# Supplementary figures and images for: MASSpy: Building, simulating, and visualizing dynamic biological models in Python using mass action kinetics (part 1 of 3)
Source: PLoS Comput Biol. 2021 Jan 28;17(1):e1008208. doi: 10.1371/journal.pcbi.1008208 (PMC7872247; doi:10.1371/journal.pcbi.1008208)

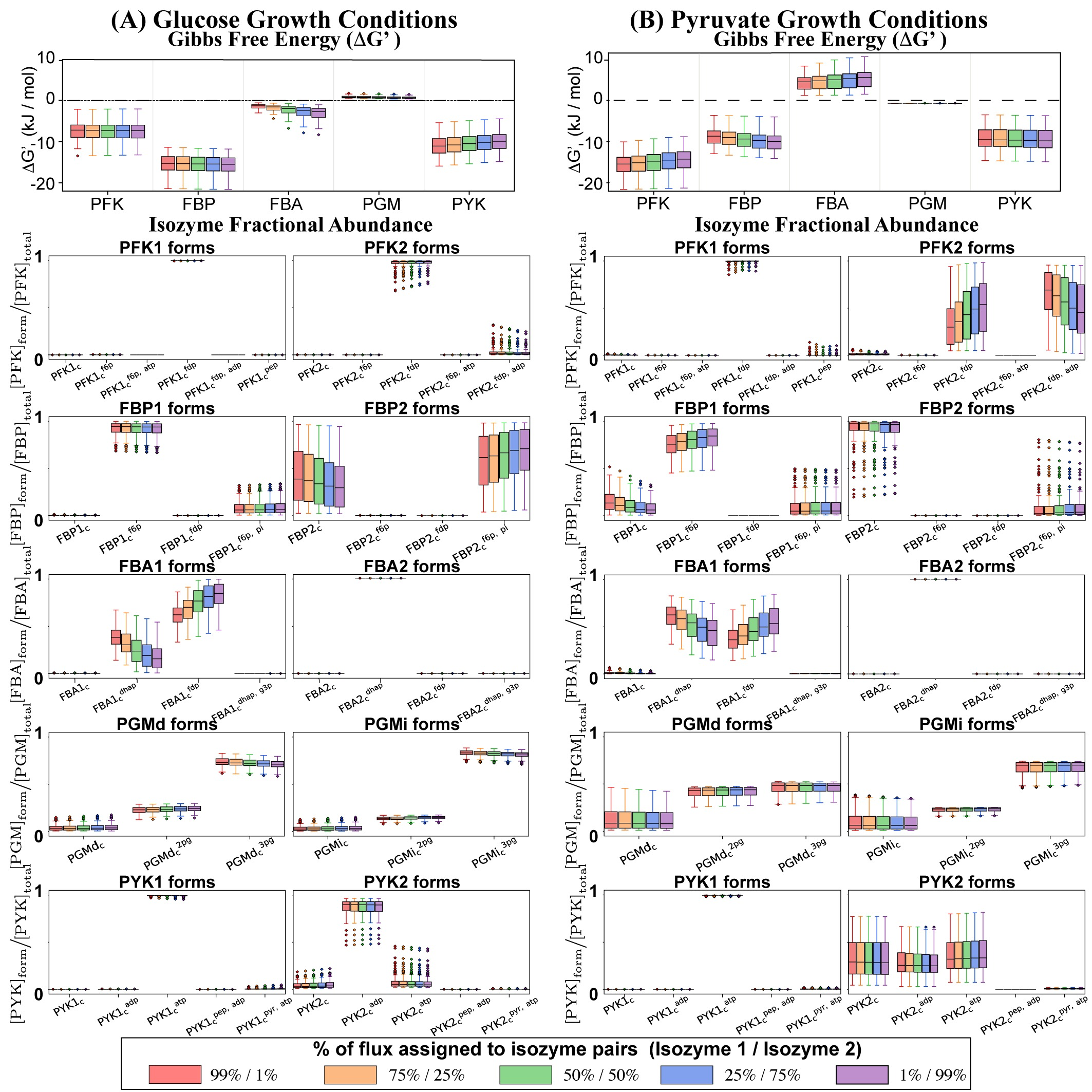

Supplement: S1 Fig — The Gibbs free energy of enzyme-catalyzed reactions and the fractional abundance for isozyme states for all isozyme pairs for (A) glucose growth conditions and (B) pyruvate growth conditions when computing the functional states of the E. coli proteome in the case study. (TIF) [file pcbi.1008208.s001.tif]

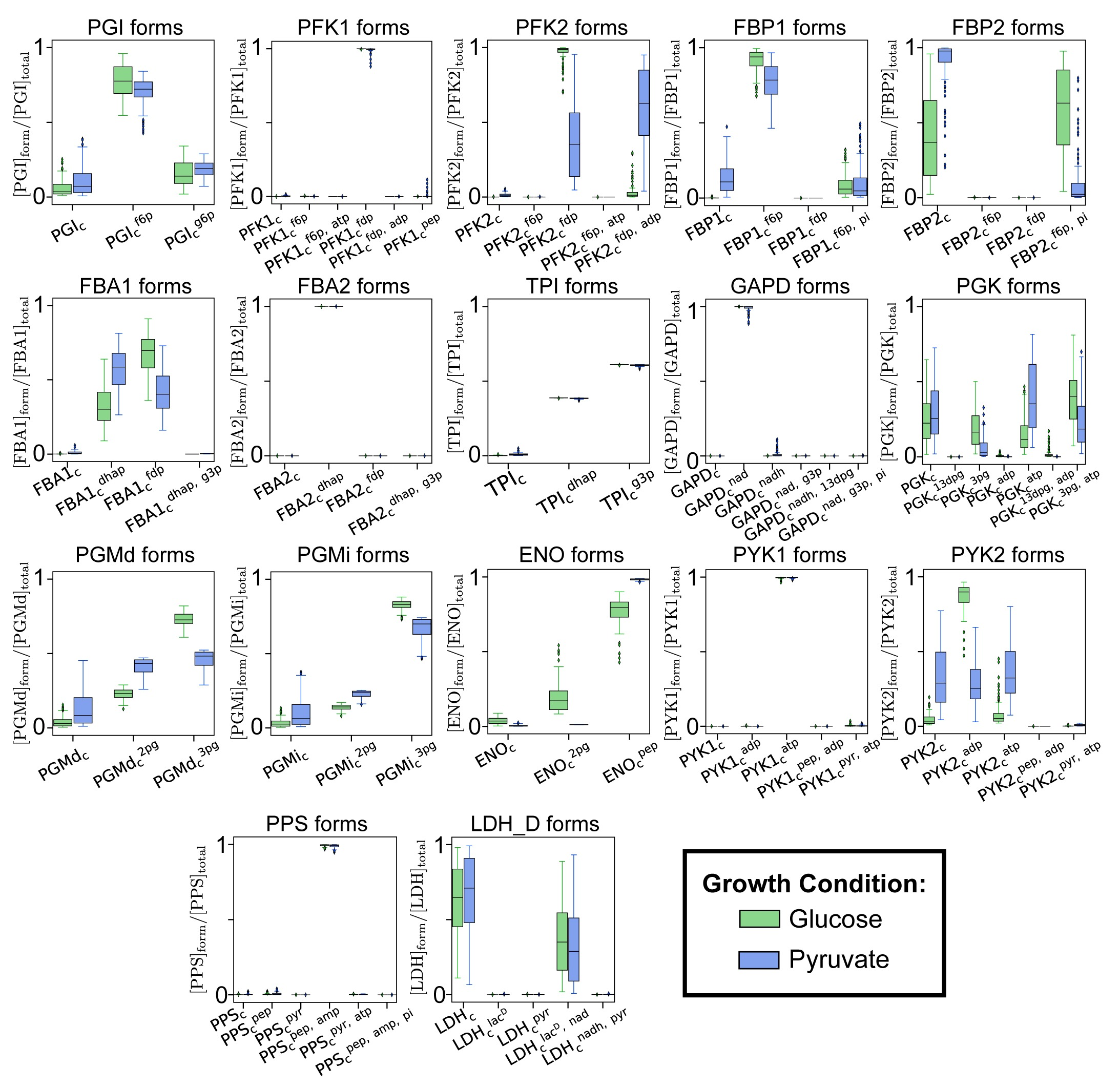

Supplement: S2 Fig — The fractional abundance for all enzymes states of all enzyme modules when computing the functional states of the E. coli proteome in the case study. (TIF) [file pcbi.1008208.s002.tif]

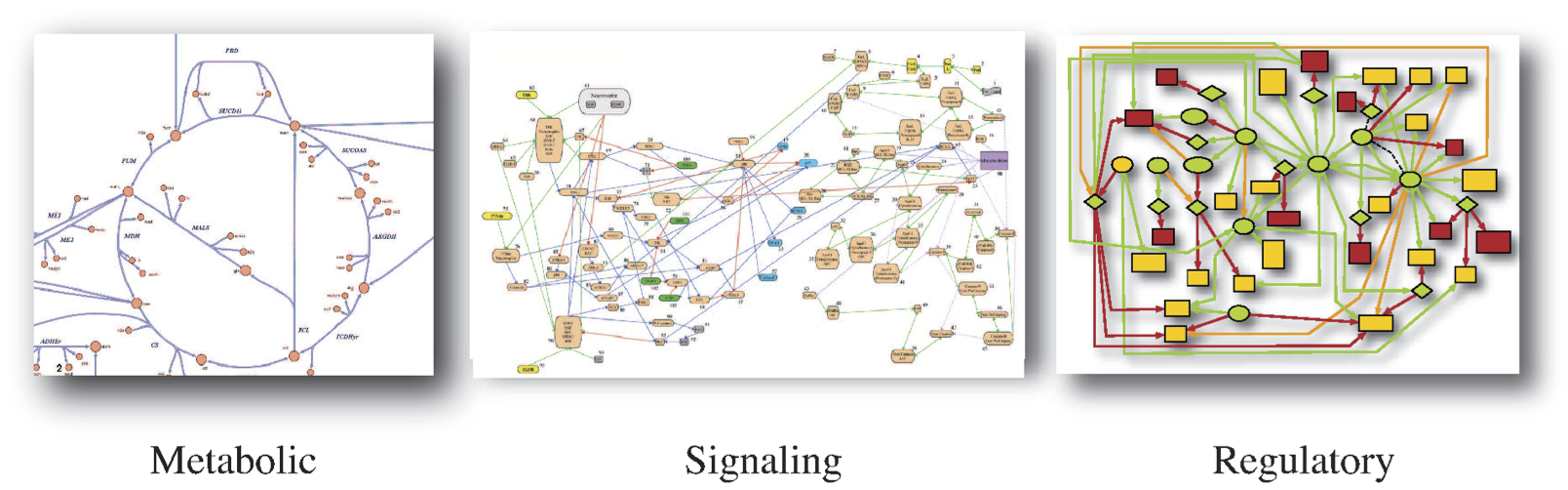

Supplement: S1 File — The latest version of the MASSpy software can be found at https://github.com/SBRG/MASSpy. (ZIP) [file pcbi.1008208.s003.zip › MASSpy-0.1.1/docs/education/sb2/images/Ch1/Figure-1-1.png]

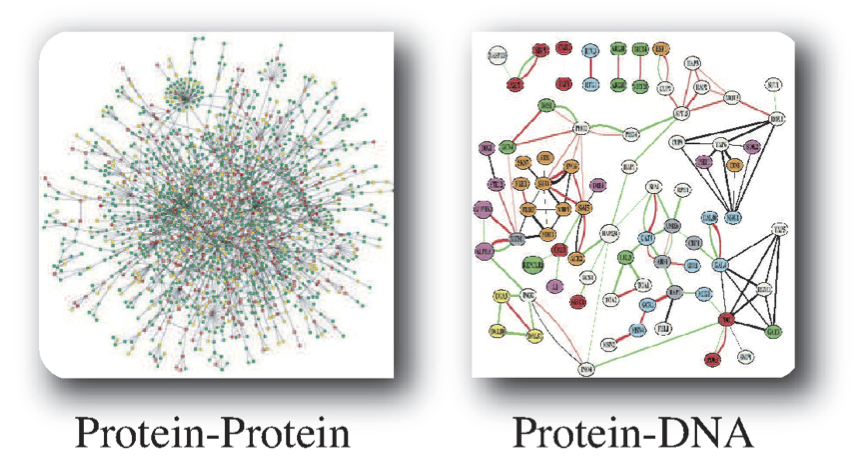

Supplement: S1 File — The latest version of the MASSpy software can be found at https://github.com/SBRG/MASSpy. (ZIP) [file pcbi.1008208.s003.zip › MASSpy-0.1.1/docs/education/sb2/images/Ch1/Figure-1-2.png]

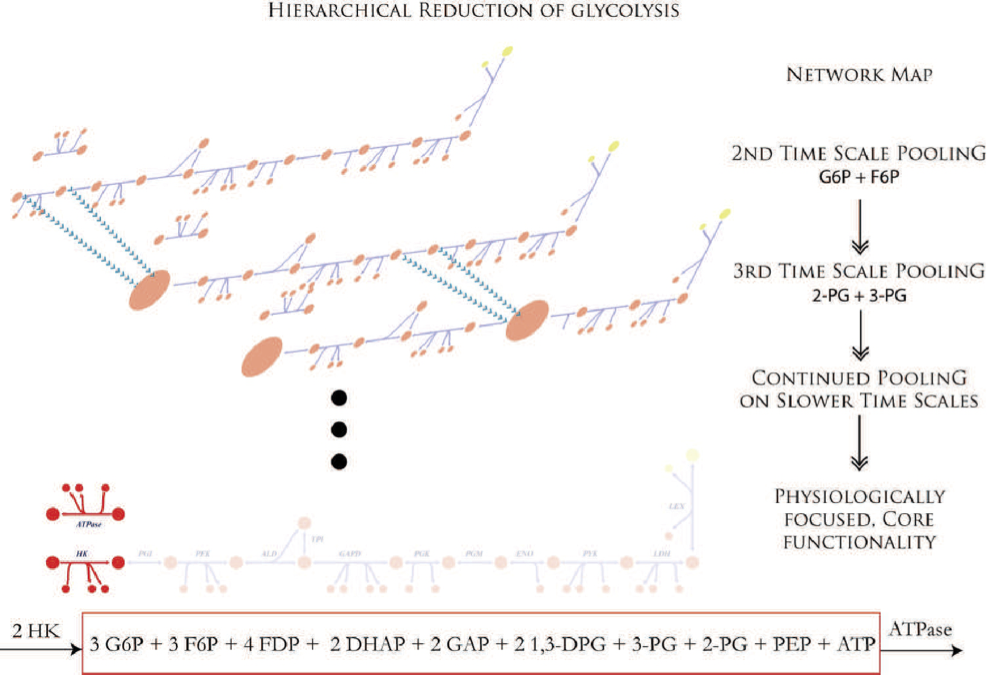

Supplement: S1 File — The latest version of the MASSpy software can be found at https://github.com/SBRG/MASSpy. (ZIP) [file pcbi.1008208.s003.zip › MASSpy-0.1.1/docs/education/sb2/images/Ch1/Figure-1-3.png]

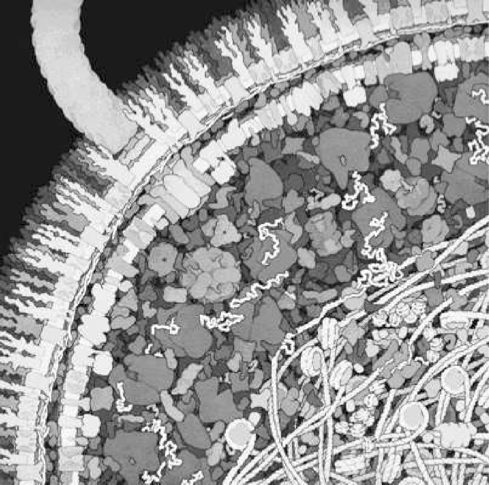

Supplement: S1 File — The latest version of the MASSpy software can be found at https://github.com/SBRG/MASSpy. (ZIP) [file pcbi.1008208.s003.zip › MASSpy-0.1.1/docs/education/sb2/images/Ch1/Figure-1-4.png]

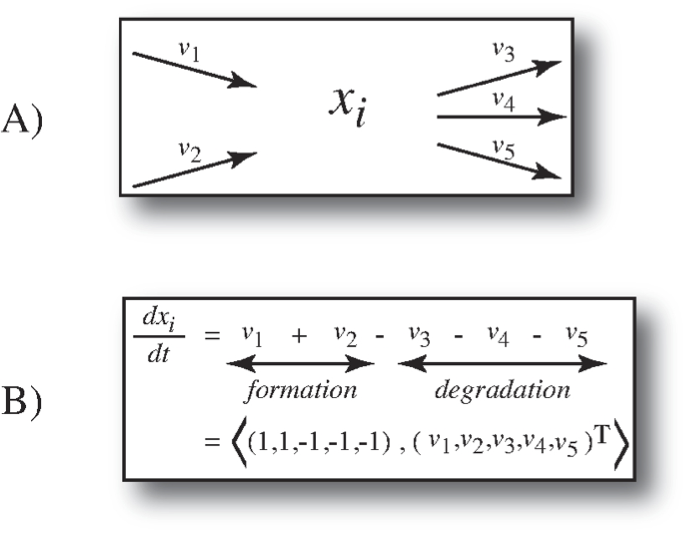

Supplement: S1 File — The latest version of the MASSpy software can be found at https://github.com/SBRG/MASSpy. (ZIP) [file pcbi.1008208.s003.zip › MASSpy-0.1.1/docs/education/sb2/images/Ch1/Figure-1-5.png]

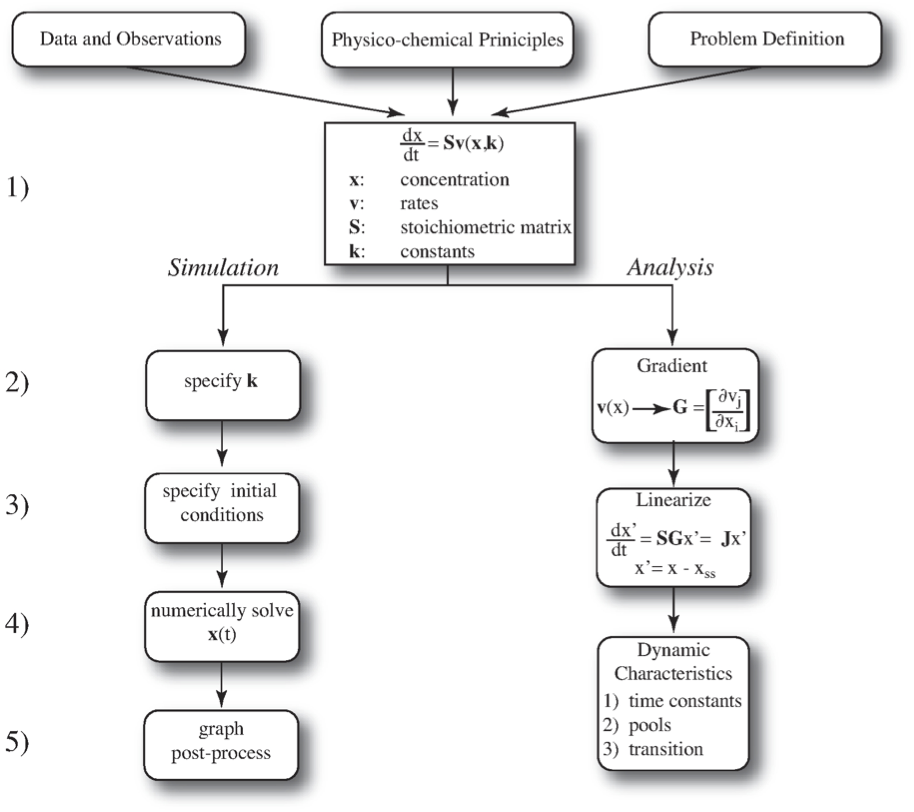

Supplement: S1 File — The latest version of the MASSpy software can be found at https://github.com/SBRG/MASSpy. (ZIP) [file pcbi.1008208.s003.zip › MASSpy-0.1.1/docs/education/sb2/images/Ch1/Figure-1-6.png]

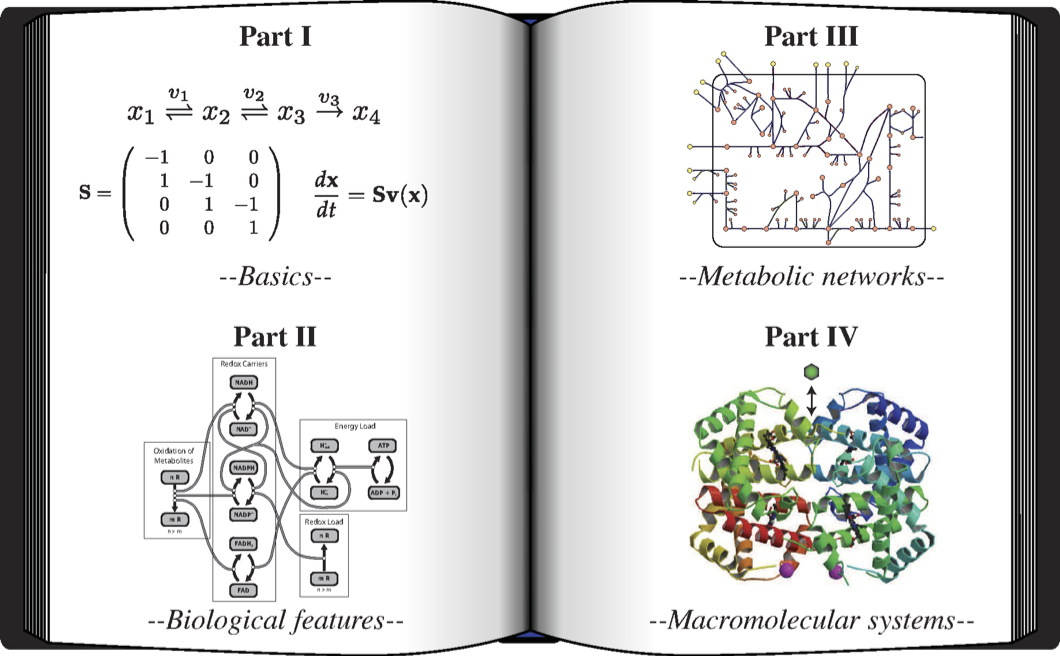

Supplement: S1 File — The latest version of the MASSpy software can be found at https://github.com/SBRG/MASSpy. (ZIP) [file pcbi.1008208.s003.zip › MASSpy-0.1.1/docs/education/sb2/images/Ch1/Figure-1-7.png]

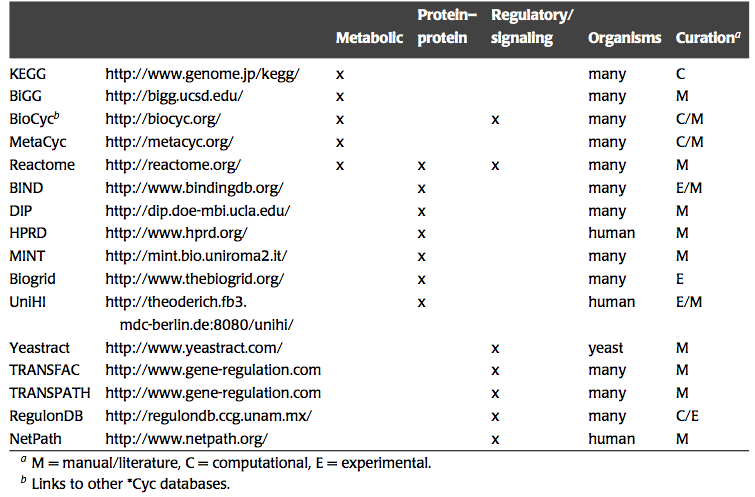

Supplement: S1 File — The latest version of the MASSpy software can be found at https://github.com/SBRG/MASSpy. (ZIP) [file pcbi.1008208.s003.zip › MASSpy-0.1.1/docs/education/sb2/images/Ch1/Table-1-1.png]

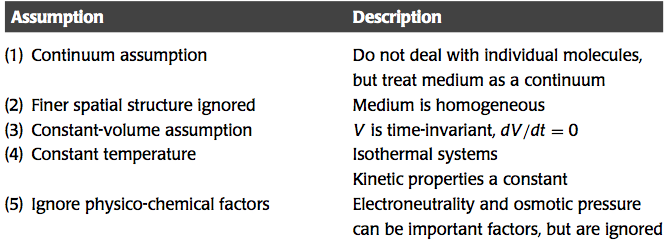

Supplement: S1 File — The latest version of the MASSpy software can be found at https://github.com/SBRG/MASSpy. (ZIP) [file pcbi.1008208.s003.zip › MASSpy-0.1.1/docs/education/sb2/images/Ch1/Table-1-2.png]

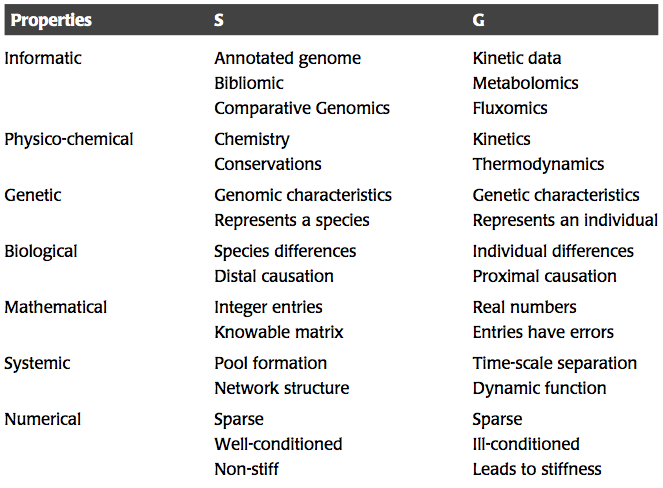

Supplement: S1 File — The latest version of the MASSpy software can be found at https://github.com/SBRG/MASSpy. (ZIP) [file pcbi.1008208.s003.zip › MASSpy-0.1.1/docs/education/sb2/images/Ch1/Table-1-3.png]

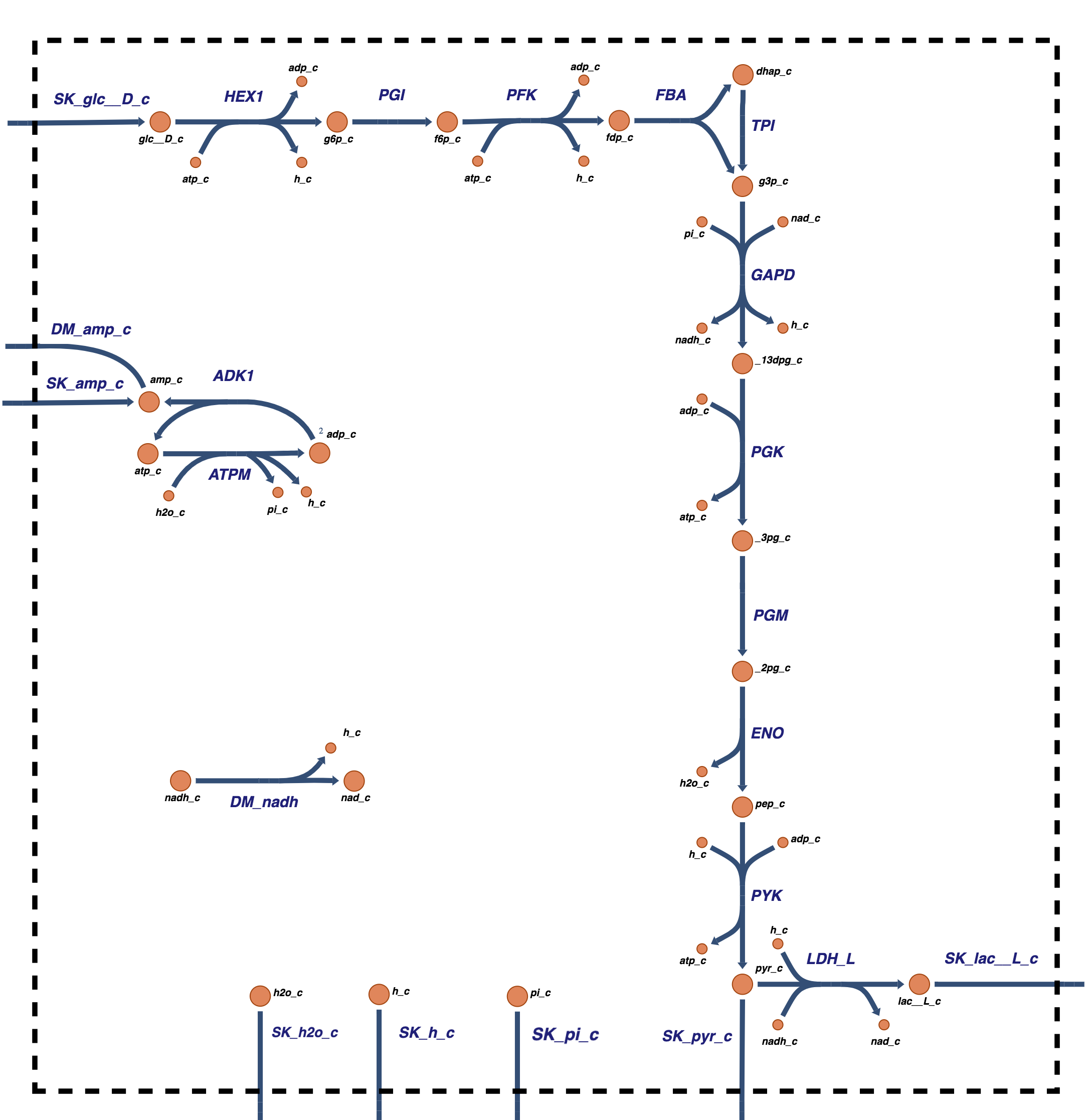

Supplement: S1 File — The latest version of the MASSpy software can be found at https://github.com/SBRG/MASSpy. (ZIP) [file pcbi.1008208.s003.zip › MASSpy-0.1.1/docs/education/sb2/images/Ch10/Figure-10-1.png]

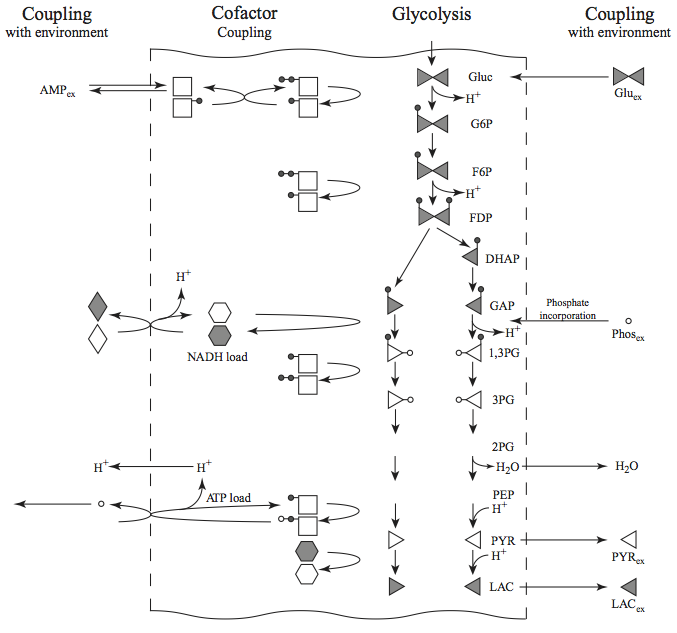

Supplement: S1 File — The latest version of the MASSpy software can be found at https://github.com/SBRG/MASSpy. (ZIP) [file pcbi.1008208.s003.zip › MASSpy-0.1.1/docs/education/sb2/images/Ch10/Figure-10-16.png]

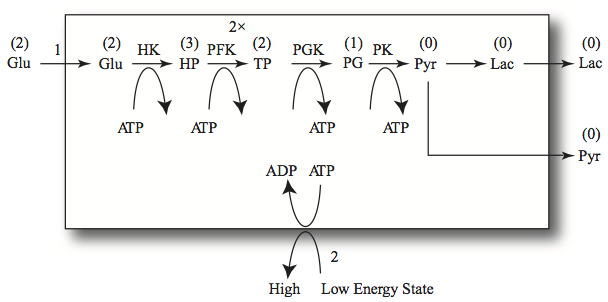

Supplement: S1 File — The latest version of the MASSpy software can be found at https://github.com/SBRG/MASSpy. (ZIP) [file pcbi.1008208.s003.zip › MASSpy-0.1.1/docs/education/sb2/images/Ch10/Figure-10-17.png]

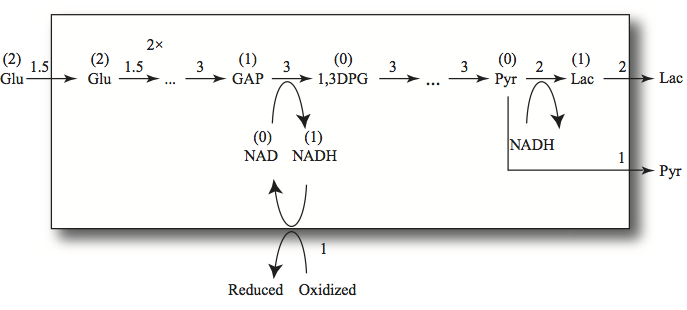

Supplement: S1 File — The latest version of the MASSpy software can be found at https://github.com/SBRG/MASSpy. (ZIP) [file pcbi.1008208.s003.zip › MASSpy-0.1.1/docs/education/sb2/images/Ch10/Figure-10-18.png]

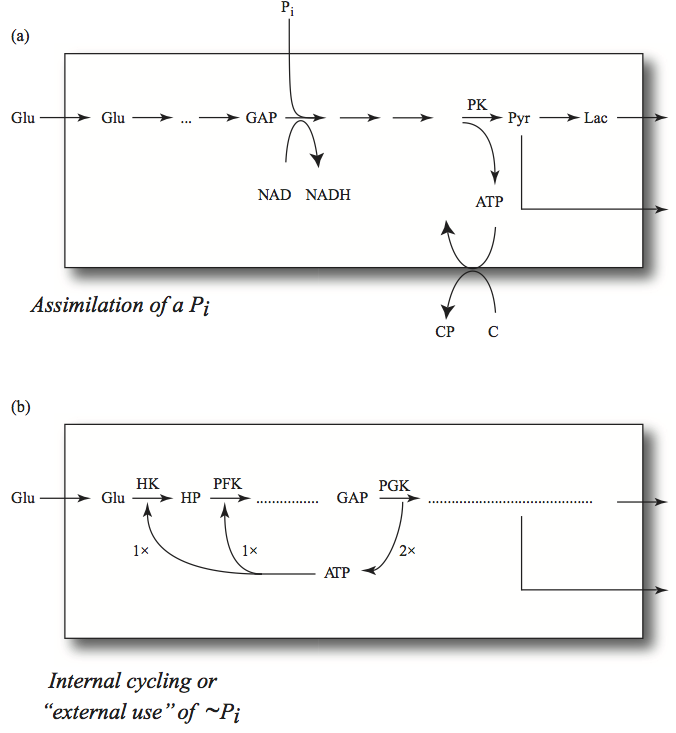

Supplement: S1 File — The latest version of the MASSpy software can be found at https://github.com/SBRG/MASSpy. (ZIP) [file pcbi.1008208.s003.zip › MASSpy-0.1.1/docs/education/sb2/images/Ch10/Figure-10-19.png]

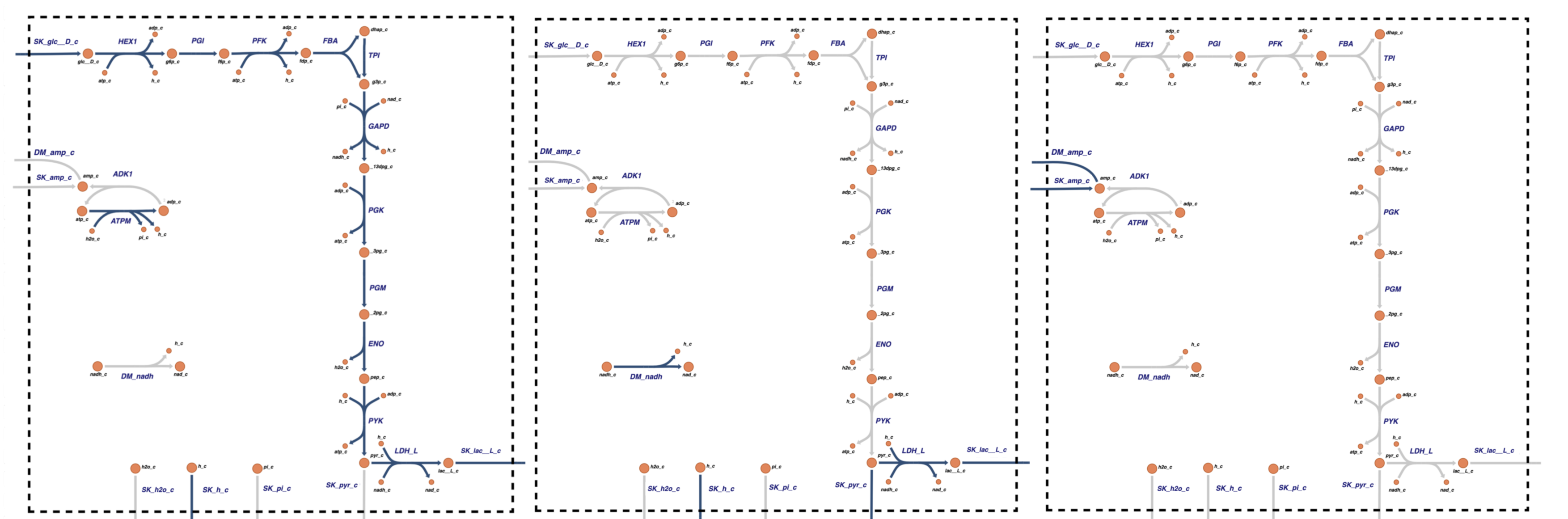

Supplement: S1 File — The latest version of the MASSpy software can be found at https://github.com/SBRG/MASSpy. (ZIP) [file pcbi.1008208.s003.zip › MASSpy-0.1.1/docs/education/sb2/images/Ch10/Figure-10-2.png]

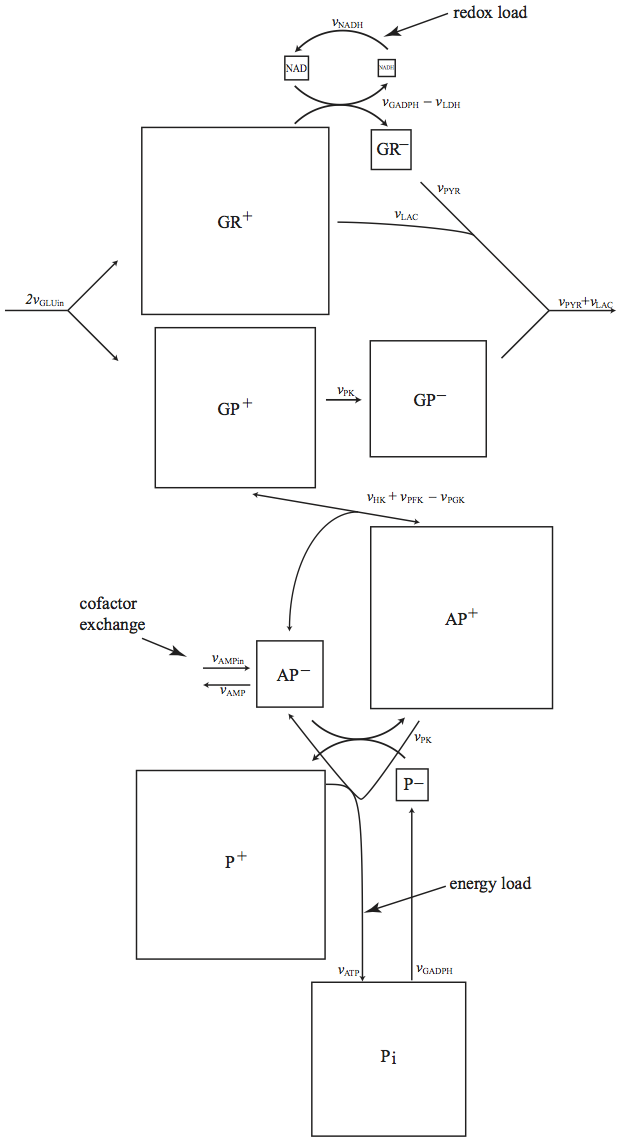

Supplement: S1 File — The latest version of the MASSpy software can be found at https://github.com/SBRG/MASSpy. (ZIP) [file pcbi.1008208.s003.zip › MASSpy-0.1.1/docs/education/sb2/images/Ch10/Figure-10-20.png]

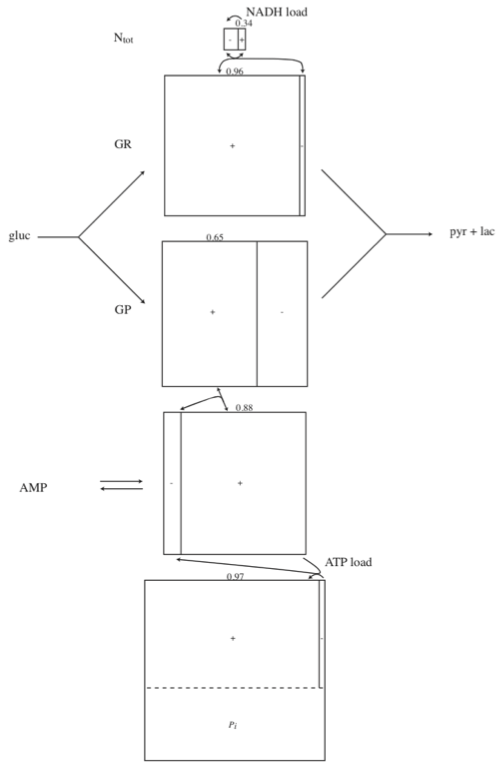

Supplement: S1 File — The latest version of the MASSpy software can be found at https://github.com/SBRG/MASSpy. (ZIP) [file pcbi.1008208.s003.zip › MASSpy-0.1.1/docs/education/sb2/images/Ch10/Figure-10-21.png]

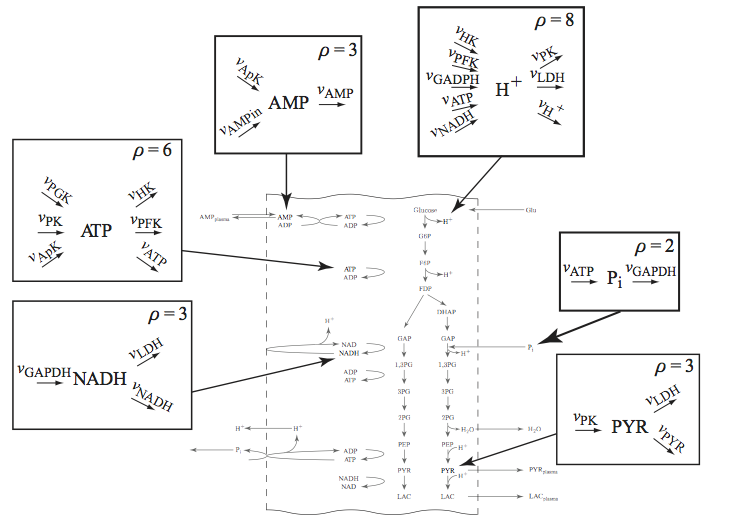

Supplement: S1 File — The latest version of the MASSpy software can be found at https://github.com/SBRG/MASSpy. (ZIP) [file pcbi.1008208.s003.zip › MASSpy-0.1.1/docs/education/sb2/images/Ch10/Figure-10-4.png]

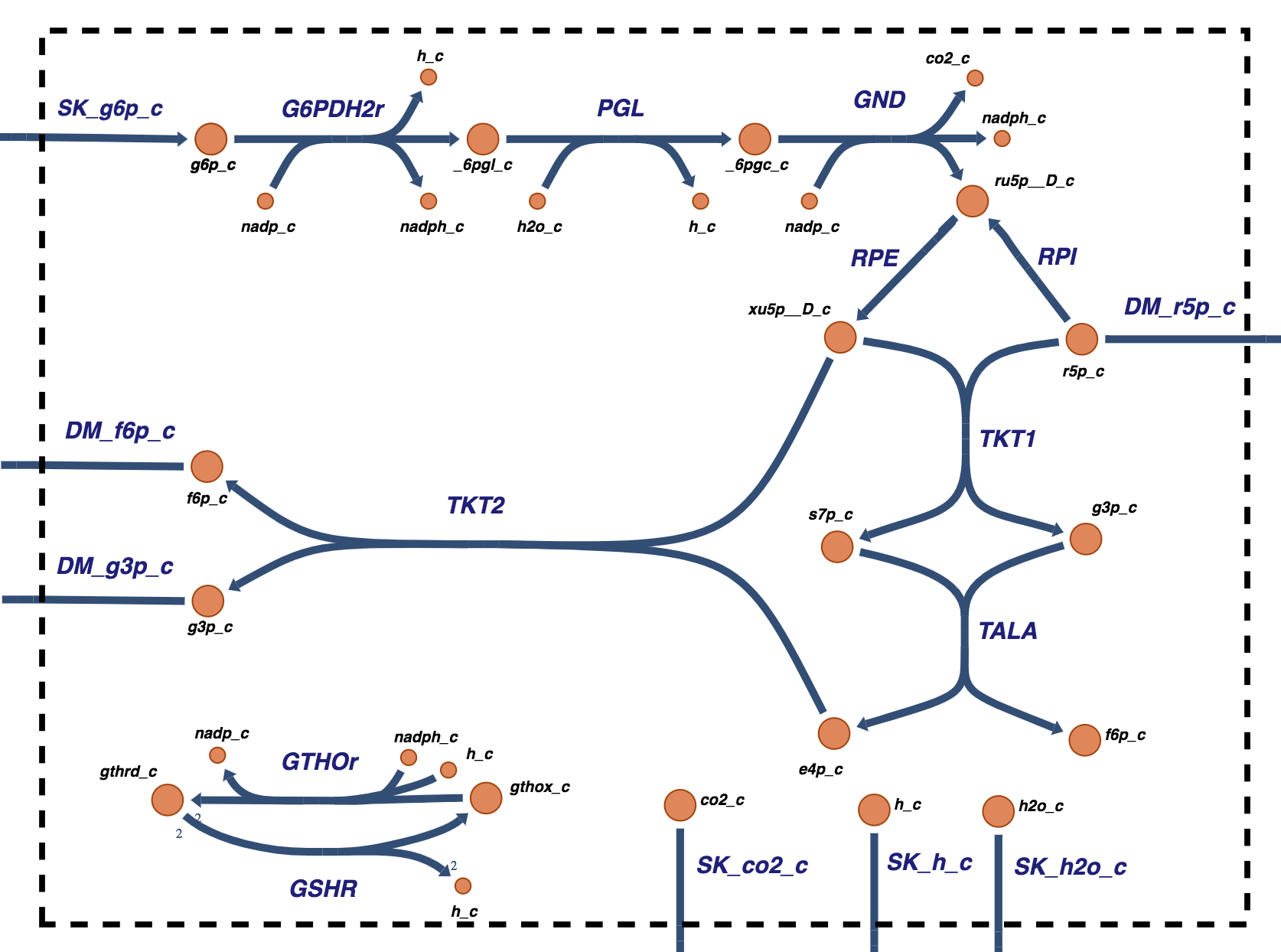

Supplement: S1 File — The latest version of the MASSpy software can be found at https://github.com/SBRG/MASSpy. (ZIP) [file pcbi.1008208.s003.zip › MASSpy-0.1.1/docs/education/sb2/images/Ch11/Figure-11-1.png]

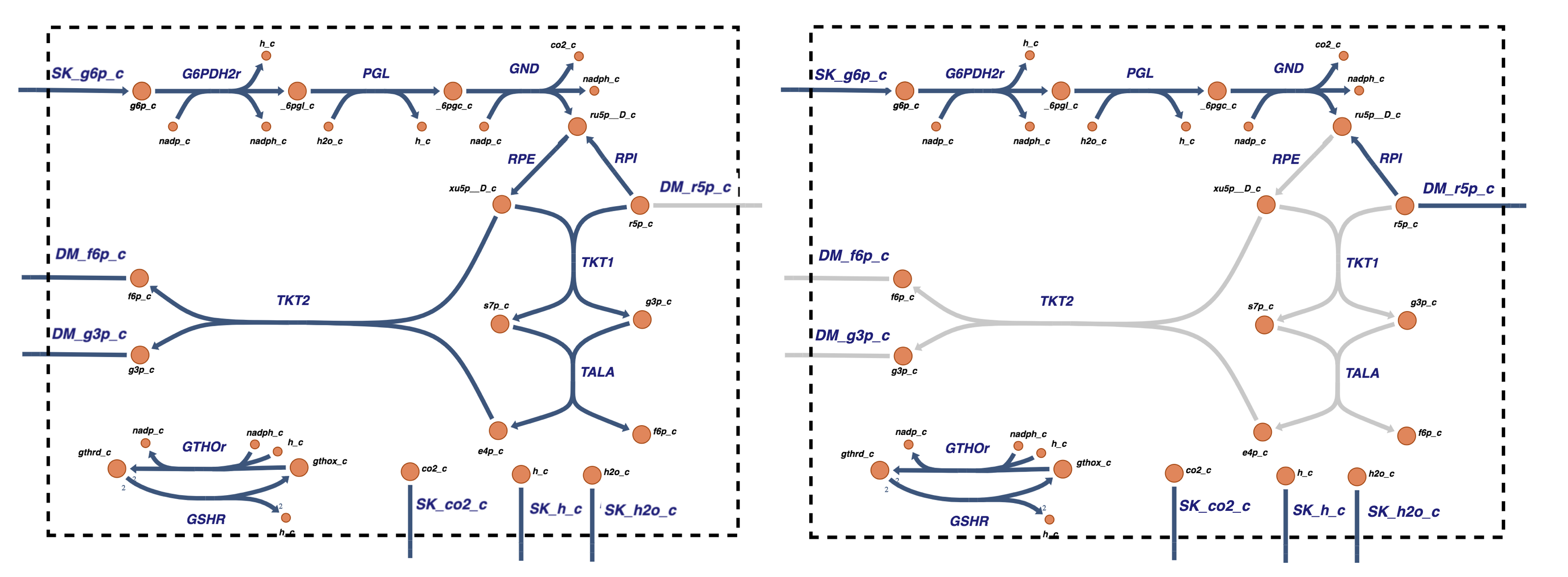

Supplement: S1 File — The latest version of the MASSpy software can be found at https://github.com/SBRG/MASSpy. (ZIP) [file pcbi.1008208.s003.zip › MASSpy-0.1.1/docs/education/sb2/images/Ch11/Figure-11-2.png]

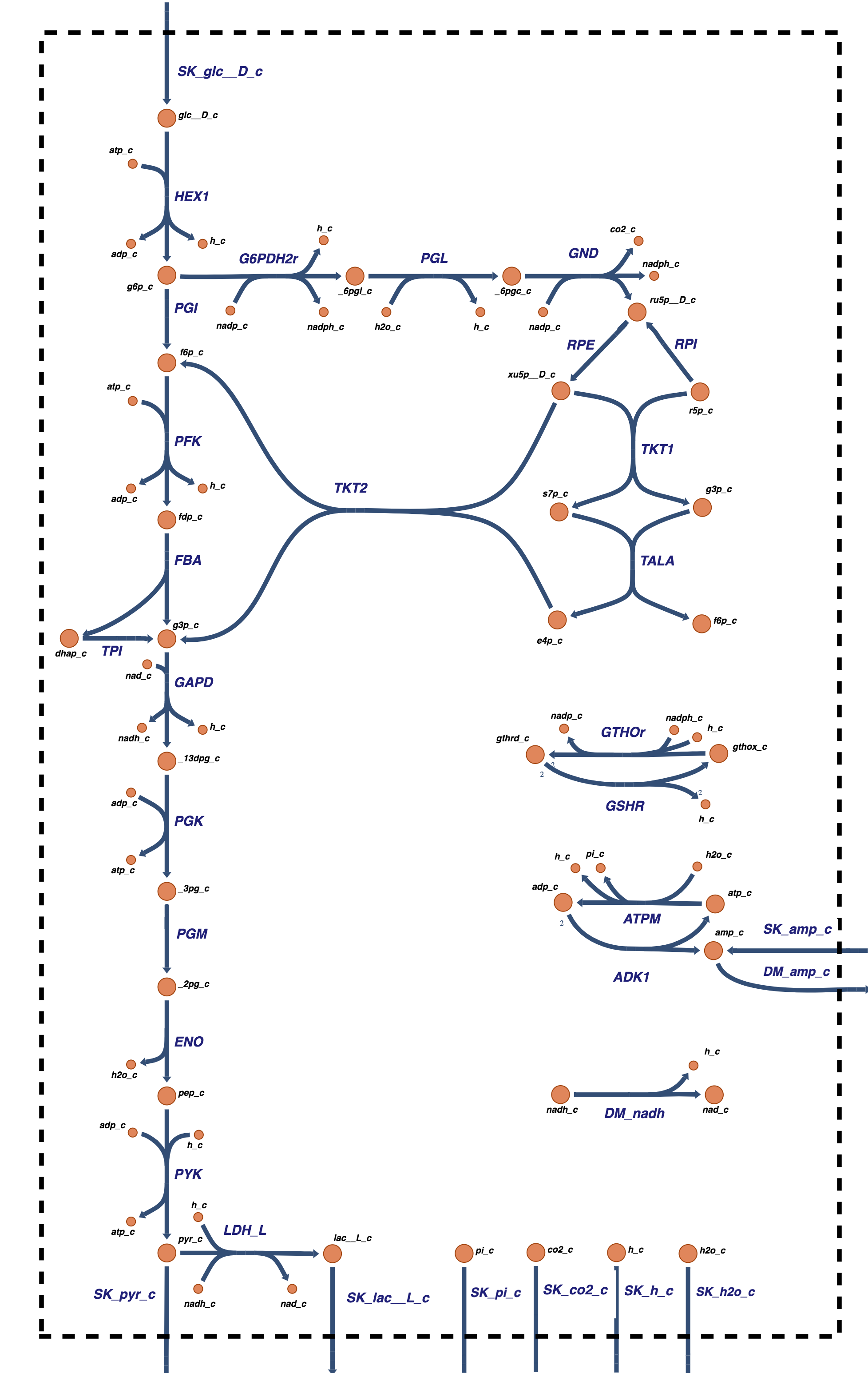

Supplement: S1 File — The latest version of the MASSpy software can be found at https://github.com/SBRG/MASSpy. (ZIP) [file pcbi.1008208.s003.zip › MASSpy-0.1.1/docs/education/sb2/images/Ch11/Figure-11-6.png]

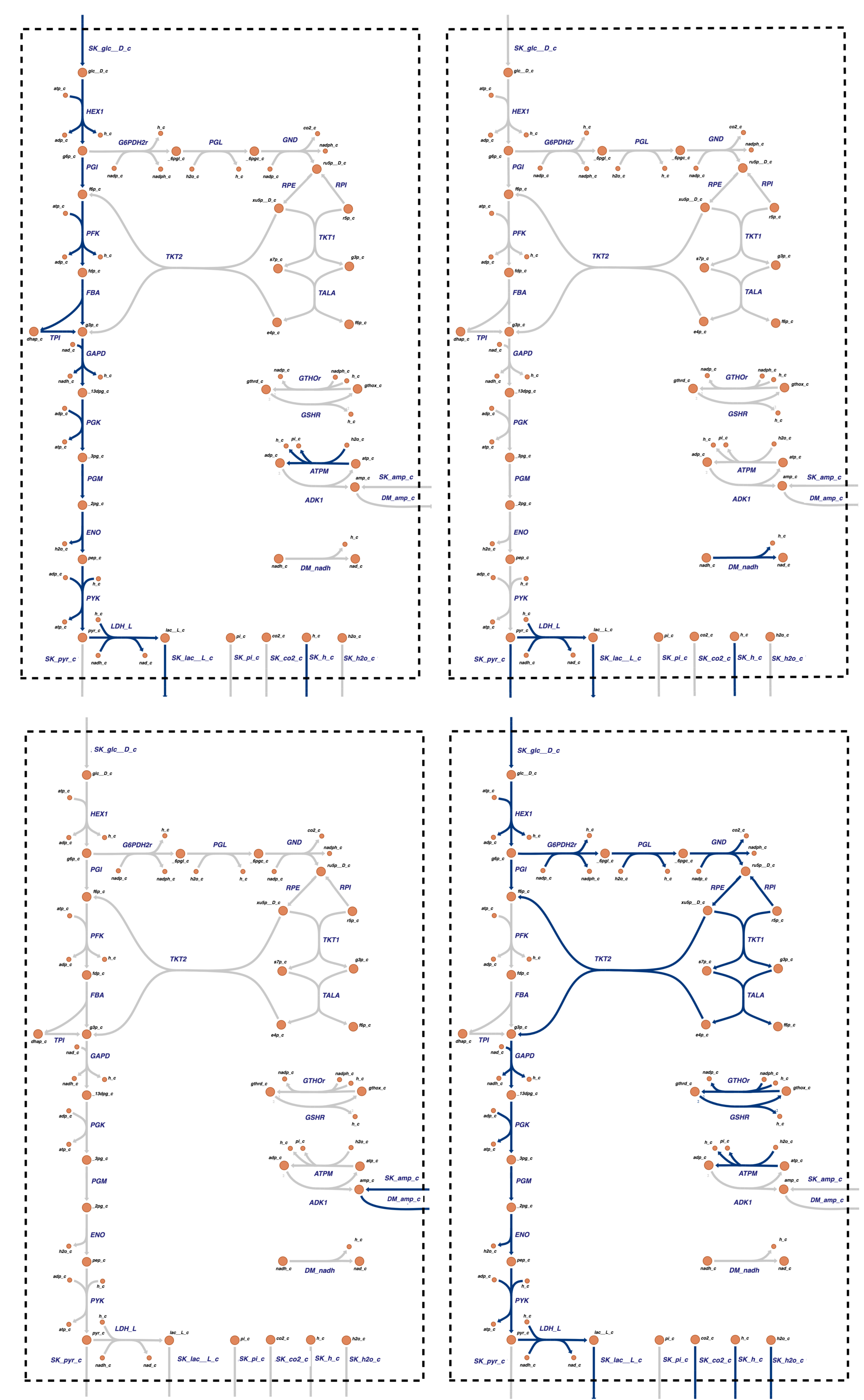

Supplement: S1 File — The latest version of the MASSpy software can be found at https://github.com/SBRG/MASSpy. (ZIP) [file pcbi.1008208.s003.zip › MASSpy-0.1.1/docs/education/sb2/images/Ch11/Figure-11-8.png]

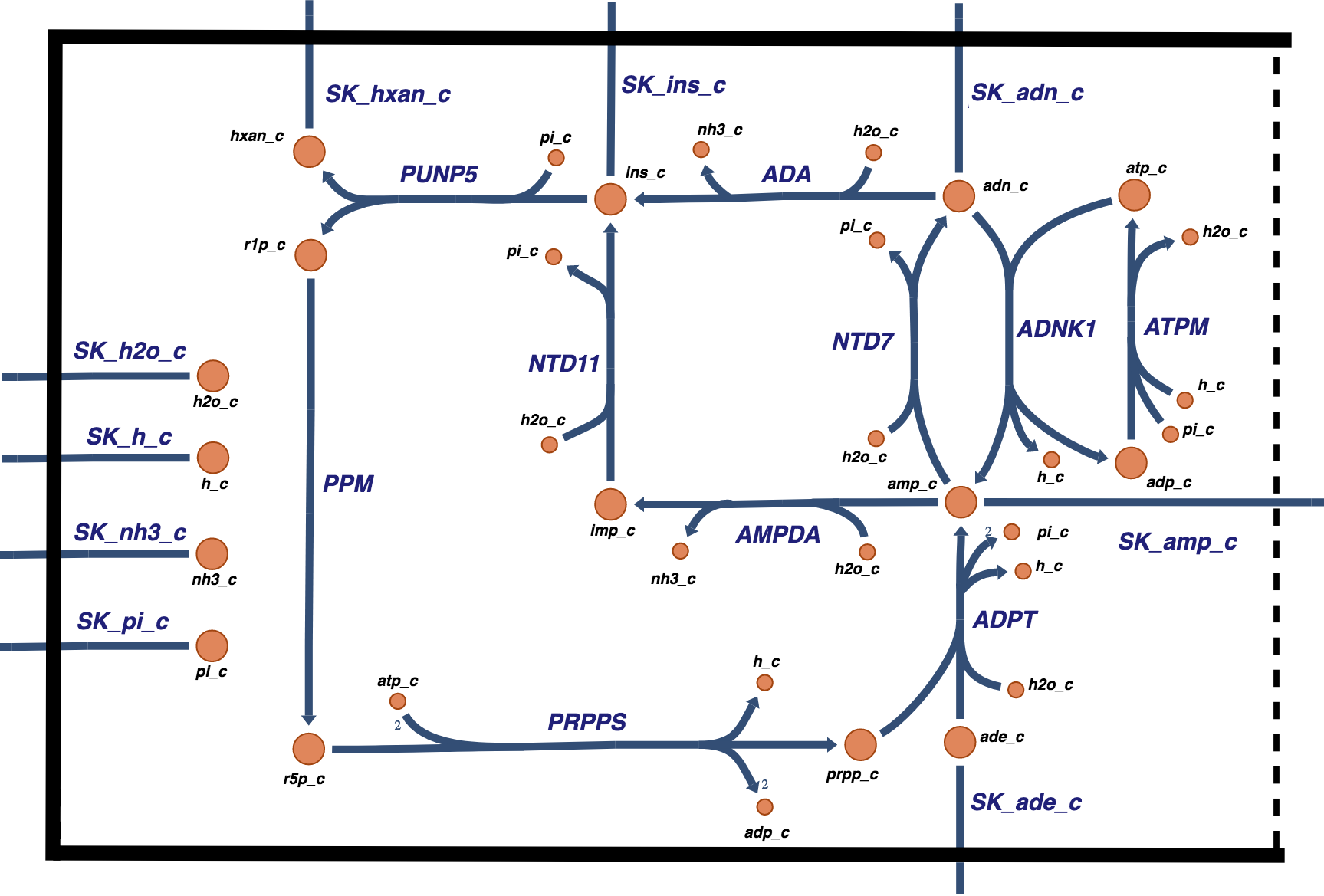

Supplement: S1 File — The latest version of the MASSpy software can be found at https://github.com/SBRG/MASSpy. (ZIP) [file pcbi.1008208.s003.zip › MASSpy-0.1.1/docs/education/sb2/images/Ch12/Figure-12-1.png]

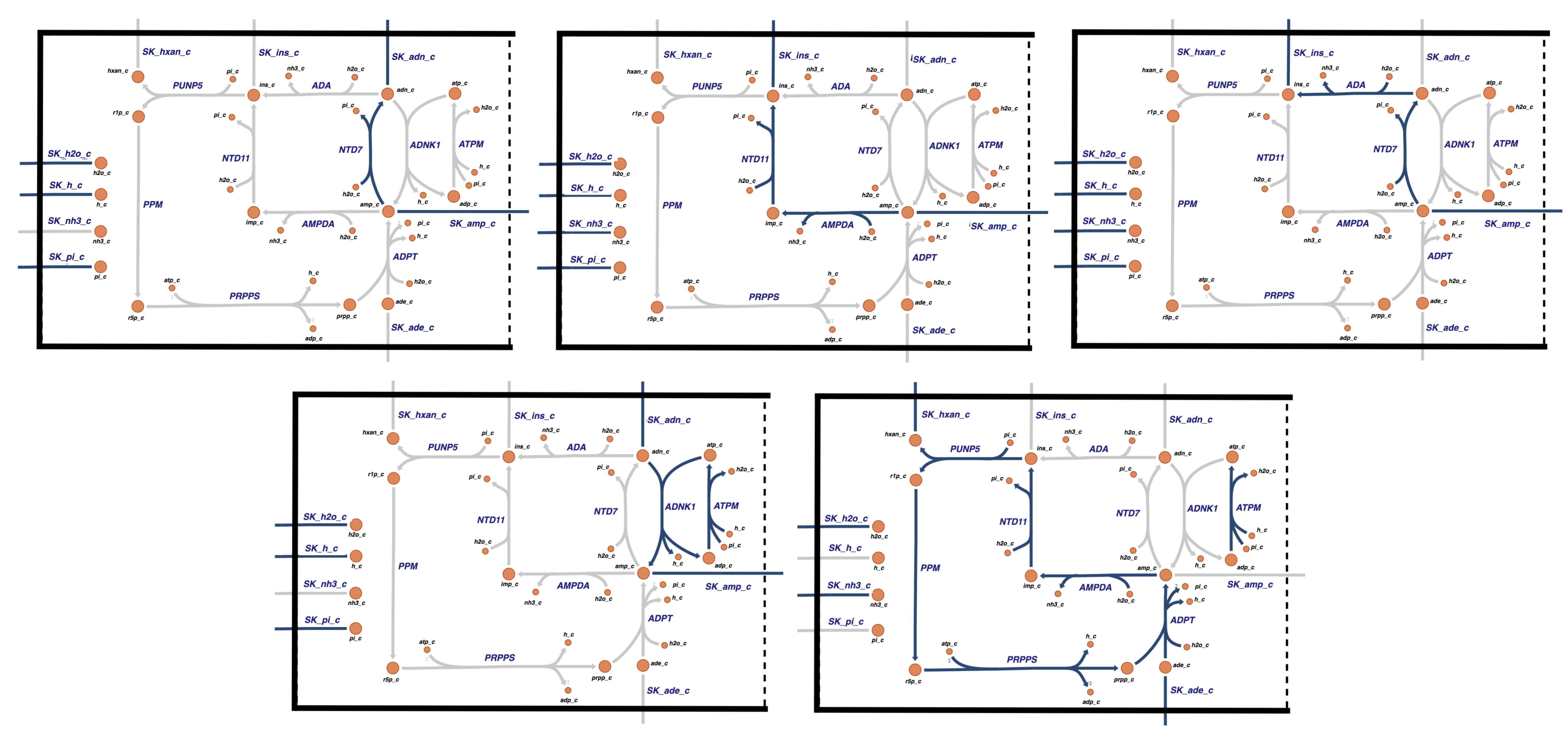

Supplement: S1 File — The latest version of the MASSpy software can be found at https://github.com/SBRG/MASSpy. (ZIP) [file pcbi.1008208.s003.zip › MASSpy-0.1.1/docs/education/sb2/images/Ch12/Figure-12-2.png]

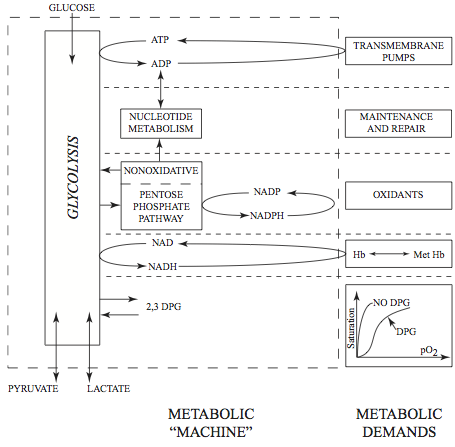

Supplement: S1 File — The latest version of the MASSpy software can be found at https://github.com/SBRG/MASSpy. (ZIP) [file pcbi.1008208.s003.zip › MASSpy-0.1.1/docs/education/sb2/images/Ch12/Figure-12-20.png]

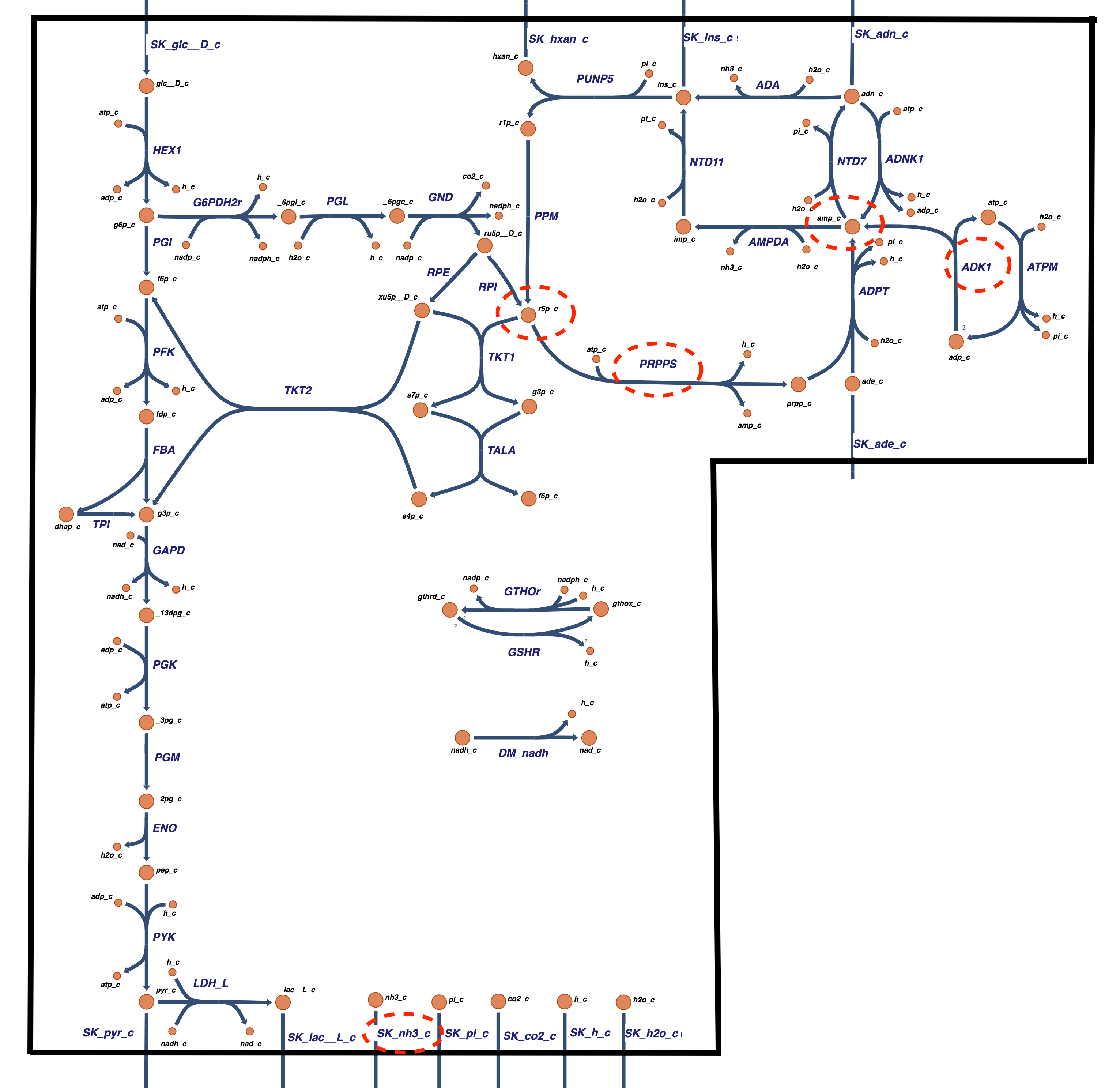

Supplement: S1 File — The latest version of the MASSpy software can be found at https://github.com/SBRG/MASSpy. (ZIP) [file pcbi.1008208.s003.zip › MASSpy-0.1.1/docs/education/sb2/images/Ch12/Figure-12-6.png]

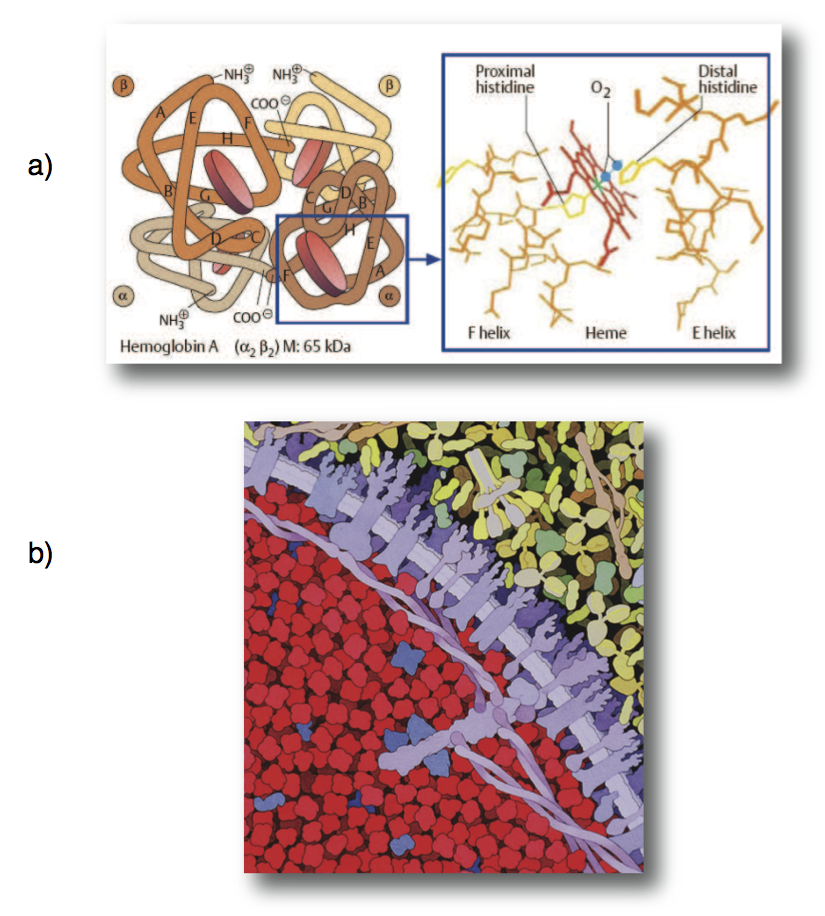

Supplement: S1 File — The latest version of the MASSpy software can be found at https://github.com/SBRG/MASSpy. (ZIP) [file pcbi.1008208.s003.zip › MASSpy-0.1.1/docs/education/sb2/images/Ch13/Figure-13-1.png]

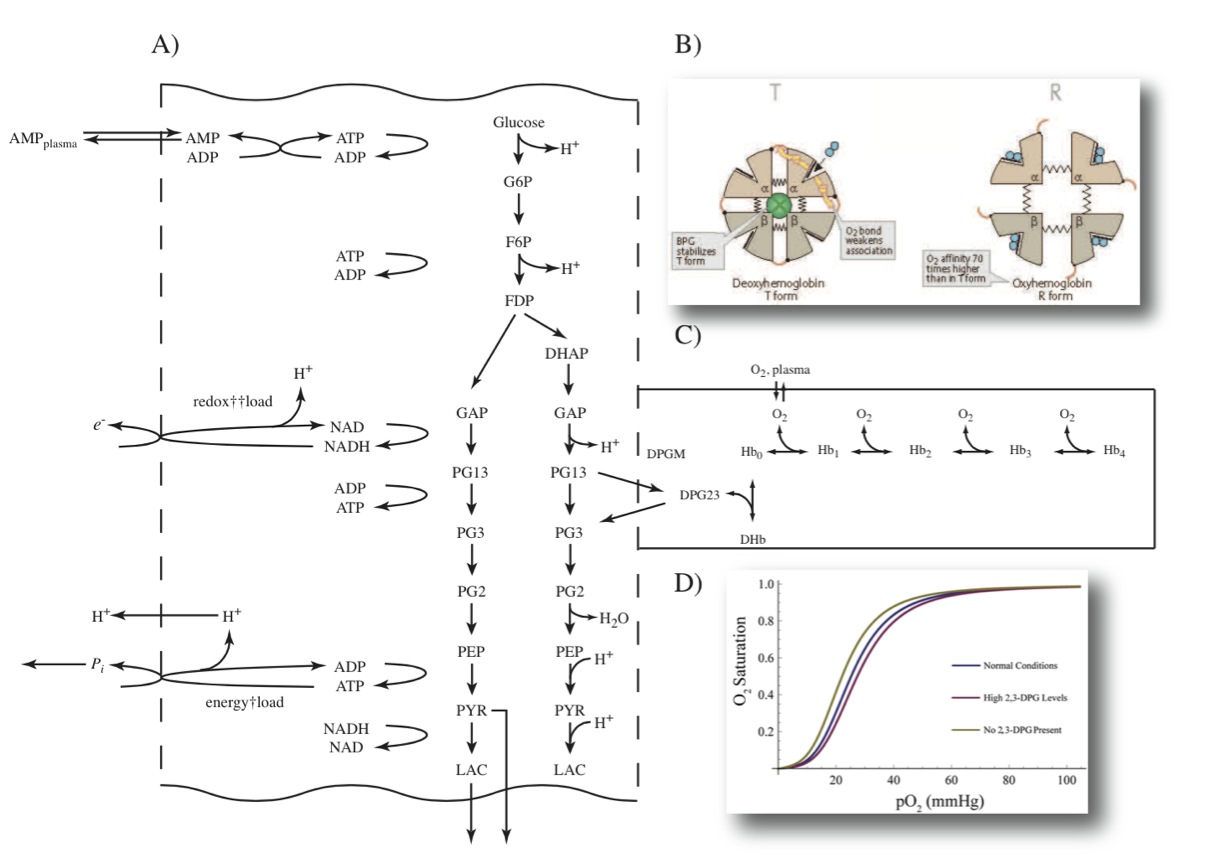

Supplement: S1 File — The latest version of the MASSpy software can be found at https://github.com/SBRG/MASSpy. (ZIP) [file pcbi.1008208.s003.zip › MASSpy-0.1.1/docs/education/sb2/images/Ch13/Figure-13-2.png]

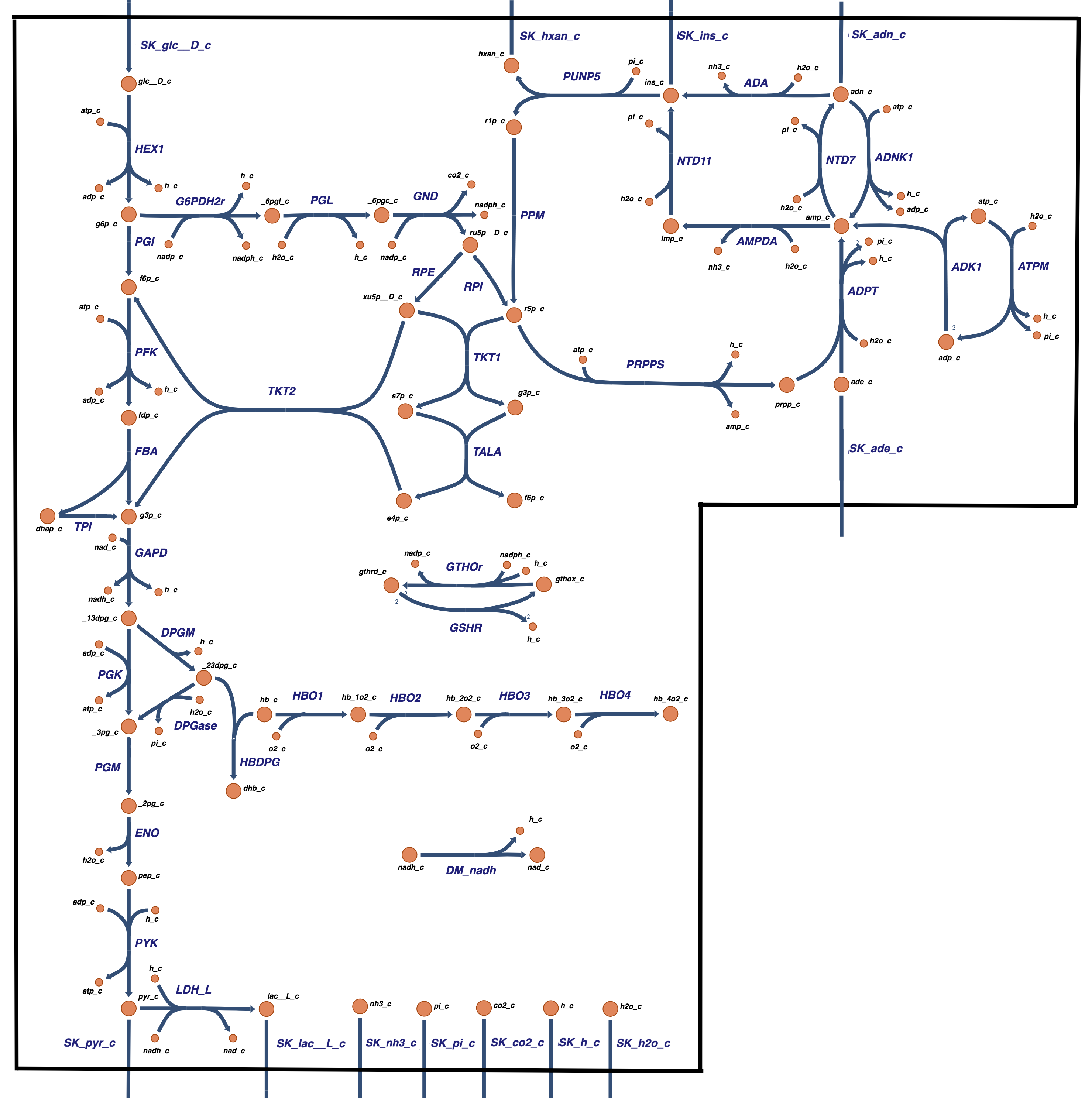

Supplement: S1 File — The latest version of the MASSpy software can be found at https://github.com/SBRG/MASSpy. (ZIP) [file pcbi.1008208.s003.zip › MASSpy-0.1.1/docs/education/sb2/images/Ch13/Figure-13-6.png]

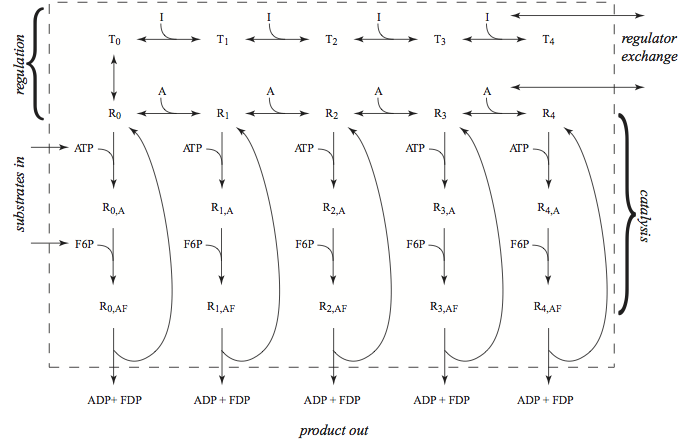

Supplement: S1 File — The latest version of the MASSpy software can be found at https://github.com/SBRG/MASSpy. (ZIP) [file pcbi.1008208.s003.zip › MASSpy-0.1.1/docs/education/sb2/images/Ch14/Figure-14-1.png]

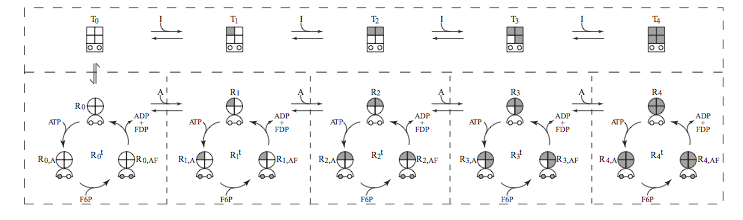

Supplement: S1 File — The latest version of the MASSpy software can be found at https://github.com/SBRG/MASSpy. (ZIP) [file pcbi.1008208.s003.zip › MASSpy-0.1.1/docs/education/sb2/images/Ch14/Figure-14-2.png]

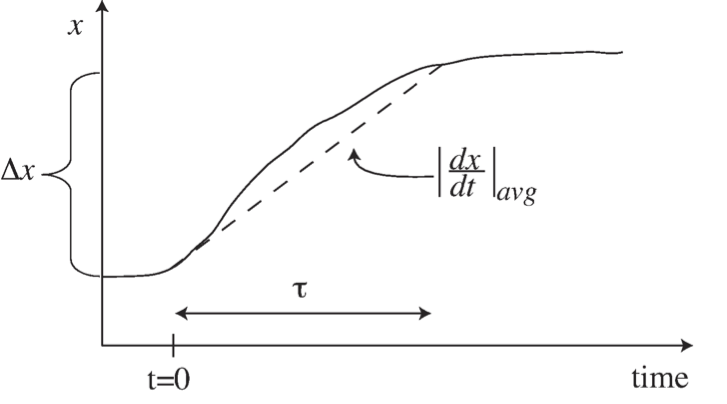

Supplement: S1 File — The latest version of the MASSpy software can be found at https://github.com/SBRG/MASSpy. (ZIP) [file pcbi.1008208.s003.zip › MASSpy-0.1.1/docs/education/sb2/images/Ch2/Figure-2-1.png]

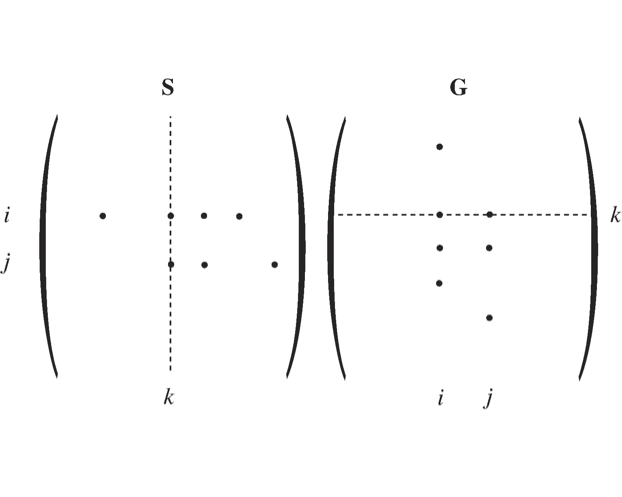

Supplement: S1 File — The latest version of the MASSpy software can be found at https://github.com/SBRG/MASSpy. (ZIP) [file pcbi.1008208.s003.zip › MASSpy-0.1.1/docs/education/sb2/images/Ch2/Figure-2-10.png]

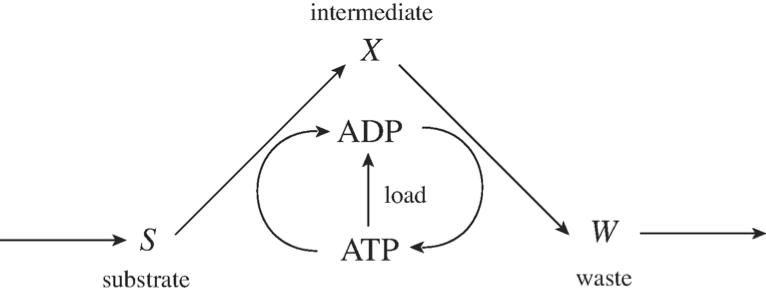

Supplement: S1 File — The latest version of the MASSpy software can be found at https://github.com/SBRG/MASSpy. (ZIP) [file pcbi.1008208.s003.zip › MASSpy-0.1.1/docs/education/sb2/images/Ch2/Figure-2-11.png]

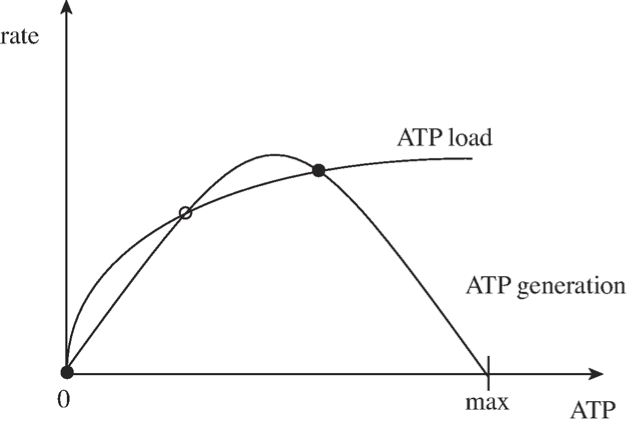

Supplement: S1 File — The latest version of the MASSpy software can be found at https://github.com/SBRG/MASSpy. (ZIP) [file pcbi.1008208.s003.zip › MASSpy-0.1.1/docs/education/sb2/images/Ch2/Figure-2-12.png]

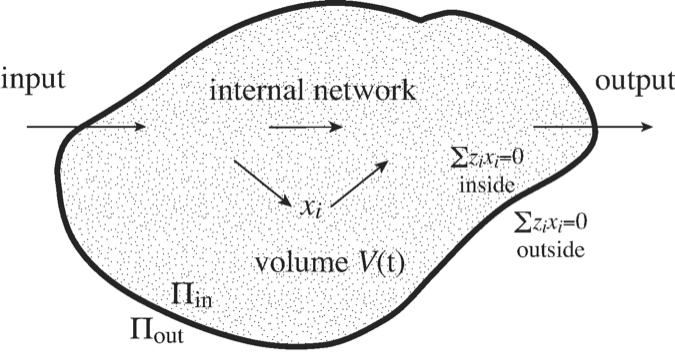

Supplement: S1 File — The latest version of the MASSpy software can be found at https://github.com/SBRG/MASSpy. (ZIP) [file pcbi.1008208.s003.zip › MASSpy-0.1.1/docs/education/sb2/images/Ch2/Figure-2-13.png]

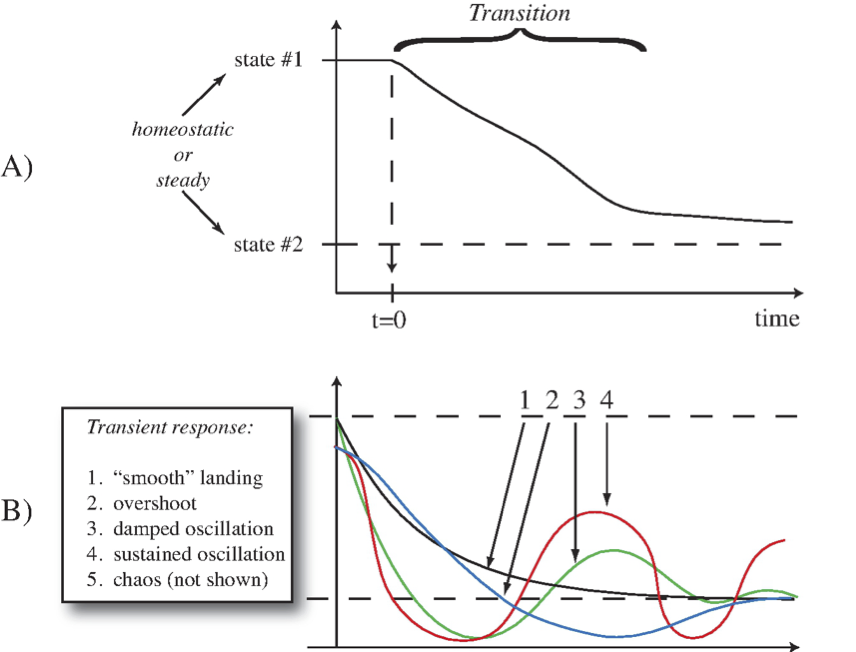

Supplement: S1 File — The latest version of the MASSpy software can be found at https://github.com/SBRG/MASSpy. (ZIP) [file pcbi.1008208.s003.zip › MASSpy-0.1.1/docs/education/sb2/images/Ch2/Figure-2-2.png]

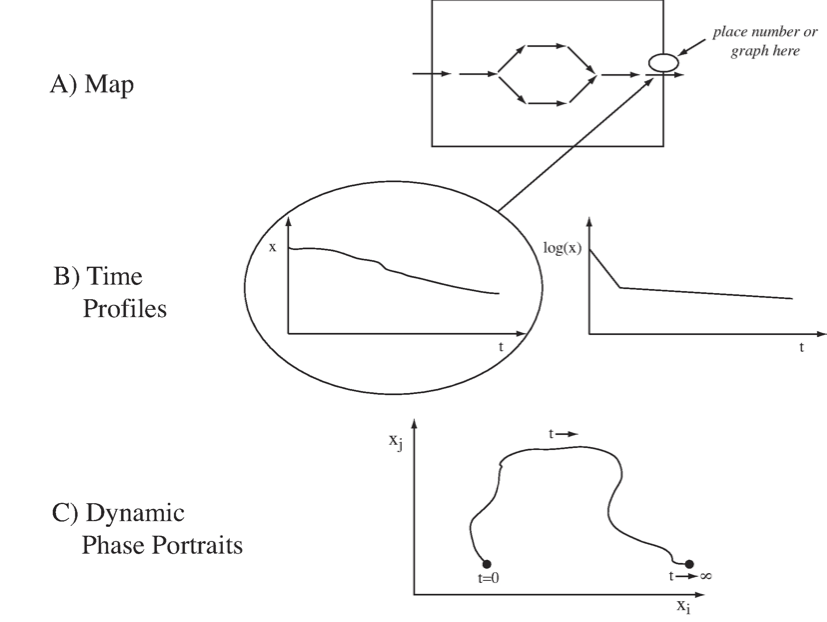

Supplement: S1 File — The latest version of the MASSpy software can be found at https://github.com/SBRG/MASSpy. (ZIP) [file pcbi.1008208.s003.zip › MASSpy-0.1.1/docs/education/sb2/images/Ch2/Figure-2-3.png]

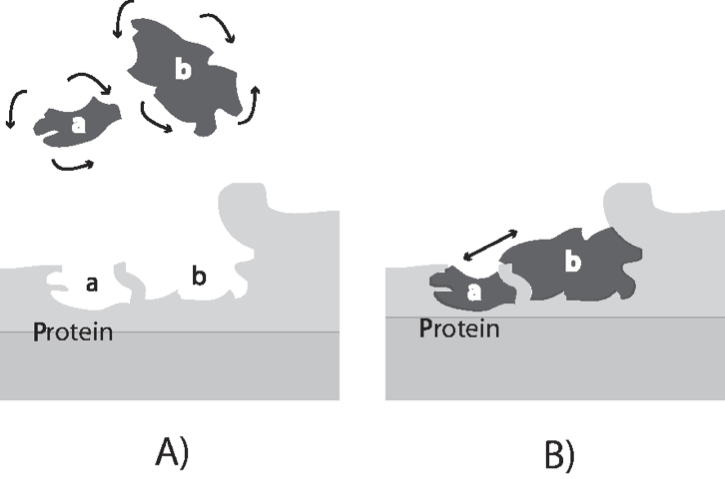

Supplement: S1 File — The latest version of the MASSpy software can be found at https://github.com/SBRG/MASSpy. (ZIP) [file pcbi.1008208.s003.zip › MASSpy-0.1.1/docs/education/sb2/images/Ch2/Figure-2-4.png]

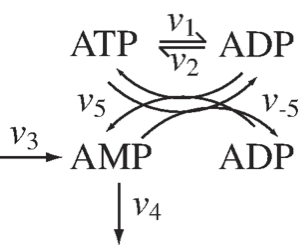

Supplement: S1 File — The latest version of the MASSpy software can be found at https://github.com/SBRG/MASSpy. (ZIP) [file pcbi.1008208.s003.zip › MASSpy-0.1.1/docs/education/sb2/images/Ch2/Figure-2-5.png]

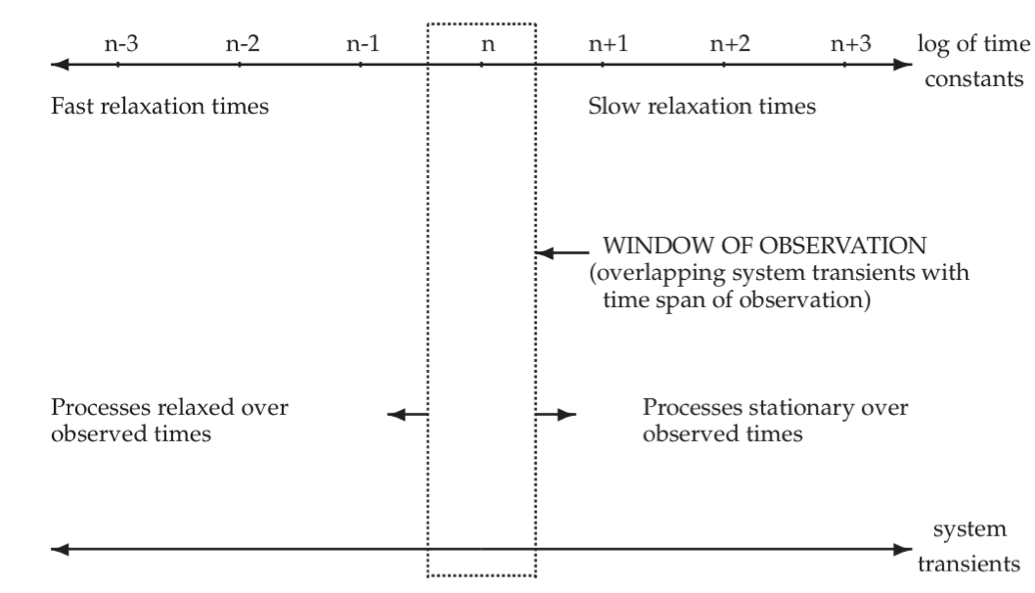

Supplement: S1 File — The latest version of the MASSpy software can be found at https://github.com/SBRG/MASSpy. (ZIP) [file pcbi.1008208.s003.zip › MASSpy-0.1.1/docs/education/sb2/images/Ch2/Figure-2-6.png]

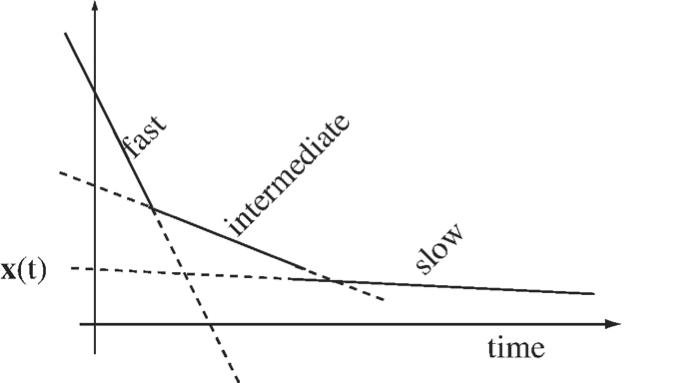

Supplement: S1 File — The latest version of the MASSpy software can be found at https://github.com/SBRG/MASSpy. (ZIP) [file pcbi.1008208.s003.zip › MASSpy-0.1.1/docs/education/sb2/images/Ch2/Figure-2-7.png]

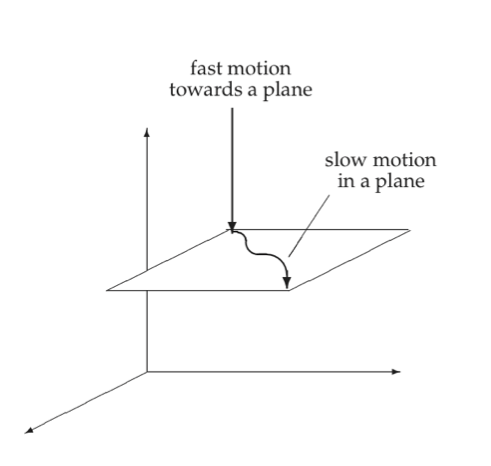

Supplement: S1 File — The latest version of the MASSpy software can be found at https://github.com/SBRG/MASSpy. (ZIP) [file pcbi.1008208.s003.zip › MASSpy-0.1.1/docs/education/sb2/images/Ch2/Figure-2-8.png]

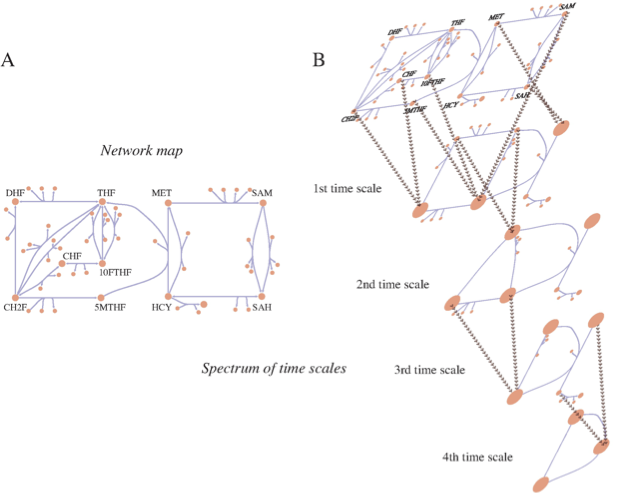

Supplement: S1 File — The latest version of the MASSpy software can be found at https://github.com/SBRG/MASSpy. (ZIP) [file pcbi.1008208.s003.zip › MASSpy-0.1.1/docs/education/sb2/images/Ch2/Figure-2-9.png]

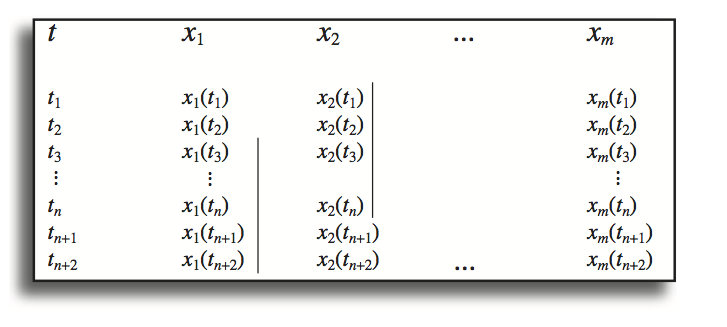

Supplement: S1 File — The latest version of the MASSpy software can be found at https://github.com/SBRG/MASSpy. (ZIP) [file pcbi.1008208.s003.zip › MASSpy-0.1.1/docs/education/sb2/images/Ch3/Figure-3-1.png]

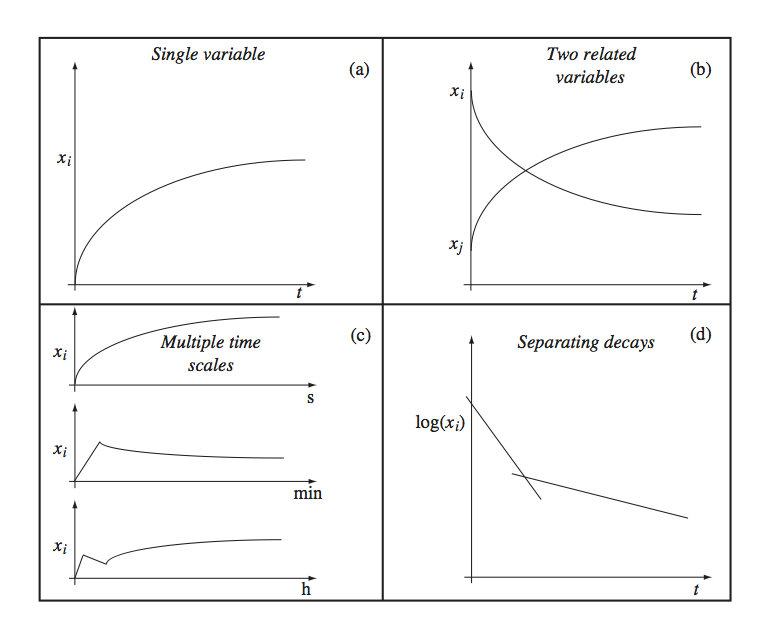

Supplement: S1 File — The latest version of the MASSpy software can be found at https://github.com/SBRG/MASSpy. (ZIP) [file pcbi.1008208.s003.zip › MASSpy-0.1.1/docs/education/sb2/images/Ch3/Figure-3-2.png]

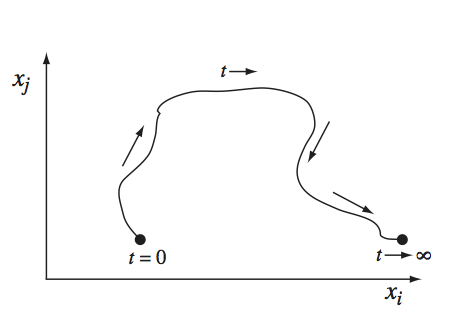

Supplement: S1 File — The latest version of the MASSpy software can be found at https://github.com/SBRG/MASSpy. (ZIP) [file pcbi.1008208.s003.zip › MASSpy-0.1.1/docs/education/sb2/images/Ch3/Figure-3-3.png]

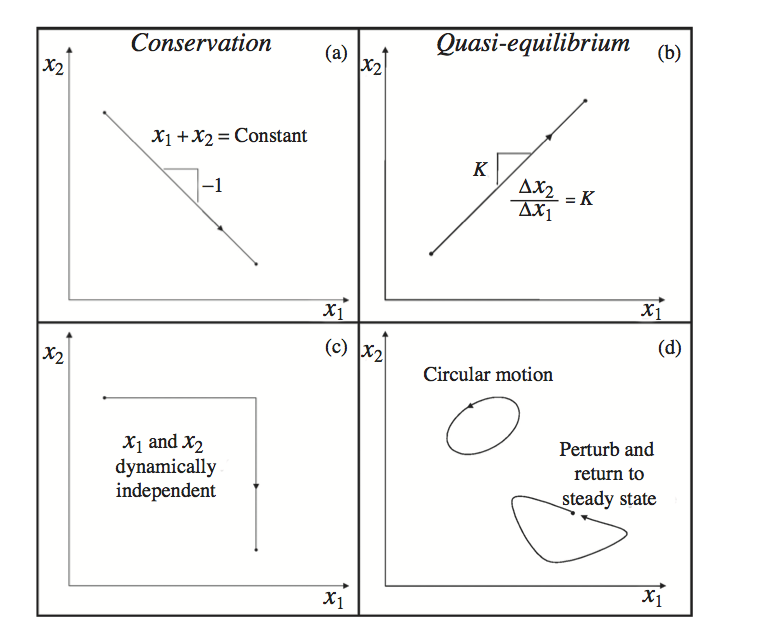

Supplement: S1 File — The latest version of the MASSpy software can be found at https://github.com/SBRG/MASSpy. (ZIP) [file pcbi.1008208.s003.zip › MASSpy-0.1.1/docs/education/sb2/images/Ch3/Figure-3-4.png]

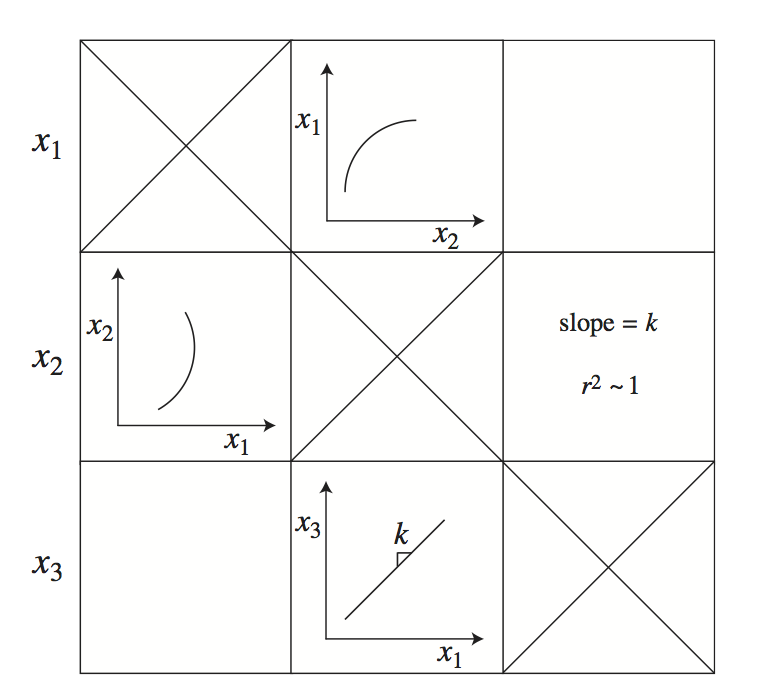

Supplement: S1 File — The latest version of the MASSpy software can be found at https://github.com/SBRG/MASSpy. (ZIP) [file pcbi.1008208.s003.zip › MASSpy-0.1.1/docs/education/sb2/images/Ch3/Figure-3-5.png]

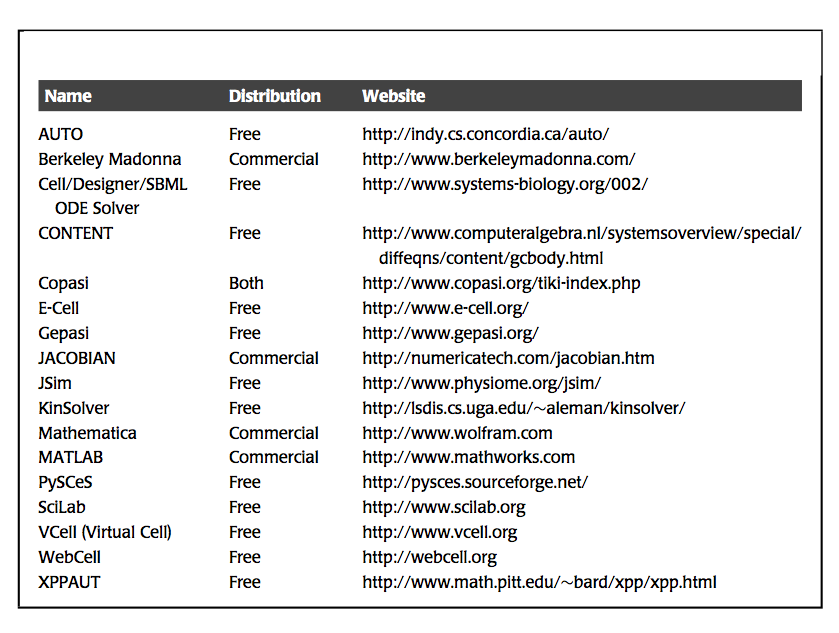

Supplement: S1 File — The latest version of the MASSpy software can be found at https://github.com/SBRG/MASSpy. (ZIP) [file pcbi.1008208.s003.zip › MASSpy-0.1.1/docs/education/sb2/images/Ch3/Table-3-1.png]

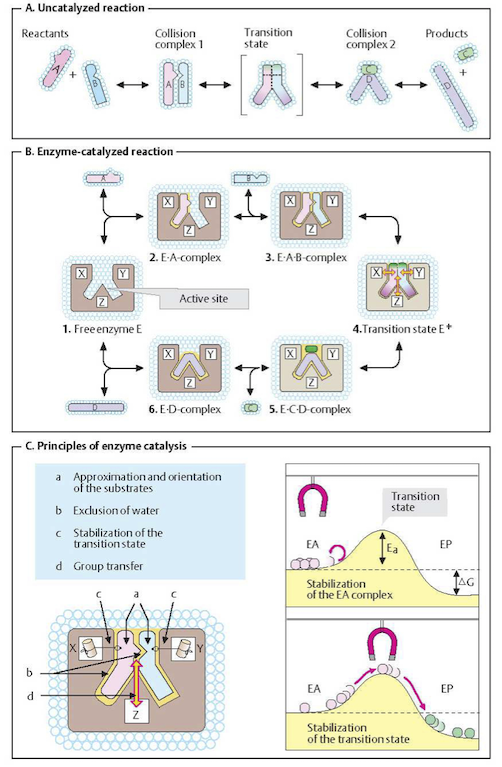

Supplement: S1 File — The latest version of the MASSpy software can be found at https://github.com/SBRG/MASSpy. (ZIP) [file pcbi.1008208.s003.zip › MASSpy-0.1.1/docs/education/sb2/images/Ch5/Figure-5-1.png]

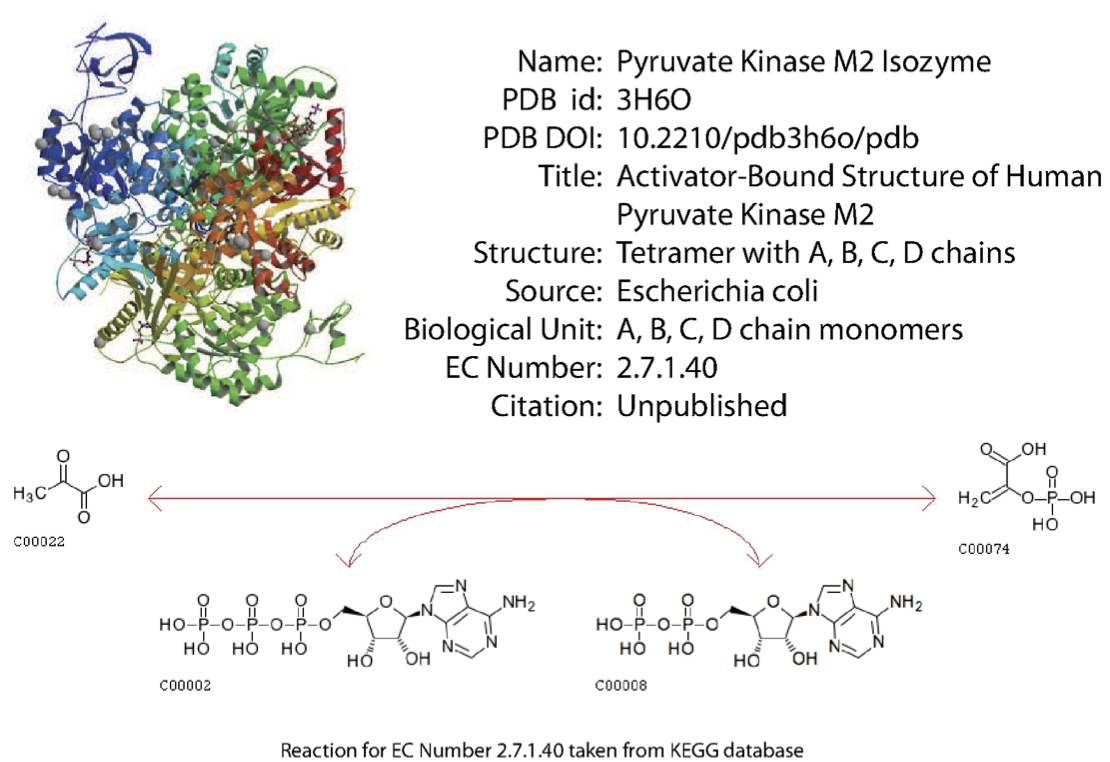

Supplement: S1 File — The latest version of the MASSpy software can be found at https://github.com/SBRG/MASSpy. (ZIP) [file pcbi.1008208.s003.zip › MASSpy-0.1.1/docs/education/sb2/images/Ch5/Figure-5-2.png]

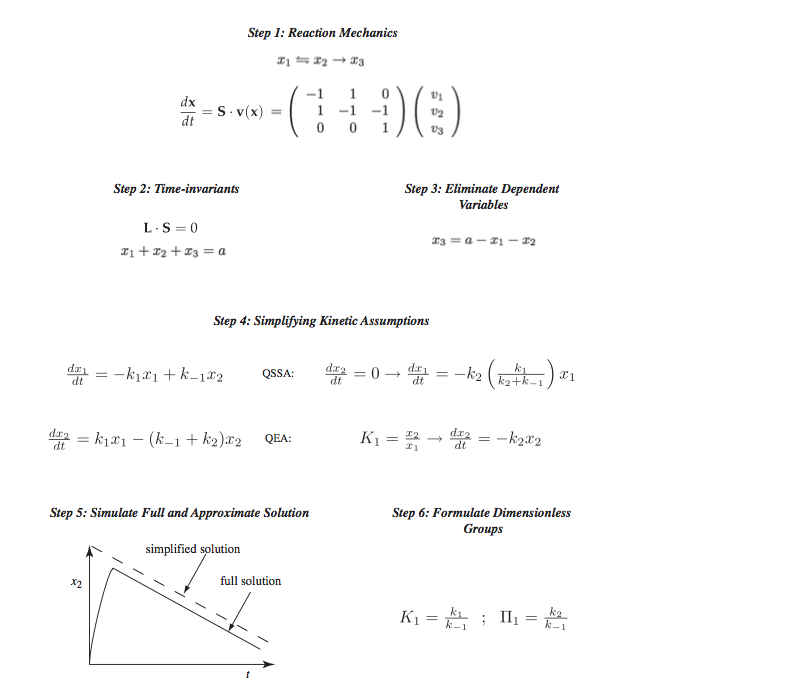

Supplement: S1 File — The latest version of the MASSpy software can be found at https://github.com/SBRG/MASSpy. (ZIP) [file pcbi.1008208.s003.zip › MASSpy-0.1.1/docs/education/sb2/images/Ch5/Figure-5-3.png]

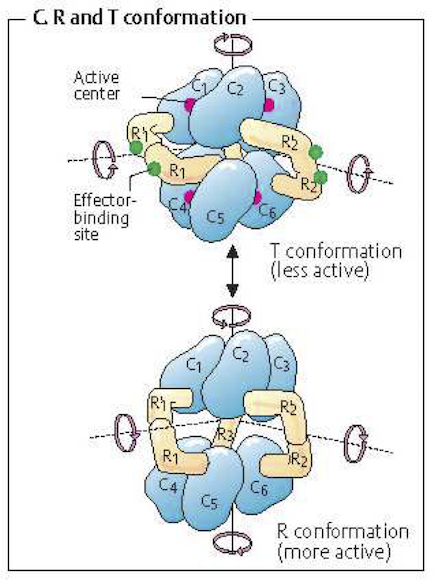

Supplement: S1 File — The latest version of the MASSpy software can be found at https://github.com/SBRG/MASSpy. (ZIP) [file pcbi.1008208.s003.zip › MASSpy-0.1.1/docs/education/sb2/images/Ch5/Figure-5-6.png]

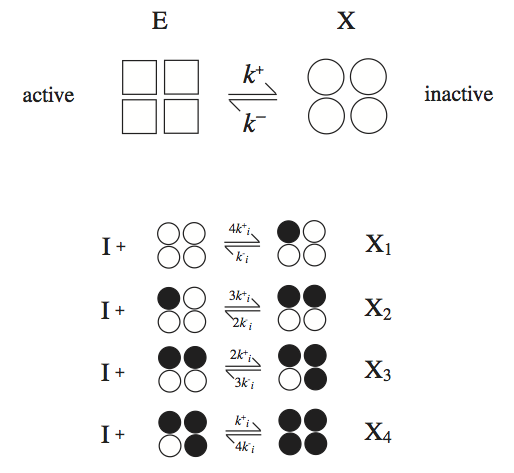

Supplement: S1 File — The latest version of the MASSpy software can be found at https://github.com/SBRG/MASSpy. (ZIP) [file pcbi.1008208.s003.zip › MASSpy-0.1.1/docs/education/sb2/images/Ch5/Figure-5-8.png]

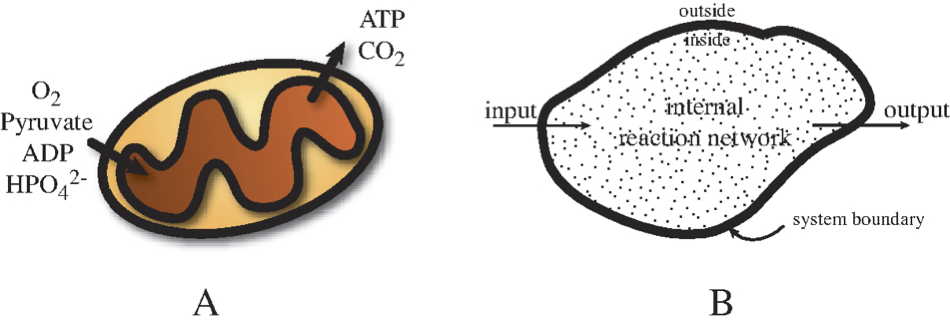

Supplement: S1 File — The latest version of the MASSpy software can be found at https://github.com/SBRG/MASSpy. (ZIP) [file pcbi.1008208.s003.zip › MASSpy-0.1.1/docs/education/sb2/images/Ch6/Figure-6-1.png]

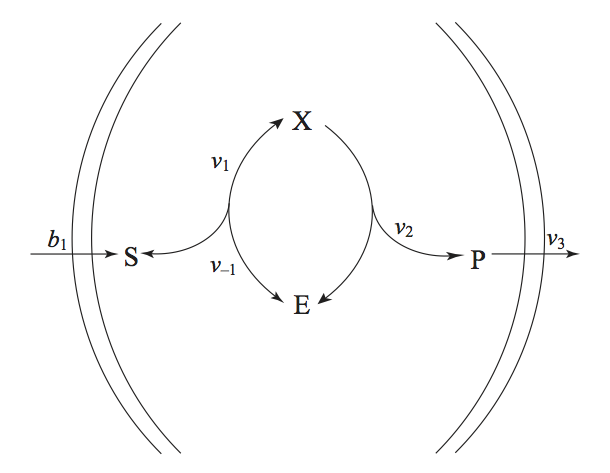

Supplement: S1 File — The latest version of the MASSpy software can be found at https://github.com/SBRG/MASSpy. (ZIP) [file pcbi.1008208.s003.zip › MASSpy-0.1.1/docs/education/sb2/images/Ch6/Figure-6-5.png]

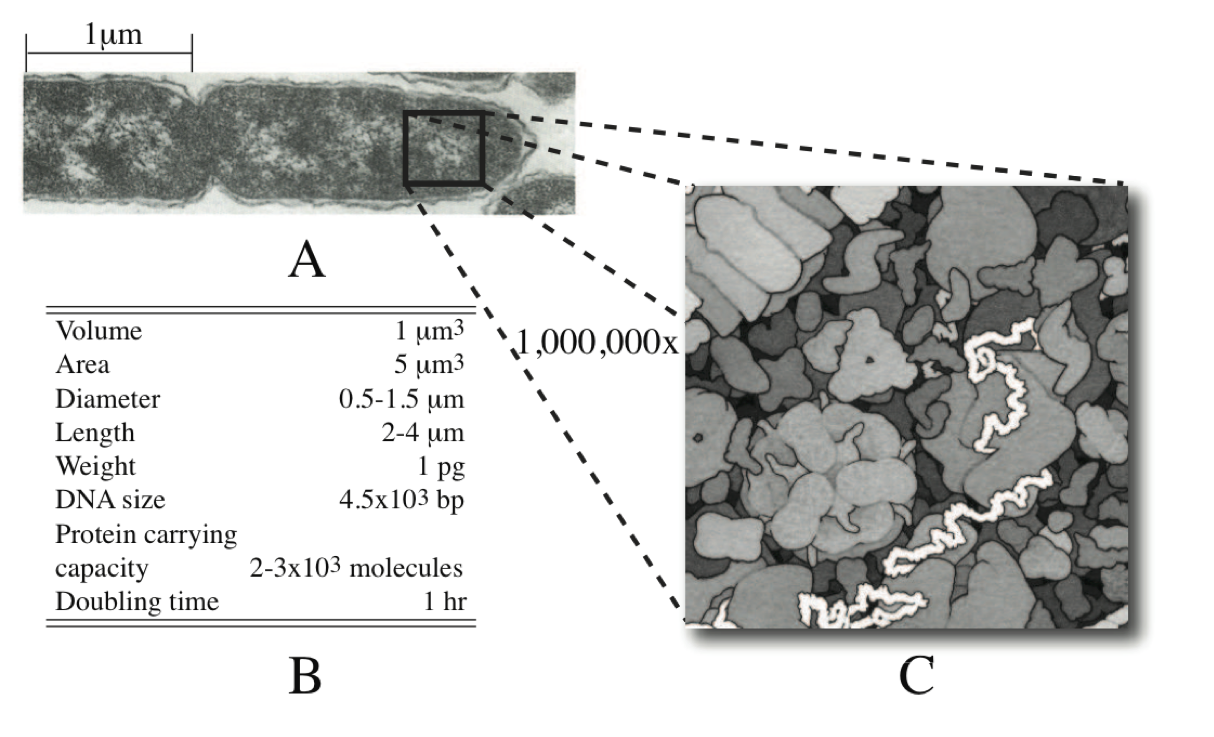

Supplement: S1 File — The latest version of the MASSpy software can be found at https://github.com/SBRG/MASSpy. (ZIP) [file pcbi.1008208.s003.zip › MASSpy-0.1.1/docs/education/sb2/images/Ch7/Figure-7-1.png]

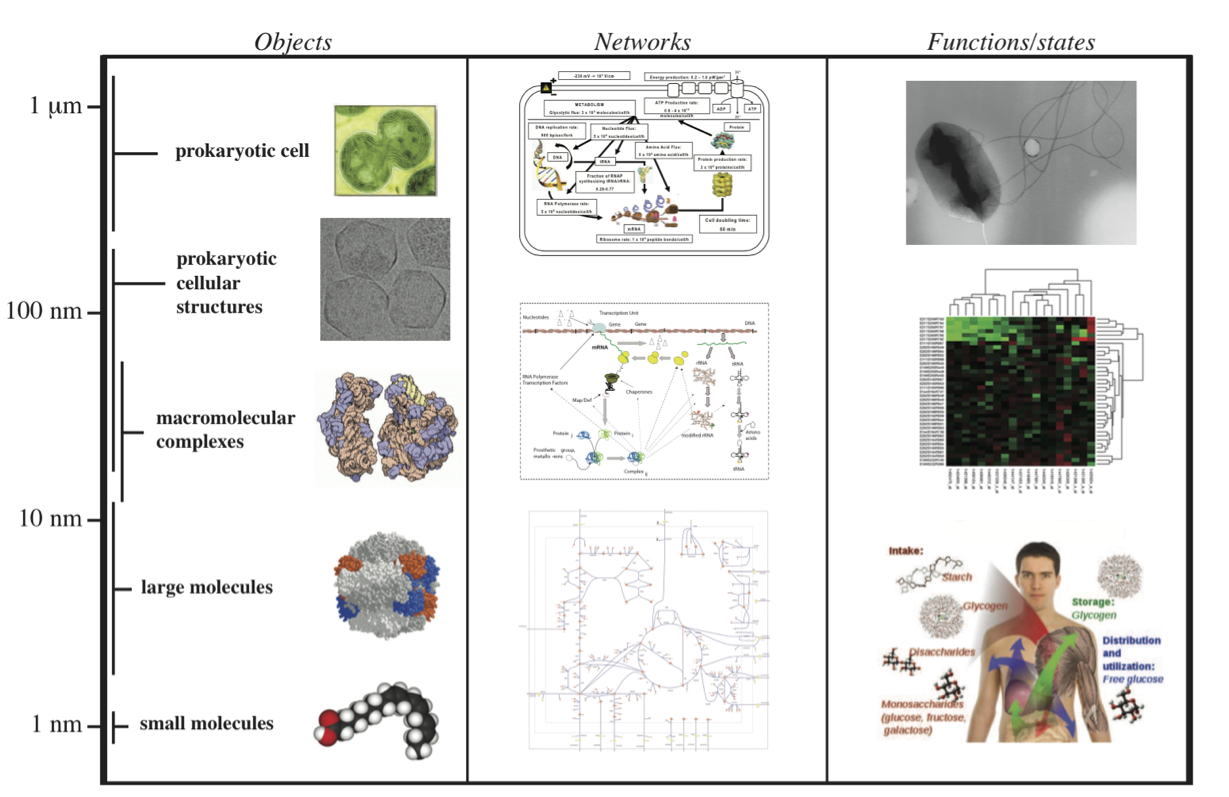

Supplement: S1 File — The latest version of the MASSpy software can be found at https://github.com/SBRG/MASSpy. (ZIP) [file pcbi.1008208.s003.zip › MASSpy-0.1.1/docs/education/sb2/images/Ch7/Figure-7-2.png]

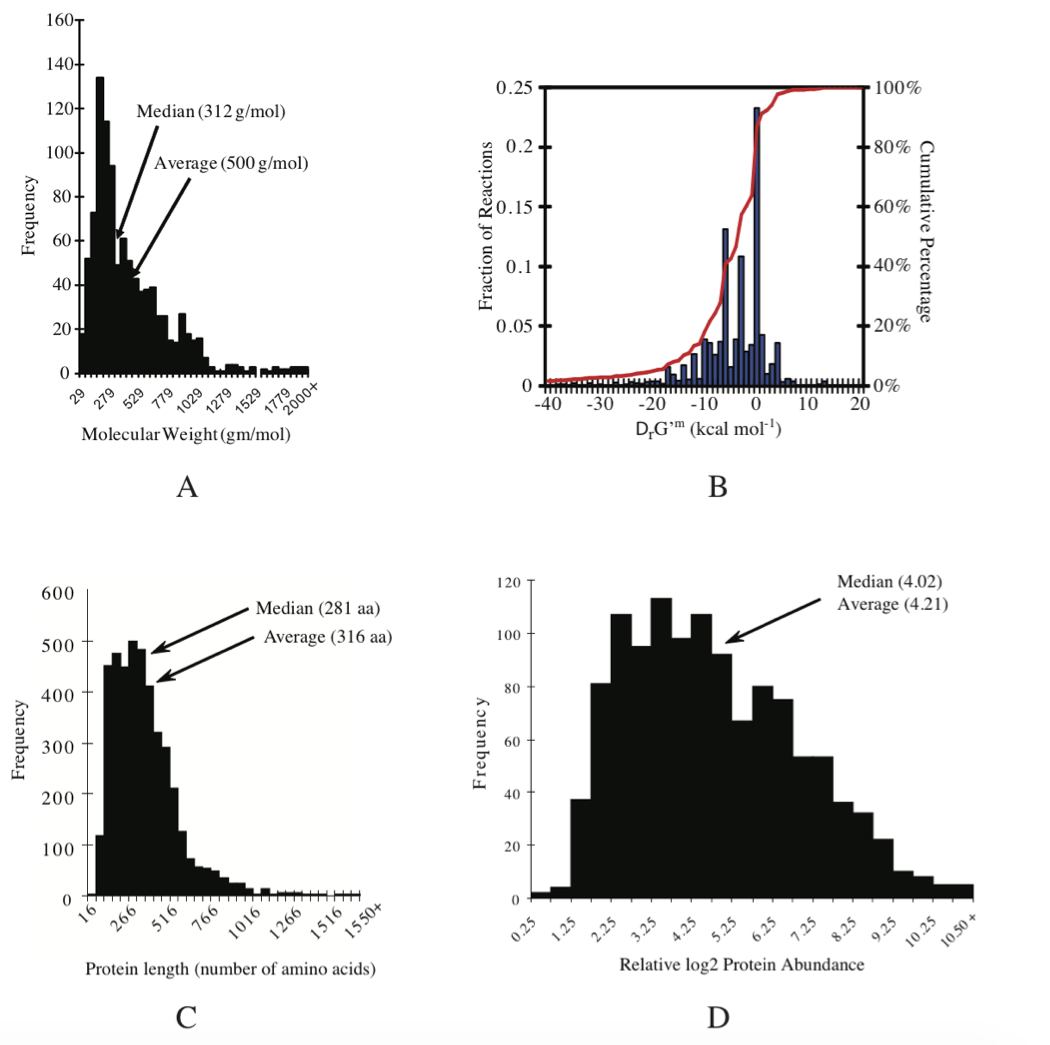

Supplement: S1 File — The latest version of the MASSpy software can be found at https://github.com/SBRG/MASSpy. (ZIP) [file pcbi.1008208.s003.zip › MASSpy-0.1.1/docs/education/sb2/images/Ch7/Figure-7-3.png]

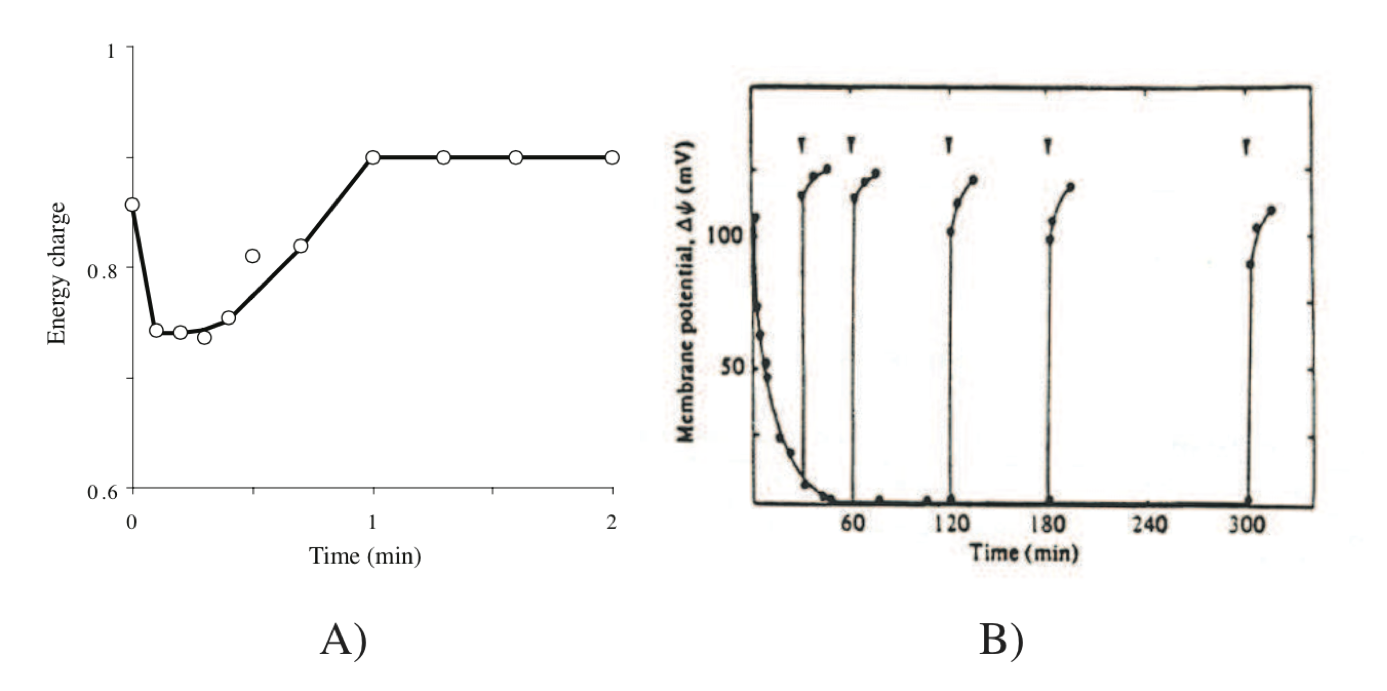

Supplement: S1 File — The latest version of the MASSpy software can be found at https://github.com/SBRG/MASSpy. (ZIP) [file pcbi.1008208.s003.zip › MASSpy-0.1.1/docs/education/sb2/images/Ch7/Figure-7-4.png]

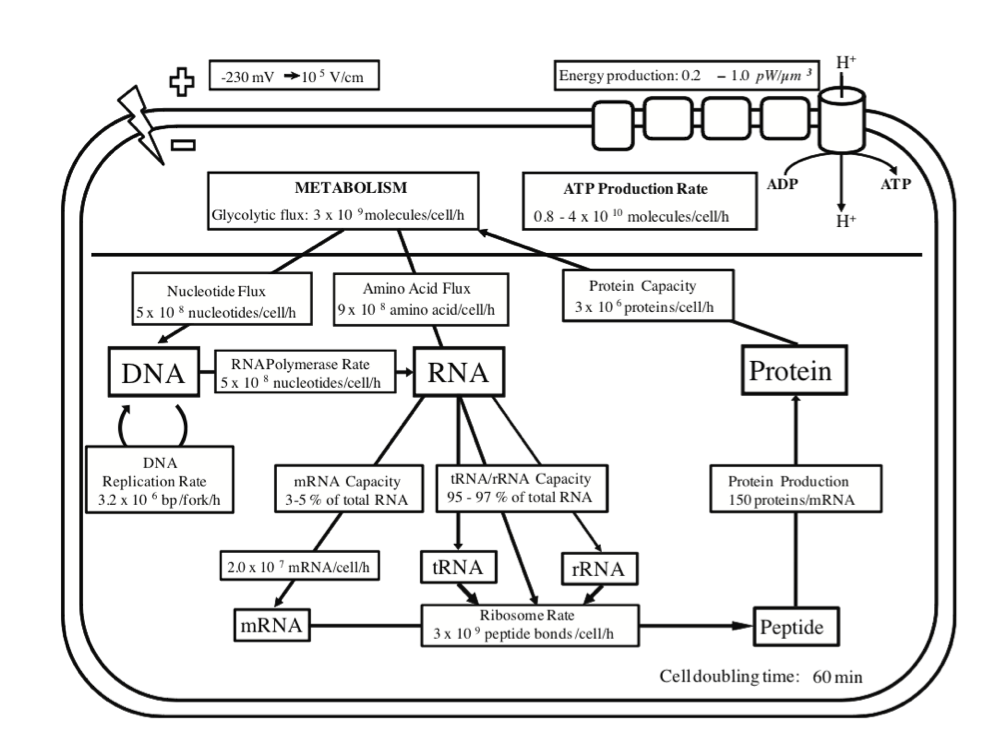

Supplement: S1 File — The latest version of the MASSpy software can be found at https://github.com/SBRG/MASSpy. (ZIP) [file pcbi.1008208.s003.zip › MASSpy-0.1.1/docs/education/sb2/images/Ch7/Figure-7-5.png]

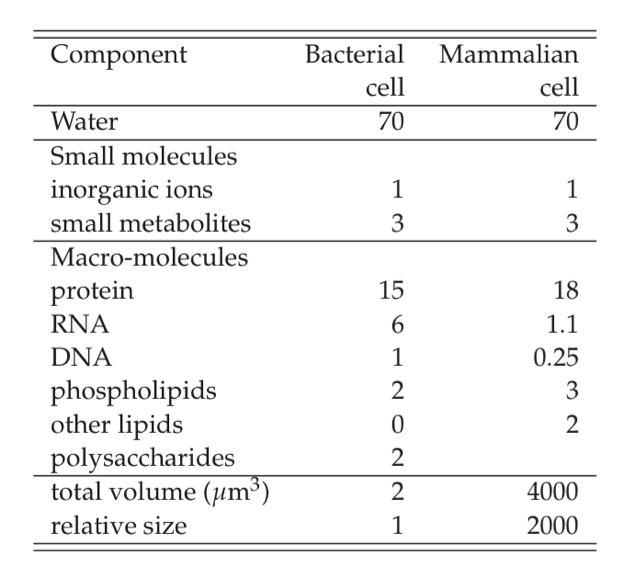

Supplement: S1 File — The latest version of the MASSpy software can be found at https://github.com/SBRG/MASSpy. (ZIP) [file pcbi.1008208.s003.zip › MASSpy-0.1.1/docs/education/sb2/images/Ch7/Table-7-1.png]

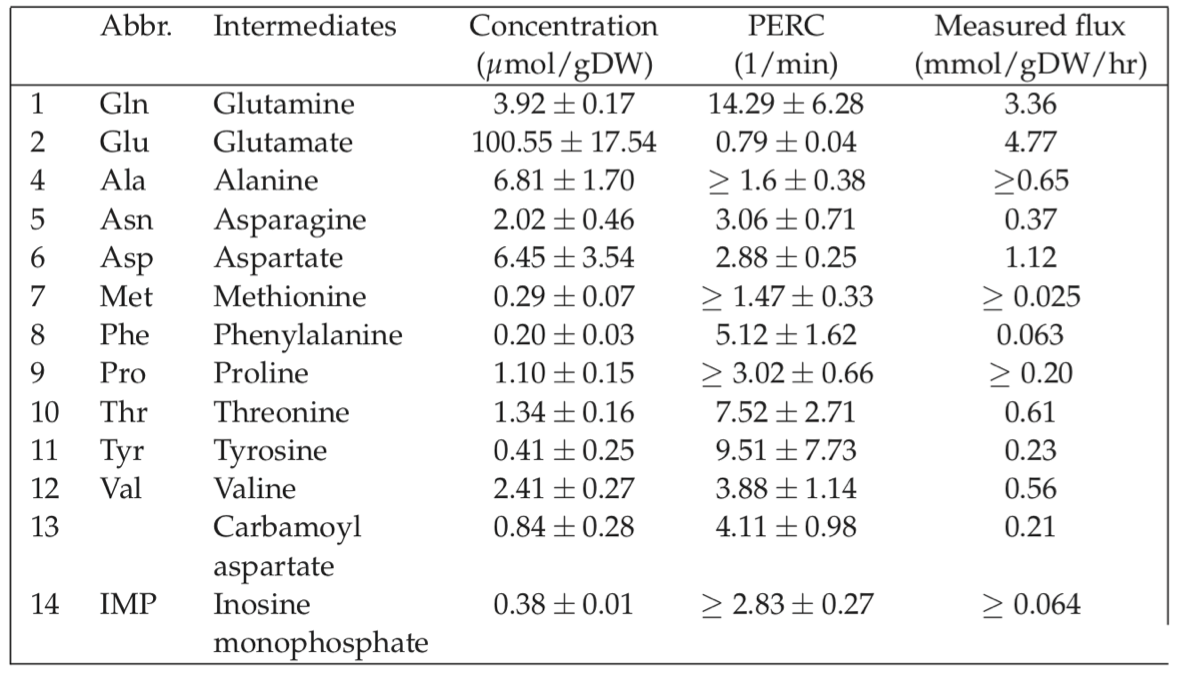

Supplement: S1 File — The latest version of the MASSpy software can be found at https://github.com/SBRG/MASSpy. (ZIP) [file pcbi.1008208.s003.zip › MASSpy-0.1.1/docs/education/sb2/images/Ch7/Table-7-2.png]

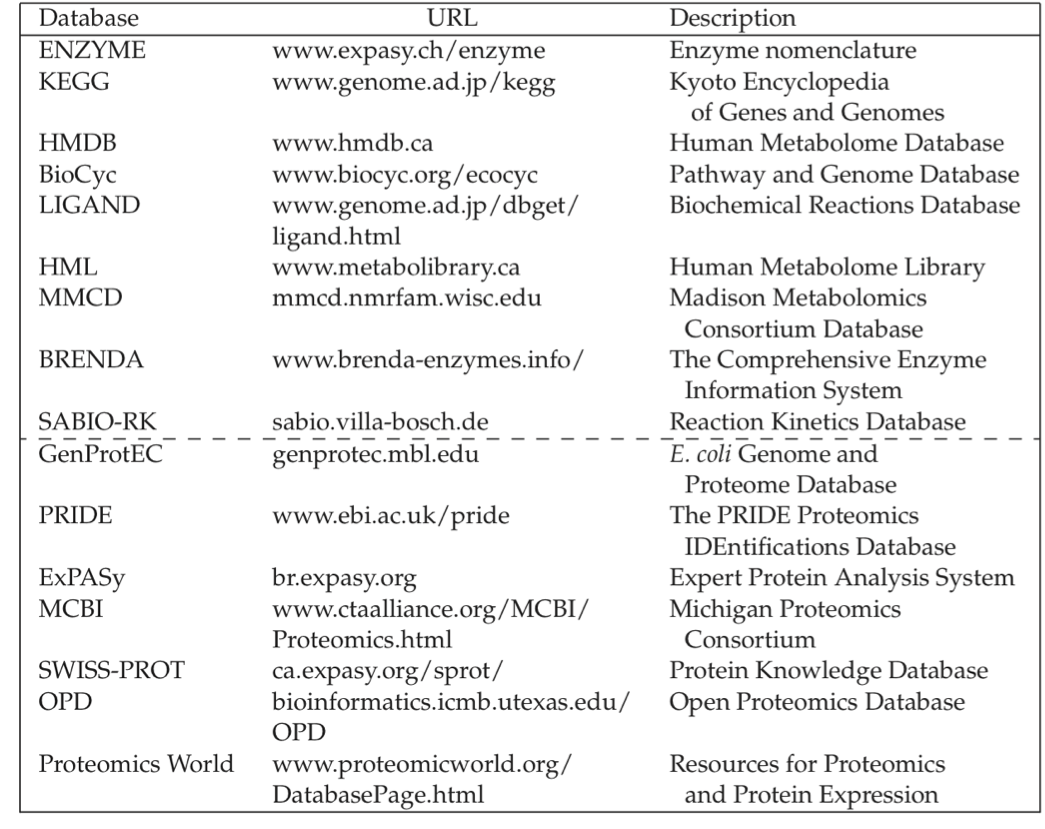

Supplement: S1 File — The latest version of the MASSpy software can be found at https://github.com/SBRG/MASSpy. (ZIP) [file pcbi.1008208.s003.zip › MASSpy-0.1.1/docs/education/sb2/images/Ch7/Table-7-3.png]

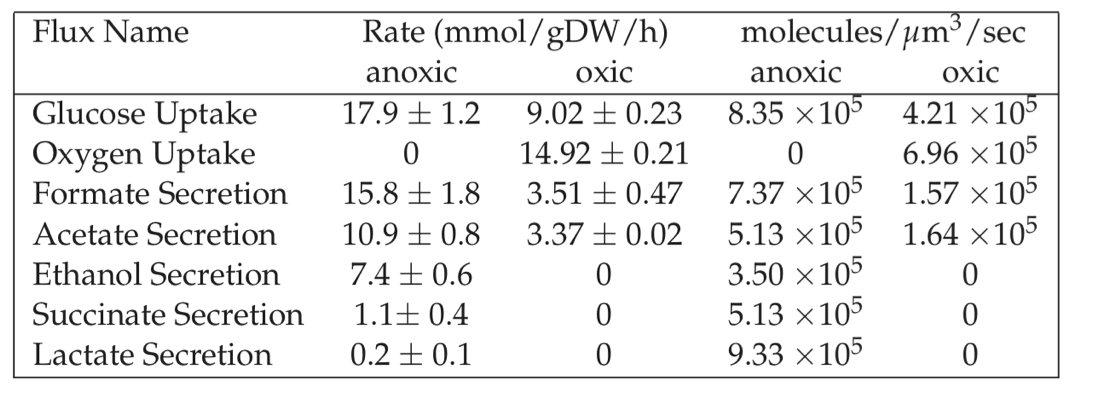

Supplement: S1 File — The latest version of the MASSpy software can be found at https://github.com/SBRG/MASSpy. (ZIP) [file pcbi.1008208.s003.zip › MASSpy-0.1.1/docs/education/sb2/images/Ch7/Table-7-4.png]

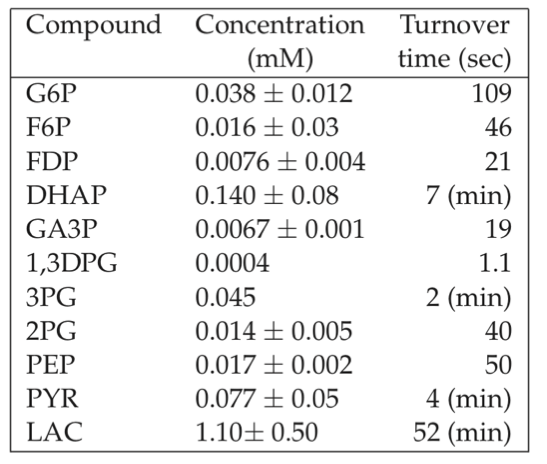

Supplement: S1 File — The latest version of the MASSpy software can be found at https://github.com/SBRG/MASSpy. (ZIP) [file pcbi.1008208.s003.zip › MASSpy-0.1.1/docs/education/sb2/images/Ch7/Table-7-5.png]

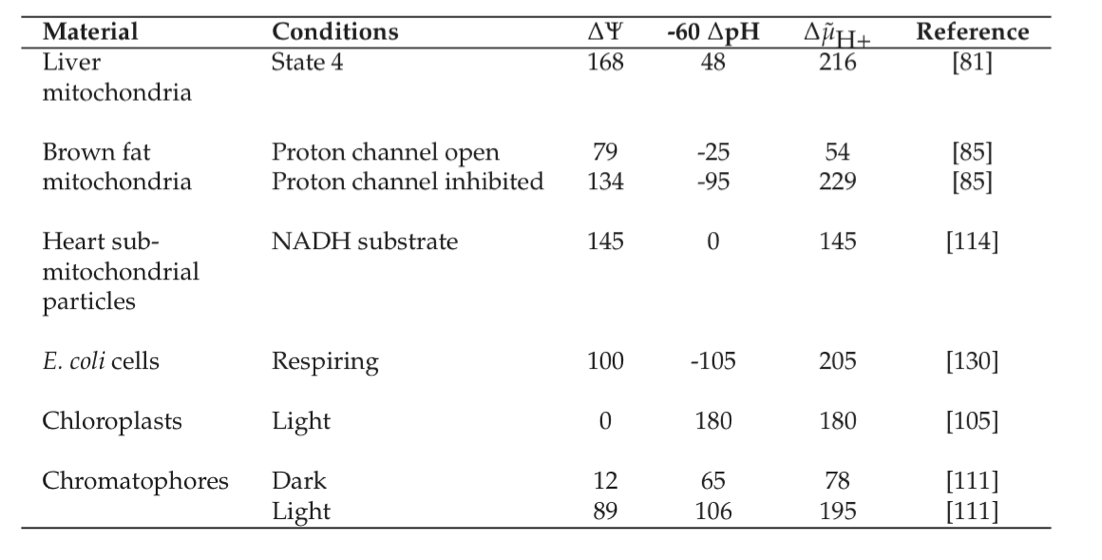

Supplement: S1 File — The latest version of the MASSpy software can be found at https://github.com/SBRG/MASSpy. (ZIP) [file pcbi.1008208.s003.zip › MASSpy-0.1.1/docs/education/sb2/images/Ch7/Table-7-6.png]

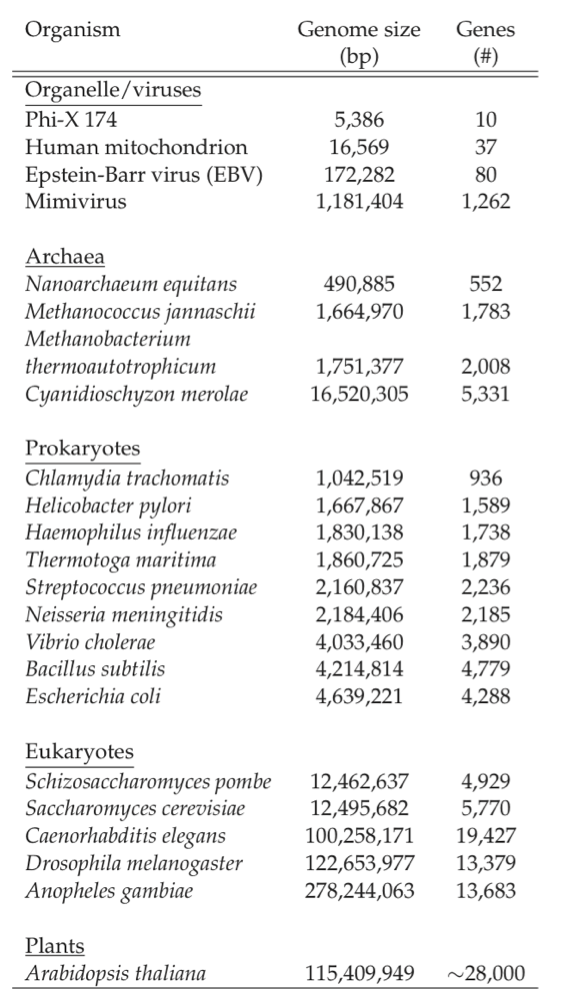

Supplement: S1 File — The latest version of the MASSpy software can be found at https://github.com/SBRG/MASSpy. (ZIP) [file pcbi.1008208.s003.zip › MASSpy-0.1.1/docs/education/sb2/images/Ch7/Table-7-7.png]

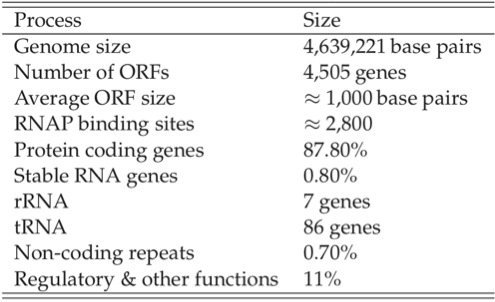

Supplement: S1 File — The latest version of the MASSpy software can be found at https://github.com/SBRG/MASSpy. (ZIP) [file pcbi.1008208.s003.zip › MASSpy-0.1.1/docs/education/sb2/images/Ch7/Table-7-8.png]

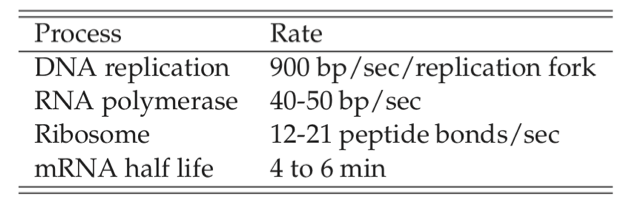

Supplement: S1 File — The latest version of the MASSpy software can be found at https://github.com/SBRG/MASSpy. (ZIP) [file pcbi.1008208.s003.zip › MASSpy-0.1.1/docs/education/sb2/images/Ch7/Table-7-9.png]

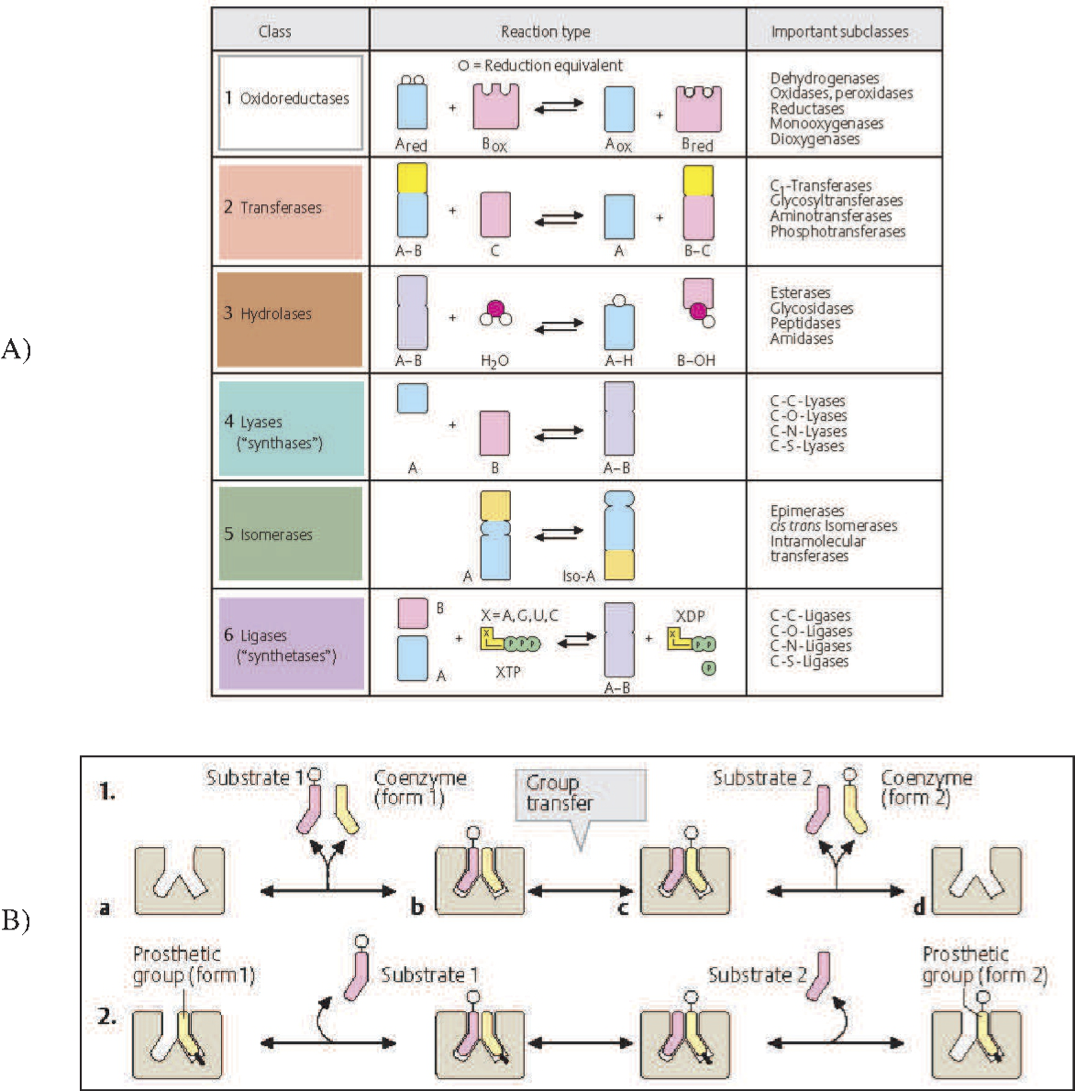

Supplement: S1 File — The latest version of the MASSpy software can be found at https://github.com/SBRG/MASSpy. (ZIP) [file pcbi.1008208.s003.zip › MASSpy-0.1.1/docs/education/sb2/images/Ch8/Figure-8-1.png]

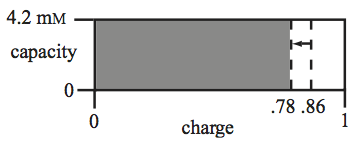

Supplement: S1 File — The latest version of the MASSpy software can be found at https://github.com/SBRG/MASSpy. (ZIP) [file pcbi.1008208.s003.zip › MASSpy-0.1.1/docs/education/sb2/images/Ch8/Figure-8-10.png]

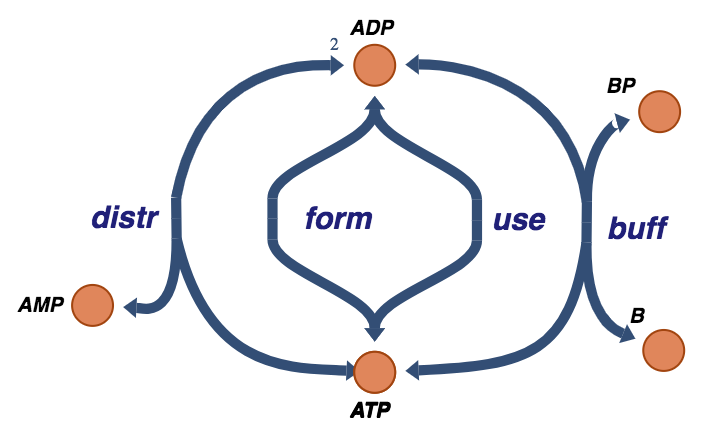

Supplement: S1 File — The latest version of the MASSpy software can be found at https://github.com/SBRG/MASSpy. (ZIP) [file pcbi.1008208.s003.zip › MASSpy-0.1.1/docs/education/sb2/images/Ch8/Figure-8-11.png]

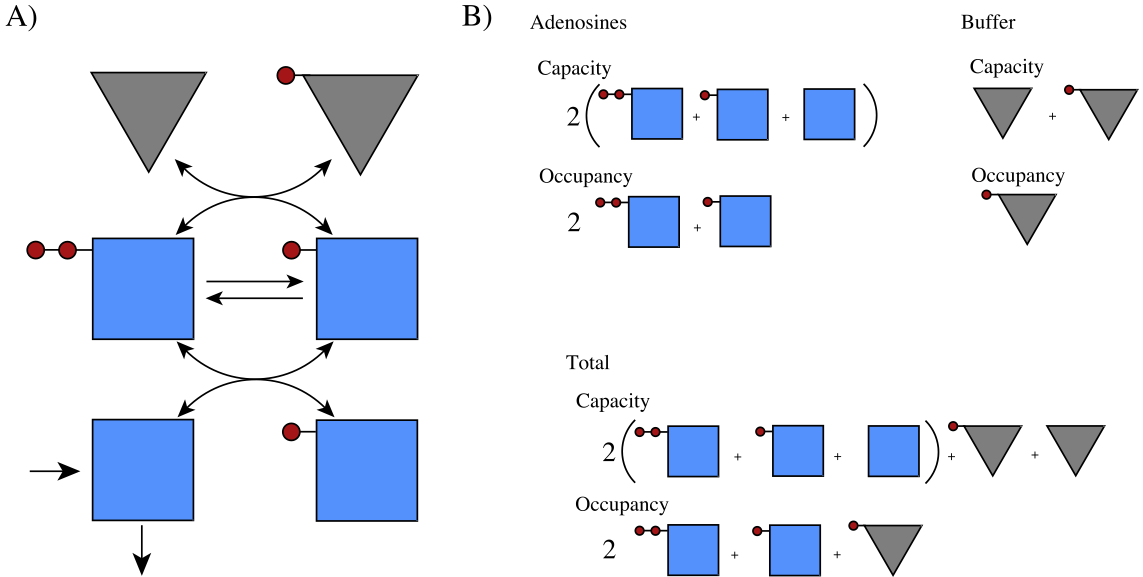

Supplement: S1 File — The latest version of the MASSpy software can be found at https://github.com/SBRG/MASSpy. (ZIP) [file pcbi.1008208.s003.zip › MASSpy-0.1.1/docs/education/sb2/images/Ch8/Figure-8-13.png]

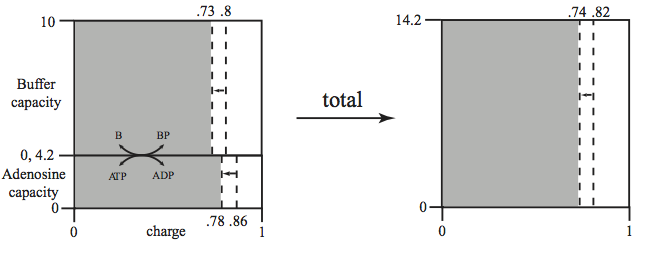

Supplement: S1 File — The latest version of the MASSpy software can be found at https://github.com/SBRG/MASSpy. (ZIP) [file pcbi.1008208.s003.zip › MASSpy-0.1.1/docs/education/sb2/images/Ch8/Figure-8-14.png]

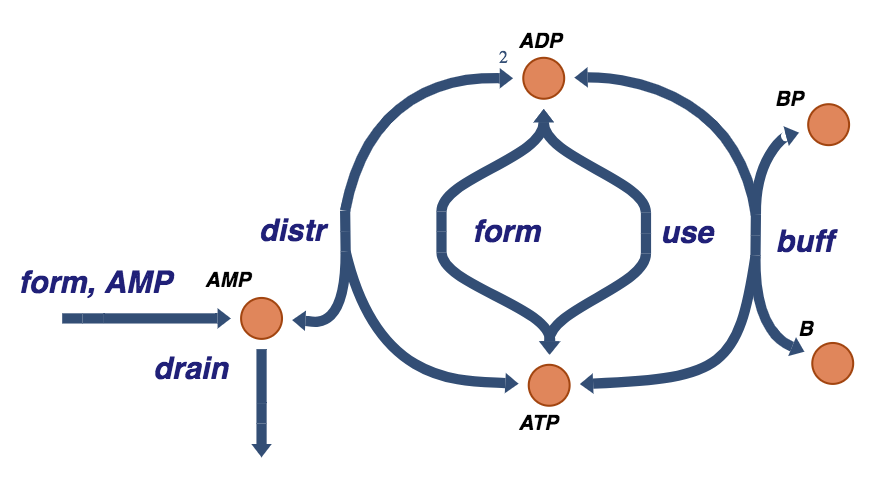

Supplement: S1 File — The latest version of the MASSpy software can be found at https://github.com/SBRG/MASSpy. (ZIP) [file pcbi.1008208.s003.zip › MASSpy-0.1.1/docs/education/sb2/images/Ch8/Figure-8-16.png]

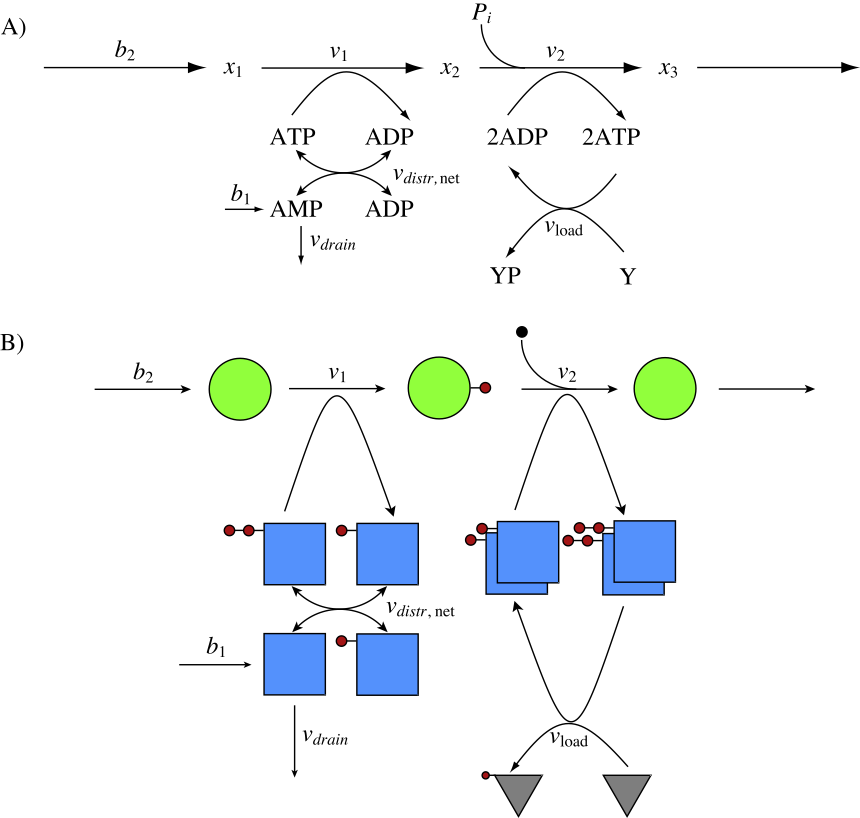

Supplement: S1 File — The latest version of the MASSpy software can be found at https://github.com/SBRG/MASSpy. (ZIP) [file pcbi.1008208.s003.zip › MASSpy-0.1.1/docs/education/sb2/images/Ch8/Figure-8-19.png]

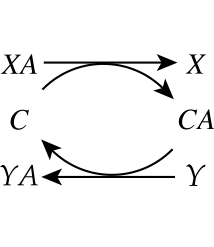

Supplement: S1 File — The latest version of the MASSpy software can be found at https://github.com/SBRG/MASSpy. (ZIP) [file pcbi.1008208.s003.zip › MASSpy-0.1.1/docs/education/sb2/images/Ch8/Figure-8-2.png]

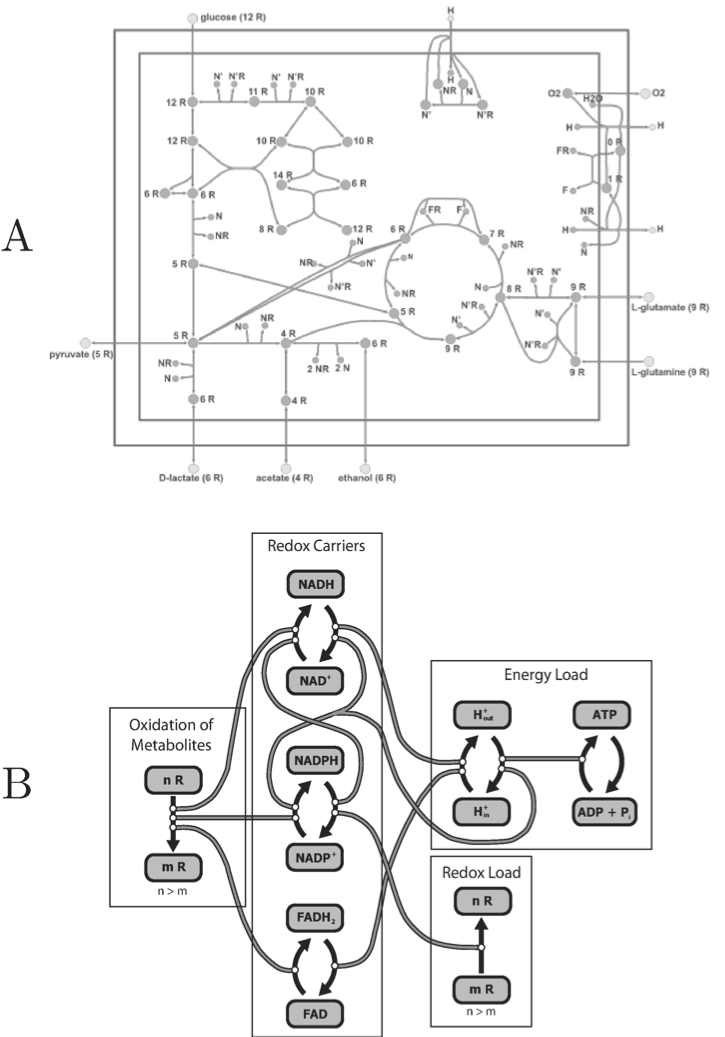

Supplement: S1 File — The latest version of the MASSpy software can be found at https://github.com/SBRG/MASSpy. (ZIP) [file pcbi.1008208.s003.zip › MASSpy-0.1.1/docs/education/sb2/images/Ch8/Figure-8-3.png]

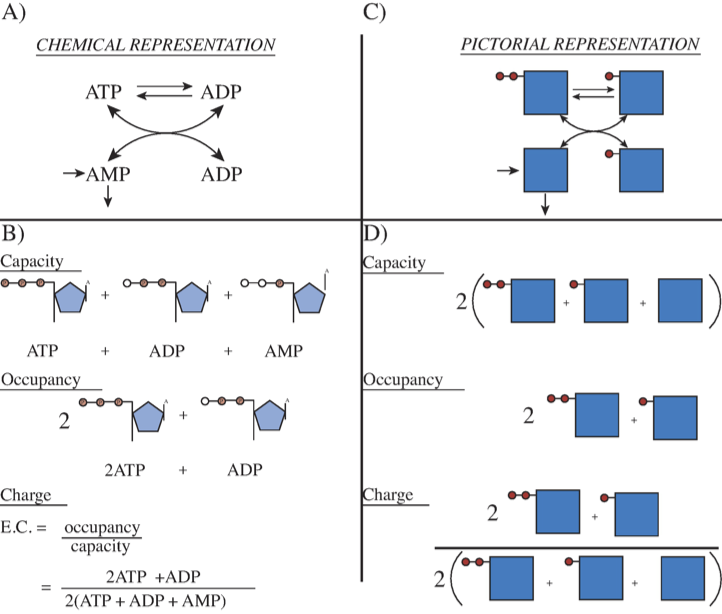

Supplement: S1 File — The latest version of the MASSpy software can be found at https://github.com/SBRG/MASSpy. (ZIP) [file pcbi.1008208.s003.zip › MASSpy-0.1.1/docs/education/sb2/images/Ch8/Figure-8-4.png]

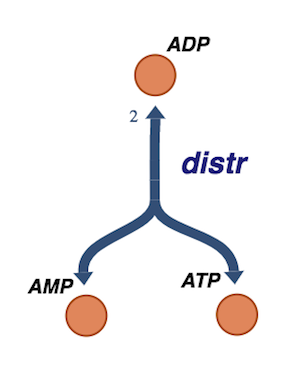

Supplement: S1 File — The latest version of the MASSpy software can be found at https://github.com/SBRG/MASSpy. (ZIP) [file pcbi.1008208.s003.zip › MASSpy-0.1.1/docs/education/sb2/images/Ch8/Figure-8-5.png]

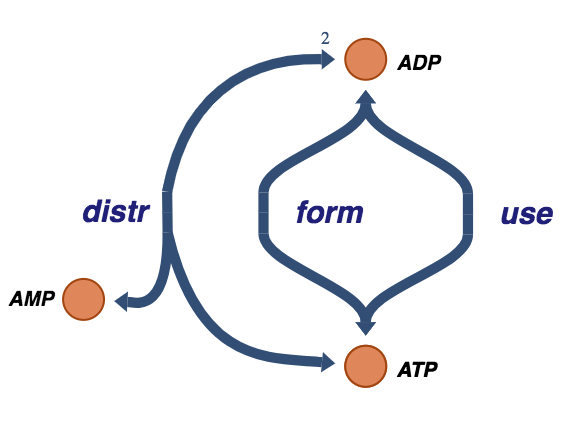

Supplement: S1 File — The latest version of the MASSpy software can be found at https://github.com/SBRG/MASSpy. (ZIP) [file pcbi.1008208.s003.zip › MASSpy-0.1.1/docs/education/sb2/images/Ch8/Figure-8-6.png]

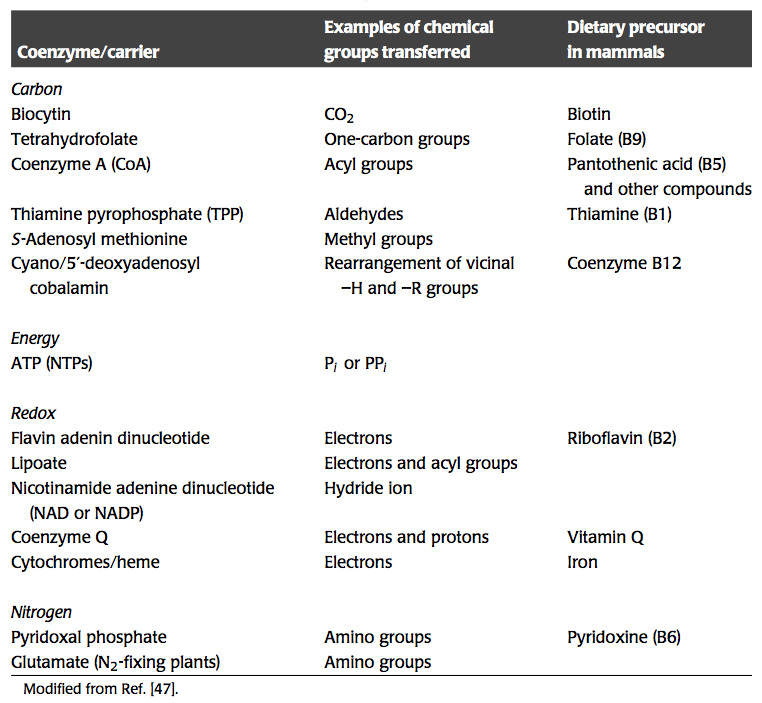

Supplement: S1 File — The latest version of the MASSpy software can be found at https://github.com/SBRG/MASSpy. (ZIP) [file pcbi.1008208.s003.zip › MASSpy-0.1.1/docs/education/sb2/images/Ch8/Table-8-1.png]

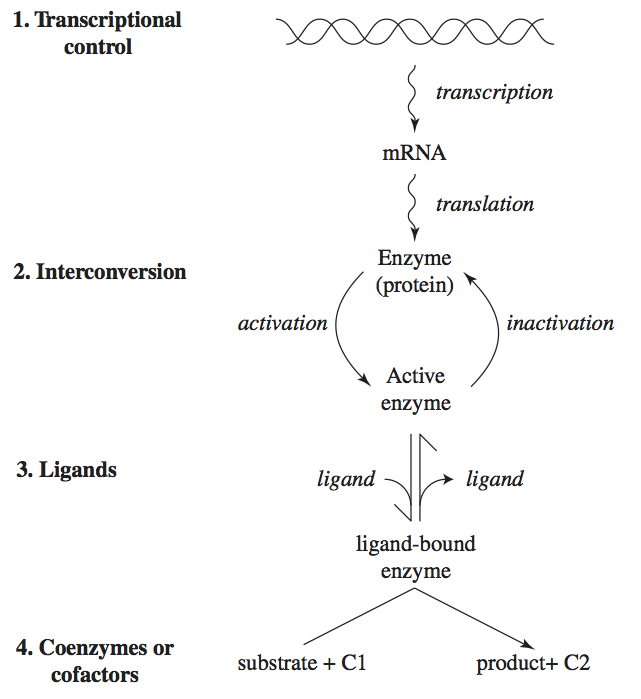

Supplement: S1 File — The latest version of the MASSpy software can be found at https://github.com/SBRG/MASSpy. (ZIP) [file pcbi.1008208.s003.zip › MASSpy-0.1.1/docs/education/sb2/images/Ch9/Figure-9-1.png]

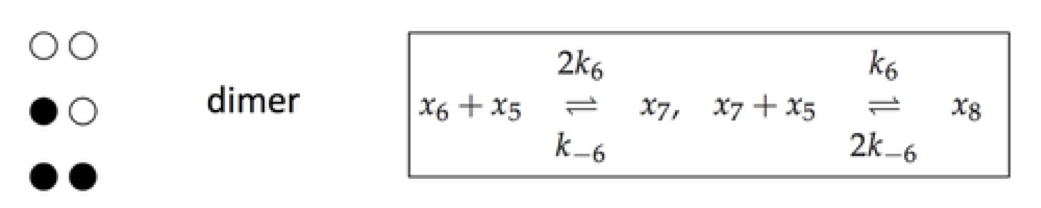

Supplement: S1 File — The latest version of the MASSpy software can be found at https://github.com/SBRG/MASSpy. (ZIP) [file pcbi.1008208.s003.zip › MASSpy-0.1.1/docs/education/sb2/images/Ch9/Figure-9-11.png]

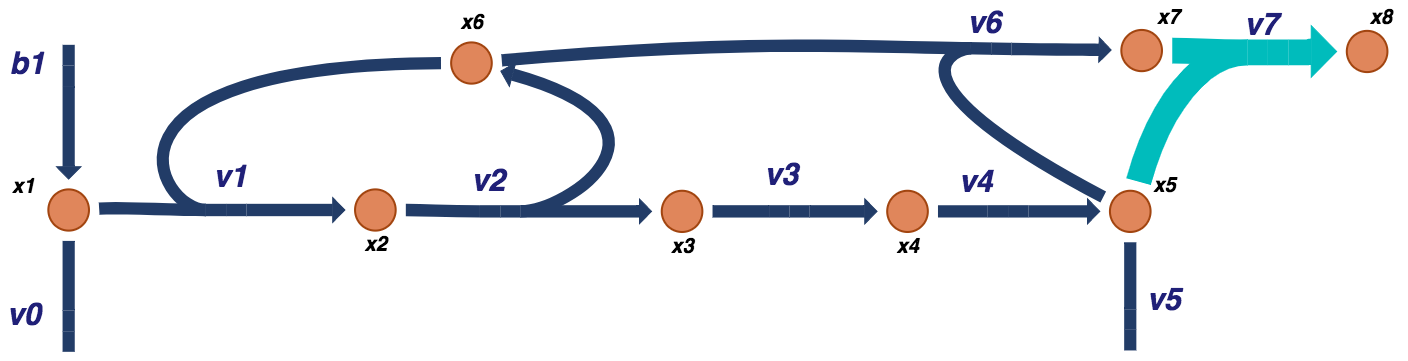

Supplement: S1 File — The latest version of the MASSpy software can be found at https://github.com/SBRG/MASSpy. (ZIP) [file pcbi.1008208.s003.zip › MASSpy-0.1.1/docs/education/sb2/images/Ch9/Figure-9-12.png]

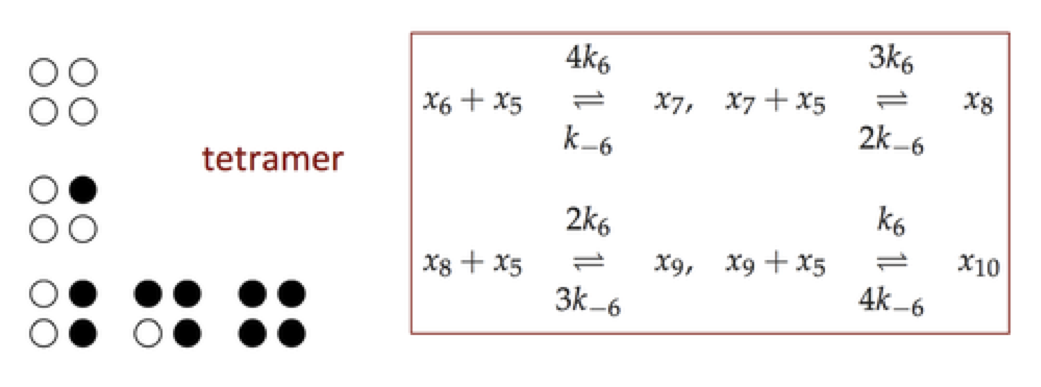

Supplement: S1 File — The latest version of the MASSpy software can be found at https://github.com/SBRG/MASSpy. (ZIP) [file pcbi.1008208.s003.zip › MASSpy-0.1.1/docs/education/sb2/images/Ch9/Figure-9-17.png]

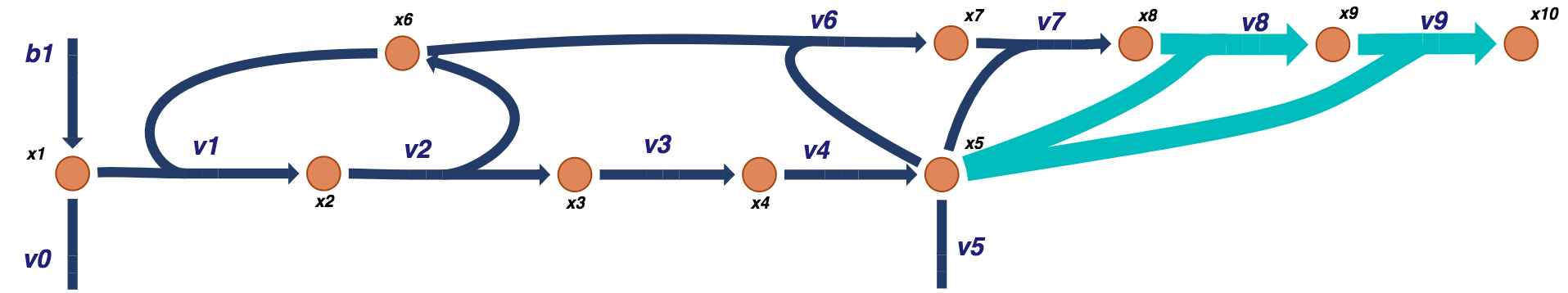

Supplement: S1 File — The latest version of the MASSpy software can be found at https://github.com/SBRG/MASSpy. (ZIP) [file pcbi.1008208.s003.zip › MASSpy-0.1.1/docs/education/sb2/images/Ch9/Figure-9-18.png]

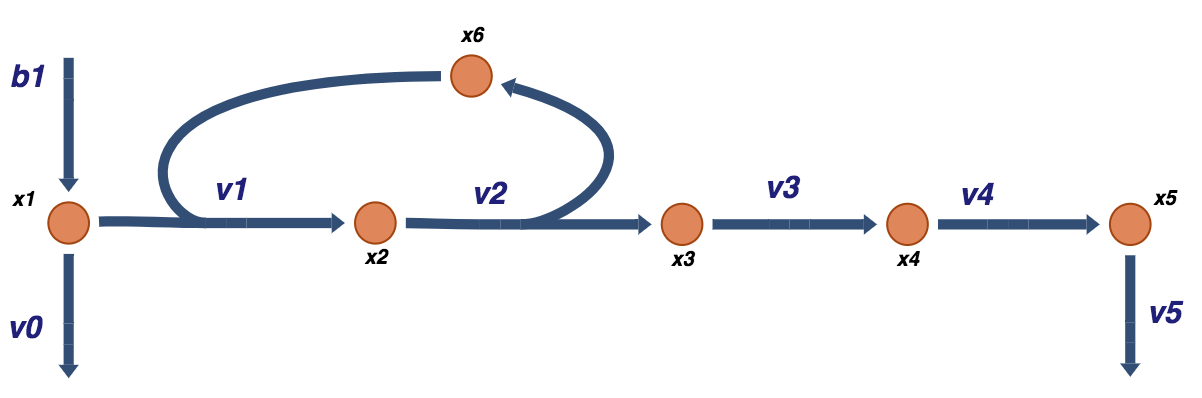

Supplement: S1 File — The latest version of the MASSpy software can be found at https://github.com/SBRG/MASSpy. (ZIP) [file pcbi.1008208.s003.zip › MASSpy-0.1.1/docs/education/sb2/images/Ch9/Figure-9-2.png]

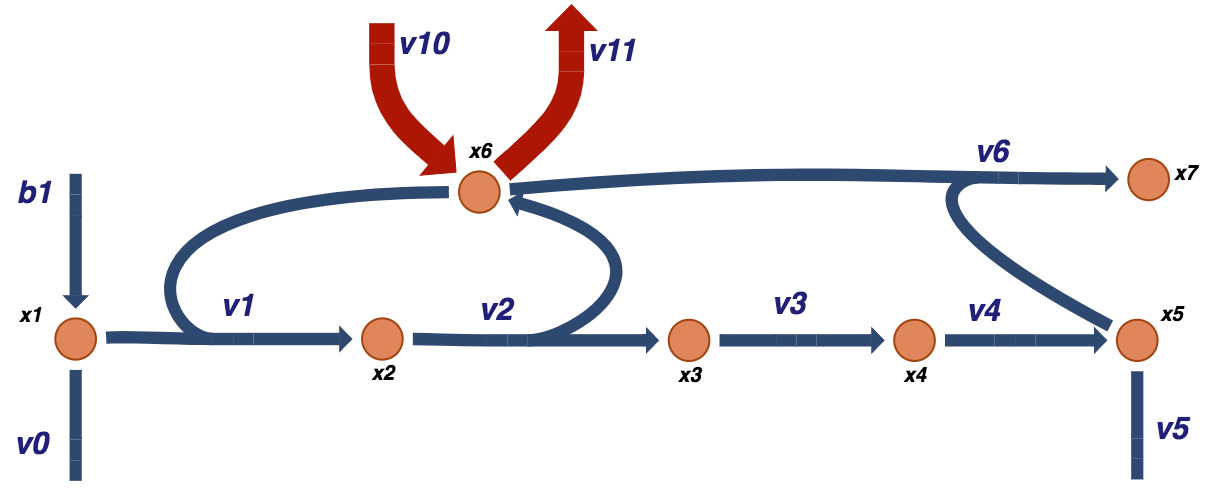

Supplement: S1 File — The latest version of the MASSpy software can be found at https://github.com/SBRG/MASSpy. (ZIP) [file pcbi.1008208.s003.zip › MASSpy-0.1.1/docs/education/sb2/images/Ch9/Figure-9-23.png]

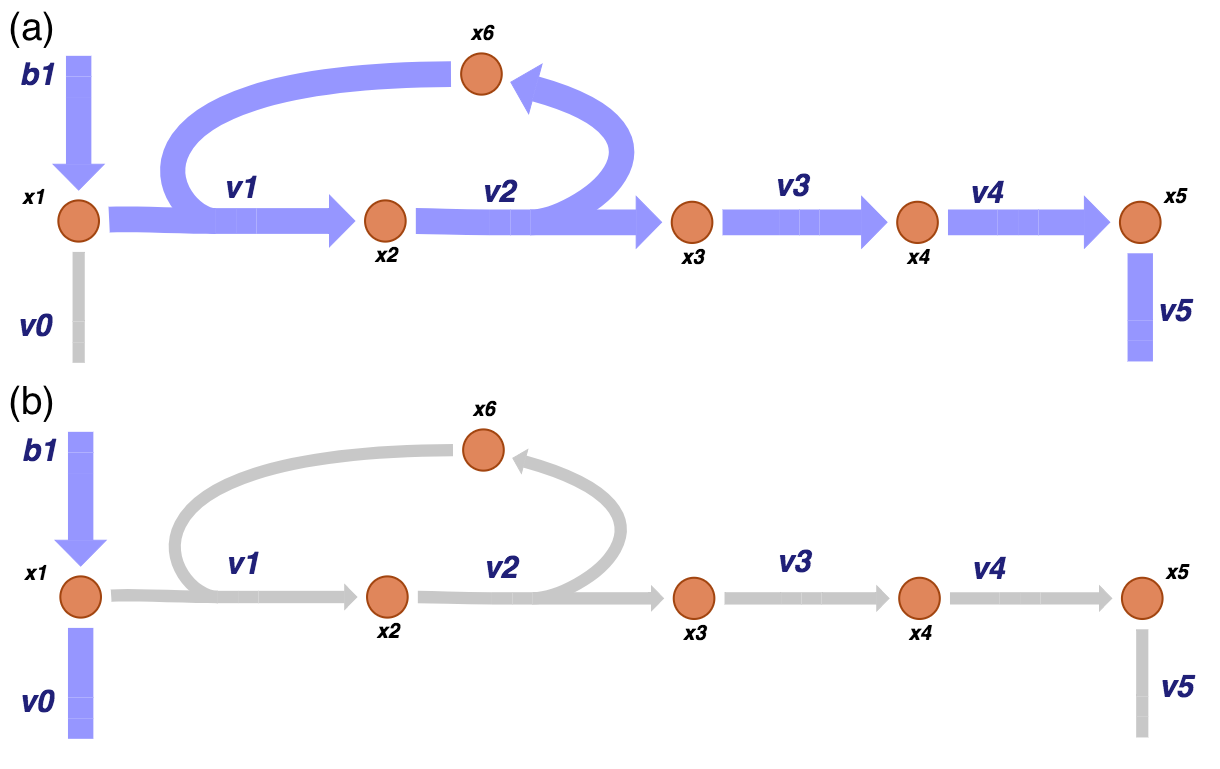

Supplement: S1 File — The latest version of the MASSpy software can be found at https://github.com/SBRG/MASSpy. (ZIP) [file pcbi.1008208.s003.zip › MASSpy-0.1.1/docs/education/sb2/images/Ch9/Figure-9-3.png]

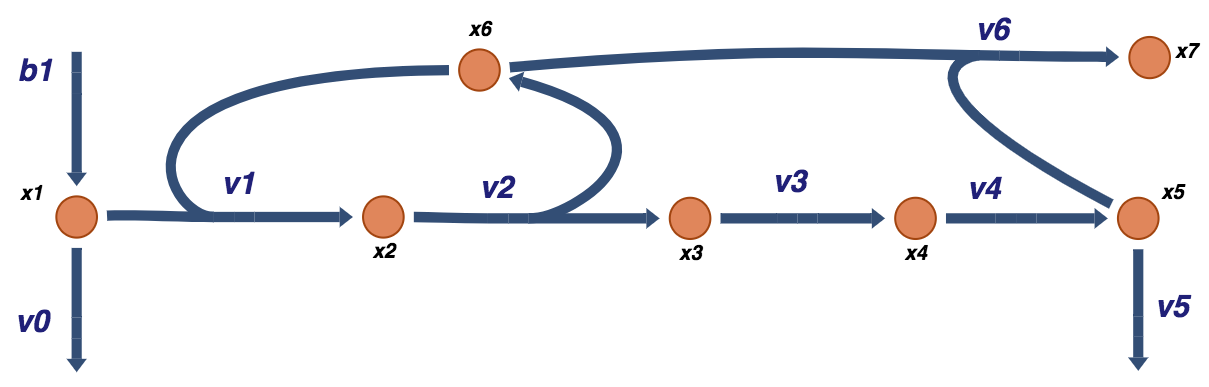

Supplement: S1 File — The latest version of the MASSpy software can be found at https://github.com/SBRG/MASSpy. (ZIP) [file pcbi.1008208.s003.zip › MASSpy-0.1.1/docs/education/sb2/images/Ch9/Figure-9-6.png]

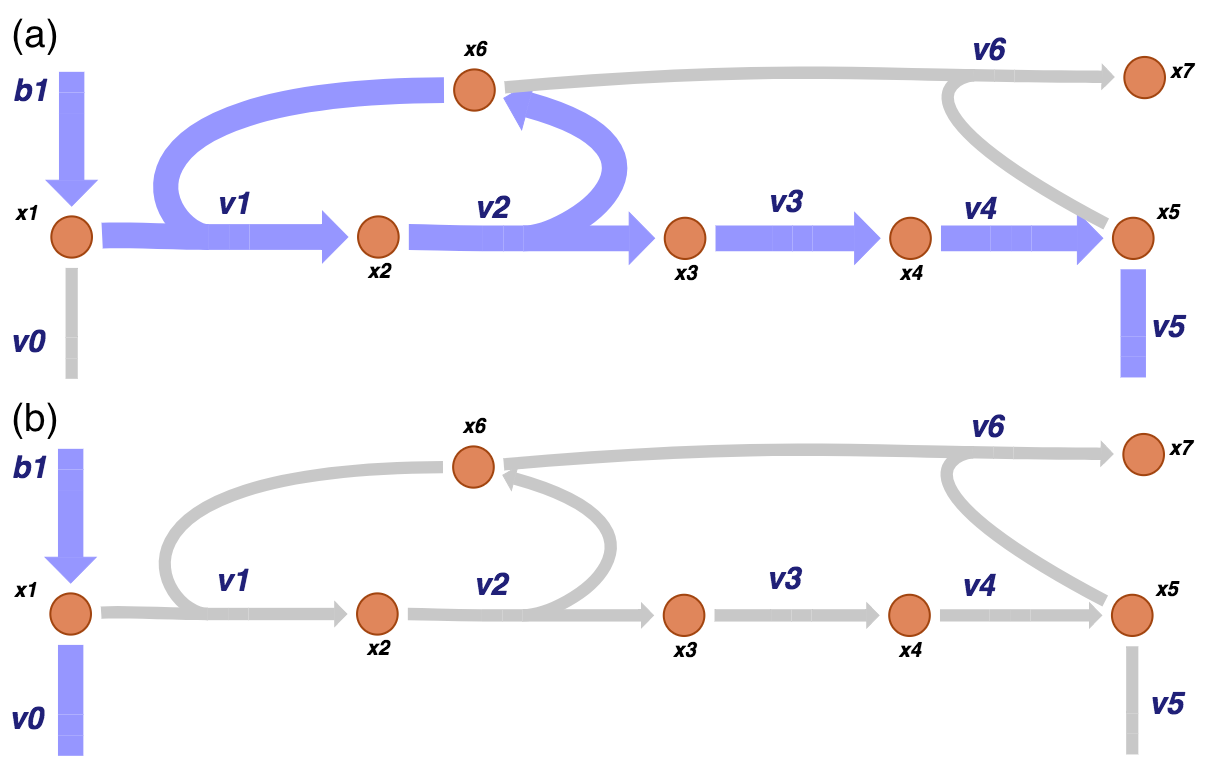

Supplement: S1 File — The latest version of the MASSpy software can be found at https://github.com/SBRG/MASSpy. (ZIP) [file pcbi.1008208.s003.zip › MASSpy-0.1.1/docs/education/sb2/images/Ch9/Figure-9-7.png]

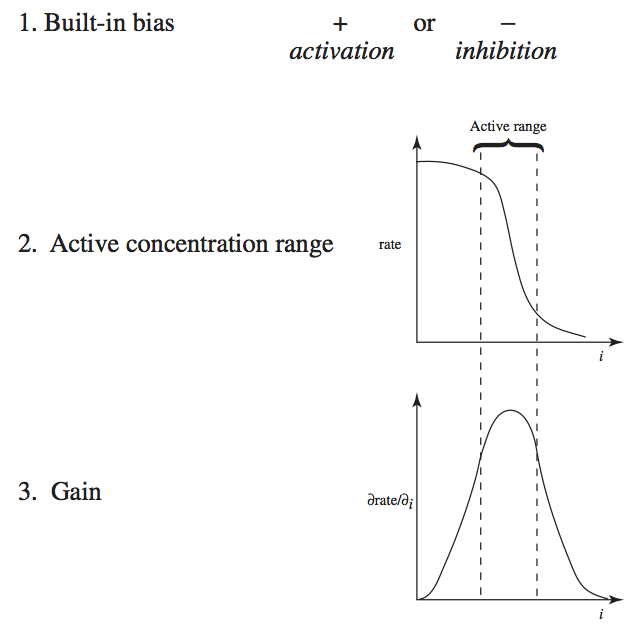

Supplement: S1 File — The latest version of the MASSpy software can be found at https://github.com/SBRG/MASSpy. (ZIP) [file pcbi.1008208.s003.zip › MASSpy-0.1.1/docs/education/sb2/images/Ch9/Figure-9-A1.png]

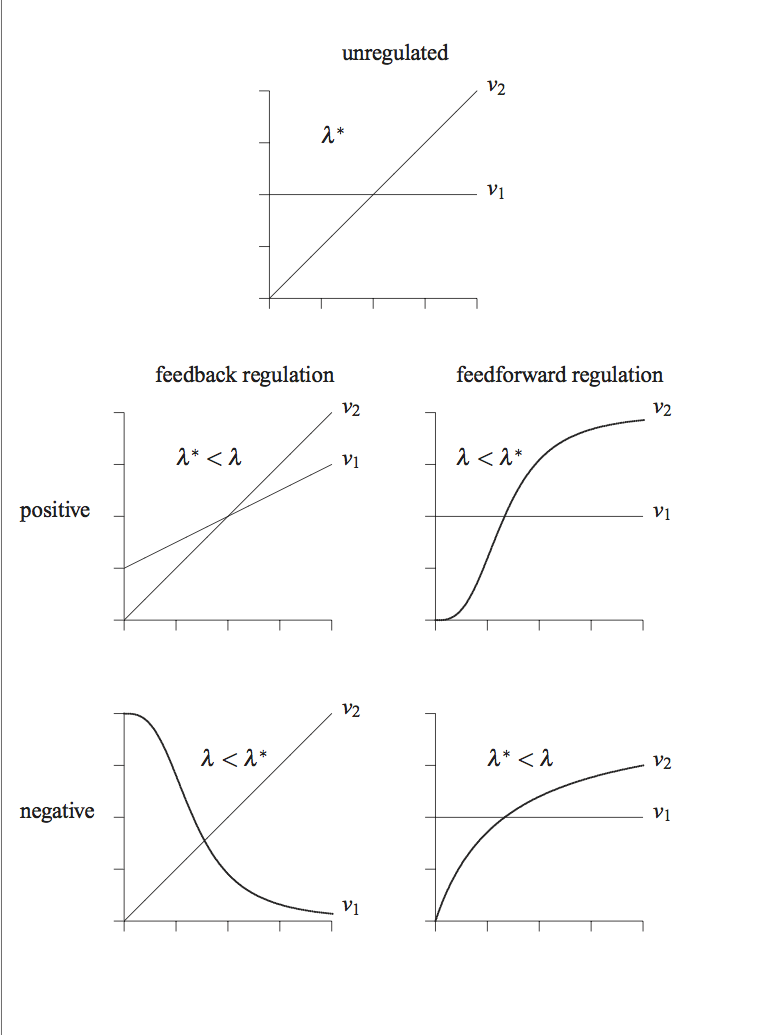

Supplement: S1 File — The latest version of the MASSpy software can be found at https://github.com/SBRG/MASSpy. (ZIP) [file pcbi.1008208.s003.zip › MASSpy-0.1.1/docs/education/sb2/images/Ch9/Figure-9-A2.png]

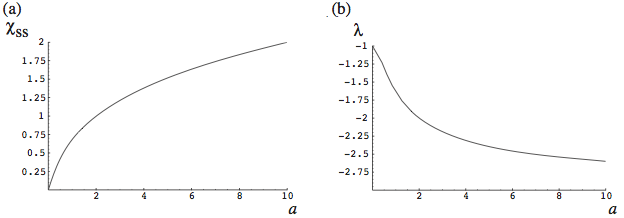

Supplement: S1 File — The latest version of the MASSpy software can be found at https://github.com/SBRG/MASSpy. (ZIP) [file pcbi.1008208.s003.zip › MASSpy-0.1.1/docs/education/sb2/images/Ch9/Figure-9-A3.png]
