# Supplementary figures and images for: MASSpy: Building, simulating, and visualizing dynamic biological models in Python using mass action kinetics (part 2 of 3)
Source: PLoS Comput Biol. 2021 Jan 28;17(1):e1008208. doi: 10.1371/journal.pcbi.1008208 (PMC7872247; doi:10.1371/journal.pcbi.1008208)

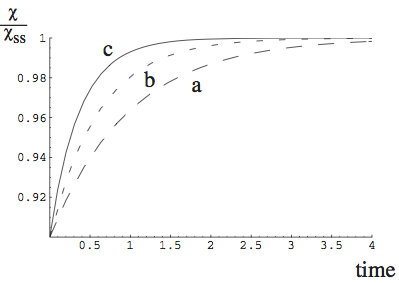

Supplement: S1 File — The latest version of the MASSpy software can be found at https://github.com/SBRG/MASSpy. (ZIP) [file pcbi.1008208.s003.zip › MASSpy-0.1.1/docs/education/sb2/images/Ch9/Figure-9-A4.png]

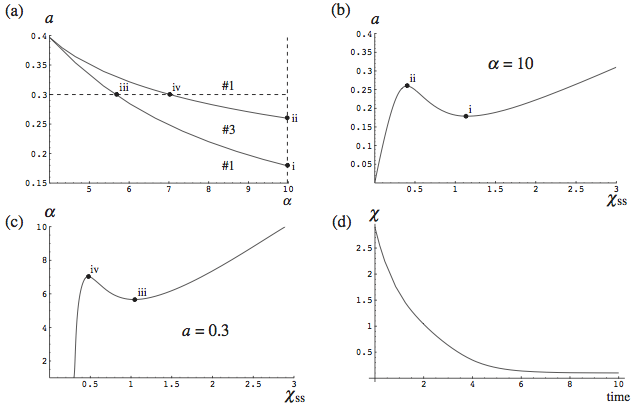

Supplement: S1 File — The latest version of the MASSpy software can be found at https://github.com/SBRG/MASSpy. (ZIP) [file pcbi.1008208.s003.zip › MASSpy-0.1.1/docs/education/sb2/images/Ch9/Figure-9-A5.png]

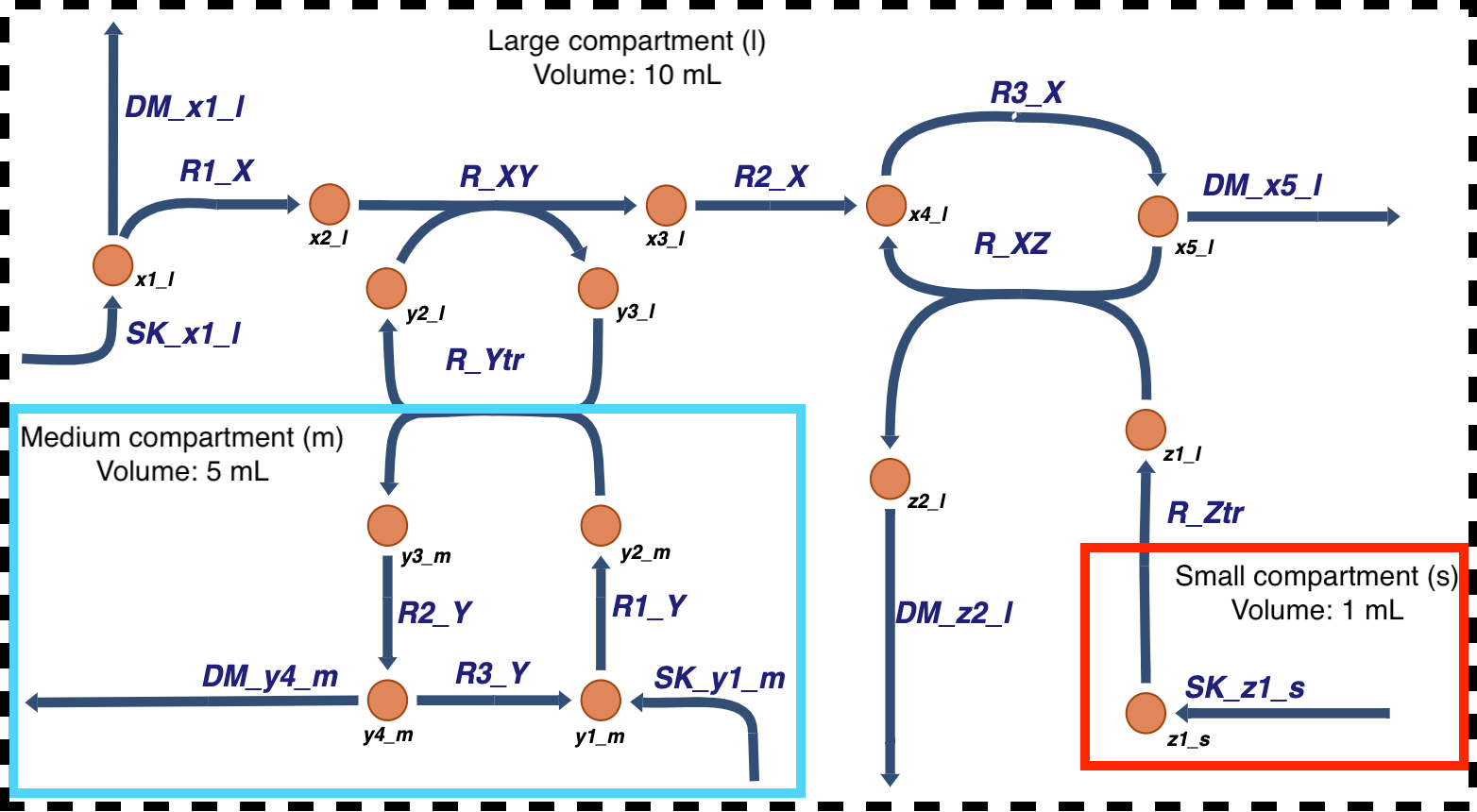

Supplement: S1 File — The latest version of the MASSpy software can be found at https://github.com/SBRG/MASSpy. (ZIP) [file pcbi.1008208.s003.zip › MASSpy-0.1.1/docs/images/multicompartment.png]

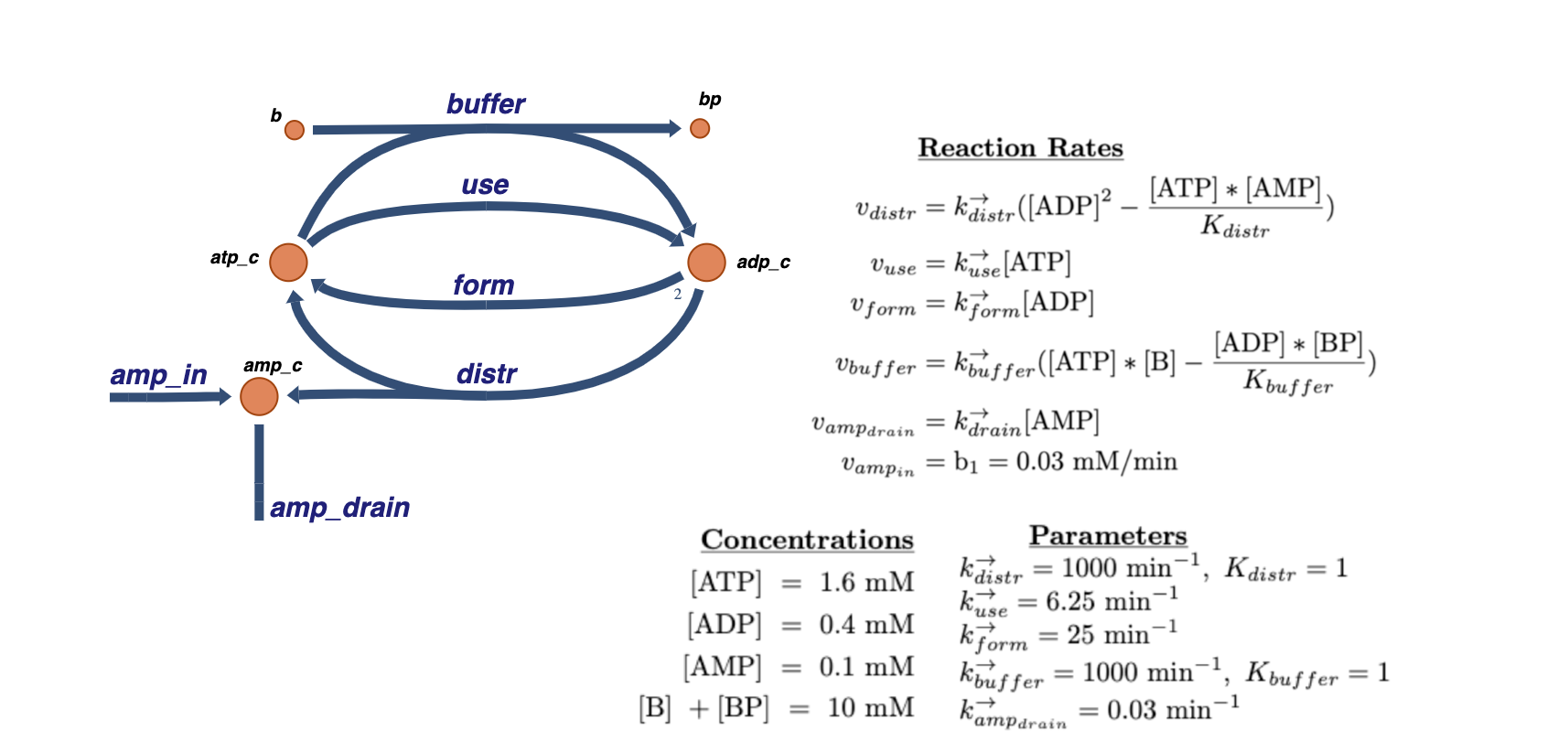

Supplement: S1 File — The latest version of the MASSpy software can be found at https://github.com/SBRG/MASSpy. (ZIP) [file pcbi.1008208.s003.zip › MASSpy-0.1.1/docs/images/phosphate_trafficking.png]

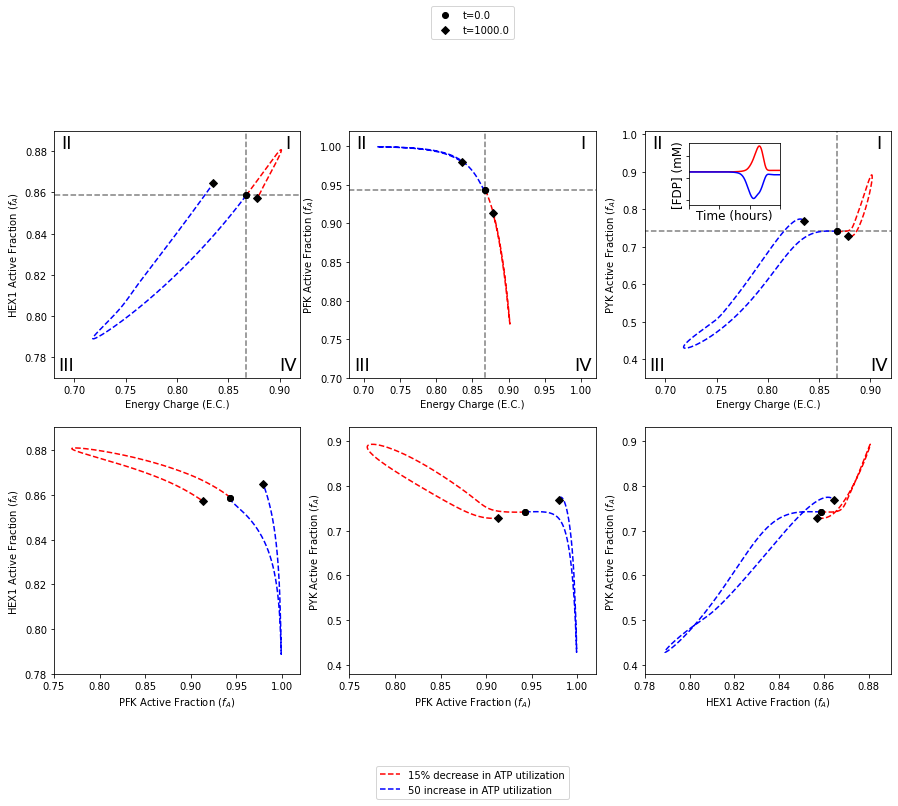

Supplement: S2 File — The latest version of the MASSpy documentation can be found at https://masspy.readthedocs.io. (ZIP) [file pcbi.1008208.s004.zip › masspy-v0.1.1/_images/gallery_visualization_catalytic_potential_visualizations_30_1.png]

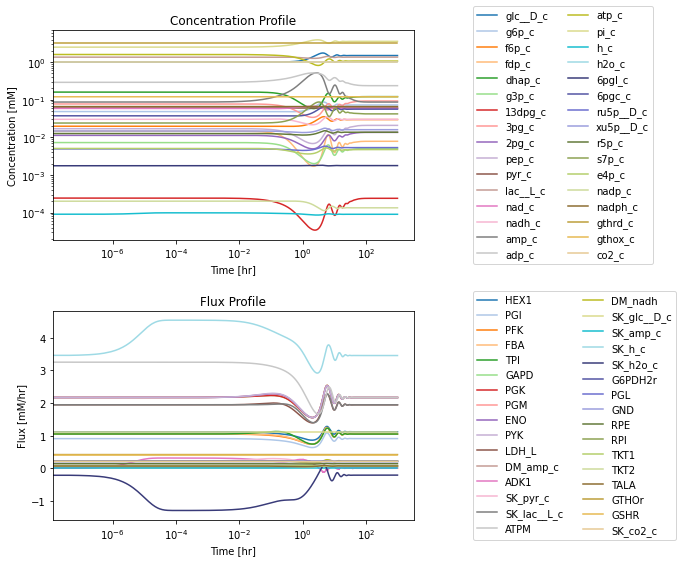

Supplement: S2 File — The latest version of the MASSpy documentation can be found at https://masspy.readthedocs.io. (ZIP) [file pcbi.1008208.s004.zip › masspy-v0.1.1/_images/education_sb2_chapters_sb2_chapter11_78_0.png]

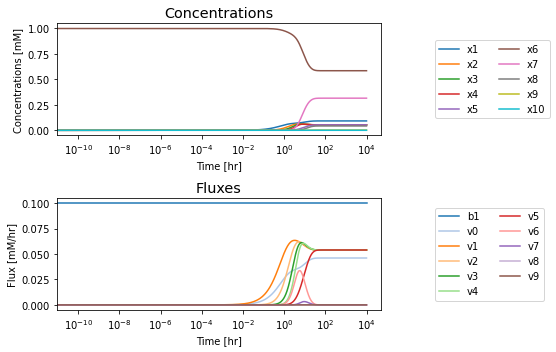

Supplement: S2 File — The latest version of the MASSpy documentation can be found at https://masspy.readthedocs.io. (ZIP) [file pcbi.1008208.s004.zip › masspy-v0.1.1/_images/education_sb2_chapters_sb2_chapter9_98_0.png]

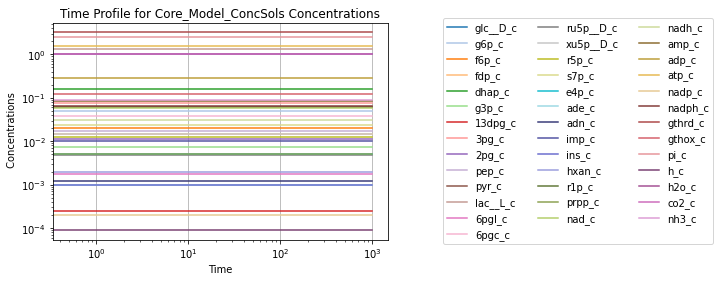

Supplement: S2 File — The latest version of the MASSpy documentation can be found at https://masspy.readthedocs.io. (ZIP) [file pcbi.1008208.s004.zip › masspy-v0.1.1/_images/education_sb2_chapters_sb2_chapter12_69_0.png]

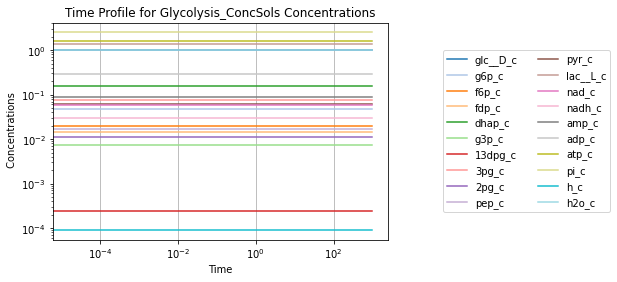

Supplement: S2 File — The latest version of the MASSpy documentation can be found at https://masspy.readthedocs.io. (ZIP) [file pcbi.1008208.s004.zip › masspy-v0.1.1/_images/gallery_workflows_constructing_glycolysis_26_1.png]

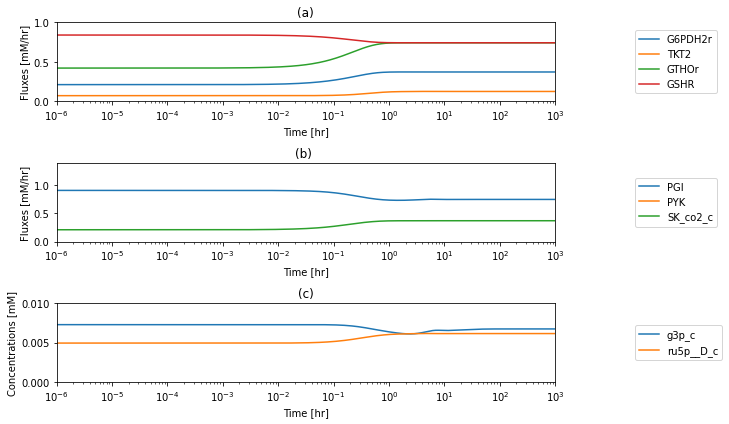

Supplement: S2 File — The latest version of the MASSpy documentation can be found at https://masspy.readthedocs.io. (ZIP) [file pcbi.1008208.s004.zip › masspy-v0.1.1/_images/education_sb2_chapters_sb2_chapter11_96_0.png]

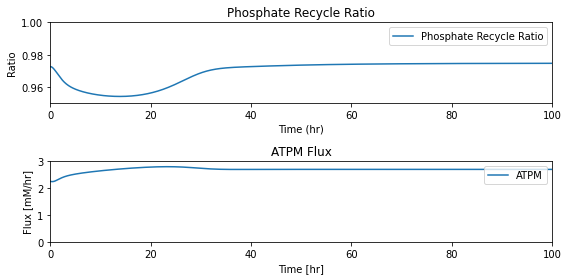

Supplement: S2 File — The latest version of the MASSpy documentation can be found at https://masspy.readthedocs.io. (ZIP) [file pcbi.1008208.s004.zip › masspy-v0.1.1/_images/education_sb2_chapters_sb2_chapter10_100_0.png]

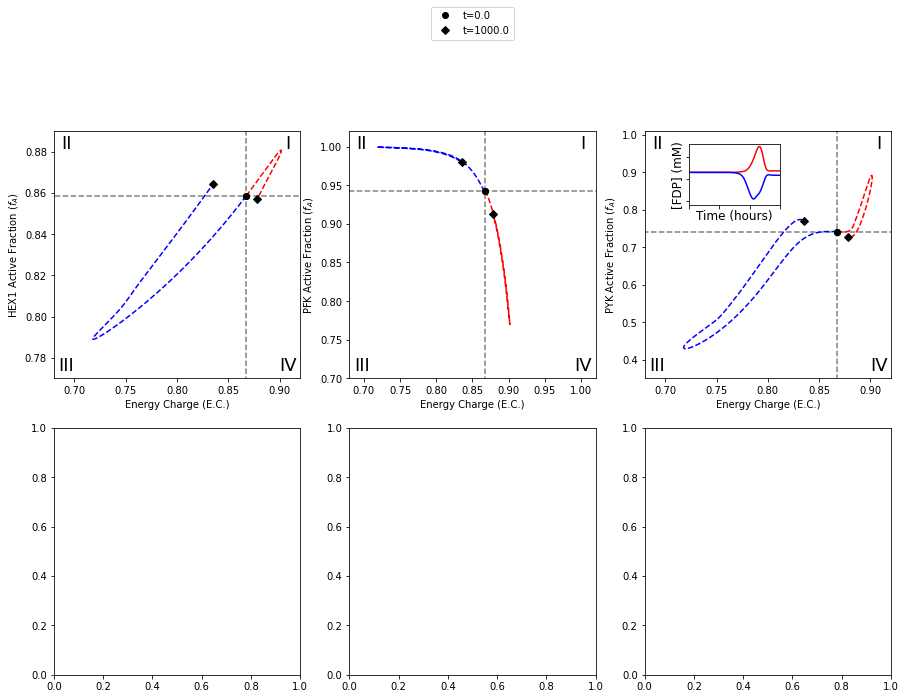

Supplement: S2 File — The latest version of the MASSpy documentation can be found at https://masspy.readthedocs.io. (ZIP) [file pcbi.1008208.s004.zip › masspy-v0.1.1/_images/gallery_visualization_catalytic_potential_visualizations_46_0.png]

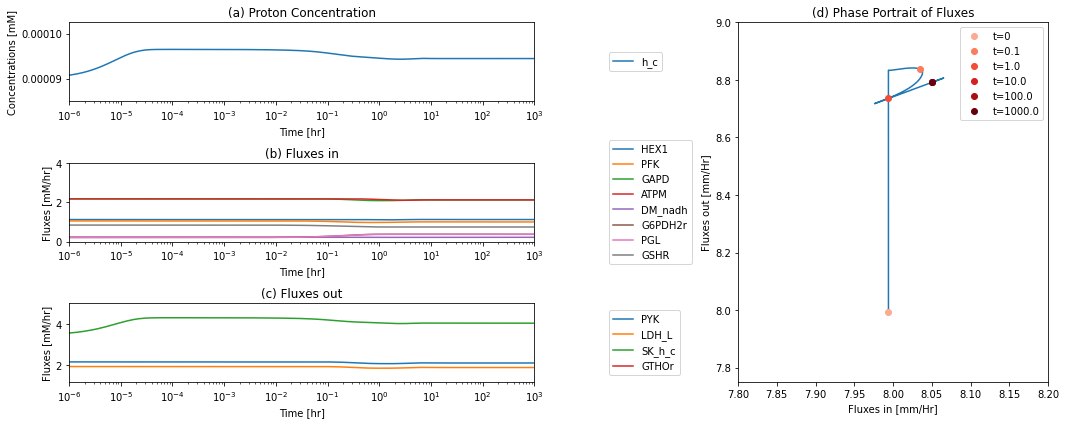

Supplement: S2 File — The latest version of the MASSpy documentation can be found at https://masspy.readthedocs.io. (ZIP) [file pcbi.1008208.s004.zip › masspy-v0.1.1/_images/education_sb2_chapters_sb2_chapter11_98_0.png]

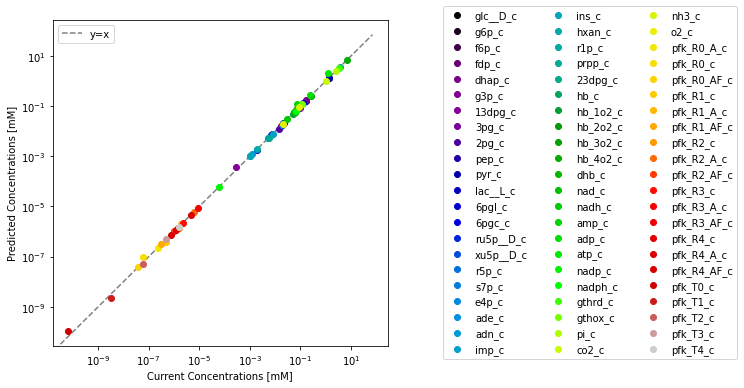

Supplement: S2 File — The latest version of the MASSpy documentation can be found at https://masspy.readthedocs.io. (ZIP) [file pcbi.1008208.s004.zip › masspy-v0.1.1/_images/tutorials_thermo_concentrations_34_1.png]

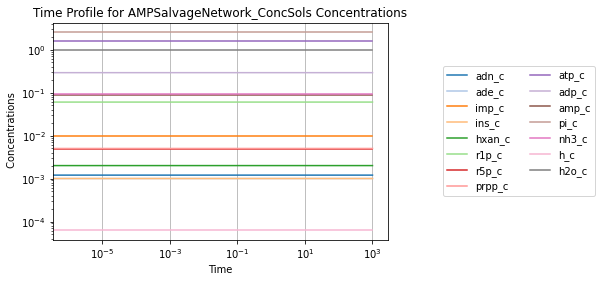

Supplement: S2 File — The latest version of the MASSpy documentation can be found at https://masspy.readthedocs.io. (ZIP) [file pcbi.1008208.s004.zip › masspy-v0.1.1/_images/education_sb2_model_construction_sb2_amp_salvage_network_26_1.png]

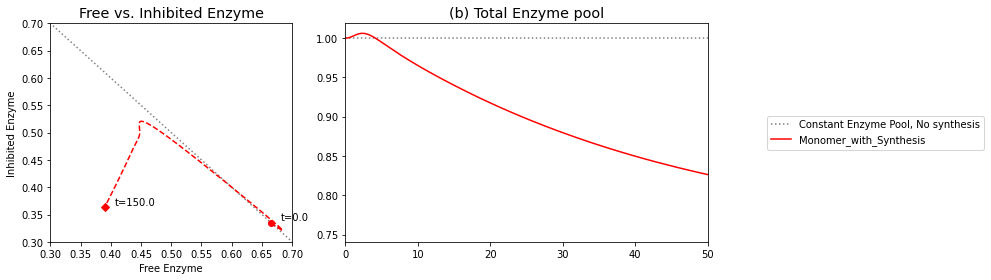

Supplement: S2 File — The latest version of the MASSpy documentation can be found at https://masspy.readthedocs.io. (ZIP) [file pcbi.1008208.s004.zip › masspy-v0.1.1/_images/education_sb2_chapters_sb2_chapter9_129_0.png]

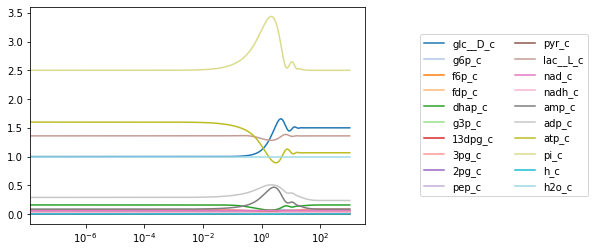

Supplement: S2 File — The latest version of the MASSpy documentation can be found at https://masspy.readthedocs.io. (ZIP) [file pcbi.1008208.s004.zip › masspy-v0.1.1/_images/tutorials_plot_visualization_15_1.png]

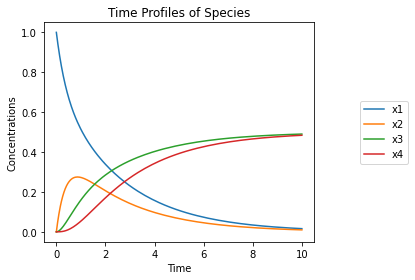

Supplement: S2 File — The latest version of the MASSpy documentation can be found at https://masspy.readthedocs.io. (ZIP) [file pcbi.1008208.s004.zip › masspy-v0.1.1/_images/education_sb2_chapters_sb2_chapter4_18_0.png]

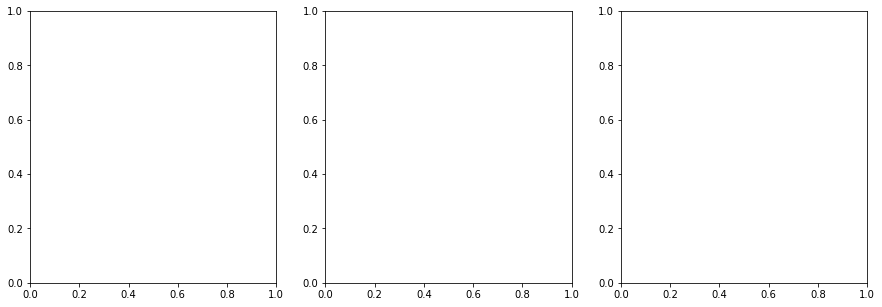

Supplement: S2 File — The latest version of the MASSpy documentation can be found at https://masspy.readthedocs.io. (ZIP) [file pcbi.1008208.s004.zip › masspy-v0.1.1/_images/gallery_visualization_catalytic_potential_visualizations_12_0.png]

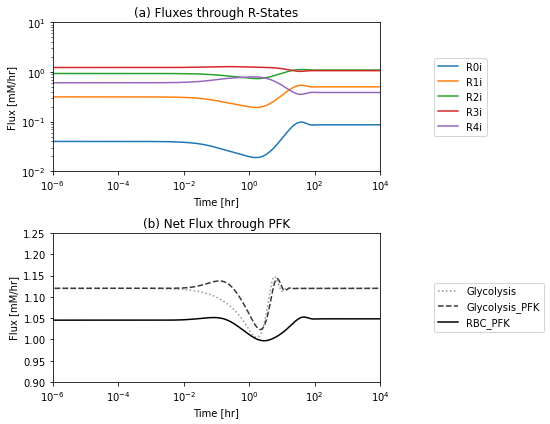

Supplement: S2 File — The latest version of the MASSpy documentation can be found at https://masspy.readthedocs.io. (ZIP) [file pcbi.1008208.s004.zip › masspy-v0.1.1/_images/education_sb2_chapters_sb2_chapter14_72_0.png]

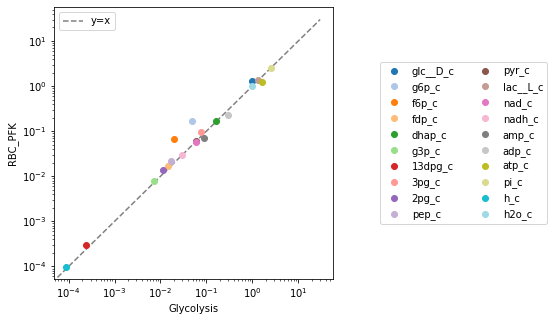

Supplement: S2 File — The latest version of the MASSpy documentation can be found at https://masspy.readthedocs.io. (ZIP) [file pcbi.1008208.s004.zip › masspy-v0.1.1/_images/tutorials_plot_visualization_51_1.png]

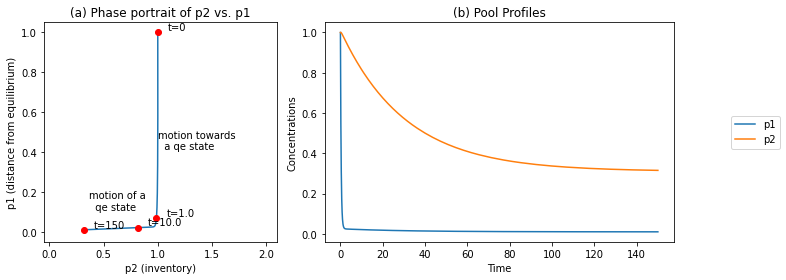

Supplement: S2 File — The latest version of the MASSpy documentation can be found at https://masspy.readthedocs.io. (ZIP) [file pcbi.1008208.s004.zip › masspy-v0.1.1/_images/education_sb2_chapters_sb2_chapter6_10_0.png]

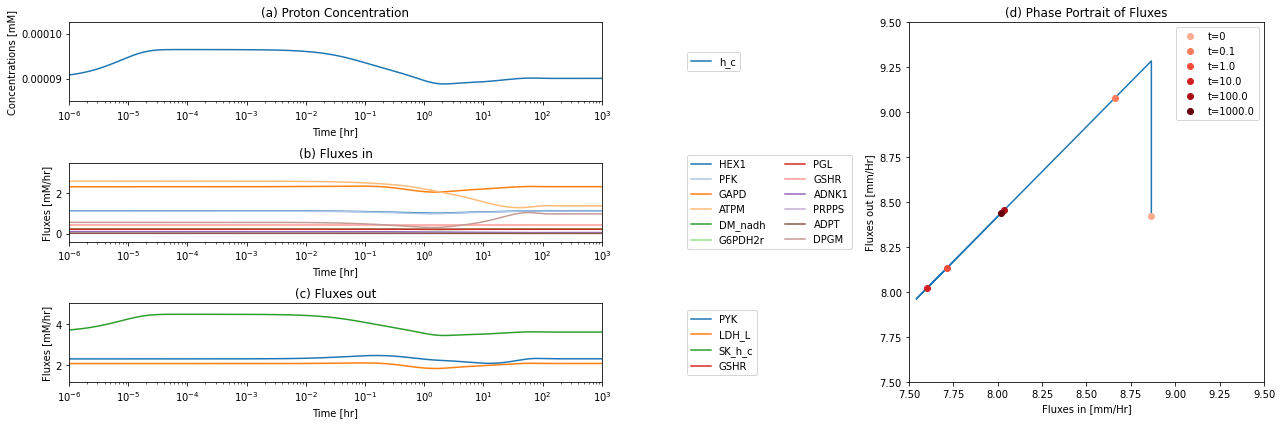

Supplement: S2 File — The latest version of the MASSpy documentation can be found at https://masspy.readthedocs.io. (ZIP) [file pcbi.1008208.s004.zip › masspy-v0.1.1/_images/education_sb2_chapters_sb2_chapter13_76_0.png]

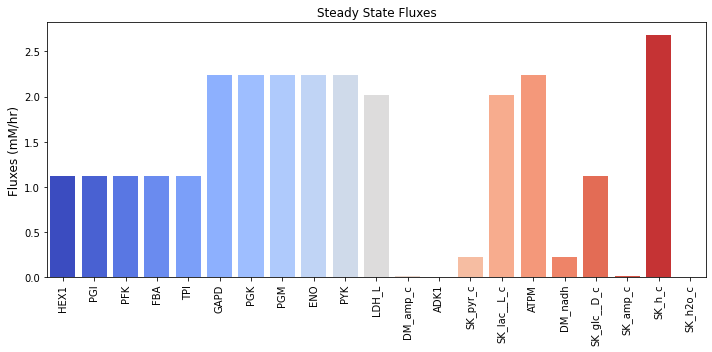

Supplement: S2 File — The latest version of the MASSpy documentation can be found at https://masspy.readthedocs.io. (ZIP) [file pcbi.1008208.s004.zip › masspy-v0.1.1/_images/education_sb2_chapters_sb2_chapter10_37_0.png]

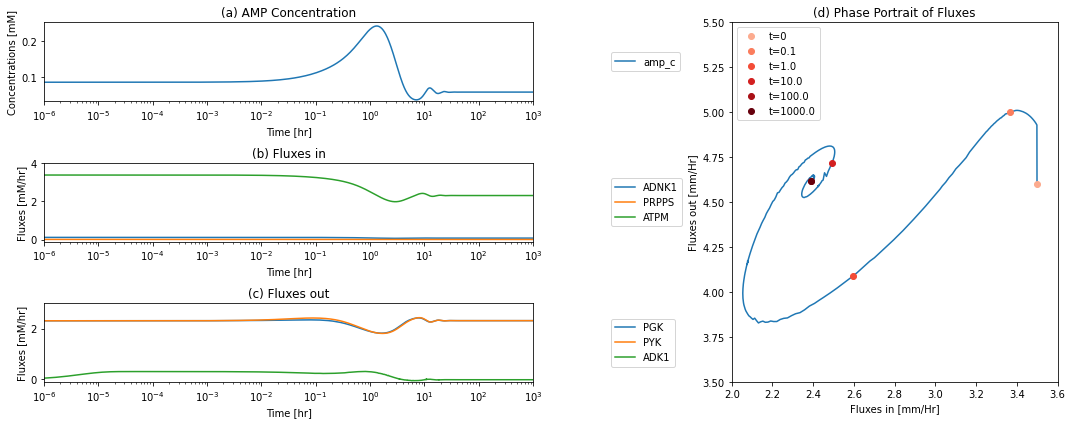

Supplement: S2 File — The latest version of the MASSpy documentation can be found at https://masspy.readthedocs.io. (ZIP) [file pcbi.1008208.s004.zip › masspy-v0.1.1/_images/education_sb2_chapters_sb2_chapter12_83_0.png]

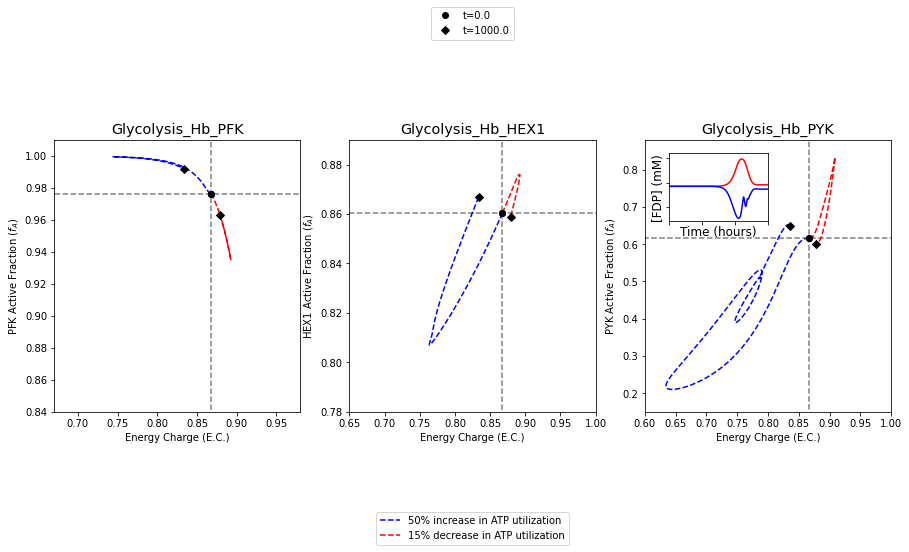

Supplement: S2 File — The latest version of the MASSpy documentation can be found at https://masspy.readthedocs.io. (ZIP) [file pcbi.1008208.s004.zip › masspy-v0.1.1/_images/gallery_visualization_catalytic_potential_visualizations_27_0.png]

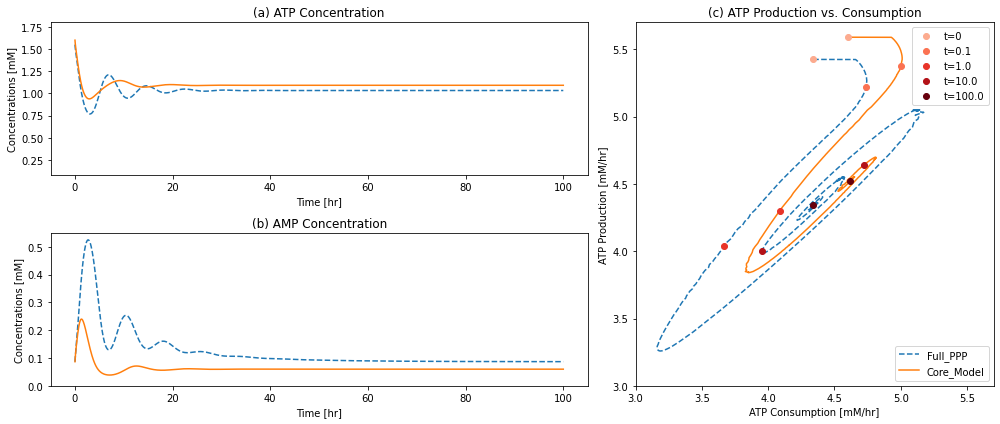

Supplement: S2 File — The latest version of the MASSpy documentation can be found at https://masspy.readthedocs.io. (ZIP) [file pcbi.1008208.s004.zip › masspy-v0.1.1/_images/education_sb2_chapters_sb2_chapter12_87_0.png]

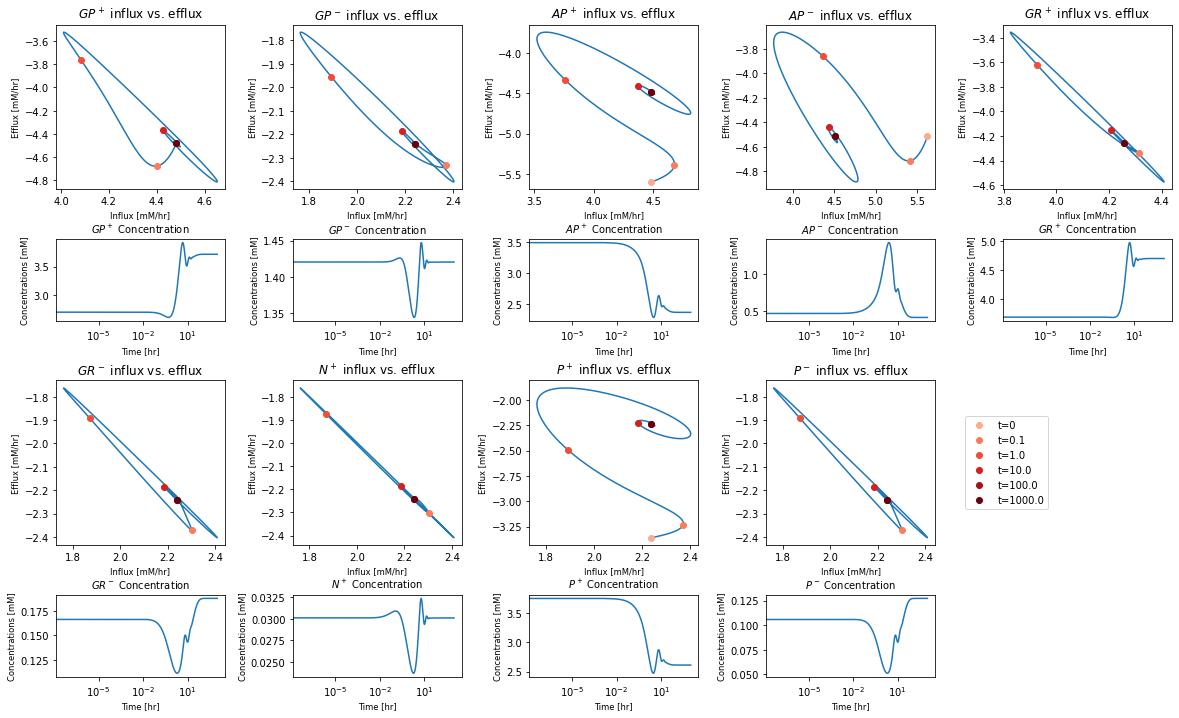

Supplement: S2 File — The latest version of the MASSpy documentation can be found at https://masspy.readthedocs.io. (ZIP) [file pcbi.1008208.s004.zip › masspy-v0.1.1/_images/education_sb2_chapters_sb2_chapter10_81_0.png]

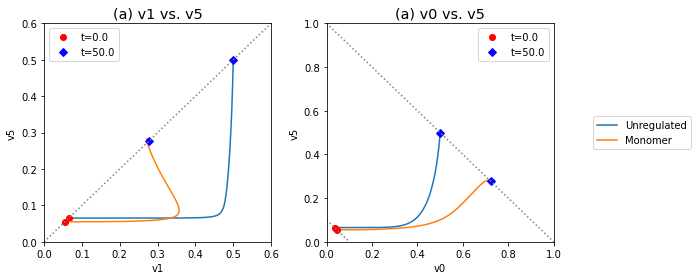

Supplement: S2 File — The latest version of the MASSpy documentation can be found at https://masspy.readthedocs.io. (ZIP) [file pcbi.1008208.s004.zip › masspy-v0.1.1/_images/education_sb2_chapters_sb2_chapter9_52_0.png]

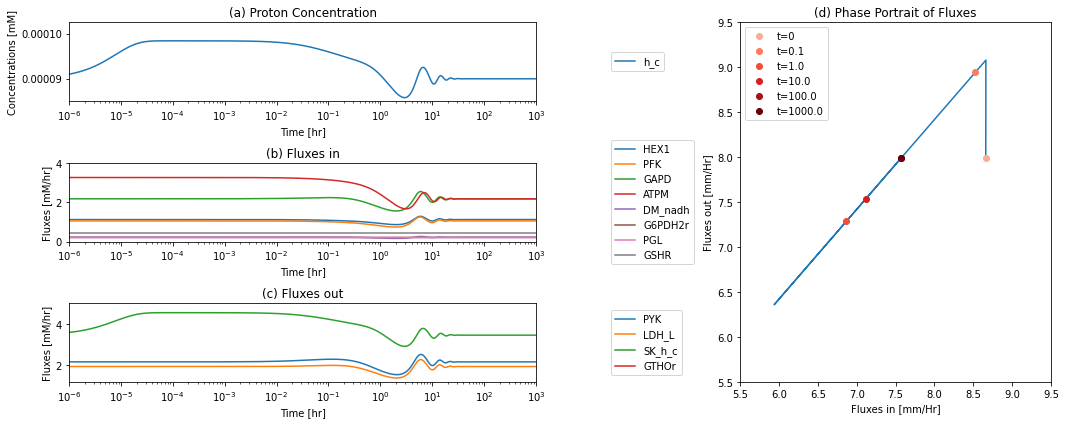

Supplement: S2 File — The latest version of the MASSpy documentation can be found at https://masspy.readthedocs.io. (ZIP) [file pcbi.1008208.s004.zip › masspy-v0.1.1/_images/education_sb2_chapters_sb2_chapter11_86_0.png]

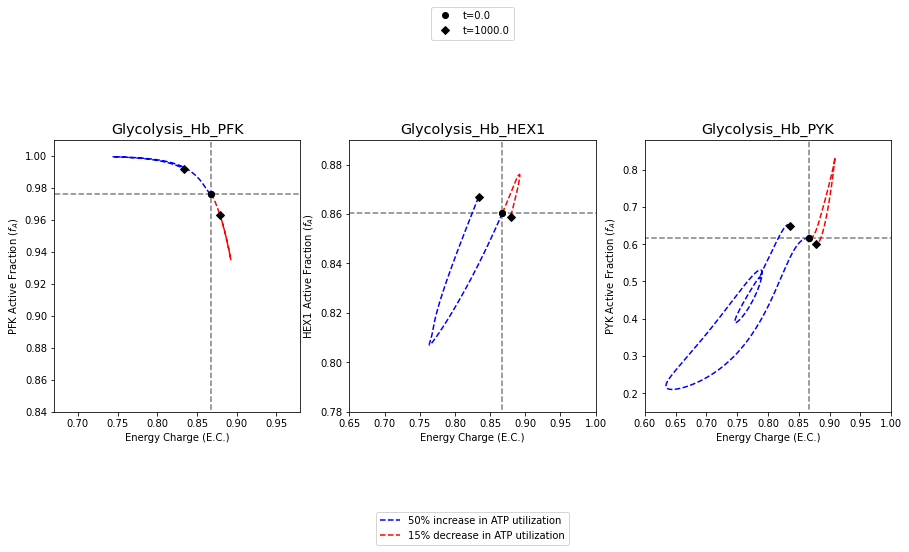

Supplement: S2 File — The latest version of the MASSpy documentation can be found at https://masspy.readthedocs.io. (ZIP) [file pcbi.1008208.s004.zip › masspy-v0.1.1/_images/gallery_visualization_catalytic_potential_visualizations_20_0.png]

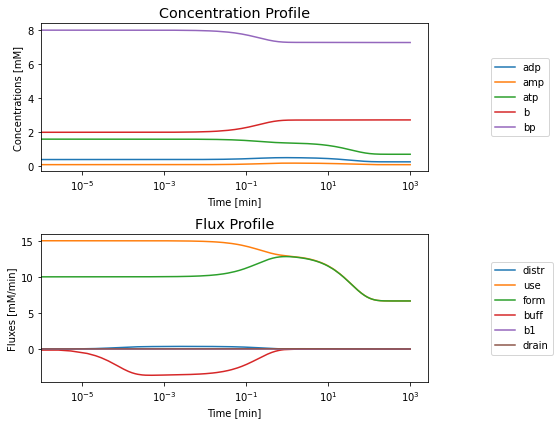

Supplement: S2 File — The latest version of the MASSpy documentation can be found at https://masspy.readthedocs.io. (ZIP) [file pcbi.1008208.s004.zip › masspy-v0.1.1/_images/education_sb2_chapters_sb2_chapter8_71_0.png]

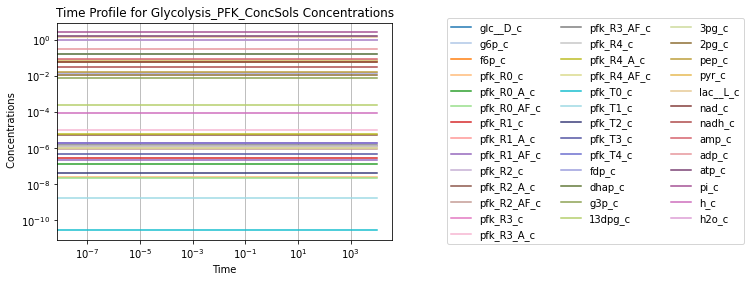

Supplement: S2 File — The latest version of the MASSpy documentation can be found at https://masspy.readthedocs.io. (ZIP) [file pcbi.1008208.s004.zip › masspy-v0.1.1/_images/education_sb2_chapters_sb2_chapter14_39_0.png]

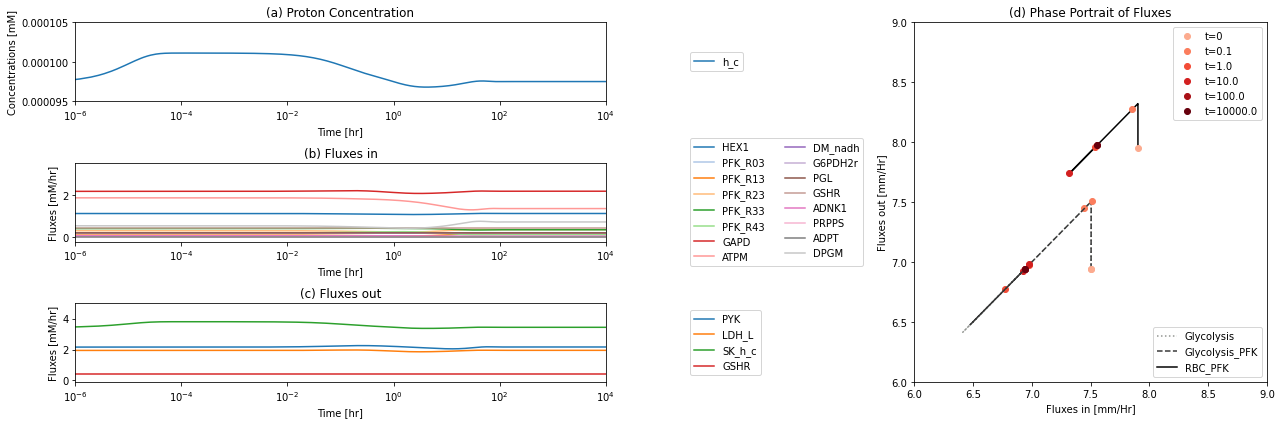

Supplement: S2 File — The latest version of the MASSpy documentation can be found at https://masspy.readthedocs.io. (ZIP) [file pcbi.1008208.s004.zip › masspy-v0.1.1/_images/education_sb2_chapters_sb2_chapter14_68_0.png]

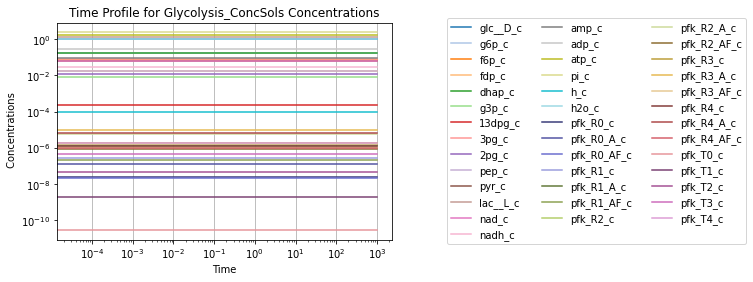

Supplement: S2 File — The latest version of the MASSpy documentation can be found at https://masspy.readthedocs.io. (ZIP) [file pcbi.1008208.s004.zip › masspy-v0.1.1/_images/education_sb2_model_construction_sb2_pfk_63_1.png]

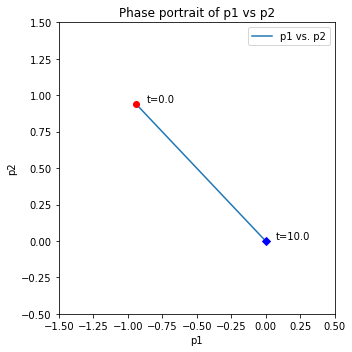

Supplement: S2 File — The latest version of the MASSpy documentation can be found at https://masspy.readthedocs.io. (ZIP) [file pcbi.1008208.s004.zip › masspy-v0.1.1/_images/education_sb2_chapters_sb2_chapter4_29_0.png]

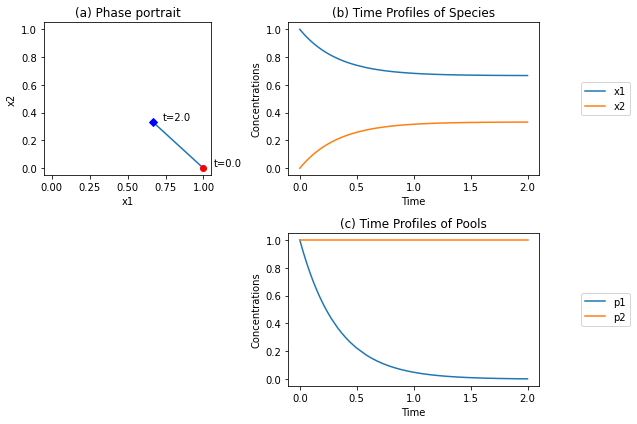

Supplement: S2 File — The latest version of the MASSpy documentation can be found at https://masspy.readthedocs.io. (ZIP) [file pcbi.1008208.s004.zip › masspy-v0.1.1/_images/education_sb2_chapters_sb2_chapter4_8_0.png]

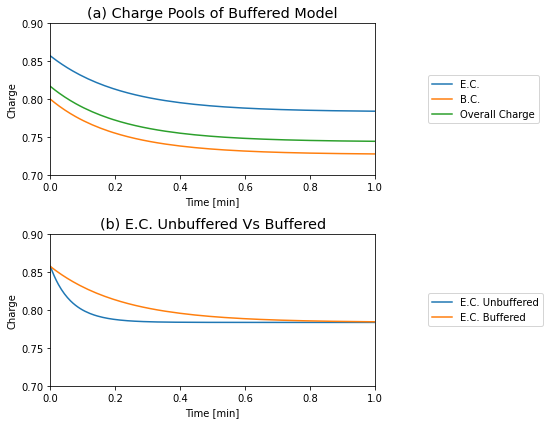

Supplement: S2 File — The latest version of the MASSpy documentation can be found at https://masspy.readthedocs.io. (ZIP) [file pcbi.1008208.s004.zip › masspy-v0.1.1/_images/education_sb2_chapters_sb2_chapter8_59_1.png]

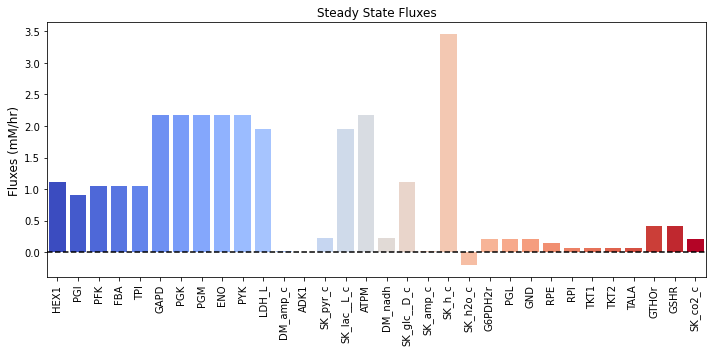

Supplement: S2 File — The latest version of the MASSpy documentation can be found at https://masspy.readthedocs.io. (ZIP) [file pcbi.1008208.s004.zip › masspy-v0.1.1/_images/education_sb2_chapters_sb2_chapter11_64_0.png]

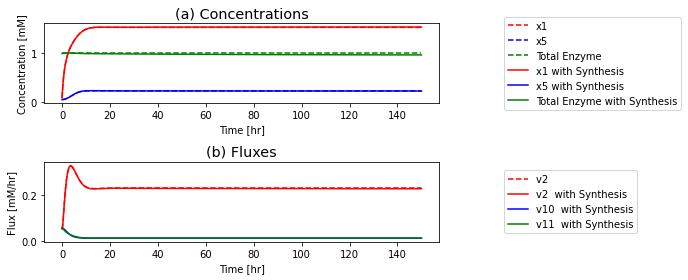

Supplement: S2 File — The latest version of the MASSpy documentation can be found at https://masspy.readthedocs.io. (ZIP) [file pcbi.1008208.s004.zip › masspy-v0.1.1/_images/education_sb2_chapters_sb2_chapter9_158_0.png]

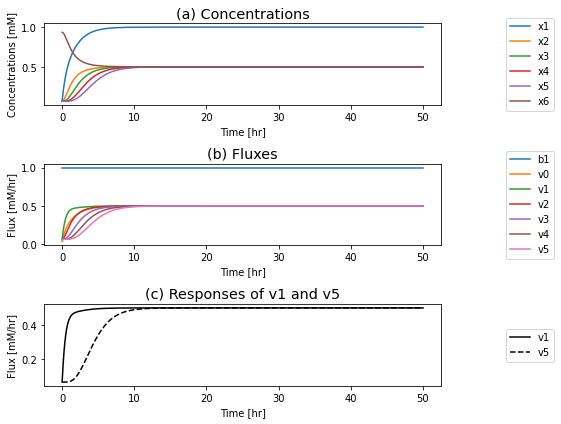

Supplement: S2 File — The latest version of the MASSpy documentation can be found at https://masspy.readthedocs.io. (ZIP) [file pcbi.1008208.s004.zip › masspy-v0.1.1/_images/education_sb2_chapters_sb2_chapter9_24_0.png]

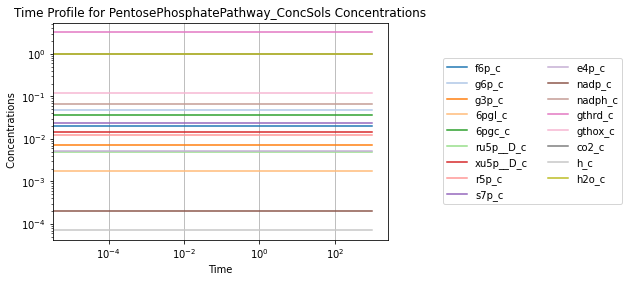

Supplement: S2 File — The latest version of the MASSpy documentation can be found at https://masspy.readthedocs.io. (ZIP) [file pcbi.1008208.s004.zip › masspy-v0.1.1/_images/education_sb2_model_construction_sb2_pentose_phosphate_pathway_26_1.png]

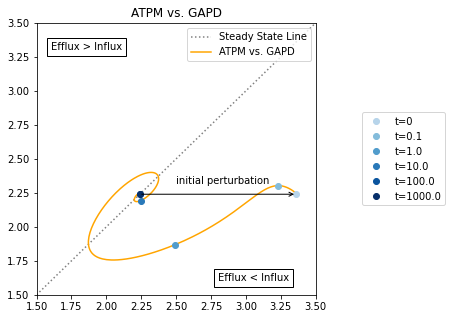

Supplement: S2 File — The latest version of the MASSpy documentation can be found at https://masspy.readthedocs.io. (ZIP) [file pcbi.1008208.s004.zip › masspy-v0.1.1/_images/tutorials_plot_visualization_42_1.png]

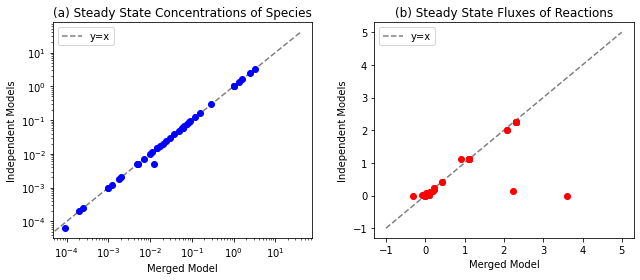

Supplement: S2 File — The latest version of the MASSpy documentation can be found at https://masspy.readthedocs.io. (ZIP) [file pcbi.1008208.s004.zip › masspy-v0.1.1/_images/education_sb2_chapters_sb2_chapter12_72_0.png]

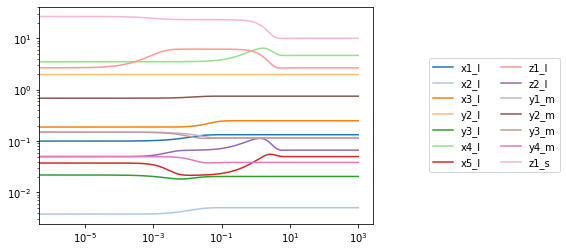

Supplement: S2 File — The latest version of the MASSpy documentation can be found at https://masspy.readthedocs.io. (ZIP) [file pcbi.1008208.s004.zip › masspy-v0.1.1/_images/tutorials_compartments_47_1.png]

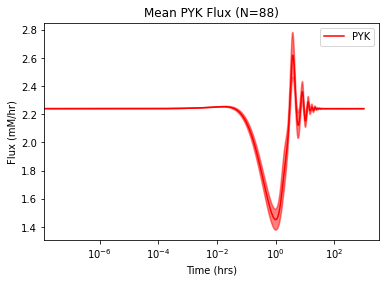

Supplement: S2 File — The latest version of the MASSpy documentation can be found at https://masspy.readthedocs.io. (ZIP) [file pcbi.1008208.s004.zip › masspy-v0.1.1/_images/tutorials_ensemble_modeling_42_1.png]

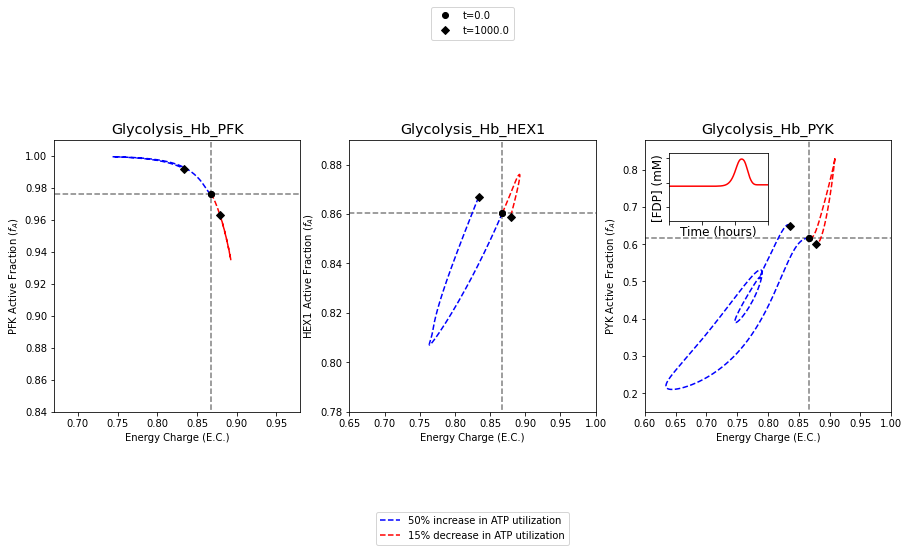

Supplement: S2 File — The latest version of the MASSpy documentation can be found at https://masspy.readthedocs.io. (ZIP) [file pcbi.1008208.s004.zip › masspy-v0.1.1/_images/gallery_visualization_catalytic_potential_visualizations_25_0.png]

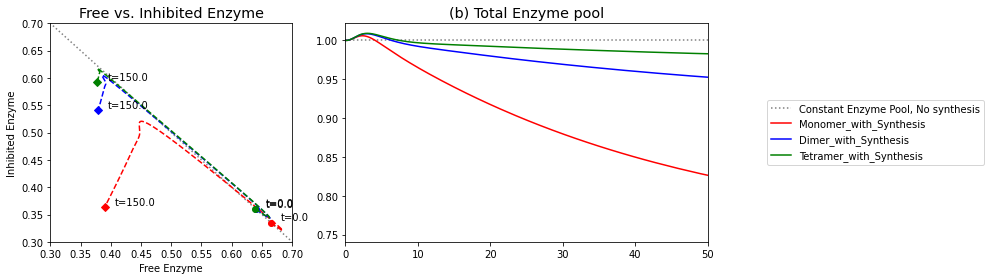

Supplement: S2 File — The latest version of the MASSpy documentation can be found at https://masspy.readthedocs.io. (ZIP) [file pcbi.1008208.s004.zip › masspy-v0.1.1/_images/education_sb2_chapters_sb2_chapter9_160_0.png]

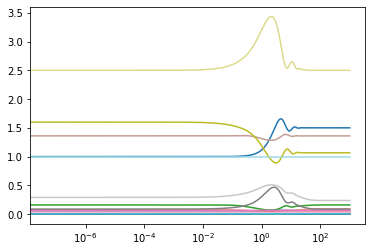

Supplement: S2 File — The latest version of the MASSpy documentation can be found at https://masspy.readthedocs.io. (ZIP) [file pcbi.1008208.s004.zip › masspy-v0.1.1/_images/tutorials_plot_visualization_13_1.png]

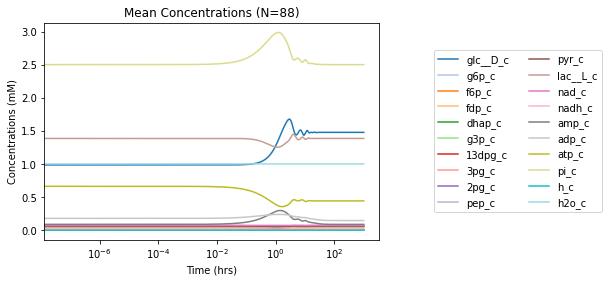

Supplement: S2 File — The latest version of the MASSpy documentation can be found at https://masspy.readthedocs.io. (ZIP) [file pcbi.1008208.s004.zip › masspy-v0.1.1/_images/tutorials_ensemble_modeling_40_1.png]

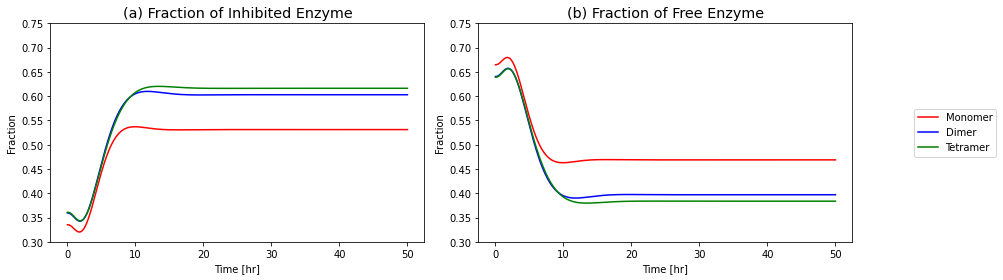

Supplement: S2 File — The latest version of the MASSpy documentation can be found at https://masspy.readthedocs.io. (ZIP) [file pcbi.1008208.s004.zip › masspy-v0.1.1/_images/education_sb2_chapters_sb2_chapter9_112_0.png]

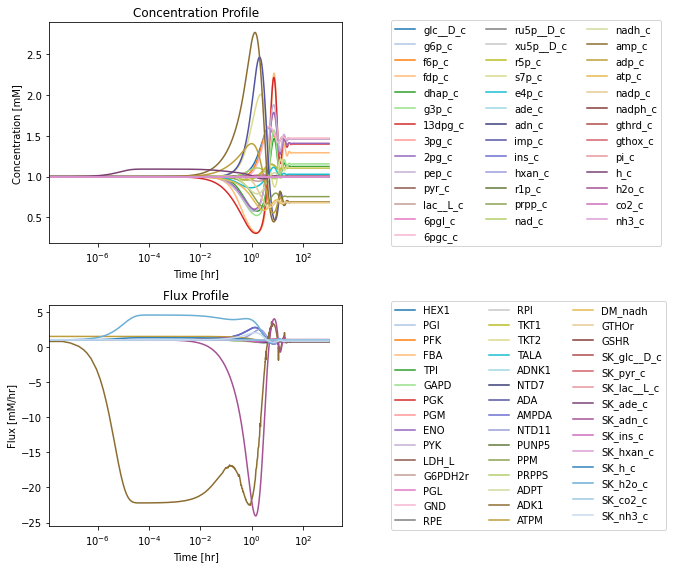

Supplement: S2 File — The latest version of the MASSpy documentation can be found at https://masspy.readthedocs.io. (ZIP) [file pcbi.1008208.s004.zip › masspy-v0.1.1/_images/education_sb2_chapters_sb2_chapter12_77_0.png]

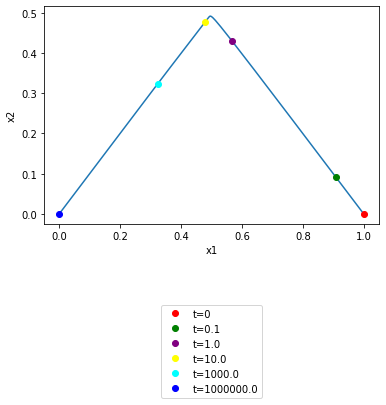

Supplement: S2 File — The latest version of the MASSpy documentation can be found at https://masspy.readthedocs.io. (ZIP) [file pcbi.1008208.s004.zip › masspy-v0.1.1/_images/education_sb2_chapters_sb2_chapter3_95_1.png]

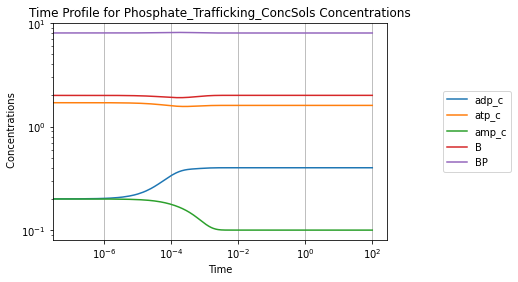

Supplement: S2 File — The latest version of the MASSpy documentation can be found at https://masspy.readthedocs.io. (ZIP) [file pcbi.1008208.s004.zip › masspy-v0.1.1/_images/tutorials_dynamic_simulation_36_0.png]

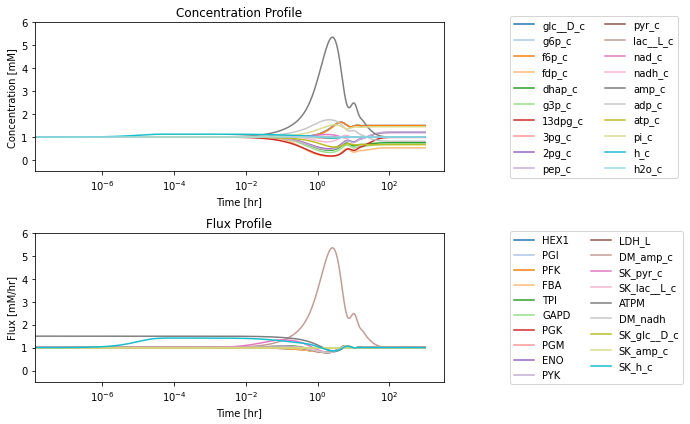

Supplement: S2 File — The latest version of the MASSpy documentation can be found at https://masspy.readthedocs.io. (ZIP) [file pcbi.1008208.s004.zip › masspy-v0.1.1/_images/education_sb2_chapters_sb2_chapter10_51_0.png]

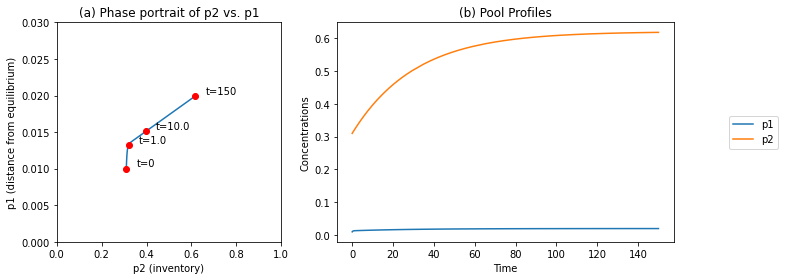

Supplement: S2 File — The latest version of the MASSpy documentation can be found at https://masspy.readthedocs.io. (ZIP) [file pcbi.1008208.s004.zip › masspy-v0.1.1/_images/education_sb2_chapters_sb2_chapter6_13_0.png]

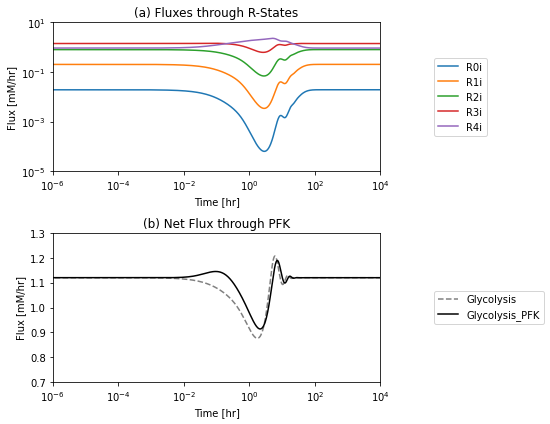

Supplement: S2 File — The latest version of the MASSpy documentation can be found at https://masspy.readthedocs.io. (ZIP) [file pcbi.1008208.s004.zip › masspy-v0.1.1/_images/education_sb2_chapters_sb2_chapter14_48_0.png]

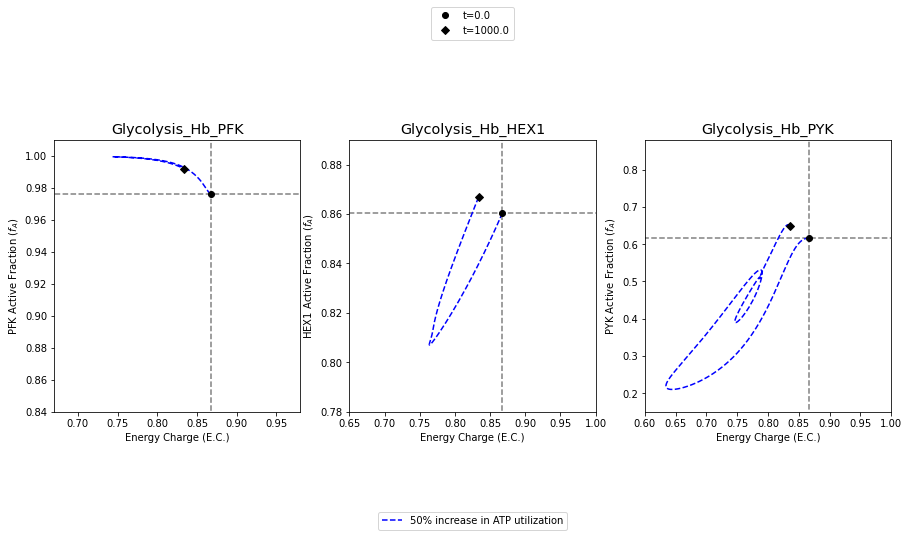

Supplement: S2 File — The latest version of the MASSpy documentation can be found at https://masspy.readthedocs.io. (ZIP) [file pcbi.1008208.s004.zip › masspy-v0.1.1/_images/gallery_visualization_catalytic_potential_visualizations_18_0.png]

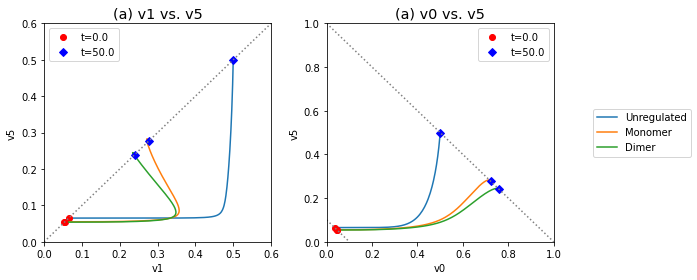

Supplement: S2 File — The latest version of the MASSpy documentation can be found at https://masspy.readthedocs.io. (ZIP) [file pcbi.1008208.s004.zip › masspy-v0.1.1/_images/education_sb2_chapters_sb2_chapter9_79_0.png]

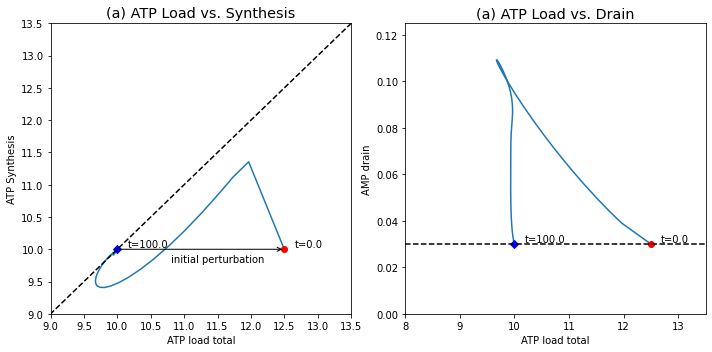

Supplement: S2 File — The latest version of the MASSpy documentation can be found at https://masspy.readthedocs.io. (ZIP) [file pcbi.1008208.s004.zip › masspy-v0.1.1/_images/education_sb2_chapters_sb2_chapter8_83_0.png]

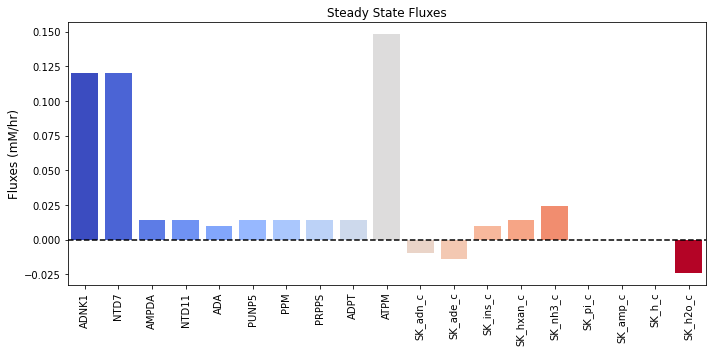

Supplement: S2 File — The latest version of the MASSpy documentation can be found at https://masspy.readthedocs.io. (ZIP) [file pcbi.1008208.s004.zip › masspy-v0.1.1/_images/education_sb2_chapters_sb2_chapter12_28_0.png]

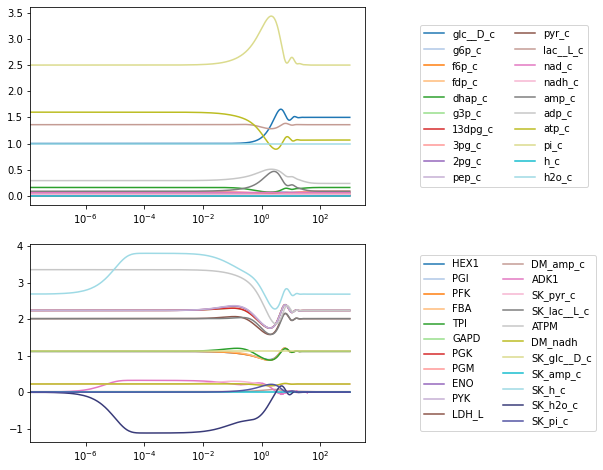

Supplement: S2 File — The latest version of the MASSpy documentation can be found at https://masspy.readthedocs.io. (ZIP) [file pcbi.1008208.s004.zip › masspy-v0.1.1/_images/tutorials_plot_visualization_19_1.png]

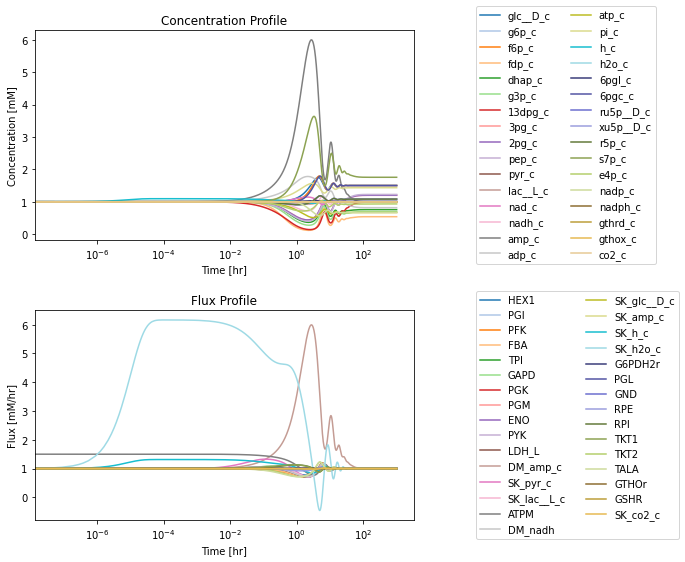

Supplement: S2 File — The latest version of the MASSpy documentation can be found at https://masspy.readthedocs.io. (ZIP) [file pcbi.1008208.s004.zip › masspy-v0.1.1/_images/education_sb2_chapters_sb2_chapter11_82_0.png]

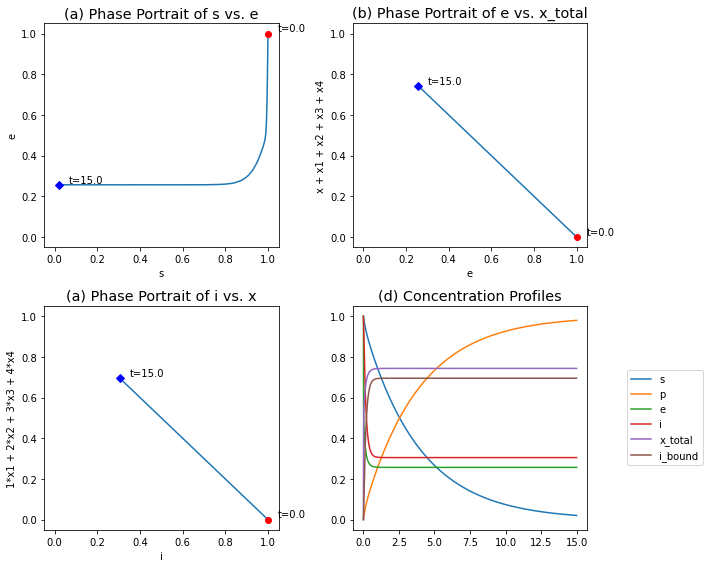

Supplement: S2 File — The latest version of the MASSpy documentation can be found at https://masspy.readthedocs.io. (ZIP) [file pcbi.1008208.s004.zip › masspy-v0.1.1/_images/education_sb2_chapters_sb2_chapter5_23_0.png]

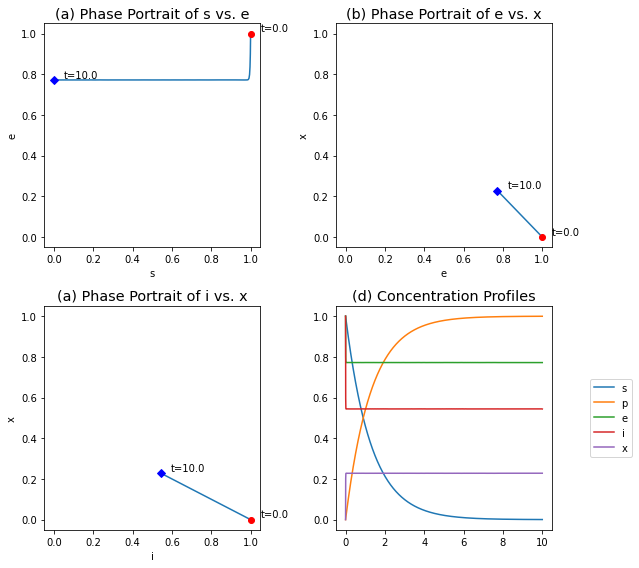

Supplement: S2 File — The latest version of the MASSpy documentation can be found at https://masspy.readthedocs.io. (ZIP) [file pcbi.1008208.s004.zip › masspy-v0.1.1/_images/education_sb2_chapters_sb2_chapter5_18_0.png]

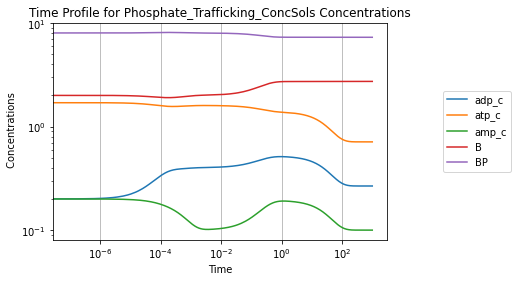

Supplement: S2 File — The latest version of the MASSpy documentation can be found at https://masspy.readthedocs.io. (ZIP) [file pcbi.1008208.s004.zip › masspy-v0.1.1/_images/tutorials_dynamic_simulation_50_0.png]

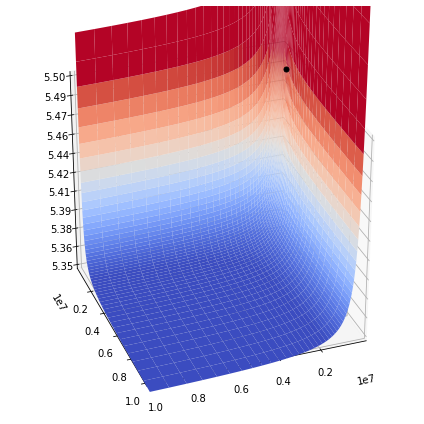

Supplement: S2 File — The latest version of the MASSpy documentation can be found at https://masspy.readthedocs.io. (ZIP) [file pcbi.1008208.s004.zip › masspy-v0.1.1/_images/education_sb2_chapters_sb2_chapter14_29_0.png]

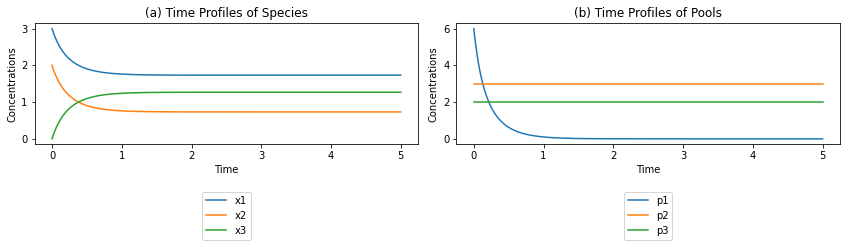

Supplement: S2 File — The latest version of the MASSpy documentation can be found at https://masspy.readthedocs.io. (ZIP) [file pcbi.1008208.s004.zip › masspy-v0.1.1/_images/education_sb2_chapters_sb2_chapter4_13_0.png]

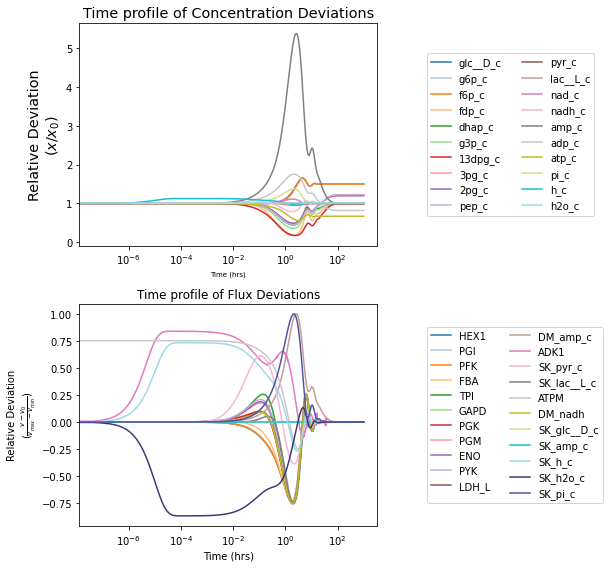

Supplement: S2 File — The latest version of the MASSpy documentation can be found at https://masspy.readthedocs.io. (ZIP) [file pcbi.1008208.s004.zip › masspy-v0.1.1/_images/tutorials_plot_visualization_25_0.png]

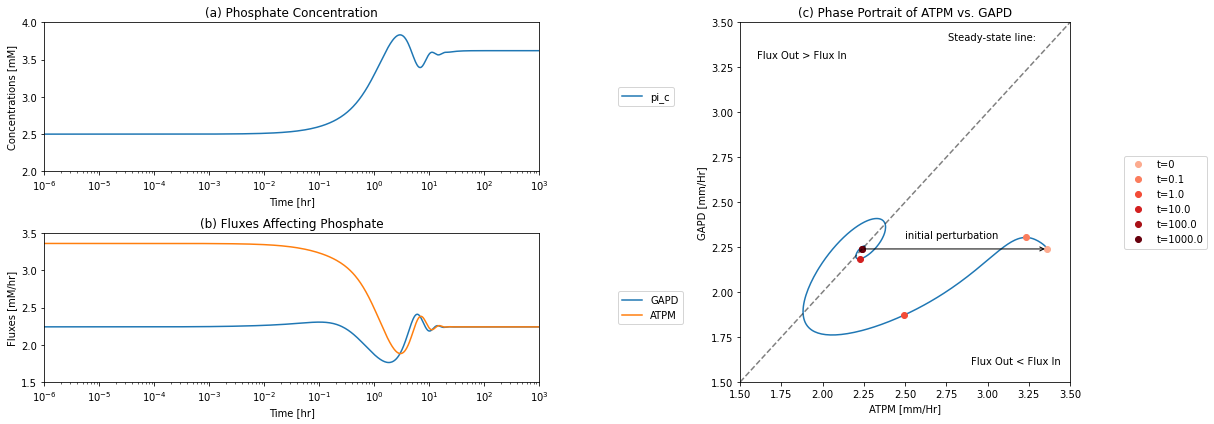

Supplement: S2 File — The latest version of the MASSpy documentation can be found at https://masspy.readthedocs.io. (ZIP) [file pcbi.1008208.s004.zip › masspy-v0.1.1/_images/education_sb2_chapters_sb2_chapter10_66_0.png]

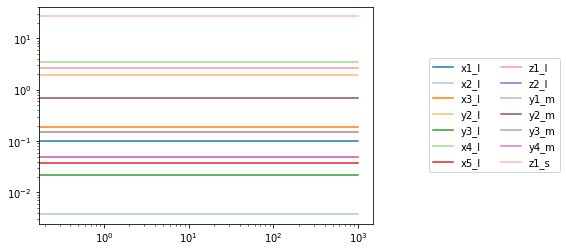

Supplement: S2 File — The latest version of the MASSpy documentation can be found at https://masspy.readthedocs.io. (ZIP) [file pcbi.1008208.s004.zip › masspy-v0.1.1/_images/tutorials_compartments_41_2.png]

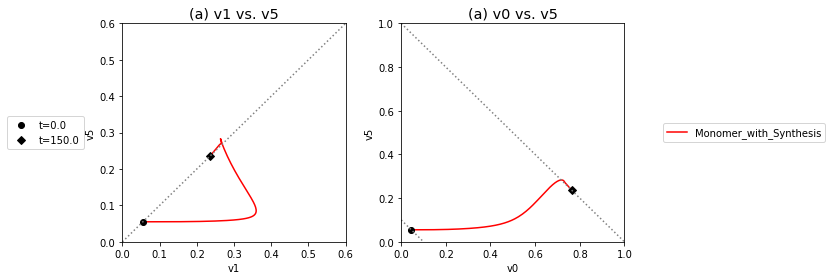

Supplement: S2 File — The latest version of the MASSpy documentation can be found at https://masspy.readthedocs.io. (ZIP) [file pcbi.1008208.s004.zip › masspy-v0.1.1/_images/education_sb2_chapters_sb2_chapter9_131_0.png]

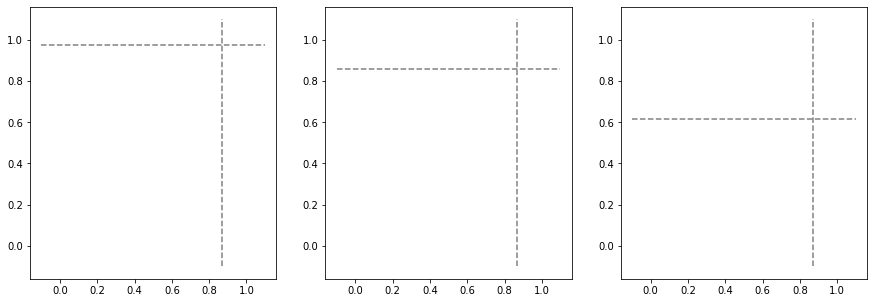

Supplement: S2 File — The latest version of the MASSpy documentation can be found at https://masspy.readthedocs.io. (ZIP) [file pcbi.1008208.s004.zip › masspy-v0.1.1/_images/gallery_visualization_catalytic_potential_visualizations_16_0.png]

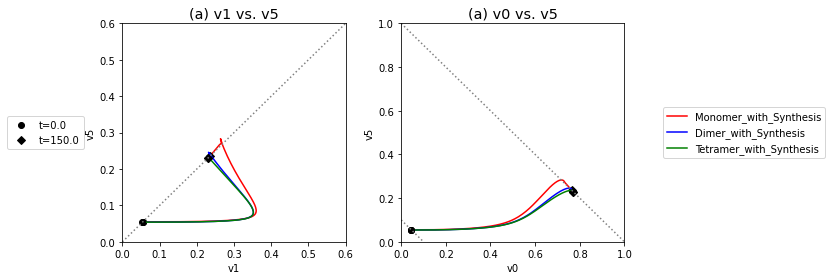

Supplement: S2 File — The latest version of the MASSpy documentation can be found at https://masspy.readthedocs.io. (ZIP) [file pcbi.1008208.s004.zip › masspy-v0.1.1/_images/education_sb2_chapters_sb2_chapter9_162_0.png]

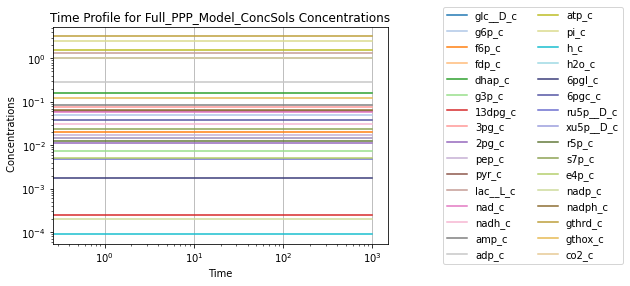

Supplement: S2 File — The latest version of the MASSpy documentation can be found at https://masspy.readthedocs.io. (ZIP) [file pcbi.1008208.s004.zip › masspy-v0.1.1/_images/education_sb2_chapters_sb2_chapter11_72_0.png]

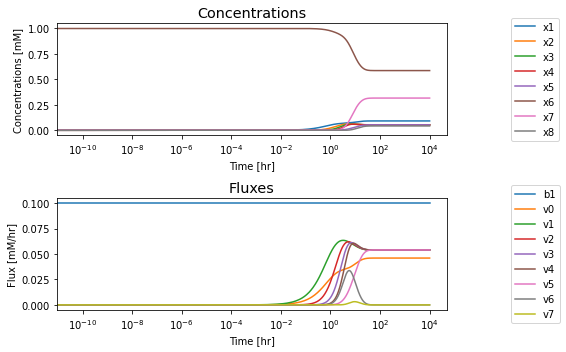

Supplement: S2 File — The latest version of the MASSpy documentation can be found at https://masspy.readthedocs.io. (ZIP) [file pcbi.1008208.s004.zip › masspy-v0.1.1/_images/education_sb2_chapters_sb2_chapter9_69_0.png]

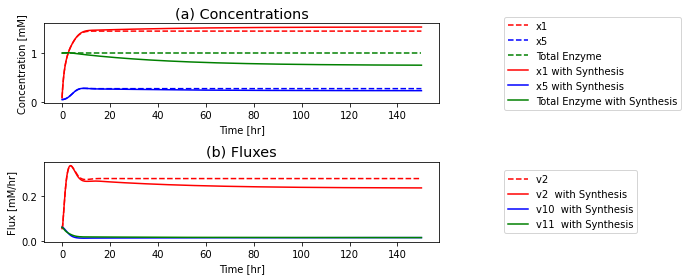

Supplement: S2 File — The latest version of the MASSpy documentation can be found at https://masspy.readthedocs.io. (ZIP) [file pcbi.1008208.s004.zip › masspy-v0.1.1/_images/education_sb2_chapters_sb2_chapter9_127_0.png]

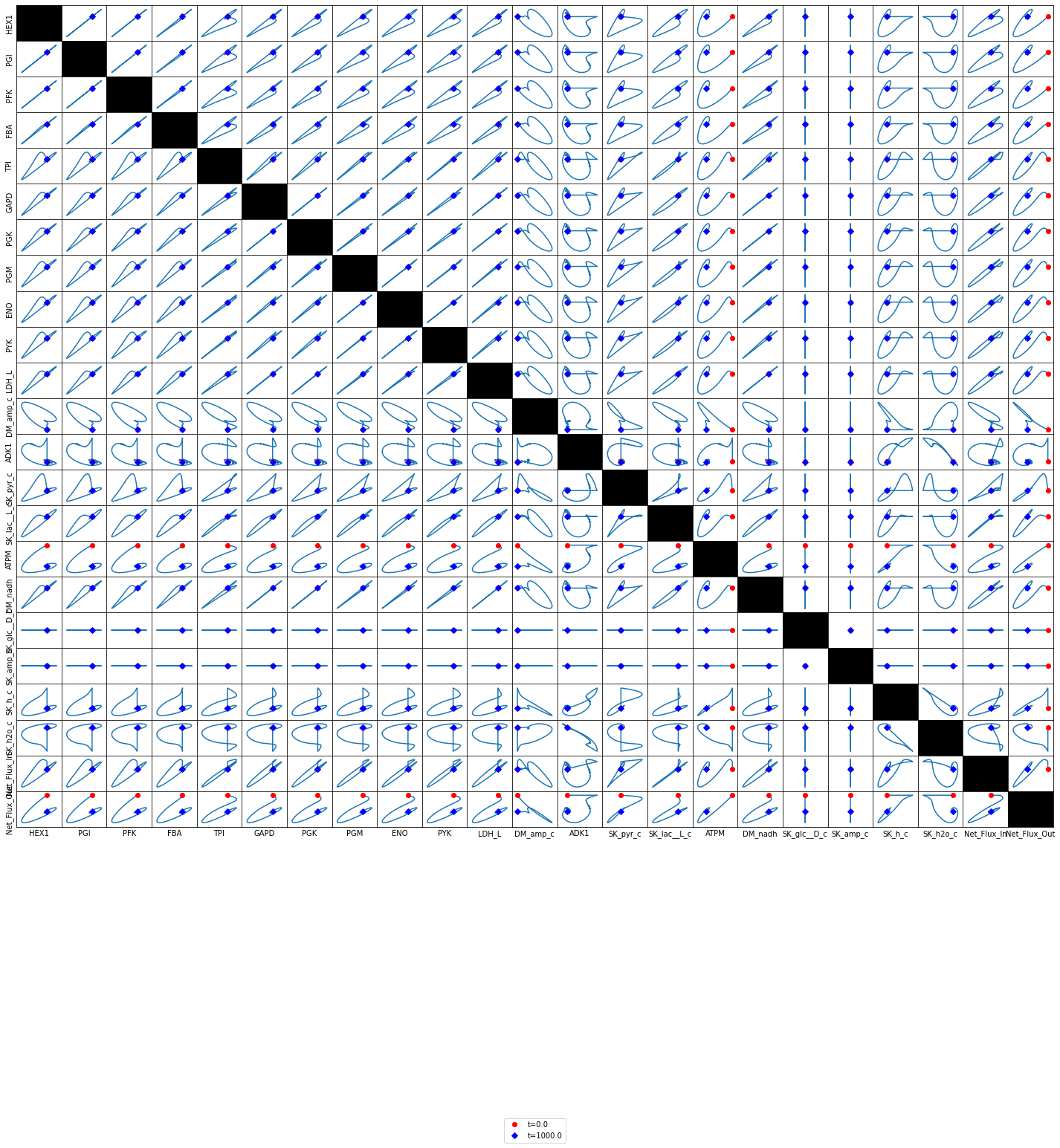

Supplement: S2 File — The latest version of the MASSpy documentation can be found at https://masspy.readthedocs.io. (ZIP) [file pcbi.1008208.s004.zip › masspy-v0.1.1/_images/education_sb2_chapters_sb2_chapter10_70_0.png]

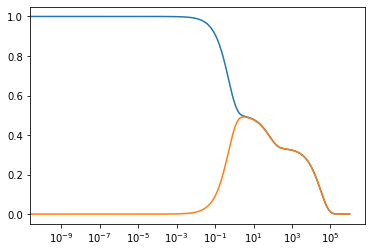

Supplement: S2 File — The latest version of the MASSpy documentation can be found at https://masspy.readthedocs.io. (ZIP) [file pcbi.1008208.s004.zip › masspy-v0.1.1/_images/education_sb2_chapters_sb2_chapter3_89_1.png]

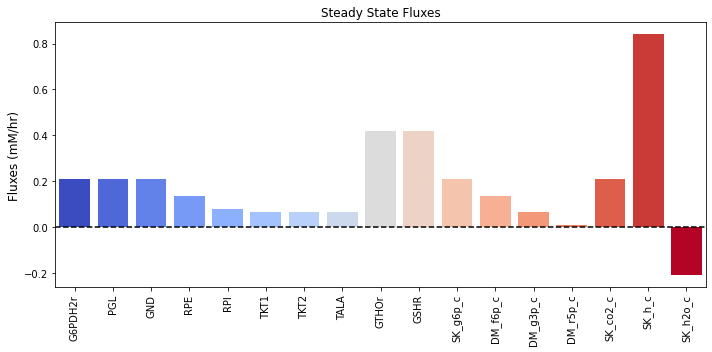

Supplement: S2 File — The latest version of the MASSpy documentation can be found at https://masspy.readthedocs.io. (ZIP) [file pcbi.1008208.s004.zip › masspy-v0.1.1/_images/education_sb2_chapters_sb2_chapter11_31_0.png]

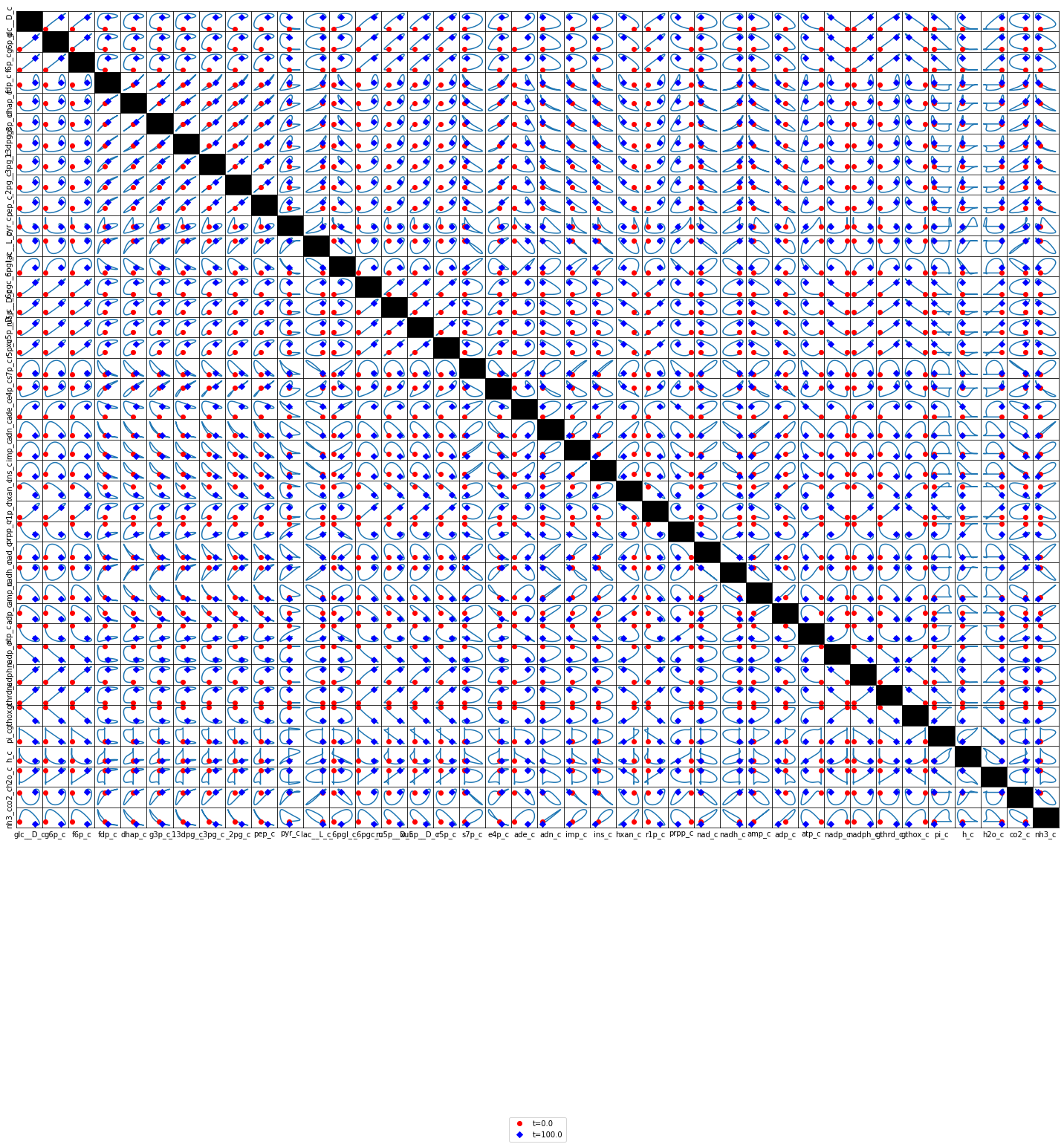

Supplement: S2 File — The latest version of the MASSpy documentation can be found at https://masspy.readthedocs.io. (ZIP) [file pcbi.1008208.s004.zip › masspy-v0.1.1/_images/education_sb2_chapters_sb2_chapter12_89_0.png]

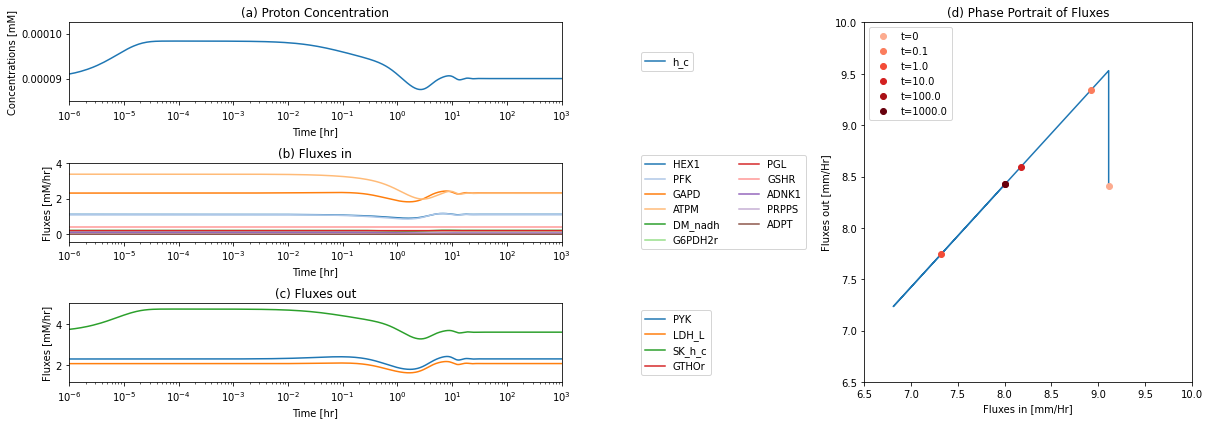

Supplement: S2 File — The latest version of the MASSpy documentation can be found at https://masspy.readthedocs.io. (ZIP) [file pcbi.1008208.s004.zip › masspy-v0.1.1/_images/education_sb2_chapters_sb2_chapter12_79_0.png]

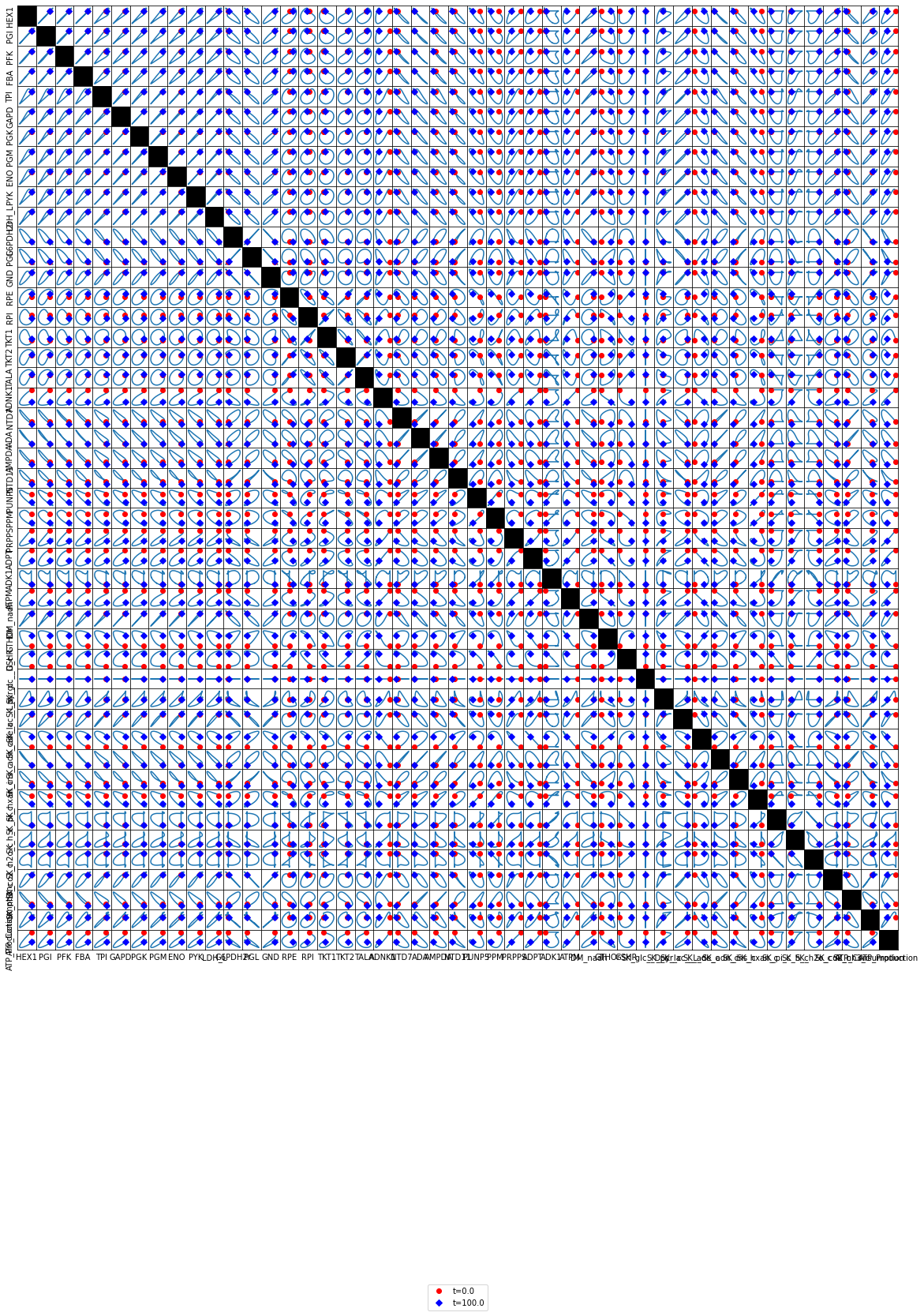

Supplement: S2 File — The latest version of the MASSpy documentation can be found at https://masspy.readthedocs.io. (ZIP) [file pcbi.1008208.s004.zip › masspy-v0.1.1/_images/education_sb2_chapters_sb2_chapter12_91_1.png]

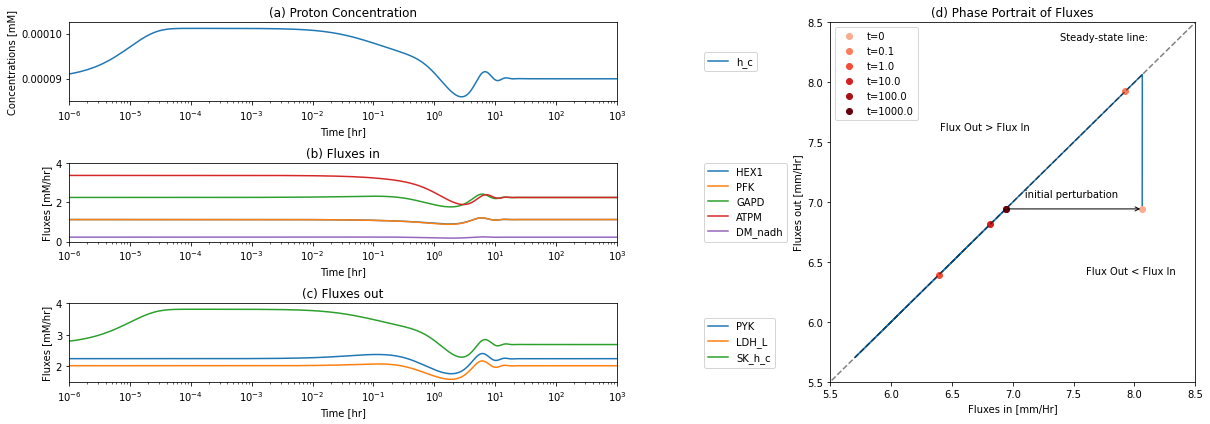

Supplement: S2 File — The latest version of the MASSpy documentation can be found at https://masspy.readthedocs.io. (ZIP) [file pcbi.1008208.s004.zip › masspy-v0.1.1/_images/education_sb2_chapters_sb2_chapter10_58_0.png]

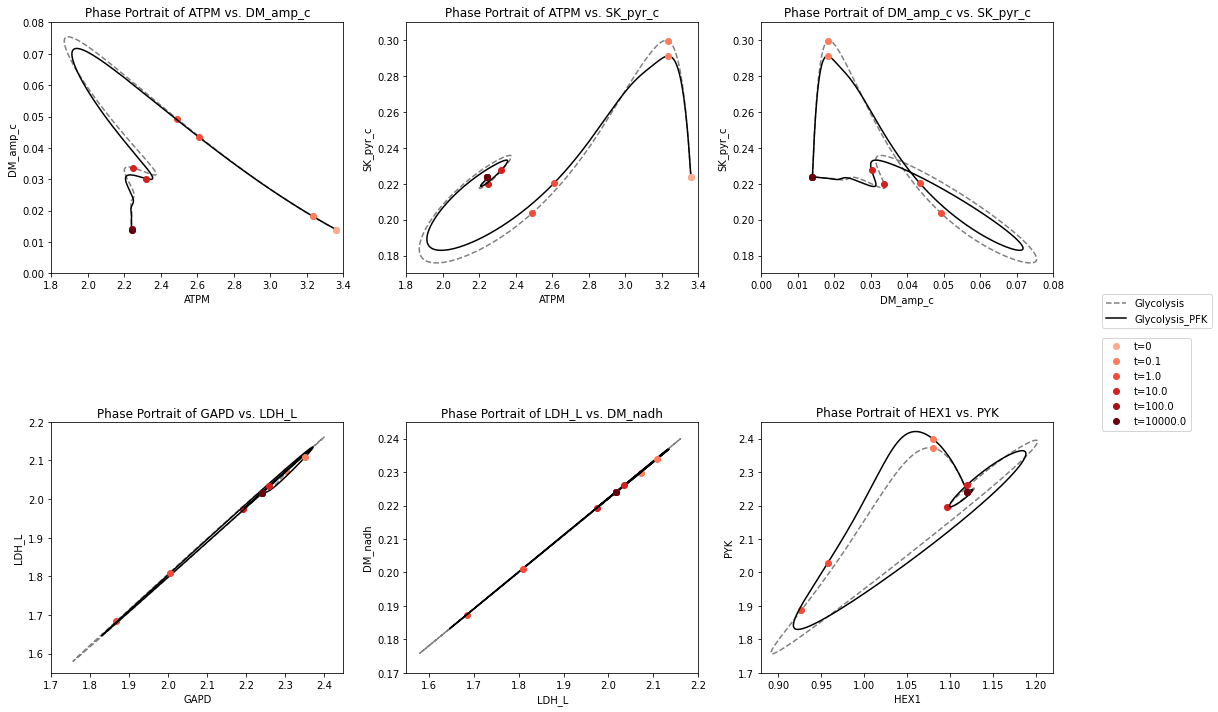

Supplement: S2 File — The latest version of the MASSpy documentation can be found at https://masspy.readthedocs.io. (ZIP) [file pcbi.1008208.s004.zip › masspy-v0.1.1/_images/education_sb2_chapters_sb2_chapter14_50_0.png]

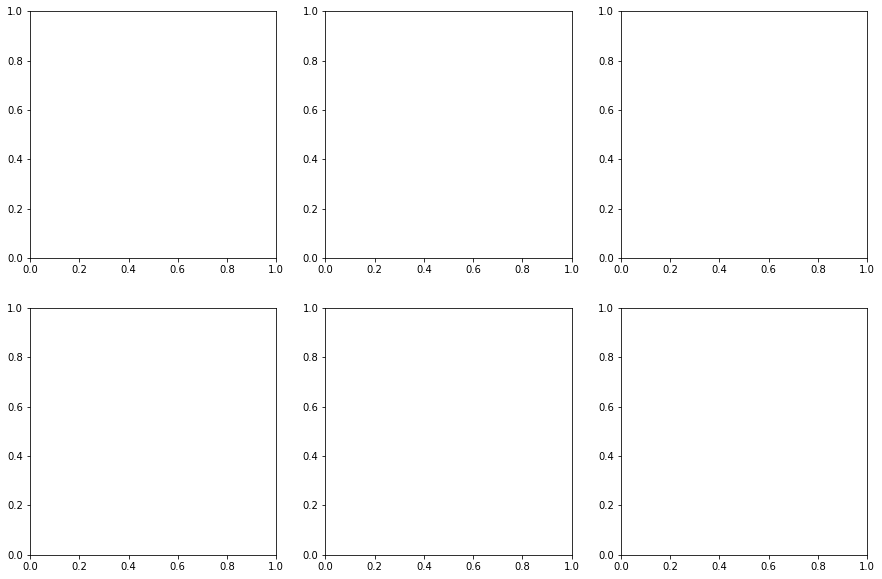

Supplement: S2 File — The latest version of the MASSpy documentation can be found at https://masspy.readthedocs.io. (ZIP) [file pcbi.1008208.s004.zip › masspy-v0.1.1/_images/gallery_visualization_catalytic_potential_visualizations_38_0.png]

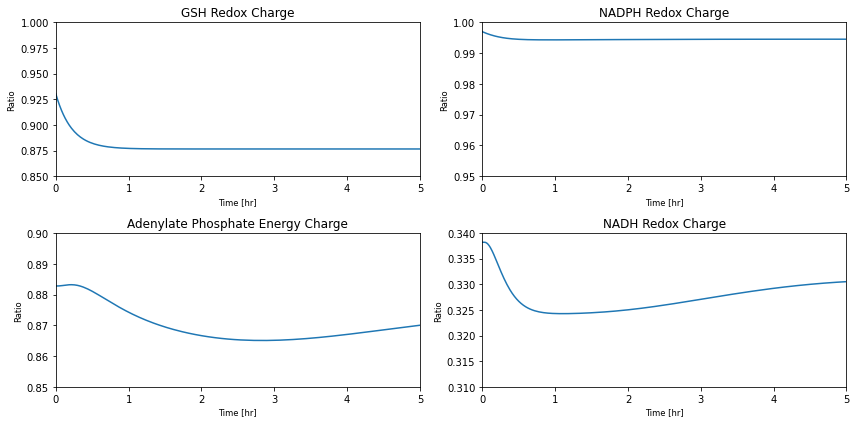

Supplement: S2 File — The latest version of the MASSpy documentation can be found at https://masspy.readthedocs.io. (ZIP) [file pcbi.1008208.s004.zip › masspy-v0.1.1/_images/education_sb2_chapters_sb2_chapter11_109_0.png]

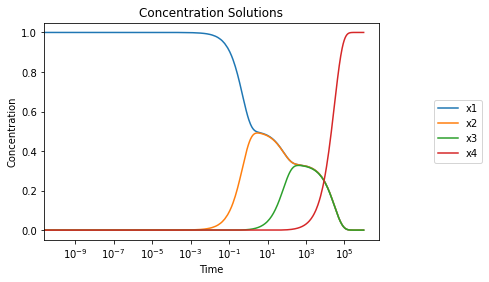

Supplement: S2 File — The latest version of the MASSpy documentation can be found at https://masspy.readthedocs.io. (ZIP) [file pcbi.1008208.s004.zip › masspy-v0.1.1/_images/education_sb2_chapters_sb2_chapter3_91_1.png]

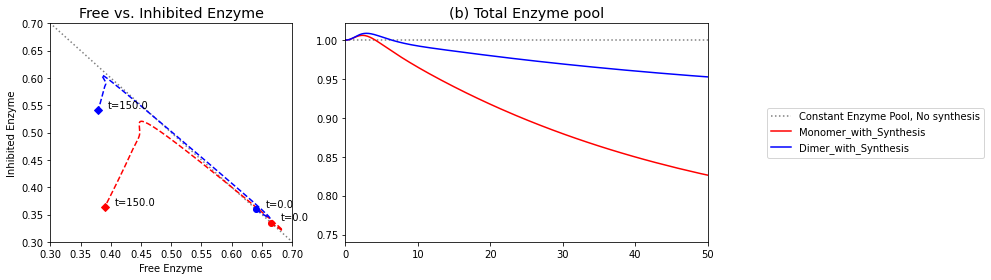

Supplement: S2 File — The latest version of the MASSpy documentation can be found at https://masspy.readthedocs.io. (ZIP) [file pcbi.1008208.s004.zip › masspy-v0.1.1/_images/education_sb2_chapters_sb2_chapter9_144_0.png]

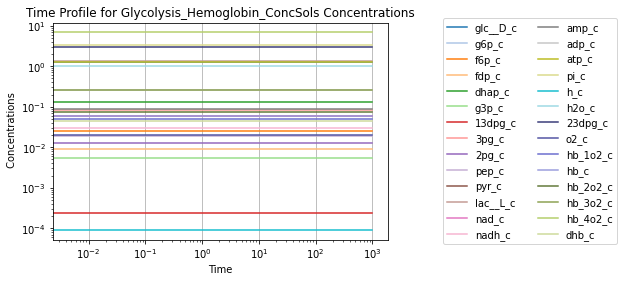

Supplement: S2 File — The latest version of the MASSpy documentation can be found at https://masspy.readthedocs.io. (ZIP) [file pcbi.1008208.s004.zip › masspy-v0.1.1/_images/education_sb2_model_construction_sb2_hemoglobin_28_1.png]

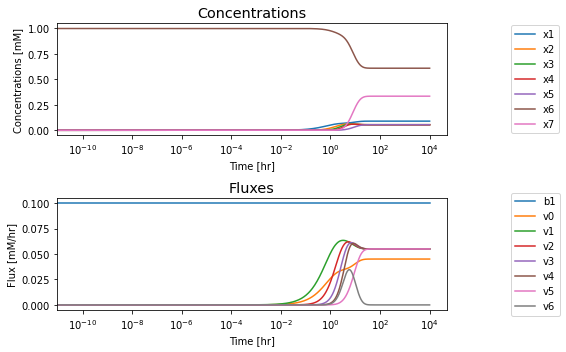

Supplement: S2 File — The latest version of the MASSpy documentation can be found at https://masspy.readthedocs.io. (ZIP) [file pcbi.1008208.s004.zip › masspy-v0.1.1/_images/education_sb2_chapters_sb2_chapter9_41_0.png]

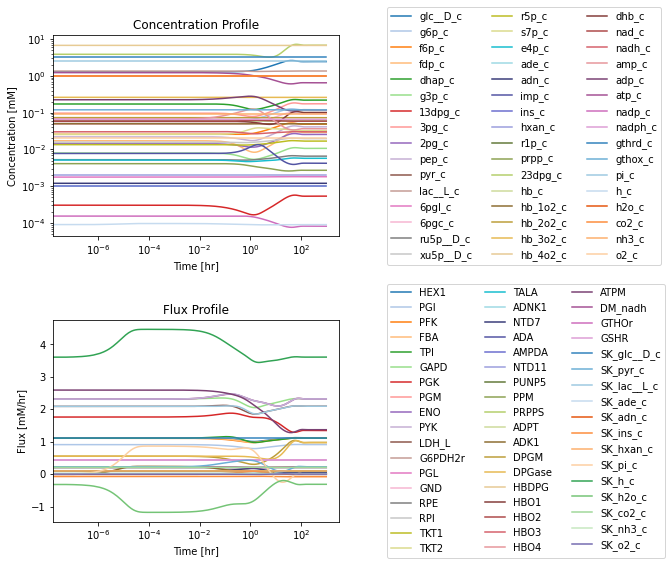

Supplement: S2 File — The latest version of the MASSpy documentation can be found at https://masspy.readthedocs.io. (ZIP) [file pcbi.1008208.s004.zip › masspy-v0.1.1/_images/education_sb2_chapters_sb2_chapter13_74_0.png]

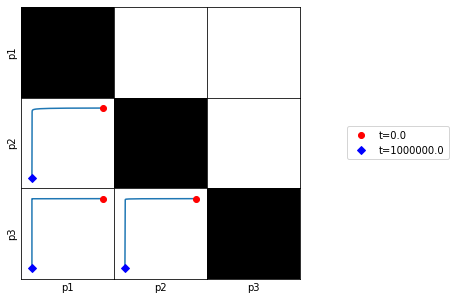

Supplement: S2 File — The latest version of the MASSpy documentation can be found at https://masspy.readthedocs.io. (ZIP) [file pcbi.1008208.s004.zip › masspy-v0.1.1/_images/education_sb2_chapters_sb2_chapter3_109_1.png]

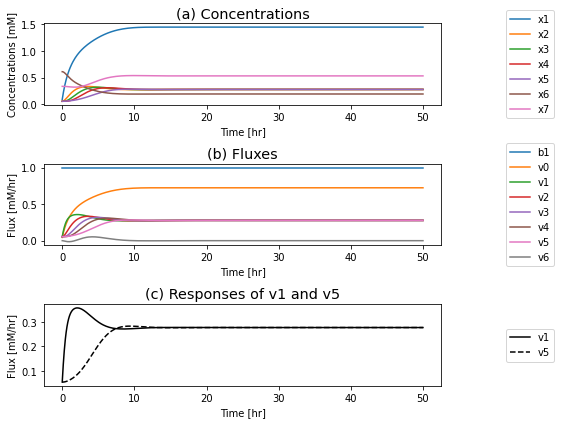

Supplement: S2 File — The latest version of the MASSpy documentation can be found at https://masspy.readthedocs.io. (ZIP) [file pcbi.1008208.s004.zip › masspy-v0.1.1/_images/education_sb2_chapters_sb2_chapter9_50_0.png]

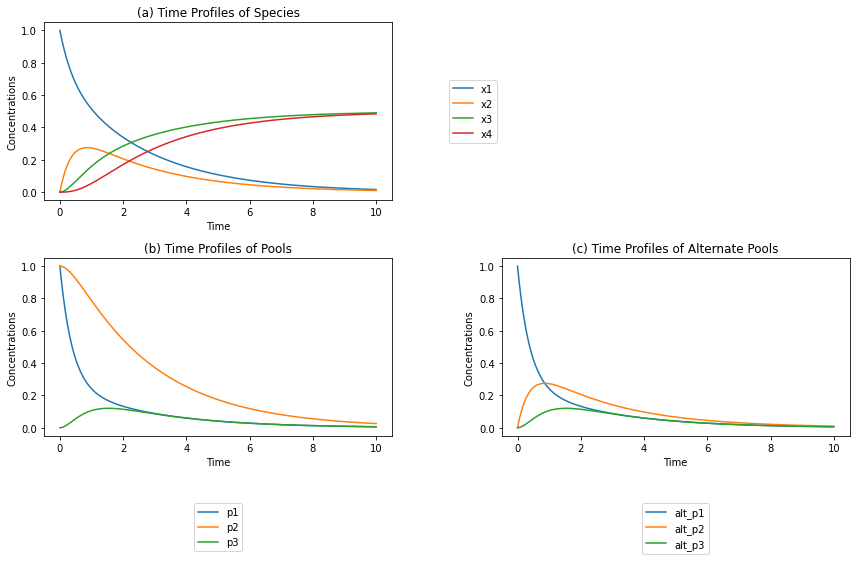

Supplement: S2 File — The latest version of the MASSpy documentation can be found at https://masspy.readthedocs.io. (ZIP) [file pcbi.1008208.s004.zip › masspy-v0.1.1/_images/education_sb2_chapters_sb2_chapter4_21_0.png]

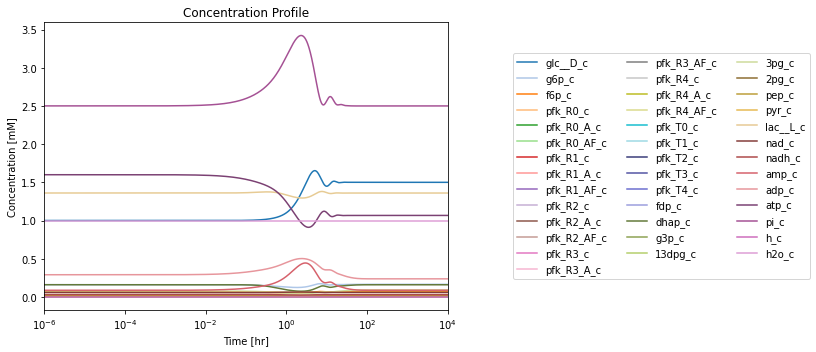

Supplement: S2 File — The latest version of the MASSpy documentation can be found at https://masspy.readthedocs.io. (ZIP) [file pcbi.1008208.s004.zip › masspy-v0.1.1/_images/education_sb2_chapters_sb2_chapter14_42_0.png]

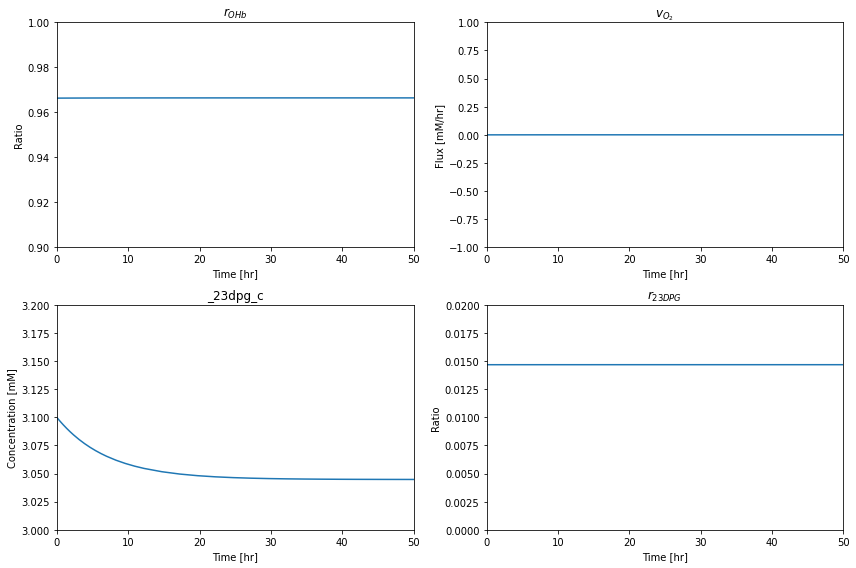

Supplement: S2 File — The latest version of the MASSpy documentation can be found at https://masspy.readthedocs.io. (ZIP) [file pcbi.1008208.s004.zip › masspy-v0.1.1/_images/education_sb2_chapters_sb2_chapter13_36_0.png]

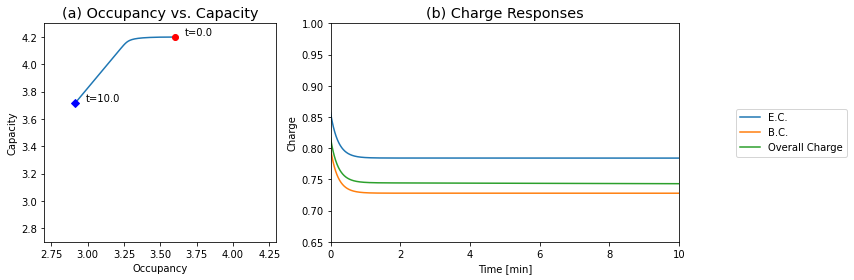

Supplement: S2 File — The latest version of the MASSpy documentation can be found at https://masspy.readthedocs.io. (ZIP) [file pcbi.1008208.s004.zip › masspy-v0.1.1/_images/education_sb2_chapters_sb2_chapter8_75_0.png]

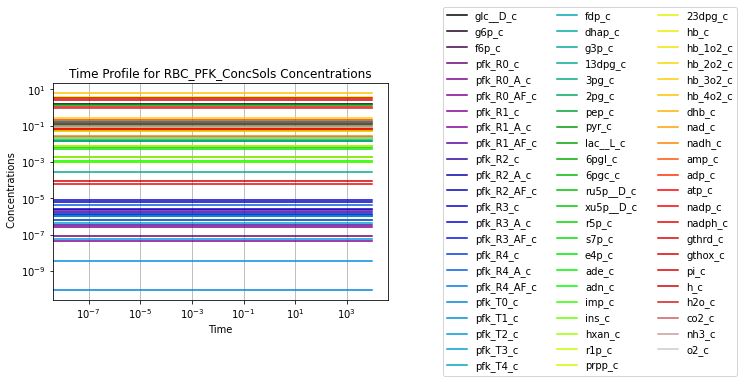

Supplement: S2 File — The latest version of the MASSpy documentation can be found at https://masspy.readthedocs.io. (ZIP) [file pcbi.1008208.s004.zip › masspy-v0.1.1/_images/education_sb2_chapters_sb2_chapter14_63_0.png]

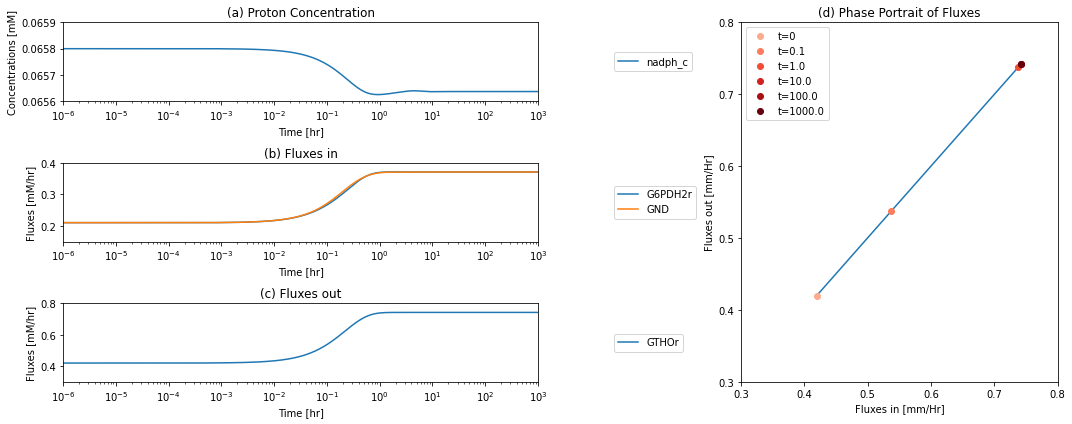

Supplement: S2 File — The latest version of the MASSpy documentation can be found at https://masspy.readthedocs.io. (ZIP) [file pcbi.1008208.s004.zip › masspy-v0.1.1/_images/education_sb2_chapters_sb2_chapter11_100_0.png]
