# Supplementary material for: MASSpy: Building, simulating, and visualizing dynamic biological models in Python using mass action kinetics
Source: PLoS Comput Biol. 2021 Jan 28;17(1):e1008208. doi: 10.1371/journal.pcbi.1008208 (PMC7872247; doi:10.1371/journal.pcbi.1008208)
Supplement: S2 File — The latest version of the MASSpy documentation can be found at https://masspy.readthedocs.io. (ZIP) [file pcbi.1008208.s004.zip › masspy-v0.1.1/index.html]

  


MASSpy 0.1.1 documentation


MASSpy

v0.1.1

Installation and Setup

- Quick Start Guide
- Optimization Solvers
- Using MASSpy with Docker
- Advanced Docker Usage

Step-by-Step Tutorials

- 1. Getting Started with MASSpy
- 2. Constructing Models
- 3. Reading and Writing Models
- 4. Dynamic Simulation of Models
- 5. Plotting and Visualization
- 6. Enzyme Modules
- 7. Thermodynamic Feasibility and Sampling of Metabolite Concentrations
- 8. Ensemble Modeling
- 9. Network Visualization
- 10. Checking Model Quality
- 11. Global Configuration
- 12. Using COBRApy with MASSpy
- 13. Modeling Volumes and Multiple Compartments
- 14. Import and Export of Optimization Problems

Gallery

- Visualization
- Workflows

Educational Resources

- Systems Biology: Simulation of Dynamic Network States

API

- API Reference

Additional Resources:

- Frequently Asked Questions (FAQs)
- Code Repositories
- Works Cited

MASSpy

- Docs »
- MASSpy 0.1.1 documentation
- Edit on GitHub

---

# MASSpy: Modeling Dynamic Biological Processes in Python¶

## Welcome to MASSpy’s Documentation!¶

The **M**ass **A**ction **S**toichiometric **S**imulation **py**thon
(**MASSpy**) package contains modules for the construction, simulation, and
analysis of kinetic models of biochemical reaction systems.

**MASSpy** is built to integrate seamlessly with **COBRApy** [ELPH13], a widely used
modeling software package for constraint-based reconstruction and analysis of
biochemical reaction systems. **MASSpy** can be used separately from
or in conjunction with **COBRApy**, providing a vast assortment of modeling
techniques and tools that enable different workflows. Additional information about
**COBRApy** can be found in its
documentation or
GitHub page.

### Citation¶

A manuscript is in preparation for publication and will be the proper reference for citing the MASSpy software package in the future.
In the meantime, feel free to cite the preprint [HZK+20], which can be found at bioRxiv.

> The code and instsructions to reproduce the results presented in the publication is located
> in the MASSpy-publication GitHub Repository.

## Installation and Setup¶

There are various ways to get started with the **MASSpy** package. The guides below provide instructions on how to set up a **MASSpy** environment best suited to your needs.

**Quick Start Guide**:
:   Ready to dive into **MASSpy** right away? Check out the Quick Start Guide.

**Optimization Solvers**:
:   In order to utilize certain **MASSpy** features, additional optimization capabilities (e.g., quadratic programming) are necessary.
    Read more at Optimization Solvers.

**Docker Containers**:
:   Need a standardized, ready-to-deploy container for your project? Learn how to set up Using MASSpy with Docker for **MASSpy**.

### Quick Start Guide¶

To quickly get started with the latest version of MASSpy, check out the information below!

#### With Python¶

The recommended method is to install **MASSpy** is to use `pip` to
install the software from the Python Package Index.
It is recommended to do this inside a virtual environment):

```
pip install masspy
```

#### With Docker¶

To quickly get started with the latest version of MASSpy using Docker, run the following commands in a shell:

```
docker pull sbrg/masspy
docker run --rm \
    --mount type=volume,src=licenses,dst=/home/masspy_user/opt/licenses \
    --mount type=volume,src=mass_project,dst=/home/masspy_user/mass_project \
    --publish 8888:8888 \
    -it sbrg/masspy
```

From within the container, either run `python` or `jupyter notebook --ip=0.0.0.0 --port=8888` depending on
the desired Python workspace.

#### Optimization in MASSpy¶

By default, **MASSpy** comes with the GLPK solver. However, specific features of
**MASSpy** require a commercial optimization solver with additional solving capabilities. For more information, check out the
section on Optimization Solvers.

### Optimization Solvers¶

**MASSpy** utilizes Optlang as a common interface for different optimization solvers.
By default, **MASSpy** will come with swiglpk, an interface to the open source (mixed integer)
linear programming (LP) solver GLPK. However, in order to utilize specific **MASSpy** features,
a (mixed integer) quadratic programming (QP) solver are necessary.

The following features require QP support:

- Concentration solution space sampling

The following optional solvers are currently supported by **Optlang**:

#### IBM ILOG CPLEX Optimization Studio¶

The *IBM ILOG CPLEX Optimization Studio* (CPLEX) can be utilized through the
CPLEX Python API.

- To use CPLEX, a license must be obtained. Free academic licences are available.
- To use CPLEX with Docker, an installer file must also be downloaded.

  Homepage |
  Documentation |
  Academic License

#### Gurobi Optimizer¶

The *Gurobi Optimizer* (Gurobi) is utilized through the Gurobi Python Interface.

- To use Gurobi, a license must be obtained. Free academic licences are available.
- To use Gurobi with Docker, a floating license is required.

  Homepage |
  Documentation |
  Academic License |
  Floating License

#### Working with other solvers¶

Prefer to work with a different optimization solver? It is possible to import/export optimization problems for use in other solvers.
Read more at Import and Export of Optimization Problems.

### Using MASSpy with Docker¶

**MASSpy** comes in deployable Docker container, allowing for quick access
to an isolated Python environment prepackaged the MASSpy software, all within a virtual machine that can run
in a variety of environments.

The following guide demonstrates how to setup a Docker container for **MASSpy**. It assumes that the Docker Daemon and Client have
already been installed. The guide can be broken down into three key steps:

> 1. Obtaining the image: An image for the MASSpy Docker container must be either obtained from an online registry or by built.
> 2. Creating the container: Once obtained, a container must be created from the image.
> 3. Running MASSpy with the container: After the container is built, the final step is to run the container and get started using MASSpy!

**Important:** In order to use the *Gurobi Optimizer* or the *IBM ILOG CPLEX Optimization Studio*, the Docker image must be built locally
from a Dockerfile and a “context” containing certain files. See the secion below on Building the image.

About Docker
:   Interested in learning more about Docker? Read more about containerization and getting started with Docker in the
    Docker Quick Start in the official Docker documentation.

#### Obtaining the image¶

An image for a MASSpy Docker container can be either be downloaded from an online registry, or it
can be built from a Dockerfile and the proper build “context”.

> - The recommended method to obtain a MASSpy image is to download an image from an online registry.
> - To enable the use of a commercial optimization solver (e.g., Gurobi, CPLEX) inside the container, the
>   MASSpy image must be built locally.

##### Downloading the image¶

Images for the MASSpy software are be found in the following registries:

SBRG DockerHub :
:   - **Image Name**: `sbrg/masspy`
    - **Tags**: A full list of tags can be found here

To pull the MASSpy image `sbrg/masspy`, run the following in a shell:

```
docker pull sbrg/masspy
```

A tag must be included in order to download a specific image version. For example, to pull the `sbrg/masspy` image with the `latest` tag:

```
docker pull sbrg/masspy:latest
```

By default, the `latest` version of MASSpy image is pulled from the registry.

##### Building the image¶

**Build Context**: The following directory stucture shows the minimal requirements needed as context when building the image:

```
MASSpy               # Source directory
└── docker           # Root directory for build context
    └── Dockerfile   # Dockerfile from VCS (https://github.com/SBRG/MASSpy)
```

To build the image with tag `latest`, navigate to the `MASSpy` directory and use the command line:

```
docker build -t sbrg/masspy:latest ./docker
```

**Windows Users:** Please note the following issue about running linux containers using Docker for Windows.

###### Including ILOG CPLEX Optimization Studio 12.10¶

To utilize the ILOG CPLEX Optimization Studio in a Docker container, a license must be obtained first.
See IBM ILOG CPLEX Optimization Studio for more information on obtaining an academic license.

Once a CPLEX license has been obtained:

> 1. Download the installer `cplex_studioXXXX.linux-x86-64.bin` from CPLEX, replacing “XXXX”
>    for the version number without punctuation (e.g., 1210).
> 2. Place the installer into the `cplex` directory in the build context as outlined below.
> 3. Place the file `cplex.install.properties` into the build context to accept the license
>    agreement and to enable silent install.

Note

The CPLEX installer must be for **LINUX** to be compatible with the containers built using the
MASSpy Dockerfile.

**Build Context**: To include CPLEX, the build context must be modified to contain the `cplex` subdirectory as follows:

```
MASSpy
└── docker
    ├── Dockerfile
    └── cplex
        ├── cplex_studio1210.linux-x86-64.bin
        └── cplex.install.properties
```

###### Including Gurobi Optimizer 9.0.3¶

To utilize the Gurobi Optimizer in a Docker container, a floating license
must be obtained first. See Gurobi Optimizer for more information on obtaining a floating license.

Once a floating Gurobi license has been obtained:

> 1. Copy the gurobi.lic.template and
>    rename the file `gurobi.lic`.
> 2. Modify the license file according to the
>    Gurobi documentation.
> 3. Place the license file into the `gurobi` directory in the build context as outlined below.

**Build Context**: To include Gurobi, the build context must be modified to contain the `gurobi` subdirectory as follows:

```
MASSpy
└── docker
    ├── Dockerfile
    └── gurobi
        └── gurobi.lic
```

###### Additional information¶

For more information about the build context for the MASSpy image, see the Recognized Image Build Context section.

#### Creating the container¶

Once the MASSpy image is obtained, the next step is to run the image as a container using the following command:

```
docker run \
    --name mass-container \
    --mount type=volume,src=mass_project,dst=/home/masspy_user/mass_project \
    --publish 8888:8888 \
    -it sbrg/masspy:latest
```

To break down the above command:

> - –name :
>   :   The `--name` flag sets an optional name for the container that can be used to reference the container
>       with the Docker Client. Here, the container is named `mass-container`.
> - –mount :
>   :   The `--mount` flag creates a volume to allow data to persist even after a container has been stopped.
>       In this particular example, a mount of type `volume` called `mass_project` is mounted to the container at
>       the location `/home/masspy_user/mass_project`. Not required for use, but highly recommended.
> - –publish :
>   :   The `--publish` flag publishes the container’s port `8888`, binding it to the host port at `8888`.
>       Required to utilize Jupyter (iPython) notebooks from inside the container.
> - -it :
>   :   Allocate a pseudo-TTY and create an interactive shell in the container.

If optimization solvers are included when building the image, it is recommended to mount the `licenses` volume
as well. This can be done via the following:

```
docker run \
    --name mass-container \
    --mount type=volume,src=licenses,dst=/home/masspy_user/opt/licenses \
    --mount type=volume,src=mass_project,dst=/home/masspy_user/mass_project \
    --publish 8888:8888 \
    -it sbrg/masspy:latest
```

Note

Containers names must be unique. To re-use a name for a new container, the previous container must first be removed.

##### Running MASSpy with the container¶

Once a container has been started with an interactive shell allocated ( the `-it` flag ), either a Jupyter (iPython)
notebook or Python itself can be started by running one of the following from the shell within the container

> - To start python, run `python`
> - To start a Jupyter notebook, run `jupyter notebook --ip=0.0.0.0 --port=8888`.

To stop the inteactive shell and exit the container, run the `exit` command.

##### Resuming the container¶

To resume the container `mass-container` after it has been stopped:

```
docker start -i mass-container
```

##### Cleanup¶

To remove the container `` `mass-container `` entirely:

```
docker rm mass-container
```

To remove the image `sbrg/masspy:latest` entirely:

```
docker rmi sbrg/masspy:latest
```

#### Troubleshooting¶

Need help trouble shooting Docker for your system? Try searching the official Docker resources:

> Docker CE for Linux |
> Docker Desktop for Mac |
> Docker Desktop for Windows

### Advanced Docker Usage¶

This page contains additional information about the MASSpy Docker image and container.

#### Recognized Image Build Context¶

The directory structure below outlines the expected build context with all optional aspects included when building a
Docker image:

```
MASSpy                   # Source directory
└── docker               # Root directory for build context
    ├── Dockerfile
    ├── cplex
    │   ├── cplex_studio1210.linux-x86-64.bin
    │   └── cplex.install.properties
    ├── gurobi
    │   └── gurobi.lic
    └── docker-entrypoint.sh
```

The MASSpy image only requires the Dockerfile in its “context” to be built. Anything else is optional and will add specific funtionality
as outlined below:

**Dockerfile** :
:   The MASSpy Dockerfile required to build the image.

**cplex** :
:   Directory used to install IBM CPLEX Optimization studio 12.10

    - **cplex\_studio1210.linux-x86-64.bin**:
      :   The installer binary for CPLEX. The presence of this file triggers the CPLEX installation process.
    - **cplex.install.properties**:
      :   Installer properties for CPLEX. Acecpts license agreement and sets silent install. Ignored if no installer exists in build context.

**gurobi** :
:   Directory used to install Gurobi Optimizer 9.0.3

    - **gurobi.lic**:
      :   Gurobi license file. The presence of this file triggers the Gurobi installation process.
    - **gurobi.lic.template**:
      :   Template for Gurobi license.
          Can be included to configure the token client license at a later point from within the container.

**docker-entrypoint.sh** :
:   A shell script for the container entrypoint to replace
    the customize the standard docker entrypoint behavior. Must be named `docker-entrypoint.sh` to work.

#### Build-time variables¶

Certain build-time variables are set and passed as arguments
when building the image. Build-time variables are passed to `--build-arg` flag in the form of `VARIABLE=VALUE`.
All build-args are optional and are not required to be defined at the time when the image is built.

The following build-time variables can be utilized by the MASSpy Dockerfile at the time of build:

**verbose**
:   Integer 0 or 1 determining whether to include additional output as the image builds.
    Can be either the value `0` to disabled verbosity, or `1` to enabled it.
    Primarily for debugging purposes. Default value is `0`.

**python\_version**
:   Indicates python base image to use. Must be Python 3.6+. Default is `3.7`.

**mass\_version**
:   The branch or tagged version of MASSpy to use in the Docker container. Value will be passed to `git checkout`. Must be one of the following:

    - A branch on the MASSpy GitHub Repository.
    - `{MAJOR}.{MINOR}.{PATCH}` to use a specific version of MASSpy.

    Default is `latest` to use the latest stable release (master branch) of MASSpy.

An example build command using all of the build-time variables:

```
docker build \
    --build-arg python_version=3.7 \
    --build-arg mass_version=latest \
    --build-arg verbose=0 \
    -t sbrg/masspy:latest ./docker
```

#### Using a local installation of MASSpy¶

To use the local installation of MASSpy when building the docker image, navigate to the directory containing the local installation of MASSpy
and run the following build command:

```
docker build \
    --build-arg mass_version=local \
    -t sbrg/masspy:local \
    -f ./docker/Dockerfile ./
```

The resulting image `sbrg/masspy:local` can then be used to build a container using `docker run`.
Note that will install the local version of **MASSpy** in editable mode.

Once MASSpy is installed, check out the step-by-step tutorials below to learn how to use **MASSpy**!

### Getting Started with MASSpy¶

In this notebook example, objects essential to **MASSpy** are explored.

#### Models¶

In **MASSpy**, a model is represented by a `mass.MassModel` object. **MASSpy** comes bundled with example models, including a “textbook” model\(^1\) of human red blood cell metabolism. To load this test model:

```
[1]:
```

```
from operator import attrgetter

import mass
import mass.test

model = mass.test.create_test_model("textbook")
```

Several attributes of the `MassModel`, including model reactions and metabolites, are special types of lists that contain objects related to the model. Each specialized list is called a `cobra.DictList` and is made up of the corresponding objects. For example, the `reactions` attribute contains the `mass.MassReaction` objects, and the `metabolites` attribute contains the `mass.MassMetabolite` objects.

```
[2]:
```

```
print("Number of metabolites: " + str(len(model.metabolites)))
print("Number of reactions: " + str(len(model.reactions)))
```

```
Number of metabolites: 68
Number of reactions: 76
```

When using a Jupyter notebook, this type of information is rendered as a table.

```
[3]:
```

```
model
```

```
[3]:
```

|  |  |
| --- | --- |
| **Name** | RBC\_PFK |
| **Memory address** | 0x07fb49dd1f6d0 |
| **Stoichiometric Matrix** | 68x76 |
| **Matrix Rank** | 63 |
| **Number of metabolites** | 68 |
| **Initial conditions defined** | 68/68 |
| **Number of reactions** | 76 |
| **Number of genes** | 0 |
| **Number of enzyme modules** | 1 |
| **Number of groups** | 16 |
| **Objective expression** | 0 |
| **Compartments** | Cytosol |

Just like a regular list, objects in a `cobra.DictList` can be retrieved by index. For example, to get the 30th reaction in the model (at index 29 because of 0-indexing):

```
[4]:
```

```
model.reactions[29]
```

```
[4]:
```

|  |  |
| --- | --- |
| **Reaction identifier** | DPGase |
| **Name** | Diphosphoglycerate phosphatase |
| **Memory address** | 0x07fb49dd6b350 |
| **Subsystem** | Hemoglobin |
| **Kinetic Reversibility** | False |
| **Stoichiometry** | \_23dpg\_c + h2o\_c --> \_3pg\_c + pi\_c  2,3-Disphospho-D-glycerate + H2O --> 3-Phospho-D-glycerate + Phosphate |
| **GPR** |  |
| **Bounds** | (-1000.0, 1000.0) |

Items also can be retrieved by their `id` attribute using the `cobra.DictList.get_by_id()` method. For example, to get the cytosolic atp metabolite object with identifier “atp\_c”:

```
[5]:
```

```
model.metabolites.get_by_id("atp_c")
```

```
[5]:
```

|  |  |
| --- | --- |
| **MassMetabolite identifier** | atp\_c |
| **Name** | ATP |
| **Memory address** | 0x07fb49dd34350 |
| **Formula** | C10H12N5O13P3 |
| **Compartment** | c |
| **Initial Condition** | 1.2338626826140733 |
| **In 16 reaction(s)** | PFK\_T1, PGK, HEX1, PFK\_T3, PFK\_R41, PFK\_R31, PRPPS, ADNK1, PYK, PFK\_R11, ADK1, PFK\_T4, PFK\_R21, ATPM, PFK\_T2, PFK\_R01 |

If care is taken when assigning object identifiers (e.g., does not start with a number, does not contain certain characters such as “-“), it is possible to access objects inside of a `cobra.DictList` as if they were attributes.

```
[6]:
```

```
print(model.reactions.DPGase)
```

```
DPGase: _23dpg_c + h2o_c --> _3pg_c + pi_c
```

To ensure all identifiers comply with Systems Biology Markup Language (SBML) and allow for interactive use, utilizing the identifiers from the BiGG Models database is highly recommended.

Guidelines for BiGG identifiers are found here.

#### Reactions¶

In **MASSpy**, a reaction is represented by a `mass.MassReaction` object. A particular reaction can be retrieved by its `id` using the `cobra.DictList.get_by_id()` method. Below, the reaction with identifier “PGI” is inspected.

```
[7]:
```

```
PGI = model.reactions.get_by_id("PGI")
PGI
```

```
[7]:
```

|  |  |
| --- | --- |
| **Reaction identifier** | PGI |
| **Name** | Glucose-6-phosphate isomerase |
| **Memory address** | 0x07fb49dd45cd0 |
| **Subsystem** | Glycolysis |
| **Kinetic Reversibility** | True |
| **Stoichiometry** | g6p\_c <=> f6p\_c  D-Glucose 6-phosphate <=> D-Fructose 6-phosphate |
| **GPR** |  |
| **Bounds** | (-1000.0, 1000.0) |

The full name and the chemical reaction are viewed as strings. If defined, the flux value for the reaction at steady state also can be viewed.

```
[8]:
```

```
print(PGI.name)
print(PGI.reaction)
print(PGI.steady_state_flux)
```

```
Glucose-6-phosphate isomerase
g6p_c <=> f6p_c
0.9098871145647632
```

The symbolic rate equation for the reaction is viewed using the `rate` attribute. The rate is returned as a **SymPy** symbolic expression.

```
[9]:
```

```
print(PGI.rate)
```

```
kf_PGI*(g6p_c(t) - f6p_c(t)/Keq_PGI)
```

The above rate is considered a Type 1 rate law because of the reaction parameters used. There are three types of mass action rate laws that can be generated; \* Type 1 rates utilize the forward rate and equilibrium constants. \* Type 2 rates utilize the forward rate and reverse rate constants. \* Type 3 rates utilize the equilibrium and reverse rate constants.

To view the reaction rate as a Type 2 rate equation, the `get_mass_action_rate()` method can be used.

```
[10]:
```

```
print(PGI.get_mass_action_rate(rate_type=2))
```

```
kf_PGI*g6p_c(t) - kr_PGI*f6p_c(t)
```

Because the reaction has its `reversible` attribute set as `True`, the net mass action rate contains both forward and reverse rate components.

```
[11]:
```

```
print("Reversible: {0!r}".format(PGI.reversible))
print("Forward rate: {0!r}".format(
    PGI.get_forward_mass_action_rate_expression(rate_type=2)))
print("Reverse rate: {0!r}".format(
    PGI.get_reverse_mass_action_rate_expression(rate_type=2)))
print("Net reaction rate: {0!r}".format(
    PGI.get_mass_action_rate(rate_type=2)))
```

```
Reversible: True
Forward rate: kf_PGI*g6p_c(t)
Reverse rate: kr_PGI*f6p_c(t)
Net reaction rate: kf_PGI*g6p_c(t) - kr_PGI*f6p_c(t)
```

To view the defined parameters for the rate and equilibrium constants, the `parameters` attribute is used to return a `dict` containing the parameter identifiers and their values.

```
[12]:
```

```
PGI.parameters
```

```
[12]:
```

```
{'kf_PGI': 2961.1111111111486, 'Keq_PGI': 0.41}
```

Parameter identifiers for a reaction can be obtained using various attributes.

```
[13]:
```

```
print(PGI.flux_symbol_str)
print(PGI.kf_str)
print(PGI.Keq_str)
print(PGI.kr_str)
```

```
v_PGI
kf_PGI
Keq_PGI
kr_PGI
```

Changing the `reversible` attribute of the reaction affects the net rate equation:

```
[14]:
```

```
PGI.reversible = False
print("Reversible: {0!r}".format(PGI.reversible))
print("Net reaction rate: {0!r}".format(PGI.rate))
```

```
Reversible: False
Net reaction rate: kf_PGI*g6p_c(t)
```

Note that changing the `reversible` attribute of the reaction can affect the parameters:

```
[15]:
```

```
PGI.parameters
```

```
[15]:
```

```
{'kf_PGI': 2961.1111111111486, 'Keq_PGI': inf}
```

The reaction can be checked for whether it is mass balanced using the `check_mass_balance()` method. This method returns the elements that violate mass balance. If it comes back empty, then the reaction is mass balanced.

```
[16]:
```

```
PGI.check_mass_balance()
```

```
[16]:
```

```
{}
```

The `add_metabolites()` method can be used to add metabolites to a reaction by passing in a `dict` that contains the `MassMetabolite` objects and their coefficients:

```
[17]:
```

```
PGI.add_metabolites({model.metabolites.get_by_id("h_c"): -1})
print(PGI)
```

```
PGI: g6p_c + h_c --> f6p_c
```

The reaction is no longer mass balanced.

```
[18]:
```

```
PGI.subtract_metabolites({model.metabolites.get_by_id("h_c"): -1})
print(PGI)
print(PGI.check_mass_balance())
```

```
PGI: g6p_c --> f6p_c
{}
```

#### Metabolites¶

In **MASSpy**, a metabolite is represented by a `MassMetabolite` object. A particular metabolite can be retrieved by its `id` using the `cobra.DictList.get_by_id()` method. Below, the cytosolic glucose 6-phosphate metabolite with identifier “g6p\_c” is inspected.

```
[19]:
```

```
g6p_c = model.metabolites.get_by_id("g6p_c")
g6p_c
```

```
[19]:
```

|  |  |
| --- | --- |
| **MassMetabolite identifier** | g6p\_c |
| **Name** | D-Glucose 6-phosphate |
| **Memory address** | 0x07fb49dd1f7d0 |
| **Formula** | C6H11O9P |
| **Compartment** | c |
| **Initial Condition** | 0.16501847288094948 |
| **In 3 reaction(s)** | G6PDH2r, HEX1, PGI |

The full name and the compartment where the metabolite is located (“c” for cytosol) are viewed as strings:

```
[20]:
```

```
print(g6p_c.name)
print(g6p_c.compartment)
```

```
D-Glucose 6-phosphate
c
```

The chemical formula and associated charge of the metabolite also can be viewed:

```
[21]:
```

```
print(g6p_c.formula)
print(g6p_c.charge)
```

```
C6H11O9P
-2
```

Reactions in which the metabolite participates are obtained as a `frozenset` from the `reactions` attribute. This can be used to count the number of reactions that utilize the metabolite.

```
[22]:
```

```
print("Number of reactions involving {0}: {1}".format(
    g6p_c.id, len(g6p_c.reactions)))
```

```
Number of reactions involving g6p_c: 3
```

The ordinary differential equation (ODE), which represents the change in metabolite concentration over time, is determined by reactions that consume or produce the metabolite. The `oridinary_differential_equation` attribute is used to view the current ODE for the metabolite. The ODE is returned as a symbolic expression.

```
[23]:
```

```
print(g6p_c.ordinary_differential_equation)
```

```
-kf_G6PDH2r*(g6p_c(t)*nadp_c(t) - _6pgl_c(t)*nadph_c(t)/Keq_G6PDH2r) + kf_HEX1*(atp_c(t)*glc__D_c(t) - adp_c(t)*g6p_c(t)/Keq_HEX1) - kf_PGI*g6p_c(t)
```

Numerical solutions are obtained by integrating ODEs. To integrate an ODE, a metabolite concentration at time \(t = 0\) must be defined as an initial condition. Initial conditions are accessed and changed using the `initial_condition` attribute.

```
[24]:
```

```
g6p_c.initial_condition = 0.8
print(g6p_c.initial_condition)
```

```
0.8
```

Certain attributes have alias attribute accessors. For example, the `initial_condition` and `ordinary_differential_equation` attributes can be accessed via `ic` and `ode`, respectively.

```
[25]:
```

```
print(g6p_c.ic)
```

```
0.8
```

The `fixed` attribute indicates whether the concentration of the metabolite is allowed to vary over time, with `True` meaning that the metabolite’s initial condition is treated as a constant concentration value. Fixed metabolites have an ODE equal to 0.

```
[26]:
```

```
g6p_c.fixed = True
print(g6p_c.ode)
```

```
0
```

#### Additional Model Objects¶

The following are additional objects that are stored within a `MassModel`. Unlike metabolites and reactions, which are essential for defining the system of ODEs, these objects are not always necessary for dynamic simulation of models.

However, these objects are still important to **MASSpy** and have a variety of uses, which include: aiding in the management of large models, sharing models among users, tracking additional information relevant to the system, and enabling various workflows for genome-scale kinetic models.

##### Genes¶

Because the `mass.MassReaction` inherits from the `cobra.Reaction`, **MASSpy** is also capable of handling genes and gene-protein-reaction (GPR) relationships. Note that **MASSpy** directly utilizes the `cobra.Gene` object for the representation and management of genes.

The `gene_reaction_rule` is a Boolean representation of the gene requirements for this reaction to be active [SQF+11].

GPRs are stored as the `gene_reaction_rule` of reaction objects. Altering a `gene_reaction_rule` will create new gene objects, if necessary, and update all relationships.

```
[27]:
```

```
PGI.gene_reaction_rule = "New_Gene"
PGI.gene_reaction_rule
```

```
[27]:
```

```
'New_Gene'
```

`Gene` objects are returned from a reaction as a `frozenset`:

```
[28]:
```

```
PGI.genes
```

```
[28]:
```

```
frozenset({<Gene New_Gene at 0x7fb49de75550>})
```

Newly created genes are added to the model upon creation. The gene objects are stored in a `cobra.DictList` as a part of the `genes` attribute. To access a gene from the model:

```
[29]:
```

```
new_gene = model.genes.get_by_id("New_Gene")
new_gene
```

```
[29]:
```

|  |  |
| --- | --- |
| **Gene identifier** | New\_Gene |
| **Name** |  |
| **Memory address** | 0x07fb49de75550 |
| **Functional** | True |
| **In 1 reaction(s)** | PGI |

Gene objects are tracked by both its associated reaction objects and the the model. Changing a reaction’s `gene_reaction_rule` may remove the gene’s association from the reaction, but it does not remove the gene from the model.

```
[30]:
```

```
PGI.gene_reaction_rule = ""
print("Reaction Genes: {0!r}".format(PGI.genes))
new_gene
```

```
Reaction Genes: frozenset()
```

```
[30]:
```

|  |  |
| --- | --- |
| **Gene identifier** | New\_Gene |
| **Name** |  |
| **Memory address** | 0x07fb49de75550 |
| **Functional** | True |
| **In 0 reaction(s)** |  |

##### EnzymeModules¶

A `mass.EnzymeModule` is a specialized `MassModel` that represents a reconstruction of an enzyme’s mechanism. Upon merging an `EnzymeModule` into a `MassModel`, the `EnzymeModule` is converted into a `mass.EnzymeModuleDict`, a specialized dictionary object. The `EnzymeModuleDict` is subsequently stored in a `cobra.DictList` and is accessible through the `enzyme_modules` attribute.

```
[31]:
```

```
PFK = model.enzyme_modules.get_by_id("PFK")
PFK
```

```
[31]:
```

|  |  |
| --- | --- |
| **Name** | PFK |
| **Memory address** | 0x07fb49dcfbe60 |
| **Stoichiometric Matrix** | 26x24 |
| **Matrix Rank** | 20 |
| **Subsystem** | Glycolysis |
| **Number of Ligands** | 6 |
| **Number of EnzymeForms** | 20 |
| **Number of EnzymeModuleReactions** | 24 |
| **Enzyme Concentration Total** | 3.3e-05 |
| **Enzyme Net Flux** | 1.12 |

The process of creating an `EnzymeModuleDict` preserves information stored in various attributes specific to the enzyme module and allows them to be accessed quickly after the merging process. Because the `EnzymeModuleDict` inherits from an `OrderedDict`, it has the same methods and behaviors.

```
[32]:
```

```
for key, value in PFK.items():
    if isinstance(value, list):
        print("{0}: {1}".format(key, len(value)))
```

```
enzyme_module_ligands: 6
enzyme_module_forms: 20
enzyme_module_reactions: 24
enzyme_module_ligands_categorized: 5
enzyme_module_forms_categorized: 5
enzyme_module_reactions_categorized: 6
```

`EnzymeModuleDict` objects also can have their contents accessed by using its `dict` keys as attribute accessors:

```
[33]:
```

```
print(PFK.id)
print(PFK.subsystem)
print(PFK.enzyme_concentration_total)
```

```
PFK
Glycolysis
3.3e-05
```

See the section on EnzymeModules for more information on working with `EnzymeModule` and related objects.

##### Groups¶

Groups are objects for holding information regarding pathways, subsystems, or any custom grouping of objects within a `MassModel`. **MASSpy** directly utilizes the `cobra.Group` object, which are implemented based on the SBML Group specifications.

`Group` objects are stored in a `cobra.DictList` as the `groups` attribute of the `MassModel`.

```
[34]:
```

```
print("Number of groups: {0}".format(len(model.groups)))
```

```
Number of groups: 16
```

There are several different ways to work with group objects. One way that **MASSpy** utilizes group objects is to aid with categorizing and grouping various objects associated with `EnzymeModule` objects.

For example, the group `Products` contains all of metabolites that are the products of the reaction catalyzed by the `PFK` enzyme. A `set` containing the associated metabolite objects is returned by the `members` attribute.

```
[35]:
```

```
products = model.groups.get_by_id("Products")
products.members
```

```
[35]:
```

```
[<MassMetabolite h_c at 0x7fb49dd344d0>,
 <MassMetabolite fdp_c at 0x7fb49dd1fad0>,
 <MassMetabolite adp_c at 0x7fb49dd34310>]
```

Groups are also used to categorize reactions. For example, the group `atp_c_binding` contains all of the reactions that represent the binding of ATP to the free active sites of the PFK enzyme.

```
[36]:
```

```
complexed_w_atp = model.groups.get_by_id("atp_c_binding")
for member in complexed_w_atp.members:
    print(member)
```

```
PFK_R41: atp_c + pfk_R4_c <=> pfk_R4_A_c
PFK_R01: atp_c + pfk_R0_c <=> pfk_R0_A_c
PFK_R11: atp_c + pfk_R1_c <=> pfk_R1_A_c
PFK_R31: atp_c + pfk_R3_c <=> pfk_R3_A_c
PFK_R21: atp_c + pfk_R2_c <=> pfk_R2_A_c
```

Because groups are sets, members of groups are returned in no particular order. To maintain a consistent order, the `sorted()` function is used with the `attrgetter()` function from the `operator` module to sort members by a particular object attribute.

```
[37]:
```

```
for member in sorted(complexed_w_atp.members, key=attrgetter("id")):
    print(member)
```

```
PFK_R01: atp_c + pfk_R0_c <=> pfk_R0_A_c
PFK_R11: atp_c + pfk_R1_c <=> pfk_R1_A_c
PFK_R21: atp_c + pfk_R2_c <=> pfk_R2_A_c
PFK_R31: atp_c + pfk_R3_c <=> pfk_R3_A_c
PFK_R41: atp_c + pfk_R4_c <=> pfk_R4_A_c
```

##### Units¶

`Unit` and `UnitDefinition` objects are implemented, as per the SBML Unit and SBML UnitDefinition specifications. The primary purpose of these objects is to inform users of the model’s units, providing context to model values and observed results.

It is important to note that unit consistency is **NOT** checked by the `MassModel`, meaning that it is incumbent upon users to maintain consistency of units and associated numerical values in a model.

`UnitDefinition` objects are stored in a `cobra.DictList`, accessible through the `units` attribute.

```
[38]:
```

```
model.units
```

```
[38]:
```

```
[<UnitDefinition Millimolar "mM" at 0x7fb49ddabfd0>,
 <UnitDefinition hour "hr" at 0x7fb49bfaea10>]
```

`UnitDefinition` objects have identifiers and optional names. Therefore, a specific unit can be accessed using the `get_by_id()` method.

```
[39]:
```

```
concentration_unit = model.units.get_by_id("mM")
print(concentration_unit.id)
print(concentration_unit.name)
```

```
mM
Millimolar
```

A `UnitDefinition` is comprised of base units, which are stored in the `list_of_units` attribute. Each unit must have a defined `kind`, `exponent`, `scale`, and `multiplier`.

```
[40]:
```

```
for unit in concentration_unit.list_of_units:
    print(unit)
```

```
kind: litre; exponent: -1; scale: 0; multiplier: 1
kind: mole; exponent: 1; scale: -3; multiplier: 1
```

**MASSpy** contains some commonly defined `Unit` objects to aid in the creation of `UnitDefinition` objects, which are viewed using the `print_defined_unit_values()` function.

```
[41]:
```

```
mass.core.units.print_defined_unit_values("Units")
```

```
╒════════════════════════════════════════════════════════════════════╕
│ Pre-defined Units                                                  │
╞════════════════════════════════════════════════════════════════════╡
│ Unit        Definition                                             │
│ ----------  ------------------------------------------------------ │
│ mole        kind: mole; exponent: 1; scale: 0; multiplier: 1       │
│ millimole   kind: mole; exponent: 1; scale: -3; multiplier: 1      │
│ litre       kind: litre; exponent: 1; scale: 0; multiplier: 1      │
│ per_litre   kind: litre; exponent: -1; scale: 0; multiplier: 1     │
│ second      kind: second; exponent: 1; scale: 0; multiplier: 1     │
│ per_second  kind: second; exponent: -1; scale: 0; multiplier: 1    │
│ hour        kind: second; exponent: 1; scale: 0; multiplier: 3600  │
│ per_hour    kind: second; exponent: -1; scale: 0; multiplier: 3600 │
│ per_gDW     kind: gram; exponent: -1; scale: 0; multiplier: 1      │
╘════════════════════════════════════════════════════════════════════╛
```

\(^1\) The “textbook” model is created from Chapters 10-14 of [Pal11]

### Constructing Models¶

In this notebook example, a step-by-step approach of building a simple model\(^1\) of trafficking of high-energy phosphate bonds is demonstrated. Illustrated below is a graphical view of the full system along with the reaction rate equations and numerical values:

The example starts by creating a model of the “use”, “distr”, and “form” reactions. Then the model is expanded to include the remaining metabolites, reactions, and any additional information that should be defined in the model.

#### Creating a Model¶

```
[1]:
```

```
import numpy as np
import pandas as pd

from mass import (
    MassConfiguration, MassMetabolite, MassModel, MassReaction)
from mass.util.matrix import left_nullspace, nullspace

mass_config = MassConfiguration()
```

The first step to creating the model is to define the `MassModel` object. A `MassModel` only requires an identifier to be initialized. For best practice, it is recommended to utilize SBML compliant identifiers for all objects.

```
[2]:
```

```
model = MassModel("Phosphate_Trafficking")
```

The model is initially empty.

```
[3]:
```

```
print("Number of metabolites: {0}".format(len(model.metabolites)))
print("Number of initial conditions: {0}".format(len(model.initial_conditions)))
print("Number of reactions: {0}".format(len(model.reactions)))
```

```
Number of metabolites: 0
Number of initial conditions: 0
Number of reactions: 0
```

The next step is to create `MassMetabolite` and `MassReaction` objects to represent the metabolites and reactions that should exist in the model.

##### Defining metabolites¶

To create a `MassMetabolite`, a unique identifier is required. The `formula` and `charge` attributes are set to ensure mass and charge balancing of reactions in which the metabolite is a participant. The `compartment` attribute indicates where the metabolite is located. In this model, all metabolites exist in a single compartment, abbreviated as “c”.

```
[4]:
```

```
atp_c = MassMetabolite(
    "atp_c",
    name="ATP",
    formula="C10H12N5O13P3",
    charge=-4,
    compartment="c")

adp_c = MassMetabolite(
    "adp_c",
    name="ADP",
    formula="C10H12N5O10P2",
    charge=-3,
    compartment="c")

amp_c = MassMetabolite(
    "amp_c",
    name="AMP",
    formula="C10H12N5O7P",
    charge=-2,
    compartment="c")
```

The metabolite concentrations can be defined as the initial conditions for the metabolites using the `initial_condition` attribute. As previously stated, the concentrations are \(\text{[ATP]}=1.6\), \(\text{[ADP]}=0.4\), and \(\text{[AMP]}=0.1\).

```
[5]:
```

```
atp_c.initial_condition = 1.6
adp_c.ic = 0.4  # Alias for initial_condition
amp_c.ic = 0.1

for metabolite in [atp_c, adp_c, amp_c]:
    print("{0}: {1}".format(metabolite.id, metabolite.initial_condition))
```

```
atp_c: 1.6
adp_c: 0.4
amp_c: 0.1
```

The metabolites are currently not a part of any reaction. Consequently, the `ordinary_differential_equation` attribute is `None`.

```
[6]:
```

```
print(atp_c.ordinary_differential_equation)
print(adp_c.ode)  # Alias for ordinary_differential_equation
print(amp_c.ode)
```

```
None
None
None
```

The next step is to create the reactions in which the metabolites participate.

##### Defining reactions¶

Just like `MassMetabolite` objects, a unique identifier is also required to create a `MassReaction`. The `reversible` attribute determines whether the reaction can proceed in both the forward and reverse directions, or only in the forward direction.

```
[7]:
```

```
distr = MassReaction("distr", name="Distribution", reversible=True)
use = MassReaction("use", name="ATP Utilization", reversible=False)
form = MassReaction("form", name="ATP Formation", reversible=False)
```

Metabolites are added to reactions using a dictionary of metabolite objects and their stoichiometric coefficients. A group of metabolites can be added either all at once or one at a time. A negative coefficient indicates the metabolite is a reactant, while a positive coefficient indicates the metabolite is a product.

```
[8]:
```

```
distr.add_metabolites({
    adp_c: -2,
    atp_c: 1,
    amp_c: 1})

use.add_metabolites({
    atp_c: -1,
    adp_c: 1})

form.add_metabolites({
    adp_c: -1,
    atp_c: 1})

for reaction in [distr, use, form]:
    print(reaction)
```

```
distr: 2 adp_c <=> amp_c + atp_c
use: atp_c --> adp_c
form: adp_c --> atp_c
```

Once the reactions are created, their parameters can be defined. As stated earlier, the distribution reaction is considerably faster when compared to other reactions in the model. The forward rate constant \(k^{\rightarrow}\), represented as `kf`, can be set as \(k^{\rightarrow}\_{distr}=1000\ \text{min}^{-1}\). The equilibrium constant \(K\_{eq}\), represented as `Keq`, is approximately \(K\_{distr}=1\).

```
[9]:
```

```
distr.forward_rate_constant = 1000
distr.equilibrium_constant = 1
distr.parameters  # Return defined mass action kinetic parameters
```

```
[9]:
```

```
{'kf_distr': 1000, 'Keq_distr': 1}
```

As shown earlier, the forward rate constants are set as \(k^{\rightarrow}\_{use}=6.25\ \text{min}^{-1}\) and \(k^{\rightarrow}\_{form}=25\ \text{min}^{-1}\). The `kf_str` attribute can be used to get the identifier of the forward rate constant as a string.

```
[10]:
```

```
use.forward_rate_constant = 6.25
form.kf = 25  # Alias for forward_rate_constant

print("{0}: {1}".format(use.kf_str, use.kf))
print("{0}: {1}".format(form.kf_str, form.kf))
```

```
kf_use: 6.25
kf_form: 25
```

Reactions can be added to the model using the `add_reactions()` method. Adding the reactions to the model also adds the associated metabolites and genes.

```
[11]:
```

```
model.add_reactions([distr, use, form])

print("Number of metabolites: {0}".format(len(model.metabolites)))
print("Number of initial conditions: {0}".format(len(model.initial_conditions)))
print("Number of reactions: {0}".format(len(model.reactions)))
```

```
Number of metabolites: 3
Number of initial conditions: 3
Number of reactions: 3
```

The stoichiometric matrix of the model is automatically constructed with the addition of the reactions and metabolites to the model. It can be accessed through the `stoichiometric_matrix` property (alias `S`).

```
[12]:
```

```
print(model.S)
```

```
[[-2.  1. -1.]
 [ 1. -1.  1.]
 [ 1.  0.  0.]]
```

The stoichiometric matrix attribute can be updated and stored in various formats using the `update_S()` method. For example, the stoichiometric matrix can be converted and stored as a `pandas.DataFrame`.

```
[13]:
```

```
model.update_S(array_type="DataFrame", dtype=np.int_, update_model=True)
model.S
```

```
[13]:
```

|  | distr | use | form |
| --- | --- | --- | --- |
| adp\_c | -2 | 1 | -1 |
| atp\_c | 1 | -1 | 1 |
| amp\_c | 1 | 0 | 0 |

Associating the metabolites with reactions allows for the mass action reaction rate expressions to be generated based on the stoichiometry.

```
[14]:
```

```
print(distr.rate)
```

```
kf_distr*(adp_c(t)**2 - amp_c(t)*atp_c(t)/Keq_distr)
```

Generation of the reaction rates also allows for the metabolite ODEs to be generated.

```
[15]:
```

```
print(atp_c.ode)
```

```
kf_distr*(adp_c(t)**2 - amp_c(t)*atp_c(t)/Keq_distr) + kf_form*adp_c(t) - kf_use*atp_c(t)
```

The `nullspace()` method can be used to obtain the null space of the stoichiometric matrix. The nullspace reflects the pathways through the system.

```
[16]:
```

```
ns = nullspace(model.S)  # Get the null space
# Divide by the minimum and round to nearest integer
ns = np.rint(ns / np.min(ns[np.nonzero(ns)]))
pd.DataFrame(
    ns, index=model.reactions.list_attr("id"),  # Rows represent reactions
    columns=["Pathway 1"], dtype=np.int_)
```

```
[16]:
```

|  | Pathway 1 |
| --- | --- |
| distr | 0 |
| use | 1 |
| form | 1 |

In a similar fashion the left nullspace can be obtained using the `left_nullspace` function. The left nullspace represents the conserved moieties in the model.

```
[17]:
```

```
lns = left_nullspace(model.S)
# Divide by the minimum and round to nearest integer
lns = np.rint(lns / np.min(lns[np.nonzero(lns)]))
pd.DataFrame(
    lns, index=["Total AxP"],
    columns=model.metabolites.list_attr("id"),  # Columns represent metabolites
    dtype=np.int_)
```

```
[17]:
```

|  | adp\_c | atp\_c | amp\_c |
| --- | --- | --- | --- |
| Total AxP | 1 | 1 | 1 |

#### Expanding an Existing Model¶

Now, the existing model is expanded to include a buffer reaction, where a phosphagen is utilized to store a high-energy phosphate in order to buffer the ATP/ADP ratio as needed. Because the buffer molecule represents a generic phosphagen, there is no chemical formula for the molecule. Therefore, the buffer molecule can be represented as a moiety in the `formula` attribute using square brackets.

```
[18]:
```

```
b = MassMetabolite(
    "B",
    name="Phosphagen buffer (Free)",
    formula="[B]",
    charge=0,
    compartment="c")

bp = MassMetabolite(
    "BP",
    name="Phosphagen buffer (Loaded)",
    formula="[B]-PO3",
    charge=-1,
    compartment="c")

buffer = MassReaction("buffer", name="ATP Buffering")
```

When adding metabolites to the reaction, the `get_by_id()` method is used to add already existing metabolites in the model to the reaction.

```
[19]:
```

```
buffer.add_metabolites({
    b: -1,
    model.metabolites.get_by_id("atp_c"): -1,
    model.metabolites.get_by_id("adp_c"): 1,
    bp: 1})

# Add reaction to model
model.add_reactions([buffer])
```

For this reaction, \(k^{\rightarrow}\_{buffer}=1000\ \text{min}^{-1}\) and \(K\_{buffer}=1\). Because the reaction has already been added to the model, the `MassModel.update_parameters()` method can be used to update the reaction parameters using a dictionary:

```
[20]:
```

```
model.update_parameters({
    buffer.kf_str: 1000,
    buffer.Keq_str: 1})

buffer.parameters
```

```
[20]:
```

```
{'kf_buffer': 1000, 'Keq_buffer': 1}
```

By adding the reaction to the model, the left nullspace expanded to include a conservation pool for the total buffer in the system.

```
[21]:
```

```
lns = left_nullspace(model.S)
for i, row in enumerate(lns):
    # Divide by the minimum and round to nearest integer
    lns[i] = np.rint(row / np.min(row[np.nonzero(row)]))
pd.DataFrame(lns, index=["Total AxP", "Total Buffer"],
             columns=model.metabolites.list_attr("id"),
             dtype=np.int_)
```

```
[21]:
```

|  | adp\_c | atp\_c | amp\_c | B | BP |
| --- | --- | --- | --- | --- | --- |
| Total AxP | 1 | 1 | 1 | 0 | 0 |
| Total Buffer | 0 | 0 | 0 | 1 | 1 |

##### Performing symbolic calculations¶

Although the concentrations for the free and loaded buffer molecules are currently unknown, the total amount of buffer is known and set as \(B\_{total} = 10\). Because the buffer reaction is assumed to be at equilibrium, it becomes possible to solve for the concentrations of the free and loaded buffer molecules.

Below, the symbolic capabilities of **SymPy** are used to solve for the steady state concentrations of the buffer molecules.

```
[22]:
```

```
from sympy import Eq, Symbol, pprint, solve

from mass.util import strip_time
```

The first step is to define the equation for the total buffer pool symbolically:

```
[23]:
```

```
buffer_total_equation = Eq(Symbol("B") + Symbol("BP"), 10)
pprint(buffer_total_equation)
```

```
B + BP = 10
```

The equation for the reaction rate at equilibrium is also defined. The `strip_time()` function is used to strip time dependency from the equation.

```
[24]:
```

```
buffer_rate_equation = Eq(0, strip_time(buffer.rate))
# Substitute defined concentration values into equation
buffer_rate_equation = buffer_rate_equation.subs({
    "atp_c":  atp_c.initial_condition,
    "adp_c":  adp_c.initial_condition,
    "kf_buffer": buffer.kf,
    "Keq_buffer": buffer.Keq})
pprint(buffer_rate_equation)
```

```
0 = 1600.0⋅B - 400.0⋅BP
```

These two equations can be solved to get the buffer concentrations:

```
[25]:
```

```
buffer_sol = solve([buffer_total_equation, buffer_rate_equation],
                   [Symbol("B"), Symbol("BP")])
buffer_sol
```

```
[25]:
```

```
{B: 2.00000000000000, BP: 8.00000000000000}
```

Because the metabolites already exist in the model, their initial conditions can be updated to the calculated concentrations using the `MassModel.update_initial_conditions()` method.

```
[26]:
```

```
# Replace the symbols in the dict
for met_symbol, concentration in buffer_sol.items():
    metabolite = model.metabolites.get_by_id(str(met_symbol))
    # Make value as a float
    buffer_sol[metabolite] = float(buffer_sol.pop(met_symbol))

model.update_initial_conditions(buffer_sol)
model.initial_conditions
```

```
[26]:
```

```
{<MassMetabolite adp_c at 0x7fcf242f3190>: 0.4,
 <MassMetabolite atp_c at 0x7fcf242f31d0>: 1.6,
 <MassMetabolite amp_c at 0x7fcf242f3210>: 0.1,
 <MassMetabolite B at 0x7fcf244615d0>: 2.0,
 <MassMetabolite BP at 0x7fcf244612d0>: 8.0}
```

##### Adding boundary reactions¶

After adding the buffer reactions, the next step is to define the AMP source and demand reactions. The `add_boundary()` method is employed to create and add a boundary reaction to a model.

```
[27]:
```

```
amp_drain = model.add_boundary(
    model.metabolites.amp_c,
    boundary_type="demand",
    reaction_id="amp_drain")

amp_in = model.add_boundary(
    model.metabolites.amp_c,
    boundary_type="demand",
    reaction_id="amp_in")

print(amp_drain)
print(amp_in)
```

```
amp_drain: amp_c -->
amp_in: amp_c -->
```

When a boundary reaction is created, a ‘boundary metabolite’ is also created as a proxy metabolite. The proxy metabolite is the external metabolite concentration (i.e., boundary condition) without instantiating a new `MassMetabolite` object to represent the external metabolite.

```
[28]:
```

```
amp_in.boundary_metabolite
```

```
[28]:
```

```
'amp_b'
```

The value of the ‘boundary metabolite’ can be set using the `MassModel.add_boundary_conditions()` method. Boundary conditions are accessed through the `MassModel.boundary_conditions` attribute.

```
[29]:
```

```
model.add_boundary_conditions({amp_in.boundary_metabolite: 1})
model.boundary_conditions
```

```
[29]:
```

```
{'amp_b': 1.0}
```

The automatic generation of the boundary reaction can be useful. However, sometimes the reaction stoichiometry needs to be switched in order to be intuitive. In this case, the stoichiometry of the AMP source reaction should be reversed to show that AMP enters the system, which is accomplished by using the `MassReaction.reverse_stoichiometry()` method.

```
[30]:
```

```
amp_in.reverse_stoichiometry(inplace=True)
print(amp_in)
```

```
amp_in:  --> amp_c
```

Note that the addition of these two reactions adds an another pathway to the null space:

```
[31]:
```

```
ns = nullspace(model.S).T
for i, row in enumerate(ns):
    # Divide by the minimum to get all integers
    ns[i] = np.rint(row / np.min(row[np.nonzero(row)]))
ns = ns.T
pd.DataFrame(
    ns, index=model.reactions.list_attr("id"),  # Rows represent reactions
    columns=["Path 1", "Path 2"], dtype=np.int_)
```

```
[31]:
```

|  | Path 1 | Path 2 |
| --- | --- | --- |
| distr | 0 | 0 |
| use | 1 | 0 |
| form | 1 | 0 |
| buffer | 0 | 0 |
| amp\_drain | 0 | 1 |
| amp\_in | 0 | 1 |

##### Defining custom rates¶

In this model, the rate for the AMP source reaction should remain at a fixed input value. However, the current rate expression for the AMP source reaction is dependent on an external AMP metabolite that exists as a boundary condition:

```
[32]:
```

```
print(amp_in.rate)
```

```
amp_b*kf_amp_in
```

Therefore, the rate can be set as a fixed input by using a custom rate expression. Custom rate expressions can be set for reactions in a model using the `MassModel.add_custom_rate()` method as follows: by passing the reaction object, a string representation of the custom rate expression, and a dictionary containing any custom parameter associated with the rate.

```
[33]:
```

```
model.add_custom_rate(amp_in, custom_rate="b1",
                      custom_parameters={"b1": 0.03})
print(model.rates[amp_in])
```

```
b1
```

#### Ensuring Model Completeness¶

##### Inspecting rates and ODEs¶

According to the network schema at the start of the notebook, the network has been fully reconstructed. The reaction rates and metabolite ODEs can be inspected to ensure that the model was built without any issues.

The `MassModel.rates` property is used to return a dictionary containing reactions and symbolic expressions of their rates. The model always prioritizes custom rate expressions over automatically generated mass action rates.

```
[34]:
```

```
for reaction, rate in model.rates.items():
    print("{0}: {1}".format(reaction.id, rate))
```

```
distr: kf_distr*(adp_c(t)**2 - amp_c(t)*atp_c(t)/Keq_distr)
use: kf_use*atp_c(t)
form: kf_form*adp_c(t)
buffer: kf_buffer*(B(t)*atp_c(t) - BP(t)*adp_c(t)/Keq_buffer)
amp_drain: kf_amp_drain*amp_c(t)
amp_in: b1
```

Similarly, the model can access the ODEs for metabolites using the `ordinary_differential_equations` property (alias `odes`) to return a dictionary of metabolites and symbolic expressions of their ODEs.

```
[35]:
```

```
for metabolite, ode in model.odes.items():
    print("{0}: {1}".format(metabolite.id, ode))
```

```
adp_c: kf_buffer*(B(t)*atp_c(t) - BP(t)*adp_c(t)/Keq_buffer) - 2*kf_distr*(adp_c(t)**2 - amp_c(t)*atp_c(t)/Keq_distr) - kf_form*adp_c(t) + kf_use*atp_c(t)
atp_c: -kf_buffer*(B(t)*atp_c(t) - BP(t)*adp_c(t)/Keq_buffer) + kf_distr*(adp_c(t)**2 - amp_c(t)*atp_c(t)/Keq_distr) + kf_form*adp_c(t) - kf_use*atp_c(t)
amp_c: b1 - kf_amp_drain*amp_c(t) + kf_distr*(adp_c(t)**2 - amp_c(t)*atp_c(t)/Keq_distr)
B: -kf_buffer*(B(t)*atp_c(t) - BP(t)*adp_c(t)/Keq_buffer)
BP: kf_buffer*(B(t)*atp_c(t) - BP(t)*adp_c(t)/Keq_buffer)
```

##### Compartments¶

Compartments, defined in metabolites, are recognized by the model and can be viewed in the `compartments` attribute.

```
[36]:
```

```
model.compartments
```

```
[36]:
```

```
{'c': ''}
```

For this model, “c” is an abbreviation for “compartment”. The `compartments` attribute can be updated to reflect this mapping using a `dict`:

```
[37]:
```

```
model.compartments = {"c": "compartment"}
model.compartments
```

```
[37]:
```

```
{'c': 'compartment'}
```

##### Units¶

`Unit` and `UnitDefinition` objects are implemented as per the SBML Unit and SBML UnitDefinition specifications. It can be useful for comparative reasons to create `Unit` and `UnitDefinition` objects for the model (e.g., amount, volume, time) to provide additional context. However, the model does not
maintain unit consistency automatically. It is the responsibility of the users to ensure consistency among units and associated numerical values in a model.

```
[38]:
```

```
from mass import Unit, UnitDefinition
from mass.core.units import print_defined_unit_values
```

SBML defines units using a compositional approach. The `Unit` objects represent references to base units. A `Unit` has four attributes: `kind`, `exponent`, `scale`, and `multiplier`. The `kind` attribute indicates the base unit. Valid base units are viewed using the `print_defined_unit_values()` function.

```
[39]:
```

```
print_defined_unit_values("BaseUnitKinds")
```

```
╒═════════════════════════════╕
│ SBML Base Unit Kinds        │
╞═════════════════════════════╡
│ Base Unit        SBML Value │
│ -------------  ------------ │
│ ampere                    0 │
│ avogadro                  1 │
│ becquerel                 2 │
│ candela                   3 │
│ coulomb                   5 │
│ dimensionless             6 │
│ farad                     7 │
│ gram                      8 │
│ gray                      9 │
│ henry                    10 │
│ hertz                    11 │
│ item                     12 │
│ joule                    13 │
│ katal                    14 │
│ kelvin                   15 │
│ kilogram                 16 │
│ liter                    17 │
│ litre                    18 │
│ lumen                    19 │
│ lux                      20 │
│ meter                    21 │
│ metre                    22 │
│ mole                     23 │
│ newton                   24 │
│ ohm                      25 │
│ pascal                   26 │
│ radian                   27 │
│ second                   28 │
│ siemens                  29 │
│ sievert                  30 │
│ steradian                31 │
│ tesla                    32 │
│ volt                     33 │
│ watt                     34 │
│ weber                    35 │
│ invalid                  36 │
╘═════════════════════════════╛
```

The `exponent`, `scale` and `multiplier` attributes indicate how the base unit should be transformed. For this model, the unit for concentration, “Millimolar”, which is represented as millimole per liter and composed of the following base units:

```
[40]:
```

```
millimole = Unit(kind="mole", exponent=1, scale=-3, multiplier=1)
per_liter = Unit(kind="liter", exponent=-1, scale=1, multiplier=1)
```

Combinations of `Unit` objects are contained inside a `UnitDefintion` object. `UnitDefinition` objects have three attributes: an `id`, an optional `name` to represent the combination, and a `list_of_units` attribute that contain references to the `Unit` objects. The concentration unit “Millimolar” is abbreviated as “mM” and defined as follows:

```
[41]:
```

```
concentration_unit = UnitDefinition(id="mM", name="Millimolar",
                                    list_of_units=[millimole, per_liter])
print("{0}:\n{1!r}\n{2!r}".format(
    concentration_unit.name, *concentration_unit.list_of_units))
```

```
Millimolar:
<Unit at 0x7fcf244c4b90 kind: liter; exponent: -1; scale: 1; multiplier: 1>
<Unit at 0x7fcf244c49d0 kind: mole; exponent: 1; scale: -3; multiplier: 1>
```

`UnitDefinition` objects also have the `UnitDefinition.create_unit()` method to directly create `Unit` objects within the `UnitDefintion`.

```
[42]:
```

```
time_unit = UnitDefinition(id="min", name="Minute")
time_unit.create_unit(kind="second", exponent=1, scale=1, multiplier=60)
print(time_unit)
print(time_unit.list_of_units)
```

```
min
[<Unit at 0x7fcf244c4450 kind: second; exponent: 1; scale: 1; multiplier: 60>]
```

Once created, `UnitDefintion` objects are added to the model:

```
[43]:
```

```
model.add_units([concentration_unit, time_unit])
model.units
```

```
[43]:
```

```
[<UnitDefinition Millimolar "mM" at 0x7fcf244c4cd0>,
 <UnitDefinition Minute "min" at 0x7fcf244c4fd0>]
```

##### Checking model completeness¶

Once constructed, the model should be checked for completeness.

```
[44]:
```

```
from mass import qcqa_model
```

The `qcqa_model()` function can be used to print a report about the model’s completeness based on the set kwargs. The `qcqa_model()` function is used to ensure that all numerical values necessary for simulating the model are defined by setting the `parameters` and `concentrations` kwargs as `True`.

```
[45]:
```

```
qcqa_model(model, parameters=True, concentrations=True)
```

```
╒══════════════════════════════════════════════╕
│ MODEL ID: Phosphate_Trafficking              │
│ SIMULATABLE: False                           │
│ PARAMETERS NUMERICALY CONSISTENT: True       │
╞══════════════════════════════════════════════╡
│ ============================================ │
│             MISSING PARAMETERS               │
│ ============================================ │
│ Reaction Parameters                          │
│ ---------------------                        │
│ amp_drain: kf                                │
│ ============================================ │
╘══════════════════════════════════════════════╛
```

As shown in the report above, the forward rate constant for the AMP drain reaction was never defined. Therefore, the forward rate constant is defined, and the model is checked again.

```
[46]:
```

```
amp_drain.kf = 0.03

qcqa_model(model, parameters=True, concentrations=True)
```

```
╒══════════════════════════════════════════╕
│ MODEL ID: Phosphate_Trafficking          │
│ SIMULATABLE: True                        │
│ PARAMETERS NUMERICALY CONSISTENT: True   │
╞══════════════════════════════════════════╡
╘══════════════════════════════════════════╛
```

Now, the report shows that the model is not missing any values necessary for simulation. See Checking Model Quality for more information on quality assurance functions and the `qcqa` submodule.

#### Additional Examples¶

For additional examples on constructing models, see the following:

- Constructing Glycolysis

\(^1\) Trafficking model is created from Chapter 8 of [Pal11]

### Reading and Writing Models¶

In this notebook example, the import and export capabilities of **MASSpy** are demonstrated.

**MASSpy** supports reading and writing models in the SBML and JSON formats. The preferred format for general use is the SBML with the FBC (Version 2) extension and the Groups (Version 1) extension.

The JSON format may be more useful for **MASSpy** specific functionality and for visualizing networks via Escher. See the Network Visualization section for additional details.

The **MASSpy** package also comes with models in various formats for testing purposes.

```
[1]:
```

```
from os.path import join

import mass
import mass.test

# To view the list of available models, remove the semicolon
mass.test.view_test_models();
```

```
[1]:
```

```
['Glycolysis.json',
 'Glycolysis_FKRM.json',
 'Glycolysis_Hb_HEX1.json',
 'Glycolysis_Hb_PFK.json',
 'Glycolysis_Hb_PYK.json',
 'Hemoglobin.json',
 'Model_to_Repair.json',
 'MultiCompartment.json',
 'Phosphate_Trafficking.json',
 'SB2_AMPSalvageNetwork.json',
 'SB2_Glycolysis.json',
 'SB2_Hemoglobin.json',
 'SB2_PFK.json',
 'SB2_PentosePhosphatePathway.json',
 'Simple_Toy.json',
 'Simple_Toy.xml',
 'WholeCellRBC_MA_Rates.json',
 'WholeCellRBC_MA_Rates.xml',
 'WholeCellRBC_MM_Rates.json',
 'WholeCellRBC_MM_Rates.xml',
 'textbook.json',
 'textbook.xml']
```

#### SBML¶

```
[2]:
```

```
from mass.io import sbml
```

The Systems Biology Markup Language is an XML-based standard format for distributing models.

**MASSpy** supports the reading and writing of SBML Level 3. **MASSpy** attempts to convert SBML Level 1 and Level 2 models to Level 3 before loading.

```
[3]:
```

```
model = sbml.read_sbml_model(join(mass.test.MODELS_DIR, "textbook.xml"))
model
```

```
[3]:
```

|  |  |
| --- | --- |
| **Name** | RBC\_PFK |
| **Memory address** | 0x07feacee420d0 |
| **Stoichiometric Matrix** | 68x76 |
| **Matrix Rank** | 63 |
| **Number of metabolites** | 68 |
| **Initial conditions defined** | 68/68 |
| **Number of reactions** | 76 |
| **Number of genes** | 0 |
| **Number of enzyme modules** | 1 |
| **Number of groups** | 16 |
| **Objective expression** | 0 |
| **Compartments** | Cytosol |

```
[4]:
```

```
sbml.write_sbml_model(model, "test_textbook.xml")
```

**MASSpy** utilizes the libSBML package to read and write SBML files, supporting both the FBC (Version 2) and the Groups (Version 1) extensions. When reading in a model, **MASSpy** automatically detects whether the FBC and/or Groups extensions were used.

To preserve information specific to `EnzymeModule` objects, the SBML Groups extension is used along with the notes section for SBML objects. The `use_groups_package` argument can be utilized to indicate whether to write `cobra.Group` objects to the SBML file, including `EnzymeModule` information. Disabling this extension may result in a loss of some enzyme specific information (e.g., categorized groups), but it does not prevent species and reactions of the enzyme module from being
written.

When writing a model, the `use_fbc_package` argument can be used to indicate whether to write additional model information (e.g., metabolite formula and charge, genes, reaction bounds) via the FBC extension.

#### JSON¶

```
[5]:
```

```
from mass.io import json
```

**MASSpy** models have a JSON representation, allowing for interoperability with the Escher.

See the Network Visualization section for additional details on working with **Escher**.

```
[6]:
```

```
model = json.load_json_model(join(mass.test.MODELS_DIR, "textbook.json"))
model
```

```
[6]:
```

|  |  |
| --- | --- |
| **Name** | RBC\_PFK |
| **Memory address** | 0x07feacfb007d0 |
| **Stoichiometric Matrix** | 68x76 |
| **Matrix Rank** | 63 |
| **Number of metabolites** | 68 |
| **Initial conditions defined** | 68/68 |
| **Number of reactions** | 76 |
| **Number of genes** | 0 |
| **Number of enzyme modules** | 1 |
| **Number of groups** | 16 |
| **Objective expression** | 0 |
| **Compartments** | Cytosol |

```
[7]:
```

```
json.save_json_model(model, "test_textbook.json")
```

Consider having the simplejson package to speed up reading/writing of JSON models.

##### JSON schema¶

The JSON schema for **MASSpy** models is stored in mass.io.json as the `JSON_SCHEMA` variable. It can be accessed via the following:

```
[8]:
```

```
# To view the JSON schema, remove the semicolon
json.JSON_SCHEMA;
```

```
[8]:
```

```
{'$schema': 'http://json-schema.org/draft-07/schema#',
 'title': 'MASS',
 'description': 'JSON representation of MASS model',
 'type': 'object',
 'properties': {'id': {'type': 'string'},
  'name': {'type': 'string'},
  'version': {'type': 'integer', 'default': 1},
  'reactions': {'type': 'array',
   'items': {'type': 'object',
    'properties': {'id': {'type': 'string'},
     'name': {'type': 'string'},
     'reversible': {'type': 'boolean'},
     'metabolites': {'type': 'object',
      'patternProperties': {'.*': {'type': 'number'}}},
     'gene_reaction_rule': {'type': 'string'},
     'lower_bound': {'type': 'number'},
     'upper_bound': {'type': 'number'},
     'subsystem': {'type': 'string'},
     'steady_state_flux': {'type': 'number'},
     'forward_rate_constant': {'type': 'number'},
     'reverse_rate_constant': {'type': 'number'},
     'equilibriun_constant': {'type': 'number'},
     'objective_coefficient': {'type': 'number', 'default': 0},
     'variable_kind': {'type': 'string',
      'pattern': 'integer|continuous',
      'default': 'continuous'},
     '_rate': {'type': 'string'},
     'notes': {'type': 'object'},
     'annotation': {'type': 'object'},
     'enzyme_module_id': {'type': 'string'}}},
   'required': ['id',
    'name',
    'reversible',
    'metabolites',
    'lower_bound',
    'upper_bound',
    'gene_reaction_rule'],
   'additionalProperties': False},
  'metabolites': {'type': 'array',
   'items': {'type': 'object',
    'properties': {'id': {'type': 'string'},
     'name': {'type': 'string'},
     'formula': {'type': 'string'},
     'charge': {'type': 'integer'},
     'compartment': {'type': 'string', 'pattern': '[a-z]{1,2}'},
     'fixed': {'type': 'boolean'},
     '_initial_condition': {'type': 'number',
      'minimum': 0,
      'exclusiveMinimum': False},
     '_constraint_sense': {'type': 'string',
      'default': 'E',
      'pattern': 'E|L|G'},
     '_bound': {'type': 'number', 'default': 0},
     'notes': {'type': 'object'},
     'annotation': {'type': 'object'},
     '_bound_metabolites': {'type': 'object',
      'patternProperties': {'.*': {'type': 'number'}}},
     'enzyme_module_id': {'type': 'string'}},
    'required': ['id', 'name'],
    'additionalProperties': False}},
  'genes': {'type': 'array',
   'items': {'type': 'object',
    'properties': {'id': {'type': 'string'},
     'name': {'type': 'string'},
     'notes': {'type': 'object'},
     'annotation': {'type': 'object'}},
    'required': ['id', 'name'],
    'additionalProperties': False}},
  'enzyme_modules': {'type': 'array',
   'items': {'type': 'object',
    'properties': {'id': {'type': 'string'},
     'name': {'type': 'string'},
     'subsystem': {'type': 'string'},
     'enzyme_module_ligands': {'type': 'array', 'allOf': {'type': 'string'}},
     'enzyme_module_forms': {'type': 'array', 'allOf': {'type': 'string'}},
     'enzyme_module_reactions': {'type': 'array', 'allOf': {'type': 'string'}},
     'enzyme_module_ligands_categorized': {'type': 'object',
      'allOf': {'type': 'array', 'allOf': {'type': 'string'}}},
     'enzyme_module_forms_categorized': {'type': 'object',
      'allOf': {'type': 'array', 'allOf': {'type': 'string'}}},
     'enzyme_module_reactions_categorized': {'type': 'object',
      'allOf': {'type': 'array', 'allOf': {'type': 'string'}}},
     'enzyme_concentration_total': {'type': 'number',
      'minimum': 0,
      'exclusiveMinimum': False},
     'enzyme_rate': {'type': 'number'},
     'enzyme_concentration_total_equation': {'type': 'string'},
     'enzyme_rate_equation': {'type': 'string'}}},
   'required': ['id', 'name'],
   'additionalProperties': False},
  'units': {'type': 'array',
   'items': {'type': 'object',
    'properties': {'kind': {'type': 'string'},
     'exponent': {'type': 'number'},
     'scale': {'type': 'number'},
     'multiplier': {'type': 'number'}}}},
  'boundary_conditions': {'type': 'object',
   'allOf': {'type': 'number', 'minimum': 0}},
  'custom_rates': {'type': 'object',
   'patternProperties': {'.*': {'type': 'string'}}},
  'custom_parameters': {'type': 'object',
   'patternProperties': {'.*': {'type': 'number'}}},
  'compartments': {'type': 'object',
   'patternProperties': {'[a-z]{1,2}': {'type': 'string'}}},
  'notes': {'type': 'object'},
  'annotation': {'type': 'object'},
  'enzyme_module_ligands': {'type': 'array', 'allOf': {'type': 'string'}},
  'enzyme_module_forms': {'type': 'array', 'allOf': {'type': 'string'}},
  'enzyme_module_reactions': {'type': 'array', 'allOf': {'type': 'string'}},
  '_enzyme_module_ligands_categorized': {'type': 'object',
   'allOf': {'type': 'array', 'allOf': {'type': 'string'}}},
  '_enzyme_module_forms_categorized': {'type': 'object',
   'allOf': {'type': 'array', 'allOf': {'type': 'string'}}},
  '_enzyme_module_reactions_categorized': {'type': 'object',
   'allOf': {'type': 'array', 'allOf': {'type': 'string'}}},
  '_enzyme_concentration_total': {'type': 'number',
   'minimum': 0,
   'exclusiveMinimum': False},
  '_enzyme_rate': {'type': 'number'},
  '_enzyme_rate_equation': {'type': 'string'}},
 'required': ['id', 'reactions', 'metabolites', 'genes'],
 'additionalProperties': False}
```

#### Converting between file formats¶

Often there are times where one program or package is used to execute a specific task, yet a downstream task requires the use of a different program or package. Consequently, the ability to import a model written using one format and subsequently export it to another is essential in these scenarios. The submodules of `mass.io` can be used to facilitate the import/export of models in different formats for this purpose.

One possible scenario in which the conversion between file formats is necessary involves the visualizion of an SBML model on a network map using the Escher [KDragerE+15] visualization tool.

See Visualizing SBML models with Escher in Python for an example demonstrating how **MASSpy** facilitates file conversion for model exchangability.

### Dynamic Simulation of Models¶

This notebook example provides a basic demonstration on how to dynamically simulate models.

```
[1]:
```

```
import mass.test

model = mass.test.create_test_model("Phosphate_Trafficking")
```

#### Creating a Simulation¶

The `Simulation` object manages all aspects related to simulating one or more model. This includes interfacing with the `RoadRunner` object from the libRoadRunner package, which is utilized for JIT compilation of the model, integration of model ODEs, and returning simulation output.

```
[2]:
```

```
from mass import Simulation
```

##### Loading a model¶

A `Simulation` object is initialized by providing a `MassModel` to be loaded into the underlying `RoadRunner` object. The provided `MassModel` is treated as the `reference_model` of the `Simulation`.

`RoadRunner` is designed to simulate models in SBML format. Therefore, models must be SBML compliant to be loaded into the `RoadRunner` instance.

```
[3]:
```

```
sim = Simulation(reference_model=model, verbose=True)
```

```
Successfully loaded MassModel 'Phosphate_Trafficking' into RoadRunner.
```

The reference `MassModel` is accessed using the `reference_model` attribute.

```
[4]:
```

```
sim.reference_model
```

```
[4]:
```

|  |  |
| --- | --- |
| **Name** | Phosphate\_Trafficking |
| **Memory address** | 0x07fe71b3f3850 |
| **Stoichiometric Matrix** | 5x6 |
| **Matrix Rank** | 4 |
| **Number of metabolites** | 5 |
| **Initial conditions defined** | 5/5 |
| **Number of reactions** | 6 |
| **Number of genes** | 0 |
| **Number of enzyme modules** | 0 |
| **Number of groups** | 0 |
| **Objective expression** | 0 |
| **Compartments** | compartment |

The underlying `RoadRunner` instance can be accessed using the `roadrunner` attribute:

```
[5]:
```

```
sim.roadrunner
```

```
[5]:
```

```
<roadrunner.RoadRunner() { this = 0x7fe71b427dd0 }>
```

Upon loading a model into a `Simulation`, the numerical values for species’ initial conditions and reaction parameters are extracted. Dictionaries containing values are retrieved using the `get_model_simulation_values()` method.

```
[6]:
```

```
initial_conditions, parameters = sim.get_model_simulation_values(model)
for metabolite, initial_condition in initial_conditions.items():
    print("{0}: {1}".format(metabolite, initial_condition))
```

```
adp_c: 0.2
atp_c: 1.7
amp_c: 0.2
B: 2
BP: 8
```

#### Running Dynamic Simulations¶

##### Simulating a model¶

Once a model has been loaded, it can be simulated using the `Simulation.simulate()` method. The `simulate()` method requires a model identifier or `MassModel` object of a loaded model and a tuple that contains the initial and final time points.

```
[7]:
```

```
# Simulate the model from 0 to 100 time units
solutions = sim.simulate(model, time=(0, 100))
solutions
```

```
[7]:
```

```
(<MassSolution Phosphate_Trafficking_ConcSols at 0x7fe71b5960b0>,
 <MassSolution Phosphate_Trafficking_FluxSols at 0x7fe71b5966b0>)
```

After a model has been simulated, the concentration and flux solutions are returned in two specialized dictionaries known as `MassSolution` objects.

```
[8]:
```

```
conc_sol, flux_sol = solutions

# List the first 5 points of concentration solutions
for metabolite, solution in conc_sol.items():
    print("{0}: {1}".format(metabolite, solution[:5]))
```

```
adp_c: [0.2        0.20020374 0.20040726 0.20095514 0.2015016 ]
atp_c: [1.7        1.69982168 1.69964357 1.69916416 1.69868608]
amp_c: [0.2        0.19997458 0.19994916 0.19988069 0.1998123 ]
B: [2.         1.99984758 1.99969535 1.99928569 1.99887727]
BP: [8.         8.00015242 8.00030465 8.00071431 8.00112273]
```

By default, solutions are returned as `numpy.ndarrays`. To return interpolating functions instead, the `interpolate` argument is set as `True`.

```
[9]:
```

```
conc_sol, flux_sol = sim.simulate(model, time=(0, 100), interpolate=True)

for metabolite, solution in conc_sol.items():
    print("{0}: {1}".format(metabolite, solution))
```

```
adp_c: <scipy.interpolate.interpolate.interp1d object at 0x7fe71b596e90>
atp_c: <scipy.interpolate.interpolate.interp1d object at 0x7fe71b596fb0>
amp_c: <scipy.interpolate.interpolate.interp1d object at 0x7fe71b5a2050>
B: <scipy.interpolate.interpolate.interp1d object at 0x7fe71b5a2110>
BP: <scipy.interpolate.interpolate.interp1d object at 0x7fe71b5a2170>
```

##### Setting integration options¶

Although the integrator options are set to accomadate a variety of models, there are circumestances in which the integrator options need to be changed. The underlying integrator can be accessed using the `integrator` property:

```
[10]:
```

```
print(sim.integrator)
```

```
< roadrunner.Integrator() >
  name: cvode
  settings:
      relative_tolerance: 0.000001
      absolute_tolerance: 0.000000000001
                   stiff: true
       maximum_bdf_order: 5
     maximum_adams_order: 12
       maximum_num_steps: 20000
       maximum_time_step: 0
       minimum_time_step: 0
       initial_time_step: 0
          multiple_steps: false
      variable_step_size: true
```

Each setting comes with a brief description that can be viewed:

```
[11]:
```

```
print(sim.integrator.getDescription("variable_step_size"))
```

```
(bool) Enabling this setting will allow the integrator to adapt the size of each time step. This will result in a non-uniform time column.
```

For example, to change the integration options so a uniform time vector is returned instead of one with a variable step size:

```
[12]:
```

```
sim.integrator.variable_step_size = False

# Simulate the model from 0 to 100 time units
# with output returned at evenly spaced time points
conc_sol, flux_sol = sim.simulate(model, time=(0, 100))

# Print the time vector
print(conc_sol.time[:10])
```

```
[ 0.  2.  4.  6.  8. 10. 12. 14. 16. 18.]
```

When encountering exceptions from the integrator, libRoadRunner recommends specifying an initial time step and tighter absolute and relative tolerances.

See the libRoadRunner documentation about the `roadrunner.Integrator` class for more information on the integrator.

##### Simulation results and the MassSolution object¶

For every model simulated, two `MassSolution` objects are returned per model. `MassSolution` objects are always outputted as pairs, with one `MassSolution` object containing the solutions for metabolite concentrations, and the other containing the solutions for reaction fluxes.

```
[13]:
```

```
# Simulate the model from 0 to 100 time units
sim = Simulation(model)
conc_sol, flux_sol = sim.simulate(model, time=(0, 100))
```

A `MassSolution` for a successful simulation contains string identifiers of objects and their corresponding solutions. Because `MassSolution` objects are specialized dictionaries, solutions can be retrieved using the object identifier as `dict` keys. For example, to access the solution for “atp\_c”:

```
[14]:
```

```
# Print first 10 solution values for ATP
conc_sol["atp_c"][:10]
```

```
[14]:
```

```
array([1.7       , 1.69982168, 1.69964357, 1.69916416, 1.69868608,
       1.69820944, 1.69773427, 1.69673394, 1.69460788, 1.6925119 ])
```

If care is taken when assigning object identifiers (e.g., does not start with a number, does not contain certain characters such as “-”), it is possible to access solutions inside of a `MassSolution` as if the corresponding keys were attributes.

```
[15]:
```

```
# Print first 10 solution values for ATP
conc_sol.atp_c[:10]
```

```
[15]:
```

```
array([1.7       , 1.69982168, 1.69964357, 1.69916416, 1.69868608,
       1.69820944, 1.69773427, 1.69673394, 1.69460788, 1.6925119 ])
```

The time points returned by the integrator are accessible using the `MassSolution.time` attribute:

```
[16]:
```

```
# Print the first 10 time points
print(conc_sol.time[0:10])
```

```
[0.00000000e+00 8.47847116e-08 1.69569423e-07 3.98257356e-07
 6.26945288e-07 8.55633221e-07 1.08432115e-06 1.56811392e-06
 2.60709564e-06 3.64607737e-06]
```

The solutions contained within the `MassSolution` can be obtained as a `pandas.DataFrame` using the `to_frame()` method.

```
[17]:
```

```
conc_sol.to_frame()
```

```
[17]:
```

|  | adp\_c | atp\_c | amp\_c | B | BP |
| --- | --- | --- | --- | --- | --- |
| Time |  |  |  |  |  |
| 0.000000e+00 | 0.200000 | 1.700000 | 0.200000 | 2.000000 | 8.000000 |
| 8.478471e-08 | 0.200204 | 1.699822 | 0.199975 | 1.999848 | 8.000152 |
| 1.695694e-07 | 0.200407 | 1.699644 | 0.199949 | 1.999695 | 8.000305 |
| 3.982574e-07 | 0.200955 | 1.699164 | 0.199881 | 1.999286 | 8.000714 |
| 6.269453e-07 | 0.201502 | 1.698686 | 0.199812 | 1.998877 | 8.001123 |
| ... | ... | ... | ... | ... | ... |
| 3.012599e+01 | 0.399998 | 1.599991 | 0.099999 | 2.000000 | 8.000000 |
| 3.828512e+01 | 0.399998 | 1.599992 | 0.100000 | 2.000000 | 8.000000 |
| 5.517474e+01 | 0.399998 | 1.599994 | 0.100000 | 2.000000 | 8.000000 |
| 8.169531e+01 | 0.399999 | 1.599996 | 0.100000 | 2.000000 | 8.000000 |
| 1.000000e+02 | 0.399999 | 1.599997 | 0.100000 | 2.000000 | 8.000000 |

171 rows × 5 columns

Solutions also can be viewed visually using the `view_time_profile()` method. Note that this requires `matplotlib` to be installed in the environment. See Plotting and Visualization for more information.

```
[18]:
```

```
conc_sol.view_time_profile()
```

##### Aggregate variables and solutions¶

Often, it is desirable to look at mathematical combinations of metabolites concentrations or reaction fluxes. To create an aggregate variable, the `MassSolution.make_aggregate_solution()` method is used. To use the method, three inputs are required:

1. A unique ID for the aggregate variable.
2. The mathematical equation for the aggregate variable given as a `str`.
3. A list of the `MassSolution` keys representing variables used in the equation.

For example, to make the Adenylate Energy Charge [Atk68], the occupancy and capacity pools are first defined:

```
[19]:
```

```
occupancy = conc_sol.make_aggregate_solution(
    aggregate_id="occupancy",
    equation="(atp_c + 0.5 * adp_c)",
    variables=["atp_c", "adp_c"])
conc_sol.update(occupancy)
print(list(conc_sol.keys()))
```

```
['adp_c', 'atp_c', 'amp_c', 'B', 'BP', 'occupancy']
```

The aggregate variables are returned as a `dict`, which can be added to the `MassSolution` object. Alternatively, the `update` flag can be set as `True` to automatically add an aggregate variable to the solution after creation.

```
[20]:
```

```
capacity = conc_sol.make_aggregate_solution(
    aggregate_id="capacity",
    equation="(atp_c + adp_c + amp_c)",
    variables=["atp_c", "adp_c", "amp_c"],
    update=True)
print(list(conc_sol.keys()))
```

```
['adp_c', 'atp_c', 'amp_c', 'B', 'BP', 'occupancy', 'capacity']
```

Aggregate variables formed from other aggregate variables also can be created using the `make_aggregate_solution()` method as long as the aggregate variables have been added to the `MassSolution`. To make the energy charge from the occupancy and capacity aggregate variables:

```
[21]:
```

```
ec = conc_sol.make_aggregate_solution(
    aggregate_id="energy_charge",
    equation="occupancy / capacity",
    variables=["occupancy", "capacity"],
    update=True)
```

If care is taken when assigning aggregate variable identifiers, it is possible to access aggregate variable solutions inside of a `MassSolution`, as if aggregate variable keys were attributes.

```
[22]:
```

```
# Print first 10 solution points for the energy charge
conc_sol.energy_charge[:10]
```

```
[22]:
```

```
array([0.85714286, 0.85710645, 0.8570701 , 0.85697226, 0.85687471,
       0.85677749, 0.85668059, 0.85647669, 0.85604374, 0.85561747])
```

#### Perturbing a Model¶

To simulate various disturbances in the system, the `perturbations` argument of the `simulate()` method can be used. There are several types of perturbations that can be implemented for a given simulation as long as they adhere to the following guidelines:

1. Perturbations are provided to the method as a `dict` with dictionary keys that correspond to variables to be changed. Dictionary values are the new numerical values or mathematical expressions as strings that indicate how the value is to be changed.
2. A formula for the perturbation can be provided as a `str` as long as the formula string can be sympified via the `sympy.sympify()` function. The formula can have one variable that is identical to the corresponding `dict` key.
3. Boundary conditions can be set as a function of time. The above rules still apply, but allow for the time “t”, as a second variable.

Some examples are demonstrated below.

A simulation without perturbations:

```
[23]:
```

```
conc_sol, flux_sol = sim.simulate(model, time=(0, 1000))
conc_sol.view_time_profile()
```

Perturbing the initial concentration of ATP from 1.6 to 2.5:

```
[24]:
```

```
conc_sol, flux_sol = sim.simulate(
    model, time=(0, 1000), perturbations={"atp_c": 2.5})
conc_sol.view_time_profile()
```

Increasing the rate constant of ATP use by 50%:

```
[25]:
```

```
conc_sol, flux_sol = sim.simulate(
    model, time=(0, 1000), perturbations={"kf_use": "kf_use * 1.5"})
conc_sol.view_time_profile()
```

#### Determining Steady State¶

The steady state for models can be found using the `Simulation.find_steady_state()` method. This method requires a model identifier or a `MassModel` object and a string thats indicates a strategy for finding the steady state. For example, to find the steady state by simulating the model for a long time:

```
[26]:
```

```
sim = Simulation(reference_model=model)

conc_sol, flux_sol = sim.find_steady_state(model, strategy="simulate")
for metabolite, solution in conc_sol.items():
    print("{0}: {1}".format(metabolite, solution))
```

```
adp_c: 0.40000000000001784
atp_c: 1.6000000000000718
amp_c: 0.10000000000000445
B: 1.9999999999999998
BP: 8.0
```

Alternatively, a steady state solver can be utilized with a root-finding algorithm to determine the steady state. For example, to use a non-linear equation solver that implements a global Newton method with adaptive damping strategies (NLEQ2):

```
[27]:
```

```
conc_sol, flux_sol = sim.find_steady_state(model, strategy="nleq2")
for metabolite, solution in conc_sol.items():
    print("{0}: {1}".format(metabolite, solution))
```

```
adp_c: 0.39999999999999997
atp_c: 1.6
amp_c: 0.1
B: 2.0000000000000004
BP: 8.000000000000004
```

Setting `update_values=True` updates the model initial conditions and fluxes with the steady state solution:

```
[28]:
```

```
conc_sol, flux_sol = sim.find_steady_state(model, strategy="nleq2",
                                           update_values=True)
model.initial_conditions  # Same object as reference model in Simulation
```

```
[28]:
```

```
{<MassMetabolite adp_c at 0x7fe71b3f3950>: 0.39999999999999997,
 <MassMetabolite atp_c at 0x7fe71b3f38d0>: 1.6,
 <MassMetabolite amp_c at 0x7fe71b3f3c50>: 0.1,
 <MassMetabolite B at 0x7fe71b3f3c10>: 2.0000000000000004,
 <MassMetabolite BP at 0x7fe71b3f3c90>: 8.000000000000004}
```

The `find_steady_state()` method also allows for perturbations to be made before determining a steady state solution:

```
[29]:
```

```
conc_sol, flux_sol = sim.find_steady_state(
    model, strategy="simulate", perturbations={"kf_use": "kf_use * 1.5"})

for metabolite, solution in conc_sol.items():
    print("{0}: {1}".format(metabolite, solution))
```

```
adp_c: 0.2666666666666707
atp_c: 0.711111111111122
amp_c: 0.10000000000000149
B: 2.72727272727273
BP: 7.272727272727282
```

##### The steady state solver¶

Although the steady state solver options are set to accommodate a variety of models, there are circumstances in which the steady state solver options need to be changed. The underlying solver can be accessed using the `steady_state_solver` property:

```
[30]:
```

```
print(sim.steady_state_solver)
```

```
< roadrunner.SteadyStateSolver() >
  name: nleq2
  settings:
     allow_presimulation: false
    presimulation_maximum_steps: 100
      presimulation_time: 100
            allow_approx: true
        approx_tolerance: 0.000001
    approx_maximum_steps: 10000
             approx_time: 10000
      relative_tolerance: 0.000000000001
      maximum_iterations: 100
         minimum_damping: 1e-20
          broyden_method: 0
               linearity: 3
```

Analogous to the integrator, each setting for the steady state solver comes with a brief description that can be viewed:

```
[31]:
```

```
print(sim.steady_state_solver.getHint("maximum_iterations"))
```

```
The maximum number of iterations the solver is allowed to use (int)
```

For example, to change the solver options to allow for a larger maximum number of iterations:

```
[32]:
```

```
sim.steady_state_solver.maximum_iterations = 500
```

For more information on steady state solver options, see the libRoadRunner documentation about the `roadrunner.SteadyStateSolver` class.

#### Simulating Multiple Models¶

Multiple models can be added to a `Simulation` object in order to perform simulations on several models. Below, a simple example utilizing a copy of the model with a smaller total buffer concentration is made for demonstration purposes:

```
[33]:
```

```
model = mass.test.create_test_model("Phosphate_Trafficking")
sim = Simulation(model)

# Make a modified model
modified = model.copy()
modified.id = "Phosphate_Trafficking_Modified"
modified.update_initial_conditions({"BP": 4, "B": 1})
```

To add an additional model to an existing `Simulation` object, three criteria must be met:

1. The model must have equivalent ODEs to the `reference_model` used in creating the `Simulation`.
2. All models in the `Simulation` must have unique identifiers.
3. Numerical values necessary for simulation must be already defined for a model.

Use the `MassModel.has_equivalent_odes()` method to check if ODEs are equivalent.

```
[34]:
```

```
sim.reference_model.has_equivalent_odes(modified, verbose=True)
```

```
[34]:
```

```
True
```

Use the `Simulation.add_models()` method to load the additional model.

```
[35]:
```

```
sim.add_models(models=[modified], verbose=True)
```

```
Successfully loaded MassModel 'Phosphate_Trafficking_Modified'.
```

Use the `simulate()` method to simulate multiple models by providing a list of model objects or their identifiers.

```
[36]:
```

```
conc_sol_list, flux_sol_list = sim.simulate(
    models=[model, modified], time=(0, 100))
```

After simulating multiple models, the `MassSolution` objects are returned in two `cobra.DictList` objects. The first `DictList` contains the concentrations solutions, and the second `DictList` contains the flux solutions for simulated models.

```
[37]:
```

```
conc_sol_list
```

```
[37]:
```

```
[<MassSolution Phosphate_Trafficking_ConcSols at 0x7fe71d1201d0>,
 <MassSolution Phosphate_Trafficking_Modified_ConcSols at 0x7fe71d120530>]
```

The `get_by_id()` method can be used to access a specific solution:

```
[38]:
```

```
conc_sol_list.get_by_id("_".join((modified.id, "ConcSols")))
```

```
[38]:
```

```
<MassSolution Phosphate_Trafficking_Modified_ConcSols at 0x7fe71d120530>
```

For additional information on simulating multiple models, see Simulating an Ensemble of Models.

### Plotting and Visualization¶

This notebook example demonstrates how to create various plots using the visualization functions in **MASSpy**.

All visualization methods in **MASSpy** utilize **matplotlib** for creating and manipulating plots. Plots generated by **MASSpy** can be subjected to various **matplotlib** methods before and after generating the plot.

```
[1]:
```

```
import matplotlib as mpl
import matplotlib.pyplot as plt

import numpy as np

import pandas as pd

import mass.test
from mass import MassConfiguration, Simulation

mass_config = MassConfiguration()
mass_config.decimal_precision = 12  # Round after 12 digits after decimal
model = mass.test.create_test_model("Glycolysis")
```

#### Quickly Viewing Simulation Results¶

A simulation is performed with a perturbation in order to generate output to plot.

```
[2]:
```

```
simulation = Simulation(model, verbose=True)
simulation.find_steady_state(model, strategy="simulate",
                             decimal_precision=True)
conc_sol, flux_sol = simulation.simulate(
    model, time=(0, 1000),
    perturbations={"kf_ATPM": "kf_ATPM * 1.5"},
    decimal_precision=True)
```

```
Successfully loaded MassModel 'Glycolysis' into RoadRunner.
```

Simulation results can be quickly rendered into a time profile using the `MassSolution.view_time_profile()` method.

```
[3]:
```

```
conc_sol.view_time_profile()
```

However, this method does not provide any flexibility or control over the plot that is generated. For more control over the plotting process, the various methods of the `visualization` submodule can be used.

#### Time Profiles¶

Time profiles of simulation results are created using the `plot_time_profile()` function.

```
[4]:
```

```
from mass.visualization.time_profiles import (
    plot_time_profile, get_time_profile_default_kwargs)
```

The minimal input required is a `MassSolution` object:

```
[5]:
```

```
plot_time_profile(conc_sol);
```

```
[5]:
```

```
<matplotlib.axes._subplots.AxesSubplot at 0x7f9781685310>
```

A linear x-axis and a linear y-axis are used by default. The `plot_function` kwarg is used to change the scale of the axes. For example, to view the plot with a linear x-axis and a logarithmic y-axis:

```
[6]:
```

```
plot_time_profile(conc_sol, plot_function="semilogx");
```

```
[6]:
```

```
<matplotlib.axes._subplots.AxesSubplot at 0x7f9781729e90>
```

A legend can be added to the plot simply by passing a valid legend location to the `legend` argument (valid legend locations can be found here). Solution labels correspond to `MassSolution` keys.

```
[7]:
```

```
plot_time_profile(conc_sol, plot_function="semilogx",
                  legend="right outside");
```

```
[7]:
```

```
<matplotlib.axes._subplots.AxesSubplot at 0x7f97818fd0d0>
```

Legend entries can be changed from their defaults by passing an iterable containing legend labels. The format must be `(labels, location)`, and the number of labels must correspond to the number of new items being plotted.

```
[8]:
```

```
labels = ["S" + str(i) for i in range(len(conc_sol.keys()))]
plot_time_profile(conc_sol, plot_function="semilogx",
                  legend=(labels, "right outside"));
```

```
[8]:
```

```
<matplotlib.axes._subplots.AxesSubplot at 0x7f9781cd0c50>
```

Whenever a plot is generated, the `Axes` containing the plot is returned by the plotting function. If a plot is created and no `Axes` object is provided, the most current `Axes` instance is used and returned.

An `Axes` instance for plotting can be provided to the `ax` argument, allowing for multiple plots to be placed on a single figure.

```
[9]:
```

```
fig, (ax1, ax2) = plt.subplots(nrows=2, ncols=1, figsize=(6, 8))

# Concentration solutions
plot_time_profile(
    conc_sol, ax=ax1, legend="right outside",
    plot_function="semilogx");

# Flux solutions
plot_time_profile(
    flux_sol, ax=ax2, legend="right outside",
    plot_function="semilogx");
```

```
[9]:
```

```
<matplotlib.axes._subplots.AxesSubplot at 0x7f97820b4a10>
```

Axes labels and a title are set at the time of plotting using the `xlabel`, `ylabel`, and `title` kwargs. Each argument takes either a `str` for the label or a tuple that contains the label and a `dict` of font properties. The `Figure.tight_layout()` method is used to prevent overlapping labels.

```
[10]:
```

```
fig, (ax1, ax2) = plt.subplots(nrows=2, ncols=1, figsize=(9, 8))

# Concentration solutions
plot_time_profile(
    conc_sol, ax=ax1, legend="right outside",
    plot_function="semilogx",
    xlabel=("Time (hrs)", {"size": "x-small"}),
    ylabel=("Concentrations (mM)", {"size": "medium"}),
    title=("Time profile of Concentrations", {"size": "x-large"}));

# Flux solutions
plot_time_profile(
    flux_sol, ax=ax2, legend="right outside",
    plot_function="semilogx",
    xlabel="Time (hrs)",
    ylabel="Fluxes (mM/hr)",
    title="Time profile of Fluxes");
fig.tight_layout()
```

The `observable` argument is used to view a subset of solutions. The `observable` argument requires an iterable of strings or objects with identifiers that correspond to `MassSolution` keys. Both are shown below:

```
[11]:
```

```
fig, (ax1, ax2) = plt.subplots(nrows=2, ncols=1, figsize=(6, 8))

# Concentration solutions
plot_time_profile(
    conc_sol,
    observable=["atp_c", "adp_c"],  # Using strings
    ax=ax1, legend="best",
    plot_function="semilogx",
    xlabel=("Time (hrs)", {"size": "x-small"}),
    ylabel=("Concentrations (mM)", {"size": "medium"}),
    title=("Time profile of Concentrations", {"size": "x-large"}));

# Flux solutions
plot_time_profile(
    flux_sol,
    observable=list(model.metabolites.atp_c.reactions),  # Using objects
    ax=ax2, legend="lower outside",
    plot_function="semilogx",
    xlabel="Time (hrs)",
    ylabel="Fluxes (mM/hr)",
    title="Time profile of Fluxes",
    legend_ncol=6);
fig.tight_layout()
```

To view how solutions deviate from their initial value (i.e., `MassModel.initial_conditions` for concentrations and `MassModel.steady_state_fluxes` for fluxes), the `deviation` kwarg can be set as `True`.

```
[12]:
```

```
fig, (ax1, ax2) = plt.subplots(nrows=2, ncols=1, figsize=(9, 8))

# Concentration solutions
plot_time_profile(
    conc_sol, ax=ax1, legend="right outside",
    plot_function="semilogx",
    xlabel=("Time (hrs)", {"size": "x-small"}),
    ylabel=("Relative Deviation\n" + r"($x/x_{0}$)",
            {"size": "x-large"}),
    title=("Time profile of Concentration Deviations",
           {"size": "x-large"}),
    deviation=True,
    deviation_normalization="initial value" # divide by initial value
);

# Flux solutions
plot_time_profile(
    flux_sol,
    ax=ax2, legend="right outside",
    plot_function="semilogx",
    xlabel="Time (hrs)",
    ylabel=("Relative Deviation\n" +\
            r"($\frac{v - v_{0}}{v_{max} - v_{min}}$)"),
    title="Time profile of Flux Deviations",
    deviation=True,
    deviation_zero_centered=True,   # Center deviation around 0
    deviation_normalization="range" # divide by value range
);
fig.tight_layout()
```

All possible kwargs and their default values for functions of the `mass.visualization.time_profiles` submodule can be retrieved using the `get_time_profile_default_kwargs()` function:

```
[13]:
```

```
sorted(get_time_profile_default_kwargs(
    function_name="plot_time_profile"))
```

```
[13]:
```

```
['annotate_time_points',
 'annotate_time_points_color',
 'annotate_time_points_labels',
 'annotate_time_points_legend',
 'annotate_time_points_marker',
 'annotate_time_points_markersize',
 'annotate_time_points_zorder',
 'color',
 'deviation',
 'deviation_normalization',
 'deviation_zero_centered',
 'grid',
 'grid_color',
 'grid_linestyle',
 'grid_linewidth',
 'legend_ncol',
 'linestyle',
 'linewidth',
 'marker',
 'markersize',
 'plot_function',
 'prop_cycle',
 'time_vector',
 'title',
 'xlabel',
 'xlim',
 'xmargin',
 'ylabel',
 'ylim',
 'ymargin',
 'zorder']
```

See the visualization submodule documentation for more information on possible kwargs.

#### Phase Portraits¶

To plot phase portraits of dynamic responses against each other, use the `plot_phase_portrait()` function.

```
[14]:
```

```
from mass.visualization.phase_portraits import (
    plot_tiled_phase_portraits, plot_phase_portrait,
    get_phase_portrait_default_kwargs)
```

The minimal input for a phase portrait includes a `MassSolution` object and two solution keys.

```
[15]:
```

```
plot_phase_portrait(
    flux_sol,
    x="ATPM",  # Using a string
    y=model.reactions.GAPD,  # Using an object
);
```

```
[15]:
```

```
<matplotlib.axes._subplots.AxesSubplot at 0x7f9763c76810>
```

As with time profiles, the `plot_phase_portrait()` function has an `ax` argument that takes an `Axes` instance and a `legend` argument for legend labels and position. A title and axes labels also can be placed on the plot.

```
[16]:
```

```
fig, ax = plt.subplots(nrows=1, ncols=1, figsize=(5, 5))
plot_phase_portrait(
    flux_sol, x="ATPM", y="GAPD", ax=ax, legend="best",
    xlabel="ATPM flux (mM/hr)", ylabel="GAPD flux (mM/hr)",
    title=("ATPM vs. GAPD", {"size": "large"}));
```

```
[16]:
```

```
<matplotlib.axes._subplots.AxesSubplot at 0x7f9763ea7890>
```

The `color` and `linestyle` kwargs are used to set the line color and style.

```
[17]:
```

```
fig, ax = plt.subplots(nrows=1, ncols=1, figsize=(5, 5))
plot_phase_portrait(
    flux_sol, x="ATPM", y="GAPD", ax=ax, legend="best",
    color="orange", linestyle="-");
```

```
[17]:
```

```
<matplotlib.axes._subplots.AxesSubplot at 0x7f9763fa8690>
```

Axes limits are set using the `xlim` and `ylim` arguments with tuples of format `(minimum, maximum)`.

```
[18]:
```

```
fig, ax = plt.subplots(nrows=1, ncols=1, figsize=(5, 5))
plot_phase_portrait(
    flux_sol, x="ATPM", y="GAPD", ax=ax, legend="best",
    color="orange", linestyle="-",
    xlim=(1.5, 3.5), ylim=(1.5, 3.5));
```

```
[18]:
```

```
<matplotlib.axes._subplots.AxesSubplot at 0x7f97640a3d10>
```

To call out a particular time point in the solution, the `annotate_time_points` kwarg is used with a list of time points to annotate. There are kwargs, such as `annotate_time_points_color` and `annotate_time_points_legend`, that allow for some customization of the annotated time points.

```
[19]:
```

```
fig, ax = plt.subplots(nrows=1, ncols=1, figsize=(5, 5))

# Createt time points and colors for the time points
time_points = [0, 1e-1, 1e0, 1e1, 1e2, 1e3]
time_point_colors = [
    mpl.colors.to_hex(c)
    for c in mpl.cm.Blues(np.linspace(0.3, 1, len(time_points)))]

# Plot the phase portrait
plot_phase_portrait(
    flux_sol, x="ATPM", y="GAPD", ax=ax, legend="upper right",
    xlim=(1.5, 3.5), ylim=(1.5, 3.5),
    title="ATPM vs. GAPD",
    color="orange", linestyle="-",
    annotate_time_points=time_points,
    annotate_time_points_color=time_point_colors,
    annotate_time_points_legend="right outside");
```

```
[19]:
```

```
<matplotlib.axes._subplots.AxesSubplot at 0x7f976418a3d0>
```

Because all figures are generated using **matplotlib**, additional lines can be plotted, and annotations can be placed on the plot using various **matplotlib** methods.

```
[20]:
```

```
fig, ax = plt.subplots(nrows=1, ncols=1, figsize=(5, 5))

# Plot a line representing steady state
ax.plot([1.5, 3.5], [1.5, 3.5], label="Steady State Line",
        color="grey", linestyle=":")

# Plot the phase portrait
plot_phase_portrait(
    flux_sol, x="ATPM", y="GAPD", ax=ax, legend="upper right",
    xlim=(1.5, 3.5), ylim=(1.5, 3.5),
    title="ATPM vs. GAPD",
    color="orange", linestyle="-",
    annotate_time_points=time_points,
    annotate_time_points_color=time_point_colors,
    annotate_time_points_legend="right outside");

# Annotate arrow for initial perturbation
xy = (flux_sol["ATPM"][0],
      flux_sol["GAPD"][0])
xytext = (model.reactions.get_by_id("ATPM").steady_state_flux,
          model.reactions.get_by_id("GAPD").steady_state_flux)
ax.annotate("", xy=xy,
            xytext=xytext, textcoords="data",
            arrowprops=dict(arrowstyle="->", connectionstyle="arc3"));
# Add arrow label
ax.annotate(
    "initial perturbation", xy=xy, xytext=(-120, 10),
    textcoords="offset pixels");
# Add text about the behavior on each side of the steady state line
ax.annotate(
    "Efflux < Influx", xy=(0.65, 0.05), xycoords="axes fraction",
    bbox=dict(fc="white", ec="black"));
ax.annotate(
    "Efflux > Influx", xy=(0.05, 0.9), xycoords="axes fraction",
    bbox=dict(fc="white", ec="black"));
```

```
[20]:
```

```
Text(0.05, 0.9, 'Efflux > Influx')
```

All possible kwargs and their default values for the functions of the `mass.visualization.phase_portraits` submodule can be retrieved using the `get_phase_portrait_default_kwargs()` function:

```
[21]:
```

```
sorted(get_phase_portrait_default_kwargs(
    function_name="plot_phase_portrait"))
```

```
[21]:
```

```
['annotate_time_points',
 'annotate_time_points_color',
 'annotate_time_points_labels',
 'annotate_time_points_legend',
 'annotate_time_points_marker',
 'annotate_time_points_markersize',
 'annotate_time_points_zorder',
 'color',
 'deviation',
 'deviation_normalization',
 'deviation_zero_centered',
 'grid',
 'grid_color',
 'grid_linestyle',
 'grid_linewidth',
 'legend_ncol',
 'linestyle',
 'linewidth',
 'marker',
 'markersize',
 'plot_function',
 'prop_cycle',
 'time_vector',
 'title',
 'xlabel',
 'xlim',
 'xmargin',
 'ylabel',
 'ylim',
 'ymargin',
 'zorder']
```

See the visualization submodule documentation for more information on possible kwargs.

#### Plotting Comparisons¶

To compare two sets of data in **MASSpy**, use the `plot_comparison()` function.

```
[22]:
```

```
from mass.visualization.comparison import (
    plot_comparison, get_comparison_default_kwargs)

model_2 = mass.test.create_test_model("textbook")
```

The `plot_comparison()` function requires two objects and a string that indicates what to compare. For example, to compare the steady state fluxes between two `MassModel` objects:

```
[23]:
```

```
fig, ax = plt.subplots(nrows=1, ncols=1, figsize=(5, 5))

plot_comparison(
    x=model, y=model, compare="fluxes",
    ax=ax, legend="right outside",
    plot_function="plot",
    xlabel=model.id, ylabel=model.id);
```

```
[23]:
```

```
<matplotlib.axes._subplots.AxesSubplot at 0x7f97812f6a50>
```

By providing an iterable of object identifiers to the `observable` argument, the plotted results are filtered, which is especially useful when comparing the similar variables in different objects. The `xy_line` kwarg is used to add a “perfect” fit line to the visualization. For example, to compare concentrations of two different models, each containing glycolytic species:

```
[24]:
```

```
fig, ax = plt.subplots(nrows=1, ncols=1, figsize=(5, 5))

# Plot species from glycolysis model only
plot_comparison(
    x=model, y=model_2, compare="concentrations",
    observable=[m.id for m in model.metabolites],
    ax=ax, legend="right outside",
    plot_function="loglog",
    xlabel=model.id, ylabel=model_2.id,
    xy_line=True, xy_legend="best");
```

```
[24]:
```

```
<matplotlib.axes._subplots.AxesSubplot at 0x7f9781856fd0>
```

The `plot_comparison()` function compares different objects to one another as long as the `compare` argument is given an appropriate value. In the following example, a `pandas.Series`, containing steady state concentrations of the model after an ATP utilization perturbation, is compared to model concentrations before the perturbation.

```
[25]:
```

```
fig, ax = plt.subplots(nrows=1, ncols=1, figsize=(5, 5))

# Create a pandas.Series to compare to the model steady state fluxes
flux_series = pd.Series(conc_sol.to_frame().iloc[-1, :])

# Compare the pandas.Series to the model steady state fluxes
# Plot species from glycolysis model only
plot_comparison(
    x=model, y=flux_series, compare="concentrations",
    ax=ax, legend="right outside",
    plot_function="loglog",
    xlabel="Before perturbation", ylabel="After perturbation",
    xlim=(1e-2, 1e1), ylim=(1e-2, 1e1),
    xy_line=True, xy_legend="best");
```

```
[25]:
```

```
<matplotlib.axes._subplots.AxesSubplot at 0x7f9781923990>
```

All possible kwargs and their default values for the functions of the `mass.visualization.comparison` submodule can be retrieved using the `get_comparison_default_kwargs()` function:

```
[26]:
```

```
sorted(get_comparison_default_kwargs(
    function_name="plot_comparison"))
```

```
[26]:
```

```
['color',
 'grid',
 'grid_color',
 'grid_linestyle',
 'grid_linewidth',
 'legend_ncol',
 'marker',
 'markersize',
 'plot_function',
 'prop_cycle',
 'title',
 'xlabel',
 'xlim',
 'xmargin',
 'xy_legend',
 'xy_line',
 'xy_linecolor',
 'xy_linestyle',
 'xy_linewidth',
 'ylabel',
 'ylim',
 'ymargin']
```

See the visualization submodule documentation for more information on possible kwargs.

#### Additional Examples¶

For additional examples of detailed visualizations using **MASSpy**, see the following:

- Visualizing Catalytic Potentials of Glycolytic Regulatory Kinases

### Enzyme Modules¶

An “enzyme module” is defined as a mechanistic description of a reaction consisting of mass action rate laws for all known reaction steps [DZK+16]. In **MASSpy**, enzyme modules are represented by the `EnzymeModule` object.

To demonstrate the utility of an `EnzymeModule` object and how it aids in constructing mechanistic models of enzyme behavior, an `EnzymeModule` of hexokinase\(^{1, 2}\) is constructed and then merged with a model of glycolysis\(^{3}\) for verification.

#### Constructing Enzyme Modules¶

In order to construct the `EnzymeModule` of hexokinase, the following information is necessary:

1. The enzyme is a monomer.
2. The enzyme binding of substrates follows a random sequential mechanism.
3. The enzyme experiences product inhibtion and is competitively inhibited by 23DPG when complexed with D-glucose.

Total HEX1 Concentration\(^2\): \(\text{[HEX1]}\_{total} = 24 nM = 0.000024 mM\).

```
[1]:
```

```
from operator import attrgetter

from mass import MassMetabolite
from mass.enzyme_modules import EnzymeModule
from mass.test import create_test_model

# Load the glycolysis and hemoglobin models, then merge them
glycolysis = create_test_model("Glycolysis")
hemoglobin = create_test_model("Hemoglobin")
glyc_hb = glycolysis.merge(hemoglobin, inplace=False)
```

The `EnzymeModule` is a subclass of the `MassModel`, meaning that it inherits the methods and behaviors of the `MassModel` object. Like a `MassModel`, an `EnzymeModule` object requires a unique identifier in order to be created. Optionally, the `name` and `subsystem` attributes are set during initialization.

```
[2]:
```

```
HEX1 = EnzymeModule("HEX1", name="Hexokinase (D-glucose:ATP)",
                    subsystem="Glycolysis")
```

##### Defining the enzyme ligands¶

The ligands that interact with the enzyme (e.g. as the substrates, activators, and inhibitors) are created as `MassMetabolite` objects and added to the model.

```
[3]:
```

```
glc__D_c = MassMetabolite(
    "glc__D_c",
    name="D-Glucose",
    formula="C6H12O6",
    charge=0,
    compartment="c")
g6p_c = MassMetabolite(
    "g6p_c",
    name="D-Glucose 6-phosphate",
    formula="C6H11O9P",
    charge=-2,
    compartment="c")
atp_c = MassMetabolite(
    "atp_c",
    name="ATP",
    formula="C10H12N5O13P3",
    charge=-4,
    compartment="c")
adp_c = MassMetabolite(
    "adp_c",
    name="ADP",
    formula="C10H12N5O10P2",
    charge=-3,
    compartment="c")
_23dpg_c = MassMetabolite(
    "_23dpg_c",
    name="2,3-Disphospho-D-glycerate",
    formula="C3H3O10P2",
    charge=-5,
    compartment="c")
h_c = MassMetabolite(
    "h_c",
    name="H+",
    formula="H",
    charge=1,
    compartment="c")

HEX1.add_metabolites([glc__D_c, g6p_c, atp_c, adp_c, _23dpg_c, h_c])
```

Once added to the `EnzymeModule`, ligands can be accessed using the `enzyme_module_ligands` attribute.

```
[4]:
```

```
HEX1.enzyme_module_ligands
```

```
[4]:
```

```
[<MassMetabolite glc__D_c at 0x7ff42b809390>,
 <MassMetabolite g6p_c at 0x7ff42b809350>,
 <MassMetabolite atp_c at 0x7ff42b8093d0>,
 <MassMetabolite adp_c at 0x7ff42b809410>,
 <MassMetabolite _23dpg_c at 0x7ff42b809490>,
 <MassMetabolite h_c at 0x7ff42b8094d0>]
```

To keep track of the roles played by various ligands in the module, the `enzyme_module_ligands_categorized` attribute is set. The attribute takes a `dict`, with categories as keys and relevant `MassMetabolite` objects as values. Note that an object can be a part of multiple categories.

```
[5]:
```

```
HEX1.enzyme_module_ligands_categorized =  {
    "substrates": glc__D_c,
    "cofactors": atp_c,
    "inhibitors": _23dpg_c,
    "products": [adp_c, g6p_c, h_c]}
HEX1.enzyme_module_ligands_categorized
```

```
[5]:
```

```
[<Group substrates at 0x7ff42b809ad0>,
 <Group cofactors at 0x7ff42b809bd0>,
 <Group inhibitors at 0x7ff42b809c50>,
 <Group products at 0x7ff42b809cd0>]
```

For each category, a `cobra.Group` is created containing the relevant objects. Once set, the attribute returns a `cobra.DictList` that contains the categorized groups. The groups and their members are printed as follows:

```
[6]:
```

```
for group in HEX1.enzyme_module_ligands_categorized:
    print("{0}: {1}".format(
        group.id, str(sorted([m.id for m in group.members]))))
```

```
substrates: ['glc__D_c']
cofactors: ['atp_c']
inhibitors: ['_23dpg_c']
products: ['adp_c', 'g6p_c', 'h_c']
```

##### Defining the enzyme module forms¶

After adding `MassMetabolite` objects of ligands to the model, the various forms of the enzyme must be defined. These forms are represented by `EnzymeModuleForm` objects.

The `EnzymeModuleForm` object inherits from the `MassMetabolite` and is treated like any other metabolite in the model. However, the `EnzymeModuleForm` object contains the additional `bound_metabolites` attribute to assist in tracking metabolites bound to the enzyme form.

The `EnzymeModule.make_enzyme_module_form()` method allows for the creation of an `EnzymeModuleForm` object while assigning categories for the `EnzymeModuleForm` in the process. Using `make_enzyme_module_form()` also adds the species to the module upon creation, accessible via the `EnzymeModule.enzyme_module_forms` attribute.

```
[7]:
```

```
hex1_c = HEX1.make_enzyme_module_form(
    "hex1_c",
    name="automatic",
    categories="Active",
    compartment="c")

hex1_A_c = HEX1.make_enzyme_module_form(
    "hex1_A_c",  # A stands complexted with ATP
    name="automatic",
    categories="Active",
    bound_metabolites={atp_c: 1},
    compartment="c")

hex1_G_c = HEX1.make_enzyme_module_form(
    "hex1_G_c",  # G stands for complexed with Glucose
    name="automatic",
    categories="Active",
    bound_metabolites={glc__D_c: 1},
    compartment="c")

hex1_AG_c = HEX1.make_enzyme_module_form(
    "hex1_AG_c",
    name="automatic",
    categories="Active",
    bound_metabolites={glc__D_c: 1, atp_c: 1},
    compartment="c")

hex1_G_CI_c = HEX1.make_enzyme_module_form(
    "hex1_G_CI_c",  # CI stands for competitive inhibition
    name="automatic",
    categories="Inhibited",
    bound_metabolites={glc__D_c: 1, _23dpg_c: 1},
    compartment="c")

hex1_A_PI_c = HEX1.make_enzyme_module_form(
    "hex1_A_PI_c",  # PI stands for competitive inhibition
    name="automatic",
    categories="Inhibited",
    bound_metabolites={adp_c: 1},
    compartment="c")

hex1_G_PI_c = HEX1.make_enzyme_module_form(
    "hex1_G_PI_c",  # PI stands for competitive inhibition
    name="automatic",
    categories="Inhibited",
    bound_metabolites={g6p_c: 1},
    compartment="c")

HEX1.enzyme_module_forms
```

```
[7]:
```

```
[<EnzymeModuleForm hex1_c at 0x7ff42b82e750>,
 <EnzymeModuleForm hex1_A_c at 0x7ff42b82e790>,
 <EnzymeModuleForm hex1_G_c at 0x7ff42b82e850>,
 <EnzymeModuleForm hex1_AG_c at 0x7ff42b82e710>,
 <EnzymeModuleForm hex1_G_CI_c at 0x7ff42b82eb50>,
 <EnzymeModuleForm hex1_A_PI_c at 0x7ff42b82ee10>,
 <EnzymeModuleForm hex1_G_PI_c at 0x7ff42b82ed10>]
```

The `bound_metabolites` attribute represents the ligands bound to the site(s) of enzyme.

```
[8]:
```

```
# Print automatically generated names
for enzyme_form in HEX1.enzyme_module_forms:
    print("Bound to sites of {0}:\n{1}\n".format(
        enzyme_form.id, {
            ligand.id: coeff
            for ligand, coeff in enzyme_form.bound_metabolites.items()}))
```

```
Bound to sites of hex1_c:
{}

Bound to sites of hex1_A_c:
{'atp_c': 1}

Bound to sites of hex1_G_c:
{'glc__D_c': 1}

Bound to sites of hex1_AG_c:
{'glc__D_c': 1, 'atp_c': 1}

Bound to sites of hex1_G_CI_c:
{'glc__D_c': 1, '_23dpg_c': 1}

Bound to sites of hex1_A_PI_c:
{'adp_c': 1}

Bound to sites of hex1_G_PI_c:
{'g6p_c': 1}
```

Setting the `bound_metabolites` attribute upon creation allow the `formula` and `charge` attributes of the various forms also to be set while ensuring mass and charge balancing is maintained. Note that the enzyme is represented as a moiety, and the ligands bound to the enzyme are represented in the chemical formula.

```
[9]:
```

```
# Get the elemental matrix for the enzyme
df = HEX1.get_elemental_matrix(array_type="DataFrame")
# Use iloc to only look at EnzymeModuleForms
df.iloc[:, 6:]
```

```
[9]:
```

|  | hex1\_c | hex1\_A\_c | hex1\_G\_c | hex1\_AG\_c | hex1\_G\_CI\_c | hex1\_A\_PI\_c | hex1\_G\_PI\_c |
| --- | --- | --- | --- | --- | --- | --- | --- |
| C | 0.0 | 10.0 | 6.0 | 16.0 | 9.0 | 10.0 | 6.0 |
| H | 0.0 | 12.0 | 12.0 | 24.0 | 15.0 | 12.0 | 11.0 |
| O | 0.0 | 13.0 | 6.0 | 19.0 | 16.0 | 10.0 | 9.0 |
| P | 0.0 | 3.0 | 0.0 | 3.0 | 2.0 | 2.0 | 1.0 |
| N | 0.0 | 5.0 | 0.0 | 5.0 | 0.0 | 5.0 | 0.0 |
| S | 0.0 | 0.0 | 0.0 | 0.0 | 0.0 | 0.0 | 0.0 |
| q | 0.0 | -4.0 | 0.0 | -4.0 | -5.0 | -3.0 | -2.0 |
| [HEX] | 1.0 | 1.0 | 1.0 | 1.0 | 1.0 | 1.0 | 1.0 |

Setting the `name` argument as “automatic” in the `EnzymeModule.make_enzyme_module_form()` method causes a name for the `EnzymeModuleForm` to be generated based on the metabolites in the `bound_metabolites` attribute.

```
[10]:
```

```
# Print automatically generated names
for enzyme_form in HEX1.enzyme_module_forms:
    print(enzyme_form.name)
```

```
HEX1
HEX1-atp complex
HEX1-glc__D complex
HEX1-glc__D-atp complex
HEX1-glc__D-_23dpg complex
HEX1-adp complex
HEX1-g6p complex
```

The `categories` argument allows for `EnzymeModuleForm` objects to be placed into `cobra.Group` objects representing those categories. As with the ligands, the categorized enzyme module forms are returned in a `DictList` of `Group` objects by the `enzyme_module_forms_categorized` attribute.

```
[11]:
```

```
for group in HEX1.enzyme_module_forms_categorized:
    print("{0}: {1}".format(
        group.id, str(sorted([m.id for m in group.members]))))
```

```
Active: ['hex1_AG_c', 'hex1_A_c', 'hex1_G_c', 'hex1_c']
Inhibited: ['hex1_A_PI_c', 'hex1_G_CI_c', 'hex1_G_PI_c']
```

Alternatively, the `enzyme_module_forms_categorized` attribute can be set using a `dict`:

```
[12]:
```

```
HEX1.enzyme_module_forms_categorized =  {
    "competitively_inhibited": hex1_G_CI_c}

for group in HEX1.enzyme_module_forms_categorized:
    print("{0}: {1}".format(
        group.id, str(sorted([m.id for m in group.members]))))
```

```
Active: ['hex1_AG_c', 'hex1_A_c', 'hex1_G_c', 'hex1_c']
Inhibited: ['hex1_A_PI_c', 'hex1_G_CI_c', 'hex1_G_PI_c']
competitively_inhibited: ['hex1_G_CI_c']
```

##### Defining enzyme module reactions¶

The next step is to define all of the reaction steps that represent the catalytic mechanism and regulation of the enzyme module. These reactions are represented as `EnzymeModuleReaction` objects.

The `EnzymeModuleReaction` object inherits from the `MassReaction` and is treated like any other reaction in the model. Like the `make_enzyme_module_form()` method, the `make_enzyme_module_reaction()` method allows for the creation of an `EnzymeModuleReaction` object while assigning categories for the `EnzymeModuleReaction` in the process.

Species that exist in the model can also be added to the reaction by providing a dictionary of metabolites and their stoichiometric coefficients to the `metabolites_to_add` argument.

```
[13]:
```

```
HEX1_1 = HEX1.make_enzyme_module_reaction(
    "HEX1_1",
    name="Automatic",
    subsystem="Glycolysis",
    reversible=True,
    categories="product_inhibition",
    metabolites_to_add={
        "hex1_c": -1,
        "adp_c": -1,
        "hex1_A_PI_c": 1})

HEX1_2 = HEX1.make_enzyme_module_reaction(
    "HEX1_2",
    name="Automatic",
    subsystem="Glycolysis",
    reversible=True,
    categories="product_inhibition",
    metabolites_to_add={
        "hex1_c": -1,
        "g6p_c": -1,
        "hex1_G_PI_c": 1})

HEX1_3 = HEX1.make_enzyme_module_reaction(
    "HEX1_3",
    name="Automatic",
    subsystem="Glycolysis",
    reversible=True,
    categories="glc__D_c_binding",
    metabolites_to_add={
        "hex1_c": -1,
        "glc__D_c": -1,
        "hex1_G_c": 1})

HEX1_4 = HEX1.make_enzyme_module_reaction(
    "HEX1_4",
    name="Automatic",
    subsystem="Glycolysis",
    reversible=True,
    categories="atp_c_binding",
    metabolites_to_add={
        "hex1_c": -1,
        "atp_c": -1,
        "hex1_A_c": 1})

HEX1_5 = HEX1.make_enzyme_module_reaction(
    "HEX1_5",
    name="Automatic",
    subsystem="Glycolysis",
    reversible=True,
    categories="competitive_inhibition",
    metabolites_to_add={
        "hex1_G_c": -1,
        "_23dpg_c": -1,
        "hex1_G_CI_c": 1})

HEX1_6 = HEX1.make_enzyme_module_reaction(
    "HEX1_6",
    name="Automatic",
    subsystem="Glycolysis",
    reversible=True,
    categories="atp_c_binding",
    metabolites_to_add={
        "hex1_G_c": -1,
        "atp_c": -1,
        "hex1_AG_c": 1})

HEX1_7 = HEX1.make_enzyme_module_reaction(
    "HEX1_7",
    name="Automatic",
    subsystem="Glycolysis",
    reversible=True,
    categories="glc__D_c_binding",
    metabolites_to_add={
        "hex1_A_c": -1,
        "glc__D_c": -1,
        "hex1_AG_c": 1})

HEX1_8 = HEX1.make_enzyme_module_reaction(
    "HEX1_8",
    name="Automatic",
    subsystem="Glycolysis",
    reversible=True,
    categories="catalyzation",
    metabolites_to_add={
        "hex1_AG_c": -1,
        "hex1_c": 1,
        "adp_c": 1,
        "g6p_c": 1,
        "h_c": 1})

for reaction in HEX1.enzyme_module_reactions:
    print(reaction)
```

```
HEX1_1: adp_c + hex1_c <=> hex1_A_PI_c
HEX1_2: g6p_c + hex1_c <=> hex1_G_PI_c
HEX1_3: glc__D_c + hex1_c <=> hex1_G_c
HEX1_4: atp_c + hex1_c <=> hex1_A_c
HEX1_5: _23dpg_c + hex1_G_c <=> hex1_G_CI_c
HEX1_6: atp_c + hex1_G_c <=> hex1_AG_c
HEX1_7: glc__D_c + hex1_A_c <=> hex1_AG_c
HEX1_8: hex1_AG_c <=> adp_c + g6p_c + h_c + hex1_c
```

The `categories` argument allows for `EnzymeModuleReactions` objects to be placed into `cobra.Group` objects representing those categories. As with the ligands and enzyme forms, a `DictList` of the relevant groups are returned with the `enzyme_module_reactions_categorized` attribute.

```
[14]:
```

```
HEX1.enzyme_module_reactions_categorized
```

```
[14]:
```

```
[<Group product_inhibition at 0x7ff42b87ae10>,
 <Group glc__D_c_binding at 0x7ff42b87c650>,
 <Group atp_c_binding at 0x7ff42b87ca50>,
 <Group competitive_inhibition at 0x7ff42b87cc90>,
 <Group catalyzation at 0x7ff42b85ef50>]
```

###### Unifying rate parameters¶

For this `EnzymeModule`, the reactions representing glucose binding to the enzyme and ATP binding to the enzyme have the same forward rate and equilibrium constants. Instead of defining the parameter values for each individual reaction, the `unify_rate_parameters()` method can be used to create custom rate laws for the given reactions that all depend on the same rate parameters.

The `unify_rate_parameters()` method takes a list of reactions and an identifier to use for the unified parameter. The `enzyme_prefix` flag can be set to `True` to prefix the new parameter identifier with the identifier of the `EnzymeModule`, ensuring that any existing custom parameters are not overwritten.

```
[15]:
```

```
for ligand, pid in zip([glc__D_c, atp_c],["G", "A"]):
    # Get the group of reactions corresponding to the ligand
    category = "_".join((ligand.id, "binding"))
    group = HEX1.enzyme_module_reactions_categorized.get_by_id(category)

    # Unify the parameters
    HEX1.unify_rate_parameters(
        group.members, new_parameter_id=pid, enzyme_prefix=True)

    # Print the new reaction rates
    print("\n" + category + "\n" + "-" * len(category))
    for reaction in sorted(group.members, key=attrgetter("id")):
        print(reaction.id + ": " + str(reaction.rate))
```

```
glc__D_c_binding
----------------
HEX1_3: kf_HEX1_G*(glc__D_c(t)*hex1_c(t) - hex1_G_c(t)/Keq_HEX1_G)
HEX1_7: kf_HEX1_G*(glc__D_c(t)*hex1_A_c(t) - hex1_AG_c(t)/Keq_HEX1_G)

atp_c_binding
-------------
HEX1_4: kf_HEX1_A*(atp_c(t)*hex1_c(t) - hex1_A_c(t)/Keq_HEX1_A)
HEX1_6: kf_HEX1_A*(atp_c(t)*hex1_G_c(t) - hex1_AG_c(t)/Keq_HEX1_A)
```

#### Determining Enzyme Form Concentrations and Rate Constants¶

The next step is to solve for the steady state concentrations for the various forms of the enzyme symbolically using **SymPy**. Because the numerical values for the dissociation constants have been defined, these equations are solved in terms of the rate constants. The rate constants can be approximated using the total enzyme concentration as a constraint and substituted back into the equations to calculate the numerical values of the steady state concentrations.

```
[16]:
```

```
from sympy import Eq, Symbol, lambdify, simplify, solveset

from mass import strip_time
from mass.util.matrix import matrix_rank
```

##### Solving steady state concentrations symbolically¶

To get the symbolic solutions for the individual enzyme forms, the ODEs are first collected in a `dict`. Keys are the enzyme forms, and values are their ODEs with the time dependency stripped via the `strip_time` function.

```
[17]:
```

```
ode_dict = {
    enzyme_form.id: Eq(strip_time(enzyme_form.ode), 0)
    for enzyme_form in HEX1.enzyme_module_forms}
# Matrix rank of enzyme stoichiometric matrix without substrates
rank = matrix_rank(HEX1.S[6:])
print("Rank Deficiency: {0}".format(len(ode_dict) - rank))
```

```
Rank Deficiency: 1
```

Because the stoichiometric matrix (without ligands) has a rank deficiency of one, there is a dependent variable in the system unless another equation is added. Therefore, the completely free enzyme form is treated as the dependent variable, and all of the enzyme forms are solved in terms of the free enzyme form.

```
[18]:
```

```
enzyme_solutions = {}
for enzyme_form in HEX1.enzyme_module_forms:
    # Skip dependent variable
    if enzyme_form.id == "hex1_c":
        continue
    # Get the ODE for the enzyme form from the ODE dict
    equation = ode_dict[enzyme_form.id]
    # Solve the equation for the enzyme form, substituting
    # previously found enzyme form solutions into the equation
    solution = solveset(equation.subs(enzyme_solutions),
                        enzyme_form.id)
    # Store the solution
    enzyme_solutions[enzyme_form.id] = list(solution)[0]
    # Substitute the new solution into existing solutions
    enzyme_solutions.update({
        enzyme_form: sol.subs(enzyme_solutions)
        for enzyme_form, sol in enzyme_solutions.items()})

args = set()
for solution in enzyme_solutions.values():
    args.update(solution.atoms(Symbol))
```

###### Defining the Rate Equation¶

To make up for the rank deficiency, an additional equation is needed. Typically, the rate of the enzyme is the summation of the rates for the catalyzation reaction step(s) of the enzyme. The `make_enzyme_rate_equation()` method can be used to create the rate equation from a list of reactions. If `use_rates=True`, the rate expressions of the reactions are added together. If `update_enzyme=True`, the rate equation is set as a symbolic expression for the `enzyme_rate_equation` attribute.

```
[19]:
```

```
# Get the catalyzation reactions
catalyzation_group = HEX1.enzyme_module_reactions_categorized.get_by_id(
    "catalyzation")

HEX1.make_enzyme_rate_equation(catalyzation_group.members,
                               use_rates=True,
                               update_enzyme=True)

print(HEX1.enzyme_rate_equation)
```

```
kf_HEX1_8*(Keq_HEX1_8*hex1_AG_c(t) - adp_c(t)*g6p_c(t)*hex1_c(t))/Keq_HEX1_8
```

With the rate equation defined, the `enzyme_rate_error()` method is used to get the equation as the difference between the flux value and the rate equation.

```
[20]:
```

```
enzyme_rate_equation = strip_time(HEX1.enzyme_rate_error(use_values=False))
print(enzyme_rate_equation)
```

```
v_HEX1 - kf_HEX1_8*(Keq_HEX1_8*hex1_AG_c - adp_c*g6p_c*hex1_c)/Keq_HEX1_8
```

The solutions for the enzyme forms are substituted into the rate equation, and the equation is solved for the free enzyme form. The solutions are subsequently updated, resulting in symbolic equations that do not depend on any enzyme form.

```
[21]:
```

```
# Solve for last unknown concentration symbolically
solution = solveset(enzyme_rate_equation.subs(enzyme_solutions),
                    "hex1_c")

# Update solution dictionary with the new solution
enzyme_solutions["hex1_c"] = list(solution)[0]

# Update solutions with free variable solutions
enzyme_solutions = {
    enzyme_form: simplify(solution.subs(enzyme_solutions))
    for enzyme_form, solution in enzyme_solutions.items()}

args = set()
for solution in enzyme_solutions.values():
    args.update(solution.atoms(Symbol))
print(args)
```

```
{g6p_c, kf_HEX1_8, kf_HEX1_A, v_HEX1, Keq_HEX1_A, atp_c, glc__D_c, _23dpg_c, kf_HEX1_G, Keq_HEX1_5, Keq_HEX1_8, adp_c, Keq_HEX1_2, Keq_HEX1_1, Keq_HEX1_G}
```

Numerical values for known quantities are substituted into the equations. For this `EnzymeModule` of Hexokinase, the following dissociation constants are used:

\[\begin{split}\begin{align}
K\_{d,\ \text{GLC-D}} &= 0.038\ \text{mM} \\
K\_{d,\ \text{ATP}} &= 2.06\ \text{mM} \\
K\_{i,\ \text{23DPG}} &= 5.5\ \text{mM} \\
K\_{i,\ \text{ADP}} &= 1\ \text{mM} \\
K\_{i,\ \text{G6P}} &= 66.67\ \text{mM} \\
\end{align}\end{split}\]

A value of \(K\_{\text{HEX1}}= 313.12\) is used for the catalyzation step. Note that the inverse of the dissociation constant is used for reactions that form complexes.

```
[22]:
```

```
numerical_values = {
    "Keq_HEX1_1": 1,
    "Keq_HEX1_2": 1 / 66.67,
    "Keq_HEX1_G": 1 / 0.038,
    "Keq_HEX1_A": 1 / 2.06,
    "Keq_HEX1_5": 1 / 5.5,
    "Keq_HEX1_8": 313.12}
# Update the model with the parameters
HEX1.update_parameters(numerical_values)
```

The ligand concentrations and the rate for the enzyme are extracted from the merged glycolysis and hemoglobin model.

```
[23]:
```

```
# Get steady state flux for EnzymeModule
HEX1.enzyme_rate = glyc_hb.reactions.get_by_id("HEX1").steady_state_flux
numerical_values[HEX1.enzyme_flux_symbol_str] = HEX1.enzyme_rate

# Get the ligand concentrations
for met in HEX1.enzyme_module_ligands:
    concentration = glyc_hb.metabolites.get_by_id(met.id).initial_condition
    # Set the ligand initial condition and add to numercal values dictionary
    met.initial_condition = concentration
    numerical_values[met.id] = concentration
```

The numerical values are substituted into the symbolic equations, resulting in the steady state concentrations that depend only on the rate constants.

```
[24]:
```

```
enzyme_solutions = {
    enzyme_form: simplify(sol.subs(numerical_values))
    for enzyme_form, sol in enzyme_solutions.items()}

args = set()
for solution in enzyme_solutions.values():
    args.update(solution.atoms(Symbol))
print(args)
```

```
{kf_HEX1_A, kf_HEX1_G, kf_HEX1_8}
```

##### Approximating Rate Constants¶

To determine the set of rate constants for the enzyme module, the absolute error between the total hexokinase concentration value (found in literature) and the computed hexokinase concentration is minimized. For this example, the `minimize()` function of the **SciPy** package is utilized to find a feasible set of rate constants.

```
[25]:
```

```
from scipy.optimize import minimize
```

The objective function for the minimization is first made symbolically. The `enzyme_total_symbol_str` property can be used to represent the total enzyme concentration, while the `enzyme_concentration_total_equation` property creates a symbolic expression for the sum of all enzyme forms.

```
[26]:
```

```
enzyme_total_error = abs(
    Symbol(HEX1.enzyme_total_symbol_str)
    - strip_time(HEX1.enzyme_concentration_total_equation))
print(enzyme_total_error)
```

```
Abs(-HEX1_Total + hex1_AG_c + hex1_A_PI_c + hex1_A_c + hex1_G_CI_c + hex1_G_PI_c + hex1_G_c + hex1_c)
```

The `enzyme_concentration_total` attribute stores the total amount of enzyme in the model and substituted into the expression. The total HEX1 concentration is \(24 \* 10^{-6} \text{mM}\).

```
[27]:
```

```
HEX1.enzyme_concentration_total = 24e-6
enzyme_total_error = enzyme_total_error.subs({
    HEX1.enzyme_total_symbol_str: HEX1.enzyme_concentration_total})
print(enzyme_total_error)
```

```
Abs(hex1_AG_c + hex1_A_PI_c + hex1_A_c + hex1_G_CI_c + hex1_G_PI_c + hex1_G_c + hex1_c - 2.4e-5)
```

Finally, the symbolic equations for the enzyme forms are substituted into the enzyme total error equation, resulting in an expression that represents the objective function with the only unknown variables being rate constants. The `lambdify()` function of the **SymPy** package converts the symbolic objective into a lambda function that can be used with the `minimize()` function of **SciPy**.

```
[28]:
```

```
enzyme_total_error = simplify(enzyme_total_error.subs(enzyme_solutions))

# Sort the arguments to ensure input format remains consistent
args = sorted(list(map(str, args)))
# Use lambdify to make objective function as a lambda function
obj_fun = lambda x: lambdify(args, enzyme_total_error)(*x)
```

The `minimize()` function is now used to approximate the rate constants. The optimization problems for enzyme rate constants are typically nonlinear, and require nonlinear optimization routines to find feasible solutions.

```
[29]:
```

```
# Minimize the objective function, initial guess based on publication values
initial_guess = [1e8, 9376585, 52001]
variable_bounds = ((0, 1e9), (0, 1e9), (0, 1e9))
solution = minimize(obj_fun, x0=initial_guess,
                    method="trust-constr",
                    bounds=variable_bounds)
# Map solution array to variables
rate_constants = dict(zip(args, solution.x))
print(rate_constants)
```

```
{'kf_HEX1_8': 100000000.0025878, 'kf_HEX1_A': 9376585.030755484, 'kf_HEX1_G': 52006.59981223971}
```

Because the rate constants associated with the inhibition of the enzyme forms are not necessary for computing the concentrations, a rapid binding assumption is made for the inhibition reactions. Therefore, a large number is set for the rate constants. The parameters are set using the `update_parameters()` method.

```
[30]:
```

```
rate_constants["kf_HEX1_1"] = 1e6
rate_constants["kf_HEX1_2"] = 1e6
rate_constants["kf_HEX1_5"] = 1e6
HEX1.update_parameters(rate_constants)
```

##### Calculating numerical values for concentrations¶

Once the rate constants have been estimated, they are substituted back into the symbolic concentration equations in order to obtain their numerical values.

```
[31]:
```

```
for enzyme_form, solution in enzyme_solutions.items():
    # Get the enzyme form object, determine the steady state concentration
    enzyme_form = HEX1.enzyme_module_forms.get_by_id(enzyme_form)
    enzyme_form.initial_condition = float(solution.subs(rate_constants))
    print("{0}: {1:e}".format(enzyme_form.id,
                              enzyme_form.initial_condition))
```

```
hex1_A_c: 9.401421e-06
hex1_G_c: 5.718872e-08
hex1_AG_c: 1.174630e-08
hex1_G_CI_c: 3.223364e-08
hex1_A_PI_c: 3.519706e-06
hex1_G_PI_c: 8.847367e-09
hex1_c: 1.213692e-05
```

###### Error values¶

As a quality assurance check, the `enzyme_concentration_total_error()` method can be used to get the error between the `enzyme_concentration_total` attribute and the sum of the enzyme form concentrations. A positive value indicates the `enzyme_concentration_total` attribute is greater than the sum of the individual enzyme form concentrations that were computed.

```
[32]:
```

```
print("Total Enzyme Concentration Error: {0}".format(
    HEX1.enzyme_concentration_total_error(use_values=True)))
```

```
Total Enzyme Concentration Error: -1.1680622689093244e-06
```

Similarly, the error between the `enzyme_rate` attribute and the computed value from the `enzyme_rate_equation` can be also checked using the `enzyme_rate_error()` method, in which a positive value indicates that the `enzyme_rate` attribute is greater than the value computed when using the rate equation.

```
[33]:
```

```
print("Enzyme Rate Error: {0}".format(
    HEX1.enzyme_rate_error(use_values=True)))
```

```
Enzyme Rate Error: 4.440892098500626e-16
```

#### Adding EnzymeModules to Models¶

With the `EnzymeModule` built, it can be integrated into a larger network and simulated. To add an `EnzymeModule` to an existing `MassModel`, the `merge()` method is used. After merging, the `remove_reactions()` method is used to remove the reaction replaced with the enzyme module. The `EnzymeModule` should always be merged into the `MassModel` as demonstrated below:

```
[34]:
```

```
glyc_hb_HEX1 = glyc_hb.merge(HEX1, inplace=False)
glyc_hb_HEX1.remove_reactions([
    glyc_hb_HEX1.reactions.get_by_id("HEX1")])
```

All objects, numerical values, and certain attributes of the `EnzymeModule` are transferred into the `MassModel` upon merging. This includes all enzyme forms, reactions steps, initial conditions, rate parameters, and category groups.

```
[35]:
```

```
glyc_hb_HEX1
```

```
[35]:
```

|  |  |
| --- | --- |
| **Name** | Glycolysis\_Hemoglobin\_HEX1 |
| **Memory address** | 0x07ff42b8e6d90 |
| **Stoichiometric Matrix** | 35x37 |
| **Matrix Rank** | 32 |
| **Number of metabolites** | 35 |
| **Initial conditions defined** | 35/35 |
| **Number of reactions** | 37 |
| **Number of genes** | 0 |
| **Number of enzyme modules** | 1 |
| **Number of groups** | 12 |
| **Objective expression** | 0 |
| **Compartments** | Cytosol |

##### The EnzymeModuleDict object¶

During the merge process, an `EnzymeModuleDict` is created from the `EnzymeModule` and added to the `MassModel.enzyme_modules` attribute.

```
[36]:
```

```
print(glyc_hb_HEX1.enzyme_modules)
HEX1_dict = glyc_hb_HEX1.enzyme_modules.get_by_id("HEX1")
HEX1_dict
```

```
[<EnzymeModuleDict HEX1 at 0x7ff42bab8950>]
```

```
[36]:
```

|  |  |
| --- | --- |
| **Name** | HEX1 |
| **Memory address** | 0x07ff42bab8950 |
| **Stoichiometric Matrix** | 13x8 |
| **Matrix Rank** | 7 |
| **Subsystem** | Glycolysis |
| **Number of Ligands** | 6 |
| **Number of EnzymeForms** | 7 |
| **Number of EnzymeModuleReactions** | 8 |
| **Enzyme Concentration Total** | 2.4e-05 |
| **Enzyme Net Flux** | 1.12 |

The `EnzymeModuleDict` inherits from an `OrderedDict`, thereby inheriting ordered dictionary methods such as `keys()`:

```
[37]:
```

```
print("\n".join(HEX1_dict.keys()))
```

```
id
name
subsystem
enzyme_module_ligands
enzyme_module_forms
enzyme_module_reactions
enzyme_module_ligands_categorized
enzyme_module_forms_categorized
enzyme_module_reactions_categorized
enzyme_concentration_total
enzyme_rate
enzyme_concentration_total_equation
enzyme_rate_equation
S
model
```

The `EnzymeModuleDict` stores several of the enzyme-specific attributes so that they are still accessible after integrating the enzyme module into a larger network. The keys of the `EnzymeModuleDict` also can be treated as attribute accessors:

```
[38]:
```

```
print("Enzyme Rate:\n{0} = {1}".format(
    HEX1_dict["enzyme_rate"],       # Returned using dict key
    HEX1_dict.enzyme_rate_equation  # Returned using attribute accessor
))
```

```
Enzyme Rate:
1.12 = kf_HEX1_8*(Keq_HEX1_8*hex1_AG_c(t) - adp_c(t)*g6p_c(t)*hex1_c(t))/Keq_HEX1_8
```

##### Steady State Validation¶

The last step is to ensure that a steady state is reached with the completed enzyme module within a larger network context.

```
[39]:
```

```
import matplotlib.pyplot as plt

from mass import Simulation
from mass.visualization import plot_time_profile
```

Here, the model is simulated, and the enzyme’s ability to reach steady state is graphically verified:

```
[40]:
```

```
# Setup simulation object
sim = Simulation(glyc_hb_HEX1, verbose=True)
# Simulate from 0 to 1000 with 10001 points in the output
conc_sol, flux_sol = sim.simulate(
    glyc_hb_HEX1, time=(0, 1e3, 1e4 + 1))

fig, ax = plt.subplots(nrows=1, ncols=1, figsize=(6, 4))
plot_time_profile(
    conc_sol, observable=HEX1_dict.enzyme_module_forms, ax=ax,
    legend="right outside", plot_function="loglog",
    xlabel="Time [hr]", ylabel="Concentration [mM]",
    title="TIme profile of Concentrations for Enzyme Forms");
```

```
Successfully loaded MassModel 'Glycolysis_Hemoglobin_HEX1' into RoadRunner.
```

```
[40]:
```

```
<matplotlib.axes._subplots.AxesSubplot at 0x7ff426323c10>
```

The plot shows that the enzyme can reach a steady state when integrated into a larger network, meaning the enzyme module that represents hexokinase in this system is complete!

#### Additional Examples¶

For additional examples of analyzing and visualizing systems with enzyme modules, see the following:

- Visualizing Catalytic Potentials of Glycolytic Regulatory Kinases

\(^1\) Procedure outlined in [DZK+16]

\(^2\) Hexokinase based on [YAHP18], [DZK+16], and [MK99]

\(^3\) Glycolysis model based on [YAHP18] and Chapter 10 of [Pal11]

### Thermodynamic Feasibility and Sampling of Metabolite Concentrations¶

This notebook demonstrates how **MASSpy** is used to ensure thermodynamic feasibility in the metabolite concentrations of a model, and how samples of thermodynamically feasible metabolite concentrations are generated for a model.

```
[1]:
```

```
import matplotlib.pyplot as plt

import numpy as np

import mass.test

from mass import MassConfiguration
from mass.thermo import ConcSolver

MASSCONFIGURATION = MassConfiguration()
```

**Note**: Throughout this notebook, the term *thermodynamic feasibility constraint* for a reaction refers to the following:

For a given reaction:

\[\begin{split}\textbf{S}^T \ln{(\textbf{x})} < \ln{(\text{Keq})}\ - \epsilon\ \text{if}\ \text{v}\ > 0\\
\textbf{S}^T \ln{(\textbf{x})} > \ln{(\text{Keq})}\ + \epsilon\ \text{if}\ \text{v}\ < 0\\\end{split}\]

where

- \(\textbf{S}\) refers to the stoichiometry of the reaction
- \(\textbf{x}\) refers to the vector of concentrations for the reaction metabolites
- \(\text{Keq}\) refers to the equilibrium constant of the reaction
- \(\text{v}\) refers to the reaction flux.
- \(\epsilon\) refers to a buffer value for the constraint.

Based on methods outlined in [KummelPH06] and [HDR13]

#### The ConcSolver Object¶

Upon initialization of the `ConcSolver` instance, the model becomes associated with the `ConcSolver` instance. Metabolite concentrations and reaction equilibrium constants are added as variables to the `ConcSolver`.Thermodynamic feasibility constraints, based on the reaction’s flux direction and stoichiometry, are created and also added to the solver. **All solver variables and constraints exist in logarithmic space.**

Metabolite concentrations that should be excluded from the solver can be defined using the `exclude_metabolites` argument (e.g., hydrogen and water). Reactions can also be excluded from the solver using the `exclude_reactions` argument.

Reactions that should exist at equilibrium or equilibrate very quickly should be set using the `equilibrium_reactions` argument. These reactions, such as the hemoglobin binding reactions and the adenylate kinase (ADK1) reaction, typically have a steady state flux value of 0.

```
[2]:
```

```
# Load the JSON version of the textbook model
model = mass.test.create_test_model("textbook")
```

```
[3]:
```

```
conc_solver = ConcSolver(
    model,
    excluded_metabolites=["h_c", "h2o_c"],
    excluded_reactions=None,
    equilibrium_reactions=["HBDPG", "HBO1", "HBO2", "HBO3", "HBO4", "ADK1",
                           "PFK_L"])
# View the model in the ConcSolver
conc_solver.model
```

```
[3]:
```

|  |  |
| --- | --- |
| **Name** | RBC\_PFK |
| **Memory address** | 0x07fd129545a90 |
| **Stoichiometric Matrix** | 68x76 |
| **Matrix Rank** | 63 |
| **Number of metabolites** | 68 |
| **Initial conditions defined** | 68/68 |
| **Number of reactions** | 76 |
| **Number of genes** | 0 |
| **Number of enzyme modules** | 1 |
| **Number of groups** | 16 |
| **Objective expression** | 0 |
| **Compartments** | Cytosol |

The `ConcSolver` also becomes associated with the loaded model.

```
[4]:
```

```
print(model.conc_solver)
```

```
<ConcSolver RBC_PFK at 0x7fd12953ef50>
```

Concentrations and equilibrium constants cannot be negative numbers; therefore, the bounds for each variable are set to ensure such behavior. Because \(\ln(0)\) results in a domain error, the `ConcSolver` has the `zero_value_log_substitute` attribute. The value of the attribute is substituted for 0 to avoid any errors.

For example, if `zero_value_log_substitute=1e-10`, then taking the logarithm of 0 is treated as \(\ln(0) \approx \ln(1\*10^{-10}) = -23.026\).

```
[5]:
```

```
print("Substitute for ln(0): ln({0:.1e})".format(
    conc_solver.zero_value_log_substitute))
```

```
Substitute for ln(0): ln(1.0e-10)
```

Variables can be accessed through the `variables` attribute. The number of variables equals the combined total of the number of included metabolites and the number of included reactions. Specific variables can be accessed using their identifiers as a key.

```
[6]:
```

```
print("Number of included metabolites: {0}".format(len(conc_solver.included_metabolites)),
      "\nNumber of included reactions: {0}".format(len(conc_solver.included_reactions)),
      "\nTotal number of variables: {0}\n".format(len(conc_solver.variables)))

# Access the glucose concentration variable
variable = conc_solver.variables["glc__D_c"]
print("The glucose concentration variable",
      "\n----------------------------------\n",
      variable)
```

```
Number of included metabolites: 66
Number of included reactions: 48
Total number of variables: 114

The glucose concentration variable
----------------------------------
 -23.025850929940457 <= glc__D_c <= inf
```

Constraints can be accessed through the `constraints` attribute. The number of constraints equals the number of included reactions. Just like variables, specific constraints can be accessed using reaction identifiers as a key.

```
[7]:
```

```
print("Total number of constraints: {0}\n".format(len(conc_solver.constraints)))
# Access the hexokinase thermodynamic feasibility constraint
print("Thermodynamic feasibility constraint for HEX1",
      "\n-------------------------------------------\n",
      conc_solver.constraints["HEX1"])
```

```
Total number of constraints: 48

Thermodynamic feasibility constraint for HEX1
-------------------------------------------
 HEX1: -1.0*Keq_HEX1 + 1.0*adp_c - 1.0*atp_c + 1.0*g6p_c - 1.0*glc__D_c <= 0
```

Currently, the constraints do not have an error buffer, which provides some flexibility when solving the underlying mathematical problem of the `ConcSolver`. The `constraint_buffer` attribute can be used to set the *epsilon* value of the constraint. The constraints must be reset in order for the changed buffer value to take effect.

```
[8]:
```

```
conc_solver.constraint_buffer = 1e-7
conc_solver.reset_constraints()
print("Thermodynamic feasibility constraint for HEX1",
      "\n-------------------------------------------\n",
      conc_solver.constraints["HEX1"])
```

```
Thermodynamic feasibility constraint for HEX1
-------------------------------------------
 HEX1: -1.0*Keq_HEX1 + 1.0*adp_c - 1.0*atp_c + 1.0*g6p_c - 1.0*glc__D_c <= -1e-07
```

Upon initialization of the `ConcSolver`, the `ConcSolver.problem_type` is considered generic and no objective is set.

```
[9]:
```

```
print(conc_solver.problem_type)
print(conc_solver.objective)
```

```
generic
Maximize
0
```

The following sections demonstrate different types of problems that can be solved using the `ConcSolver`.

#### Solving for Feasible Concentrations¶

##### Creating the QP problem¶

In order to determine thermodynamically feasible concentrations, a quadratic programming (QP) problem can be set up as follows:

Minimize

\[\ln( (\textbf{x}/\textbf{x}\_0)^{2} )\]

subject to

\[\begin{split}\textbf{S}^T \ln{(\textbf{x})} \lt \ln{(\text{Keq}\_i)}\ - \epsilon\ \text{if}\ \text{v}\_i\ \gt 0 \\
\textbf{S}^T \ln{(\textbf{x})} \gt \ln{(\text{Keq}\_i)}\ + \epsilon\ \text{if}\ \text{v}\_i\ \lt 0 \\
\ln(\text{Keq}\_{i,\ lb}) \leq \ln(\text{Keq}\_i) \leq \ln(\text{Keq}\_{i,\ ub}) \\
\ln(\text{x}\_{j,\ lb}) \leq \ln(\text{x}\_j) \leq \ln(\text{x}\_{j,\ ub}) \\\end{split}\]

where

- \(\textbf{S}\) refers to the stoichiometric matrix.
- \(\textbf{x}\) refers to the vector of metabolite concentrations.
- \(\textbf{x}\_0\) refers to the vector of initial metabolite concentrations.
- \(\text{Keq}\_i\) refers to the equilibrium constant of reaction \(i\).
- \(\text{v}\_i\) refers to the flux for reaction \(i\).
- \(\text{x}\_j\) refers to the concentration of metabolite \(j\).
- \(\epsilon\) refers to a buffer value for the constraint.

Note that solving the QP problem requires a capable solver. Although **MASSpy** does not come with any QP solvers installed, it can interface with an installed version of gurobi through the **optlang** package.

The first step is to set the optimization solver to one that is capable of handling quadratic objectives.

```
[10]:
```

```
conc_solver.solver = conc_solver.choose_solver(qp=True)
print(repr(conc_solver.solver))
```

```
<optlang.gurobi_interface.Model object at 0x7fd129542190>
```

To set up the underlying mathematical problem in the `ConcSolver`, the `setup_feasible_qp_problem()` method can be used. The `fixed_conc_bounds` and `fixed_Keq_bounds` arguments can be used to set the upper and lower bounds of the corresponding variables equal to one other, fixing the variable’s value. In this example, the metabolite concentrations are allowed to change, while the equilibrium constants are fixed at their original value.

```
[11]:
```

```
conc_solver.setup_feasible_qp_problem(
    fixed_Keq_bounds=conc_solver.model.reactions)
```

Using the `setup_feasible_qp_problem()` method also sets the objective for the optimization.

```
[12]:
```

```
print(conc_solver.objective_direction)
conc_solver.objective
```

```
min
```

```
[12]:
```

```
<optlang.gurobi_interface.Objective at 0x7fd12841b110>
```

After using the `setup_feasible_qp_problem()` method, the `ConcSolver` is ready for optimization. The `problem_type` is automatically changed to reflect the current problem setup.

```
[13]:
```

```
print(conc_solver.problem_type)
```

```
feasible_qp
```

##### The ConcSolution Object¶

Once the `ConcSolver` is set up to solve the QP, the next step is to use the `optimize()` method to solve the QP. A successful optimization returns a `ConcSolution` object. All values are transformed back into linear space upon being returned.

```
[14]:
```

```
conc_solution = conc_solver.optimize()
conc_solution
```

```
[14]:
```

***Optimal* solution with objective value 0.000**  

|  | variables | reduced\_costs |
| --- | --- | --- |
| glc\_\_D\_c | 1.296763 | 0.000000 |
| g6p\_c | 0.165018 | 0.000000 |
| f6p\_c | 0.067532 | 0.000000 |
| fdp\_c | 0.016615 | 0.000000 |
| dhap\_c | 0.169711 | 0.000000 |
| ... | ... | ... |
| Keq\_PFK\_L | 0.001100 | -0.103955 |
| Keq\_PFK\_T1 | 10.000000 | -1.097455 |
| Keq\_PFK\_T2 | 10.000000 | -0.408917 |
| Keq\_PFK\_T3 | 10.000000 | 0.000000 |
| Keq\_PFK\_T4 | 10.000000 | 0.000000 |

114 rows × 2 columns

The `ConcSolution` object has several methods for viewing the results of the optimization and returning `pandas` objects containing the numerical solutions.

```
[15]:
```

```
dir(conc_solution)
```

```
[15]:
```

```
['Keq_reduced_costs',
 'Keqs',
 'Keqs_to_frame',
 'concentration_reduced_costs',
 'concentrations',
 'concentrations_to_frame',
 'get_primal_by_id',
 'objective_value',
 'shadow_prices',
 'status',
 'to_frame']
```

```
[16]:
```

```
from mass.visualization import plot_comparison
```

Through visualization features of **MASSPy**, the predicted values can be plotted against the original model values for comparison using the `plot_comparison()` function.

```
[17]:
```

```
# Create figure
fig, ax = plt.subplots(figsize=(6, 6))

# Compare values
plot_comparison(
    x=model, y=conc_solution, compare="concentrations",
    observable=conc_solver.included_metabolites, legend="right outside",
    xlabel="Current Concentrations [mM]",
    ylabel="Predicted Concentrations [mM]",
    plot_function="loglog", xy_line=True, xy_legend="best");
```

```
[17]:
```

```
<matplotlib.axes._subplots.AxesSubplot at 0x7fd129681310>
```

The model in the `ConcSolver` can be updated with the results contained within the `ConcSolution` using the `update_model_with_solution()` method. Setting `inplace=True` updates the current model in the `ConcSolver`, while setting `inplace=False` replaces the model in the `ConcSolver` with an updated copy the model without modifying the original. Setting `inplace=False` also removes the previous model’s association with the `ConcSolver`.

```
[18]:
```

```
conc_solver.update_model_with_solution(
    conc_solution, concentrations=True, Keqs=False, inplace=False)
print("Same model object? {0}".format(conc_solver.model == model))
print(model.conc_solver)
```

```
Same model object? False
None
```

#### Concentration Sampling¶

##### Basic usage¶

The easiest method of sampling concentrations is to use the `sample_concentrations()` function in the `conc_sampling` submodule.

```
[19]:
```

```
from mass.thermo.conc_sampling import sample_concentrations
```

To set up the `ConcSolver` for sampling, the `setup_sampling_problem` method is used. The `conc_percent_deviation` and `Keq_percent_deviation` arguments can be used to set the variable bounds for sampling. For this example, the defined concentrations are allowed to deviate up to %75 from their baseline value, while the defined equilibrium constants remain fixed at their current values.

```
[20]:
```

```
conc_solver.setup_sampling_problem(
    conc_percent_deviation=0.75,
    Keq_percent_deviation=0)
print(conc_solver.problem_type)
```

```
sampling
```

Using the `sample_concentrations()` function requires at least two arguments: a `ConcSolver` that has been set up for sampling, and the number of samples to generate.

```
[21]:
```

```
samples = sample_concentrations(conc_solver, n=20)
samples.head()
```

```
[21]:
```

|  | glc\_\_D\_c | g6p\_c | f6p\_c | fdp\_c | dhap\_c | g3p\_c | \_13dpg\_c | \_3pg\_c | \_2pg\_c | pep\_c | ... | pfk\_R3\_A\_c | pfk\_R3\_AF\_c | pfk\_R4\_c | pfk\_R4\_A\_c | pfk\_R4\_AF\_c | pfk\_T0\_c | pfk\_T1\_c | pfk\_T2\_c | pfk\_T3\_c | pfk\_T4\_c |
| --- | --- | --- | --- | --- | --- | --- | --- | --- | --- | --- | --- | --- | --- | --- | --- | --- | --- | --- | --- | --- | --- |
| 0 | 0.573084 | 0.072931 | 0.029583 | 0.005041 | 0.072693 | 0.003611 | 0.000257 | 0.039993 | 0.005171 | 0.007705 | ... | 0.000005 | 7.853042e-07 | 6.694715e-07 | 0.000002 | 3.965789e-07 | 9.650193e-11 | 1.505694e-09 | 2.101538e-08 | 1.328247e-07 | 3.876323e-07 |
| 1 | 0.856706 | 0.150834 | 0.060231 | 0.015956 | 0.159873 | 0.007932 | 0.000379 | 0.127215 | 0.018441 | 0.029765 | ... | 0.000005 | 6.515172e-07 | 5.497481e-07 | 0.000002 | 3.517310e-07 | 5.981162e-11 | 9.459993e-10 | 1.811540e-08 | 1.543798e-07 | 3.845968e-07 |
| 2 | 0.998544 | 0.195311 | 0.063848 | 0.017021 | 0.163829 | 0.005826 | 0.000346 | 0.090935 | 0.011576 | 0.016030 | ... | 0.000003 | 4.135990e-07 | 3.306412e-07 | 0.000002 | 3.379217e-07 | 1.277482e-10 | 1.890705e-09 | 2.948550e-08 | 2.131460e-07 | 4.545959e-07 |
| 3 | 0.914117 | 0.167755 | 0.058189 | 0.017858 | 0.153780 | 0.005535 | 0.000390 | 0.067050 | 0.009733 | 0.014720 | ... | 0.000004 | 6.057890e-07 | 3.992094e-07 | 0.000003 | 3.881900e-07 | 1.254980e-10 | 1.799046e-09 | 2.254959e-08 | 2.210896e-07 | 4.464778e-07 |
| 4 | 1.342935 | 0.239222 | 0.075899 | 0.019497 | 0.156620 | 0.006916 | 0.000357 | 0.122204 | 0.017919 | 0.027510 | ... | 0.000005 | 7.079300e-07 | 4.701749e-07 | 0.000003 | 4.453493e-07 | 7.721228e-11 | 1.881876e-09 | 2.896366e-08 | 1.929782e-07 | 4.755604e-07 |

5 rows × 66 columns

By default `sample_concentrations` uses the `optgp` method [MHM14], as it is suited for larger models and can run in parallel. The number of processes can be changed by using the `processes` argument.

```
[22]:
```

```
print("One process:")
%time samples = sample_concentrations(conc_solver, n=1000, processes=1)
print("\nTwo processes:")
%time samples = sample_concentrations(conc_solver, n=1000, processes=2)
```

```
One process:
CPU times: user 12.7 s, sys: 62 ms, total: 12.8 s
Wall time: 12.7 s

Two processes:
CPU times: user 506 ms, sys: 35.4 ms, total: 541 ms
Wall time: 7.08 s
```

Alternatively, the Artificial Centering Hit-and-Run for sampling [KS98] can be utilized by setting the method to `achr`. The `achr` method does not support parallel execution, but it has good convergence and is almost Markovian.

```
[23]:
```

```
samples = sample_concentrations(conc_solver, n=100, method="achr")
```

In general, setting up the sampler is expensive since initial search directions are generated by solving many linear programming problems. Thus, it is recommended to generate as many samples as possible in one go. However, generating large numbers of samples might require finer control over the sampling procedure, as described in the following section.

##### Advance usage¶

###### Sampler objects¶

The concentration sampling process can be controlled on a lower level by using the sampler classes directly, found in the `conc_sampling` submodule.

```
[24]:
```

```
from mass.thermo.conc_sampling import ConcACHRSampler, ConcOptGPSampler
```

Both concentration sampler classes have standardized interfaces and take some additional arguments.

For example, one such argument is the thinning factor, where “thinning” means only recording samples every `x` iterations where `x` is the thinning factor. Higher thinning factors mean less correlated samples but also larger computation times.

By default, the samplers use a thinning factor of 100, which creates roughly uncorrelated samples. Increasing the thinning factor leads to better mixing of samples, while lowering the thinning factor leads to more correlated samples. For example, it may be desirable to set a thinning factor of 1 to obtain all iterates when studying convergence for a model.

Samplers can be seeded so that they produce the same results each time they are run.

```
[25]:
```

```
conc_achr = ConcACHRSampler(conc_solver, thinning=1, seed=5)
samples = conc_achr.sample(10, concs=True)
# Display only the first 5 samples
samples.head(5)
```

```
[25]:
```

|  | glc\_\_D\_c | g6p\_c | f6p\_c | fdp\_c | dhap\_c | g3p\_c | \_13dpg\_c | \_3pg\_c | \_2pg\_c | pep\_c | ... | pfk\_R3\_A\_c | pfk\_R3\_AF\_c | pfk\_R4\_c | pfk\_R4\_A\_c | pfk\_R4\_AF\_c | pfk\_T0\_c | pfk\_T1\_c | pfk\_T2\_c | pfk\_T3\_c | pfk\_T4\_c |
| --- | --- | --- | --- | --- | --- | --- | --- | --- | --- | --- | --- | --- | --- | --- | --- | --- | --- | --- | --- | --- | --- |
| 0 | 1.592336 | 0.197568 | 0.078832 | 0.018910 | 0.142081 | 0.007510 | 0.000472 | 0.090709 | 0.013006 | 0.021494 | ... | 0.000003 | 4.536657e-07 | 3.693232e-07 | 0.000001 | 2.228541e-07 | 3.305772e-11 | 6.624519e-10 | 1.327504e-08 | 1.311206e-07 | 3.905462e-07 |
| 1 | 2.248172 | 0.285898 | 0.116922 | 0.004324 | 0.043806 | 0.002030 | 0.000364 | 0.022330 | 0.003282 | 0.005558 | ... | 0.000002 | 3.634455e-07 | 3.032560e-07 | 0.000001 | 1.922307e-07 | 2.714412e-11 | 5.768461e-10 | 1.225869e-08 | 1.236426e-07 | 3.810419e-07 |
| 2 | 2.263896 | 0.288041 | 0.117857 | 0.004522 | 0.045433 | 0.002117 | 0.000370 | 0.023388 | 0.003439 | 0.005827 | ... | 0.000002 | 3.618207e-07 | 3.020507e-07 | 0.000001 | 1.916574e-07 | 2.703623e-11 | 5.752355e-10 | 1.223898e-08 | 1.234960e-07 | 3.808523e-07 |
| 3 | 2.258775 | 0.287343 | 0.117552 | 0.004406 | 0.044485 | 0.002067 | 0.000366 | 0.022777 | 0.003348 | 0.005673 | ... | 0.000002 | 3.623478e-07 | 3.024418e-07 | 0.000001 | 1.918435e-07 | 2.707124e-11 | 5.757583e-10 | 1.224538e-08 | 1.235436e-07 | 3.809139e-07 |
| 4 | 2.267224 | 0.288494 | 0.109154 | 0.004331 | 0.043882 | 0.002036 | 0.000365 | 0.022409 | 0.003295 | 0.005585 | ... | 0.000002 | 3.614792e-07 | 3.017973e-07 | 0.000001 | 1.915368e-07 | 2.701355e-11 | 5.748966e-10 | 1.223483e-08 | 1.234651e-07 | 3.808123e-07 |

5 rows × 66 columns

The `sample()` method also comes with the `concs` argument that controls the sample output. Setting `concs=True` returns only concentration variables, while setting `concs=False` returns the equilibrium constant variables and any additional variables.

```
[26]:
```

```
samples = conc_achr.sample(10, concs=False)
print(samples.columns)
```

```
Index(['ade_c', 'adn_c', 'imp_c', 'prpp_c', 'nh3_c', 'glc__D_c', 'g6p_c',
       'adp_c', 'atp_c', 'Keq_HEX1',
       ...
       'pfk_T0_c', 'Keq_PFK_L', 'pfk_T1_c', 'Keq_PFK_T1', 'pfk_T2_c',
       'Keq_PFK_T2', 'pfk_T3_c', 'Keq_PFK_T3', 'pfk_T4_c', 'Keq_PFK_T4'],
      dtype='object', length=114)
```

The `ConcOptGPSampler` has an additional `processes` argument that specifies how many processes are used to create parallel sampling chains. The number of processes should be in the order of available CPU cores for maximum efficiency. As noted before, the class initialization can take up to a few minutes due to generation of initial search directions. On the other hand, sampling is quicker.

```
[27]:
```

```
conc_optgp = ConcOptGPSampler(conc_solver, processes=4, seed=5)
```

For the `ConcOptGPSampler`, the number of samples should be a multiple of the number of processes. Otherwise, the number is increased automatically to the nearest multiple.

```
[28]:
```

```
samples = conc_optgp.sample(10)
print("Number of samples generated: {0}".format(len(samples)))
```

```
Number of samples generated: 12
```

###### Batch sampling¶

Sampler objects are made for generating billions of samples, however using the sampling functions might quickly fill up the computer RAM when working with genome-scale models.

In this scenario, the batch method of the sampler objects might be useful. The `batch` method takes two arguments: the number of samples in each batch and the number of batches.

Suppose the concentration of ATP, ADP and AMP are unknown. The batch sampler could be used to generate 10 batches of feasible concentrations with 100 samples each. The samples could be averaged to get the mean metabolite concentrations per batch. Finally, the mean metabolite concentrations and standard deviation could be calculated.

```
[29]:
```

```
# Remove current initial conditions for example
conc_solver.model.metabolites.atp_c.ic = None
conc_solver.model.metabolites.adp_c.ic = None
conc_solver.model.metabolites.amp_c.ic = None
# Set up concentration sampling problem
conc_solver.setup_sampling_problem(
    conc_percent_deviation=0.5,
    Keq_percent_deviation=0)

# Get batch samples
conc_optgp = ConcOptGPSampler(conc_solver, processes=1, seed=5)
batch_samples = [sample for sample in conc_optgp.batch(100, 10)]

# Determine average metabolite concentrations per batch
for met in ["atp_c", "adp_c", "amp_c"]:
    met = conc_solver.model.metabolites.get_by_id(met)
    per_batch_axp_ave = [
        np.mean(sample[met.id])
        for sample in batch_samples]
    print("Ave. {2} concentration: {0:.5f} +- {1:.5f}".format(
        np.mean(per_batch_axp_ave), np.std(per_batch_axp_ave), met.id))
    met.ic = np.mean(per_batch_axp_ave)
```

```
Ave. atp_c concentration: 2.17516 +- 0.10551
Ave. adp_c concentration: 0.24789 +- 0.01742
Ave. amp_c concentration: 0.12479 +- 0.00738
```

### Ensemble Modeling¶

This notebook demonstrates how **MASSpy** can be used to generate an ensemble of models.

```
[1]:
```

```
# Disable gurobi logging output for this notebook.
try:
    import gurobipy
    gurobipy.setParam("OutputFlag", 0)
except ImportError:
    pass

import logging
logging.getLogger("").setLevel("CRITICAL")

# Configure roadrunner to allow for more output rows
import roadrunner
roadrunner.Config.setValue(
    roadrunner.Config.MAX_OUTPUT_ROWS, 1e6)

from mass import MassConfiguration, Simulation
from mass.test import create_test_model

mass_config = MassConfiguration()
mass_config.decimal_precision = 12 # Round 12 places after decimal

# Load the model
reference_model = create_test_model("Glycolysis")
```

#### Generating Data for Ensembles¶

In addition to loading external sources of data for use (e.g., loading excel sheets), sampling can be used to get valid data for the generation of ensembles. As an example, a small set of samples are generated for use in this notebook.

Utilizing COBRApy flux sampling, the following flux samples are used in generating the ensemble of models. All steady state flux values are set to allow to deviation by up to 80% of their defined baseline values.

```
[2]:
```

```
from cobra.sampling import sample
```

```
[3]:
```

```
flux_percent_deviation = 0.8
for reaction in reference_model.reactions:
    flux = reaction.steady_state_flux
    reaction.bounds = sorted([
        round(flux * (1 - flux_percent_deviation),
              mass_config.decimal_precision),
        round(flux * (1 + flux_percent_deviation),
              mass_config.decimal_precision)])

flux_samples = sample(reference_model, n=10, seed=25)
```

Utilizing MASSpy concentration sampling, the following concentration samples are used in generating the ensemble of models. All concentration values are set to allow deviation by up to 80% of their defined baseline values.

```
[4]:
```

```
from mass.thermo import ConcSolver, sample_concentrations
```

```
[5]:
```

```
conc_solver = ConcSolver(
    reference_model,
    excluded_metabolites=["h_c", "h2o_c"],
    equilibrium_reactions=["ADK1"],
    constraint_buffer=1e-7)

conc_solver.setup_sampling_problem(
    conc_percent_deviation=0.8,
    Keq_percent_deviation=0)

conc_samples = sample_concentrations(conc_solver, n=10, seed=25)
```

Because there are 10 flux data sets and 10 concentration data sets being passed to the function, there are \(10 \* 10 = 100\) models generated in total.

#### Creating an Ensemble¶

```
[6]:
```

```
from mass.simulation import ensemble, generate_ensemble_of_models
```

##### Generating new models¶

The `ensemble` submodule has two functions for creating models from `pandas.DataFrame` objects:

- The `create_models_from_flux_data()` function creates an ensemble of models from a `DataFrame` containing flux data, where rows correspond to samples and columns correspond to reaction identifiers.
- The `create_models_from_concentration_data()` function creates an ensemble of models from a `DataFrame` containing concentration data, where rows correspond to samples and columns correspond to metabolite identifiers.

The functions can be used separately or together to generate models. In this example, an ensemble of 100 models is generated by utilizing both mode; generation methods.

First, the 10 flux samples are used to generate 10 models with varying flux states from a single reference `MassModel`.

```
[7]:
```

```
flux_models = ensemble.create_models_from_flux_data(
    reference_model, data=flux_samples)
len(flux_models)
```

```
[7]:
```

```
10
```

The `list` of models are passed to the `create_models_from_concentration_data()` function along with the concentration samples to create models with varying concentration states. By treating each of the 10 models with varying flux states as a reference model in addition to providing 10 concentration samples, 100 total models are generated.

```
[8]:
```

```
conc_models = []
for ref_model in flux_models:
    conc_models += ensemble.create_models_from_concentration_data(
        ref_model, data=conc_samples)
len(conc_models)
```

```
[8]:
```

```
100
```

Generating models does not always ensure that the models are thermodynamically feasible. The `ensure_positive_percs()` function is used to calculate PERCs (pseudo-elementary rate constants) for all reactions provided to the `reactions` argument. Those that produce all positive PERCs are separated from those that produce at least one negative PERC, and two lists that contain the seperated models are returned.

If the `update_values` argument is set to `True`, PERC values are updated for models that produce all positive PERCs.

```
[9]:
```

```
# Exclude boundary reactions from PERC calculations for the example
reactions_to_check_percs = [
    r.id for r in reference_model.reactions
    if r not in reference_model.boundary]

positive, negative = ensemble.ensure_positive_percs(
    models=conc_models, reactions=reactions_to_check_percs,
    update_values=True)

print("Models with positive PERCs: {0}".format(len(positive)))
print("Models with negative PERCs: {0}".format(len(negative)))
```

```
Models with positive PERCs: 100
Models with negative PERCs: 0
```

The `ensure_steady_state()` function is used to ensure that models are able to reach a steady state. If `update_values=True`, models that reach a steady state are updated with the new steady state values.

```
[10]:
```

```
feasible, infeasible = ensemble.ensure_steady_state(
    models=positive, strategy="simulate",
    update_values=True, decimal_precision=True)

print("Reached steady state: {0}".format(len(feasible)))
print("No steady state reached: {0}".format(len(infeasible)))
```

```
mass/simulation/simulation.py:828 UserWarning: Unable to find a steady state for one or more models. Check the log for more details.
```

```
Reached steady state: 90
No steady state reached: 10
```

The `perturbations` argument of the `ensure_steady_state()` method is used to check that models are able to reach a steady state with a given perturbation.

```
[11]:
```

```
feasible, infeasible = ensemble.ensure_steady_state(
    models=feasible, strategy="simulate",
    perturbations={"kf_ATPM": "kf_ATPM * 1.5"},
    update_values=False, decimal_precision=True)

print("Reached steady state: {0}".format(len(feasible)))
print("No steady state reached: {0}".format(len(infeasible)))
```

```
mass/simulation/simulation.py:828 UserWarning: Unable to find a steady state for one or more models. Check the log for more details.
```

```
Reached steady state: 88
No steady state reached: 2
```

All models returned as “feasible” are considered to be thermodynamically feasible and able to reach a steady state, even with the given disturbance.

#### Simulating an Ensemble of Models¶

Once an ensemble of models is generated, the `Simulation` object can be used to simulate the ensemble of models.

```
[12]:
```

```
sim = Simulation(reference_model, verbose=True)
```

```
Successfully loaded MassModel 'Glycolysis' into RoadRunner.
```

Three criteria must be met to add additional models to an existing `Simulation` object:

1. The model must have ODEs equivalent to those of the `Simulation.reference_model`.
2. All models must have unique identifiers.
3. Numerical values that are necessary for simulation must already be defined for a model.

If the criteria are met, additional models can be loaded into the `Simulation` using the `add_models()` method.

```
[13]:
```

```
sim.add_models(models=feasible)
print("Number of models added: {0}".format(len(feasible)))
print("Number of models total: {0}".format(len(sim.models)))
```

```
Number of models added: 88
Number of models total: 89
```

The `simulate()` method is used to simulate multiple models. By default, all loaded models are simulated, including the `reference_model`.

```
[14]:
```

```
conc_sol_list, flux_sol_list = sim.simulate(time=(0, 1000))
print("ConcSols returned: {0}".format(len(conc_sol_list)))
print("FluxSols returned: {0}".format(len(flux_sol_list)))
```

```
ConcSols returned: 89
FluxSols returned: 89
```

To simulate a subset of the models, a list of models or their identifiers can be provided to the `simulate()` method. For example, to simulate the subset of models with identical concentration states but different flux states:

```
[15]:
```

```
model_subset = [model for model in sim.models if model.endswith("_C0")]
conc_sol_list, flux_sol_list = sim.simulate(
    models=model_subset, time=(0, 1000))
print("ConcSols returned: {0}".format(len(conc_sol_list)))
print("FluxSols returned: {0}".format(len(flux_sol_list)))
```

```
ConcSols returned: 7
FluxSols returned: 7
```

Similar to the `simulate()` method, the `find_steady_state()` method can be used to determine a steady state for each model in an ensemble or subset of models.

```
[16]:
```

```
conc_sol_list, flux_sol_list = sim.find_steady_state(
    models=model_subset, strategy="simulate")
print("ConcSols returned: {0}".format(len(conc_sol_list)))
print("FluxSols returned: {0}".format(len(flux_sol_list)))
```

```
ConcSols returned: 7
FluxSols returned: 7
```

If an exception occurs for a model during steady state determination or simulation (e.g., no steady state exists), the MassSolution objects that correspond to the failed model will return empty.

```
[17]:
```

```
# Create a simulation with the reference model and an infeasible one
infeasible_sim = Simulation(reference_model)
infeasible_sim.add_models(infeasible[0])

conc_sol_list, flux_sol_list = infeasible_sim.find_steady_state(
    strategy="simulate", perturbations={"kf_ATPM": "kf_ATPM * 1.5"})

print("ConcSols returned: {0}".format(len(conc_sol_list)))
print("FluxSols returned: {0}".format(len(flux_sol_list)))

for model, sol in zip(sim.models, conc_sol_list):
    print("Solutions for {0}: {1}".format(str(model), bool(sol)))
```

```
ConcSols returned: 2
FluxSols returned: 2
Solutions for Glycolysis: True
Solutions for Glycolysis_F0_C0: False
```

```
mass/simulation/simulation.py:828 UserWarning: Unable to find a steady state for one or more models. Check the log for more details.
```

##### Visualizing Ensemble Results¶

Through visualization features of **MASSPy**, the results of simulating the ensemble can be visualized using the `plot_ensemble_time_profile()` and `plot_ensemble_phase_portrait()` functions.

```
[18]:
```

```
import matplotlib as mpl
import matplotlib.pyplot as plt

import numpy as np

from mass.visualization import (
    plot_ensemble_phase_portrait, plot_ensemble_time_profile)
```

A list of `MassSolution` objects is required to use an ensemble visualization function. The output of `simulate()` method for an ensemble of models can be placed into the functions directly.

```
[19]:
```

```
sim = Simulation(reference_model, verbose=True)
sim.add_models(models=feasible)

conc_sol_list, flux_sol_list = sim.simulate(
    models=feasible, time=(0, 1000),
    perturbations={"kf_ATPM": "kf_ATPM * 1.5"},
    decimal_precision=True)
```

```
Successfully loaded MassModel 'Glycolysis' into RoadRunner.
```

The `plot_ensemble_time_profile()` function works in a manner similar to the `plot_time_profile()` function described in Time Profiles. The minimal input required is a list of `MassSolution` objects and an iterable that contains strings or objects with identifiers that correspond to keys of the `MassSolution`. The plotted solution lines represent the average (mean) solution.

```
[20]:
```

```
plot_ensemble_time_profile(
    conc_sol_list, observable=reference_model.metabolites,
    interval_type=None,
    legend="right outside", plot_function="semilogx",
    xlabel="Time (hrs)", ylabel="Concentrations (mM)",
    title="Mean Concentrations (N={0})".format(len(conc_sol_list)))
```

```
[20]:
```

```
<matplotlib.axes._subplots.AxesSubplot at 0x7fad769df110>
```

Because the plotted lines are the mean solution values over time for the ensemble, there is some uncertainty associated with the solutions. The `interval_type` argument can be specified to plot the results with a confidence interval. For example, to plot the mean PYK flux with a 95% confidence interval:

```
[21]:
```

```
plot_ensemble_time_profile(
    flux_sol_list, observable=["PYK"], legend="best",
    interval_type="CI=95", # Shading for 95% confidence
    plot_function="semilogx", xlabel="Time (hrs)",
    ylabel="Flux (mM/hr)",
    title="Mean PYK Flux (N={0})".format(len(flux_sol_list)),
    color="red", mean_line_alpha=1,  # Default opacity of mean line
    interval_fill_alpha=0.5,   # Default opacity for interval shading
    interval_border_alpha=0.5)  # Default opacity of border lines
```

```
[21]:
```

```
<matplotlib.axes._subplots.AxesSubplot at 0x7fad74fe0090>
```

Setting `interval_type="range"` causes shading between the minimum and maximum solution values. The `mean_line_alpha`, `interval_fill_alpha`, and `interval_border_alpha` kwargs are used to control the opacity of the mean solution, interval borders, and interval shading, respectively.

```
[22]:
```

```
plot_ensemble_time_profile(
    flux_sol_list, observable=["PYK"],
    interval_type="range", # Shading from min to max value
    legend="best", plot_function="semilogx",
    xlabel="Time (hrs)", ylabel="Flux (mM/hr)",
    title="Mean PYK Flux (N={0})".format(len(flux_sol_list)),
    color="red", mean_line_alpha=0.6,  # For opacity of mean line
    interval_fill_alpha=0.3, # For lighter interval shading
    interval_border_alpha=1  # For darker border lines
)
```

```
[22]:
```

```
<matplotlib.axes._subplots.AxesSubplot at 0x7fad763df710>
```

Relative deviations are plotted using the `deviation` kwarg. The `deviation_zero_centered` kwarg is used to shift the results to deviate from 0., and the `deviation_normalization` kwarg is used to normalize each solution.

```
[23]:
```

```
fig, (ax1, ax2) = plt.subplots(nrows=2, ncols=1, figsize=(12, 8))

# Plot all relative flux deviations
plot_ensemble_time_profile(
    flux_sol_list, observable=reference_model.reactions,
    interval_type=None, ax=ax1, legend="right outside",
    plot_function="semilogx", xlabel="Time (hrs)",
    ylabel=("Relative Deviation\n" +\
            r"($\frac{v - v_{0}}{v_{max} - v_{min}}$)"),
    title="Mean Flux Deviations (N={0})".format(len(flux_sol_list)),
    deviation=True,
    deviation_zero_centered=True,  # Center around 0
    deviation_normalization="range")  # Normalized by value range

# Plot PYK relative flux deviations
plot_ensemble_time_profile(
    flux_sol_list, observable=["PYK"],
    interval_type="CI=99",  # 99% confidence interval
    ax=ax2, legend="lower right",
    plot_function="semilogx", xlabel="Time (hrs)",
    ylabel=("Relative Deviation\n" +\
            r"($\frac{v - v_{0}}{v_{0}}$)"),
    title="Average PYK Flux Deviation (N={0})".format(
        len(flux_sol_list)),
    color="red", deviation=True,
    deviation_zero_centered=True,  # Center around 0
    deviation_normalization="initial value")  # Normalized by init. value

fig.tight_layout()
```

The `plot_ensemble_phase_portrait()` function works in a manner similar to the `plot_phase_portrait()` function described in Phase Portraits. The `plot_ensemble_phase_portrait()` function plots the mean solutions for the two ensemble simulation results against each other.

```
[24]:
```

```
fig, ax = plt.subplots(nrows=1, ncols=1, figsize=(5, 5))

# Createt time points and colors for the time points
time_points = [0, 1e-1, 1e0, 1e1, 1e2]
time_point_colors = [
    mpl.colors.to_hex(c)
    for c in mpl.cm.Blues(np.linspace(0.3, 1, len(time_points)))]

# Plot the phase portrait
plot_ensemble_phase_portrait(
    flux_sol_list, x="ATPM", y="GAPD", ax=ax, legend="upper right",
    xlim=(1.3, 3.5), ylim=(1.3, 3.5),
    title="ATPM vs. GAPD",
    color="orange", linestyle="-",
    annotate_time_points=time_points,
    annotate_time_points_color=time_point_colors,
    annotate_time_points_legend="right outside");
```

```
[24]:
```

```
<matplotlib.axes._subplots.AxesSubplot at 0x7fad76b303d0>
```

#### Fast Ensemble Creation¶

Another function utilized for ensemble creation is the `generate_ensemble_of_models()` function. The `generate_ensemble_of_models()` function is a way to streamline the generation of models for an ensemble with increased performance gains at the cost of user control and increased overhead for setup. Consequently, the `generate_ensemble_of_models()` function may be a more desirable function to use when generating a large number of models.

The `generate_ensemble_of_models()` function requires a single `MassModel` as a reference model, and sample data as a `pandas.DataFrame` for the `flux_data` and `conc_data` arguments.

- For `flux_data` columns are reaction identifiers, and rows are samples of steady state fluxes.
- For `conc_data` columns are metabolite identifiers, and rows are samples of concentrations (initial conditions).

At least one of the above arguments must be provided for the function to work. After generating the models, a list that contains the model objects is returned.

```
[25]:
```

```
# Generate the ensemble
models = generate_ensemble_of_models(
    reference_model=reference_model,
    flux_data=flux_samples,
    conc_data=conc_samples)
```

```
Total models generated: 100
```

To ensure that the PERCs for certain reactions are positive, a `list` of reactions to check can be provided to the `ensure_positive_percs` argument.

```
[26]:
```

```
# Exclude boundary reactions from PERC calculations for the example
reactions_to_check_percs = [
    r.id for r in reference_model.reactions
    if r not in reference_model.boundary]

# Generate the ensemble
ensemble = generate_ensemble_of_models(
    reference_model=reference_model,
    flux_data=flux_samples,
    conc_data=conc_samples,
    ensure_positive_percs=reactions_to_check_percs)
```

```
Total models generated: 100
Feasible: 100
Infeasible, negative PERCs: 0
```

To ensure that all models can reach a steady state with their new values, a strategy for finding the steady state can be provided to the `strategy` argument.

```
[27]:
```

```
# Generate the ensemble
models = generate_ensemble_of_models(
    reference_model=reference_model,
    flux_data=flux_samples,
    conc_data=conc_samples,
    ensure_positive_percs=reactions_to_check_percs,
    strategy="simulate",
    decimal_precision=True)
```

```
mass/simulation/simulation.py:828 UserWarning: Unable to find a steady state for one or more models. Check the log for more details.
```

```
Total models generated: 100
Feasible: 90
Infeasible, negative PERCs: 0
Infeasible, no steady state found: 10
```

To ensure that all models can reach a steady state with their new values after a given perturbation, in addition to passing a value to the `strategy` argument, one or more perturbations can be given to the `perturbations` argument. The `perturbations` argument takes a `list` of dictionaries, each containing perturbations formatted as described in Dynamic Simulation.

If it is desirable to return the models that were not deemed ‘feasible’, the `return_infeasible` kwarg can be set to `True` to return a second list that contains only models deemed ‘infeasible’.

```
[28]:
```

```
feasible, infeasible = generate_ensemble_of_models(
    reference_model=reference_model,
    flux_data=flux_samples,
    conc_data=conc_samples,
    ensure_positive_percs=reactions_to_check_percs,
    strategy="simulate",
    perturbations=[
        {"kf_ATPM": "kf_ATPM * 1.5"},
        {"kf_ATPM": "kf_ATPM * 0.85"}],
    return_infeasible=True,
    decimal_precision=True)
```

```
mass/simulation/simulation.py:828 UserWarning: Unable to find a steady state for one or more models. Check the log for more details.
mass/simulation/simulation.py:828 UserWarning: Unable to find a steady state for one or more models. Check the log for more details.
```

```
Total models generated: 100
Feasible: 88
Infeasible, negative PERCs: 0
Infeasible, no steady state found: 10
Infeasible, no steady state with pertubration 1: 2
Infeasible, no steady state with pertubration 2: 0
```

Note that perturbations are not applied all at once; each `dict` provided corresponds to a new attempt to find a steady state. For example, two dictionaries passed to the `perturbations` argument indicate that three steady state determinations are performed, once for the model without any perturbations and once for each `dict` provided.

Generally it is recommended to utilize the functions in the `ensemble` submodule to generate small ensembles while experimenting with various settings, and then to utilize the `generate_ensemble_of_models` function to generate the larger ensemble.

### Network Visualization¶

This notebook demonstrates how to view **MASSpy** models on network maps using the Escher visualization tool [KDragerE+15].

The **Escher** package must already be installed into the environment. To install **Escher**:

```
pip install escher
```

#### Viewing Models with Escher¶

The **MASSpy** package also comes with some maps for testing purposes.

```
[1]:
```

```
from os.path import join

import numpy as np

import mass
import mass.test

# Load the glycolysis and hemoglobin models, then merge them
glycolysis = mass.test.create_test_model("Glycolysis")
hemoglobin = mass.test.create_test_model("Hemoglobin")
model = glycolysis.merge(hemoglobin, inplace=False)

# Set the path to the map file
map_filepath = join(mass.test.MAPS_DIR, "RBC.glycolysis.map.json")

# To view the list of available maps, remove the semicolon
mass.test.view_test_maps();
```

```
[1]:
```

```
['RBC.glycolysis.map.json',
 'multicompartment_map.json',
 'phosphate_trafficking_map.json',
 'sb2_RBC_map.json',
 'sb2_amp_salvage_network_map.json',
 'sb2_glycolysis_map.json',
 'sb2_pentose_phosphate_pathway_map.json',
 'simple_toy_map.json']
```

The primary object for viewing **Escher** maps is the `escher.Builder`, a Jupyter widget that can be viewed in a Jupyter notebook.

```
[2]:
```

```
import escher
from escher import Builder

# Turns off the warning message when leaving or refreshing this page.
# The default setting is False to help avoid losing work.
escher.rc['never_ask_before_quit'] = True
```

To load an existing map, the path to the JSON file of the **Escher** map is provided to the `map_json` argument of the `Builder`. The `MassModel` can be loaded using the `model` argument.

```
[3]:
```

```
escher_builder = Builder(
    model=model,
    map_json=map_filepath)

escher_builder
```

#### Mapping Data onto Escher¶

##### Viewing Reaction Data¶

Reaction data can be displayed on the **Escher** map using a dictionary that contains reaction identifiers, and values to map onto reaction arrows. The `dict` can be provided to the `reaction_data` argument upon initialization of the builder.

For example, to display the steady state fluxes on the map:

```
[4]:
```

```
initial_flux_data = {
    reaction.id: flux
    for reaction, flux in model.steady_state_fluxes.items()}

# New instance to prevent modifications to the existing maps
escher_builder = Builder(
    model=model,
    map_json=map_filepath,
    reaction_data=initial_flux_data)

# Display map in notebook
escher_builder
```

The color and size of the data scale can be altered by providing a tuple of at least two dictionaries. Each dictionary is considered a “stop” that defines the color and size at or near that particular value in the data set. The `type` key defines the type for the stop, the `color` key defines the color of the arrow, and the `size` key defines the thickness of the arrow.

```
[5]:
```

```
# New instance to prevent modifications to the existing maps
escher_builder = Builder(
    model=model,
    map_json=map_filepath,
    reaction_data=initial_flux_data,
    reaction_scale=(
        {"type": 'min', "color": 'green', "size": 5 },
        {"type": 'value', "value": 1.12, "color": 'purple', "size": 10},
        {"type": 'max', "color": 'blue', "size": 15 }),
)

# Display map in notebook
escher_builder
```

##### Viewing Metabolite Data¶

Metabolite data also can be displayed on an **Escher** map by using a dictionary containing metabolite identifiers, and values to map onto metabolite nodes. In addition to setting the attributes to apply upon initializing the builder, the attributes also can be set for a map after initialization.

For example, to display metabolite concentrations on the map:

```
[6]:
```

```
initial_conc_data = {
    metabolite.id: round(conc, 8)
    for metabolite, conc in model.initial_conditions.items()}

# New instance to prevent modifications to the existing maps
escher_builder = Builder(
    model=model,
    map_json=map_filepath,
    metabolite_data=initial_conc_data)

# Display map in notebook
escher_builder
```

The secondary metabolites can be removed by setting `hide_secondary_metabolites` as `True` to provide a cleaner visualization of the primary metabolites in the network.

```
[7]:
```

```
escher_builder.hide_secondary_metabolites = True
```

Note that changes made affect the already displayed map. Here, a preset scale is applied to the metabolite concentrations.

```
[8]:
```

```
escher_builder.metabolite_scale_preset = "RdYlBu"
```

##### Visualizing SBML models with Escher in Python¶

Suppose that we would like to visualize our SBML model on a network map as follows: 1. We would like to create this map with the **Escher** web-based API. 2. We would like to view the model on the network map within in a Jupyter notebook using the **Escher** Python-based API. 3. We would like to display the value of forward rate constants for each reaction on the network map.

The JSON format is the preferred format for **Escher** to load models onto network maps (read more here). Therefore, we must convert models between SBML and JSON formats to achieve our goal.

**Note:** The models and maps used in the following example are also available in the testing data.

```
[9]:
```

```
import mass.io
```

Fortunately, the mass.io submodule is capable of exporting such models.

First the SBML model is loaded using the `mass.io.sbml` submodule. The model is then exported to a JSON format using the `mass.io.json` submodule for use in the Escher web-based API.

```
[10]:
```

```
# Define path to SBML model
path_to_sbml_model = join(mass.test.MODELS_DIR, "Simple_Toy.xml")

# Load SBML model
model = mass.io.sbml.read_sbml_model(path_to_sbml_model)

# Export as JSON
path_to_json_model = "./Simple_Toy.json"
mass.io.json.save_json_model(model, filename=path_to_json_model)
```

Suppose that we have now created our map using the **Escher** web-based API and saved it as the file “simple\_toy\_map.json”. To display the map with the model:

```
[11]:
```

```
# Define path to Escher map
path_to_map = join(mass.test.MAPS_DIR, "simple_toy_map.json")
escher_builder = Builder(
    model_json=path_to_json_model,
    map_json=path_to_map)
escher_builder
```

Finally the forward rate constant data from the `MassModel` object is added to the map:

```
[12]:
```

```
escher_builder.reaction_data = dict(zip(
    model.reactions.list_attr("id"),
    model.reactions.list_attr("forward_rate_constant")
))
```

#### Additional Examples¶

For additional information and examples on how to visualize networks and **MASSpy** models using **Escher**, see the following:

- Animating Simulations with Escher

### Checking Model Quality¶

This notebook example demonstrates the various methods for ensuring quality and consistency in models. Here, the functions of the `qcqa` submodule are used to inspect a broken model and identify the issues that need attention.

```
[1]:
```

```
import mass.test

from mass import MassConfiguration
from mass.util import qcqa

model = mass.test.create_test_model("Model_To_Repair")
```

#### Inspecting a Model¶

To quickly identify all issues in a model, the `qcqa_model()` function of the `qcqa` submodule can be used. The function takes a `MassModel` and Booleans for various kwargs as input, identifies issues in the model based on the kwargs, and prints a report outlining possible issues.

```
[2]:
```

```
qcqa.qcqa_model(
    model,
    parameters=True,        # Check for undefined but necessary parameters in the model
    concentrations=True,    # Check for undefined but necessary concentrations in the model
    fluxes=True,            # Check for undefined steady state fluxes for reactions in the model
    superfluous=True,       # Check for excess parameters and ensure they are consistent.
    elemental=True,         # Check mass and charge balancing of reactions in the model
    simulation_only=True,  # Check for values necessary for simulation only
)
```

```
╒═══════════════════════════════════════════════════════════════╕
│ MODEL ID: RBC_PFK                                             │
│ SIMULATABLE: False                                            │
│ PARAMETERS NUMERICALY CONSISTENT: False                       │
╞═══════════════════════════════════════════════════════════════╡
│ ============================================================= │
│                      MISSING PARAMETERS                       │
│ ============================================================= │
│ Reaction Parameters    Custom Parameters    S.S. Fluxes       │
│ ---------------------  -------------------  -------------     │
│ PGI: Keq; kf           PFK_R01: Keq_PFK_A   GAPD              │
│ PGK: kf                PFK_R11: Keq_PFK_A                     │
│ PGM: Keq               PFK_R21: Keq_PFK_A                     │
│                        PFK_R31: Keq_PFK_A                     │
│                        PFK_R41: Keq_PFK_A                     │
│ ============================================================= │
├───────────────────────────────────────────────────────────────┤
│ ============================================================= │
│                    MISSING CONCENTRATIONS                     │
│ ============================================================= │
│ Initial Conditions               Boundary Conditions          │
│ -------------------------------  ---------------------        │
│ glc__D_c (in HEX1, SK_glc__D_c)  h2o_b (in SK_h2o_c)          │
│ ============================================================= │
├───────────────────────────────────────────────────────────────┤
│ ============================================================= │
│                      CONSISTENCY CHECKS                       │
│ ============================================================= │
│ Superfluous Parameters    Elemental                           │
│ ------------------------  ---------------------------------   │
│ HEX1: Inconsistent        HEX1: {H: -3.0; O: -4.0; P: -1.0}   │
│ PYK: Consistent           PGI: {H: 3.0; O: 4.0; P: 1.0}       │
│                           G6PDH2r: {H: 3.0; O: 4.0; P: 1.0}   │
│                           DM_nadh: {charge: 2.0}              │
│                           GSHR: {charge: 2.0}                 │
│ ============================================================= │
╘═══════════════════════════════════════════════════════════════╛
```

The `simulation_only` kwarg as `True` ensures that identified missing values in the report (excluding steady state fluxes) are necessary for simulation. As seen above, there are a number of missing values and consistency issues that need to be addressed.

#### Identifying Missing Values¶

The report printed by the `qcqa_model()` function shows that there are a number of values in the model that have not yet been defined. Here, the functions of the `qcqa` submodule are used to retrieve the objects in the model that have missing values so that those values can be defined.

##### Missing parameters¶

To identify the reactions that have missing parameter values, the `parameters` flag is set as `True`. Reaction parameters for mass action rate laws (e.g., forward and reverse rate constants, equilibrium constants) and custom parameters for custom rates are checked for undefined numerical values.

```
[3]:
```

```
qcqa.qcqa_model(model, parameters=True)
```

```
╒══════════════════════════════════════════════╕
│ MODEL ID: RBC_PFK                            │
│ SIMULATABLE: False                           │
│ PARAMETERS NUMERICALY CONSISTENT: False      │
╞══════════════════════════════════════════════╡
│ ============================================ │
│             MISSING PARAMETERS               │
│ ============================================ │
│ Reaction Parameters    Custom Parameters     │
│ ---------------------  -------------------   │
│ PGI: Keq; kf           PFK_R01: Keq_PFK_A    │
│ PGK: kf                PFK_R11: Keq_PFK_A    │
│ PGM: Keq               PFK_R21: Keq_PFK_A    │
│                        PFK_R31: Keq_PFK_A    │
│                        PFK_R41: Keq_PFK_A    │
│ ============================================ │
╘══════════════════════════════════════════════╛
```

The report shows that the PGI, PGK, and PGM reactions are missing numerical values for forward rate and equilibrium constants. The `get_missing_reaction_parameters()` function is used to get these reaction objects from the model:

```
[4]:
```

```
qcqa.get_missing_reaction_parameters(model)
```

```
[4]:
```

```
{<MassReaction PGI at 0x7fd819d7f4d0>: 'Keq; kf',
 <MassReaction PGK at 0x7fd819d7fb50>: 'kf',
 <MassReaction PGM at 0x7fd819d7fdd0>: 'Keq'}
```

The `get_missing_reaction_parameters()` function returns a `dict` that contains reaction objects and a string that indicates which parameters are missing. To get a subset of these reactions, a list of reaction identifiers is provided to the `reaction_list` argument. For example, to separate the reactions missing forward rate constants from those that are missing equilibrium constants:

```
[5]:
```

```
missing_kfs = qcqa.get_missing_reaction_parameters(model, reaction_list=["PGI", "PGK"])
missing_Keqs = qcqa.get_missing_reaction_parameters(model, reaction_list=["PGI", "PGM"])

print("Missing forward rate constants: {0!r}".format(list(missing_kfs)))
print("Missing equilibrium constants: {0!r}".format(list(missing_Keqs)))
```

```
Missing forward rate constants: [<MassReaction PGI at 0x7fd819d7f4d0>, <MassReaction PGK at 0x7fd819d7fb50>]
Missing equilibrium constants: [<MassReaction PGI at 0x7fd819d7f4d0>, <MassReaction PGM at 0x7fd819d7fdd0>]
```

The `get_missing_custom_parameters()` function is used to identify missing custom parameters and the reactions that require them.

```
[6]:
```

```
qcqa.get_missing_custom_parameters(model)
```

```
[6]:
```

```
{<EnzymeModuleReaction PFK_R01 at 0x7fd819dc27d0>: 'Keq_PFK_A',
 <EnzymeModuleReaction PFK_R11 at 0x7fd819dc2e50>: 'Keq_PFK_A',
 <EnzymeModuleReaction PFK_R21 at 0x7fd819dcca10>: 'Keq_PFK_A',
 <EnzymeModuleReaction PFK_R31 at 0x7fd819dd55d0>: 'Keq_PFK_A',
 <EnzymeModuleReaction PFK_R41 at 0x7fd819dd50d0>: 'Keq_PFK_A'}
```

Once defined, the parameters no longer appear in the returned `dict` of missing values. A returned empty `dict` indicates that no undefined parameter values exist in the model.

```
[7]:
```

```
# Define missing parameters and update model
missing_parameters = {
    "kf_PGI": 2961.11, "Keq_PGI": 0.41,
    "kf_PGK": 1061655.085,
    "Keq_PGM": 0.147059,
    "Keq_PFK_A": 14.706}
model.update_parameters(missing_parameters)

print("Missing reaction parameters: {0!r}".format(qcqa.get_missing_reaction_parameters(model)))
print("Missing custom parameters: {0!r}".format(qcqa.get_missing_custom_parameters(model)))
```

```
Missing reaction parameters: {}
Missing custom parameters: {}
```

##### Missing fluxes¶

To identify the reactions that have missing steady state flux values, the `fluxes` kwarg is set as `True`.

```
[8]:
```

```
qcqa.qcqa_model(model, fluxes=True)
```

```
╒══════════════════════════════════════════════╕
│ MODEL ID: RBC_PFK                            │
│ SIMULATABLE: False                           │
│ PARAMETERS NUMERICALY CONSISTENT: False      │
╞══════════════════════════════════════════════╡
│ ============================================ │
│             MISSING PARAMETERS               │
│ ============================================ │
│ S.S. Fluxes                                  │
│ -------------                                │
│ GAPD                                         │
│ ============================================ │
╘══════════════════════════════════════════════╛
```

To get the reaction objects that are missing steady state fluxes, the `get_missing_steady_state_fluxes()` function is used. A returned empty `list` indicates that no undefined flux values exist in the model.

```
[9]:
```

```
missing_fluxes = qcqa.get_missing_steady_state_fluxes(model)
print("Before: {0!r}".format(missing_fluxes))

# Define missing flux value
missing_fluxes[0].steady_state_flux = 2.305

missing_fluxes = qcqa.get_missing_steady_state_fluxes(model)
print("After: {0!r}".format(missing_fluxes))
```

```
Before: [<MassReaction GAPD at 0x7fd819d7f590>]
After: []
```

##### Missing concentrations¶

To identify the metabolites that have missing concentrations, the `concentrations` kwarg is set as `True`. Metabolite concentrations refer to the initial and boundary conditions of the model.

```
[10]:
```

```
qcqa.qcqa_model(model, concentrations=True)
```

```
╒══════════════════════════════════════════════════════════╕
│ MODEL ID: RBC_PFK                                        │
│ SIMULATABLE: False                                       │
│ PARAMETERS NUMERICALY CONSISTENT: False                  │
╞══════════════════════════════════════════════════════════╡
│ ======================================================== │
│                 MISSING CONCENTRATIONS                   │
│ ======================================================== │
│ Initial Conditions               Boundary Conditions     │
│ -------------------------------  ---------------------   │
│ glc__D_c (in HEX1, SK_glc__D_c)  h2o_b (in SK_h2o_c)     │
│ ======================================================== │
╘══════════════════════════════════════════════════════════╛
```

The `get_missing_initial_conditions()` function is used to return a list of metabolite objects that have undefined initial conditions:

```
[11]:
```

```
missing_ics = qcqa.get_missing_initial_conditions(model)
print(missing_ics)
```

```
[<MassMetabolite glc__D_c at 0x7fd819d5aed0>]
```

The `get_missing_boundary_conditions()` function is used to return a list of ‘boundary metabolites’ that have undefined boundary conditions. A ‘boundary metabolite’ is a proxy metabolite for a boundary condition not represented by `MassMetabolite` objects.

```
[12]:
```

```
qcqa.get_missing_boundary_conditions(model)
```

```
[12]:
```

```
['h2o_b']
```

Once defined, the metabolites no longer appear in the returned `list`. A returned empty `list` means no undefined metabolite concentrations were found.

```
[13]:
```

```
# Define missing initial condition
missing_ics[0].initial_condition = 1.3
# Define mising boundary condition
model.boundary_conditions["h2o_b"] = 1

# Check model to ensure they have been defined
print("Missing initial conditions: {0!r}".format(qcqa.get_missing_initial_conditions(model)))
print("Missing boundary conditions: {0!r}".format(qcqa.get_missing_boundary_conditions(model)))
```

```
Missing initial conditions: []
Missing boundary conditions: []
```

After defining the missing values, the report displayed by the `qcqa_model()` function shows that the model is simulatable. However, the model parameters are not considered numerically consistent, which may present some problems during the simulation process.

```
[14]:
```

```
qcqa.qcqa_model(model, parameters=True, concentrations=True, fluxes=True)
```

```
╒═══════════════════════════════════════════╕
│ MODEL ID: RBC_PFK                         │
│ SIMULATABLE: True                         │
│ PARAMETERS NUMERICALY CONSISTENT: False   │
╞═══════════════════════════════════════════╡
╘═══════════════════════════════════════════╛
```

#### Consistency Checks¶

In addition to the undefined numerical values in the model, the initial report printed by the `qcqa_model()` function also indicates some issues in parameter consistency and elemental balancing. Here, the functions of the `qcqa` submodule are used to retrieve the objects in the model that have consistency issues so that they can be corrected.

##### Elemental¶

To identify the reactions that are not elementally balanced, the `elemental` kwarg is set as `True`. Note that pseudoreactions are typically unbalanced, and although boundary reactions are excluded by default, other pseudoreactions may exist in the system. In this model, the two pseudoreactions expected to be unbalanced are the `DM_nadh` and the `GSHR` reactions.

```
[15]:
```

```
qcqa.qcqa_model(model, elemental=True)
```

```
╒══════════════════════════════════════════════╕
│ MODEL ID: RBC_PFK                            │
│ SIMULATABLE: True                            │
│ PARAMETERS NUMERICALY CONSISTENT: False      │
╞══════════════════════════════════════════════╡
│ ============================================ │
│             CONSISTENCY CHECKS               │
│ ============================================ │
│ Elemental                                    │
│ ---------------------------------            │
│ HEX1: {H: -3.0; O: -4.0; P: -1.0}            │
│ PGI: {H: 3.0; O: 4.0; P: 1.0}                │
│ G6PDH2r: {H: 3.0; O: 4.0; P: 1.0}            │
│ DM_nadh: {charge: 2.0}                       │
│ GSHR: {charge: 2.0}                          │
│ ============================================ │
╘══════════════════════════════════════════════╛
```

As seen above, there are reactions other than the two expected pseudoreactions that appear in the printed report. Specifically, these are reactions with an imbalance in phosphoric acid (H3PO4). To get the imbalanced reaction objects, use the `check_elemental_consistency()` function.

```
[16]:
```

```
imbalanced_reactions = qcqa.check_elemental_consistency(
    model, reaction_list=["HEX1", "PGI", "G6PDH2r"])
imbalanced_reactions
```

```
[16]:
```

```
{<MassReaction HEX1 at 0x7fd819d7f490>: 'H: -3.0; O: -4.0; P: -1.0',
 <MassReaction PGI at 0x7fd819d7f4d0>: 'H: 3.0; O: 4.0; P: 1.0',
 <MassReaction G6PDH2r at 0x7fd819d8e4d0>: 'H: 3.0; O: 4.0; P: 1.0'}
```

By looking at the reactions, their stoichiometries, and the unbalanced elements, it is clear that glucose 6-phosphate (G6P) is missing a phosphoric acid in its chemica formula.

```
[17]:
```

```
for reaction, unbalanced in imbalanced_reactions.items():
    print(reaction)

g6p_c = model.metabolites.get_by_id("g6p_c")
print("\n{0} formula before: {1}".format(g6p_c.id, repr(g6p_c.formula)))
```

```
HEX1: atp_c + glc__D_c <=> adp_c + g6p_c + h_c
PGI: g6p_c <=> f6p_c
G6PDH2r: g6p_c + nadp_c <=> _6pgl_c + h_c + nadph_c

g6p_c formula before: 'C6H8O5'
```

The current elemental composition of G6P is combined with the elemental composition of phosphoric acid:

```
[18]:
```

```
# Get existing formula composition
formula_composition = g6p_c.elements

# Update with the phosphoric acid
phosphoric_acid = {"H": 3, "P": 1, "O": 4}
for element, to_add in phosphoric_acid.items():
    if element in formula_composition:
        formula_composition[element] += to_add
    else:
        formula_composition[element] = to_add

# Change the existing formula to the new one
g6p_c.elements = formula_composition

print("{0} formula after: {1}".format(g6p_c.id, repr(g6p_c.formula)))
```

```
g6p_c formula after: 'C6H11O9P'
```

The reactions are no longer considered imbalanced.

```
[19]:
```

```
imbalanced_reactions = qcqa.check_elemental_consistency(
    model, reaction_list=["HEX1", "PGI", "G6PDH2r"])
imbalanced_reactions
```

```
[19]:
```

```
{}
```

##### Superfluous parameters¶

To identify the reactions with superfluous parameters, the `superfluous` kwarg is set as `True`. If a reaction has superfluous parameters, the parameters are checked to ensure that they are numerically consistent:

```
[20]:
```

```
qcqa.qcqa_model(model, superfluous=True)
```

```
╒══════════════════════════════════════════════╕
│ MODEL ID: RBC_PFK                            │
│ SIMULATABLE: True                            │
│ PARAMETERS NUMERICALY CONSISTENT: False      │
╞══════════════════════════════════════════════╡
│ ============================================ │
│             CONSISTENCY CHECKS               │
│ ============================================ │
│ Superfluous Parameters                       │
│ ------------------------                     │
│ HEX1: Inconsistent                           │
│ PYK: Consistent                              │
│ ============================================ │
╘══════════════════════════════════════════════╛
```

The pyruvate kinase reaction (PYK) contains a consistent superfluous parameter. A consistent superfluous parameter indicates that although an extra parameter is defined, the forward rate constant, reverse rate constant, and the equilibrium constant are numerically consistent with consistency being determined as \(|k\_{f} / K\_{eq} - k\_{r}| \le tolerance\). The tolerance is determined by the `decimal_precision` of the `MassConfiguration` object (e.g., a `decimal_precision` of eight
corresponds to rounding at the 8th digit right of the decimal, equivalent to \(|k\_{f} / K\_{eq} - k\_{r}| \le 10^{-8}\).

```
[21]:
```

```
PYK = model.reactions.get_by_id("PYK")
print(abs(PYK.kf / PYK.Keq - PYK.kr))
```

```
0.0
```

The hexokinase reaction (HEX1) contains an inconsistent superfluous parameter:

```
[22]:
```

```
HEX1 = model.reactions.get_by_id("HEX1")
print(abs(HEX1.kf / HEX1.Keq - HEX1.kr))
```

```
10.0
```

Inconsistent superfluous parameters are quickly fixed by defining them as a consistent value, or ignored by setting the value as `None`.

```
[23]:
```

```
HEX1.kr = None
qcqa.qcqa_model(model, superfluous=True)
```

```
╒══════════════════════════════════════════════╕
│ MODEL ID: RBC_PFK                            │
│ SIMULATABLE: True                            │
│ PARAMETERS NUMERICALY CONSISTENT: True       │
╞══════════════════════════════════════════════╡
│ ============================================ │
│             CONSISTENCY CHECKS               │
│ ============================================ │
│ Superfluous Parameters                       │
│ ------------------------                     │
│ PYK: Consistent                              │
│ ============================================ │
╘══════════════════════════════════════════════╛
```

After addressing several of the model issues, the `qcqa_model()` function at the beginning of this notebook can be reused. This time, the report indicates that the model is elementally balanced and contains the numerical values necessary for simulation.

```
[24]:
```

```
qcqa.qcqa_model(
    model,
    parameters=True,        # Check for undefined but necessary parameters in the model
    concentrations=True,    # Check for undefined but necessary concentrations in the model
    fluxes=True,            # Check for undefined steady state fluxes for reactions in the model
    superfluous=True,       # Check for excess parameters and ensure they are consistent.
    elemental=True,         # Check mass and charge balancing of reactions in the model
)
```

```
╒════════════════════════════════════════════════════╕
│ MODEL ID: RBC_PFK                                  │
│ SIMULATABLE: True                                  │
│ PARAMETERS NUMERICALY CONSISTENT: True             │
╞════════════════════════════════════════════════════╡
│ ================================================== │
│                CONSISTENCY CHECKS                  │
│ ================================================== │
│ Superfluous Parameters    Elemental                │
│ ------------------------  ----------------------   │
│ PYK: Consistent           DM_nadh: {charge: 2.0}   │
│                           GSHR: {charge: 2.0}      │
│ ================================================== │
╘════════════════════════════════════════════════════╛
```

### Global Configuration¶

This notebook example demonstrates how the global configuration object, the `MassConfiguration`, is used to configure the default behaviors for various **COBRApy** and **MASSpy** methods.

```
[1]:
```

```
import cobra

import mass
from mass.test import create_test_model

cobra_config = cobra.Configuration()
```

Note that changing the global configuration values is the most useful at the beginning of a work session.

#### The MassConfiguration Object¶

Similar to the `cobra.Configuration` object, the `MassConfiguration` object is a singleton, meaning that only one instance can exist and is respected everywhere in **MASSpy**.

The `MassConfiguration` is retrieved via the following:

```
[2]:
```

```
mass_config = mass.MassConfiguration()
```

The `MassConfiguration` is synchronized with the `cobra.Configuration` singleton object such that a change in one configuration object affects the other.

```
[3]:
```

```
print("cobra configuration before: {0!r}".format(cobra_config.bounds))
# Change bounds using the MassConfiguration object
mass_config.bounds = (-444, 444)
print("cobra configuration after: {0!r}".format(cobra_config.bounds))
```

```
cobra configuration before: (-1000.0, 1000.0)
cobra configuration after: (-444, 444)
```

This means that changes only need to be made to the `MassConfiguration` object for workflows that involve both the **COBRApy** and **MASSpy** packages. The shared configuration attributes can be viewed using the `MassConfiguration.shared_state` attribute.

```
[4]:
```

```
list(mass_config.shared_state)
```

```
[4]:
```

```
['solver', 'tolerance', 'lower_bound', 'upper_bound', 'processes']
```

#### Attributes for Model Construction¶

The following attributes of the `MassConfiguration` alter default behaviors for constructing models and importing/exporting models via SBML.

```
[5]:
```

```
from mass import MassMetabolite, MassReaction
```

##### For irreversible reactions¶

When an irreversible reaction is created, the equilibrium constant and reverse rate constant are automatically set based on the `irreversible_Keq` and `irreversible_kr` attributes, respectively.

```
[6]:
```

```
mass_config.irreversible_Keq = float("inf")
mass_config.irreversible_kr = 0

print("Irreversible Keq: {0}".format(mass_config.irreversible_Keq))
print("Irreversible kr: {0}".format(mass_config.irreversible_kr))
R1 = MassReaction("R1", reversible=False)
R1.parameters
```

```
Irreversible Keq: inf
Irreversible kr: 0
```

```
[6]:
```

```
{'Keq_R1': inf, 'kr_R1': 0}
```

Changing the `irreversible_Keq` and `irreversible_kr` attributes affects newly created `MassReaction` objects.

```
[7]:
```

```
mass_config.irreversible_Keq = 10e6
mass_config.irreversible_kr = 1e-6
print("Irreversible Keq: {0}".format(mass_config.irreversible_Keq))
print("Irreversible kr: {0}\n".format(mass_config.irreversible_kr))

# Create new reaction
R2 = MassReaction("R2", reversible=False)
print(R2.parameters)
```

```
Irreversible Keq: 10000000.0
Irreversible kr: 1e-06

{'Keq_R2': 10000000.0, 'kr_R2': 1e-06}
```

Existing reactions are not affected.

```
[8]:
```

```
print(R1.parameters)
```

```
{'Keq_R1': inf, 'kr_R1': 0}
```

##### For rate expressions¶

Automatic generation of rate expressions are affected using the `exclude_metabolites_from_rates` and `exclude_compartment_volumes_in_rates` attributes.

```
[9]:
```

```
model = create_test_model("textbook")
```

###### Excluding metabolites from rates¶

The `exclude_metabolites_from_rates` attribute determines which metabolites to exclude from rate expressions by using a dictionary that contains a metabolite attribute for filtering, and a list of values to be excluded.

```
[10]:
```

```
mass_config.exclude_metabolites_from_rates
```

```
[10]:
```

```
{'elements': [{'H': 2, 'O': 1}, {'H': 1}]}
```

The default setting utilizes the `MassMetabolite.elements` attribute for filtering, excluding any metabolite that returns the elements for hydrogen and water.

```
[11]:
```

```
ENO = model.reactions.get_by_id("ENO")
print(ENO.rate)
```

```
kf_ENO*(_2pg_c(t) - pep_c(t)/Keq_ENO)
```

The `exclude_metabolites_from_rates` attribute can be changed by providing a `dict` that contains a metabolite attribute for filtering and the list of values to be excluded. For example, to exclude “2pg\_c” by using its `name` attribute as the criteria for exclusion:

```
[12]:
```

```
mass_config.exclude_metabolites_from_rates = {"name": ["D-Glycerate 2-phosphate"]}
ENO = model.reactions.get_by_id("ENO")
print(ENO.rate)
```

```
kf_ENO*(1 - h2o_c(t)*pep_c(t)/Keq_ENO)
```

Or, to exclude hydrogen and water by using their identifiers:

```
[13]:
```

```
mass_config.exclude_metabolites_from_rates = {"id": ["h_c", "h2o_c"]}
ENO = model.reactions.get_by_id("ENO")
print(ENO.rate)
```

```
kf_ENO*(_2pg_c(t) - pep_c(t)/Keq_ENO)
```

Boundary reactions are unaffected by the `exclude_metabolites_from_rates` attribute:

```
[14]:
```

```
for rid in ["SK_h_c", "SK_h2o_c"]:
    reaction = model.reactions.get_by_id(rid)
    print(reaction.rate)
```

```
kf_SK_h_c*(h_c(t) - h_b/Keq_SK_h_c)
kf_SK_h2o_c*(h2o_c(t) - h2o_b/Keq_SK_h2o_c)
```

###### Excluding compartments from rates¶

The `exclude_compartment_volumes_in_rates` attribute determines whether compartment volumes are factored into rate expressions. By default, compartment volumes are not included in automatically generated rate expressions:

```
[15]:
```

```
PGI = model.reactions.get_by_id("PGI")
print(PGI.rate)
```

```
kf_PGI*(g6p_c(t) - f6p_c(t)/Keq_PGI)
```

When the `exclude_compartment_volumes_in_rates` attribute is set as `False`, compartments are included in rate expressions as `volume_CID`, with `CID` referring to the compartment identifier.

```
[16]:
```

```
mass_config.exclude_compartment_volumes_in_rates = False

PGI = model.reactions.get_by_id("PGI")
model.custom_parameters["volume_c"] = 1

print(PGI.rate)
```

```
kf_PGI*volume_c*(g6p_c(t) - f6p_c(t)/Keq_PGI)
```

The compartment volume is currently treated as a custom parameter. This behavior is subject to change in future updates following the release of COBRApy compartment objects.

##### For compartments and SBML¶

The `boundary_compartment` attribute defines the compartment for any external boundary species.

```
[17]:
```

```
# Create a boundary reaction
x1_c = MassMetabolite("x1_c", compartment="c")
R3 = MassReaction("R1")
R3.add_metabolites({x1_c: -1})

print(mass_config.boundary_compartment)
R3.boundary_metabolite
```

```
{'b': 'boundary'}
```

```
[17]:
```

```
'x1_b'
```

The `boundary_compartment` can be changed using a `dict` that contains the new compartment identifier and its full name.

```
[18]:
```

```
mass_config.boundary_compartment = {"xt": "external"}
R3.boundary_metabolite
```

```
[18]:
```

```
'x1_xt'
```

Because the `mass.Simulation` object uses the **libRoadRunner** package, a simulator for SBML models, a model cannot be simulated without defining at least one compartment. The `default_compartment` attribute is used to define the compartment of the model when no compartments have been defined.

```
[19]:
```

```
mass_config.default_compartment
```

```
[19]:
```

```
{'compartment': 'default_compartment'}
```

As with the `boundary_compartment` attribute, the `default_compartment` attribute can be changed using a `dict`:

```
[20]:
```

```
mass_config.default_compartment = {"def": "default_compartment"}
mass_config.default_compartment
```

```
[20]:
```

```
{'def': 'default_compartment'}
```

###### Model creator¶

SBML also allows for a model creator to be defined when exporting models:

```
[21]:
```

```
mass_config.model_creator
```

```
[21]:
```

```
{'familyName': '', 'givenName': '', 'organization': '', 'email': ''}
```

The `model_creator` attribute of the `MassConfiguration` allows the model creator to be set at the time of export by using a `dict`, with valid keys as “familyName”, “givenName”, “organization”, and “email”.

```
[22]:
```

```
mass_config.model_creator = {
    "familyName": "Smith",
    "givenName": "John",
    "organization": "Systems Biology Research Group @UCSD"}
mass_config.model_creator
```

```
[22]:
```

```
{'familyName': 'Smith',
 'givenName': 'John',
 'organization': 'Systems Biology Research Group @UCSD',
 'email': ''}
```

#### Attributes for Simulation and Analysis¶

The following attributes of the `MassConfiguration` alter default behaviors of various simulation and analytical methods.

```
[23]:
```

```
from mass import Simulation

# Reset configurations before loading model
mass_config.boundary_compartment = {"b": "boundary"}
mass_config.exclude_compartment_volumes_in_rates = True

model = create_test_model("Glycolysis")
sim = Simulation(model, verbose=True)
```

```
Successfully loaded MassModel 'Glycolysis' into RoadRunner.
```

##### Steady state threshold¶

The `MassConfiguration.steady_state_threshold` attribute determines whether a model has reached a steady state using the following criteria:

- With simulations (i.e., `strategy=simulate`), the absolute difference between the last two solution points must be less than or equal to the steady state threshold.
- With steady state solvers, the sum of squares of the steady state solutions must be less than or equal to the steady state threshold.

In general, compared values must be less than or equal to the `steady_state_threshold` attribute to be considered at a steady state.

```
[24]:
```

```
mass_config.steady_state_threshold = 1e-20
conc_sol, flux_sol = sim.find_steady_state(model, strategy="simulate")
bool(conc_sol)  # Empty solution objects return False
```

```
mass/simulation/simulation.py:828 UserWarning: Unable to find a steady state for one or more models. Check the log for more details.
ERROR: Unable to find a steady state for 'Glycolysis' using strategy 'simulate' due to the following: For MassModel "Glycolysis", absolute difference for "['[fdp_c]']" is greater than the steady state threshold.
```

```
[24]:
```

```
False
```

Changing the threshold affects whether solution values are considered to be at steady state:

```
[25]:
```

```
mass_config.steady_state_threshold = 1e-6
conc_sol, flux_sol = sim.find_steady_state(model, strategy="simulate")
bool(conc_sol)  # Filled solution objects return False
```

```
[25]:
```

```
True
```

##### Decimal precision¶

The `MassConfiguration.decimal_precision` attribute is a special attribute used in several `mass` methods. The value of the attribute determines how many digits in rounding after the decimal to preserve.

For many methods, the `decimal_precision` attribute will not be applied unless a `decimal_precision` kwarg is set as `True`.

```
[26]:
```

```
# Set decimal precision
mass_config.decimal_precision = 8

# Will not apply decimal precision to steady state solutions
conc_sol, flux_sol = sim.find_steady_state(model, strategy="simulate",
                                           decimal_precision=False)
print(conc_sol["glc__D_c"])

# Will apply decimal precision to steady state solutions
conc_sol, flux_sol = sim.find_steady_state(model, strategy="simulate",
                                           decimal_precision=True)
print(conc_sol["glc__D_c"])
```

```
1.0000003633303345
1.00000036
```

If `MassConfiguration.decimal_precision` is `None`, no rounding will occur.

```
[27]:
```

```
mass_config.decimal_precision = None

# Will apply decimal precision to steady state solutions
conc_sol, flux_sol = sim.find_steady_state(model, strategy="simulate",
                                           decimal_precision=True)
print(conc_sol["glc__D_c"])
```

```
1.0000003633303345
```

#### Shared COBRA Attributes¶

The following attributes are those shared with the `cobra.Configuration` object.

##### Bounds¶

When a reaction is created, its default bound values are determined by the `lower_bound` and `upper_bound` attributes of the `MassConfiguration`:

```
[28]:
```

```
mass_config.lower_bound = -1000
mass_config.upper_bound = 1000
R4 = MassReaction("R4")
print("R4 bounds: {0}".format(R4.bounds))
```

```
R4 bounds: (-1000, 1000)
```

Changing the bounds affects newly created reactions, but not existing ones:

```
[29]:
```

```
mass_config.bounds = (-444, 444)
R5 = MassReaction("R5")
print("R5 bounds: {0}".format(R5.bounds))
print("R4 bounds: {0}".format(R4.bounds))
```

```
R5 bounds: (-444, 444)
R4 bounds: (-1000, 1000)
```

##### Solver¶

The default solver and solver tolerance attributes are determined by the `solver` and `tolerance` attributes of the `MassConfiguration`. The `solver` and `tolerance` attributes are utilized by newly instantiated models and `ConcSolver` objects.

```
[30]:
```

```
model = create_test_model("textbook")
print("Solver {0!r}".format(model.solver))
print("Tolerance {0}".format(model.tolerance))
```

```
Solver <optlang.gurobi_interface.Model object at 0x7fb2b0b06550>
Tolerance 1e-07
```

The default solver can be changed, depending on the solvers installed in the current environment. GLPK is assumed to always be present in the environment.

The solver tolerance is similarly set using the `tolerance` attribute.

```
[31]:
```

```
# Change solver and solver tolerance
mass_config.solver = "glpk"
mass_config.tolerance = 1e-4

# Instantiate a new model to observe changes
model = create_test_model("textbook")
print("Solver {0!r}".format(model.solver))
print("Tolerance {0}".format(model.tolerance))
```

```
Solver <optlang.glpk_interface.Model object at 0x7fb2b0ba9510>
Tolerance 0.0001
```

##### Number of processes¶

The `MassConfiguration.processes` determines the default number of processes used when multiprocessing is possible. The default number corresponds to the number of available cores (hyperthreads).

```
[32]:
```

```
mass_config.processes
```

```
[32]:
```

```
3
```

### Using COBRApy with MASSpy¶

This notebook example demonstrates how to convert **COBRApy** objects into their equivalent **MASSpy** objects, and highlights some of the differences between them.

```
[1]:
```

```
import cobra.test

from mass import MassMetabolite, MassModel, MassReaction
```

#### Converting COBRA to MASS¶

Converting **COBRApy** objects into their **MASSpy** equivalents is a simple process. It only requires the user to instantiate the **MASSpy** object using the **COBRApy** object.

```
[2]:
```

```
# Get some COBRA objects
cobra_model = cobra.test.create_test_model("textbook")
cobra_metabolite = cobra_model.metabolites.get_by_id("atp_c")
cobra_reaction = cobra_model.reactions.get_by_id("PGI")
```

##### Metabolite to MassMetabolite¶

To convert a `cobra.Metabolite` to a `mass.MassMetabolite`:

```
[3]:
```

```
mass_metabolite = MassMetabolite(cobra_metabolite)
mass_metabolite
```

```
[3]:
```

|  |  |
| --- | --- |
| **MassMetabolite identifier** | atp\_c |
| **Name** | ATP |
| **Memory address** | 0x07fe88c817390 |
| **Formula** | C10H12N5O13P3 |
| **Compartment** | c |
| **Initial Condition** | None |
| **In 0 reaction(s)** |  |

Note that converted metabolites do not retain any references to the previously associated `cobra.Reaction` or `cobra.Model`.

```
[4]:
```

```
for metabolite in [cobra_metabolite, mass_metabolite]:
    print("Number of Reactions: {0}; Model: {1}".format(len(metabolite.reactions), metabolite.model))
```

```
Number of Reactions: 13; Model: e_coli_core
Number of Reactions: 0; Model: None
```

However, all attributes that the `mass.MassMetabolite` object inherits from the `cobra.Metabolite` object are preserved:

```
[5]:
```

```
for attr in ["id", "name", "formula", "charge", "compartment"]:
    print("Identical '{0}': {1}".format(
        attr, getattr(cobra_metabolite, attr) == getattr(mass_metabolite, attr)))
```

```
Identical 'id': True
Identical 'name': True
Identical 'formula': True
Identical 'charge': True
Identical 'compartment': True
```

##### Reaction to MassReaction¶

To convert a `cobra.Reaction` to a `mass.MassReaction`:

```
[6]:
```

```
mass_reaction = MassReaction(cobra_reaction)
mass_reaction
```

```
[6]:
```

|  |  |
| --- | --- |
| **Reaction identifier** | PGI |
| **Name** | glucose-6-phosphate isomerase |
| **Memory address** | 0x07fe88c808650 |
| **Subsystem** |  |
| **Kinetic Reversibility** | True |
| **Stoichiometry** | g6p\_c <=> f6p\_c  D-Glucose 6-phosphate <=> D-Fructose 6-phosphate |
| **GPR** | b4025 |
| **Bounds** | (-1000.0, 1000.0) |

Upon conversion of a reaction, all associated `cobra.Metabolite` objects are converted to `mass.MassMetabolite` objects.

```
[7]:
```

```
for metabolite in mass_reaction.metabolites:
    print(metabolite, type(metabolite))
```

```
g6p_c <class 'mass.core.mass_metabolite.MassMetabolite'>
f6p_c <class 'mass.core.mass_metabolite.MassMetabolite'>
```

If there are genes present, they are copied from one reaction to another in order to create a new `cobra.Gene` object for the `MassReaction`.

```
[8]:
```

```
print(cobra_reaction.genes)
print(mass_reaction.genes)
```

```
frozenset({<Gene b4025 at 0x7fe88c57c450>})
frozenset({<Gene b4025 at 0x7fe88c808fd0>})
```

All other references to **COBRApy** objects are removed.

```
[9]:
```

```
print(cobra_reaction.model)
print(mass_reaction.model)
```

```
e_coli_core
None
```

All attributes that the `mass.MassReaction` object inherits from the `cobra.Reaction` object are preserved upon conversion.

```
[10]:
```

```
for attr in ["id", "name", "subsystem", "bounds", "compartments", "gene_reaction_rule"]:
    print("Identical '{0}': {1}".format(
        attr, getattr(cobra_reaction, attr) == getattr(mass_reaction, attr)))
```

```
Identical 'id': True
Identical 'name': True
Identical 'subsystem': True
Identical 'bounds': True
Identical 'compartments': True
Identical 'gene_reaction_rule': True
```

##### Model to MassModel¶

To convert a `cobra.Model` to a `mass.MassModel`:

```
[11]:
```

```
mass_model = MassModel(cobra_model)
mass_model
```

```
[11]:
```

|  |  |
| --- | --- |
| **Name** | e\_coli\_core |
| **Memory address** | 0x07fe88c801490 |
| **Stoichiometric Matrix** | 72x95 |
| **Matrix Rank** | 67 |
| **Number of metabolites** | 72 |
| **Initial conditions defined** | 0/72 |
| **Number of reactions** | 95 |
| **Number of genes** | 137 |
| **Number of enzyme modules** | 0 |
| **Number of groups** | 0 |
| **Objective expression** | 1.0\*Biomass\_Ecoli\_core - 1.0\*Biomass\_Ecoli\_core\_reverse\_2cdba |
| **Compartments** | cytosol, extracellular |

During conversion, the original `cobra.Model` remains untouched, while a new `mass.MassModel` is created using the equivalent `mass` objects. All references to the original `cobra.Model` are updated with references to the newly created `mass.MassModel`.

```
[12]:
```

```
print("All MassMetabolites: {0}".format(
    all([isinstance(met, MassMetabolite)
         for met in mass_model.metabolites])))
print("All MassReactions: {0}".format(
    all([isinstance(rxn, MassReaction)
         for rxn in mass_model.reactions])))
```

```
All MassMetabolites: True
All MassReactions: True
```

#### Differences between COBRA and MASS¶

Although there are several similarities between **COBRApy** and **MASSpy**, there are some key differences in behavior that are worth highlighting.

##### COBRA vs. MASS reactions¶

There are some key differences between `cobra.Reaction` and `mass.MassReaction` objects. They are summarized below:

###### `reversible` vs. `reversibility` attributes¶

One key difference observed is how a reaction direction is determined. A `cobra.Reaction` utilizes the lower and upper bound values to determine the `reversibility` attribute.

```
[13]:
```

```
print(cobra_reaction.reaction)
print(cobra_reaction.bounds)
print(cobra_reaction.reversibility)
```

```
g6p_c <=> f6p_c
(-1000.0, 1000.0)
True
```

Changing the reaction bounds affects the direction a reaction can proceed:

```
[14]:
```

```
for header, bounds in zip(["Both Directions", "Forward Direction", "Reverse Direction"],
                          [(-1000, 1000), (0, 1000), (-1000, 0)]):

    print("\n".join((header, "-" * len(header))))
    cobra_reaction.bounds = bounds
    print(cobra_reaction.reaction)
    print(cobra_reaction.bounds)
    print("Reversibility: {0}\n".format(cobra_reaction.reversibility))
```

```
Both Directions
---------------
g6p_c <=> f6p_c
(-1000, 1000)
Reversibility: True

Forward Direction
-----------------
g6p_c --> f6p_c
(0, 1000)
Reversibility: False

Reverse Direction
-----------------
g6p_c <-- f6p_c
(-1000, 0)
Reversibility: False
```

Although `MassReaction` objects still have the `reversibility` attribute based on reaction bounds, the reaction rate equation is based on the `reversible` attribute. Additionally, the displayed reaction arrow for a reaction string now depends on the `reversible` attribute, rather than the `reversibility` attribute.

Therefore, even if the flux is constrained to proceed in one direction by the bounds, the kinetic rate expression still accounts for a reverse rate.

```
[15]:
```

```
for header, bounds in zip(["Forward Direction (Flux)", "Reverse Direction (Flux)", "Both Directions (Flux)"],
                          [(0, 1000), (-1000, 0), (-1000, 1000)]):

    print("\n".join((header, "-" * len(header))))
    mass_reaction.bounds = bounds
    print(mass_reaction.reaction)
    print(mass_reaction.bounds)
    print("Reversibility: {0}".format(mass_reaction.reversibility))
    print("Reversible (Kinetic): {0}".format(mass_reaction.reversible))
    print("Rate: {0}\n".format(mass_reaction.rate))
```

```
Forward Direction (Flux)
------------------------
g6p_c <=> f6p_c
(0, 1000)
Reversibility: False
Reversible (Kinetic): True
Rate: kf_PGI*(g6p_c(t) - f6p_c(t)/Keq_PGI)

Reverse Direction (Flux)
------------------------
g6p_c <=> f6p_c
(-1000, 0)
Reversibility: False
Reversible (Kinetic): True
Rate: kf_PGI*(g6p_c(t) - f6p_c(t)/Keq_PGI)

Both Directions (Flux)
----------------------
g6p_c <=> f6p_c
(-1000, 1000)
Reversibility: True
Reversible (Kinetic): True
Rate: kf_PGI*(g6p_c(t) - f6p_c(t)/Keq_PGI)
```

Changing the `reversible` attribute affects the kinetic rate expression for the reaction, but it does not affect the reaction bounds.

```
[16]:
```

```
for header, reversible in zip(["Both Directions (Kinetics)", "Forward Direction (Kinetics)"], [True, False]):
    print("\n".join((header, "-" * len(header))))
    mass_reaction.reversible = reversible
    print(mass_reaction.reaction)
    print(mass_reaction.bounds)
    print("Reversibility: {0}".format(mass_reaction.reversibility))
    print("Reversible (Kinetic): {0}".format(mass_reaction.reversible))
    print("Rate: {0}\n".format(mass_reaction.rate))
```

```
Both Directions (Kinetics)
--------------------------
g6p_c <=> f6p_c
(-1000, 1000)
Reversibility: True
Reversible (Kinetic): True
Rate: kf_PGI*(g6p_c(t) - f6p_c(t)/Keq_PGI)

Forward Direction (Kinetics)
----------------------------
g6p_c --> f6p_c
(-1000, 1000)
Reversibility: True
Reversible (Kinetic): False
Rate: kf_PGI*g6p_c(t)
```

To obtain the reaction in the reverse direction instead of the forward direction, the `MassReaction.reverse_stoichiometry()` method can be used. Setting `inplace=False` produces a new reaction, while setting `inplace=True` modifies the existing reaction. Setting `reverse_bounds=True` switches the lower and upper bound values.

```
[17]:
```

```
mass_reaction_rev = mass_reaction.reverse_stoichiometry(inplace=False)
print(mass_reaction_rev.reaction)
print(mass_reaction_rev.bounds)
print("Reversibility: {0}".format(mass_reaction_rev.reversibility))
print("Reversible (Kinetic): {0}".format(mass_reaction_rev.reversible))
print("Rate: {0}\n".format(mass_reaction_rev.rate))
```

```
f6p_c --> g6p_c
(-1000, 1000)
Reversibility: True
Reversible (Kinetic): False
Rate: kf_PGI*f6p_c(t)
```

###### `flux` vs. `steady_state_flux` attributes¶

Another difference observed between `cobra.Reaction` and `mass.MassReaction` is how flux values are stored. When a model is optimized for FBA, the `flux` attribute of the reaction reflects the solution directly produced by the solver.

```
[18]:
```

```
cobra_model = cobra.test.create_test_model("textbook")
cobra_model.optimize()
cobra_reaction = cobra_model.reactions.get_by_id("PGI")
cobra_reaction.flux
```

```
[18]:
```

```
4.860861146496817
```

`MassModel` objects retain their ability to be optimized for FBA. Consequently, the ability to retrieve a solution for a reaction using the `flux` attribute is also retained.

```
[19]:
```

```
cobra_model = cobra.test.create_test_model("textbook")
mass_model = MassModel(cobra_model)
mass_model.optimize()
mass_reaction = mass_model.reactions.get_by_id("PGI")
mass_reaction.flux
```

```
[19]:
```

```
4.860861146496817
```

The value of the `flux` attribute is not the same as the `steady_state_flux` attribute, which is used in various `mass` methods:

```
[20]:
```

```
print(mass_reaction.steady_state_flux)
```

```
None
```

To set `steady_state_flux` attributes for all reactions based on the optimization solutions, the `MassModel.set_steady_state_fluxes_from_solver()` method is used.

```
[21]:
```

```
mass_model.set_steady_state_fluxes_from_solver()
# Display for first 10 reactions
for reaction in mass_model.reactions[:10]:
    print(reaction.id, reaction.steady_state_flux)
```

```
ACALD 0.0
ACALDt 0.0
ACKr 0.0
ACONTa 6.007249575350331
ACONTb 6.007249575350331
ACt2r 0.0
ADK1 0.0
AKGDH 5.064375661482091
AKGt2r 0.0
ALCD2x 0.0
```

### Modeling Volumes and Multiple Compartments¶

This notebook example provides a basic demonstration on how to create and dynamically simulate multi-compartment models.

Illustrated below is the multi-compartment model utilized in this notebook:

In the above example: \* For metabolite **x**: \* The biochemical pathway for the conversion of **x** occurs in the `large` compartment outlined by the dotted black line. \* For metabolite **y**: \* Cofactor **y** is necessary for the conversion of metabolite **x** in the biochemical pathway. \* The synthesis of **y** occurs in the `medium` compartment outlined by the blue line. \* `R_Ytr` is an antiporter, coupling the import of **y2** into the `large` compartment with the export of
**y3** to the `medium` compartment. \* For metabolite **z**: \* Protein **z** is synthesized in the `small` compartment outlined by the red line. \* Protein **z** is also to facilitate the conversion of **x5** back into **x4** and for metabolic functions outside of the model’s scope.

The reaction converting **x5** back to **x4** converted into **x5** The pair of irreversible reactions `R3_X` and `R_XZ` form a cycle that is used to The synthesis and degradation of metabolite **y** occurs in the **medium compartment** outlined in blue.

COBRApy is currently in the process of developing improved compartment handling. These changes are outlined in the following COBRApy issues:

- https://github.com/opencobra/cobrapy/pull/725
- https://github.com/opencobra/cobrapy/projects/5

MASSpy is awaiting these COBRApy changes in order to improve how compartments are handled in dynamic simulations, SBML compatibity, etc. Once these changes have been implemented in COBRApy, a new version of MASSpy will be developed and released with improved functionality around compartments and their handling.

#### Models with Multiple Compartments¶

```
[1]:
```

```
import sympy as sym

from mass import (
    MassConfiguration, MassMetabolite, MassModel, MassReaction, Simulation)
from mass.test import create_test_model
from mass.visualization import plot_time_profile
model = create_test_model("MultiCompartment")
```

##### Viewing compartments in a model¶

The `MassModel.compartments` attribute is used to get `dict` with compartment identifiers and their corresponding names.

```
[2]:
```

```
model.compartments
```

```
[2]:
```

```
{'l': 'Large', 'm': 'Medium', 's': 'Small'}
```

The names for the compartments can be reset or changed by using the `MassModel.compartments` attribute setter method. To reset compartment names, pass an empty dict:

```
[3]:
```

```
model.compartments = {}
```

To set a new name for a compartment, set a `dict` using the `MassModel.compartments` method with the compartment identifer as the key and the compartment name as the value. Compartments can be set one at a time, or multiple at once:

```
[4]:
```

```
model.compartments = {"l": "the large compartment"}
print(model.compartments)

model.compartments = {"m": "the medium compartment", "s": "the small compartment"}
print(model.compartments)
```

```
{'l': 'the large compartment', 'm': '', 's': ''}
{'l': 'the large compartment', 'm': 'the medium compartment', 's': 'the small compartment'}
```

###### Volume units¶

To get a list of all UnitDefinition(s) that contain a volume base unit, an modified filter that scans the base units can be applied:

```
[5]:
```

```
def volumes_filter(udef):
    if list(filter(lambda u: u.kind in ["liter","litre"], udef.list_of_units)):
        return True
    return False
print(model.units.query(volumes_filter))
```

```
[<UnitDefinition Milliliters "mL" at 0x7ffd39e79710>, <UnitDefinition Concentration "mol_per_mL" at 0x7ffd39e79750>]
```

##### Enabling compartment volumes in rate laws¶

By default, the `MassConfiguration.exclude_compartment_volumes_in_rates` is set as `True`.

```
[6]:
```

```
mass_config = MassConfiguration()
print(mass_config.exclude_compartment_volumes_in_rates)
```

```
True
```

Therefore, all automatically generated mass action rate laws do not include the compartment volume:

```
[7]:
```

```
print(model.reactions.get_by_id("R2_X").rate)
```

```
kf_R2_X*x3_l(t)
```

To enable compartment volumes in rate laws, the `MassConfiguration.exclude_compartment_volumes_in_rates` attribute must be set to `False`.

```
[8]:
```

```
mass_config.exclude_compartment_volumes_in_rates = False
print(model.reactions.get_by_id("R2_X").rate)
```

```
kf_R2_X*volume_l*x3_l(t)
```

As seen above, volume parameters are added into the rate laws to represent compartment volumes. The volume parameters have identifiers of format `volume_CID` , with `CID` referring to the compartment identifier (e.g., “l” for large compartment). For a reaction that crosses compartments, more than one “volume” parameter will appear as a variable in the rate:

```
[9]:
```

```
for param in model.reactions.get_by_id("R_Ytr").rate.atoms(sym.Symbol):
    if str(param).find("volume") != -1:
        print(param)
```

```
volume_l
volume_m
```

See the section on Excluding compartments from rates in the Global Configuration tutorial for more information about the `exclude_compartment_volumes_in_rates` attribute.

##### The “boundary” compartment¶

In boundary reactions (e.g., pseudeoreactions such as sinks, demands, and exchanges), metabolites that exist in the `boundary` a.k,a. the boundary conditions, are given a default “boundary” compartment with the identifier “b”. This compartment is treated as a pseudo-compartment, and therefore the ‘boundary’ metabolites are treated as pseudo-metabolites, meaning no corresponding object is created for them.

Boundary metabolites can be accessed either through the `MassReaction.boundary_metabolite` method.

```
[10]:
```

```
x1_b = model.reactions.get_by_id("SK_x1_l").boundary_metabolite
x1_b
```

```
[10]:
```

```
'x1_b'
```

If a reaction is not a boundary reaction (i.e., `MassReaction.boundary==False`) then `None` will be returned:

```
[11]:
```

```
print(model.reactions.get_by_id("R_Ytr").boundary_metabolite)
```

```
None
```

The `boundary_metabolite` attribute is useful for getting and setting values in the `MassModel.boundary_conditions` attribute.

```
[12]:
```

```
model.boundary_conditions[x1_b] = 2
model.boundary_conditions
```

```
[12]:
```

```
{'x1_b': 2}
```

To change the ‘boundary’ compartment identifier and name, a `dict` is passed to the `MassConfiguration.boundary_compartment` attribute setter:

```
[13]:
```

```
print("Before: {0}\n{1}".format(mass_config.boundary_compartment, model.boundary_metabolites))
mass_config.boundary_compartment = {"xt": "External compartment"}
print("\nAfter: {0}\n{1}".format(mass_config.boundary_compartment, model.boundary_metabolites))
```

```
Before: {'b': 'boundary'}
['x1_b', 'x5_b', 'y1_b', 'y4_b', 'z1_b', 'z2_b']

After: {'xt': 'External compartment'}
['x1_xt', 'x5_xt', 'y1_xt', 'y4_xt', 'z1_xt', 'z2_xt']
```

The “boundary” compartment is automatically assumed to have a volume of 1, and therefore is not factored in the rate laws. It is also ignored by the `MassModel.compartments` attribute, even when explicitly set:

```
[14]:
```

```
for r in model.sinks:
    print("{0}: {1}".format(r.id, r.get_mass_action_rate()))
model.compartments = {"xt": "External compartment"}
model.compartments
```

```
SK_y1_m: kf_SK_y1_m*y1_xt
SK_z1_s: kf_SK_z1_s*z1_xt
```

```
[14]:
```

```
{'l': 'the large compartment',
 'm': 'the medium compartment',
 's': 'the small compartment'}
```

See the section on For compartments and SBML in the Global Configuration tutorial for more information about the `boundary_compartment` attribute.

The ‘boundary’ pseudo-compartment and ‘boundary’ pseudo-metabolites are designed to make working with boundary conditions convenient at the cost of having finer user control. This primarily useful for \* Setting functions as boundary conditions (e.g., an oscillating function for external oxygen concentration) \* Using custom rates to set fixed inputs, causing irrelevant boundary conditions to be ignored altogether.

However, for finer control over external compartment and boundary conditions (and general best practices for SBML compatibility in MASSpy), it is recommended to (1) create new `MassMetabolite` objects, define their `compartment` and `initial_condition` attributes, (2) set the `fixed` attribute as `True`, and (3) add the metabolites to the appropriate reactions. This ensures the concentration of the metabolite is fixed at a constant value, and that its initial condition value is treated
as a boundary condition.

###### Fixed inputs¶

To bypass using the ‘boundary’ pseudo-compartment, it is recommended to set a fixed input using a custom rate law:

```
[15]:
```

```
for r in model.reactions.get_by_any(["SK_x1_l", "SK_y1_m", "SK_z1_s"]):
    model.add_custom_rate(r, custom_rate=r.kf_str)
    print("{0}: {1}".format(r.id, r.rate))
```

```
SK_x1_l: kf_SK_x1_l
SK_y1_m: kf_SK_y1_m
SK_z1_s: kf_SK_z1_s
```

#### Getting and setting compartment volumes¶

Support for compartment volumes is currently through the `MassModel.custom_parameters` attribute. To view what compartment volumes are set:

```
[16]:
```

```
def volume_filter(parameter):
    if str(parameter).startswith("volume"):
        return True
    return False

for vol_id in filter(volume_filter, model.custom_parameters):
    print("{0}: {1}".format(vol_id, model.custom_parameters[vol_id]))
```

```
volume_l: 10.0
volume_m: 5.0
volume_s: 1.0
```

To set or change a compartment volume, the value in the `MassModel.custom_parameters` dict is set using the volume parameter ID as the key:

```
[17]:
```

```
# Set the large compartment volume to 15
model.custom_parameters["volume_l"] = 15

# Double current medium compartment volume
model.custom_parameters["volume_m"] = model.custom_parameters["volume_m"] * 2

# 10% decrease to current small compartment volume
model.custom_parameters["volume_s"] = model.custom_parameters["volume_s"] * (1 + (-10/100))

for vol_id in filter(volume_filter, model.custom_parameters):
    print("{0}: {1}".format(vol_id, model.custom_parameters[vol_id]))
```

```
volume_l: 15
volume_m: 10.0
volume_s: 0.9
```

#### Simulating with Volumes and Multiple Compartments¶

Using a newly loaded model, the following section provides guidance on dynamic simulations for models with multiple compartments and includes examples on perturbing compartment volume.

```
[18]:
```

```
# Ensure compartments are active and boundary compartment is reset
mass_config.exclude_compartment_volumes_in_rates = False
mass_config.boundary_compartment = {'b': 'boundary'}

# Start with a fresh model, checking to ensure compartment volumes are reset
model = create_test_model("MultiCompartment")
for vol_id in filter(volume_filter, model.custom_parameters):
    print("{0}: {1}".format(vol_id, model.custom_parameters[vol_id]))
```

```
volume_l: 10.0
volume_m: 5.0
volume_s: 1.0
```

As always, a model must first must be loaded into the `mass.Simulation`. object in order to run a simulation. A quick simulation shows that the model is already at a steady state:

```
[19]:
```

```
simulation = Simulation(model, verbose=True)
conc_sol = simulation.simulate(model, time=(0, 1000))[0]
plot_time_profile(conc_sol, plot_function="loglog", legend="right outside")
```

```
Successfully loaded MassModel 'MultiCompartment' into RoadRunner.
```

```
[19]:
```

```
<matplotlib.axes._subplots.AxesSubplot at 0x7ffd39f97f50>
```

A volume parameter can be perturbed just like any other parameter using a `dict`. For example, suppose volume of the large compartment `volume_m` lost 40% of its volume:

```
[20]:
```

```
conc_sol = simulation.simulate(model, time=(0, 1000), perturbations={
    "volume_m": "volume_m * (1 - 0.4)",
})[0]
plot_time_profile(conc_sol, plot_function="loglog", legend="right outside")
```

```
[20]:
```

```
<matplotlib.axes._subplots.AxesSubplot at 0x7ffd3ac54510>
```

Note that in the above simulation, several of the metabolite concentrations in the large compartment changed. The `observable` argument can be used with the `MassMetabolite.compartment` attribute to look at metabolites in a specific compartment for futher examination

```
[21]:
```

```
plot_time_profile(conc_sol, observable=list(model.metabolites.query(lambda m: m.compartment == "m")),
                  plot_function="semilogx", legend="right outside")
```

```
[21]:
```

```
<matplotlib.axes._subplots.AxesSubplot at 0x7ffd3bab9490>
```

Multiple volumes also can be perturbed simultaneously. For example, suppose 1 mL of fluid from the `large` compartment was transfered to the `small` compartment, while 1.5 mL was transfered to the `medium` compartment:

```
[22]:
```

```
conc_sol = simulation.simulate(model, time=(0, 1000), perturbations={
    "volume_l": "volume_l - 2.5",
    "volume_m": "volume_m + 1.5",
    "volume_s": "volume_s + 1.0"
})[0]
plot_time_profile(conc_sol, plot_function="loglog", legend="right outside")
```

```
[22]:
```

```
<matplotlib.axes._subplots.AxesSubplot at 0x7ffd3ae99310>
```

Helpful tips: When enabling compartment volumes, it is up to the user to track their units to ensure that no numerical consistency issues arise. To make this a bit easier, be aware of the following MASSpy expectations and behaviors:

- When compartment volumes are disabled, MASSpy expects that volumes are already factored into initial condition values, and therefore considers values to be initial concentrations. Consequently, metabolite solutions returned by solutions will be for metabolite concentrations (e.g., mol/L, g/cDW)
- When compartment volumes are enabled, MASSpy expects that volumes have not been factored factored into initial condition values, and therefore considers values to be initial amounts. Consequently, metabolite solutions returned by solutions will be for metabolite amounts (e.g., mol, grams)

### Import and Export of Optimization Problems¶

This notebook demonstrates how an optimization problem setup in **MASSpy** can be exported for use with other optimization solvers. This notebook is based on the Optlang API documentation and the COBRApy FAQ How do I generate an LP file from a COBRA model?

Variables, constraints, objectives, and a name (if provided) are imported/exported through this method; however, solver configuration options are not.

```
[1]:
```

```
try:
    import simplejson as json
except ImportError:
    import json

import cobra
from optlang import Model as OptModel

import mass.test
# Print list of available solvers
print(list(cobra.util.solver.solvers))
```

```
['cplex', 'glpk_exact', 'glpk', 'gurobi', 'scipy']
```

#### Using Optlang¶

To facilitate the formation of the mathematical optimization problem, **MASSpy** utilizes the Optlang python package [JCS17]. As stated in the documentation:

1. Optlang provides a common interface to a series of optimization tools, so different solver backends can be changed in a transparent way.
2. Optlang takes advantage of the symbolic math library SymPy to allow objective functions and constraints to be easily formulated from symbolic expressions of variables.
3. Optlang interfaces with all solvers through importable python modules (read more here).

The following optimization solvers are supported:

- GLPK (LP/MILP; via swiglpk)
- CPLEX (LP/MILP/QP)
- Gurobi (LP/MILP/QP)

However, there are times where it would be preferrable to utilize other solvers and/or change programming environments in the process of setting up and performing optimizations. Fortunately, **Optlang** provides class methods for importing and exporting the optimization problem in both LP and JSON-compatible formats. The examples below demonstrate how the JSON format is utilized with **MASSpy** objects to facilitate the transference of optimization problems.

It is generally NOT recommended to import optimization problems directly into the solvers, as the corresponding MASSpy objects are bypassed and therefore do not have any values updated to match the new state of the solver.

##### Importing and Exporting with LP files¶

LP formulations of models can be used in conjunction with **Optlang** facilitate the exchange of optimization problems. Note the following:

1. Importing and exporting using LP formulations can change variable and constraint identifiers
2. LP formulations **do not** work with the `scipy` solver interface.

```
[2]:
```

```
# Start with a fresh model
model = mass.test.create_test_model("textbook")
# Change the bounds for demonstration purposes
model.variables.HEX1.lb, model.variables.HEX1.ub = (-123, 456)
print(model.variables["HEX1"])
```

```
-123 <= HEX1 <= 456
```

##### Exporting an LP file¶

For all solver interfaces in **Optlang**, the `str` representation of an `optlang.interface.Model` is the LP formulation of the problem.

To export the optimization problem into a file:

```
[3]:
```

```
with open("problem.lp", "w") as file:
    file.write(str(model.solver))
```

Alternatively, the `optlang.interface.Model.to_lp()` method can be used, but note that variable and constraint identifiers may be changed.

##### Importing an LP file¶

The `optlang.interface.Model.from_lp()` method can be used to import an LP formulation of an optimization problem.

```
[4]:
```

```
# Use new model to demonstrate how bounds change
model = mass.test.create_test_model("textbook")
print("Before: " + str(model.variables["HEX1"]))

# Load problem from JSON file
with open("problem.lp") as file:
    model._solver = OptModel.from_lp(file.read())
print("After: " + str(model.variables["HEX1"]))
```

```
Before: 0 <= HEX1 <= 1000.0
After: -123.0 <= HEX1 <= 456.0
```

##### Importing and Exporting with JSON files¶

```
[5]:
```

```
# Start with a fresh model
model = mass.test.create_test_model("textbook")
# Change the bounds for demonstration purposes
model.variables.HEX1.lb, model.variables.HEX1.ub = (-654, 321)
print(model.variables.HEX1)
```

```
-654 <= HEX1 <= 321
```

###### Exporting using JSON¶

Problems formulated in **Optlang** can be exported using the optlang.interface.Model.to\_json class method. First, the `to_json` class method exports a JSON compatible `dict` containing the variables, constraints, objectives, and an optional name from the `optlang.interface.Model`. The `dict` is then passed to json.dump to save the
optimization problem as a JSON file.

To export the optimization problem into a file:

```
[6]:
```

```
with open("problem.json", "w") as file:
    json.dump(model.solver.to_json(), file)
```

###### Importing using JSON¶

Problems can be imported into **Optlang** using the optlang.interface.Model.from\_json class method. First, a JSON compatible `dict` is loaded from a file using the json.load. The `dict` is then passed to the `from_json` class method to load the variables, constraints, objectives, and an optional name into the `optlang.interface.Model`
(imported as “OptModel” in this example”).

To import the optimization problem from a file:

```
[7]:
```

```
# Use new model to demonstrate how bounds change
model = mass.test.create_test_model("textbook")
print("Before: " + str(model.variables.HEX1))

# Load problem from JSON file
with open("problem.json") as file:
    model._solver = OptModel.from_json(json.load(file))
print("After: " + str(model.variables.HEX1))
```

```
Before: 0 <= HEX1 <= 1000.0
After: -654 <= HEX1 <= 321
```

##### Adding a solver interface¶

For an optimization solver that does not currently have an interface, consider adding a solver interface.

## Example Gallery¶

Interested in seeing more of **MASSpy** in action? Browse through the Gallery.

Various examples of MASSpy are provided below.

### Visualization¶

The following are additional examples demonstrating how to generate various types of visualizations using MASSpy.

#### Animating Simulations with Escher¶

This example shows how simulation results can be displayed on an escher map. Using the reactive options of Escher, the results of a simulation can also be animated.

For this particular example, a simple toy model is used.

```
[1]:
```

```
from os import path
from time import sleep

from matplotlib.cm import get_cmap
import numpy as np

import escher

from mass import MassConfiguration, Simulation
from mass.test import create_test_model, MAPS_DIR


mass_config = MassConfiguration()
mass_config.decimal_precision = 8
# Turns off the warning message when leaving or refreshing this page.
escher.rc['never_ask_before_quit'] = True
```

##### Load Model and Map¶

```
[2]:
```

```
model = create_test_model("Simple_Toy")

# Set the path to the map file
map_filepath = path.join(MAPS_DIR, "simple_toy_map.json")
```

##### Make color scales¶

###### Metabolites¶

```
[3]:
```

```
num_scales = 6
min_value, max_value = (0, 1)
min_size, max_size = (10, 30)
colors = 255 * get_cmap("bwr", num_scales)(np.linspace(0, 1, num_scales))
colors = np.flip(colors, axis=0)

metabolite_data_scale = []
for i in range(num_scales):
    scale_stop = {}
    scale_stop["type"] = "value"
    if i == 0:
        scale_stop["value"] = min_value
        scale_stop["size"] = min_size
    elif i == num_scales - 1:
        scale_stop["value"] = max_value
        scale_stop["size"] = max_size
    else:
        scale_stop["value"] = i*(max_value - min_value)/(num_scales - 1)
        scale_stop["size"] = min_size + i * (max_size - min_size)/(num_scales - 1)
    scale_stop["color"] = "rgb({0}, {1}, {2})".format(*colors[i])
    metabolite_data_scale.append(scale_stop)
```

###### Reactions¶

```
[4]:
```

```
stops = ["min", "Q1", "median", "Q3", "max"]
num_scales = len(stops)
min_value, max_value = (0, 1)
min_size, max_size = (10, 30)
colors = 255 * get_cmap("Purples", num_scales)(np.linspace(0, 1, num_scales))

reaction_data_scale = []
for i, stop_type in enumerate(stops):
    scale_stop = {}
    scale_stop["type"] = stop_type
    scale_stop["color"] = "rgb({0}, {1}, {2})".format(*colors[i])
    scale_stop["size"] = min_size + i * (max_size - min_size)/(num_scales - 1)
    reaction_data_scale.append(scale_stop)
reaction_data_scale.append(
    {"type": "value", "value": 0, "color": "grey", "size": 8})
```

##### Animate Simulation¶

###### Simulate model¶

```
[5]:
```

```
sim = Simulation(model, verbose=True)
conc_sol, flux_sol = sim.simulate(model, time=(0, 1e6),
                                  decimal_precision=True)
```

```
WARNING: No compartments found in model. Therefore creating compartment 'compartment' for entire model.
```

```
Successfully loaded MassModel 'Simple_Toy' into RoadRunner.
```

###### Render map¶

```
[6]:
```

```
escher_builder = escher.Builder(
    model=model,
    map_json=map_filepath,
    reaction_scale=tuple(reaction_data_scale),
    metabolite_scale=tuple(metabolite_data_scale)
)
escher_builder
```

###### Animate results¶

```
[7]:
```

```
for i in range(len(conc_sol.time)):
    # Map metabolite data onto map per solution point
    escher_builder.metabolite_data = {
        met: solution[i] for met, solution in conc_sol.items()}
    # Map reaction data onto map per solution point
    escher_builder.reaction_data = {
        rxn: solution[i] for rxn, solution in flux_sol.items()}
    sleep(0.05)
```

The video of the created animation:

```
[8]:
```

```
from IPython.display import Video
Video("../media/escher_toy_animation.mp4", embed=True, width=500)
```

```
[8]:
```

[

Your browser does not support the video tag.

#### Visualizing Catalytic Potentials of Glycolytic Regulatory Kinases¶

This notebook provides two examples on creating more sophisticated figures using MASSpy. Specifically, the two examples will reproduce the following from the publication by [YAHP18] - Example **A**: Reproduce Figure 2 of main text. - Example **B**: Reproduce Figure 4 of main text.

```
[1]:
```

```
from os import path

import matplotlib.pyplot as plt
from matplotlib.patches import Patch

from mass import Simulation
from mass.test import create_test_model
from mass.util import strip_time
from mass.visualization import plot_phase_portrait, plot_time_profile
```

All models for this example have already been constructed based on [YAHP18] and can be found here.

##### Example A: Phase Portraits of Catalytic Potentials for Individual Enzymes¶

In this example, the three key regulatory kinases of glycolysis are integrated into the glycolytic network as enzyme modules.

Specifically, this notebook example focuses on visualizing the catalytic potential of each individual enzyme when only one enzyme module are integrated into the network in order to reproduce Figure 2 of [YAHP18]

###### A1: All in one¶

The following cell reproduces Figure 2 of [YAHP18]

```
[2]:
```

```
# Create list of model IDs
model_ids = ["Glycolysis_Hb_PFK",
             "Glycolysis_Hb_HEX1",
             "Glycolysis_Hb_PYK"]
# Define perturbations
perturbations = [
    {"kf_ATPM": "kf_ATPM * 0.85"},
    {"kf_ATPM": "kf_ATPM * 1.50"}]
# Define colors and legend labels
colors = ["red", "blue"]
labels = ["15% decrease", "50 increase"]

# Define axes limits
xy_limits = {
    "PFK":  [(0.67, 0.98), (0.84, 1.01)],
    "HEX1": [(0.65, 1.00), (0.78, 0.89)],
    "PYK":  [(0.60, 1.00), (0.15, 0.88)]}

# Define helper functions to make aggregate solutions
def make_energy_charge_solution(conc_sol):
    conc_sol.make_aggregate_solution(
        aggregate_id="energy_charge",
        equation="(atp_c + 0.5 * adp_c) / (atp_c + adp_c + amp_c)",
        variables=["atp_c", "adp_c", "amp_c"], update=True)

def make_active_fraction_solution(model, conc_sol, enzyme):
    enzyme = model.enzyme_modules.get_by_id(enzyme)
    active = enzyme.enzyme_module_forms_categorized.get_by_id(
        enzyme.id + "_Active")
    conc_sol.make_aggregate_solution(
        aggregate_id="active_fraction",
        equation="({0}) / ({1})".format(
            " + ".join([e.id for e in active.members]),
             str(strip_time(enzyme.enzyme_concentration_total_equation))),
        variables=enzyme.enzyme_module_forms, update=True)

# Create figure and flatten axes into a list
fig, axes = plt.subplots(nrows=1, ncols=3, figsize=(15, 5))
axes = axes.flatten()
# Create inset axes
ax_inset = axes[-1].inset_axes([0.1, 0.7, 0.4, 0.25])
ax_inset.tick_params(labelbottom=False, labelleft=False)

# Iterate through models and axes instances
for i, (model_id, ax) in enumerate(zip(model_ids, axes)):
    # Load model and simulation
    model = create_test_model(model_id + ".json")
    sim = Simulation(model, verbose=True)

    # Get ID of enzyme module in model
    enzyme_id = model.id.split("_")[-1]

    # Ensure models are in steady state and make aggregate solutions
    conc_sol, flux_sol = sim.find_steady_state(
        model, strategy="simulate", update_values=True, tfinal=1e4)
    make_energy_charge_solution(conc_sol)
    make_active_fraction_solution(model, conc_sol, enzyme_id)

    # Plot steady state lines
    ax.plot([conc_sol["energy_charge"]] * 2, [-0.1, 1.1],
            color="grey", linestyle="--")
    ax.plot([-0.1, 1.1], [conc_sol["active_fraction"]] * 2,
            color="grey", linestyle="--")

    # Initialize legend arguments
    legend = None
    time_points_legend = None
    # Iterate through ATP perturbations
    for j, perturbation in enumerate(perturbations):
        # Simulate model with perturbation and make aggregate solutions
        conc_sol, flux_sol = sim.simulate(
            model, time=(0, 1000), perturbations=perturbation)
        make_energy_charge_solution(conc_sol)
        make_active_fraction_solution(model, conc_sol, enzyme_id)

        # Set legends on the middle plot only
        if i == 1:
            legend = (labels[j] + " in ATP utilization", "lower outside")
            time_points_legend = "upper outside"

        # Get x and y axes limits
        xlimits, ylimits = xy_limits[enzyme_id]

        # Make phase portrait
        plot_phase_portrait(
            conc_sol, x="energy_charge", y="active_fraction",
            ax=ax, legend=legend,                  # Set axes and legend
            xlim=xlimits, ylim=ylimits,            # Axes limits
            xlabel="Energy Charge (E.C.)",         # Axes labels
            ylabel=enzyme_id + " Active Fraction ($f_{A}$)",
            title=(model.id, {"size": "x-large"}), # Title
            linestyle="--", color=colors[j],       # Line color and style
            annotate_time_points="endpoints",      # Annotate time points
            annotate_time_points_color="black",
            annotate_time_points_legend=time_points_legend)

        # Plot time profile of FDP on the inset axes
        if i == 2:
            # Plot FDP time profile
            plot_time_profile(
                conc_sol, observable="fdp_c", ax=ax_inset,
                plot_function="semilogx",
                xlim=(1e-6, 1000), ylim=(0.002, 0.016),
                xlabel="Time (hours)", ylabel="[FDP] (mM)",
                color=colors[j])
```

```
Successfully loaded MassModel 'Glycolysis_Hb_PFK' into RoadRunner.
Successfully loaded MassModel 'Glycolysis_Hb_HEX1' into RoadRunner.
Successfully loaded MassModel 'Glycolysis_Hb_PYK' into RoadRunner.
```

###### A2: Steps to Reproduce¶

In this section, the steps for reproducing Figure 2 of [YAHP18] are outlined below in an expanded workflow.

###### A2.1: Setup¶

The first step is to load the models and ensure they exist in a steady state.

```
[3]:
```

```
models_and_simulations = {}
for model_id in ["Glycolysis_Hb_PFK",
                 "Glycolysis_Hb_HEX1",
                 "Glycolysis_Hb_PYK"]:
    model = create_test_model(model_id + ".json")
    simulation = Simulation(model, verbose=True)
    simulation.find_steady_state(model, strategy="simulate",
                                 update_values=True, tfinal=1e4)
    models_and_simulations[model] = simulation
```

```
Successfully loaded MassModel 'Glycolysis_Hb_PFK' into RoadRunner.
Successfully loaded MassModel 'Glycolysis_Hb_HEX1' into RoadRunner.
Successfully loaded MassModel 'Glycolysis_Hb_PYK' into RoadRunner.
```

When the `EnzymeModule` objects were created, the active forms of the enzyme was categorized and placed in a group. This will make creating the solution for the active fraction of the enzyme significantly easier.

```
[4]:
```

```
for model in models_and_simulations:
    # Get the ID of the enzyme module
    e_mod = model.id.split("_")[-1]
    # Get the enzyme module
    e_mod = model.enzyme_modules.get_by_id(e_mod)
    # Get the active group from the categorized enzyme module forms
    active = e_mod.enzyme_module_forms_categorized.get_by_id(
        "_".join((e_mod.id, "Active")))

    print("Enzyme: " + e_mod.id)
    print("Group ID: " + active.id)
    print("# of members: {0}\n".format(len(active.members)))
```

```
Enzyme: PFK
Group ID: PFK_Active
# of members: 15

Enzyme: HEX1
Group ID: HEX1_Active
# of members: 4

Enzyme: PYK
Group ID: PYK_Active
# of members: 20
```

Because aggregate solutions for the adenylate energy charge and enzyme active fractions will need to be created multiple times, smaller “helper” functions are defined to facilate this process. A list containing enzyme IDs is also defined in the order in which the models were stored.

```
[5]:
```

```
def make_energy_charge_solution(conc_sol):
    conc_sol.make_aggregate_solution(
        aggregate_id="energy_charge",
        equation="(atp_c + 0.5 * adp_c) / (atp_c + adp_c + amp_c)",
        variables=["atp_c", "adp_c", "amp_c"], update=True)
    return

def make_active_fraction_solution(model, conc_sol, enzyme_id):
    # Get the relevant enzyme module
    e_mod = model.enzyme_modules.get_by_id(enzyme_id)

    # Get active group
    active = e_mod.enzyme_module_forms_categorized.get_by_id(
        enzyme_id + "_Active")

    # Create string representing sum of active enzyme forms
    active_eq = " + ".join([e.id for e in active.members])

    # Create string representing sum of all enzyme forms
    total_eq  = str(strip_time(e_mod.enzyme_concentration_total_equation))

    # Make aggregate solution
    conc_sol.make_aggregate_solution(
        aggregate_id="active_fraction",
        equation="({0}) / ({1})".format(active_eq, total_eq),
        variables=e_mod.enzyme_module_forms, update=True)
    return

enzyme_ids = [model.id.split("_")[-1] for model in models_and_simulations]
enzyme_ids
```

```
[5]:
```

```
['PFK', 'HEX1', 'PYK']
```

The figure and axes instances are created using **matplotlib**. The figure size is set as 15 x 5 to create three square plots in a single row. Each plot will be have a size of 5 x 5.

```
[6]:
```

```
fig, axes = plt.subplots(nrows=1, ncols=3, figsize=(15, 5))
axes = axes.flatten()
```

Lists containing tuples for the x-axis and y-axis limits are also created. They are placed in a dictionary where the enzyme module IDs are the `dict` keys.

```
[7]:
```

```
# Values are formatted as [(xmin, xmax), (ymin, ymax)]
xy_limits = {
    "PFK":  [(0.67, 0.98), (0.84, 1.01)],
    "HEX1": [(0.65, 1.00), (0.78, 0.89)],
    "PYK":  [(0.60, 1.00), (0.15, 0.88)]}
```

###### A2.2: Plot steady state lines¶

The steady state values are plotted as straight lines to visually indicate where the steady state lies.

```
[8]:
```

```
for i, (model, sim) in enumerate(models_and_simulations.items()):
    # Get axes to plot on
    ax = axes[i]
    # Get ID of enzyme module in model
    enzyme_id = enzyme_ids[i]
    # Get MassSolution objects containing steady state values
    conc_sol_ss, flux_sol_ss = sim.find_steady_state(
        model, strategy="simulate", update_values=True, tfinal=1e4)

    # Make an aggregate solution for the energy charge
    make_energy_charge_solution(conc_sol_ss)
    # Plot a vertical line at the steady steady value
    ax.plot([conc_sol_ss["energy_charge"]] * 2,
            [-0.1, 1.1],
            color="grey", linestyle="--")

    # Make an aggregate solution for the active fraction
    make_active_fraction_solution(model, conc_sol_ss, enzyme_id)
    # Plot a horizontal line at the steady steady value
    ax.plot([-0.1, 1.1],
            [conc_sol_ss["active_fraction"]] * 2,
            color="grey", linestyle="--")
fig
```

```
[8]:
```

###### A2.3: Plot catalytic potentials for 15% decrease in ATP utilization¶

The first set of results to plot on the figure are the results obtained from simulating the models with a 15% decrease in ATP utilization. This behavior is mimicked by perturbing the rate constant for ATP use to be 0.85 times its original value. The color “red” will be used to represent these results.

Legends are also created for perturbations and the annotated time points, placed below and above the middle plot, respectively.

```
[9]:
```

```
for i, (model, sim) in enumerate(models_and_simulations.items()):
    # Get axes to plot on
    ax = axes[i]
    # Get ID of enzyme module in model
    enzyme_id = enzyme_ids[i]
    # Simulate model with a 50% increase in ATP utilization
    conc_sol, flux_sol = sim.simulate(
        model, time=(0, 1000),
        perturbations={"kf_ATPM": "kf_ATPM * 1.5"});

    # Make an aggregate solution for the energy charge
    make_energy_charge_solution(conc_sol)

    # Make an aggregate solution for the active fraction
    make_active_fraction_solution(model, conc_sol, enzyme_id)

    # Place legend on middle plot only
    if i == 1:
        legend = ("50% increase in ATP utilization", "lower outside")
        time_points_legend = "upper outside"
    else:
        legend = None
        time_points_legend = None

    # Get x and y axes limits
    xlimits, ylimits = xy_limits[enzyme_id]
    # Make phase portrait
    plot_phase_portrait(
        conc_sol, x="energy_charge", y="active_fraction",
        ax=ax, legend=legend,                  # Axes instance and legend
        xlim=xlimits, ylim=ylimits,            # Axes limits
        xlabel="Energy Charge (E.C.)",         # Axes labels
        ylabel=enzyme_id + " Active Fraction ($f_{A}$)",
        title=(model.id, {"size": "x-large"}), # Title
        linestyle="--", color="blue",          # Line color and style
        annotate_time_points="endpoints",      # Annotate time points
        annotate_time_points_color="black",
        annotate_time_points_legend=time_points_legend,
    )
fig
```

```
[9]:
```

###### A2.4: Plot catalytic potentials for 50% increase in ATP utilization¶

The second set of results to plot on the figure are the results obtained from simulating the models with a 50% increase in ATP utilization. This behavior is mimicked by perturbing the rate constant for ATP use to be 1.5 times its original value. The color “blue” will be used to represent these results.

```
[10]:
```

```
for i, (model, sim) in enumerate(models_and_simulations.items()):
    # Get axes to plot on
    ax = axes[i]
    # Get ID of enzyme module in model
    enzyme_id = enzyme_ids[i]

    # Simulate model with a 50% increase in ATP utilization
    conc_sol, flux_sol = sim.simulate(
        model, time=(0, 1000),
        perturbations={"kf_ATPM": "kf_ATPM * 0.85"});

    # Make an aggregate solution for the energy charge
    make_energy_charge_solution(conc_sol)

    # Make an aggregate solution for the active fraction
    make_active_fraction_solution(model, conc_sol, enzyme_id)

    # Place legend on middle plot only
    if i == 1:
        legend = ("15% decrease in ATP utilization", "lower outside")
        time_points_legend = "upper outside"
    else:
        legend = None
        time_points_legend = None
    # Get x and y axes limits
    xlimits, ylimits = xy_limits[enzyme_id]
    # Make phase portrait
    plot_phase_portrait(
        conc_sol, x="energy_charge", y="active_fraction",
        ax=ax, legend=legend,                  # Axes instance and legend
        xlim=xlimits, ylim=ylimits,            # Axes limits
        xlabel="Energy Charge (E.C.)",         # Axes labels
        ylabel=enzyme_id + " Active Fraction ($f_{A}$)",
        title=(model.id, {"size": "x-large"}), # Title
        linestyle="--", color="red",           # Line color and style
        annotate_time_points="endpoints",      # Annotate time points
        annotate_time_points_color="black",
        annotate_time_points_legend=time_points_legend,
    )
fig
```

```
[10]:
```

###### A2.5: Time profile of FDP concentration¶

The plot of FDP concentration in the upper left quadrant of the plot for the catalytic potential of PYK is created.

First, the inset axes instance is created and the axes tick labels are removed:

```
[11]:
```

```
# Get the axes instance
right_ax = axes[-1]
ax_inset = right_ax.inset_axes([0.1, 0.7, 0.4, 0.25])
ax_inset.tick_params(labelbottom=False, labelleft=False)
fig
```

```
[11]:
```

The model with the PYK enzyme module is simulated with a 15% decrease in ATP utilization, and the results are plotted.

```
[12]:
```

```
for model, sim in models_and_simulations.items():
    # Only interested in the third model
    if model.id != "Glycolysis_Hb_PYK":
        continue
    # Simulate model with a 50% increase in ATP utilization
    conc_sol, flux_sol = sim.simulate(
        model, time=(0, 1000),
        perturbations={"kf_ATPM": "kf_ATPM * 0.85"});

    # Plot FDP time profile
    plot_time_profile(
        conc_sol, observable="fdp_c", ax=ax_inset,
        plot_function="semilogx",
        xlim=(1e-6, 1000), ylim=(0.002, 0.016),
        xlabel=("Time (hours)", {"size": "large"}),
        ylabel=("[FDP] (mM)", {"size": "large"}),
        color="red")
fig
```

```
[12]:
```

In a similar fashion, the results of simulating the model with a 50% increase in ATP utilization are plotted.

```
[13]:
```

```
for model, sim in models_and_simulations.items():
    # Only interested in the third model
    if model.id != "Glycolysis_Hb_PYK":
        continue
    # Simulate model with a 50% increase in ATP utilization
    conc_sol, flux_sol = sim.simulate(
        model, time=(0, 1000),
        perturbations={"kf_ATPM": "kf_ATPM * 1.5"});

    # Plot FDP time profile
    plot_time_profile(
        conc_sol, observable="fdp_c", ax=ax_inset,
        plot_function="semilogx",
        xlim=(1e-6, 1000), ylim=(0.002, 0.016),
        xlabel=("Time (hours)", {"size": "large"}),
        ylabel=("[FDP] (mM)", {"size": "large"}),
        color="blue")
fig
```

```
[13]:
```

Figure 2 of [YAHP18] has been reproduced.

##### Example B: Phase Portraits of Catalytic Potentials and Interplay Among Enzymes¶

In this example, the three key regulatory kinases of glycolysis are integrated into the glycolytic network as enzyme modules.

Specifically, this notebook example focuses on visualizing the catalytic potentials of enzymes as well as the interplay among enzymes when all three enzyme modules are integrated into the network in order to reproduce Figure 4 of [YAHP18].

###### B1: All in one¶

The following cell reproduces Figure 4 of [YAHP18].

```
[14]:
```

```
# Load model and simulation
model = create_test_model("Glycolysis_FKRM.json")
simulation = Simulation(model, verbose=True)

# Define perturbations
perturbation_list = [
    {"kf_ATPM": "kf_ATPM * 0.85"},
    {"kf_ATPM": "kf_ATPM * 1.50"}]
# Define colors and legend labels
colors = ["red", "blue"]
labels = ["15% decrease", "50 increase"]

# Define enzyme pairs and axes limits
enzyme_pairs = [("PFK", "HEX1"), ("PFK", "PYK"), ("HEX1", "PYK")]

# Values are formatted as [(xmin, xmax), (ymin, ymax)]
xy_limits = {
    "HEX1": ((0.68, 0.92), (0.77, 0.89)),
    "PFK":  ((0.68, 1.02), (0.70, 1.02)),
    "PYK":  ((0.68, 0.92), (0.35, 1.01)),
    ("PFK", "HEX1"): ((0.75, 1.02), (0.78, 0.89)),
    ("PFK", "PYK"):  ((0.75, 1.02), (0.38, 0.93)),
    ("HEX1", "PYK"): ((0.78, 0.89), (0.38, 0.93))}

# Define roman numerals and coordinates
roman_num_coords= {"I":   (0.95, 0.95), "II":  (0.05, 0.95),
                   "III": (0.05, 0.05), "IV":  (0.95, 0.05)}

# Define helper functions to make aggregate solutions
def make_energy_charge_solution(conc_sol):
    conc_sol.make_aggregate_solution(
        aggregate_id="energy_charge",
        equation="(atp_c + 0.5 * adp_c) / (atp_c + adp_c + amp_c)",
        variables=["atp_c", "adp_c", "amp_c"], update=True)

def make_active_fraction_solution(conc_sol, enzyme):
    active = enzyme.enzyme_module_forms_categorized.get_by_id(
        enzyme.id + "_Active")
    conc_sol.make_aggregate_solution(
        aggregate_id=enzyme.id + "_active_fraction",
        equation="({0}) / ({1})".format(
            " + ".join([e.id for e in active.members]),
            str(strip_time(enzyme.enzyme_concentration_total_equation))),
        variables=enzyme.enzyme_module_forms, update=True)

# Create figure and axes
fig, axes = plt.subplots(nrows=2, ncols=3, figsize=(15, 10))
# Get the rightmost axes instance on the top row
right_ax = axes[0][-1]
# Create inset axes
ax_inset = right_ax.inset_axes([0.18, 0.7, 0.37, 0.25])
# Remove tick labels
ax_inset.tick_params(labelbottom=False, labelleft=False)

# Simulate to steady state
conc_sol, flux_sol = simulation.find_steady_state(
    model, strategy="simulate", update_values=True, tfinal=1e4)
# Make aggregate solution for energy charge
make_energy_charge_solution(conc_sol)

# Iterate through enzyme modules and axes for the top half of figure
for enzyme, ax in zip(model.enzyme_modules, axes[0]):
    # Make aggregate solutions for the active fraction
    make_active_fraction_solution(conc_sol, enzyme)
    # Plot steady state lines for energy charge and active fraction
    ax.plot([conc_sol["energy_charge"]] * 2,
            [-0.1, 1.1],
            color="grey", linestyle="--")
    ax.plot([-0.1, 1.1],
            [conc_sol[enzyme.id + "_active_fraction"]] * 2,
            color="grey", linestyle="--")
    # Add roman numerals to the top axes.
    for roman_num, coords in roman_num_coords.items():
        ax.text(*coords, roman_num, fontsize=18, transform=ax.transAxes,
                horizontalalignment='center', verticalalignment='center')

# Iterate through perturbations and colors
for i, (perturbation, color) in enumerate(zip(perturbation_list, colors)):
    # Simulate model with ATP utilization perturbation
    conc_sol, flux_sol = simulation.simulate(
        model, time=(0, 1000), perturbations=perturbation)
    # Make aggregate solution for energy charge
    make_energy_charge_solution(conc_sol)

    # Iterate through enzyme modules and axes for the top half of figure
    for j, (enzyme, ax) in enumerate(zip(model.enzyme_modules, axes[0])):
         # Make aggregate solutions for the active fraction
        make_active_fraction_solution(conc_sol, enzyme)

        # Place time point legend on middle plot only
        if j == 1:
            time_points_legend = "upper outside"
        else:
            time_points_legend = None

        # Make phase portrait of catalytic potential for an enzyme
        plot_phase_portrait(
            conc_sol, x="energy_charge",
            y=enzyme.id + "_active_fraction", ax=ax,
            xlim=xy_limits[enzyme.id][0],          # Axes limits
            ylim=xy_limits[enzyme.id][1],
            xlabel="Energy Charge (E.C.)",         # Axes labels
            ylabel=enzyme.id + " Active Fraction ($f_{A}$)",
            linestyle="--", color=color,           # Line color and style
            annotate_time_points="endpoints",      # Annotate time points
            annotate_time_points_color="black",
            annotate_time_points_legend=time_points_legend)

        # Plot time profile of FDP on the inset axes
        if j == 2:
            # Plot FDP time profile
            plot_time_profile(
                conc_sol, observable="fdp_c", ax=ax_inset,
                plot_function="semilogx",
                xlim=(1e-6, 1000), ylim=(0.004, 0.018),
                xlabel=("Time (hours)", {"size": "large"}),
                ylabel=("[FDP] (mM)", {"size": "large"}),
                color=color)

    # Iterate through enzyme pairs and axes for bottom half of figure
    for j, (enzyme_pair, ax) in enumerate(zip(enzyme_pairs, axes[1])):
        # Place legend on middle plot only (PFK vs. PYK)
        if j == 1:
            legend = (labels[i] + " in ATP utilization",
                      "lower outside")
        else:
            legend = None
        # Get the enzyme for the x-axis and for the y-axis
        enz_x, enz_y = enzyme_pair
        plot_phase_portrait(
            conc_sol,
            x=enz_x + "_active_fraction", y=enz_y + "_active_fraction",
            ax=ax, legend=legend,              # Axes instance and legend
            xlim=xy_limits[enzyme_pair][0],    # Axes limits
            ylim=xy_limits[enzyme_pair][1],
            xlabel=enz_x + " Active Fraction ($f_{A}$)", # Axes labels
            ylabel=enz_y + " Active Fraction ($f_{A}$)",
            color=color, linestyle="--",       # Line color and style
            annotate_time_points="endpoints",  # Annotate time points
            annotate_time_points_color="black")
```

```
Successfully loaded MassModel 'Glycolysis_FKRM' into RoadRunner.
```

###### B2: Steps to Reproduce¶

In this section, the steps for reproducing Figure 4 of [YAHP18] are outlined below in an expanded workflow.

###### B2.1: Setup¶

The first step is to load the model and ensure it exists in a steady state.

```
[15]:
```

```
model = create_test_model("Glycolysis_FKRM.json")
simulation = Simulation(model, verbose=True)
simulation.find_steady_state(model, strategy="simulate",
                             update_values=True, tfinal=1e4)
```

```
Successfully loaded MassModel 'Glycolysis_FKRM' into RoadRunner.
```

```
[15]:
```

```
(<MassSolution Glycolysis_FKRM_ConcSols at 0x7fd2992d3530>,
 <MassSolution Glycolysis_FKRM_FluxSols at 0x7fd2994802f0>)
```

When the `EnzymeModule` objects were created, the active forms of the enzyme was categorized and placed in a group. This will make creating the solution for the active fraction of the enzyme significantly easier.

```
[16]:
```

```
for enzyme_module in model.enzyme_modules:

    active = enzyme_module.enzyme_module_forms_categorized.get_by_id(
        "_".join((enzyme_module.id, "Active")))

    print("Enzyme: " + enzyme_module.id)
    print("Group ID: " + active.id)
    print("# of members: {0}\n".format(len(active.members)))
```

```
Enzyme: HEX1
Group ID: HEX1_Active
# of members: 4

Enzyme: PFK
Group ID: PFK_Active
# of members: 15

Enzyme: PYK
Group ID: PYK_Active
# of members: 20
```

Because aggregate solutions for the adenylate energy charge and enzyme active fractions will need to be created multiple times, smaller “helper” functions are defined to facilate this process.

```
[17]:
```

```
def make_energy_charge_solution(conc_sol):
    conc_sol.make_aggregate_solution(
        aggregate_id="energy_charge",
        equation="(atp_c + 0.5 * adp_c) / (atp_c + adp_c + amp_c)",
        variables=["atp_c", "adp_c", "amp_c"], update=True)
    return

def make_active_fraction_solution(conc_sol, enzyme_module):
    # Get active group
    active = enzyme_module.enzyme_module_forms_categorized.get_by_id(
        enzyme_module.id + "_Active")

    # Create string representing sum of active enzyme forms
    active_eq = " + ".join([e.id for e in active.members])

    # Create string representing sum of all enzyme forms
    total_eq  = str(strip_time(
        enzyme_module.enzyme_concentration_total_equation))

    # Make aggregate solution
    conc_sol.make_aggregate_solution(
        aggregate_id=enzyme_module.id + "_active_fraction",
        equation="({0}) / ({1})".format(active_eq, total_eq),
        variables=enzyme_module.enzyme_module_forms, update=True)
    return
```

The figure and axes instances are created using **matplotlib**. The figure size is set as 15 x 10 to create three square plots in two rows for a total of 6 plots. Each plot will be have a size of 5 x 5.

```
[18]:
```

```
fig, axes = plt.subplots(nrows=2, ncols=3, figsize=(15, 10))
```

###### B2.2: Plot catalytic potential of enzymes¶

The following section demonstrates the necessary steps for generating the top half of the figure. The top half of the figure contains plots of the catalytic potentials for enzymes as a function of energy charge.

###### B2.2.1: Plot steady state lines¶

The steady state values are plotted as straight lines to visually indicate where the steady state lies.

```
[19]:
```

```
# Simulate to steady state
conc_sol_ss, flux_sol_ss = simulation.find_steady_state(
    model, strategy="simulate", update_values=True, tfinal=1e4)
# Make aggregate solutions for energy charge
make_energy_charge_solution(conc_sol_ss)

# Iterate through enzyme modules and axes for the top half of figure
for enzyme_module, ax in zip(model.enzyme_modules, axes[0]):
    # Make aggregate solutions for the active fraction
    make_active_fraction_solution(conc_sol_ss, enzyme_module)
    # Plot steady state lines for energy charge
    ax.plot([conc_sol_ss["energy_charge"]] * 2,
            [-0.1, 1.1],
            color="grey", linestyle="--")
    # Plot steady state lines for active fraction
    ax.plot([-0.1, 1.1],
            [conc_sol_ss[enzyme_module.id + "_active_fraction"]] * 2,
            color="grey", linestyle="--")
```

###### B2.2.2: Label quadrants with roman numerals¶

Roman numerals I-IV are added to the quandrants as follows: \* (I) more enzyme in active form and higher energy charge \* (II) more enzyme in active form and lower energy \* (III) more enzyme in inactive form and lower energy charge \* (IV) more enzyme in inactive form and higher energy charge

```
[20]:
```

```
# Define roman numerals and coordinates
roman_num_coords= {
    "I":   (0.95, 0.95),
    "II":  (0.05, 0.95),
    "III": (0.05, 0.05),
    "IV":  (0.95, 0.05)}
# Iterate through each axes
for ax in axes[0]:
    # Add each number to the current axes.
    for roman_num, coords in roman_num_coords.items():
        ax.text(*coords, roman_num, fontsize=18,
                horizontalalignment='center',
                verticalalignment='center',
                transform=ax.transAxes)
fig
```

```
[20]:
```

###### B2.2.3: Plot catalytic potentials for ATP utilization perturbations¶

After plotting the steady state lines and labeling the quadrants, the model is simulated with perturbations to the ATP utilization rate. These perturbations include a 15% decrease in the rate constant for ATP use and a 50% increase in the rate constant for ATP use, represented by the colors “red” and “blue” respectively.

A legend is also created for the annotated time points, placed above the top middle plot (PFK catalytic potential).

```
[21]:
```

```
# Values are formatted as [(xmin, xmax), (ymin, ymax)]
xy_limits = {
    "HEX1": [(0.68, 0.92), (0.77, 0.89)],
    "PFK":  [(0.68, 1.02), (0.70, 1.02)],
    "PYK":  [(0.68, 0.92), (0.35, 1.01)]}

# Iterate through perturbations and colors
perturbation_list = [
    {"kf_ATPM": "kf_ATPM * 0.85"},
    {"kf_ATPM": "kf_ATPM * 1.50"}]
colors = ["red", "blue"]
for perturbation, color in zip(perturbation_list, colors):
    # Simulate model with ATP utilization perturbation
    conc_sol, flux_sol = simulation.simulate(
        model, time=(0, 1000), perturbations=perturbation)

    # Make aggregate solutions for energy charge and active fraction
    make_energy_charge_solution(conc_sol)

    # Iterate through enzyme modules and axes for the top half of figure
    for enzyme_module, ax in zip(model.enzyme_modules, axes[0]):
        # Make aggregate solutions for the active fraction
        make_active_fraction_solution(conc_sol, enzyme_module)

        # Place time point legend on middle plot only (PFK)
        if enzyme_module.id == "PFK":
            time_points_legend = "upper outside"
        else:
            time_points_legend = None

        # Make phase portrait
        plot_phase_portrait(
            conc_sol, x="energy_charge",
            y=enzyme_module.id + "_active_fraction", ax=ax,
            xlim=xy_limits[enzyme_module.id][0],   # Axes limits
            ylim=xy_limits[enzyme_module.id][1],
            xlabel="Energy Charge (E.C.)",         # Axes labels
            ylabel=enzyme_module.id + " Active Fraction ($f_{A}$)",
            linestyle="--", color=color,           # Line color and style
            annotate_time_points="endpoints",      # Annotate time points
            annotate_time_points_color="black",
            annotate_time_points_legend=time_points_legend)
fig
```

```
[21]:
```

###### B2.2.4: Time profile for FDP concentration¶

The last step for creating the top half of the figure is to add the time profile of FDP concentration in the 2nd quadrant of the PYK catalytic potential plot.

```
[22]:
```

```
# Get the rightmost axes instance on the top row
right_ax = axes[0][-1]
# Create inset axes
ax_inset = right_ax.inset_axes([0.18, 0.7, 0.37, 0.25])
# Remove tick labels
ax_inset.tick_params(labelbottom=False, labelleft=False)

# Iterate through perturbations and colors
perturbation_list = [
    {"kf_ATPM": "kf_ATPM * 0.85"},
    {"kf_ATPM": "kf_ATPM * 1.50"}]
colors = ["red", "blue"]
for perturbation, color in zip(perturbation_list, colors):
    # Simulate model with ATP utilization perturbation
    conc_sol, flux_sol = simulation.simulate(
        model, time=(0, 1000), perturbations=perturbation)

    # Plot FDP time profile
    plot_time_profile(
        conc_sol, observable="fdp_c", ax=ax_inset,
        plot_function="semilogx",
        xlim=(1e-6, 1000), ylim=(0.004, 0.018),
        xlabel=("Time (hours)", {"size": "large"}),
        ylabel=("[FDP] (mM)", {"size": "large"}),
        color=color)
fig
```

```
[22]:
```

###### B2.3: Plot pairwise relationships of enzyme active fractions¶

The following section demonstrates the necessary steps for generating the bottom half of the figure. The bottom half of the figure contains plots of the pairwise relationships between the active fractions of two kinases.

A legend is also created for the different simulations performed, placed above the bottom middle plot (PFK vs. PYK active fractions).

```
[23]:
```

```
enzyme_pairs = [("PFK", "HEX1"), ("PFK", "PYK"), ("HEX1", "PYK")]
xy_limits = dict(zip(
    enzyme_pairs, [
        ((0.75, 1.02), (0.78, 0.89)),
        ((0.75, 1.02), (0.38, 0.93)),
        ((0.78, 0.89), (0.38, 0.93)),
    ]
))
labels = ["15% decrease", "50% increase"]
# Iterate through perturbations and colors
for i, (perturbation, color) in enumerate(zip(perturbation_list, colors)):
    # Simulate model with ATP utilization perturbation
    conc_sol, flux_sol = simulation.simulate(
        model, time=(0, 1000), perturbations=perturbation)

    # Make aggregate solutions for the active fractions
    for enzyme_module in model.enzyme_modules:
        make_active_fraction_solution(conc_sol, enzyme_module)

    # Iterate through enzyme pairs and axes for bottom half of figure
    for enzyme_pair, ax in zip(enzyme_pairs, axes[1]):

        # Place legend on middle plot only (PFK vs. PYK)
        if enzyme_pair == ("PFK", "PYK"):
            legend = (labels[i] + " in ATP utilization",
                      "lower outside")
        else:
            legend = None

        # Get the enzyme for the x-axis and for the y-axis
        enz_x, enz_y = enzyme_pair
        plot_phase_portrait(
            conc_sol,
            x=enz_x + "_active_fraction", y=enz_y + "_active_fraction",
            ax=ax, legend=legend,              # Axes instance and legend
            xlim=xy_limits[enzyme_pair][0],    # Axes limits
            ylim=xy_limits[enzyme_pair][1],
            xlabel=enz_x + " Active Fraction ($f_{A}$)", # Axes labels
            ylabel=enz_y + " Active Fraction ($f_{A}$)",
            color=color, linestyle="--",       # Line color and style
            annotate_time_points="endpoints",  # Annotate time points
            annotate_time_points_color="black")
fig
```

```
[23]:
```

Figure 4 of [YAHP18] has been reproduced.

### Workflows¶

The following are additional examples demonstrating how to generate various types of visualizations using MASSpy.

#### Constructing Glycolysis¶

Based on Chapter 10 of [Pal11]

To construct a model of glycolysis, first we import **MASSpy** and other essential packages. Constants used throughout the notebook are also defined.

```
[1]:
```

```
from os import path

import matplotlib.pyplot as plt

from cobra import DictList

from mass import (
    MassConfiguration, MassMetabolite, MassModel,
    MassReaction, Simulation, UnitDefinition)
from mass.io import json, sbml
from mass.util import qcqa_model

mass_config = MassConfiguration()
```

##### Model Construction¶

The first step of creating a model of glycolysis is to define the `MassModel`.

```
[2]:
```

```
glycolysis = MassModel("Glycolysis")
```

###### Metabolites¶

The next step is to define all of the metabolites using the `MassMetabolite` object. Some considerations for this step include the following:

1. It is important to use a clear and consistent format for identifiers and names when defining the `MassMetabolite` objects for various reasons, some of which include improvements to model clarity and utility, assurance of unique identifiers (required to add metabolites to the model), and consistency when collaborating and communicating with others.
2. In order to ensure our model is physiologically accurate, it is important to provide the `formula` argument with a string representing the chemical formula for each metabolite, and the `charge` argument with an integer representing the metabolite’s ionic charge (Note that neutrally charged metabolites are provided with 0). These attributes can always be set later if necessary using the `formula` and `charge` attribute set methods.
3. To indicate that the cytosol is the cellular compartment in which the reactions occur, the string “c” is provided to the `compartment` argument.

This model will be created using identifiers and names found in the BiGG Database.

In this model, there are 20 metabolites inside the cytosol compartment.

```
[3]:
```

```
glc__D_c = MassMetabolite(
    "glc__D_c",
    name="D-Glucose",
    formula="C6H12O6",
    charge=0,
    compartment="c",
    fixed=False)

g6p_c = MassMetabolite(
    "g6p_c",
    name="D-Glucose 6-phosphate",
    formula="C6H11O9P",
    charge=-2,
    compartment="c",
    fixed=False)

f6p_c = MassMetabolite(
    "f6p_c",
    name="D-Fructose 6-phosphate",
    formula="C6H11O9P",
    charge=-2,
    compartment="c",
    fixed=False)

fdp_c = MassMetabolite(
    "fdp_c",
    name="D-Fructose 1,6-bisphosphate",
    formula="C6H10O12P2",
    charge=-4,
    compartment="c",
    fixed=False)

dhap_c = MassMetabolite(
    "dhap_c",
    name="Dihydroxyacetone phosphate",
    formula="C3H5O6P",
    charge=-2,
    compartment="c",
    fixed=False)

g3p_c = MassMetabolite(
    "g3p_c",
    name="Glyceraldehyde 3-phosphate",
    formula="C3H5O6P",
    charge=-2,
    compartment="c",
    fixed=False)

_13dpg_c = MassMetabolite(
    "_13dpg_c",
    name="3-Phospho-D-glyceroyl phosphate",
    formula="C3H4O10P2",
    charge=-4,
    compartment="c",
    fixed=False)

_3pg_c = MassMetabolite(
    "_3pg_c",
    name="3-Phospho-D-glycerate",
    formula="C3H4O7P",
    charge=-3,
    compartment="c",
    fixed=False)

_2pg_c = MassMetabolite(
    "_2pg_c",
    name="D-Glycerate 2-phosphate",
    formula="C3H4O7P",
    charge=-3,
    compartment="c",
    fixed=False)

pep_c = MassMetabolite(
    "pep_c",
    name="Phosphoenolpyruvate",
    formula="C3H2O6P",
    charge=-3,
    compartment="c",
    fixed=False)

pyr_c = MassMetabolite(
    "pyr_c",
    name="Pyruvate",
    formula="C3H3O3",
    charge=-1,
    compartment="c",
    fixed=False)

lac__L_c = MassMetabolite(
    "lac__L_c",
    name="L-Lactate",
    formula="C3H5O3",
    charge=-1,
    compartment="c",
    fixed=False)

nad_c = MassMetabolite(
    "nad_c",
    name="Nicotinamide adenine dinucleotide",
    formula="[NAD]-C21H26N7O14P2",
    charge=-1,
    compartment="c",
    fixed=False)

nadh_c = MassMetabolite(
    "nadh_c",
    name="Nicotinamide adenine dinucleotide - reduced",
    formula="[NAD]-C21H27N7O14P2",
    charge=-2,
    compartment="c",
    fixed=False)

atp_c = MassMetabolite(
    "atp_c",
    name="ATP",
    formula="C10H12N5O13P3",
    charge=-4,
    compartment="c",
    fixed=False)

adp_c = MassMetabolite(
    "adp_c",
    name="ADP",
    formula="C10H12N5O10P2",
    charge=-3,
    compartment="c",
    fixed=False)

amp_c = MassMetabolite(
    "amp_c",
    name="AMP",
    formula="C10H12N5O7P",
    charge=-2,
    compartment="c",
    fixed=False)

pi_c = MassMetabolite(
    "pi_c",
    name="Phosphate",
    formula="HPO4",
    charge=-2,
    compartment="c",
    fixed=False)

h_c = MassMetabolite(
    "h_c",
    name="H+",
    formula="H",
    charge=1,
    compartment="c",
    fixed=False)

h2o_c = MassMetabolite(
    "h2o_c",
    name="H2O",
    formula="H2O",
    charge=0,
    compartment="c",
    fixed=False)
```

###### Reactions¶

Once all of the `MassMetabolite` objects for each metabolite, the next step is to define all of the reactions that occur and their stoichiometry.

1. As with the metabolites, it is also important to use a clear and consistent format for identifiers and names when defining when defining the `MassReaction` objects.
2. To make this model useful for integration with other models, it is important to provide a string to the `subsystem` argument. By providing the subsystem, the reactions can be easily obtained even when integrated with a significantly larger model through the `subsystem` attribute
3. After the creation of each `MassReaction` object, the metabolites are added to the reaction using a dictionary where keys are the `MassMetabolite` objects and values are the stoichiometric coefficients (reactants have negative coefficients, products have positive ones).

This model will be created using identifiers and names found in the BiGG Database.

In this model, there are 14 reactions occuring inside the cytosol compartment.

```
[4]:
```

```
HEX1 = MassReaction(
    "HEX1",
    name="Hexokinase (D-glucose:ATP)",
    subsystem=glycolysis.id,
    reversible=True)
HEX1.add_metabolites({
    glc__D_c: -1,
    atp_c: -1,
    adp_c: 1,
    g6p_c: 1,
    h_c: 1})

PGI = MassReaction(
    "PGI",
    name="Glucose-6-phosphate isomerase",
    subsystem=glycolysis.id,
    reversible=True)
PGI.add_metabolites({
    g6p_c: -1,
    f6p_c: 1})

PFK = MassReaction(
    "PFK",
    name="Phosphofructokinase",
    subsystem=glycolysis.id,
    reversible=True)
PFK.add_metabolites({
    f6p_c: -1,
    atp_c: -1,
    fdp_c: 1,
    adp_c: 1,
    h_c: 1})

FBA = MassReaction(
    "FBA",
    name="Fructose-bisphosphate aldolase",
    subsystem=glycolysis.id,
    reversible=True)
FBA.add_metabolites({
    fdp_c: -1,
    dhap_c: 1,
    g3p_c: 1})

TPI = MassReaction(
    "TPI",
    name="Triose-phosphate isomerase",
    subsystem=glycolysis.id,
    reversible=True)
TPI.add_metabolites({
    dhap_c: -1,
    g3p_c: 1})

GAPD = MassReaction(
    "GAPD",
    name="Glyceraldehyde-3-phosphate dehydrogenase",
    subsystem=glycolysis.id,
    reversible=True)
GAPD.add_metabolites({
    g3p_c: -1,
    nad_c: -1,
    pi_c: -1,
    _13dpg_c: 1,
    h_c: 1,
    nadh_c: 1})

PGK = MassReaction(
    "PGK",
    name="Phosphoglycerate kinase",
    subsystem=glycolysis.id,
    reversible=True)
PGK.add_metabolites({
    _13dpg_c: -1,
    adp_c: -1,
    _3pg_c: 1,
    atp_c: 1})

PGM = MassReaction(
    "PGM",
    name="Phosphoglycerate mutase",
    subsystem=glycolysis.id,
    reversible=True)
PGM.add_metabolites({
    _3pg_c: -1,
    _2pg_c: 1})

ENO = MassReaction(
    "ENO",
    name="Enolase",
    subsystem=glycolysis.id,
    reversible=True)
ENO.add_metabolites({
    _2pg_c: -1,
    h2o_c: 1,
    pep_c: 1})

PYK = MassReaction(
    "PYK",
    name="Pyruvate kinase",
    subsystem=glycolysis.id,
    reversible=True)
PYK.add_metabolites({
    pep_c: -1,
    h_c: -1,
    adp_c: -1,
    atp_c: 1,
    pyr_c: 1})

LDH_L = MassReaction(
    "LDH_L",
    name="L-lactate dehydrogenase",
    subsystem=glycolysis.id,
    reversible=True)
LDH_L.add_metabolites({
    h_c: -1,
    nadh_c: -1,
    pyr_c: -1,
    lac__L_c: 1,
    nad_c: 1})

ADK1 = MassReaction(
    "ADK1",
    name="Adenylate kinase",
    subsystem="Misc.",
    reversible=True)
ADK1.add_metabolites({
    adp_c: -2,
    amp_c: 1,
    atp_c: 1})

ATPM = MassReaction(
    "ATPM",
    name="ATP maintenance requirement",
    subsystem="Pseudoreaction",
    reversible=False)
ATPM.add_metabolites({
    atp_c: -1,
    h2o_c: -1,
    adp_c: 1,
    h_c: 1,
    pi_c: 1})

DM_nadh = MassReaction(
    "DM_nadh",
    name="Demand NADH",
    subsystem="Pseudoreaction",
    reversible=False)
DM_nadh.add_metabolites({
    nadh_c: -1,
    nad_c: 1,
    h_c: 1})
```

After generating the reactions, all reactions are added to the model through the `MassModel.add_reactions` class method. Adding the `MassReaction` objects will also add their associated `MassMetabolite` objects if they have not already been added to the model.

```
[5]:
```

```
glycolysis.add_reactions([
    HEX1, PGI, PFK, FBA, TPI, GAPD, PGK,
    PGM, ENO, PYK, LDH_L, ADK1, ATPM, DM_nadh])

for reaction in glycolysis.reactions:
    print(reaction)
```

```
HEX1: atp_c + glc__D_c <=> adp_c + g6p_c + h_c
PGI: g6p_c <=> f6p_c
PFK: atp_c + f6p_c <=> adp_c + fdp_c + h_c
FBA: fdp_c <=> dhap_c + g3p_c
TPI: dhap_c <=> g3p_c
GAPD: g3p_c + nad_c + pi_c <=> _13dpg_c + h_c + nadh_c
PGK: _13dpg_c + adp_c <=> _3pg_c + atp_c
PGM: _3pg_c <=> _2pg_c
ENO: _2pg_c <=> h2o_c + pep_c
PYK: adp_c + h_c + pep_c <=> atp_c + pyr_c
LDH_L: h_c + nadh_c + pyr_c <=> lac__L_c + nad_c
ADK1: 2 adp_c <=> amp_c + atp_c
ATPM: atp_c + h2o_c --> adp_c + h_c + pi_c
DM_nadh: nadh_c --> h_c + nad_c
```

###### Boundary reactions¶

After generating the reactions, the next step is to add the boundary reactions and boundary conditions (the concentrations of the boundary ‘metabolites’ of the system). This can easily be done using the `MassModel.add_boundary` method. With the generation of the boundary reactions, the system becomes an open system, allowing for the flow of mass through the biochemical pathways of the model.

Once added, the model will be able to return the boundary conditions as a dictionary through the `MassModel.boundary_conditions` attribute

In this model, there are 7 boundary reactions that must be defined.

All boundary reactions are originally created with the metabolite as the reactant. However, there are times where it would be preferable to represent the metabolite as the product. For these situtations, the `MassReaction.reverse_stoichiometry` method can be used with its `inplace` argument to create a new `MassReaction` or simply reverse the stoichiometry for the current `MassReaction.`

```
[6]:
```

```
SK_glc__D_c = glycolysis.add_boundary(
    metabolite=glc__D_c, boundary_type="sink", subsystem="Pseudoreaction",
    boundary_condition=1)
SK_glc__D_c.reverse_stoichiometry(inplace=True)

SK_lac__L_c = glycolysis.add_boundary(
    metabolite=lac__L_c, boundary_type="sink", subsystem="Pseudoreaction",
    boundary_condition=1)

SK_pyr_c = glycolysis.add_boundary(
    metabolite=pyr_c, boundary_type="sink", subsystem="Pseudoreaction",
    boundary_condition=0.06)

SK_h_c = glycolysis.add_boundary(
    metabolite=h_c, boundary_type="sink", subsystem="Pseudoreaction",
    boundary_condition=6.30957e-05)

SK_h2o_c = glycolysis.add_boundary(
    metabolite=h2o_c, boundary_type="sink", subsystem="Pseudoreaction",
    boundary_condition=1)

SK_amp_c = glycolysis.add_boundary(
    metabolite=amp_c, boundary_type="sink", subsystem="Pseudoreaction",
    boundary_condition=1)
SK_amp_c.reverse_stoichiometry(inplace=True)

DM_amp_c = glycolysis.add_boundary(
    metabolite=amp_c, boundary_type="demand", subsystem="Pseudoreaction",
    boundary_condition=1)

print("Boundary Reactions and Values\n-----------------------------")
for reaction in glycolysis.boundary:
    boundary_met = reaction.boundary_metabolite
    bc_value = glycolysis.boundary_conditions.get(boundary_met)
    print("{0}\n{1}: {2}\n".format(
        reaction, boundary_met, bc_value))
```

```
Boundary Reactions and Values
-----------------------------
SK_glc__D_c:  <=> glc__D_c
glc__D_b: 1.0

SK_lac__L_c: lac__L_c <=>
lac__L_b: 1.0

SK_pyr_c: pyr_c <=>
pyr_b: 0.06

SK_h_c: h_c <=>
h_b: 6.30957e-05

SK_h2o_c: h2o_c <=>
h2o_b: 1.0

SK_amp_c:  <=> amp_c
amp_b: 1.0

DM_amp_c: amp_c -->
amp_b: 1.0
```

###### Ordering of internal species and reactions¶

Sometimes, it is also desirable to reorder the metabolite and reaction objects inside the model to follow the physiology. To reorder the internal objects, one can use `cobra.DictList` containers and the `DictList.get_by_any` method with the list of object identifiers in the desirable order. To ensure all objects are still present and not forgotten in the model, a small QA check is also performed.

```
[7]:
```

```
new_metabolite_order = [
    "glc__D_c", "g6p_c", "f6p_c", "fdp_c", "dhap_c",
    "g3p_c", "_13dpg_c", "_3pg_c", "_2pg_c", "pep_c",
    "pyr_c", "lac__L_c", "nad_c", "nadh_c", "amp_c",
    "adp_c", "atp_c", "pi_c", "h_c", "h2o_c"]

if len(glycolysis.metabolites) == len(new_metabolite_order):
    glycolysis.metabolites = DictList(
        glycolysis.metabolites.get_by_any(new_metabolite_order))

new_reaction_order = [
    "HEX1", "PGI", "PFK", "FBA", "TPI",
    "GAPD", "PGK", "PGM", "ENO", "PYK",
    "LDH_L", "DM_amp_c", "ADK1", "SK_pyr_c",
    "SK_lac__L_c", "ATPM", "DM_nadh", "SK_glc__D_c",
    "SK_amp_c", "SK_h_c", "SK_h2o_c"]

if len(glycolysis.reactions) == len(new_reaction_order):
    glycolysis.reactions = DictList(
        glycolysis.reactions.get_by_any(new_reaction_order))

glycolysis.update_S(array_type="DataFrame", dtype=int)
```

```
[7]:
```

|  | HEX1 | PGI | PFK | FBA | TPI | GAPD | PGK | PGM | ENO | PYK | ... | DM\_amp\_c | ADK1 | SK\_pyr\_c | SK\_lac\_\_L\_c | ATPM | DM\_nadh | SK\_glc\_\_D\_c | SK\_amp\_c | SK\_h\_c | SK\_h2o\_c |
| --- | --- | --- | --- | --- | --- | --- | --- | --- | --- | --- | --- | --- | --- | --- | --- | --- | --- | --- | --- | --- | --- |
| glc\_\_D\_c | -1 | 0 | 0 | 0 | 0 | 0 | 0 | 0 | 0 | 0 | ... | 0 | 0 | 0 | 0 | 0 | 0 | 1 | 0 | 0 | 0 |
| g6p\_c | 1 | -1 | 0 | 0 | 0 | 0 | 0 | 0 | 0 | 0 | ... | 0 | 0 | 0 | 0 | 0 | 0 | 0 | 0 | 0 | 0 |
| f6p\_c | 0 | 1 | -1 | 0 | 0 | 0 | 0 | 0 | 0 | 0 | ... | 0 | 0 | 0 | 0 | 0 | 0 | 0 | 0 | 0 | 0 |
| fdp\_c | 0 | 0 | 1 | -1 | 0 | 0 | 0 | 0 | 0 | 0 | ... | 0 | 0 | 0 | 0 | 0 | 0 | 0 | 0 | 0 | 0 |
| dhap\_c | 0 | 0 | 0 | 1 | -1 | 0 | 0 | 0 | 0 | 0 | ... | 0 | 0 | 0 | 0 | 0 | 0 | 0 | 0 | 0 | 0 |
| g3p\_c | 0 | 0 | 0 | 1 | 1 | -1 | 0 | 0 | 0 | 0 | ... | 0 | 0 | 0 | 0 | 0 | 0 | 0 | 0 | 0 | 0 |
| \_13dpg\_c | 0 | 0 | 0 | 0 | 0 | 1 | -1 | 0 | 0 | 0 | ... | 0 | 0 | 0 | 0 | 0 | 0 | 0 | 0 | 0 | 0 |
| \_3pg\_c | 0 | 0 | 0 | 0 | 0 | 0 | 1 | -1 | 0 | 0 | ... | 0 | 0 | 0 | 0 | 0 | 0 | 0 | 0 | 0 | 0 |
| \_2pg\_c | 0 | 0 | 0 | 0 | 0 | 0 | 0 | 1 | -1 | 0 | ... | 0 | 0 | 0 | 0 | 0 | 0 | 0 | 0 | 0 | 0 |
| pep\_c | 0 | 0 | 0 | 0 | 0 | 0 | 0 | 0 | 1 | -1 | ... | 0 | 0 | 0 | 0 | 0 | 0 | 0 | 0 | 0 | 0 |
| pyr\_c | 0 | 0 | 0 | 0 | 0 | 0 | 0 | 0 | 0 | 1 | ... | 0 | 0 | -1 | 0 | 0 | 0 | 0 | 0 | 0 | 0 |
| lac\_\_L\_c | 0 | 0 | 0 | 0 | 0 | 0 | 0 | 0 | 0 | 0 | ... | 0 | 0 | 0 | -1 | 0 | 0 | 0 | 0 | 0 | 0 |
| nad\_c | 0 | 0 | 0 | 0 | 0 | -1 | 0 | 0 | 0 | 0 | ... | 0 | 0 | 0 | 0 | 0 | 1 | 0 | 0 | 0 | 0 |
| nadh\_c | 0 | 0 | 0 | 0 | 0 | 1 | 0 | 0 | 0 | 0 | ... | 0 | 0 | 0 | 0 | 0 | -1 | 0 | 0 | 0 | 0 |
| amp\_c | 0 | 0 | 0 | 0 | 0 | 0 | 0 | 0 | 0 | 0 | ... | -1 | 1 | 0 | 0 | 0 | 0 | 0 | 1 | 0 | 0 |
| adp\_c | 1 | 0 | 1 | 0 | 0 | 0 | -1 | 0 | 0 | -1 | ... | 0 | -2 | 0 | 0 | 1 | 0 | 0 | 0 | 0 | 0 |
| atp\_c | -1 | 0 | -1 | 0 | 0 | 0 | 1 | 0 | 0 | 1 | ... | 0 | 1 | 0 | 0 | -1 | 0 | 0 | 0 | 0 | 0 |
| pi\_c | 0 | 0 | 0 | 0 | 0 | -1 | 0 | 0 | 0 | 0 | ... | 0 | 0 | 0 | 0 | 1 | 0 | 0 | 0 | 0 | 0 |
| h\_c | 1 | 0 | 1 | 0 | 0 | 1 | 0 | 0 | 0 | -1 | ... | 0 | 0 | 0 | 0 | 1 | 1 | 0 | 0 | -1 | 0 |
| h2o\_c | 0 | 0 | 0 | 0 | 0 | 0 | 0 | 0 | 1 | 0 | ... | 0 | 0 | 0 | 0 | -1 | 0 | 0 | 0 | 0 | -1 |

20 rows × 21 columns

##### Model Parameterization¶

###### Steady State fluxes¶

Steady state fluxes can be computed as a summation of the MinSpan pathway vectors.

Pathways are obtained using the MinSpan package.

Using these pathways and literature sources, independent fluxes can be defined in order to calculate the steady state flux vector. For this model, flux and concentration data are obtained from the historical human red blood cell (RBC) metabolic model ([HRR78], [JP89a], [JP89b], [JP90], [JWPB02]).

From the literature, it is known that:

1. A typical uptake rate of the RBC of glucose is about 1.12 mM/hr.
2. Based on experimental data, the steady state load on NADH is set at 20% of the glucose uptake rate, or \(0.2 \* 1.12 = 0.244\).
3. The synthesis rate of AMP is measured to be 0.014 mM/hr.

With these pathays and numerical values, the steady state flux vector can be computed as the weighted sum of the corresponding basis vectors. The steady state flux vector is computed as an inner product:

```
[8]:
```

```
minspan_paths = [
    [1, 1, 1, 1, 1, 2, 2, 2, 2, 2, 2, 0, 0, 0, 2, 2, 0, 1, 0, 2, 0],
    [0, 0, 0, 0, 0, 0, 0, 0, 0, 0, -1, 0, 0, 1, -1, 0, 1, 0, 0, 2, 0],
    [0, 0, 0, 0, 0, 0, 0, 0, 0, 0, 0, 1, 0, 0, 0, 0, 0, 0, 1, 0, 0]]
glycolysis.compute_steady_state_fluxes(
    pathways=minspan_paths,
    independent_fluxes={
        SK_glc__D_c: 1.12,
        DM_nadh: .2 * 1.12,
        DM_amp_c: 0.014},
    update_reactions=True)

print("Steady State Fluxes\n-------------------")
for reaction, steady_state_flux in glycolysis.steady_state_fluxes.items():
    print("{0}: {1:.6f}".format(reaction.flux_symbol_str, steady_state_flux))
```

```
Steady State Fluxes
-------------------
v_HEX1: 1.120000
v_PGI: 1.120000
v_PFK: 1.120000
v_FBA: 1.120000
v_TPI: 1.120000
v_GAPD: 2.240000
v_PGK: 2.240000
v_PGM: 2.240000
v_ENO: 2.240000
v_PYK: 2.240000
v_LDH_L: 2.016000
v_DM_amp_c: 0.014000
v_ADK1: 0.000000
v_SK_pyr_c: 0.224000
v_SK_lac__L_c: 2.016000
v_ATPM: 2.240000
v_DM_nadh: 0.224000
v_SK_glc__D_c: 1.120000
v_SK_amp_c: 0.014000
v_SK_h_c: 2.688000
v_SK_h2o_c: 0.000000
```

###### Initial Conditions¶

Once the network has been built, the concentrations can be added to the metabolites. These concentrations are also treated as the initial conditions required to integrate and simulate the model’s ordinary differential equations (ODEs). The metabolite concentrations are added to each individual metabolite using the `MassMetabolite.initial_condition` (alias: `MassMetabolite.ic`) attribute setter methods. Once added, the model will be able to return the initial conditions as a dictionary
through the `MassModel.initial_conditions` attribute.

```
[9]:
```

```
glc__D_c.ic = 1.0
g6p_c.ic = 0.0486
f6p_c.ic = 0.0198
fdp_c.ic = 0.0146
g3p_c.ic = 0.00728
dhap_c.ic = 0.16
_13dpg_c.ic = 0.000243
_3pg_c.ic = 0.0773
_2pg_c.ic = 0.0113
pep_c.ic = 0.017
pyr_c.ic = 0.060301
lac__L_c.ic = 1.36
atp_c.ic = 1.6
adp_c.ic = 0.29
amp_c.ic = 0.0867281
h_c.ic = 0.0000899757
nad_c.ic = 0.0589
nadh_c.ic = 0.0301
pi_c.ic = 2.5
h2o_c.ic = 1.0

print("Initial Conditions\n------------------")
for metabolite, ic_value in glycolysis.initial_conditions.items():
    print("{0}: {1}".format(metabolite, ic_value))
```

```
Initial Conditions
------------------
glc__D_c: 1.0
g6p_c: 0.0486
f6p_c: 0.0198
fdp_c: 0.0146
dhap_c: 0.16
g3p_c: 0.00728
_13dpg_c: 0.000243
_3pg_c: 0.0773
_2pg_c: 0.0113
pep_c: 0.017
pyr_c: 0.060301
lac__L_c: 1.36
nad_c: 0.0589
nadh_c: 0.0301
amp_c: 0.0867281
adp_c: 0.29
atp_c: 1.6
pi_c: 2.5
h_c: 8.99757e-05
h2o_c: 1.0
```

###### Equilibirum Constants¶

After adding initial conditions and steady state fluxes, the equilibrium constants are defined using the `MassReaction.equilibrium_constant` (alias: `MassReaction.Keq`) setter method.

```
[10]:
```

```
HEX1.Keq = 850
PGI.Keq = 0.41
PFK.Keq = 310
FBA.Keq = 0.082
TPI.Keq = 0.0571429
GAPD.Keq = 0.0179
PGK.Keq = 1800
PGM.Keq = 0.147059
ENO.Keq = 1.69492
PYK.Keq = 363000
LDH_L.Keq = 26300
ADK1.Keq = 1.65

SK_glc__D_c.Keq = mass_config.irreversible_Keq
SK_lac__L_c.Keq = 1
SK_pyr_c.Keq = 1
SK_h_c.Keq = 1
SK_h2o_c.Keq = 1
SK_amp_c.Keq = mass_config.irreversible_Keq

print("Equilibrium Constants\n---------------------")
for reaction in glycolysis.reactions:
    print("{0}: {1}".format(reaction.Keq_str, reaction.Keq))
```

```
Equilibrium Constants
---------------------
Keq_HEX1: 850
Keq_PGI: 0.41
Keq_PFK: 310
Keq_FBA: 0.082
Keq_TPI: 0.0571429
Keq_GAPD: 0.0179
Keq_PGK: 1800
Keq_PGM: 0.147059
Keq_ENO: 1.69492
Keq_PYK: 363000
Keq_LDH_L: 26300
Keq_DM_amp_c: inf
Keq_ADK1: 1.65
Keq_SK_pyr_c: 1
Keq_SK_lac__L_c: 1
Keq_ATPM: inf
Keq_DM_nadh: inf
Keq_SK_glc__D_c: inf
Keq_SK_amp_c: inf
Keq_SK_h_c: 1
Keq_SK_h2o_c: 1
```

###### Calculation of PERCs¶

By defining the equilibrium constant and steady state parameters, the values of the pseudo rate constants (PERCs) can be calculated and added to the model using the `MassModel.calculate_PERCs` method.

```
[11]:
```

```
glycolysis.calculate_PERCs(update_reactions=True)

print("Forward Rate Constants\n----------------------")
for reaction in glycolysis.reactions:
    print("{0}: {1:.6f}".format(reaction.kf_str, reaction.kf))
```

```
Forward Rate Constants
----------------------
kf_HEX1: 0.700007
kf_PGI: 3644.444444
kf_PFK: 35.368784
kf_FBA: 2834.567901
kf_TPI: 34.355728
kf_GAPD: 3376.749242
kf_PGK: 1273531.269741
kf_PGM: 4868.589299
kf_ENO: 1763.740525
kf_PYK: 454.385552
kf_LDH_L: 1112.573989
kf_DM_amp_c: 0.161424
kf_ADK1: 100000.000000
kf_SK_pyr_c: 744.186047
kf_SK_lac__L_c: 5.600000
kf_ATPM: 1.400000
kf_DM_nadh: 7.441860
kf_SK_glc__D_c: 1.120000
kf_SK_amp_c: 0.014000
kf_SK_h_c: 100000.000000
kf_SK_h2o_c: 100000.000000
```

##### QA Model¶

Before simulating the model, it is important to ensure that the model is elementally balanced, and that the model can simulate. Therefore, the `qcqa_model` function from the `mass.util.qcqa`

submodule is used to provide a report on the model quality and indicate whether simulation is possible and if not, what parameters and/or initial conditions are missing.

Generally, pseudoreactions (e.g. boundary exchanges, sinks, demands) are not elementally balanced. The `qcqa_model` function does not include elemental balancing of boundary reactions.

However, some models contain pseudoreactions reprsenting a simplified mechanism, and show up in the returned report. The elemental imbalance of these pseudoreactions is therefore expected in certain reaction and should not be a cause for concern.

In this model of glycolysis, the NADH demand reaction is a simplified pseudoreaction that generates electrons for certain redox processes and is not expected to be balanced.

```
[12]:
```

```
qcqa_model(glycolysis, parameters=True, concentrations=True,
           fluxes=True, superfluous=True, elemental=True)
```

```
╒══════════════════════════════════════════════╕
│ MODEL ID: Glycolysis                         │
│ SIMULATABLE: True                            │
│ PARAMETERS NUMERICALY CONSISTENT: True       │
╞══════════════════════════════════════════════╡
│ ============================================ │
│             CONSISTENCY CHECKS               │
│ ============================================ │
│ Elemental                                    │
│ ----------------------                       │
│ DM_nadh: {charge: 2.0}                       │
│ ============================================ │
╘══════════════════════════════════════════════╛
```

From the results of the QC/QA test, it can be seen that the model can be simulated and is numerically consistent.

##### Steady State and Model Validation¶

To find the steady state of the model and perform simulations, the model must first be loaded into a `Simulation`. In order to load a model into a `Simulation`, the model must be simulatable, meaning there are no missing numerical values that would prevent the integration of the ODEs that comprise the model. The `verbose` argument can be used while loading a model to produce a message indicating the successful loading of a model, or why a model could not load.

Once loaded into a `Simulation`, the `find_steady_state` method can be used with the `update_values` argument in order to update the initial conditions and fluxes of the model to a steady state (if necessary). The model can be simulated using the `simulate` method by passing the model to simulate, and a tuple containing the start time and the end time. The number of time points can also be included, but is optional.

After a successful simulation, two `MassSolution` objects are returned. The first `MassSolution` contains the concentration results of the simulation, and the second contains the flux results of the simulation. The `MassSolution.view_time_profile` property can be used to quickly generate a time profile for the results.

```
[13]:
```

```
# Setup simulation object
sim = Simulation(glycolysis, verbose=True)
# Simulate from 0 to 1000 with 10001 points in the output
conc_sol, flux_sol = sim.simulate(glycolysis, time=(0, 1e3, 1e4 + 1))
# Quickly render and display time profiles
conc_sol.view_time_profile()
```

```
Successfully loaded MassModel 'Glycolysis' into RoadRunner.
```

###### Storing information and references¶

###### Compartment¶

Because the character “c” represents the cytosol compartment, it is recommended to define and set the compartment in the `MassModel.compartments` attribute.

```
[14]:
```

```
glycolysis.compartments = {"c": "Cytosol"}
print(glycolysis.compartments)
```

```
{'c': 'Cytosol'}
```

###### Units¶

All of the units for the numerical values used in this model are “Millimoles” for amount and “Liters” for volume (giving a concentration unit of ‘Millimolar’), and “Hours” for time. In order to ensure that future users understand the numerical values for model, it is important to define the `MassModel.units` attribute.The `MassModel.units` is a `cobra.DictList` that contains only `UnitDefinition` objects from the `mass.core.unit` submodule.

Each `UnitDefinition` is created from `Unit` objects representing the base units that comprise the `UnitDefinition`. These `Units` are stored in the `list_of_units` attribute. Pre-built units can be viewed using the `print_defined_unit_values` function from the `mass.core.unit` submodule. Alternatively, custom units can also be created using the `UnitDefinition.create_unit` function.

For more information about units, please see the module docstring for `mass.core.unit` submodule.

**Note:** It is important to note that this attribute will NOT track units, but instead acts as a reference for the user and others so that they can perform necessary unit conversions.

```
[15]:
```

```
# Using pre-build units to define UnitDefinitions
concentration = UnitDefinition("mM", name="Millimolar", list_of_units=["millimole", "per_litre"])
time = UnitDefinition("hr", name="hour", list_of_units=["hour"])

# Add units to model
glycolysis.add_units([concentration, time])
print(glycolysis.units)
```

```
[<UnitDefinition Millimolar "mM" at 0x7ff785435550>, <UnitDefinition hour "hr" at 0x7ff785435290>]
```

##### Export¶

After validation, the model is ready to be saved. The model can either be exported as a “.json” file or as an “.sbml” (“.xml”) file using their repsective submodules in `mass.io`.

To export the model, only the path to the directory and the name of the model need to be specified.

###### Export using JSON¶

```
[16]:
```

```
json.save_json_model(glycolysis, filename=glycolysis.id + ".json")
```

###### Export using SBML¶

```
[17]:
```

```
sbml.write_sbml_model(glycolysis, filename=glycolysis.id + ".xml")
```

## Educational Resources¶

Need to review the basic principles of dynamic simulation and analysis? Educational resources utilizing **MASSpy** are outlined below and available for academic purposes.

> - Systems Biology: Simulation of Dynamic Network States

### Systems Biology: Simulation of Dynamic Network States¶

Bernhard Ø. Palsson. Systems Biology: Simulation of Dynamic Network States. Cambridge University Press, 2011. doi:10.1017/CBO9780511736179.
Citation: [Pal11]

#### Introduction¶

Systems biology has been brought to the forefront of life science-based research and development. The need for systems analysis is made apparent by the inability of focused studies to explain whole network, cell, or organism behavior, and the availability of component data is what is fueling and enabling the effort. This massive amount of experimental information is a reflection of the complex molecular networks that underlie cellular functions. Reconstructed networks represent a common
denominator in systems biology. They are used for data interpretation, comparing organism capabilities, and as the basis for computing their functional states. The companion book *Systems Biology: Properties of Reconstructed Networks* details the topological features and assessment of functional states of biochemical reaction networks and how these features are represented by the stoichiometric matrix. In this book, we turn our attention to the kinetic properties of the reactions that make up a
network. We will focus on the formulation of dynamic simulators and how they are used to generate and study the dynamic states of biological networks.

##### Biological Networks¶

Cells are made up of many chemical constituents that interact to form networks. Networks are fundamentally comprised of *nodes* (the compounds) and the *links* (chemical transformations) between them. The networks take on functional states that we wish to compute, and it is these physiological states that we observe. This text is focused on dynamic states of networks.

There are many different kinds of biological networks of interest, and they can be defined in different ways. One common way to define networks is based on a preconceived notion of what they do. Examples include metabolic, signaling, and regulatory networks, see Figure 1.1. This approach is driven by a large body of literature that has grown around a particular cellular function.

**Figure 1.1:** Three examples of networks that are defined by major function. (a) Metabolism. (b) Signaling. From Arisi, et al. BMC Neuroscience 2006 7(Suppl 1):S6 DOI:10.1186/1471-2202-7-S1-S6. (c) Transcriptional regulatory networks. Image courtesy of Christopher Workman, Center for Biological Sequence Analysis, Technical University of Denmark.

###### Metabolic networks¶

Metabolism is ubiquitous in living cells and is involved in essentially all cellular functions. It has a long history - glycolysis was the first pathway elucidated in the 1930s - and is thus well-known in biochemical terms. Many of the enzymes and the corresponding genes have been discovered and characterized. Consequently, the development of dynamic models for metabolism is the most advanced at the present time.

A few large-scale kinetic models of metabolic pathways and networks now exist. Genome-scale reconstructions of metabolic networks in many organisms are now available. With the current developments in metabolomics and fluxomics, there is a growing number of large-scale data sets becoming available. However, there are no genome-scale dynamic models yet available for metabolism.

###### Signaling networks¶

Living cells have a large number of sensing mechanisms to measure and evaluate their environment. Bacteria have a surprising number of 2-component sensing systems that inform the organism about its nutritional, physical, and biological environment. Human cells in tissues have a large number of receptor systems in their membranes to which specific ligands bind, such as growth factors or chemokines. Such signaling influences the cellular fate processes: differentiation, replication, apoptosis, and
migration.

The function of many of the signaling pathways that is initiated by a sensing event are presently known, and this knowledge is becoming more detailed. Only a handful of signaling networks are well-known, such as the JAK-STAT signaling network in lymphocytes and the Toll-like receptor system in macrophages. A growing number of dynamic models for individual signaling pathways are becoming available.

###### Regulatory networks¶

There is a complex network of interactions that determine the DNA binding state of most proteins, which in turn determine if genes are being expressed. The RNA polymerase must bind to DNA, as do transcription factors and various other proteins. The details of these chemical interactions are being worked out, but in the absence of such information, most of the network models that have been built are discrete, stochastic, and logistical in nature.

With the rapid development of experimental methods that measure expression states, the binding sites, and their occupancy, we may soon see large-scale reconstructions of transcriptional regulatory networks. Once these are available, we can begin to plan the process to build models that will describe their dynamic states.

###### Unbiased network definitions¶

An alternative way to define networks is based on chemical assays. Measuring all protein-protein interactions regardless of function provides one such example, see Figure 1.2. Another example is a genome-wide measurement of the binding sites of a DNA-binding protein. This approach is driven by data-generating capabilities. It does not have an *a priori* bias about the function of molecules being examined.

**Figure 1.2:** Two examples of networks that are defined by high-throughput chemical assays. Images courtesy of Markus Herrgard.

###### Network reconstruction¶

Metabolic networks are currently the best-characterized biological networks for which the most detailed reconstructions are available. The conceptual basis for their reconstruction has been reviewed (Reed, 2006), the workflows used detailed (Feist, 2009) and a detailed standard operating procedure (SOP) is available (Thiele, 2010). Some of the fundamental issues associated with the generation of dynamic models describing their functions have been articulated (Jashmidi, 2008).

There is much interest in reconstructing signaling and regulatory networks in a similar way. The prospects for reconstruction of large-scale signaling networks have been discussed (Hyduke, 2010). Given the development of new omics data types and other information, it seems likely that we will be able to obtain reliable reconstructions of these networks in the not too distant future.

###### Public information about pathways and networks¶

There is a growing number of networks that underlie cellular functions that are being unraveled and reconstructed. Many publicly available sources contain this information, Table 1.1. We wish to study the dynamic states of such networks. To do so, we need to describe them in chemical detail and incorporate thermodynamic information and formulate a mathematical model.

**Table 1.1:** Web resources that contain information about biological networks. Prepared by Jan Schellenberger.

##### Why Build and Study Models?¶

Mathematical modeling is practiced in various branches of science and engineering. The construction of models is a laborious and detailed task. It also involves the use of numerical and mathematical analysis, both of which are intellectually intensive and unforgiving undertakings. So why bother?

###### Bailey’s five reasons¶

The purpose and utility of model building has been succinctly summarized and discussed (Bailey, 1998):

**1.** *“To organize disparate information into a coherent whole.”* The information that goes into building models is often found in many different sources and the model-builder has to look for these, evaluate them, and put them in context. In our case, this comes down to building data matrices (see Table 1.3) and determining conditions of interest.

**2.** *“To think (and calculate) logically about what components and interactions are important in a complex system.”* Once the information has been gathered it can be mathematically represented in a self-consistent format. Once equations have been formulated using the information gathered and according to the laws of nature, the information can be mathematically interrogated. The interactions among the different components are evaluated and the behavior of the model is compared to experimental
data.

**3.** *“To discover new strategies.”* Once a model has been assembled and studied, it often reveals relationships among its different components that were not previously known. Such observations often lead to new experiments, or form the basis for new designs. Further, when a model fails to reproduce the functions of the process being described, it means there is either something critical missing in the model or the data that led to its formulation is inconsistent. Such an occurrence then leads
to a re-examination of the information that led to the model formulation. If no logical flaw is found, the analysis of the discrepancy may lead to new experiments to try to discover the missing information.

**4.** *“To make important corrections to the conventional wisdom.”* The properties of a model may differ from the governing thinking about process phenomena that is inferred based on qualitative reasoning. Good models may thus lead to important new conceptual developments.

**5.** *“To understand the essential qualitative features.”* Since a model accounts for all described interactions among its parts, it often leads to a better understanding of the whole. In the present case, such qualitative features relate to multi-scale analysis in time and an understanding of how multiple chemical events culminate in coherent physiological features.

##### Characterizing Dynamic States¶

The dynamic analysis of complex reaction networks involves the tracing of time-dependent changes of concentrations and reaction fluxes over time. The concentrations typically considered are those of metabolites, proteins, or other cellular constituents. There are three key characteristics of dynamics states that we mention here right at the outset, and they are described in more detail in Section 2.1.

###### Time constants¶

Dynamic states are characterized by change in time, thus the rate of change becomes the key consideration. The rate of change of a variable is characterized by a *time constant*. Typically, there is a broad spectrum of time constants found in biochemical reaction networks. This leads to time scale separation where events may be happening on the order of milliseconds all the way to hours, if not days. The determination of the spectrum of time constants is thus central to the analysis of network
dynamics.

###### Aggregate variables¶

An associated issue is the identification of the biochemical, and ultimately physiological, events that are unfolding on every time scale. Once identified, one begins to form *aggregate concentration variables,* or *pooled variables*. These variables will be combinations of the original concentration variables. For example, two concentration variables may inter-convert rapidly, on the order of milliseconds, and thus on every time scale longer than milliseconds, these two concentrations will be
“connected.” They can therefore be “pooled” together to form an aggregate variable. An example is given in Figure 1.3.

The determination of such aggregate variables becomes an intricate mathematical problem. Once solved, it allows us to determine the *dynamic structure of a network*. In other words, we move hierarchically away from the original concentration variables to increasingly interlinked aggregate variables that ultimately culminate in the overall dynamic features of a network on slower time scales. Temporal decomposition therefore involves finding the time scale spectrum of a network and determining
what moves on each one of these time scales. A network can then be studied on any one of these time scales.

**Figure 1.3:** Time-scale hierarchy and the formation of aggregate variables in glycolysis.The ‘pooling’ process culminates in the formation of one pool (shown in a box at the bottom) that is filled by hexokinase (HK) and drained by ATPase. This pool represents the inventory of high energy phosphate bonds. From (Jashmidi, 2008).

###### Transitions¶

Complex networks can transition from one steady state (i.e., homeostatic state) to another. There are distinct types of transitions that characterize the dynamic states of a network. Transitions are analyzed by *bifurcation theory*. The most common bifurcations involve the emergence of *multiple steady states,* *sustained oscillations,* and *chaotic behavior*. Such dynamic features call for a yet more sophisticated mathematical treatment. Such changes in dynamic states have been called *creative
functions,* which in turn represent willful physiological changes in organism behavior. In this book, we will only encounter relatively simple types of such transitions.

##### 1.4 Formulating Dynamic Network Models¶

###### Approach¶

Mechanistic kinetic models based on differential equations represent a *bottom-up approach*. This means that we identify all the detailed events in a network and systematically build it up in complexity by adding more and more new information about the components of a network and how they interact. A complementary approach to the analysis of a biochemical reaction network is a *top-down approach* where one collects data and information about the state of the whole network at one time. This
approach is not covered in this text but typically requires a Bayesian or Boolean analysis that represents causal or statistically-determined relationships between network components. The bottom-up approach requires a mechanistic understanding of component interactions. Both the top-down and bottom-up approaches are useful and complimentary in studying the dynamic states of networks.

###### Simplifying assumptions¶

Kinetic models are typically formulated as a set of deterministic ordinary differential equations (ODEs). There are a number of important assumptions made in such formulations that often are not fully described and delineated. Five assumptions will be discussed here (Table 1.2).

**Table 1.2:** Assumptions used in the formulation of biological network models.

**1.** Using deterministic equations to model biochemistry essentially implies a “clockwork” of functionality. However, this modeling assumption needs justification. There are three principal sources of variability in biological dynamics: internal thermal noise, changes in the environment, and cell-to-cell variation. Inside cells, all components experience thermal effects that result in *random molecular motion*. This process is, of course, one of molecular diffusion, called *Brownian motion*
with larger observable objects. The ordinary differential equation assumption involves taking an ensemble of molecules and averaging out the stochastic effects. In cases where there are very few molecules of a particular species inside a cell or a cellular compartment, this assumption may turn out to be erroneous.

**2.** The finer architecture of cells is also typically not considered in kinetic models. Cells are highly structured down to the 100 nm length scale and are thus not homogeneous (see Figure 1.4). Rapidly diffusing compounds, such as metabolites and ions, will distribute rapidly throughout the compartment and one can justifiably consider the concentration to be relatively uniform. However, with larger molecules whose diffusion is hindered and confined, one may have to consider their spatial
location. Studying and describing cellular functions of the 100 nm length scale is likely to represent an interesting topic in systems biology as it unfolds.

**3.** Another major assumption in most kinetic models is that of *constant volume*. Cells and cellular compartments typically have fluctuations in their volume. Treating variable volume turns out to be mathematically difficult. It is, therefore, often ignored. However, minor fluctuations in the volume of a cellular compartment may change all the concentrations in that compartment and therefore all kinetic and regulatory effects.

**4.** Temperature is typically considered to be a constant. Larger organisms have the capability to control their temperature. Small organisms have a high surface to volume ratio making it hard to control heat flux at their periphery. Further, small cellular dimensions lead to rapid thermal diffusivity and a strong dependency on the thermal characteristics of the environment. Rate constants are normally a strong function of temperature, often described by Arrhenius’ law. Thus, treating cells as
isothermal systems is a simplification under which the kinetic properties are described by kinetic constants.

**5.** All cells and cellular compartments must maintain electro-neutrality and therefore the exchange of any species in and out of a compartment or a cell must also obey electro-neutrality. Considering the charge of molecules and their pH dependence is yet another complicated mathematical subject and thus often ignored. Similarly, significant internal osmotic pressure must be balanced with that of the environment. Cells in tissues are in an isotonic environment, while single cellular organisms
and cells in plants build rigid walls to maintain their integrity.

**Figure 1.4:** The crowded state of the intracellular environment.Some of the physical characteristics are viscosity \((>100\ \mu\_{H2O})\), osmotic pressure \((<150 atm)\), electrical gradients \((\approx 300,000\ V/cm)\), and a near crystalline state. © David S.Goodsell 1999.

###### The dynamic mass balance equations¶

Applying these simplifying assumptions, we arrive at the dynamic mass balance equations as the starting point for modeling the dynamic states of biochemical reaction networks. The basic notion of a dynamic mass balance on a single compound, \(x\_i\), is shown in Figure 1.5.

**Figure 1.5:** The dynamic mass balance on a single compound. (a) All the rates of formation and degradation of a compound \(x\_i\) (a graphical representation called a node map). (b) The corresponding dynamic mass balance equation that simply states that the rate of change of the concentrations \(x\_i\) is equal to the sum of the rates of formation minus the sum of the rates of degradation. This summation can be represented as an inner product between a row vector and the flux vector.
This row vector becomes a row in the stoichiometric matrix in Eq. 1.1.

The combination of all the dynamic mass balances for all concentrations (\(\textbf{x}\))in a biochemical reaction network are given by a matrix equation:

\[\begin{equation} \frac{d\textbf{x}}{dt} = \textbf{Sv}(\textbf{x}) \tag{1.1} \end{equation}\]

where \(\textbf{S}\) is the stoichiometric matrix, \(\textbf{v}\) is the vector of reaction fluxes \((v\_j)\), and \(\textbf{x}\) is the vector of concentrations \((x\_i)\). Equation 1.1 will be the ‘master’ equation that will be used to describe network dynamics states in this book.

###### Alternative views¶

There are a number of considerations that come with the differential equation formalism described in this book and in the vast majority of the research literature on this subject matter. Perhaps the most important issue is the treatment of cells as behaving deterministically and displaying continuous changes over time. It is possible, though, that cells ultimately will be viewed as essentially a *liquid crystalline state* where transitions will be discreet from one state to the next, and not
continuous.

##### The Basic Information is in a Matrix Format¶

The natural mathematical language for describing network states using dynamic mass balances is that of matrix algebra. In studying the dynamic states of networks, there are three fundamental matrices of interest: the stoichiometric, the gradient, and the Jacobian matrices.

**Table 1.3:** Comparison of some of the attributes and properties of the stoichiometric and the gradient matrices. Adapted from (Jashmidi, 2008).

###### The stoichiometric matrix¶

The stoichiometric matrix, the properties of which are detailed in (SB1), represents the reaction topology of a network. Every row in this matrix represents a compound (alternative states require multiple rows) and every column represents a link between compounds, or a chemical reaction. All the entries in this matrix are *stoichiometric coefficients*. This matrix is “knowable,” since it is comprised of integers that have no error associated with them. The stoichiometric matrix is
*genomically-derived* and thus all members of a species or a biopopulation will have the same stoichiometric matrix.

Mathematically, the stoichiometric matrix has important features. It is a *sparse matrix,* which means that few of its elements are non-zero. Typically, less than 1% of the elements of a genome-scale stoichiometric matrix are non-zero, and those non-zero elements are almost always 1 or -1. Occasionally there will be an entry of numerical value 2, which may represent the formation of a homodimer. The fact that all the elements of \(\textbf{S}\) are of the same order of magnitude makes it a
convenient matrix to deal with from a numerical standpoint. The properties of the stoichiometric matrix have been extensively studied (SB1).

###### The gradient matrix¶

Each link in a reaction map has kinetic properties with which it is associated. The reaction rates that describe the kinetic properties are found in the rate laws, \(\textbf{v}(\textbf{x}; \textbf{k})\), where the vector \(\textbf{k}\) contains all the kinetic constants that appear in the rate laws. Ultimately, these properties represent time constants that tell us how quickly a link in a network will respond to the concentrations that are involved in that link. The reciprocal of these
time constants are found in the gradient matrix, \(\textbf{G}\), whose elements are:

\[\begin{equation} g\_{ij} = \frac{\partial v\_i}{\partial x\_j}[\text{time}^{-1}] \tag{1.2} \end{equation}\]

These constants may change from one member to the next in a biopopulation given the natural sequence diversity that exists. Therefore, the gradient matrix, is a *genetically-determined* matrix. Two members of the population may have a different \(\textbf{G}\) matrix. This difference is especially important in cases where mutations exist that significantly change the kinetic properties of critical steps in the network and change its dynamic structure. Such changes in the properties of a
single link in a network may change the properties of the entire network.

Mathematically speaking, \(\textbf{G}\) has several challenging features. Unlike the stoichiometric matrix, its numerical values vary over many orders of magnitude. Some links have very fast response times, while others have long response times. The entries of \(\textbf{G}\) are real numbers and therefore are not “knowable.” The values of \(\textbf{G}\) will always come with an error bar associated with the experimental method used to determine them. Sometimes only order-of-magnitude
information about the numerical values of the entries in \(\textbf{G}\) is sufficient to allow us to determine the overall dynamic properties of a network. The matrix \(\textbf{G}\) has the same sparsity properties as the matrix \(\textbf{S}\).

###### The Jacobian matrix¶

The Jacobian matrix, \(\textbf{J}\), is the matrix that characterizes network dynamics. It is a product of the stoichiometric matrix and the gradient matrix. The stoichiometric matrix gives us network structure, and the gradient matrix gives us kinetic parameters of the links in the network. The product of these two matrices gives us the network dynamics.

The three matrices described above are thus not independent. The Jacobian is given by

\[\begin{equation} \textbf{J} = \textbf{SG} \tag{1.3} \end{equation}\]

The properties of \(\textbf{S}\) and \(\textbf{G}\) are compared in Table 1.3.

##### Summary¶

- Large-scale biological networks that underlie various cellular functions can now be reconstructed from detailed data sets and published literature.
- Mathematical models can be built to study the dynamic states of networks. These dynamic states are most often described by ordinary differential equations.
- There are significant assumptions leading to an ordinary differential equations formalism to describe dynamic states. Most notably is the elimination of molecular noise, assuming volumes and temperature to be constant, and considering spatial structure to be insignificant.
- Networks have dynamic states that are characterized by time constants, pooling of variables, and characteristic transitions. The basic dynamic properties of a network come down to analyzing the spectrum of time-scales associated with a network and how the concentrations move on these time-scales.
- Sometimes the concentrations move in tandem at certain time-scales, leading to the formation of aggregate variables. This property is key to the hierarchical decomposition of networks and the understanding of how physiological functions are formed.
- The data used to formulate models to describe dynamic states of networks is found in two matrices: the stoichiometric matrix, that is typically well-known, the gradient matrix, whose elements are harder to determine.
- Dynamic states can be studied by simulation or mathematical analysis. This text is focused on the process of dynamic simulation and its uses.

\(\tiny{\text{© B. Ø. Palsson 2011;}\ \text{This publication is in copyright.}\\ \text{Subject to statutory exception and to the provisions of relevant collective licensing agreements,}\\ \text{no reproduction of any part may take place without the written permission of Cambridge University Press.}}\)

#### Basic Concepts¶

The bottom-up analysis of dynamic states of a network is based on network topology and kinetic theory describing the links in the network. In this chapter, we provide a primer for the basic concepts of dynamic analysis of network states. We also discuss basics of the kinetic theory that is needed to formulate and understand detailed dynamic models of biochemical reaction networks.

##### Properties of Dynamic States¶

The three key dynamic properties outlined in the introduction - time constants, aggregate variables and transitions - are detailed in this section.

###### Time scales¶

A fundamental quantity in dynamic analysis is the *time constant*. A time constant is a measure of time span over which significant changes occur in a state variable. It is thus a scaling factor for time and determines where in the time scale spectrum one needs to focus attention when dealing with a particular process or event of interest.

A general definition of a time constant is given by

\[\begin{equation} \tau = \frac{\Delta x}{|dx/dt|\_{avg}} \tag{2.1} \end{equation}\]

where \(\Delta{x}\) is a characteristic change in the state variable \(x\) of interest and \(|dx/dt|\_{avg}\) is an estimate of the rate of change of the variable \(x\). Notice the ratio between \(\Delta{x}\) and the average derivative has units of time, and the time constant characterizes the time span over which these changes in \(x\) occur, see Figure 2.1.

**Figure 2.1:** Illustration of the concept of a time constant, \(\tau\), and its estimation as \(\tau = \Delta x\ / |dx/dt|\_{avg}.\)

In a network, there are many time constants. In fact, there is a spectrum of time constants, \(\tau\_1,\ \tau\_2, \dots \tau\_r\) where \(r\) is the rank of the Jacobian matrix defining the dynamic dimensionality of the dynamic response of the network. This spectrum of time constants typically spans many orders of magnitude. The consequences of a well-separated set of time constants is a key concern in the analysis of network dynamics.

###### Forming aggregate variables through “pooling”¶

One important consequence of time scale hierarchy is the fact that we will have fast and slow events. If fast events are filtered out or ignored, one removes a dynamic degree of freedom from the dynamic description, thus reducing the dynamic dimension of a system. Removal of a dynamic dimension leads to “coarse-graining” of the dynamic description. Reduction in dynamic dimension results in the combination, or pooling, of variables into aggregate variables.

A simple example can be obtained from upper glycolysis. The first three reactions of this pathway are:

\[\begin{equation} \text{glucose}\ \underset{\stackrel{\frown}{ATP \ ADP}}{\stackrel{HK}{\longrightarrow}} \text{G6P} \underset{\text{fast}, \tau\_f}{\stackrel{PGI}{\leftrightharpoons}} \text{F6P} \underset{\stackrel{\frown}{ATP \ ADP}}{\stackrel{PFK}{\longrightarrow}} \text{FDP} \tag{2.2} \end{equation}\]

This schema includes the second step in glycolysis where glucose-6-phosphate (G6P) is converted to fructose-6-phosphate (F6P) by the phosphogluco-isomerase (PGI). Isomerases are highly active enzymes and have rate constants that tend to be fast. In this case, PGI has a much faster response time than the response time of the flanking kinases in this pathway, hexokinase (HK) and phosphofructokinase (PFK). If one considers a time period that is much greater than \(\tau\_f\) (the time constant
associated with PGI), this system is simplified to:

\[\begin{equation} \underset{\stackrel{\frown}{ATP \ ADP}}{\stackrel{HK}{\longrightarrow}} \ \underset{t \gg \tau\_f}{\text{HP}} \ \underset{\stackrel{\frown}{ATP \ ADP}}{\stackrel{PFK}{\longrightarrow}} \tag{2.3} \end{equation}\]

where HP = (G6P+F6P) is the hexosephosphate pool. At a slow time scale (i.e, long compared to \(\tau\_f\)), the isomerase reaction has effectively equilibrated, leading to the removal of its dynamics from the network. As a result, F6P and G6P become dynamically coupled and can be considered to be a single variable. HP is an example of an aggregate variable that results from pooling G6P and F6P into a single variable. Such aggregation of variables is a consequence of time-scale hierarchy in
networks. Determining how to aggregate variables into meaningful quantities becomes an important consideration in the dynamic analysis of network states. Further examples of pooling variables are given in Section 2.3.

###### Transitions¶

The dynamic analysis of a network comes down to examining its transient behavior as it moves from one state to another.

**Figure 2.2:** Illustration of a transition from one state to another. (a) A simple transition. (b) A more complex set of transitions.

One type of transition, or *transient response,* is illustrated in Figure 2.2a, where a system is in a homeostatic state, labeled as state \(\text{#1}\), and is perturbed at time zero. Over some time period, as a result of the perturbation, it transitions into another homeostatic state (state \(\text{#2}\)). We are interested in characteristics such as the time duration of this response, as well as looking at the dynamic states that the network exhibits during this transition. Complex
types of transitions are shown in Figure 2.2b.

It should be noted that when complex kinetic models are studied, there are two ways to perturb a system and induce a transient response. One is to instantaneously change the initial condition of one of the state variables (typically a concentration), and the second is to change the state of an environmental variable that represents an input to the system. The latter perturbation is the one that is biologically meaningful, whereas the former may be of some mathematical interest.

###### Visualizing dynamic states¶

There are several ways to graphically represent dynamic states:

- First, we can represent them on a map (Figure 2.3a). If we have a reaction or a compound map for a network of interest, we can simply draw it out on a computer screen and leave open spaces above the arrows and the concentrations into which we can write numerical values for these quantities. These quantities can then be displayed dynamically as the simulation proceeds, or by a graph showing the changes in the variable over time. This representation requires writing complex software to make
  such an interface.
- A second, and probably more common, way of viewing dynamic states is to simply graph the state variables, \(x\), as a function of time (Figure 2.3b). Such graphs show how the variables move up and down, and on which time scales. Often, one uses a logarithmic scale for the y-axis, and that often delineates the different time constants on which a variable moves.
- A third way to represent dynamic solutions is to plot two state variables against one another in a two-dimensional plot (Figure 2.3c). This representation is known as a *phase portrait*. Plotting two variables against one another traces out a curve in this plane along which time is a parameter. At the beginning of the trajectory, time is zero, and at the end, time has gone to infinity. These phase portraits will be discussed in more detail in Chapter 3.

**Figure 2.3:** Graphical representation of dynamic states.

##### Primer on Rate Laws¶

The reaction rates, \(v\_i\), are described mathematically using kinetic theory. In this section, we will discuss some of the fundamental concepts of kinetic theory that lead to their formation.

###### Elementary reactions¶

The fundamental events in chemical reaction networks are elementary reactions. There are two types of elemental reactions:

\[\begin{split}\begin{align} &\text{linear} &x \stackrel{v}{\rightarrow} \\ &\text{bi-linear} & x\_1 + x\_2 \stackrel{v}{\rightarrow} \\ \end{align} \tag{2.4}\end{split}\]

A special case of a bi-linear reaction is when \(x\_1\) is the same as \(x\_2\) in which case the reaction is second order.

Elementary reactions represent the irreducible events of chemical transformations, analogous to a base pair being the irreducible unit of DNA sequence. Note that rates, \(v\), and concentrations, \(x\), are non-negative variables, that is;

\[\begin{equation} x \geq 0, \ v \geq 0 \tag{2.5} \end{equation}\]

###### Mass action kinetics¶

The fundamental assumption underlying the mathematical description of reaction rates is that they are proportional to the collision frequency of molecules taking part in a reaction. Most commonly, reactions are bi-linear, where two different molecules collide to produce a chemical transformation. The probability of a collision is proportional to the concentration of a chemical species in a 3-dimensional unconstrained domain. This proportionality leads to the elementary reaction rates:

\[\begin{split}\begin{align} \text{linear} \ \ &v = kx \ &\text{where the units on}& \ k \ \text{are time}^{-1} \ \text{and} \\ \text{bi-linear} \ \ &v = kx\_1x\_2 \ &\text{where the units on}& \ k \ \text{are time}^{-1}\text{conc}^{-1} \\ \end{align} \tag{2.6}\end{split}\]

###### Enzymes increase the probability of the ‘right’ collision¶

Not all collisions of molecules have the same probability of producing a chemical reaction. Collisions at certain angles are more likely to produce a reaction than others. As illustrated in Figure 2.4, molecules bound to the surface of an enzyme can be oriented to produce collisions at certain angles, thus accelerating the reaction rate. The numerical values of the rate constants are thus genetically determined as the structure of a protein is encoded in the sequence of the DNA. Sequence
variation in the underlying gene in a population leads to differences amongst the individuals that make up the population. Principles of enzyme catalysis are further discussed in Section 5.1.

**Figure 2.4:** A schematic showing how the binding sites of two molecules on an enzyme bring them together to collide at an optimal angle to produce a reaction. Panel A: Two molecules can collide at random and various angles in free solution. Only a fraction of the collisions lead to a chemical reaction. Panel B: Two molecules bound to the surface of a reaction can only collide at a highly restricted angle, substantially enhancing the probability of a chemical reaction between the two
compounds. Redrawn based on (Lowenstein, 2000).

###### Generalized mass action kinetics¶

The reaction rates may not be proportional to the concentration in certain circumstances, and we may have what are called *power-law kinetics*. The mathematical form of the elementary rate laws are

\[\begin{split}\begin{align} v &= kx^a \\ v &= kx\_1^ax\_2^b \\ \end{align} \tag{2.7}\end{split}\]

where \(a\) and \(b\) can be greater or smaller than unity. In cases where a restricted geometry reduces the probability of collision relative to a geometrically-unrestricted case, the numerical values of \(a\) and \(b\) are less than unity, and vice versa.

###### Combining elementary reactions¶

In the analysis of chemical kinetics, the elementary reactions are often combined into reaction mechanisms. Following are two such examples:

###### Reversible reactions:¶

If a chemical conversion is thermodynamically reversible, then the two opposite reactions can be combined as

\[\begin{equation} x\_1 \underset{v\_{-}}{\stackrel{v\_+}{\rightleftharpoons}} x\_2 \end{equation}\]

The net rate of the reaction can then be described by the difference between the forward and reverse reactions;

\[\begin{split}\begin{equation} v\_{net} = v^+ - v^- = k^+x\_1 - k^-x\_2, \\ K\_{eq} = x\_2 / x\_1 = k^+/k^- \end{equation} \tag{2.8}\end{split}\]

where \(K\_{eq}\) is the equilibrium constant for the reaction. Note that \(v\_{net}\) can be positive or negative. Both \(k^+\) and \(k^-\) have units of reciprocal time. They are thus inverses of time constants. Similarly, a net reversible bi-linear reaction can be written as

\[\begin{equation} x\_1 + x\_2 \underset{v\_{-}}{\stackrel{v\_+}{\rightleftharpoons}} x\_3 \end{equation}\]

The net rate of the reaction can then be described by

\[\begin{split}\begin{equation} v\_{net} = v^+ - v^- = k^+x\_1x\_2 - k^-x\_3, \\ K\_{eq} = x\_3 / x\_1x\_2 = k^+/k^- \end{equation}\end{split}\]

where \(K\_{eq}\) is the equilibrium constant for the reaction. The units on the rate constant \((k^+)\) for a bi-linear reaction are concentration per time. Note that we can also write this equation as

\[\begin{equation} v\_{net} = k^+x\_1x\_2 - k^-x\_3 = k^+(x\_1x\_2 - x\_3/K\_{eq}) \end{equation}\]

that can be a convenient form as often the \(K\_{eq}\) is a known number with a thermodynamic basis, and thus only a numerical value for \(k^+\) needs to be estimated.

###### Converting enzymatic reaction mechanisms into rate laws:¶

Often, more complex combinations of elementary reactions are analyzed. The classical irreversible Michaelis-Menten mechanism is comprised of three elementary reactions.

\[\begin{equation} S + E \underset{v\_{-1} = k\_{-1}x}{\stackrel{v\_1 = k\_1se}{\rightleftharpoons}} X \stackrel{v\_2 = k\_2x}{\longrightarrow} E + P \end{equation}\]

where a substrate, \(S\), binds to an enzyme to form a complex, \(X\), that can break down to generate the product, \(P\). The concentrations of the corresponding chemical species is denoted with the same lower case letter; i.e., \(e=[E]\), etc. This reaction mechanism has two conservation quantities associated with it: one on the enzyme \(e\_{tot} = e + x\) and one on the substrate \(s\_{tot} = s+x+p\).

A quasi-steady-state assumption (QSSA), \(dx/dt=0\), is then applied to generate the classical rate law

\[\begin{equation} \frac{ds}{dt} = \frac{-v\_ms}{K\_m + s} \tag{2.9} \end{equation}\]

that describes the kinetics of this reaction mechanism. This expression is the best-known rate equation in enzyme kinetics. It has two parameters: the maximal reaction rate \(v\_m\), and the Michaelis-Menten constant \(K\_m = (k\_{-1} + k\_2)/k\_1\). The use and applicability of kinetic assumptions to deriving rate laws for enzymatic reaction mechanisms is discussed in detail in Chapter 5.

It should be noted that the elimination of the elementary rates through the use of the simplifying kinetic assumptions *fundamentally changes* the mathematical nature of the dynamic description from that of bi-linear equations to that of hyperbolic equations (i.e., Eq. 2.9) and, more generally, to ratios of polynomial functions.

###### Pseudo-first order rate constants (PERCs)¶

The effects of temperature, pH, enzyme concentrations, and other factors that influence the kinetics can often be accounted for in a condition specific numerical value of a constant that looks like a regular elementary rate constant, as in Eq (2.4). The advantage of having such constants is that it simplifies the network dynamic analysis. The disadvantage is that dynamic descriptions based on PERCs are condition specific. This issue is discussed in Parts 3 and 4 of the book.

###### The mass action ratio (\(\Gamma\))¶

The equilibrium relationship among reactants and products of a chemical reaction are familiar to the reader. For example, the equilibrium relationship for the PGI reaction (Eq. (2.8)) is

\[\begin{equation} K\_{eq} = \frac{[\text{F6P}]\_{eq}}{[\text{G6P}]\_{eq}} \tag{2.10} \end{equation}\]

This relationship is observed in a closed system after the reaction is allowed to proceed to equilibrium over a long time, \(t \rightarrow \infty\), (which in practice has a meaning relative to the time constant of the reaction, \(t \gg \tau\_f\)).

However, in a cell, as shown in Eq. (2.2), the PGI reactions operate in an “open” environment, i.e., G6P is being produced and F6P is being consumed. The reaction reaches a steady state in a cell that will have concentration values that are different from the equilibrium value. The *mass action ratio* for open systems, defined to be analogous to the equilibrium constant, is

\[\begin{equation} \Gamma = \frac{[\text{F6P}]\_{ss}}{[\text{G6P}]\_{ss}} \tag{2.11} \end{equation}\]

The mass action ratio is denoted by \(\Gamma\) in the literature.

###### ‘Distance’ from equilibrium¶

The numerical value of the ratio \(\Gamma / K\_{eq}\) relative to unity can be used as a measure of how far a reaction is from equilibrium in a cell. Fast reversible reactions tend to be close to equilibrium in an open system. For instance, the net reaction rate for a reversible bi-linear reaction (Eq. (2.2)) can be written as:

\[\begin{equation} v\_{net} = k^+x\_1x\_2 - k^-x\_3 = k^+x\_1x\_2(1 - \Gamma/K\_{eq}) \end{equation}\]

If the reaction is “fast” then \((k^+x\_1x\_2)\) is a “large” number and thus \((1 - \Gamma/K\_{eq})\) tends to be a “small” number, since the net reaction rate is balanced relative to other reactions in the network.

###### Recap¶

These basic considerations of reaction rates and enzyme kinetic rate laws are described in much more detail in other standard sources, e.g., (Segal, 1975). In this text, we are not so concerned about the details of the mathematical form of the rate laws, but rather with the order-of-magnitude of the rate constants and how they influence the properties of the dynamic response.

##### More on Aggregate Variables¶

Pools, or aggregate variables, form as a result of well-separated time constants. Such pools can form in a hierarchical fashion. Aggregate variables can be physiologically significant, such as the total inventory of high-energy phosphate bonds, or the total inventory of particular types of redox equivalents. These important concepts are perhaps best illustrated through a simple example that should be considered a primer on a rather important and intricate subject matter. Formation of aggregate
variables in complex models is seen throughout Parts III and IV of this text.

**Figure 2.5:** The chemical transformations involved in the distribution of high-energy phosphate bonds among adenosines.

###### Distribution of high-energy phosphate among the adenylate phosphates¶

In Figure 2.5 we show the skeleton structure of the transfer of high-energy phosphate bonds among the adenylates. In this figure we denote the use of ATP by \(v\_1\) and the synthesis of ATP from ADP by \(v\_2\), \(v\_5\) and \(v\_{-5}\) denote the reaction rates of adenylate kinase that distributes the high energy phosphate bonds among ATP, ADP, and AMP, through the reaction

\[\begin{equation} 2 \text{ADP} \leftrightharpoons \text{ATP} + \text{AMP} \tag{2.12} \end{equation}\]

Finally, the synthesis of AMP and its degradation is denoted by \(v\_3\) and \(v\_4\), respectively. The dynamic mass balance equations that describe this schema are:

\[\begin{split}\begin{align} \frac{d \text{ATP}}{dt} &= -v\_1 + v\_2 + v\_{5, net} \\ \frac{d \text{ADP}}{dt} &= v\_1 - v\_2 - 2 v\_{5, net} \\ \frac{d \text{AMP}}{dt} &= v\_3 - v\_4 + v\_{5, net} \end{align} \tag{2.13}\end{split}\]

The responsiveness of these reactions falls into three categories: \(v\_{5, net} (=v\_5 - v\_{-5})\) is a *fast* reversible reaction, \(v\_1\) and \(v\_2\) have *intermediate* time scales, and the kinetics of \(v\_3\) and \(v\_4\) are *slow* and have large time constants associated with them. Based on this time scale decomposition, we can combine the three concentrations so that they lead to the elimination of the reactions of a particular response time category on the right hand
side of (Eq. 2.13). These combinations are as follows:

- First, we can eliminate all but the slow reactions by forming the sum of the adenosine phosphates.

  \[\begin{equation} \frac{d}{dt}(\text{ATP} + \text{ADP} + \text{AMP}) = v\_3 - v\_4\ \text{(slow)} \tag{2.14} \end{equation}\]

  The only reaction rates that appear on the right hand side of the equation are \(v\_3\) and \(v\_4\), that are the slowest reactions in the system. Thus, the summation of ATP, ADP, and AMP is a pool or aggregate variable that is expected to exhibit the slowest dynamics in the system.
- The second pooled variable of interest is the summation of 2ATP and ADP that represents the total number of high energy phosphate bonds found in the system at any given point in time:

  \[\begin{equation} \frac{d}{dt}(2 \text{ATP} + \text{ADP}) = -v\_1 + v\_2\ \text{(intermediate)} \tag{2.15} \end{equation}\]

  This aggregate variable is only moved by the reaction rates of intermediate response times, those of \(v\_1\) and \(v\_2\).
- The third aggregate variable we can form is the sum of the energy carrying nucleotides which are

  \[\begin{equation} \frac{d}{dt}(\text{ATP} + \text{ADP}) = -v\_{5, net}\ \text{(fast)} \tag{2.16} \end{equation}\]

  This summation will be the fastest aggregate variable in the system.

Notice that by combining the concentrations in certain ways, we define aggregate variables that may move on distinct time scales in the simple model system, and, in addition, we can interpret these variables in terms of their metabolic physiological significance. However, in general, time scale decomposition is more complex as the concentrations that influence the rate laws may move on many time scales and the arguments in the rate law functions must be pooled as well.

###### Using ratios of aggregate variables to describe metabolic physiology¶

We can define an aggregate variable that represents the *capacity* to carry high-energy phosphate bonds. That simply is the summation of \(\text{ATP} + \text{ADP} + \text{AMP}.\) This number multiplied by 2 would be the total number of high energy phosphate bonds that can be stored in this system. The second variable that we can define here would be the *occupancy* of that capacity, \(\textit{2ATP + ADP}\), which is simply an enumeration of how much of that capacity is occupied by
high-energy phosphate bonds. Notice that the occupancy variable has a conjugate pair, which would be the vacancy variable. The ratio of these two aggregate variables forms a charge

\[\begin{equation} \text{charge} = \frac{\text{occupancy}}{\text{capacity}} \tag{2.17} \end{equation}\]

called the *energy charge,* given by

\[\begin{equation} \text{E.C} = \frac{2 \text{ATP} \ + \ \text{ADP}}{2(\text{ATP} \ + \ \text{ADP} \ + \ \text{AMP})} \tag{2.18} \end{equation}\]

which is a variable that varies between 0 and 1. This quantity is the *energy charge* defined by Daniel Atkinson (Atkinson, 1968). In cells, the typical numerical range for this variable when measured is 0.80-0.90.

In a similar way, one can define other redox charges. For instance, the *catabolic redox charge* on the NADH carrier can be defined as

\[\begin{equation} \text{C.R.C} = \frac{\text{NADH}}{\text{NADH} \ + \ \text{NAD}} \tag{2.19} \end{equation}\]

which simply is the fraction of the NAD pool that is in the reduced form of NADH. It typically has a low numerical value in cells, i.e., about 0.001-0.0025, and therefore this pool is typically discharged by passing the redox potential to the electron transfer system (ETS). The *anabolic redox charge*

\[\begin{equation} \text{A.R.C} = \frac{\text{NADPH}}{\text{NADPH} \ + \ \text{NADP}} \tag{2.20} \end{equation}\]

in contrast, tends to be in the range of 0.5 or higher, and thus this pool is charged and ready to drive biosynthetic reactions. Therefore, pooling variables together based on a time scale hierarchy and chemical characteristics can lead to aggregate variables that are physiologically meaningful.

In Chapter 8 we further explore these fundamental concepts of time scale hierarchy. They are then used in Parts III and IV in interpreting the dynamic states of realistic biological networks.

##### Time Scale Decomposition¶

###### Reduction in dimensionality¶

As illustrated by the examples given in the previous section, most biochemical reaction networks are characterized by many time constants. Typically, these time constants are of very different orders of magnitude. The hierarchy of time constants can be represented by the time axis, Figure 2.6. Fast transients are characterized by the processes at the extreme left and slow transients at the extreme right. The process time scale, i.e., the time scale of interest, can be represented by a *window of
observation* on this time axis. Typically, we have three principal ranges of time constants of interest if we want to focus on a limited set of events taking place in a network. We can thus decompose the system response in time. To characterize network dynamics completely we would have to study all the time constants.

**Figure 2.6:** Schematic illustration of network transients that overlap with the time span of observation. n, n + 1, … represent the decadic order of time constants.

###### Three principal time constants¶

One can readily conceptualize this by looking at a three-dimensional linear system where the first time constant represents the fast motion, the second represents the time scale of interest, and the third is a slow motion, see Figure 2.7. The general solution to a three-dimensional linear system is

\[\begin{split}\begin{align} \textbf{x}(t) &=\textbf{v}\_1 \langle \textbf{u}\_1, \ \textbf{x}\_0 \rangle \ \text{exp}(\lambda\_1 t) && \text{fast}\\ &+\textbf{v}\_2 \langle \textbf{u}\_2, \ \textbf{x}\_0 \rangle \ \text{exp}(\lambda\_2 t) && \text{intermediate} \\ &+\textbf{v}\_3 \langle \textbf{u}\_3, \ \textbf{x}\_0 \rangle \ \text{exp}(\lambda\_3 t) && \text{slow} \end{align} \tag{2.21}\end{split}\]

where \(\textbf{v}\_i\) are the *eigenvectors,* \(\textbf{u}\_i\) are the *eigenrows,* and \(\boldsymbol{\lambda}\_i\) are the *eigenvalues* of the Jacobian matrix. The eigenvalues are negative reciprocals of time constants.

The terms that have time constants faster than the observed window can be eliminated from the dynamic description as these terms are small. However, the mechanisms which have transients slower than the observed time exhibit high “inertia” and hardly move from their initial state and can be considered constants.

**Figure 2.7:** A schematic of a decay comprised of three dynamic modes with well-separated time constants.

###### Example: 3D motion simplifying to a 2D motion¶

Figure 2.8 illustrates a three-dimensional space where there is rapid motion into a slow two-dimensional subspace. The motion in the slow subspace is spanned by two “slow” eigenvectors, whereas the fast motion is in the direction of the “fast” eigenvector.

**Figure 2.8:** Fast motion into a two-dimensional subspace.

###### Multiple time scales¶

In reality there are many more than three time scales in a realistic network. In metabolic systems there are typically many time scales and a hierarchical formation of pools, Figure 2.9. The formation of such hierarchies will be discussed in Parts III and IV of the text.

**Figure 2.9:** Multiple time scales in a metabolic network and the process of pool formation. This figure represents human folate metabolism. (a) A map of the folate network. (b) An illustration progressive pool formation. Beyond the first time scale pools form between CHF and CH2F; and 5MTHF, 10FTHF, SAM; and MET and SAH (these are abbreviations for the long, full names of these metabolites). DHF and THF form a pool beyond the second time scale. Beyond the third time scale CH2F/CHF join the
5MTHF/10FTHF/SAM pool. Beyond the fourth time scale HCY joins the MET/SAH pool. Ultimately, on time scales on the order of a minute and slower, interactions between the pools of folate carriers and methionine metabolites interact. Courtesy of Neema Jamshidi (Jamshidi, 2008a).

##### Network Structure versus Dynamics¶

The stoichiometric matrix represents the topological structure of the network, and this structure has significant implications with respect to what dynamic states a network can take. Its null spaces give us information about pathways and pools. It also determines the structural features of the gradient matrix. Network topology can have a dominant effect on network dynamics.

###### The null spaces of the stoichiometric matrix¶

Any matrix has a right and a left null space. The right null space, normally called just the null space, is defined by all vectors that give zero when post-multiplying that matrix:

\[\begin{equation} \textbf{Sv}=0 \tag{2.22} \end{equation}\]

The null space thus contains all the steady state flux solutions for the network. The null space can be spanned by a set of basis vectors that are pathway vectors (SB1).

The left null space is defined by all vectors that give zero when pre-multiplying that matrix:

\[\begin{equation} \textbf{lS}=0 \tag{2.23} \end{equation}\]

These vectors \(\textbf{l}\) correspond to pools that are always conserved at all time scales. We will call them *time invariants*. Throughout the book we will look at these properties of the stoichiometric matrices that describe the networks being studied.

**Figure 2.10:** A schematic showing how the structure of \(\textbf{S}\) and \(\textbf{G}\) form matrices that have non-zero elements in the same location if one of these matrices is transposed. The columns of \(\textbf{S}\) and the rows of \(\textbf{G}\) have similar but not identical vectors in an n-dimensional space. Note that this similarity only holds once the two opposing elementary reactions have been combined into a net reaction.

###### The structure of the gradient matrix¶

We will now examine some of the properties of \(\textbf{G}\). If a compound \(x\_i\) participates in reaction \(v\_j\), then the entry \(s\_{i,j}\) is non-zero. Thus, a net reaction

\[\begin{equation} x\_i + x\_{i + 1} \stackrel{v\_j}{\leftrightharpoons} x\_{i + 2} \tag{2.24} \end{equation}\]

with a net reaction rate

\[\begin{equation} v\_j = v\_j^+ - v\_j^- \tag{2.25} \end{equation}\]

generates three non-zero entries in \(\textbf{S}\): \(s\_{i,j}\), \(s\_{i + 1,j}\), and \(s\_{i + 2,j}\). Since compounds \(x\_i\), \(x\_{i + 1}\), and \(x\_{i + 2}\) influence reaction \(v\_j\), they will also generate non-zero elements in \(\textbf{G}\), see Figure 2.10. Thus, non-zero elements generated by the reactions are:

\[\begin{equation} g\_{j, i} = \frac{\partial v\_j}{\partial x\_i}, \ g\_{j, i + 1} = \frac{\partial v\_j}{\partial x\_{i + 1}}, \ \text{and} \ g\_{j, i + 2} = \frac{\partial v\_j}{\partial x\_{i + 2}} \tag{2.26} \end{equation}\]

In general, every reaction in a network is a reversible reaction. Hence we have the the following relationships between the elements of \(\textbf{S}\) and \(\textbf{G}\):

\[\begin{split}\begin{align} \text{if} \ &s\_{i, j} = 0 \ \text{then} \ g\_{j, i} = 0 \\ \text{if} \ &s\_{i, j} \ne 0 \ \text{then} \ g\_{j, i} \ne 0 \\ \text{if} \ &s\_{i, j} > 0 \ \text{then} \ g\_{j, i} < 0 \\ \text{if} \ &s\_{i, j} < 0 \ \text{then} \ g\_{j, i} >0 \end{align}\end{split}\]

Note that for the rare cases where a reaction is effectively irreversible, an element in \(\textbf{G}\) can become very small, but in principle finite.

It can thus be seen that

\[\begin{equation} -\textbf{G}^T \ \tilde \ \ \textbf{S} \tag{2.27} \end{equation}\]

in the sense that both will have non-zero elements in the same location. These elements will have opposite signs.

###### Stoichiometric autocatalysis¶

The fundamental structure of most catabolic pathways in a cell is such that a compound is imported into a cell and then some property stored on cofactors is transferred to the compound and the molecule is thus “charged” with this property. This charged form is then degraded into a waste product that is secreted from the cell. During that degradation process, the property that the molecule was charged with is re-extracted from the compound, often in larger quantities than was used in the initial
charging of the compound. This pathway structure is the cellular equivalent of “it takes money to make money,” and its basic network structure is in Figure 2.11.

**Figure 2.11:** The prototypic pathway structure for degradation of a carbon substrate.

This figure illustrates the import of a substrate, \(S\), to a cell. It is charged with high-energy phosphate bonds to form an intermediate, \(X\). \(X\) is then subsequently degraded to a waste product, \(W\), that is secreted. In the degradation process, ATP is recouped in a larger quantity than was used in the charging process. This means that there is a net production of ATP in the two steps, and that difference can be used to drive various load functions on metabolism.

The consequence of this schema is basically *stoichiometric autocatalysis* that can lead to multiple steady states. The rate of formation of \(\text{ATP}\) from this schema as balanced by the load parameters is illustrated in Figure 2.12. This figure shows that the \(\text{ATP}\) generation is 0 if all the adenosine phosphates are in the form of \(\text{ATP}\) because there is no \(\text{ADP}\) to drive the conversion of X to W. The \(\text{ATP}\) generation is also 0 if
there is no \(\text{ATP}\) available, because \(S\) cannot be charged to form \(X\). The curve in between \(\text{ATP} = 0\) and \(\text{ATP} = \text{ATP}\_{max}\) will be positive. The \(\text{ATP}\) load, or use rate, will be a curve that grows with \(\text{ATP}\) concentration and is sketched here as a hyperbolic function. As shown, there are three intersections in this curve, with the upper stable steady-state being the physiological state of this system. This
system can thus have multiple steady-states and this property is a consequence of the topological structure of this reaction network.

###### Network structure¶

The three topics discussed in this section show that the stoichiometric matrix has a dominant effect on integrated network functions and sets constraints on the dynamic states that a network can achieve. The numerical values of the elements of the gradient matrix determine which of these states are chosen.

##### Physico-Chemical Effects¶

Molecules have other physico-chemical properties besides the collision rates that are used in kinetic theory. They also have osmotic properties and are electrically charged. Both of these features influence dynamic descriptions of biochemical reaction networks.

###### The constant volume assumption¶

Most systems that we identify in systems biology correspond to some biological entity. Such entities may be an organelle like the nucleus or the mitochondria, or it may be the whole cell, as illustrated in Figure 2.13.

A compound, \(x\_i\), internal to the system, has a mass balance on the total amount per cell. We denote this quantity with an \(M\_i\). \(M\_i\) is a product of the volume per cell, \(V\), and the concentration of the compound, \(x\_i\), which is amount per volume

\[\begin{equation} M\_i = V \ x\_i \tag{2.28} \end{equation}\]

The time derivative of the amount per cell is given by:

\[\begin{equation} \frac{M\_i}{dt} = \frac{d}{dt}(V \ x\_i) = V \frac{d x\_i}{dt} + x\_i \frac{dV}{dt} \tag{2.29} \end{equation}\]

**Figure 2.13:** An illustration of a ‘system’ with a defined boundary, inputs and outputs, and an internal network of reactions. The \(V\) volume of the system may change over time. \(\Pi\) denotes osmotic pressure, see (Eq. 2.32).

The time change of the amount \(M\_i\) per cell is thus dependent on two dynamic variables. One is \(dx\_i/dt\) which is the time change in the concentration of \(x\_i\), and the second is \(dV/dt\) which is the change in volume with time. The volume is typically taken to be time invariant and therefore the term \(dV/dt\) is equal to 0 and therefore results in a system that is of *constant volume*. In this case

\[\begin{equation} \frac{d x\_i}{dt} = \frac{1}{V}\frac{d M\_i}{dt} \tag{2.30} \end{equation}\]

This constant volume assumption (recall Table 1.2) needs to be carefully scrutinized when one builds kinetic models since volumes of cellular compartments tend to fluctuate and such fluctuations can be very important. Very few kinetic models in the current literature account for volume variation because it is mathematically challenging and numerically difficult to deal with. A few kinetic models have appeared, however, that do take volume fluctuations into account (Joshi, 1989m and Klipp, 2005).

###### Osmotic balance¶

Molecules come with osmotic pressure, electrical charge, and other properties, all of which impact the dynamic states of networks. For instance, in cells that do not have rigid walls, the osmotic pressure has to be balanced inside \((\Pi\_{in})\) and outside \((\Pi\_{out})\) of the cell (Figure 2.13), i.e.,

\[\begin{equation} \Pi\_{in} = \Pi\_{out} \tag{2.31} \end{equation}\]

At first approximation, osmotic pressure is proportional to the total solute concentration,

\[\begin{equation} \Pi = R T \sum\_i x\_i \tag{2.32} \end{equation}\]

although some compounds are more osmotically-active than others and have osmotic coefficients that are not unity. The consequences are that if a reaction takes one molecule and splits it into two, the reaction comes with an increase in osmotic pressure that will impact the total solute concentration allowable inside the cell, as it needs to be balanced relative to that outside the cell. Osmotic balance equations are algebraic equations that are often complicated and therefore are often
conveniently ignored in the formulation of a kinetic model.

###### Electroneutrality¶

Another constraint on dynamic network models is the accounting for electrical charge. Molecules tend to be charged positively or negatively. Elementary charges cannot be separated, and therefore the total number of positive and negative charges within a compartment must balance. Any import and export in and out of a compartment of a charged species has to be counterbalanced by the equivalent number of molecules of the opposite charge crossing the membrane. Typically, bilipid membranes are
impermeable to cations, but permeable to anions. For instance, the deliberate displacement of sodium and potassium by the ATP-driven sodium potassium pump is typically balanced by chloride ions migrating in and out of a cell or a compartment leading to a state of electroneutrality both inside and outside the cell. The equations that describe electroneutrality are basically a summation of the charge, \(z\_i\), of a molecule multiplied by its concentration,

\[\begin{equation} \sum\_i z\_ix\_i = 0 \tag{2.33} \end{equation}\]

and such terms are summed up over all the species in a compartment. That sum has to add up to 0 to maintain electroneutrality. Since that summation includes concentrations of species, it represents an algebraic equation that is a constraint on the allowable concentration states of a network.

##### Summary¶

- Time constants are key quantities in dynamic analysis. Large biochemical reaction networks typically have a broad spectrum of time constants.
- Well-separated time constants lead to pooling of variables to form aggregates. Aggregate variables represent a coarse-grained (i.e., lower dimensional) view of network dynamics and can lead to physiologically meaningful variables.
- Elementary reactions and mass action kinetics are the irreducible events in dynamic descriptions of networks. Elementary reactions are often combined into reaction mechanisms from which rate laws are derived using simplifying assumptions.
- Network structure has an overarching effect on network dynamics. Certain physico-chemical effects can as well. Thus topological analysis is useful, and so is a careful examination of the assumptions (recall Table 1.2) that underlie the dynamic mass balances (Eq. (1.1)) for the system being modeled and simulated.

\(\tiny{\text{© B. Ø. Palsson 2011;}\ \text{This publication is in copyright.}\\ \text{Subject to statutory exception and to the provisions of relevant collective licensing agreements,}\\ \text{no reproduction of any part may take place without the written permission of Cambridge University Press.}}\)

#### Dynamic Simulation: The Basic Procedure¶

Once a set of dynamic mass balance equations has been formulated, they can be numerically solved, and thus the behavior of a network can be simulated in response to environmental and genetic changes. Simulation results can be obtained using a number of different software packages. Dynamic simulation generates the time dependent behavior of the concentrations, i.e., \(\textbf{x}\)(t). This solution can be obtained in response to several different types of perturbations and the results
graphically displayed. The basic principles and procedures associated with dynamic simulation are covered in this chapter. The following three chapters then apply the simulation process to a set of simple but progressively more complex and relevant examples.

##### Numerical Solutions¶

Network dynamics are described by a set of ordinary differential equations (ODEs): the dynamic mass balance equations; see Eq. (1.1). To obtain the dynamic solutions, we need three things: first, the equations themselves; second, the numerical values for the kinetic constants that are in the equations; and third, the initial conditions and parameters that are being perturbed. We describe each briefly.

**1.** To formulate the mass balances we have to specify the system boundary, the fluxes in and out of the system, and the reactions that take place in the network. From the set of reactions that are taking place, a stoichiometric matrix is formed. This matrix is then put into Eq. (1.1) . One can multiply out the individual dynamic mass balances, as was done in Eq. (2.13) for the adenosine phosphate network, to prevent a large number of numerical operations that involve multiplication of
reaction rates by the zero elements in \(\textbf{S}\). The reaction rate laws for the reactions are then identified and substituted into the equations. Typically, one would use elementary kinetics as shown in Eq. (2.6), or apply more complex rate laws if they are appropriate and available. This process leads to the definition of the dynamic mass balances.

**2.** The numerical values for the kinetic parameters in the rate laws have to be specified, as do any imposed fluxes across the system boundary. Obtaining numerical values for the kinetic constants is typically difficult. They are put into a parameter vector designated by \(\textbf{k}\). In select cases, detailed kinetic characterization has been carried out. More often, though, one only knows these values approximately. It is important to make sure that all units are consistent throughout
the equations and that the numerical values used are in the appropriate units.

**3.** With the equations and numerical values for the kinetic constants specified \((\textbf{k})\), we can simulate the responses of the network that they represent. To do so, we have to set initial conditions \((x\_0)\). This leads to the numerical solution of

\[\begin{equation} \frac{d\textbf{x}}{dt} = \textbf{Sv(x;k)},\ \textbf{x}(t = 0) = \textbf{x}\_0 \tag{3.1} \end{equation}\]

There are three conditions that are typically considered.

1. First, the initial conditions for the concentrations are set, and the motion of the network into a steady state (open system) or equilibrium (closed system) is simulated. This scenario is typically physiologically unrealistic since individual concentrations cannot simply change individually in a living cell.
2. Second, a change in an input flux is imposed on a network that is in a steady state. This scenario can be used to simulate the response of a cell to a change in its environment.
3. Third, a change in a kinetic parameter is implemented at the initial time. The initial concentrations are typically set at the steady state values with the nominal value of the parameter. The equations are then simulated to a long time to obtain the steady state values that correspond to the altered kinetic parameters. These are set as the initial conditions when examining the responses of the system with the altered kinetic properties.

**4.** Once the solution has been obtained it can be graphically displayed and the results analyzed. There are several ways to accomplish this step, as detailed in the next two sections. The analysis of the results can lead to post-processing of the output to form an alternative set of dynamic variables.

The simulation is implemented using a numerical solver. Currently, such implementation is carried out using standard and readily available software, such as Mathematica or MATLAB. Specialized simulation packages are also available (Table 3.1). After the simulation is set up and the conditions specified, the software computes the concentrations as a function of time. The output is a file that contains the numerical values of the concentrations at a series of time points (Figure 3.1). This set of
numbers is typically graphically displayed, and/or used for subsequent computations.

**Table 3.1:** Available software for dynamic simulation. Assembled by Neema Jamshidi.

We will be using iPython notebooks using the python software package **MASSpy** as our simulation software.

##### Graphically Displaying the Solution¶

The simulation procedure described in the previous section results in a file that contains the concentrations as a function of time (Figure 3.1). These results are graphically displayed, typically in two ways: by plotting the concentrations as a function of time, or by plotting two concentrations against one another with time as a parameter along the trajectory.

**Figure 3.1:** The fundamental structure of the file \(\textbf{x}\)(t) that results from a numerical simulation. The two vertical bars show the list of values that would be used to compute \(\sigma\_{12}\)(2) (see Eq. 3.8); that is, the correlation between \(x\_1\) and \(x\_2\) with a time lag of 2.

Before describing these methods, we observe certain fundamental aspects of the equations that we are solving. The dynamic mass balances can be expanded as:

\[\begin{equation} \frac{d\textbf{x}}{dt} = \textbf{Sv(x)} = \sum\textbf{s}\_i v\_i(\textbf{x}) \tag{3.2} \end{equation}\]

In other words, the time derivatives are linear combinations of the reaction vectors \((\textbf{s}\_i)\) weighted by the reaction rates, that in turn change with the concentrations that are time varying. Thus, the motions are linear combinations of the directions specified by \(\textbf{s}\_i\). This characteristic is important because if the \(v\_i\). have different time constants, the motion can be decomposed in time along these reaction vectors.

###### Time Profiles¶

The simulation results in a file that contains the vector \(\textbf{x}\)(t) and the time points at which the numerical values for the concentrations are given. These time points can be specified by the user or are automatically generated by the solver used. Typically, the user specifies the initial time, the final time, and sometimes the time increment between the time points where the simulator stores the computed concentration values in the file. The results can then be graphically
displayed depending on a few features of the solution. Some of these are shown in Figure 3.2 and are now briefly described:

- Panel A: The most common way to display a dynamic solution is to plot the concentration as a function of time.
- Panel B: If there are many concentration variables they are often displayed on the same graph.
- Panel C: In many cases there are different response times and one plots multiple time profiles where the x-axis on each plot is scaled to a particular response time. Alternatively, one can use a logarithmic scale for time.
- Panel D: If a variable moves on many time scales changing over many orders of magnitude, the y-axis is often displayed on a logarithmic scale.

**Figure 3.2:** Graphing concentrations over time. (a) A single concentration shown as a function of time. (b) Many concentrations shown as a function of time. (c) A single concentration shown as a function of time separately on different time scales. (d) The logarithm of a single concentration shown as a function of time to distinguish the decay on different time scales.

The solution can thus be displayed in different ways depending on the characteristics of the time profiles. One normally plays with these representations to get an understanding of the responses of the network that they have formulated and to represent the features in which one is interested.

###### Dynamic phase portraits¶

Dynamic phase portraits represent trajectories formed when two concentrations plotted against each other, parameterized with respect to time (Figure 3.3). The dynamic trajectories in the diagram move from an initial state to a final state. Analysis of these trajectories can point to key dynamic relationships between compounds in a biochemical reaction network. For example, if a system is dynamically stable, the dynamic trajectories will converge to a single point in the plane, known as an
attracting fixed pointattracting fixed point. A stable steady-state point would represent a homeostatic state. Conversely, if the system is unstable, the trajectories will not approach a fixed point but diverge away from it. The former is essentially always the case for biochemical reaction networks representing real cells. The way the trajectories converge on the steady state is highly informative as different dynamic characteristics are evident from the trajectory.

**Figure 3.3:** A dynamic phase portrait.

###### Characteristic features of phase portraits¶

A trajectory in the phase portraitphase portrait may indicate the presence of one or more general dynamic features. Namely, the shapes of the trajectories contain significant information about the dynamic characteristics of a network. Some important features of trajectories in a phase portrait are shown in Figure 3.4

**Figure 3.4:** General features of dynamic phase portraits. Dynamic phase portraits are formed by graphing the time dependent concentrations of two concentrations \((x\_1\) and \(x\_2)\) against one another. Phase portraits have certain characteristic features. (a) Conservation relationship. (b) A pair of concentrations that could be in quasi-equilibrium with one another. (c) Motion of the two concentrations dynamically independent of one another. (d) Closed loop traces representing
either a periodic motion or a return to the original steady state. Modified from Kauffman 2002 [64].

1. When the trajectory has a negative slope, it indicates that one concentration is increasing while the other is decreasing. The concentrations are moving on the same time scales but in opposite directions; that is, one is consumed while the other is produced. This feature might represent the substrate concentration versus the product concentration of a given reaction. Such behavior helps define aggregate concentration variablesaggregate concentration variables.
2. When a trajectory in the phase portrait between two concentrations is a straight line with a positive slope, it means that the two concentrations are moving in tandem; i.e., as one increases so does the other. This feature is observed when two or more concentrations move on the same time scales and are in quasi-equilibrium with one another. Such behavior helps define aggregate concentration variables.
3. When a trajectory is vertical or horizontal, it indicates that one of the concentrations is changing while the other remains constant. This feature implies either that the motions of the concentrations during the trajectory are independent of one another or that the dynamic motions of the concentrations progress on different characteristic time scales. Such behavior helps define time scale decomposition.
4. When a trajectory forms a closed loop, it implies one of two possibilities. The system never converges to a steady state over time but oscillates forming a closed loop trajectory. On the other hand, if the orbit begins at one point, moves away from it, then returns to the same point after a sufficiently long time interval, then it implies that a change in another variable in the system forced it away from its steady state temporarily, but it returned to the original steady state. Such
   behavior helps define disturbance rejection characteristics.

**Figure 3.5:** A schematic of a tiled phase portrait.The matrix is symmetric, making it possible to display statistical information about a phase portrait in the mirror position.The diagonal elements are meaningless.Originally developed in Kauffman 2002 [64].

The qualitative characteristics of dynamic phase portraitsphase portrait can provide insight into the dynamic features of a network. A trajectory may have more than one of these basic features. For instance, there can be a fast independent motion (i.e., a horizontal phase portrait trajectory) followed by a line with a positive slope after an equilibrium state has been reached.

###### Tiling dynamic phase portraits¶

Phase portraits show the dynamic relationships between two variables on multiple time scales, see Figure 3.5. If a system has \(n\) variables, then there are \(n^2\) dynamic phase portraits. All pair-wise phase portraits can be tiled in a matrix form where the \(\textit{i}\), \(\textit{j}\) entry represents the dynamic phase portrait between variables \(x\_i\) and \(x\_j\). Note that such an array is symmetric and that the diagonal elements are un-informative. Thus, there
are \((n^2-n)/2\) phase portraits of interest. This feature of this graphical representation opens the possibility of putting the phase portrait in the \(\textit{i}\), \(\textit{j}\) position in the array and showing other information (such as a regression coefficient or a slope) in the corresponding \(\textit{j}\), \(\textit{i}\) position.

Since the time scales in biochemical reaction networks typically vary over many orders of magnitude, it often makes sense to make a series of tiled phase portraits, each of which represents a key time scale. For instance, rapid equilibration leads to straight lines with positive slopes in the phase portrait (Figure 3.4b) where the slope is the equilibrium constant of the reaction. This may be one of many dynamic events taking place. If a phase portrait is graphed separately on this time scale
alone, the positive line will show up with a high regression coefficient and a slope that corresponds to the equilibrium constant.

##### Post-Processing the Solution¶

The initial suggestions obtained from graphing and visualizing the concentration vector \(\textbf{x}\)(t) can lead to a more formal analysis of the results. We describe three post-processing procedures of \(\textbf{x}\)(t).

###### Computing the fluxes from the concentration variables:¶

The solution for the concentrations \(\textbf{x}\)(t)can be used to compute the fluxes from

\[\begin{equation} \textbf{v}(t)= \textbf{v}(\textbf{x}(t)) \tag{3.3} \end{equation}\]

and subsequently we can plot the fluxes in the same way as the concentrations. Graphical information about both the \(\textbf{x}\)(t) and \(\textbf{v}\)(t) is useful.

###### Combining concentrations to form aggregate variables:¶

The graphical and statistical multi-time scale analysis discussed above may lead to the identification of aggregate variables. Pooled variables, p, are computed from

\[\begin{equation} \textbf{p}(t)= \textbf{Px}(t)) \tag{3.4} \end{equation}\]

where the pool transformation matrix, \(\textbf{P}\), defines the linear combination of the concentration variables that forms the aggregate variables. For instance, if we find that a logical way to pool two variables, \(x\_1\) and \(x\_2\), into new aggregate variables is \(p\_1 = x\_1 + x\_2\) and \(p\_2 = x\_1 - x\_2\), then we form the following matrix equation describing these relationships as:

\[\begin{split}\begin{equation} \textbf{p}(t) = \textbf{Px}(t) = \begin{pmatrix} {p\_1(t)} \\ {p\_2(t)} \end{pmatrix} = \begin{pmatrix} {1} & {1} \\ {1} & {-1} \end{pmatrix} \begin{pmatrix} {x\_1(t)} \\ {x\_2(t)} \end{pmatrix} = \begin{pmatrix} {x\_1(t) + x\_2(t)} \\ {x\_1(t) - x\_2(t)} \end{pmatrix} \end{equation}\end{split}\]

The dynamic variables, \(\textbf{p}\)(t), can be graphically studied as described in the previous section.

###### Example: The Phosphorylated Adenosines¶

The pool formation discussed in Chapter 2 can be described by the pool transformation matrix:

\[\begin{split}\begin{equation} \textbf{P} = \begin{pmatrix} {1} & {1} & {0} \\ {2} & {1} & {0} \\ {1} & {1} & {1} \end{pmatrix} \end{equation} \tag{3.5}\end{split}\]

and thus

\[\begin{split}\begin{equation} \textbf{p} = \textbf{Px} = \textbf{P}\begin{pmatrix} {\text{ATP}} \\ {\text{ADP}} \\ {\text{AMP}} \end{pmatrix} = \begin{pmatrix} {\text{ATP} + \text{ADP}} \\ {2 \text{ATP} + \text{ADP}} \\ {\text{ATP} + \text{ADP} + \text{AMP}} \end{pmatrix} \end{equation} \tag{3.6}\end{split}\]

The pool sizes \(p\_i\)(t) can then be graphed as a function of time.

###### Correlating concentrations over time:¶

One can construct the time-separated correlation matrix, \(\textbf{R}\), based on a time scale structure of a system. In this matrix, we compute the correlation between two concentrations on a time scale as:

\[\begin{equation} \textbf{R}(\tau) = (r\_{ij}) = \frac{\sigma\_{ij}(\tau)}{\sqrt{\sigma\_{ii}\sigma\_{jj}}} \tag{3.7} \end{equation}\]

in which \(\sigma\_{ii}\) is the variance of the dataset \(x\_i(k)\) and \(\sigma\_{ij}(\tau)\) is the time-lagged covariance between the discrete, uniformly sampled datasets \(x\_i(k)\) and \(x\_j(k + \tau)\), determined as,

\[\begin{equation} \sigma\_{ij}(\tau) = \frac{1}{n}\sum\limits\_{k=1}^{n-\tau} (x\_i(k) - \bar{x\_i})(x\_j(k + \tau) - \bar{x\_j}) \tag{3.8} \end{equation}\]

in which \(n\) is the number of data points in the series, and \(\bar{x\_i}\) indicates the average value of the series \(x\_i\). The values in \(\textbf{R}\) range from -1 to 1, indicating perfect anti-correlation or correlation, respectively, between two datasets with a delay of time steps. Elements in \(\textbf{R}\) equal to zero indicate that the two corresponding datasets are completely uncorrelated. If such correlation computations were done for the cases shown in Figure
3.4, one would expect to find a strong negative correlation for the data shown in Figure 3.4a, a strong positive correlation for Figure 3.4b, and no correlation for Figure 3.4c,

The correlation computations can be performed with an increment, \(\tau\), offset in time between two concentrations. An example of a time offset is shown in Figure 3.2 showing the values used from the output file to compute the correlation between \(x\_1\) and \(x\_2\) with a time lag of 2.

The matrix of phase portraits is symmetric with uninformative diagonal elements. One can therefore enter a correlation coefficient corresponding to a particular phase portrait in the transpose position to the phase portrait in the matrix. A correlation coefficient provides a quantitative description of the phase portrait’s linearity between the two variables over the time scale displayed. In addition to the correlation coefficient, the slope can be computed and displayed, giving the equilibrium
constant between the two compounds displayed.

##### Demonstration of the Simulation Procedure in MASSpy¶

###### Setting up the model¶

The following builds the model of three reactions in series that is described on pages 51-56 in the book. We show how the model is built, simulated, solutions graphically displayed, solutions post processed and analyzed mathematically.

To construct a model in **MASSpy**, the `MassModel`, `MassReaction`, and `MassMetabolite` objects need to be imported into the environment.

```
[1]:
```

```
from mass import MassModel, MassMetabolite, MassReaction
```

###### Defining metabolites and reactions¶

One method for creating the model is to objects that represent the metabolites and reactions. Metabolite are represented by `MassMetabolite` objects, and can be created by providing a unique identifier for that object. Therefore we can define the four metabolites, \(x\_1, x\_2, x\_3\), and \(x\_4\) by the following;

```
[2]:
```

```
x1 = MassMetabolite('x1')
x2 = MassMetabolite('x2')
x3 = MassMetabolite('x3')
x4 = MassMetabolite('x4')
```

Reactions are represented by `MassReaction` objects, and like metabolites, they can be also created by providing a unique identifier for that object.

```
[3]:
```

```
v1 = MassReaction('v1')
v2 = MassReaction('v2')
```

By default, a reaction is considered reversible. However, if we wish to make an irreversible reaction, we set the `reversible` argument to `False`.

```
[4]:
```

```
v3 = MassReaction('v3', reversible=False)
```

Once the `MassReaction` objects have been created, metabolites can be added to the reaction using the `MassReaction.add_metabolites` method. To quickly see how this method is used, we can use the `help()` function. Alternatively, we can go to the API documentation and read about how the
MassReaction.add\_metabolites method works.

```
[5]:
```

```
help(MassReaction.add_metabolites)
```

```
Help on function add_metabolites in module mass.core.mass_reaction:

add_metabolites(self, metabolites_to_add, combine=True, reversibly=True)
    Add metabolites and their coefficients to the reaction.

    If the final coefficient for a metabolite is 0 then it is removed from
    the reaction.

    The change is reverted upon exit when using the :class:`~.MassModel`
    as a context.

    Notes
    -----
    * A final coefficient of < 0 implies a reactant and a final
      coefficient of > 0 implies a product.

    * Extends :meth:`~cobra.core.reaction.Reaction.add_metabolites` of the
      :class:`cobra.Reaction <cobra.core.reaction.Reaction>` by first
      ensuring that the metabolites to be added are
      :class:`.MassMetabolite`\ s and not
      :class:`cobra.Metabolites <cobra.core.metabolite.Metabolite>`.
      and error message raised reflects the :mod:`mass` object.

    * If a :class:`cobra.Metabolite <cobra.core.metabolite.Metabolite>` is
      provided. a warning is raised and a :class:`.MassMetabolite`
      will be instantiated using the
      :class:`cobra.Metabolite <cobra.core.metabolite.Metabolite>`.

    Parameters
    ----------
    metabolites_to_add : dict
        A ``dict`` with :class:`.MassMetabolite`\ s or metabolite
        identifiers as keys and stoichiometric coefficients as values. If
        keys are strings (id of a metabolite), the reaction must already
        be part of a :class:`~.MassModel` and a metabolite with the given
        id must already exist in the :class:`~.MassModel`.
    combine : bool
        Describes the behavior of existing metabolites.
        If ``True``, the metabolite coefficients are combined together.
        If ``False`` the coefficients are replaced.
    reversibly : bool
        Whether to add the change to the context to make the change
        reversible (primarily intended for internal use).

    See Also
    --------
    :meth:`subtract_metabolites`
```

To use `MassReaction.add_metabolites`, a dictionary input is required, where the `MassMetabolite` objects are keys and the value is their stoichiometric coefficient. Reactants are defined with negative coefficients, while products are defined with positive coefficients.

```
[6]:
```

```
v1.add_metabolites({x1 : -1, x2 : 1})
v2.add_metabolites({x2 : -1, x3 : 1})
v3.add_metabolites({x3 : -1, x4 : 1})
```

Reactions, e.g., \(v\_1\) can be used to define any kind of chemical transformation, association, activation etc. A series of methods are provided for inspection of the reaction.

```
[7]:
```

```
v1.id
```

```
[7]:
```

```
'v1'
```

```
[8]:
```

```
v1.reactants
```

```
[8]:
```

```
[<MassMetabolite x1 at 0x7fe452a8d290>]
```

```
[9]:
```

```
v1.products
```

```
[9]:
```

```
[<MassMetabolite x2 at 0x7fe452a8d250>]
```

```
[10]:
```

```
v1.stoichiometry
```

```
[10]:
```

```
[-1, 1]
```

```
[11]:
```

```
v1.reversible
```

```
[11]:
```

```
True
```

Check the documentation for the `MassReaction` class for further details.

###### Model Setup¶

To construct a model capable of dynamic simulation, a `MassModel` object must be created. The minimal input for creating a `MassModel` object is a unique identifier.

```
[12]:
```

```
model = MassModel('Model')
```

To add reactions and their corresponding metabolites to the model, the `MassModel.add_reactions` method can be used by providing a list of reactions to add to the model.

```
[13]:
```

```
model.add_reactions([v1, v2, v3])
```

###### Model Inspection¶

Similar to the `MassReaction` object, the `MassModel` object also has various methods that can be used to inspect the model. For example, to obtain the list of reactions and species in the system:

```
[14]:
```

```
model.reactions
```

```
[14]:
```

```
[<MassReaction v1 at 0x7fe452a8d410>,
 <MassReaction v2 at 0x7fe452a8d3d0>,
 <MassReaction v3 at 0x7fe452a8d710>]
```

```
[15]:
```

```
model.metabolites
```

```
[15]:
```

```
[<MassMetabolite x1 at 0x7fe452a8d290>,
 <MassMetabolite x2 at 0x7fe452a8d250>,
 <MassMetabolite x3 at 0x7fe452a8d2d0>,
 <MassMetabolite x4 at 0x7fe452a8d310>]
```

In some circumstances, it is helpful to iterate through a reaction and its associated metabolites using a loop:

```
[16]:
```

```
print("Model ID: %s" % model.id)
for rxn in model.reactions:
    print("\nReaction: %s\n------------" % rxn.id)
    for metab, stoichiometry in rxn.metabolites.items():
        print("%s: %s " % (metab.id, stoichiometry))
```

```
Model ID: Model

Reaction: v1
------------
x1: -1
x2: 1

Reaction: v2
------------
x3: 1
x2: -1

Reaction: v3
------------
x4: 1
x3: -1
```

To examine the stoichiometric matrix:

```
[17]:
```

```
model.S
```

```
[17]:
```

```
array([[-1.,  0.,  0.],
       [ 1., -1.,  0.],
       [ 0.,  1., -1.],
       [ 0.,  0.,  1.]])
```

The stoichiometric matrix can also be viewed as a `pandas.DataFrame` with annotated information about the metabolites and reactions.

Note: The `update_model` argument can be used to store matrix as the specified `array_type` for the next time the stoichiometric matrix is viewed.

```
[18]:
```

```
model.update_S(array_type="DataFrame", update_model=True)
```

```
[18]:
```

|  | v1 | v2 | v3 |
| --- | --- | --- | --- |
| x1 | -1.0 | 0.0 | 0.0 |
| x2 | 1.0 | -1.0 | 0.0 |
| x3 | 0.0 | 1.0 | -1.0 |
| x4 | 0.0 | 0.0 | 1.0 |

The rate equations can be examined,

```
[19]:
```

```
for rxn, rate in model.rates.items():
    print("%s: %s" % (rxn.id, rate))
```

```
v1: kf_v1*(x1(t) - x2(t)/Keq_v1)
v2: kf_v2*(x2(t) - x3(t)/Keq_v2)
v3: kf_v3*x3(t)
```

or just one rate equation can be called out:

```
[20]:
```

```
print(model.rates[v2])
```

```
kf_v2*(x2(t) - x3(t)/Keq_v2)
```

The ordinary differential equations can be also be listed in full,

```
[21]:
```

```
for metab, ode in model.odes.items():
    print("%s: %s" % (metab.id, ode))
```

```
x1: -kf_v1*(x1(t) - x2(t)/Keq_v1)
x2: kf_v1*(x1(t) - x2(t)/Keq_v1) - kf_v2*(x2(t) - x3(t)/Keq_v2)
x3: kf_v2*(x2(t) - x3(t)/Keq_v2) - kf_v3*x3(t)
x4: kf_v3*x3(t)
```

or just one ordiniary differential equation can be called out:

```
[22]:
```

```
print(model.odes[x3])
```

```
kf_v2*(x2(t) - x3(t)/Keq_v2) - kf_v3*x3(t)
```

Note that none of these expressions have been provided during the model construction process. Instead the expresions have been generated automatically from the provided list of reactions and their metabolites.

###### Set parameters and initial condtions¶

When using Jupyter notebooks, an overview of the model is rendered as a table when only the model object is called. Note that this also applies to metabolites and reactions.

```
[23]:
```

```
model
```

```
[23]:
```

|  |  |
| --- | --- |
| **Name** | Model |
| **Memory address** | 0x07fe452ab6450 |
| **Stoichiometric Matrix** | 4x3 |
| **Matrix Rank** | 3 |
| **Number of metabolites** | 4 |
| **Initial conditions defined** | 0/4 |
| **Number of reactions** | 3 |
| **Number of genes** | 0 |
| **Number of enzyme modules** | 0 |
| **Number of groups** | 0 |
| **Objective expression** | 0 |
| **Compartments** |  |

From the model overview it can be seen that no parameters or initial conditions have been defined. Parameters can be defined directly for a specific reaction:

```
[24]:
```

```
v1.forward_rate_constant = 1
v2.kf = 0.01 # Shorthand method
v3.kf = 0.0001

v1.equilibrium_constant = 1
v2.Keq = 1 # Shorthand method

for param_type, param_dict in model.parameters.items():
    print("%s: %s" %(param_type, param_dict))
```

```
kf: {'kf_v1': 1, 'kf_v2': 0.01, 'kf_v3': 0.0001}
Keq: {'Keq_v1': 1, 'Keq_v2': 1, 'Keq_v3': inf}
kr: {}
v: {}
Custom: {}
Boundary: {}
```

Initial conditions for metabolites can be defined directly for a specific metabolite,

```
[25]:
```

```
x1.initial_condition = 1
x2.ic  = 0 # Shorthand method
model.initial_conditions
```

```
[25]:
```

```
{<MassMetabolite x1 at 0x7fe452a8d290>: 1,
 <MassMetabolite x2 at 0x7fe452a8d250>: 0}
```

or a dictionary can be used to define them in a model directly. The `update_metabolites` argument will subsequently update the initial condition in the metabolite object as well.

```
[26]:
```

```
model.update_initial_conditions({x3: 0, x4:0})
model.initial_conditions
```

```
[26]:
```

```
{<MassMetabolite x1 at 0x7fe452a8d290>: 1,
 <MassMetabolite x2 at 0x7fe452a8d250>: 0,
 <MassMetabolite x3 at 0x7fe452a8d2d0>: 0,
 <MassMetabolite x4 at 0x7fe452a8d310>: 0}
```

Check the documentation for further details on the `MassModel` class.

###### Simulating Dynamic Responses¶

###### Simulate¶

Simulating the model once it is set up properly is very simple. To set up the simulation, we use a `Simulation` object. The simulation object requires a `MassModel` for initialization.

```
[27]:
```

```
from mass import Simulation
```

```
[28]:
```

```
sim = Simulation(model, verbose=True)
```

```
WARNING: No compartments found in model. Therefore creating compartment 'compartment' for entire model.
```

```
Successfully loaded MassModel 'Model' into RoadRunner.
```

The `Simulation.simulate` method from the will integrate the ordinary differential equations of the system in the provided time interval and return the dynamic responses of concentrations and fluxes.

```
[29]:
```

```
t0 = 0
tf = 1e6
numpoints = 1e3 + 1

conc_sol, flux_sol = sim.simulate(
    model, time=(t0, tf, numpoints), interpolate=True, verbose=True)
```

```
Getting time points
Setting output selections
Setting simulation values for 'Model'
Simulating 'Model'
Simulation for 'Model' successful
Adding 'Model' simulation solutions to output
Updating stored solutions
```

Note: If a model is unable to be simulated, a warning will be raised. By setting the `verbose` argument to `True`, a QC/QA report outlining inconsistencies, missing values, and other issues will also be generated and displayed to assist in diagnosing the reason why a model could not be simulated.

###### Inspect the solution¶

As the default setting, the Simulation object utilizes scipy interpolating functions to capture the concentration and flux responses (see documentation for scipy.interpolate for additional information). The `Simulation.simulate_model` method returns two `cobra.DictLists` containing specialized dictionaries known as `MassSolution` objects.

The first `MassSolution` object contains the `MassMetabolite` identifiers as keys, and their corresponding concentration solutions as values.

```
[30]:
```

```
for metabolite, solution in conc_sol.items():
    print(metabolite, solution)
```

```
x1 <scipy.interpolate.interpolate.interp1d object at 0x7fe452b5b710>
x2 <scipy.interpolate.interpolate.interp1d object at 0x7fe452b5b7d0>
x3 <scipy.interpolate.interpolate.interp1d object at 0x7fe452b5b830>
x4 <scipy.interpolate.interpolate.interp1d object at 0x7fe452b5b890>
```

Similarly, the second `MassSolution` object contains the `MassReaction` identifiers as keys, and their corresponding flux solutions as values.

```
[31]:
```

```
for reaction, solution in flux_sol.items():
    print(reaction, solution)
```

```
v1 <scipy.interpolate.interpolate.interp1d object at 0x7fe452b5b770>
v2 <scipy.interpolate.interpolate.interp1d object at 0x7fe452b5b9b0>
v3 <scipy.interpolate.interpolate.interp1d object at 0x7fe452b5b950>
```

###### Query time responses¶

The interpolating functions are functions of time. Therefore, we can evaluate the interpolating function at a specific time point using the following:

```
[32]:
```

```
time_points = 100;
for metabolite, interpolating_function in conc_sol.items():
    print("%s: %s" % (metabolite, interpolating_function(time_points)))
print()
for reaction, interpolating_function in flux_sol.items():
    print("%s: %s" % (reaction, interpolating_function(time_points)))
```

```
x1: 0.3710242389082219
x2: 0.3704524448547136
x3: 0.2569363810507253
x4: 0.0015869284328310024

v1: 0.0005717940535082393
v2: 0.0011351606380398832
v3: 2.5693638105072534e-05
```

It is also possible to get values for multiple time points at once:

```
[33]:
```

```
time_points = [0.01, 0.1, 1, 10, 100, 1000];
for metabolite, interpolating_function in conc_sol.items():
    print("%s: %s" % (metabolite, interpolating_function(time_points)))
print()
for reaction, interpolating_function in flux_sol.items():
    print("%s: %s" % (reaction, interpolating_function(time_points)))
```

```
x1: [0.99009934 0.90936384 0.56699581 0.4790072  0.37102424 0.32389592]
x2: [0.00990017 0.09058937 0.43018534 0.47682748 0.37045244 0.32388517]
x3: [4.96651050e-07 4.67952432e-05 2.81873928e-03 4.41437817e-02
 2.56936381e-01 3.21735095e-01]
x4: [1.65778860e-13 1.58583691e-10 1.07541682e-07 2.15291648e-05
 1.58692843e-03 3.04838051e-02]

v1: [9.80199167e-01 8.18774471e-01 1.36810476e-01 2.17971609e-03
 5.71794054e-04 1.07505761e-05]
v2: [9.89967148e-05 9.05425714e-04 4.27366598e-03 4.32683701e-03
 1.13516064e-03 2.15007648e-05]
v3: [4.96651050e-11 4.67952432e-09 2.81873928e-07 4.41437817e-06
 2.56936381e-05 3.21735095e-05]
```

For example, a `pandas.Dataframe` of concentration values at different time points could be generated using this method:

```
[34]:
```

```
import pandas as pd
```

```
[35]:
```

```
data = [interpolating_function(time_points)
        for interpolating_function in conc_sol.values()]
index_col = [metabolite for metabolite in conc_sol.keys()]
pd.DataFrame(data, index=index_col, columns=time_points)
```

```
[35]:
```

|  | 0.01 | 0.10 | 1.00 | 10.00 | 100.00 | 1000.00 |
| --- | --- | --- | --- | --- | --- | --- |
| x1 | 9.900993e-01 | 9.093638e-01 | 5.669958e-01 | 0.479007 | 0.371024 | 0.323896 |
| x2 | 9.900168e-03 | 9.058937e-02 | 4.301853e-01 | 0.476827 | 0.370452 | 0.323885 |
| x3 | 4.966510e-07 | 4.679524e-05 | 2.818739e-03 | 0.044144 | 0.256936 | 0.321735 |
| x4 | 1.657789e-13 | 1.585837e-10 | 1.075417e-07 | 0.000022 | 0.001587 | 0.030484 |

The same can be done for the fluxes:

```
[36]:
```

```
data = [interpolating_function(time_points)
        for interpolating_function in flux_sol.values()]
index_col = [reaction for reaction in flux_sol.keys()]
pd.DataFrame(data, index=index_col, columns=time_points)
```

```
[36]:
```

|  | 0.01 | 0.10 | 1.00 | 10.00 | 100.00 | 1000.00 |
| --- | --- | --- | --- | --- | --- | --- |
| v1 | 9.801992e-01 | 8.187745e-01 | 1.368105e-01 | 0.002180 | 0.000572 | 0.000011 |
| v2 | 9.899671e-05 | 9.054257e-04 | 4.273666e-03 | 0.004327 | 0.001135 | 0.000022 |
| v3 | 4.966510e-11 | 4.679524e-09 | 2.818739e-07 | 0.000004 | 0.000026 | 0.000032 |

###### Filtering for specific species and fluxes¶

Because concentration and flux `MassSolution` objects are specialized dictionaries, they can be handled like any other dictionary. Therefore, obtaining the solution for individual species and fluxes can be done easily by using the `MassMetabolite` or `MassReaction` identifiers as keys.

```
[37]:
```

```
print(x1.id, conc_sol[x1.id])
```

```
x1 <scipy.interpolate.interpolate.interp1d object at 0x7fe452b5b710>
```

```
[38]:
```

```
for flux in [v1, v2]:
    print(flux.id, flux_sol[flux.id])
```

```
v1 <scipy.interpolate.interpolate.interp1d object at 0x7fe452b5b770>
v2 <scipy.interpolate.interpolate.interp1d object at 0x7fe452b5b9b0>
```

###### Switching between numerical arrays and interpolating functions¶

Suppose that instead of working with interpolating functions, we would rather work with the original time points and the corresponding solutions utilized by the ODE solver. One way this can be done would be to access the original time point values stored in the Solution object, and use those in the interpolating function:

```
[39]:
```

```
time_points = conc_sol.t
# Get a slice of the first 50 points
print(conc_sol["x1"](time_points)[:50])
```

```
[1.         1.         0.99999943 0.99999734 0.99999525 0.99999316
 0.99998821 0.99997787 0.99995537 0.99990413 0.99978077 0.99942553
 0.99907055 0.99871581 0.99806461 0.99741426 0.99676475 0.9961161
 0.9948862  0.99290131 0.98987466 0.9868666  0.983877   0.98090577
 0.97795277 0.97334374 0.96877916 0.96425858 0.95727034 0.94370569
 0.92186543 0.90109967 0.88135539 0.86258222 0.84473223 0.82775987
 0.81162185 0.78756746 0.76536568 0.7448733  0.72595816 0.70849829
 0.69238106 0.67750262 0.66376717 0.64105858 0.62146897 0.6045664
 0.58997873 0.57738534]
```

To quickly convert an entire `MassSolution` object from interpolating functions to numerical arrays or vice-versa, we use the `MassSolution.interpolate` setter method:

```
[40]:
```

```
conc_sol.interpolate = False
# Get a slice of the first 50 points
conc_sol["x1"][:50]
```

```
[40]:
```

```
array([1.        , 1.        , 0.99999943, 0.99999734, 0.99999525,
       0.99999316, 0.99998821, 0.99997787, 0.99995537, 0.99990413,
       0.99978077, 0.99942553, 0.99907055, 0.99871581, 0.99806461,
       0.99741426, 0.99676475, 0.9961161 , 0.9948862 , 0.99290131,
       0.98987466, 0.9868666 , 0.983877  , 0.98090577, 0.97795277,
       0.97334374, 0.96877916, 0.96425858, 0.95727034, 0.94370569,
       0.92186543, 0.90109967, 0.88135539, 0.86258222, 0.84473223,
       0.82775987, 0.81162185, 0.78756746, 0.76536568, 0.7448733 ,
       0.72595816, 0.70849829, 0.69238106, 0.67750262, 0.66376717,
       0.64105858, 0.62146897, 0.6045664 , 0.58997873, 0.57738534])
```

```
[41]:
```

```
conc_sol.interpolate = True
conc_sol["x1"]
```

```
[41]:
```

```
<scipy.interpolate.interpolate.interp1d at 0x7fe452b77470>
```

```
[42]:
```

```
for key, value in conc_sol.items():
    print(key, value)
```

```
x1 <scipy.interpolate.interpolate.interp1d object at 0x7fe452b77470>
x2 <scipy.interpolate.interpolate.interp1d object at 0x7fe452b77650>
x3 <scipy.interpolate.interpolate.interp1d object at 0x7fe452b776b0>
x4 <scipy.interpolate.interpolate.interp1d object at 0x7fe452b77710>
```

```
[43]:
```

```
conc_sol["x1"]
```

```
[43]:
```

```
<scipy.interpolate.interpolate.interp1d at 0x7fe452b77470>
```

```
[44]:
```

```
conc_sol.x1
```

```
[44]:
```

```
<scipy.interpolate.interpolate.interp1d at 0x7fe452b77470>
```

###### Visualizing the Solution Graphically¶

Once the model has been simulated, the solutions can be visualized using the visualization tools in **MASSpy**.

```
[45]:
```

```
import matplotlib.pyplot as plt
import numpy as np

from mass.visualization import (
    plot_phase_portrait, plot_time_profile, plot_tiled_phase_portraits)
```

All visualization tools utilize the matplotlib python package. See documentation for the visualization class for more details on the available plotting kwargs.

###### Draw time course¶

Plotting the dynamic responses is straightforward using the `plot_time_profile` function:

```
[46]:
```

```
plot_time_profile(conc_sol);
```

```
[46]:
```

```
<matplotlib.axes._subplots.AxesSubplot at 0x7fe452b7f550>
```

For this model and simulation, plotting on a linear scale does not provide us information about the dyanmics at various time scales. Therefore, we can use the `plot_function` kwarg to change the scale. Let us keep a linear scale on the y-axis, but change the x-axis to a logarithmic scale.

```
[47]:
```

```
plot_time_profile(conc_sol, plot_function="semilogx");
```

```
[47]:
```

```
<matplotlib.axes._subplots.AxesSubplot at 0x7fe452d6ee10>
```

The `observable` argument allows one to specify particular solutions from the solution profile to observe while filtering out all other solutions. For example, only the solutions for \(x\_1\) and \(x\_2\) can be observed by setting observable to an list of these two keys in the solution profile.

```
[48]:
```

```
plot_time_profile(conc_sol, observable=["x1", "x2"],
                  plot_function="semilogx");
```

```
[48]:
```

```
<matplotlib.axes._subplots.AxesSubplot at 0x7fe452f51250>
```

Though the dynamic behavior is clear, the above plots do not provide any other information. Let us add axes labels, a title, and a legend to the plot.

```
[49]:
```

```
plot_time_profile(
    conc_sol, legend="right outside", plot_function="semilogx",
    xlabel="Time", ylabel="Concentration",
    title=("Concentration Solutions", {"size": "large"}));
```

```
[49]:
```

```
<matplotlib.axes._subplots.AxesSubplot at 0x7fe45312fd90>
```

###### Draw phase portraits¶

Plotting the dynamic responses against one another is also straightforward by using the `plot_phase_portrait` function:

```
[50]:
```

```
plot_phase_portrait(conc_sol, x="x1", y="x2",
                    xlabel="x1", ylabel="x2");
```

```
[50]:
```

```
<matplotlib.axes._subplots.AxesSubplot at 0x7fe453567690>
```

\(x\_1\) vs \(x\_2\): note that you can use the `annotate_time_points` argument to highlight particular time points of interest. This argument can be utilized either by providing iterable of time points of interest. The `annotate_time_points_color` can be used to set the color of the time points. To use color to distinguish time points, the number of colors should equal the number of time points specified.

```
[51]:
```

```
plot_phase_portrait(
    conc_sol, x="x1", y="x2", xlabel="x1", ylabel="x2",
    annotate_time_points=[t0, 1e-1, 1e0, 1e1, 1e3, tf],
    annotate_time_points_color= [
        "red", "green", "purple", "yellow", "cyan", "blue"],
    annotate_time_points_legend="lower outside");
```

```
[51]:
```

```
<matplotlib.axes._subplots.AxesSubplot at 0x7fe4538bbdd0>
```

All pairwise phase portraits can be generated and viewed at once in a tiled format using the `plot_tiled_phase_portrait` function:

```
[52]:
```

```
plot_tiled_phase_portraits(conc_sol,
                           annotate_time_points_legend="right outside");
```

```
[52]:
```

```
<matplotlib.axes._subplots.AxesSubplot at 0x7fe4539dee10>
```

This method is particularly useful for looking at correlations at various time scales. For example, looking at the overall behavior, a fast time timescale of (0, 1), an intermediate timescale of (3, 100), and a slow timescale of (300, 10000), we can generate the following:

```
[53]:
```

```
correlations = [
    np.empty((3, 3)).astype(str),
    np.empty((3, 3)).astype(str),
    np.empty((3, 3)).astype(str),
    np.empty((3, 3)).astype(str)]
for entry in correlations:
    entry.fill("")

fmt_str = "{0:.2f}\n{1:.2f}"
correlations[1][0, 1] = fmt_str.format(*[1, 1])
correlations[2][0, 1] = fmt_str.format(*[1, 1.02])
correlations[2][0, 2] = fmt_str.format(*[1, 0.51])
correlations[2][1, 2] = fmt_str.format(*[1, 0.5])
correlations[3][0, 1] = fmt_str.format(*[1, 1.02])
correlations[3][0, 2] = fmt_str.format(*[1, 1.02])
correlations[3][1, 2] = fmt_str.format(*[1, 1.02])

fig, axes = plt.subplots(2, 2, figsize=(10, 10))
axes = axes.flatten()

times = [(0, 400000), (0, 1), (3, 100), (300, 10000)]
titles = ["{0}\nt0={1}; tf={2}".format(label, *time)
         for label, time in zip(["(a)", "(b)", "(c)", "(d)"], times)]

for i, ax in enumerate(axes.flatten()):
    plot_tiled_phase_portraits(
        conc_sol, observable=["x1", "x2", "x3"], ax=ax,
        plot_tile_placement="lower", additional_data=correlations[i],
        time_vector=np.linspace(*times[i], int(1e6)),
        tile_xlabel_fontdict={"size": "large"},
        tile_ylabel_fontdict={"size": "large"},
        title=titles[i])
```

###### Post process the solution¶

###### Analyze pool behavior¶

In order to analyze the behavior of pools, pools can be created using the `MassSolution.make_aggregate_solution` method using the string representation of the pooling formulas. Additional parameters can also be incorporated into the pool formulation using a dictionary input for the `parameters` argument.

```
[54]:
```

```
pools = ["x1 - x2", "x1 + x2 - 2*x3", "x1 + x2 + x3"]

for i, equation_str in enumerate(pools):
    pool_id = "p" + str(i + 1)
    conc_sol.make_aggregate_solution(
        pool_id, equation=equation_str, update=True)
    print(pool_id, conc_sol[pool_id])
```

```
p1 <scipy.interpolate.interpolate.interp1d object at 0x7fe453393350>
p2 <scipy.interpolate.interpolate.interp1d object at 0x7fe452ee13b0>
p3 <scipy.interpolate.interpolate.interp1d object at 0x7fe452ee1530>
```

This method utilizes the solutions for the individual metabolites over the time range input, and then creates new solutions to represent the behavior of those pools.

```
[55]:
```

```
plot_time_profile(
    conc_sol, observable=["p1", "p2", "p3"], legend="best",
    plot_function="semilogx",
    xlabel="time",  ylabel="Concentrations",
    title=("Pool profile", {"size": "large"}));
```

```
[55]:
```

```
<matplotlib.axes._subplots.AxesSubplot at 0x7fe452ed9ad0>
```

###### Compute and plot the fluxes¶

A similar process as above can be utilized to obtain behavior of the net flux through a group of reactions. Note that the `MassSolution.make_aggregate_solution` method relies on the `sympy.sympify` function and can therefore utilize specific methods, such as the absolute value function, in the string as well.

```
[56]:
```

```
flux_sol.make_aggregate_solution(
    "v_net", equation='Abs(v1) + Abs(v2) + Abs(v3)', update=True)
```

```
[56]:
```

```
{'v_net': <scipy.interpolate.interpolate.interp1d at 0x7fe4540f5890>}
```

Again, this method obtains the solutions for the individual fluxes over the time range input, and then creates new solutions to represent the behavior of various flux combinations.

```
[57]:
```

```
plot_time_profile(
    flux_sol, observable=["v_net"], legend="best",
    plot_function="semilogx",  xlabel="time", ylabel="Fluxes",
    title=("Net Flux", {"size": "large"}));
```

```
[57]:
```

```
<matplotlib.axes._subplots.AxesSubplot at 0x7fe452df1890>
```

###### Plot phase portraits of pools¶

```
[58]:
```

```
fig, ax = plt.subplots(1, 1, figsize=(5, 5))
plot_tiled_phase_portraits(
    conc_sol, observable=["p1", "p2", "p3"], ax=ax,
    plot_tile_placement="lower",
    annotate_time_points_legend="right outside");
```

```
[58]:
```

```
<matplotlib.axes._subplots.AxesSubplot at 0x7fe42d3132d0>
```

Here, we can see that all of the defined pools are dynamically independent of one another.

##### Summary¶

- Network dynamics are described by dynamic mass balances \((d\textbf{x}/dt = \textbf{Sv}(\textbf{x}; \textbf{k}))\) that are formulated after applying a series of simplifying assumptions
- To simulate the dynamic mass balances we have to specify the numerical values of the kinetic constants \((\textbf{k})\), the initial conditions \((\textbf{x}\_0)\), and any fixed boundary fluxes.
- The equations with the initial conditions can be integrated numerically.
- The solution contains numerical values for the concentration variables at discrete time points. The solution is graphically displayed as concentrations over time, or in a phase portrait.
- The solution can be post-processed following its initial analysis to bring out special dynamic features of the network. Such features will be described in more detail in the subsequent notebooks.

\(\tiny{\text{© B. Ø. Palsson 2011;}\ \text{This publication is in copyright.}\\ \text{Subject to statutory exception and to the provisions of relevant collective licensing agreements,}\\ \text{no reproduction of any part may take place without the written permission of Cambridge University Press.}}\)

#### Chemical Reactions¶

The simulation procedure described in the previous chapter is now applied to a series of simple examples that represent chemical reactions. We first remind the reader of some key properties of chemical reactions that will show up in dynamic simulations and determine characteristics of network dynamic responses. We then go through a set of examples of chemical reactions that occur in a *closed system*. A closed system is isolated from its environment. No molecules enter or leave the system.
Reactions being carried out in the laboratory in a sealed container represent an example of closed systems. In this chapter we assign numerical values to all the parameters for illustration purposes. We start by importing **MASSpy**:

```
[1]:
```

```
from mass import (
    MassModel, MassMetabolite, MassReaction, Simulation)
from mass.visualization import plot_time_profile, plot_phase_portrait
```

Other useful packages are also imported at this time.

```
[2]:
```

```
import numpy as np
import matplotlib.pyplot as plt
```

##### Basic Properties of Reactions¶

Links between molecular components in a biochemical reaction network are given by chemical reactions or associations between chemical components. These links are therefore characterized and constrained by basic chemical rules.

###### Bi-linear reactions are prevalent in biology¶

Although there are linear reactions found in biological reaction networks, the prototypical transformations in living systems at the molecular level are bi-linear. This association involves two compounds coming together to either be chemically transformed through the breakage and formation of a new covalent bond, as is typical of metabolic reactions or macromolecular synthesis,

\[\begin{equation\*} \text{X} +\text{Y} \rightleftharpoons \text{X-Y}\ \text{covalent bonds} \end{equation\*}\]

or two molecules associated together to form a complex that may be held together by hydrogen bonds and/or other physical association forces to form a complex that has a different functionality than individual components,

\[\begin{equation\*}\text{X} +\text{Y} \rightleftharpoons \text{X:Y}\ \text{association of molecules} \end{equation\*}\]

Such association, for instance, could designate the binding of a transcription factor to DNA to form an activated site to which an activated polymerase binds. Such bi-linear association between two molecules might also involve the binding of an allosteric regulator to an allosteric enzyme that induces a conformational change in the enzyme.

###### Properties of biochemical reactions¶

Chemical transformations have three key properties that will influence the dynamic features of reaction networks and how we interpret dynamic states:

###### Stoichiometry¶

The stoichiometry of chemical reactions is fixed and is described by integral numbers counting the molecules that react and that form as a consequence of the chemical reaction. Thus, stoichiometry basically represents “digital information.” Chemical transformations are constrained by elemental and charge balancing, as well as other features. Stoichiometry is invariant between organisms for the same reactions and does not change with pressure, temperature, or other conditions. Stoichiometry gives
the primary topological properties of a biochemical reaction network.

###### Thermodynamics¶

All reactions inside a cell are governed by thermodynamics that determine the equilibrium state of a reaction. The relative rates of the forward and reverse reactions are therefore fixed by basic thermodynamic properties. Unlike stoichiometry, thermodynamic properties do change with physico-chemical conditions such as pressure and temperature. Thus the thermodynamics of transformation between small molecules in cells are fixed but condition-dependent. The thermodynamic properties of associations
between macromolecules can be changed by altering the amino acid sequence of a protein or by phosphorylation of amino acids in the interface region, or by conformational change induced by the binding of a small molecule ligand.

###### Absolute Rates¶

In contrast to stoichiometry and thermodynamics, the absolute rates of chemical reactions inside cells are highly manipulable. Highly evolved enzymes are very specific in catalyzing particular chemical transformations. Cells can thus extensively manipulate the absolute rates of reactions through changes in their DNA sequence.

All biochemical transformations are subject to the basic rules of chemistry and thermodynamics.

##### The Reversible Linear Reaction¶

We start with the reversible linear reaction:

\[\begin{equation} x\_1 \underset{v\_{-1}}{\stackrel{v\_1}{\rightleftharpoons}} x\_2 \tag{4.1} \end{equation}\]

Here we have that

\[\begin{split}\begin{equation\*} \textbf{S} = \begin{pmatrix} {-1} & {1} \\ {1} & {-1} \\ \end{pmatrix}, \textbf{v}(\textbf{x}) = \begin{pmatrix} {v\_1(x\_1)} \\ {v\_{-1}(x\_2)} \end{pmatrix} = \begin{pmatrix} {k\_1x\_1} \\ {k\_{-1}x\_2} \end{pmatrix} \end{equation\*}\end{split}\]

and thus the differential equations that we will need to simulate are:

\[\begin{equation} \frac{dx\_1}{dt} = -k\_1x\_1 + k\_{-1}x\_2, \frac{dx\_2}{dt} = k\_1x\_1 - k\_{-1}x\_2 = -\frac{dx\_1}{dt} \tag{4.2} \end{equation}\]

with the reaction rate given as the difference between two elementary reaction rates

\[\begin{equation} v\_{1, net} = v\_1 - v\_{-1} = k\_1x\_1 - k\_{-1}x\_2 = k\_1(x\_1 - x\_2/K\_1) \tag{4.3} \end{equation}\]

where \(K\_1 = k\_1/k\_{-1} = x\_{2, eq}/x\_{1, eq}\) or the ratio of the product to reactant concentrations at equilibrium, the conventional definition of an equilibrium constant in chemistry. Note that in Eq. (4.3), \(k\_1\) represents the kinetics, or the rate of change, while \((x\_1 - x\_2/K\_1)\) represents the thermodynamics measuring how far from equilibrium the system is, i.e.,\((x\_{1, eq} - x\_{2, eq}/K\_1) = 0\).

Below, a sample solution is shown for \(k\_1 = 1\) and \(k\_{-1} = 2\). These simulation results can be examined further, and they reveal three important observations; 1) the existence of a conservation quantity, 2) a thermodynamic driving force, and 3) the pooling of variables based on chemistry and thermodynamics.

```
[3]:
```

```
# Create MassModel
model = MassModel('Linear_Reversible')
# Generate the MassMetabolites
x1 = MassMetabolite("x1")
x2 = MassMetabolite("x2")
# Generate the MassReactions
v1 = MassReaction("v1")
# Add metabolites to the reaction, add reaction to the model
v1.add_metabolites({x1: -1, x2: 1})
model.add_reactions([v1])
# Set parameters and initial conditions
v1.kf = 1
v1.kr = 2
model.update_initial_conditions({x1: 1, x2: 0})
# Utilize type 2 rate law for kf and kr parameters defined
model.get_rate_expressions(rate_type=2, update_reactions=True)
model
```

```
[3]:
```

|  |  |
| --- | --- |
| **Name** | Linear\_Reversible |
| **Memory address** | 0x07fd1902d7f10 |
| **Stoichiometric Matrix** | 2x1 |
| **Matrix Rank** | 1 |
| **Number of metabolites** | 2 |
| **Initial conditions defined** | 2/2 |
| **Number of reactions** | 1 |
| **Number of genes** | 0 |
| **Number of enzyme modules** | 0 |
| **Number of groups** | 0 |
| **Objective expression** | 0 |
| **Compartments** |  |

```
[4]:
```

```
t0 = 0
tf = 2
sim = Simulation(model, verbose=True)
conc_sol, flux_sol = sim.simulate(model, time=(t0, tf), verbose=True)

# Define pools
pools = ["x1 - x2 / Keq_v1", "x1 + x2"]

for i, equation_str in enumerate(pools):
    pool_id = "p" + str(i + 1)
    conc_sol.make_aggregate_solution(
        pool_id, equation=equation_str,
        parameters={v1.Keq_str: v1.kf/v1.kr}, update=True)
```

```
WARNING: No compartments found in model. Therefore creating compartment 'compartment' for entire model.
```

```
Successfully loaded MassModel 'Linear_Reversible' into RoadRunner.
Getting time points
Setting output selections
Setting simulation values for 'Linear_Reversible'
Simulating 'Linear_Reversible'
Simulation for 'Linear_Reversible' successful
Adding 'Linear_Reversible' simulation solutions to output
Updating stored solutions
```

```
[5]:
```

```
fig_4_1 = plt.figure(figsize=(9, 6))
gs = fig_4_1.add_gridspec(nrows=2, ncols=2, width_ratios=[1, 1.5],
                          height_ratios=[1, 1])

ax1 = fig_4_1.add_subplot(gs[0, 0])
ax2 = fig_4_1.add_subplot(gs[0, 1])
ax3 = fig_4_1.add_subplot(gs[1, 1])

plot_phase_portrait(
    conc_sol, x=x1, y=x2, ax=ax1,
    xlabel=x1.id, ylabel=x2.id,
    xlim=(-0.05, 1.05), ylim=(-0.05, 1.05),
    title=("(a) Phase portrait", {"size":"large"}),
    annotate_time_points="endpoints",
    annotate_time_points_labels=True);

plot_time_profile(
    conc_sol, ax=ax2, observable=model.metabolites, legend="right outside",
    xlabel="Time", ylabel="Concentrations",
    title=("(b) Time Profiles of Species", {"size": "large"}));

plot_time_profile(
    conc_sol, ax=ax3, observable=["p1", "p2"],
    legend="right outside",
    xlabel="Time", ylabel="Concentrations",
    title=("(c) Time Profiles of Pools", {"size": "large"}));
fig_4_1.tight_layout()
```

**Figure 4.1:** Dynamic simulation of the reaction \(x\_1 \underset{v\_{-1}}{\stackrel{v\_1}{\rightleftharpoons}} x\_2\) for \(k\_1 =1\) and \(k\_{-1} = 2\), and \(x\_1(0)=1\), \(x\_2(0)=0\). (a) The phase portrait. (b) The time profiles. (c) The time profile of the pooled variables \(p\_1 = x\_1 - x\_2/K\_1\) and \(p\_1 = x\_1 + x\_2\).

###### Mass conservation:¶

The time profiles in Figure 4.1b show \(x\_1\) fall and \(x\_2\) rise to their equilibrium values. The phase portrait (Figure 4.1a) is a straight line of slope -1. This implies that

\[\begin{equation} p\_1 = x\_1 + x\_2 = \big \langle (1, 1), (x\_1, x\_2)^T \big \rangle \tag{4.4} \end{equation}\]

is a constant. This summation represents a conservation quantity that stems from the fact that as \(x\_1\) reacts, \(x\_2\) appears in an equal and opposite amount. The stoichiometric matrix is singular with a rank of 1, showing that this is a one-dimensional dynamic system. It has a left null space that is spanned by the vector \((1, 1), i.e., (1, 1) \centerdot \textbf{S} = 0\), thus \(p\_2\) is in the left null space of \(\textbf{S}\).

We also note that since \(x\_1 + x\_2\) is a constant, we can describe the concentration of \(x\_1\) as a fraction of the total mass, i.e.,

\[\begin{equation\*} f\_1 = \frac{x\_1}{x\_1 + x\_2} = \frac{x\_1}{p\_2} \end{equation\*}\]

Pool sizes and the fraction of molecules in a particular state will be used later in the text to define physiologically useful quantities.

###### Disequilibrium and the thermodynamic driving force:¶

A pooled variable:

\[\begin{equation} p\_1 = x\_1 - x\_2/K\_1 \tag{4.5} \end{equation}\]

can be formed (see Figure 4.1c). Combination of the differential equations for \(x\_1\) and \(x\_2\) leads to

\[\begin{equation} \frac{dp\_1}{dt} = -(k\_1 + k\_{-1}) p\_1 \tag{4.6} \end{equation}\]

and thus the time constant for this reaction is

\[\begin{equation} \tau\_{1} = \frac{1}{k\_1 + k\_{-1}} \tag{4.7} \end{equation}\]

Note that when \(t \rightarrow \infty, p\_1 \rightarrow 0\) and then

\[\begin{equation} \frac{x\_2}{x\_1} \rightarrow \frac{k\_1}{k\_{-1}} = K\_1 = \frac{x\_{2,eq}}{x\_{1, eq}} \tag{4.8} \end{equation}\]

the reaction has reached equilibrium. The pool \(p\_1\) thus represents a disequilibrium quantity and represents the thermodynamic driver for the reaction, see Eq. (4.3). With an initial condition of \(x\_{1, 0} = 1\) and \(K\_1 = 1/2\), the eventual concentrations \((t \rightarrow \infty)\) will be \(x\_{1, eq} = 2/3\) and \(x\_{2, eq} = 1/3\).

###### Representing dynamics with pools for reversible linear reactions¶

These considerations show that we can think about the dynamics of reaction (4.1) in terms of two pooled variables rather than the concentrations themselves. Thus, a useful pool transforming matrix for this reaction would be

\[\begin{split}\begin{equation} \textbf{P} = \begin{pmatrix} {1} & {-1/K\_1} \\ {1} & {1} \\ \end{pmatrix} \end{equation} \tag{4.9}\end{split}\]

leading to disequilibrium \((p\_1)\) and conservation \((p\_2)\) quantities associated with the reaction in Eq. (4.1). The former quantity moves on the time scale given by \(\tau\_1\) while the latter is time invariant. For practical purposes, the dynamics of the reaction have relaxed within a time duration of three to five times \(\tau\_1\) (see Figure 4.1b).

The differential equations for the pools can be obtained as

\[\begin{split}\begin{equation} \textbf{P} \frac{d\textbf{x}}{dt} = \frac{d}{dt}\begin{pmatrix} {p\_1} \\ {p\_2} \\ \end{pmatrix} = -(k\_1 + k\_{-1}) \begin{pmatrix} {x\_1 - x\_2/K\_1} \\ {0} \\ \end{pmatrix} = -(k\_1 + k\_{-1})\begin{pmatrix} {p\_1} \\ {0} \\ \end{pmatrix} \end{equation}\end{split}\]

Therefore, the conservation quantity is a constant (time derivative is zero) and the disequilibrium pool is driven by a thermodynamic driving force that is itself multiplied by \(-(k\_1 + k\_{-1})\), that is the inverse of the time constant for the reaction. Thus, the three key features of chemical reactions, the stoichiometry, thermodynamics, and kinetics, are separately accounted for.

##### The Reversible Bi-Linear Reaction¶

The reaction mechanism for the reversible bi-linear reaction is:

\[\begin{equation} x\_1 + x\_2 \underset{v\_{-1}}{\stackrel{v\_1}{\rightleftharpoons}} x\_3 \tag{4.10} \end{equation}\]

where the elementary reaction rates are

\[\begin{equation} v\_1 = k\_1x\_1x\_2, \ v\_{-1} = k\_{-1}x\_3 \tag{4.11} \end{equation}\]

The forward rate, \(v\_1\), is a non-linear function, or more specifically, a bi-linear function. The variable

\[\begin{equation} p\_1 = x\_1x\_2 - x\_3/K\_1 \tag{4.12} \end{equation}\]

represents a disequilibrium quantity. The dynamic states of this system can be computed from

\[\begin{equation} \frac{dx\_1}{dt} = -v\_1 + v\_{-1} = -k\_1x\_1x\_2 + k\_{-1}x\_3 = -k\_1(x\_1x\_2 - x\_3/K\_1) = \frac{dx\_2}{dt} = -\frac{dx\_3}{dt} \tag{4.13} \end{equation}\]

This example will be used to illustrate the essential features of a bi-linear reaction; 1) That there are two conservation quantities associated with it, 2) How to compute the equilibrium state, 3) The use of linearization and deviation variables from the equilibrium state, 4) The derivation of a single linear disequilibrium quantity, and 5) Formation of pools.

###### Conservation quantities for reversible bi-linear reactions¶

The stoichiometric matrix is

\[\begin{split}\begin{equation} S = \begin{pmatrix} {-1} & {1} \\ {-1} & {1} \\ {1} & {-1} \\ \end{pmatrix} \end{equation}\end{split}\]

The stoichiometric matrix has a rank of 1, and thus the dynamic dimension of this system is 1. Two vectors that span the left null space of **S** are (1,0,1) and (0,1,1) and the corresponding conservation quantities are:

\[\begin{equation} p\_2 = x\_1 + x\_3, \ p\_3 = x\_2 + x\_3 \tag{4.14} \end{equation}\]

This selection of conservation quantities is not unique, as one can find other sets of two vectors that span the left null space.

###### The equilibrium state for reversible bi-linear reactions¶

We can examine the equilibrium state for the specific parameter values to be used for numerical simulation below, Figure 4.2. At equilibrium, \(p\_1 \rightarrow 0\) and we have that \((K\_1 = 1)\)

\[\begin{equation} x\_{1, eq}x\_{2, eq} = x\_{3, eq} \tag{4.15} \end{equation}\]

and that

\[\begin{equation} x\_1(0) = 3 = x\_{1, eq} + x\_{3, eq}, \ \ \ x\_2(0) = 2 = x\_{2, eq} + x\_{3, eq} \tag{4.16} \end{equation}\]

These three equations can be combined to give a second order algebraic equation

\[\begin{equation} x\_{3, eq}^2 - 6x\_{3, eq} + 6 = 0 \tag{4.17} \end{equation}\]

that has a positive root that yields

\[\begin{equation} x\_{1, eq} = 1.73,\ \ x\_{2, eq} = 0.73, \ \ x\_{3, eq} = 1.27 \tag{4.18} \end{equation}\]

###### Linearization and deviation variables for reversible bi-linear reactions¶

Equation (13) can be linearized around the equilibrium point \(\textbf{x}\_{eq}\)==(1.73,0.73,1.27) to give

\[\begin{equation} \frac{dx\_1}{dt} = x\_1x\_2 - x\_3 \tag{4.19} \end{equation}\]

\[\begin{equation} \rightarrow \frac{dx\_1}{dt} = 0.73(x\_1 -1.73) + 1.73(x\_2 - 0.73) - (x\_3 - 1.27) \tag{4.20} \end{equation}\]

where a numerical value of \(k\_1\) used is unity.

###### The disequilibrium and conservation quantities for reversible bi-linear reactions¶

Equation (20) can be written in terms of deviation variables from the equilibrium state, i.e.,

\[\begin{equation} x\_i' x\_i - x\_{i, eq} \tag{4.21} \end{equation}\]

as

\[\begin{equation} \frac{dx\_1'}{dt} = 0.73x\_1' + 1.73x\_2' - x\_3' = p\_1' \tag{4.22} \end{equation}\]

which simply is the linearized version of the disequilibrium quantity in Eq.(4.12), and we have that

\[\begin{equation} \frac{dx\_2'}{dt} =\frac{dx\_1'}{dt} \ \ and \ \ \frac{dx\_3'}{dt} = -\frac{dx\_1'}{dt} \tag{4.23} \end{equation}\]

###### Representing dynamics with pools for reversible bi-linear reactions¶

We can therefore form a pool transformation matrix as:

\[\begin{split}\begin{equation} \textbf{P} = \begin{pmatrix} {0.73} & {1.73} & {-1} \\ {1} & {0} & {1} \\ {0} & {1} & {1} \\ \end{pmatrix} \end{equation} \tag{4.24}\end{split}\]

where the first pool represents the disequilibrium quantity and the second and third are conservation quantities. Now we transform the deviation variables with this matrix, i.e., \(\textbf{p'} = \textbf{Px'}\) and can look at the time derivatives of the pools

\[\begin{split}\begin{align} \frac{d\textbf{p'}}{dt} &= \textbf{P}\frac{d\textbf{x'}}{dt} \\ &= \begin{pmatrix} {-3.46} & {3.46} \\ {0} & {0} \\ {0} & {0} \\ \end{pmatrix}(0.73x\_1' + 1.73x\_2' - x\_3') \\ &= 3.46\begin{pmatrix} {1} \\ {0} \\ {0} \\ \end{pmatrix} p\_1' \end{align} \tag{4.25}\end{split}\]

This result is similar to that obtained above for the linear reversible reaction. There are two conservation pools and a disequilibrium pool that is moved by itself multiplied by a characteristic rate constant. We note that the conservation quantities, for both the linear and bi-linear reaction, do not change if the reactions are irreversible (i.e., if \(K\_{eq} \rightarrow \infty\))

###### Numerical simulation of reversible bi-linear reactions¶

The dynamic response of this reaction can readily be computed and the results graphed; see Figure 4.2.

```
[6]:
```

```
# Create MassModel
model = MassModel('BiLinear_Reversible')
# Generate the MassMetabolites
x1 = MassMetabolite("x1")
x2 = MassMetabolite("x2")
x3 = MassMetabolite("x3")
# Generate the MassReactions
v1 = MassReaction("v1")
# Add metabolites to the reaction, add reaction to the model
v1.add_metabolites({x1: -1, x2: -1, x3: 1})
model.add_reactions([v1])
# Set parameters and initial conditions
v1.kf = 1
v1.kr = 1
model.update_initial_conditions({x1: 3, x2: 2, x3: 0})
# Utilize type 2 rate law for kf and kr parameters defined
model.get_rate_expressions(rate_type=2, update_reactions=True)
model
```

```
[6]:
```

|  |  |
| --- | --- |
| **Name** | BiLinear\_Reversible |
| **Memory address** | 0x07fd19052fd10 |
| **Stoichiometric Matrix** | 3x1 |
| **Matrix Rank** | 1 |
| **Number of metabolites** | 3 |
| **Initial conditions defined** | 3/3 |
| **Number of reactions** | 1 |
| **Number of genes** | 0 |
| **Number of enzyme modules** | 0 |
| **Number of groups** | 0 |
| **Objective expression** | 0 |
| **Compartments** |  |

```
[7]:
```

```
t0 = 0
tf = 5
sim = Simulation(model, verbose=True)
conc_sol, flux_sol = sim.simulate(model, time=(t0, tf), verbose=True)

# Define pools
pools = ['x1*x2 - x3 / Keq_v1', 'x1 + x3', 'x2 + x3']

for i, equation_str in enumerate(pools):
    pool_id = "p" + str(i + 1)
    conc_sol.make_aggregate_solution(
        pool_id, equation=equation_str,
        parameters={v1.Keq_str: v1.kf/v1.kr}, update=True)
```

```
WARNING: No compartments found in model. Therefore creating compartment 'compartment' for entire model.
```

```
Successfully loaded MassModel 'BiLinear_Reversible' into RoadRunner.
Getting time points
Setting output selections
Setting simulation values for 'BiLinear_Reversible'
Simulating 'BiLinear_Reversible'
Simulation for 'BiLinear_Reversible' successful
Adding 'BiLinear_Reversible' simulation solutions to output
Updating stored solutions
```

```
[8]:
```

```
fig_4_2 = plt.figure(figsize=(12, 4))
gs = fig_4_2.add_gridspec(nrows=1, ncols=2)

ax1 = fig_4_2.add_subplot(gs[0, 0])
ax2 = fig_4_2.add_subplot(gs[0, 1])

plot_time_profile(
    conc_sol, ax=ax1, observable=model.metabolites,
    legend="lower outside",
    xlabel="Time", ylabel="Concentrations",
    title=("(a) Time Profiles of Species", {"size": "large"}));

plot_time_profile(
    conc_sol, ax=ax2, observable=["p1", "p2", "p3"],
    legend="lower outside",
    xlabel="Time", ylabel="Concentrations",
    title=("(b) Time Profiles of Pools", {"size": "large"}));
fig_4_2.tight_layout()
```

**Figure 4.2:** The concentration time profiles for the reaction \(x\_1 + x\_2 {\rightleftharpoons} x\_3\) for \(k\_1\) = \(k\_{-1} = 1\) and \(x\_1(0)=3\), \(x\_2(0)=2\), and \(x\_3(0)=0\). (a) The concentrations as a function of time. (b) The pools as a function of time.

##### Connected Reversible Linear Reactions¶

Now we consider more than one reaction working simultaneously. We will consider two reversible first order reactions that are connected by an irreversible reaction;

\[\begin{equation} x\_1 \underset{v\_{-1}}{\stackrel{v\_1}{\rightleftharpoons}} x\_2 \stackrel{v\_2} \rightarrow x\_3 \underset{v\_{-3}}{\stackrel{v\_3}{\rightleftharpoons}} x\_4 \tag{4.26} \end{equation}\]

The stoichiometric matrix and the reaction vector, are

\[\begin{split}\begin{equation} \textbf{S} = \begin{pmatrix} {-1} & {1} & {0} & {0} & {0} \\ {1} & {-1} & {-1} & {0} & {0} \\ {0} & {0} & {1} & {-1} & {1} \\ {0} & {0} & {0} & {1} & {-1} \\ \end{pmatrix}, \ \textbf{v}(\textbf{x}) = \begin{pmatrix} {k\_1x\_1} \\ {k\_{-1}x\_2} \\ {k\_2x\_2} \\ {k\_3x\_3} \\ {k\_{-3}x\_4} \\\end{pmatrix}\end{equation} \tag{4.27}\end{split}\]

and thus the dynamic mass balances are;

\[\begin{split}\begin{align} \frac{dx\_1}{dt} &= -k\_1x\_1 + k\_{-1}x\_2 \\ \frac{dx\_2}{dt} &= k\_1x\_1 - k\_{-1}x\_2 - k\_2x\_2 \\ \frac{dx\_3}{dt} &= k\_2x\_2 - k\_3x\_3 + k\_{-3}x\_4 \\ \frac{dx\_4}{dt} &= k\_3x\_3 - k\_{-3}x\_4 \\ \end {align} \tag{4.28}\end{split}\]

The net reaction rates are:

\[\begin{equation} v\_{1, net} = k\_1x\_1 - k\_{-1}x\_2 = k\_1(x\_1 - x\_2/K\_1) \tag{4.29} \end{equation}\]

and

\[\begin{equation} v\_{3, net} = k\_3x\_3 - k\_{-3}x\_4 = k\_3(x\_3 - x\_4/K\_3) \tag{4.30} \end{equation}\]

where \(K\_1 = k\_1/k\_{-1}\) and \(K\_3 = k\_3/k\_{-3}\) are the equilibrium constants. This example can be used to illustrate three concepts: 1) dynamic decoupling, 2) stoichiometric decoupling, and 3) formation of multi-reaction pools.

###### Dynamic decoupling through seperated time scales:¶

This linear system can be described by

\[\begin{equation} \frac{d\textbf{x}}{dt} = \textbf{Jx} \tag{4.31} \end{equation}\]

where the Jacobian matrix for this system is obtained directly from the equations in (4.28):

\[\begin{split}\begin{equation} \textbf{J} = \begin{pmatrix} {-k\_1} & {k\_{-1}} & {0} & {0} \\ {k\_1} & {-k\_{-1} - k\_2} & {0} & {0} \\ {0} & {k\_2} & {-k\_3} & {-k\_{-3}} \\ {0} & {0} & {k\_3} & {k\_{-3}} \\ \end{pmatrix} \end{equation} \tag{4.32}\end{split}\]

Note that for linear systems, \(\textbf{x} = \textbf{x'}\). Observe that the second column in \(\textbf{J}\) is a combination of the second and third column in \(\textbf{S}\);

\[\begin{split}\begin{equation} \begin{pmatrix} {j\_{12}} \\ {j\_{22}} \\ {j\_{32}} \\ {j\_{42}} \end{pmatrix} = \begin{pmatrix} {1} \\ {-1} \\ {0} \\ {0} \end{pmatrix} + \begin{pmatrix} {0} \\ {-1} \\ {1} \\ {0} \end{pmatrix} k\_2 = k\_{-1}\textbf{s}\_2 + k\_2\textbf{s}\_3 \end{equation} \tag{4.33}\end{split}\]

```
[9]:
```

```
# Create MassModel
model = MassModel('Connected_Linear_Reversible')
# Generate the MassMetabolites
x1 = MassMetabolite("x1")
x2 = MassMetabolite("x2")
x3 = MassMetabolite("x3")
x4 = MassMetabolite("x4")
# Generate the MassReactions
v1 = MassReaction("v1")
v2 = MassReaction("v2", reversible=False)
v3 = MassReaction("v3")
# Add metabolites to the reaction, add reaction to the model
v1.add_metabolites({x1: -1, x2: 1})
v2.add_metabolites({x2: -1, x3: 1})
v3.add_metabolites({x3: -1, x4: 1})
model.add_reactions([v1, v2, v3])
# Set parameters and initial conditions
v1.kf = 1
v1.Keq = 1
v2.kf = 1
v3.kf = 1
v3.Keq = 1
model.update_initial_conditions({x1: 1, x2: 0, x3: 0, x4: 0})
model
```

```
[9]:
```

|  |  |
| --- | --- |
| **Name** | Connected\_Linear\_Reversible |
| **Memory address** | 0x07fd1902a1ad0 |
| **Stoichiometric Matrix** | 4x3 |
| **Matrix Rank** | 3 |
| **Number of metabolites** | 4 |
| **Initial conditions defined** | 4/4 |
| **Number of reactions** | 3 |
| **Number of genes** | 0 |
| **Number of enzyme modules** | 0 |
| **Number of groups** | 0 |
| **Objective expression** | 0 |
| **Compartments** |  |

```
[10]:
```

```
t0 = 0
tf = 10

sim = Simulation(model, verbose=True)
conc_sol, flux_sol = sim.simulate(model, time=(t0, tf), verbose=True)
```

```
WARNING: No compartments found in model. Therefore creating compartment 'compartment' for entire model.
```

```
Successfully loaded MassModel 'Connected_Linear_Reversible' into RoadRunner.
Getting time points
Setting output selections
Setting simulation values for 'Connected_Linear_Reversible'
Simulating 'Connected_Linear_Reversible'
Simulation for 'Connected_Linear_Reversible' successful
Adding 'Connected_Linear_Reversible' simulation solutions to output
Updating stored solutions
```

```
[11]:
```

```
fig_4_3 = plt.figure(figsize=(6, 4))
gs = fig_4_3.add_gridspec(nrows=1, ncols=1)

ax1 = fig_4_3.add_subplot(gs[0, 0])

plot_time_profile(
    conc_sol, ax=ax1, legend="right outside",
    xlabel="Time", ylabel="Concentrations",
    title=("Time Profiles of Species", {"size": "large"}));
fig_4_3.tight_layout()
```

**Figure 4.3:** The dynamic response of the reactions in Eq. (4.17) for \(K\_1=K\_3=1\) and \(k\_1=k\_2=k\_3=1\). The graphs show the concentrations varying with time for \(x\_1(0)=1, x\_2(0)=x\_3(0)=x\_4(0)=0\).

The kinetic effects of \(x\_2\) are thus felt through both reactions 2 and 3 (i.e., the second and third column in \(\textbf{S}\) that are the corresponding reaction vectors), \(\textbf{s}\_2\) and \(\textbf{s}\_3\). These two reaction vectors are weighted by the rate constants (reciprocal of the time constants). Therefore, we expect that if \(k\_2\) is numerically much smaller than \(k\_{-1}\) then the dynamic coupling is ‘weak.’ We consider two sets of parameter values.

- First, we simulate this system with all the rate constants being equal, Figure 4.3. All the concentrations are moving on the same time scale. For a series of reactions, the overall dynamics are expected to unfold on a time scale that is the sum of the individual time constants. Here, this sum is three, and the dynamics have relaxed after a time period of three to five times this value.
- Next, we make the second reaction ten times slower compared to the other two by decreasing \(k\_2\). We see that the two faster reactions come to a quasi-equilibrium state relatively quickly (relative to reaction 3), and form two conservation pools that exchange mass slowly. The sum of the rate constants for the three reactions in series is now seven, and the dynamics unfold on this time scale.

###### Stoichiometric decoupling¶

Reaction 3 does not influence reaction 1 at all. They are separated by the irreversible reaction 2. Thus, changes in the kinetics of reaction 3 will not influence the progress of reaction 1. This can be illustrated through simulation by changing the rate constants for reaction 3 and observing what happens to reaction 1.

###### Formation of multi-reaction pools:¶

We can form the following pooled variables based on the properties of the individual reversible reactions

\[\begin{split}\begin{align} &p\_1 = x\_1 - x\_2/K\_1\ && disequilibrium\ quantity\ for\ reaction\ 1 \\ &p\_2 = x\_1 + x\_2 \ && conservation\ quantity\ for\ reaction\ 1 \\ &p\_3 = x\_3 - x\_4/K\_3\ && disequilibrium\ quantity\ for\ reaction\ 3 \\ &p\_4 = x\_3 + x\_4 \ && conservation\ quantity\ for\ reaction\ 3\end{align} \tag{4.34}\end{split}\]

A representation of the dynamics of this reaction system can be obtained by plotting these pools as a function of time; Figure 4.4a. To prepare this plot, we use the pooling matrix

\[\begin{split}\begin{equation} \textbf{P} = \begin{pmatrix} {1} & {-1/K\_1} & {0} & {0} \\ {1} & {1} & {0} & {0} \\ {0} & {0} & {1} & {-1/K\_3} \\ {0} & {0} & {1} & {1} \\ \end{pmatrix} \end{equation} \tag{4.35}\end{split}\]

to post-process the output. However, we note that the conservation quantities associated with the individual reactions are no longer time-invariant.

The rank of \(\textbf{S}\) is 3 and its one-dimensional left null space is spanned by (1,1,1,1); thus the conservation quantity is \(x\_1 + x\_2 + x\_3 + x\_4\). Therefore an alternative pooling matrix may be formulated as

\[\begin{split}\begin{equation} \textbf{P} = \begin{pmatrix} {1} & {-1/K\_1} & {0} & {0} \\ {0} & {1} & {0} & {0} \\ {0} & {0} & {1} & {-1/K\_3} \\ {1} & {1} & {1} & {1} \\ \end{pmatrix}\end{equation} \tag{4.36}\end{split}\]

where we use \(x\_2\) as the coupling variable and the overall conservation pool instead of the conservation pools associated with the individual reactions. The two conservation pools are combined into one overall mass conservation pool.

We can now derive the dynamic mass balances on the pools as

\[\begin{split}\begin{align} \frac{d\textbf{p}}{dt} = \textbf{PSv} &= \begin{pmatrix} {-(k\_1 + k\_{-1})(x\_1 - x\_2/K\_1) +\frac{k\_2}{K\_1}x\_2} \\ {k\_1(x\_1 - x\_2/K\_1) - k\_2x\_2} \\ {-(k\_3 + k\_{-3})(x\_3 - x\_4/K\_3) +k\_2x\_2} \\ {0}\end{pmatrix} \\ &= \begin{pmatrix} {-(k\_1 + k\_{-1})p\_1 +\frac{k\_2}{K\_1}x\_2} \\ {k\_1p\_1 - k\_2p\_2} \\ {-(k\_3 + k\_{-3})p\_3 +k\_2p\_2} \\{0}\end{pmatrix} \\ &= \begin{pmatrix} {-(k\_1 + k\_{-1})} \\ {k\_1} \\ {0} \\ {0} \end{pmatrix}p\_1 + \begin{pmatrix} {\frac{k\_2}{K\_1}} \\ {-k\_2} \\ {k\_2} \\ {0} \end{pmatrix}p\_2 + \begin{pmatrix} {0} \\ {0} \\ {-(k\_3 + k\_{-3})} \\ {0} \end{pmatrix}p\_3 \end{align} \tag{4.37}\end{split}\]

This equation shows that \(p\_1\) and \(p\_3\) create fast motion compared to \(p\_2\) given the relative numerical values of the rate constants; \(p\_2\) creates a slow drift in this system for the numerical values used in Figure 4.4b.

```
[12]:
```

```
# Define pools
reg_pools = ['x1 - x2 / Keq_v1', 'x1 + x2',
             'x3 - x4 / Keq_v3', 'x3 + x4']

alt_pools = ['x1 - x2 / Keq_v1', 'x2',
             'x3 - x4 / Keq_v3', 'x1 + x2 + x3 + x4']

for prefix, pools in zip(["p", "alt_p"], [reg_pools, alt_pools]):
    for i, equation_str in enumerate(pools):
        pool_id = prefix + str(i + 1)
        conc_sol.make_aggregate_solution(
            pool_id, equation=equation_str,
            parameters={v1.Keq_str: v1.Keq, v3.Keq_str: v3.Keq},
            update=True)
```

```
[13]:
```

```
fig_4_4 = plt.figure(figsize=(12, 8))
gs = fig_4_4.add_gridspec(nrows=2, ncols=2)

ax1 = fig_4_4.add_subplot(gs[0, 0])
ax2 = fig_4_4.add_subplot(gs[1, 0])
ax3 = fig_4_4.add_subplot(gs[1, 1])

plot_time_profile(
    conc_sol, ax=ax1, observable=model.metabolites,
    legend="right outside", xlabel="Time", ylabel="Concentrations",
    title=("(a) Time Profiles of Species", {"size": "large"}));

plot_time_profile(
    conc_sol, observable=["p1", "p2", "p3"], ax=ax2,
    legend="lower outside", xlabel="Time", ylabel="Concentrations",
    title=("(b) Time Profiles of Pools", {"size": "large"}));

plot_time_profile(
    conc_sol, observable=["alt_p1", "alt_p2", "alt_p3"], ax=ax3,
    legend="lower outside", xlabel="Time", ylabel="Concentrations",
    title=("(c) Time Profiles of Alternate Pools",  {"size": "large"}));
fig_4_4.tight_layout()
```

**Figure 4.4:** (a) The time profiles (b) The conservation pools \(p\_2 = x\_1 + x\_2\) and \(p\_4 = x\_3 + x\_4\) and the disequilibrium pools \(p\_1 = x\_1 - x\_2/K\_1\) and \(p\_3 = x\_3 - x\_4/K\_3\) for the individual reactions. The disequilibrium pools move quickly towards a quasi-equilibrium state, while the conservation pools move more slowly. These pools are defined in Eq (4.35). (c) The dynamic response with alternative pools; \(p\_2 = x\_2\), and
\(p\_4 = x\_1 + x\_2 + x\_3 + x\_4\). These pools are defined in Eq (4.36).

##### Connected Reversible Bi-linear Reactions¶

An important case of connected bi-linear reactions is represented by the reaction mechanism

\[\begin{equation} x\_1 + x\_2 \underset{v\_{-1}}{\stackrel{v\_1}{\rightleftharpoons}} x\_3 \underset{v\_{-2}}{\stackrel{v\_2}{\rightleftharpoons}} x\_4 + x\_5 \tag{4.38} \end{equation}\]

This reaction network is similar to reaction mechanisms for enzymes, and thus leads us into the treatment of enzyme kinetics (Chapter 5). The elementary reaction rates are:

\[\begin{equation} v\_1 = k\_1x\_1x\_2, \ \ v\_{-1} = k\_{-1}x\_3 \ \ v\_2 = k\_2x\_3, \text{and} \ \ v\_{-2} = k\_{-2}x\_4x\_5 \tag{4.39} \end{equation}\]

and the equilibrium constants are \(K\_1 = k\_1/k\_{-1}\) and \(K\_2 = k\_2/k\_{-2}\). There are two disequilibrium quantities.

\[\begin{equation} p\_1 = x\_1x\_2 - x\_3 / K\_1 \tag{4.40} \end{equation}\]

\[\begin{equation} p\_2 = x\_3 - x\_4x\_5 / K\_2 \tag{4.41} \end{equation}\]

We now explore the same features of this coupled system of bi-linear reactions as we did for the single reversible bi-linear reaction.

###### Conservation quantities for connected reversible bi-linear reactions¶

The (5x4) stoichiometric matrix

\[\begin{split}\begin{equation} \textbf{S} = \begin{pmatrix} {-1} & {1} & {0} & {0} \\ {-1} & {1} & {0} & {0} \\ {1} & {-1} & {-1} & {1} \\ {0} & {0} & {1} & {-1} \\ {0} & {0} & {1} & {-1} \\ \end{pmatrix} \end{equation} \tag{4.42}\end{split}\]

has a rank of 2 and thus there are three conservation variables and two independent dynamic variables.

The conservation quantities are not unique and which one we will use depends on the reaction chemistry that is being studied. An example is

\[\begin{equation} AB + C \underset{v\_{-1}}{\stackrel{v\_1}{\rightleftharpoons}} ABC \underset{v\_{-2}}{\stackrel{v\_2}{\rightleftharpoons}} A + BC \tag{4.43} \end{equation}\]

in which case the three independent conservation quantities would be:

\[\begin{split}\begin{equation} \text{Conservation of A}:\ p\_3 = x\_1 + x\_3 + x\_4 \\ \text{Conservation of B}:\ p\_4 = x\_1 + x\_3 + x\_5 \\ \text{Conservation of C}:\ p\_5 = x\_2 + x\_3 + x\_5 \end{equation}\end{split}\]

\[\tag{4.44}\]

These are convex quantities as all the coefficients are non-negative (the concentrations are \(x\_i>0\)). The individual bi-linear reactions have two each, but once coupled, the number of conservation quantities drops by one.

###### The equilibrium state for connected reversible bi-linear reactions¶

The computation of the equilibrium state involves setting the net fluxes to zero and combining those equations with the conservation quantities to get a set of independent equations. For convenience of illustration, we pick \(K\_1 = K\_2 = 1\) and the equilibrium equations become

\[\begin{equation} x\_{1, eq}x\_{2, eq} = x\_{3, eq} = x\_{4, eq}x\_{5, eq} \tag{4.45} \end{equation}\]

and if we pick \(p\_3 = p\_4 = p\_5 = 3\) then the solution for the equilibrium state is simple: \(x\_{1, eq}x\_{2, eq} = x\_{3, eq} = x\_{4, eq}x\_{5, eq} = 1\). These equations can also be solved for arbitrary parameter values.

###### Linearization and deviation variables for connected reversible bi-linear reactions¶

By linearizing the differential equations around the steady state we obtain

\[\begin{split}\begin{align} \frac{dx\_1'}{dt} &= \frac{dx\_2'}{dt} = -(k\_1x\_{1,eq})x\_2' -(k\_1x\_{2,eq})x\_1' + k\_{-1}x\_3' \\ \frac{dx\_3'}{dt} &= (k\_1x\_{2,eq})x\_1' + (k\_1x\_{1,eq})x\_2' - (k\_{-1} + k\_2)x\_3' + (k\_{-2}x\_{5,eq})x\_4'+ (k\_{-2}x\_{4,eq})x\_5'\\ \frac{dx\_4'}{dt} &= \frac{dx\_5'}{dt} = k\_{2}x\_3' - (k\_{-2}x\_{5,eq})x\_4' -(k\_{-2}x\_{4,eq})x\_5' \end{align} \tag{4.46}\end{split}\]

where \(x\_i' = x\_i - x\_{i, eq}\) represent the concentration deviation around equilibrium, \(i\) =1,2,3,4 and 5.

###### The disequilibrium and conservation quantities for connected reversible bi-linear reactions¶

Similar to the reversible bi-linear reaction, we obtain two pools that represent the disequilibrium driving forces of the two reactions,

\[\begin{split}\begin{align} p\_1 &= x\_1x\_2 - x\_3 / K\_1 \approx (x\_{2, eq})x\_1' + (x\_{1, eq})x\_2' - (1/K\_1)x\_3' = p\_1'\\ p\_2 &= x\_3 - x\_4x\_5 / K\_2 \approx x\_3' - (x\_{5, eq}/K\_2)x\_4' - (x\_{4, eq}/K\_2)x\_5' = p\_2' \end{align} \tag{4.47}\end{split}\]

and the three pools that represent conservative quantities do not change:

\[\begin{split}\begin{align} p\_3 &= x\_1 + x\_3 + x\_4 \\ p\_4 &= x\_1 + x\_3 + x\_5 \\ p\_5 &= x\_2 + x\_3 + x\_5 \end{align} \tag{4.48}\end{split}\]

We thus can define the pooling matrix as:

\[\begin{split}\begin{align} \textbf{P} &= \begin{pmatrix} {x\_{2, eq}} & {x\_{1, eq}} & {1/K\_1} & {0} & {0} \\ {0} & {0} & {1} & {-x\_{5, eq}/K\_2} & {-x\_{4, eq}/K\_2} \\ {1} & {0} & {1} & {1} & {0} \\ {1} & {0} & {1} & {0} & {1} \\ {0} & {1} & {1} & {0} & {1} \\ \end{pmatrix} \\ &= \begin{pmatrix} {1} & {1} & {-1} & {0} & {0} \\ {0} & {0} & {1} & {-1} & {-1} \\ {1} & {0} & {1} & {1} & {0} \\ {1} & {0} & {1} & {0} & {1} \\ {0} & {1} & {1} & {0} & {1} \\ \end{pmatrix} \end{align} \tag{4.49}\end{split}\]

for the particular equilibrium constants and concentrations values given above.

The differential equations for the pools are then formed by (and at this stage we remove the conservation pools as they are always constant):

\[\begin{split}\begin{equation} \frac{d\textbf{p'}}{dt} = \textbf{PSv(x)} \ \text{where} \ \textbf{v(x)} \approx \begin{pmatrix} {k\_1p\_1'} \\ {k\_2p\_2'} \\ \end{pmatrix} = \begin{pmatrix} {k\_1} & {0} \\ {0} & {k\_2} \\ \end{pmatrix} \begin{pmatrix} {p\_1'} \\ {p\_2'} \\ \end{pmatrix} \end{equation} \tag{4.50}\end{split}\]

which gives

\[\begin{split}\begin{equation} \frac{d\textbf{p'}}{dt} = \begin{pmatrix} {-(x\_{2, eq} + x\_{1, eq} + 1/K\_1)} \\ {1} \\ \end{pmatrix}k\_1p\_1' + \begin{pmatrix} {1/K\_1} \\ {-(1 + (x\_{5, eq} + x\_{4, eq})/K\_2} \\ \end{pmatrix}k\_2p\_2' \end{equation} \tag{4.51}\end{split}\]

###### Numerical simulation of connected reversible bi-linear reactions¶

These equations can be simulated once parameter values and initial conditions are specified. In order to illustrate the dynamic behavior in terms of the pools, we consider the particular situation where \(K\_1 = K\_2 = x\_{1, eq} = x\_{2, eq} = x\_{3, eq} = x\_{4, eq} = x\_{5, eq} = 1\), see Figure 4.5.

```
[14]:
```

```
# Create MassModel
model = MassModel('Connected_BiLinear_Reversible')
# Generate the MassMetabolites
x1 = MassMetabolite("x1")
x2 = MassMetabolite("x2")
x3 = MassMetabolite("x3")
x4 = MassMetabolite("x4")
x5 = MassMetabolite("x5")
# Generate the MassReactions
v1 = MassReaction("v1")
v2 = MassReaction("v2")
# Add metabolites to the reaction, add reaction to the model
v1.add_metabolites({x1: -1, x2: -1, x3: 1})
v2.add_metabolites({x3: -1, x4: 1, x5: 1})
model.add_reactions([v1, v2])
# Set parameters and initial conditions
v1.kf = 1
v1.kr = 1
v2.kf = 1
v2.kr = 1
model.update_initial_conditions({x1: 3, x2: 3, x3: 0, x4: 0, x5: 0})

# Utilize type 2 rate law for kf and kr parameters defined
model.get_rate_expressions(rate_type=2, update_reactions=True)
model
```

```
[14]:
```

|  |  |
| --- | --- |
| **Name** | Connected\_BiLinear\_Reversible |
| **Memory address** | 0x07fd190e33b50 |
| **Stoichiometric Matrix** | 5x2 |
| **Matrix Rank** | 2 |
| **Number of metabolites** | 5 |
| **Initial conditions defined** | 5/5 |
| **Number of reactions** | 2 |
| **Number of genes** | 0 |
| **Number of enzyme modules** | 0 |
| **Number of groups** | 0 |
| **Objective expression** | 0 |
| **Compartments** |  |

```
[15]:
```

```
t0 = 0
tf = 10
sim = Simulation(model, verbose=True)
conc_sol, flux_sol = sim.simulate(model, time=(t0, tf), verbose=True)

# Define pools
pools = ['x1*x2 - x3 / Keq_v1', 'x3 - x4*x5 / Keq_v2',
         'x1 + x3 + x4', 'x1 + x3 + x5', 'x2 + x3 + x4']

for i, equation_str in enumerate(pools):
    pool_id = "p" + str(i + 1)
    conc_sol.make_aggregate_solution(
        pool_id, equation=equation_str,
        parameters={v1.Keq_str: v1.kf/v1.kr,
                    v2.Keq_str: v2.kf/v2.kr}, update=True)
```

```
WARNING: No compartments found in model. Therefore creating compartment 'compartment' for entire model.
```

```
Successfully loaded MassModel 'Connected_BiLinear_Reversible' into RoadRunner.
Getting time points
Setting output selections
Setting simulation values for 'Connected_BiLinear_Reversible'
Simulating 'Connected_BiLinear_Reversible'
Simulation for 'Connected_BiLinear_Reversible' successful
Adding 'Connected_BiLinear_Reversible' simulation solutions to output
Updating stored solutions
```

```
[16]:
```

```
fig_4_5 = plt.figure(figsize=(12, 4))
gs = fig_4_5.add_gridspec(nrows=1, ncols=2)

ax1 = fig_4_5.add_subplot(gs[0, 0])
ax2 = fig_4_5.add_subplot(gs[0, 1])

plot_time_profile(
    conc_sol, observable=model.metabolites, ax=ax1, legend="left outside",
    xlabel="Time", ylabel="Concentrations",
    title=("(a) Time Profiles of Species",  {"size": "large"}));

plot_time_profile(
    conc_sol, ax=ax2, observable=["p1", "p2", "p3", "p4", "p5"],
    legend="right outside",
    xlabel="Time", ylabel="Concentrations",
    title=("(a) Time Profiles of Pools",  {"size": "large"}));
fig_4_5.tight_layout()
```

**Figure 4.5:** The concentration time profiles for the reaction system \(x\_1 + x\_2 \rightleftharpoons x\_3 \rightleftharpoons x\_4 + x\_5\) for \(k\_1 = k\_{-1} = k\_2 = k\_{-2} = 1\) and \(x\_1(0)=3, x\_2(0)=3, x\_3(0)=0, x\_4(0)=0, x\_5(0)=0\) (a) The concentrations as a function of time. (b) The pools as a function of time.

In this situation, the dynamic equation for the linearized pools becomes

\[\begin{split}\begin{equation} \frac{d\textbf{p'}}{dt} = \begin{pmatrix} {-3} \\ {1} \\ \end{pmatrix}k\_1p\_1' + \begin{pmatrix} {1} \\ {-3} \\ \end{pmatrix}k\_2p\_2' \end{equation} \tag{4.52}\end{split}\]

We can solve this equation and present the results with a dynamic phase portrait, Figure 4.6. The dynamic behavior of the non-equilibrium pools is shown for a range of parameters. We make three observations here.

1. Figure 4.6 shows that the dynamics for the pools can be decomposed to consider a fast equilibration of the two disequilibrium pools followed by the slow decay of the slower disequilibrium pool (Change values for \(k\_1\) and \(k\_2\) to visualize).
2. When reaction 1 is 10 times faster than reaction 2, then initial motion is along the vector \((-3,1)^T\), and when reaction 1 is 10 times slower than reaction 2, then initial motion is along the vector \((1,-3)^T\),
3. The linearized pools move in a similar fashion to the bi-linear disequilibrium pools, see Figure 4.6. The bi-linear and linear simulation do not change that much even though \(x\_1\), \(x\_2\), \(x\_4\), \(x\_5\) are 25% from their equilibrium value, and \(x\_3\) is 50% away from equilibrium.

```
[17]:
```

```
# Set new initial conditions
model.update_initial_conditions({x1: 0.75, x2: 0.75, x3: 1.5,
                                 x4: 0.75, x5: 0.75})
sim.update_model_simulation_values(model)
conc_sol, flux_sol = sim.simulate(model, time=(t0, tf))

for i, equation_str in enumerate(pools):
    pool_id = "p" + str(i + 1)
    conc_sol.make_aggregate_solution(
        pool_id, equation=equation_str,
        parameters={v1.Keq_str: v1.kf/v1.kr,
                    v2.Keq_str: v2.kf/v2.kr}, update=True)
```

```
[18]:
```

```
# Visualize solution
fig_4_6 = plt.figure(figsize=(5, 5))
gs = fig_4_6.add_gridspec(nrows=1, ncols=1)

ax1 = fig_4_6.add_subplot(gs[0, 0])

plot_phase_portrait(
    conc_sol, x="p1", y="p2", ax=ax1, legend="best",
    xlabel="p1", ylabel="p2", xlim=(-1.5, .5), ylim=(-.5, 1.5),
    title=("Phase portrait of p1 vs p2", {"size": "large"}),
    annotate_time_points="endpoints",
    annotate_time_points_labels=True);
fig_4_6.tight_layout()
```

**Figure 4.6:** The dynamic response of \(x\_1 + x\_2 \rightleftharpoons x\_3 \rightleftharpoons x\_4 + x\_5\) for \(K\_1 = K\_{-1} = 1\). The graphs show the concentrations varying with time for \(x\_3(0)=1.5, \ x\_1(0) = x\_2(0) = x\_4(0) = x\_5(0) =0.75\) The disequilibrium pools \(p\_1 = x\_1x\_2 - x\_3 / K\_1\) (x-axis) and \(p\_2 = x\_3 - x\_4x\_5 / K\_2\) (y-axis) shown in a phase portrait.

##### Summary¶

- Chemical properties associated with chemical reactions are; stoichiometry thermodynamics, and kinetics. The first two are physico-chemical properties, while the third can be biologically altered through enzyme action.
- Each net reaction can be described by pooled variables that represent a dis-equilibrium quantity and a mass conservation quantity that is associated with the reaction.
- If a reaction is fast compared to its network environment, its disequilibrium variable can be relaxed and then described by the conservation quantity associated with the reaction.
- Linearizing bi-linear rate laws does not create much error for small changes around the reference state.
- Removing a time scale from a model corresponds to reducing the dynamic dimension of the transient response by one.
- As the number of reactions grow, the number of conservation quantities may change.
- Irreversibility of reactions does not change the number of conservation quantities for a system.

\(\tiny{\text{© B. Ø. Palsson 2011;}\ \text{This publication is in copyright.}\\ \text{Subject to statutory exception and to the provisions of relevant collective licensing agreements,}\\ \text{no reproduction of any part may take place without the written permission of Cambridge University Press.}}\)

#### Enzyme Kinetics¶

We now study common reaction mechanisms that describe enzyme catalysis. Enzymes can dramatically accelerate the rate of biochemical reactions inside and outside living cells. The absolute rates of biochemical reactions are key biological design variables because they can evolve from a very low rate as determined by the mass action kinetics based on collision frequencies, to a very high and specific reaction rate as determined by appropriately-evolved enzyme properties. We first describe the
procedure used to derive enzymatic rate laws, which we then apply to the Michaelis-Menten reaction mechanism, then to the Hill model, and finally to the symmetry model. The first is used to describe plain chemical transformations, while the latter two are used to describe regulatory effects.

**MASSpy** will be used to demonstrate some of the topics in this chapter.

```
[1]:
```

```
from mass import (
    MassModel, MassMetabolite, MassReaction, Simulation, MassSolution)
from mass.visualization import plot_time_profile, plot_phase_portrait
```

Other useful packages are also imported at this time.

```
[2]:
```

```
import numpy as np
import pandas as pd
import matplotlib.pyplot as plt
from scipy.integrate import solve_ivp
```

##### Enzyme Catalysis¶

Enzymes are catalysts that accelerate biochemical transformations in cells. Almost all enzymes are proteins. There are also catalytically active ribonucleic acids, called “ribozymes.” The fundamental properties of enzyme catalysis are described in this section.

###### Enzymatic activity¶

The activity of an enzyme is measured by determining the increase in the reaction rate relative to the absence of the enzyme. In other words we compare the reaction rate of the un-catalyzed reaction to the catalyzed rate. The ratio can be thought of as an acceleration factor and this number can be quite high, sometimes by many million-fold.

###### Reaction and substrate specificity¶

Enzymes are usually very specific both with respect to the type of reaction being catalyzed (reaction specificity) and with respect to the reactants (the “substrates”) that they act on. Highly specific enzymes catalyze the cleavage of only one type of a chemical bond, and only in one substrate. Other enzymes may have a narrow reaction specificity, but broad substrate specificity, i.e., they act on a number of chemically similar substrates. Rare enzymes exist that have both low reaction
specificity and low substrate specificity.

**Figure 5.1:** Basic principles of enzyme catalysis. From (Koolman, 2005).

###### Catalysis¶

As discussed in Chapter 2 (Figure 2.4), two molecules can only react with each other if they collide in a favorable orientation. Such collisions may be rare, and thus the reaction rate is slow. An un-catalyzed reaction starts with a favorable collision as shown in Figure 5.1a. Before the products are formed, the collision complex A-B has to pass through what is called a *transition state*. Its formation requires *activation energy*. Since activation energies can be quite high, only a few A-B
complexes have this amount of energy, and thus a productive transition state arises only for a fraction of favorable collisions. As a result, conversion only happens occasionally even when the reaction is thermodynamically feasible; i.e., when the net change in Gibbs free energy is negative (\(\Delta G < 0\)).

Enzymes can facilitate the probability of a favorable collision and lower the activation energy barrier, see Figure 5.1b,c. Enzymes are able to bind their substrates in the catalytic site. As a result, the substrates are favorably oriented relative to one another, greatly enhancing the probability that productive A-B complexes form. The transition state is stabilized leading to a lowered activation energy barrier.

###### Information on enzymes¶

Detailed information is available on a large number of enzymes. This include structural information, the organism source, and other characteristics. An example is shown in Figure 5.2. Many online sources of such information exist.

**Figure 5.2:** Detailed information on enzymes is available. From PDB.

##### Deriving Enzymatic Rate Laws¶

The chemical events underlying the catalytic activities of enzymes are described by a reaction mechanism. A reaction mechanism is comprised of the underlying elementary reactions that are believed to take place. A rate law is then formulated to describe the rate of reaction.

A rate law describes the conversion of a substrate \((x\_1)\) by an enzyme into a product \((x\_2)\):

\[\begin{equation} x\_1 \stackrel{v}{\rightarrow} x\_2 \tag{5.1} \end{equation}\]

where \(v\) is a function of the concentrations of the chemical species involved in the reaction. The steps involved in the development and analysis of enzymatic rate laws are illustrated in Figure 5.3 and they are as follows:

**Figure 5.3:** The process of formulating enzymatic rate laws. QSSA represents the quasi-steady state assumption and QEA represents the quasi-equilibrium assumption.

- Formulate the dynamic mass balances based on the elementary reactions in the postulated reaction mechanism,
- Identify time invariants, or conservation relationships,
- Reduce the dynamic dimension of the reaction mechanism by eliminating dynamically dependent variables using the conservation relationships,
- Apply commonly used simplifying kinetic assumptions to formulate a rate law, representing a reduction in the dynamic dimension of the kinetic model,
- Apply mathematical and numerical analysis to determine when the simplifying assumptions are valid and the reaction rate law can be used; and
- Identify key dimensionless parameter ratios. This last step is optional and used by those interested in deeper mathematical analysis of the properties of the rate laws.

The use of enzymatic rate laws in dynamic network models is hampered by their applicability in vivo based on in vitro measurements. From a practical standpoint, with the numerical simulation capacity that is now routinely available, applying simplifying assumptions may no longer be needed for computational simplification and convenience. However, it is useful to help understand the historical origin of enzymatic rate laws, the simplifications on which they are based, and when it may be desirable
to use them.

##### Michaelis-Menten Kinetics¶

The simplest enzymatic reaction mechanism, first proposed by Henri (Henri, 1903) but named after Michaelis and Menten (Michaelis, 1913) is;

\[\begin{equation} S + E \underset{k\_{-1}}{\stackrel{k\_1}{\rightleftharpoons}} X \stackrel{k\_2}{\rightarrow} E + P \tag{5.2} \end{equation}\]

where a substrate, \(S\), binds reversibly to the enzyme, \(E\), to form the intermediate, \(X\), which can break down to give the product, \(P\), and regenerate the enzyme. Note that it is similar to the reaction mechanism of two connected reversible bi-linear reactions (Eq. (4.38)) with \(x\_5 = x\_2\), as one of the original reactants \((E)\) is regained in the second step. Historically speaking, the Michaelis-Menten scheme is the most important enzymatic reaction
mechanism. A detailed account of the early history of Michaelis-Menten kinetics is found in (Segal, 1959).

###### Step 1: Dynamic mass balances for Michaelis-Menten kinetics¶

Applying the law of mass action to the Michaelis-Menten reaction mechanism, one obtains four differential equations that describe the dynamics of the concentrations of the four chemical species involved in the reaction mechanism:

\[\begin{split}\begin{align} \frac{ds}{dt} &= -k\_1es + k\_{-1}x, & s(t = 0) = s\_0 \\ \frac{dx}{dt} &= k\_1es - (k\_{-1} + k\_2)x, & x(t = 0) = 0 \\ \frac{de}{dt} &= -k\_1es + (k\_{-1} + k\_2)x, & e(t = 0) = e\_0 \\ \frac{dp}{dt} &= k\_2x, & p(t = 0) = 0 \\ \end{align} \tag{5.3}\end{split}\]

where the lower case letters denote the concentrations of the corresponding chemical species. The initial conditions shown are for typical initial rate experiments where substrate and free enzyme are mixed together at time \(t=0\). \(e\_0\) and \(s\_0\) denote the initial concentration of enzyme and substrate, respectively. No mass exchange occurs with the environment.

###### Step 2: Finding the time invariants for Michaelis-Menten kinetics¶

Using \(\textbf{x} = (s, e, x, p)\) and \(\textbf{v} = (k\_1es, \ k\_{-1}x, \ k\_2x)\) the stoichiometrix matrix is

\[\begin{split}\begin{equation} \textbf{S} = \begin{pmatrix} {-1} & {1} & {0} \\ {-1} & {1} & {1} \\ {1} & {-1} & {-1} \\ {0} & {0} & {1} \\ \end{pmatrix} \end{equation} \tag{5.4}\end{split}\]

It has a rank of 2 and thus there are two conservation quantities. They are the total concentration of the enzyme and total concentration of the substrate:

\[\begin{split}\begin{align} e\_0 & = e + x \tag{5.5} \\ s\_0 &= s + x + p \tag{5.6} \end{align}\end{split}\]

###### Step 3: Reducing the dynamic description for Michaelis-Menten kinetics¶

As a consequence of the two conservation relationships, only two of equations 5.3 are dynamically independent. Choosing the substrate, \(s\), and the intermediate complex, \(x\), concentrations as the two independent variables, the reaction dynamics are described by:

\[\begin{split}\begin{align} \frac{ds}{dt} &= -k\_1e\_0s + (k\_1s + k\_{-1})x, \ &s(t = 0)=s\_0 \tag{5.7} \\ \frac{dx}{dt} &= k\_1e\_0s - (k\_1s + k\_{-1} + k\_2)x, \ &x(t = 0)=0 \tag{5.8} \\ \end{align}\end{split}\]

The major problem with this mass action kinetic model is that it is mathematically intractable (Hommes, 1962). Equations 5.7 and 5.8 can be reduced to an Abel type differential equation whose solution cannot be obtained in a closed form.

###### Step 4: Applying kinetic assumptions for Michaelis-Menten kinetics¶

A closed form analytical solution to the mass action kinetic equations, 5.7 and 5.8, is only attainable by using simplifying kinetic assumptions. Two assumptions are used: the *quasi-steady state assumption* (QSSA) and the *quasi-equilibrium assumption* (QEA).

###### The quasi-steady state assumption:¶

The rationale behind the quasi-steady state assumption (Briggs, 1925) is that, after a rapid transient phase, the intermediate, \(X\), reaches a quasi-stationary state in which its concentration does not change appreciably with time. Applying this assumption to Eq. (5.8) (i.e., \(dx/dt=0\)) gives the concentration of the intermediate complex as:

\[\begin{equation} x\_{qss} = \frac{e\_0s}{K\_m + s} \tag{5.9} \end{equation}\]

where \(K\_m = (k\_{-1} + k\_2)/k\_1\) is the well-known Michaelis constant. Substituting \(x\_{qss}\) into the differential equation for the substrate (Eq. (5.7)) gives the rate law

\[\begin{equation} \frac{ds}{dt} = \frac{-k\_2e\_0s}{K\_m + s} \tag{5.10} \end{equation}\]

which is the well-known Michaelis-Menten equation, where \(v\_m\) is the maximum reaction rate (or reaction velocity).

Initially, the quasi-steady state assumption was justified based on physical intuition, but justification for its applicability is actually found within the theory of singular perturbations (Bowen, 1963) Eq. (5.10) can be shown to be the first term in an asymptotic series solution derived from singular perturbation theory (Heineken,1967), (Meiske, 1978); see review in (Palsson, 1984).

###### The quasi-equilibrium assumption:¶

Here, one assumes that the binding step quickly reaches a quasi-equilibrium state (Henri, 1903), (Michaelis, 1913) where

\[\begin{equation} \frac{se}{x} = \frac{s(e\_0 - x)}{x} = \frac{k\_{-1}}{k\_1} = K\_d, \ \text{or} \ x\_{qe} = \frac{e\_0s}{K\_d + s} \tag{5.11} \end{equation}\]

holds. \(K\_d\) is the disassociation equilibrium constant. Note the similarity to Eq. (5.9). Hence, one obtains the rate law

\[\begin{equation} \frac{dp}{dt} = \frac{k\_2e\_0s}{K\_d + s} \tag{5.12} \end{equation}\]

by using Eq. (5.11) in the differential equation for the product \(P\).

###### Step 5: Numerical solutions for Michaelis-Menten kinetics¶

The full dynamic description of the kinetics of the reaction (Eq. (5.7) and (5.8)) can be obtained by direct numerical integration. The results are most conveniently shown on a phase portrait along with the transient response of the concentrations on both the fast and slow time scales, see Figure 5.4.

###### QSSA Solution for Michaelis-Menten kinetics¶

```
[3]:
```

```
t0 = 0
tf = 1e3
# QSSA Assumption
# Define function to integrate
def qssa(t, s, *params):
    k2, e0, Km = params
    dsdt = (-k2*e0*s)/(Km + s)
    return dsdt

# Define initial conditions and parameters for integration
s0 = 1

e0 = (1/100)
k2 = 1
Km = 1
params = [k2, e0, Km]

# Obtain numerical solutions
sol_obj = solve_ivp(fun=lambda t, s: qssa(t, s, *params),
                    t_span=(t0, tf), y0=[s0])
t, s_sol = (sol_obj.t, sol_obj.y)
x_sol = np.array([(e0 * val)/(Km + val) for val in s_sol])
# Store solutions into Solution Objects
qssa_sol = MassSolution(
    "QSSA", solution_type="Conc",
    data_dict={"s": s_sol[0], "x": x_sol[0]},
    time=t, interpolate=False)
```

###### Numerical Solution for Michaelis-Menten kinetics¶

```
[4]:
```

```
model = MassModel('Michaeli_Menten')
## Define metabolites
s = MassMetabolite("s")
e = MassMetabolite("e")
x = MassMetabolite("x")
p = MassMetabolite("p")
# Define reactions
v1 = MassReaction("v1")
v2 = MassReaction("v2", reversible=False)
v1.add_metabolites({s: -1, e: -1, x: 1})
v2.add_metabolites({x: -1, e: 1, p: 1})
model.add_reactions([v1, v2])
## Define parameters
v1.kf = 2
v1.Keq = 2
v2.kf = 1
# Define initial conditions
model.update_initial_conditions({s: s0, e: e0, x: 0, p: 0})

# Solve
MM_simulation = Simulation(model, verbose=True)
conc_sol, flux_sol = MM_simulation.simulate(model, (t0, tf))
```

```
WARNING: No compartments found in model. Therefore creating compartment 'compartment' for entire model.
```

```
Successfully loaded MassModel 'Michaeli_Menten' into RoadRunner.
```

```
[5]:
```

```
fig_5_4 = plt.figure(figsize=(9, 7))
gs = fig_5_4.add_gridspec(nrows=2, ncols=2, width_ratios=[1, 1.5],
                          height_ratios=[1, 1])

ax1 = fig_5_4.add_subplot(gs[0, 0])
ax2 = fig_5_4.add_subplot(gs[0, 1])
ax3 = fig_5_4.add_subplot(gs[1, 1])
# Phase portrait of both solutions' substrate vs. intermediate
plot_phase_portrait(
    conc_sol, x=s, y=x, ax=ax1, legend=["Numerical Solution"],
    annotate_time_points="endpoints",
    annotate_time_points_color=["r", "b"],
    annotate_time_points_labels=True);
plot_phase_portrait(
    qssa_sol, x=s, y=x, ax=ax1, legend=["QSSA", "lower outside"],
    xlabel=s.id, ylabel=x.id, linestyle=["--"],
    title=("(a) Phase Portrait", {"size": "x-large"}),
    annotate_time_points="endpoints",
    annotate_time_points_labels=True);

# Time profile of solutions' substrate concentration
plot_time_profile(conc_sol, observable=s, ax=ax2);
plot_time_profile(
    qssa_sol, observable=s, ax=ax2,
    xlabel="Time", ylabel="Concentration",
    title=("(b) Substrate Concentration", {"size": "x-large"}),
    linestyle=["--"]);
# Time profile of solutions' intermediate concentration
plot_time_profile(conc_sol, observable=x, ax=ax3);
plot_time_profile(
    qssa_sol, observable=x, ax=ax3,
    xlabel="Time", ylabel="Concentration",
    title=("(c) Intermediate Concentration", {"size": "x-large"}),
    linestyle=["--"]);
fig_5_4.tight_layout()
```

**Figure 5.4:** The transient response of the Michaelis-Menten reaction mechanism, for \(k\_2 = k\_{-1}, \ 100e\_0 = K\_m \ \text{and} \ s\_0 = K\_m\). (a) The phase portrait . (b) The substrate concentrations. (c) The intermediate concentrations. The solid and dashed line represent the quasi-steady state and the full numerical solution respectively.

- *The phase portrait*. The phase portrait is shown in of Figure 5.4a and it shows how the reaction rapidly approaches the quasi-steady state line and then moves along that line towards the equilibrium in the origin where the reaction has gone to completion.
- *The fast motion*. With the slider on moved to the left, Figure 5.4 shows the changes in the concentrations during the faster time scale. The intermediate concentration exhibits a significant fast motion, while the substrate does not move far from its initial value.
- *The slow motion*. The changes in the concentrations during the slower time scale are shown when the slider is on the right of Figure 5.4. Both the substrate and the intermediate complex decay towards zero. During the decay process, the complex is in a quasi-stationary state and the motion of the substrate drives the reaction dynamics. The quasi-steady state solution gives a good description of the motion on the slower time scale.

###### Step 6: Identification of dimensionless parameters for Michaelis-Menten kinetics¶

Simulation studies suggests that there are three dimensionless parameters of interest:

\[\begin{equation} a = k\_2/k\_{-1}, \ b = e\_0/K\_m, \ c = s\_0/K\_m \tag{5.13} \end{equation}\]

This result is also found by rigorous mathematical analysis (Palsson, 1984). The dynamic behavior of the reaction is determined by three dimensionless groups: a ratio of kinetic constants and the two initial conditions scaled to \(K\_m\).

1. The first dimensionless group, a, is a ratio consisting only of kinetic constants, \(k\_2/k\_{-1}\). This ratio has been called the ‘stickiness number’  (Palsson, 1984), (Palsson, 1984a), since a substrate is said to stick well to an enzyme if \(k\_2 > k\_{-1}\). Once \(X\) is formed it is more likely to break down to yield the product than to revert back to substrate.
2. The second dimensionless number, \(e\_0/K\_m\), is a dimensionless concentration parameter - the total enzyme concentration relative to the Michaelis constant. This quantity varies from one situation to another and takes particularly different values under *in vitro* and *in vivo* conditions. The enzyme concentrations used *in vitro* are several orders of magnitude lower than the \(K\_m\) values (Masters, 1977), (Srere, 1967), (Srere, 1970). In vivo enzyme concentrations can approach the
   same order of magnitude as \(K\_m\).
3. The third dimensionless ratio, \(s\_0/K\_m\), is the initial condition for the substrate concentration. Typical values for this ratio *in vivo* is on the order of unity.

```
[6]:
```

```
# Define new initial conditions and parameters for integration
s0 = (1/100)

e0 = (1/100)
k2 = 1
Km = 1
params = [k2, e0, Km]

# Obtain numerical solutions
sol_obj = solve_ivp(fun=lambda t, s: qssa(t, s, *params),
                    t_span=(t0, tf), y0=[s0])
s_sol = sol_obj.y
x_sol = np.array([(e0 * val)/(Km + val) for val in s_sol])
# Store solutions into MassSolution Objects
qssa_sol = MassSolution(
    "QSSA", solution_type="Conc",
    data_dict={"s": s_sol[0], "x": x_sol[0]},
    time=sol_obj.t, interpolate=False)

# Update initial conditions for MassModel
model.update_initial_conditions({s: s0})

# Solve
MM_simulation = Simulation(model, verbose=True)
conc_sol, flux_sol = MM_simulation.simulate(model, (t0, tf))
```

```
WARNING: No compartments found in model. Therefore creating compartment 'compartment' for entire model.
```

```
Successfully loaded MassModel 'Michaeli_Menten' into RoadRunner.
```

```
[7]:
```

```
fig_5_5 = plt.figure(figsize=(9, 7))
gs = fig_5_5.add_gridspec(nrows=2, ncols=2, width_ratios=[1, 1.5],
                          height_ratios=[1, 1])

ax1 = fig_5_5.add_subplot(gs[0, 0])
ax2 = fig_5_5.add_subplot(gs[0, 1])
ax3 = fig_5_5.add_subplot(gs[1, 1])
# Phase portrait of both solutions' substrate vs. intermediate
plot_phase_portrait(
    conc_sol, x=s, y=x, ax=ax1, legend=["Numerical Solution"],
    annotate_time_points="endpoints",
    annotate_time_points_color=["r", "b"],
    annotate_time_points_labels=True);
plot_phase_portrait(
    qssa_sol, x=s, y=x, ax=ax1, legend=["QSSA", "lower outside"],
    xlabel=s.id, ylabel=x.id, linestyle=["--"],
    title=("(a) Phase Portrait", {"size": "x-large"}),
    annotate_time_points="endpoints",
    annotate_time_points_labels=True);

# Time profile of solutions' substrate concentration
plot_time_profile(conc_sol, observable=s, ax=ax2);
plot_time_profile(qssa_sol, observable=s, ax=ax2,
                xlabel="Time", ylabel="Concentration",
                title=("(b) Substrate Concentration", {"size": "x-large"}),
                linestyle=["--"]);
# Time profile of solutions' intermediate concentration
plot_time_profile(conc_sol, observable=x, ax=ax3);
plot_time_profile(qssa_sol, observable=x, ax=ax3,
                xlabel="Time", ylabel="Concentration",
                title=("(c) Intermediate Concentration", {"size": "x-large"}),
                linestyle=["--"]);
fig_5_5.tight_layout()
```

**Figure 5.5:** The transient response of the Michaelis-Menten reaction mechanism, for \(k\_2 = k\_{-1},\ 100e\_0 = K\_m \ \text{and} \ s\_0 = K\_m\). (a) The phase portrait . (b) The substrate concentrations. (c) The slow transients. The solid and dashed line represent the quasi-steady state and the full numerical solution respectively.

###### Comment on the criterion \(e\_0 << s\_0\)¶

Historically, the commonly accepted criterion for the applicability of the quasi-steady state assumption is that the initial concentration of the enzyme must be much smaller than that of the substrate. The actual criterion is \(e\_0 << K\_m, \ \text{or} \ b << 1\) (Palsson, 1984). Figure 5.5 shows the reaction dynamics for \(e\_0 = K\_m, \ e\_0 = 100s\_0, \ 100k\_2 = k\_{-1}\), which is analogous to Figure 5.4, except the initial substrate concentration is now a hundred times smaller than
\(K\_m\). In other words, we have \(e\_0 = s\_0 << K\_m\) and, as demonstrated in Figure 5.5, the quasi-steady state assumption is applicable.

##### Hill-kinetics for Enzyme Regulation¶

###### Regulated enzymes¶

Enzyme activity is regulated by the binding of small molecules to the enzyme resulting in an altered enzymatic activity. Such binding can inhibit or activate the catalytic activities of the enzyme. The regulation of enzymes such regulators represents a ‘tug of war’ between the functional states of the enzyme, see Figure 5.6. A simple extension of the oldest reaction mechanisms for ligand binding to oligomeric protein, i.e., oxygen binding to hemoglobin, is commonly used to obtain simple rate
laws for regulated enzymes (Hill, 1910).

**Figure 5.6:** An example of a regulated multimeric enzyme. The T form of the enzyme created by inhibitor binding is inactive, where as the R form, where no inhibitor is bound, is catalytically active. From (Koolman, 2005) (reprinted with permission).

###### The reaction mechanism for Hill-kinetics¶

The Hill reaction mechanism is based on two reactions: a catalytic conversion and the sequestration of the enzyme in an inactive form. It assumes that the catalyzed reaction is an irreversible bi-molecular reaction between the substrate, \(S\), and the enzyme, \(E\), to form the product,\(P\), and the free enzyme in a single elementary reaction:

\[\begin{equation} S + E \stackrel{k}{\rightarrow} E + P \tag{5.14} \end{equation}\]

The enzyme in turn can be put into a catalytically inactive state, \(X\), through binding simultaneously and reversibly to \(\nu\) molecules of an inhibitor, \(I\):

\[\begin{equation} E + {\nu}I \underset{k\_{-i}^-}{\stackrel{k\_{i}^+}{\rightleftharpoons}} X \tag{5.15} \end{equation}\]

Numerical values for \(\nu\) often exceed unity. Thus, the regulatory action of \(I\) is said to be lumped in the simple \(E\) to \(X\) transformation, as values of \(\nu\) greater than 1 are chemically unrealistic. Numerical values estimated from data show that the best fit values for \(\nu\) are not integers; for instance \(\nu\) is found to be around 2.3 to 2.6 for \(O\_2\) binding to hemoglobin. Section 5.5 describes more realistic reaction mechanisms of serial
binding of an inhibitor to a regulated enzyme to sequester it in an inactive form.

###### Step 1: Dynamic mass balances for Hill-kinetics¶

The mass action kinetic equations are

\[\begin{equation} \frac{ds}{dt} = -v\_1, \ \frac{de}{dt} = -v\_2 + v\_3, \ \frac{dp}{dt} = v\_1, \ \frac{di}{dt} = -\nu (v\_2 - v\_3), \ \frac{ds}{dt} = v\_2 - v\_3 \end{equation}\]

where the reaction rates are

\[\begin{equation} v\_1 = kse, \ v\_2 = k\_i^+i^{\nu}e, \ v\_3 = k\_i^-x \tag{5.16} \end{equation}\]

###### Step 2: Finding the time invariants for Hill-kinetics¶

We define \(\textbf{x} = (s, e, p, i, x) \ \text{and} \ \textbf{v} = (ks, k\_i^+i^{\nu}e, k\_i^-x)\). The stoichiometric matrix is then

\[\begin{split}\begin{equation} \textbf{S} = \begin{pmatrix} {-1} & {0} & {0} \\ {0} & {-1} & {1} \\ {1} & {0} & {0} \\ {0} & {-\nu} & {\nu} \\ {0} & {1} & {-1} \\ \end{pmatrix} \end{equation}\end{split}\]

\[\tag{5.17}\]

and has a rank of two. The conservation quantities are a balance on the substrate, the enzyme, and the inhibitor:

\[\begin{equation} s\_0 = s + p, \ e\_0 = e + x, \ i\_0 = i + \nu x \tag{5.18} \end{equation}\]

###### Step 3: Reducing the dynamic description for Hill-kinetics¶

We need two differential equations to simulate the dynamic response and then the remaining three variables can be computed from the conservation relationships. We can choose the substrate, \(s\), and the concentration of the enzyme, \(e\):

\[\begin{equation} \frac{ds}{dt} = kse, \ \frac{de}{dt} = -k\_i^+i^{\nu}e + k\_i^-x \tag{5.19} \end{equation}\]

then \(p\), \(x\) and \(i\) are computed from Eq. (5.18).

###### Step 4: Applying simplifying kinetic assumptions for Hill-kinetics¶

If we assume that the binding of the inhibitor is fast, so that a quasi-equilibrium forms for the reaction of Eq. (5.15), we have

\[\begin{equation} v\_2 = v\_3, \ \text{thus} \ x = (k\_i^+/k\_i^-)i^{\nu}e = (i/K\_i)^{\nu}e, \ \text{and} \ \frac{de}{dt} = \frac{dx}{dt} = \frac{di}{dt} = 0 \tag{5.20} \end{equation}\]

where \(K\_i\) is a “per-site” dissociation constant for Eq. (5.15). The enzyme is in one of two states, so that we have the mass balance

\[\begin{equation} e\_0 = e + x = (1 + (i/K\_i)^{\nu})e \ \text{or} \ e(i) = \frac{e\_0}{1 + (i/K\_i)^{\nu}} \tag{5.21} \end{equation}\]

where \(e\_0\) is the total concentration of the enzyme. Using the mass balance and the quasi-equilibrium assumption gives the flux through the regulated reaction as

\[\begin{equation} v(i) = ke(i)s = \frac{ke\_0s}{1 + (i/K\_i)^{\nu}} = \frac{v\_m}{1 + (i/K\_i)^{\nu}} \tag{5.22} \end{equation}\]

with \(v\_m = ke\_0s\). The Hill model has three parameters: 1) \(\nu\), the degree of cooperativity, 2) \(K\_i\), the dissociation constant for the inhibitor and, 3) \(v\_m\), the maximum reaction rate or the capacity of the enzyme. We note that

\[\begin{equation} f\_e = \frac{e(i)}{e\_0} = \frac{1}{1 + (i/K\_i)^{\nu}} \tag{5.23} \end{equation}\]

represents the fraction of the enzyme that is in the active state. Note that \(f\_e \lt 1\) for any finite concentration of the inhibitor.

```
[8]:
```

```
t0 = 0
tf = 10

def hill(t, state_vars, *params):
    s, p, e, i, x = state_vars
    k1, k_plus,k_minus, nu = params
    # Reaction Rates
    v1 = k1 * s * e
    v2 = k_plus * i**nu * e
    v3 = k_minus * x
    # Differential equations
    diffeqs =[-v1,          # ds/dt
              v1,           # dp/dt
              -v2 + v3,     # de/dt
              -nu*(v2 - v3), # di/dt
              v2 - v3]      # dx/dt
    return diffeqs

# Define initial conditions
s0, p0, e0, i0, x0 = (1, 0, 1, 1, 0)

# Define paramters
k1 = 1
k_plus, k_minus = (100, 100)
nu = 2
params = [k1, k_plus, k_minus, nu]

# Obtain numerical solutions
sol_obj = solve_ivp(
    fun=lambda t, state_vars: hill(t, state_vars, *params),
    t_span=(t0, tf), y0=[s0, p0, e0, i0, x0])
# Store solutions into Solution Objects
sol_dict = dict(zip(["s", "p", "e", "i", "x"], sol_obj.y))
hill_sol = MassSolution(
    "Hill", solution_type="Conc", data_dict=sol_dict,
    time=sol_obj.t, interpolate=False)
```

```
[9]:
```

```
fig_5_7 = plt.figure(figsize=(9, 8))
gs = fig_5_7.add_gridspec(nrows=2, ncols=2, width_ratios=[1, 1],
                          height_ratios=[1, 1])

ax1 = fig_5_7.add_subplot(gs[0, 0])
ax2 = fig_5_7.add_subplot(gs[0, 1])
ax3 = fig_5_7.add_subplot(gs[1, 0])
ax4 = fig_5_7.add_subplot(gs[1, 1])

plot_phase_portrait(
    hill_sol, x="s", y="e", ax=ax1, xlabel="s", ylabel="e",
    xlim=(-0.05, 1.05), ylim=(-0.05, 1.05),
    title=("(a) Phase Portrait of s vs. e", {"size": "x-large"}),
    annotate_time_points="endpoints",
    annotate_time_points_labels=True);
plot_phase_portrait(
    hill_sol, x="e", y="x", ax=ax2, xlabel="e", ylabel="x",
    xlim=(-0.05, 1.05), ylim=(-0.05, 1.05),
    title=("(b) Phase Portrait of e vs. x", {"size": "x-large"}),
    annotate_time_points="endpoints",
    annotate_time_points_labels=True);

plot_phase_portrait(
    hill_sol, x="i", y="x", ax=ax3, xlabel="i", ylabel="x",
    xlim=(-0.05, 1.05), ylim=(-0.05, 1.05),
    title=("(a) Phase Portrait of i vs. x", {"size": "x-large"}),
    annotate_time_points="endpoints",
    annotate_time_points_labels=True);
plot_time_profile(
    hill_sol, ax=ax4, legend="right outside",
    title=("(d) Concentration Profiles", {"size": "x-large"}));
fig_5_7.tight_layout()
```

**Figure 5.7:** The transient response of the Hill reaction mechanism, for \(k\_i^+ = k\_i^- = 100\), \(k = 1\), \(\nu = 2\), \(x\_0 = 0\) and \(e\_0 = s\_0 = i\_0 = 1\). (a) The phase portraits of \(s\) and \(e\). (b) The phase portraits of \(e\) and \(x\). (c) The phase portraits of \(i\) and \(x\). (d) The concentration profiles.

###### Step 5: Numerical solutions for Hill-kinetics¶

The dynamic response of the Hill reaction mechanism is shown in Figure 5.7. The trace in the \(s\) vs. \(e\) phase portrait is L-shaped, showing a rapid initial equilibration of the enzyme to the inhibitor (the vertical line), followed by the slower conversion of the product (the horizontal line). These two reactions are naturally (stoichiometrically) decoupled and separated in time for the numerical values of the kinetic constants used.

The phase portraits for \(e\) vs. \(x\) and \(i\) vs. \(x\) are straight lines as given by the conservation Eq. (5.18), see Figure 5.7b,c. The two phase transient responses in Figure 5.7d shows the rapid equilibration of the enzyme and the slow conversion of substrate. Under these parameter conditions, the QEA should give good results.

###### Step 6: Estimating key parameters¶

There are two features of the Hill rate law that are of interest:

###### Applicability of the quasi-equilibrium assumption.¶

Given the fact that the two reactions have characteristic times scales, their relative magnitude is of key concern when it comes to the justification of the QEA:

\[\begin{equation} a = (\frac{\text{characteristic binding time of the inhibitor}}{\text{characteristic turnover time of the substrate}}) = \frac{k}{k\_i^+} \tag{5.24} \end{equation}\]

If \(a\) is much smaller than unity, we would expect the QEA to be valid. In Figure 5.7, \(a\) is 0.01.

###### Regulatory characteristics¶

The Hill rate law has a sigmoidal shape with sensitivity of the reaction rate to the end product concentration as

\[\begin{equation} v\_i = \frac{\partial v}{\partial i} = \frac{-\nu v\_m}{i} \frac{(i/K\_i)^{\nu}}{[1 + (i/K\_i)^{\nu}]^2} \tag{5.25} \end{equation}\]

which has a maximum

\[\begin{equation} v\_i^\* = -\frac{v\_m}{K\_i}N(\nu) \ \text{where} \ N(\nu) = \frac{1}{4\nu}(\nu - 1)^{1 - 1/\nu}(\nu + 1)^{1 + 1/\nu} \tag{5.26} \end{equation}\]

at the inflection point

\[\begin{equation} i^\* = K\_i(\frac{\nu - 1}{\nu + 1})^{1/\nu} \tag{5.27} \end{equation}\]

For plausible values of \(\nu\), the function \(N(\nu)\) is on the order of unity (Table 5.1), and hence the maximum sensitivity \(v\_i^\*\) is on the order of \((-v\_m/K\_i)\). The ratio \((K\_i/v\_m)\) can be interpreted as a time constant characterizing the inhibition process;

\[\begin{equation} t\_i = \frac{K\_i}{v\_m} = [\frac{\text{concentration}}{\text{concentration/time}}]\tag{5.28} \end{equation}\]

This estimate represents an upper bound since the steady state concentration of \(i\) can be different from \(i^\*\). The turnover of the substrate happens on a time scale defined by the rate constant \(t\_s = 1/k\). Thus, a key dimensionless property is

\[\begin{equation} b = \frac{t\_s}{t\_i} = \frac{1/k}{K\_i/v\_m} = \frac{v\_m}{kK\_i} = \frac{e\_t}{K\_i} \tag{5.29} \end{equation}\]

Therefore, the dimensionless parameter \(b\) can be interpreted as a ratio of time constants or as a ratio of concentration ranges.

**Table 5.1:** The values of the function \(N(\nu)\) and \(i^\*/K\_i\) at the inflection point.

```
[10]:
```

```
def N(nu): # N(v)
    return (1/(4*nu))*((nu-1)**(1-1/nu))*((nu+1)**(1+1/nu))

def i_Ki(nu): # i*/Ki
    return ((nu-1)/(nu+1))**(1/nu)

cols = [nu for nu in np.linspace(2, 5, 4)]
tab_5_1 = pd.DataFrame([[round(N(nu), 2) for nu in cols],
                        [round(i_Ki(nu), 2) for nu in cols]],
                      index=['N($\\nu$)', '$i^{*}$$/K_{i}$'], columns=cols)
tab_5_1.index.rename('$\\nu$', inplace=True)
tab_5_1
```

```
[10]:
```

|  | 2.0 | 3.0 | 4.0 | 5.0 |
| --- | --- | --- | --- | --- |
| $\nu$ |  |  |  |  |
| N($\nu$) | 0.65 | 0.84 | 1.07 | 1.30 |
| $i^{\*}$$/K\_{i}$ | 0.58 | 0.79 | 0.88 | 0.92 |

##### The Symmetry Model¶

The regulatory molecules are often chemically quite different than the substrate molecule. They thus often have a different binding site on the protein molecule than the catalytic site. It called an *allosteric site*. One of the earliest enzyme kinetic models that accounted for allosterism was the symmetry model (Monod, 1965), named after certain assumed symmetry properties of the subunits of the enzyme. It is a mechanistically realistic description of regulatory enzymes. An example of a
multimeric regulatory enzyme is given in Figure 5.6.

###### The reaction mechanism for the symmetry model¶

The main chemical conversion in the symmetry model is as before and is described by Equation (5.14). The symmetry model postulates that the regulated enzyme lies naturally in two forms, \(E\) and \(X\), and is converted between the two states simply as

\[\begin{equation} E \underset{k\_{-}}{\stackrel{k\_+}{\rightleftharpoons}} X \tag{5.30} \end{equation}\]

The equilibrium constant for this reaction,

\[\begin{equation} L = k\_+/k\_- = x/e \tag{5.31} \end{equation}\]

has a special name, the allosteric constant. Then \(\nu\) molecules of an inhibitor, \(I\), can bind sequentially to \(X\) as

\[\begin{split}\begin{equation} \begin{matrix} {X} & {+} & {I} & {\underset{k\_i^-}{\stackrel{\nu k\_i^+}{\rightleftharpoons}}} & {X\_1} \\ {X\_1} & {+} & {I} & {\underset{2 k\_i^-}{\stackrel{(\nu-1) k\_i^+}{\rightleftharpoons}}} & {X\_2} \\ {\vdots} & {} & {} & {} & {\vdots} \\ {X\_{\nu - 1}} & {+} & {I} & {\underset{\nu k\_i^-}{\stackrel{k\_i^+}{\rightleftharpoons}}} & {X\_{\nu}} \\ \end{matrix}\end{equation} \tag{5.32}\end{split}\]

where the binding steps have the same dissociation constant, \(K\_i = k\_i^- / k\_i^+\). We will discuss the most common case of a tetramer here, i.e., \(\nu = 4\), see Figure 5.8.

**Figure 5.8:** The reaction mechanisms for the symmetry model. The enzyme has four binding sites for the inhibitor.

###### Step 1: Dynamic mass balances for the symmetry model¶

The conversion rate of the substrate is

\[\begin{equation} v = kse \tag{5.33} \end{equation}\]

whereas the enzyme sequestration is characterized by the reaction rates

\[\begin{split}\begin{equation} \begin{matrix} {v\_1 = k^+e,} & {v\_2 = k^-x,} & {v\_3 = 4k\_i^+xi,} \\ {v\_4 = k\_i^-x\_1,} & {v\_5 = 3 k\_i^+x\_1i,} & {v\_6 = 2k\_i^-x\_2,} \\ {v\_7 = k\_i^+x\_2i,} & {v\_8 = k\_i^-x\_3,} & {v\_9 = k\_i^+x\_3i,} \\ {} & {v\_{10} = 4k\_i^-x\_4} & {} \\ \end{matrix} \end{equation} \tag{5.34}\end{split}\]

The dynamic mass balances on the various states of the enzyme are:

\[\begin{split}\begin{align} \frac{de}{dt} &= -v\_1 + v\_2\\ \frac{dx}{dt} &= v\_1 - v\_2 - v\_3 + v\_4\\ \frac{di}{dt} &= -v\_3 + v\_4 - v\_5 + v\_6 - v\_7 + v\_8 - v\_9 + v\_{10} \\ \frac{dx\_1}{dt} &= v\_3 - v\_4 - v\_5 + v\_6 \\ \frac{dx\_2}{dt} &= v\_5 - v\_6 - v\_7 + v\_8 \\ \frac{dx\_3}{dt} &= v\_7 - v\_8 - v\_9 + v\_{10} \\ \frac{dx\_4}{dt} &= v\_9 - v\_{10} \\ \end{align} \tag{5.35}\end{split}\]

###### Step 2: Finding the time invariants for the symmetry model¶

The stoichiometric matrix for \(\textbf{x} = (e, x, i, x\_1, x\_2, x\_3, x\_4)\) is a 7x10 matrix:

\[\begin{split}\begin{equation} \textbf{S} = \begin{pmatrix} {-1} & {1} & {0} & {0} & {0} & {0} & {0} & {0} & {0} & {0} \\ {1} & {-1} & {-1} & {1} & {0} & {0} & {0} & {0} & {0} & {0} \\ {0} & {0} & {-1} & {1} & {-1} & {1} & {-1} & {1} & {-1} & {1} \\ {0} & {0} & {1} & {-1} & {-1} & {1} & {0} & {0} & {0} & {0} \\ {0} & {0} & {0} & {0} & {1} & {-1} & {-1} & {1} & {0} & {0} \\ {0} & {0} & {0} & {0} & {0} & {0} & {1} & {-1} & {-1} & {1} \\ {0} & {0} & {0} & {0} & {0} & {0} & {0} & {0} & {1} & {-1} \\ \end{pmatrix} \end{equation} \tag{5.36}\end{split}\]

that has a rank of 5. Thus, there are two conservation relationships, for the enzyme: \(e\_0 = e + x + x\_1 + x\_2 + x\_3 + x\_4\);, and, for the inhibitor: \(i\_0 = i + x\_1 + 2x\_2 + 3x\_3 + 4x\_4\). If the dynamic mass balances on the substrate and product are taken into account, a third conservation \(s\_0 = s + p\) appears.

###### Step 3: Reducing the dynamic description for the symmetry model¶

We leave it to the reader to pick two dynamic variables from the full kinetic model as the dependent variables and then eliminate them from the dynamic description using the conservation relationships. The impetus for doing so algebraically becomes smaller as the number of differential equations grows. Most standard software packages will integrate a dynamically redundant set of differential equations and such substitution is not necessary to obtain the numerical solutions.

###### Step 4: Using simplifying kinetic assumptions to derive a rate law for the symmetry model¶

The serial binding of an inhibitor to X that has four binding sites is shown in Figure 5.8. The derivation of the rate law is comprised of four basic steps:

1. Mass balance on enzyme:

\[\begin{equation} e\_0 = e + x + x\_1 + x\_2 + x\_3 + x\_4 \tag{5.37} \end{equation}\]

2. QEA for binding steps:

\[\begin{split}\begin{align} 4k\_i^+ix = k\_i^-x\_1 \ &\Rightarrow \ x\_1 = \frac{4}{1}x (i/K\_i) = 4x(i/K\_i) \\ 3k\_i^+ix\_1 = 2k\_i^-x\_2 \ &\Rightarrow \ x\_2 = \frac{3}{2}x\_1(i/K\_i) = 6x(i/K\_i)^2 \\ 2k\_i^+ix\_2 = 3k\_i^-x\_3 \ &\Rightarrow \ x\_3 = \frac{2}{3}x\_2(i/K\_i) = 4x(i/K\_i)^3 \\ k\_i^+ix\_3 = 4k\_i^-x\_4 \ &\Rightarrow \ x\_4 = \frac{1}{4}x\_3(i/K\_i) = x(i/K\_i)^4 \\ \end{align} \tag{5.38}\end{split}\]

3. Combine 1 and 2:

\[\begin{split}\begin{align} e\_0 &= e + x + 4x(i/K\_i) + 6x(i/K\_i)^2 + 4x(i/K\_i)^3 + x(i/K\_i)^4 \\ &= e + x(1 + (i/K\_i))^4 \ \text{where} \ x=Le \\ &= e(1 + L(1 + (i/K\_i)))^4 \end{align} \tag{5.39}\end{split}\]

4. Form the rate law: The reaction rate is given by: \(v = kse\). We can rewrite the last part of Eq. (5.39) as:

\[\begin{equation} e = \frac{e\_0}{1 + L(1 + i/K\_i)^4} \tag{5.40} \end{equation}\]

leading to the rate law:

\[\begin{equation} v(s, i) = \frac{ke\_0s}{1 + L(1 + i/K\_i)^4} \tag{5.41} \end{equation}\]

This rate law generalizes to:

\[\begin{equation} v(s, i) = \frac{ke\_0s}{1 + L(1 + i/K\_i)^{\nu}} = \frac{v\_m}{1 + L(1 + i/K\_i)^{\nu}} \tag{5.42} \end{equation}\]

for any \(\nu\). The reader can find the same key dimensionless groups as for the Hill rate law. Note again the fraction

\[\begin{equation} f\_e = \frac{e}{e\_0} = \frac{1}{1 + L(1 + i/K\_i)^{\nu}} \tag{5.43} \end{equation}\]

that describes the what fraction of the enzyme is in the catalytically active state.

```
[11]:
```

```
t0 = 0
tf = 15

def symmetry(t, state_vars, *params):
    s, p, e, i, x, x1, x2, x3, x4 = state_vars
    k1, k_plus, k_minus, ki_plus, ki_minus = params
    # Enzyme Reaction Rates
    v1 = k_plus * e;           v2 = k_minus * x;
    v3 = 4 * ki_plus * i * x;  v4 = ki_minus * x1;
    v5 = 3 * ki_plus * i * x1; v6 = 2 * ki_minus * x2;
    v7 = 2 * ki_plus * i * x2; v8 = 3 * ki_minus * x3;
    v9 = ki_plus * i * x3;    v10 = 4 * ki_minus * x4;
    # Differential equations to integrate
    diffeqs = [-k1 * s * e,                             #  ds/dt
               k1 * s * e,                              #  dp/dt
               -v1 + v2,                                #  de/dt
               -v3 + v4 - v5 + v6 - v7 + v8 - v9 + v10, #  di/dt
               v1 - v2 - v3 + v4,                       #  dx/dt
               v3 - v4 - v5 + v6,                       # dx1/dt
               v5 - v6 - v7 + v8,                       # dx2/dt
               v7 - v8 - v9 + v10,                      # dx3/dt
               v9 - v10]                                # dx4/dt
    return diffeqs

# Define initial conditions
s0, p0, e0, i0, x0 = (1, 0, 1, 1, 0)
x1_0, x2_0, x3_0, x4_0 = (0, 0, 0, 0)

# Define paramters
k1 = 1;
k_plus, k_minus = (100, 100)
ki_plus, ki_minus = (2, 2)
params = [k1, k_plus,k_minus, ki_plus, ki_minus]

# Obtain numerical solutions
sol_obj = solve_ivp(fun=lambda t, state_vars: symmetry(t, state_vars, *params),
                    t_span=(t0, tf), y0=[s0, p0, e0, i0, x0, x1_0, x2_0, x3_0, x4_0])
# Store solutions into Solution Objects
sol_dict = dict(zip(["s", "p", "e", "i", "x", "x1", "x2", "x3", "x4"], sol_obj.y))

x_total = sum(sol_dict[k] for k in ["x", "x1", "x2", "x3", "x4"])
i_bound = sum(i*sol_dict[k] for i, k in zip([1, 2, 3, 4], ["x1", "x2", "x3", "x4"]))

sol_dict.update({"x_total": x_total, "i_bound": i_bound})

symmetry_sol = MassSolution(
    "Symmetry", solution_type="Conc", data_dict=sol_dict,
    time=sol_obj.t, interpolate=False)
```

```
[12]:
```

```
fig_5_9 = plt.figure(figsize=(10, 8))
gs = fig_5_9.add_gridspec(nrows=2, ncols=2, width_ratios=[1, 1],
                          height_ratios=[1, 1])

ax1 = fig_5_9.add_subplot(gs[0, 0])
ax2 = fig_5_9.add_subplot(gs[0, 1])
ax3 = fig_5_9.add_subplot(gs[1, 0])
ax4 = fig_5_9.add_subplot(gs[1, 1])

plot_phase_portrait(
    symmetry_sol, x="s", y="e", ax=ax1, xlabel="s", ylabel="e",
    xlim=(-0.05, 1.05), ylim=(-0.05, 1.05),
    title=("(a) Phase Portrait of s vs. e", {"size": "x-large"}),
    annotate_time_points="endpoints",
    annotate_time_points_labels=True);

plot_phase_portrait(
    symmetry_sol, x="e", y="x_total", ax=ax2,
    xlabel="e", ylabel='x + x1 + x2 + x3 + x4',
    xlim=(-0.05, 1.05), ylim=(-0.05, 1.05),
    title=("(b) Phase Portrait of e vs. x_total", {"size": "x-large"}),
    annotate_time_points="endpoints",
    annotate_time_points_labels=True);

plot_phase_portrait(
    symmetry_sol, x="i", y="i_bound", ax=ax3,
    xlabel="i", ylabel='1*x1 + 2*x2 + 3*x3 + 4*x4',
    xlim=(-0.05, 1.05), ylim=(-0.05, 1.05),
    title=("(a) Phase Portrait of i vs. x", {"size": "x-large"}),
    annotate_time_points="endpoints",
    annotate_time_points_labels=True);

plot_time_profile(
    symmetry_sol, observable=list(
        k for k in symmetry_sol.keys() if k not in [
            "x", "x1", "x2", "x3", "x4"]),
    ax=ax4, legend="right outside",
    title=("(d) Concentration Profiles", {"size": "x-large"}));
fig_5_9.tight_layout()
```

**Figure 5.9:** The transient response of the symmetry model, for \(k^+ = k^- = 100\), \(k\_i^+ = k\_i^- = 2\), \(k = 1\), \(\nu = 4\), \(x\_0 = x\_{1, 0} = x\_{2, 0} = x\_{3, 0} = x\_{4, 0} = 0\) and \(e\_0 = s\_0 = i\_0 = 1\). (a) The phase portraits of \(s\) and \(e\). (b) The phase portraits of \(e\) and \((x + x\_1 + x\_2 + x\_3 + x\_4)\). (c) The phase portraits of \(i\) and \((x\_1 + 2x\_2 + 3x\_3 + 4x\_4)\). (d) Concentration and pool profiles.

###### Step 5: Numerical solutions for the symmetry model¶

These equations can be simulated. Typically the conformational changes between \(E\) and \(X\) are fast as are the inhibitor binding steps relative to the catalysis rate. Numerical simulations were carried out for this situation and the results are plotted in Figure 5.9.

- Figure 5.9a shows how the substrate-enzyme phase portrait is L-shaped showing that the sequestration of the enzyme in the inhibited form (the vertical line) is faster than the conversion of the substrate (the horizontal line).
- Figure 5.9b shows the redistribution of the total enzyme among the active and inactive forms, that is, \(e\) vs. \((x + x\_1 + x\_2 + x\_3 + x\_4)\). The fraction of the enzyme in the inactive form is about 0.29.
- Figure 5.9c shows the redistribution of the inhibitor between the free and bound form; \(i\) vs. \((x\_1 + 2x\_2 + 3x\_3 + 4x\_4)\). This panel shows that the fraction the inhibitor that is bound is high, 0.70.
- Finally, Figure 5.9d show the transient changes in the concentrations and pools on the fast and slow time scales. Note that two natural aggregation variables appear: the total enzyme in the inactive form, and the total number of inhibitor molecules bound to the enzyme.

##### Scaling Dynamic Descriptions¶

The analysis of simple equations requires the “proper frame of mind.” In step 6 of the process of formulating rate laws, this notion is translated into quantitative measures. We need to scale the variables with respect to intrinsic reference scales and thereby cast our mathematical descriptions into appropriate coordinate systems. All parameters then aggregate into dimensionless property ratios that, if properly interpreted, have a clear physical significance.

###### The scaling process:¶

The examples above illustrate the decisive role of time constants and their use to analyze simple situations and to elucidate intrinsic reference scales. Identification of unimportant terms is sometimes more difficult and familiarity with a formal scaling procedure is useful. This procedure basically consists of four steps:

1. Identify logical reference scales. This step is perhaps the most difficult. It relies partly on physical intuition, and the use of time constants is surprisingly powerful even when analyzing steady situations.
2. Introduce reference scales into the equations and make the variables dimensionless.
3. Collect the parameters into dimensionless property ratios. The number of dimensionless parameters is always the same and it is given by the well-known Buckingham Pi theorem.
4. Interpret the results. The dimensionless groups that appear can normally be interpreted as ratios of the time constants, such as those discussed above.

Scaling of equations is typically only practiced for small models and for analysis purposes only. Numerical simulations of complex models are essentially always performed with absolute values of the variables.

###### The importance of intrinsic reference scales¶

The process by which the equations are made dimensionless is not unique. The ‘correct’ way of putting the equations into a dimensionless form, where judgments of relative orders of magnitude can be made, is called *scaling*. The scaling process is defined by Lin and Segel (Segel, 1974) as:

“…select intrinsic reference quantities so that each term in the dimensional equations transforms into a product of a constant dimensional factor which closely estimates the term’s order of magnitude and a dimensionless factor of unit order of magnitude.”

In other words, if one has an equation which is a sum of terms \(T\_i\) as:

\[\begin{equation} T\_1 + T\_2 + \dots = 0 \tag{5.44} \end{equation}\]

one tries to scale the *variables* involved so that they are of unit order of magnitude or

\[\begin{equation} t\_i = \frac{\text{variable}\_i}{\text{intrinistic reference scale}\_i} \approx \text{unit order of magnitude} \tag{5.45} \end{equation}\]

Introducing these dimensionless variables into equation (5.44) results in the dimensionless form:

\[\begin{equation} \pi\_1 t\_1 + \pi\_2 t\_2 + \dots = 0 \tag{5.44} \end{equation}\]

where the dimensionless multipliers, \(\pi\_i\) are the dimensionless groups and they will indicate the order of magnitude of the product, \(\pi\_it\_i\). Once the equations are in this form, order of magnitude judgements can be made based on the dimensionless groups.

##### Summary¶

- Enzymes are highly specialized catalysts that can dramatically accelerate the rates of biochemical reactions.
- Reaction mechanisms are formulated for the chemical conversions carried out by enzymes in terms of elementary reactions.
- Rate laws for enzyme reaction mechanisms are derived based on simplifying assumptions.
- Two simplifying assumptions are commonly used: the quasi-steady state (QSSA) and the quasi-equilibrium assumptions (QEA).
- The validity of the simplifying assumptions can be determined using scaling of the equations followed by mathematical and numerical analysis.
- A number of rate laws have been developed for enzyme catalysis and for the regulation of enzymes. Only three reaction mechanisms were described in this chapter.

\(\tiny{\text{© B. Ø. Palsson 2011;}\ \text{This publication is in copyright.}\\ \text{Subject to statutory exception and to the provisions of relevant collective licensing agreements,}\\ \text{no reproduction of any part may take place without the written permission of Cambridge University Press.}}\)

#### Open Systems¶

All of the examples in the previous two chapters were closed systems. The ultimate state of closed, chemically reacting systems is chemical equilibrium. Living systems are characterized by mass and energy flow across their boundaries that keep them away from equilibrium. We now discuss open systems, which allow molecules to enter and leave. Open systems ultimately reach a steady state that is often close to a homeostatic state of interest.

**MASSpy** will be used to demonstrate some of the topics in this chapter.

```
[1]:
```

```
from mass import (
    MassModel, MassMetabolite, MassReaction, Simulation, MassSolution)
from mass.visualization import plot_time_profile, plot_phase_portrait
```

Other useful packages are also imported at this time.

```
[2]:
```

```
import matplotlib.pyplot as plt
```

##### Basic Concepts¶

There are several fundamental concepts that need to be understood when one considers open systems. We discuss the more significant ones in this section.

###### The system boundary¶

Implicit in the term ‘open system’ is the notion of an inside and an outside, the division of the world into two domains. The separation between the two is the *system boundary,* which thus defines what is inside a system and belongs to it, and what is outside.

The definition of a boundary can be physical. An example of a physical boundary may be the cell wall, that clearly defines what is inside and what is outside. Similarly, the outer membrane of the mitochondria can serve as a clearly defined physical system boundary. Thus, systems can have hard, immovable boundaries or soft, flexible ones. In the latter case, the volume of the system may be changing.

The definition of a boundary can also be virtual. For instance, we can define a pathway, such as the TCA cycle, as a system, or the amino acid biosynthetic pathways in a cell as a system. In both cases we might draw dashed lines around it on the metabolic map to indicate the system boundary.

###### Crossing the boundary: inputs and outputs¶

Once a system boundary has been established, we can identify and define interactions across it. They are the *inputs* and *outputs* of the system. In most cases, we will be considering mass flows in and out of a system; molecules coming and going. Such flows are normally amenable to experimental determination.

There may be other quantities crossing the system boundary. For instance, if we are considering photosynthesis, photons are crossing the system boundary providing a net influx of energy. Forces can also act on the system boundary. For instance, if the number of molecules coming into and leaving a system through a coupled transport mechanism is not balanced, then osmotic pressure may be generated across the system boundary. Cells have sodium-potassium pumps that displace an uneven number of two
types of cations to deal with osmotic imbalances.

###### Perturbations and boundaries¶

In most analyses of living systems, we are concerned about changes in the environment. The temperature may change, substrate availability may change, and so on. Such changes in the environment can be thought of as forcing functions to which the living system responds. These are one-way interactions, from the environment to the system. In most cases, we consider the system to be small (relative to the environment) and that the environment buffers the activities of the system. For instance, the
environment may provide an infinite sink for cellular waste products.

In other cases, the activities of the system influence the state of the environment. In batch fermentation, for example, the metabolic activities will substantially change the chemical composition of the medium during the course of the fermentation. On a global scale, we are now becoming more concerned about the impact that human activities have on the climate and the larger environment of human socio-economic activities. In such cases, there are two-way interactions between the system and the
environment that need to be described.

###### Inside the boundary: the internal network¶

The definition of a system boundary determines what is inside. Once we know what is inside the system, we can determine the network of chemical transformations that takes place. This network has topology and its links have kinetic properties.

In most cases, it is hard to measure the internal state of a system. There typically are only a few non-invasive measurements available. Occasionally, there are probes that we can use to observe the internal activities. Tracers, such as \(^{13}C\) atoms strategically placed in substrates, can be used to trace input to output and allow us to determine pieces of the internal state of a system. Tearing a system apart to enumerate its components is of course possible, but the system is destroyed
in the process. Thus, we are most often in the situation where we cannot fully experimentally determine the internal state of a system, and may have to be satisfied with only partial knowledge.

###### From networks to system models¶

Mathematical models may help us to simulate or estimate the internal state of the system. Sometimes we are able to bracket its state based on the inputs and outputs and our knowledge of the internal network.

Full dynamic simulation requires extensive knowledge about the internal properties of a system. Detailed models that describe the dynamic state of a system require the definition of the system boundary, the inputs and outputs, the structure of the internal network, and the kinetic properties of the links in the network. We can then simulate the response of the system to various perturbations, such as changes in the environmental conditions.

###### The functional state¶

Once a system model has been formulated, it can be used to compute the functional state of the system for a given set of conditions. Closed systems will eventually go to chemical equilibrium. However, open systems are fundamentally different. Due to continuous interactions with the environment, they have internal states that are either dynamic or steady. A system that has fast internal time constants relative to changes in the environment will reach a steady state, or a quasi-steady state. For a
biological system, such functional states are called *homeostatic states*. Such states are maintained through energy dissipation. The flow of energy in has to exceed the energy leaving the system. This difference allows living systems to reach a functional homeostatic state.

**Figure 6.1:** Open systems. (a) example production of ATP from pyruvate by mitochondria (Prepared by Nathan Lewis). (b) Basic definitions associated with an open system.

##### Reversible reaction in an Open Environment¶

In an open environment, the simple reaction of Eq. (4.1) has an inflow \((b\_1)\) of \(x\_1\) and an outflow \((b\_2)\) of \(x\_2\):

\[\begin{equation} \stackrel{b\_1}{\rightarrow} x\_1 \underset{v\_{-1}}{\stackrel{v\_1}{\rightleftharpoons}} x\_2 \stackrel{b\_2}{\rightarrow} \tag{6.1} \end{equation}\]

The input is fixed by the environment and there is a first-order rate for the product to leave the system and thus we have

\[\begin{equation} b\_1 = \text{constant and}\ b\_2 = k\_2x\_2 \end{equation}\]

This defines the system boundary, and the inputs and outputs.

The stoichiometric matrix is

\[\begin{split}\begin{equation} \textbf{S} = \begin{pmatrix} {1} & {-1} & {1} & {0} \\ {0} & {1} & {-1} & {-1} \\ \end{pmatrix} \end{equation} \tag{6.2}\end{split}\]

where \(\textbf{x} = (x\_1,\ x\_2)\) and \(\textbf{v} = (b\_1, \ k\_1x\_1, \ k\_{-1}x\_2, \ k\_2x\_2)\) The stoichiometric matrix has a rank of 2 and is thus a two-dimensional dynamic system. The differential equations that will need to be solved are:

\[\begin{equation} \frac{dx\_1}{dt} = b\_1 - k\_1x\_1 + k\_{-1}x\_2, \
\frac{dx\_2}{dt} = k\_1x\_1 - k\_{-1}x\_2 - k\_2x\_2 \tag{6.3} \end{equation}\]

There are no conservation quantities.

###### The steady states¶

There are three properties of the steady state of interest. We can find first the steady state fluxes and second the steady state concentrations. Third, we can determine the difference between the steady state and the equilibrium state of this open system.

###### Steady state fluxes:¶

The steady state of the fluxes is given by

\[\begin{equation} \textbf{Sv}\_{ss} = 0 \tag{6.4} \end{equation}\]

and thus \(\textbf{v}\_{ss}\) resides in the null space of \(\textbf{S}\). For this matrix, the null space is two dimensional; \(\text{Dim}(\text{Null}(\textbf{S}))=n-r=4-2=2\), where \(n=4\) is the number of fluxes and the rank is \(r=2\). The null space is spanned by two pathway vectors: (1,1,0,1) and (0,1,1,0). The former is the path through the system while the latter corresponds to the reversible reaction. These are known as type 1 and type 3 extreme pathways,
respectively (*Systems Biology: Properties of Reconstructed Networks*). All steady state flux states of the system are a non-negative combination of these two vectors.

\[\begin{split}\begin{align} \textbf{v}\_{ss} &= (b\_1, \ k\_1x\_{1, ss}, \ k\_{-1}x\_{2, ss}, \ k\_2x\_{2, ss}) \tag{6.5} \\ &= a(1, 1, 0, 1) + b(0, 1, 1, 0), \ a \geq 0, \ b \geq 0 \tag{6.6} \end{align}\end{split}\]

###### Steady state concentrations:¶

The concentrations in the steady state can be evaluated from

\[\begin{equation} 0 = b\_1 - k\_1x\_1 + k\_{-1}x\_2, \ 0 = k\_1x\_1 - k\_{-1}x\_2 - k\_2x\_2 \end{equation}\]

\[\tag{6.7}\]

If we add these equations we find that \(x\_{2, ss} = b\_1 / k\_2\). This concentration can then be substituted into either of the two equations to show that \(x\_{1, ss} = (1 + k\_{-1}/k\_2)(b\_1/k\_1)\). Thus, the steady state concentration vector is

\[\begin{split}\begin{equation} \textbf{x}\_{ss} = \begin{pmatrix} {x\_{1, ss}} \\ {x\_{2, ss}} \end{pmatrix} = (\frac{b\_1}{k\_2})\begin{pmatrix} {\frac{k\_2 + k\_{-1}}{k\_1}} \\ {1} \end{pmatrix} \end{equation}\end{split}\]

\[\tag{6.8}\]

These steady state concentrations can be substituted into the steady state flux vector to get

\[\begin{split}\begin{align} \textbf{v}\_{ss} &= b\_1(1, 1+(\frac{k\_{-1}}{k\_2}), \ (\frac{k\_{-1}}{k\_2}), \ 1) \tag{6.9} \\ &= a(1, 1, 0, 1) + b(0, 1, 1, 0), \ a = b\_1, \ b = (\frac{b\_1k\_{-1}}{k\_2}) \end{align}\end{split}\]

Therefore, the steady state flux distribution is a summation of the straight through pathway and the discounted flux through the reversible reaction. The key quantity is \(k\_{-1} / k\_2\) that measures the relative rate of \(x\_2\) reacting back to form \(x\_1\) versus the rate at which it leaves the system.

###### The distance from the equilibrium state:¶

The difference between the steady state and the equilibrium state can be measured by:

\[\begin{equation} \frac{x\_{2, ss}/x\_{1, ss}}{x\_{2, eq}/x\_{1, eq}} = \frac{1}{1 + k\_2/k\_{-1}} \stackrel{k\_2 << k\_{-1}}{\longrightarrow} 1 \tag{6.11} \end{equation}\]

Thus, when \(k\_2 << k\_{-1}\), the steady state approaches the equilibrium state (recall that \((x\_{2, ss}/x\_{1, ss})/(x\_{2, eq}/x\_{1, eq}) = \Gamma/K\_{eq}\) Section 2.2). If the exchange with the environment is slow relative to the internal reaction rates, the internal system approaches that of an equilibrium state.

###### Dynamic states for reversible reactions¶

The dynamic states are computed from the dynamic mass balances for a given condition. We are interested in two dynamic states: the approach to the steady state, and the response to a change in the input flux, \(b\_1\)

###### A two-phase transient response:¶

Simulation of this system with an input rate of \(b\_1 = 0.01\) and a slow removal rate, \(k\_2 = 0.1\), from an initial state of \(x\_1(0) = 1.0\) and \(x\_2(0) = 0\) is shown in Figure 6.2. There are two discernible time scales: a rapid motion of the equilibrating reaction, and a slow removal of \(x\_2\) from the system, Figure 6.2b. A phase portrait shows a rapid movement along a line with a negative slope of 1, showing the existence of a conservation quantity
\((x\_1 + x\_2)\) on a fast time scale, followed by a slow motion down a quasi-equilibrium line with a slope of 1/2 to a steady state point. Note the difference from Figure 4.4c.

```
[3]:
```

```
# Create MassModel
model = MassModel('Linear_Reversible_Open')
# Generate the MassMetabolites
x1 = MassMetabolite("x1")
x2 = MassMetabolite("x2")

# Generate the MassReactions
b1 = MassReaction("b1", reversible=False)
v1 = MassReaction("v1")
b2 = MassReaction("b2", reversible=False)
# Add metabolites to the reaction, add reaction to the model
b1.add_metabolites({x1: 1})
v1.add_metabolites({x1: -1, x2: 1})
b2.add_metabolites({x2: -1})
model.add_reactions([b1, v1, b2])
# Set parameters
b1.kf = 0.01
v1.kf, v1.kr = (1, 2)
b2.kf = 0.1

# Set initial conditions for model
x1.ic = 1
x2.ic = 0
# Utilize type 2 rate law for kf and kr parameters defined
model.get_rate_expressions(rate_type=2, update_reactions=True)

# Set a custom rate for b1 to remove substrate concentration dependence
model.add_custom_rate(reaction=b1, custom_rate=b1.kf_str)
```

```
[4]:
```

```
t0 = 0
tf = 150

sim = Simulation(model, verbose=True)
conc_sol, flux_sol = sim.simulate(model, time=(t0, tf),
                                  interpolate=True,
                                  verbose=True)
```

```
WARNING: No compartments found in model. Therefore creating compartment 'compartment' for entire model.
```

```
Successfully loaded MassModel 'Linear_Reversible_Open' into RoadRunner.
Getting time points
Setting output selections
Setting simulation values for 'Linear_Reversible_Open'
Simulating 'Linear_Reversible_Open'
Simulation for 'Linear_Reversible_Open' successful
Adding 'Linear_Reversible_Open' simulation solutions to output
Updating stored solutions
```

```
[5]:
```

```
fig_6_2 = plt.figure(figsize=(13, 5))
gs = fig_6_2.add_gridspec(nrows=1, ncols=2, width_ratios=[1, 1.5])

ax1 = fig_6_2.add_subplot(gs[0, 0])
ax2 = fig_6_2.add_subplot(gs[0, 1])

plot_phase_portrait(
    conc_sol, x=x1, y=x2, ax=ax1,
    xlabel=x1.id, ylabel=x2.id, xlim=(-.05, 1.2), ylim=(-.05, 0.5),
    title=("(a) Phase portrait of x1 vs. x2", {"size": "large"}),
    annotate_time_points=[t0, 1e0, 1e1, tf],
    annotate_time_points_color=["red"],
    annotate_time_points_labels=True);

ax1.annotate(
    'motion towards\n  a qe state',
    xy=(conc_sol[x1.id](0.5), conc_sol[x2.id](0.5)),
    xytext=(conc_sol[x1.id](0.3), conc_sol[x2.id](0.3)));

ax1.annotate(
    'motion of a\n  qe state',
    xy=(conc_sol[x1.id](tf), conc_sol[x2.id](tf)),
    xytext=(conc_sol[x1.id](tf) + 0.05, conc_sol[x2.id](tf) + 0.15));

plot_time_profile(
    conc_sol, ax=ax2, legend="right outside",
    xlabel="Time", ylabel="Concentrations",
    title=("(b) Concentration Profiles", {"size": "large"}));
fig_6_2.tight_layout()
```

**Figure 6.2:** The concentration time profiles for the reaction system \(\rightarrow x\_1 \rightleftharpoons x\_2 \rightarrow\) for \(k\_1 = 1\), \(k\_{-1} = 2\), \(x\_1(0) = 0\), and \(b\_1 = 0.01\). (a) The phase portrait of \(x\_1\) and \(x\_2\) (b) The concentrations as a function of time.

The same pools can be formed as defined in Eq. (4.9):

\[\begin{split}\begin{equation} \begin{pmatrix} {p\_1} \\ {p\_2} \end{pmatrix} = \begin{pmatrix} {1} & {-1/K\_1} \\ {1} & {1} \end{pmatrix} = \begin{pmatrix} {x\_1} \\ {x\_2} \end{pmatrix} \end{equation} \tag{6.12}\end{split}\]

This matrix can be used to post-process the concentrations and the results can be graphed, Figure 6.3. The pool transformation leads to dynamic decoupling and is clearly illustrated in this figure. The disequilibrium pool relaxes very quickly, while the conservation pool moves slowly, Figure 6.3b. The phase portrait formed by the pools thus has an L shape (Figure 6.3a), illustrating the dynamic decoupling. Note that:

- There is first a vertical motion where \(x\_1 + x\_2\) is essentially a constant, followed by a slow horizontal motion where the disequilibrium variable \(x\_1 - x\_2 /K\_1\) is a constant.
- These two separate motions correspond to forming the pathways found in the steady state.
- The disequilibrium aggregate is not zero, as it is forced away from equilibrium by the input and settling in a steady state.

```
[6]:
```

```
# Define pools
pools = ["x1 - x2 / Keq_v1", "x1 + x2"]
for i, equation_str in enumerate(pools):
    pool_id = "p" + str(i + 1)
    conc_sol.make_aggregate_solution(
        pool_id, equation=equation_str,
        parameters={v1.Keq_str: v1.kf/v1.kr}, update=True)

fig_6_3 = plt.figure(figsize=(11, 4))
gs = fig_6_3.add_gridspec(nrows=1, ncols=2, width_ratios=[1, 1.5])

ax1 = fig_6_3.add_subplot(gs[0, 0])
ax2 = fig_6_3.add_subplot(gs[0, 1])

plot_phase_portrait(
    conc_sol, x="p2", y="p1", ax=ax1,
    xlabel="p2 (inventory)", ylabel="p1 (distance from equilibrium)",
    xlim=(-.05, 2.1), ylim=(-.05, 1.05),
    title=("(a) Phase portrait of p2 vs. p1", {"size": "large"}),
    annotate_time_points=[t0, 1e0, 1e1, tf],
    annotate_time_points_color=["red"],
    annotate_time_points_labels=True);

ax1.annotate(
    'motion towards\n  a qe state',
    xy=(conc_sol["p2"](0.5), conc_sol["p1"](0.5)),
    xytext=(conc_sol["p2"](0.3), conc_sol["p1"](0.3)));

ax1.annotate(
    'motion of a\n  qe state',
    xy=(conc_sol["p2"](tf), conc_sol["p1"](tf)),
    xytext=(conc_sol["p2"](tf) + 0.05, conc_sol["p1"](tf) + 0.1));

plot_time_profile(
    conc_sol, observable=["p1", "p2"], ax=ax2, legend="right outside",
    xlabel="Time", ylabel="Concentrations",
    title=("(b) Pool Profiles", {"size": "large"}));
fig_6_3.tight_layout()
```

**Figure 6.3:** The time profiles of the pools involved in reaction system \(\rightarrow x\_1 \rightleftharpoons x\_2 \rightarrow\) for the same conditions as in Figure 6.2. (a) The phase portrait of \(p\_2\) and \(p\_1\). (b) The pools as a function of time.

###### External disturbance:¶

The previous simulation represents a biologically unrealistic situation. An internal concentration cannot suddenly deviate from its value independent of what else happens in the system. A much more realistic situation is one where we start out at a steady state and an environmental change is observed. In our case, the only environmental parameter is \(b\_1\).

In Figure 6.4 we change the input flux, \(b\_1\), from 0.01 to 0.02 at time zero, when the system is initially in a steady state (the endpoint in Figure 6.4). We make three observations:

- We see that the fast motion is not activated.
- The ‘inventory’ or the pool of \(x\_1 + x\_2\) moves from one steady state to another. It increases, as the forcing function was stepped up.
- The ‘distance from equilibrium’

\[\begin{equation} x\_{1, ss} - \frac{x\_{2, ss}}{K\_1} = x\_{1, ss} - \frac{x\_{2, ss}}{x\_{2, eq}/x\_{1, eq}} = x\_{1, ss}(1 - \frac{\Gamma}{K\_1}) \tag{6.13} \end{equation}\]

is close to zero in both steady states. The higher throughput, however, does push the system farther from equilibrium.

```
[7]:
```

```
# Ensure model starts simulation at a steady state.
sim.find_steady_state(model, strategy="simulate", update_values=True, verbose=True);

# Simulate model with disturbance from steady state
conc_sol, flux_sol = sim.simulate(
    model, time=(t0, tf), perturbations={"kf_b1": 0.02},
    interpolate=True, verbose=True)

# Determine pools
for i, equation_str in enumerate(pools):
    pool_id = "p" + str(i + 1)
    conc_sol.make_aggregate_solution(
        pool_id, equation=equation_str,
        parameters={v1.Keq_str: v1.kf/v1.kr}, update=True)
```

```
WARNING: No compartments found in model. Therefore creating compartment 'compartment' for entire model.
```

```
Setting output selections
Setting simulation values for 'Linear_Reversible_Open'
Setting output selections
Getting time points
Simulating 'Linear_Reversible_Open'
Found steady state for 'Linear_Reversible_Open'.
Updating 'Linear_Reversible_Open' values
Adding 'Linear_Reversible_Open' simulation solutions to output
Getting time points
Parsing perturbations
Setting output selections
Setting simulation values for 'Linear_Reversible_Open'
Simulating 'Linear_Reversible_Open'
Simulation for 'Linear_Reversible_Open' successful
Adding 'Linear_Reversible_Open' simulation solutions to output
Updating stored solutions
```

```
[8]:
```

```
fig_6_4 = plt.figure(figsize=(11, 4))
gs = fig_6_4.add_gridspec(nrows=1, ncols=2, width_ratios=[1, 1.5])

ax1 = fig_6_4.add_subplot(gs[0, 0])
ax2 = fig_6_4.add_subplot(gs[0, 1])

plot_phase_portrait(
    conc_sol, x="p2", y="p1", ax=ax1,
    xlabel="p2 (inventory)", ylabel="p1 (distance from equilibrium)",
    xlim=(0, 1), ylim=(0, 0.03),
    title=("(a) Phase portrait of p2 vs. p1", {"size": "large"}),
    annotate_time_points=[t0, 1e0, 1e1, tf],
    annotate_time_points_color=["red"],
    annotate_time_points_labels=True);

plot_time_profile(
    conc_sol,  observable=["p1", "p2"], ax=ax2, legend="right outside",
    xlabel="Time", ylabel="Concentrations",
    title=("(b) Pool Profiles", {"size": "large"}));
fig_6_4.tight_layout()
```

**Figure 6.4:** The time profiles of the pools involved in reaction system \(\rightarrow x\_1 \rightleftharpoons x\_2 \rightarrow\) where the system starts out at equilibrium and the input rate, \(b\_1\), is changed from 0.01 to 0.02 at time zero. (a) The phase portrait of \(p\_2\) and \(p\_1\); (b) The pools as a function of time. Note the small numerical change in \(p\_1\) relative to \(p\_2\). Parameter values as in Figures 6.2 and Figure 6.3.

##### Michaelis-Menten Kinetics in an Open Environment¶

We now consider the case when the Michaelis-Menten reaction mechanism operates in an open environment (Figure 6.5). The substrate enters the system and the product leaves. The enzyme stays internal to the system.

**Figure 6.5:** The Michaelis-Menten reaction mechanisms in an open setting. The substrate and the product enter and leave the system, while the enzyme stays inside.

###### Dynamic description¶

The mass action kinetic model is

\[\begin{split}\begin{align} \frac{ds}{dt} &= b\_1 - k\_1es + k\_{-1}x, \ &s(t=0) &= s\_0 \\ \frac{dx}{dt} &= k\_1es - (k\_{-1} + k\_2)x, \ &x(t=0) &= x\_0 \\ \frac{de}{dt} &= -k\_1es + (k\_{-1} + k\_2)x, \ &e(t=0) &= e\_0 \\ \frac{dp}{dt} &= k\_2x + k\_3p, \ &p(t=0) &= p\_0 \\ \end{align} \tag{6.14}\end{split}\]

The initial conditions would normally be the steady state conditions. The stoichiometric matrix is

\[\begin{split}\begin{equation} \textbf{S} = \begin{pmatrix} {1} & {-1} & {1} & {0} & {0} \\ {0} & {-1} & {1} & {1} & {0} \\ {0} & {1} & {-1} & {-1} & {0} \\ {0} & {0} & {0} & {1} & {-1} \\ \end{pmatrix} \end{equation} \tag{6.15}\end{split}\]

where \(\textbf{x}=(s,\ e, \ x, \ p)\) and \(\textbf{v} = (b\_1, \ k\_1es, \ k\_{-1}x, \ k\_2x, \ k\_3p)\)

###### The steady state¶

As in the previous section, we can compute the steady state fluxes and concentrations. We can also compute how the parameters determine the distance from equilibrium, and here we also run into an additional issue: capacity constraints that result from a conservation quantity.

###### The steady state fluxes:¶

The rank of \(\textbf{S}\) is 3, thus the dimension of the null space is 5-3=2. The null space of \(\textbf{S}\) is spanned by two vectors, (1,1,0,1,1) and (0,1,1,0,0), that correspond to a pathway through the system and an internal reversible reaction. The steady state fluxes are given by

\[\begin{split}\begin{align} \textbf{v}\_{ss} &= (b\_1, \ k\_1e\_{ss}s\_{ss}, \ k\_{-1}x\_{ss}, \ k\_2x\_{ss}, \ k\_3p\_{ss}) \tag{6.16} \\ &= a(1, \ 1, \ 0, \ 1, \ 1) + b(0, 1, 1, 0, 0), \ a \geq 0, \ b \geq 0 \tag{6.17} \\ \end{align}\end{split}\]

###### The steady state concentrations:¶

The dimension of the left null space is 4-3=1. The left null space has one conservation quantity (\(e+x\)), which can readily be seen from the fact that the second and third row of \(\textbf{S}\) add up to zero. The steady state flux balances for this system are

\[\begin{equation} b\_1 = v\_1 - v\_{-1} = v\_2 = v\_3 \tag{6.18} \end{equation}\]

Thus, the incoming flux and the kinetic parameters immediately set the concentrations for \(X\), \(P\), and \(E\) as

\[\begin{equation} x\_{ss} = b\_1/k\_2, \ p\_{ss} = b\_1/k\_3, \ \text{and} \ e\_{ss} = e\_t - x\_{ss} = e\_t - b\_1/k\_2 \tag{6.19} \end{equation}\]

and the steady state substrate concentration can be determined. The steady state concentration of the substrate is given by \(k\_1s\_{ss}e\_{ss} = b\_1 + k\_{-1}x\_{ss}\) that can be solved to give

\[\begin{equation} s\_{ss} = (\frac{k\_2}{k\_1})(\frac{k\_{-1}/k\_2 + 1}{e\_tk\_2/b\_1 - 1}) \tag{6.20} \end{equation}\]

The steady state flux vector can now be computed

\[\begin{split}\begin{align} \textbf{v}\_{ss} &= (b\_1, \ b\_1(k\_{-1}/k\_2 + 1), \ b\_1k\_{-1}/k\_2, \ b\_1, \ b\_1) \tag{6.21} \\ &= a(1, \ 1, \ 0, \ 1, \ 1) + b(0, 1, 1, 0, 0), \ a=b\_1, \ b=b\_1k\_{-1}/k\_2 \tag{6.22} \\ \end{align}\end{split}\]

The distance from equilibrium can now be computed as in the previous section.

###### Internal capacity constraints:¶

Since \(e\_{ss} \geq 0\), the maximum input is

\[\begin{equation} b\_1 \leq b\_{1, max} = k\_2e\_t = v\_m \tag{6.23} \end{equation}\]

which is the maximum reaction rate for the Michaelis-Menten mechanism. The total amount of the enzyme and the turnover rate set this flux constraint.

```
[9]:
```

```
model = MassModel('Michaelis_Menten_Open')
## Define metabolites
s = MassMetabolite("s")
e = MassMetabolite("e")
x = MassMetabolite("x")
p = MassMetabolite("p")

# Define reactions
b1 = MassReaction("b1", reversible=False)
v1 = MassReaction("v1")
v2 = MassReaction("v2", reversible=False)
v3 = MassReaction("v3", reversible=False)

b1.add_metabolites({s: 1})
v1.add_metabolites({s: -1, e: -1, x: 1})
v2.add_metabolites({x: -1, e: 1, p: 1})
v3.add_metabolites({p: -1})
model.add_reactions([b1, v1, v2, v3])

## Define parameters
b1.kf = 0.025
v1.kf, v1.kr = (1, 0.5)
v2.kf = 0.5
v3.kf = 1

# Set initial conditions for model
s.ic = 1
e.ic = 0.05
x.ic = 0.05
p.ic = 0

# Utilize type 2 rate law for kf and kr parameters defined
model.get_rate_expressions(rate_type=2, update_reactions=True)

# Set a custom rate for b1 to remove substrate concentration dependence
model.add_custom_rate(reaction=b1, custom_rate=b1.kf_str)
```

```
[10]:
```

```
t0 = 0
tf = 2e3

sim = Simulation(model, verbose=True)
# Simulate model with disturbance from steady state
conc_sol, flux_sol = sim.simulate(
    model, time=(t0, tf), perturbations={"kf_b1": 0.04},
    interpolate=True, verbose=True)
```

```
WARNING: No compartments found in model. Therefore creating compartment 'compartment' for entire model.
```

```
Successfully loaded MassModel 'Michaelis_Menten_Open' into RoadRunner.
Getting time points
Parsing perturbations
Setting output selections
Setting simulation values for 'Michaelis_Menten_Open'
Simulating 'Michaelis_Menten_Open'
Simulation for 'Michaelis_Menten_Open' successful
Adding 'Michaelis_Menten_Open' simulation solutions to output
Updating stored solutions
```

```
[11]:
```

```
fig_6_6 = plt.figure(figsize=(8, 6))
gs = fig_6_6.add_gridspec(nrows=2, ncols=2, width_ratios=[1, 1.5],
                          height_ratios=[1, 1])

ax1 = fig_6_6.add_subplot(gs[0, 0])
ax2 = fig_6_6.add_subplot(gs[0, 1])
ax3 = fig_6_6.add_subplot(gs[1, 1])

plot_phase_portrait(
    conc_sol, x=s, y=x, ax=ax1,
    xlabel=s.id, ylabel=x.id,
    xlim=(-0.1, 6), ylim=(0, 0.105),
    title=("(a) Phase Portrait of s vs. x", {"size":"large"}),
    annotate_time_points="endpoints",
    annotate_time_points_labels=True)

plot_time_profile(
    conc_sol, observable=x, ax=ax2, legend="best",
    ylim=(0.03, 0.09), xlabel="Time", ylabel="Concentration",
    title=("(b) Time Response of x", {"size": "large"}));

plot_time_profile(
    conc_sol, observable=s, ax=ax3, legend="best",
    ylim=(0, 6), xlabel="Time", ylabel="Concentration",
    title=("(c) Time Response of s", {"size": "large"}));
fig_6_6.tight_layout()
```

**Figure 6.6:** The time profiles for the transient response of the Michaelis-Menten mechanisms for \(b\_1 = 0.025\) changed to 0.04 at time zero. The kinetic parameters are; \(e\_t = 0.1\), \(k\_1 = 1\), \(k\_{-1} = 0.5\), \(k\_2 = 0.5\), \(k\_3 = 0.1\). (a) The phase portrait of \(s\) vs. \(x\). (b) The time response of \(x\). (c) The time response of \(s\).

###### Dynamic states for Michaelis-Menten kinetics¶

The response of this system to a change in the input rate starting from a steady state is of greatest interest, see Figure 6.6. For the kinetic parameters given in the figure, the steady state concentrations of \(s\) and \(x\) are 1.0 and 0.5 respectively. The input rate is changed from 0.025 to 0.04 at time zero and the concentrations of \(s\) and \(x\) go to 4.0 and 0.8, respectively, as time goes to infinity. The maximum flux rate is 0.05.

The change in the input rate triggers an internal motion that basically follows the quasi-steady state line (Figure 6.6c). Figure 6.6b and Figure 6.6c shows that since the internal steps are rapid relative to the exchange rates, there are no rapid transients produced by a perturbation in the input rate.

If the input is increased towards \(v\_m\), the substrate concentration builds up to a very high value and most of the enzyme is found in the intermediate state. If the input rate exceeds \(v\_m\) there will be no steady state as the enzyme cannot convert the substrate to the product at the same rate as it is entering the system.

The dynamic properties of this open system can be analyzed using pool formation.

##### Summary¶

- Open systems eventually reach a steady state, which is different from the equilibrium state of a closed system. Such steady states can be thought of as homeostatic, living states.
- Living cells are open systems that continually exchange mass and energy with their environment. The continual net throughput of mass and concomitant energy dissipation is what allows steady states to form and differentiates them from equilibrium states.
- The relative rates of the internal network to that of the exchanges across the system boundary are important. Time scale separation between internal and exchange processes can form. Rapid internal transients lead to pool formation.
- Open systems are most naturally in a steady state and respond to external stimuli. It is normally not possible to suddenly change the internal state of the system since it is in balance with the environment.
- If the internal dynamics are fast, they do not excite when external stimuli are experienced, and thus accurate information about the fast kinetics may not be needed. It may be enough to know that they are “fast.”
- Non-exchanged moieties form dynamic invariants. They can set internal capacity constraints.

\(\tiny{\text{© B. Ø. Palsson 2011;}\ \text{This publication is in copyright.}\\ \text{Subject to statutory exception and to the provisions of relevant collective licensing agreements,}\\ \text{no reproduction of any part may take place without the written permission of Cambridge University Press.}}\)

#### Orders of Magnitude¶

The simulation examples in the previous chapters are conceptual. As we begin to build simulation models of realistic biological processes, the need to obtain information such as the numerical values of the parameters that appear in the dynamic mass balances. We thus go through a process of estimating the approximate numerical values of various quantities and parameters. Size, mass, chemical composition, metabolic complexity, and genetic makeup represent characteristics for which we now have
extensive data available. Based on typical values for these quantities we show how one can make useful estimates of concentrations and the dynamic features of the intracellular environment.

##### Cellular Composition and Ultra-structure¶

It is often stated that all biologists have two favorite organisms, *E. coli* and another one. Fortunately, much data exists for *E. coli,* and we can go through parameter and variable estimation procedures using it as an example. These estimation procedures can be performed for other target organisms, cell types, and cellular processes in an analogous manner if the appropriate data is available. We organize the discussion around key questions.

###### The interior of a cell¶

The typical bacterial cell, like *E. coli,* is on the order of microns in size (Figure 7.1a). The *E. coli* cell is a short cylinder, about 2-4 micron in length with a 0.5 to 1.5 micron diameter, Figure 7.1b. The size of the *E. coli* cell is growth rate dependent; the faster the cell grows, the larger it is.

It has a complex intra-cellular environment. One can isolate and crystallize macromolecules and obtain their individual structure. However, this approach gives limited information about the configuration and location of a protein in a living functional cell. The intracellular milieu can to be reconstructed from available data to yield an indirect picture of the interior of an cell. Such a reconstruction has been carried out Goodsell93. Based on well known chemical composition data, this image
provides us with about a million-fold magnification of the interior of an *E. coli* cell, see Figure 7.1c. Examination of this picture of the interior of the *E. coli* cell is instructive:

- The intracellular environment is very crowded and represents a dense solution. Protein density in some sub-cellular structures can approach that found in protein crystals, and the intracellular environment is sometimes referred to as ‘soft glass,’ suggesting that it is close to a crystalline state.
- The chemical composition of this dense mixture is very complex. The majority of cellular mass are macromolecules with metabolites, the small molecular weight molecules interspersed among the macromolecules.
- In this crowded solution, the motion of the macromolecules is estimated to be one hundred to even one thousand-fold slower than in a dilute solution. The time it takes a 160 kDa protein to move 10 nm - a distance that corresponds approximately to the size of the protein molecule - is estimated to be 0.2 to 2 milliseconds. Moving one cellular diameter of approximately 0.64 \(\mu m\), or 640 nm, would then require 1-10 min. The motion of metabolites is expected to be significantly faster
  due to their smaller size.

**Figure 7.1:** (a) An electron micrograph of the *E. coli* cell, from Ingraham83. (b) Characteristic features of an *E. coli* cell. (c) The interior of an *E. coli* cell, © David S. Goodsell 1999.

###### The overall chemical composition of a cell¶

With these initial observations, let’s take a closer look at the chemical composition of the cell. Most cells are about 70% water. It is likely that cellular functions and evolutionary design is constrained by the solvent capacity of water, and the fact that most cells are approximately 70% water suggests that all cells are close to these constraints.

The ‘biomass’ is about 30% of cell weight. It is sometimes referred to as the dry weight of a cell, and is denoted by gDW. The 30% of the weight that is biomass is comprised of 26% macromolecules, 1% inorganic ions, and 3% low molecular weight metabolites. The basic chemical makeup of prokaryotic cells is shown in Table 7.1, and contrasted to that of a typical animal cell. The gross chemical composition is similar except that animal cells, and eukaryotic cells in general, have a higher lipid
content because of the membrane requirement for cellular compartmentalization. Approximate cellular composition is available or relatively easy to get for other cell types.

**Table 7.1:** Approximate composition of cells, from (Alberts, 1983). The numbers given are weight percent.

###### The detailed composition of *E. coli*¶

The total weight of a bacterial cell is about 1 picogram. The density of cells barely exceeds that of water, and cellular density is typically around 1.04 to 1.08 \(gm/cm^3\). Since the density of cells is close to unity, a cellular concentration of about \(10^12\) cells per milliliter represents packing density of *E. coli* cells. Detailed and recent data is found later in Figure 7.2.

This information provides the basis for estimating the numerical values for a number of important quantities that relate to dynamic network modeling. Having such order of magnitude information provides a frame of reference, allows one to develop a conceptual model of cells, evaluate the numerical outputs from models, and perform any approximation or simplification that is useful and justified based on the numbers. “Numbers count,” even in biology.

###### Order of magnitude estimates¶

It is relatively easy to estimate the approximate order of magnitude of the numerical values of key quantities. Enrico Fermi the famous physicist was well-known for his skills with such calculations and they thus know as Fermi problems. We give a couple examples to illustrate the order of magnitude estimation process.

###### 1. How many piano tuners are in Chicago?¶

This question represents a classical Fermi problem. First we state assumptions or key numbers:

**1.** There are approximately 5,000,000 people living in Chicago.

**2.** On average, there are two persons in each household in Chicago.

**3.** Roughly one household in twenty has a piano that is tuned regularly.

**4.** Pianos that are tuned regularly are tuned on average about once per year.

**5.** It takes a piano tuner about two hours to tune a piano, including travel time.

**6.** Each piano tuner works eight hours in a day, five days in a week, and 50 weeks in a year.

From these assumptions we can compute:

- that the number of piano tunings in a single year in Chicago is: (5,000,000 persons in Chicago)/(2 persons/household) \(\*\) (1 piano/20 households) \(\*\) (1 piano tuning per piano per year) = 125,000 piano tunings per year in Chicago.
- that the average piano tuner performs (50 weeks/year) \(\*\) (5 days/week) \(\*\) (8 hours/day)/(1 piano tuning per 2 hours per piano tuner) = 1000 piano tunings per year per piano tuner.
- then dividing gives (125,000 piano tuning per year in Chicago) / (1000 piano tunings per year per piano tuner) = 125 piano tuners in Chicago, which is the answer that we sought.

###### 2. How far can a retrovirus diffuse before it falls apart?¶

A similar procedure that relies more on scientific principles can be used to answer this question. The half-live of retroviruses, \(t\_{0.5}\), are measured to be about 5 to 6 hours. The time constant for diffusion is:

\[\begin{equation} t\_{diff} = t^2 / D \tag{7.1} \end{equation}\]

where l is the diffusion distance and \(D\) is the diffusion constant. Then the distance \(l\_{0.5}\) that a virus can travel over a half life is

\[\begin{equation} l\_{0.5} = \sqrt{D\ t\_{0.5}} \tag{7.2} \end{equation}\]

Using a numerical value for \(D\) of \(6.5\*10^{-8}\ cm^2/sec\) that is computed from the Stokes-Einstein equation for a 100 nm particle (approximately the diameter of the retrovirus) the estimate is about 500 \(\mu m\) (Chuck, 1996). A fairly short distance that limits how far a virus can go to infect a target cell.

###### A multi-scale view¶

The cellular composition of cells is complex. More complex yet is the intricate and coordinated web of complex functions that underlie the physiological state of a cell. We can view this as a multi-scale relationship, Figure 7.2. Based on cellular composition and other data we can estimate the overall parameters that are associated with cellular functions. Here we will focus on metabolism, macro-molecular synthesis and overall cellular states.

**Figure 7.2:** A multi-scale view of metabolism, macromolecular synthesis, and cellular functions. Prokaryotic cell (Synechocytis image from W. Vermaas, Arizona State University). Prokaryotic cell structures (purified carboxysomes) image from T. Yates, M. Yeager, and K. Dryden. Macromolecular complexes image © 2000, David S. Goodsell.

##### Metabolism¶

Biomass composition allows the estimation of important overall features of metabolic processes. These quantities are basically concentrations (abundance), rates of change (fluxes), and time constants (response times, sensitivities, etc). For metabolism, we can readily estimate reasonable values for these quantities, and we again organize the discussion around key questions.

###### What are typical concentrations?¶

###### Estimation¶

The approximate number of different metabolites present in a given cell is on the order of 1000 (Feist, 2007). By assuming that metabolite has a median molecular weight of about 312 gm/mol (Figure 7.3a) and that the fraction of metabolites of the wet weight is 0.01, we can estimate a typical metabolite concentration of:

\[\begin{equation} x\_{avg} \approx \frac{1 gm/cm^3 \* 0.01}{1000\*312\ gm/mol} \approx 32 \mu M \tag{7.3} \end{equation}\]

The volume of a bacterial cell is about one cubic micron, or about one femto-liter \((=(10^-15)\) liter). Since a cubic micron is a logical reference volume, we convert the concentration unit as follows:

\[\begin{split}\begin{align} 1 \mu M &= \frac{10^{-6} mole}{1\ L} \* \frac{10^{-15}\ L}{1\ \mu m^3} \* \frac{6 \* 10^{23}\ molecules}{mol} \tag{7.4} \\ &= 600\ molecules/\ \mu m^3 \tag{7.5} \end{align}\end{split}\]

This number is remarkably small. A typical metabolite concentration of \(32 \mu M\) then translates into mere \(19,000\) molecules per cubic micron. One would expect that such low concentrations would lead to slow reaction rates. However, metabolic reaction rates are fairly rapid. As discussed in Chapter 5, cells have evolved highly efficient enzymes to achieve high reaction rates that occur even in the presence of low metabolite concentrations.

**Figure 7.3:** Details of *E. coli* K12 MG1655 composition and properties. (a) The average molecular weight of metabolites is 500 gm/mol and the median is 312 gm/mol. Molecular weight distribution. (b) Thermodynamic properties of the reactions in the iAF1260 reconstruction the metabolic network. (c) Size distribution of ORF lengths or protein sizes: protein size distribution. The average protein length is 316 amino acids, the median is 281. Average molecular weight of *E. coli’s* proteins
(monomers): 34.7 kDa Median: 30.828 kDa (d) distribution of protein concentrations: relative protein abundance distribution. See (Feist, 2007) and (Riley, 2006) for details. Prepared by Vasiliy Portnoy.

###### Measurement¶

Experimentally determined ranges of metabolite concentrations fall around the estimated range; an example is provided in Table 7.2. Surprisingly, glutamate is at a concentration that falls within the 100 millimolar range in *E. coli*. Other important metabolites such as ATP tend to fall in the millimolar range. Intermediates of pathways are often in the micromolar range. Several on-line resources are now available for metabolic concentration data, Table 7.3.

**Table 7.2:** Measured and predicted parameters for *E. coli* growing on minimal media. Taken from (Yuan, 2006).

**Table 7.3:** Publicly available metabolic resources (above the dashed line) and proteomic resources (below the dashed line). Assembled by Vasiliy Portnoy.

###### What are typical metabolic fluxes?¶

###### Rates of diffusion¶

In estimating reaction rates we first need to know if they are diffusion limited. Typical cellular dimensions are on the order of microns, or less. The diffusion constants for metabolites is on the order of \(10^{-5}\ cm^2/sec\) and \(10^{-6}\ cm^2/sec\). These figures translate into diffusional response times that are on the order of:

\[\begin{equation} t\_{diff} = \frac{t^2}{D} = \frac{(10^{-4} cm)^2}{10^{-5}\ \text{to}\ 10^{-6} cm^2 / sec} \approx 1-10 msec \tag{7.6} \end{equation}\]

or faster. The metabolic dynamics of interest are much slower than milliseconds. Although more detail about the cell’s finer spatial structure is becoming increasingly available, it is unlikely, from a dynamic modeling standpoint, that spatial concentration gradients will be a key concern for dynamic modeling of metabolic states in bacterial cells (Weisz, 1973).

###### Estimating maximal reaction rates¶

Reaction rates in cells are limited by the achievable kinetics. Few collections of enzyme kinetic parameters are available in the literature, see Table 7.3. One observation from such collections is that the biomolecular association rate constant, \(k\_1\), for a substrate \((S)\) to an enzyme \((E)\);

\[\begin{equation} S + E \stackrel{k\_1}{\longrightarrow} \tag{7.7} \end{equation}\]

is on the order of \(10^8 M^{-1} sec^{-1}\). This numerical value corresponds to the estimated theoretical limit, due to diffusional constraints (Gutfreund, 1972). The corresponding number for macromolecules is about three orders of magnitude lower.

Using the order of magnitude values for concentrations of metabolites given above and for enzymes in the next section, we find the representative association rate of substrate to enzymes to be on the order of

\[\begin{equation} k\_1 s e = 10^8 (M\*sec)^{-1} \* 10^{-4}M \* 10^{-6}M = 0.01\ M/sec \tag{7.8} \end{equation}\]

that translates into about

\[\begin{equation} k\_1 s e = 10^6\ molecules / \mu m^3 sec \tag{7.9} \end{equation}\]

that is only one million molecules per cubic micron per second. However, the binding of the substrate to the enzyme is typically reversible and a better order of magnitude estimate for *net* reaction rates is obtained by considering the release rate of the product from the substrate-enzyme complex, \(X\). This release step tends to be the slowest step in enzyme catalysis (Albery 1976, Albery 1977, Cleland 1975). Typical values for the release rate constant, \(k\_2\),

\[\begin{equation} X \stackrel{k\_2}{\rightarrow} P + E \tag{7.10} \end{equation}\]

are \(100-1000\ sec^{-1}\). If the concentration of the intermediate substrate-enzyme complex, \(X\), is on the order of 1 \(\mu M\) we get a release rate of about

\[\begin{equation} k\_2x = 10^4\ \text{to}\ 10^5 molecules/\mu m^3 sec \tag{7.11} \end{equation}\]

We can compare the estimate in Eq. (7.9) to observed metabolic fluxes, see Table 7.4. Uptake and secretion rates of major metabolites during bacterial growth represent high flux pathways.

**Table 7.4:** Typical metabolic fluxes measured in *E. coli* K12 MG1655 grown under oxic and anoxic conditions.

###### Measured kinetic constants¶

There are now several accessible sources of information that contain kinetic data for enzymes and the chemical transformation that they catalyze. For kinetic information, both BRENDA and SABIO-RK (Wittig, 2006) are resources of literature curated constants, including rates and saturation levels, Table 7.2.2. Unlike stoichiometric information which is universal, kinetic parameters are highly condition-dependent. *In vitro* kinetic assays typically do not represent in vivo conditions. Factors such
as cofactor binding, pH, and unknown interactions with metabolites and proteins are likely causes.

###### Thermodynamics¶

While computational prediction of enzyme kinetic rates is difficult, obtaining thermodynamic values is more feasible. Estimates of metabolite standard transformed Gibbs energy of formation can be derived using an approach called *group contribution method* (Mavrovouniotis 1991). This method considers a single compound as being made up of smaller structural subgroups. The metabolite standard Gibbs energy of formation associated with structural subgroups commonly found in metabolites are available
in the literature and in the NIST database urlnist. To estimate the metabolite standard Gibbs energy of formation of the entire compound, the contributions from each of the subgroups are summed along with an origin term. The group contribution approach has been used to estimate standard transformed Gibbs energy of formation for 84% of the metabolites in the genome scale model of *E. coli* (Feist 2007, Henry 2006, Henry 2007).

Thermodynamic values can also be obtained by integrating experimentally measured parameters and algorithms which implement sophisticated theory from biophysical chemistry (Alberty 2003, Alberty 2006). Combining this information with the results from group contribution method provides standard transformed Gibbs energy of formation for 96% of the reactions in the genome-scale *E. coli* model (Feist 2007), see Figure 7.3b.

###### What are typical turnover times?¶

As outlined in Chapter 2, turnover times can be estimated by taking that ratio of the concentration relative to the flux of degradation. Both concentrations and fluxes have been estimated above. Some specific examples of estimated turnover times are now provided.

###### Glucose turnover in rapidly growing E. coli cells¶

With an intracellular concentration of glucose of 1 to 5 mM, the estimate of the internal glucose turnover time is

\[\begin{equation} \tau\_{glu} = \frac{6-30\*10^5\ molecules/cell}{4.2 \* 10^5\ \text{to}\ 8.4\*10^5\ molecules/ \mu m^3 /sec} = 1\ \text{to} \ 8\ sec \tag{7.12} \end{equation}\]

###### Response of red cell glycolytic intermediates¶

A typical glycolytic flux in the red cell is about 1.25 mM/hr. By using this number and measured concentrations, we can estimate the turnover times for the intermediates of glycolysis by simply using:

\[\begin{equation} t\_R = \frac{x\_{avg}}{1.25 \ mM/hr} \tag{7.13} \end{equation}\]

The results are shown in Table 7.5. We see the sharp distribution of turnover times that appears. Note that the turnover times are set by the relative concentrations since the flux through the pathway is the same. Thus the least abundant metabolites will have the fastest turnover. At a constant flux, the relative concentrations are set by the kinetic constants.

**Table 7.5:** Turnover times for the glycolytic intermediates in the red blood cell. The glycolytic flux is assumed to be 1.25 mM/hr = 0.35 \(\mu M/sec\) and the Rapoport-Luebering shunt flux is about 0.5 mM/hr. Table adapted from (Joshi, 1990).

###### Response of the energy charge¶

Exchange of high energy bonds between the various carriers is on the order of minutes. The dynamics of this energy pool occur on the middle time scale of minutes as described earlier, see Figure 7.4.

**Figure 7.4:** Responses in energy transduction processes in cells. (a) Effect of addition of glucose on the energy charge of Ehrlich ascites tumor cells. Redrawn based on (Atkinson, 1977). (b) Transient response of the transmembrane gradient, from (Konings, 1983). Generation of a proton motive force in energy starved *S. cremoris* upon addition of lactose (indicated by arrows) at different times after the start of starvation (t=0).

###### Response of transmembrane charge gradients¶

Cells store energy by extruding protons across membranes. The consequence is the formation of an osmotic and charge gradient that results in the so-called *proton motive force,* denoted as \(\Delta \mu\_{H^+}\). It is defined by:

\[\begin{equation} \Delta \mu\_{H^+} = \Delta \Psi - Z\Delta pH \tag{7.14} \end{equation}\]

where \(\Delta \Psi\) is the charge gradient and \(\Delta pH\) is the hydrogen ion gradient. The parameter \(Z\) takes a value of about 60 mV under physiological conditions. The transient response of gradient establishment is very rapid, Figure 7.4.

(Konings, 1983)

###### Conversion between different forms of energy¶

If energy is to be readily exchanged between transmembrane gradients and the high energy phosphate bond system, their displacement from equilibrium should be about the same. Based on typical ATP, ADP, and \(P\_i\) concentrations, one can calculate the transmembrane gradient to be about -180mV. Table 7.6 shows that observed values for the transmembrane gradient, \(\Delta \widetilde{\mu}\), are on the order of -180 to -220 mV. It is interesting to note that the maximum gradient that a
bi-lipid layer can withstand is on the order of -280mV (Konings, 1983), based on electrostatic considerations.

**Table 7.6:** Typical values (mV) for the transmembrane electrochemical potential gradient, reproduced from (Konings, 1983).

###### What are typical power densities?¶

The metabolic rates estimated above come with energy transmission through key cofactors. The highest energy production and dissipation rates are associated with energy transducing membranes. The ATP molecule is considered to be an energy currency of the cell, allowing one to estimate the power density in the cell/organelle based on the ATP production rate.

###### Power density in mitochondria¶

The rate of ATP production in mitochondria can be measured. Since we know the energy in each phosphate bond and the volume of the mitochondria, we can estimate the volumetric rate of energy production in the mitochondria.

Reported rates of ATP production in rat mitochondria from succinate are on the order of \(6\*10^{-19}\) mol ATP/mitochondria/sec  Schwerzmann86, taking place in a volume of about 0.27 \(\mu m^3\). The energy in the phosphate bond about is -52kJ/mol ATP at physiological conditions. These numbers lead to the computation of a per unit volume energy production rate of \(2.2\*10^{-18}\) mol ATP/\(\mu m^3/sec\), or \(10^{-13}W/\mu m^3\ (0.1 pW/\mu m^3)\).

###### Power density in chloroplast of green algae¶

In *Chlamydomonas reinhardtii*, the rate of ATP production of chloroplast varies between \(9.0\*10^{-17}\ \text{to}\ 1.4\*10^{-16}\) mol ATP/chloroplast/sec depending on the light intensity (Baroli 2003, Burns 1990, Melis 2000, Ross 1995) in the volume of 17.4 \(\mu m^3\) (Harris 1989a). Thus, the volumetric energy production rate of chloroplast is on the order of \(5\*10^{-18}\) mol ATP/\(\mu m^3/sec\), or \(3\*10^{-13}W/\mu m^3\ (0.3 pW/\mu m^3)\).

###### Power density in rapidly growing E. coli cells:¶

A similar estimate of energy production rates can be performed for microorganisms. The aerobic glucose consumption of *E. coli* is about 10 mmol/gDW/hr. The weight of a cell is about \(2.8\*10^{-13}\) gDW/cell. The ATP yield on glucose is about 17.5 ATP/glucose. These numbers allow us to compute the energy generation density from the ATP production rate of \(1.4\*10^{-17}\) mol ATP/cell/sec. These numbers lead to the computation of the power density of or
\(7.3\*10^{-13}W/\mu m^3 O\_2\ (0.7 pW/\mu m^3)\)., that is a similar numbers computed for the mitochondria and chloroplast above.

##### Macromolecules¶

We now look at the abundance, concentration and turnover rates of macromolecules in the bacterial cell. We are interested in the genome, RNA and protein molecules.

###### What are typical characteristics of a genome?¶

Sizes of genomes vary significantly amongst different organisms, see Table 7.7. For bacteria, they vary from about 0.5 to 9 million base pairs. The key features of the *E. coli* K-12 MG1655 genome are summarized in Table 7.8. There are about 4500 ORFs on the genome of an average length of about 1kb. This means that the average protein size is 316 amino acids, see Figure 7.3c.

**Table 7.7:** Genome sizes. A selection of representative genome sizes from the rapidly growing list of organisms whose genomes have been sequenced. Adapted from Kimball’s Biology Pages.

**Table 7.8:** Some features of the *E. coli* genome. From (Blattner 1997).

The number of RNA polymerase binding sites is estimated to be about 2800, leading to a estimate of about 1.6 ORFs \((\approx 4.4\*10^6/2400)\) per transcription unit. There are roughly 3000 copies of the RNA polymerase present in an *E. coli* cell (Wagner 2000). Thus if 1000 of the transcription units are active at any given time, there are only 2-3 RNA polymerase molecules available for each transcription unit. The promoters have different binding strength and thus recruit a different number
of RNA polymerase molecules each. ChIP-chip data can be used to estimate this distribution.

###### What are typical protein concentrations?¶

Cells represent a fairly dense solution of protein. One can estimate the concentration ranges for individual enzymes in cells. If we assume that the cell has about 1000 proteins with an average molecular weight of 34.7kD, as is typical for an *E. coli* cell, see Figure 7.3c, and given the fact that the cellular biomass is about 15% protein, we get:

\[\begin{equation} e\_{tot} \approx \frac{1\ gm/cm^3 \* 0.15}{1000 \* 34700 gm/mole} = 4.32 \mu M \tag{7.15} \end{equation}\]

This estimate is, indeed, the range into which the *in vivo* concentration of most proteins fall. It corresponds to about 2500 molecules of a particular protein molecule per cubic micron. As with metabolites there is a significant distribution around the estimate of Eq. (7.15). Important proteins such as the enzymes catalyzing major catabolic reactions tend to be present in higher concentrations, and pathways with smaller fluxes have their enzymes in lower concentrations. It should be noted that
we are not assuming that all these proteins are in solution; the above number should be viewed more as a molar density.

The distribution about this mean is significant, Figure 7.3d. Many of the proteins in *E. coli* are in concentrations as low as a few dozen per cell. The *E. coli* cell is believed to have about 200 major proteins which brings our estimate for the abundant ones to about 12,000 copies per cell. The *E. coli* cell has a capacity to carry about 2.0-2.3 million protein molecules.

###### What are typical fluxes?¶

The rates of synthesis of the major classes of macromolecules in *E. coli* are summarized in Table 7.9. The genome can be replicated in 40 min with two replication forks. This means that the speed of DNA polymerase is estimated to be

\[\begin{equation} \text{rate of DNA polymerase} = \frac{4.4\*10^6\ \text{bp}}{2\*40\*60} \approx 900\ \text{bp/sec/fork} \tag{7.16} \end{equation}\]

RNA polymerase is much slower at 40-50 bp/sec and the ribosomes operate at about 12-21 peptide bonds per ribosome per second.

###### Protein synthesis capacity in *E. coli*¶

We can estimate the number of peptide bonds (pb) produced by *E. coli* per second. To do so we will need the rate of peptide bond formation by the ribosome (12 to 21 bp/ribosome/sec (Bremer 1996, Dennis 1974, Young 1976)); and number of ribosomes present in the *E. coli* cell \((7\*10^3\ \text{to} \ 7\*10^4\) ribosomes/cell depending on the growth rate (Bremer 1996)). So the total number of the peptide bonds that *E. coli* can make per second is on the order of:
\(8\*10^4\ \text{to}\ 1.5\*10^6\) pb/cell/sec

The average size of a protein in *E. coli* is about 320 amino acids. At about 45 to 60 min doubling time, the total amount of protein produced by *E. coli* per second is ~300 to 900 protein molecules/cell/sec. This is equivalent to \(1\ \text{to}\ 3\*10^6\) molecules/cell/h as a function of growth rate, about the total number of protein per cell given above.

###### Maximum protein production rate from a single gene in murine cells¶

The total amount of the protein formed from a single gene in the mammalian cell can be estimated based on the total amount of mRNA present in the cytoplasm from a single gene, the rate of translation of the mRNA molecule by ribosomes, and the ribosomal spacing (Savinell 1989). Additional factors needed include gene dosage (here taken as 1), rate of mRNA degradation, velocity of the RNA polymerase II molecule, and the growth rate (Savinell 1989).

Murine hybridoma cell lines are commonly used for antibody production. For this cell type, the total amount of mRNA from a single antibody encoding gene in the cytoplasm is on the order of 40,000 mRNA molecules/cell (Gilmore 1979, Schibler 1978) the ribosomal synthesis rate is on the order of 20 nucleotides/sec [Potter72], and the ribosomal spacing on the mRNA is between 90-100 nucleotides (Christensen 1987, Potter 1972). Multiplying these numbers, we can estimate the protein production rate in
a hybridoma cell line to be approximately 3000 - 6000 protein molecules/cell/sec.

###### What are the typical turnover times?¶

The assembly of new macromolecules such as RNA and proteins requires the source of nucleotides and amino acids. These building blocks are generated by the degradation of existing RNA molecules and proteins. Many cellular components are constantly degraded and synthesized. This process is commonly characterized by the turnover rates and half-lives. Intracellular protein turnover is experimentally assessed by an addition of an isotope-labeled amino acid mixture to the normally growing or
non-growing cells (Levine 1965, Pratt 2002). It has been shown that the rate of breakdown of an individual proteins is on the order of 2-20% per hour in *E. coli* culture (Levine 1965, Neidhardt 1996).

##### Cell Growth and Phenotypic Functions¶

###### What are typical cell-specific production rates?¶

The estimated rates of metabolism and macromolecular synthesis can be used to compute various cellular functions and their limitations. Such computations can in turn be used for bioengineering design purposes, environmental roles and impacts of microorganisms, and for other purposes. We provide a couple of simple examples.

###### Limits on volumetric productivity¶

*E. coli* is one of the most commonly used host organisms for metabolic engineering and overproduction of metabolites. In many cases, the glycolytic flux acts as a carbon entry point to the pathway for metabolite overproduction (Eiteman 2008, Jantama 2008, Yomano 2008, Zhu 2008). Thus, the substrate uptake rate (SUR) is one of the critical characteristics of the productive capabilities of the engineered cell.

Let us examine the wild-type *E. coli* grown on glucose under anoxic conditions. As shown in Table 7.4, the (SUR) is on the order of 15-20 mmol glucose/gDW/h which translates into 1.5 gm glucose/L/h at cell densities of (VP has number). Theoretically, if all the carbon source (glucose) is converted to the desired metabolite the volumetric productivity will be approximately 3 g/L/h.

The amount of cells present in the culture, play a significant role in production potential. In the industrial settings, the cell density is usually higher which increases the volumetric productivity. Some metabolic engineered strain designs demonstrate higher SUR (Portnoy 2008) that also leads to the increase in volumetric productivity.

###### Photoautotrophic growth¶

*Chlorella vulgaris* is a single-celled green algae that uses light to generate energy necessary for growth. At the top rate of photosynthesis, the specific oxygen production rate (SOPR) can be estimated to be between 20-400 fmol \(O\_2\)/cell/h (Lee 1994a). Algae biotechnology is drawing increasing interest due to its potential for production of biofuels and fine chemicals (Lee 1994a). However, a lack of suitable photobioreactors (PBR) makes the cost of algally-derived compounds high. One of
the key limiting factors for PBR is the light source; however, light-emitting diodes (LED) can be employed for these purposes.

Let us now use order of magnitude calculations to estimate the light requirement for an algae photobioreactor using *C. vulgaris* as a model organism. Given the fact that maximum photosynthetic efficiency of *C. vulgaris* is below 50%  (Kok 1960, Myers 1980, Pirt 1980) and one mole of photons (680 nm) is equivalent to 50W, we can estimate that in order to sustain the SOPR of 100 fmol \(\text{O}\_2\)/cell/h each cell must receive 40pW equivalent of photons (Lee1994a). A conventional LED can
provide \(0.3\ \text{mW/cm}^2\) or \(0.1\ \text{mW}\) per LED. With a cell density close to \(10^9\ \text{cells/ml}\) and \(80\ \text{cm}^3\) volume of the reactor (Lee 1994a), the photobioreactor must include close to a 100 LED to sustain the growth of algae and oxygen production.

###### Balancing the fluxes and composition in an entire cell¶

The approximate calculation procedures presented can be used to estimate the overall flows of mass and energy in a bacterial cell. Proteins are 55% of the dry weight of cells and their most energetically costly component, so let’s begin such computation with the assumption that there are about \(10^9\) amino acids found in the proteins of a single cell. With this starting point and the various data given in this chapter, we can roughly estimate all the major flows using a 60 min doubling
time:

- With approximately 316 amino acids found in a protein, we have to make about 3 million protein molecules.
- If we take the ribosome to make 20 pb/sec = 72,000 pb/hr, (pb=peptide bond) the we require

  \[\begin{equation} \frac{1,000,000,000}{72,000} = 14,000\ \text{ribosomes} \tag{7.17} \end{equation}\]

  to carry out this protein synthesis.
- To make 14,000 ribosomes with each having 4,500 nt length RNA molecules (nt = nucleotide), we need

  \[\begin{equation} 14,000\*4,500 = 63,000,000\ \text{nt}\ \tag{7.18} \end{equation}\]

  assembled. In addition, there are 10 tRNAs of 80 nt in length per ribosome leading to an additional nucleotide requirement of

  \[\begin{equation} 10\*80\*14,000 = 11,200,000\ \text{nt}\ \tag{7.19} \end{equation}\]

  for a grand total of approximately 75,000,000 for stable RNA molecule synthesis.
- The total nucleotide synthesis for RNA will be 3000 RNA polymerase molecules synthesizing at the rate of 50 nt/sec or

  \[\begin{equation} 3000\*50\*3600 = 540,000,000\ \text{nt/hour}\ \tag{7.20} \end{equation}\]
- The fraction of RNA that is mRNA is 0.03 to 0.05 Rosenow01 or,

  \[\begin{equation} 540,000,000\*(0.03\ \text{to}\ 0.05) \approx (16\ \text{to}\ 25.0)\*10^6\ \text{nt/cell/hr}\ \tag{7.21} \end{equation}\]

  If the average mRNA length is 1100 nt then the cell needs to make on average 20,000 transcripts in one hour.
- We have to make 3,000,000 proteins from 20,000 transcripts, or about 150 protein molecules per transcript.
- The transcripts have a finite half live. On average, each transcript has a 5 min lifetime, or 300 sec. Due to structural constraints a ribosome can only bind every 50 nt to the mRNA, producing a maximum ribosomal loading of about 20 ribosomes per transcript. The rate of translation is 20 pb/sec. With the average length of the peptide being 316 amino acids, we can produce 1.25 protein/sec. This calculation estimated the maximum protein production from one transcript on the order of 375 protein
  molecules per transcript.
- To synthesize the genome, we need \(2\*4,500,000 = 9,000,000\ \text{nt}\) to make the double stranded DNA.
- Thus, the total metabolic requirement of amino acids and nucleotides in *E. coli* per doubling is \(1\*10^9\) amino acids/cell/h and \(5\*10^8\) nt/cell/h.

These are the approximate overall material requirements. We also need energy to drive the process. Using Table 7.4 we can estimate energy requirements for *E. coli* under oxic and anoxic conditions.

- Aerobically, at a doubling time of 1 hour, the glucose uptake rate is about 10 mmol/gDW/h, that is equivalent to \(1.5\*10^9\) molecules of glucose per cell per doubling. At 17.5 ATP produced per glucose he corresponding energy production is: \(3\*10^{10}\) ATP per cell per doubling.
- Anaerobically, at a doubling time of 1.5 hours, the glucose uptake rate is about 18 mmol/gDW/h, which is equivalent to \(4.5\*10^9\) molecules of glucose/ cell/doubling. At 3 ATP per glucose the corresponding energy production is \(1.4\*10^{10}\) molecules ATP/cell/doubling.

##### Summary¶

- Data on cellular composition and over all rates are available.
- Order of magnitude estimation procedures exist through which one can obtain the approximate values for key quantities.
- In this fashion, typical concentrations, fluxes and turnover times can be estimated.
- An approximate quantitative overall multi-scale framework can be obtained for the function of complex biological processes.

\(\tiny{\text{© B. Ø. Palsson 2011;}\ \text{This publication is in copyright.}\\ \text{Subject to statutory exception and to the provisions of relevant collective licensing agreements,}\\ \text{no reproduction of any part may take place without the written permission of Cambridge University Press.}}\)

#### Stoichiometric Structure¶

Part I of this book introduced the basics of dynamic simulation. The process for setting up dynamic equations, their simulation, and processing of the output was presented in Chapter 3. Several concepts of dynamic analysis of networks were illustrated through the use of simple examples of chemical reaction mechanisms in Chapters 4 through 6. Most of these examples were conceptual and had limited direct biological relevance. In Chapter 7 we began to estimate the numerical values and ranges for
key quantities in dynamic models. With this background, we now begin the process of addressing issues that are important when one builds realistic dynamic models of biological functions. We start by exploring the consequences of reaction bilinearity and that of the stoichiometric structure of a network. In Part III we then extend the material in this chapter to well-known metabolic pathways.

**MASSpy** will be used to demonstrate some of the topics in this chapter.

```
[1]:
```

```
from mass import (
    MassModel, MassMetabolite, MassReaction,
    Simulation, MassSolution, strip_time)
from mass.util.matrix import nullspace, left_nullspace
from mass.visualization import plot_time_profile, plot_phase_portrait
```

Other useful packages are also imported at this time.

```
[2]:
```

```
import numpy as np
import pandas as pd
import sympy as sym
import matplotlib.pyplot as plt
XL_FONT = {"size": "x-large"}
```

##### Bilinearity in Biochemical Reactions¶

###### Bilinear reactions¶

They are of the form:

\[\begin{equation} x + y \rightarrow z \tag{8.1} \end{equation}\]

Two molecules come together to form a new molecule through the breaking and forming of covalent bonds, or a complex through the formation of hydrogen bonds. As illustrated with the pool formations in the bilinear examples in Chapter 4, such reactions come with moiety exchanges.

###### Enzyme classification¶

Enzyme catalyzed reactions are classified in to seven categories by Enzyme Commission (EC) numbers, see Figure 8.1a. These categories are: oxidoreductases, transferases, hydrolases, lyases, isomerases, and ligases. All these chemical transformations are bilinear with the exception of isomerases that simply rearrange a molecule without the participation of other reactants. Thus, the vast majority of biochemical reactions are bilinear. An overall pseudo-elementary representation (i.e., without
treating the enzyme itself as a reactant, and just representing the un-catalyzed reaction) is bilinear.

**Figure 8.1:** The bilinear nature of biochemical reactions. (a) The classification of enzyme catalyzed reactions into seven categories by the enzyme commission (EC) number system. (b) The detailed view of the role of coenzymes and prosthetic groups in enzyme catalyzed reactions. Coenzymes are often referred to as cofactors. Both images from Koolman, 2005 (reprinted with permission).

###### Coenzymes and prosthetic groups¶

There are coenzymes and prosthetic groups that are involved in many biochemical reactions. These molecules are involved in group transfer reactions as illustrated in Figure 8.1b. They can transfer various chemical moieties or redox equivalents, see Table 8.1. Coenzymes act like a reactant and product in a reaction. They can work with many enzymes performing reactions that need them. Prosthetic groups associate with a particular enzyme to give it chemical functionalities that the protein itself
does not have, Figure 8.1b. The heme group on hemoglobin is perhaps the most familiar example (see Chapter 13) that allows the protein tetramer to acquire a ferrous ion thus enabling the binding of oxygen. This binding allows the red blood cell to perform its oxygen delivery functions. There are many such capabilities ‘grafted’ onto proteins in the form of prosthetic groups. Many of the vitamins confer functions on protein complexes.

##### Bilinearity Leads to a Tangle of Cycles¶

###### Moiety exchange:¶

Biochemical reaction networks are primarily made up of bilinear reactions. A fundamental consequence of this characteristic is a deliberate exchange of chemical moieties and properties between molecules. This exchange is illustrated in Figure 8.2. Here, an incoming molecule, \(XA\), puts the moiety, \(A\), onto a carrier molecule, \(C\). The carrier molecule, now in a ‘charged’ form \((CA)\), can donate the \(A\) moiety to another molecule, \(Y\), to form \(YA\). The
terms *coenzyme,* *cofactor* or *carrier* are used to describe the \(C\) molecule.

**Figure 8.2:** Carrier \((C)\) mediated transfer of chemical moiety \(A\) from compound \(X\) to compound \(Y\).

###### Formation of cycles:¶

The ability of bilinear reactions to exchange moieties in this fashion leads to the formation of distribution networks of chemical moieties and other properties of interest through the formation of a deliberate ‘supply-chain’ network. The structure of such a network must be thermodynamically feasible and conform to environmental constraints.

Bilinearization in biochemical reaction networks leads to a ‘tangle of cycles,’ where different moieties and properties are being moved around the network. While a property of all biochemical networks, this trafficking of chemical and other properties is best known in metabolism. The major chemical properties that are being exchanged in metabolism are summarized in Table 8.1. These properties include energy, redox potential, one-carbon units, two-carbon units, amide groups, amine groups, etc. We
now consider some specific cases.

**Table 8.1:** Some activated carriers or coenzymes in metabolism, modified from Kurganov, 1983.

###### Example: Redox and energy trafficking in the core *E. coli* metabolic pathways¶

Energy metabolism revolves around the generation of redox potential and chemical energy in the form of high-energy phosphate bonds. The degradation of substrates through a series of chemical reactions culminates in the storage of these properties on key carrier molecules; see Table 8.1.

The core metabolic pathways in *E. coli* illustrate this feature, Figure 8.3. The transmission of redox equivalents through this core set of pathways is shown in Figure 8.3a. Each pathway is coupled to a redox carrier in a particular way. This pathway map can be drawn to show the cofactors rather than the primary metabolites and the main pathways (Figure 8.3b). This figure clearly shows how the cofactors interact and how the bilinear property of the stoichiometry of the core set of pathways
leads to a tangle of cycles among the redox carriers.

**Figure 8.3:** The tangle of cycles in trafficking of redox potential (R) in *E. coli* core metabolic pathways. (a) A map organized around the core pathways. (b) The tangle of cycles seen by viewing the cofactors and how they are coupled. Prepared by Jeff Orth.

###### Example: Protein trafficking in signaling pathways¶

Although the considerations above are illustrated using well-known metabolic pathways, these same features are also observed in signaling pathways. Incoming molecules (ligands) trigger a well-defined series of charging and discharging of the protein that make up a signaling network, most often with a phosphate group.

##### Trafficking of High-Energy Phosphate Bonds¶

Given the bilinear nature of biochemical reaction networks and the key role that cofactors play, we begin the process of building biologically meaningful simulation models by studying the use and formation of high-energy phosphate bonds. Cellular energy is stored in high-energy phosphate bonds in ATP. The dynamic balance of the rates of use and formation of ATP is thus a common denominator in all cellular processes, and thus foundational to the living process. We study the dynamic properties of
this system in a bottom-up fashion by starting with its simple elements and making the description progressively more complicated. Throughout the text we make explicit use of the basic methods in **MASSpy**.

**Figure 8.4:** Representation of the exchange of high energy phosphate bonds among the adenosine phosphates. (a) The chemical reactions. (b) The molecules with open circles showing the “vacant” places for high energy bonds. The capacity to carry high-energy phosphate bonds, the occupancy of high-energy bonds, and the energy charge are shown. (c) The reaction schema of (a) in pictorial form. The solid squares represent AMP and the solid circles the high energy phosphate bonds. (d) The same
concepts as in (b) represented in pictorial form.

###### Distribution of high-energy phosphate groups: adenylate kinase (EC 2.7.4.3)¶

The Adenylate Kinase is an important part in intracellular energy homeostasis. Adenylate Kinase is a phosphotransferase enzyme and it is the enzyme responsible for the redistribution of the phosphate groups among the adenosine phosphates. The redistribution reaction the Adenylate Kinase catalyzes is seen in Figure 8.4a.

###### The mass balance: adenylate kinase¶

The redistribution of the phosphate groups among the adenosine phosphates by the adenylate kinase is given by the following kinetic equations:

\[\begin{equation} \frac{d\text{ATP}}{dt} = v\_{\mathrm{distr}}, \ \frac{d\text{ADP}}{dt} = -2\ v\_{\mathrm{distr}}, \ \frac{d\text{AMP}}{dt} = v\_{\mathrm{distr}} \tag{8.2} \end{equation}\]

###### The reaction rates: adenylate kinase¶

The mass action form of these basic reaction rates are

\[\begin{equation} v\_{\mathrm{distr}} = k\_{\mathrm{distr}}^\rightarrow\text{ADP}^2 - k\_{\mathrm{distr}}^\leftarrow\text{ATP}\*\text{AMP} \tag{8.3} \end{equation}\]

###### Numerical values: adenylate kinase¶

The approximate numerical values of the parameters in this system can be estimated. In metabolically active tissues, the ATP concentration is about 1.6 mM, the ADP concentration is about 0.4 mM, and the AMP concentration is about 0.1 mM. Total adenosine phosphates are thus about 2.1 mM. Because this reaction is considerably faster compared to other metabolic processes, we set \(k\_{\mathrm{distr}}^\rightarrow\) to 1000/Min. \(K\_{\mathrm{distr}}\) for the distribution reaction is
approximately unity. We then construct a model of the redistribution of phosphate groups among the adenosine phosphates by adenylate kinase using the above constraints. This is simple reversible reaction that equilibrates quickly.

**Figure 8.5:** The redistribution of phosphate groups among the adenosine phosphates by adenylate kinase.

```
[3]:
```

```
phos_traffic = MassModel("Phosphate_Trafficking", array_type="DataFrame",
                         dtype=np.int64)
# Define metabolites
atp = MassMetabolite("atp")
adp = MassMetabolite("adp")
amp = MassMetabolite("amp")
# Define reactions
v_distr = MassReaction("distr")
v_distr.add_metabolites({adp: -2, amp: 1, atp:1})

# Add reactions to model
phos_traffic.add_reactions([v_distr])

# Define initial conditions and parameters
atp.ic = 1.6
adp.ic = 0.4
amp.ic = 0.1

v_distr.kf = 1000
v_distr.Keq = 1
```

###### Null spaces: adenylate kinase¶

The stoichiometric matrix is basically a column vector.

```
[4]:
```

```
phos_traffic.S
```

```
[4]:
```

|  | distr |
| --- | --- |
| adp | -2 |
| amp | 1 |
| atp | 1 |

It has an empty null space; i.e. zero dimensional.

```
[5]:
```

```
nullspace(phos_traffic.S, rtol=1e-1)
```

```
[5]:
```

```
array([], shape=(1, 0), dtype=float64)
```

However, the left null space has two dimensions and it thus has two conservation pools.

```
[6]:
```

```
# Obtain left nullspace
lns = left_nullspace(phos_traffic.S, rtol=1e-1)
# Iterate through left nullspace,
# dividing by the smallest value in each row.
for i, row in enumerate(lns):
    minval = np.min(abs(row[np.nonzero(row)]))
    new_row = np.array(row/minval)
    # Round to ensure the left nullspace is composed of only integers
    lns[i] = np.array([round(value) for value in new_row])

# Ensure positive stoichiometric coefficients if all are negative
for i, space in enumerate(lns):
    lns[i] = np.negative(space) if all([num <= 0 for num in space]) else space

# Create a pandas.DataFrame to represent the left nullspace
pd.DataFrame(lns, index=["Vacancy", "Occupancy"],
             columns=phos_traffic.metabolites, dtype=np.int64)
```

```
[6]:
```

|  | adp | amp | atp |
| --- | --- | --- | --- |
| Vacancy | 1 | 2 | 0 |
| Occupancy | 1 | 0 | 2 |

The interpretation of these pools is remarkably interesting: the first one counts the number of high energy phosphate bonds in the system, while the second counts the number of vacant spots where high energy phosphate bonds can be added. The left null space is spanned by these two vectors that we can think of as a conjugate pair. Furthermore, the summation of the two is the total amount of the ‘A’ nucleotide in the system times two; ie the total number of possible high-energy phosphate bonds
that the system can carry.

```
[7]:
```

```
# Sum the elements of each row to obtain the capacity pool
capacity = np.array([np.sum(lns, axis=0)])
pd.DataFrame(capacity, index=["Capacity"],
             columns=phos_traffic.metabolites, dtype=np.int64)
```

```
[7]:
```

|  | adp | amp | atp |
| --- | --- | --- | --- |
| Capacity | 2 | 2 | 2 |

Note that any activity of this reaction does not change the sizes of these two pools as the left null space is orthogonal to the reaction vector (or the column vector of \((\textbf{S})\), that represents the direction of motion.

###### Using and generating high-energy phosphate groups¶

We now introduce the ‘use’ and ‘formation’ reactions for ATP into the above system. These represent aggregate processes in the cell using and forming high energy bonds.

###### The mass balances: trafficking high-energy phosphate bonds¶

\[\begin{split}\begin{align} \frac{d\text{ATP}}{dt} &= -v\_{\mathrm{use}} + v\_{\mathrm{form}} + v\_{\mathrm{distr}} \tag{8.4} \\ \frac{d\text{ADP}}{dt} &= v\_{\mathrm{use}} - v\_{\mathrm{form}} - 2\ v\_{\mathrm{distr}} \tag{8.5} \\ \frac{d\text{AMP}}{dt} &= v\_{\mathrm{distr}} \tag{8.6} \end{align}\end{split}\]

where \(v\_{\mathrm{use}}\) is the rate of use of ATP, \(v\_{\mathrm{form}}\) is the rate of formation of ATP, and, as above, \(v\_{\mathrm{distr}}\) is the redistribution of the phosphate group among the adenosine phosphates by adenylate kinase.

###### The reaction rates: trafficking high-energy phosphate bonds¶

Elementary mass action form for the two additional rate equations are

\[\begin{equation} v\_{\mathrm{use}} = k\_{\mathrm{use}}^\rightarrow \text{ATP},\ v\_{\mathrm{form}} = k\_{\mathrm{form}}^\rightarrow\text{ADP}\tag{8.7} \end{equation}\]

###### Numerical values: trafficking high-energy phosphate bonds¶

We use the equilibrium concentrations from the distribution model and estimate in the numerical values for the rate constants of ATP use and formation based on the fact that typical use and formation rates of ATP are about 10 mM/min. Using the steady state concentrations, we can calculate \(k\_{\mathrm{use}}^\rightarrow\) and \(k\_{\mathrm{form}}^\rightarrow\), resulting in \(k\_{\mathrm{use}}^\rightarrow=6.25\ min^{-1}\) and \(k\_{\mathrm{form}}^\rightarrow=25\ min^{-1}\). These
constants are known as Pseudo-Elementary Rate Constants (PERCs). They are a ratio between the flux through a reaction and the concentrations of the involved species, and the simplify the network dynamic analysis. However they are condition dependent and result in a condition dependent kinetic model. What comprises the PERCs is explored further in the later chapters.

We update the distribution model with the additional reactions and parameters.

**Figure 8.6:** The trafficking of high-energy phosphate bonds.

```
[8]:
```

```
# Create utilization reaction
v_use = MassReaction("use", reversible=False)
v_use.add_metabolites({atp: -1, adp: 1})
v_use.kf = 6.25

# Create formation reaction
v_form = MassReaction("form", reversible=False)
v_form.add_metabolites({adp: -1, atp: 1})
v_form.kf = 25

# Add reactions to model
phos_traffic.add_reactions([v_use, v_form])

# View rate of distribution reaction
print(v_distr.rate)
```

```
kf_distr*(adp(t)**2 - amp(t)*atp(t)/Keq_distr)
```

From the model we also see that the net rate for the redistribution of high-energy bonds is

\[\begin{split}\begin{align} v\_{\mathrm{distr}} &= k\_{\mathrm{distr}}^\rightarrow\ \text{ADP}^2 - k\_{\mathrm{distr}}^\leftarrow\text{ATP}\*\text{AMP} \\ &= k\_{\mathrm{distr}}^\rightarrow( \text{ADP}^2 - \text{ATP}\*\text{AMP}/K\_{\mathrm{distr}}) \end{align} \tag{8.8}\end{split}\]

###### Null spaces: trafficking high-energy phosphate bonds¶

Now the stoichiometric matrix three columns.

```
[9]:
```

```
phos_traffic.S
```

```
[9]:
```

|  | distr | use | form |
| --- | --- | --- | --- |
| adp | -2 | 1 | -1 |
| amp | 1 | 0 | 0 |
| atp | 1 | -1 | 1 |

It has a one-dimensional null space, that represents an internal loop as the use and formation reactions are the exact opposites of each other.

```
[10]:
```

```
# Obtain nullspace
ns = nullspace(phos_traffic.S, rtol=1e-1)
# Transpose and iterate through nullspace,
# dividing by the smallest value in each row.
ns = ns.T
for i, row in enumerate(ns):
    minval = np.min(abs(row[np.nonzero(row)]))
    new_row = np.array(row/minval)
    # Round to ensure the nullspace is composed of only integers
    ns[i] = np.array([round(value) for value in new_row])


# Ensure positive stoichiometric coefficients if all are negative
for i, space in enumerate(ns):
    ns[i] = np.negative(space) if all([num <= 0 for num in space]) else space

# Revert transpose
ns = ns.T
# Create a pandas.DataFrame to represent the nullspace
pd.DataFrame(ns, index=[rxn.id for rxn in phos_traffic.reactions],
             columns=["Path 1"], dtype=np.int64)
```

```
[10]:
```

|  | Path 1 |
| --- | --- |
| distr | 0 |
| use | 1 |
| form | 1 |

The left null space is now one-dimensional;

```
[11]:
```

```
# Obtain left nullspace
lns = left_nullspace(phos_traffic.S, rtol=1e-1)
# Iterate through left nullspace,
# dividing by the smallest value in each row.
for i, row in enumerate(lns):
    minval = np.min(abs(row[np.nonzero(row)]))
    new_row = np.array(row/minval)
    # Round to ensure the left nullspace is composed of only integers
    lns[i] = np.array([round(value) for value in new_row])

# Ensure positive stoichiometric coefficients if all are negative
for i, space in enumerate(lns):
    lns[i] = np.negative(space) if all([num <= 0 for num in space]) else space

# Create a pandas.DataFrame to represent the left nullspace
pd.DataFrame(lns, index=["Total AxP"],
             columns=phos_traffic.metabolites, dtype=np.int64)
```

```
[11]:
```

|  | adp | amp | atp |
| --- | --- | --- | --- |
| Total AxP | 1 | 1 | 1 |

###### Dynamic simulations: trafficking high-energy phosphate bonds¶

The system is steady at the initial conditions given

```
[12]:
```

```
t0, tf = (0, 1e3)
sim = Simulation(phos_traffic, verbose=True)
conc_sol, flux_sol = sim.simulate(
    phos_traffic, time=(t0, tf, tf*10 + 1), interpolate=True,
    verbose=True)
```

```
WARNING: No compartments found in model. Therefore creating compartment 'compartment' for entire model.
```

```
Successfully loaded MassModel 'Phosphate_Trafficking' into RoadRunner.
Getting time points
Setting output selections
Setting simulation values for 'Phosphate_Trafficking'
Simulating 'Phosphate_Trafficking'
Simulation for 'Phosphate_Trafficking' successful
Adding 'Phosphate_Trafficking' simulation solutions to output
Updating stored solutions
```

```
[13]:
```

```
fig, axes = plt.subplots(nrows=2, ncols=1, figsize=(8, 4),
                         )
(ax1, ax2) = axes.flatten()

plot_time_profile(
    conc_sol, ax=ax1,
    legend="right outside",plot_function="semilogx",
    xlabel="Time [min]", ylabel="Concentrations [mM]",
    title=("Concentration Profile", XL_FONT));

plot_time_profile(
    flux_sol, ax=ax2,
    legend="right outside", plot_function="semilogx",
    xlabel="Time [min]", ylabel="Fluxes [mM/min]",
    title=("Flux Profile", XL_FONT));
```

```
[13]:
```

```
<matplotlib.axes._subplots.AxesSubplot at 0x7faf1bbed2d0>
```

We can induce motion in the system by taking 0.2 mM of ADP and splitting it into 0.1 mM addition to AMP and ATP, and set the initial conditions as ATP is 1.7 mM, ADP is 0.2 mM, and AMP is 0.2 mM and simulate the dynamic response. We graph the concentration profiles, as well as the two pools and the disequilibrium variable: \(\text{ADP}^2 - \text{ATP}\*\text{AMP}\) that is zero at the equilibrium

```
[14]:
```

```
# Define pools and perturbations
pools = {"Occupancy": "adp + 2*atp",
         "Vacancy": "adp + 2*amp",
         "Disequilibrium": "adp**2 - atp*amp"}

# Simulate with disturbance
conc_sol, flux_sol = sim.simulate(
    phos_traffic, time=(t0, tf, tf*10 + 1),
    perturbations={"atp": 1.7, "adp": 0.2, "amp": 0.2})

# Determine pools
for pool_id, equation_str in pools.items():
    conc_sol.make_aggregate_solution(
        pool_id, equation=equation_str, update=True)

# Visualize solutions
fig_8_7, axes = plt.subplots(nrows=3, ncols=1, figsize=(8, 6),)
(ax1, ax2, ax3) = axes.flatten()

plot_time_profile(
    conc_sol, ax=ax1, observable=phos_traffic.metabolites,
    legend="right outside", plot_function="semilogx", ylim=(0, 1.8),
    xlabel="Time [min]", ylabel="Concentration [mM]",
    title=("(a) Concentration Profile", XL_FONT));

plot_time_profile(
    conc_sol, observable=["Occupancy", "Vacancy"], ax=ax2,
    legend="right outside", plot_function="semilogx", ylim=(0., 4.),
    xlabel="Time [min]", ylabel="Concentration [mM]",
    title=("(b) Occupancy and Vacancy Pools", XL_FONT));

plot_time_profile(
    conc_sol, observable=["Disequilibrium"], ax=ax3,
    legend="right outside", plot_function="semilogx", ylim=(-.4, 0.1),
    xlabel="Time [min]", ylabel="Concentration [mM]",
    title=("(c) Disequilibrium Variable", XL_FONT));
```

```
[14]:
```

```
<matplotlib.axes._subplots.AxesSubplot at 0x7faf1c1a5090>
```

**Figure 8.7:** The time response of the adenylate kinase reaction (“distr”) and with the addition of ATP use and formation to a change in the initial conditions. (a) The concentrations. (b) The occupancy and capacity pools. (c) The disequilibrium variable.

###### Towards a realistic simulation of a dynamic response¶

Next, we simulate the response of this system to a more realistic perturbation: a 50% increase in the rate of ATP use. This would represent a sudden increase in energy use by a cell. At time zero, we have the network in a steady state and we change \(k\_{\mathrm{use}}^\rightarrow\) from \(6.25/min\) to \(1.5\*6.25=9.375/min\), and the rate of ATP use instantly becomes 15 mM/min.

The response of the system is perhaps best visualized by showing the phase portrait of the rate of ATP use versus ATP formation. Prior to the increased load, the system is on the 45 degree line, where the rate of ATP formation and use balances. Then at time zero it is instantly imbalanced by changing \(k\_{\mathrm{use}}^\rightarrow\) above or below its initial value. If \(k\_{\mathrm{use}}^\rightarrow\) is increased then the initial point moved into the region where more ATP is used than
formed. From this initial perturbation the response of the system is to move directly towards the 45 degree line to regain balance between ATP use and formation.

```
[15]:
```

```
t0, tf = (0, 1e3)
# Simulate with disturbance
conc_sol, flux_sol = sim.simulate(
    phos_traffic, time=(t0, tf, tf*10 + 1),
    perturbations={"kf_use": "kf_use * 1.5"},
    verbose=True)

# Determine pools
for pool_id, equation_str in pools.items():
    conc_sol.make_aggregate_solution(
        pool_id, equation=equation_str, update=True)
```

```
Getting time points
Parsing perturbations
Setting output selections
Setting simulation values for 'Phosphate_Trafficking'
Simulating 'Phosphate_Trafficking'
Simulation for 'Phosphate_Trafficking' successful
Adding 'Phosphate_Trafficking' simulation solutions to output
Updating stored solutions
```

```
[16]:
```

```
fig_8_8 = plt.figure(figsize=(15, 5))
gs = fig_8_8.add_gridspec(nrows=3, ncols=2, width_ratios=[1, 1.5])

ax1 = fig_8_8.add_subplot(gs[:, 0])
ax2 = fig_8_8.add_subplot(gs[0, 1])
ax3 = fig_8_8.add_subplot(gs[1, 1])
ax4 = fig_8_8.add_subplot(gs[2, 1])

label = "{0} [mM/min]"
plot_phase_portrait(
    flux_sol, x=v_use, y=v_form, ax=ax1,
    time_vector=np.linspace(t0, 1, int(1e4)),
    xlabel=label.format(v_use.id), ylabel=label.format(v_form.id),
    xlim=(4, 21), ylim=(4, 21),
    title=("(a) Phase Portrait of ATP use vs. formation", XL_FONT),
    annotate_time_points="endpoints",
    annotate_time_points_labels=True);

line_data = [i for i in range(0, 22)]
ax1.plot(line_data, line_data, ls="--", color="black")
ax1.annotate("use < form", xy=(6, 15))
ax1.annotate("use > form", xy=(15, 6))
ax1.annotate("Steady-state line:\n     use=form", xy=(15, 19))
ax1.annotate("initial perturbation", xy=(9.5, 9), xycoords="data")
ax1.annotate("", xy=(flux_sol[v_use.id][0], flux_sol[v_form.id][0]),
             xytext=(10, 10), textcoords="data",
             arrowprops=dict(arrowstyle="->",connectionstyle="arc3"))

plot_time_profile(
    conc_sol, observable=phos_traffic.metabolites,
    ax=ax2, legend="right outside",
    time_vector=np.linspace(t0, 1, int(1e5)),
    xlim=(t0, 1), ylim=(0, 2),
    xlabel="Time [min]", ylabel="Concentration [mM]",
    title=("(b) Concentration Profiles", XL_FONT));

plot_time_profile(
    flux_sol, observable=[v_use],
    ax=ax3, legend="right outside",
    time_vector=np.linspace(t0, 1, int(1e5)),
    xlim=(t0, 1), ylim=(12, 16),
    xlabel="Time [min]", ylabel="Flux [mM/min]",
    title=("(c) Net ATP use", XL_FONT));

plot_time_profile(
    conc_sol, observable="Disequilibrium",
    ax=ax4, legend="right outside",
    time_vector=np.linspace(t0, 1, int(1e5)), plot_function="semilogx",
    xlabel="Time [min]", ylabel="Concentration [mM]",
    xlim=(1e-6, 1), ylim=(-.0001, 0.0015),
    title=("(d) Disequilibrium", XL_FONT));
fig_8_8.tight_layout()
```

**Figure 8.8:** Dynamic responses for Eqs (8.4 - 8.8). (a) The phase portrait for the rates of use and formation of ATP. (b) The concentrations of ATP, ADP, and AMP. (c) Net ATP use (d) The disequilibrium variable for Adenylate kinase.

###### Pooling and interpretation: trafficking high-energy phosphate bonds¶

Since AMP is not being synthesized and degraded, the sum of \(\text{ATP} + \text{ADP} +\text{AMP}\), or the capacity to carry high-energy phosphate bonds, is a constant. The Atkinson’s energy charge

\[\begin{equation} \text{E.C.} = \frac{2\ \text{ATP} + \text{ADP}}{2\ \text{ATP}+\text{ADP}+\text{AMP}} = \frac{\text{occupancy}}{\text{capacity}} \tag{8.9} \end{equation}\]

shows a monotonic decay to a lower state in response to the increased load (see Figure 8.9).

```
[17]:
```

```
pools.update({"EC": "(2*atp + adp) / (2*(atp + adp + amp))"})
# Determine pools
for pool_id, equation_str in pools.items():
    conc_sol.make_aggregate_solution(
        pool_id, equation=equation_str, update=True)
```

```
[18]:
```

```
fig_8_9, axes = plt.subplots(nrows=1, ncols=2, figsize=(12, 4))
(ax1, ax2) = axes.flatten()

plot_time_profile(
    conc_sol, observable=["EC"], ax=ax1, legend="best",
    plot_function="semilogx", ylim= (.7, 1),
    xlabel="Time [min]", ylabel="Concentration [mM]",
    title=("(a) Energy Charge", XL_FONT));

plot_time_profile(
    conc_sol, observable=["Occupancy", "Vacancy"], ax=ax2,
    legend="right outside", plot_function="semilogx",
    ylim=(0., 4.), xlabel="Time [min]", ylabel="Concentration [mM]",
    title=("(b) Charge Pools", XL_FONT));
fig_8_9.tight_layout()
```

**Figure 8.9:** (a) The Atkinson’s energy charge (Eq. 8.9). (b) The occupancy and vacancy pools move in the opposite directions. Increasing the load drops the occupancy pool and increases the vacancy pool as the system becomes discharged. Reduced loads have the opposite reaction.

**Figure 8.10:** Graphical representation of the energy charge (x-direction) versus the capacity (y-direction). The drop in the charge is indicated by the arrow. The capacity is a constant in this case.

###### Buffering the energy charge¶

###### Reaction mechanism: E.C. buffering¶

In many situations, there is a buffering effect on the energy charge by a coupled carrier of high energy bonds. This exchange is:

\[\begin{equation} \text{ATP}\ + \text{B} \leftrightharpoons \text{ADP}\ + \text{BP} \tag{8.10} \end{equation}\]

where the buffering molecule, \(\text{B}\), picks up the high-energy phosphate group through a fast equilibrating reaction.

**Figure 8.11:** The trafficking of high-energy phosphate bonds with the buffer molecule exchange reaction.

```
[19]:
```

```
# Copy the model to create a new, yet identical model instance
phos_buffered = phos_traffic.copy()
phos_buffered.id += "_Buffered"

# Create the buffer metabolites
b = MassMetabolite("b")
bp = MassMetabolite("bp")

# Create the buffer reaction and add the metaolites
v_buff = MassReaction("buff")
v_buff.add_metabolites({atp:-1, b:-1, adp:1, bp:1})

# Update model
phos_buffered.add_reactions(v_buff)
```

The rate equation of the buffering reaction is:

```
[20]:
```

```
print(strip_time(phos_buffered.rates[v_buff]))
```

```
kf_buff*(atp*b - adp*bp/Keq_buff)
```

###### Examples of buffer molecules¶

In Eq. (8.10), \(\text{B}\) represents a phosphagen, which is a compound containing a high-energy phosphate bond that is used as energy storage to buffer the ATP/ADP ratio. The most well-known phosphagen is creatine, which is found in the muscles of mammals. Marine organisms have other phosphagens (arginine, taurocyamine, glycocyamine), while earthworms use lombricine (Nguyen, 1960).

###### Buffering:¶

When the reaction in Eq. 8.10 is at equilibrium we have

\[\begin{equation} k\_{\mathrm{buff}}^\rightarrow\text{ATP}\*\text{B} = k\_{\mathrm{buff}}^\leftarrow \text{ADP}\*\text{BP} \tag{8.11} \end{equation}\]

This equation can be rearranged as

\[\begin{equation} 4 K\_{\mathrm{buff}} = \text{BP}/\text{B} \tag{8.12} \end{equation}\]

where \(\text{ATP}/\text{ADP}=1.6/0.4=4\) in the steady state, and \(K\_{\mathrm{buff}} = k\_{\mathrm{buff}}/k\_{-buff}\). If the buffering molecule is present in a constant amount, then

\[\begin{equation} \text{B}\_{\mathrm{tot}} = \text{B} + \text{BP} \tag{8.13} \end{equation}\]

We can rearrange equations (8.12) and (8.13) as:

\[\begin{equation} \frac{\text{BP}}{\text{B}\_{\mathrm{tot}}} = \frac{4 K\_{\mathrm{buff}}}{4 K\_{\mathrm{buff}} + 1} \tag{8.14} \end{equation}\]

In this equation, \(\text{B}\_{\mathrm{tot}}\) is the capacity of the buffer to carry the high energy phosphate bond whereas \(\text{BP}/\text{B}\_{\mathrm{tot}}\) is the energy charge of the buffer.

We note that the value of \(K\_{\mathrm{buff}}\) is a key variable. If \(K\_{\mathrm{buff}} = 1/4\) then the buffer is half charged at equilibrium, whereas if \(K\_{\mathrm{buff}}=1\) then the buffer is 80% charged. Thus, this numerical value (a thermodynamic quantity) is key and will specify the relative charge on the buffer and the adenosine phosphates. The effect of \(K\_{\mathrm{buff}}\) can be determined through simulation.

###### Updating the model with the buffering reaction¶

It is assumed that the buffering reaction is at equilibrium and that the amount of buffering molecules is constant:

```
[21]:
```

```
# Use sympy to set up a symbolic equation for the buffer equilibrium
buff_equilibrium = sym.Eq(
    sym.S.Zero, strip_time(phos_buffered.rates[v_buff]))

# Set amount of buffer molecules
btot = 10

# Use sympy to set up a symbolic equation for the buffer pool
b_sym = sym.Symbol(b.id)
bp_sym = sym.Symbol(bp.id)
buff_pool = sym.Eq(b_sym + bp_sym, btot)

# Pretty print the equations
sym.pprint(buff_equilibrium)
sym.pprint(buff_pool)
```

```
            ⎛         adp⋅bp ⎞
0 = kf_buff⋅⎜atp⋅b - ────────⎟
            ⎝        Keq_buff⎠
b + bp = 10
```

Solve the equilibrium system:

```
[22]:
```

```
# Obtain a dict of ic values for substitution into the sympy expressions
ic_dict = {sym.Symbol(met.id): ic
          for met, ic in phos_buffered.initial_conditions.items()}
# Substitute known concentrations
buff_equilibrium = buff_equilibrium.subs(ic_dict)

# Obtain solutions for B and BP
buff_sol = sym.solve([buff_equilibrium, buff_pool], [b_sym, bp_sym])
# Pretty print the equation
print(buff_sol)
```

```
{bp: 40.0*Keq_buff/(4.0*Keq_buff + 1.0), b: 10.0/(4.0*Keq_buff + 1.0)}
```

Set \(K\_{\mathrm{buff}}\) and \(k\_{\mathrm{buff}}^\rightarrow\):

```
[23]:
```

```
v_buff.kf = 1000
v_buff.Keq = 1

# Obtain a dict of parameter values for substitution into the sympy expressions
param_dict = {
    sym.Symbol(parameter): value
    for parameter, value in v_buff.parameters.items()}

buffer_ics = {
    phos_buffered.metabolites.get_by_id(str(met)): float(expr.subs(param_dict))
    for met, expr in buff_sol.items()}

# Update initial conditions with buffer molecule concentrations
phos_buffered.update_initial_conditions(buffer_ics)
for met, ic in phos_buffered.initial_conditions.items():
    print("{0}: {1} mM".format(met, ic))
```

```
adp: 0.4 mM
amp: 0.1 mM
atp: 1.6 mM
b: 2.0 mM
bp: 8.0 mM
```

###### Null spaces: E.C. buffering¶

With the addition of the buffer, stoichiometric matrix four columns.

```
[24]:
```

```
phos_buffered.S
```

```
[24]:
```

|  | distr | use | form | buff |
| --- | --- | --- | --- | --- |
| adp | -2 | 1 | -1 | 1 |
| amp | 1 | 0 | 0 | 0 |
| atp | 1 | -1 | 1 | -1 |
| b | 0 | 0 | 0 | -1 |
| bp | 0 | 0 | 0 | 1 |

It has still has a one-dimensional null space, that represents and internal loop as the use and formation reactions are the exact opposites of each other.

```
[25]:
```

```
# Obtain nullspace
ns = nullspace(phos_buffered.S, rtol=1e-1)
# Transpose and iterate through nullspace,
# dividing by the smallest value in each row.
ns = ns.T
for i, row in enumerate(ns):
    minval = np.min(abs(row[np.nonzero(row)]))
    new_row = np.array(row/minval)
    # Round to ensure the nullspace is composed of only integers
    ns[i] = np.array([round(value) for value in new_row])

# Ensure positive stoichiometric coefficients if all are negative
for i, space in enumerate(ns):
    ns[i] = np.negative(space) if all([num <= 0 for num in space]) else space

# Revert transpose
ns = ns.T
# Create a pandas.DataFrame to represent the nullspace
pd.DataFrame(ns, index=[rxn.id for rxn in phos_buffered.reactions],
             columns=["Path 1"], dtype=np.int64)
```

```
[25]:
```

|  | Path 1 |
| --- | --- |
| distr | 0 |
| use | 1 |
| form | 1 |
| buff | 0 |

The left null space is two-dimensional. It represents conservation of the nucleotide and the buffer molecule. Neither AxP or B is produced or destroyed in the model;

```
[26]:
```

```
# Obtain left nullspace
lns = left_nullspace(phos_buffered.S, rtol=1e-1)
# Iterate through left nullspace,
# dividing by the smallest value in each row.
for i, row in enumerate(lns):
    minval = np.min(abs(row[np.nonzero(row)]))
    new_row = np.array(row/minval)
    # Round to ensure the left nullspace is composed of only integers
    lns[i] = np.array([round(value) for value in new_row])

# Ensure positive stoichiometric coefficients if all are negative
for i, space in enumerate(lns):
    lns[i] = np.negative(space) if all([num <= 0 for num in space]) else space

# Create a pandas.DataFrame to represent the left nullspace
pd.DataFrame(lns, index=["Total AxP", "Total B"],
             columns=phos_buffered.metabolites, dtype=np.int64)
```

```
[26]:
```

|  | adp | amp | atp | b | bp |
| --- | --- | --- | --- | --- | --- |
| Total AxP | 1 | 1 | 1 | 0 | 0 |
| Total B | 0 | 0 | 0 | 1 | 1 |

###### Dynamic simulation: E.C. buffering¶

The model is initially in steady state.

```
[27]:
```

```
t0, tf = (0, 1e3)
sim = Simulation(phos_buffered, verbose=True)
conc_sol, flux_sol = sim.simulate(phos_buffered, time=(t0, tf, tf*10 + 1),
                                  verbose=True)
```

```
WARNING: No compartments found in model. Therefore creating compartment 'compartment' for entire model.
```

```
Successfully loaded MassModel 'Phosphate_Trafficking_Buffered' into RoadRunner.
Getting time points
Setting output selections
Setting simulation values for 'Phosphate_Trafficking_Buffered'
Simulating 'Phosphate_Trafficking_Buffered'
Simulation for 'Phosphate_Trafficking_Buffered' successful
Adding 'Phosphate_Trafficking_Buffered' simulation solutions to output
Updating stored solutions
```

```
[28]:
```

```
fig, axes = plt.subplots(nrows=2, ncols=1, figsize=(8, 4),
                         )
(ax1, ax2) = axes.flatten()

plot_time_profile(
    conc_sol, ax=ax1, legend="right outside",
    plot_function="semilogx",
    xlabel="Time [min]", ylabel="Concentrations [mM]",
    title=("Concentration Profile", XL_FONT));

plot_time_profile(
    flux_sol, ax=ax2, legend="right outside",
    plot_function="semilogx",
    xlabel="Time [min]", ylabel="Fluxes [mM/min]",
    title=("Flux Profile", XL_FONT));
```

```
[28]:
```

```
<matplotlib.axes._subplots.AxesSubplot at 0x7faeff049990>
```

We can compare the flux dynamics of the buffered vs. unbuffered system. The buffered system has a much longer response time. Once again, we consider a simulation where we increase the ATP use rate by a ‘multiplier’ in this figure:

```
[29]:
```

```
fig, ax = plt.subplots(nrows=1, ncols=1, figsize=(8, 4),
                       )

buff_strs = ["unbuffered", "buffered"]
linestyles = ["--", "-"]

t0, tf = (0, 1e3)
# Simulate both models with the disturbance
for i, model in enumerate([phos_traffic, phos_buffered]):
    sim = Simulation(model)
    conc_sol, flux_sol = sim.simulate(
        model, time=(t0, tf, tf*10 + 1),
        perturbations={"kf_use": "kf_use * 1.5"})

    plot_time_profile(
        flux_sol, observable=["use", "form"], ax=ax,
        legend=(["use " + buff_strs[i], "form " + buff_strs[i]],
                "right outside"),
        plot_function="semilogx",
        xlabel="Time [min]", ylabel="Fluxes [mM/min]",
        color=["red", "blue"], linestyle=linestyles[i])
```

```
WARNING: No compartments found in model. Therefore creating compartment 'compartment' for entire model.
WARNING: No compartments found in model. Therefore creating compartment 'compartment' for entire model.
```

**Figure 8.12:** The fluxes of ATP use and formation respond more slowly when the ATP buffer is present.

The response of the adenosine phosphate system can be simulated in the presence of a buffer. We choose the parameters as \(\text{B}\_{\mathrm{tot}}=10\ mM\), \(K\_{\mathrm{buff}}=1\), and \(k\_{\mathrm{buff}}=1000/min\) and all other conditions as in Figure 8.8. The results of the simulation are shown in Figure 8.13. The time response of the energy charge is shown, along with the buffer charge \(\text{BP}/\text{B}\_{\mathrm{tot}}\). We see that the fast response in the energy charge
is now slower as the initial reaction is buffered by release of the high energy bonds that are bound to the buffer. The overall change in the energy charge is the same: it goes from 0.86 to 0.78. The charge of the buffer drops from 0.80 to 0.73 at the same time.

**Figure 8.13:** Pictorial representation of the phosphate exchange among the adenosine phosphates and a buffering molecule. (a) The reaction schema. (b) A pictorial representation of the molecules, their charged states, and the definition of pooled variables\*

###### Pooling and interpretation: E.C. buffering¶

A pictorial representation of the phosphate buffering is given in Figure 8.13. Here, a generalized definition of the overall phosphate charge is:

\[\begin{equation} \text{overall charge} = \frac{\text{overall occupancy}}{\text{overall capacity}} = \frac{2\ \text{ATP}+\text{ADP}+\text{BP}}{2\ (\text{ATP}+\text{ADP}+\text{AMP})+\text{BP} + \text{B}} \tag{8.15} \end{equation}\]

This combined charge system can be represented similarly to the representation in Figure 8.10. Figure 8.14 shows a stacking of the buffer and adenosine phosphate capacity versus their charge. The total capacity to carry high-energy bonds is now 14.2 mM. The overall charge is 0.82 (or 11.64 mM concentration of high-energy bonds) in the system before the perturbation. The increased load brings the overall charge down to 0.74.

**Figure 8.14:** The representation of the energy and buffer charge versus the capacity (in mM on y-axis). The lumping of the two quantities into ‘overall’ quantities is illustrated. The case considered corresponds to the simulation in Figure 8.15.

To understand this effect, we first define more pools:

```
[30]:
```

```
pools.update({
    "BC": "bp / (bp + b)",
    "Overall_Charge": "(2*atp + adp + bp) / (2*(atp + adp + amp) + bp + b)"})
```

and then plot the dynamic responses of the pools:

```
[31]:
```

```
fig_8_15, axes = plt.subplots(nrows=2, ncols=1, figsize=(8, 6),)
(ax1, ax2) = axes.flatten()
legend_labels = ["E.C. Unbuffered", "E.C. Buffered"]
for i, model in enumerate([phos_traffic, phos_buffered]):
    sim = Simulation(model)
    conc_sol, flux_sol = sim.simulate(
        model, time=(t0, tf, tf*10 + 1),
        perturbations={"kf_use": "kf_use * 1.5"})

    # Determine pools
    for pool_id, equation_str in pools.items():
        # Skip buffered charge for model with no buffer
        if i == 0 and pool_id in ["BC", "Overall_Charge"]:
            continue
        conc_sol.make_aggregate_solution(
            pool_id, equation=equation_str, update=True)

    if i == 1:
        # Plot the charge pools for the buffered solution
        plot_time_profile(
            conc_sol, observable=["EC", "BC", "Overall_Charge"], ax=ax1,
            legend=(["E.C.", "B.C.", "Overall Charge"], "right outside"),
            xlabel="Time [min]", ylabel="Charge",
            xlim=(t0, 1), ylim=(.7, .9),
            title=("(a) Charge Pools of Buffered Model", XL_FONT));

    # Compare the buffered and unbuffered solutions
    plot_time_profile(
        conc_sol, observable=["EC"], ax=ax2,
        legend=(legend_labels[i], "right outside"),
        xlabel="Time [min]", ylabel="Charge",
        xlim=(t0, 1), ylim=(.7, .9),
        title=("(b) E.C. Unbuffered Vs Buffered", XL_FONT));
fig_8_15.tight_layout()
```

```
WARNING: No compartments found in model. Therefore creating compartment 'compartment' for entire model.
WARNING: No compartments found in model. Therefore creating compartment 'compartment' for entire model.
```

**Figure 8.15:** Dynamic responses for Eqs.(8.4 - 8.8) with the buffering effect (Eq. (8.10)). (a) The Atkinson’s energy charge (Eq. (8.9)) the buffer charge (Eq. (8.14)), and the overall charge (Eq. (8.15) are shown as a function of time. (b) Comparison of the buffered and unbuffered energy charge. \(B\_{\mathrm{tot}}=10 mM\), \(K\_{\mathrm{buff}}=1\) and \(k\_{\mathrm{buff}}=1000\). All other conditions are as in Figure 8.8; i.e., we simulate the response to a ‘multiplier’ increase in
\(k\_{\mathrm{use}}\). Note the slower response of the E.C. in panel (b) when the system is buffered.

###### Open system: long term adjustment of the capacity¶

###### Inputs and outputs:¶

Although the rates of formation and degradation of AMP are low, their effects can be significant. These fluxes will determine the total amount of the adenosine phosphates and thus their capacity to carry high energy bonds. The additional elementary rate laws needed to account for the rate of AMP formation and drain are:

\[\begin{equation} v\_{\mathrm{form,\ AMP}} = b\_{1}, \ v\_{\mathrm{drain}} = k\_{\mathrm{drain}} \* \text{AMP} \tag{8.16} \end{equation}\]

where \(b\_1\) is the net synthesis rate of AMP. The numerical values used are \(b\_{1}=0.03\ mM/min\) and \(k\_{\mathrm{drain}} = (0.03\ mM/min)/(0.1\ mM) = 0.3\ mM/min\).

###### Updating the model for long term capacity adjustment¶

Define the AMP exchange reaction:

**Figure 8.16:** The trafficking of high-energy phosphate bonds with the buffer molecule and AMP exchange reactions.

```
[32]:
```

```
# Copy the model to create a new, yet identical model instance
phos_open = phos_buffered.copy()
phos_open.id += "_Open"

# Get MassMetabolite amp assoicated with the new copied model
amp = phos_open.metabolites.amp

# Define AMP formation
b1 = MassReaction("b1", reversible=False)
b1.add_metabolites({amp:1})
b1.kf = 0.03

# Define AMP drain
drain = MassReaction("drain", reversible=False)
drain.add_metabolites({amp:-1})
drain.kf = 0.3
# Add reactions to the model
phos_open.add_reactions([b1, drain])
# Set custom rate for formation of AMP
phos_open.add_custom_rate(b1, custom_rate=b1.kf_str)

# Display the net rate for AMP synthesis and draining
rate = strip_time(phos_open.rates[b1] - phos_open.rates[drain])
print(rate)
# Substitute values to check if steady state
print(rate.subs({
    sym.Symbol('amp'): amp.ic, # AMP concentration at steady state
    sym.Symbol('kf_drain'): drain.kf, # forward rate constant for drain reaction
    sym.Symbol('kf_b1'): b1.kf})) # Synthesis rate
```

```
-amp*kf_drain + kf_b1
0
```

With the specified parameters and initial conditions, the system is in a steady state, i.e. no net exchange of AMP.

###### Null spaces: long term capacity adjustment¶

With the addition of the AMP exchanges, stoichiometric matrix six columns.

```
[33]:
```

```
phos_open.S
```

```
[33]:
```

|  | distr | use | form | buff | b1 | drain |
| --- | --- | --- | --- | --- | --- | --- |
| adp | -2 | 1 | -1 | 1 | 0 | 0 |
| amp | 1 | 0 | 0 | 0 | 1 | -1 |
| atp | 1 | -1 | 1 | -1 | 0 | 0 |
| b | 0 | 0 | 0 | -1 | 0 | 0 |
| bp | 0 | 0 | 0 | 1 | 0 | 0 |

It has still has a two-dimensional null space, that 1) represents and internal loop as the use and formation reactions are the exact opposites of each other, as before, and 2) an exchange pathways of AMP coming into the system and leaving the system.

```
[34]:
```

```
# Obtain nullspace
ns = nullspace(phos_open.S, rtol=1e-1)
# Transpose and iterate through nullspace,
# dividing by the smallest value in each row.
ns = ns.T
for i, row in enumerate(ns):
    minval = np.min(abs(row[np.nonzero(row)]))
    new_row = np.array(row/minval)
    # Round to ensure the nullspace is composed of only integers
    ns[i] = np.array([round(value) for value in new_row])

# Ensure positive stoichiometric coefficients if all are negative
for i, space in enumerate(ns):
    ns[i] = np.negative(space) if all([num <= 0 for num in space]) else space

# Revert transpose
ns = ns.T
# Create a pandas.DataFrame to represent the nullspace
pd.DataFrame(ns, index=[r.id for r in phos_open.reactions],
             columns=["Path 1", "Path 2"], dtype=np.int64)
```

```
[34]:
```

|  | Path 1 | Path 2 |
| --- | --- | --- |
| distr | 0 | 0 |
| use | 1 | 0 |
| form | 1 | 0 |
| buff | 0 | 0 |
| b1 | 0 | 1 |
| drain | 0 | 1 |

The left null space becomes one-dimensional. The total amount of A is no longer conserved as AMP can now enter or leave the system, i.e. pathway 2) can have a net flux. The buffer molecule, B, on the other hand is always contained within the system

```
[35]:
```

```
# Obtain left nullspace
lns = left_nullspace(phos_open.S, rtol=1e-1)
# Iterate through left nullspace,
# dividing by the smallest value in each row.
for i, row in enumerate(lns):
    minval = np.min(abs(row[np.nonzero(row)]))
    new_row = np.array(row/minval)
    # Round to ensure the left nullspace is composed of only integers
    lns[i] = np.array([round(value) for value in new_row])

# Ensure positive stoichiometric coefficients if all are negative
for i, space in enumerate(lns):
    lns[i] = np.negative(space) if all([num <= 0 for num in space]) else space

# Create a pandas.DataFrame to represent the left nullspace
pd.DataFrame(lns, index=["Total B"],
             columns=phos_open.metabolites, dtype=np.int64)
```

```
[35]:
```

|  | adp | amp | atp | b | bp |
| --- | --- | --- | --- | --- | --- |
| Total B | 0 | 0 | 0 | 1 | 1 |

###### Dynamic simulations: long term capacity adjustment¶

Initially, the open system is in a steady-state. Once again, we consider a simulation where we increase the ATP use rate by a ‘multiplier’. This system has a bi-phasic response for the values of the kinetic constants. We can start the system in a steady state at \(t=0^-\) and simulate the response for increasing the ATP load by shifting the value of \(k\_{\mathrm{use}}^\rightarrow\) by a ‘multiplier’ at \(t=0\), as before. The initial rapid response is similar to what is shown in
Figure 8.8a, where the concentration of ATP drops in response to the load and the concentrations of ADP and AMP rise. This initial response is followed by a much slower response where all three concentrations drop.

```
[36]:
```

```
t0, tf = (0, 1e3)
sim = Simulation(phos_open, verbose=True)
sim.find_steady_state(models=phos_open, strategy="simulate")
conc_sol, flux_sol = sim.simulate(
    phos_open, time=(t0, tf, tf*10 + 1),
    perturbations={"kf_use": "kf_use * 1.5"})
pools.update({"Capacity": "2*(atp + adp + amp)"})
# Determine pools
for pool_id, equation_str in pools.items():
    # Skip buffered charge for model with no buffer
    conc_sol.make_aggregate_solution(
        pool_id, equation=equation_str, update=True)
```

```
WARNING: No compartments found in model. Therefore creating compartment 'compartment' for entire model.
```

```
Successfully loaded MassModel 'Phosphate_Trafficking_Buffered_Open' into RoadRunner.
```

```
[37]:
```

```
fig, axes = plt.subplots(nrows=2, ncols=1, figsize=(8, 6),
                         )
(ax1, ax2) = axes.flatten()
plot_time_profile(
    conc_sol, ax=ax1, observable=phos_open.metabolites,
    legend="right outside",
    plot_function="semilogx",
    xlabel="Time [min]", ylabel="Concentrations [mM]",
    title=("Concentration Profile", XL_FONT));

plot_time_profile(
    flux_sol, ax=ax2, observable=phos_open.reactions,
    legend="right outside",
    plot_function="semilogx",
    xlabel="Time [min]", ylabel="Fluxes [mM/min]",
    title=("Flux Profile", XL_FONT));
fig.tight_layout()
```

###### Interpretation of the bi-phasic response¶

This bi-phasic response can be examined further by looking at dynamic phase portraits of key fluxes (Figure 8.17) and key pools (Figure 8.18).

```
[38]:
```

```
fig_8_17, axes = plt.subplots(nrows=1, ncols=2, figsize=(10, 5))
(ax1, ax2) = axes.flatten()

label = "{0} [mM/min]"
plot_phase_portrait(
    flux_sol, x="use", y="form", ax=ax1,
    xlim=(4, 21), ylim=(4, 21),
    xlabel=label.format("use"), ylabel=label.format("form"),
    title=("(a) Phase Portrait of ATP use vs. formation", XL_FONT),
    annotate_time_points=[0, 1e-1, 1e0, 25, 150],
    annotate_time_points_color=["red"],
    annotate_time_points_labels=True);

# Annotate plot
line_data = [i for i in range(0, 22)]
ax1.plot(line_data, line_data, ls="--", color="black");
ax1.annotate("use < form", xy=(6, 15));
ax1.annotate("use > form", xy=(15, 6));
ax1.annotate("Steady-state line:\n     use=form", xy=(15, 19));
ax1.annotate("initial perturbation", xy=(9.5, 9), xycoords="data");
ax1.annotate("", xy=(flux_sol["use"][0], flux_sol["form"][0]),
             xytext=(10, 10), textcoords="data",
             arrowprops=dict(arrowstyle="->",connectionstyle="arc3"));

plot_phase_portrait(
    flux_sol, x="use", y="drain", ax=ax2,
    xlim=(0, 21), ylim=(0, 0.1),
    xlabel=label.format("use"), ylabel=label.format("drain"),
    title=("(b) Phase Portrait of use vs. drain", XL_FONT),
    annotate_time_points=[0, 1e-1, 1e0, 25, 150],
    annotate_time_points_color=["red"],
    annotate_time_points_labels=True);

# Annotate plot
ax2.plot(line_data, [0.03]*22, ls="--", color="black");
ax2.annotate("net AMP\ngain", xy=(1.5, 0.02));
ax2.annotate("net AMP\ndrain", xy=(1.5, 0.04));
fig_8_17.tight_layout()
```

**Figure 8.17:** Dynamic phase portraits of fluxes for the simulation of the adenosine phosphate system with formation and drain of AMP (Eq. (8.16)). (a) the ATP use \((v\_{\mathrm{use}})\) versus the ATP formation rate \((v\_{\mathrm{form}})\). (b) the ATP use \((v\_{\mathrm{use}})\) versus the AMP drain \((v\_{\mathrm{drain}})\).

```
[39]:
```

```
fig_8_18 = plt.figure(figsize=(12, 4))
gs = fig_8_18.add_gridspec(nrows=1, ncols=2, width_ratios=[1, 1.5])

ax1 = fig_8_18.add_subplot(gs[0, 0])
ax2 = fig_8_18.add_subplot(gs[0, 1])

plot_phase_portrait(
    conc_sol, x="Occupancy", y="Capacity", ax=ax1,
    time_vector=np.linspace(t0, 10, int(1e6)),
    xlim=(2.7, 4.3), ylim=(2.7, 4.3),
    xlabel="Occupancy", ylabel="Capacity",
    title=("(a) Occupancy vs. Capacity", XL_FONT),
    annotate_time_points="endpoints",
    annotate_time_points_labels=True);

plot_time_profile(
    conc_sol, observable=["EC", "BC", "Overall_Charge"], ax=ax2,
    legend=(["E.C.", "B.C.", "Overall Charge"], "right outside"),
    time_vector=np.linspace(t0, 10, int(1e6)),
    xlabel="Time [min]", ylabel="Charge",
    xlim=(t0, 10), ylim=(0.65, 1),
    title=("(b) Charge Responses", XL_FONT));
fig_8_18.tight_layout()
```

**Figure 8.18:** The Energy Charge response. (a) Dynamic phase portrait of 2ATP+ADP versus 2(ATP+ADP+AMP). (b) The response of E.C., B.C., and overall charge.

- First, we examine how the system balances the use of ATP \((v\_{\mathrm{use}})\) with its rate of formation \((v\_{\mathrm{form}})\), see Figure 8.17. At \(t=0\) the system is at rest at \(v\_{\mathrm{use}}=v\_{\mathrm{form}}=10.0\ mM/min\). Then the system is perturbed by moving the ATP drain, \(v\_{\mathrm{use}}\), to 15.0 mM/min, as before. The initial response is to increase the formation rate of ATP to about 13 mM/min with the simultaneous drop in the use rate to about the
  same number, due to a net drop in the concentration of ATP during this period. The rate of ATP use and formation is approximately the same at this point in time. Then, during the slower response time, the use and formation rates of ATP are similar and the system moves along the 45 degree line to a new steady state point at 6.67 mM/min.
- The slow dynamics are associated with the inventory of the adenosine phosphates (ATP + ADP + AMP). The AMP drain can be graphed versus the ATP use, see Figure 8.17b. Initially, the AMP drain increases rapidly as the increased ATP use leads to ADP buildup that gets converted into AMP by adenylate kinase \((v\_{\mathrm{distr}})\). The AMP drain then drops and sets at the same rate to balance the formation rate, set at 0.03 mM/min.
- We can graph the occupancy against the capacity (Figure 8.18a). During the initial response, the occupancy moves while the capacity is a constant. Then, during the slower phase, the two move at a constant ratio. This gives a bi-phasic response of the energy charge (Figure 8.18b). In about a minute, the energy charge changes from 0.86 to about 0.77 and then stays a constant. The energy charge is roughly a constant even though all the other concentrations are changing.

This feature of keeping the energy charge a constant while the capacity is changing has a role in a variety of physiological responses, from blood storage to the ischemic response in the heart. Note that this property is a stoichiometric one; no regulation is required to produce this effect.

##### Charging Substrates and Recovery of High-Energy Bonds¶

###### Reaction mechanism:¶

As discussed in Section 8.2, most catabolic pathways generate energy (and other metabolic resources) in the form of activated (or charged) carrier molecules. Before energy can be extracted from a compound, it is typically activated by the use of metabolic resources (a biological equivalent of “it takes money to make money”). This basic structure shown in Figure 2.5 is redrawn in Figure 8.19a where one ATP molecule is used to ‘charge’ a substrate \((x\_1)\) with one high-energy bond to form an
intermediate \((x\_2)\). This intermediate is then degraded through a process wherein two ATP molecules are synthesized and an inorganic phosphate is incorporated. The net gain of ATP is 1 for every \((x\_2)\) metabolized, and this ATP molecule can then be used to drive a process \(v\_{\mathrm{load}}\) that uses an ATP molecule. The trafficking of high-energy phosphate bonds is shown pictorially in Figure 8.19b.

**Figure 8.19:** Coupling of the adenosine phosphates with a skeleton metabolic pathway. (a) The reaction map. (b) A pictorial view of the molecules emphasizing the exchange of the high-energy phosphate group (solid circle). The blue square is AMP. The rate laws used are: \(b\_1 = 0.03\ mM/min.\); \(b\_2 = 5\ mM/min.\); \(k\_{\mathrm{drain}}=b\_1/0.1\); \(k\_{\mathrm{load}}=5/1.6\); \(k\_1=5/0.4\). The flux of \(b\_2\) was set to 5 mM/min, as the ATP production rate is double
that number, thus the steady state value for ATP production is 10 mM/min, to match what is discussed in section 8.3.

```
[40]:
```

```
# Create model
phos_recovery = MassModel("Phosphate_Recovery", array_type="dense",
                          dtype=np.int64)
# Define metabolites
atp = MassMetabolite("atp")
adp = MassMetabolite("adp")
amp = MassMetabolite("amp")
pi = MassMetabolite("pi")
x1 = MassMetabolite("x1")
x2 = MassMetabolite("x2")
x3 = MassMetabolite("x3")
# Define reactions
b1 = MassReaction("b1", reversible=False)
b1.add_metabolites({amp:1})

distr = MassReaction("distr")
distr.add_metabolites({adp: -2, amp: 1, atp:1})

load = MassReaction("load", reversible=False)
load.add_metabolites({atp: -1, adp: 1, pi: 1})

drain = MassReaction("drain", reversible=False)
drain.add_metabolites({amp:-1})

b2 = MassReaction("b2", reversible=False)
b2.add_metabolites({x1: 1})

v1 = MassReaction("v1", reversible=False)
v1.add_metabolites({atp: -1, x1: -1, adp: 1, x2: 1})

v2 = MassReaction("v2", reversible=False)
v2.add_metabolites({adp: -2, pi: -1, x2: -1, atp: 2, x3: 1})

DM_x3 = MassReaction("DM_x3", reversible=False)
DM_x3.add_metabolites({x3: -1})

# Add reactions to model
phos_recovery.add_reactions([b1, distr, load, drain, b2, v1, v2, DM_x3])

# Define initial conditions and parameters
atp.ic = 1.6
adp.ic = 0.4
amp.ic = 0.1
pi.ic = 2.5
x1.ic = 1
x2.ic = 1
x3.ic = 1

b1.kf = 0.03
distr.kf = 1000
distr.Keq = 1
load.kf = 5/1.6
drain.kf = 0.3
b2.kf = 5
v1.kf = 5/1.6
v2.kf = 5/0.4
DM_x3.kf = 5

# Set custom rate for source reactions
phos_recovery.add_custom_rate(b1, custom_rate=b1.kf_str)
phos_recovery.add_custom_rate(b2, custom_rate=b2.kf_str)
```

###### The dynamic mass balances:¶

The dynamic mass balance equations that describe this process are:

\[\begin{split}\begin{align} \frac{dx\_1}{dt} &= b\_2 - v\_1 \\ \frac{dx\_2}{dt} &= v\_1 - v\_2 \\ \frac{d\text{ATP}}{dt} &= -(v\_1 + v\_{\mathrm{load}}) + 2v\_2 + v\_{\mathrm{distr}} \\ \frac{d\text{ADP}}{dt} &= (v\_1 + v\_{\mathrm{load}}) - 2v\_2 - 2v\_{\mathrm{distr}} \\ \frac{d\text{AMP}}{dt} &= b\_1 - v\_{\mathrm{drain}} + v\_{\mathrm{distr}} \\ \end{align} \tag{8.17}\end{split}\]

To integrate the reaction schema in Figure 8.13a with this skeleton pathway, we have replaced the use rate of ATP \((v\_{\mathrm{use}})\) with \(v\_1 + v\_{\mathrm{load}}\) and the formation rate of ATP \((v\_{\mathrm{form}})\) with \(2v\_2\).

###### Dynamic simulation:¶

The flow of substrate into the cell, given by \(b\_2\), will be set to 5 mM/min in the simulation to follow to set the gross ATP production at 10 mM/min. The response of this system can be simulated to a change in the ATP load parameter, as in previous examples. The difference from the previous examples here is that the net ATP production rate is 5 mM/min.

The time response of the concentrations and fluxes are shown in Figure 8.20, the flux phase portraits in Figure 8.21, and the pools and ratios in Figure 8.22.

```
[41]:
```

```
t0, tf = (0, 100)
sim = Simulation(phos_recovery, verbose=True)
sim.find_steady_state(models=phos_recovery, strategy="simulate",
                      update_values=True)
conc_sol, flux_sol = sim.simulate(
    phos_recovery, time=(t0, tf, tf*10 + 1),
    perturbations={"kf_load": "kf_load * 1.5"},
    interpolate=True)

pools = {"Occupancy": "adp + 2*atp",
         "Capacity": "2*(atp + adp + amp)",
         "EC": "(2*atp + adp) / (2*(atp + adp + amp))"}

for pool_id, equation_str in pools.items():
    conc_sol.make_aggregate_solution(
        pool_id, equation=equation_str, update=True)

netfluxes = {
    "load_total": "v1 + load",
    "generation": "2*v2",
    "drain_total": "drain"}
for flux_id, equation_str in netfluxes.items():
    # Skip buffered charge for model with no buffer
    flux_sol.make_aggregate_solution(
        flux_id, equation=equation_str, update=True)
```

```
WARNING: No compartments found in model. Therefore creating compartment 'compartment' for entire model.
WARNING: No compartments found in model. Therefore creating compartment 'compartment' for entire model.
ERROR: Something unexpected occurred and the model could not be loaded into the current RoadRunner instance. Therefore initializing a new RoadRunner instance for the Simulation.
```

```
Successfully loaded MassModel 'Phosphate_Recovery' into RoadRunner.
```

```
[42]:
```

```
fig_8_20, axes = plt.subplots(nrows=3, ncols=1, figsize=(8, 6))
(ax1, ax2, ax3) = axes.flatten()

plot_time_profile(
    conc_sol, observable=phos_recovery.metabolites,
    ax=ax1, legend="right outside",
    xlim=(t0, 25), ylim=(0, 2.0),
    xlabel="Time [min]", ylabel="Concentration [mM]",
    title=("(a) Concentrations", XL_FONT));

plot_time_profile(
    flux_sol, observable=["v1", "v2", "load"],
    ax=ax2, legend="right outside",
    xlim=(t0, 25), ylim=(4, 8),
    xlabel="Time [min]", ylabel="Fluxes [mM/min]",
    title=("(b) High-Flux Reactions", XL_FONT));

plot_time_profile(
    flux_sol, observable=["distr", "drain"],
    ax=ax3, legend="right outside",
    xlim=(t0, 25), ylim=(0, .4),
    xlabel="Time [min]", ylabel="Fluxes [mM/min]",
    title=("(c) Low-Flux Reactions", XL_FONT));
fig_8_20.tight_layout()
```

**Figure 8.20:** The response of the systems shown in Figure 8.19 to a 50% increase in the ATP load rate constant. (a) Dynamic response of the concentrations on a fast and slow time scale. (b) Dynamic response of the main fluxes on a fast and slow time scale. (c) Dynamic response of the AMP determining fluxes on a fast and slow time scale. Parameter values are the same as in Figure 8.19.

```
[43]:
```

```
fig_8_21, axes = plt.subplots(nrows=1, ncols=2, figsize=(10, 5))
(ax1, ax2) = axes.flatten()

plot_phase_portrait(
    flux_sol, x="load_total", y="generation", ax=ax1,
    xlabel="ATP load total", ylabel="ATP Synthesis",
    xlim=(9, 13.5), ylim=(9, 13.5),
    title=("(a) ATP Load vs. Synthesis", XL_FONT),
    annotate_time_points="endpoints",
    annotate_time_points_labels=True);

# Annotate plot
line_data = [i for i in range(8, 15)]
ax1.plot(line_data, line_data, ls="--", color="black");
ax1.annotate(
    "", xy=(flux_sol["load_total"](0), flux_sol["generation"](0)),
    xytext=(10, 10), textcoords="data",
    arrowprops=dict(arrowstyle="->",connectionstyle="arc3"));
ax1.annotate("initial perturbation", xy=(
    flux_sol["load_total"](0) - 1.7,
    flux_sol["generation"](0) - 0.2));

plot_phase_portrait(
    flux_sol, x="load_total", y="drain_total", ax=ax2,
    xlabel="ATP load total", ylabel="AMP drain",
    xlim=(8, 13.5), ylim=(0, 0.125),
    title=("(a) ATP Load vs. Drain", XL_FONT),
    annotate_time_points="endpoints",
    annotate_time_points_labels=True);

ax2.plot(line_data, [0.03] * 7, ls="--", color="black");
fig_8_21.tight_layout()
```

**Figure 8.21:** The response of the system shown in Figure 8.19 to a change in the ATP load rate constant. (a) ATP load versus ATP synthesis rate. (b) ATP load versus AMP drainage rate. You can compare this response to Figure 8.17.

```
[44]:
```

```
fig_8_22 = plt.figure(figsize=(10, 4))
gs = fig_8_22.add_gridspec(nrows=1, ncols=2, width_ratios=[1, 1.5])

ax1 = fig_8_22.add_subplot(gs[0, 0])
ax2 = fig_8_22.add_subplot(gs[0, 1])

plot_phase_portrait(
    conc_sol, x="Occupancy", y="Capacity", ax=ax1,
    xlim=(2.3, 4.4), ylim=(2.3, 4.4),
    xlabel="Occupancy", ylabel="Capacity",
    title=("(a) Occupancy vs. Capacity", XL_FONT),
    annotate_time_points=[t0, 1e0, 50],
    annotate_time_points_color=["red"],
    annotate_time_points_labels=True);
# Annotate plot
ax1.annotate(" fast\nmotion\n", xy=(conc_sol["Occupancy"](0.3) - .25,
                                   conc_sol["Capacity"](0.3) - .35))

plot_time_profile(
    conc_sol, observable=["EC"], ax=ax2, legend="best",
    xlim=(t0, 50), ylim=(0.65, 1),
    xlabel="Time [min]", ylabel="Energy Charge",
    title=("(b) Stoichiometric Disturbance Rejection Property", XL_FONT));
fig_8_22.tight_layout()
```

**Figure 8.22:** The response of the system shown in Figure 8.19 to a change in the ATP load rate constant. (a) Dynamic phase portrait of the pools 2ATP+ADP versus 2(ATP+ADP+AMP). (b) Energy charge ratio as a function of time. You can compare this response to Figure 8.18.

###### Interpretation:¶

We can make the following observations from this dynamic response:

- The concentrations move on two principal time scales (Figure 8.20): a fast time scale that is about three to five minutes, and a slower time scale that is about 50 min. ATP and \(x\_1\) move primarily on the fast time scale, whereas ADP, AMP, and \(x\_2\) move on the slower time scale. You can see this clearly by changing time in Figure 8.20.
- Initially \(v\_{\mathrm{load}}\) increases sharply, and \(v\_2\) increases and \(v\_1\) decreases to meet the increased load. The three high flux reactions \(v\_1\), \(v\_2\), and \(v\_{\mathrm{load}}\) restabilize at about 5 mM/min after about a three to five minute time frame, after which they are closely, but not fully, balanced (Figure 8.20).
- The dynamic phase portrait, Figure 8.21a, shows that the overall ATP use \((v\_1 + v\_{\mathrm{load}})\) quickly moves to about 12.5 mM/min while the production rate \((2v\_2)\) is about 10 mM/min. Following this initial response, the ATP use drops and the ATP synthesis rate increases to move towards the 45 degree line. The 45 degree line is not reached. After 0.1 min, \(v\_2\) starts to drop and the system moves somewhat parallel to the 45 degree line until 1.5 min have passed. At
  this time the ATP concentration has dropped to about 1.06 mM, which makes the ATP use and production rate approximately balanced. Following this point, both the use and production rate increase slowly and return the system back to the initial point where both have a value of 10 mM/min. Since the input rate of \(x\_1\) is a constant, the system has to return to the initial state.
- AMP initially increases leading to a net drain of AMP from the system. This drain unfolds on a long time scale leading to a net flux through the adenylate kinase that decays on the slower time scale. The effects of AMP drainage can be seen in the flux phase portrait in Figure 8.21b. Initially the AMP drain increases as the ATP usage drops close to its eventual steady state. Then the vertical motion in the phase portrait shows that there is a slower motion in which the ATP usage does not
  change much but the AMP drainage rate drops to match its input rate at 0.03 mM/hr.
- The dynamic response of the energy charge (Figure 8.22b) shows that it drops on the faster time scale from an initial value of 0.86 to reach a minimum of about 0.67 at about 1.5 min. This initial response results from the increase in the ATP load parameter of 50%. After this initial response, the energy charge increases on the slower time scale to an eventual value of about 0.82.
- Notice that this secondary response is not a result of a regulatory mechanism, but is a property that is built into the stoichiometric structure and the values of the rate constants that lead to the time scale separation.

##### Summary¶

- Most biochemical reactions are bilinear. Six of the seven categories of enzymes catalyze bilinear reactions.
- The bilinear properties of biochemical reactions lead to complex patterns of exchange of key chemical moieties and properties. Many such simultaneous exchange processes lead to a ‘tangle of cycles’ in biochemical reaction networks.
- Skeleton (or scaffold) dynamic models of biochemical processes can be carried out using dynamic mass balances based on elementary reaction representations and mass action kinetics.
- Complex kinetic models are built in a bottom-up fashion, adding more details in a step-wise fashion, making sure that every new feature is consistently integrated. This chapter demonstrated a four-step analysis of the ATP cofactor sub-network and then its integration to a skeleton ATP generating pathway.
- Once dynamic network models are formulated, the perturbations to which we simulate their responses are in fluxes, typically the exchange and demand fluxes.
- A recurring theme is the formation of pools and the state of those pools in terms of how their total concentration is distributed among its constituent members.
- Some dynamic properties are a result of the stoichiometric structure and do not result from intricate regulatory mechanisms or complex kinetic expressions.

\(\tiny{\text{© B. Ø. Palsson 2011;}\ \text{This publication is in copyright.}\\ \text{Subject to statutory exception and to the provisions of relevant collective licensing agreements,}\\ \text{no reproduction of any part may take place without the written permission of Cambridge University Press.}}\)

#### Regulation as Elementary Phenomena¶

In the previous chapter, we demonstrated that the dynamic states of biochemical reaction networks can be characterized by elementary reactions. We now show how the phenomena of regulation can be described and simulated in the same framework by developing a simulation model of regulation of a prototypical biosynthetic pathway. The chemical reactions that underlie the regulatory steps are identified and their kinetic properties are estimated. These reactions are then added to the scaffold formed
by the basic mass action kinetic description of the network of interest to simulate the effects of the regulation. This approach will then be applied to realistic situations in Part IV.

**MASSpy** will be used to demonstrate some of the topics in this chapter.

```
[1]:
```

```
from mass import (
    MassModel, MassMetabolite, MassReaction,
    Simulation, MassSolution, strip_time)
from mass.util.matrix import nullspace, left_nullspace
from mass.visualization import plot_time_profile, plot_phase_portrait
```

Other useful packages are also imported at this time.

```
[2]:
```

```
import numpy as np
import pandas as pd
import sympy as sym
import matplotlib.pyplot as plt
XL_FONT = {"size": "x-large"}
```

##### Regulation of Enzymes¶

Many factors regulate enzymes, their concentration, and their catalytic activity. We describe four different mechanisms here (Figure 9.1).

- *Regulation of gene expression:* the transcription of genes is regulated in an intricate way. Many proteins, called transcription factors, bind to the promoter region of a gene. Their binding can *induce* or *repress* gene expression. Metabolites often determine the active states of transcription factors.
- *Interconversion:* regulated enzymes can exist in many functional states. As we saw in Chapter 5, a regulatory enzyme can naturally exist in two conformations: catalytically-active and catalytically-inactive. Often, regulated enzymes are chemically modified through phosphorylation, methylation, or acylation to inter-convert them between inactive and active states.
- *Binding by ligands:* small molecules can bind to regulatory enzymes in an allosteric binding site (see Chapters 5 and 14). Such binding can promote the relaxed (R) or taught (T) state of the enzyme, leading to a ‘tug of war’ among its states.
- *Cofactor and coenzyme availability:* as detailed at the beginning of Chapter 8, enzymes rely on ‘accessory molecules’ for their function. Thus the availability of such molecules determines the functional state of the enzyme.
- These are four genetic and biochemical mechanisms by which regulation of enzyme catalytic activity is exerted. We will now describe the dynamic consequences of such regulatory actions. We will focus on the binding of regulatory ligands, level 3 in Figure 9.1.

**Figure 9.1:** Four levels of regulation of enzymes: gene expression, interconversion, ligand binding, and cofactor availability. C1 and C2 represent a cofactor or a coenzyme and its two different states (charged and discharged).

##### Unregulated Model¶

Before we begin examining the mechanisms of feedback inhibition, we will first look at an unregulated biosynthetic pathway that branches off a main pathway. In the main pathway, a metabolic intermediate, \(x\_1\), is being formed, and degraded as

\[\begin{equation} \stackrel{b\_1}{\rightarrow} x\_1 \stackrel{v\_0}{\rightarrow} \tag{9.1} \end{equation}\]

Then, an enzyme, \(x\_6\), can be convert \(x\_1\) to \(x\_2\) as

\[\begin{equation} x\_1 + x\_6 \stackrel{v\_1}{\rightarrow} x\_2 \tag{9.2} \end{equation}\]

that is followed by a series of reactions

\[\begin{equation} x\_2 \stackrel{v\_2}{\rightarrow} x\_6 + x\_3 \stackrel{v\_3}{\rightarrow} x\_4 \stackrel{v\_4}{\rightarrow} x\_5 \stackrel{v\_5}{\rightarrow} \tag{9.3} \end{equation}\]

to form \(x\_5\), that is an end product of a the biosynthetic pathway. This system represents a simple schema where a biosynthetic pathway branches off a main pathway, and it can be graphically illustrated as:

**Figure 9.2:** A schematic of a prototypical biosynthetic pathway that takes an intermediate of a main pathway \(x\_1\) and converts it into a product \(x\_5\) that is then used for other purposes such as biosynthesis. Note that the convention for the boundary fluxes \((b\_1,\ v\_0,\ v\_5)\) is to point into the system. The fluxes through these reactions can be into the system (i.e. \(b\_1\)) or out of the system \((v\_0\) and \(v\_5)\) in this case.

###### Define model¶

The model is constructed by defining the reactions involved and specifying the numerical values for the rate constants.

```
[3]:
```

```
# Define model
unregulated = MassModel("Unregulated")

# Define metabolites
x1 = MassMetabolite("x1")
x2 = MassMetabolite("x2")
x3 = MassMetabolite("x3")
x4 = MassMetabolite("x4")
x5 = MassMetabolite("x5")
x6 = MassMetabolite("x6")

# Define reactions
b1 = MassReaction("b1", reversible=False)
b1.add_metabolites({x1: 1})

v0 = MassReaction("v0", reversible=False)
v0.add_metabolites({x1: -1})

v1 = MassReaction("v1", reversible=False)
v1.add_metabolites({x1: -1, x6: -1, x2: 1})

v2 = MassReaction("v2", reversible=False)
v2.add_metabolites({x2: -1, x3: 1, x6:1})

v3 = MassReaction("v3", reversible=False)
v3.add_metabolites({x3:-1, x4:1})

v4 = MassReaction("v4", reversible=False)
v4.add_metabolites({x4: -1, x5: 1})

v5 = MassReaction("v5", reversible=False)
v5.add_metabolites({x5: -1})

# Add reactions to model
unregulated.add_reactions([b1, v0, v1, v2, v3, v4, v5])

# Sort metabolites
unregulated.metabolites.sort()
unregulated.repair()

# Add the custom rate for b1
unregulated.add_custom_rate(b1, custom_rate=b1.kf_str)
```

###### Null spaces and their content: unregulated model¶

We begin our analysis by looking at the contents of the two null spaces. These are topological quantities that are condition independent. In other words, these properties are always the same regardless of the numerical values of the parameters and the steady state.

The right null space has two pathways, one goes from the primary input \((b\_1)\) and out of the biosynthetic pathway \((v\_5)\) and one goes from the primary input \((b\_1)\) and out of the primary pathway \((v\_0)\). These two nulls space vectors correspond to the two pathways of this system.

```
[4]:
```

```
# Obtain nullspace
ns = nullspace(unregulated.S)
# Transpose and iterate through nullspace,
# dividing by the smallest value in each row.
ns = ns.T
for i, row in enumerate(ns):
    minval = np.min(abs(row[np.nonzero(row)]))
    new_row = np.array(row/minval)
    # Round to ensure the nullspace is composed of only integers
    ns[i] = np.array([round(value) for value in new_row])

# Row operations to find meaningful pathways
ns[1] = (ns[0] + 2*ns[1])/7
ns[0] = (ns[0] - ns[1])/2
# Ensure positive stoichiometric coefficients if all are negative
for i, space in enumerate(ns):
    ns[i] = np.negative(space) if all([num <= 0 for num in space]) else space

# Revert transpose
ns = ns.T
# Create a pandas.DataFrame to represent the nullspace
pd.DataFrame(ns, index=[rxn.id for rxn in unregulated.reactions],
             columns=["Path 1", "Path 2"], dtype=np.int64)
```

```
[4]:
```

|  | Path 1 | Path 2 |
| --- | --- | --- |
| b1 | 1 | 1 |
| v0 | 0 | 1 |
| v1 | 1 | 0 |
| v2 | 1 | 0 |
| v3 | 1 | 0 |
| v4 | 1 | 0 |
| v5 | 1 | 0 |

We can visualize the two pathway vectors on the network map as shown in Figure 9.3.

**Figure 9.3:** The two pathways of the of the system under consideration correspond to the two vectors that span the null space of \(\textbf{S}\). One pathway (a) goes from the primary input and out of the biosynthetic pathway while the other (b) goes in the primary input and out of the primary pathway.

The left null space has one pool: the conservation of the enzyme that is found in the active form \((x\_6)\) or in the intermediary complex \((x\_2)\). We can compute the left null space vector and form the conserved pool. Notice that the initial conditions that are used for simulation will fix the size of this pool.

```
[5]:
```

```
# Obtain left nullspace
lns = left_nullspace(unregulated.S)
# Iterate through left nullspace and divide by the smallest value in each row.
for i, row in enumerate(lns):
    minval = np.min(row[np.nonzero(row)])
    lns[i] = np.array(row/minval)
    # Ensure the left nullspace is composed of only integers
    lns[i] = np.array([round(value) for value in lns[i]])

#Create a pandas.DataFrame to represent the left nullspace
pd.DataFrame(lns, columns=unregulated.metabolites,
             index=["Total Enzyme"], dtype=np.int64)
```

```
[5]:
```

|  | x1 | x2 | x3 | x4 | x5 | x6 |
| --- | --- | --- | --- | --- | --- | --- |
| Total Enzyme | 0 | 1 | 0 | 0 | 0 | 1 |

###### Steady state: unregulated model¶

We now evaluate the steady state by simulating the system to very long times. We will set the total enzyme concentration to 1 by specifying \(x\_6 = 1\) and all other concentrations as 0 at the initial time. The flux into the system \((b\_1)\) is given by \(k\_{b\_{1}}^\rightarrow\) of 0.1; a zeroth order reaction, that is, it is a constant and does not respond to any of the state variables (the concentrations) of the system.

```
[6]:
```

```
# Define parameters
b1.kf = 0.1
v0.kf = 0.5
v1.kf = 1
v2.kf = 1
v3.kf = 1
v4.kf = 1
v5.kf = 1

# Define initial conditions
unregulated.update_initial_conditions(
    dict((met, 0) if met.id != "x6"
         else (met, 1) for met in unregulated.metabolites))
```

We simulate the model to obtain the concentration and flux solutions, then we plot the time profile of the concentrations and the fluxes.

```
[7]:
```

```
(t0, tf) = (0, 1e4)
sim_unreg = Simulation(unregulated, id="Enzyme_Regulation", verbose=True)
conc_sol, flux_sol = sim_unreg.simulate(
    unregulated, time=(t0, tf, tf*10 + 1),
    interpolate=True, verbose=True)

# Place models and simulations into lists for later
models = [unregulated]
simulations = [sim_unreg]
```

```
WARNING: No compartments found in model. Therefore creating compartment 'compartment' for entire model.
```

```
Successfully loaded MassModel 'Unregulated' into RoadRunner.
Getting time points
Setting output selections
Setting simulation values for 'Unregulated'
Simulating 'Unregulated'
Simulation for 'Unregulated' successful
Adding 'Unregulated' simulation solutions to output
Updating stored solutions
```

```
[8]:
```

```
fig, axes = plt.subplots(nrows=2, ncols=1, figsize=(8, 5), )
(ax1, ax2) = axes.flatten()

plot_time_profile(
    conc_sol, ax=ax1, legend="right outside",
    plot_function="semilogx",
    xlabel="Time [hr]", ylabel="Concentrations [mM]",
    title=("Concentrations", XL_FONT));

plot_time_profile(
    flux_sol, ax=ax2, legend="right outside",
    plot_function="semilogx",
    xlabel="Time [hr]", ylabel="Flux [mM/hr]",
    title=("Fluxes", XL_FONT));
```

```
[8]:
```

```
<matplotlib.axes._subplots.AxesSubplot at 0x7fe9d13d7250>
```

We can call out the values as particular time points if we like.

```
[9]:
```

```
time_points = [1e0, 1e1, 1e2, 1e3]
# Make a pandas DataFrame using a dictionary and generators
pd.DataFrame({rxn: [round(value, 3) for value in flux_func(time_points)]
              for rxn, flux_func in flux_sol.items()},
             index=["t=%i" % t for t in time_points])
```

```
[9]:
```

|  | b1 | v0 | v1 | v2 | v3 | v4 | v5 |
| --- | --- | --- | --- | --- | --- | --- | --- |
| t=1 | 0.1 | 0.026 | 0.051 | 0.023 | 0.007 | 0.002 | 0.000 |
| t=10 | 0.1 | 0.035 | 0.065 | 0.065 | 0.065 | 0.065 | 0.064 |
| t=100 | 0.1 | 0.035 | 0.065 | 0.065 | 0.065 | 0.065 | 0.065 |
| t=1000 | 0.1 | 0.035 | 0.065 | 0.065 | 0.065 | 0.065 | 0.065 |

We note that in the eventual steady state that all the fluxes in the biosynthetic pathway are equal, and that the overall flux balance on inputs and outputs, that is \(b\_1 = v\_0 + v\_5\), holds.

**A numerical QC/QA**: Check the size of the enzyme pool at various time points:

```
[10]:
```

```
conc_sol.make_aggregate_solution(
    "Total_Enzyme", equation="x2 + x6", variables=["x2", "x6"]);

pd.DataFrame({
    "Total Enzyme": conc_sol["Total_Enzyme"](time_points)},
    index=["t=%i" % t for t in time_points]).T
```

```
[10]:
```

|  | t=1 | t=10 | t=100 | t=1000 |
| --- | --- | --- | --- | --- |
| Total Enzyme | 1.0 | 1.0 | 1.0 | 1.0 |

We can compute the steady state concentrations and replace the initial conditions of the model with those steady state concentrations. This will put the system into a steady state from which we can perturb the system. We will look at perturbations in the input flux \(b\_1\).

```
[11]:
```

```
unregulated_ss = sim_unreg.find_steady_state(
    unregulated, strategy="simulate", update_values=True,
    verbose=True)
```

```
WARNING: No compartments found in model. Therefore creating compartment 'compartment' for entire model.
```

```
Setting output selections
Setting simulation values for 'Unregulated'
Setting output selections
Getting time points
Simulating 'Unregulated'
Found steady state for 'Unregulated'.
Updating 'Unregulated' values
Adding 'Unregulated' simulation solutions to output
```

###### Dynamic states: unregulated model¶

###### Simulate the response to a step change in the input \(b\_1\) by 10-fold¶

We will now perform dynamic simulation by simulating the response to a 10-fold change in the input flux \(b\_1\). This perturbation will create a strong dynamic response, after which the systems settles down in a new steady state. In a separate plot we will focus on \(v\_1\) and \(v\_5\) as the inputs and outputs to the biosynthetic pathway that we will trying to regulate below.

```
[12]:
```

```
scalar = 10
perturbation_dict = {"kf_b1": "kf_b1 * {0}".format(scalar)}

t0, tf = (0, 50)
conc_sol, flux_sol = sim_unreg.simulate(
    unregulated, time=(t0, tf, tf*10+ 1),
    perturbations=perturbation_dict)
```

```
[13]:
```

```
fig_9_4, axes = plt.subplots(nrows=3, ncols=1, figsize=(8, 6))
(ax1, ax2, ax3) = axes.flatten()

plot_time_profile(
    conc_sol, ax=ax1, legend="right outside",
    xlabel="Time [hr]", ylabel="Concentrations [mM]",
    title=("(a) Concentrations", XL_FONT));

plot_time_profile(
    flux_sol, ax=ax2, legend="right outside",
    xlabel="Time [hr]", ylabel="Flux [mM/hr]",
    title=("(b) Fluxes", XL_FONT));

plot_time_profile(
    flux_sol, observable=["v1", "v5"], ax=ax3,
    legend="right outside",
    xlabel="Time [hr]", ylabel="Flux [mM/hr]",
    title=("(c) Responses of v1 and v5", XL_FONT),
    color="black", linestyle=["-", "--"]);
fig_9_4.tight_layout()
```

**Figure 9.4:** The time profiles for the concentrations and fluxes involved in the simple feedback control loop without the regulatory mechanism. The parameter values used are \(k\_0 = 0.5\), \(k\_1 = k\_2 = k\_3 = k\_4 = k\_5 = 1\), and \(e\_t = 1\). Just prior to time zero, the input rate is \(b\_1 = 0.1\), and the model is at steady state where \(x\_1 = 0.0697\), \(x\_2 = x\_3 = x\_4 = x\_5 = 0.0652\), \(x\_6 = 0.935\). The input rate is changed to a specified number at time
zero and the dynamic response is simulated. (a) The concentrations as a function of time. (b) The reaction fluxes as a function of time. (c) Dynamic response of \(v\_1\) and \(v\_5\) to the perturbation.

###### Seeking a graphical representation to understand the solution better¶

We introduce two useful phase portraits. First we notice that \(v\_1\) and \(v\_5\) have to be equal in a steady state, that is the input and output of the biosynthetic pathway have to be equal. If we plot them on a phase portrait, we see that the initial and final points have to be on the 45 degree line where the two fluxes are equal. We also see that the final state after the perturbation lies far away from the initial state. The dynamics are such that the flux through the first step in
the pathway \(v\_1\) changes first creating a horizontal motion, that is followed by a change in the output flux, \(v\_5\), creating a vertical motion to the 45 degree line where the system settles down. We notice that a lot of the additional incoming flux through \(b\_1\) goes down the biosynthetic pathway. Below we will study how regulation can diminish the flux through the biosynthetic pathway.

Second, we know that the overall flux balance on inputs and outputs, that is \(b\_1 = v\_0 + v\_5\), hold at the starting point and final point. These will be represented by lines of negative slop of 45 degrees in a phase portrait formed by \(v\_0\) and \(v\_5\) since there sum is a constant. This line is given by \(v\_5 = b\_1 - v\_0\). These lines corresponding to the initial flux and perturbed flux through \(b\_1\) can be graphed. The initial and final states must be on these two
lines.

```
[14]:
```

```
fig_9_5, axes = plt.subplots(nrows=1, ncols=2, figsize=(8, 4))
(ax1, ax2) = axes.flatten()


# Plot v1 vs. v5
plot_phase_portrait(
    flux_sol, x="v1", y="v5", ax=ax1,
    xlim=(0, 0.6), ylim=(0, 0.6),
    xlabel="v1", ylabel="v5",
    title=("(a) v1 vs. v5", XL_FONT),
    annotate_time_points="endpoints",
    annotate_time_points_legend="best");

# Plot v0 vs. v5
plot_phase_portrait(
    flux_sol, x="v0", y="v5", ax=ax2,
    legend=([unregulated.id], "right outside"),
    xlim=(0, 1), ylim=(0, 1),
    xlabel="v0", ylabel="v5",
    title=("(a) v0 vs. v5", XL_FONT),
    annotate_time_points="endpoints",
    annotate_time_points_legend="best");

# Annotate steady state line on first plot
ax1.plot([0, 1], [0, 1], color="grey", linestyle=":");

# Annotate flux balance lines on second plot
ax2.plot([0, .1], [.1, 0], color="grey", linestyle=":");
ax2.plot([0, .1*scalar], [.1*scalar, 0], color="grey", linestyle=":");
fig_9_5.tight_layout()
```

**Figure 9.5:** Dynamic phase portraits for (a) the flux into \((v\_1)\) and out of \((v\_5)\) in the biosynthetic pathway and (b) the flux out of the primary pathway \((v\_0)\) and out of the biosynthetic pathway \((v\_5).\) The model is the same as in Figure 9.4. The red points are at the start time and the blue points are at the final time.

###### Breaking down the steady states into the pathway vectors¶

We can study the steady state solutions by breaking them down into a linear combination of the two pathway vectors in the null space. These two vectors span the null space and can be used to represent any given valid steady state solution. We can tabulate the two pathway vectors and the initial and final flux states as;

```
[15]:
```

```
unregulated_ss_perturbed = sim_unreg.find_steady_state(
    unregulated, strategy="simulate",
    perturbations=perturbation_dict)

unperturbed = [round(value,3) for value in unregulated_ss[1].values()]
perturbed = [round(value,3) for value in unregulated_ss_perturbed[1].values()]

pd.DataFrame(np.vstack((ns.T, unperturbed, perturbed)),
             index=["Nullspace Path 1", "Nullspace Path 2", "Unperturbed fluxes", "Perturbed fluxes"],
             columns=[rxn.id for rxn in unregulated.reactions],
             dtype=np.float64)
```

```
[15]:
```

|  | b1 | v0 | v1 | v2 | v3 | v4 | v5 |
| --- | --- | --- | --- | --- | --- | --- | --- |
| Nullspace Path 1 | 1.0 | 0.000 | 1.000 | 1.000 | 1.000 | 1.000 | 1.000 |
| Nullspace Path 2 | 1.0 | 1.000 | 0.000 | 0.000 | 0.000 | 0.000 | 0.000 |
| Unperturbed fluxes | 0.1 | 0.035 | 0.065 | 0.065 | 0.065 | 0.065 | 0.065 |
| Perturbed fluxes | 1.0 | 0.500 | 0.500 | 0.500 | 0.500 | 0.500 | 0.500 |

The overall flux balance is \(b\_1 = v\_0 + v\_5\). Thus, the first pathway goes from 35% of \(b\_1\) to 50% of \(b\_1\) after the perturbation and the second pathway goes from a 65% of \(b\_1\) to 50% of \(b\_1\) after the perturbation.

**A numerical QC/QA:** The loadings on \(v\_0\) and \(v\_5\) should add up to \(b\_1\):

```
[16]:
```

```
before = unregulated_ss[1]["v0"] +\
         unregulated_ss[1]["v5"] == unregulated_ss[1]["b1"]
after = unregulated_ss_perturbed[1]["v0"] +\
        unregulated_ss_perturbed[1]["v5"] == unregulated_ss_perturbed[1]["b1"]

pd.DataFrame([before, after],
             index=["Before Perturbation", "After Perturbation"],
             columns=["Add up to b1?"])
```

```
[16]:
```

|  | Add up to b1? |
| --- | --- |
| Before Perturbation | True |
| After Perturbation | True |

##### Regulated Model: Feedback Inhibition of a Pathway¶

In this section we study the mechanisms of feedback inhibition by the end product of the biosynthetic pathway on the first reaction in the pathway. We can use simulation and case studies to determine the effectiveness of the regulatory action. We will use elementary reactions to describe the regulatory mechanism.

###### Feedback regulation in a biosynthetic pathway¶

In a biosynthetic pathway, the first reaction is often inhibited by binding of the end product of the pathway to the regulated enzyme. The end product, \(x\_5\), feedback inhibits the enzyme, \(x\_6\), by binding to it and converting it into an inactive form:

\[\begin{equation} x\_6 + x\_5 \underset{v\_{-6}}{\stackrel{v\_6}{\rightleftharpoons}} x\_7 \tag{9.4} \end{equation}\]

This regulation represents a simple negative feedback loop. It can be added into the schematic of the system, resulting in Figure 9.6.

**Figure 9.6:** A schematic of a prototypical feedback loop for a biosynthetic pathway. The end product of the pathway, \(x\_5\), feedback inhibits the flux into the pathway by binding to the enzyme catalyzing the first reaction of the pathway.

###### Expand model¶

The model is expanded by defining the binding and conversion of the enzyme, \(x\_6\), to its inactive form and specifying the numerical values for the rate constants.

```
[17]:
```

```
# Copy the unregulated model
monomer = unregulated.copy()
# Change the model ID
monomer.id = "Monomer"

# Define new metabolite
x7 = MassMetabolite("x7")
mets = monomer.metabolites

# Define new reaction and set parameters
v6 = MassReaction("v6");
v6.add_metabolites({mets.x5: -1, mets.x6: -1, x7:1})
v6.get_mass_action_rate(rate_type=2, update_reaction=True)
# Add Reaction to the model
monomer.add_reactions([v6])
monomer.update_S(array_type="DataFrame");
```

```
[17]:
```

|  | b1 | v0 | v1 | v2 | v3 | v4 | v5 | v6 |
| --- | --- | --- | --- | --- | --- | --- | --- | --- |
| x1 | 1.0 | -1.0 | -1.0 | 0.0 | 0.0 | 0.0 | 0.0 | 0.0 |
| x2 | 0.0 | 0.0 | 1.0 | -1.0 | 0.0 | 0.0 | 0.0 | 0.0 |
| x3 | 0.0 | 0.0 | 0.0 | 1.0 | -1.0 | 0.0 | 0.0 | 0.0 |
| x4 | 0.0 | 0.0 | 0.0 | 0.0 | 1.0 | -1.0 | 0.0 | 0.0 |
| x5 | 0.0 | 0.0 | 0.0 | 0.0 | 0.0 | 1.0 | -1.0 | -1.0 |
| x6 | 0.0 | 0.0 | -1.0 | 1.0 | 0.0 | 0.0 | 0.0 | -1.0 |
| x7 | 0.0 | 0.0 | 0.0 | 0.0 | 0.0 | 0.0 | 0.0 | 1.0 |

###### Null spaces and their content: regulated model¶

When we look at the right null space, we observe that the addition of the inhibition step did not change the pathway vectors, meaning that \(v\_6\) is a dead-end reaction and is not found in any pathway as it carries no flux in a steady state; it is at equilibrium in the steady state.

```
[18]:
```

```
# Obtain nullspace
ns = nullspace(monomer.S, rtol=1e-10)
# Transpose and iterate through nullspace,
# dividing by the smallest value in each row.
ns = ns.T
for i, row in enumerate(ns):
    minval = np.min(abs(row[np.nonzero(row)]))
    new_row = np.array(row/minval)
    # Round to ensure the nullspace is composed of only integers
    ns[i] = np.array([round(value) for value in new_row])

# Find the viable pathways using linear combinations of the nullspace
ns[1] = (ns[0] + 2*ns[1])/7
ns[0] = (ns[0] - ns[1])/2

# Ensure positive stoichiometric coefficients if all are negative
for i, space in enumerate(ns):
    ns[i] = np.negative(space) if all([num <= 0 for num in space]) else space

# Revert transpose
ns = ns.T
# Create a pandas.DataFrame to represent the nullspace
pd.DataFrame(ns, index=[rxn.id for rxn in monomer.reactions],
             columns=["Path 1", "Path 2"], dtype=np.int64)
```

```
[18]:
```

|  | Path 1 | Path 2 |
| --- | --- | --- |
| b1 | 1 | 1 |
| v0 | 0 | 1 |
| v1 | 1 | 0 |
| v2 | 1 | 0 |
| v3 | 1 | 0 |
| v4 | 1 | 0 |
| v5 | 1 | 0 |
| v6 | 0 | 0 |

**Figure 9.7:** The two pathways of a prototypical negative feedback loop for the biosynthetic pathway. As for the unregulated system, one pathway (a) goes from the primary input and out of the biosynthetic pathway while the other (b) goes in the primary input and out of the primary pathway.

The left null space has one pool: the conservation of the enzyme that is found in the active form \((x\_6)\), in the intermediary complex \((x\_2)\) or in the inhibited form \((x\_7)\) when it is bound to the inhibitor \((x\_5)\) We can compute the left null space vector and form the conserved pool.

```
[19]:
```

```
# Obtain left nullspace
lns = left_nullspace(monomer.S, rtol=1e-10)
# Iterate through left nullspace and divide by the smallest value in each row.
for i, row in enumerate(lns):
    minval = np.min(row[np.nonzero(row)])
    lns[i] = np.array(row/minval)
    # Ensure the left nullspace is composed of only integers
    lns[i] = np.array([round(value) for value in lns[i]])

#Create a pandas.DataFrame to represent the left nullspace
pd.DataFrame(lns, columns=monomer.metabolites,
             index=["Total Enzyme"], dtype=np.int64)
```

```
[19]:
```

|  | x1 | x2 | x3 | x4 | x5 | x6 | x7 |
| --- | --- | --- | --- | --- | --- | --- | --- |
| Total Enzyme | 0 | 1 | 0 | 0 | 0 | 1 | 1 |

###### Steady state: regulated model¶

We now evaluate the steady state by simulating the system to very long times. The total enzyme concentration to 1 by specifying \(x\_6 = 1\) and all other concentrations as 0 at the initial time.

```
[20]:
```

```
# Define new parameters
v6.kf = 10
v6.kr = 1

# Define initial conditions
monomer.update_initial_conditions(
    dict((met, 0) if met.id != "x6"
         else (met, 1) for met in monomer.metabolites))
```

We simulate the model to obtain the concentration and flux solutions, then we plot the time profile of the concentrations and the fluxes.

```
[21]:
```

```
(t0, tf) = (0, 1e4)
sim_monomer = Simulation(monomer, verbose=True)
conc_sol, flux_sol = sim_monomer.simulate(
    monomer, time=(t0, tf, tf*10 + 1),
    interpolate=True, verbose=True)

# Place models and simulations into lists for later
models += [monomer]
simulations += [sim_monomer]
```

```
WARNING: No compartments found in model. Therefore creating compartment 'compartment' for entire model.
```

```
Successfully loaded MassModel 'Monomer' into RoadRunner.
Getting time points
Setting output selections
Setting simulation values for 'Monomer'
Simulating 'Monomer'
Simulation for 'Monomer' successful
Adding 'Monomer' simulation solutions to output
Updating stored solutions
```

```
[22]:
```

```
fig, axes = plt.subplots(nrows=2, ncols=1, figsize=(8, 5))
(ax1, ax2) = axes.flatten()

plot_time_profile(
    conc_sol, ax=ax1, legend="right outside",
    plot_function="semilogx",
    xlabel="Time [hr]", ylabel="Concentrations [mM]",
    title=("Concentrations", XL_FONT));

plot_time_profile(
    flux_sol, ax=ax2, legend="right outside",
    plot_function="semilogx",
    xlabel="Time [hr]", ylabel="Flux [mM/hr]",
    title=("Fluxes", XL_FONT));
fig.tight_layout()
```

We note that in the eventual steady state that; i) all the fluxes in the biosynthetic pathway are equal, ii) that the overall flux balance on inputs and outputs, \(b\_1 = v\_0 + v\_5\), holds, and iii) the inhibitor binding reactions, \(v\_6\) and \(v\_7\), go to equilibrium, i.e. \(v\_6= v\_7= 0.\)

```
[23]:
```

```
time_points = [1e0, 1e1, 1e2, 1e3]
# Make a pandas DataFrame using a dictionary and generators
pd.DataFrame({rxn: [round(value, 3) for value in flux_func(time_points)]
              for rxn, flux_func in flux_sol.items()},
             index=["t=%i" % t for t in time_points])
```

```
[23]:
```

|  | b1 | v0 | v1 | v2 | v3 | v4 | v5 | v6 |
| --- | --- | --- | --- | --- | --- | --- | --- | --- |
| t=1 | 0.1 | 0.026 | 0.051 | 0.023 | 0.007 | 0.002 | 0.000 | 0.001 |
| t=10 | 0.1 | 0.040 | 0.058 | 0.059 | 0.059 | 0.060 | 0.035 | 0.022 |
| t=100 | 0.1 | 0.045 | 0.055 | 0.055 | 0.055 | 0.055 | 0.055 | -0.000 |
| t=1000 | 0.1 | 0.045 | 0.055 | 0.055 | 0.055 | 0.055 | 0.055 | 0.000 |

**A numerical QC/QA**: Check the size of the enzyme pool at various time points

```
[24]:
```

```
conc_sol.make_aggregate_solution(
    "Total_Enzyme", equation="x2 + x6 + x7",
    variables=["x2", "x6", "x7"]);

pd.DataFrame({
    "Total Enzyme": conc_sol["Total_Enzyme"](time_points)},
    index=["t=%i" % t for t in time_points]).T
```

```
[24]:
```

|  | t=1 | t=10 | t=100 | t=1000 |
| --- | --- | --- | --- | --- |
| Total Enzyme | 1.0 | 1.0 | 1.0 | 1.0 |

As we did with the unregulated system, we can compute the steady state and define it as the initial condition for any subsequent simulations.

```
[25]:
```

```
monomer_ss = sim_monomer.find_steady_state(
    monomer, strategy="simulate", update_values=True,
    verbose=True)
```

```
WARNING: No compartments found in model. Therefore creating compartment 'compartment' for entire model.
```

```
Setting output selections
Setting simulation values for 'Monomer'
Setting output selections
Getting time points
Simulating 'Monomer'
Found steady state for 'Monomer'.
Updating 'Monomer' values
Adding 'Monomer' simulation solutions to output
```

###### Dynamic states: regulated model¶

###### Simulate the response to a step change in the input \(b\_1\):¶

We will now simulate the feedback model’s response to an x-fold change in the input flux \(b\_1\) and compare it to the unregulated model in order to analyze the effectiveness of the regulatory mechanism. The simulation results can be displayed as time profiles of the (a) concentrations, (b) fluxes and (c) of \((v\_1, v\_5)\), providing an initial visualization of the solution as before.

```
[26]:
```

```
scalar = 10
perturbation_dict = {"kf_b1": "kf_b1 * {0}".format(scalar)}

t0, tf = (0, 50)
conc_sol, flux_sol = sim_monomer.simulate(
    monomer, time=(t0, tf, tf*10 + 1),
    perturbations=perturbation_dict)
```

```
[27]:
```

```
fig_9_8, axes = plt.subplots(nrows=3, ncols=1, figsize=(8, 6))
(ax1, ax2, ax3) = axes.flatten()

plot_time_profile(
    conc_sol, ax=ax1, legend="right outside",
    xlabel="Time [hr]", ylabel="Concentrations [mM]",
    title=("(a) Concentrations", XL_FONT));

plot_time_profile(
    flux_sol, ax=ax2, legend="right outside",
    xlabel="Time [hr]", ylabel="Flux [mM/hr]",
    title=("(b) Fluxes", XL_FONT));

plot_time_profile(
    flux_sol, observable=["v1", "v5"], ax=ax3,
    legend="right outside",
    xlabel="Time [hr]", ylabel="Flux [mM/hr]",
    title=("(c) Responses of v1 and v5", XL_FONT),
    color="black", linestyle=["-", "--"]);
fig_9_8.tight_layout()
```

**Figure 9.8:** The time profiles for the concentrations and fluxes involved in the simple feedback control loop. The parameter values used are \(k\_0 = 0.5\), \(k\_1 = k\_2 = k\_3 = k\_4 = k\_5 = 1\), and \(e\_t = 1\). Just prior to time zero, the input rate is \(b\_1 = 0.1\), the inhibitor binding rate is \(k\_6\)=10, and the feedback loop is at steady state where \(x\_1 = 0.090\), \(x\_2 = x\_3 = x\_4 = x\_5 = 0.055\), \(x\_6 = 0.610\), \(x\_7 = 0.335\). The input rate
is changed to a specified number at time zero and the dynamic response is simulated. (a) The concentrations as a function of time. (b) The reaction fluxes as a function of time. (c) Dynamic response of \(v\_1\) and \(v\_5\) to the perturbation.

If you put a 10-fold change on \(b\_1\) you can see in: Panel (a) that the concentration of the inhibited enzyme \((x\_7)\) increases as the concentration of the inhibitor \((x\_5)\) rises; Panel (b) that the reduction of the concentration active enzymes leads to a reduction in the fluxes into the biosynthetic pathway creating an overshoot in the flux values; and in Panel (c) how the input \((v\_1)\) and the output \((v\_5)\) of the biosynthetic pathway come into a steady state.
First the input rises rapidly, and it reaches a peak as the output flux begins to rise as a consequence of buildup of \(x\_5\). The buildup of \(x\_5\) leads to a reduction of the input flux through the feedback mechanism until it matches the output and steady state is reached.

We can graph the input and the output flux in a phase portrait, and compare it to the response of the unregulated system that we simulated in the previous section. The difference is the pull back in \(v\_5\) and that leads to a steady state on the 45 degree line that is much closer to the initial state as compared to the final state of the unregulated system. We can also graph the over all flux balance as before by plotting the two pathway outputs \(v\_0\) and \(v\_5\) against one
another. The endpoint of the simulation will be on the 45 degree line whose y-intercept is given by the numerical value of \(b\_1\). The trajectory of the regulated system is more in the horizontal direction than the unregulated system, and the final resting point shows that much more of the disturbance comes out of the primary pathway than out of the biosynthetic pathway.

Even if the feedback regulation acts to overcome the effects of the disturbance in \(b\_1\), it does not eliminate the additional flux that it creates in the biosynthetic pathway.

```
[28]:
```

```
fig_9_9, axes = plt.subplots(nrows=1, ncols=2, figsize=(8, 4))
(ax1, ax2) = axes.flatten()


for model, sim in zip(models, simulations):
    conc_sol, flux_sol = sim.simulate(
        model, time=(t0, tf, tf*10 + 1),
        perturbations=perturbation_dict)

    # Plot v1 vs. v5
    plot_phase_portrait(
        flux_sol, x="v1", y="v5", ax=ax1,
        xlim=(0, 0.6), ylim=(0, 0.6),
        xlabel="v1", ylabel="v5",
        title=("(a) v1 vs. v5", XL_FONT),
        annotate_time_points="endpoints",
        annotate_time_points_legend="best");

    # Plot v0 vs. v5
    plot_phase_portrait(
        flux_sol, x="v0", y="v5", ax=ax2,
        legend=([model.id], "right outside"),
        xlim=(0, 1), ylim=(0, 1),
        xlabel="v0", ylabel="v5",
        title=("(a) v0 vs. v5", XL_FONT),
        annotate_time_points="endpoints",
        annotate_time_points_legend="best");

# Annotate steady state line on first plot
ax1.plot([0, 1], [0, 1], color="grey", linestyle=":");

# Annotate flux balance lines on second plot
ax2.plot([0, .1], [.1, 0], color="grey", linestyle=":");
ax2.plot([0, .1*scalar], [.1*scalar, 0], color="grey", linestyle=":");
fig_9_9.tight_layout()
```

**Figure 9.9:** Dynamic phase portraits for (a) the flux into \((v\_1)\) and out of \((v\_5)\) in the biosynthetic pathway and (b) the flux out of the primary pathway \((v\_0)\) and out of the biosynthetic pathway \((v\_5)\). The red points are at the start time and the blue points are at the final time.

As we can surmise from the above simulations, the state of the enzyme is a key consideration. Either it is inhibited \((x\_7)\) or it is actively carrying out catalysis \((x\_2 + x\_6)\). Since all the forms of the enzyme from a constant pool (the time invariant pool in the left null space) we can graph \(x\_2 + x\_6\) against \(x\_7\). This plot will be on the -45 degree line with the x- and y-axis intercepts corresponding to the total amount of enzyme present. In the initial steady
state 66.5% of the enzyme pool is active. For a 10-fold increase in \(b\_1\), the active form of the enzyme is 47.9% of the total enzyme present.

```
[29]:
```

```
enzyme_pools = {
    "Free": ["x2 + x6", ["x2", "x6"]],
    "Monomer_Inhibited": ["x7", ["x7"]]}
for pool_id, args in enzyme_pools.items():
    equation, variables = args
    conc_sol.make_aggregate_solution(
        pool_id, equation, variables)

fig_9_10, ax = plt.subplots(nrows=1, ncols=1, figsize=(6.5, 5))

plot_phase_portrait(
    conc_sol, x="Free", y=monomer.id + "_Inhibited", ax=ax,
    legend=([monomer.id], "right outside"),
    xlim=(0.3, .7), ylim=(0.3, .7),
    xlabel="Free Enzyme", ylabel="Inhibited Enzyme",
    color=["red"], linestyle=["--"],
    title=("Free vs. Inhibited Enzyme", XL_FONT),
    annotate_time_points="endpoints",
    annotate_time_points_color=["red"],
    annotate_time_points_labels=True)

# Add line representing the constant enzyme
ax.plot([0, 1], [1, 0], color="grey", linestyle=":");
fig_9_10.tight_layout()
```

**Figure 9.10:** Dynamic phase portrait of the inhibited enzyme vs. the free enzyme for the monomer.

###### Breaking down the steady states into the pathway vectors: regulated model¶

As before, we can evaluate the steady state solutions by breaking them down into a linear combination of the two pathway vectors in the null space.

```
[30]:
```

```
monomer_ss_perturbed = sim_monomer.find_steady_state(
    monomer, strategy="simulate", perturbations=perturbation_dict)

unperturbed = [round(value,3) for value in monomer_ss[1].values()]
perturbed = [round(value,3) for value in monomer_ss_perturbed[1].values()]

pd.DataFrame(
    np.vstack((ns.T, unperturbed, perturbed)),
    index=["Nullspace Path 1", "Nullspace Path 2", "Unperturbed fluxes", "Perturbed fluxes"],
    columns=[rxn.id for rxn in monomer.reactions],
    dtype=np.float64)
```

```
[30]:
```

|  | b1 | v0 | v1 | v2 | v3 | v4 | v5 | v6 |
| --- | --- | --- | --- | --- | --- | --- | --- | --- |
| Nullspace Path 1 | 1.0 | 0.000 | 1.000 | 1.000 | 1.000 | 1.000 | 1.000 | 0.0 |
| Nullspace Path 2 | 1.0 | 1.000 | 0.000 | 0.000 | 0.000 | 0.000 | 0.000 | 0.0 |
| Unperturbed fluxes | 0.1 | 0.045 | 0.055 | 0.055 | 0.055 | 0.055 | 0.055 | 0.0 |
| Perturbed fluxes | 1.0 | 0.723 | 0.277 | 0.277 | 0.277 | 0.277 | 0.277 | -0.0 |

The overall flux balance is \(b\_1 = v\_0 + v\_5\). Thus, the biosynthetic pathway goes from 55% of \(b\_1\) to 27.7% of \(b\_1\) after the perturbation. In contrast, the unregulated system goes from 65% to 50% of \(b\_1\). Therefore, the regulation diverts a part of the incoming flux of \(b\_1\) out the primary pathway.

**A numerical QC/QA:** The loadings on \(v\_0\) and \(v\_5\) should add up to \(b\_1\):

```
[31]:
```

```
before = monomer_ss[1]["v0"] +\
         monomer_ss[1]["v5"] == monomer_ss[1]["b1"]
after = monomer_ss_perturbed[1]["v0"] +\
        monomer_ss_perturbed[1]["v5"] == monomer_ss_perturbed[1]["b1"]

pd.DataFrame([before, after],
             index=["Before Perturbation", "After Perturbation"],
             columns=["Add up to b1?"])
```

```
[31]:
```

|  | Add up to b1? |
| --- | --- |
| Before Perturbation | True |
| After Perturbation | True |

##### Dimer Model of the Regulated Enzyme¶

###### Building on a scaffold¶

The reaction network studied in the previous section represents the basic framework for feedback regulation of a biosynthetic pathway. This schema operates in a cellular environment that has additional regulatory features. These can be built on top of this basic structure. We will consider two additional regulatory mechanisms. First, we consider more and more realistic mechanisms for \(x\_5\) binding to the regulated enzyme. Second, we look at the regulation of protein synthesis and more
elaborate and realistic schemas for the inhibition of the first enzyme in the pathway. The two can be combined. Such additions take into account more processes and make the models more realistic.

###### More realistic mechanism for \(x\_5\) binding¶

Regulatory enzymes have a more complex mechanism than simply having an active and inactive state, as denoted by \(x\_6\) and \(x\_7\) in Section 9.3. Regulatory enzymes often have a series of binding sites for inhibitory molecules. One mechanism for such serial binding is the symmetry model, described in Section 5.5. The reaction mechanisms (using the same compound names as in Section 9.3) for a dimer are shown below.

**Figure 9.11:** The reaction mechanism for a dimer.

For the dimer case, \(x\_7\) has one ligand bound and \(x\_8\) has two.

###### Define dimer model¶

Add the first additional binding reaction and adjust the corresponding rate equations for the effective concentrations of the multiple binding sites.

**Figure 9.12:** Visual representation of a prototypical negative feedback loop for a biosynthetic pathway for the dimer model. The additional dimer reactions are shown in light blue.

```
[32]:
```

```
# Copy the monomer model
dimer = monomer.copy()
# Change the model ID
dimer.id = "Dimer"

# Define new metabolite
x8 = MassMetabolite("x8")
mets = dimer.metabolites

# Define new reaction and set parameters
v7 = MassReaction("v7");
v7.add_metabolites({mets.x5: -1, mets.x7: -1, x8:1})
v7.get_mass_action_rate(rate_type=2, update_reaction=True)
# Add Reaction to the model
dimer.add_reactions([v7])
```

###### Null spaces and their content: dimer model¶

When we look at the right null space, we observe that the addition of another inhibition step did not change the pathway vectors.

```
[33]:
```

```
# Obtain nullspace
ns = nullspace(dimer.S, rtol=1e-10)
# Transpose and iterate through nullspace,
# dividing by the smallest value in each row.
ns = ns.T
for i, row in enumerate(ns):
    minval = np.min(abs(row[np.nonzero(row)]))
    new_row = np.array(row/minval)
    # Round to ensure the nullspace is composed of only integers
    ns[i] = np.array([round(value) for value in new_row])

# Find the viable pathways using linear combinations of the nullspace
ns[1] = (3*ns[0] - ns[1])/13
ns[0] = ns[0] - 3 * ns[1]

# Ensure positive stoichiometric coefficients if all are negative
for i, space in enumerate(ns):
    ns[i] = np.negative(space) if all([num <= 0 for num in space]) else space

# Revert transpose
ns = ns.T
# Create a pandas.DataFrame to represent the nullspace
pd.DataFrame(ns, index=[rxn.id for rxn in dimer.reactions],
             columns=["Path 1", "Path 2"], dtype=np.int64)
```

```
[33]:
```

|  | Path 1 | Path 2 |
| --- | --- | --- |
| b1 | 1 | 1 |
| v0 | 0 | 1 |
| v1 | 1 | 0 |
| v2 | 1 | 0 |
| v3 | 1 | 0 |
| v4 | 1 | 0 |
| v5 | 1 | 0 |
| v6 | 0 | 0 |
| v7 | 0 | 0 |

The left null space has one pool: the conservation of the enzyme that is found in the active form \((x\_6)\), in the intermediary complex \((x\_2)\) and in the inhibited forms \((x\_7,\ x\_8)\) when it is bound to the inhibitor \((x\_5)\).

```
[34]:
```

```
# Obtain left nullspace
lns = left_nullspace(dimer.S, rtol=1e-10)
# Iterate through left nullspace and divide by the smallest value in each row.
for i, row in enumerate(lns):
    minval = np.min(row[np.nonzero(row)])
    lns[i] = np.array(row/minval)
    # Ensure the left nullspace is composed of only integers
    lns[i] = np.array([round(value) for value in lns[i]])

#Create a pandas.DataFrame to represent the left nullspace
pd.DataFrame(lns, columns=dimer.metabolites,
             index=["Total Enzyme"], dtype=np.int64)
```

```
[34]:
```

|  | x1 | x2 | x3 | x4 | x5 | x6 | x7 | x8 |
| --- | --- | --- | --- | --- | --- | --- | --- | --- |
| Total Enzyme | 0 | 1 | 0 | 0 | 0 | 1 | 1 | 1 |

###### Steady state: dimer model¶

We now evaluate the steady state by simulating the system to very long times.

```
[35]:
```

```
# Following Figure 9.11, adjust the parameters for v6
# and use them to set the parameters for v7.
rxns = dimer.reactions
rxns.v6.kf = 10/1
rxns.v7.kf = 10/2

rxns.v6.kr = 1*1
rxns.v7.kr = 1*2

# Define initial conditions
dimer.update_initial_conditions(
    dict((met, 0) if met.id != "x6"
         else (met, 1) for met in dimer.metabolites))
```

We simulate the model to obtain the concentration and flux solutions, then we plot the time profile of the concentrations and the fluxes.

```
[36]:
```

```
(t0, tf) = (0, 1e4)
sim_dimer = Simulation(dimer, verbose=True)
conc_sol, flux_sol = sim_dimer.simulate(
    dimer, time=(t0, tf, tf*10 + 1),
    interpolate=True, verbose=True)

# Place models and simulations into lists for later
models += [dimer]
simulations += [sim_dimer]
```

```
WARNING: No compartments found in model. Therefore creating compartment 'compartment' for entire model.
```

```
Successfully loaded MassModel 'Dimer' into RoadRunner.
Getting time points
Setting output selections
Setting simulation values for 'Dimer'
Simulating 'Dimer'
Simulation for 'Dimer' successful
Adding 'Dimer' simulation solutions to output
Updating stored solutions
```

```
[37]:
```

```
fig, axes = plt.subplots(nrows=2, ncols=1, figsize=(8, 5))
(ax1, ax2) = axes.flatten()

plot_time_profile(
    conc_sol, ax=ax1, legend="right outside",
    plot_function="semilogx",
    xlabel="Time [hr]", ylabel="Concentrations [mM]",
    title=("Concentrations", XL_FONT));

plot_time_profile(
    flux_sol, ax=ax2, legend="right outside",
    plot_function="semilogx",
    xlabel="Time [hr]", ylabel="Flux [mM/hr]",
    title=("Fluxes", XL_FONT));
fig.tight_layout()
```

We note that in the eventual steady state that; i) all the fluxes in the biosynthetic pathway are equal, ii) that the overall flux balance on inputs and outputs, \(b\_1 = v\_0 + v\_5\), holds, and iii) the inhibitor binding reactions, \(v\_6\) and \(v\_7\), go to equilibrium, i.e. \(v\_6 = v\_7 = 0\).

```
[38]:
```

```
time_points = [1e0, 1e1, 1e2, 1e3]
# Make a pandas DataFrame using a dictionary and generators
pd.DataFrame({rxn: [round(value, 3) for value in flux_func(time_points)]
              for rxn, flux_func in flux_sol.items()},
             index=["t=%i" % t for t in time_points])
```

```
[38]:
```

|  | b1 | v0 | v1 | v2 | v3 | v4 | v5 | v6 | v7 |
| --- | --- | --- | --- | --- | --- | --- | --- | --- | --- |
| t=1 | 0.1 | 0.026 | 0.051 | 0.023 | 0.007 | 0.002 | 0.000 | 0.001 | 0.000 |
| t=10 | 0.1 | 0.040 | 0.058 | 0.059 | 0.060 | 0.060 | 0.032 | 0.022 | 0.003 |
| t=100 | 0.1 | 0.046 | 0.054 | 0.054 | 0.054 | 0.054 | 0.054 | 0.000 | 0.000 |
| t=1000 | 0.1 | 0.046 | 0.054 | 0.054 | 0.054 | 0.054 | 0.054 | 0.000 | 0.000 |

**A numerical QC/QA**: Check the size of the enzyme pool at various time points.

```
[39]:
```

```
conc_sol.make_aggregate_solution(
    "Total_Enzyme", equation="x2 + x6 + x7 + x8",
    variables=["x2", "x6", "x7", "x8"]);

pd.DataFrame({
    "Total Enzyme": conc_sol["Total_Enzyme"](time_points)},
    index=["t=%i" % t for t in time_points]).T
```

```
[39]:
```

|  | t=1 | t=10 | t=100 | t=1000 |
| --- | --- | --- | --- | --- |
| Total Enzyme | 1.0 | 1.0 | 1.0 | 1.0 |

```
[40]:
```

```
dimer_ss = sim_dimer.find_steady_state(
    dimer, strategy="simulate", update_values=True,
    verbose=True)
```

```
WARNING: No compartments found in model. Therefore creating compartment 'compartment' for entire model.
```

```
Setting output selections
Setting simulation values for 'Dimer'
Setting output selections
Getting time points
Simulating 'Dimer'
Found steady state for 'Dimer'.
Updating 'Dimer' values
Adding 'Dimer' simulation solutions to output
```

###### Dynamic states: dimer model¶

We now simulate the dimer model’s response to a 10-fold change in the input flux \(b\_1\) and compare it to the previous models in order to analyze the effectiveness of the different regulatory mechanisms.

###### Simulate the response to a step change in the input \(b\_1\): dimer model¶

The simulation results can be displayed as time profiles of the concentrations and fluxes, providing an initial visualization of the solution.

```
[41]:
```

```
scalar = 10
perturbation_dict = {"kf_b1": "kf_b1 * {0}".format(scalar)}

t0, tf = (0, 50)
conc_sol, flux_sol = sim_dimer.simulate(
    dimer, time=(t0, tf, tf*10 + 1),
    perturbations=perturbation_dict)
```

```
[42]:
```

```
fig_9_13, axes = plt.subplots(nrows=3, ncols=1, figsize=(8, 6), )
(ax1, ax2, ax3) = axes.flatten()

plot_time_profile(
    conc_sol, ax=ax1, legend="right outside",
    xlabel="Time [hr]", ylabel="Concentrations [mM]",
    title=("(a) Concentrations", XL_FONT));

plot_time_profile(
    flux_sol, ax=ax2, legend="right outside",
    xlabel="Time [hr]", ylabel="Flux [mM/hr]",
    title=("(b) Fluxes", XL_FONT));

plot_time_profile(
    flux_sol, observable=["v1", "v5"], ax=ax3,
    legend="right outside",
    xlabel="Time [hr]", ylabel="Flux [mM/hr]",
    title=("(c) Responses of v1 and v5", XL_FONT),
    color="black", linestyle=["-", "--"]);
fig_9_13.tight_layout()
```

**Figure 9.13:** Simulation of the dimeric system where the influx of \(b\_1\) is varied. (a) The concentration profiles. (b) The flux profiles. (c) Comparison of the flux into \((v\_1)\) and out of \((v\_5)\) the reaction sequence.

Compare the performance of the unregulated, feedback, and dimer systems to reject the disturbance in the input flux.

```
[43]:
```

```
fig_9_14, axes = plt.subplots(nrows=1, ncols=2, figsize=(8, 4))
(ax1, ax2) = axes.flatten()

for model, sim in zip(models, simulations):
    conc_sol, flux_sol = sim.simulate(
        model, time=(t0, tf, tf*10 + 1),
        perturbations=perturbation_dict)

    # Plot v1 vs. v5
    plot_phase_portrait(
        flux_sol, x="v1", y="v5", ax=ax1,
        xlim=(0, 0.6), ylim=(0, 0.6),
        xlabel="v1", ylabel="v5",
        title=("(a) v1 vs. v5", XL_FONT),
        annotate_time_points="endpoints",
        annotate_time_points_legend="best");

    # Plot v0 vs. v5
    plot_phase_portrait(
        flux_sol, x="v0", y="v5", ax=ax2,
        legend=([model.id], "right outside"),
        xlim=(0, 1), ylim=(0, 1),
        xlabel="v0", ylabel="v5",
        title=("(a) v0 vs. v5", XL_FONT),
        annotate_time_points="endpoints",
        annotate_time_points_legend="best");

# Annotate steady state line on first plot
ax1.plot([0, 1], [0, 1], color="grey", linestyle=":");

# Annotate flux balance lines on second plot
ax2.plot([0, .1], [.1, 0], color="grey", linestyle=":");
ax2.plot([0, .1*scalar], [.1*scalar, 0], color="grey", linestyle=":");
fig_9_14.tight_layout()
```

**Figure 9.14:** Dynamic phase portraits for (a) the flux into \((v\_1)\) and out of \((v\_5)\) in the biosynthetic pathway and (b) the flux out of the primary pathway \((v\_0)\) and out of the biosynthetic pathway \((v\_5)\).The model is the simple feedback loop for the same conditions as in Figure 9.9 except for the dimeric form of the inhibition mechanism. The red points are at the start time and the blue points are at the final time.

```
[44]:
```

```
enzyme_pools = {
    "Free": ["x2 + x6", ["x2", "x6"]],
    "Monomer_Inhibited": ["x7", ["x7"]],
    "Dimer_Inhibited": ["x7 + x8", ["x7", "x8"]]}

colors = ["red", "blue"]

fig_9_15, ax = plt.subplots(nrows=1, ncols=1, figsize=(6.5, 5))

for i, (model, sim) in enumerate(zip(models[1:], simulations[1:])):
    conc_sol, flux_sol = sim.simulate(
        model, time=(t0, tf, tf*10 + 1),
        perturbations=perturbation_dict)

    for pool_id, args in enzyme_pools.items():
        if pool_id != "Free" and model.id not in pool_id:
            continue
        equation, variables = args
        conc_sol.make_aggregate_solution(
            pool_id, equation, variables)

    plot_phase_portrait(
        conc_sol, x="Free", y=model.id + "_Inhibited", ax=ax,
        legend=([model.id], "right outside"),
        xlim=(0.3, .7), ylim=(0.3, .7),
        xlabel="Free Enzyme", ylabel="Inhibited Enzyme",
        color=colors[i], linestyle=["--"],
        title=("Free vs. Inhibited Enzyme", XL_FONT),
        annotate_time_points="endpoints",
        annotate_time_points_color=colors[i],
        annotate_time_points_labels=True)

ax.plot([0, 1], [1, 0], color="grey", linestyle=":");
fig_9_15.tight_layout()
```

**Figure 9.15:** Dynamic phase portrait of the inhibited enzyme vs. the free enzyme for the monomer and the dimer.

###### Pool sizes and their states: dimer model¶

The fraction of the enzyme that is in the inhibited form can be computed for the dimer:

\[\begin{equation} f\_{inhibited} = \frac{x\_7 + x\_8}{x\_2 + x\_6 + x\_7 + x\_8} \tag{9.5} \end{equation}\]

Therefore the fraction of free enzyme can be computed for the dimer:

\[\begin{equation} f\_{free} = \frac{x\_2 + x\_6}{x\_2 + x\_6 + x\_7 + x\_8} \tag{9.6} \end{equation}\]

The first fraction is the ratio of the occupied sites to the total number of binding sites for \(x\_5\), while the second fraction is the ratio of free binding sites to the total number of binding sites for \(x\_5\). The increased number of binding sites for \(x\_5\) on the enzyme \((x\_6)\) increases the efficacy of the regulatory mechanisms on the ability of the system to reject the disturbance imposed on it.

```
[45]:
```

```
fig_9_16, axes = plt.subplots(nrows=1, ncols=2, figsize=(14, 4),
                              )
(ax1, ax2) = axes.flatten()

for i, (model, sim) in enumerate(zip(models[1:], simulations[1:])):
    conc_sol = sim.concentration_solutions.get_by_id(
        "_".join((model.id, "ConcSols")))
    inhibited_key = model.id + "_Inhibited"
    # Make Fraction of Free Enzyme pool
    conc_sol.make_aggregate_solution(
        "Fraction_Free", equation="{0} / ({0} + {1})".format(
            "Free", inhibited_key),
        variables=["Free", inhibited_key])
    # Make Fraction of Inhibited Enzyme pool
    conc_sol.make_aggregate_solution(
        "Fraction_Inhibited", equation="{1} / ({0} + {1})".format(
            "Free", inhibited_key),
        variables=["Free", inhibited_key])

    plot_time_profile(
        conc_sol, observable="Fraction_Inhibited", ax=ax1,
        ylim=(0.3, .75), xlabel="Time [hr]", ylabel="Fraction",
        title=("(a) Fraction of Inhibited Enzyme", XL_FONT),
        color=colors[i])

    plot_time_profile(
        conc_sol, observable="Fraction_Free", ax=ax2,
        legend=(model.id, "right outside"),
        ylim=(0.3, .75), xlabel="Time [hr]", ylabel="Fraction",
        title=("(b) Fraction of Free Enzyme", XL_FONT),
        color=colors[i])
fig_9_16.tight_layout()
```

**Figure 9.16:** (a) Fraction of inhibited enzyme and (b) fraction of free enzyme for the monomer and dimer models.

###### Breaking down the steady states into the pathway vectors: dimer model¶

We can study the steady state solutions by breaking them down into a linear combination of the two pathway vectors in the null space.

```
[46]:
```

```
dimer_ss_perturbed = sim.find_steady_state(
    dimer, strategy="simulate", perturbations=perturbation_dict)

unperturbed = [round(value,3) for value in dimer_ss[1].values()]
perturbed = [round(value,3) for value in dimer_ss_perturbed[1].values()]

pd.DataFrame(
    np.vstack((ns.T, unperturbed, perturbed)),
    index=["Nullspace Path 1", "Nullspace Path 2", "Unperturbed fluxes", "Perturbed fluxes"],
    columns=[rxn.id for rxn in dimer.reactions],
    dtype=np.float64)
```

```
[46]:
```

|  | b1 | v0 | v1 | v2 | v3 | v4 | v5 | v6 | v7 |
| --- | --- | --- | --- | --- | --- | --- | --- | --- | --- |
| Nullspace Path 1 | 1.0 | 0.000 | 1.000 | 1.000 | 1.000 | 1.000 | 1.000 | 0.0 | 0.0 |
| Nullspace Path 2 | 1.0 | 1.000 | 0.000 | 0.000 | 0.000 | 0.000 | 0.000 | 0.0 | 0.0 |
| Unperturbed fluxes | 0.1 | 0.046 | 0.054 | 0.054 | 0.054 | 0.054 | 0.054 | 0.0 | 0.0 |
| Perturbed fluxes | 1.0 | 0.760 | 0.240 | 0.240 | 0.240 | 0.240 | 0.240 | 0.0 | 0.0 |

The overall flux balance is \(b\_1 = v\_0 + v\_5\). Thus, the first pathway goes from 45% of \(b\_1\) to 73% of \(b\_1\) after the perturbation and the second pathway goes from a 55% of \(b\_1\) to 27.2% of \(b\_1\) after the perturbation. So the dimer is more effective in preventing flux from going down the biosynthetic pathway as compared to the monomer. Still, a fair number of the disturbance does go down the biosynthetic pathway so the regulation does not reject the entire
disturbance.

**A numerical QC/QA:** The loadings on \(v\_0\) and \(v\_5\) should add up to \(b\_1\):

```
[47]:
```

```
before = dimer_ss[1]["v0"] +\
         dimer_ss[1]["v5"] == dimer_ss[1]["b1"]
after = dimer_ss_perturbed[1]["v0"] +\
        dimer_ss_perturbed[1]["v5"] == dimer_ss_perturbed[1]["b1"]

pd.DataFrame([before, after],
             index=["Before Perturbation", "After Perturbation"],
             columns=["Add up to b1?"])
```

```
[47]:
```

|  | Add up to b1? |
| --- | --- |
| Before Perturbation | True |
| After Perturbation | True |

##### Tetramer Model of the Regulated Enzyme¶

Expanding on the mechanism in symmetry model, described in Section 5.5, a the reaction mechanisms (using the same compound names as in Section 9.3) for a tetramer are shown below.

**Figure 9.17:** The reaction mechanism for a tetramer.

###### Define tetramer model¶

We will now examine the tetramer symmetry model by adding all additional binding reactions and adjust the corresponding rate equations for the effective concentrations of the multiple binding sites.

**Figure 9.18:** Visual representation of a prototypical negative feedback loop for a biosynthetic pathway for a tetramer model. The additional tetramer reactions are shown in light blue.

```
[48]:
```

```
# Copy the dimer model
tetramer = dimer.copy()
# Change the model ID
tetramer.id = "Tetramer"

# Define new metabolites
x9 = MassMetabolite("x9")
x10 = MassMetabolite("x10")
mets = tetramer.metabolites

# Define new reaction and set parameters
v8 = MassReaction("v8");
v8.add_metabolites({mets.x5: -1, mets.x8: -1, x9:1})
v8.get_mass_action_rate(rate_type=2, update_reaction=True)

v9 = MassReaction("v9");
v9.add_metabolites({mets.x5: -1, x9: -1, x10:1})
v9.get_mass_action_rate(rate_type=2, update_reaction=True)

# Add Reactions to the model
tetramer.add_reactions([v8, v9])
```

###### Null spaces and their content: tetramer model¶

When we look at the right null space, we observe that the additional inhibition steps did not change the pathway vectors.

```
[49]:
```

```
# Obtain nullspace
ns = nullspace(tetramer.S, rtol=1e-10)
# Transpose and iterate through nullspace,
# dividing by the smallest value in each row.
ns = ns.T
for i, row in enumerate(ns):
    minval = np.min(abs(row[np.nonzero(row)]))
    new_row = np.array(row/minval)
    # Round to ensure the nullspace is composed of only integers
    ns[i] = np.array([round(value) for value in new_row])

ns[1] = (ns[0] + 2*ns[1])/7
ns[0] = (ns[0] - ns[1])/2

# Ensure positive stoichiometric coefficients if all are negative
for i, space in enumerate(ns):
    ns[i] = np.negative(space) if all([num <= 0 for num in space]) else space

# Revert transpose
ns = ns.T
# Create a pandas.DataFrame to represent the nullspace
pd.DataFrame(ns, index=[rxn.id for rxn in tetramer.reactions],
             columns=["Path 1", "Path 2"], dtype=np.int64)
```

```
[49]:
```

|  | Path 1 | Path 2 |
| --- | --- | --- |
| b1 | 1 | 1 |
| v0 | 0 | 1 |
| v1 | 1 | 0 |
| v2 | 1 | 0 |
| v3 | 1 | 0 |
| v4 | 1 | 0 |
| v5 | 1 | 0 |
| v6 | 0 | 0 |
| v7 | 0 | 0 |
| v8 | 0 | 0 |
| v9 | 0 | 0 |

The left null space has one pool: the conservation of the enzyme that is found in the active form \((x\_6)\), in the intermediary complex \((x\_2)\) and in the inhibited forms \((x\_7,\ x\_8,\ x\_9,\ x\_{10})\) when it is bound to the inhibitor \((x\_5)\).

```
[50]:
```

```
# Obtain left nullspace
lns = left_nullspace(tetramer.S, rtol=1e-10)
# Iterate through left nullspace and divide by the smallest value in each row.
for i, row in enumerate(lns):
    minval = np.min(row[np.nonzero(row)])
    lns[i] = np.array(row/minval)
    # Ensure the left nullspace is composed of only integers
    lns[i] = np.array([round(value) for value in lns[i]])

#Create a pandas.DataFrame to represent the left nullspace
pd.DataFrame(lns, columns=tetramer.metabolites,
             index=["Total Enzyme"], dtype=np.int64)
```

```
[50]:
```

|  | x1 | x2 | x3 | x4 | x5 | x6 | x7 | x8 | x9 | x10 |
| --- | --- | --- | --- | --- | --- | --- | --- | --- | --- | --- |
| Total Enzyme | 0 | 1 | 0 | 0 | 0 | 1 | 1 | 1 | 1 | 1 |

###### Steady state: tetramer model¶

We now evaluate the steady state by simulating the system to very long times.

```
[51]:
```

```
# Following Figure 9.11, adjust the parameters for v6
# and use them to set the parameters for v7.
rxns = tetramer.reactions
rxns.v6.kf = 10/1
rxns.v7.kf = 10/2
rxns.v8.kf = 10/3
rxns.v9.kf = 10/4

rxns.v6.kr = 1*1
rxns.v7.kr = 1*2
rxns.v8.kr = 1*3
rxns.v9.kr = 1*4

# Define initial conditions
tetramer.update_initial_conditions(
    dict((met, 0) if met.id != "x6"
         else (met, 1) for met in tetramer.metabolites))
```

We simulate the model to obtain the concentration and flux solutions, then we plot the time profile of the concentrations and the fluxes.

```
[52]:
```

```
(t0, tf) = (0, 1e4)
sim_tetramer = Simulation(tetramer, verbose=True)
conc_sol, flux_sol = sim_tetramer.simulate(
    tetramer, time=(t0, tf, tf*10 + 1),
    interpolate=True, verbose=True)

# Place models and simulations into lists for later
models += [tetramer]
simulations += [sim_tetramer]
```

```
WARNING: No compartments found in model. Therefore creating compartment 'compartment' for entire model.
```

```
Successfully loaded MassModel 'Tetramer' into RoadRunner.
Getting time points
Setting output selections
Setting simulation values for 'Tetramer'
Simulating 'Tetramer'
Simulation for 'Tetramer' successful
Adding 'Tetramer' simulation solutions to output
Updating stored solutions
```

```
[53]:
```

```
fig, axes = plt.subplots(nrows=2, ncols=1, figsize=(8, 5))
(ax1, ax2) = axes.flatten()

plot_time_profile(
    conc_sol, ax=ax1, legend="right outside",
    plot_function="semilogx",
    xlabel="Time [hr]", ylabel="Concentrations [mM]",
    title=("Concentrations", XL_FONT));

plot_time_profile(
    flux_sol, ax=ax2, legend="right outside",
    plot_function="semilogx",
    xlabel="Time [hr]", ylabel="Flux [mM/hr]",
    title=("Fluxes", XL_FONT));
fig.tight_layout()
```

We note that in the eventual steady state that; i) all the fluxes in the biosynthetic pathway are equal, ii) that the overall flux balance on inputs and outputs, \(b\_1 = v\_0 + v\_5\), holds, and iii) the inhibitor binding reactions, \(v\_6,\ v\_7,\ v\_8,\) and \(v\_9\), go to equilibrium, i.e. \(v\_6 = v\_7 = v\_8 = v\_9 = 0\).

```
[54]:
```

```
time_points = [1e0, 1e1, 1e2, 1e3]
# Make a pandas DataFrame using a dictionary and generators
pd.DataFrame({rxn: [round(value, 3) for value in flux_func(time_points)]
              for rxn, flux_func in flux_sol.items()},
             index=["t=%i" % t for t in time_points])
```

```
[54]:
```

|  | b1 | v0 | v1 | v2 | v3 | v4 | v5 | v6 | v7 | v8 | v9 |
| --- | --- | --- | --- | --- | --- | --- | --- | --- | --- | --- | --- |
| t=1 | 0.1 | 0.026 | 0.051 | 0.023 | 0.007 | 0.002 | 0.000 | 0.001 | 0.000 | 0.0 | 0.0 |
| t=10 | 0.1 | 0.040 | 0.058 | 0.059 | 0.060 | 0.060 | 0.032 | 0.022 | 0.003 | 0.0 | 0.0 |
| t=100 | 0.1 | 0.046 | 0.054 | 0.054 | 0.054 | 0.054 | 0.054 | 0.000 | 0.000 | 0.0 | 0.0 |
| t=1000 | 0.1 | 0.046 | 0.054 | 0.054 | 0.054 | 0.054 | 0.054 | 0.000 | 0.000 | -0.0 | -0.0 |

**A numerical QC/QA**: Check the size of the enzyme pool at various time points.

```
[55]:
```

```
conc_sol.make_aggregate_solution(
    "Total_Enzyme", equation="x2 + x6 + x7 + x8 + x9 + x10",
    variables=["x2", "x6", "x7", "x8", "x9", "x10"]);

pd.DataFrame({
    "Total Enzyme": conc_sol["Total_Enzyme"](time_points)},
    index=["t=%i" % t for t in time_points]).T
```

```
[55]:
```

|  | t=1 | t=10 | t=100 | t=1000 |
| --- | --- | --- | --- | --- |
| Total Enzyme | 1.0 | 1.0 | 1.0 | 1.0 |

```
[56]:
```

```
tetramer_ss = sim_tetramer.find_steady_state(
    tetramer, strategy="simulate", update_values=True,
    verbose=True)
```

```
WARNING: No compartments found in model. Therefore creating compartment 'compartment' for entire model.
```

```
Setting output selections
Setting simulation values for 'Tetramer'
Setting output selections
Getting time points
Simulating 'Tetramer'
Found steady state for 'Tetramer'.
Updating 'Tetramer' values
Adding 'Tetramer' simulation solutions to output
```

###### Dynamic states: tetramer model¶

We now simulate the tetramers model’s response to a 10-fold change in the input flux \(b\_1\) and compare it to the previous models in order to analyze the effectiveness of the different regulatory mechanisms.

###### Simulate the response to a step change in the input \(b\_1\) by 10-fold: tetramer model¶

The simulation results can be displayed as time profiles of the concentrations and fluxes, providing an initial visualization of the solution.

```
[57]:
```

```
scalar = 10
perturbation_dict = {"kf_b1": "kf_b1 * {0}".format(scalar)}

t0, tf = (0, 50)
conc_sol, flux_sol = sim_tetramer.simulate(
    tetramer, time=(t0, tf, tf*10 + 1),
    perturbations=perturbation_dict)
```

```
[58]:
```

```
fig_9_19, axes = plt.subplots(nrows=3, ncols=1, figsize=(8, 8))
(ax1, ax2, ax3) = axes.flatten()

plot_time_profile(
    conc_sol, ax=ax1, legend="right outside",
    xlabel="Time [hr]", ylabel="Concentrations [mM]",
    title=("(a) Concentrations", XL_FONT));

plot_time_profile(
    flux_sol, ax=ax2, legend="right outside",
    xlabel="Time [hr]", ylabel="Flux [mM/hr]",
    title=("(b) Fluxes", XL_FONT));

plot_time_profile(
    flux_sol, observable=["v1", "v5"], ax=ax3,
    legend="right outside",
    xlabel="Time [hr]", ylabel="Flux [mM/hr]",
    title=("(c) Responses of v1 and v5", XL_FONT),
    color="black", linestyle=["-", "--"]);
fig_9_19.tight_layout()
```

**Figure 9.19:** Simulation of the tetrameric system where the influx of \(b\_1\) is varied. (a) The concentration profiles. (b) The flux profiles. (c) Comparison of the flux into \((v\_1)\) and out of \((v\_5)\) the reaction sequence.

Compare the performance of the unregulated, monomer, dimer, and tetramer systems to reject the disturbance in the input flux.

```
[59]:
```

```
fig_9_20, axes = plt.subplots(nrows=1, ncols=2, figsize=(8, 4))
(ax1, ax2) = axes.flatten()

for model, sim in zip(models, simulations):
    conc_sol, flux_sol = sim.simulate(
        model, time=(t0, tf, tf*10 + 1),
        perturbations=perturbation_dict)

    # Plot v1 vs. v5
    plot_phase_portrait(
        flux_sol, x="v1", y="v5", ax=ax1,
        xlim=(0, 0.6), ylim=(0, 0.6),
        xlabel="v1", ylabel="v5",
        title=("(a) v1 vs. v5", XL_FONT),
        annotate_time_points="endpoints",
        annotate_time_points_legend="best");

    # Plot v0 vs. v5
    plot_phase_portrait(
        flux_sol, x="v0", y="v5", ax=ax2,
        legend=([model.id], "right outside"),
        xlim=(0, 1), ylim=(0, 1),
        xlabel="v0", ylabel="v5",
        title=("(a) v0 vs. v5", XL_FONT),
        annotate_time_points="endpoints",
        annotate_time_points_legend="best");

# Annotate steady state line on first plot
ax1.plot([0, 1], [0, 1], color="grey", linestyle=":");

# Annotate flux balance lines on second plot
ax2.plot([0, .1], [.1, 0], color="grey", linestyle=":");
ax2.plot([0, .1*scalar], [.1*scalar, 0], color="grey", linestyle=":");
fig_9_20.tight_layout()
```

**Figure 9.20:** Dynamic phase portraits for (a) the flux into \((v\_1)\) and out of \((v\_5)\) in the biosynthetic pathway and (b) the flux out of the primary pathway \((v\_0)\) and out of the biosynthetic pathway \((v\_5)\).The model is the simple feedback loop for the same conditions as in Figure 9.9 except for the dimeric and tetrameric forms of the inhibition mechanism. The red points are at the start time and the blue points are at the final time.

```
[60]:
```

```
enzyme_pools = {
    "Free": ["x2 + x6", ["x2", "x6"]],
    "Monomer_Inhibited": ["x7", ["x7"]],
    "Dimer_Inhibited": ["x7 + x8", ["x7", "x8"]],
    "Tetramer_Inhibited": ["x7 + x8 + x9 + x10", ["x7", "x8", "x9", "x10"]]}

colors = ["red", "blue", "green"]

fig_9_21, ax = plt.subplots(nrows=1, ncols=1, figsize=(6.5, 5))

for i, (model, sim) in enumerate(zip(models[1:], simulations[1:])):
    conc_sol, flux_sol = sim.simulate(
        model, time=(t0, tf, tf*10 + 1),
        perturbations=perturbation_dict)

    for pool_id, args in enzyme_pools.items():
        if pool_id != "Free" and model.id not in pool_id:
            continue
        equation, variables = args
        conc_sol.make_aggregate_solution(
            pool_id, equation, variables)

    plot_phase_portrait(
        conc_sol, x="Free", y=model.id + "_Inhibited", ax=ax,
        legend=([model.id], "right outside"),
        xlim=(0.3, .7), ylim=(0.3, .7),
        xlabel="Free Enzyme", ylabel="Inhibited Enzyme",
        color=colors[i], linestyle=["--"],
        title=("Free vs. Inhibited Enzyme", XL_FONT),
        annotate_time_points="endpoints",
        annotate_time_points_color=colors[i],
        annotate_time_points_labels=True)

ax.plot([0, 1], [1, 0], color="grey", linestyle=":");
fig_9_21.tight_layout()
```

**Figure 9.21:** Dynamic phase portrait of the inhibited enzyme vs. the free enzyme for the monomer, the dimer, and the tetramer.

###### Pool sizes and their states: tetramer model¶

The fraction of the enzyme that is in the inhibited form can be computed for the dimer:

\[\begin{equation} f\_{inhibited} = \frac{x\_7 + x\_8 + x\_9 + x\_{10}}{x\_2 + x\_6 + x\_7 + x\_8 + x\_9 + x\_{10}} \tag{9.7} \end{equation}\]

Therefore the fraction of free enzyme can be computed for the dimer:

\[\begin{equation} f\_{free} = \frac{x\_2 + x\_6}{x\_2 + x\_6 + x\_7 + x\_8 + x\_9 + x\_{10}} \tag{9.8} \end{equation}\]

The first fraction is the ratio of the occupied sites to the total number of binding sites for \(x\_5\), while the second fraction is the ratio of free binding sites to the total number of binding sites for \(x\_5\). The increased number of binding sites for \(x\_5\) on the enzyme \((x\_6)\) increases the efficacy of the regulatory mechanisms on the ability of the system to reject the disturbance imposed on it.

```
[61]:
```

```
fig_9_22, axes = plt.subplots(nrows=1, ncols=2, figsize=(14, 4))
(ax1, ax2) = axes.flatten()

for i, (model, sim) in enumerate(zip(models[1:], simulations[1:])):
    conc_sol = sim.concentration_solutions.get_by_id(
        "_".join((model.id, "ConcSols")))
    inhibited_key = model.id + "_Inhibited"
    # Make Fraction of Free Enzyme pool
    conc_sol.make_aggregate_solution(
        "Fraction_Free", equation="{0} / ({0} + {1})".format(
            "Free", inhibited_key),
        variables=["Free", inhibited_key])
    # Make Fraction of Inhibited Enzyme pool
    conc_sol.make_aggregate_solution(
        "Fraction_Inhibited", equation="{1} / ({0} + {1})".format(
            "Free", inhibited_key),
        variables=["Free", inhibited_key])

    plot_time_profile(
        conc_sol, observable="Fraction_Inhibited", ax=ax1,
        ylim=(0.3, .75), xlabel="Time [hr]", ylabel="Fraction",
        title=("(a) Fraction of Inhibited Enzyme", XL_FONT),
        color=colors[i])

    plot_time_profile(
        conc_sol, observable="Fraction_Free", ax=ax2,
        legend=(model.id, "right outside"),
        ylim=(0.3, .75), xlabel="Time [hr]", ylabel="Fraction",
        title=("(b) Fraction of Free Enzyme", XL_FONT),
        color=colors[i])
fig_9_22.tight_layout()
```

**Figure 9.22:** Fraction of free enzyme and fraction of inhibited enzyme over time for the monomer, dimer, and tetramer models.

###### Breaking down the steady states into the pathway vectors: tetramer model¶

We can study the steady state solutions by breaking them down into a linear combination of the two pathway vectors in the null space.

```
[62]:
```

```
tetramer_ss_perturbed = sim.find_steady_state(
    tetramer, strategy="simulate", perturbations=perturbation_dict)

unperturbed = [round(value,3) for value in tetramer_ss[1].values()]
perturbed = [round(value,3) for value in tetramer_ss_perturbed[1].values()]

pd.DataFrame(
    np.vstack((ns.T, unperturbed, perturbed)),
    index=["Nullspace Path 1", "Nullspace Path 2", "Unperturbed fluxes", "Perturbed fluxes"],
    columns=[rxn.id for rxn in tetramer.reactions],
    dtype=np.float64)
```

```
[62]:
```

|  | b1 | v0 | v1 | v2 | v3 | v4 | v5 | v6 | v7 | v8 | v9 |
| --- | --- | --- | --- | --- | --- | --- | --- | --- | --- | --- | --- |
| Nullspace Path 1 | 1.0 | 0.000 | 1.000 | 1.000 | 1.000 | 1.000 | 1.000 | 0.0 | 0.0 | 0.0 | 0.0 |
| Nullspace Path 2 | 1.0 | 1.000 | 0.000 | 0.000 | 0.000 | 0.000 | 0.000 | 0.0 | 0.0 | 0.0 | 0.0 |
| Unperturbed fluxes | 0.1 | 0.046 | 0.054 | 0.054 | 0.054 | 0.054 | 0.054 | 0.0 | 0.0 | -0.0 | 0.0 |
| Perturbed fluxes | 1.0 | 0.768 | 0.232 | 0.232 | 0.232 | 0.232 | 0.232 | 0.0 | 0.0 | 0.0 | -0.0 |

The overall flux balance is \(b\_1 = v\_0 + v\_5\). Thus, the first pathway goes from 48% of \(b\_1\) to 81% of \(b\_1\) after the perturbation and the second pathway goes from a 52% of \(b\_1\) to 19% of \(b\_1\) after the perturbation. So the tetramer is more effective in preventing flux from going down the biosynthetic pathway as compared to the monomer and the dimer.

**A numerical QC/QA:** The loadings on \(v\_0\) and \(v\_5\) should add up to \(b\_1\):

```
[63]:
```

```
before = tetramer_ss[1]["v0"] +\
         tetramer_ss[1]["v5"] == tetramer_ss[1]["b1"]
after = tetramer_ss_perturbed[1]["v0"] +\
        tetramer_ss_perturbed[1]["v5"] == tetramer_ss_perturbed[1]["b1"]

pd.DataFrame([before, after],
             index=["Before Perturbation", "After Perturbation"],
             columns=["Add up to b1?"])
```

```
[63]:
```

|  | Add up to b1? |
| --- | --- |
| Before Perturbation | True |
| After Perturbation | True |

##### Regulated Protein Synthesis of the Regulatory Enzyme¶

In this section, we will look at the effects of regulating protein synthesis on the basic Monomer model. We will do this by comparing the Monomer model without the protein synthesis and degradation reactions to the monomer model with the synthesis and degradation reactions. We will then add the synthesis and degradation reactions to the unregulated, dimeric and tetrameric models and perform dynamic simulations to examine the differences in disturbance rejection among the models.

###### Regulation of Protein Synthesis¶

In the feedback loop, \(x\_5\) feedback inhibits its own synthesis by transforming the first enzyme in the reaction chain into an inactive form. In many organisms, end products like \(x\_5\) can also feedback regulate the synthesis rate of the enzyme itself and thus regulate the total amount of enzyme present. Thus, \(e\_t\) is no longer a constant but a dynamic variable. This regulation of protein synthesis can be simulated by adding a synthesis and degradation rate in the dynamic
equation for the enzyme, \(x\_6\), where we can use an inhibition rate of the Hill form with \(\nu = 1:\)

\[\begin{equation} v\_{10} = \frac{k\_{10}}{1 + K\_{10}x\_5} \tag{9.8} \end{equation}\]

and a first order turnover

\[\begin{equation} v\_{11} = k\_{11}x\_6 \tag{9.9} \end{equation}\]

for the enzyme. Note that these equations assume that the inhibited form, \(x\_7\), is stable.

###### Updating the model¶

Define synthesis and degradation reactions for \(x\_6\) and add them to the model (resulting in a new model). Define a Hill type inhibition rate for the synthesis reaction (no need to define a custom rate equation for the degradation reaction). The final product \((x\_5)\) acts hereby as the inhibitor. A parameter set is chosen that preserves the steady initial state of the initial model. If we pick \(k\_{11} = 0.1\), we can solve for the other parameters so the network will have the
same initial state as in the initial model, and the protein turnover will have a time constant of 10 (=1/0.1). Macromolecular turnover is much slower than metabolite turnover.

Visualize the difference between the initial and the extended model:

**Figure 9.23:** Visual representation of the monomer with protein synthesis and degradation for a biosynthetic pathway. The red pathway in the model from the inclusion the of the protein synthesis and degradation reactions.

```
[64]:
```

```
monomer_w_synth = monomer.copy()
monomer_w_synth.id += "_with_Synthesis"

mets = monomer_w_synth.metabolites
# Define synthesis
v10 = MassReaction("v10")
v10.add_metabolites({mets.x6: 1})

# Define drain
v11 = MassReaction("v11", reversible=False)
v11.add_metabolites({mets.x6: -1})
v11.kf = 0.1

monomer_w_synth.add_reactions([v10, v11])
monomer_w_synth.add_custom_rate(v10, custom_rate="{0} / (1 + {1}*x5(t))".format(v10.kf_str, v10.Keq_str))
```

To get the parameters for the steady state system, we use the parameters from above and simulate to very long times to obtain the steady state concentrations. Since we will substitute these values into sympy equations in the next step, we will also ensure that the metabolites are represented as **sympy.Symbol** objects.

```
[65]:
```

```
ics = {sym.Symbol(met): ic_value
       for met, ic_value in sim_monomer.find_steady_state(
           monomer, strategy="simulate")[0].items()}
for metabolite, ic in ics.items():
    print("%s: %s" % (metabolite, ic))
```

```
x1: 0.09009804864073484
x2: 0.054950975679632585
x3: 0.054950975679632585
x4: 0.054950975679632585
x5: 0.054950975679632585
x6: 0.6099019513591144
x7: 0.33514707296095153
```

We then set the rate equations for the protein synthesis and degradation equal to each other. We also set the quantity of \(K\_{10}x\_{5}\) equal to 10 since we want a protein turnover time constant of 10, and the rate law seen in Eq. (9.9) represents the synthesis rate.

```
[66]:
```

```
# Set up symbolic equalities using sympy
eq1 = sym.Eq(10, sym.Symbol(v10.Keq_str)*sym.Symbol("x5"))
sym.pprint(eq1)

eq2 = sym.Eq(strip_time(monomer_w_synth.custom_rates[v10]),
             strip_time(strip_time(monomer_w_synth.rates[v11])))
sym.pprint(eq2)
```

```
10 = Keqᵥ₁₀⋅x₅
    kfᵥ₁₀
───────────── = kfᵥ₁₁⋅x₆
Keqᵥ₁₀⋅x₅ + 1
```

We solve the equations using the steady state conditions for \(K\_{10}\) and then for \(k\_{10}\):

```
[67]:
```

```
# Substitute ics and obtain Keq for v10
sym.pprint(eq1.subs(ics))

#Update model with new parameter value
v10.Keq = float(sym.solve(eq1.subs(ics))[0])
print("{0} = {1:.3f}\n".format(v10.Keq_str, v10.Keq))

# Susbtitute ics and Keq for v10, and kf for v11 to obtain kf for v10
parameters = {
    sym.Symbol(v10.Keq_str): v10.Keq,
    sym.Symbol(v11.kf_str): v11.kf}
sym.pprint(eq2.subs(ics).subs(parameters))

#Update model with new parameter value
v10.kf = float(sym.solve(eq2.subs(ics).subs(parameters))[0])
print("{0} = {1:.3f}\n".format(v10.kf_str, v10.kf))
```

```
10 = 0.0549509756796326⋅Keqᵥ₁₀
Keq_v10 = 181.980

0.0909090909090909⋅kfᵥ₁₀ = 0.0609901951359114
kf_v10 = 0.671
```

###### Dynamic states: protein synthesis¶

The effect of this additional process can be simulated. The stability of the enzyme \((k\_{11} = 0.10)\) introduces a slower secondary response of this loop. During this secondary response, the total enzyme concentration drops from 1.0 to a value of 0.748428, (Figure 9.24a) During this slow response, the concentration of \(x\_1\) rises due to a lower flux into the reaction chain \((v\_2)\) and the end product \((x\_5)\) drops slightly, (Figure 9.24b).

```
[68]:
```

```
scalar = 10
perturbation_dict = {"kf_b1": "kf_b1 * {0}".format(scalar)}

t0, tf = (0, 150)
sim_monomer_synth = Simulation(monomer_w_synth)

# Place models and simulations into lists for later
models_with_synth = [monomer_w_synth]
simulations_with_synth = [sim_monomer_synth]

for model, sim in zip([monomer, monomer_w_synth],
                      [sim_monomer, sim_monomer_synth]):
    conc_sol, flux_sol = sim.simulate(
        model, time=(t0, tf, tf*10 + 1),
        perturbations=perturbation_dict);

    conc_sol.make_aggregate_solution(
        "Total_Enzyme", equation="x2 + x6 + x7",
        variables=["x2", "x6", "x7"]);
```

```
WARNING: No compartments found in model. Therefore creating compartment 'compartment' for entire model.
```

```
[69]:
```

```
fig_9_24, axes = plt.subplots(nrows=2, ncols=1, figsize=(10, 4),
                              )
(ax1, ax2) = axes.flatten()

synthesis_strs = ["", " with Synthesis"]
linestyles = ["--", "-"]
colors = ["red", "blue", "green"]
observable_fluxes = [["v2"], ["v2", "v10", "v11"]]


for i, (model, sim) in enumerate(zip([monomer, monomer_w_synth],
                                     [sim_monomer, sim_monomer_synth])):
    conc_sol = sim.concentration_solutions.get_by_id(
        "_".join((model.id, "ConcSols")))
    flux_sol = sim.flux_solutions.get_by_id(
        "_".join((model.id, "FluxSols")))

    plot_time_profile(
        conc_sol, observable=["x1", "x5"], ax=ax1,
        legend=(["x1" + synthesis_strs[i], "x5" + synthesis_strs[i]],
                "right outside"),
        color=colors[:2], linestyle=linestyles[i]);

    plot_time_profile(
        conc_sol, observable=["Total_Enzyme"], ax=ax1,
        legend=(["Total Enzyme" + synthesis_strs[i]], "right outside"),
        xlabel="Time [hr]", ylabel="Concentration [mM]",
        title=("(a) Concentrations", XL_FONT),
        color=colors[2:], linestyle=linestyles[i]);

    plot_time_profile(
        flux_sol, observable=observable_fluxes[i], ax=ax2,
        legend=([entry + " " + synthesis_strs[i]
                 for entry in observable_fluxes[i]],
                "right outside"),
        xlabel="Time [hr]", ylabel="Flux [mM/hr]",
        title=("(b) Fluxes", XL_FONT),
        color=colors[:2*i + 1], linestyle=linestyles[i]);
fig_9_24.tight_layout()
```

**Figure 9.24:** The time profiles for concentrations and fluxes involved in the monomer model with feedback control of protein synthesis. The parameter values used are \(k\_{10}=0.671,\ K\_{10} =182,\) and \(k\_{11} = 0.10\). Other parameter values are as in Figure 9.8.(a) Key concentrations as a function of time for the monomer model with and without the protein synthesis. (b) Key reaction fluxes as a function of time for the monomer model with and without the protein synthesis.

###### Slowly changing pool sizes: protein synthesis¶

These changes can also be traced out using dynamic phase portraits, see Figure 9.25. Compared to the response without the regulation of protein synthesis (Figure 9.10a) the input and output rates from the reaction chain drop slowly. This motion is approximately along the 45 degree line. The phase portrait of the free and inhibited forms of the enzyme \(x\_2 + x\_6\) and \(x\_7\) initially follows the straight line \(x\_2 + x\_6 + x\_7 = X,\) as without the control of enzyme synthesis but
then has a secondary slow response where the total amount of the enzyme drops from X to about Y. Thus, the control of enzyme synthesis drops the total amount of the enzyme by about \(40\%\), leading to a lower flux through \(v\_2\).

```
[70]:
```

```
fig_9_25 = plt.figure(figsize=(14, 4))
gs = fig_9_25.add_gridspec(nrows=1, ncols=2, width_ratios=[1, 1.5])

ax1 = fig_9_25.add_subplot(gs[0, 0])
ax2 = fig_9_25.add_subplot(gs[0, 1])

# Annotate line representing constant enzyme on plots
ax1.plot([0, 1], [1, 0], color="grey", linestyle=":")
ax2.plot([t0, tf], [1, 1], color="grey", linestyle=":",
         label="Constant Enzyme Pool, No synthesis");
ax2.legend()

enzyme_pools = {
    "Free": ["x2 + x6", ["x2", "x6"]],
    "Monomer_with_Synthesis_Inhibited": ["x7", ["x7"]]}
for pool_id, args in enzyme_pools.items():
    equation, variables = args
    conc_sol.make_aggregate_solution(
        pool_id, equation, variables)

plot_phase_portrait(
    conc_sol, x="Free", y=monomer_w_synth.id + "_Inhibited", ax=ax1,
    xlim=(0.3, .7), ylim=(0.3, .7),
    xlabel="Free Enzyme", ylabel="Inhibited Enzyme",
    color=["red"], linestyle=["--"],
    title=("Free vs. Inhibited Enzyme", XL_FONT),
    annotate_time_points="endpoints",
    annotate_time_points_color=["red"],
    annotate_time_points_labels=True)

plot_time_profile(
    conc_sol, observable=["Total_Enzyme"], ax=ax2,
    legend=([monomer_w_synth.id], "right outside"),
    xlim=(t0, 50), color="red",
    title=("(b) Total Enzyme pool", XL_FONT));
fig_9_25.tight_layout()
```

**Figure 9.25:** (a) Dynamic phase portrait of the inhibited enzyme vs. the free enzyme for the monomer model with the protein synthesis reactions and (b) the conservation pool over time.

Analyze the effects of the protein synthesis reactions on the models and their ability to reject the disturbance.

```
[71]:
```

```
fig_9_26, axes = plt.subplots(nrows=1, ncols=2, figsize=(12, 4))
(ax1, ax2) = axes.flatten()

# Plot v1 vs. v5
plot_phase_portrait(
    flux_sol, x="v1", y="v5", ax=ax1,
    xlim=(0, 0.6), ylim=(0, 0.6),
    xlabel="v1", ylabel="v5", color="red",
    title=("(a) v1 vs. v5", XL_FONT),
    annotate_time_points="endpoints",
    annotate_time_points_color="black",
    annotate_time_points_legend="left outside");

# Plot v0 vs. v5
plot_phase_portrait(
    flux_sol, x="v0", y="v5", ax=ax2,
    legend=([model.id], "right outside"),
    xlim=(0, 1), ylim=(0, 1),
    xlabel="v0", ylabel="v5", color="red",
    title=("(a) v0 vs. v5", XL_FONT),
    annotate_time_points="endpoints",
    annotate_time_points_color="black");

# Annotate steady state line on first plot
ax1.plot([0, 1], [0, 1], color="grey", linestyle=":");

# Annotate flux balance lines on second plot
ax2.plot([0, .1], [.1, 0], color="grey", linestyle=":");
ax2.plot([0, .1*scalar], [.1*scalar, 0], color="grey", linestyle=":");
fig_9_26.tight_layout()
```

**Figure 9.26:** Dynamic phase portraits for (a) the flux into \((v\_1)\) and out of \((v\_5)\) in the biosynthetic pathway and (b) the flux out of the primary pathway \((v\_0)\) and out of the biosynthetic pathway \((v\_5)\). The model is the simple feedback loop for the same conditions as in Figure 9.9 except with the protein synthesis and degradation reactions.

###### Dimer With Protein Synthesis¶

We repeat the process for the dimer model and compare it to the monomer models with and without protein synthesis:

```
[72]:
```

```
dimer_w_synth = dimer.copy()
dimer_w_synth.id += "_with_Synthesis"

mets = dimer_w_synth.metabolites
# Define synthesis
v10 = MassReaction("v10")
v10.add_metabolites({mets.x6: 1})

# Define drain
v11 = MassReaction("v11", reversible=False)
v11.add_metabolites({mets.x6: -1})
v11.kf = 0.1

dimer_w_synth.add_reactions([v10, v11])
dimer_w_synth.add_custom_rate(
    v10, custom_rate="{0} / (1 + {1}*x5(t))".format(
        v10.kf_str, v10.Keq_str))
```

To get the parameters for the steady state system, we use the parameters from above and simulate to very long times to obtain the steady state concentrations. Since we will substitute these values into sympy equations in the next step, we will also ensure that the metabolites are represented as **sympy.Symbol** objects

```
[73]:
```

```
ics = {sym.Symbol(met): ic_value
       for met, ic_value in sim_dimer.find_steady_state(
           dimer, strategy="simulate")[0].items()}
for metabolite, ic in ics.items():
    print("%s: %s" % (metabolite, ic))
```

```
x1: 0.09203016916503
x2: 0.053984915417485006
x3: 0.053984915417485006
x4: 0.053984915417485006
x5: 0.053984915417485006
x6: 0.5866001976012711
x7: 0.3166756205138461
x8: 0.0427392664705489
```

We then set the rate equations for the protein synthesis and degradation equal to each other. We also set the quantity of \(K\_{10}x\_{5}\) equal to 10 since we want a protein turnover time constant of 10, and the rate law seen in Eq. (9.9) represents the synthesis rate.

```
[74]:
```

```
# Set up symbolic equalities using sympy
eq1 = sym.Eq(10, sym.Symbol(v10.Keq_str)*sym.Symbol("x5"))
sym.pprint(eq1)

eq2 = sym.Eq(strip_time(dimer_w_synth.custom_rates[v10]),
             strip_time(strip_time(dimer_w_synth.rates[v11])))
sym.pprint(eq2)
```

```
10 = Keqᵥ₁₀⋅x₅
    kfᵥ₁₀
───────────── = kfᵥ₁₁⋅x₆
Keqᵥ₁₀⋅x₅ + 1
```

We solve the equations using the steady state conditions for \(K\_{10}\) and then for \(k\_{10}:\)

```
[75]:
```

```
# Substitute ics and obtain Keq for v10
sym.pprint(eq1.subs(ics))

#Update model with new parameter value
v10.Keq = float(sym.solve(eq1.subs(ics))[0])
print("{0} = {1:.3f}\n".format(v10.Keq_str, v10.Keq))

# Susbtitute ics and Keq for v10, and kf for v11 to obtain kf for v10
parameters = {sym.Symbol(v10.Keq_str): v10.Keq, sym.Symbol(v11.kf_str): v11.kf}
sym.pprint(eq2.subs(ics).subs(parameters))

#Update model with new parameter value
v10.kf = float(sym.solve(eq2.subs(ics).subs(parameters))[0])
print("{0} = {1:.3f}\n".format(v10.kf_str, v10.kf))
```

```
10 = 0.053984915417485⋅Keqᵥ₁₀
Keq_v10 = 185.237

0.0909090909090909⋅kfᵥ₁₀ = 0.0586600197601271
kf_v10 = 0.645
```

###### Dynamic states: dimer model protein synthesis¶

The effect of this additional process can be simulated and plotted for comparison with the monomer models.

```
[76]:
```

```
scalar = 10
perturbation_dict = {"kf_b1": "kf_b1 * {0}".format(scalar)}

t0, tf = (0, 150)
sim_dimer_synth = Simulation(dimer_w_synth)

# Place models and simulations into lists for later
models_with_synth += [dimer_w_synth]
simulations_with_synth += [sim_dimer_synth]

for model, sim in zip([dimer, dimer_w_synth],
                      [sim_dimer, sim_dimer_synth]):
    conc_sol, flux_sol = sim.simulate(
        model, time=(t0, tf, tf*10 + 1),
        perturbations=perturbation_dict);

    conc_sol.make_aggregate_solution(
        "Total_Enzyme", equation="x2 + x6 + x7 + x8",
        variables=["x2", "x6", "x7", "x8"]);
```

```
WARNING: No compartments found in model. Therefore creating compartment 'compartment' for entire model.
```

```
[77]:
```

```
fig_9_27, axes = plt.subplots(nrows=2, ncols=1, figsize=(10, 4))
(ax1, ax2) = axes.flatten()

synthesis_strs = ["", " with Synthesis"]
linestyles = ["--", "-"]
colors = ["red", "blue", "green"]
observable_fluxes = [["v2"], ["v2", "v10", "v11"]]


for i, (model, sim) in enumerate(zip([dimer, dimer_w_synth],
                                     [sim_dimer, sim_dimer_synth])):
    conc_sol = sim.concentration_solutions.get_by_id(
        "_".join((model.id, "ConcSols")))
    flux_sol = sim.flux_solutions.get_by_id(
        "_".join((model.id, "FluxSols")))

    plot_time_profile(
        conc_sol, observable=["x1", "x5"], ax=ax1,
        legend=(["x1" + synthesis_strs[i], "x5" + synthesis_strs[i]],
                "right outside"),
        color=colors[:2], linestyle=linestyles[i]);

    plot_time_profile(
        conc_sol, observable=["Total_Enzyme"], ax=ax1,
        legend=(["Total Enzyme" + synthesis_strs[i]], "right outside"),
        xlabel="Time [hr]", ylabel="Concentration [mM]",
        title=("(a) Concentrations", XL_FONT),
        color=colors[2:], linestyle=linestyles[i]);

    plot_time_profile(
        flux_sol, observable=observable_fluxes[i], ax=ax2,
        legend=([entry + " " + synthesis_strs[i]
                 for entry in observable_fluxes[i]],
                "right outside"),
        xlabel="Time [hr]", ylabel="Flux [mM/hr]",
        title=("(b) Fluxes", XL_FONT),
        color=colors[:2*i + 1], linestyle=linestyles[i]);
fig_9_27.tight_layout()
```

**Figure 9.27:** The time profiles for concentrations and fluxes involved in the dimer model with feedback control of protein synthesis. The parameter values used are \(k\_{10}=0.645,\ K\_{10} =185,\) and \(k\_{11} = 0.10.\) Other parameter values are as in Figure 9.8.(a) Key concentrations as a function of time for the monomer model with and without the protein synthesis. (b) Key reaction fluxes as a function of time for the monomer model with and without the protein synthesis.

###### Slowly changing pool sizes: dimer model protein synthesis¶

Next, we examine the changes in pool size:

```
[78]:
```

```
fig_9_28 = plt.figure(figsize=(14, 4))
gs = fig_9_28.add_gridspec(nrows=1, ncols=2, width_ratios=[1, 1.5])

ax1 = fig_9_28.add_subplot(gs[0, 0])
ax2 = fig_9_28.add_subplot(gs[0, 1])

# Annotate line representing constant enzyme on plots
ax1.plot([0, 1], [1, 0], color="grey", linestyle=":")
ax2.plot([t0, tf], [1, 1], color="grey", linestyle=":",
         label="Constant Enzyme Pool, No synthesis");
ax2.legend()

enzyme_pools = {
    "Free": ["x2 + x6", ["x2", "x6"]],
    "Monomer_with_Synthesis_Inhibited": ["x7", ["x7"]],
    "Dimer_with_Synthesis_Inhibited": ["x7 + x8", ["x7", "x8"]]}

colors = ["red", "blue"]

for i, (model, sim) in enumerate(zip(models_with_synth,
                                     simulations_with_synth)):
    conc_sol = sim.concentration_solutions.get_by_id(
        "_".join((model.id, "ConcSols")))
    for pool_id, args in enzyme_pools.items():
        if pool_id != "Free" and model.id not in pool_id:
            continue
        equation, variables = args
        conc_sol.make_aggregate_solution(
            pool_id, equation, variables)

    plot_phase_portrait(
        conc_sol, x="Free", y=model.id + "_Inhibited", ax=ax1,
        xlim=(0.3, .7), ylim=(0.3, .7),
        xlabel="Free Enzyme", ylabel="Inhibited Enzyme",
        color=colors[i], linestyle=["--"],
        title=("Free vs. Inhibited Enzyme", XL_FONT),
        annotate_time_points="endpoints",
        annotate_time_points_color=colors[i],
        annotate_time_points_labels=True)

    plot_time_profile(
        conc_sol, observable=["Total_Enzyme"], ax=ax2,
        legend=([model.id], "right outside"),
        xlim=(t0, 50), color=colors[i],
        title=("(b) Total Enzyme pool", XL_FONT));
fig_9_28.tight_layout()
```

**Figure 9.28:** (a) Dynamic phase portrait of the inhibited enzyme vs. the free enzyme for the monomer and dimer models with the protein synthesis reactions and (b) the conservation pools over time.

Analyze the effects of the protein synthesis reactions on the models and their ability to reject the disturbance.

```
[79]:
```

```
fig_9_29, axes = plt.subplots(nrows=1, ncols=2, figsize=(12, 4))
(ax1, ax2) = axes.flatten()
colors = ["red", "blue"]
for i, (model, sim) in enumerate(zip(models_with_synth,
                                     simulations_with_synth)):
    flux_sol = sim.flux_solutions.get_by_id(
        "_".join((model.id, "FluxSols")))

    # Plot v1 vs. v5
    plot_phase_portrait(
        flux_sol, x="v1", y="v5", ax=ax1,
        xlim=(0, 0.6), ylim=(0, 0.6),
        xlabel="v1", ylabel="v5", color=colors[i],
        title=("(a) v1 vs. v5", XL_FONT),
        annotate_time_points="endpoints",
        annotate_time_points_color="black",
        annotate_time_points_legend="left outside");

    # Plot v0 vs. v5
    plot_phase_portrait(
        flux_sol, x="v0", y="v5", ax=ax2,
        legend=([model.id], "right outside"),
        xlim=(0, 1), ylim=(0, 1),
        xlabel="v0", ylabel="v5", color=colors[i],
        title=("(a) v0 vs. v5", XL_FONT),
        annotate_time_points="endpoints",
        annotate_time_points_color="black");

# Annotate steady state line on first plot
ax1.plot([0, 1], [0, 1], color="grey", linestyle=":");

# Annotate flux balance lines on second plot
ax2.plot([0, .1], [.1, 0], color="grey", linestyle=":");
ax2.plot([0, .1*scalar], [.1*scalar, 0], color="grey", linestyle=":");
fig_9_29.tight_layout()
```

**Figure 9.29:** Dynamic phase portraits for (a) the flux into \((v\_1)\) and out of \((v\_5)\) in the biosynthetic pathway and (b) the flux out of the primary pathway \((v\_0)\) and out of the biosynthetic pathway \((v\_5)\). The model is the simple feedback loop for the same conditions as in Figure 9.14 except with the protein synthesis and degradation reactions.

###### Tetramer With Protein Synthesis¶

We repeat the process for the tetramer model and compare it to the monomer and dimer models with and without protein synthesis:

```
[80]:
```

```
tetramer_w_synth = tetramer.copy()
tetramer_w_synth.id += "_with_Synthesis"

mets = tetramer_w_synth.metabolites
# Define synthesis
v10 = MassReaction("v10")
v10.add_metabolites({mets.x6: 1})

# Define drain
v11 = MassReaction("v11", reversible=False)
v11.add_metabolites({mets.x6: -1})
v11.kf = 0.1

tetramer_w_synth.add_reactions([v10, v11])
tetramer_w_synth.add_custom_rate(
    v10, custom_rate="{0} / (1 + {1}*x5(t))".format(
        v10.kf_str, v10.Keq_str))
```

To get the parameters for the steady state system, we use the parameters from above and simulate to very long times to obtain the steady state concentrations. Since we will substitute these values into sympy equations in the next step, we will also ensure that the metabolites are represented as **sympy.Symbol** objects.

```
[81]:
```

```
ics = {sym.Symbol(met): ic_value
       for met, ic_value in sim_tetramer.find_steady_state(
           tetramer, strategy="simulate")[0].items()}
for metabolite, ic in ics.items():
    print("%s: %s" % (metabolite, ic))
```

```
x1: 0.09214361177035077
x2: 0.05392819411482462
x3: 0.05392819411482462
x4: 0.05392819411482462
x5: 0.05392819411482462
x6: 0.5852624297952385
x7: 0.31562145922111534
x8: 0.042552238299201274
x9: 0.0025497392966884456
x10: 8.593927233375681e-05
```

We then set the rate equations for the protein synthesis and degradation equal to each other. We also set the quantity of \(K\_{10}x\_{5}\) equal to 10 since we want a protein turnover time constant of 10, and the rate law seen in Eq. (9.9) represents the synthesis rate.

```
[82]:
```

```
# Set up symbolic equalities using sympy
eq1 = sym.Eq(10, sym.Symbol(v10.Keq_str)*sym.Symbol("x5"))
sym.pprint(eq1)

eq2 = sym.Eq(strip_time(tetramer_w_synth.custom_rates[v10]),
             strip_time(strip_time(tetramer_w_synth.rates[v11])))
sym.pprint(eq2)
```

```
10 = Keqᵥ₁₀⋅x₅
    kfᵥ₁₀
───────────── = kfᵥ₁₁⋅x₆
Keqᵥ₁₀⋅x₅ + 1
```

We solve the equations using the steady state conditions for \(K\_{10}\) and then for \(k\_{10}:\)

```
[83]:
```

```
# Substitute ics and obtain Keq for v10
sym.pprint(eq1.subs(ics))

#Update model with new parameter value
v10.Keq = float(sym.solve(eq1.subs(ics))[0])
print("{0} = {1:.3f}\n".format(v10.Keq_str, v10.Keq))

# Susbtitute ics and Keq for v10, and kf for v11 to obtain kf for v10
parameters = {sym.Symbol(v10.Keq_str): v10.Keq, sym.Symbol(v11.kf_str): v11.kf}
sym.pprint(eq2.subs(ics).subs(parameters))

#Update model with new parameter value
v10.kf = float(sym.solve(eq2.subs(ics).subs(parameters))[0])
print("{0} = {1:.3f}\n".format(v10.kf_str, v10.kf))
```

```
10 = 0.0539281941148246⋅Keqᵥ₁₀
Keq_v10 = 185.432

0.0909090909090909⋅kfᵥ₁₀ = 0.0585262429795239
kf_v10 = 0.644
```

###### Dynamic states: tetramer model protein synthesis¶

The effect of this additional process can be simulated and plotted for comparison with the monomer models.

```
[84]:
```

```
scalar = 10
perturbation_dict = {"kf_b1": "kf_b1 * {0}".format(scalar)}

t0, tf = (0, 150)
sim_tetramer_synth = Simulation(tetramer_w_synth)

# Place models and simulations into lists for later
models_with_synth += [tetramer_w_synth]
simulations_with_synth += [sim_tetramer_synth]

for model, sim in zip([tetramer, tetramer_w_synth],
                      [sim_tetramer, sim_tetramer_synth]):
    conc_sol, flux_sol = sim.simulate(
        model, time=(t0, tf, tf*10 + 1),
        perturbations=perturbation_dict);

    conc_sol.make_aggregate_solution(
        "Total_Enzyme", equation="x2 + x6 + x7 + x8 + x9 + x10",
        variables=["x2", "x6", "x7", "x8", "x9", "x10"]);
```

```
WARNING: No compartments found in model. Therefore creating compartment 'compartment' for entire model.
```

```
[85]:
```

```
fig_9_30, axes = plt.subplots(nrows=2, ncols=1, figsize=(10, 4))
(ax1, ax2) = axes.flatten()

synthesis_strs = ["", " with Synthesis"]
linestyles = ["--", "-"]
colors = ["red", "blue", "green"]
observable_fluxes = [["v2"], ["v2", "v10", "v11"]]


for i, (model, sim) in enumerate(zip([tetramer, tetramer_w_synth],
                                     [sim_tetramer, sim_tetramer_synth])):
    conc_sol = sim.concentration_solutions.get_by_id(
        "_".join((model.id, "ConcSols")))
    flux_sol = sim.flux_solutions.get_by_id(
        "_".join((model.id, "FluxSols")))

    plot_time_profile(
        conc_sol, observable=["x1", "x5"], ax=ax1,
        legend=(["x1" + synthesis_strs[i], "x5" + synthesis_strs[i]],
                "right outside"),
        color=colors[:2], linestyle=linestyles[i]);

    plot_time_profile(
        conc_sol, observable=["Total_Enzyme"], ax=ax1,
        legend=(["Total Enzyme" + synthesis_strs[i]], "right outside"),
        xlabel="Time [hr]", ylabel="Concentration [mM]",
        title=("(a) Concentrations", XL_FONT),
        color=colors[2:], linestyle=linestyles[i]);

    plot_time_profile(
        flux_sol, observable=observable_fluxes[i], ax=ax2,
        legend=([entry + " " + synthesis_strs[i]
                 for entry in observable_fluxes[i]],
                "right outside"),
        xlabel="Time [hr]", ylabel="Flux [mM/hr]",
        title=("(b) Fluxes", XL_FONT),
        color=colors[:2*i + 1], linestyle=linestyles[i]);
fig_9_30.tight_layout()
```

**Figure 9.30:** The time profiles for concentrations and fluxes involved in the dimer model with feedback control of protein synthesis. The parameter values used are \(k\_{10}=0.645,\ K\_{10} =185,\) and \(k\_{11} = 0.10\). Other parameter values are as in Figure 9.8.(a) Key concentrations as a function of time for the monomer model with and without the protein synthesis. (b) Key reaction fluxes as a function of time for the monomer model with and without the protein synthesis.

###### Slowly changing pool sizes: tetramer model protein synthesis¶

Next, we examine the changes in pool size:

```
[86]:
```

```
fig_9_31 = plt.figure(figsize=(14, 4))
gs = fig_9_31.add_gridspec(nrows=1, ncols=2, width_ratios=[1, 1.5])

ax1 = fig_9_31.add_subplot(gs[0, 0])
ax2 = fig_9_31.add_subplot(gs[0, 1])

# Annotate line representing constant enzyme on plots
ax1.plot([0, 1], [1, 0], color="grey", linestyle=":")
ax2.plot([t0, tf], [1, 1], color="grey", linestyle=":",
         label="Constant Enzyme Pool, No synthesis");
ax2.legend()

enzyme_pools = {
    "Free": ["x2 + x6", ["x2", "x6"]],
    "Monomer_with_Synthesis_Inhibited": ["x7", ["x7"]],
    "Dimer_with_Synthesis_Inhibited": ["x7 + x8", ["x7", "x8"]],
    "Tetramer_with_Synthesis_Inhibited": [
        "x7 + x8 + x9 + x10", ["x7", "x8", "x9", "x10"]]}

colors = ["red", "blue", "green"]

for i, (model, sim) in enumerate(zip(models_with_synth,
                                     simulations_with_synth)):
    conc_sol = sim.concentration_solutions.get_by_id(
        "_".join((model.id, "ConcSols")))
    for pool_id, args in enzyme_pools.items():
        if pool_id != "Free" and model.id not in pool_id:
            continue
        equation, variables = args
        conc_sol.make_aggregate_solution(
            pool_id, equation, variables)

    plot_phase_portrait(
        conc_sol, x="Free", y=model.id + "_Inhibited", ax=ax1,
        xlim=(0.3, .7), ylim=(0.3, .7),
        xlabel="Free Enzyme", ylabel="Inhibited Enzyme",
        color=colors[i], linestyle=["--"],
        title=("Free vs. Inhibited Enzyme", XL_FONT),
        annotate_time_points="endpoints",
        annotate_time_points_color=colors[i],
        annotate_time_points_labels=True)

    plot_time_profile(
        conc_sol, observable=["Total_Enzyme"], ax=ax2,
        legend=([model.id], "right outside"),
        xlim=(t0, 50), color=colors[i],
        title=("(b) Total Enzyme pool", XL_FONT));
fig_9_31.tight_layout()
```

**Figure 9.31:** (a) Dynamic phase portrait of the inhibited enzyme vs. the free enzyme for the monomer, dimer and tetramer models with and without the protein synthesis reactions and (b) the conservation pools over time.

Analyze the effects of the protein synthesis reactions on the models and their ability to reject the disturbance.

```
[87]:
```

```
fig_9_32, axes = plt.subplots(nrows=1, ncols=2, figsize=(12, 4))
(ax1, ax2) = axes.flatten()
colors = ["red", "blue", "green"]
for i, (model, sim) in enumerate(zip(models_with_synth,
                                     simulations_with_synth)):
    flux_sol = sim.flux_solutions.get_by_id(
        "_".join((model.id, "FluxSols")))

    # Plot v1 vs. v5
    plot_phase_portrait(
        flux_sol, x="v1", y="v5", ax=ax1,
        xlim=(0, 0.6), ylim=(0, 0.6),
        xlabel="v1", ylabel="v5", color=colors[i],
        title=("(a) v1 vs. v5", XL_FONT),
        annotate_time_points="endpoints",
        annotate_time_points_color="black",
        annotate_time_points_legend="left outside");

    # Plot v0 vs. v5
    plot_phase_portrait(
        flux_sol, x="v0", y="v5", ax=ax2,
        legend=([model.id], "right outside"),
        xlim=(0, 1), ylim=(0, 1),
        xlabel="v0", ylabel="v5", color=colors[i],
        title=("(a) v0 vs. v5", XL_FONT),
        annotate_time_points="endpoints",
        annotate_time_points_color="black");

# Annotate steady state line on first plot
ax1.plot([0, 1], [0, 1], color="grey", linestyle=":");

# Annotate flux balance lines on second plot
ax2.plot([0, .1], [.1, 0], color="grey", linestyle=":");
ax2.plot([0, .1*scalar], [.1*scalar, 0], color="grey", linestyle=":");
fig_9_32.tight_layout()
```

**Figure 9.32:** Dynamic phase portraits for (a) the flux into \((v\_1)\) and out of \((v\_5)\) in the biosynthetic pathway and (b) the flux out of the primary pathway \((v\_0)\) and out of the biosynthetic pathway \((v\_5)\). The model is the simple feedback loop for the same conditions as in Figure 9.20 except with the protein synthesis and degradation reactions.

##### APPENDIX¶

This text is about the use of simulation. We do provide this appendix to introduce the interested reader to the analysis of regulatory phenomena. We define basics concepts (A.1), the defining role of eigenvalues (A.2) and detailed mathematical analysis of local regulation (A.3).

###### A.1 Regulatory Signals: phenomenology¶

###### Basic features of a regulatory event:¶

The regulatory action of compounds is characterized by three important measures: bias, active range, and sensitivity. These features are illustrated in Figure 9.A1 and are detailed as follows:

- First, there is a built-in bias; the regulation is either negative (inhibition) or positive (activation).
- Second, there is a range of concentrations over which the signal is active. The measure of this concentration range is the dissociation constant for the regulatory molecule.
- Third is the sensitivity of the flux to changes in the concentration of the regulator, or the ‘gain’ of the regulation.

**Figure 9.A1:** Graphical representation of the three basic features of regulation.

###### Network topology:¶

Regulation can be exerted ‘close’ to the formation of the regulatory molecule, such as feedback activation of an enzyme by its product, or the regulator can be controlling ‘far’ away from its site formation, as is the case with citrate inhibition of PFK or for amino acids feedback inhibiting the first reaction in the sequence that leads to their formation, see Figure 9.6.

###### Physiological roles:¶

From a network perspective, there are two overall physiological functions of regulatory signals:

- To overcome any disturbances in the cellular environment. This function is the ‘disturbance rejection’ problem. A cell or an organism experiences a change in an environmental parameter but wants to maintain its homeostatic state.
- To drive a network from one state to the next, that is, change the homeostatic state. This need is broad and often encountered from the need to change from a non-growing state to a growing one in response to the availability of a nutrient or to the need for a precursor cell to initiate differentiation to a new state. In control theory, this is known as the ‘servo’ problem.

###### A.2 The Effects of Regulation on Dynamic States¶

We now quantitatively assess the effects of regulatory signals on network dynamic states, representing the most mathematically-difficult material in this book.

###### Basic mathematical features:¶

To examine the qualitative effects of signals on network dynamics, let us examine the simple scheme:

\[\begin{equation} \stackrel{v\_1(x)}{\longrightarrow} X \stackrel{v\_2(x)}{\longrightarrow} \tag{9.A1} \end{equation}\]

where the concentration of metabolite \(X,\ x,\) directly influences the rates of its own formation, \(v\_1(x),\) and degradation, \(v\_2(x)\). The following discussion is graphically illustrated in Figure 9.A2.

The dynamic mass balance on \(X\) is

\[\begin{equation} \frac{dx}{dt} = v\_1(x) - v\_2(x) \tag{9.A2} \end{equation}\]

that in a linearized form is

\[\begin{equation} \frac{dx'}{dt} = (\frac{\partial v\_1}{\partial x} - \frac{\partial v\_2}{\partial x})x' = \lambda x' \tag{9.A3} \end{equation}\]

where \(\lambda\) is the ‘net’ rate constant. The value of \(\lambda\) determines the rate of response of this system to changes in the concentration of \(X.\)

###### Measures of systemic effects:¶

The quantity, \(\lambda\), is a combination of the time constants for the individual reactions. It is thus a ‘systems’ property rather than a property of a single component or a link in the network. It is equivalent to the eigenvalue of this one-dimensional system. Eigenvalues are systems quantities that are combinations of the properties of the individual components and links. These combinations become complicated as the size of a networks grows.

**Figure 9.A2:** Qualitative effects of regulatory signals. The graphs show the dependency of flux on the concentration of X.

###### Local regulation:¶

We examine the various scenarios that can arise when regulation is added on top of the natural mass action trend:

- *The unregulated situation:* When no regulation is exerted, the eigenvalue that describes the dynamics is given by:

  \[\begin{equation} \lambda = \frac{\partial v\_1}{\partial x} - \frac{\partial v\_2}{\partial x}' = 0 - k\_2 = \lambda^\* \lt 0 \tag{9.A4} \end{equation}\]

  if we assume that the turnover rate is first order and the formation rate is zeroth order. The unregulated situation corresponds to elementary mass action kinetics overlaid on the stoichiometric structure.
- *Feedback inhibition:* If the formation rate is feedback inhibited, the eigenvalue becomes more negative than for the unregulated situation since now \(\partial v\_1/ \partial x \lt 0\) and hence the time constant is faster. The feedback inhibition therefore augments the mass action trend and supports the natural dynamics of the system.
- *Feedback activation:* Feedback activation does just the opposite. Now \(\partial v\_1/ \partial x \gt 0\) and this signal thus tends to counter the natural dynamics of the system. If the signal is sufficiently strong, then the eigenvalue can become zero or positive, creating an instability that is reflected as multiple steady states.
- *Feedforward inhibition:* This signal tends to reduce \(\partial v\_2/ \partial x\) and hence tends to move \(\lambda\) closer to zero making instability more likely. Feedforward inhibition counters the mass action trend and is classified as a destabilizing signal. Note that saturation kinetics are of this type and they are observed to create instabilities in several models (Palsson 1988a, Savageau 1974, Tyson 1983).
- *Feedforward activation:* Feedforward activation increases the magnitude of \(\lambda\) and thus supports the mass action trend.

###### Regulatory principles:¶

Regulatory signals can either support or antagonize the mass action trend in a network. Thus, we arrive at the following principles:

\(\textbf{1.}\) Local negative feedback and local positive feedforward controls support the mass action trend and are stabilizing in the sense that they try to maintain the intrinsic dynamic properties of the stoichiometric structure.

\(\textbf{2.}\) Local positive feedback and local negative feedforward controls counteract the mass action trend and can create instabilities. Many of the creative functions associated with metabolism can be attributed to these control modes. These signals allow the cell to behave in apparent defiance to the laws of mass action and stoichiometric trends.

Regulators that act ‘far’ from their site of formation can induce dynamic instabilities even when the signal supports the mass action trend. There is a limit on the extent of stabilization or support of homeostasis achievable.

###### Measuring the dynamic effects of regulation¶

Equation (9.A4) may be rewritten as

\[\begin{split}\begin{align}\lambda &= -\frac{\partial v\_2}{\partial x}(1 - \frac{\partial v\_1/\partial x}{\partial v\_2/\partial x}) \tag{9.A5} \\ &\approx -\frac{\partial v\_2}{\partial x}(1 \pm {\frac{t\_{turnover}}{t\_{regulation}}}) \tag{9.A6} \\ \end{align}\end{split}\]

if the time constants indicated are good estimates of the corresponding partial derivatives. The dimensionless ratio:

\[\begin{equation} a = \large{\frac{t\_{turnover}}{t\_{regulation}}} \tag{9.A7} \end{equation}\]

thus characterizes local regulatory signals. If \(a\) is less than unity or on the order of unity, the regulation is dynamically about as important as the natural turnover time. However, if it significantly exceeds unity, one would expect that dynamics then would become dominated by the regulatory action.

###### A.3 Local Regulation with Hill Kinetics¶

We will now look at specific examples to quantitatively explore the concepts introduced in the last section. We will use Hill-type rate laws that are the simplest mathematical forms for regulated reactions. Even for the simplest case, the algebra becomes a bit cumbersome.

###### Inhibition¶

We can look quantitatively at the effects of local feedback inhibition. We can consider specific functional forms for \(v\_1\) and \(v\_2\) in Eq (9.A2):

\[\begin{equation} v\_1(x) = \frac{v\_m}{1 + (x/K)^2}\ \text{and}\ v\_2(x) = kx \tag{9.A8} \end{equation}\]

where the production rate is a Hill-type equation with \(\nu =2\) and the removal is an elementary first-order equation. The dynamic mass balance is

\[\begin{equation} \frac{dx}{dt} = \frac{v\_m}{1 + (x/K)^2} - kx \tag{9.A9} \end{equation}\]

The dynamics of this simple system can be analyzed to determine the dynamic effects of the feedback inhibition.

###### The steady state: inhibition¶

The steady state equation, \(v\_1 = v\_2\), for this network is a cubic equation

\[\begin{equation} (\frac{x}{K})^3 + \frac{x}{K} - \frac{v\_m}{kK} = 0 \tag{9.A10} \end{equation}\]

Introducing a dimensionless concentration \(\chi = x/K\) we have that

\[\begin{equation} \chi^3 + \chi - a = 0 \tag{9.A11} \end{equation}\]

where \(a=v\_m/kK.\) This equation has one real root

\[\begin{equation} \chi\_{ss} = \large{\frac{\sqrt[3]{9a\ +\ \sqrt{3}\sqrt{27a^2\ +\ 4}}}{3\ \sqrt[3]{\frac{2}{3}}} - \frac{\sqrt[3]{\frac{2}{3}}}{\sqrt[3]{9a\ +\ \sqrt{3}\sqrt{27a^2\ +\ 4}}}} \tag{9.A12} \end{equation}\]

The steady state level of \(x\), relative to \(K\), is dependent on a single parameter, \(a\), as shown in Figure 9.A3. We see that

\[\begin{equation} a = \frac{1/k}{K/v\_m} = \frac{t\_{turnoever}}{t\_{regulation}} \tag{9.A13} \end{equation}\]

###### Determining the eigenvalue: inhibition¶

By defining \(\tau = kt\) we have

\[\begin{equation} \frac{d\chi}{d\tau} = \frac{a}{1 + \chi^2} - \chi\ \text{and thus}\ \chi\_{ss} = \frac{a}{1 + \chi\_{ss}^2} \tag{9.A14} \end{equation}\]

The eigenvalue can be computed

\[\begin{equation} \lambda = \frac{\partial}{d\chi}[\frac{a}{1 + \chi^2} - \chi]\_{ss} = \frac{2a \chi\_{ss}}{(1 + chi\_{ss}^2} - 1 = -\frac{2\chi\_{ss}^2}{1 + \chi\_{ss}^2} - 1 \tag{9.A15} \end{equation}\]

The eigenvalue thus changes from a negative unity (when \(x\_{ss} \ll K\) and the regulation is not felt) to a negative three (when \(x\_{ss} \gg K\) where regulation is strong).

**Figure 9.A3:** Regulation by local feedback inhibition. (a) The dependency of the steady state concentration, \(\chi\_{ss} = x/K,\) on \(a = v\_m/kK\) by solving Eq. (9.A11). (b) The eigenvalue as a function of a computed from equation (9.A13).

###### Dynamic simulations: inhibition¶

The dynamic effects of the feedback regulation can be simulated, Figure 9.A4. Three cases are considered by changing the single dimensionless parameter, \(a.\) As \(a\) increases, the regulation is tighter and the value of \(\lambda\) increases, thus making the approach to steady state faster.

**Figure 9.A4:** Regulation by local feedback inhibition. The dynamic responses of \(d\chi/d\tau = a/(1+\chi^2\) for A): Long dashes: \(a = 0.1 (\chi\_{ss} = 0.1,\lambda = −1.02).\) B): Short dashes: \(a = 1 (\chi\_{ss} = 0.68,\ \lambda = −1.64).\) C): Solid line: \(a = 10\ (\chi\_{ss} = 2,\lambda = −2.6)\). The initial conditions for each case are \(0.9\chi\_{ss}\). Each curve is graphed as \(\chi(t)/\chi\_{ss}.\)\*

###### Activation¶

We can look quantitatively at the effects of local feedback activation. We can consider specific functional forms for \(v\_1\) and \(v\_2\) in Eq. (9.A2):

\[\begin{equation} v\_1(x) = v\_m\frac{1 + \alpha(x/K)^\nu}{1 + (x/K)^\nu}\ \text{and}\ v\_2(x) = kx\tag{9.A16} \end{equation}\]

with a dynamic mass balance

\[\begin{equation} \frac{dx}{dt} = v\_m\frac{1 + \alpha(x/K)^\nu}{1 + (x/K)^\nu} - kx \tag{9.A17} \end{equation}\]

We can make this equation dimensionless using the same dimensionless variables as above

\[\begin{equation} \frac{d\chi}{d\tau} = a\frac{1 + \alpha\chi^\nu}{1 + \chi^\nu} - \chi \tag{9.A18} \end{equation}\]

###### The steady state: activation¶

In a steady state, the dynamic mass balance becomes:

\[\begin{equation} \chi\_{ss} = a\frac{1 + \alpha\chi\_{ss}^\nu}{1 + \chi\_{ss}^\nu} \tag{9.A19} \end{equation}\]

###### Determining the eigenvalue: activation¶

The eigenvalue can be determined by the linearization of Eq. (9.A18)

\[\begin{equation} \lambda = \frac{\nu a(\alpha - 1)\chi\_{ss}^{\nu-1}}{(1+chi\_{ss}^\nu)^2} -1 \tag{9.A20} \end{equation}\]

since \(\alpha\gt \ 1\), the first term in equation Eq. (9.A20) is positive. This leads to the possibility that the eigenvalue is zero. This condition in turn leads to a situation where one can have multiple steady states as will now be demonstrated.

###### Existence of multiple steady states¶

We are looking for conditions where Eqs (9.A19) and (9.A20) are simultaneously zero, that is, the steady state condition and a zero eigenvalue. The two equations can be combined by multiplying Eq. (9.A20) by \(\chi\) and adding the equations together. After rearrangement, the equations become

\[\begin{equation} z^2 + [(1-\nu) + (1+\nu)/\alpha]z + 1/\alpha = 0,\ z=\chi^\nu \tag{9.A21} \end{equation}\]

This equation can only have a real positive solution if

\[\begin{equation} \alpha \gt \alpha\_{min} = \frac{(1 + \nu)}{(1-\nu)}^2 \tag{9.A22} \end{equation}\]

If \(\alpha\) exceeds this minimum value, the steady state equation will have multiple solutions for \(\chi\) for a range of values for \(a.\)

###### Region of multiple steady states in the parameter plane¶

If \(\alpha\) exceeds its minimum value, multiple steady states are possible. Equation (9.A21) will have two roots, \(\chi\_{1, ss}\) and \(\chi\_{2, ss}.\) These roots depend on two parameters, \(a\) and \(\alpha.\) To compute the relationships between \(a\) and \(\alpha.\) when the eigenvalue is zero, we can first specify \(\alpha\) and compute \(\chi\_{1, ss}\) and \(\chi\_{2, ss}\) and then compute the two corresponding values for \(a\_1\) and
\(a\_2\):

\[\begin{equation} a\_1 = \chi\_{1, ss}(\frac{1 + \chi\_{1, ss}^\nu}{1 + \alpha \chi\_{1, ss}^\nu}) \tag{9.A23} \end{equation}\]

and the same for \(a\_2\) as a function of \(\chi\_{2, ss}\). The results are shown in Figure 9.A5a.

###### Computing the multiple steady state values for the steady state concentration¶

For a fixed value of \(\alpha\) we can vary \(a\) along a line in the parameter plane and compute the steady state concentration. Since one cannot solve the steady state solution explicitly for \(\chi\_{ss},\) this is hard to do; however, one can rearrange it as

\[\begin{equation} a = \chi\_{1, ss}(\frac{1 + \chi\_{ss}^\nu}{1 + \alpha \chi\_{ss}^\nu}) \tag{9.A24} \end{equation}\]

and plot \(a\) versus \(\chi\_{ss}\) to get the same results. The limit, or turnaround point, corresponds to substituting the roots of Eq (9.A21) into Eq (9.A24). The resulting graphs are shown in Figure 9.A5b. Similarly, one can fix a and compute the relationship between \(\chi\_{ss}\) and \(\alpha,\) see Figure 9.A5c.

###### Simulating the dynamic response to a critical change in the parameter values¶

We can simulate the dynamic response of this loop to changes in the parameter values. As an example, we chose \(\alpha=10,\) and change a from an initial value of 0.3 to 0.1 at time zero. These two points lie on each side of the region of three steady states (Figure 9.A5a). The results from the dynamic simulation are shown in Figure 9.A5d.

**Figure 9.A.5:** Regulation by local feedback activation. (a): The plane of \(a\) and \(\alpha\) and the regions in the plane that can have one or three steady states. (b): The steady state concentration, \(\chi\_{ss},\) as a function of \(a\) for \(\alpha = 10\). (c): The steady state concentration, \(\chi\_{ss},\) as a function of \(\alpha\) for \(a = 0.3\). D): The dynamic response from an initial parameter set of \((a,\ \alpha) = (0.3,\ 10)\) to
\((a,\ \alpha) = (0.3,\ 10),\ \nu = 3.\) The points indicated by i, ii, iii, iv are selected critical points where the steady state solution ‘turns around’ thus demarcating the region in the parameter space where multiple steady state solutions exist.

\(\tiny{\text{© B. Ø. Palsson 2011;}\ \text{This publication is in copyright.}\\ \text{Subject to statutory exception and to the provisions of relevant collective licensing agreements,}\\ \text{no reproduction of any part may take place without the written permission of Cambridge University Press.}}\)

#### Glycolysis¶

Glycolysis is a central metabolic pathway. In this chapter, we formulate a mass action stoichiometric simulation (MASS) model of the glycolytic pathway. In doing so, we detail the process that goes into formulating, characterizing, and validating a simulation model of a single pathway. First, we define the pathway, or, more accurately stated, the *system* that is to be studied and characterized through dynamic simulation. Such a definition includes the internal reactions, the systems boundary,
and the exchange fluxes. The stoichiometric matrix is then constructed and quality controlled, and its properties studied. The contents of the null spaces give us information about the pathway and pool structure of the defined system. The steady flux state is deduced through the specification of a minimum number of experimentally determined fluxes. We use data from the red blood cell (RBC) for building the model. The steady state concentrations are then specified based on data. With the steady
state flux map and concentrations,the mass action kinetic constants are evaluated. These are called pseudo-elementary rate constants, or PERCs. A functional pooling structure and corresponding property ratios are formulated using biochemical rationale. The dynamic responses of glycolysis as a system can then be simulated and interpreted through the pooling structure. As in Chapter 8, we focus on the response to an increase in the rate of ATP utilization. With the formulated glycolytic MASS
model, the reader can simulate and study responses to other perturbations. In this chapter we focus only on the fundamental stoichiometric characteristics of the glycolytic system, but in subsequent chapters we will systematically expand the scope of the system being studied by including the regulatory enzymes.

This chapter will be split up into 3 parts. The first part will introduce the MASS model of the glycolytic network and set up the steady state parameters. The second part will use this model to dynamically simulate the response of this model, and to delve deeper into the pooling structure and relevant ratios. The final section will summarize the lessons from this chapter, and explore some applications of the network.

**MASSpy** will be used to demonstrate some of the topics in this chapter.

```
[1]:
```

```
from mass import (
    MassModel, MassMetabolite, MassReaction,
    Simulation, MassSolution, strip_time)
from mass.test import create_test_model
from mass.util.matrix import nullspace, left_nullspace, matrix_rank
from mass.visualization import (
    plot_time_profile, plot_phase_portrait, plot_tiled_phase_portraits)
```

Other useful packages are also imported at this time.

```
[2]:
```

```
from os import path

import matplotlib as mpl
import matplotlib.pyplot as plt
import numpy as np
import pandas as pd
import sympy as sym
```

Some options and variables used throughout the notebook are also declared here.

```
[3]:
```

```
pd.set_option("display.max_rows", 100)
pd.set_option("display.max_columns", 100)
pd.set_option('display.max_colwidth', None)
pd.options.display.float_format = '{:,.3f}'.format
S_FONT = {"size": "small"}
L_FONT = {"size": "large"}
INF = float("inf")
```

##### Glycolysis as a System¶

###### Defining the system¶

The glycolytic pathway degrades glucose (a six-carbon compound) to form pyruvate or lactate (three-carbon compounds) as end products. During this degradation process, the glycolytic pathway builds redox potential in the form of NADH and high energy phosphate bonds in the form of ATP via substrate-level phosphorylation. Glycolysis also assimilates an inorganic phosphate group that is converted into a high energy bond and then hydrolyzed in the ATP use reaction (or the energy ‘load’ reaction).
Glycolysis as a system is shown in Figure 10.1.

Glycolysis thus has interactions with three key cofactor or carrier moieties \((\text{ATP, NADH, }\text{P}\_i)\). These three key metabolic interactions of the glycolytic pathway with other cellular functions can be examined from a biochemical and metabolic physiological standpoint. Such intuitive analysis gives definition of key biochemical property pools in glycolysis leading us to gain insight into its systems biology. The charged state of these pools leads us to physiological states. This
material is summarized in Section 10.5

**Figure 10.1:** Glycolysis: the reaction schema, cofactor interactions, and environmental exchanges.

```
[4]:
```

```
glycolysis = create_test_model("SB2_Glycolysis")
```

###### The nodes (compounds) and links (reactions)¶

In its simplest form, glycolysis has 12 primary metabolites, 5 cofactor molecules \((\text{ATP, ADP, AMP, NAD, NADH})\), inorganic phosphate \((\text{P}\_i)\), protons \((\text{H}^+)\), and water \((\text{H}\_2\text{O})\). The system thus has 20 compounds, Table 10.1. The links formed between these compounds are the glycolytic reactions. There are 21 reactions, including all the transport reactions in and out of the system. The reactions are summarized in Table 10.2.

**Table 10.1:** The compounds in the glycolytic system, their abbreviations, and representative steady state concentrations. These compounds can be divided into three groups: i) the glycolytic intermediates (1-12), ii) the cofactors (13-17), and iii) inorganic components (18-20). The concentration of water is arbitrary as it will not appear in any reaction rate laws.

```
[5]:
```

```
metabolite_ids = [m.id for m in glycolysis.metabolites]

table_10_1 = pd.DataFrame(
    np.array([metabolite_ids,
              [met.name for met in glycolysis.metabolites],
              [glycolysis.initial_conditions[met]
               for met in glycolysis.metabolites]]).T,
    index=[i for i in range(1, len(metabolite_ids) + 1)],
    columns=["Abbreviations", "Species", "Initial Concentration"])
table_10_1
```

```
[5]:
```

|  | Abbreviations | Species | Initial Concentration |
| --- | --- | --- | --- |
| 1 | glc\_\_D\_c | D-Glucose | 1.0 |
| 2 | g6p\_c | D-Glucose 6-phosphate | 0.0486 |
| 3 | f6p\_c | D-Fructose 6-phosphate | 0.0198 |
| 4 | fdp\_c | D-Fructose 1,6-bisphosphate | 0.0146 |
| 5 | dhap\_c | Dihydroxyacetone phosphate | 0.16 |
| 6 | g3p\_c | Glyceraldehyde 3-phosphate | 0.00728 |
| 7 | \_13dpg\_c | 3-Phospho-D-glyceroyl phosphate | 0.000243 |
| 8 | \_3pg\_c | 3-Phospho-D-glycerate | 0.0773 |
| 9 | \_2pg\_c | D-Glycerate 2-phosphate | 0.0113 |
| 10 | pep\_c | Phosphoenolpyruvate | 0.017 |
| 11 | pyr\_c | Pyruvate | 0.060301 |
| 12 | lac\_\_L\_c | L-Lactate | 1.36 |
| 13 | nad\_c | Nicotinamide adenine dinucleotide | 0.0589 |
| 14 | nadh\_c | Nicotinamide adenine dinucleotide - reduced | 0.0301 |
| 15 | amp\_c | AMP | 0.0867281 |
| 16 | adp\_c | ADP | 0.29 |
| 17 | atp\_c | ATP | 1.6 |
| 18 | pi\_c | Phosphate | 2.5 |
| 19 | h\_c | H+ | 8.99757e-05 |
| 20 | h2o\_c | H2O | 1.0 |

**Table 10.2:** The glycolytic enzymes and transporters, their abbreviations, and chemical reactions. These reactions can be grouped into several categories: i) the glycolytic reactions (1-11), ii) adenosine phosphate metabolism (12,13), iii) primary exports (14,15), iii) the load functions (16,17), iv) the primary inputs, here fixed (18,19), and v) the water, proton, and phosphate exchanges.

```
[6]:
```

```
reaction_ids = [r.id for r in glycolysis.reactions]
table_10_2 = pd.DataFrame(
    np.array([reaction_ids,
              [r.name for r in glycolysis.reactions],
              [r.reaction for r in glycolysis.reactions]]).T,
    index=[i for i in range(1, len(glycolysis.reactions) + 1)],
    columns=["Abbreviations", "Enzymes/Transporter/Load", "Elementally Balanced Reaction"])
table_10_2
```

```
[6]:
```

|  | Abbreviations | Enzymes/Transporter/Load | Elementally Balanced Reaction |
| --- | --- | --- | --- |
| 1 | HEX1 | Hexokinase (D-glucose:ATP) | atp\_c + glc\_\_D\_c <=> adp\_c + g6p\_c + h\_c |
| 2 | PGI | Glucose-6-phosphate isomerase | g6p\_c <=> f6p\_c |
| 3 | PFK | Phosphofructokinase | atp\_c + f6p\_c <=> adp\_c + fdp\_c + h\_c |
| 4 | FBA | Fructose-bisphosphate aldolase | fdp\_c <=> dhap\_c + g3p\_c |
| 5 | TPI | Triose-phosphate isomerase | dhap\_c <=> g3p\_c |
| 6 | GAPD | Glyceraldehyde-3-phosphate dehydrogenase | g3p\_c + nad\_c + pi\_c <=> \_13dpg\_c + h\_c + nadh\_c |
| 7 | PGK | Phosphoglycerate kinase | \_13dpg\_c + adp\_c <=> \_3pg\_c + atp\_c |
| 8 | PGM | Phosphoglycerate mutase | \_3pg\_c <=> \_2pg\_c |
| 9 | ENO | Enolase | \_2pg\_c <=> h2o\_c + pep\_c |
| 10 | PYK | Pyruvate kinase | adp\_c + h\_c + pep\_c <=> atp\_c + pyr\_c |
| 11 | LDH\_L | L-lactate dehydrogenase | h\_c + nadh\_c + pyr\_c <=> lac\_\_L\_c + nad\_c |
| 12 | DM\_amp\_c | AMP demand | amp\_c --> |
| 13 | ADK1 | Adenylate kinase | 2 adp\_c <=> amp\_c + atp\_c |
| 14 | SK\_pyr\_c | Pyruvate sink | pyr\_c <=> |
| 15 | SK\_lac\_\_L\_c | L-Lactate sink | lac\_\_L\_c <=> |
| 16 | ATPM | ATP maintenance requirement | atp\_c + h2o\_c --> adp\_c + h\_c + pi\_c |
| 17 | DM\_nadh | Demand NADH | nadh\_c --> h\_c + nad\_c |
| 18 | SK\_glc\_\_D\_c | D-Glucose sink | <=> glc\_\_D\_c |
| 19 | SK\_amp\_c | AMP sink | <=> amp\_c |
| 20 | SK\_h\_c | H+ sink | h\_c <=> |
| 21 | SK\_h2o\_c | H2O sink | h2o\_c <=> |

##### The Stoichiometric Matrix¶

The stoichiometric matrix, \(\textbf{S}\), can be formulated for the glycolytic system. It is embedded in the glycolysis model. Its dimensions are 20 x 21, representing the 20 metabolites and the 21 fluxes given in Tables 10.1 and 10.2, respectively. The rank of this stoichiometric matrix is 18, leaving a 3 dimensional null space (i.e., 21-18) and a 2 dimensional left null space (i.e., 20-18).

```
[7]:
```

```
print(glycolysis.S.shape)
print(matrix_rank(glycolysis.S))
```

```
(20, 21)
18
```

The stoichiometric matrix has many attributes and properties, as discussed in Chapter 1. The ones from a modeling and systems standpoint will now be discussed. At the end of the section we show how all of these properties can be assembled into one succinct tabular format.

###### Elemental balancing¶

The stoichiometric matrix needs to be quality controlled to make sure that the chemical equations are mass balanced. The elemental compositions of the compounds in the glycolytic system are given in Table 10.3. This table is the elemental matrix, \(\textbf{E}\), for this system. We can multiply \(\textbf{ES}\) to quality control the reconstructed network for elemental balancing properties of the reactions, (i.e., verify that \(\textbf{ES} = 0\) ). The results are shown in Table 10.4.
All the internal reactions are elementally balanced. The exchange reactions are not elementally balanced as they represent net addition or removal from the system as defined.

```
[8]:
```

```
table_10_3 = glycolysis.get_elemental_matrix(
    array_type="DataFrame", dtype=np.int64)
```

**Table 10.3:** The elemental composition and charges of the glycolytic intermediates. This table represents the matrix \(\textbf{E}.\)\*

```
[9]:
```

```
table_10_3
```

```
[9]:
```

|  | glc\_\_D\_c | g6p\_c | f6p\_c | fdp\_c | dhap\_c | g3p\_c | \_13dpg\_c | \_3pg\_c | \_2pg\_c | pep\_c | pyr\_c | lac\_\_L\_c | nad\_c | nadh\_c | amp\_c | adp\_c | atp\_c | pi\_c | h\_c | h2o\_c |
| --- | --- | --- | --- | --- | --- | --- | --- | --- | --- | --- | --- | --- | --- | --- | --- | --- | --- | --- | --- | --- |
| C | 6 | 6 | 6 | 6 | 3 | 3 | 3 | 3 | 3 | 3 | 3 | 3 | 21 | 21 | 10 | 10 | 10 | 0 | 0 | 0 |
| H | 12 | 11 | 11 | 10 | 5 | 5 | 4 | 4 | 4 | 2 | 3 | 5 | 26 | 27 | 12 | 12 | 12 | 1 | 1 | 2 |
| O | 6 | 9 | 9 | 12 | 6 | 6 | 10 | 7 | 7 | 6 | 3 | 3 | 14 | 14 | 7 | 10 | 13 | 4 | 0 | 1 |
| P | 0 | 1 | 1 | 2 | 1 | 1 | 2 | 1 | 1 | 1 | 0 | 0 | 2 | 2 | 1 | 2 | 3 | 1 | 0 | 0 |
| N | 0 | 0 | 0 | 0 | 0 | 0 | 0 | 0 | 0 | 0 | 0 | 0 | 7 | 7 | 5 | 5 | 5 | 0 | 0 | 0 |
| S | 0 | 0 | 0 | 0 | 0 | 0 | 0 | 0 | 0 | 0 | 0 | 0 | 0 | 0 | 0 | 0 | 0 | 0 | 0 | 0 |
| q | 0 | -2 | -2 | -4 | -2 | -2 | -4 | -3 | -3 | -3 | -1 | -1 | -1 | -2 | -2 | -3 | -4 | -2 | 1 | 0 |
| [NAD] | 0 | 0 | 0 | 0 | 0 | 0 | 0 | 0 | 0 | 0 | 0 | 0 | 1 | 1 | 0 | 0 | 0 | 0 | 0 | 0 |

###### Charge balancing¶

A row in \(\textbf{E}\) represents the charges of the metabolites. Then, charge balance is ensured by making sure that \(\textbf{ES} = 0\). Charge balancing the whole system for transporters can be difficult as some of the transport systems, co-transport ions, and some ions can cross the membrane by themselves. Overall, the system has to be charge neutral. Accounting for full charge balances and the volume of a system can be quite mathematically involved; (see Joshi, 1989-I and Joshi,
1989-II.

The resulting table (Table 10.4) shows that \(\textbf{ES} = 0\) for all non-exchange reactions in the glycolysis model. Thus the reactions are charge and elementally balanced. The model passes this QC/QA test.

```
[10]:
```

```
table_10_4 = glycolysis.get_elemental_charge_balancing(
    array_type="DataFrame", dtype=np.int64)
```

**Table 10.4:** The elemental and charge balance test on the reactions. All internal reactions are balanced. Exchange reactions are not. Note that the NADH exchange reaction creates two electrons. This corresponds to the delivery of two electrons to the compound or process that uses redox potential.

```
[11]:
```

```
table_10_4
```

```
[11]:
```

|  | HEX1 | PGI | PFK | FBA | TPI | GAPD | PGK | PGM | ENO | PYK | LDH\_L | DM\_amp\_c | ADK1 | SK\_pyr\_c | SK\_lac\_\_L\_c | ATPM | DM\_nadh | SK\_glc\_\_D\_c | SK\_amp\_c | SK\_h\_c | SK\_h2o\_c |
| --- | --- | --- | --- | --- | --- | --- | --- | --- | --- | --- | --- | --- | --- | --- | --- | --- | --- | --- | --- | --- | --- |
| C | 0 | 0 | 0 | 0 | 0 | 0 | 0 | 0 | 0 | 0 | 0 | -10 | 0 | -3 | -3 | 0 | 0 | 6 | 10 | 0 | 0 |
| H | 0 | 0 | 0 | 0 | 0 | 0 | 0 | 0 | 0 | 0 | 0 | -12 | 0 | -3 | -5 | 0 | 0 | 12 | 12 | -1 | -2 |
| O | 0 | 0 | 0 | 0 | 0 | 0 | 0 | 0 | 0 | 0 | 0 | -7 | 0 | -3 | -3 | 0 | 0 | 6 | 7 | 0 | -1 |
| P | 0 | 0 | 0 | 0 | 0 | 0 | 0 | 0 | 0 | 0 | 0 | -1 | 0 | 0 | 0 | 0 | 0 | 0 | 1 | 0 | 0 |
| N | 0 | 0 | 0 | 0 | 0 | 0 | 0 | 0 | 0 | 0 | 0 | -5 | 0 | 0 | 0 | 0 | 0 | 0 | 5 | 0 | 0 |
| S | 0 | 0 | 0 | 0 | 0 | 0 | 0 | 0 | 0 | 0 | 0 | 0 | 0 | 0 | 0 | 0 | 0 | 0 | 0 | 0 | 0 |
| q | 0 | 0 | 0 | 0 | 0 | 0 | 0 | 0 | 0 | 0 | 0 | 2 | 0 | 1 | 1 | 0 | 2 | 0 | -2 | -1 | 0 |
| [NAD] | 0 | 0 | 0 | 0 | 0 | 0 | 0 | 0 | 0 | 0 | 0 | 0 | 0 | 0 | 0 | 0 | 0 | 0 | 0 | 0 | 0 |

```
[12]:
```

```
for boundary in glycolysis.boundary:
    print(boundary)
```

```
DM_amp_c: amp_c -->
SK_pyr_c: pyr_c <=>
SK_lac__L_c: lac__L_c <=>
SK_glc__D_c:  <=> glc__D_c
SK_amp_c:  <=> amp_c
SK_h_c: h_c <=>
SK_h2o_c: h2o_c <=>
```

###### Topological properties¶

The simplest topological properties of the stoichiometric matrix relate to the number of non-zero elements in rows and columns. The more advanced topological properties are associated with the null spaces and their basis vectors and are quantities of systems biology. All of these properties are summarized in Table 10.8 below.

The number of reactions in which a compound participates, \(\rho\_i\), is the *connectivity*. It is formally defined as the total number of non-zero elements in a row (*Systems Biology: Constraint-based reconstruction and analysis*). Most of the compounds are formed by one reaction and degraded by another, thus having a connectivity of 2. The proton participates in 8 reactions while ATP and ADP participate in 6, making them the most connected compounds in the system. Highly connected nodes
are often of interest as they tie many parts of a network together. As illustrated below, normally it is useful to draw node maps that show the flows in and out of a node.

The number of compounds that participate in a reaction, \(\pi\_j\), is called the *participation* number. It is formally defined as the total number of non-zero elements in a column (*Systems Biology: Constraint-based reconstruction and analysis*). Exchange reactions have a participation of unity and are thus elementally imbalanced. Some of the kinases and dehydrogenases have the highest participation number, 5.

###### The pathway structure: basis for the null space¶

The null space is spanned by three vectors, \(\textbf{p}\_1,\textbf{p}\_2, \textbf{p}\_3\), that have pathway interpretations (Chapter 9 of *Systems Biology: Constraint-based reconstruction and analysis*). They are not unique. The simplest pathway vectors, called MinSpan (Bordbar et al., 2014) can be computed.

**Table 10.5:** The calculated MinSpan pathway vectors for the stoichiometric matrix for the glycolytic system.

```
[13]:
```

```
# MinSpan pathways are calculated outside this notebook and the results are provided here.
minspan_paths = np.array([
    [1, 1, 1, 1, 1, 2, 2, 2, 2, 2, 2, 0, 0, 0, 2, 2, 0, 1, 0, 2, 0],
    [0, 0, 0, 0, 0, 0, 0, 0, 0, 0,-1, 0, 0, 1,-1, 0, 1, 0, 0, 2, 0],
    [0, 0, 0, 0, 0, 0, 0, 0, 0, 0, 0, 1, 0, 0, 0, 0, 0, 0, 1, 0, 0]])
# Create labels for the paths
path_labels = ["$p_1$","$p_2$", "$p_3$"]
# Create DataFrame
table_10_5 = pd.DataFrame(minspan_paths, index=path_labels,
                          columns=reaction_ids)
table_10_5
```

```
[13]:
```

|  | HEX1 | PGI | PFK | FBA | TPI | GAPD | PGK | PGM | ENO | PYK | LDH\_L | DM\_amp\_c | ADK1 | SK\_pyr\_c | SK\_lac\_\_L\_c | ATPM | DM\_nadh | SK\_glc\_\_D\_c | SK\_amp\_c | SK\_h\_c | SK\_h2o\_c |
| --- | --- | --- | --- | --- | --- | --- | --- | --- | --- | --- | --- | --- | --- | --- | --- | --- | --- | --- | --- | --- | --- |
| $p\_1$ | 1 | 1 | 1 | 1 | 1 | 2 | 2 | 2 | 2 | 2 | 2 | 0 | 0 | 0 | 2 | 2 | 0 | 1 | 0 | 2 | 0 |
| $p\_2$ | 0 | 0 | 0 | 0 | 0 | 0 | 0 | 0 | 0 | 0 | -1 | 0 | 0 | 1 | -1 | 0 | 1 | 0 | 0 | 2 | 0 |
| $p\_3$ | 0 | 0 | 0 | 0 | 0 | 0 | 0 | 0 | 0 | 0 | 0 | 1 | 0 | 0 | 0 | 0 | 0 | 0 | 1 | 0 | 0 |

These three pathways are illustrated graphically in Figure 10.2, and they are biochemically interpreted as follows:

1. The first pathway is a redox balanced use of glycolysis to produce two high energy bonds per glucose metabolized. A glucose enters and two pyruvate leave. There is no net production of NADH, but there is a net production of 2ATP that is balanced by the ATP load reaction.
2. The second pathway describes how a redox load on NADH is balanced by the uptake of lactate to produce pyruvate via LDH and generate the NADH that is used in the NADH load reaction. When this pathway is added to the first one, it leads to a reduced lactate and pyruvate secretion and the net production to NADH that meets the load imposed.
3. The third pathway is simply AMP entering and leaving the system. These rates have to be balanced in a steady state. This pathway will determine the total amount \((\text{A}\_{\mathrm{tot}}=\text{ATP + ADP + AMP})\) of the adenosine phosphates in the system. This is not a real pathway, but is a proxy for the processes that generate and degrade AMP in the cell. In Chapter 12, we detail the synthesis of AMP we remove the virtual dashed boundary (recall chapter 6).

The addition of these three pathways gives the steady state flux distribution as discussed in Section 10.3 below. To do so for an actual situation, we need three experimental measurements of three independent fluxes; one measurement that allows the unique determination of the flux through each pathway. Ideally, one can measure an exchange flux that is just found in a single pathway vector. As we will see in chapter 12, this situation can get more complicated.

Although these three vectors are a basis for the null space, they are not a unique basis. There are other choices of linear basis vectors. Alternatively, the so-called convex basis vectors do form a unique basis (*Systems Biology: Constraint-based reconstruction and analysis*).

**Figure 10.2:** The pathway vectors for the stoichiometric matrix for the glycolytic system. They span all possible steady state flux states.

###### Elemental balancing of the pathway vectors¶

Each of the pathway vectors needs to be elementally balanced, which means the elements coming into and leaving a pathway through the transporters need to be balanced. Thus we must have \(\textbf{ESp}\_i = 0\) for each pathway. This condition is satisfied for these three pathways.

**Table 10.6:** Elemental and charge balancing on the MinSpan pathways of the glycolytic system.

```
[14]:
```

```
table_10_6 = glycolysis.get_elemental_charge_balancing(
    array_type="DataFrame").dot(minspan_paths.T).astype(np.int64)
table_10_6.columns = path_labels
table_10_6
```

```
[14]:
```

|  | $p\_1$ | $p\_2$ | $p\_3$ |
| --- | --- | --- | --- |
| C | 0 | 0 | 0 |
| H | 0 | 0 | 0 |
| O | 0 | 0 | 0 |
| P | 0 | 0 | 0 |
| N | 0 | 0 | 0 |
| S | 0 | 0 | 0 |
| q | 0 | 0 | 0 |
| [NAD] | 0 | 0 | 0 |

###### The time invariant pools: the basis for the left null space¶

The stoichiometric matrix has a left null space of dimension 2, meaning the glycolytic system as defined has two time invariant pools. These can be found in the *Left Nullspace* of the glycolysis model. We can compute them directly:

**Table 10.7:** The left null space vectors of the stoichiometric matrix for the glycolytic system.

```
[15]:
```

```
lns = left_nullspace(glycolysis.S, rtol=1e-10)
# Iterate through left nullspace,
# dividing by the smallest value in each row.
for i, row in enumerate(lns):
    minval = np.min(abs(row[np.nonzero(row)]))
    new_row = np.array(row/minval)
    # Round to ensure the left nullspace is composed of only integers
    lns[i] = np.array([round(value) for value in new_row])

# Ensure positive stoichiometric coefficients if all are negative
for i, space in enumerate(lns):
    lns[i] = np.negative(space) if all([num <= 0 for num in space]) else space

# Row operations to find biologically meaningful pools
lns[1] = (lns[0] - lns[1])/12
lns[0] = (lns[0] - lns[1])
lns = lns.astype(np.int64)

# Create labels for the time invariants
time_inv_labels = ["$P_{\mathrm{tot}}$", "$N_{\mathrm{tot}}$"]
table_10_7 = pd.DataFrame(lns, index=time_inv_labels,
                          columns=metabolite_ids)
table_10_7
```

```
[15]:
```

|  | glc\_\_D\_c | g6p\_c | f6p\_c | fdp\_c | dhap\_c | g3p\_c | \_13dpg\_c | \_3pg\_c | \_2pg\_c | pep\_c | pyr\_c | lac\_\_L\_c | nad\_c | nadh\_c | amp\_c | adp\_c | atp\_c | pi\_c | h\_c | h2o\_c |
| --- | --- | --- | --- | --- | --- | --- | --- | --- | --- | --- | --- | --- | --- | --- | --- | --- | --- | --- | --- | --- |
| $P\_{\mathrm{tot}}$ | 0 | 1 | 1 | 2 | 1 | 1 | 2 | 1 | 1 | 1 | 0 | 0 | 0 | 0 | 0 | 1 | 2 | 1 | 0 | 0 |
| $N\_{\mathrm{tot}}$ | 0 | 0 | 0 | 0 | 0 | 0 | 0 | 0 | 0 | 0 | 0 | 0 | 1 | 1 | 0 | 0 | 0 | 0 | 0 | 0 |

\(\textbf{1.}\) The first time invariant is the total amount of phosphates on all the compounds in the network. Looking ahead to section 10.5 we call this pool 10. Mathematically it is:

\[\begin{split}\begin{align}p\_{10} &= \text{G6P} + \text{F6P} + 2\text{FDP} + \text{DHAP} + \text{GAP} + 2\text{13DPG} \\ &\ \ \ \ \ + \text{3PG} + \text{2PG} + \text{PEP} + \text{ADP} + 2\text{ATP} + \text{P}\_i \\ &= P\_{\mathrm{tot}} \end{align} \tag{10.1}\end{split}\]

Notice that the phosphate on AMP is not counted as this phosphate group enters and leaves the system as a part of the AMP moiety. We will discuss the consequences of this invariant pool in the appendix to the chapter.

\(\textbf{2.}\) The second time invariant is the total amount of the NAD cofactor moiety. This cofactor never leaves or enters the system as defined. This pool is:

\[\begin{equation} p\_{11} = \text{NADH} + \text{NAD} = N\_{\mathrm{tot}} \tag{10.2} \end{equation}\]

and is number 11. The linear basis for the left null space is not unique, but a convex basis is (Famili, 2005 and *Systems Biology: Constraint-based reconstruction and analysis*).

###### An ‘annotated’ form of the stoichiometric matrix¶

All of the properties of the stoichiometric matrix can be conveniently summarized in a tabular format, Table 10.8. The table succinctly summarizes the chemical and topological properties of \(\textbf{S}\). The matrix has dimensions of 20x21 and a rank of 3. It thus has a 3 dimensional null space and a two dimensional left null space.

**Table 10.8:** The stoichiometric matrix for the glycolytic system in Figure 10.1. The matrix is partitioned to show the glycolytic intermediates (yellow) separate from the cofactors and to separate the exchange reactions and cofactor loads (orange). The connectivities, \(\rho\_i\) (red), for a compound, and the participation number, \(\pi\_j\) (cyan), for a reaction are shown. The second block in the table is the product \(\textbf{ES}\) (blue) to evaluate elemental balancing status
of the reactions. All exchange reactions have a participation number of unity and are thus not elementally balanced. The last block in the table has the three pathway vectors (purple) for glycolysis. These vectors are graphically shown in Figure 10.2. Furthest to the right, we display the two time invariant pools (green) that span the left null space.

```
[16]:
```

```
# Define labels
pi_str = r"$\pi_{j}$"
rho_str = r"$\rho_{i}$"
chopsnq = ['C', 'H', 'O', 'P', 'N', 'S', 'q', '[NAD]']

# Make table content from the stoichiometric matrix, elemental balancing of pathways
# participation number, and MinSpan pathways
S_matrix = glycolysis.update_S(
    array_type="dense", dtype=np.int64, update_model=False)
ES_matrix = glycolysis.get_elemental_charge_balancing(dtype=np.int64)
pi = np.count_nonzero(S_matrix, axis=0)
rho = np.count_nonzero(S_matrix, axis=1)
table_10_8 = np.vstack((S_matrix, pi, ES_matrix, minspan_paths))

# Determine number of blank entries needed to be added to pad the table,
# Add connectivity number and time invariants to table content
blanks = [""]*(len(table_10_8) - len(glycolysis.metabolites))
rho = np.concatenate((rho, blanks))
time_inv = np.array([np.concatenate([row, blanks]) for row in lns])
table_10_8 = np.vstack([table_10_8.T, rho, time_inv]).T

colors = {"intermediates": "#ffffe6", # Yellow
          "cofactors": "#ffe6cc",     # Orange
          "chopsnq": "#99e6ff",       # Blue
          "pathways": "#b399ff",      # Purple
          "pi": "#99ffff",            # Cyan
          "rho": "#ff9999",           # Red
          "time_invs": "#ccff99",     # Green
          "blank": "#f2f2f2"}         # Grey
bg_color_str = "background-color: "
def highlight_table(df, model, main_shape):
    df = df.copy()
    n_mets, n_rxns = (len(model.metabolites), len(model.reactions))
    # Highlight rows
    for row in df.index:
        other_key, condition = ("blank", lambda i, v: v != "")
        if row == pi_str:        # For participation
            main_key = "pi"
        elif row in chopsnq:     # For elemental balancing
            main_key = "chopsnq"
        elif row in path_labels: # For pathways
            main_key = "pathways"
        else:
            # Distinguish between intermediate and cofactor reactions for model reactions
            main_key, other_key = ("cofactors", "intermediates")
            condition = lambda i, v: (main_shape[1] <= i and i < n_rxns)
        df.loc[row, :] = [bg_color_str + colors[main_key] if condition(i, v)
                          else bg_color_str + colors[other_key]
                          for i, v in enumerate(df.loc[row, :])]

    for col in df.columns:
        condition = lambda i, v: v != bg_color_str + colors["blank"]
        if col == rho_str:
            main_key = "rho"
        elif col in time_inv_labels:
            main_key = "time_invs"
        else:
            # Distinguish intermediates and cofactors for model metabolites
            main_key = "cofactors"
            condition = lambda i, v: (main_shape[0] <= i and i < n_mets)
        df.loc[:, col] = [bg_color_str + colors[main_key] if condition(i, v)
                          else v for i, v in enumerate(df.loc[:, col])]
    return df

# Create index and column labels
index_labels = np.concatenate((metabolite_ids, [pi_str], chopsnq, path_labels))
column_labels = np.concatenate((reaction_ids, [rho_str], time_inv_labels))
# Create DataFrame
table_10_8 = pd.DataFrame(
    table_10_8, index=index_labels, columns=column_labels)
# Apply colors
table_10_8 = table_10_8.style.apply(
    highlight_table,  model=glycolysis, main_shape=(12, 11), axis=None)
table_10_8
```

```
[16]:
```

|  | HEX1 | PGI | PFK | FBA | TPI | GAPD | PGK | PGM | ENO | PYK | LDH\_L | DM\_amp\_c | ADK1 | SK\_pyr\_c | SK\_lac\_\_L\_c | ATPM | DM\_nadh | SK\_glc\_\_D\_c | SK\_amp\_c | SK\_h\_c | SK\_h2o\_c | $\rho\_{i}$ | $P\_{\mathrm{tot}}$ | $N\_{\mathrm{tot}}$ |
| --- | --- | --- | --- | --- | --- | --- | --- | --- | --- | --- | --- | --- | --- | --- | --- | --- | --- | --- | --- | --- | --- | --- | --- | --- |
| glc\_\_D\_c | -1 | 0 | 0 | 0 | 0 | 0 | 0 | 0 | 0 | 0 | 0 | 0 | 0 | 0 | 0 | 0 | 0 | 1 | 0 | 0 | 0 | 2 | 0 | 0 |
| g6p\_c | 1 | -1 | 0 | 0 | 0 | 0 | 0 | 0 | 0 | 0 | 0 | 0 | 0 | 0 | 0 | 0 | 0 | 0 | 0 | 0 | 0 | 2 | 1 | 0 |
| f6p\_c | 0 | 1 | -1 | 0 | 0 | 0 | 0 | 0 | 0 | 0 | 0 | 0 | 0 | 0 | 0 | 0 | 0 | 0 | 0 | 0 | 0 | 2 | 1 | 0 |
| fdp\_c | 0 | 0 | 1 | -1 | 0 | 0 | 0 | 0 | 0 | 0 | 0 | 0 | 0 | 0 | 0 | 0 | 0 | 0 | 0 | 0 | 0 | 2 | 2 | 0 |
| dhap\_c | 0 | 0 | 0 | 1 | -1 | 0 | 0 | 0 | 0 | 0 | 0 | 0 | 0 | 0 | 0 | 0 | 0 | 0 | 0 | 0 | 0 | 2 | 1 | 0 |
| g3p\_c | 0 | 0 | 0 | 1 | 1 | -1 | 0 | 0 | 0 | 0 | 0 | 0 | 0 | 0 | 0 | 0 | 0 | 0 | 0 | 0 | 0 | 3 | 1 | 0 |
| \_13dpg\_c | 0 | 0 | 0 | 0 | 0 | 1 | -1 | 0 | 0 | 0 | 0 | 0 | 0 | 0 | 0 | 0 | 0 | 0 | 0 | 0 | 0 | 2 | 2 | 0 |
| \_3pg\_c | 0 | 0 | 0 | 0 | 0 | 0 | 1 | -1 | 0 | 0 | 0 | 0 | 0 | 0 | 0 | 0 | 0 | 0 | 0 | 0 | 0 | 2 | 1 | 0 |
| \_2pg\_c | 0 | 0 | 0 | 0 | 0 | 0 | 0 | 1 | -1 | 0 | 0 | 0 | 0 | 0 | 0 | 0 | 0 | 0 | 0 | 0 | 0 | 2 | 1 | 0 |
| pep\_c | 0 | 0 | 0 | 0 | 0 | 0 | 0 | 0 | 1 | -1 | 0 | 0 | 0 | 0 | 0 | 0 | 0 | 0 | 0 | 0 | 0 | 2 | 1 | 0 |
| pyr\_c | 0 | 0 | 0 | 0 | 0 | 0 | 0 | 0 | 0 | 1 | -1 | 0 | 0 | -1 | 0 | 0 | 0 | 0 | 0 | 0 | 0 | 3 | 0 | 0 |
| lac\_\_L\_c | 0 | 0 | 0 | 0 | 0 | 0 | 0 | 0 | 0 | 0 | 1 | 0 | 0 | 0 | -1 | 0 | 0 | 0 | 0 | 0 | 0 | 2 | 0 | 0 |
| nad\_c | 0 | 0 | 0 | 0 | 0 | -1 | 0 | 0 | 0 | 0 | 1 | 0 | 0 | 0 | 0 | 0 | 1 | 0 | 0 | 0 | 0 | 3 | 0 | 1 |
| nadh\_c | 0 | 0 | 0 | 0 | 0 | 1 | 0 | 0 | 0 | 0 | -1 | 0 | 0 | 0 | 0 | 0 | -1 | 0 | 0 | 0 | 0 | 3 | 0 | 1 |
| amp\_c | 0 | 0 | 0 | 0 | 0 | 0 | 0 | 0 | 0 | 0 | 0 | -1 | 1 | 0 | 0 | 0 | 0 | 0 | 1 | 0 | 0 | 3 | 0 | 0 |
| adp\_c | 1 | 0 | 1 | 0 | 0 | 0 | -1 | 0 | 0 | -1 | 0 | 0 | -2 | 0 | 0 | 1 | 0 | 0 | 0 | 0 | 0 | 6 | 1 | 0 |
| atp\_c | -1 | 0 | -1 | 0 | 0 | 0 | 1 | 0 | 0 | 1 | 0 | 0 | 1 | 0 | 0 | -1 | 0 | 0 | 0 | 0 | 0 | 6 | 2 | 0 |
| pi\_c | 0 | 0 | 0 | 0 | 0 | -1 | 0 | 0 | 0 | 0 | 0 | 0 | 0 | 0 | 0 | 1 | 0 | 0 | 0 | 0 | 0 | 2 | 1 | 0 |
| h\_c | 1 | 0 | 1 | 0 | 0 | 1 | 0 | 0 | 0 | -1 | -1 | 0 | 0 | 0 | 0 | 1 | 1 | 0 | 0 | -1 | 0 | 8 | 0 | 0 |
| h2o\_c | 0 | 0 | 0 | 0 | 0 | 0 | 0 | 0 | 1 | 0 | 0 | 0 | 0 | 0 | 0 | -1 | 0 | 0 | 0 | 0 | -1 | 3 | 0 | 0 |
| $\pi\_{j}$ | 5 | 2 | 5 | 3 | 2 | 6 | 4 | 2 | 3 | 5 | 5 | 1 | 3 | 1 | 1 | 5 | 3 | 1 | 1 | 1 | 1 |  |  |  |
| C | 0 | 0 | 0 | 0 | 0 | 0 | 0 | 0 | 0 | 0 | 0 | -10 | 0 | -3 | -3 | 0 | 0 | 6 | 10 | 0 | 0 |  |  |  |
| H | 0 | 0 | 0 | 0 | 0 | 0 | 0 | 0 | 0 | 0 | 0 | -12 | 0 | -3 | -5 | 0 | 0 | 12 | 12 | -1 | -2 |  |  |  |
| O | 0 | 0 | 0 | 0 | 0 | 0 | 0 | 0 | 0 | 0 | 0 | -7 | 0 | -3 | -3 | 0 | 0 | 6 | 7 | 0 | -1 |  |  |  |
| P | 0 | 0 | 0 | 0 | 0 | 0 | 0 | 0 | 0 | 0 | 0 | -1 | 0 | 0 | 0 | 0 | 0 | 0 | 1 | 0 | 0 |  |  |  |
| N | 0 | 0 | 0 | 0 | 0 | 0 | 0 | 0 | 0 | 0 | 0 | -5 | 0 | 0 | 0 | 0 | 0 | 0 | 5 | 0 | 0 |  |  |  |
| S | 0 | 0 | 0 | 0 | 0 | 0 | 0 | 0 | 0 | 0 | 0 | 0 | 0 | 0 | 0 | 0 | 0 | 0 | 0 | 0 | 0 |  |  |  |
| q | 0 | 0 | 0 | 0 | 0 | 0 | 0 | 0 | 0 | 0 | 0 | 2 | 0 | 1 | 1 | 0 | 2 | 0 | -2 | -1 | 0 |  |  |  |
| [NAD] | 0 | 0 | 0 | 0 | 0 | 0 | 0 | 0 | 0 | 0 | 0 | 0 | 0 | 0 | 0 | 0 | 0 | 0 | 0 | 0 | 0 |  |  |  |
| $p\_1$ | 1 | 1 | 1 | 1 | 1 | 2 | 2 | 2 | 2 | 2 | 2 | 0 | 0 | 0 | 2 | 2 | 0 | 1 | 0 | 2 | 0 |  |  |  |
| $p\_2$ | 0 | 0 | 0 | 0 | 0 | 0 | 0 | 0 | 0 | 0 | -1 | 0 | 0 | 1 | -1 | 0 | 1 | 0 | 0 | 2 | 0 |  |  |  |
| $p\_3$ | 0 | 0 | 0 | 0 | 0 | 0 | 0 | 0 | 0 | 0 | 0 | 1 | 0 | 0 | 0 | 0 | 0 | 0 | 1 | 0 | 0 |  |  |  |

##### Defining the Steady State¶

In the last section, we defined the topological structure of the glycolytic system. We formulated its stoichiometric matrix and performed quality controls on it. We determined its topological properties, namely the pathways and the invariant pools, by looking at the basis vectors for the null spaces. To perform a simulation we need supply appropriate data and compute the pseudo-elementary rate constants (PERCs).

Numerical values for the concentrations and exchange fluxes need to be obtained experimentally. We will now introduce, 1) experimental flux data to determine the steady state flux map, and 2) steady state metabolite concentrations that will lead to the computation of the PERCs. Note that the PERCs are condition-dependent as they depend on the flux and concentration measurements. PERCs are phenomenological coefficients that allow us to *“To organize disparate information into a coherent whole.”*
(Baily’s motivation #1, see Chapter 1)

###### Computing the steady state flux vector¶

Flux and concentration data for glycolysis are available for several organisms and cell types. Here, we will use data from the historical human red blood cell (RBC) metabolic model (Heinrich 1977, Jamshidi 2002, Joshi 1988, Joshi 1990, Joshi 1989-II) as an example. The null space of \(\textbf{S}\) is three dimensional. Thus, the specification of three independent fluxes allows the determination of the unique steady state flux state. The steady state flux distribution is a combination of the
three pathway vectors.

A typical uptake rate of the RBC of glucose is about 1.12 mM/hr. This number specifies the length of the first pathway vector, \(\textbf{p}\_1\), in the steady state solution, \(\textbf{v}\_{stst}\). Based on experimental data, the steady state load on NADH is set at 20% of the glucose uptake rate, or \(0.2 \* 1.12 = 0.244.\) This number specifies the length of the second pathway vector, \(\textbf{p}\_2\), in the steady state flux vector. The synthesis rate of AMP is measured to be
0.014 mM/hr. This number specifies the length of the third pathway vector, \(\textbf{p}\_3\), in the steady state flux vector. The steady state flux vector is the weighted sum of the corresponding basis vectors. The steady state flux vector is computed as an inner product:

\[\begin{equation} \textbf{v}\_{\mathrm{stst}} = 1.12\textbf{p}\_1 + 0.224\textbf{p}\_2 + 0.014\textbf{p}\_3 \tag{10.3} \end{equation}\]

```
[17]:
```

```
# Set independent fluxes to determine steady state flux vector
independent_fluxes = {
    glycolysis.reactions.SK_glc__D_c: 1.12,
    glycolysis.reactions.DM_nadh: .2*1.12,
    glycolysis.reactions.SK_amp_c: 0.014}

# Compute steady state fluxes
ssfluxes = glycolysis.compute_steady_state_fluxes(
    minspan_paths,
    independent_fluxes,
    update_reactions=True)

table_10_9 = pd.DataFrame(list(ssfluxes.values()), index=reaction_ids,
                          columns=[r"$\textbf{v}_{\mathrm{stst}}$"]).T
```

**Table 10.9:** Computing the steady state fluxes as a summation of the MinSpan pathway vectors.

```
[18]:
```

```
table_10_9
```

```
[18]:
```

|  | HEX1 | PGI | PFK | FBA | TPI | GAPD | PGK | PGM | ENO | PYK | LDH\_L | DM\_amp\_c | ADK1 | SK\_pyr\_c | SK\_lac\_\_L\_c | ATPM | DM\_nadh | SK\_glc\_\_D\_c | SK\_amp\_c | SK\_h\_c | SK\_h2o\_c |
| --- | --- | --- | --- | --- | --- | --- | --- | --- | --- | --- | --- | --- | --- | --- | --- | --- | --- | --- | --- | --- | --- |
| $\textbf{v}\_{\mathrm{stst}}$ | 1.120 | 1.120 | 1.120 | 1.120 | 1.120 | 2.240 | 2.240 | 2.240 | 2.240 | 2.240 | 2.016 | 0.014 | 0.000 | 0.224 | 2.016 | 2.240 | 0.224 | 1.120 | 0.014 | 2.688 | 0.000 |

and can be visualized as a bar chart:

```
[19]:
```

```
fig_10_3, ax = plt.subplots(nrows=1, ncols=1, figsize=(10, 5))
# Define indicies for bar chart
indicies = np.arange(len(reaction_ids))+0.5
# Define colors to use
c = plt.cm.coolwarm(np.linspace(0, 1, len(reaction_ids)))
# Plot bar chart
ax.bar(indicies, ssfluxes.values(), width=0.8, color=c);
ax.set_xlim([0, len(reaction_ids)]);
# Set labels and adjust ticks
ax.set_xticks(indicies);
ax.set_xticklabels(reaction_ids, rotation="vertical");
ax.set_ylabel("Fluxes (mM/hr)", L_FONT);
ax.set_title("Steady State Fluxes", L_FONT);
fig_10_3.tight_layout()
```

**Figure 10.3:** Bar chart of the steady-state fluxes.

Note that there is a net production of a proton, leading to acidification of the surrounding medium, but no net production of water. We can perform a numerical check make sure that we have a steady state flux vector by performing the multiplication \(\textbf{Sv}\_{\mathrm{stst}}\) that should yield zero.

**A numerical QC/QA**: Ensure \(\textbf{Sv}\_{\mathrm{stst}} = 0.\)

```
[20]:
```

```
pd.DataFrame(
    glycolysis.S.dot(np.array(list(ssfluxes.values()))),
    index=metabolite_ids,
    columns=[r"$\textbf{Sv}_{\mathrm{stst}}$"],
    dtype=np.int64).T
```

```
[20]:
```

|  | glc\_\_D\_c | g6p\_c | f6p\_c | fdp\_c | dhap\_c | g3p\_c | \_13dpg\_c | \_3pg\_c | \_2pg\_c | pep\_c | pyr\_c | lac\_\_L\_c | nad\_c | nadh\_c | amp\_c | adp\_c | atp\_c | pi\_c | h\_c | h2o\_c |
| --- | --- | --- | --- | --- | --- | --- | --- | --- | --- | --- | --- | --- | --- | --- | --- | --- | --- | --- | --- | --- |
| $\textbf{Sv}\_{\mathrm{stst}}$ | 0 | 0 | 0 | 0 | 0 | 0 | 0 | 0 | 0 | 0 | 0 | 0 | 0 | 0 | 0 | 0 | 0 | 0 | 0 | 0 |

###### Computing the PERCs¶

Glycolysis contains both reversible and effectively irreversible reactions. The approximate steady state values of the metabolites are given above. The mass action ratios can be computed from these steady state concentrations and are all smaller than the corresponding equilibrium constants (i.e., \(\Gamma < K\_{eq}\)), and thus the reactions are proceeding in the forward direction. We can compute the forward rate constant for reaction i from

\[\begin{equation} k\_i = \frac{\text{flux through reaction}}{(\Pi\_i\text{reactants}\_i - \Pi\_i\text{products}\_i/K\_{eq})} \tag{10.4} \end{equation}\]

```
[21]:
```

```
percs = glycolysis.calculate_PERCs(update_reactions=True)
```

**Table 10.10:** Glycolytic enzymes, loads, transport rates, and their abbreviations. In addition, the glucose influx rate is set at 1.12 mM/hr and the AMP influx rate is set at 0.014 mM/hr based on data, see (Joshi 1988). For irreversible reactions, the numerical value for the equilibrium constants is \(\infty\), which, for practical reasons, can be set to a finite value.

```
[22]:
```

```
# Get concentration values for substitution into sympy expressions
value_dict = {sym.Symbol(str(met)): ic
              for met, ic in glycolysis.initial_conditions.items()}
value_dict.update({sym.Symbol(str(met)): bc
                   for met, bc in glycolysis.boundary_conditions.items()})

table_10_10 = []
# Get symbols and values for table and substitution
for p_key in ["Keq", "kf"]:
    symbol_list, value_list = [], []
    for p_str, value in glycolysis.parameters[p_key].items():
        symbol_list.append(r"$%s_{\text{%s}}$" % (p_key[0], p_str.split("_", 1)[-1]))
        value_list.append("{0:.3f}".format(value) if value != INF else r"$\infty$")
        value_dict.update({sym.Symbol(p_str): value})
    table_10_10.extend([symbol_list, value_list])

table_10_10.append(["{0:.6f}".format(float(ratio.subs(value_dict)))
                    for ratio in strip_time(glycolysis.get_mass_action_ratios()).values()])
table_10_10.append(["{0:.6f}".format(float(ratio.subs(value_dict)))
                    for ratio in strip_time(glycolysis.get_disequilibrium_ratios()).values()])
table_10_10 = pd.DataFrame(np.array(table_10_10).T, index=reaction_ids,
                           columns=[r"$K_{eq}$ Symbol", r"$K_{eq}$ Value", "PERC Symbol",
                                    "PERC Value", r"$\Gamma$", r"$\Gamma/K_{eq}$"])
table_10_10
```

```
[22]:
```

|  | $K\_{eq}$ Symbol | $K\_{eq}$ Value | PERC Symbol | PERC Value | $\Gamma$ | $\Gamma/K\_{eq}$ |
| --- | --- | --- | --- | --- | --- | --- |
| HEX1 | $K\_{\text{HEX1}}$ | 850.000 | $k\_{\text{HEX1}}$ | 0.700 | 0.008809 | 0.000010 |
| PGI | $K\_{\text{PGI}}$ | 0.410 | $k\_{\text{PGI}}$ | 3644.444 | 0.407407 | 0.993677 |
| PFK | $K\_{\text{PFK}}$ | 310.000 | $k\_{\text{PFK}}$ | 35.369 | 0.133649 | 0.000431 |
| FBA | $K\_{\text{FBA}}$ | 0.082 | $k\_{\text{FBA}}$ | 2834.568 | 0.079781 | 0.972937 |
| TPI | $K\_{\text{TPI}}$ | 0.057 | $k\_{\text{TPI}}$ | 34.356 | 0.045500 | 0.796249 |
| GAPD | $K\_{\text{GAPD}}$ | 0.018 | $k\_{\text{GAPD}}$ | 3376.749 | 0.006823 | 0.381183 |
| PGK | $K\_{\text{PGK}}$ | 1800.000 | $k\_{\text{PGK}}$ | 1273531.270 | 1755.073081 | 0.975041 |
| PGM | $K\_{\text{PGM}}$ | 0.147 | $k\_{\text{PGM}}$ | 4868.589 | 0.146184 | 0.994048 |
| ENO | $K\_{\text{ENO}}$ | 1.695 | $k\_{\text{ENO}}$ | 1763.741 | 1.504425 | 0.887608 |
| PYK | $K\_{\text{PYK}}$ | 363000.000 | $k\_{\text{PYK}}$ | 454.386 | 19.570304 | 0.000054 |
| LDH\_L | $K\_{\text{LDH\_L}}$ | 26300.000 | $k\_{\text{LDH\_L}}$ | 1112.574 | 44.132974 | 0.001678 |
| DM\_amp\_c | $K\_{\text{DM\_amp\_c}}$ | $\infty$ | $k\_{\text{DM\_amp\_c}}$ | 0.161 | 11.530288 | 0.000000 |
| ADK1 | $K\_{\text{ADK1}}$ | 1.650 | $k\_{\text{ADK1}}$ | 100000.000 | 1.650000 | 1.000000 |
| SK\_pyr\_c | $K\_{\text{SK\_pyr\_c}}$ | 1.000 | $k\_{\text{SK\_pyr\_c}}$ | 744.186 | 0.995008 | 0.995008 |
| SK\_lac\_\_L\_c | $K\_{\text{SK\_lac\_\_L\_c}}$ | 1.000 | $k\_{\text{SK\_lac\_\_L\_c}}$ | 5.600 | 0.735294 | 0.735294 |
| ATPM | $K\_{\text{ATPM}}$ | $\infty$ | $k\_{\text{ATPM}}$ | 1.400 | 0.453125 | 0.000000 |
| DM\_nadh | $K\_{\text{DM\_nadh}}$ | $\infty$ | $k\_{\text{DM\_nadh}}$ | 7.442 | 1.956811 | 0.000000 |
| SK\_glc\_\_D\_c | $K\_{\text{SK\_glc\_\_D\_c}}$ | $\infty$ | $k\_{\text{SK\_glc\_\_D\_c}}$ | 1.120 | 1.000000 | 0.000000 |
| SK\_amp\_c | $K\_{\text{SK\_amp\_c}}$ | $\infty$ | $k\_{\text{SK\_amp\_c}}$ | 0.014 | 0.086728 | 0.000000 |
| SK\_h\_c | $K\_{\text{SK\_h\_c}}$ | 1.000 | $k\_{\text{SK\_h\_c}}$ | 100000.000 | 0.701253 | 0.701253 |
| SK\_h2o\_c | $K\_{\text{SK\_h2o\_c}}$ | 1.000 | $k\_{\text{SK\_h2o\_c}}$ | 100000.000 | 1.000000 | 1.000000 |

These estimates for the numerical values for the PERCs are shown in Table 10.10. These numerical values, along with the elementary form of the rate laws, complete the definition of the dynamic mass balances that can now be simulated. The steady state is specified in Table 10.9. Clearly, there are practical limitations for the computation of the PERCs from Eq. (10.4). If the reaction is close to equilibrium, then the denominator can be close to zero. If this is the case, the PERC is effectively
indeterminable, but we know it is fast. The PERC can then be fixed to a large value and, in almost all cases, will correspond to dynamics that are too fast to be of interest.

We note that these computations are automated in **MASSpy** using the `MassModel.calculate_PERCs` method but detailed here for illustrative purposes.

###### Node maps¶

These maps show all the flows of mass in and out of a node (a compound) in the network. Some of the key nodes in the glycolytic system are shown in Figure 10.4. The rate constants of the links into and out of a node can differ in their magnitude. They give us a measure of the spectrum of response times associated with a node. It is hard to excite some of the rapid dynamics associated with a node by perturbing a boundary flux; thus, such rapid motions are not often observed in dynamical
simulations around a steady state.

**Figure 10.4:** Node maps for key glycolytic intermediates. The node maps for \(\textit{AMP}\), \(\textit{NADH}\), \(\textit{ATP}\), \(\textit{P}\_i\), \(\textit{PYR}\), and \(\textit{H}^+\) are shown. The flows in and out balance in the steady state.

##### Simulating Mass Balances: Biochemistry¶

In the first part, we specified the glycolytic system, its contents, its steady state, and all numerical values needed to simulate its dynamic responses. Such simulations can be done for perturbations in the energy or redox loads, in environmental parameters like the external PYR and LAC concentrations, or in the influx of the two forcing functions, for glucose or AMP. Here, we will simulate the response to an increased rate of use of ATP.

###### Validating the steady state¶

As a QC/QA test, we start by simulating the glycolytic system without perturbing it to make sure that the initial conditions represent a steady state.

```
[23]:
```

```
t0, tf = (0, 1e3)
sim_glycolysis = Simulation(glycolysis)
conc_sol_ss, flux_sol_ss = sim_glycolysis.simulate(
    glycolysis, time=(t0, tf, tf*10 + 1))
conc_sol_ss.view_time_profile()
```

**Figure 10.5:** Simulating the glycolytic system from the steady state with no perturbation.

###### Response to an increased \(k\_{ATPM}\) at a constant glucose input rate¶

We focus here on a perturbation in the ATP load, where we increase the \(k\_{ATPM}\) parameter by 50% at \(t = 0\) and simulate the dynamic response to a new steady state. In Chapter 8 we studied this same perturbation in an idealized toy system. This perturbation reflects a change in the rate of usage of ATP.

```
[24]:
```

```
conc_sol, flux_sol = sim_glycolysis.simulate(
    glycolysis, time=(t0, tf, tf*10 + 1),
    perturbations={"kf_ATPM": "kf_ATPM * 1.5"})
```

```
[25]:
```

```
fig_10_6, axes = plt.subplots(nrows=2, ncols=1, figsize=(10, 6));
(ax1, ax2) = axes.flatten()

plot_time_profile(
    conc_sol, ax=ax1, legend="right outside",
    plot_function="loglog",
    xlabel="Time [hr]", ylabel="Concentration [mM]",
    title=("Concentration Profile", L_FONT));

plot_time_profile(
    flux_sol, ax=ax2, legend="right outside",
    plot_function="semilogx",
    xlabel="Time [hr]", ylabel="Flux [mM/hr]",
    title=("Flux Profile", L_FONT));
fig_10_6.tight_layout()
```

**Figure 10.6:** Simulating the glycolytic system from the steady state with 50% increase in the rate of ATP utilization at t = 0.

###### A multi-scale response¶

To get the full response, the time scale is shown on a log scale. The full time response shows that there are roughly three time scales of interest. These are consistent with distribution of numerical values of PERCs in Table 10.11. We have roughly three time scales of interest: <0.1 hr, 0.1 to 10 hr, and 10 to 100 hr. The initial perturbation imbalances a few nodes immediately, that then activates fluxes with fast time scales that then serially over time lead to dynamic responses on subsequent
time scales.

###### Relative deviations from the initial state¶

The solution shows that the concentrations are spread over many orders of magnitude. We can judge the magnitude of the perturbation from the steady state by normalizing the concentrations to their initial conditions.

```
[26]:
```

```
fig_10_7, axes = plt.subplots(nrows=2, ncols=1, figsize=(10, 6));
(ax1, ax2) = axes.flatten()

conc_deviation = {met.id: conc_sol[met.id]/ic
                  for met, ic in glycolysis.initial_conditions.items()}
conc_deviation = MassSolution(
    "Deviation", solution_type="Conc",
    data_dict=conc_deviation,
    time=conc_sol.t, interpolate=False)

flux_deviation = {rxn.id: flux_sol[rxn.id]/ssflux
                  for rxn, ssflux in glycolysis.steady_state_fluxes.items()
                  if ssflux != 0} # To avoid dividing by 0 for equilibrium fluxes.

flux_deviation = MassSolution(
    "Deviation", solution_type="Flux",
    data_dict=flux_deviation,
    time=flux_sol.t, interpolate=False)

plot_time_profile(
    conc_deviation, ax=ax1, legend="right outside",
    plot_function="semilogx", ylim=(-.5, 6),
    xlabel="Time [hr]", ylabel="Concentration [mM]",
    title=("Concentration Profile", L_FONT));

plot_time_profile(
    flux_deviation, ax=ax2, legend="right outside",
    plot_function="semilogx", ylim=(-.5, 6),
    xlabel="Time [hr]", ylabel="Flux [mM/hr]",
    title=("Flux Profile", L_FONT));
fig_10_7.tight_layout()
```

**Figure 10.7:** (a) Deviation from the steady state of the concentrations as a fraction of the steady state. (b) Deviation from the steady state of the fluxes as a fraction of the steady state.

- The concentration of AMP shows the most significant perturbation from its initial steady state. The phosphate and glucose concentrations change the most in the new steady state. Similarly, we can examine how much the fluxes deviate from their inital steady state values.
- We see that the flux map is immediately imbalanced at short times with the proton efflux \((v\_{SK\_{h}})\) and the pyruvate efflux \((v\_{SK\_{pyr}})\) taking large values relative to the steady state. The nodes that they are connected to will thus imbalance, and the perturbation of the corresponding concentration will be determined by the change in the flux relative to the initial concentration.

Since the forced unbalancing of the two inputs (glucose and AMP) are the same, the eventual flux map will balance out the same way as the pre-perturbation steady state. The eventual value of the ATP demand flux will end up at 2/3rds of its initial value since it was perturbed by 50% at \(t=0.\)

**Table 10.11:** Numerical values for the concentrations (mM) and fluxes (mM/hr) at the beginning and end of the dynamic simulation. The concentration of water is arbitrarily set at 1.0. At \(t=0\) the \(v\_{ATPM}\) flux changes from 2.24 to 3.36, unbalancing the flux map. The flux map returns to its original state as time goes to infinity (here \(t=1000\)). Note that unlike the fluxes, the concentrations reach a different steady state.

```
[27]:
```

```
met_ids = metabolite_ids.copy()
init_concs = [round(ic[0], 3) for ic in conc_sol.values()]
final_concs = [round(bc[-1], 3) for bc in conc_sol.values()]
column_labels = ["Metabolite", "Conc. at t=0 [mM]", "Conc. at t=1000 [mM]"]

rxn_ids = reaction_ids.copy()
init_fluxes = [round(ic[0], 3) for ic in flux_sol.values()]
final_fluxes = [round(bc[-1], 3) for bc in flux_sol.values()]
column_labels += ["Reactions", "Flux at t=0 [mM/hr]", "Flux at t=1000 [mM/hr]"]

# Extend metabolite columns to match length of reaction columns for table
pad = [""]*(len(reaction_ids) - len(metabolite_ids))
# Make table
table_10_11 = np.array([metabolite_ids + pad, init_concs + pad, final_concs + pad,
                        reaction_ids, init_fluxes, final_fluxes])
table_10_11 = pd.DataFrame(table_10_11.T,
                           index=[i for i in range(1, len(reaction_ids) + 1)],
                           columns=column_labels)
def highlight_table(x):
    # ATPM is the 16th reaction according to Table 10.2
    return ['color: red' if x.name == 16 else '' for v in x]

table_10_11 = table_10_11.style.apply(highlight_table, subset=column_labels[3:], axis=1)
table_10_11
```

```
[27]:
```

|  | Metabolite | Conc. at t=0 [mM] | Conc. at t=1000 [mM] | Reactions | Flux at t=0 [mM/hr] | Flux at t=1000 [mM/hr] |
| --- | --- | --- | --- | --- | --- | --- |
| 1 | glc\_\_D\_c | 1.0 | 1.5 | HEX1 | 1.12 | 1.12 |
| 2 | g6p\_c | 0.049 | 0.073 | PGI | 1.12 | 1.12 |
| 3 | f6p\_c | 0.02 | 0.03 | PFK | 1.12 | 1.12 |
| 4 | fdp\_c | 0.015 | 0.008 | FBA | 1.12 | 1.12 |
| 5 | dhap\_c | 0.16 | 0.12 | TPI | 1.12 | 1.12 |
| 6 | g3p\_c | 0.007 | 0.005 | GAPD | 2.24 | 2.24 |
| 7 | \_13dpg\_c | 0.0 | 0.0 | PGK | 2.24 | 2.24 |
| 8 | \_3pg\_c | 0.077 | 0.093 | PGM | 2.24 | 2.24 |
| 9 | \_2pg\_c | 0.011 | 0.014 | ENO | 2.24 | 2.24 |
| 10 | pep\_c | 0.017 | 0.021 | PYK | 2.24 | 2.24 |
| 11 | pyr\_c | 0.06 | 0.06 | LDH\_L | 2.016 | 2.016 |
| 12 | lac\_\_L\_c | 1.36 | 1.36 | DM\_amp\_c | 0.014 | 0.014 |
| 13 | nad\_c | 0.059 | 0.059 | ADK1 | 0.002 | -0.0 |
| 14 | nadh\_c | 0.03 | 0.03 | SK\_pyr\_c | 0.224 | 0.224 |
| 15 | amp\_c | 0.087 | 0.087 | SK\_lac\_\_L\_c | 2.016 | 2.016 |
| 16 | adp\_c | 0.29 | 0.237 | ATPM | 3.36 | 2.24 |
| 17 | atp\_c | 1.6 | 1.067 | DM\_nadh | 0.224 | 0.224 |
| 18 | pi\_c | 2.5 | 3.62 | SK\_glc\_\_D\_c | 1.12 | 1.12 |
| 19 | h\_c | 0.0 | 0.0 | SK\_amp\_c | 0.014 | 0.014 |
| 20 | h2o\_c | 1.0 | 1.0 | SK\_h\_c | 2.688 | 2.688 |
| 21 |  |  |  | SK\_h2o\_c | 0.0 | -0.0 |

**A numerical QC/QA:** We can make sure that we have the steady state flux map by computing the flux balances as \(\textbf{Sv}\) and determine if they are zero.

```
[28]:
```

```
final_fluxes = [round(bc[-1], 3) for bc in flux_sol.values()]
Sv_qcqa = np.array([[round(value, 5) for value in glycolysis.S.dot(final_fluxes)]])
Sv_qcqa = pd.DataFrame(Sv_qcqa, index=["Sv after perturbation"], columns=metabolite_ids, dtype=np.int64)
Sv_qcqa
```

```
[28]:
```

|  | glc\_\_D\_c | g6p\_c | f6p\_c | fdp\_c | dhap\_c | g3p\_c | \_13dpg\_c | \_3pg\_c | \_2pg\_c | pep\_c | pyr\_c | lac\_\_L\_c | nad\_c | nadh\_c | amp\_c | adp\_c | atp\_c | pi\_c | h\_c | h2o\_c |
| --- | --- | --- | --- | --- | --- | --- | --- | --- | --- | --- | --- | --- | --- | --- | --- | --- | --- | --- | --- | --- |
| Sv after perturbation | 0 | 0 | 0 | 0 | 0 | 0 | 0 | 0 | 0 | 0 | 0 | 0 | 0 | 0 | 0 | 0 | 0 | 0 | 0 | 0 |

###### Node balances¶

Although the response is not high dimensional, it is difficult to conceptualize. We begin to untangle the dynamic state by examining the node balances. The sudden increase in the usage rate of the ATP demand reaction: \(\text{ATP} + \text{H}\_2\text{O} \rightarrow \text{ADP} + \text{P}\_i + \text{H}\), there is a sudden increase in all the products of the reactions that in turn cause perturbations through the network. We now look at the node balances for each of these products.

###### The proton node¶

The proton node is imbalanced at time zero with the increased flux of the ATP demand reaction. The proton node has a connectivity of 8; five production reactions and three utilization reactions. We can look at the node balance at the beginning of the simulation, i.e. the imbalanced state, and compare it with the balanced state at the end of the simulation.

The fluxes in and out of the node can be graphed, and so can the proton concentration (the ‘inventory’ in the node).

```
[29]:
```

```
h_c = glycolysis.metabolites.h_c
fluxes_in = []
fluxes_out = []
for reaction in glycolysis.reactions:
    if h_c in reaction.reactants:
        fluxes_out.append(reaction.id)
    if h_c in reaction.products:
        fluxes_in.append(reaction.id)

print(fluxes_in, "Produce\n")
print(fluxes_out, "Consume\n")
```

```
['HEX1', 'PFK', 'GAPD', 'ATPM', 'DM_nadh'] Produce

['PYK', 'LDH_L', 'SK_h_c'] Consume
```

```
[30]:
```

```
fig_10_8 = plt.figure(figsize=(17, 6))
gs = fig_10_8.add_gridspec(nrows=3, ncols=2, width_ratios=[1.5, 1])

ax1 = fig_10_8.add_subplot(gs[0, 0])
ax2 = fig_10_8.add_subplot(gs[1, 0])
ax3 = fig_10_8.add_subplot(gs[2, 0])
ax4 = fig_10_8.add_subplot(gs[:, 1])

plot_time_profile(
    conc_sol, observable="h_c", ax=ax1,
    legend="right outside", plot_function="semilogx",
    xlim=(1e-6, tf), ylim=(8.5e-5, 1e-4*1.025),
    xlabel="Time [hr]", ylabel="Concentrations [mM]",
    title=("(a) Proton Concentration", L_FONT));

fluxes_in = ["HEX1", "PFK","GAPD", "ATPM", "DM_nadh"]
plot_time_profile(
    flux_sol, observable=fluxes_in, ax=ax2,
    legend="right outside", plot_function="semilogx",
    xlim=(1e-6, tf),  ylim=(0, 4),
    xlabel="Time [hr]", ylabel="Fluxes [mM/hr]",
    title=("(b) Fluxes in", L_FONT));

fluxes_out = ["PYK", "LDH_L", "SK_h_c"]
plot_time_profile(
    flux_sol, observable=fluxes_out, ax=ax3,
    legend="right outside", plot_function="semilogx",
    xlim=(1e-6, tf),  ylim=(1.5, 4),
    xlabel="Time [hr]", ylabel="Fluxes [mM/hr]",
    title=("(c) Fluxes out", L_FONT));

for flux_id, variables in zip(["Net_Flux_In", "Net_Flux_Out"],
                              [fluxes_in, fluxes_out]):
    flux_sol.make_aggregate_solution(
        flux_id, equation=" + ".join(variables), variables=variables)

time_points = [t0, 1e-1, 1e0, 1e1, 1e2, tf]
time_point_colors = [
    mpl.colors.to_hex(c)
    for c in mpl.cm.Reds(np.linspace(0.3, 1, len(time_points)))]

plot_phase_portrait(
    flux_sol, x="Net_Flux_In", y="Net_Flux_Out", ax=ax4,
    xlim=(5.5, 8.5), ylim=(5.5, 8.5),
    xlabel="Fluxes in [mm/Hr]", ylabel="Fluxes out [mm/Hr]",
    title=("(d) Phase Portrait of Fluxes", L_FONT),
    annotate_time_points=time_points,
    annotate_time_points_color=time_point_colors,
    annotate_time_points_legend="best");

ax4.plot([5.0, 9.0], [5.0, 9.0], ls="--", color="black", alpha=0.5)
xy = (flux_sol["Net_Flux_In"][0], flux_sol["Net_Flux_Out"][0])
xytext = (sum([flux_sol_ss[rxn][-1] for rxn in fluxes_in]),
          sum([flux_sol_ss[rxn][-1] for rxn in fluxes_out]))
ax4.annotate("Steady-state line:", xy=(0.63, 0.95), xycoords="axes fraction");
ax4.annotate("Flux Out < Flux In", xy=(0.7, 0.3), xycoords="axes fraction");
ax4.annotate("Flux Out > Flux In", xy=(0.3, 0.7), xycoords="axes fraction");
ax4.annotate("      initial perturbation\n", xy=xy, xytext=xytext, textcoords="data");
ax4.annotate("", xy=xy, xytext=xytext, textcoords="data",
             arrowprops=dict(arrowstyle="->", connectionstyle="arc3"));
fig_10_8.tight_layout()
```

**Figure 10.8:** The time profiles of the (a) proton concentration, (b) the fluxes that make protons, (c) the fluxes that use protons and (d) the phase portrait of the net flux in and net flux out (darker red colors indicate slower time scales).

The ATP load reaction initially imbalances the node, and the proton efflux reacts to the proton fluctuation. We thus look at a phase plane representing these two fluxes.

```
[31]:
```

```
fig_10_9, ax = plt.subplots(nrows=1, ncols=1, figsize=(7, 5))

plot_phase_portrait(
    flux_sol, x="ATPM", y="SK_h_c", ax=ax,
    xlabel="ATPM [mm/Hr]", ylabel="SK_h_c [mm/Hr]", xlim=(1.8, 4), ylim=(1.8, 4),
    title=("Phase Portrait of ATPM vs. SK_h_c", L_FONT),
    annotate_time_points=time_points,
    annotate_time_points_color=time_point_colors,
    annotate_time_points_legend="right outside")

xy = (flux_sol["ATPM"][0], flux_sol["SK_h_c"][0])
xytext = (flux_sol_ss["ATPM"][-1], flux_sol_ss["SK_h_c"][-1])
ax.annotate("              initial perturbation", xy=xy, xytext=tuple(i-.1 for i in xytext), textcoords="data");
ax.annotate("", xy=xy, xytext=xytext, textcoords="data",
             arrowprops=dict(arrowstyle="->", connectionstyle="arc3"));
fig_10_9.tight_layout()
```

**Figure 10.9:** A phase portrait of the instantly imbalanced ATP demand flux and the proton exchange rate.

The ATP load is instantaneously moved to the \(t\_0\) point (i.e. changed from 2.24 to 3.36mM/hr). The very rapidly responsive proton efflux immediately increases to pump out the proton and then the phase portrait moves approximately along an affine 45 degree line (offset by 0.3 mM/hr), as the proton efflux adjusts to the ATP load reaction. The initial rapid response of the proton efflux is likely to be an unrealistic response, as there is substantial intracellular buffer for protons.

###### The ATP node¶

The ATP and the opposite ADP node has a connectivity of 6; three producing reactions and three utilization reactions. The sudden imbalance between ATP use and ATP production initially drops the ATP concentration. The flux through the ATP utilizing kinases (HEX1 and PFK) in upper glycolysis thus goes down, with a mirrored reaction of the kinases in lower glycolysis (PGK and PYK). This dynamic interplay leads to ultimate steady state where ATP is at a lower concentration and so interestingly is
ADP. The reason is that ADK1 forms AMP initially when ADP builds up and this leads to loss of AMP from the system.

```
[32]:
```

```
fig_10_10 = plt.figure(figsize=(17, 6))
gs = fig_10_10.add_gridspec(nrows=3, ncols=2, width_ratios=[1.5, 1])

ax1 = fig_10_10.add_subplot(gs[0, 0])
ax2 = fig_10_10.add_subplot(gs[1, 0])
ax3 = fig_10_10.add_subplot(gs[2, 0])
ax4 = fig_10_10.add_subplot(gs[:, 1])

plot_time_profile(
    conc_sol, observable="atp_c", ax=ax1,
    legend="right outside", plot_function="semilogx",
    xlim=(1e-6, tf), ylim=(.75, 2),
    xlabel="Time [hr]", ylabel="Concentrations [mM]",
    title=("(a) ATP Concentration", L_FONT));

fluxes_in = ["PGK", "PYK","ADK1"]
plot_time_profile(
    flux_sol, observable=fluxes_in, ax=ax2,
    legend="right outside", plot_function="semilogx",
    xlim=(1e-6, tf), ylim=(-0.1, 2.5),
    xlabel="Time [hr]", ylabel="Fluxes [mM/hr]",
    title=("(b) Fluxes in", L_FONT));

fluxes_out = ["HEX1", "PFK", "ATPM"]
plot_time_profile(
    flux_sol, observable=fluxes_out, ax=ax3,
    legend="right outside", plot_function="semilogx",
    xlim=(1e-6, tf), ylim=(.8, 3.5),
    xlabel="Time [hr]", ylabel="Fluxes [mM/hr]",
    title=("(c) Fluxes out", L_FONT));

for flux_id, variables in zip(["Net_Flux_In", "Net_Flux_Out"],
                              [fluxes_in, fluxes_out]):
    flux_sol.make_aggregate_solution(
        flux_id, equation=" + ".join(variables), variables=variables)

time_points = [t0, 1e-1, 1e0, 1e1, 1e2, tf]
time_point_colors = [
    mpl.colors.to_hex(c)
    for c in mpl.cm.Reds(np.linspace(0.3, 1, len(time_points)))]

plot_phase_portrait(
    flux_sol, x="Net_Flux_In", y="Net_Flux_Out", ax=ax4,
    xlim=(3.5, 6.0), ylim=(3.5, 6.0),
    xlabel="Fluxes in [mm/Hr]", ylabel="Fluxes out [mm/Hr]",
    title=("(d) Phase Portrait of Fluxes", L_FONT),
    annotate_time_points=time_points,
    annotate_time_points_color=time_point_colors,
    annotate_time_points_legend="right outside");


ax4.plot((3.5, 6.0), (3.5, 6.0), ls="--", color="black", alpha=0.5)
xy = (flux_sol["Net_Flux_In"][0], flux_sol["Net_Flux_Out"][0])
xytext = (sum([flux_sol_ss[rxn][-1] for rxn in fluxes_in]),
          sum([flux_sol_ss[rxn][-1] for rxn in fluxes_out]))
ax4.annotate("Steady-state line:", xy=(0.63, 0.95), xycoords="axes fraction");
ax4.annotate("Flux Out < Flux In", xy=(0.7, 0.05), xycoords="axes fraction");
ax4.annotate("Flux Out > Flux In", xy=(0.05, 0.9), xycoords="axes fraction");
ax4.annotate("initial\nperturbation", xy=xy, xytext=(4, 5.2), textcoords="data");
ax4.annotate("", xy=xy, xytext=xytext, textcoords="data",
             arrowprops=dict(arrowstyle="->", connectionstyle="arc3"));
fig_10_10.tight_layout()
```

###### The AMP node¶

The AMP node has a connectivity of 3. The influx is fixed at 0.014 mM/hr, the exit rate of AMP is proportional to its concentration, and ADK1 establishes a quasi-equilibrium state between ATP, ADP and AMP. Thus by plotting a phase plane of \(v\_{ATPM}\) and \(v\_{DM\_{AMP}}\) we can relate the three fluxes. After the imbalancing of the ATP demand reaction AMP builds up that leads to the efflux being higher than the input rate, Figure 10.11a. This can also be seen in a phase portrait of the
ADK1 and AMP exit flux, Figure 10.11b.

```
[33]:
```

```
fig_10_11, axes = plt.subplots(nrows=1, ncols=2, figsize=(11, 5))
(ax1, ax2) = axes.flatten()

plot_phase_portrait(
    flux_sol, x="ATPM", y="DM_amp_c", ax=ax1,
    xlim=(1.5, 3.5), ylim=(0, 0.09),
    xlabel="ATPM [mm/Hr]", ylabel="DM_amp_c [mm/Hr]",
    title=("Phase Portrait of ATPM vs. DM_amp_c", L_FONT),
    annotate_time_points=time_points,
    annotate_time_points_color=time_point_colors);

# Annotate plot
ax1.plot((1.5, 3.5), [flux_sol_ss["DM_amp_c"][-1]]*2,
         ls="--", color="black");
ax1.annotate("net AMP\ngain", xy=(1.7, flux_sol_ss["DM_amp_c"][-1]*1.2));
ax1.annotate("net AMP\ndrain", xy=(1.7, flux_sol_ss["DM_amp_c"][-1]*.5));

xy = (flux_sol["ATPM"][0], flux_sol["DM_amp_c"][0])
xytext = (flux_sol_ss["ATPM"][-1], flux_sol_ss["DM_amp_c"][-1])

ax1.annotate("          initial perturbation\n",
             xy=xy, xytext=tuple(i for i in xytext),
             textcoords="data");
ax1.annotate("", xy=xy, xytext=xytext, textcoords="data",
             arrowprops=dict(arrowstyle="->", connectionstyle="arc3"));

plot_phase_portrait(
    flux_sol, x="ADK1", y="DM_amp_c", ax=ax2,
    xlim=(-0.1, 0.35), ylim=(0, 0.08),
    xlabel="ADK1 [mm/Hr]", ylabel="DM_amp_c [mm/Hr]",
    title=("Phase Portrait of ADK1 vs. DM_amp_c", L_FONT),
    annotate_time_points=time_points,
    annotate_time_points_color=time_point_colors,
    annotate_time_points_legend="right outside");
fig_10_11.tight_layout()
```

**Figure 10.11:** Illustrating the AMP node response through flux phase portraits. (a) Phase portrait of \(v\_{ATPM}\) vs. \(v\_{DM\_{AMP}}\). (b) Phase portrait of \(v\_{ADK1}\) vs. \(v\_{DM\_{AMP}}\)\*

###### The inorganic phosphate node¶

The \(\text{P}\_i\) node has a connectivity of 2; one input and one output. The two fluxes can be plotted as a function of time, or on a phase portrait. The imbalancing of the phosphate pool is initially met by a slight increase in phosphate incorporation by GAPD. That is short lived as the flux in upper glycolysis drops. Thus this node takes a long time to get into balance leading to substantial increase in free inorganic phosphate concentration. Since the total phosphate is a constant in
the system, this leads to systemic constraints on the total concentration of all phosphorylated compounds.

```
[34]:
```

```
fig_10_12 = plt.figure(figsize=(17, 6))
gs = fig_10_12.add_gridspec(nrows=2, ncols=2, width_ratios=[1.5, 1])

ax1 = fig_10_12.add_subplot(gs[0, 0])
ax2 = fig_10_12.add_subplot(gs[1, 0])
ax3 = fig_10_12.add_subplot(gs[:, 1])

plot_time_profile(
    conc_sol, observable="pi_c", ax=ax1,
    legend="right outside", plot_function="semilogx",
    xlim=(1e-6, tf), ylim=(2, 4),
    xlabel="Time [hr]", ylabel="Concentrations [mM]",
    title=("(a) Phosphate Concentration", L_FONT));

plot_time_profile(
    flux_sol, observable=["GAPD", "ATPM"], ax=ax2,
    legend="right outside", plot_function="semilogx",
    xlim=(1e-6, tf), ylim=(1.5, 3.5),
    xlabel="Time [hr]", ylabel="Fluxes [mM/hr]",
    title=("(b) Fluxes Affecting Phosphate", L_FONT));

plot_phase_portrait(
    flux_sol, x="ATPM", y="GAPD", ax=ax3,
    xlim=(1.5, 3.5), ylim=(1.5, 3.5),
    xlabel="ATPM [mm/Hr]", ylabel="GAPD [mm/Hr]",
    title=("(c) Phase Portrait of ATPM vs. GAPD", L_FONT),
    annotate_time_points=time_points,
    annotate_time_points_color=time_point_colors,
    annotate_time_points_legend="right outside");

ax3.plot((1.5, 3.5), (1.5, 3.5), ls="--", color="black", alpha=0.5)
xy = (flux_sol["ATPM"][0], flux_sol["GAPD"][0])
xytext = (flux_sol_ss["ATPM"][-1], flux_sol_ss["GAPD"][-1])
ax3.annotate("Steady-state line:", xy=(0.63, 0.95),
             xycoords="axes fraction");
ax3.annotate("Flux Out < Flux In", xy=(0.7, 0.05),
             xycoords="axes fraction");
ax3.annotate("Flux Out > Flux In", xy=(0.05, 0.9),
             xycoords="axes fraction");
ax3.annotate("initial perturbation", xy=xy, xytext=(2.5, 2.3),
             textcoords="data");
ax3.annotate("", xy=xy, xytext=xytext, textcoords="data",
             arrowprops=dict(arrowstyle="->", connectionstyle="arc3"));
fig_10_12.tight_layout()
```

**Figure 10.12:** The time profiles of the (a) inorganic phosphate concentration, (b) the two fluxes that make and consume inorganic phosphate, and (c) the phase portrait of the net flux in and net flux out (darker red colors indicate slower time scales).

###### Key Fluxes and all pairwise phase portraits¶

Figure 10.13 and Figure 10.14 are set up to allow the reader to examine all pairwise phase portraits After browsing through many of them, you will find that they resemble each other, showing that the variables move in a highly coordinated manner. We can study the relationship between many variables at once using multi-variate statistics.

```
[35]:
```

```
fig_10_13, ax = plt.subplots(nrows=1, ncols=1, figsize=(20, 22))
plot_tiled_phase_portraits(
    conc_sol, ax=ax, annotate_time_points_legend="lower outside");
fig_10_13.tight_layout()
```

**Figure 10.13:** Phase portraits of all the glycolytic species.

```
[36]:
```

```
fig_10_14, ax = plt.subplots(nrows=1, ncols=1, figsize=(20, 22))
plot_tiled_phase_portraits(
    flux_sol, ax=ax, annotate_time_points_legend="lower outside");
fig_10_14.tight_layout()
```

**Figure 10.14:** Phase portraits of all the glycolytic fluxes.

You can quickly figure out the relationships between the kinases as a group and the two dehydrogenases. You can also figure out why pyruvate efflux has a sudden increase by looking at the pyruvate node.

```
[37]:
```

```
fig_10_15, axes = plt.subplots(nrows=2, ncols=3, figsize=(17, 10))
axes = axes.flatten()

time_points = [t0, 1e-1, 1e0, 1e1, 1e2, tf]
time_point_colors = [
    mpl.colors.to_hex(c)
    for c in mpl.cm.Reds(np.linspace(0.3, 1, len(time_points)))]

pairings = [
    ("ATPM", "DM_amp_c"), ("ATPM", "SK_pyr_c"), ("DM_amp_c", "SK_pyr_c"),
    ("GAPD", "LDH_L"), ("LDH_L", "DM_nadh"), ("HEX1", "PYK")]
xlims = [
    (1.80, 3.40), (1.80, 3.40), (0.00, 0.08),
    (1.70, 2.45), (1.55, 2.200), (0.88, 1.22)]
ylims = [
    (0.00, 0.08), (0.17, 0.31), (0.17, 0.31),
    (1.55, 2.20), (0.17, 0.245), (1.70, 2.45)]

for i, ax in enumerate(axes):
    x_i, y_i = pairings[i]
    # Create a legend for the points of interest
    if i == len(axes) - 1:
        legend="upper right outside"
    else:
        legend = None
    plot_phase_portrait(
        flux_sol, x=x_i, y=y_i, ax=ax,
        xlabel=x_i, ylabel=y_i,
        xlim=xlims[i], ylim=ylims[i],
        title=("Phase Portrait of {0} vs. {1}".format(x_i, y_i), L_FONT),
        annotate_time_points=time_points,
        annotate_time_points_color=time_point_colors,
        annotate_time_points_legend=legend)
fig_10_15.tight_layout()
```

**Figure 10.15:** The dynamic response of key fluxes. Detailed pair-wise phase portraits: a): \(v\_{ATPM}\) vs. \(v\_{DM\_{AMP}}\). b): \(v\_{ATPM}\) vs. \(v\_{SK\_{pyr}}\). c): \(v\_{DM\_{AMP}}\) vs. \(v\_{SK\_{pyr}}\). d): \(v\_{GAPD}\) vs. \(v\_{LDH\_{L}}\). e): \(v\_{LDH\_{L}}\) vs. \(v\_{DM\_{NADH}}\). f): \(v\_{HEX1}\) vs. \(v\_{PYK}\). The fluxes are in units of mM/hr. The perturbation is reflected in the instantaneous move of the flux state from the initial
steady state to an unsteady state, as indicated by the arrow placing the initial point at \(t = 0^+.\) The system then returns to its steady state at \(t \rightarrow \infty\)\*

##### Pooling: Towards Systems Biology¶

We now take a look at the biochemical features of this pathway to formulate meaningful pooling of the concentrations to form aggregate variables. The formulation of these quantities allows for a systems interpretation of the dynamic responses rather than the chemical interpretation that is achieved by looking at individual concentrations and fluxes. The analysis is organized around the three cofactor coupling features of glycolysis. A symbolic version of the compounds, shown in Figure 10.16, can
help us conceptually in this process.

**Figure 10.16:** A schematic of glycolysis that symbolically shows how redox (shaded=reduced, clear=oxidized) energy and inorganic phosphate (open circle) incorporation are coupled to the pathway. The closed circles represent phosphates that cycle between the adenylates and the glycolytic intermediates. A triangle is used to symbolize a three carbon compound.

###### High-energy phosphate bond trafficking¶

There are four kinases (HEX1, PFK, PGK, and PYK) in the pathway. With respect to the potential to generate high-energy phosphate bonds, PYR and LAC are in a “ground state,” incapable of generating such bonds. The phosphoglycerates (PG3 + PG2 + PEP) are capable of generating one high energy bond, the triose phosphates (2FDP + DHAP + GAP + DPG13) are capable of generating two, the hexose phosphates (G6P + F6P) three, and glucose, two. Thus, the high-energy inventory (occupancy) in glycolysis is:

\[\begin{split}\begin{align} p\_1 &= 2\ \text{Gluc}\ +\ 3(\text{G6P}\ +\ \text{F6P}) \\ &+\ 2(2\text{FDP}\ +\ \text{DHAP}\ +\ \text{GAP}\ +\ \text{13DPG}) \\ &+\ (\text{3PG}\ +\ \text{2PG} +\ \text{PEP}) \\ \end{align} \tag{10.5}\end{split}\]

The intermediates that have no high energy phosphate bond value are

\[\begin{equation}p\_2 = \text{PYR}\ +\ \text{LAC} \end{equation}\]

The high-energy bonds made in glycolysis are then stored in the form of ATP that in turn is used to meet energy demands. As we saw in Chapter 2, the adenylate phosphates can be described by two pools:

\[\begin{equation} p\_3 = \text{ADP}\ +\ 2\ \text{ATP} \ \text{and}\ p\_4 = 2\ \text{AMP}\ +\ \text{ADP} \tag{10.6} \end{equation}\]

that represent the capacity and vacancy of high energy bonds on the adenylates. The addition of the two gives us the capacity as we saw in Chapter 2.

**Figure 10.17:** High-energy bond trafficking in glycolysis. The numbers above the arrows are the fluxes. The 2x indicates doubling in flux where a hexose is split into two trioses. The numbers in parentheses above the compounds represent their high-energy bond value.

###### Redox trafficking¶

The net flow of glucose to two lactate molecules is neutral with respect to generation of redox potential. If there is a separate redox load (see line 17 in Table 10.12.) then some of the NADH that is produced by GAPDH is used for other purposes than to reduce PYR to LAC via LDH. Then in order to balance the fluxes in the pathway there will be reduced production of LAC that will be reflected by secretion of PYR (see lines 14 and 15 in Table 10.12).

The conversion of GAP to DPG13 is coupled to the NADH/NAD cofactor pair via the GAPDH reaction as is the conversion of PYR to LAC via the LDH reaction. GAPDH and LDH are the two hydrogenases that determine the exchange of redox equivalents in the pathway. Thus, the compounds “upstream” of GAPDH have redox value relative to NAD. The hexoses (Gluc, G6P, F6P, and FDP) all have the potential to reduce two NAD molecules, and the trioses (DHAP, GAP) can reduce one NAD. The three-carbon compounds
“downstream” from GAPDH (DPG13, PG3, PG2, PEP, and PYR) have no redox value. The conversion of PYR to LAC using NADH gives LAC a redox value of one NADH. The two dehydrogenases are reversible enzymes. Note that this assignment of redox value is dependent on the environment in which the pathway operates, Figure 10.1, as defined by the systems boundary drawn and the inputs and outputs given.

The total redox inventory (occupancy) of the glycolytic intermediates is thus given by

\[\begin{equation} p\_5 = 2(\text{Gluc}\ +\ \text{G6P}\ +\ \text{F6P}\ +\ \text{FDP})\ +\ (\text{DHAP}\ +\ \text{GAP})\ +\ \text{LAC} \tag{10.7} \end{equation}\]

By the same token, the intermediates in an oxidized state are

\[\begin{equation} p\_6 = \text{13DPG}\ +\ \text{3PG}\ +\ \text{2PG}\ +\ \text{PEP}\ +\ \text{PYR} \tag{10.8} \end{equation}\]

The carrier of the redox potential is NADH, we thus define

\[\begin{equation} p\_7 = \text{NADH} = N^+ \tag{10.9} \end{equation}\]

as the occupancy of redox potential. Clearly then, NAD would be the vacancy state, denoted by \(N^-.\) However, in this case, \(p\_{11}=N^+ + N^-\) will be the total capacity to carry redox potential by this carrier. It is a time invariant pool in this model since the cofactor moiety cannot enter or leave the system.

**Figure 10.18:** Redox trafficking in glycolysis. The numbers above the arrows are the fluxes in a steady state. The 2x indicates doubling in flux where a hexose is split into two trioses. If 1.5 flux units enter glycolysis and the redox load is one, then two flux units of lactate and one of pyruvate are formed. The numbers in parentheses above the compounds represent their redox value.

###### The trafficking of phosphate groups¶

Glycolysis generates a net of two ATP per glucose consumed; two ATP molecules are spent and four are generated. In the process, GAPDH incorporates the net of two inorganic phosphate groups that are needed to generate the net two ATP from ADP. These features lead to distinguishing two types of phosphate groups in glycolysis: those that were recycled between the glycolytic intermediates and the adenylate carrier or being used through the ATP load reaction

\[\begin{equation} p\_8 = \text{G6P}\ + \text{F6P}\ + 2\ \text{FDP}\ + \text{DHAP}\ + \text{GAP}\ + \text{13DPG}\ +\ \text{ADP}\ + 2\ \text{ATP} \tag{10.10} \end{equation}\]

and those that were incorporated

\[\begin{equation} p\_8 = \text{13DPG}\ + \text{3PG}\ + \text{2PG}\ + \text{PEP} \tag{10.11} \end{equation}\]

If the inorganic phosphate is added to the sum of these two pools, then we get the total phosphate inventory, which is the second time invariant, \(p\_{10}.\)

**Figure 10.19:** The trafficking of phosphate groups in glycolysis. (a) The incorporation of inorganic phosphate that then gets passed onto the molecule, C, where it creates a high-energy phosphate bond (in the example simulated in this chapter, the load is simply a hydrolysis reaction and there is no C molecule). (b) The cycling of high-energy phosphate groups between the adenylate carrier and the glycolytic intermediates. The relative steady state flux values are indicated by 1x and 2x.

###### The pooling matrix¶

The inspection of the biochemical properties of the glycolytic pathway leads to the definition of a series of pools. This biochemical insight can be formally represented mathematically using a pooling matrix, \(\textbf{P}\), Table 10.12. The concentration vector \(\textbf{x}\) can be converted into a vector that contains the pools (\(\textbf{p}\)) by:

\[\begin{equation} \textbf{p}=\textbf{Px} \end{equation}\]

as a post processing step (see Equation (3.4)).

The table sums up the non-zero elements in a row \((\rho\_i)\) and in a column \((\pi\_j)\). The number of compounds that make up a pool is given by \(\rho\_i\), while \(\pi\_j\) gives the number of pools in which a compound participates. Thus, \(\pi\_j\) tell us how many aggregate metabolic properties in which a compound participates. For instance, G6P has a glycolytic energy value, glycolytic redox value, and has a high energy phosphate group. G6P is thus in three
\(\pi\_{G6P} = 3\) different glycolytic pools.

This multi-functionality of a compound is common in biochemical reaction networks. This feature makes it hard to untangle the individual functions of a compound (recall the ‘tangle of cycles’ notion from Chapter 8). This coupling of individual functions of a molecule (or a node) in a network is a foundational feature of systems biology.

```
[38]:
```

```
# Define the individual pools. The placement of each coefficient
# corresponds to the order of the metabolites in the model as seen
# in table_10_8.  _p indicates the positive superscript ^+ and
# _n indicates the negative superscript (^-).
def make_pooling_matrix(include_all=False):
    GP_p = np.array([2, 3, 3, 4, 2, 2, 2, 1, 1, 1, 0, 0, 0, 0, 0, 0, 0, 0, 0 ,0])
    GP_n = np.array([0, 0, 0, 0, 0, 0, 0, 0, 0, 0, 1, 1, 0, 0, 0, 0, 0, 0, 0, 0])
    AP_p = np.array([0, 0, 0, 0, 0, 0, 0, 0, 0, 0, 0, 0, 0, 0, 0, 1, 2, 0, 0, 0])
    AP_n = np.array([0, 0, 0, 0, 0, 0, 0, 0, 0, 0, 0, 0, 0, 0, 2, 1, 0, 0, 0, 0])
    GR_p = np.array([2, 2, 2, 2, 1, 1, 0, 0, 0, 0, 0, 1, 0, 0, 0, 0, 0, 0, 0, 0])
    GR_n = np.array([0, 0, 0, 0, 0, 0, 1, 1, 1, 1, 1, 0, 0, 0, 0, 0, 0, 0, 0, 0])
    N_p = np.array([0, 0, 0, 0, 0, 0, 0, 0, 0, 0, 0, 0, 0, 1, 0, 0, 0, 0, 0, 0])
    N_n = np.array([0, 0, 0, 0, 0, 0, 0, 0, 0, 0, 0, 0, 1, 0, 0, 0, 0, 0, 0, 0]) # Not shown in pooling matrix
    P_p = np.array([0, 1, 1, 2, 1, 1, 1, 0, 0, 0, 0, 0, 0, 0, 0, 1, 2, 0, 0, 0])
    P_n = np.array([0, 0, 0, 0, 0, 0, 1, 1, 1, 1, 0, 0, 0, 0, 0, 0, 0, 0, 0, 0])
    P_tot = P_p + P_n
    P_tot[-3] = 1
    N_tot = N_p + N_n
    # Define pooling matrix and pool labels
    if include_all:
        pool_labels = [r"$GP^+$", r"$GP^-$", r"$AP^+$", r"$AP^-$", r"$GR^+$", r"$GR^-$",
                       r"$N^+$", r"$N^-$", r"$P^+$", r"$P^-$", r"$P_{\mathrm{tot}}$", r"$N_{\mathrm{tot}}$"]
        pooling_matrix = np.vstack([GP_p, GP_n, AP_p, AP_n, GR_p, GR_n,
                                    N_p, N_n, P_p, P_n, P_tot, N_tot])
    else:
        pool_labels = [r"$GP^+$", r"$GP^-$", r"$AP^+$", r"$AP^-$", r"$GR^+$", r"$GR^-$",
                        r"$N^+$", r"$P^+$", r"$P^-$", r"$P_{\mathrm{tot}}$", r"$N_{\mathrm{tot}}$"]
        pooling_matrix = np.vstack([GP_p, GP_n, AP_p, AP_n, GR_p, GR_n,
                                    N_p, P_p, P_n, P_tot, N_tot])
    return pooling_matrix, pool_labels
include_all = False
```

**Table 10.12:** A pooling matrix, P, for glycolysis. Pools 10 \((P\_{\mathrm{\mathrm{tot}}})\) and 11 \((N\_{\mathrm{\mathrm{tot}}})\) are the two time-invariant pools discussed in Section 10.3. They are the ‘hard’ pools in the left null space of \(\textbf{S}\). The number of ‘soft’ pools in which a compound participates, \(\pi\_j\), is shown (cyan). The number of compounds in a pool, \(\rho\_i\), is given in the last column (red).

```
[39]:
```

```
# Make table content from the pooling matrix, connectivity and
# participation numbers, and pool labels
pooling_matrix, pool_labels = make_pooling_matrix(include_all)
pool_numbers = np.array([[i for i in range(1, len(pool_labels) + 1)] + [""]])
pi = np.count_nonzero(pooling_matrix, axis=0)
rho = np.array([np.concatenate((np.count_nonzero(pooling_matrix, axis=1), [""]))])
table_10_12 = np.vstack((pooling_matrix, pi))
table_10_12 = np.hstack((pool_numbers.T, table_10_12, rho.T))

index_labels = pool_labels + [pi_str]
column_labels = ["Pool #"] + metabolite_ids + [rho_str]
table_10_12 = pd.DataFrame(table_10_12, index=index_labels,
                           columns=column_labels)

# Highlight table
n_colors = int(np.ceil(len(pool_labels)/2))
color_list = [mpl.colors.to_hex(c) for c in mpl.cm.Pastel1(np.linspace(0.2, 0.7, n_colors))]
colors = dict(zip(["GP", "AP", "GR", "N", "P", "tot"], color_list))
colors.update({pi_str: "#99ffff",    # Cyan
               rho_str: "#ff9999",   # Red
               "blank": "#f2f2f2"}) # Grey
bg_color_str = "background-color: "
def highlight_table(df):
    df = df.copy()

    for row in df.index:
        if row == pi_str:
            main_key = pi_str
        elif row[1:-3] in colors:
            main_key = row[1:-3]
        else:
            main_key = "tot"
        df.loc[row, :] = [bg_color_str + colors[main_key] if v != ""
                          else bg_color_str + colors["blank"]
                          for v in df.loc[row, :]]
    for col in df.columns:
        if col == rho_str:
            df.loc[:, col] = [bg_color_str + colors[rho_str]
                              if v != bg_color_str + colors["blank"]
                              else v for v in df.loc[:, col]]
    return df

table_10_12 = table_10_12.style.apply(highlight_table, axis=None)
table_10_12
```

```
[39]:
```

|  | Pool # | glc\_\_D\_c | g6p\_c | f6p\_c | fdp\_c | dhap\_c | g3p\_c | \_13dpg\_c | \_3pg\_c | \_2pg\_c | pep\_c | pyr\_c | lac\_\_L\_c | nad\_c | nadh\_c | amp\_c | adp\_c | atp\_c | pi\_c | h\_c | h2o\_c | $\rho\_{i}$ |
| --- | --- | --- | --- | --- | --- | --- | --- | --- | --- | --- | --- | --- | --- | --- | --- | --- | --- | --- | --- | --- | --- | --- |
| $GP^+$ | 1 | 2 | 3 | 3 | 4 | 2 | 2 | 2 | 1 | 1 | 1 | 0 | 0 | 0 | 0 | 0 | 0 | 0 | 0 | 0 | 0 | 10 |
| $GP^-$ | 2 | 0 | 0 | 0 | 0 | 0 | 0 | 0 | 0 | 0 | 0 | 1 | 1 | 0 | 0 | 0 | 0 | 0 | 0 | 0 | 0 | 2 |
| $AP^+$ | 3 | 0 | 0 | 0 | 0 | 0 | 0 | 0 | 0 | 0 | 0 | 0 | 0 | 0 | 0 | 0 | 1 | 2 | 0 | 0 | 0 | 2 |
| $AP^-$ | 4 | 0 | 0 | 0 | 0 | 0 | 0 | 0 | 0 | 0 | 0 | 0 | 0 | 0 | 0 | 2 | 1 | 0 | 0 | 0 | 0 | 2 |
| $GR^+$ | 5 | 2 | 2 | 2 | 2 | 1 | 1 | 0 | 0 | 0 | 0 | 0 | 1 | 0 | 0 | 0 | 0 | 0 | 0 | 0 | 0 | 7 |
| $GR^-$ | 6 | 0 | 0 | 0 | 0 | 0 | 0 | 1 | 1 | 1 | 1 | 1 | 0 | 0 | 0 | 0 | 0 | 0 | 0 | 0 | 0 | 5 |
| $N^+$ | 7 | 0 | 0 | 0 | 0 | 0 | 0 | 0 | 0 | 0 | 0 | 0 | 0 | 0 | 1 | 0 | 0 | 0 | 0 | 0 | 0 | 1 |
| $P^+$ | 8 | 0 | 1 | 1 | 2 | 1 | 1 | 1 | 0 | 0 | 0 | 0 | 0 | 0 | 0 | 0 | 1 | 2 | 0 | 0 | 0 | 8 |
| $P^-$ | 9 | 0 | 0 | 0 | 0 | 0 | 0 | 1 | 1 | 1 | 1 | 0 | 0 | 0 | 0 | 0 | 0 | 0 | 0 | 0 | 0 | 4 |
| $P\_{\mathrm{tot}}$ | 10 | 0 | 1 | 1 | 2 | 1 | 1 | 2 | 1 | 1 | 1 | 0 | 0 | 0 | 0 | 0 | 1 | 2 | 1 | 0 | 0 | 12 |
| $N\_{\mathrm{tot}}$ | 11 | 0 | 0 | 0 | 0 | 0 | 0 | 0 | 0 | 0 | 0 | 0 | 0 | 1 | 1 | 0 | 0 | 0 | 0 | 0 | 0 | 2 |
| $\pi\_{j}$ |  | 2 | 4 | 4 | 4 | 4 | 4 | 5 | 4 | 4 | 4 | 2 | 2 | 1 | 2 | 1 | 4 | 3 | 1 | 0 | 0 |  |

###### The reactions that move the pools¶

The dynamic mass balances can be multiplied by the matrix P to obtain dynamic balances on the pools, as

\[\begin{equation} \frac{d\textbf{p}}{dt} = \textbf{P}\frac{d\textbf{x}}{dt} = \textbf{PSv (x)} \tag{10.12} \end{equation}\]

The matrix \(\textbf{PS}\) (see Table 10.13) therefore tells us which fluxes move each of the pools.

**Table 10.13:** The fluxes that move the pools. This table is obtained from the product **PS**. The time invariant pools are in the last two rows. The number of reactions, \(\rho\_i,\) that move a pool is shown (cyan), as is the pool size (light blue), the steady state flux in and out of the pool (light pink), and its turnover time (light green). The number of pools that a reaction moves, \(\pi\_j,\) is also given (red).

```
[40]:
```

```
# Make table content from the pooling matrix, connectivity and
# participation numbers, pool sizes, and time constants
pooling_matrix, pool_labels = make_pooling_matrix(include_all)
PS = pooling_matrix.dot(glycolysis.S).astype(np.int64)
pi = np.count_nonzero(PS, axis=0)
rho = np.count_nonzero(PS, axis=1)
ic_values = [glycolysis.initial_conditions[met]
             for met in glycolysis.metabolites.get_by_any(metabolite_ids)]
pool_sizes = [round(np.sum([coeff*ic for coeff, ic in zip(row, ic_values)]), 5)
              for row in pooling_matrix]
flux_values = [glycolysis.steady_state_fluxes[rxn]
               for rxn in glycolysis.reactions.get_by_any(reaction_ids)]
fluxes_in = [round(np.sum([coeff*flux for coeff, flux in zip(row, flux_values)
                           if coeff >=0]), 5) for row in PS]
taus = [round(size/flux_in, 5) if flux_in != 0
        else r"$\infty$" for size, flux_in in zip(pool_sizes, fluxes_in)]
table_10_13 = np.hstack((pool_numbers.T,
                         np.vstack((PS, pi)),
                         np.array([np.append(col, "")
                                   for col in np.array([rho, pool_sizes, fluxes_in, taus])]).T))
index_labels = pool_labels + [pi_str]
column_labels = np.concatenate((["Pool #"], reaction_ids,
                                [rho_str, "Size (mM)", "Net steady state flux (mM/hr)", r"$\tau$(h)"]))
table_10_13 = pd.DataFrame(table_10_13, index=index_labels,
                           columns=column_labels)
# Highlight table
colors.update({"Size (mM)": "#e6faff",                     # L. Blue
               "Net steady state flux (mM/hr)": "#f9ecf2", # L. Pink
               r"$\tau$(h)": "#e6ffe6"})                   # L. Green
bg_color_str = "background-color: "
def highlight_table(df):
    df = df.copy()
    for row in df.index:
        if row == pi_str:
            main_key = pi_str
        elif row[1:-3] in colors:
            main_key = row[1:-3]
        else:
            main_key = "tot"
        df.loc[row, :] = [bg_color_str + colors[main_key] if v != ""
                          else bg_color_str + colors["blank"]
                          for v in df.loc[row, :]]
    for col in df.columns:
        if col in colors:
            df.loc[:, col] = [bg_color_str + colors[col]
                              if v != bg_color_str + colors["blank"]
                              else v for v in df.loc[:, col]]
    return df

table_10_13 = table_10_13.style.apply(highlight_table, axis=None)
table_10_13
```

```
[40]:
```

|  | Pool # | HEX1 | PGI | PFK | FBA | TPI | GAPD | PGK | PGM | ENO | PYK | LDH\_L | DM\_amp\_c | ADK1 | SK\_pyr\_c | SK\_lac\_\_L\_c | ATPM | DM\_nadh | SK\_glc\_\_D\_c | SK\_amp\_c | SK\_h\_c | SK\_h2o\_c | $\rho\_{i}$ | Size (mM) | Net steady state flux (mM/hr) | $\tau$(h) |
| --- | --- | --- | --- | --- | --- | --- | --- | --- | --- | --- | --- | --- | --- | --- | --- | --- | --- | --- | --- | --- | --- | --- | --- | --- | --- | --- |
| $GP^+$ | 1 | 1 | 0 | 1 | 0 | 0 | 0 | -1 | 0 | 0 | -1 | 0 | 0 | 0 | 0 | 0 | 0 | 0 | 2 | 0 | 0 | 0 | 5 | 2.70425 | 4.48 | 0.60363 |
| $GP^-$ | 2 | 0 | 0 | 0 | 0 | 0 | 0 | 0 | 0 | 0 | 1 | 0 | 0 | 0 | -1 | -1 | 0 | 0 | 0 | 0 | 0 | 0 | 3 | 1.4203 | 2.24 | 0.63406 |
| $AP^+$ | 3 | -1 | 0 | -1 | 0 | 0 | 0 | 1 | 0 | 0 | 1 | 0 | 0 | 0 | 0 | 0 | -1 | 0 | 0 | 0 | 0 | 0 | 5 | 3.49 | 4.48 | 0.77902 |
| $AP^-$ | 4 | 1 | 0 | 1 | 0 | 0 | 0 | -1 | 0 | 0 | -1 | 0 | -2 | 0 | 0 | 0 | 1 | 0 | 0 | 2 | 0 | 0 | 7 | 0.46346 | 4.508 | 0.10281 |
| $GR^+$ | 5 | 0 | 0 | 0 | 0 | 0 | -1 | 0 | 0 | 0 | 0 | 1 | 0 | 0 | 0 | -1 | 0 | 0 | 2 | 0 | 0 | 0 | 4 | 3.69328 | 4.256 | 0.86778 |
| $GR^-$ | 6 | 0 | 0 | 0 | 0 | 0 | 1 | 0 | 0 | 0 | 0 | -1 | 0 | 0 | -1 | 0 | 0 | 0 | 0 | 0 | 0 | 0 | 3 | 0.16614 | 2.24 | 0.07417 |
| $N^+$ | 7 | 0 | 0 | 0 | 0 | 0 | 1 | 0 | 0 | 0 | 0 | -1 | 0 | 0 | 0 | 0 | 0 | -1 | 0 | 0 | 0 | 0 | 3 | 0.0301 | 2.24 | 0.01344 |
| $P^+$ | 8 | 0 | 0 | 0 | 0 | 0 | 0 | 0 | 0 | 0 | 1 | 0 | 0 | 0 | 0 | 0 | -1 | 0 | 0 | 0 | 0 | 0 | 2 | 3.75512 | 2.24 | 1.67639 |
| $P^-$ | 9 | 0 | 0 | 0 | 0 | 0 | 1 | 0 | 0 | 0 | -1 | 0 | 0 | 0 | 0 | 0 | 0 | 0 | 0 | 0 | 0 | 0 | 2 | 0.10584 | 2.24 | 0.04725 |
| $P\_{\mathrm{tot}}$ | 10 | 0 | 0 | 0 | 0 | 0 | 0 | 0 | 0 | 0 | 0 | 0 | 0 | 0 | 0 | 0 | 0 | 0 | 0 | 0 | 0 | 0 | 0 | 6.36097 | 0.0 | $\infty$ |
| $N\_{\mathrm{tot}}$ | 11 | 0 | 0 | 0 | 0 | 0 | 0 | 0 | 0 | 0 | 0 | 0 | 0 | 0 | 0 | 0 | 0 | 0 | 0 | 0 | 0 | 0 | 0 | 0.089 | 0.0 | $\infty$ |
| $\pi\_{j}$ |  | 3 | 0 | 3 | 0 | 0 | 4 | 3 | 0 | 0 | 6 | 3 | 1 | 0 | 2 | 2 | 3 | 1 | 2 | 1 | 0 | 0 |  |  |  |  |

Table 10.13 also contains the summation of the non-zero elements in a row \((\rho\_i)\) and a column \((\pi\_j)\). The former gives the number of fluxes that fill or drain a pool. Thus, when drawing the system in terms of pools, (see Figure 10.20). \(\pi\_j\) is the number of links in a pool. Notice that the last two rows have no non-zero entries since these are time invariant pools. The latter number, \(\pi\_j\), is the number of pools that a flux moves. Many of the \(\pi\_j\)
are zero, and thus these reactions do not appear in Figure 10.20. Conversely, some fluxes drain and fill many pools. PYK moves six pools, while GAPD moves four pools.

###### Graphical representation¶

The glycolytic system can now be laid out in terms of the pools (see Figure 10.20). This layout shows how the fluxes interconnect the pools and how the loads pull on these pools and lead to the movement of mass among the pools. This diagram is akin to a process flow chart. The pools sizes, the steady state flux through them, and their response times, and the ratio of the sizes to the net fluxes, are shown in Table 10.13. Note that we have now moved our point of view from the individual molecules
to a view where aggregate redox, energy, and phosphate values are displayed.

**Figure 10.20:** A pool-flux map of glycolysis. The pools from Table 10.12 are shown as well as the fluxes that fill and drain them, given in Table 10.13. The relative areas of the boxes indicate the relative concentration of a pool.

###### Dynamic behavior of the pools¶

The pooling matrix, \(\textbf{P}\), defined in Table 10.12, can be used to compute the pools by \(\textbf{p}(t)=\textbf{Px}(t).\) The time dependent responses of the pools can then be studied. The dynamic responses to a sudden increase in ATP load of the phosphate containing pools are shown in Figure 10.20. These pools go through well-defined changes:

- The sudden change in the ATP usage rate creates a flux imbalance that leads to drainage of the \(AP^+\) and \(P^+\) pools that is mirrored in the build-up of the \(AP^-\) pool.
- Counterintuitively, taken together, the activities of the kinases lead to an increase in the \(GP^+\) and \(GR^+\) pools.
- There is a slow drainage of the \(AP^-\) pool that corresponds to the exit of AMP from the system, that is reflected in an increase in the \(P^-\) and the \(GR^-\) pools.
- There is a dynamic interaction with the NADH pool leading to some drainage of redox potential.

The latter two effects are the results of the interactions between high-energy phosphate metabolism and the AMP exchange and redox production in the form of NADH.

```
[41]:
```

```
# Make matrix
pooling_matrix, pool_labels = make_pooling_matrix(include_all)
PS = pooling_matrix.dot(glycolysis.S)
# Make solution IDs, equations, and variables for pooled solutions
pools = {}
net_fluxes = {}
for i, pool_id in enumerate(pool_labels[:-2]):
    terms = ["*".join((str(coeff), met))
             for coeff, met in zip(pooling_matrix[i], metabolite_ids)
             if coeff != 0]
    variables = [term.split("*")[-1] for term in terms]
    pools.update({pool_id: ["+".join(terms), variables]})

    for flux_str, condition in zip([" influx", " efflux"], [lambda i: i > 0, lambda i: i < 0]):
        terms = ["*".join((str(coeff), rxn))
                 for coeff, rxn in zip(PS[i], reaction_ids)
                 if condition(coeff)]
        variables = [term.split("*")[-1] for term in terms]
        net_fluxes.update({
            pool_id + flux_str: ["+".join(terms), variables]})

for sol_obj, equation_dict in zip([conc_sol, flux_sol],
                                  [pools, net_fluxes]):
    for sol_id, (equation, variables) in equation_dict.items():
        sol_obj.make_aggregate_solution(
            sol_id, equation=equation, variables=variables)

time_points = [t0, 1e-1, 1e0, 1e1, 1e2, tf]
time_point_colors = [
    mpl.colors.to_hex(c)
    for c in mpl.cm.Reds(np.linspace(0.3, 1, len(time_points)))]

# Create figure
fig_10_21 = plt.figure(figsize=(20, 12))
outer_grid = mpl.gridspec.GridSpec(nrows=2, ncols=5, wspace=.4)
for i, pool_id in enumerate(pools):
    inner_grid = mpl.gridspec.GridSpecFromSubplotSpec(
        nrows=2, ncols=1, subplot_spec=outer_grid[i],
        height_ratios=[2, 1], hspace=.4)

    for j in range(2):
        ax = plt.Subplot(fig_10_21, inner_grid[j])
        if i == len(pools) - 1 and j == 0:
            legend = "right outside"
        else:
            legend = None
        if j == 0:
            plot_phase_portrait(
                flux_sol, x=pool_id + " influx", y=pool_id + " efflux", ax=ax,
                xlabel=("Influx [mM/hr]", S_FONT),
                ylabel=("Efflux [mM/hr]", S_FONT),
                title=(pool_id + " influx vs. efflux", L_FONT),
                annotate_time_points=time_points,
                annotate_time_points_color=time_point_colors,
                annotate_time_points_legend=legend)

        else:
            plot_time_profile(
                conc_sol, observable=pool_id, ax=ax,
                plot_function="semilogx",
                xlabel=("Time [hr]", S_FONT),
                ylabel=("Concentrations [mM]", S_FONT),
                title=(pool_id + " Concentration", {"size": "medium"}));
        fig_10_21.add_subplot(ax)
```

**Figure 10.21:** The dynamic response of the glycolytic pools. Top, the flux phase portrait (flux in on the x-axis vs. flux out on the y-axis) is shown for each pool. Bottom, the concentration profile of the pool.

###### The dual issue that underlies the formation of pools¶

We close this section by observing that although Eq. (10.12) does relate the fluxes to the pools, the functions for the fluxes are dependent on the concentrations. Thus, there is a dual issue here. If we are to get dynamic descriptions of the pools, we must convert the arguments in the flux vector from the concentration variables to the pools themselves. This problem is mathematically difficult to analyze and is addressed elsewhere (*Systems Biology: Volume III*).

##### Ratios: Towards Physiology¶

We now take a look at the conjugate pools (i.e., the corresponding high and low states) of various metabolic properties of the glycolytic system to form property ratios. The energy charge is one such ratio and was introduced in Chapter 2. The formulation of these quantities allows a physiological interpretation of the dynamic responses.

The notion of a charge is related to the relative size of the conjugate pools. A ratio is formed as:

\[\begin{equation} \text{property ratio}=\frac{\text{high}}{\text{high} + \text{low}} = \frac{\text{high}}{\text{total}} \tag{10.13} \end{equation}\]

The ratios, \(r\_i\), are computed from the conjugates, \(p\_i\). We can then graph the ratios to interpret transient responses from a metabolic physiological point of view. The total pool size may change on a different time scale than the interconversion between the high and low forms. Any time scale hierarchy in such responses is of interest since most physiological responses involve multi-scale analysis.

###### Energy charges¶

We can define an energy charge for glycolysis as the ratio:

\[\begin{equation} r\_1 =\frac{p\_1}{p\_1 + p\_2} \tag{10.14} \end{equation}\]

that is an analogous quantity to the adenylate energy charge

\[\begin{equation} r\_2 =\frac{p\_3}{p\_3 + p\_4} = \frac{2\ \text{ATP}\ +\ \text{ADP}}{2\ (\text{ATP}\ +\ \text{ADP}\ +\ \text{AMP})} \tag{10.15} \end{equation}\]

Thus, we have two ratios that describe metabolic physiology in terms of the energy charge in the glycolytic intermediates and on the adenylate phosphates.

These charge parameters \((r\_1\ \text{and}\ r\_2)\) can vary between zero and unity. If the ratio is close to unity, the energy charge is high, and vice versa. The glycolytic pathway thus transfers metabolic energy equivalents from glucose to ADP. The energy charge of the glycolytic intermediates and NAD are quantities that describe how this process takes place.

###### Redox charges¶

We can define the redox charge in glycolysis as

\[\begin{equation} r\_3 =\frac{p\_5}{p\_5 + p\_6} \tag{10.16} \end{equation}\]

We note that three times the denominator in equation 0 is the total carbon inventory in glycolysis. In an analogous fashion, we can define the redox state on the NAD carrier as

\[\begin{equation} r\_4 =\frac{\text{NADH}}{\text{NADH}\ +\ \text{NAD}} = \frac{p\_7}{p\_{10}} \tag{10.17} \end{equation}\]

These ratios will have identical interpretations as the energy charges. Glycolysis will move redox equivalents in glucose onto NADH that then become the conduit of redox equivalent to other processes in a cell, here represented by the NADH load function.

###### The state of the phosphate groups¶

We can define the state of the phosphates as

\[\begin{equation} r\_5 =\frac{p\_8}{p\_8 + p\_9} \tag{10.18} \end{equation}\]

to get the fraction of phosphate that has been incorporated and is available for recycling or to meet the ATP demand function.

```
[42]:
```

```
# Make pool solutions
pooling_matrix, pool_labels = make_pooling_matrix(True)
pools = {}
for i, pool_id in enumerate(pool_labels[:-2]):
    terms = ["*".join((str(coeff), met))
             for coeff, met in zip(pooling_matrix[i], metabolite_ids)
             if coeff != 0]
    variables = [term.split("*")[-1] for term in terms]
    pools.update({pool_id: ["+".join(terms), variables]})

ratios = {}
keys = [
    ('$GP^+$', '$GP^-$'), ('$AP^+$', '$AP^-$'),
    ('$GR^+$', '$GR^-$'),('$N^+$', '$N^-$'),
    ('$P^+$', '$P^-$')]
ratio_names = [
    "Glycolytic Energy Charge", "Adenylate Energy Charge",
    "Glycolytic Redox Charge", "NADH Redox Charge",
    "Phosphate Recycle Ratio"]
for name, (k1, k2) in zip(ratio_names, keys):
    ratio = " / ".join(["({0})".format(pools[k1][0]),
                        "({0} + {1})".format(pools[k1][0], pools[k2][0])])
    variables = list(set(pools[k1][1] + pools[k2][1]))
    ratios[name] = [ratio, variables]

for ratio_id, (equation, variables) in ratios.items():
    conc_sol.make_aggregate_solution(
        ratio_id, equation=equation, variables=variables)
```

**Table 10.14:** The numerical values of key ratios in glycolysis before and after the increased rate of ATP use.

```
[43]:
```

```
table_10_14 = pd.DataFrame(
    [[ratio, conc_sol[ratio][0], conc_sol[ratio][-1]]
     for ratio in ratios],
    index=range(1, len(ratios) + 1),
    columns=["Ratio Name", "t=0", "t=1000"])
table_10_14.index.rename("$r_i$", inplace=True)
table_10_14
```

```
[43]:
```

|  | Ratio Name | t=0 | t=1000 |
| --- | --- | --- | --- |
| $r\_i$ |  |  |  |
| 1 | Glycolytic Energy Charge | 0.656 | 0.723 |
| 2 | Adenylate Energy Charge | 0.883 | 0.852 |
| 3 | Glycolytic Redox Charge | 0.957 | 0.962 |
| 4 | NADH Redox Charge | 0.338 | 0.338 |
| 5 | Phosphate Recycle Ratio | 0.973 | 0.954 |

###### Operating diagrams¶

The combination of the conjugate pools can be used to develop the pool diagram of Figure 10.20 further and form effectively an operating diagram for glycolysis; Figure 10.21. This diagram represents the glycolytic system as a set of interconnected charges of various properties, and is a physiological point of view.

**Figure 10.21:** An operating diagram for the glycolytic systems.

```
[44]:
```

```
fig_10_22, axes = plt.subplots(nrows=3, ncols=2, figsize=(16, 6))
axes = axes.flatten()
axes, ax6 = (axes[:5], axes[5:])
fig_10_22.delaxes(ax6[0])

ylims = [(0.6, 0.9), (0.6, 0.9), (0.9, 1), (0.2, 0.4), (0.95, 1)]
for i, (ax, ratio) in enumerate(zip(axes, list(ratios))):
    plot_time_profile(
        conc_sol, observable=ratio, ax=axes[i],
        xlim=(t0, 50), ylim=ylims[i],
        xlabel=("Time [hr]", S_FONT),
        ylabel=("Ratio", S_FONT), title=(ratio, L_FONT))
fig_10_22.tight_layout()
```

**Figure 10.22:** The dynamic response of the glycolytic property ratios.

###### Dynamic responses of the ratios¶

The ratios can easily be computed from the time profiles of the pools. The response of the ratios is shown on the glycolytic operating diagram (Figure 10.21) in Figure 10.22. We now see the simplicity in the dynamic reaction of the glycolytic system to the increased rate of ATP usage.

- The energy charge experiences a significant drop initially, but then recovers over a long time period, due to the reduction in the total adenosine phosphate ATP + ADP + AMP pool.
- The phosphate charge has a similar but much less pronounced adjustment.
- The energy charge of glycolysis increases.
- There are minor ripple effects through the redox pools.

Note that these responses are built into the stoichiometry and the numerical values of the rate constants. No regulatory mechanisms are required to obtain these systemic responses.

##### Assumptions¶

The glycolytic MASS model has several assumptions built into it. The user of models and dynamic simulators needs to be aware of the assumptions that underlie a model, and these assumptions need to be taken into account when interpreting the results from the simulation. The effects of assumptions can be examined by simulations that test these assumptions.

###### The time invariant pools¶

The total amount of the NADH redox carrier and the total amount of phosphate are constants in the glycolytic system studied. The operating diagram shows the consequences of the time invariant pools. The total NADH is constant as it does not leave or enter the system. The total phosphate pool is a constant. The phosphate group is found in three different forms. We note that the incorporation of \(\text{P}\_i\) and its formation from the ATP load reaction are from the same source. We study the
consequences of these conservations through the homework sets given below.

###### Constant inputs and environment¶

The model studied assumes that the glucose and AMP inputs are constants and that the plasma concentrations of PYR and LAC are constants, as well as the external pH.

Normally, inflows of nutrients into a cell are regulated. For instance, if the energy requirement of a human cell goes up, then the glucose input would be expected to be regulated to increase. The glucose transporters into human cells are carefully regulated (Leney 2009, Petersen 2002, Watson 2006).

Similarly, the AMP input is influenced by other cellular processes. We will look at the effect of the nucleotide salvage pathways in Chapter 12. Through the simulations performed in this chapter, we have discovered that this input rate is important for the long-term function of the system and for the determination of its energy state. The salvage pathways have an important physiological role, and many serious inborn errors of metabolism are associated with genetic defects in this nucleotide
salvage process (Dudzinska 2006, McMullin 1999).

The composition of the plasma is variable. For instance, the normal range of pyruvate concentration in plasma is 0.06 to 0.11 mM, and that for lactate is 0.4 to 1.8 mM. Here, we did fix these conditions to one condition. One can simulate the response to changes in plasma concentrations.

###### Constant volume and charge neutrality¶

As discussed in Chapter 1, there are several fundamental assumptions that underlie the formulation of the dynamic mass balances. These include charge neutrality of the compounds and electroneutrality of the interior and exterior of the cell. The simulator studied here assumed neutral molecules and had no osmotic effects on the volume of the system.

###### Regulation¶

There were no regulatory mechanisms incorporated into this model. In Chapters 14 we will discuss how regulated enzymes are described in MASS models.

##### Summary¶

- The glycolytic pathway is converted in to a system with systems boundary, and inputs and outputs
- The topological characteristics of the stoichiometric matrix are elucidated in terms of three key pathways that span the null space of \(\textbf{S}\) and two pools that span the left null space of \(\textbf{S}\).
- From three flux measurements we can determine the weightings on the three pathways and specify the steady state flux map
- From measured concentrations and the computed flux map, we can calculate the PERCs and complete the steady state description of the glycolytic system.
- Initial draft MASS models can be obtained from using measured concentration values, elementary reactions, and associated mass action kinetics. These first draft kinetic models can be used as a scaffold to build more complicated models that include regulatory effects and interactions with other pathways.
- Dynamic simulations can be performed for perturbations in environmental parameters, and the responses can be examined in terms of the concentrations and the fluxes. Tiled phase portraits are useful to get an overall view of the dynamic response.
- A metabolic map can be analyzed for its stoichiometric texture to assess consequences of cofactor coupling. Such reduction of the biochemical network helps define pools that are physiologically meaningful from a metabolic perspective and are context dependent.
- Some of the responses, namely damped oscillatory behavior, are built into the topological features of a network and require no regulatory action.
- The raw output of the simulation can be post-processed with a pooling matrix that allows the pools and their ratios to be graphed to obtain a deeper interpretation of dynamic responses.
- MASS models, post processing, and analysis of responses allows study at three levels: 1) biochemistry, 2) systems biology, and 3) physiology.

##### Applications¶

###### Examining the constraints a Constant Total Phosphate Pool¶

The total phosphate pool can be a constraint on the capabilities of the glycolytic model. This constraint prevents the model to respond to some environmental stimuli or boundary fluxes. Red blood cells can import/export phosphate from/to the surrounding plasma. Thus if a phosphate transporter is added to the model this unrealistic constraint is removed.

###### Simulating increased glucose uptake rate¶

Here we simulate the response of the glycolytic system without the phosphate transporter to a 20% increase in the glucose uptake rate. Observe what happens to the fraction of phosphate in the recycle pool. We then add a phosphate transporter to alleviate this constraint.

```
[45]:
```

```
t0, tf = (0, 100)
# Simulate and make ratio
ratio_id = 'Phosphate Recycle Ratio'
perturbation_dict = {"kf_SK_glc__D_c": "kf_SK_glc__D_c*1.2"}

phosphate_ratio = {ratio_id: ratios[ratio_id]}
terms, variables = [], []
for k in ['$P^+$', '$P^-$']:
    terms.append(pools[k][0])
    variables.append(pools[k][1])

phosphate_ratio.update({
    "P_tot": ["+".join(terms), list(set(variables[0] + variables[1]))]})

conc_sol, flux_sol = sim_glycolysis.simulate(
    glycolysis, time=(t0, tf, tf*10 + 1),
    perturbations=perturbation_dict)

for ratio, (equation, variables) in phosphate_ratio.items():
    conc_sol.make_aggregate_solution(
        ratio, equation=equation, variables=variables)

fig_10_23, axes = plt.subplots(nrows=2, ncols=1, figsize=(8, 4));
(ax1, ax2) = axes.flatten()

plot_time_profile(
    conc_sol, observable='Phosphate Recycle Ratio', ax=ax1,
    legend="upper right",
    xlabel="Time (hr)", ylabel="Ratio",
    xlim=(t0, tf), ylim=(.95, 1),
    title=(ratio_id, L_FONT));

plot_time_profile(
    flux_sol, observable="ATPM", ax=ax2, legend="upper right",
    xlabel="Time [hr]", ylabel="Flux [mM/hr]",
    xlim=(t0, tf), ylim=(-.001, 3), title=("ATPM Flux", L_FONT));
fig_10_23.tight_layout()
```

**Figure 10.23:** (a) Computation of the phosphate recycle ratio for changes in the glucose uptake rate. Note how high this ratio is. (b) Computation of the ATP usage rate with changes in the glucose uptake rate.

Once the glucose increase hits a certain threshold, the ATP load flux cannot balance, and the system breaks down. At 20% increase in the glucose uptake rate, this ratio hits unity. All the phosphate is found in the recycle pool making it impossible to deliver any energy phosphate bonds. We can examine the ATP usage rate under the same conditions.

###### Adding phosphate exchange reaction with surrounding plasma¶

Now we add a reaction \(v\_P = k\_P^{\rightarrow}(P\_i − P\_{i,plasma})\) where \(P\_{i,plasma} = 2.5\) and \(k\_P^{\rightarrow}\) is 0.23/hr (Prankerd, T., & Altman, K., 1954) and repeat the simulations from above.

```
[46]:
```

```
# Duplicate the model
glycolysis_w_phos = glycolysis.copy()
glycolysis_w_phos.id += "_w_Phosphate"
# Add phosphate exchange reaction with external phosphate concentration set to 2.5 mM
SK_pi_c = glycolysis_w_phos.add_boundary(
    metabolite=glycolysis_w_phos.metabolites.get_by_id("pi_c"),
    boundary_type="sink",  boundary_condition=2.5)

# Set forward rate constant
SK_pi_c.kf = 0.23
SK_pi_c.Keq = 1
```

###### Changes in topological properties¶

The dimensions of \(\textbf{S}\) are now 20x22 and the rank is 19. Thus, the left space in one dimensional but the null space is still 3-dimensional.

Lets examine the left null space of the stoichiometric model. First we see that its dimension has dropped to one, and the only left null space vector is the NAD+NADH pool (Table 10.15). The total phosphate conservation pool has disappeared since we now have a transporter that dynamically adjusts the total phosphate inventory.

**Table 10.15:** The left null space pool of the updated glycolytic system. The total phosphate pool is not found.

```
[47]:
```

```
lns = left_nullspace(glycolysis_w_phos.S, rtol=1e-10)
# Iterate through left nullspace,
# dividing by the smallest value in each row.
for i, row in enumerate(lns):
    minval = np.min(abs(row[np.nonzero(row)]))
    new_row = np.array(row/minval)
    # Round to ensure the left nullspace is composed of only integers
    lns[i] = np.array([round(value) for value in new_row])

# Ensure positive stoichiometric coefficients if all are negative
for i, space in enumerate(lns):
    lns[i] = np.negative(space) if all([num <= 0 for num in space]) else space

# Create labels for the time invariants
table_10_15 = pd.DataFrame(lns, index=["$N_{\mathrm{tot}}$"],
                           columns=metabolite_ids, dtype=np.int64)
table_10_15
```

```
[47]:
```

|  | glc\_\_D\_c | g6p\_c | f6p\_c | fdp\_c | dhap\_c | g3p\_c | \_13dpg\_c | \_3pg\_c | \_2pg\_c | pep\_c | pyr\_c | lac\_\_L\_c | nad\_c | nadh\_c | amp\_c | adp\_c | atp\_c | pi\_c | h\_c | h2o\_c |
| --- | --- | --- | --- | --- | --- | --- | --- | --- | --- | --- | --- | --- | --- | --- | --- | --- | --- | --- | --- | --- |
| $N\_{\mathrm{tot}}$ | 0 | 0 | 0 | 0 | 0 | 0 | 0 | 0 | 0 | 0 | 0 | 0 | 1 | 1 | 0 | 0 | 0 | 0 | 0 | 0 |

###### Dynamic simulation¶

We can now re-simulate the dynamic response of the system to changed glucose uptake rate and we see that the system can now rebalance the phosphate recycle ratio. The model is now more realistic. It re-adjusts the size of the phosphate pool. We can examine how the boundary fluxes change. At 20% increase in the glucose uptake rate the system does reach a new steady state.

```
[48]:
```

```
sim_glycolysis_w_phos = Simulation(glycolysis_w_phos)
conc_sol, flux_sol = sim_glycolysis_w_phos.simulate(
    glycolysis_w_phos, time=(t0, tf, tf*10 + 1),
    perturbations=perturbation_dict)

for ratio, (equation, variables) in phosphate_ratio.items():
    conc_sol.make_aggregate_solution(
        ratio, equation=equation, variables=variables)

fig_10_24, axes = plt.subplots(nrows=2, ncols=1, figsize=(8, 4),
                               );
(ax1, ax2) = axes.flatten()

plot_time_profile(
    conc_sol, observable=ratio_id, ax=ax1, legend="upper right",
    xlabel="Time (hr)", ylabel="Ratio",
    xlim=(t0, tf), ylim=(.95, 1), title=(ratio_id, L_FONT));

plot_time_profile(
    flux_sol, observable="ATPM", ax=ax2, legend="upper right",
    xlabel="Time [hr]", ylabel="Flux [mM/hr]",
    xlim=(t0, tf), ylim=(-.001, 3), title=("ATPM Flux", L_FONT));
fig_10_24.tight_layout()
```

**Figure 10.24:** (a) Computation of the phosphate recycle ratio for changes in the glucose uptake rate in the updated model. (b) Computation of the ATP usage rate with changes in the glucose uptake rate in the updated model.

We can now examine other properties of the response of the updated glycolytic system. The ATP demand can be met at a 20% increase in glucose uptake rate. The size of the total phosphate pool is increased over time. The phase portrait of the total phosphate pool and the phosphate transporter shows that phosphate is taken up (i.e. negative flux on the inorganic phosphate exchange rate, \(v\_P\) and the total phosphate concentration builds up. There is an overshoot and the phosphate is secreted
and the pool size drops and settles down in a new steady state. If you try the simulation at 5% increase in the glucose uptake rate, you will see no such overshoot. The second flux phase portrait shows that AMP leaves the system during this transition.

```
[49]:
```

```
fig_10_25 = plt.figure(figsize=(9, 6))
gs = fig_10_25.add_gridspec(nrows=2, ncols=2, width_ratios=[1, 1],
                            height_ratios=[.5, 1])

ax1 = fig_10_25.add_subplot(gs[0, :])
ax2 = fig_10_25.add_subplot(gs[1, 0])
ax3 = fig_10_25.add_subplot(gs[1, 1])

time_points = time_points[:-1]
time_point_colors = time_point_colors[:-1]
conc_sol.update({SK_pi_c.id: flux_sol[SK_pi_c.id]})

plot_time_profile(
    conc_sol, observable="P_tot", ax=ax1,
    xlim=(t0, tf), ylim =(3, 6),
    xlabel="Time (hr)", ylabel="Pool Size [mM]",
    title=("Total Phosphate Pool", L_FONT))

plot_phase_portrait(
    conc_sol, x="P_tot", y=SK_pi_c.id, ax=ax2,
    xlabel="P_tot [mM]", ylabel="SK_pi_c [mM/hr]",
    title=("Phosphate Phase Portrait", L_FONT),
    annotate_time_points=time_points,
    annotate_time_points_color=time_point_colors)

plot_phase_portrait(
    flux_sol, x="ATPM", y="DM_amp_c", ax=ax3,
    xlabel="ATPM [mM/hr]", ylabel="DM_amp_c [mM/hr]",
    title=("AMP Phase Portrait", L_FONT),
    annotate_time_points=time_points,
    annotate_time_points_color=time_point_colors,
    annotate_time_points_legend="right outside")
fig_10_25.tight_layout()
```

**Figure 10.25:** Plotting response characteristics of the updated glycolytic system.

\(\tiny{\text{© B. Ø. Palsson 2011;}\ \text{This publication is in copyright.}\\ \text{Subject to statutory exception and to the provisions of relevant collective licensing agreements,}\\ \text{no reproduction of any part may take place without the written permission of Cambridge University Press.}}\)

#### Coupling Pathways¶

In Chapter 10 we formulated a mass-action stoichiometric simulation (MASS) model of glycolysis. We took a linear pathway and converted it into an open system with defined inputs and outputs, formed the dynamic mass balances, and then simulated its response to increased rate of energy use. In this chapter, we will show how one can build a dynamic simulation model for two coupled pathways that is based on an integrated stoichiometric scaffold for the two pathways. We start with the pentose pathway
and then couple it to the glycolytic model from Chapter 10 to form a simulation model of two pathways to study their simultaneous dynamic responses.

**MASSpy** will be used to demonstrate some of the topics in this chapter.

```
[1]:
```

```
from mass import (
    MassModel, MassMetabolite, MassReaction,
    Simulation, MassSolution, strip_time)
from mass.test import create_test_model
from mass.util.matrix import nullspace, left_nullspace, matrix_rank
from mass.visualization import (
    plot_time_profile, plot_phase_portrait, plot_tiled_phase_portraits,
    plot_comparison)
```

Other useful packages are also imported at this time.

```
[2]:
```

```
from os import path

from cobra import DictList
import matplotlib as mpl
import matplotlib.pyplot as plt
import numpy as np
import pandas as pd
import sympy as sym
```

Some options and variables used throughout the notebook are also declared here.

```
[3]:
```

```
pd.set_option("display.max_rows", 100)
pd.set_option("display.max_columns", 100)
pd.set_option('display.max_colwidth', None)
pd.options.display.float_format = '{:,.3f}'.format
S_FONT = {"size": "small"}
L_FONT = {"size": "large"}
INF = float("inf")
```

##### The Pentose Phosphate Pathway¶

###### Defining the system¶

The pentose phosphate pathway (PPP) originates from G6P in glycolysis. The pathway is typically thought of as being comprised of two parts: the oxidative and the non-oxidative branches.

**Figure 11.1:** The pentose pathway. The reaction schema, cofactor interactions, and environmental exchanges.

```
[4]:
```

```
ppp = create_test_model("SB2_PentosePhosphatePathway")
```

###### The oxidative branch¶

G6P undergoes two oxidation steps including decarboxylation, releasing \(\text{CO}\_2\), leading to the formation of one pentose and two NADPH molecules. These reactions are called the *oxidative branch* of the pentose pathway. The branch forms two NADPH molecules that are used to form glutathione (GSH) from an oxidized dimeric state, GSSG, by breaking a di-sulfite bond. GSH and GSSG are present in high concentrations, and thus buffer the NADPH redox charge (recall the discussion of the
creatine phosphate buffer in Chapter 8). The pentose formed, R5P, can be used for biosynthesis. We will discuss the connection of R5P with the salvage pathways in Chapter 12.

###### The non-oxidative branch¶

If the pentose formed by the oxidative branch is not used for biosynthetic purposes, it undergoes a number of isomerization, epimerisation, transaldolation, and transketolation reactions that lead to the formation of F6P and GAP. Specifically, two F6P and one GAP that return to glycolysis come from three pentose molecules (i.e., \(3\*5=15\) carbon atoms go to \(2\*6+3=15\) carbon atoms). This part of the pathway is the *non-oxidative branch,* and it is comprised of a series of reversible
reactions, while the oxidative branch is irreversible. The non-oxidative branch can operate in either direction depending on the cell’s physiological state.

###### The overall reaction schema¶

When no pentose is used by other pathways, the overall flow of carbon in the pentose phosphate pathway can be described by

\[\begin{equation} 3\ \text{G6P}\rightarrow 2\ \text{F6P} + \text{GAP} +3\ \text{CO}\_2 \tag{11.1} \end{equation}\]

The input and output from the pathway are glycolytic intermediates. Two redox equivalents of NADPH (or GSH) are produced for every G6P that enters the pathway.

###### Metabolic functions¶

The function of the pentose pathway is to produce redox potential in the form of NADPH and a pentose phosphate that is used for nucleotide synthesis. Also, a 4-carbon intermediate, E4P, is used as a biosynthetic precursor for amino acid synthesis. Exchange reactions for R5P and E4P can then be added to complete the system, as well as a redox load on NADPH.

###### The biochemical reactions¶

The pentose phosphate pathway has twelve compounds (Table 11.1). The pathway has eleven reactions given in (Table 11.2). Their elemental composition is given in Table 11.3. The charge balance is given in Table 11.4. We will consider the removal of R5P, but not of E4P, in this chapter. An exchange reaction for E4P can be added if desired, see the homework section for this chapter.

**Table 11.1:** Pentose pathway intermediates, their abbreviations, and steady state concentrations for the parameter values used in this chapter. The concentrations given are those for the human red blood cell. The index on the compounds is added to that for glycolysis, Table 10.1\*

```
[5]:
```

```
metabolite_ids = [m.id for m in ppp.metabolites
                  if m.id not in ["f6p_c", "g6p_c", "g3p_c", "h_c", "h2o_c"]]

table_11_1 = pd.DataFrame(
    np.array([metabolite_ids,
              [met.name for met in ppp.metabolites
               if met.id in metabolite_ids],
              [ppp.initial_conditions[met] for met in ppp.metabolites
               if met.id in metabolite_ids]]).T,
    index=[i for i in range(21, len(metabolite_ids) + 21)],
    columns=["Abbreviations", "Species", "Initial Concentration"])
table_11_1
```

```
[5]:
```

|  | Abbreviations | Species | Initial Concentration |
| --- | --- | --- | --- |
| 21 | \_6pgl\_c | 6-Phospho-D-gluco-1,5-lactone | 0.00175424 |
| 22 | \_6pgc\_c | 6-Phospho-D-gluconate | 0.0374753 |
| 23 | ru5p\_\_D\_c | D-Ribulose 5-phosphate | 0.00493679 |
| 24 | xu5p\_\_D\_c | D-Xylulose 5-phosphate | 0.0147842 |
| 25 | r5p\_c | Alpha-D-Ribose 5-phosphate | 0.0126689 |
| 26 | s7p\_c | Sedoheptulose 7-phosphate | 0.023988 |
| 27 | e4p\_c | D-Erythrose 4-phosphate | 0.00507507 |
| 28 | nadp\_c | Nicotinamide adenine dinucleotide phosphate | 0.0002 |
| 29 | nadph\_c | Nicotinamide adenine dinucleotide phosphate - reduced | 0.0658 |
| 30 | gthrd\_c | Reduced glutathione | 3.2 |
| 31 | gthox\_c | Oxidized glutathione | 0.12 |
| 32 | co2\_c | CO2 | 1 |

**Table 11.2:** Pentose pathway enzymes and transporters, their abbreviations and chemical reactions. The reactions of the oxidative branch are irreversible, while those of the non-oxidative branch are reversible.

```
[6]:
```

```
reaction_ids = [r.id for r in ppp.reactions
                if r.id not in ["SK_g6p_c", "DM_f6p_c", "DM_g3p_c",
                                "DM_r5p_c", "SK_h_c", "SK_h2o_c"]]
table_11_2 = pd.DataFrame(
    np.array([reaction_ids,
              [r.name for r in ppp.reactions
               if r.id in reaction_ids],
              [r.reaction for r in ppp.reactions
               if r.id in reaction_ids]]).T,
    index=[i for i in range(22, len(reaction_ids) + 22)],
    columns=["Abbreviations", "Enzymes/Transporter/Load", "Elementally Balanced Reaction"])
table_11_2
```

```
[6]:
```

|  | Abbreviations | Enzymes/Transporter/Load | Elementally Balanced Reaction |
| --- | --- | --- | --- |
| 22 | G6PDH2r | Glucose 6-phosphate dehydrogenase | g6p\_c + nadp\_c <=> \_6pgl\_c + h\_c + nadph\_c |
| 23 | PGL | 6-phosphogluconolactonase | \_6pgl\_c + h2o\_c <=> \_6pgc\_c + h\_c |
| 24 | GND | Phosphogluconate dehydrogenase | \_6pgc\_c + nadp\_c <=> co2\_c + nadph\_c + ru5p\_\_D\_c |
| 25 | RPE | Ribulose 5-phosphate 3-epimerase | ru5p\_\_D\_c <=> xu5p\_\_D\_c |
| 26 | RPI | Ribulose 5-Phosphate Isomerase | ru5p\_\_D\_c <=> r5p\_c |
| 27 | TKT1 | Transketolase | r5p\_c + xu5p\_\_D\_c <=> g3p\_c + s7p\_c |
| 28 | TKT2 | Transketolase | e4p\_c + xu5p\_\_D\_c <=> f6p\_c + g3p\_c |
| 29 | TALA | Transaldolase | g3p\_c + s7p\_c <=> e4p\_c + f6p\_c |
| 30 | GTHOr | Glutathione oxidoreductase | gthox\_c + h\_c + nadph\_c <=> 2 gthrd\_c + nadp\_c |
| 31 | GSHR | Glutathione-disulfide reductase | 2 gthrd\_c <=> gthox\_c + 2 h\_c |
| 32 | SK\_co2\_c | CO2 sink | co2\_c <=> |

**Table 11.3:** The elemental composition and charges of the pentose phosphate pathway intermediates. This table represents the matrix \(\textbf{E}.\)\*

```
[7]:
```

```
table_11_3 = ppp.get_elemental_matrix(array_type="DataFrame",
                                      dtype=np.int64)
table_11_3
```

```
[7]:
```

|  | f6p\_c | g6p\_c | g3p\_c | \_6pgl\_c | \_6pgc\_c | ru5p\_\_D\_c | xu5p\_\_D\_c | r5p\_c | s7p\_c | e4p\_c | nadp\_c | nadph\_c | gthrd\_c | gthox\_c | co2\_c | h\_c | h2o\_c |
| --- | --- | --- | --- | --- | --- | --- | --- | --- | --- | --- | --- | --- | --- | --- | --- | --- | --- |
| C | 6 | 6 | 3 | 6 | 6 | 5 | 5 | 5 | 7 | 4 | 21 | 21 | 10 | 20 | 1 | 0 | 0 |
| H | 11 | 11 | 5 | 9 | 10 | 9 | 9 | 9 | 13 | 7 | 25 | 26 | 16 | 30 | 0 | 1 | 2 |
| O | 9 | 9 | 6 | 9 | 10 | 8 | 8 | 8 | 10 | 7 | 17 | 17 | 6 | 12 | 2 | 0 | 1 |
| P | 1 | 1 | 1 | 1 | 1 | 1 | 1 | 1 | 1 | 1 | 3 | 3 | 0 | 0 | 0 | 0 | 0 |
| N | 0 | 0 | 0 | 0 | 0 | 0 | 0 | 0 | 0 | 0 | 7 | 7 | 3 | 6 | 0 | 0 | 0 |
| S | 0 | 0 | 0 | 0 | 0 | 0 | 0 | 0 | 0 | 0 | 0 | 0 | 1 | 2 | 0 | 0 | 0 |
| q | -2 | -2 | -2 | -2 | -3 | -2 | -2 | -2 | -2 | -2 | -3 | -4 | -1 | -2 | 0 | 1 | 0 |
| [NADP] | 0 | 0 | 0 | 0 | 0 | 0 | 0 | 0 | 0 | 0 | 1 | 1 | 0 | 0 | 0 | 0 | 0 |

**Table 11.4:** The elemental and charge balance test on the reactions. All internal reactions are balanced. Exchange reactions are not. Note that the GSHR exchange reaction creates two electrons. This corresponds to the delivery of two electrons to the compound or process that uses redox potential.

```
[8]:
```

```
table_11_4 = ppp.get_elemental_charge_balancing(array_type="DataFrame",
                                                dtype=np.int64)
table_11_4
```

```
[8]:
```

|  | G6PDH2r | PGL | GND | RPE | RPI | TKT1 | TKT2 | TALA | GTHOr | GSHR | SK\_g6p\_c | DM\_f6p\_c | DM\_g3p\_c | DM\_r5p\_c | SK\_co2\_c | SK\_h\_c | SK\_h2o\_c |
| --- | --- | --- | --- | --- | --- | --- | --- | --- | --- | --- | --- | --- | --- | --- | --- | --- | --- |
| C | 0 | 0 | 0 | 0 | 0 | 0 | 0 | 0 | 0 | 0 | 6 | -6 | -3 | -5 | -1 | 0 | 0 |
| H | 0 | 0 | 0 | 0 | 0 | 0 | 0 | 0 | 0 | 0 | 11 | -11 | -5 | -9 | 0 | -1 | -2 |
| O | 0 | 0 | 0 | 0 | 0 | 0 | 0 | 0 | 0 | 0 | 9 | -9 | -6 | -8 | -2 | 0 | -1 |
| P | 0 | 0 | 0 | 0 | 0 | 0 | 0 | 0 | 0 | 0 | 1 | -1 | -1 | -1 | 0 | 0 | 0 |
| N | 0 | 0 | 0 | 0 | 0 | 0 | 0 | 0 | 0 | 0 | 0 | 0 | 0 | 0 | 0 | 0 | 0 |
| S | 0 | 0 | 0 | 0 | 0 | 0 | 0 | 0 | 0 | 0 | 0 | 0 | 0 | 0 | 0 | 0 | 0 |
| q | 0 | 0 | 0 | 0 | 0 | 0 | 0 | 0 | 0 | 2 | -2 | 2 | 2 | 2 | 0 | -1 | 0 |
| [NADP] | 0 | 0 | 0 | 0 | 0 | 0 | 0 | 0 | 0 | 0 | 0 | 0 | 0 | 0 | 0 | 0 | 0 |

Table 11.4 shows that \(\textbf{ES} = 0\) for all non-exchange reactions in the pentose phosphate pathway model. Thus the reactions are charge and elementally balanced. The model passes this QC/QA test.

```
[9]:
```

```
for boundary in ppp.boundary:
    print(boundary)
```

```
SK_g6p_c:  <=> g6p_c
DM_f6p_c: f6p_c -->
DM_g3p_c: g3p_c -->
DM_r5p_c: r5p_c -->
SK_co2_c: co2_c <=>
SK_h_c: h_c <=>
SK_h2o_c: h2o_c <=>
```

###### The pathway structure: basis for the null space¶

The null space is spanned by two vectors, \(p\_1\) and \(p\_2\), that have pathway interpretations as for glycolysis. They are graphically shown in Figure 11.2.

**Table 11.5:** The calculated MinSpan pathway vectors for the stoichiometric matrix for the glycolytic system.

```
[10]:
```

```
reaction_ids = [r.id for r in ppp.reactions]
# MinSpan pathways are calculated outside this notebook and the results are provided here.
minspan_paths = np.array([
    [1, 1, 1, 2/3, 1/3, 1/3, 1/3, 1/3, 2, 2, 1, 2/3, 1/3, 0, 1, 4, -1],
    [1, 1, 1,  0,   1,   0,   0,   0,  2, 2, 1,  0,   0,  1, 1, 4, -1]])
# Round to the 3rd decimal point for minspan_paths
minspan_paths = np.array([[round(val, 3) for val in row]
                          for row in minspan_paths])
# Create labels for the paths
path_labels = ["$p_1$","$p_2$"]
# Create DataFrame
table_11_5 = pd.DataFrame(minspan_paths, index=path_labels,
                          columns=reaction_ids)
table_11_5
```

```
[10]:
```

|  | G6PDH2r | PGL | GND | RPE | RPI | TKT1 | TKT2 | TALA | GTHOr | GSHR | SK\_g6p\_c | DM\_f6p\_c | DM\_g3p\_c | DM\_r5p\_c | SK\_co2\_c | SK\_h\_c | SK\_h2o\_c |
| --- | --- | --- | --- | --- | --- | --- | --- | --- | --- | --- | --- | --- | --- | --- | --- | --- | --- |
| $p\_1$ | 1.000 | 1.000 | 1.000 | 0.667 | 0.333 | 0.333 | 0.333 | 0.333 | 2.000 | 2.000 | 1.000 | 0.667 | 0.333 | 0.000 | 1.000 | 4.000 | -1.000 |
| $p\_2$ | 1.000 | 1.000 | 1.000 | 0.000 | 1.000 | 0.000 | 0.000 | 0.000 | 2.000 | 2.000 | 1.000 | 0.000 | 0.000 | 1.000 | 1.000 | 4.000 | -1.000 |

The pathways are biochemically interpreted as follows:

1. The first pathway describes the oxidative branch of the PPP to form one R5P and one CO2. Two redox equivalents in the form of GSH are made.
2. The second pathway is the classical complete conversion of one G6P to 2/3 F6P and 1/3 GAP with the formation of two redox equivalents

**Figure 11.2:** The two pathway vectors that span the null space of \(\textbf{S}\) for the PPP as shown in Table 11.2.

###### Elemental balancing of the pathway vectors¶

Each of the pathway vectors needs to be elementally balanced, which means the elements coming into and leaving a pathway through the transporters need to be balanced. Thus we must have \(\textbf{ESp}\_i = 0\) for each pathway. This condition is satisfied for these two pathways.

**Table 11.6:** Elemental and charge balancing on the MinSpan pathways of the Pentose Phosphate Pathway.

```
[11]:
```

```
table_11_6 = ppp.get_elemental_charge_balancing(array_type="DataFrame").dot(minspan_paths.T).astype(np.int64)
table_11_6.columns = path_labels
table_11_6
```

```
[11]:
```

|  | $p\_1$ | $p\_2$ |
| --- | --- | --- |
| C | 0 | 0 |
| H | 0 | 0 |
| O | 0 | 0 |
| P | 0 | 0 |
| N | 0 | 0 |
| S | 0 | 0 |
| q | 0 | 0 |
| [NADP] | 0 | 0 |

###### The time invariant pools: the basis for the left null space¶

The stoichiometric matrix has a left null space of dimension 2, meaning the pentose phosphate pathway as defined has two time invariant pools. These can be found in the *Left Nullspace* of the pentose phosphate pathway model. We can compute them directly:

**Table 11.7:** The left null space vectors of the stoichiometric matrix for the Pentose Phosphate Pathway.

```
[12]:
```

```
metabolite_ids = [m.id for m in ppp.metabolites]
lns = left_nullspace(ppp.S, rtol=1e-10)
# Iterate through left nullspace,
# dividing by the smallest value in each row.
for i, row in enumerate(lns):
    minval = np.min(abs(row[np.nonzero(row)]))
    new_row = np.array(row/minval)
    # Round to ensure the left nullspace is composed of only integers
    lns[i] = np.array([round(value) for value in new_row])

# Row operations to find biologically meaningful pools
lns[1] = (-lns[1]*17 + lns[0])/120
lns[0] =(lns[1] - lns[0])/17
# Ensure positive stoichiometric coefficients if all are negative
for i, space in enumerate(lns):
    lns[i] = np.negative(space) if all([num <= 0 for num in space]) else space

# Create labels for the time invariants
time_inv_labels = ["$NP_{\mathrm{tot}}$", "$G_{\mathrm{tot}}$"]
table_11_7 = pd.DataFrame(lns, index=time_inv_labels,
                          columns=metabolite_ids, dtype=np.int64)
table_11_7
```

```
[12]:
```

|  | f6p\_c | g6p\_c | g3p\_c | \_6pgl\_c | \_6pgc\_c | ru5p\_\_D\_c | xu5p\_\_D\_c | r5p\_c | s7p\_c | e4p\_c | nadp\_c | nadph\_c | gthrd\_c | gthox\_c | co2\_c | h\_c | h2o\_c |
| --- | --- | --- | --- | --- | --- | --- | --- | --- | --- | --- | --- | --- | --- | --- | --- | --- | --- |
| $NP\_{\mathrm{tot}}$ | 0 | 0 | 0 | 0 | 0 | 0 | 0 | 0 | 0 | 0 | 1 | 1 | 0 | 0 | 0 | 0 | 0 |
| $G\_{\mathrm{tot}}$ | 0 | 0 | 0 | 0 | 0 | 0 | 0 | 0 | 0 | 0 | 0 | 0 | 1 | 2 | 0 | 0 | 0 |

\(\textbf{1.}\) The first time invariant is the total amount of the NADP moiety

\(\textbf{2.}\) The second time invariant is the total amount of the glutathione moiety, GS-.

###### An ‘annotated’ form of the stoichiometric matrix¶

All of the properties of the stoichiometric matrix can be conveniently summarized in a tabular format, Table 11.8. The table succinctly summarizes the chemical and topological properties of \(\textbf{S}\). The matrix has dimensions of 17x17 and a rank of 15. It thus has a 2 dimensional null space and a two dimensional left null space.

**Table 11.8:** The stoichiometric matrix for the Pentose Phosphate Pathway seen in Figure 11.1. The matrix is partitioned to show the intermediates (yellow) separate from the cofactors and to separate the exchange reactions and cofactor loads (orange). The connectivities, \(\rho\_i\)(red), for a compound, and the participation number, \(\pi\_j\) (cyan), for a reaction are shown. The second block in the table is the product \(\textbf{ES}\) (blue) to evaluate elemental balancing
status of the reactions. All exchange reactions have a participation number of unity and are thus not elementally balanced. The last block in the table has the three pathway vectors (purple) for the pentose phosphate pathway. These vectors are graphically shown in Figure 11.2. Furthest to the right, we display the two time invariant pools (green) that span the left null space.

```
[13]:
```

```
# Define labels
pi_str = r"$\pi_{j}$"
rho_str = r"$\rho_{i}$"
chopsnq = ['C', 'H', 'O', 'P', 'N', 'S', 'q', '[NADP]']

# Make table content from the stoichiometric matrix, elemental balancing of pathways
# participation number, and MinSpan pathways
S_matrix = ppp.update_S(array_type="dense", dtype=np.int64, update_model=False)
ES_matrix = ppp.get_elemental_charge_balancing(dtype=np.int64)
pi = np.count_nonzero(S_matrix, axis=0)
rho = np.count_nonzero(S_matrix, axis=1)
table_11_8 = np.vstack((S_matrix, pi, ES_matrix, minspan_paths))

# Determine number of blank entries needed to be added to pad the table,
# Add connectivity number and time invariants to table content
blanks = [""]*(len(table_11_8) - len(metabolite_ids))
rho = np.concatenate((rho, blanks))
time_inv = np.array([np.concatenate([row, blanks]) for row in lns])
table_11_8 = np.vstack([table_11_8.T, rho, time_inv]).T

colors = {"intermediates": "#ffffe6", # Yellow
          "cofactors": "#ffe6cc",     # Orange
          "chopsnq": "#99e6ff",       # Blue
          "pathways": "#b399ff",      # Purple
          "pi": "#99ffff",            # Cyan
          "rho": "#ff9999",           # Red
          "time_invs": "#ccff99",     # Green
          "blank": "#f2f2f2"}         # Grey
bg_color_str = "background-color: "
def highlight_table(df, model, main_shape):
    df = df.copy()
    n_mets, n_rxns = (len(model.metabolites), len(model.reactions))
    # Highlight rows
    for row in df.index:
        other_key, condition = ("blank", lambda i, v: v != "")
        if row == pi_str:        # For participation
            main_key = "pi"
        elif row in chopsnq:     # For elemental balancing
            main_key = "chopsnq"
        elif row in path_labels: # For pathways
            main_key = "pathways"
        else:
            # Distinguish between intermediate and cofactor reactions for model reactions
            main_key, other_key = ("cofactors", "intermediates")
            condition = lambda i, v: (main_shape[1] <= i and i < n_rxns)
        df.loc[row, :] = [bg_color_str + colors[main_key] if condition(i, v)
                          else bg_color_str + colors[other_key]
                          for i, v in enumerate(df.loc[row, :])]

    for col in df.columns:
        condition = lambda i, v: v != bg_color_str + colors["blank"]
        if col == rho_str:
            main_key = "rho"
        elif col in time_inv_labels:
            main_key = "time_invs"
        else:
            # Distinguish intermediates and cofactors for model metabolites
            main_key = "cofactors"
            condition = lambda i, v: (main_shape[0] <= i and i < n_mets)
        df.loc[:, col] = [bg_color_str + colors[main_key] if condition(i, v)
                          else v for i, v in enumerate(df.loc[:, col])]
    return df

# Create index and column labels
index_labels = np.concatenate((metabolite_ids, [pi_str], chopsnq, path_labels))
column_labels = np.concatenate((reaction_ids, [rho_str], time_inv_labels))
# Create DataFrame
table_11_8 = pd.DataFrame(
    table_11_8, index=index_labels, columns=column_labels)
# Apply colors
table_11_8 = table_11_8.style.apply(
    highlight_table,  model=ppp, main_shape=(10, 10), axis=None)
table_11_8
```

```
[13]:
```

|  | G6PDH2r | PGL | GND | RPE | RPI | TKT1 | TKT2 | TALA | GTHOr | GSHR | SK\_g6p\_c | DM\_f6p\_c | DM\_g3p\_c | DM\_r5p\_c | SK\_co2\_c | SK\_h\_c | SK\_h2o\_c | $\rho\_{i}$ | $NP\_{\mathrm{tot}}$ | $G\_{\mathrm{tot}}$ |
| --- | --- | --- | --- | --- | --- | --- | --- | --- | --- | --- | --- | --- | --- | --- | --- | --- | --- | --- | --- | --- |
| f6p\_c | 0.0 | 0.0 | 0.0 | 0.0 | 0.0 | 0.0 | 1.0 | 1.0 | 0.0 | 0.0 | 0.0 | -1.0 | 0.0 | 0.0 | 0.0 | 0.0 | 0.0 | 3 | 0.0 | 0.0 |
| g6p\_c | -1.0 | 0.0 | 0.0 | 0.0 | 0.0 | 0.0 | 0.0 | 0.0 | 0.0 | 0.0 | 1.0 | 0.0 | 0.0 | 0.0 | 0.0 | 0.0 | 0.0 | 2 | 0.0 | 0.0 |
| g3p\_c | 0.0 | 0.0 | 0.0 | 0.0 | 0.0 | 1.0 | 1.0 | -1.0 | 0.0 | 0.0 | 0.0 | 0.0 | -1.0 | 0.0 | 0.0 | 0.0 | 0.0 | 4 | 0.0 | 0.0 |
| \_6pgl\_c | 1.0 | -1.0 | 0.0 | 0.0 | 0.0 | 0.0 | 0.0 | 0.0 | 0.0 | 0.0 | 0.0 | 0.0 | 0.0 | 0.0 | 0.0 | 0.0 | 0.0 | 2 | 0.0 | 0.0 |
| \_6pgc\_c | 0.0 | 1.0 | -1.0 | 0.0 | 0.0 | 0.0 | 0.0 | 0.0 | 0.0 | 0.0 | 0.0 | 0.0 | 0.0 | 0.0 | 0.0 | 0.0 | 0.0 | 2 | 0.0 | 0.0 |
| ru5p\_\_D\_c | 0.0 | 0.0 | 1.0 | -1.0 | -1.0 | 0.0 | 0.0 | 0.0 | 0.0 | 0.0 | 0.0 | 0.0 | 0.0 | 0.0 | 0.0 | 0.0 | 0.0 | 3 | 0.0 | 0.0 |
| xu5p\_\_D\_c | 0.0 | 0.0 | 0.0 | 1.0 | 0.0 | -1.0 | -1.0 | 0.0 | 0.0 | 0.0 | 0.0 | 0.0 | 0.0 | 0.0 | 0.0 | 0.0 | 0.0 | 3 | 0.0 | 0.0 |
| r5p\_c | 0.0 | 0.0 | 0.0 | 0.0 | 1.0 | -1.0 | 0.0 | 0.0 | 0.0 | 0.0 | 0.0 | 0.0 | 0.0 | -1.0 | 0.0 | 0.0 | 0.0 | 3 | 0.0 | 0.0 |
| s7p\_c | 0.0 | 0.0 | 0.0 | 0.0 | 0.0 | 1.0 | 0.0 | -1.0 | 0.0 | 0.0 | 0.0 | 0.0 | 0.0 | 0.0 | 0.0 | 0.0 | 0.0 | 2 | 0.0 | 0.0 |
| e4p\_c | 0.0 | 0.0 | 0.0 | 0.0 | 0.0 | 0.0 | -1.0 | 1.0 | 0.0 | 0.0 | 0.0 | 0.0 | 0.0 | 0.0 | 0.0 | 0.0 | 0.0 | 2 | 0.0 | 0.0 |
| nadp\_c | -1.0 | 0.0 | -1.0 | 0.0 | 0.0 | 0.0 | 0.0 | 0.0 | 1.0 | 0.0 | 0.0 | 0.0 | 0.0 | 0.0 | 0.0 | 0.0 | 0.0 | 3 | 1.0 | 0.0 |
| nadph\_c | 1.0 | 0.0 | 1.0 | 0.0 | 0.0 | 0.0 | 0.0 | 0.0 | -1.0 | 0.0 | 0.0 | 0.0 | 0.0 | 0.0 | 0.0 | 0.0 | 0.0 | 3 | 1.0 | 0.0 |
| gthrd\_c | 0.0 | 0.0 | 0.0 | 0.0 | 0.0 | 0.0 | 0.0 | 0.0 | 2.0 | -2.0 | 0.0 | 0.0 | 0.0 | 0.0 | 0.0 | 0.0 | 0.0 | 2 | 0.0 | 1.0 |
| gthox\_c | 0.0 | 0.0 | 0.0 | 0.0 | 0.0 | 0.0 | 0.0 | 0.0 | -1.0 | 1.0 | 0.0 | 0.0 | 0.0 | 0.0 | 0.0 | 0.0 | 0.0 | 2 | 0.0 | 2.0 |
| co2\_c | 0.0 | 0.0 | 1.0 | 0.0 | 0.0 | 0.0 | 0.0 | 0.0 | 0.0 | 0.0 | 0.0 | 0.0 | 0.0 | 0.0 | -1.0 | 0.0 | 0.0 | 2 | 0.0 | 0.0 |
| h\_c | 1.0 | 1.0 | 0.0 | 0.0 | 0.0 | 0.0 | 0.0 | 0.0 | -1.0 | 2.0 | 0.0 | 0.0 | 0.0 | 0.0 | 0.0 | -1.0 | 0.0 | 5 | 0.0 | 0.0 |
| h2o\_c | 0.0 | -1.0 | 0.0 | 0.0 | 0.0 | 0.0 | 0.0 | 0.0 | 0.0 | 0.0 | 0.0 | 0.0 | 0.0 | 0.0 | 0.0 | 0.0 | -1.0 | 2 | 0.0 | 0.0 |
| $\pi\_{j}$ | 5.0 | 4.0 | 5.0 | 2.0 | 2.0 | 4.0 | 4.0 | 4.0 | 5.0 | 3.0 | 1.0 | 1.0 | 1.0 | 1.0 | 1.0 | 1.0 | 1.0 |  |  |  |
| C | 0.0 | 0.0 | 0.0 | 0.0 | 0.0 | 0.0 | 0.0 | 0.0 | 0.0 | 0.0 | 6.0 | -6.0 | -3.0 | -5.0 | -1.0 | 0.0 | 0.0 |  |  |  |
| H | 0.0 | 0.0 | 0.0 | 0.0 | 0.0 | 0.0 | 0.0 | 0.0 | 0.0 | 0.0 | 11.0 | -11.0 | -5.0 | -9.0 | 0.0 | -1.0 | -2.0 |  |  |  |
| O | 0.0 | 0.0 | 0.0 | 0.0 | 0.0 | 0.0 | 0.0 | 0.0 | 0.0 | 0.0 | 9.0 | -9.0 | -6.0 | -8.0 | -2.0 | 0.0 | -1.0 |  |  |  |
| P | 0.0 | 0.0 | 0.0 | 0.0 | 0.0 | 0.0 | 0.0 | 0.0 | 0.0 | 0.0 | 1.0 | -1.0 | -1.0 | -1.0 | 0.0 | 0.0 | 0.0 |  |  |  |
| N | 0.0 | 0.0 | 0.0 | 0.0 | 0.0 | 0.0 | 0.0 | 0.0 | 0.0 | 0.0 | 0.0 | 0.0 | 0.0 | 0.0 | 0.0 | 0.0 | 0.0 |  |  |  |
| S | 0.0 | 0.0 | 0.0 | 0.0 | 0.0 | 0.0 | 0.0 | 0.0 | 0.0 | 0.0 | 0.0 | 0.0 | 0.0 | 0.0 | 0.0 | 0.0 | 0.0 |  |  |  |
| q | 0.0 | 0.0 | 0.0 | 0.0 | 0.0 | 0.0 | 0.0 | 0.0 | 0.0 | 2.0 | -2.0 | 2.0 | 2.0 | 2.0 | 0.0 | -1.0 | 0.0 |  |  |  |
| [NADP] | 0.0 | 0.0 | 0.0 | 0.0 | 0.0 | 0.0 | 0.0 | 0.0 | 0.0 | 0.0 | 0.0 | 0.0 | 0.0 | 0.0 | 0.0 | 0.0 | 0.0 |  |  |  |
| $p\_1$ | 1.0 | 1.0 | 1.0 | 0.667 | 0.333 | 0.333 | 0.333 | 0.333 | 2.0 | 2.0 | 1.0 | 0.667 | 0.333 | 0.0 | 1.0 | 4.0 | -1.0 |  |  |  |
| $p\_2$ | 1.0 | 1.0 | 1.0 | 0.0 | 1.0 | 0.0 | 0.0 | 0.0 | 2.0 | 2.0 | 1.0 | 0.0 | 0.0 | 1.0 | 1.0 | 4.0 | -1.0 |  |  |  |

###### The steady state¶

If we fix the flux into the pathway, \(v\_{SK\_{G6P}}\), and the exit flux of R5P, \(v\_{DM\_{R5P}}\), then we fix the steady state flux distribution since these fluxes uniquely specify the pathway fluxes. We set these numbers at 0.21 mM/hr and 0.01 mM/hr, respectively. The former value represents a typical flux through the pentose pathway while the latter will couple at a low flux level to the AMP pathways treated in the next chapter. Using \(\textbf{p}\_1 + \textbf{p}\_2=0.21\) and
\(\textbf{p}\_1=0.01\) we can compute the steady state.

```
[14]:
```

```
# Set independent fluxes to determine steady state flux vector
independent_fluxes = {
    ppp.reactions.SK_g6p_c: 0.21,
    ppp.reactions.DM_r5p_c: 0.01}

# Compute steady state fluxes
ssfluxes = ppp.compute_steady_state_fluxes(
    minspan_paths,
    independent_fluxes,
    update_reactions=True)

table_11_9 = pd.DataFrame(list(ssfluxes.values()), index=reaction_ids,
                          columns=[r"$\textbf{v}_{\mathrm{stst}}$"]).T
```

**Table 11.9:** Computing the steady state fluxes as a summation of the MinSpan pathway vectors.

```
[15]:
```

```
table_11_9
```

```
[15]:
```

|  | G6PDH2r | PGL | GND | RPE | RPI | TKT1 | TKT2 | TALA | GTHOr | GSHR | SK\_g6p\_c | DM\_f6p\_c | DM\_g3p\_c | DM\_r5p\_c | SK\_co2\_c | SK\_h\_c | SK\_h2o\_c |
| --- | --- | --- | --- | --- | --- | --- | --- | --- | --- | --- | --- | --- | --- | --- | --- | --- | --- |
| $\textbf{v}\_{\mathrm{stst}}$ | 0.210 | 0.210 | 0.210 | 0.133 | 0.077 | 0.067 | 0.067 | 0.067 | 0.420 | 0.420 | 0.210 | 0.133 | 0.067 | 0.010 | 0.210 | 0.840 | -0.210 |

and can be visualized as a bar chart:

```
[16]:
```

```
fig_11_3, ax = plt.subplots(nrows=1, ncols=1, figsize=(10, 5))
# Define indicies for bar chart
indicies = np.arange(len(reaction_ids))+0.5
# Define colors to use
c = plt.cm.coolwarm(np.linspace(0, 1, len(reaction_ids)))
# Plot bar chart
ax.bar(indicies, list(ssfluxes.values()), width=0.8, color=c);
ax.set_xlim([0, len(reaction_ids)]);
# Set labels and adjust ticks
ax.set_xticks(indicies);
ax.set_xticklabels(reaction_ids, rotation="vertical");
ax.set_ylabel("Fluxes (mM/hr)", L_FONT);
ax.set_title("Steady State Fluxes", L_FONT);
# Add a dashed line at 0
ax.plot([0, len(reaction_ids)], [0, 0], "k--");
fig_11_3.tight_layout()
```

**Figure 11.3:** Bar chart of the steady-state fluxes.

We can perform a numerical check make sure that we have a steady state flux vector by performing the multiplication \(\textbf{Sv}\_{\mathrm{stst}}\) that should yield zero.

**A numerical QC/QA:** Ensure \(\textbf{Sv}\_{\mathrm{stst}} = 0\)

```
[17]:
```

```
pd.DataFrame(
    ppp.S.dot(np.array(list(ssfluxes.values()))),
    index=metabolite_ids,
    columns=[r"$\textbf{Sv}_{\mathrm{stst}}$"],
    dtype=np.int64).T
```

```
[17]:
```

|  | f6p\_c | g6p\_c | g3p\_c | \_6pgl\_c | \_6pgc\_c | ru5p\_\_D\_c | xu5p\_\_D\_c | r5p\_c | s7p\_c | e4p\_c | nadp\_c | nadph\_c | gthrd\_c | gthox\_c | co2\_c | h\_c | h2o\_c |
| --- | --- | --- | --- | --- | --- | --- | --- | --- | --- | --- | --- | --- | --- | --- | --- | --- | --- |
| $\textbf{Sv}\_{\mathrm{stst}}$ | 0 | 0 | 0 | 0 | 0 | 0 | 0 | 0 | 0 | 0 | 0 | 0 | 0 | 0 | 0 | 0 | 0 |

###### Computing the PERCs¶

The approximate steady state values of the metabolites are given above. The mass action ratios can be computed from these steady state concentrations. We can also compute the forward rate constant for the reactions:

```
[18]:
```

```
percs = ppp.calculate_PERCs(update_reactions=True)
```

**Table 11.10:** Pentose phosphate pathway enzymes, loads, transport rates, and their abbreviations. For irreversible reactions, the numerical value for the equilibrium constants is \(\infty\), which, for practical reasons, can be set to a finite value.

```
[19]:
```

```
# Get concentration values for substitution into sympy expressions
value_dict = {sym.Symbol(str(met)): ic
              for met, ic in ppp.initial_conditions.items()}
value_dict.update({sym.Symbol(str(met)): bc
                   for met, bc in ppp.boundary_conditions.items()})

table_11_10 = []
# Get symbols and values for table and substitution
for p_key in ["Keq", "kf"]:
    symbol_list, value_list = [], []
    for p_str, value in ppp.parameters[p_key].items():
        symbol_list.append(r"$%s_{\text{%s}}$" % (p_key[0], p_str.split("_", 1)[-1]))
        value_list.append("{0:.3f}".format(value) if value != INF else r"$\infty$")
        value_dict.update({sym.Symbol(p_str): value})
    table_11_10.extend([symbol_list, value_list])

table_11_10.append(["{0:.6f}".format(float(ratio.subs(value_dict)))
                    for ratio in strip_time(ppp.get_mass_action_ratios()).values()])
table_11_10.append(["{0:.6f}".format(float(ratio.subs(value_dict)))
                    for ratio in strip_time(ppp.get_disequilibrium_ratios()).values()])
table_11_10 = pd.DataFrame(np.array(table_11_10).T, index=reaction_ids,
                           columns=[r"$K_{eq}$ Symbol", r"$K_{eq}$ Value", "PERC Symbol",
                                    "PERC Value", r"$\Gamma$", r"$\Gamma/K_{eq}$"])
table_11_10
```

```
[19]:
```

|  | $K\_{eq}$ Symbol | $K\_{eq}$ Value | PERC Symbol | PERC Value | $\Gamma$ | $\Gamma/K\_{eq}$ |
| --- | --- | --- | --- | --- | --- | --- |
| G6PDH2r | $K\_{\text{G6PDH2r}}$ | 1000.000 | $k\_{\text{G6PDH2r}}$ | 21864.589 | 11.875411 | 0.011875 |
| PGL | $K\_{\text{PGL}}$ | 1000.000 | $k\_{\text{PGL}}$ | 122.323 | 21.362698 | 0.021363 |
| GND | $K\_{\text{GND}}$ | 1000.000 | $k\_{\text{GND}}$ | 29287.807 | 43.340651 | 0.043341 |
| RPE | $K\_{\text{RPE}}$ | 3.000 | $k\_{\text{RPE}}$ | 15292.319 | 2.994699 | 0.998233 |
| RPI | $K\_{\text{RPI}}$ | 2.570 | $k\_{\text{RPI}}$ | 10555.433 | 2.566222 | 0.998530 |
| TKT1 | $K\_{\text{TKT1}}$ | 1.200 | $k\_{\text{TKT1}}$ | 1594.356 | 0.932371 | 0.776976 |
| TKT2 | $K\_{\text{TKT2}}$ | 10.300 | $k\_{\text{TKT2}}$ | 1091.154 | 1.921130 | 0.186517 |
| TALA | $K\_{\text{TALA}}$ | 1.050 | $k\_{\text{TALA}}$ | 843.772 | 0.575416 | 0.548015 |
| GTHOr | $K\_{\text{GTHOr}}$ | 100.000 | $k\_{\text{GTHOr}}$ | 53.330 | 0.259372 | 0.002594 |
| GSHR | $K\_{\text{GSHR}}$ | 2.000 | $k\_{\text{GSHR}}$ | 0.041 | 0.011719 | 0.005859 |
| SK\_g6p\_c | $K\_{\text{SK\_g6p\_c}}$ | 1.000 | $k\_{\text{SK\_g6p\_c}}$ | 0.221 | 0.048600 | 0.048600 |
| DM\_f6p\_c | $K\_{\text{DM\_f6p\_c}}$ | $\infty$ | $k\_{\text{DM\_f6p\_c}}$ | 6.737 | 50.505051 | 0.000000 |
| DM\_g3p\_c | $K\_{\text{DM\_g3p\_c}}$ | $\infty$ | $k\_{\text{DM\_g3p\_c}}$ | 9.148 | 137.362637 | 0.000000 |
| DM\_r5p\_c | $K\_{\text{DM\_r5p\_c}}$ | $\infty$ | $k\_{\text{DM\_r5p\_c}}$ | 0.789 | 78.933451 | 0.000000 |
| SK\_co2\_c | $K\_{\text{SK\_co2\_c}}$ | 1.000 | $k\_{\text{SK\_co2\_c}}$ | 100000.000 | 1.000000 | 1.000000 |
| SK\_h\_c | $K\_{\text{SK\_h\_c}}$ | 1.000 | $k\_{\text{SK\_h\_c}}$ | 100000.000 | 0.882510 | 0.882510 |
| SK\_h2o\_c | $K\_{\text{SK\_h2o\_c}}$ | 1.000 | $k\_{\text{SK\_h2o\_c}}$ | 100000.000 | 1.000000 | 1.000000 |

These estimates for the numerical values for the PERCs are shown in Table 11.10. These numerical values, along with the elementary form of the rate laws, complete the definition of the dynamic mass balances that can now be simulated. The steady state is specified in Table 11.9.

###### Dynamic response: increased rate of R5P production¶

One function of the pentose pathway is to provide R5P for biosynthesis. We thus simulate its response to an increased rate of R5P use. We increase the value of \(k\_{DM\_{R5P}}\), ten-fold at time zero and simulate the response. The responses in best interpreted in terms overall flux balance on the pathway; Figure 11.4

```
[20]:
```

```
t0, tf = (0, 100)
sim_ppp = Simulation(ppp)
conc_sol, flux_sol = sim_ppp.simulate(
    ppp, time=(t0, tf, tf*10 + 1),
    perturbations={"kf_DM_r5p_c": "kf_DM_r5p_c * 10"})

fig_11_4 = plt.figure(figsize=(17, 6))
gs = fig_11_4.add_gridspec(nrows=3, ncols=2, width_ratios=[1.5, 1])

ax1 = fig_11_4.add_subplot(gs[0, 0])
ax2 = fig_11_4.add_subplot(gs[1, 0])
ax3 = fig_11_4.add_subplot(gs[2, 0])
ax4 = fig_11_4.add_subplot(gs[:, 1])

plot_time_profile(
    conc_sol, observable="r5p_c", ax=ax1,
    legend="right outside", plot_function="semilogx",
    xlim=(1e-6, tf), ylim=(0.009, 0.014),
    xlabel="Time [hr]", ylabel="Concentrations [mM]",
    title=("(a) R5P Concentration", L_FONT));

fluxes_in = ["RPI"]
plot_time_profile(
    flux_sol, observable=fluxes_in, ax=ax2,
    legend="right outside", plot_function="semilogx",
    xlim=(1e-6, tf),  ylim=(0, .15),
    xlabel="Time [hr]", ylabel="Fluxes [mM/hr]",
    title=("(b) Fluxes in", L_FONT));

fluxes_out = ["DM_r5p_c", "TKT1"]
plot_time_profile(
    flux_sol, observable=fluxes_out, ax=ax3,
    legend="right outside", plot_function="semilogx",
    xlim=(1e-6, tf), ylim=(0.03, .11),
    xlabel="Time [hr]", ylabel="Fluxes [mM/hr]",
    title=("(c) Fluxes out", L_FONT));

for flux_id, variables in zip(["Net_Flux_In", "Net_Flux_Out"],
                              [fluxes_in, fluxes_out]):
    flux_sol.make_aggregate_solution(
        flux_id, equation=" + ".join(variables), variables=variables)

time_points = [t0, 1e-1, 1e0, 1e1, 1e2, tf]
time_point_colors = [
    mpl.colors.to_hex(c)
    for c in mpl.cm.Reds(np.linspace(0.3, 1, len(time_points)))]

plot_phase_portrait(
    flux_sol, x="Net_Flux_In", y="Net_Flux_Out", ax=ax4,
    xlim=(0.07, 0.14), ylim=(0.11, 0.17),
    xlabel="Fluxes in [mm/Hr]", ylabel="Fluxes out [mm/Hr]",
    title=("(d) Phase Portrait of Fluxes", L_FONT),
    annotate_time_points=time_points,
    annotate_time_points_color=time_point_colors,
    annotate_time_points_legend="best");
fig_11_4.tight_layout()
```

**Figure 11.4:** The time profiles of the (a) R5P concentration, (b) the fluxes that make R5P, (c) the fluxes that use R5P and (d) the phase portrait of the net flux in and net flux out (darker red colors indicate slower time scales).

The initial perturbation creates an imbalance on the R5P node as seen in Figure 11.4. These fluxes must balance in the steady state as indicated by the 45 degree line where the rate of formation and use balance, Figure 11.5a. Initially, \(v\_{DM\_{R5P}}\) is instantaneously increased \((t = 0^+)\). The immediate response is a compensating increase of production by \(v\_{RPI}\), a rapidly equilibrating enzyme. The utilization of R5P by the non-oxidative branch then drops and steady state
is reached to balance the increased removal rate of R5P from the system.

The overall steady state flux balance states that the sum of the three fluxes leaving the network have to be balanced by the constant input of 0.21 mM/hr \((= v\_{DM\_{F6P}} + v\_{DM\_{G3P}} + v\_{DM\_{R5P}})\) as indicated by the -45 degree line in Figure 11.5b. Initially, \(v\_{DM\_{R5P}}\) increases ten-fold. Over time, the return flux to glycolysis, \(v\_{DM\_{F6P}} + v\_{DM\_{G3P}}\), decreases, until a steady state is reached.

```
[21]:
```

```
fig_11_5, axes = plt.subplots(nrows=1, ncols=2, figsize=(11, 5))
(ax1, ax2) = axes.flatten()

R5P_form = ["DM_r5p_c", "TKT1"]
flux_out = ["DM_f6p_c", "DM_g3p_c"]


for flux_id, variables in zip(["R5P_form", "flux_out"],
                              [R5P_form, flux_out]):
    flux_sol.make_aggregate_solution(
        flux_id, equation=" + ".join(variables), variables=variables)

time_points = [t0, 1e-1, 1e0, 1e1, 1e2, tf]
time_point_colors = [
    mpl.colors.to_hex(c)
    for c in mpl.cm.Reds(np.linspace(0.3, 1, len(time_points)))]

plot_phase_portrait(
    flux_sol, x="R5P_form", y="RPI", ax=ax1,
    xlabel="R5P_form", ylabel="RPI", xlim=(.06, .17), ylim=(.06, .17),
    title=("(a) R5P_form vs. RPI", L_FONT),
    annotate_time_points=time_points,
    annotate_time_points_color=time_point_colors)

# Annotate the plot
ax1.plot([0, .24], [0, .24], "k--");
ax1.annotate("degradation < formation", xy=(.1, .8),
             xycoords="axes fraction")
ax1.annotate("degradation > formation", xy=(.4, .3),
             xycoords="axes fraction")
ax1.annotate("", xy=(flux_sol["R5P_form"][0], flux_sol["RPI"][0]),
             xytext=(ppp.reactions.TKT1.steady_state_flux + ppp.reactions.DM_r5p_c.steady_state_flux,
                     ppp.reactions.RPI.steady_state_flux),
             textcoords="data",
             arrowprops=dict(arrowstyle="->",connectionstyle="arc3"));

plot_phase_portrait(
    flux_sol, x="flux_out", y="DM_r5p_c", ax=ax2,
    xlabel="DM_f6p_c + DM_g3p_c", ylabel="DM_r5p_c",
    title=("(a) Fluxes out of PPP", L_FONT),
    annotate_time_points=time_points,
    annotate_time_points_color=time_point_colors,
    annotate_time_points_legend="right outside")

# Annotate the plot
ax2.plot([0, 0.21], [.21 ,0], "k--")
ax2.annotate("", xy=(flux_sol["flux_out"][0], flux_sol["DM_r5p_c"][0]),
             xytext=(ppp.reactions.DM_f6p_c.steady_state_flux + ppp.reactions.DM_g3p_c.steady_state_flux,
                     ppp.reactions.DM_r5p_c.steady_state_flux),
             textcoords="data",
             arrowprops=dict(arrowstyle="->",connectionstyle="arc3"));
fig_11_5.tight_layout()
```

**Figure 11.5:** Dynamic response of the pentose pathway, increasing the rate of R5P production. (a) The fluxes that form and degrade R5P, \(v\_{DM\_{R5P}} + v\_{TKT1}\) vs. \(v\_{RPI}\). (b) The fluxes out of the pentose pathway, \(v\_{DM\_{F6P}} + v\_{DM\_{G3P}}\) vs. \(v\_{DM\_{R5P}}\).

##### The Combined Stoichiometric Matrix¶

###### Coupling to glycolysis: forming a unified reaction map¶

Since the inputs to and outputs from the pentose pathway are from glycolysis, the pentose pathway and glycolysis are readily interfaced to form a single reaction map (see Figure 11.6). The dashed arrows in the reaction map represent the return of F6P and GAP from the pentose pathway to glycolysis and do not represent actual reactions. The additional exchanges with the environment, over those in glycolysis alone, are \(\text{CO}\_2\) secretion and redox load on the NADPH pool, which are shown
in Figure 11.6 as a load on GSH (reduced glutathione). We will not consider R5P production here. It will appear in the next chapter as it is involved in the nucleotide salvage pathways.

**Figure 11.6:** Coupling glycolysis and the pentose pathway. The reaction schema, cofactor interactions, and environmental exchanges.

###### Joining the models¶

First, load both models. The pentose phosphate pathway is already loaded, so only the glycolysis model must be loaded:

```
[22]:
```

```
glycolysis = create_test_model("SB2_Glycolysis")
```

To merge two models, we use the `MassModel.merge` method to merge the two pathways and get rid of redundant reactions. Models take precedence from left to right in terms of parameters, initial conditions, and other model attributes.

```
[23]:
```

```
fullppp = glycolysis.merge(ppp, inplace=False)
fullppp.id = "Full_PPP_Model"
```

```
Ignoring reaction 'SK_h_c' since it already exists.
Ignoring reaction 'SK_h2o_c' since it already exists.
```

A few obsolete exchange reactions have to be removed.

```
[24]:
```

```
for boundary in fullppp.boundary:
    print(boundary)
```

```
DM_amp_c: amp_c -->
SK_pyr_c: pyr_c <=>
SK_lac__L_c: lac__L_c <=>
SK_glc__D_c:  <=> glc__D_c
SK_amp_c:  <=> amp_c
SK_h_c: h_c <=>
SK_h2o_c: h2o_c <=>
SK_g6p_c:  <=> g6p_c
DM_f6p_c: f6p_c -->
DM_g3p_c: g3p_c -->
DM_r5p_c: r5p_c -->
SK_co2_c: co2_c <=>
```

We can remove them using the `MassModel.remove_reactions` method.

```
[25]:
```

```
fullppp.remove_reactions([
    r for r in fullppp.boundary
    if r.id in ["SK_g6p_c", "DM_f6p_c", "DM_g3p_c", "DM_r5p_c"]])
fullppp.remove_boundary_conditions([
    "g6p_c", "f6p_c", "g3p_c", "r5p_c"])
```

The merged model contains 32 metabolites and 32 reactions:

```
[26]:
```

```
print(fullppp.S.shape)
```

```
(32, 32)
```

The merged model is not in a steady-state since the flux through PGI has not been corrected for the flux (0.21) that was diverted into the PPP. The PGI flux was 1.12 in the glycolytic model, and will need to be adjusted in the merged **fullppp** model.

```
[27]:
```

```
t0, tf = (0, 1e3)

fig_11_7, ax = plt.subplots(nrows=1, ncols=1, figsize=(6, 4))

for model in [glycolysis, fullppp]:
    sim = Simulation(model)
    flux_sol = sim.simulate(model, time=(t0, tf, tf*10 + 1))[1]
    plot_time_profile(
        flux_sol, observable=["PGI"], ax=ax,
        legend=model.id, plot_function="semilogx",
        xlabel="Time (hr)", ylabel="Flux (mM/hr)", ylim=(0.85, 1.15),
        title=("Flux through PGI", L_FONT))
fig_11_7.tight_layout()
```

###### Organization of the stoichiometric matrix¶

When the models are merged, metabolites and reactions are added at the end of their respecitive lists. We can perform a set of transformations to group the species and reactions into organized groups.

```
[28]:
```

```
# Define new order for metabolites
new_metabolite_order = ["glc__D_c", "g6p_c", "f6p_c", "fdp_c", "dhap_c",
                        "g3p_c", "_13dpg_c", "_3pg_c", "_2pg_c", "pep_c",
                        "pyr_c", "lac__L_c", "nad_c", "nadh_c", "amp_c",
                        "adp_c", "atp_c", "pi_c", "h_c", "h2o_c", "_6pgl_c", "_6pgc_c",
                        "ru5p__D_c", "xu5p__D_c", "r5p_c", "s7p_c", "e4p_c",
                        "nadp_c", "nadph_c", "gthrd_c", "gthox_c", "co2_c"]
if len(fullppp.metabolites) == len(new_metabolite_order):
    fullppp.metabolites = DictList(fullppp.metabolites.get_by_any(new_metabolite_order))
# Define new order for reactions
new_reaction_order = ["HEX1", "PGI", "PFK", "FBA", "TPI",
                      "GAPD", "PGK", "PGM", "ENO", "PYK",
                      "LDH_L", "DM_amp_c", "ADK1", "SK_pyr_c",
                      "SK_lac__L_c", "ATPM", "DM_nadh", "SK_glc__D_c",
                      "SK_amp_c", "SK_h_c", "SK_h2o_c",
                      "G6PDH2r", "PGL", "GND", "RPE", "RPI",
                      "TKT1", "TKT2", "TALA", "GTHOr", "GSHR", "SK_co2_c"]
if len(fullppp.reactions) == len(new_reaction_order):
    fullppp.reactions = DictList(fullppp.reactions.get_by_any(new_reaction_order))
```

The stoichiometric matrix for glycolysis (Table 10.8) can be appended with the reactions in the pentose pathway (Table 11.1). The resulting stoichiometric matrix is shown in Table 11.11. This matrix has dimensions of 32x32 and its rank is 28. The null is of dimension 4 (=32-28) and the left null space is of dimension 4 (=32-28). The matrix is elementally balanced.

We have used colors in Table 11.11 to illustrate the structure of the matrix. The two blocks of matrices on the diagonal are those for each pathway. The lower left block is filled with zero elements showing that the pentose pathway intermediates do not appear in glycolysis. Conversely, the upper right hand block shows that three of the glycolytic intermediates leave (GAP and F6P) and enter (G6P) the pentose pathway. Both glycolysis and the pentose pathway produce and/or consume protons and
water.

**Table 11.11:** The stoichiometric matrix for the coupled glycolytic and pentose pathways in Figure 11.6. The matrix is partitioned to show the glycolytic reactions (yellow) separate from the pentose phosphate pathway (light blue). The connectivities, \(\rho\_i\) (red), for a compound, and the participation number, \(pi\_j\) (cyan), for a reaction are shown. The second block in the table is the product \(\textbf{ES}\) (blue) to evaluate elemental balancing status of the reactions. All
exchange reactions have a participation number of unity and are thus not elementally balanced. The last block in the table has the four pathway vectors (purple) for the merged model. These vectors are graphically shown in Figure 11.10. Furthest to the right, we display the time invariant pools (green) that span the left null space.

```
[29]:
```

```
# Define labels
metabolite_ids = [m.id for m in fullppp.metabolites]
reaction_ids = [r.id for r in fullppp.reactions]

pi_str = r"$\pi_{j}$"
rho_str = r"$\rho_{i}$"
chopsnq = ['C', 'H', 'O', 'P', 'N', 'S', 'q', '[NAD]', '[NADP]']
time_inv_labels = [
    "$P_{\mathrm{tot}}$", "$N_{\mathrm{tot}}$",
    "$NP_{\mathrm{tot}}$", "$G_{\mathrm{tot}}$"]
path_labels = ["$p_1$","$p_2$", "$p_3$", "$p_4$"]

# Make table content from the stoichiometric matrix, elemental balancing of pathways
# participation number, and MinSpan pathways
S_matrix = fullppp.update_S(array_type="dense", dtype=np.int64, update_model=False)
ES_matrix = fullppp.get_elemental_charge_balancing(dtype=np.int64)
pi = np.count_nonzero(S_matrix, axis=0)
rho = np.count_nonzero(S_matrix, axis=1)
minspan_paths = np.array([
    [1, 1, 1, 1, 1, 2, 2, 2, 2, 2, 2, 0, 0, 0, 2, 2, 0, 1, 0, 2, 0, 0, 0, 0, 0, 0, 0, 0, 0, 0, 0, 0],
    [0, 0, 0, 0, 0, 0, 0, 0, 0, 0,-1, 0, 0, 1,-1, 0, 1, 0, 0, 2, 0, 0, 0, 0, 0, 0, 0, 0, 0, 0, 0, 0],
    [0, 0, 0, 0, 0, 0, 0, 0, 0, 0, 0, 1, 0, 0, 0, 0, 0, 0, 1, 0, 0, 0, 0, 0, 0, 0, 0, 0, 0, 0, 0, 0],
    [1,-2, 0, 0, 0, 1, 1, 1, 1, 1, 1, 0, 0, 0, 1, 1, 0, 1, 0,13,-3, 3, 3, 3, 2, 1, 1, 1, 1, 6, 6, 3]])

table_11_11 = np.vstack((S_matrix, pi, ES_matrix, minspan_paths))

# Determine number of blank entries needed to be added to pad the table,
# Add connectivity number and time invariants to table content
blanks = [""]*(len(table_11_11) - len(fullppp.metabolites))
rho = np.concatenate((rho, blanks))

lns = np.array([
    [0, 1, 1, 2, 1, 1, 2, 1, 1, 1, 0, 0, 0, 0, 0, 1, 2, 1, 0, 0, 0, 0, 0, 0, 0, 0, 0, 0, 0, 0, 0, 0],
    [0, 0, 0, 0, 0, 0, 0, 0, 0, 0, 0, 0, 1, 1, 0, 0, 0, 0, 0, 0, 0, 0, 0, 0, 0, 0, 0, 0, 0, 0, 0, 0],
    [0, 0, 0, 0, 0, 0, 0, 0, 0, 0, 0, 0, 0, 0, 0, 0, 0, 0, 0, 0, 0, 0, 0, 0, 0, 0, 0, 1, 1, 0, 0, 0],
    [0, 0, 0, 0, 0, 0, 0, 0, 0, 0, 0, 0, 0, 0, 0, 0, 0, 0, 0, 0, 0, 0, 0, 0, 0, 0, 0, 0, 0, 1, 2, 0],
])


time_inv = np.array([np.concatenate([row, blanks]) for row in lns])
table_11_11 = np.vstack([table_11_11.T, rho, time_inv]).T

colors = {"glycolysis": "#ffffe6",    # Yellow
          "ppp": "#e6faff",           # Light blue
          "chopsnq": "#99e6ff",       # Blue
          "pathways": "#b399ff",      # Purple
          "pi": "#99ffff",            # Cyan
          "rho": "#ff9999",           # Red
          "time_invs": "#ccff99",     # Green
          "blank": "#f2f2f2"}         # Grey
bg_color_str = "background-color: "
def highlight_table(df, model):
    df = df.copy()
    n_mets, n_rxns = (len(model.metabolites), len(model.reactions))
    # Highlight rows
    for row in df.index:
        other_key, condition = ("blank", lambda v, row: v != "")
        if row == pi_str:        # For participation
            main_key = "pi"
        elif row in chopsnq:     # For elemental balancing
            main_key = "chopsnq"
        elif row in path_labels: # For pathways
            main_key = "pathways"
        else:
            # Distinguish between reactions for model modules
            main_key, other_key = ("glycolysis", "ppp")
            condition = lambda v, row: row in glycolysis.metabolites
        df.loc[row, :] = [bg_color_str + colors[main_key] if condition(v, row)
                          else bg_color_str + colors[other_key]
                          for v in df.loc[row, :]]

    for col in df.columns:
        condition = lambda i, v, col: v != bg_color_str + colors["blank"]
        if col == rho_str:
            main_key = "rho"
        elif col in time_inv_labels:
            main_key = "time_invs"
        else:
            # Distinguish between metabolites for model modules
            main_key = "ppp"
            condition = lambda i, v, col: (col not in glycolysis.reactions and i < n_mets)
        df.loc[:, col] = [bg_color_str + colors[main_key] if condition(i, v, col)
                          else v for i, v in enumerate(df.loc[:, col])]
    return df

# Create index and column labels
index_labels = np.concatenate((metabolite_ids, [pi_str], chopsnq, path_labels))
column_labels = np.concatenate((reaction_ids, [rho_str], time_inv_labels))
# Create DataFrame
table_11_11 = pd.DataFrame(
    table_11_11, index=index_labels, columns=column_labels)
# Apply colors
table_11_11 = table_11_11.style.apply(
    highlight_table,  model=fullppp, axis=None)
table_11_11
```

```
[29]:
```

|  | HEX1 | PGI | PFK | FBA | TPI | GAPD | PGK | PGM | ENO | PYK | LDH\_L | DM\_amp\_c | ADK1 | SK\_pyr\_c | SK\_lac\_\_L\_c | ATPM | DM\_nadh | SK\_glc\_\_D\_c | SK\_amp\_c | SK\_h\_c | SK\_h2o\_c | G6PDH2r | PGL | GND | RPE | RPI | TKT1 | TKT2 | TALA | GTHOr | GSHR | SK\_co2\_c | $\rho\_{i}$ | $P\_{\mathrm{tot}}$ | $N\_{\mathrm{tot}}$ | $NP\_{\mathrm{tot}}$ | $G\_{\mathrm{tot}}$ |
| --- | --- | --- | --- | --- | --- | --- | --- | --- | --- | --- | --- | --- | --- | --- | --- | --- | --- | --- | --- | --- | --- | --- | --- | --- | --- | --- | --- | --- | --- | --- | --- | --- | --- | --- | --- | --- | --- |
| glc\_\_D\_c | -1 | 0 | 0 | 0 | 0 | 0 | 0 | 0 | 0 | 0 | 0 | 0 | 0 | 0 | 0 | 0 | 0 | 1 | 0 | 0 | 0 | 0 | 0 | 0 | 0 | 0 | 0 | 0 | 0 | 0 | 0 | 0 | 2 | 0 | 0 | 0 | 0 |
| g6p\_c | 1 | -1 | 0 | 0 | 0 | 0 | 0 | 0 | 0 | 0 | 0 | 0 | 0 | 0 | 0 | 0 | 0 | 0 | 0 | 0 | 0 | -1 | 0 | 0 | 0 | 0 | 0 | 0 | 0 | 0 | 0 | 0 | 3 | 1 | 0 | 0 | 0 |
| f6p\_c | 0 | 1 | -1 | 0 | 0 | 0 | 0 | 0 | 0 | 0 | 0 | 0 | 0 | 0 | 0 | 0 | 0 | 0 | 0 | 0 | 0 | 0 | 0 | 0 | 0 | 0 | 0 | 1 | 1 | 0 | 0 | 0 | 4 | 1 | 0 | 0 | 0 |
| fdp\_c | 0 | 0 | 1 | -1 | 0 | 0 | 0 | 0 | 0 | 0 | 0 | 0 | 0 | 0 | 0 | 0 | 0 | 0 | 0 | 0 | 0 | 0 | 0 | 0 | 0 | 0 | 0 | 0 | 0 | 0 | 0 | 0 | 2 | 2 | 0 | 0 | 0 |
| dhap\_c | 0 | 0 | 0 | 1 | -1 | 0 | 0 | 0 | 0 | 0 | 0 | 0 | 0 | 0 | 0 | 0 | 0 | 0 | 0 | 0 | 0 | 0 | 0 | 0 | 0 | 0 | 0 | 0 | 0 | 0 | 0 | 0 | 2 | 1 | 0 | 0 | 0 |
| g3p\_c | 0 | 0 | 0 | 1 | 1 | -1 | 0 | 0 | 0 | 0 | 0 | 0 | 0 | 0 | 0 | 0 | 0 | 0 | 0 | 0 | 0 | 0 | 0 | 0 | 0 | 0 | 1 | 1 | -1 | 0 | 0 | 0 | 6 | 1 | 0 | 0 | 0 |
| \_13dpg\_c | 0 | 0 | 0 | 0 | 0 | 1 | -1 | 0 | 0 | 0 | 0 | 0 | 0 | 0 | 0 | 0 | 0 | 0 | 0 | 0 | 0 | 0 | 0 | 0 | 0 | 0 | 0 | 0 | 0 | 0 | 0 | 0 | 2 | 2 | 0 | 0 | 0 |
| \_3pg\_c | 0 | 0 | 0 | 0 | 0 | 0 | 1 | -1 | 0 | 0 | 0 | 0 | 0 | 0 | 0 | 0 | 0 | 0 | 0 | 0 | 0 | 0 | 0 | 0 | 0 | 0 | 0 | 0 | 0 | 0 | 0 | 0 | 2 | 1 | 0 | 0 | 0 |
| \_2pg\_c | 0 | 0 | 0 | 0 | 0 | 0 | 0 | 1 | -1 | 0 | 0 | 0 | 0 | 0 | 0 | 0 | 0 | 0 | 0 | 0 | 0 | 0 | 0 | 0 | 0 | 0 | 0 | 0 | 0 | 0 | 0 | 0 | 2 | 1 | 0 | 0 | 0 |
| pep\_c | 0 | 0 | 0 | 0 | 0 | 0 | 0 | 0 | 1 | -1 | 0 | 0 | 0 | 0 | 0 | 0 | 0 | 0 | 0 | 0 | 0 | 0 | 0 | 0 | 0 | 0 | 0 | 0 | 0 | 0 | 0 | 0 | 2 | 1 | 0 | 0 | 0 |
| pyr\_c | 0 | 0 | 0 | 0 | 0 | 0 | 0 | 0 | 0 | 1 | -1 | 0 | 0 | -1 | 0 | 0 | 0 | 0 | 0 | 0 | 0 | 0 | 0 | 0 | 0 | 0 | 0 | 0 | 0 | 0 | 0 | 0 | 3 | 0 | 0 | 0 | 0 |
| lac\_\_L\_c | 0 | 0 | 0 | 0 | 0 | 0 | 0 | 0 | 0 | 0 | 1 | 0 | 0 | 0 | -1 | 0 | 0 | 0 | 0 | 0 | 0 | 0 | 0 | 0 | 0 | 0 | 0 | 0 | 0 | 0 | 0 | 0 | 2 | 0 | 0 | 0 | 0 |
| nad\_c | 0 | 0 | 0 | 0 | 0 | -1 | 0 | 0 | 0 | 0 | 1 | 0 | 0 | 0 | 0 | 0 | 1 | 0 | 0 | 0 | 0 | 0 | 0 | 0 | 0 | 0 | 0 | 0 | 0 | 0 | 0 | 0 | 3 | 0 | 1 | 0 | 0 |
| nadh\_c | 0 | 0 | 0 | 0 | 0 | 1 | 0 | 0 | 0 | 0 | -1 | 0 | 0 | 0 | 0 | 0 | -1 | 0 | 0 | 0 | 0 | 0 | 0 | 0 | 0 | 0 | 0 | 0 | 0 | 0 | 0 | 0 | 3 | 0 | 1 | 0 | 0 |
| amp\_c | 0 | 0 | 0 | 0 | 0 | 0 | 0 | 0 | 0 | 0 | 0 | -1 | 1 | 0 | 0 | 0 | 0 | 0 | 1 | 0 | 0 | 0 | 0 | 0 | 0 | 0 | 0 | 0 | 0 | 0 | 0 | 0 | 3 | 0 | 0 | 0 | 0 |
| adp\_c | 1 | 0 | 1 | 0 | 0 | 0 | -1 | 0 | 0 | -1 | 0 | 0 | -2 | 0 | 0 | 1 | 0 | 0 | 0 | 0 | 0 | 0 | 0 | 0 | 0 | 0 | 0 | 0 | 0 | 0 | 0 | 0 | 6 | 1 | 0 | 0 | 0 |
| atp\_c | -1 | 0 | -1 | 0 | 0 | 0 | 1 | 0 | 0 | 1 | 0 | 0 | 1 | 0 | 0 | -1 | 0 | 0 | 0 | 0 | 0 | 0 | 0 | 0 | 0 | 0 | 0 | 0 | 0 | 0 | 0 | 0 | 6 | 2 | 0 | 0 | 0 |
| pi\_c | 0 | 0 | 0 | 0 | 0 | -1 | 0 | 0 | 0 | 0 | 0 | 0 | 0 | 0 | 0 | 1 | 0 | 0 | 0 | 0 | 0 | 0 | 0 | 0 | 0 | 0 | 0 | 0 | 0 | 0 | 0 | 0 | 2 | 1 | 0 | 0 | 0 |
| h\_c | 1 | 0 | 1 | 0 | 0 | 1 | 0 | 0 | 0 | -1 | -1 | 0 | 0 | 0 | 0 | 1 | 1 | 0 | 0 | -1 | 0 | 1 | 1 | 0 | 0 | 0 | 0 | 0 | 0 | -1 | 2 | 0 | 12 | 0 | 0 | 0 | 0 |
| h2o\_c | 0 | 0 | 0 | 0 | 0 | 0 | 0 | 0 | 1 | 0 | 0 | 0 | 0 | 0 | 0 | -1 | 0 | 0 | 0 | 0 | -1 | 0 | -1 | 0 | 0 | 0 | 0 | 0 | 0 | 0 | 0 | 0 | 4 | 0 | 0 | 0 | 0 |
| \_6pgl\_c | 0 | 0 | 0 | 0 | 0 | 0 | 0 | 0 | 0 | 0 | 0 | 0 | 0 | 0 | 0 | 0 | 0 | 0 | 0 | 0 | 0 | 1 | -1 | 0 | 0 | 0 | 0 | 0 | 0 | 0 | 0 | 0 | 2 | 0 | 0 | 0 | 0 |
| \_6pgc\_c | 0 | 0 | 0 | 0 | 0 | 0 | 0 | 0 | 0 | 0 | 0 | 0 | 0 | 0 | 0 | 0 | 0 | 0 | 0 | 0 | 0 | 0 | 1 | -1 | 0 | 0 | 0 | 0 | 0 | 0 | 0 | 0 | 2 | 0 | 0 | 0 | 0 |
| ru5p\_\_D\_c | 0 | 0 | 0 | 0 | 0 | 0 | 0 | 0 | 0 | 0 | 0 | 0 | 0 | 0 | 0 | 0 | 0 | 0 | 0 | 0 | 0 | 0 | 0 | 1 | -1 | -1 | 0 | 0 | 0 | 0 | 0 | 0 | 3 | 0 | 0 | 0 | 0 |
| xu5p\_\_D\_c | 0 | 0 | 0 | 0 | 0 | 0 | 0 | 0 | 0 | 0 | 0 | 0 | 0 | 0 | 0 | 0 | 0 | 0 | 0 | 0 | 0 | 0 | 0 | 0 | 1 | 0 | -1 | -1 | 0 | 0 | 0 | 0 | 3 | 0 | 0 | 0 | 0 |
| r5p\_c | 0 | 0 | 0 | 0 | 0 | 0 | 0 | 0 | 0 | 0 | 0 | 0 | 0 | 0 | 0 | 0 | 0 | 0 | 0 | 0 | 0 | 0 | 0 | 0 | 0 | 1 | -1 | 0 | 0 | 0 | 0 | 0 | 2 | 0 | 0 | 0 | 0 |
| s7p\_c | 0 | 0 | 0 | 0 | 0 | 0 | 0 | 0 | 0 | 0 | 0 | 0 | 0 | 0 | 0 | 0 | 0 | 0 | 0 | 0 | 0 | 0 | 0 | 0 | 0 | 0 | 1 | 0 | -1 | 0 | 0 | 0 | 2 | 0 | 0 | 0 | 0 |
| e4p\_c | 0 | 0 | 0 | 0 | 0 | 0 | 0 | 0 | 0 | 0 | 0 | 0 | 0 | 0 | 0 | 0 | 0 | 0 | 0 | 0 | 0 | 0 | 0 | 0 | 0 | 0 | 0 | -1 | 1 | 0 | 0 | 0 | 2 | 0 | 0 | 0 | 0 |
| nadp\_c | 0 | 0 | 0 | 0 | 0 | 0 | 0 | 0 | 0 | 0 | 0 | 0 | 0 | 0 | 0 | 0 | 0 | 0 | 0 | 0 | 0 | -1 | 0 | -1 | 0 | 0 | 0 | 0 | 0 | 1 | 0 | 0 | 3 | 0 | 0 | 1 | 0 |
| nadph\_c | 0 | 0 | 0 | 0 | 0 | 0 | 0 | 0 | 0 | 0 | 0 | 0 | 0 | 0 | 0 | 0 | 0 | 0 | 0 | 0 | 0 | 1 | 0 | 1 | 0 | 0 | 0 | 0 | 0 | -1 | 0 | 0 | 3 | 0 | 0 | 1 | 0 |
| gthrd\_c | 0 | 0 | 0 | 0 | 0 | 0 | 0 | 0 | 0 | 0 | 0 | 0 | 0 | 0 | 0 | 0 | 0 | 0 | 0 | 0 | 0 | 0 | 0 | 0 | 0 | 0 | 0 | 0 | 0 | 2 | -2 | 0 | 2 | 0 | 0 | 0 | 1 |
| gthox\_c | 0 | 0 | 0 | 0 | 0 | 0 | 0 | 0 | 0 | 0 | 0 | 0 | 0 | 0 | 0 | 0 | 0 | 0 | 0 | 0 | 0 | 0 | 0 | 0 | 0 | 0 | 0 | 0 | 0 | -1 | 1 | 0 | 2 | 0 | 0 | 0 | 2 |
| co2\_c | 0 | 0 | 0 | 0 | 0 | 0 | 0 | 0 | 0 | 0 | 0 | 0 | 0 | 0 | 0 | 0 | 0 | 0 | 0 | 0 | 0 | 0 | 0 | 1 | 0 | 0 | 0 | 0 | 0 | 0 | 0 | -1 | 2 | 0 | 0 | 0 | 0 |
| $\pi\_{j}$ | 5 | 2 | 5 | 3 | 2 | 6 | 4 | 2 | 3 | 5 | 5 | 1 | 3 | 1 | 1 | 5 | 3 | 1 | 1 | 1 | 1 | 5 | 4 | 5 | 2 | 2 | 4 | 4 | 4 | 5 | 3 | 1 |  |  |  |  |  |
| C | 0 | 0 | 0 | 0 | 0 | 0 | 0 | 0 | 0 | 0 | 0 | -10 | 0 | -3 | -3 | 0 | 0 | 6 | 10 | 0 | 0 | 0 | 0 | 0 | 0 | 0 | 0 | 0 | 0 | 0 | 0 | -1 |  |  |  |  |  |
| H | 0 | 0 | 0 | 0 | 0 | 0 | 0 | 0 | 0 | 0 | 0 | -12 | 0 | -3 | -5 | 0 | 0 | 12 | 12 | -1 | -2 | 0 | 0 | 0 | 0 | 0 | 0 | 0 | 0 | 0 | 0 | 0 |  |  |  |  |  |
| O | 0 | 0 | 0 | 0 | 0 | 0 | 0 | 0 | 0 | 0 | 0 | -7 | 0 | -3 | -3 | 0 | 0 | 6 | 7 | 0 | -1 | 0 | 0 | 0 | 0 | 0 | 0 | 0 | 0 | 0 | 0 | -2 |  |  |  |  |  |
| P | 0 | 0 | 0 | 0 | 0 | 0 | 0 | 0 | 0 | 0 | 0 | -1 | 0 | 0 | 0 | 0 | 0 | 0 | 1 | 0 | 0 | 0 | 0 | 0 | 0 | 0 | 0 | 0 | 0 | 0 | 0 | 0 |  |  |  |  |  |
| N | 0 | 0 | 0 | 0 | 0 | 0 | 0 | 0 | 0 | 0 | 0 | -5 | 0 | 0 | 0 | 0 | 0 | 0 | 5 | 0 | 0 | 0 | 0 | 0 | 0 | 0 | 0 | 0 | 0 | 0 | 0 | 0 |  |  |  |  |  |
| S | 0 | 0 | 0 | 0 | 0 | 0 | 0 | 0 | 0 | 0 | 0 | 0 | 0 | 0 | 0 | 0 | 0 | 0 | 0 | 0 | 0 | 0 | 0 | 0 | 0 | 0 | 0 | 0 | 0 | 0 | 0 | 0 |  |  |  |  |  |
| q | 0 | 0 | 0 | 0 | 0 | 0 | 0 | 0 | 0 | 0 | 0 | 2 | 0 | 1 | 1 | 0 | 2 | 0 | -2 | -1 | 0 | 0 | 0 | 0 | 0 | 0 | 0 | 0 | 0 | 0 | 2 | 0 |  |  |  |  |  |
| [NAD] | 0 | 0 | 0 | 0 | 0 | 0 | 0 | 0 | 0 | 0 | 0 | 0 | 0 | 0 | 0 | 0 | 0 | 0 | 0 | 0 | 0 | 0 | 0 | 0 | 0 | 0 | 0 | 0 | 0 | 0 | 0 | 0 |  |  |  |  |  |
| [NADP] | 0 | 0 | 0 | 0 | 0 | 0 | 0 | 0 | 0 | 0 | 0 | 0 | 0 | 0 | 0 | 0 | 0 | 0 | 0 | 0 | 0 | 0 | 0 | 0 | 0 | 0 | 0 | 0 | 0 | 0 | 0 | 0 |  |  |  |  |  |
| $p\_1$ | 1 | 1 | 1 | 1 | 1 | 2 | 2 | 2 | 2 | 2 | 2 | 0 | 0 | 0 | 2 | 2 | 0 | 1 | 0 | 2 | 0 | 0 | 0 | 0 | 0 | 0 | 0 | 0 | 0 | 0 | 0 | 0 |  |  |  |  |  |
| $p\_2$ | 0 | 0 | 0 | 0 | 0 | 0 | 0 | 0 | 0 | 0 | -1 | 0 | 0 | 1 | -1 | 0 | 1 | 0 | 0 | 2 | 0 | 0 | 0 | 0 | 0 | 0 | 0 | 0 | 0 | 0 | 0 | 0 |  |  |  |  |  |
| $p\_3$ | 0 | 0 | 0 | 0 | 0 | 0 | 0 | 0 | 0 | 0 | 0 | 1 | 0 | 0 | 0 | 0 | 0 | 0 | 1 | 0 | 0 | 0 | 0 | 0 | 0 | 0 | 0 | 0 | 0 | 0 | 0 | 0 |  |  |  |  |  |
| $p\_4$ | 1 | -2 | 0 | 0 | 0 | 1 | 1 | 1 | 1 | 1 | 1 | 0 | 0 | 0 | 1 | 1 | 0 | 1 | 0 | 13 | -3 | 3 | 3 | 3 | 2 | 1 | 1 | 1 | 1 | 6 | 6 | 3 |  |  |  |  |  |

###### The pathway structure:¶

The null space is spanned by four vectors (shown towards the bottom of Table 11.11) that have pathway interpretations (Figure 11.8). The first three pathways are the same as that shown in Figure 10.2 for glycolysis alone, representing:They represent: \(\textbf{p}\_1\): redox neutral glycolysis (glucose to lactate), \(\textbf{p}\_2\): redox exchange with plasma (pyruvate to lactate), and \(\textbf{p}\_3\): AMP in and out.

**Figure 11.8:** Pathway maps for the four pathway vectors for the system formed by coupling glycolysis and the pentose pathway. They span all possible steady state solutions.

A new pathway, \(\textbf{p}\_4\), is a cycle through the pentose pathway, where the two F6P output from the pentose pathway flow back up glycolysis through PGI and enter the pentose pathway again, while the GAP output flows down glycolysis and leaves as lactate producing an ATP in lower glycolysis. The net result is the conversion of glucose to three \(\text{CO}\_2\) molecules and the production of six NADPH redox equivalents. It is a combination of \(\textbf{p}\_1\)(Table 11.5) for
the pentose pathway alone and the redox neutral use of glycolysis.

This new pathway balances the system fully and is a hybrid of the definitions of the classical glycolytic and pentose pathways. Note that the vectors that span the null space consider the entire network. Thus, as the scope of models increases the classical pathway definitions give way to network-based pathways that are mathematically defined. This definition is a departure from the historical and heuristic definitions of pathways. This feature is an important one in systems biology.

###### The time invariant pools:¶

There are four time invariant pools associated with the coupled glycolytic and pentose pathways (Table 11.7). The first are the same as for glycolysis alone: the pool of total NADH. Two new pools appear when the pentose pathway is coupled to glycolysis: the total amount of glutathione \(G\_{\mathrm{tot}}\) in the system)

\[\begin{equation} 2 \text{GSH} + \text{GSSG} = G\_{\mathrm{\mathrm{tot}}} \tag{11.2} \end{equation}\]

and the total amount of the NADPH carrier, \(NP\_{\mathrm{tot}}\),

\[\begin{equation} 2 \text{NADPH} + \text{NADP} = \text{NADPH}\_{\mathrm{tot}} = NP\_{\mathrm{tot}} \tag{11.3} \end{equation}\]

These time invariant pools are shown in the last four columns of Table 11.11.

##### Defining the Steady State¶

###### Computing the steady state flux map¶

The null space is four dimensional. Thus, if four independent fluxes are specified then the steady state flux map is uniquely defined. We therefore have to select fluxes for the four steady state pathway vectors in Table 11.11.

The glucose uptake rate is 1.12 mM/hr. The glutathione load is approximately 0.42 mM/hr. Thus we set the weight on \((\textbf{p}\_4)\) to be 0.42/6 = 0.07 and that of \(\textbf{p}\_1\) to be 1.12-0.07=1.05 mM/hr. As in Chapter 10, we set the NADH load to be 20% of the glucose uptake, thus \(\textbf{p}\_2\) has a load of \(0.2 \* 1.12 = 0.244.\) mM/hr. Finally the AMP input rate is the same as before at 0.014 mM/hr \(\textbf{p}\_3\). Thus the steady state flux vector is:

\[\begin{equation} \textbf{v}\_{\mathrm{stst}} = 1.05\textbf{p}\_1 + 0.224\textbf{p}\_1 + 0.014\textbf{p}\_3 + 0.07\textbf{p}\_4 \tag{11.4} \end{equation}\]

This equation is analogous to Eq. (10.3) except the incoming glucose flux is now distributed between the glucose pathway vector \((\textbf{p}\_1)\) and the pentose pathway vector \((\textbf{p}\_4)\). Summing up the pathway vectors in this ratio gives the steady state flux values, as shown in the last row of Table 11.11.

```
[30]:
```

```
# Set independent fluxes to determine steady state flux vector
independent_fluxes = {
    fullppp.reactions.SK_glc__D_c: 1.12,
    fullppp.reactions.DM_nadh: .2*1.12,
    fullppp.reactions.SK_amp_c: 0.014,
    fullppp.reactions.GTHOr: 0.42}

ssfluxes = fullppp.compute_steady_state_fluxes(
    minspan_paths,
    independent_fluxes,
    update_reactions=True)
table_11_12 = pd.DataFrame(list(ssfluxes.values()), index=reaction_ids,
                           columns=[r"$\textbf{v}_{\mathrm{stst}}$"]).T
```

**Table 11.12:** The steady state fluxes as a summation of the MinSpan pathway vectors.

```
[31]:
```

```
table_11_12
```

```
[31]:
```

|  | HEX1 | PGI | PFK | FBA | TPI | GAPD | PGK | PGM | ENO | PYK | LDH\_L | DM\_amp\_c | ADK1 | SK\_pyr\_c | SK\_lac\_\_L\_c | ATPM | DM\_nadh | SK\_glc\_\_D\_c | SK\_amp\_c | SK\_h\_c | SK\_h2o\_c | G6PDH2r | PGL | GND | RPE | RPI | TKT1 | TKT2 | TALA | GTHOr | GSHR | SK\_co2\_c |
| --- | --- | --- | --- | --- | --- | --- | --- | --- | --- | --- | --- | --- | --- | --- | --- | --- | --- | --- | --- | --- | --- | --- | --- | --- | --- | --- | --- | --- | --- | --- | --- | --- |
| $\textbf{v}\_{\mathrm{stst}}$ | 1.120 | 0.910 | 1.050 | 1.050 | 1.050 | 2.170 | 2.170 | 2.170 | 2.170 | 2.170 | 1.946 | 0.014 | 0.000 | 0.224 | 1.946 | 2.170 | 0.224 | 1.120 | 0.014 | 3.458 | -0.210 | 0.210 | 0.210 | 0.210 | 0.140 | 0.070 | 0.070 | 0.070 | 0.070 | 0.420 | 0.420 | 0.210 |

```
[32]:
```

```
fig_11_9, ax = plt.subplots(nrows=1, ncols=1, figsize=(10, 5))
# Define indicies for bar chart
indicies = np.arange(len(reaction_ids))+0.5
# Define colors to use
c = plt.cm.coolwarm(np.linspace(0, 1, len(reaction_ids)))
# Plot bar chart
ax.bar(indicies, list(ssfluxes.values()), width=0.8, color=c);
ax.set_xlim([0, len(reaction_ids)]);
# Set labels and adjust ticks
ax.set_xticks(indicies);
ax.set_xticklabels(reaction_ids, rotation="vertical");
ax.set_ylabel("Fluxes (mM/hr)", L_FONT);
ax.set_title("Steady State Fluxes", L_FONT);
ax.plot([0, len(reaction_ids)], [0, 0], "k--");
fig_11_9.tight_layout()
```

**Figure 11.9:** Bar chart of the steady-state fluxes.

Note that this procedure leads to the decomposition of the steady state into four interlinked pathway vectors. All homeostatic states are simultaneously carrying out many functions. This multiplexing can be broken down into underlying pathways and thus leads to a clear interpretation of the steady state solution.

**A numerical QC/QA:** Ensure \(\textbf{Sv}\_{\mathrm{stst}} = 0\)

```
[33]:
```

```
pd.DataFrame(
    fullppp.S.dot(np.array(list(ssfluxes.values()))),
    index=metabolite_ids,
    columns=[r"$\textbf{Sv}_{\mathrm{stst}}$"],
    dtype=np.int64).T
```

```
[33]:
```

|  | glc\_\_D\_c | g6p\_c | f6p\_c | fdp\_c | dhap\_c | g3p\_c | \_13dpg\_c | \_3pg\_c | \_2pg\_c | pep\_c | pyr\_c | lac\_\_L\_c | nad\_c | nadh\_c | amp\_c | adp\_c | atp\_c | pi\_c | h\_c | h2o\_c | \_6pgl\_c | \_6pgc\_c | ru5p\_\_D\_c | xu5p\_\_D\_c | r5p\_c | s7p\_c | e4p\_c | nadp\_c | nadph\_c | gthrd\_c | gthox\_c | co2\_c |
| --- | --- | --- | --- | --- | --- | --- | --- | --- | --- | --- | --- | --- | --- | --- | --- | --- | --- | --- | --- | --- | --- | --- | --- | --- | --- | --- | --- | --- | --- | --- | --- | --- |
| $\textbf{Sv}\_{\mathrm{stst}}$ | 0 | 0 | 0 | 0 | 0 | 0 | 0 | 0 | 0 | 0 | 0 | 0 | 0 | 0 | 0 | 0 | 0 | 0 | 0 | 0 | 0 | 0 | 0 | 0 | 0 | 0 | 0 | 0 | 0 | 0 | 0 | 0 |

###### Computing the rate constants¶

The kinetic constants can be computed from the steady state values of the concentrations using elementary mass action kinetics. The computation is based on Eq. (10.4) The results from this computation is summarized in Table 11.13. Note that most of the PERCs are large, leading to rapid responses, except for the glutathiones. \(v\_{GSHR}\) clearly sets the slowest time scale. This table has all the reaction properties that we need to complete the MASS model.

```
[34]:
```

```
percs = fullppp.calculate_PERCs(update_reactions=True)
```

**Table 11.13:** Combined glycolysis and pentose pathway enzymes and transport rates.

```
[35]:
```

```
# Get concentration values for substitution into sympy expressions
value_dict = {sym.Symbol(str(met)): ic
              for met, ic in fullppp.initial_conditions.items()}
value_dict.update({sym.Symbol(str(met)): bc
                   for met, bc in fullppp.boundary_conditions.items()})

table_11_13 = []
# Get symbols and values for table and substitution
for p_key in ["Keq", "kf"]:
    symbol_list, value_list = [], []
    for p_str, value in fullppp.parameters[p_key].items():
        symbol_list.append(r"$%s_{\text{%s}}$" % (p_key[0], p_str.split("_", 1)[-1]))
        value_list.append("{0:.3f}".format(value) if value != INF else r"$\infty$")
        value_dict.update({sym.Symbol(p_str): value})
    table_11_13.extend([symbol_list, value_list])

table_11_13.append(["{0:.6f}".format(float(ratio.subs(value_dict)))
                    for ratio in strip_time(fullppp.get_mass_action_ratios()).values()])
table_11_13.append(["{0:.6f}".format(float(ratio.subs(value_dict)))
                    for ratio in strip_time(fullppp.get_disequilibrium_ratios()).values()])
table_11_13 = pd.DataFrame(np.array(table_11_13).T, index=reaction_ids,
                           columns=[r"$K_{eq}$ Symbol", r"$K_{eq}$ Value", "PERC Symbol",
                                    "PERC Value", r"$\Gamma$", r"$\Gamma/K_{eq}$"])
table_11_13
```

```
[35]:
```

|  | $K\_{eq}$ Symbol | $K\_{eq}$ Value | PERC Symbol | PERC Value | $\Gamma$ | $\Gamma/K\_{eq}$ |
| --- | --- | --- | --- | --- | --- | --- |
| HEX1 | $K\_{\text{HEX1}}$ | 850.000 | $k\_{\text{HEX1}}$ | 0.700 | 0.008809 | 0.000010 |
| PGI | $K\_{\text{PGI}}$ | 0.410 | $k\_{\text{PGI}}$ | 2961.111 | 0.407407 | 0.993677 |
| PFK | $K\_{\text{PFK}}$ | 310.000 | $k\_{\text{PFK}}$ | 33.158 | 0.133649 | 0.000431 |
| FBA | $K\_{\text{FBA}}$ | 0.082 | $k\_{\text{FBA}}$ | 2657.407 | 0.079781 | 0.972937 |
| TPI | $K\_{\text{TPI}}$ | 0.057 | $k\_{\text{TPI}}$ | 32.208 | 0.045500 | 0.796249 |
| GAPD | $K\_{\text{GAPD}}$ | 0.018 | $k\_{\text{GAPD}}$ | 3271.226 | 0.006823 | 0.381183 |
| PGK | $K\_{\text{PGK}}$ | 1800.000 | $k\_{\text{PGK}}$ | 1233733.418 | 1755.073081 | 0.975041 |
| PGM | $K\_{\text{PGM}}$ | 0.147 | $k\_{\text{PGM}}$ | 4716.446 | 0.146184 | 0.994048 |
| ENO | $K\_{\text{ENO}}$ | 1.695 | $k\_{\text{ENO}}$ | 1708.624 | 1.504425 | 0.887608 |
| PYK | $K\_{\text{PYK}}$ | 363000.000 | $k\_{\text{PYK}}$ | 440.186 | 19.570304 | 0.000054 |
| LDH\_L | $K\_{\text{LDH\_L}}$ | 26300.000 | $k\_{\text{LDH\_L}}$ | 1073.943 | 44.132974 | 0.001678 |
| DM\_amp\_c | $K\_{\text{DM\_amp\_c}}$ | $\infty$ | $k\_{\text{DM\_amp\_c}}$ | 0.161 | 11.530288 | 0.000000 |
| ADK1 | $K\_{\text{ADK1}}$ | 1.650 | $k\_{\text{ADK1}}$ | 100000.000 | 1.650000 | 1.000000 |
| SK\_pyr\_c | $K\_{\text{SK\_pyr\_c}}$ | 1.000 | $k\_{\text{SK\_pyr\_c}}$ | 744.186 | 0.995008 | 0.995008 |
| SK\_lac\_\_L\_c | $K\_{\text{SK\_lac\_\_L\_c}}$ | 1.000 | $k\_{\text{SK\_lac\_\_L\_c}}$ | 5.406 | 0.735294 | 0.735294 |
| ATPM | $K\_{\text{ATPM}}$ | $\infty$ | $k\_{\text{ATPM}}$ | 1.356 | 0.453125 | 0.000000 |
| DM\_nadh | $K\_{\text{DM\_nadh}}$ | $\infty$ | $k\_{\text{DM\_nadh}}$ | 7.442 | 1.956811 | 0.000000 |
| SK\_glc\_\_D\_c | $K\_{\text{SK\_glc\_\_D\_c}}$ | $\infty$ | $k\_{\text{SK\_glc\_\_D\_c}}$ | 1.120 | 1.000000 | 0.000000 |
| SK\_amp\_c | $K\_{\text{SK\_amp\_c}}$ | $\infty$ | $k\_{\text{SK\_amp\_c}}$ | 0.014 | 0.086728 | 0.000000 |
| SK\_h\_c | $K\_{\text{SK\_h\_c}}$ | 1.000 | $k\_{\text{SK\_h\_c}}$ | 128645.833 | 0.701253 | 0.701253 |
| SK\_h2o\_c | $K\_{\text{SK\_h2o\_c}}$ | 1.000 | $k\_{\text{SK\_h2o\_c}}$ | 100000.000 | 1.000000 | 1.000000 |
| G6PDH2r | $K\_{\text{G6PDH2r}}$ | 1000.000 | $k\_{\text{G6PDH2r}}$ | 21864.589 | 11.875411 | 0.011875 |
| PGL | $K\_{\text{PGL}}$ | 1000.000 | $k\_{\text{PGL}}$ | 122.323 | 21.362698 | 0.021363 |
| GND | $K\_{\text{GND}}$ | 1000.000 | $k\_{\text{GND}}$ | 29287.807 | 43.340651 | 0.043341 |
| RPE | $K\_{\text{RPE}}$ | 3.000 | $k\_{\text{RPE}}$ | 16048.911 | 2.994699 | 0.998233 |
| RPI | $K\_{\text{RPI}}$ | 2.570 | $k\_{\text{RPI}}$ | 9645.957 | 2.566222 | 0.998530 |
| TKT1 | $K\_{\text{TKT1}}$ | 1.200 | $k\_{\text{TKT1}}$ | 1675.750 | 0.932371 | 0.776976 |
| TKT2 | $K\_{\text{TKT2}}$ | 10.300 | $k\_{\text{TKT2}}$ | 1146.859 | 1.921130 | 0.186517 |
| TALA | $K\_{\text{TALA}}$ | 1.050 | $k\_{\text{TALA}}$ | 886.847 | 0.575416 | 0.548015 |
| GTHOr | $K\_{\text{GTHOr}}$ | 100.000 | $k\_{\text{GTHOr}}$ | 53.330 | 0.259372 | 0.002594 |
| GSHR | $K\_{\text{GSHR}}$ | 2.000 | $k\_{\text{GSHR}}$ | 0.041 | 0.011719 | 0.005859 |
| SK\_co2\_c | $K\_{\text{SK\_co2\_c}}$ | 1.000 | $k\_{\text{SK\_co2\_c}}$ | 100000.000 | 1.000000 | 1.000000 |

##### Simulating an Increase in ATP Utilization¶

###### Validating the steady state¶

We can see from Figure 11.7 that the model is not in a steaty state. Therefore, as a QC/QA test, we simulate the model to ensure that the system is in a steady state after our PERC and steady state flux calculations.

```
[36]:
```

```
t0, tf = (0, 1e3)
sim_fullppp = Simulation(fullppp)
sim_fullppp.find_steady_state(fullppp, strategy="simulate",
                              update_values=True)
conc_sol_ss, flux_sol_ss = sim_fullppp.simulate(
    fullppp, time=(t0, tf, tf*10 + 1))
# Quickly render and display time profiles
conc_sol_ss.view_time_profile()
```

**Figure 11.10:** The merged model after determining the steady state conditions.

We can compare the differences in the initial state of each model before merging and after.

```
[37]:
```

```
fig_11_11, axes = plt.subplots(1, 2, figsize=(9, 4))
(ax1, ax2) = axes.flatten()

# Compare initial conditions
initial_conditions = {
    m.id: ic for m, ic in glycolysis.initial_conditions.items()
    if m.id in fullppp.metabolites}
initial_conditions.update({
    m.id: ic for m, ic in ppp.initial_conditions.items()
    if m.id in fullppp.metabolites})

plot_comparison(
    fullppp, pd.Series(initial_conditions), compare="concentrations",
    ax=ax1, plot_function="loglog",
    xlabel="Merged Model", ylabel="Independent Models",
    title=("(a) Steady State Concentrations of Species", L_FONT),
    color="blue", xy_line=True, xy_legend="best");

# Compare fluxes
fluxes = {
    r.id: flux for r, flux in glycolysis.steady_state_fluxes.items()
    if r.id in fullppp.reactions}
fluxes.update({
    r.id: flux for r, flux in ppp.steady_state_fluxes.items()
    if r.id in fullppp.reactions})

plot_comparison(
    fullppp, pd.Series(fluxes), compare="fluxes",
    ax=ax2, plot_function="plot",
    xlabel="Merged Model", ylabel="Independent Models",
    title=("(b) Steady State Fluxes of Reactions", L_FONT),
    color="red", xy_line=True, xy_legend="best");
fig_11_11.tight_layout()
```

**Figure 11.11:** Comparisons between the initial conditions of the merged model and the initial conditions of the independent glycolysis and pentose phosphate pathway networks for (a) the species and (b) the fluxes.

###### Response to an increased \(k\_{ATPM}\)¶

First, we must ensure that the system is originally at steady state. We perform the same simulation as in the last chapter by increasing the rate of ATP utilization.

```
[38]:
```

```
conc_sol, flux_sol = sim_fullppp.simulate(
    fullppp, time=(t0, tf, tf*10 + 1),
    perturbations={"kf_ATPM": "kf_ATPM * 1.5"})
```

```
[39]:
```

```
fig_11_12, axes = plt.subplots(nrows=2, ncols=1, figsize=(10, 8));
(ax1, ax2) = axes.flatten()

plot_time_profile(
    conc_sol, ax=ax1, legend="right outside",
    plot_function="loglog",
    xlabel="Time [hr]", ylabel="Concentration [mM]",
    title=("Concentration Profile", L_FONT));

plot_time_profile(
    flux_sol, ax=ax2, legend="right outside",
    plot_function="semilogx",
    xlabel="Time [hr]", ylabel="Flux [mM/hr]",
    title=("Flux Profile", L_FONT));
fig_11_12.tight_layout()
```

**Figure 11.12:** Simulating the combined system from the steady state with 50% increase in the rate of ATP utilization at t = 0.

The dynamic phase portraits are shown in Figure 11.13 for the same key fluxes as for glycolysis alone (Figure 10.15). The response is similar, except the dampened oscillations are more pronounced. The oxidative branch of the pentose pathway is not affected, as the GSH load is not changed (Figure 11.13e), while the damped oscillations do occur in the non-oxidative branch (Figure 11.13f). The dashed lines show the glycolytic phase portraits (before the pentose phosphate pathway was added).

```
[40]:
```

```
fig_11_13, axes = plt.subplots(nrows=2, ncols=3, figsize=(14, 8),
                               )

time_points = [t0, 1e-1, 1e0, 1e1, 1e2, tf]
time_point_colors = [
    mpl.colors.to_hex(c)
    for c in mpl.cm.Reds(np.linspace(0.3, 1, len(time_points)))]

pairings = [
    ("ATPM", "DM_amp_c"), ("ATPM", "SK_pyr_c"), ("DM_amp_c", "SK_pyr_c"),
    ("TKT2", "G6PDH2r"), ("GTHOr", "G6PDH2r"), ("GAPD", "DM_nadh")]
xlims = [
    (1.50, 3.40), (1.50, 3.40), (0.00, 0.10),
    (0.000, 0.100), (0.419, 0.421), (1.50, 2.60)]
ylims = [
    (0.00, 0.10), (0.14, 0.31), (0.14, 0.31),
    (0.204, 0.220), (0.204, 0.220), (0.15, 0.27)]

colors = ["grey", "black"]
styles = ["--", "-"]
for k, model in enumerate([glycolysis, fullppp]):
    sim = Simulation(model)
    flux_solution = sim.simulate(
        model, time=(t0, tf, tf*10 + 1),
        perturbations={"kf_ATPM": "kf_ATPM * 1.5"})[1]

    for i, ax in enumerate(axes.flatten()):
        if i >= 3 and k == 0:
            continue
        legend, time_point_legend = None, None
        if i == 2:
            legend = [model.id, "lower right outside"]
        if i == 5:
            time_point_legend = "upper right outside"
        x_i, y_i = pairings[i]
        plot_phase_portrait(
            flux_solution, x=x_i, y=y_i, ax=ax, legend=legend,
            xlabel=x_i, ylabel=y_i,
            xlim=xlims[i], ylim=ylims[i],
            title=("Phase Portrait of {0} vs. {1}".format(
                x_i, y_i), L_FONT),
            color=colors[k],
            linestyle=styles[k],
            annotate_time_points=time_points,
            annotate_time_points_color=time_point_colors,
            annotate_time_points_legend=time_point_legend)
fig_11_13.tight_layout()
```

**Figure 11.13:** Dynamic response of the integrated system of glycolysis and the pentose pathway to increasing the rate of ATP utilization. The dynamic response of key fluxes are shown. Detailed pair-wise phase portraits: a): \(v\_{ATPM}\ \textit{vs.}\ v\_{DM\_{AMP}}\). b): \(v\_{ATPM}\ \textit{vs.}\ v\_{SK\_{PYR}}\). c): \(v\_{DM\_{AMP}}\ \textit{vs.}\ v\_{SK\_{PYR}}\). d): \(v\_{TKT2}\ \textit{vs.}\ v\_{G6PDH2r}\). e): \(v\_{GTHOr}\ \textit{vs.}\ v\_{G6PDH2r}\). f):
\(v\_{GAPD}\ \textit{vs.}\ v\_{DM\_{NADH}}\). These phase portrait can be compared to the corresponding ones in Figure 10.15. The perturbation is reflected in the instantaneous move of the flux state from the initial steady state to an unsteady state, as indicated by the arrow placing the initial point at \(t=0^+\). The system then returns to its steady state at \(t \rightarrow \infty\). The dashed lines show the phase portraits of the original glycolysis model.

###### Relative deviations from the initial state¶

As shown in the figure below, AMP still has the highest perturbation. However, we can see that sedulose 7-phosphate also has a fairly high deviation from its initial condition. Note that there is slightly more variation in the concentration profiles at around \(t = 10\) than in glycolysis alone. We can see that the flux deviations also follow the same general trend as the glycolysis simulations, with slightly more variation.

```
[41]:
```

```
fig_11_14, axes = plt.subplots(nrows=2, ncols=1, figsize=(10, 8),
                               );
(ax1, ax2) = axes.flatten()

conc_deviation = {met.id: conc_sol[met.id]/ic
                  for met, ic in fullppp.initial_conditions.items()}
conc_deviation = MassSolution(
    "Deviation", solution_type="Conc",
    data_dict=conc_deviation,
    time=conc_sol.t, interpolate=False)

flux_deviation = {rxn.id: flux_sol[rxn.id]/ssflux
                  for rxn, ssflux in fullppp.steady_state_fluxes.items()
                  if ssflux != 0 and rxn.id != "ADK1"} # To avoid dividing by 0 for equilibrium fluxes.

flux_deviation = MassSolution(
    "Deviation", solution_type="Flux",
    data_dict=flux_deviation,
    time=flux_sol.t, interpolate=False)

plot_time_profile(
    conc_deviation, ax=ax1, legend="right outside",
    plot_function="semilogx",
    xlabel="Time [hr]", ylabel="Concentration [mM]",
    title=("Concentration Profile", L_FONT));

plot_time_profile(
    flux_deviation, ax=ax2, legend="right outside",
    plot_function="semilogx",
    xlabel="Time [hr]", ylabel="Flux [mM/hr]",
    title=("Flux Profile", L_FONT));
fig_11_14.tight_layout()
```

**Figure 11.14:** (a) Deviation from the steady state of the concentrations as a fraction of the steady state. (b) Deviation from the steady state of the fluxes as a fraction of the steady state.

**Table 11.13:** Numerical values for the concentrations (mM) and fluxes (mM/hr) at the beginning and end of the dynamic simulation. The concentration of water is arbitrarily set at 1.0. At \(t=0\) the \(v\_{ATPM}\) flux changes from 2.24 to 3.36, unbalancing the flux map. The flux map returns to its original state as time goes to infinity (here \(t=1000\)). Note that unlike the fluxes, the concentrations reach a different steady state.

```
[42]:
```

```
met_ids = metabolite_ids.copy()
init_concs = [round(ic[0], 3) for ic in conc_sol.values()]
final_concs = [round(bc[-1], 3) for bc in conc_sol.values()]
column_labels = ["Metabolite", "Conc. at t=0 [mM]", "Conc. at t=1000 [mM]"]

rxn_ids = reaction_ids.copy()
init_fluxes = [round(ic[0], 3) for ic in flux_sol.values()]
final_fluxes = [round(bc[-1], 3) for bc in flux_sol.values()]
column_labels += ["Reactions", "Flux at t=0 [mM/hr]", "Flux at t=1000 [mM/hr]"]

# Extend metabolite columns to match length of reaction columns for table
pad = [""]*(len(reaction_ids) - len(metabolite_ids))
# Make table
table_11_13 = np.array([metabolite_ids + pad, init_concs + pad, final_concs + pad,
                        reaction_ids, init_fluxes, final_fluxes])
table_11_13 = pd.DataFrame(table_11_13.T,
                           index=[i for i in range(1, len(reaction_ids) + 1)],
                           columns=column_labels)
def highlight_table(x):
    # ATPM is the 16th reaction according to Table 10.2
    return ['color: red' if x.name == 16 else '' for v in x]

table_11_13 = table_11_13.style.apply(highlight_table, subset=column_labels[3:], axis=1)
table_11_13
```

```
[42]:
```

|  | Metabolite | Conc. at t=0 [mM] | Conc. at t=1000 [mM] | Reactions | Flux at t=0 [mM/hr] | Flux at t=1000 [mM/hr] |
| --- | --- | --- | --- | --- | --- | --- |
| 1 | glc\_\_D\_c | 1.0 | 1.5 | HEX1 | 1.12 | 1.12 |
| 2 | g6p\_c | 0.049 | 0.073 | PGI | 0.91 | 0.91 |
| 3 | f6p\_c | 0.02 | 0.03 | PFK | 1.05 | 1.05 |
| 4 | fdp\_c | 0.015 | 0.008 | FBA | 1.05 | 1.05 |
| 5 | dhap\_c | 0.16 | 0.121 | TPI | 1.05 | 1.05 |
| 6 | g3p\_c | 0.007 | 0.005 | GAPD | 2.17 | 2.17 |
| 7 | \_13dpg\_c | 0.0 | 0.0 | PGK | 2.17 | 2.17 |
| 8 | \_3pg\_c | 0.077 | 0.093 | PGM | 2.17 | 2.17 |
| 9 | \_2pg\_c | 0.011 | 0.014 | ENO | 2.17 | 2.17 |
| 10 | pep\_c | 0.017 | 0.021 | PYK | 2.17 | 2.17 |
| 11 | pyr\_c | 0.06 | 0.06 | LDH\_L | 1.946 | 1.946 |
| 12 | lac\_\_L\_c | 1.36 | 1.36 | DM\_amp\_c | 0.014 | 0.014 |
| 13 | nad\_c | 0.059 | 0.059 | ADK1 | -0.0 | -0.0 |
| 14 | nadh\_c | 0.03 | 0.03 | SK\_pyr\_c | 0.224 | 0.224 |
| 15 | amp\_c | 0.087 | 0.087 | SK\_lac\_\_L\_c | 1.946 | 1.946 |
| 16 | adp\_c | 0.29 | 0.237 | ATPM | 3.255 | 2.17 |
| 17 | atp\_c | 1.6 | 1.067 | DM\_nadh | 0.224 | 0.224 |
| 18 | pi\_c | 2.5 | 3.58 | SK\_glc\_\_D\_c | 1.12 | 1.12 |
| 19 | h\_c | 0.0 | 0.0 | SK\_amp\_c | 0.014 | 0.014 |
| 20 | h2o\_c | 1.0 | 1.0 | SK\_h\_c | 3.458 | 3.458 |
| 21 | \_6pgl\_c | 0.002 | 0.002 | SK\_h2o\_c | -0.21 | -0.21 |
| 22 | \_6pgc\_c | 0.037 | 0.056 | G6PDH2r | 0.21 | 0.21 |
| 23 | ru5p\_\_D\_c | 0.005 | 0.005 | PGL | 0.21 | 0.21 |
| 24 | xu5p\_\_D\_c | 0.015 | 0.016 | GND | 0.21 | 0.21 |
| 25 | r5p\_c | 0.013 | 0.014 | RPE | 0.14 | 0.14 |
| 26 | s7p\_c | 0.024 | 0.042 | RPI | 0.07 | 0.07 |
| 27 | e4p\_c | 0.005 | 0.005 | TKT1 | 0.07 | 0.07 |
| 28 | nadp\_c | 0.0 | 0.0 | TKT2 | 0.07 | 0.07 |
| 29 | nadph\_c | 0.066 | 0.066 | TALA | 0.07 | 0.07 |
| 30 | gthrd\_c | 3.2 | 3.2 | GTHOr | 0.42 | 0.42 |
| 31 | gthox\_c | 0.12 | 0.12 | GSHR | 0.42 | 0.42 |
| 32 | co2\_c | 1.0 | 1.0 | SK\_co2\_c | 0.21 | 0.21 |

As before, we will examine the node balances to gain a better understanding of the system’s response.

###### The proton node for \(k\_{ATPM}\) perturbation¶

The proton node now has 8 production reactions and 4 utilization reactions. The fluxes in and out of the node can be graphed, and so can the proton concentration (the inventory in the node).

```
[43]:
```

```
fig_11_15 = plt.figure(figsize=(15, 6))
gs = fig_11_15.add_gridspec(nrows=3, ncols=2, width_ratios=[1.5, 1])

ax1 = fig_11_15.add_subplot(gs[0, 0])
ax2 = fig_11_15.add_subplot(gs[1, 0])
ax3 = fig_11_15.add_subplot(gs[2, 0])
ax4 = fig_11_15.add_subplot(gs[:, 1])

plot_time_profile(
    conc_sol, observable="h_c", ax=ax1,
    legend="right outside", plot_function="semilogx",
    xlim=(1e-6, tf), ylim=(8.5e-5, 1e-4*1.025),
    xlabel="Time [hr]", ylabel="Concentrations [mM]",
    title=("(a) Proton Concentration", L_FONT));

fluxes_in = ["HEX1", "PFK", "GAPD", "ATPM", "DM_nadh",
             "G6PDH2r", "PGL", "GSHR"]
plot_time_profile(
    flux_sol, observable=fluxes_in, ax=ax2,
    legend="right outside", plot_function="semilogx",
    xlim=(1e-6, tf),  ylim=(0, 4),
    xlabel="Time [hr]", ylabel="Fluxes [mM/hr]",
    title=("(b) Fluxes in", L_FONT));

fluxes_out = ["PYK", "LDH_L","SK_h_c", "GTHOr"]
plot_time_profile(
    flux_sol, observable=fluxes_out, ax=ax3,
    legend="right outside", plot_function="semilogx",
    xlim=(1e-6, tf),  ylim=(1.2, 5),
    xlabel="Time [hr]", ylabel="Fluxes [mM/hr]",
    title=("(c) Fluxes out", L_FONT));

for flux_id, variables in zip(["Net_Flux_In", "Net_Flux_Out"],
                              [fluxes_in, fluxes_out]):
    flux_sol.make_aggregate_solution(
        flux_id, equation=" + ".join(variables), variables=variables)

time_points = [t0, 1e-1, 1e0, 1e1, 1e2, tf]
time_point_colors = [
    mpl.colors.to_hex(c)
    for c in mpl.cm.Reds(np.linspace(0.3, 1, len(time_points)))]

plot_phase_portrait(
    flux_sol, x="Net_Flux_In", y="Net_Flux_Out", ax=ax4,
    xlim=(5.5, 9.5), ylim=(5.5, 9.5),
    xlabel="Fluxes in [mm/Hr]", ylabel="Fluxes out [mm/Hr]",
    title=("(d) Phase Portrait of Fluxes", L_FONT),
    annotate_time_points=time_points,
    annotate_time_points_color=time_point_colors,
    annotate_time_points_legend="best");
fig_11_15.tight_layout()
```

**Figure 11.15:** The time profiles of the (a) proton concentration, (b) the fluxes that make protons, (c) the fluxes that use protons and (d) the phase portrait of the net flux in and net flux out (darker red colors indicate slower time scales) for an increase in ATP utilization.

###### The NADPH node for \(k\_{ATPM}\) perturbation¶

Here, we examine the NADPH node. It has a connectivity of 3: two inputs and one output.

```
[44]:
```

```
fig_11_16 = plt.figure(figsize=(15, 6))
gs = fig_11_16.add_gridspec(nrows=3, ncols=2, width_ratios=[1.5, 1])

ax1 = fig_11_16.add_subplot(gs[0, 0])
ax2 = fig_11_16.add_subplot(gs[1, 0])
ax3 = fig_11_16.add_subplot(gs[2, 0])
ax4 = fig_11_16.add_subplot(gs[:, 1])

plot_time_profile(
    conc_sol, observable="nadph_c", ax=ax1,
    legend="right outside", plot_function="semilogx",
    xlim=(1e-6, tf), ylim=(0.0657, 0.066),
    xlabel="Time [hr]", ylabel="Concentrations [mM]",
    title=("(a) Proton Concentration", L_FONT));

fluxes_in = ["G6PDH2r", "GND"]
plot_time_profile(
    flux_sol, observable=fluxes_in, ax=ax2,
    legend="right outside", plot_function="semilogx",
    xlim=(1e-6, tf),  ylim=(0.203, 0.218),
    xlabel="Time [hr]", ylabel="Fluxes [mM/hr]",
    title=("(b) Fluxes in", L_FONT));

fluxes_out = ["GTHOr"]
plot_time_profile(
    flux_sol, observable=fluxes_out, ax=ax3,
    legend="right outside", plot_function="semilogx",
    xlim=(1e-6, tf), ylim=(0.4199, 0.4202),
    xlabel="Time [hr]", ylabel="Fluxes [mM/hr]",
    title=("(c) Fluxes out", L_FONT));

for flux_id, variables in zip(["Net_Flux_In", "Net_Flux_Out"],
                              [fluxes_in, fluxes_out]):
    flux_sol.make_aggregate_solution(
        flux_id, equation=" + ".join(variables), variables=variables)

time_points = [t0, 1e-1, 1e0, 1e1, 1e2, tf]
time_point_colors = [
    mpl.colors.to_hex(c)
    for c in mpl.cm.Reds(np.linspace(0.3, 1, len(time_points)))]

plot_phase_portrait(
    flux_sol, x="Net_Flux_In", y="Net_Flux_Out", ax=ax4,
    xlim=(0.4199, 0.4202), ylim=(0.4199, 0.4202),
    xlabel="Fluxes in [mm/Hr]", ylabel="Fluxes out [mm/Hr]",
    title=("(d) Phase Portrait of Fluxes", L_FONT),
    annotate_time_points=time_points,
    annotate_time_points_color=time_point_colors,
    annotate_time_points_legend="best");
fig_11_16.tight_layout()
```

**Figure 11.16:** The time profiles of the (a) NADPH concentration, (b) the fluxes that make NADPH, (c) the fluxes that use NADPH and (d) the phase portrait of the net flux in and net flux out (darker red colors indicate slower time scales) for an increase in ATP utilization.

###### Key Fluxes and all pairwise phase portraits¶

Figure 11.17 and Figure 11.18 are set up to allow the reader to examine all pairwise phase portraits After browsing through many of them, you will find that they resemble each other, showing that the variables move in a highly coordinated manner. We can study the relationship between many variables at once using multi-variate statistics.

```
[45]:
```

```
fig_11_17, ax = plt.subplots(nrows=1, ncols=1, figsize=(20, 22))
plot_tiled_phase_portraits(
    conc_sol, ax=ax, annotate_time_points_legend="lower outside");
fig_11_17.tight_layout()
```

**Figure 11.17:** Phase portraits of all the combined glycolytic and pentose phosphate pathway species.

```
[46]:
```

```
fig_11_18, ax = plt.subplots(nrows=1, ncols=1, figsize=(20, 22))
plot_tiled_phase_portraits(
    flux_sol, ax=ax, annotate_time_points_legend="lower outside");
fig_11_18.tight_layout()
```

**Figure 11.18:** Phase portraits of all the combined glycolytic and pentose phosphate pathway fluxes.

###### Response to an increased rate of GSH utilization¶

The main function of the pentose pathway is to generate redox potential in the form of GSH. We are thus interested in the increased rate of GSH use. We simulate the doubling of the rate of GSH use (see Figure 11.19). The response is characteristic of a highly buffered system. The size of \(G\_{\mathrm{tot}}\) is 3.44 mM \((=3.2+2\*0.12)\), which is a high concentration for this coupled pathway system.

```
[47]:
```

```
conc_sol, flux_sol = sim_fullppp.simulate(
    fullppp, time=(t0, tf, tf*10 + 1),
    perturbations={"kf_GSHR": "kf_GSHR * 2"})
```

The glutathione oxidase (the load) and the reductase reach a steady state within an hour at a higher flux level (Figure 11.19a), while the fluxes through the oxidative and non-oxidative branches of the pentose pathway increase, leading to increased \(\text{CO}\_2\) production. The loss of \(\text{CO}\_2\) leads to a lower flux through the upper and lower branches of glycolysis (Figure 11.19b). Thus the glycolytic intermediates drop slightly in concentration and the pentose pathway
intermediates increase, as indicated by the levels of GAP and Ru5P (Figure 11.19c).

```
[48]:
```

```
fig_11_19 = plt.figure(figsize=(15, 6))
gs = fig_11_19.add_gridspec(nrows=3, ncols=2, width_ratios=[1.5, 1])

ax1 = fig_11_19.add_subplot(gs[0, 0])
ax2 = fig_11_19.add_subplot(gs[1, 0])
ax3 = fig_11_19.add_subplot(gs[2, 0])

plot_time_profile(
    flux_sol, observable=["G6PDH2r", "TKT2", "GTHOr", "GSHR"],
    ax=ax1, legend="right outside", plot_function="semilogx",
    xlim=(1e-6, tf), ylim=(0, 1),
    xlabel="Time [hr]", ylabel="Fluxes [mM/hr]",
    title=("(a)", L_FONT));

plot_time_profile(
    flux_sol, observable=["PGI", "PYK", "SK_co2_c"],
    ax=ax2, legend="right outside", plot_function="semilogx",
    xlim=(1e-6, tf), ylim=(0, 1.4),
    xlabel="Time [hr]", ylabel="Fluxes [mM/hr]",
    title=("(b) ", L_FONT));

plot_time_profile(
    conc_sol, observable=["g3p_c", "ru5p__D_c"],
    ax=ax3, legend="right outside", plot_function="semilogx",
    xlim=(1e-6, tf), ylim=(0, 0.01),
    xlabel="Time [hr]", ylabel="Concentrations [mM]",
    title=("(c)", L_FONT));
fig_11_19.tight_layout()
```

**Figure 11.19:** Dynamic response of the integrated system of glycolysis and the pentose pathway, doubling the rate of GSH utilization. (a) The dynamic response of the fluxes in the oxidative \((v\_{G6PDH2r})\) and the non-oxidative \((v\_{TKT2})\) branches of the pentose pathway as well as GSH reductase and oxidase (the load). (b) The dynamic response of the upper \((v\_{PGI})\) and lower \((v\_{PYK})\) glycolytic fluxes, as well as \(\text{CO}\_2\) production by the pentose
pathway. (c) The dynamic response of sample intermediates in glycolysis, GAP, and the pentose pathway, Ru5P\*

As before, we will examine the node balances to gain a better understanding of the system’s response.

###### The proton node for GSH utilization perturbation¶

```
[49]:
```

```
fig_11_20 = plt.figure(figsize=(15, 6))
gs = fig_11_20.add_gridspec(nrows=3, ncols=2, width_ratios=[1.5, 1])

ax1 = fig_11_20.add_subplot(gs[0, 0])
ax2 = fig_11_20.add_subplot(gs[1, 0])
ax3 = fig_11_20.add_subplot(gs[2, 0])
ax4 = fig_11_20.add_subplot(gs[:, 1])

plot_time_profile(
    conc_sol, observable="h_c", ax=ax1,
    legend="right outside", plot_function="semilogx",
    xlim=(1e-6, tf), ylim=(8.5e-5, 1e-4*1.025),
    xlabel="Time [hr]", ylabel="Concentrations [mM]",
    title=("(a) Proton Concentration", L_FONT));

fluxes_in = ["HEX1", "PFK", "GAPD", "ATPM", "DM_nadh",
             "G6PDH2r", "PGL", "GSHR"]
plot_time_profile(
    flux_sol, observable=fluxes_in, ax=ax2,
    legend="right outside", plot_function="semilogx",
    xlim=(1e-6, tf),  ylim=(0, 4),
    xlabel="Time [hr]", ylabel="Fluxes [mM/hr]",
    title=("(b) Fluxes in", L_FONT));

fluxes_out = ["PYK", "LDH_L","SK_h_c", "GTHOr"]
plot_time_profile(
    flux_sol, observable=fluxes_out, ax=ax3,
    legend="right outside", plot_function="semilogx",
    xlim=(1e-6, tf),  ylim=(1.2, 5),
    xlabel="Time [hr]", ylabel="Fluxes [mM/hr]",
    title=("(c) Fluxes out", L_FONT));

for flux_id, variables in zip(["Net_Flux_In", "Net_Flux_Out"],
                              [fluxes_in, fluxes_out]):
    flux_sol.make_aggregate_solution(
        flux_id, equation=" + ".join(variables), variables=variables)

time_points = [t0, 1e-1, 1e0, 1e1, 1e2, tf]
time_point_colors = [
    mpl.colors.to_hex(c)
    for c in mpl.cm.Reds(np.linspace(0.3, 1, len(time_points)))]

plot_phase_portrait(
    flux_sol, x="Net_Flux_In", y="Net_Flux_Out", ax=ax4,
    xlim=(7.8, 8.2), ylim=(7.75, 9),
    xlabel="Fluxes in [mm/Hr]", ylabel="Fluxes out [mm/Hr]",
    title=("(d) Phase Portrait of Fluxes", L_FONT),
    annotate_time_points=time_points,
    annotate_time_points_color=time_point_colors,
    annotate_time_points_legend="best");
fig_11_20.tight_layout()
```

**Figure 11.20:** The time profiles of the (a) proton concentration, (b) the fluxes that make protons, (c) the fluxes that use protons and (d) the phase portrait of the net flux in and net flux out (darker red colors indicate slower time scales) for an increase in GSH utilization.

###### The NADPH node for GSH utilization perturbation¶

```
[50]:
```

```
fig_11_21 = plt.figure(figsize=(15, 6))
gs = fig_11_21.add_gridspec(nrows=3, ncols=2, width_ratios=[1.5, 1])

ax1 = fig_11_21.add_subplot(gs[0, 0])
ax2 = fig_11_21.add_subplot(gs[1, 0])
ax3 = fig_11_21.add_subplot(gs[2, 0])
ax4 = fig_11_21.add_subplot(gs[:, 1])

plot_time_profile(
    conc_sol, observable="nadph_c", ax=ax1,
    legend="right outside", plot_function="semilogx",
    xlim=(1e-6, tf), ylim=(0.0656, 0.0659),
    xlabel="Time [hr]", ylabel="Concentrations [mM]",
    title=("(a) Proton Concentration", L_FONT));

fluxes_in = ["G6PDH2r", "GND"]
plot_time_profile(
    flux_sol, observable=fluxes_in, ax=ax2,
    legend="right outside", plot_function="semilogx",
    xlim=(1e-6, tf), ylim=(0.15, 0.4),
    xlabel="Time [hr]", ylabel="Fluxes [mM/hr]",
    title=("(b) Fluxes in", L_FONT));

fluxes_out = ["GTHOr"]
plot_time_profile(
    flux_sol, observable=fluxes_out, ax=ax3,
    legend="right outside", plot_function="semilogx",
    xlim=(1e-6, tf), ylim=(0.3, 0.8),
    xlabel="Time [hr]", ylabel="Fluxes [mM/hr]",
    title=("(c) Fluxes out", L_FONT));

for flux_id, variables in zip(["Net_Flux_In", "Net_Flux_Out"],
                              [fluxes_in, fluxes_out]):
    flux_sol.make_aggregate_solution(
        flux_id, equation=" + ".join(variables), variables=variables)

time_points = [t0, 1e-1, 1e0, 1e1, 1e2, tf]
time_point_colors = [
    mpl.colors.to_hex(c)
    for c in mpl.cm.Reds(np.linspace(0.3, 1, len(time_points)))]

plot_phase_portrait(
    flux_sol, x="Net_Flux_In", y="Net_Flux_Out", ax=ax4,
    xlim=(0.3, 0.8), ylim=(0.3, 0.8),
    xlabel="Fluxes in [mm/Hr]", ylabel="Fluxes out [mm/Hr]",
    title=("(d) Phase Portrait of Fluxes", L_FONT),
    annotate_time_points=time_points,
    annotate_time_points_color=time_point_colors,
    annotate_time_points_legend="best");
fig_11_21.tight_layout()
```

**Figure 11.21:** The time profiles of the (a) NADPH concentration, (b) the fluxes that make NADPH, (c) the fluxes that use NADPH and (d) the phase portrait of the net flux in and net flux out (darker red colors indicate slower time scales) for an increase in GSH utilization.

##### Pooling: Towards Systems Biology¶

The combined glycolytic and pentose pathways have couplings to four cofactors: ATP, NADH and NADPH (and thus GSH), and \(P\_i\). The formation of the corresponding pools is clear as they show up in the left null space. The last four rows of Table 11.14 show these time-invariant, or ‘hard’ pools. We can form the time dependent pools based on analysis of the biochemical features of these coupled pathways.

```
[51]:
```

```
# Define the individual pools. The placement of each coefficient
# corresponds to the order of the metabolites in the model as seen
# in table_11_1.  _p indicates the positive superscript ^+ and
# _n indicates the negative superscript (^-).
def make_pooling_matrix(include_all=False):
    GP_p = np.array([2, 3, 3, 4, 2, 2, 2, 1, 1, 1, 0, 0, 0, 0, 0, 0, 0, 0, 0, 0, 3, 3, 8/3, 8/3, 8/3, 10/3, 7/3, 0, 0, 0, 0, 0])
    GP_n = np.array([0, 0, 0, 0, 0, 0, 0, 0, 0, 0, 1, 1, 0, 0, 0, 0, 0, 0, 0, 0, 0, 0, 0, 0, 0, 0, 0, 0, 0, 0, 0, 0])
    GR_p = np.array([2, 2, 2, 2, 1, 1, 0, 0, 0, 0, 0, 1, 0, 0, 0, 0, 0, 0, 0, 0, 2, 2, 5/3, 5/3, 5/3, 7/3, 4/3, 0, 0, 0, 0, 0])
    GR_n = np.array([0, 0, 0, 0, 0, 0, 1, 1, 1, 1, 1, 0, 0, 0, 0, 0, 0, 0, 0, 0, 0, 0, 0,0, 0, 0, 0, 0, 0, 0, 0, 0])
    GPR_p = np.array([6, 6, 6, 0, 0, 0, 0, 0, 0, 0, 0, 0, 0, 0, 0, 0, 0, 0, 0, 0, 5, 5, 4, 4, 4, 8, 2, 0, 0, 0, 0, 0])
    GPR_n = np.array([0, 0, 0, 1, 1, 1, 1, 1, 1, 1, 1, 1, 0, 0, 0, 0, 0, 0, 0, 0, 0, 0, 0, 0, 0, 0, 0, 0, 0, 0, 0, 0])
    P_p = np.array([0, 1, 1, 2, 1, 1, 1, 0, 0, 0, 0, 0, 0, 0, 0, 1, 2, 0, 0, 0, 0, 0, 0, 0, 0, 0, 0, 0, 0, 0, 0, 0])
    P_n = np.array([0, 0, 0, 0, 0, 0, 1, 1, 1, 1, 0, 0, 0, 0, 0, 0, 0, 0, 0, 0, 0, 0, 0,0, 0, 0, 0, 0, 0, 0, 0, 0])
    AP_p = np.array([0, 0, 0, 0, 0, 0, 0, 0, 0, 0, 0, 0, 0, 0, 0, 1, 2, 0, 0, 0, 0, 0, 0, 0, 0, 0, 0, 0, 0, 0, 0, 0])
    AP_n = np.array([0, 0, 0, 0, 0, 0, 0, 0, 0, 0, 0, 0, 0, 0, 2, 1, 0, 0, 0, 0, 0, 0, 0, 0, 0, 0, 0, 0, 0, 0, 0, 0])
    N_p =  np.array([0, 0, 0, 0, 0, 0, 0, 0, 0, 0, 0, 0, 0, 1, 0, 0, 0, 0, 0, 0, 0, 0, 0, 0, 0, 0, 0, 0, 0, 0, 0, 0])
    N_n =  np.array([0, 0, 0, 0, 0, 0, 0, 0, 0, 0, 0, 0, 1, 0, 0, 0, 0, 0, 0, 0, 0, 0, 0, 0, 0, 0, 0, 0, 0, 0, 0, 0])
    NP_p = np.array([0, 0, 0, 0, 0, 0, 0, 0, 0, 0, 0, 0, 0, 0, 0, 0, 0, 0, 0, 0, 0, 0, 0, 0, 0, 0, 0, 0, 1, 0, 0, 0])
    NP_n = np.array([0, 0, 0, 0, 0, 0, 0, 0, 0, 0, 0, 0, 0, 0, 0, 0, 0, 0, 0, 0, 0, 0, 0, 0, 0, 0, 0, 1, 0, 0, 0, 0])
    G_p = np.array([0, 0, 0, 0, 0, 0, 0, 0, 0, 0, 0, 0, 0, 0, 0, 0, 0, 0, 0, 0, 0, 0, 0, 0, 0, 0, 0, 0, 0, 1, 0, 0])
    G_n = np.array([0, 0, 0, 0, 0, 0, 0, 0, 0, 0, 0, 0, 0, 0, 0, 0, 0, 0, 0, 0, 0, 0, 0, 0, 0, 0, 0, 0, 0, 0, 2, 0])
    P_tot = P_p + P_n
    N_tot = N_p + N_n
    G_tot = G_p + G_n
    NP_tot = NP_p + NP_n

    # Define pooling matrix and pool labels
    if include_all:
        pool_labels = ["$GP^+$", "$GP^-$", "$GR^+$", "$GR^-$", "$GRP^+$", "$GRP^-$",
                       "$P^+$", "$P^-$","$AP^+$", "$AP^-$", "$N^+$", "$N^-$", "$NP^+$", "$NP^-$",
                       "$G^+$", "$G^-$","$P_{\mathrm{tot}}$", "$N_{\mathrm{tot}}$", "$G_{\mathrm{tot}}$",
                       "$NP_{\mathrm{tot}}$"]
        pooling_matrix = np.vstack([GP_p, GP_n, GR_p, GR_n, GPR_p, GPR_n,  P_p, P_n, AP_p, AP_n,
                                    N_p, N_n, NP_p, NP_n, G_p, G_n, P_tot, N_tot, G_tot, NP_tot])
    else:
        pool_labels = ["$GP^+$", "$GP^-$", "$GR^+$", "$GR^-$", "$GRP^+$", "$GRP^-$",
                       "$P^+$", "$P^-$","$AP^+$", "$AP^-$", "$N^+$", "$NP^+$", "$G^+$",
                       "$P_{\mathrm{tot}}$", "$N_{\mathrm{tot}}$", "$G_{\mathrm{tot}}$", "$NP_{\mathrm{tot}}$"]
        pooling_matrix = np.vstack([GP_p, GP_n, GR_p, GR_n, GPR_p, GPR_n,  P_p, P_n, AP_p, AP_n,
                                    N_p,  NP_p, G_p, P_tot, N_tot, G_tot, NP_tot])
    # Round to 3 decimals
    pooling_matrix = np.array([[round(col, 3) for col in row] for row in pooling_matrix])
    return pooling_matrix, pool_labels
include_all = False
```

**Table 11.14:** Definition of functional pools for merged glycolysis and the pentose pathway. The left null space vectors for the stoichiometric matrix for coupled glycolytic and the pentose pathways in Table 11.11 are the last four rows in the table. The four time-invariant pools represent: 1) total phosphate, 2) total NADH, 3) total glutathione, and 4) total NADPH.

```
[52]:
```

```
# Make table content from the pooling matrix, connectivity and
# participation numbers, and pool labels
pooling_matrix, pool_labels = make_pooling_matrix(include_all)
pool_numbers = np.array([[i for i in range(1, len(pool_labels) + 1)] + [""]])
pi = np.count_nonzero(pooling_matrix, axis=0)
rho = np.array([np.concatenate((np.count_nonzero(pooling_matrix, axis=1), [""]))])
table_11_14 = np.vstack((pooling_matrix, pi))
table_11_14 = np.hstack((pool_numbers.T, table_11_14, rho.T))

index_labels = pool_labels + [pi_str]
column_labels = ["Pool #"] + metabolite_ids + [rho_str]
table_11_14 = pd.DataFrame(table_11_14, index=index_labels,
                           columns=column_labels)

# Highlight table
n_colors = int(np.ceil(len(pool_labels)/2))
color_list = [mpl.colors.to_hex(c) for c in mpl.cm.Set3(np.linspace(0, 1, n_colors))]
colors = dict(zip(["GP", "GR", "GRP", "P", "AP", "N", "NP", "G", "tot"], color_list))
colors.update({pi_str: "#99ffff",    # Cyan
               rho_str: "#ff9999",   # Red
               "blank": "#f2f2f2"}) # Grey
bg_color_str = "background-color: "
def highlight_table(df):
    df = df.copy()
    for row in df.index:
        if row == pi_str:
            main_key = pi_str
        elif row[1:-3] in colors:
            main_key = row[1:-3]
        else:
            main_key = "tot"
        df.loc[row, :] = [bg_color_str + colors[main_key] if v != ""
                          else bg_color_str + colors["blank"]
                          for v in df.loc[row, :]]
    for col in df.columns:
        if col == rho_str:
            df.loc[:, col] = [bg_color_str + colors[rho_str]
                              if v != bg_color_str + colors["blank"]
                              else v for v in df.loc[:, col]]
    return df

table_11_14 = table_11_14.style.apply(highlight_table, axis=None)
table_11_14
```

```
[52]:
```

|  | Pool # | glc\_\_D\_c | g6p\_c | f6p\_c | fdp\_c | dhap\_c | g3p\_c | \_13dpg\_c | \_3pg\_c | \_2pg\_c | pep\_c | pyr\_c | lac\_\_L\_c | nad\_c | nadh\_c | amp\_c | adp\_c | atp\_c | pi\_c | h\_c | h2o\_c | \_6pgl\_c | \_6pgc\_c | ru5p\_\_D\_c | xu5p\_\_D\_c | r5p\_c | s7p\_c | e4p\_c | nadp\_c | nadph\_c | gthrd\_c | gthox\_c | co2\_c | $\rho\_{i}$ |
| --- | --- | --- | --- | --- | --- | --- | --- | --- | --- | --- | --- | --- | --- | --- | --- | --- | --- | --- | --- | --- | --- | --- | --- | --- | --- | --- | --- | --- | --- | --- | --- | --- | --- | --- |
| $GP^+$ | 1 | 2.0 | 3.0 | 3.0 | 4.0 | 2.0 | 2.0 | 2.0 | 1.0 | 1.0 | 1.0 | 0.0 | 0.0 | 0.0 | 0.0 | 0.0 | 0.0 | 0.0 | 0.0 | 0.0 | 0.0 | 3.0 | 3.0 | 2.667 | 2.667 | 2.667 | 3.333 | 2.333 | 0.0 | 0.0 | 0.0 | 0.0 | 0.0 | 17 |
| $GP^-$ | 2 | 0.0 | 0.0 | 0.0 | 0.0 | 0.0 | 0.0 | 0.0 | 0.0 | 0.0 | 0.0 | 1.0 | 1.0 | 0.0 | 0.0 | 0.0 | 0.0 | 0.0 | 0.0 | 0.0 | 0.0 | 0.0 | 0.0 | 0.0 | 0.0 | 0.0 | 0.0 | 0.0 | 0.0 | 0.0 | 0.0 | 0.0 | 0.0 | 2 |
| $GR^+$ | 3 | 2.0 | 2.0 | 2.0 | 2.0 | 1.0 | 1.0 | 0.0 | 0.0 | 0.0 | 0.0 | 0.0 | 1.0 | 0.0 | 0.0 | 0.0 | 0.0 | 0.0 | 0.0 | 0.0 | 0.0 | 2.0 | 2.0 | 1.667 | 1.667 | 1.667 | 2.333 | 1.333 | 0.0 | 0.0 | 0.0 | 0.0 | 0.0 | 14 |
| $GR^-$ | 4 | 0.0 | 0.0 | 0.0 | 0.0 | 0.0 | 0.0 | 1.0 | 1.0 | 1.0 | 1.0 | 1.0 | 0.0 | 0.0 | 0.0 | 0.0 | 0.0 | 0.0 | 0.0 | 0.0 | 0.0 | 0.0 | 0.0 | 0.0 | 0.0 | 0.0 | 0.0 | 0.0 | 0.0 | 0.0 | 0.0 | 0.0 | 0.0 | 5 |
| $GRP^+$ | 5 | 6.0 | 6.0 | 6.0 | 0.0 | 0.0 | 0.0 | 0.0 | 0.0 | 0.0 | 0.0 | 0.0 | 0.0 | 0.0 | 0.0 | 0.0 | 0.0 | 0.0 | 0.0 | 0.0 | 0.0 | 5.0 | 5.0 | 4.0 | 4.0 | 4.0 | 8.0 | 2.0 | 0.0 | 0.0 | 0.0 | 0.0 | 0.0 | 10 |
| $GRP^-$ | 6 | 0.0 | 0.0 | 0.0 | 1.0 | 1.0 | 1.0 | 1.0 | 1.0 | 1.0 | 1.0 | 1.0 | 1.0 | 0.0 | 0.0 | 0.0 | 0.0 | 0.0 | 0.0 | 0.0 | 0.0 | 0.0 | 0.0 | 0.0 | 0.0 | 0.0 | 0.0 | 0.0 | 0.0 | 0.0 | 0.0 | 0.0 | 0.0 | 9 |
| $P^+$ | 7 | 0.0 | 1.0 | 1.0 | 2.0 | 1.0 | 1.0 | 1.0 | 0.0 | 0.0 | 0.0 | 0.0 | 0.0 | 0.0 | 0.0 | 0.0 | 1.0 | 2.0 | 0.0 | 0.0 | 0.0 | 0.0 | 0.0 | 0.0 | 0.0 | 0.0 | 0.0 | 0.0 | 0.0 | 0.0 | 0.0 | 0.0 | 0.0 | 8 |
| $P^-$ | 8 | 0.0 | 0.0 | 0.0 | 0.0 | 0.0 | 0.0 | 1.0 | 1.0 | 1.0 | 1.0 | 0.0 | 0.0 | 0.0 | 0.0 | 0.0 | 0.0 | 0.0 | 0.0 | 0.0 | 0.0 | 0.0 | 0.0 | 0.0 | 0.0 | 0.0 | 0.0 | 0.0 | 0.0 | 0.0 | 0.0 | 0.0 | 0.0 | 4 |
| $AP^+$ | 9 | 0.0 | 0.0 | 0.0 | 0.0 | 0.0 | 0.0 | 0.0 | 0.0 | 0.0 | 0.0 | 0.0 | 0.0 | 0.0 | 0.0 | 0.0 | 1.0 | 2.0 | 0.0 | 0.0 | 0.0 | 0.0 | 0.0 | 0.0 | 0.0 | 0.0 | 0.0 | 0.0 | 0.0 | 0.0 | 0.0 | 0.0 | 0.0 | 2 |
| $AP^-$ | 10 | 0.0 | 0.0 | 0.0 | 0.0 | 0.0 | 0.0 | 0.0 | 0.0 | 0.0 | 0.0 | 0.0 | 0.0 | 0.0 | 0.0 | 2.0 | 1.0 | 0.0 | 0.0 | 0.0 | 0.0 | 0.0 | 0.0 | 0.0 | 0.0 | 0.0 | 0.0 | 0.0 | 0.0 | 0.0 | 0.0 | 0.0 | 0.0 | 2 |
| $N^+$ | 11 | 0.0 | 0.0 | 0.0 | 0.0 | 0.0 | 0.0 | 0.0 | 0.0 | 0.0 | 0.0 | 0.0 | 0.0 | 0.0 | 1.0 | 0.0 | 0.0 | 0.0 | 0.0 | 0.0 | 0.0 | 0.0 | 0.0 | 0.0 | 0.0 | 0.0 | 0.0 | 0.0 | 0.0 | 0.0 | 0.0 | 0.0 | 0.0 | 1 |
| $NP^+$ | 12 | 0.0 | 0.0 | 0.0 | 0.0 | 0.0 | 0.0 | 0.0 | 0.0 | 0.0 | 0.0 | 0.0 | 0.0 | 0.0 | 0.0 | 0.0 | 0.0 | 0.0 | 0.0 | 0.0 | 0.0 | 0.0 | 0.0 | 0.0 | 0.0 | 0.0 | 0.0 | 0.0 | 0.0 | 1.0 | 0.0 | 0.0 | 0.0 | 1 |
| $G^+$ | 13 | 0.0 | 0.0 | 0.0 | 0.0 | 0.0 | 0.0 | 0.0 | 0.0 | 0.0 | 0.0 | 0.0 | 0.0 | 0.0 | 0.0 | 0.0 | 0.0 | 0.0 | 0.0 | 0.0 | 0.0 | 0.0 | 0.0 | 0.0 | 0.0 | 0.0 | 0.0 | 0.0 | 0.0 | 0.0 | 1.0 | 0.0 | 0.0 | 1 |
| $P\_{\mathrm{tot}}$ | 14 | 0.0 | 1.0 | 1.0 | 2.0 | 1.0 | 1.0 | 2.0 | 1.0 | 1.0 | 1.0 | 0.0 | 0.0 | 0.0 | 0.0 | 0.0 | 1.0 | 2.0 | 0.0 | 0.0 | 0.0 | 0.0 | 0.0 | 0.0 | 0.0 | 0.0 | 0.0 | 0.0 | 0.0 | 0.0 | 0.0 | 0.0 | 0.0 | 11 |
| $N\_{\mathrm{tot}}$ | 15 | 0.0 | 0.0 | 0.0 | 0.0 | 0.0 | 0.0 | 0.0 | 0.0 | 0.0 | 0.0 | 0.0 | 0.0 | 1.0 | 1.0 | 0.0 | 0.0 | 0.0 | 0.0 | 0.0 | 0.0 | 0.0 | 0.0 | 0.0 | 0.0 | 0.0 | 0.0 | 0.0 | 0.0 | 0.0 | 0.0 | 0.0 | 0.0 | 2 |
| $G\_{\mathrm{tot}}$ | 16 | 0.0 | 0.0 | 0.0 | 0.0 | 0.0 | 0.0 | 0.0 | 0.0 | 0.0 | 0.0 | 0.0 | 0.0 | 0.0 | 0.0 | 0.0 | 0.0 | 0.0 | 0.0 | 0.0 | 0.0 | 0.0 | 0.0 | 0.0 | 0.0 | 0.0 | 0.0 | 0.0 | 0.0 | 0.0 | 1.0 | 2.0 | 0.0 | 2 |
| $NP\_{\mathrm{tot}}$ | 17 | 0.0 | 0.0 | 0.0 | 0.0 | 0.0 | 0.0 | 0.0 | 0.0 | 0.0 | 0.0 | 0.0 | 0.0 | 0.0 | 0.0 | 0.0 | 0.0 | 0.0 | 0.0 | 0.0 | 0.0 | 0.0 | 0.0 | 0.0 | 0.0 | 0.0 | 0.0 | 0.0 | 1.0 | 1.0 | 0.0 | 0.0 | 0.0 | 2 |
| $\pi\_{j}$ |  | 3.0 | 5.0 | 5.0 | 5.0 | 5.0 | 5.0 | 6.0 | 5.0 | 5.0 | 5.0 | 3.0 | 3.0 | 1.0 | 2.0 | 1.0 | 4.0 | 3.0 | 0.0 | 0.0 | 0.0 | 3.0 | 3.0 | 3.0 | 3.0 | 3.0 | 3.0 | 3.0 | 1.0 | 2.0 | 2.0 | 1.0 | 0.0 |  |

###### Using shadow prices to determine the cofactor value of intermediates¶

The value of the intermediates needed to generate a charged form of a cofactor is hard to obtain by mere inspection of the coupled pathways. As the scope of models grows, this cofactor value determination becomes increasingly difficult.

The cofactor values of the intermediates can be obtained using established methods of systems biology. These values can be generated from the shadow prices generated by linear optimization (see *Systems Biology: Properties of Reconstructed Networks, Chapter 15*). Here, the stoichiometric matrix and the inputs and outputs are used to formulate a linear optimization problem:

\[\begin{equation} \text{max (cofactor production) subject to}\ \textbf{Sv}=0;\ \text{and the input of glucose} =1 \tag{11.5} \end{equation}\]

The maximum amount of a charged version of the cofactor produced (e.g., ATP) is computed from a single glucose molecule entering the system. The shadow prices for the intermediates give the sensitivity of the objective function (i.e., ATP production) with respect to the infinitesimal addition of that intermediate. The shadow prices can thus give the value of the intermediates for the production of a particular cofactor.

An alternative way to evaluate the cofactor generation value of the intermediates is:

\[\begin{split}\begin{equation} \text{max (cofactor production) subject to}\ \textbf{Sv}=0;\\ \text{and the input of the intermediate of interest} =1 \end{equation} \tag{11.6}\end{split}\]

and by adding an exchange rate for the intermediate of interest. This alternative approach would need many optimization computations, whereas all the shadow prices can be obtained from a single optimization computation.

###### Defining a pooling matrix¶

The results from shadow price computations are used in forming the pooling matrix (Table 11.15). The values of the glycolytic intermediates are the same as before, but we can now add the ATP and NADH value of the pentose pathway intermediates. In addition, we can compute the value of the intermediates with respect to generating redox potential in the form of NADPH (or GSH). The high energy bond value of the intermediates in the combined pathways are shown in the first two lines
\((GP^+, GP^-)\). The next two lines do the same for the NADH value \((GR^+, GR^-)\), and then the following two lines give new pools for the NADPH value of the intermediates \((GPR^+, GPR^-)\). The following four pools give the state of the phosphates \((P^+, P^-)\) as well as the energy value of the adenosine phosphates \((AP^+, AP^-)\). These are the same as for glycolysis alone. The next three lines give the redox carriers \((N^+, NP^+, G^+)\). The bottom four pools
are the time invariant pools that are in the left null space of \(\textbf{S}\).

###### The reactions that move the pools¶

The fluxes that move the pools can be determined by computing \(\textbf{PS}\). The results are shown in Table 11.15. These pools can be added to the pool-flux map of glycolysis (Figure 10.20).

**Table 11.15:** The fluxes that flow in and out of the pools defined in Table 11.14.

```
[53]:
```

```
# Make table content from the pooling matrix, connectivity and
# participation numbers, pool sizes, and time constants
pooling_matrix, pool_labels = make_pooling_matrix(include_all)
PS = pooling_matrix.dot(fullppp.S)
PS = np.array([[round(col, 3) for col in row] for row in PS])

pi = np.count_nonzero(PS, axis=0)
rho = np.count_nonzero(PS, axis=1)
ic_values = [fullppp.initial_conditions[met]
             for met in fullppp.metabolites.get_by_any(metabolite_ids)]
pool_sizes = [round(np.sum([coeff*ic for coeff, ic in zip(row, ic_values)]), 5)
              for row in pooling_matrix]
flux_values = [fullppp.steady_state_fluxes[rxn]
               for rxn in fullppp.reactions.get_by_any(reaction_ids)]
fluxes_in = [round(np.sum([coeff*flux for coeff, flux in zip(row, flux_values)
                           if coeff >=0]), 5) for row in PS]
taus = [round(size/flux_in, 5) if flux_in != 0
        else r"$\infty$" for size, flux_in in zip(pool_sizes, fluxes_in)]
table_11_15 = np.hstack((pool_numbers.T,
                         np.vstack((PS, pi)),
                         np.array([np.append(col, "")
                                   for col in np.array([rho, pool_sizes, fluxes_in, taus])]).T))
index_labels = pool_labels + [pi_str]
column_labels = np.concatenate((["Pool #"], reaction_ids,
                                [rho_str, "Size (mM)", "Net steady state flux (mM/hr)", r"$\tau$(h)"]))
table_11_15 = pd.DataFrame(table_11_15, index=index_labels,
                           columns=column_labels)
# Highlight table
colors.update({"Size (mM)": "#e6faff",                     # L. Blue
               "Net steady state flux (mM/hr)": "#f9ecf2", # L. Pink
               r"$\tau$(h)": "#e6ffe6"})                   # L. Green
bg_color_str = "background-color: "
def highlight_table(df):
    df = df.copy()
    for row in df.index:
        if row == pi_str:
            main_key = pi_str
        elif row[1:-3] in colors:
            main_key = row[1:-3]
        else:
            main_key = "tot"
        df.loc[row, :] = [bg_color_str + colors[main_key] if v != ""
                          else bg_color_str + colors["blank"]
                          for v in df.loc[row, :]]
    for col in df.columns:
        if col in colors:
            df.loc[:, col] = [bg_color_str + colors[col]
                              if v != bg_color_str + colors["blank"]
                              else v for v in df.loc[:, col]]
    return df

table_11_15 = table_11_15.style.apply(highlight_table, axis=None)
table_11_15
```

```
[53]:
```

|  | Pool # | HEX1 | PGI | PFK | FBA | TPI | GAPD | PGK | PGM | ENO | PYK | LDH\_L | DM\_amp\_c | ADK1 | SK\_pyr\_c | SK\_lac\_\_L\_c | ATPM | DM\_nadh | SK\_glc\_\_D\_c | SK\_amp\_c | SK\_h\_c | SK\_h2o\_c | G6PDH2r | PGL | GND | RPE | RPI | TKT1 | TKT2 | TALA | GTHOr | GSHR | SK\_co2\_c | $\rho\_{i}$ | Size (mM) | Net steady state flux (mM/hr) | $\tau$(h) |
| --- | --- | --- | --- | --- | --- | --- | --- | --- | --- | --- | --- | --- | --- | --- | --- | --- | --- | --- | --- | --- | --- | --- | --- | --- | --- | --- | --- | --- | --- | --- | --- | --- | --- | --- | --- | --- | --- |
| $GP^+$ | 1 | 1.0 | 0.0 | 1.0 | 0.0 | 0.0 | 0.0 | -1.0 | 0.0 | 0.0 | -1.0 | 0.0 | 0.0 | 0.0 | 0.0 | 0.0 | 0.0 | 0.0 | 2.0 | 0.0 | 0.0 | 0.0 | 0.0 | 0.0 | -0.333 | 0.0 | 0.0 | -0.001 | 0.0 | 0.0 | 0.0 | 0.0 | 0.0 | 7 | 3.00011 | 4.41 | 0.6803 |
| $GP^-$ | 2 | 0.0 | 0.0 | 0.0 | 0.0 | 0.0 | 0.0 | 0.0 | 0.0 | 0.0 | 1.0 | 0.0 | 0.0 | 0.0 | -1.0 | -1.0 | 0.0 | 0.0 | 0.0 | 0.0 | 0.0 | 0.0 | 0.0 | 0.0 | 0.0 | 0.0 | 0.0 | 0.0 | 0.0 | 0.0 | 0.0 | 0.0 | 0.0 | 3 | 1.4203 | 2.17 | 0.65452 |
| $GR^+$ | 3 | 0.0 | 0.0 | 0.0 | 0.0 | 0.0 | -1.0 | 0.0 | 0.0 | 0.0 | 0.0 | 1.0 | 0.0 | 0.0 | 0.0 | -1.0 | 0.0 | 0.0 | 2.0 | 0.0 | 0.0 | 0.0 | 0.0 | 0.0 | -0.333 | 0.0 | 0.0 | -0.001 | 0.0 | -0.0 | 0.0 | 0.0 | 0.0 | 6 | 3.88846 | 4.186 | 0.92892 |
| $GR^-$ | 4 | 0.0 | 0.0 | 0.0 | 0.0 | 0.0 | 1.0 | 0.0 | 0.0 | 0.0 | 0.0 | -1.0 | 0.0 | 0.0 | -1.0 | 0.0 | 0.0 | 0.0 | 0.0 | 0.0 | 0.0 | 0.0 | 0.0 | 0.0 | 0.0 | 0.0 | 0.0 | 0.0 | 0.0 | 0.0 | 0.0 | 0.0 | 0.0 | 3 | 0.16614 | 2.17 | 0.07656 |
| $GRP^+$ | 5 | 0.0 | 0.0 | -6.0 | 0.0 | 0.0 | 0.0 | 0.0 | 0.0 | 0.0 | 0.0 | 0.0 | 0.0 | 0.0 | 0.0 | 0.0 | 0.0 | 0.0 | 6.0 | 0.0 | 0.0 | 0.0 | -1.0 | 0.0 | -1.0 | 0.0 | 0.0 | 0.0 | 0.0 | 0.0 | 0.0 | 0.0 | 0.0 | 4 | 6.93816 | 6.72 | 1.03246 |
| $GRP^-$ | 6 | 0.0 | 0.0 | 1.0 | 1.0 | 0.0 | 0.0 | 0.0 | 0.0 | 0.0 | 0.0 | 0.0 | 0.0 | 0.0 | -1.0 | -1.0 | 0.0 | 0.0 | 0.0 | 0.0 | 0.0 | 0.0 | 0.0 | 0.0 | 0.0 | 0.0 | 0.0 | 1.0 | 1.0 | -1.0 | 0.0 | 0.0 | 0.0 | 7 | 1.70802 | 2.24 | 0.76251 |
| $P^+$ | 7 | 0.0 | 0.0 | 0.0 | 0.0 | 0.0 | 0.0 | 0.0 | 0.0 | 0.0 | 1.0 | 0.0 | 0.0 | 0.0 | 0.0 | 0.0 | -1.0 | 0.0 | 0.0 | 0.0 | 0.0 | 0.0 | -1.0 | 0.0 | 0.0 | 0.0 | 0.0 | 1.0 | 2.0 | 0.0 | 0.0 | 0.0 | 0.0 | 5 | 3.75512 | 2.38 | 1.57778 |
| $P^-$ | 8 | 0.0 | 0.0 | 0.0 | 0.0 | 0.0 | 1.0 | 0.0 | 0.0 | 0.0 | -1.0 | 0.0 | 0.0 | 0.0 | 0.0 | 0.0 | 0.0 | 0.0 | 0.0 | 0.0 | 0.0 | 0.0 | 0.0 | 0.0 | 0.0 | 0.0 | 0.0 | 0.0 | 0.0 | 0.0 | 0.0 | 0.0 | 0.0 | 2 | 0.10584 | 2.17 | 0.04877 |
| $AP^+$ | 9 | -1.0 | 0.0 | -1.0 | 0.0 | 0.0 | 0.0 | 1.0 | 0.0 | 0.0 | 1.0 | 0.0 | 0.0 | 0.0 | 0.0 | 0.0 | -1.0 | 0.0 | 0.0 | 0.0 | 0.0 | 0.0 | 0.0 | 0.0 | 0.0 | 0.0 | 0.0 | 0.0 | 0.0 | 0.0 | 0.0 | 0.0 | 0.0 | 5 | 3.49 | 4.34 | 0.80415 |
| $AP^-$ | 10 | 1.0 | 0.0 | 1.0 | 0.0 | 0.0 | 0.0 | -1.0 | 0.0 | 0.0 | -1.0 | 0.0 | -2.0 | 0.0 | 0.0 | 0.0 | 1.0 | 0.0 | 0.0 | 2.0 | 0.0 | 0.0 | 0.0 | 0.0 | 0.0 | 0.0 | 0.0 | 0.0 | 0.0 | 0.0 | 0.0 | 0.0 | 0.0 | 7 | 0.46346 | 4.368 | 0.1061 |
| $N^+$ | 11 | 0.0 | 0.0 | 0.0 | 0.0 | 0.0 | 1.0 | 0.0 | 0.0 | 0.0 | 0.0 | -1.0 | 0.0 | 0.0 | 0.0 | 0.0 | 0.0 | -1.0 | 0.0 | 0.0 | 0.0 | 0.0 | 0.0 | 0.0 | 0.0 | 0.0 | 0.0 | 0.0 | 0.0 | 0.0 | 0.0 | 0.0 | 0.0 | 3 | 0.0301 | 2.17 | 0.01387 |
| $NP^+$ | 12 | 0.0 | 0.0 | 0.0 | 0.0 | 0.0 | 0.0 | 0.0 | 0.0 | 0.0 | 0.0 | 0.0 | 0.0 | 0.0 | 0.0 | 0.0 | 0.0 | 0.0 | 0.0 | 0.0 | 0.0 | 0.0 | 1.0 | 0.0 | 1.0 | 0.0 | 0.0 | 0.0 | 0.0 | 0.0 | -1.0 | 0.0 | 0.0 | 3 | 0.0658 | 0.42 | 0.15667 |
| $G^+$ | 13 | 0.0 | 0.0 | 0.0 | 0.0 | 0.0 | 0.0 | 0.0 | 0.0 | 0.0 | 0.0 | 0.0 | 0.0 | 0.0 | 0.0 | 0.0 | 0.0 | 0.0 | 0.0 | 0.0 | 0.0 | 0.0 | 0.0 | 0.0 | 0.0 | 0.0 | 0.0 | 0.0 | 0.0 | 0.0 | 2.0 | -2.0 | 0.0 | 2 | 3.2 | 0.84 | 3.80952 |
| $P\_{\mathrm{tot}}$ | 14 | 0.0 | 0.0 | 0.0 | 0.0 | 0.0 | 1.0 | 0.0 | 0.0 | 0.0 | 0.0 | 0.0 | 0.0 | 0.0 | 0.0 | 0.0 | -1.0 | 0.0 | 0.0 | 0.0 | 0.0 | 0.0 | -1.0 | 0.0 | 0.0 | 0.0 | 0.0 | 1.0 | 2.0 | 0.0 | 0.0 | 0.0 | 0.0 | 5 | 3.86097 | 2.38 | 1.62226 |
| $N\_{\mathrm{tot}}$ | 15 | 0.0 | 0.0 | 0.0 | 0.0 | 0.0 | 0.0 | 0.0 | 0.0 | 0.0 | 0.0 | 0.0 | 0.0 | 0.0 | 0.0 | 0.0 | 0.0 | 0.0 | 0.0 | 0.0 | 0.0 | 0.0 | 0.0 | 0.0 | 0.0 | 0.0 | 0.0 | 0.0 | 0.0 | 0.0 | 0.0 | 0.0 | 0.0 | 0 | 0.089 | 0.0 | $\infty$ |
| $G\_{\mathrm{tot}}$ | 16 | 0.0 | 0.0 | 0.0 | 0.0 | 0.0 | 0.0 | 0.0 | 0.0 | 0.0 | 0.0 | 0.0 | 0.0 | 0.0 | 0.0 | 0.0 | 0.0 | 0.0 | 0.0 | 0.0 | 0.0 | 0.0 | 0.0 | 0.0 | 0.0 | 0.0 | 0.0 | 0.0 | 0.0 | 0.0 | 0.0 | 0.0 | 0.0 | 0 | 3.44 | 0.0 | $\infty$ |
| $NP\_{\mathrm{tot}}$ | 17 | 0.0 | 0.0 | 0.0 | 0.0 | 0.0 | 0.0 | 0.0 | 0.0 | 0.0 | 0.0 | 0.0 | 0.0 | 0.0 | 0.0 | 0.0 | 0.0 | 0.0 | 0.0 | 0.0 | 0.0 | 0.0 | 0.0 | 0.0 | 0.0 | 0.0 | 0.0 | 0.0 | 0.0 | 0.0 | 0.0 | 0.0 | 0.0 | 0 | 0.066 | 0.0 | $\infty$ |
| $\pi\_{j}$ |  | 3.0 | 0.0 | 5.0 | 1.0 | 0.0 | 5.0 | 3.0 | 0.0 | 0.0 | 6.0 | 3.0 | 1.0 | 0.0 | 3.0 | 3.0 | 4.0 | 1.0 | 3.0 | 1.0 | 0.0 | 0.0 | 4.0 | 0.0 | 4.0 | 0.0 | 0.0 | 5.0 | 3.0 | 1.0 | 2.0 | 1.0 | 0.0 |  |  |  |  |

##### Ratios: Towards Physiology¶

The five ratios from the glycolytic system alone carry over to the system of the combined pathways. The addition of the pentose pathway adds new property ratios related to the type of redox charge that NADPH carries.

###### Additional redox charges¶

The quotient between the charged and uncharged states can be used to define property ratios as in Chapter 10. The ratios are the same here as for glycolysis, with the addition of the following redox charges:

- the NADPH conversion value of the intermediates;

\[\begin{equation} r\_6 = \frac{GRP^+}{GRP^+ + GRP^-} \tag{11.6} \end{equation}\]

- the NADPH carrier;

\[\begin{equation}r\_7 = \frac{\text{NADPH}}{\text{NADPH} + \text{NADP}} = \frac{NP^+}{NP^+ + NP^-} \tag{11.7} \end{equation}\]

- the glutathione carrier

\[\begin{equation} r\_8 = \frac{\text{GSH}}{\text{GSH} + 2\text{GSSG}} \tag{11.8} \end{equation}\]

###### Dynamic responses of the ratios¶

The response to the increased rate of GSH utilization on the cofactor charge ratios is shown in Figure 11.7.1. The glutathione redox charge drops from 0.93 to 0.88 (Figure 11.22a) and complementary to that there is a modest increase in the NADPH redox charge (Figure 11.22b). Figure 11.22c and Figure 11.22d show the dip in the NADH redox charge and the adenosine phosphate energy charge that result from the effect that increased pentose pathway flux has on reduced glycolysis flux. These ratios
drop modestly but then return almost to their exact initial value. Thus, there is dynamic interaction between the pathways, but the steady state remains similar.

```
[54]:
```

```
# Make pool solutions
pooling_matrix, pool_labels = make_pooling_matrix(True)
pools = {}
for i, pool_id in enumerate(pool_labels[:-2]):
    terms = ["*".join((str(coeff), met))
             for coeff, met in zip(pooling_matrix[i], metabolite_ids)
             if coeff != 0]
    variables = [term.split("*")[-1] for term in terms]
    pools.update({pool_id: ["+".join(terms), variables]})

ratios = {}
keys = [('$G^+$', '$G^-$'), ('$NP^+$', '$NP^-$'),
        ('$AP^+$', '$AP^-$'), ('$N^+$', '$N^-$')]
ratio_names = ["GSH Redox Charge", "NADPH Redox Charge",
               "Adenylate Phosphate Energy Charge", "NADH Redox Charge"]
for name, (k1, k2) in zip(ratio_names, keys):
    ratio = " / ".join(["({0})".format(pools[k1][0]),
                        "({0} + {1})".format(pools[k1][0], pools[k2][0])])
    variables = list(set(pools[k1][1] + pools[k2][1]))
    ratios[name] = [ratio, variables]

for ratio_id, (equation, variables) in ratios.items():
    conc_sol.make_aggregate_solution(
        ratio_id, equation=equation, variables=variables)

fig_11_22, axes = plt.subplots(nrows=2, ncols=2, figsize=(12, 6))
axes = axes.flatten()

ylims=[(0.85, 1), (0.95, 1), (0.85, 0.9),(0.31, 0.34)]
for i, (ax, ratio) in enumerate(zip(axes, list(ratios))):
    plot_time_profile(
        conc_sol, observable=ratio, ax=axes[i],
        xlim=(t0, 5), ylim=ylims[i],
        xlabel=("Time [hr]", S_FONT),
        ylabel=("Ratio", S_FONT), title=(ratio, L_FONT))
fig_11_22.tight_layout()
```

**Figure 11.22:** The dynamic response of the combined glycolytic and pentose phosphate pathway ratios to a doubling of rate of GSH utilization, see Section 11.4. (a) The response of the glutathione redox ratio. (b) The response of the NADPH redox ratio. (c) The response of the adenosine phosphate energy charge. (d) The response of the NADH redox ratio.

##### Summary¶

- A stoichiometric matrix for the pentose pathway can be formed and a MASS model built to simulate the dynamic responses of this pathway.
- The stoichiometric matrix for the pentose pathway can be merged with the stoichiometric matrix for glycolysis to form a system that describes the coupling of the two pathways.
- By going through the process of coupling the pentose pathway to glycolysis, we begin to see the emergence of systems biology with scale.
- The basis for the null space of \(\textbf{S}\) gets complicated. The primary pentose pathway vector now becomes cyclic as it connects the glycolytic inputs and outputs from the pentose pathway. The pentose pathway thus integrates with glycolysis.
- To define the composition of the redox pools we have to deploy linear programming as it becomes difficult to determine the redox value of the intermediates by simple inspection. This difficulty arises from the coupling of two processes and the growing scale of the model. LP optimization can solve this problem.
- The response of the coupled pathways to an increase to the rate of use of the redox potential generated by the pentose pathway shows the importance of GSSG/GSH buffering. The concentrations of glutathione is high and buffers the response.
- Nevertheless, the pathways are coupled and this perturbation does influence glycolysis. The simulations show the interactions between glycolysis and the pentose pathway when we put energy and redox loads on the coupled pathways. Thus, it may be detrimental to think of the pentose pathway as just the producer of R5P, as one needs many of the glycolytic reactions to form R5P.
- Therefore, we have to begin to think about the network as a whole. This point of view becomes even more apparent in the next chapter.

\(\tiny{\text{© B. Ø. Palsson 2011;}\ \text{This publication is in copyright.}\\ \text{Subject to statutory exception and to the provisions of relevant collective licensing agreements,}\\ \text{no reproduction of any part may take place without the written permission of Cambridge University Press.}}\)

#### Building Networks¶

##### The AMP Salvage Network¶

The previous chapter described the integration of two pathways. We will now add yet another set of reactions to the previously integrated pathways. The AMP input and output that was described earlier by two simple reactions across the system boundary represent a network of reactions that are called purine degradation and salvage pathways. The salvage pathways recycle a pentose through the attachment of an imported purine base to effectively reverse AMP degradation. AMP degradation and salvage
are low flux pathways but have several genetic mutations in the human population that cause serious diseases. Thus, even though fluxes through these pathways are low, their function is critical. As we have seen, many of the long-term dynamic responses are determined by the adjustment of the total adenylate cofactor pool. In this chapter, we integrate the degradation and salvage pathways to form a core metabolic network for the human red blood cell.

**MASSpy** will be used to demonstrate some of the topics in this chapter.

```
[1]:
```

```
from mass import (
    MassModel, MassMetabolite, MassReaction,
    Simulation, MassSolution, strip_time)
from mass.test import create_test_model
from mass.util.matrix import nullspace, left_nullspace, matrix_rank
from mass.visualization import (
    plot_time_profile, plot_phase_portrait, plot_tiled_phase_portraits,
    plot_comparison)
```

Other useful packages are also imported at this time.

```
[2]:
```

```
from os import path
from cobra import DictList
import matplotlib as mpl
import matplotlib.pyplot as plt
import numpy as np
import pandas as pd
import sympy as sym
```

Some options and variables used throughout the notebook are also declared here.

```
[3]:
```

```
pd.set_option("display.max_rows", 100)
pd.set_option("display.max_columns", 100)
pd.set_option('display.max_colwidth', None)
pd.options.display.float_format = '{:,.3f}'.format
S_FONT = {"size": "small"}
L_FONT = {"size": "large"}
INF = float("inf")
```

##### AMP Metabolism¶

AMP metabolism forms a sub-network in the overall red blood cell metabolic network. We will first consider its properties before we integrate it with the combined glycolytic and pentose pathway model.

###### The AMP metabolic sub-network¶

AMP is simultaneously degraded and synthesized, creating a dynamic balance (Figure 12.1). The metabolites that are found in these pathways and that are over and above those that are in the integrated glycolytic and pentose pathway network are listed in Table 12.1. To build this sub-network, prior to integration with the glycolytic and pentose pathway network, we need consider a sub-network comprised of the metabolites shown in Table 12.1. The reactions that take place in the degradation and
biosynthesis of AMP are shown in Table 12.2.

**Figure 12.1:** The nucleotide metabolism considered in this chapter. There are two biochemical ways in which AMP is degraded and two ways in which it is synthesized.

```
[4]:
```

```
ampsn = create_test_model("SB2_AMPSalvageNetwork")
```

###### Degradation¶

AMP can be degraded either by dephosphorylation or by deamination. In the former case, adenosine (ADO) is formed and can cross the cell membrane to enter plasma. In the latter case, IMP is formed and can then subsequently be dephosphorylated to form inosine (INO). INO can then cross the cell membrane, or be further degraded to form a pentose (R1P) and the purine base hypoxanthine (HYP), the latter of which can be exchanged with plasma. HYP will become uric acid, the accumulation of which causes
hyperuricemia (gout).

###### Biosynthesis¶

The red blood cell does not have the capacity for de novo adenine synthesis. It can synthesize AMP in two different ways. First, it can phosphorylate ADO directly to form AMP using an ATP to ADP conversion. This ATP use creates an energy load met by glycolysis. A second process to offset degradation of adenine is the salvage pathway, in which adenine (ADE) is picked up from plasma and is combined with phosphoribosyl diphosphate (PRPP) to form AMP. PRPP is formed from R5P using two ATP
equivalents, where the R5P is formed from isomerization of R1P that is formed during INO degradation. In this way, the pentose is recycled to re-synthesize AMP at the cost of two ATP molecules. The R5P can also come from the pentose phosphate pathway in an integrated network.

###### The biochemical reactions¶

**Table 12.1:** Intermediates of AMP degradation and synthesis, their abbreviations and steady state concentrations. The concentrations given are those typical for the human red blood cell. The index on the compounds is added to that for the combined glycolysis and pentose phosphate pathway, (Table 11.1).

```
[5]:
```

```
metabolite_ids = [m.id for m in ampsn.metabolites
                  if m.id not in ["r5p_c", "atp_c", "adp_c",
                                  "amp_c", "pi_c", "h_c", "h2o_c"]]

table_12_1 = pd.DataFrame(
    np.array([metabolite_ids,
              [met.name for met in ampsn.metabolites
               if met.id in metabolite_ids],
              [ampsn.initial_conditions[met] for met in ampsn.metabolites
               if met.id in metabolite_ids]]).T,
    index=[i for i in range(33, len(metabolite_ids) + 33)],
    columns=["Abbreviations", "Species", "Initial Concentration"])
table_12_1
```

```
[5]:
```

|  | Abbreviations | Species | Initial Concentration |
| --- | --- | --- | --- |
| 33 | adn\_c | Adenosine | 0.0012 |
| 34 | ade\_c | Adenine | 0.001 |
| 35 | imp\_c | Inosine monophosphate | 0.01 |
| 36 | ins\_c | Inosine | 0.001 |
| 37 | hxan\_c | Hypoxanthine | 0.002 |
| 38 | r1p\_c | Alpha-D-Ribose 1-phosphate | 0.06 |
| 39 | prpp\_c | 5-Phospho-alpha-D-ribose 1-diphosphate | 0.005 |
| 40 | nh3\_c | Ammonia | 0.091002 |

**Table 12.2:** AMP degradation and biosynthetic pathway enzymes and transporters, their abbreviations and chemical reactions.The index on the reactions is added to that for the combined glycolysis and pentose pathways (Table 11.2)\*

```
[6]:
```

```
reaction_ids = [r.id for r in ampsn.reactions
                if r.id not in ["SK_g6p_c", "DM_f6p_c", "SK_amp_c",
                                "SK_h_c", "SK_h2o_c"]]
table_12_2 = pd.DataFrame(
    np.array([reaction_ids,
              [r.name for r in ampsn.reactions
               if r.id in reaction_ids],
              [r.reaction for r in ampsn.reactions
               if r.id in reaction_ids]]).T,
    index=[i for i in range(34, len(reaction_ids) + 34)],
    columns=["Abbreviations", "Enzymes/Transporter/Load", "Elementally Balanced Reaction"])
table_12_2
```

```
[6]:
```

|  | Abbreviations | Enzymes/Transporter/Load | Elementally Balanced Reaction |
| --- | --- | --- | --- |
| 34 | ADNK1 | Adenosine kinase | adn\_c + atp\_c --> adp\_c + amp\_c + h\_c |
| 35 | NTD7 | 5'-nucleotidase (AMP) | amp\_c + h2o\_c --> adn\_c + pi\_c |
| 36 | AMPDA | Adenosine monophosphate deaminase | amp\_c + h2o\_c --> imp\_c + nh3\_c |
| 37 | NTD11 | 5'-nucleotidase (IMP) | h2o\_c + imp\_c --> ins\_c + pi\_c |
| 38 | ADA | Adenosine deaminase | adn\_c + h2o\_c --> ins\_c + nh3\_c |
| 39 | PUNP5 | Purine-nucleoside phosphorylase (Inosine) | ins\_c + pi\_c <=> hxan\_c + r1p\_c |
| 40 | PPM | Phosphopentomutase | r1p\_c <=> r5p\_c |
| 41 | PRPPS | Phosphoribosylpyrophosphate synthetase | 2 atp\_c + r5p\_c --> 2 adp\_c + h\_c + prpp\_c |
| 42 | ADPT |  | ade\_c + h2o\_c + prpp\_c --> amp\_c + h\_c + 2 pi\_c |
| 43 | ATPM | ATP maintenance requirement | adp\_c + h\_c + pi\_c --> atp\_c + h2o\_c |
| 44 | SK\_adn\_c | Adenosine sink | adn\_c <=> |
| 45 | SK\_ade\_c | Adenine sink | ade\_c <=> |
| 46 | SK\_ins\_c | Inosine sink | ins\_c <=> |
| 47 | SK\_hxan\_c | Hypoxanthine sink | hxan\_c <=> |
| 48 | SK\_nh3\_c | Ammonia sink | nh3\_c <=> |
| 49 | SK\_pi\_c | Phosphate sink | pi\_c <=> |

**Table 12.3:** The elemental composition and charges of the AMP Salvage Network intermediates. This table represents the matrix \(\textbf{E}.\)

```
[7]:
```

```
table_12_3 = ampsn.get_elemental_matrix(array_type="DataFrame",
                                        dtype=np.int64)
table_12_3
```

```
[7]:
```

|  | adn\_c | ade\_c | imp\_c | ins\_c | hxan\_c | r1p\_c | r5p\_c | prpp\_c | atp\_c | adp\_c | amp\_c | pi\_c | nh3\_c | h\_c | h2o\_c |
| --- | --- | --- | --- | --- | --- | --- | --- | --- | --- | --- | --- | --- | --- | --- | --- |
| C | 10 | 5 | 10 | 10 | 5 | 5 | 5 | 5 | 10 | 10 | 10 | 0 | 0 | 0 | 0 |
| H | 13 | 5 | 11 | 12 | 4 | 9 | 9 | 8 | 12 | 12 | 12 | 1 | 3 | 1 | 2 |
| O | 4 | 0 | 8 | 5 | 1 | 8 | 8 | 14 | 13 | 10 | 7 | 4 | 0 | 0 | 1 |
| P | 0 | 0 | 1 | 0 | 0 | 1 | 1 | 3 | 3 | 2 | 1 | 1 | 0 | 0 | 0 |
| N | 5 | 5 | 4 | 4 | 4 | 0 | 0 | 0 | 5 | 5 | 5 | 0 | 1 | 0 | 0 |
| S | 0 | 0 | 0 | 0 | 0 | 0 | 0 | 0 | 0 | 0 | 0 | 0 | 0 | 0 | 0 |
| q | 0 | 0 | -2 | 0 | 0 | -2 | -2 | -5 | -4 | -3 | -2 | -2 | 0 | 1 | 0 |

**Table 12.4:** The elemental and charge balance test on the reactions. All internal reactions are balanced. Exchange reactions are not.

```
[8]:
```

```
table_12_4 = ampsn.get_elemental_charge_balancing(array_type="DataFrame",
                                                  dtype=np.int64)
table_12_4
```

```
[8]:
```

|  | ADNK1 | NTD7 | AMPDA | NTD11 | ADA | PUNP5 | PPM | PRPPS | ADPT | ATPM | SK\_adn\_c | SK\_ade\_c | SK\_ins\_c | SK\_hxan\_c | SK\_nh3\_c | SK\_pi\_c | SK\_amp\_c | SK\_h\_c | SK\_h2o\_c |
| --- | --- | --- | --- | --- | --- | --- | --- | --- | --- | --- | --- | --- | --- | --- | --- | --- | --- | --- | --- |
| C | 0 | 0 | 0 | 0 | 0 | 0 | 0 | 0 | 0 | 0 | -10 | -5 | -10 | -5 | 0 | 0 | -10 | 0 | 0 |
| H | 0 | 0 | 0 | 0 | 0 | 0 | 0 | 0 | 0 | 0 | -13 | -5 | -12 | -4 | -3 | -1 | -12 | -1 | -2 |
| O | 0 | 0 | 0 | 0 | 0 | 0 | 0 | 0 | 0 | 0 | -4 | 0 | -5 | -1 | 0 | -4 | -7 | 0 | -1 |
| P | 0 | 0 | 0 | 0 | 0 | 0 | 0 | 0 | 0 | 0 | 0 | 0 | 0 | 0 | 0 | -1 | -1 | 0 | 0 |
| N | 0 | 0 | 0 | 0 | 0 | 0 | 0 | 0 | 0 | 0 | -5 | -5 | -4 | -4 | -1 | 0 | -5 | 0 | 0 |
| S | 0 | 0 | 0 | 0 | 0 | 0 | 0 | 0 | 0 | 0 | 0 | 0 | 0 | 0 | 0 | 0 | 0 | 0 | 0 |
| q | 0 | 0 | 0 | 0 | 0 | 0 | 0 | 0 | 0 | 0 | 0 | 0 | 0 | 0 | 0 | 2 | 2 | -1 | 0 |

```
[9]:
```

```
for boundary in ampsn.boundary:
    print(boundary)
```

```
SK_adn_c: adn_c <=>
SK_ade_c: ade_c <=>
SK_ins_c: ins_c <=>
SK_hxan_c: hxan_c <=>
SK_nh3_c: nh3_c <=>
SK_pi_c: pi_c <=>
SK_amp_c: amp_c <=>
SK_h_c: h_c <=>
SK_h2o_c: h2o_c <=>
```

###### The pathway structure: basis for the null space¶

Five pathway vectors chosen to span the null space are shown in Table 12.5. They can be divided into groups of degradative and biosynthetic pathways.

**Table 12.5:** The calculated null space pathway vectors for the stoichiometric matrix for the AMP Salvage Network.

```
[10]:
```

```
reaction_ids = [r.id for r in ampsn.reactions]
# MinSpan pathways are calculated outside this notebook and the results are provided here.
minspan_paths = np.array([
    [0, 1, 0, 0, 0, 0, 0, 0, 0, 0, 1, 0, 0, 0, 0, 1,-1, 1,-1],
    [0, 0, 1, 1, 0, 0, 0, 0, 0, 0, 0, 0, 1, 0, 1, 1,-1, 1,-2],
    [0, 1, 0, 0, 1, 0, 0, 0, 0, 0, 0, 0, 1, 0, 1, 1,-1, 1,-2],
    [1, 0, 0, 0, 0, 0, 0, 0, 0, 1,-1, 0, 0, 0, 0,-1, 1,-1, 1],
    [0, 0, 1, 1, 0, 1, 1, 1, 1, 2, 0,-1, 0, 1, 1, 0, 0, 0,-1]])
# Create labels for the paths
path_labels = ["$p_1$", "$p_2$", "$p_3$", "$p_4$", "$p_5$"]
# Create DataFrame
table_12_5 = pd.DataFrame(minspan_paths, index=path_labels,
                          columns=reaction_ids, dtype=np.int64)
table_12_5
```

```
[10]:
```

|  | ADNK1 | NTD7 | AMPDA | NTD11 | ADA | PUNP5 | PPM | PRPPS | ADPT | ATPM | SK\_adn\_c | SK\_ade\_c | SK\_ins\_c | SK\_hxan\_c | SK\_nh3\_c | SK\_pi\_c | SK\_amp\_c | SK\_h\_c | SK\_h2o\_c |
| --- | --- | --- | --- | --- | --- | --- | --- | --- | --- | --- | --- | --- | --- | --- | --- | --- | --- | --- | --- |
| $p\_1$ | 0 | 1 | 0 | 0 | 0 | 0 | 0 | 0 | 0 | 0 | 1 | 0 | 0 | 0 | 0 | 1 | -1 | 1 | -1 |
| $p\_2$ | 0 | 0 | 1 | 1 | 0 | 0 | 0 | 0 | 0 | 0 | 0 | 0 | 1 | 0 | 1 | 1 | -1 | 1 | -2 |
| $p\_3$ | 0 | 1 | 0 | 0 | 1 | 0 | 0 | 0 | 0 | 0 | 0 | 0 | 1 | 0 | 1 | 1 | -1 | 1 | -2 |
| $p\_4$ | 1 | 0 | 0 | 0 | 0 | 0 | 0 | 0 | 0 | 1 | -1 | 0 | 0 | 0 | 0 | -1 | 1 | -1 | 1 |
| $p\_5$ | 0 | 0 | 1 | 1 | 0 | 1 | 1 | 1 | 1 | 2 | 0 | -1 | 0 | 1 | 1 | 0 | 0 | 0 | -1 |

The first three pathways, \(\textbf{p}\_1\), \(\textbf{p}\_2\), and \(\textbf{p}\_3\) are degradation pathways of AMP. The first pathway degrades AMP to ADO via dephosphorylation. The second pathway degrades AMP to IMP via AMP deaminase followed by dephosphorylation and secretion of INO. The third pathway degrades AMP first with dephosphorylation to ADO followed by deamination to INO and its secretion. These are shown graphically on the reaction map in Figure 12.2. Note that the second
and third pathways are equivalent overall, but take an alternative route through the network. These two pathways are said to have an equivalent input/output signature (*Systems Biology: Properties of Reconstructed Networks*).

The fourth pathway, \(\textbf{p}\_4\), shows the import of adenosine (ADO) and its phosphorylation via direct use of ATP. Thus, the cost of the AMP molecule now relative to the plasma environment is one high-energy phosphate bond. In the previous chapters, AMP was directly imported and did not cost any high-energy bonds in the defined system. Pathway four is essentially the opposite of pathway one, except it requires ATP for fuel. If one sums these two pathways, a futile cycle results.

The fifth pathway, \(\textbf{p}\_5\), is a salvage pathway. It takes the INO produced through degradation of AMP, cleaves the pentose off to form and secrete hypoxanthine (HYP), and recycles the pentose through the formation of PRPP at the cost of two ATP molecules. PPRP is then combined with an imported adenine base to form AMP. This pathway is energy requiring, needing two ATPs. As we detail below, some of the chemical reactions of this pathway change when it is integrated with glycolysis
and the pentose pathway, as we have modified the PRPP synthase reaction to make it easier to analyze this sub-network.

**Figure 12.2:** The graphical depiction of the five chosen pathway vectors of the null space of the stoichiometric matrix, shown in Table 12.1.5. From left to right: The first three pathways \(\textbf{p}\_1\), \(\textbf{p}\_2\), and \(\textbf{p}\_3\), are degradation pathways, while the \(\textbf{p}\_4\) is a direct synthesis pathway and \(\textbf{p}\_5\) is a salvage pathway.

###### The time invariant pools: the basis for the left null space¶

The left null space is one dimensional. It has one time invariant: ATP + ADP. In this sub-network, this sum acts like a conserved cofactor.

**Table 12.6:** The left null space composed of the time invariant ATP + ADP pool.

```
[11]:
```

```
metabolite_ids = [m.id for m in ampsn.metabolites]
lns = left_nullspace(ampsn.S, rtol=1e-10)
# Iterate through left nullspace,
# dividing by the smallest value in each row.
for i, row in enumerate(lns):
    minval = np.min(abs(row[np.nonzero(row)]))
    new_row = np.array(row/minval)
    # Round to ensure the left nullspace is composed of only integers
    lns[i] = np.array([round(value) for value in new_row])

# Ensure positive stoichiometric coefficients if all are negative
for i, space in enumerate(lns):
    lns[i] = np.negative(space) if all([num <= 0 for num in space]) else space

lns = lns.astype(np.int64)
# Create labels for the time invariants
time_inv_labels = ["ATP + ADP"]
table_12_6 = pd.DataFrame(lns, index=time_inv_labels,
                          columns=metabolite_ids, dtype=np.int64)
table_12_6
```

```
[11]:
```

|  | adn\_c | ade\_c | imp\_c | ins\_c | hxan\_c | r1p\_c | r5p\_c | prpp\_c | atp\_c | adp\_c | amp\_c | pi\_c | nh3\_c | h\_c | h2o\_c |
| --- | --- | --- | --- | --- | --- | --- | --- | --- | --- | --- | --- | --- | --- | --- | --- |
| ATP + ADP | 0 | 0 | 0 | 0 | 0 | 0 | 0 | 0 | 1 | 1 | 0 | 0 | 0 | 0 | 0 |

###### An ‘annotated’ form of the stoichiometric matrix¶

All of the properties of the stoichiometric matrix can be conveniently summarized in a tabular format, Table 12.7. The table succinctly summarizes the chemical and topological properties of \(\textbf{S}\). The matrix has dimensions of 15x19 and a rank of 14. It thus has a 5 dimensional null space and a 1 dimensional left null space.

**Table 12.7:** The stoichiometric matrix for the AMP Salvage Network seen in Figure 12.1. The matrix is partitioned to show the intermediates (yellow) separate from the cofactors and to separate the exchange reactions and cofactor loads (orange). The connectivities, \(\rho\_i\) (red), for a compound, and the participation number, \(\pi\_j\) (cyan), for a reaction are shown. The second block in the table is the product \(\textbf{ES}\) (blue) to evaluate elemental balancing status of
the reactions. All exchange reactions have a participation number of unity and are thus not elementally balanced. The last block in the table has the five pathway vectors (purple) for the AMP Salvage Network. These vectors are graphically shown in Figure 12.2. Furthest to the right, we display the time invariant pools (green) that span the left null space.

```
[12]:
```

```
# Define labels
pi_str = r"$\pi_{j}$"
rho_str = r"$\rho_{i}$"
chopsnq = ['C', 'H', 'O', 'P', 'N', 'S', 'q' ]

# Make table content from the stoichiometric matrix, elemental balancing of pathways
# participation number, and MinSpan pathways
S_matrix = ampsn.update_S(array_type="dense", dtype=np.int64, update_model=False)
ES_matrix = ampsn.get_elemental_charge_balancing(dtype=np.int64)
pi = np.count_nonzero(S_matrix, axis=0)
rho = np.count_nonzero(S_matrix, axis=1)
table_12_7 = np.vstack((S_matrix, pi, ES_matrix, minspan_paths))

# Determine number of blank entries needed to be added to pad the table,
# Add connectivity number and time invariants to table content
blanks = [""]*(len(table_12_7) - len(ampsn.metabolites))
rho = np.concatenate((rho, blanks))
time_inv = np.array([np.concatenate([row, blanks]) for row in lns])
table_12_7 = np.vstack([table_12_7.T, rho, time_inv]).T

colors = {"intermediates": "#ffffe6", # Yellow
          "cofactors": "#ffe6cc",     # Orange
          "chopsnq": "#99e6ff",       # Blue
          "pathways": "#b399ff",      # Purple
          "pi": "#99ffff",            # Cyan
          "rho": "#ff9999",           # Red
          "time_invs": "#ccff99",     # Green
          "blank": "#f2f2f2"}         # Grey
bg_color_str = "background-color: "
def highlight_table(df, model, main_shape):
    df = df.copy()
    n_mets, n_rxns = (len(model.metabolites), len(model.reactions))
    # Highlight rows
    for row in df.index:
        other_key, condition = ("blank", lambda i, v: v != "")
        if row == pi_str:        # For participation
            main_key = "pi"
        elif row in chopsnq:     # For elemental balancing
            main_key = "chopsnq"
        elif row in path_labels: # For pathways
            main_key = "pathways"
        else:
            # Distinguish between intermediate and cofactor reactions for model reactions
            main_key, other_key = ("cofactors", "intermediates")
            condition = lambda i, v: (main_shape[1] <= i and i < n_rxns)
        df.loc[row, :] = [bg_color_str + colors[main_key] if condition(i, v)
                          else bg_color_str + colors[other_key]
                          for i, v in enumerate(df.loc[row, :])]

    for col in df.columns:
        condition = lambda i, v: v != bg_color_str + colors["blank"]
        if col == rho_str:
            main_key = "rho"
        elif col in time_inv_labels:
            main_key = "time_invs"
        else:
            # Distinguish intermediates and cofactors for model metabolites
            main_key = "cofactors"
            condition = lambda i, v: (main_shape[0] <= i and i < n_mets)
        df.loc[:, col] = [bg_color_str + colors[main_key] if condition(i, v)
                          else v for i, v in enumerate(df.loc[:, col])]
    return df

# Create index and column labels
index_labels = np.concatenate((metabolite_ids, [pi_str], chopsnq, path_labels))
column_labels = np.concatenate((reaction_ids, [rho_str], time_inv_labels))
# Create DataFrame
table_12_7 = pd.DataFrame(
    table_12_7, index=index_labels, columns=column_labels)
# Apply colors
table_12_7 = table_12_7.style.apply(
    highlight_table,  model=ampsn, main_shape=(11, 10), axis=None)
table_12_7
```

```
[12]:
```

|  | ADNK1 | NTD7 | AMPDA | NTD11 | ADA | PUNP5 | PPM | PRPPS | ADPT | ATPM | SK\_adn\_c | SK\_ade\_c | SK\_ins\_c | SK\_hxan\_c | SK\_nh3\_c | SK\_pi\_c | SK\_amp\_c | SK\_h\_c | SK\_h2o\_c | $\rho\_{i}$ | ATP + ADP |
| --- | --- | --- | --- | --- | --- | --- | --- | --- | --- | --- | --- | --- | --- | --- | --- | --- | --- | --- | --- | --- | --- |
| adn\_c | -1 | 1 | 0 | 0 | -1 | 0 | 0 | 0 | 0 | 0 | -1 | 0 | 0 | 0 | 0 | 0 | 0 | 0 | 0 | 4 | 0 |
| ade\_c | 0 | 0 | 0 | 0 | 0 | 0 | 0 | 0 | -1 | 0 | 0 | -1 | 0 | 0 | 0 | 0 | 0 | 0 | 0 | 2 | 0 |
| imp\_c | 0 | 0 | 1 | -1 | 0 | 0 | 0 | 0 | 0 | 0 | 0 | 0 | 0 | 0 | 0 | 0 | 0 | 0 | 0 | 2 | 0 |
| ins\_c | 0 | 0 | 0 | 1 | 1 | -1 | 0 | 0 | 0 | 0 | 0 | 0 | -1 | 0 | 0 | 0 | 0 | 0 | 0 | 4 | 0 |
| hxan\_c | 0 | 0 | 0 | 0 | 0 | 1 | 0 | 0 | 0 | 0 | 0 | 0 | 0 | -1 | 0 | 0 | 0 | 0 | 0 | 2 | 0 |
| r1p\_c | 0 | 0 | 0 | 0 | 0 | 1 | -1 | 0 | 0 | 0 | 0 | 0 | 0 | 0 | 0 | 0 | 0 | 0 | 0 | 2 | 0 |
| r5p\_c | 0 | 0 | 0 | 0 | 0 | 0 | 1 | -1 | 0 | 0 | 0 | 0 | 0 | 0 | 0 | 0 | 0 | 0 | 0 | 2 | 0 |
| prpp\_c | 0 | 0 | 0 | 0 | 0 | 0 | 0 | 1 | -1 | 0 | 0 | 0 | 0 | 0 | 0 | 0 | 0 | 0 | 0 | 2 | 0 |
| atp\_c | -1 | 0 | 0 | 0 | 0 | 0 | 0 | -2 | 0 | 1 | 0 | 0 | 0 | 0 | 0 | 0 | 0 | 0 | 0 | 3 | 1 |
| adp\_c | 1 | 0 | 0 | 0 | 0 | 0 | 0 | 2 | 0 | -1 | 0 | 0 | 0 | 0 | 0 | 0 | 0 | 0 | 0 | 3 | 1 |
| amp\_c | 1 | -1 | -1 | 0 | 0 | 0 | 0 | 0 | 1 | 0 | 0 | 0 | 0 | 0 | 0 | 0 | -1 | 0 | 0 | 5 | 0 |
| pi\_c | 0 | 1 | 0 | 1 | 0 | -1 | 0 | 0 | 2 | -1 | 0 | 0 | 0 | 0 | 0 | -1 | 0 | 0 | 0 | 6 | 0 |
| nh3\_c | 0 | 0 | 1 | 0 | 1 | 0 | 0 | 0 | 0 | 0 | 0 | 0 | 0 | 0 | -1 | 0 | 0 | 0 | 0 | 3 | 0 |
| h\_c | 1 | 0 | 0 | 0 | 0 | 0 | 0 | 1 | 1 | -1 | 0 | 0 | 0 | 0 | 0 | 0 | 0 | -1 | 0 | 5 | 0 |
| h2o\_c | 0 | -1 | -1 | -1 | -1 | 0 | 0 | 0 | -1 | 1 | 0 | 0 | 0 | 0 | 0 | 0 | 0 | 0 | -1 | 7 | 0 |
| $\pi\_{j}$ | 5 | 4 | 4 | 4 | 4 | 4 | 2 | 5 | 6 | 5 | 1 | 1 | 1 | 1 | 1 | 1 | 1 | 1 | 1 |  |  |
| C | 0 | 0 | 0 | 0 | 0 | 0 | 0 | 0 | 0 | 0 | -10 | -5 | -10 | -5 | 0 | 0 | -10 | 0 | 0 |  |  |
| H | 0 | 0 | 0 | 0 | 0 | 0 | 0 | 0 | 0 | 0 | -13 | -5 | -12 | -4 | -3 | -1 | -12 | -1 | -2 |  |  |
| O | 0 | 0 | 0 | 0 | 0 | 0 | 0 | 0 | 0 | 0 | -4 | 0 | -5 | -1 | 0 | -4 | -7 | 0 | -1 |  |  |
| P | 0 | 0 | 0 | 0 | 0 | 0 | 0 | 0 | 0 | 0 | 0 | 0 | 0 | 0 | 0 | -1 | -1 | 0 | 0 |  |  |
| N | 0 | 0 | 0 | 0 | 0 | 0 | 0 | 0 | 0 | 0 | -5 | -5 | -4 | -4 | -1 | 0 | -5 | 0 | 0 |  |  |
| S | 0 | 0 | 0 | 0 | 0 | 0 | 0 | 0 | 0 | 0 | 0 | 0 | 0 | 0 | 0 | 0 | 0 | 0 | 0 |  |  |
| q | 0 | 0 | 0 | 0 | 0 | 0 | 0 | 0 | 0 | 0 | 0 | 0 | 0 | 0 | 0 | 2 | 2 | -1 | 0 |  |  |
| $p\_1$ | 0 | 1 | 0 | 0 | 0 | 0 | 0 | 0 | 0 | 0 | 1 | 0 | 0 | 0 | 0 | 1 | -1 | 1 | -1 |  |  |
| $p\_2$ | 0 | 0 | 1 | 1 | 0 | 0 | 0 | 0 | 0 | 0 | 0 | 0 | 1 | 0 | 1 | 1 | -1 | 1 | -2 |  |  |
| $p\_3$ | 0 | 1 | 0 | 0 | 1 | 0 | 0 | 0 | 0 | 0 | 0 | 0 | 1 | 0 | 1 | 1 | -1 | 1 | -2 |  |  |
| $p\_4$ | 1 | 0 | 0 | 0 | 0 | 0 | 0 | 0 | 0 | 1 | -1 | 0 | 0 | 0 | 0 | -1 | 1 | -1 | 1 |  |  |
| $p\_5$ | 0 | 0 | 1 | 1 | 0 | 1 | 1 | 1 | 1 | 2 | 0 | -1 | 0 | 1 | 1 | 0 | 0 | 0 | -1 |  |  |

###### The steady state¶

The null space is five dimensional. We thus have to specify five fluxes to set the steady state.

1. In a steady state, the synthesis of AMP is balanced by degradation, that is \(v\_{SK\_{amp}}=0\). Thus, the sum of the flux through the first three pathways must be balanced by the fourth to make the AMP exchange rate zero. Note that the fifth pathway has no net AMP exchange rate.
2. The fifth pathway is uniquely defined by either the exchange rate of hypoxanthine or adenine. These two exchange rates are not independent. The uptake rate of adenine is approximately 0.014 mM/hr (Joshi, 1990).
3. The exchange rate of adenosine would specify the relative rate of pathways one and four. The rate of \(v\_{ADNK1}\) is set to 0.12 mM/hr, specifying the flux through \(\textbf{p}\_{4}\). The net uptake rate of adenosine is set at 0.01 mM/hr, specifying the flux of \(\textbf{p}\_{1}\) to be 0.11 mM/hr.
4. Since \(\textbf{p}\_{1}\) and \(\textbf{p}\_{4}\) differ by 0.01 mM/hr in favor of AMP synthesis, it means that the sum of \(\textbf{p}\_{2}\) and \(\textbf{p}\_{3}\) has to be 0.01 mM/hr. To specify the contributions to that sum of the two pathways, we would have to know one of the internal rates, such as the deaminases or the phosphorylases. We set the flux of adenosine deaminase to 0.01 mM/hr as it a very low flux enzyme based on an earlier model (Joshi, 1990).
5. This assignment sets the flux of \(\textbf{p}\_{2}\) to zero and \(\textbf{p}\_{3}\) to 0.01 mM/hr. We pick the flux through \(\textbf{p}\_{2}\) to be zero since it overlaps with \(\textbf{p}\_{5}\) and gives flux values to all the reactions in the pathways.

With these pathays and numerical values, the steady state flux vector can be computed as the weighted sum of the corresponding basis vectors. The steady state flux vector is computed as an inner product:

```
[13]:
```

```
# Set independent fluxes to determine steady state flux vector
independent_fluxes = {
    ampsn.reactions.SK_amp_c: 0.0,
    ampsn.reactions.SK_ade_c: -0.014,
    ampsn.reactions.ADNK1: 0.12,
    ampsn.reactions.SK_adn_c: -0.01,
    ampsn.reactions.AMPDA: 0.014}
# Compute steady state fluxes
ssfluxes = ampsn.compute_steady_state_fluxes(
    minspan_paths,
    independent_fluxes,
    update_reactions=True)
table_12_8 = pd.DataFrame(list(ssfluxes.values()), index=reaction_ids,
                          columns=[r"$\textbf{v}_{\mathrm{stst}}$"])
```

**Table 12.8:** The steady state flux through the AMP metabolism pathway.

```
[14]:
```

```
table_12_8.T
```

```
[14]:
```

|  | ADNK1 | NTD7 | AMPDA | NTD11 | ADA | PUNP5 | PPM | PRPPS | ADPT | ATPM | SK\_adn\_c | SK\_ade\_c | SK\_ins\_c | SK\_hxan\_c | SK\_nh3\_c | SK\_pi\_c | SK\_amp\_c | SK\_h\_c | SK\_h2o\_c |
| --- | --- | --- | --- | --- | --- | --- | --- | --- | --- | --- | --- | --- | --- | --- | --- | --- | --- | --- | --- |
| $\textbf{v}\_{\mathrm{stst}}$ | 0.120 | 0.120 | 0.014 | 0.014 | 0.010 | 0.014 | 0.014 | 0.014 | 0.014 | 0.148 | -0.010 | -0.014 | 0.010 | 0.014 | 0.024 | 0.000 | 0.000 | 0.000 | -0.024 |

and can be visualized as a bar chart:

```
[15]:
```

```
fig_12_3, ax = plt.subplots(nrows=1, ncols=1, figsize=(10, 5))
# Define indicies for bar chart
indicies = np.arange(len(reaction_ids))+0.5
# Define colors to use
c = plt.cm.coolwarm(np.linspace(0, 1, len(reaction_ids)))
# Plot bar chart
ax.bar(indicies, list(ssfluxes.values()), width=0.8, color=c);
ax.set_xlim([0, len(reaction_ids)]);
# Set labels and adjust ticks
ax.set_xticks(indicies);
ax.set_xticklabels(reaction_ids, rotation="vertical");
ax.set_ylabel("Fluxes (mM/hr)", L_FONT);
ax.set_title("Steady State Fluxes", L_FONT);
# Add a dashed line at 0
ax.plot([0, len(reaction_ids)], [0, 0], "k--");
fig_12_3.tight_layout()
```

**Figure 12.3:** Bar chart of the steady-state fluxes.

We can perform a numerical check make sure that we have a steady state flux vector by performing the multiplication \(\textbf{Sv}\_{\mathrm{stst}}\) that should yield zero.

**A numerical QC/QA:** Ensure \(\textbf{Sv}\_{\mathrm{stst}} = 0\)

```
[16]:
```

```
pd.DataFrame(
    ampsn.S.dot(np.array(list(ssfluxes.values()))),
    index=metabolite_ids,
    columns=[r"$\textbf{Sv}_{\mathrm{stst}}$"],
    dtype=np.int64).T
```

```
[16]:
```

|  | adn\_c | ade\_c | imp\_c | ins\_c | hxan\_c | r1p\_c | r5p\_c | prpp\_c | atp\_c | adp\_c | amp\_c | pi\_c | nh3\_c | h\_c | h2o\_c |
| --- | --- | --- | --- | --- | --- | --- | --- | --- | --- | --- | --- | --- | --- | --- | --- |
| $\textbf{Sv}\_{\mathrm{stst}}$ | 0 | 0 | 0 | 0 | 0 | 0 | 0 | 0 | 0 | 0 | 0 | 0 | 0 | 0 | 0 |

###### Computing the PERCs¶

The approximate steady state values of the metabolites are given above. The mass action ratios can be computed from these steady state concentrations. We can also compute the forward rate constant for the reactions:

```
[17]:
```

```
percs = ampsn.calculate_PERCs(update_reactions=True)
```

**Table 12.9:** AMP Salvage Network enzymes, loads, transport rates, and their abbreviations. For irreversible reactions, the numerical value for the equilibrium constants is \(\infty\), which, for practical reasons, can be set to a finite value.

```
[18]:
```

```
# Get concentration values for substitution into sympy expressions
value_dict = {sym.Symbol(str(met)): ic
              for met, ic in ampsn.initial_conditions.items()}
value_dict.update({sym.Symbol(str(met)): bc
                   for met, bc in ampsn.boundary_conditions.items()})

table_12_9 = []
# Get symbols and values for table and substitution
for p_key in ["Keq", "kf"]:
    symbol_list, value_list = [], []
    for p_str, value in ampsn.parameters[p_key].items():
        symbol_list.append(r"$%s_{\text{%s}}$" % (p_key[0], p_str.split("_", 1)[-1]))
        value_list.append("{0:.3f}".format(value) if value != INF else r"$\infty$")
        value_dict.update({sym.Symbol(p_str): value})
    table_12_9.extend([symbol_list, value_list])

table_12_9.append(["{0:.6f}".format(float(ratio.subs(value_dict)))
                    for ratio in strip_time(ampsn.get_mass_action_ratios()).values()])
table_12_9.append(["{0:.6f}".format(float(ratio.subs(value_dict)))
                    for ratio in strip_time(ampsn.get_disequilibrium_ratios()).values()])
table_12_9 = pd.DataFrame(np.array(table_12_9).T, index=reaction_ids,
                          columns=[r"$K_{eq}$ Symbol", r"$K_{eq}$ Value", "PERC Symbol",
                                   "PERC Value", r"$\Gamma$", r"$\Gamma/K_{eq}$"])
table_12_9
```

```
[18]:
```

|  | $K\_{eq}$ Symbol | $K\_{eq}$ Value | PERC Symbol | PERC Value | $\Gamma$ | $\Gamma/K\_{eq}$ |
| --- | --- | --- | --- | --- | --- | --- |
| ADNK1 | $K\_{\text{ADNK1}}$ | $\infty$ | $k\_{\text{ADNK1}}$ | 62.500 | 13.099557 | 0.000000 |
| NTD7 | $K\_{\text{NTD7}}$ | $\infty$ | $k\_{\text{NTD7}}$ | 1.384 | 0.034591 | 0.000000 |
| AMPDA | $K\_{\text{AMPDA}}$ | $\infty$ | $k\_{\text{AMPDA}}$ | 0.161 | 0.010493 | 0.000000 |
| NTD11 | $K\_{\text{NTD11}}$ | $\infty$ | $k\_{\text{NTD11}}$ | 1.400 | 0.250000 | 0.000000 |
| ADA | $K\_{\text{ADA}}$ | $\infty$ | $k\_{\text{ADA}}$ | 8.333 | 0.075835 | 0.000000 |
| PUNP5 | $K\_{\text{PUNP5}}$ | 0.090 | $k\_{\text{PUNP5}}$ | 12.000 | 0.048000 | 0.533333 |
| PPM | $K\_{\text{PPM}}$ | 13.300 | $k\_{\text{PPM}}$ | 0.235 | 0.082333 | 0.006190 |
| PRPPS | $K\_{\text{PRPPS}}$ | $\infty$ | $k\_{\text{PRPPS}}$ | 1.107 | 0.033251 | 0.000000 |
| ADPT | $K\_{\text{ADPT}}$ | $\infty$ | $k\_{\text{ADPT}}$ | 2800.000 | 108410.125000 | 0.000000 |
| ATPM | $K\_{\text{ATPM}}$ | $\infty$ | $k\_{\text{ATPM}}$ | 0.204 | 2.206897 | 0.000000 |
| SK\_adn\_c | $K\_{\text{SK\_adn\_c}}$ | 1.000 | $k\_{\text{SK\_adn\_c}}$ | 100000.000 | 1.000000 | 1.000000 |
| SK\_ade\_c | $K\_{\text{SK\_ade\_c}}$ | 1.000 | $k\_{\text{SK\_ade\_c}}$ | 100000.000 | 1.000000 | 1.000000 |
| SK\_ins\_c | $K\_{\text{SK\_ins\_c}}$ | 1.000 | $k\_{\text{SK\_ins\_c}}$ | 100000.000 | 1.000000 | 1.000000 |
| SK\_hxan\_c | $K\_{\text{SK\_hxan\_c}}$ | 1.000 | $k\_{\text{SK\_hxan\_c}}$ | 100000.000 | 1.000000 | 1.000000 |
| SK\_nh3\_c | $K\_{\text{SK\_nh3\_c}}$ | 1.000 | $k\_{\text{SK\_nh3\_c}}$ | 100000.000 | 1.000000 | 1.000000 |
| SK\_pi\_c | $K\_{\text{SK\_pi\_c}}$ | 1.000 | $k\_{\text{SK\_pi\_c}}$ | 100000.000 | 1.000000 | 1.000000 |
| SK\_amp\_c | $K\_{\text{SK\_amp\_c}}$ | 1.000 | $k\_{\text{SK\_amp\_c}}$ | 100000.000 | 1.000000 | 1.000000 |
| SK\_h\_c | $K\_{\text{SK\_h\_c}}$ | 1.000 | $k\_{\text{SK\_h\_c}}$ | 100000.000 | 1.000000 | 1.000000 |
| SK\_h2o\_c | $K\_{\text{SK\_h2o\_c}}$ | 1.000 | $k\_{\text{SK\_h2o\_c}}$ | 100000.000 | 1.000000 | 1.000000 |

These estimates for the numerical values for the PERCs are shown in Table 12.9. These numerical values, along with the elementary form of the rate laws, complete the definition of the dynamic mass balances that can now be simulated. The steady state is specified in Table 12.8.

###### Ratios¶

The AMP degradation and biosynthetic pathways determine the amount of AMP and its degradation products present. We thus define the ‘AMP charge,’ calculated as the AMP concentration divided by the concentration of AMP and its degradation. This pool consists of IMP, ADO, INO, R1P, R5P, and PRPP. The AMP charge is

\[\begin{equation} r\_{\text{AMP}} = \frac{\text{AMP}}{\text{AMP} + \text{IMP} + \text{ADO} + \text{INO} + \text{R1P} + \text{R5P} + \text{PRPP}} \end{equation}\]

The AMP charge, \(r\_{AMP}\), is a little over 50% at steady-state calculated below. Thus, about half of the pentose in the system is in the AMP molecule and the other half is in the degradation or biosynthesis products.

###### Dynamic simulation: Increasing AMP concentration¶

The maintenance of AMP at a physiologically meaningful value is determined by the balance of its degradative and biosynthetic pathways. In Chapter 10 we saw that a disturbance in the ATP load on glycolysis leads to AMP exiting the glycolytic system, requiring degradation. Here we are interested in seeing how the balance of the AMP pathways is influenced by a sudden change in the level of AMP.

We thus simulate the response to a sudden 10% increase in AMP concentration as a proxy for a disturbance that increases AMP concentration, such as the simulations performed in the previous chapters.

```
[19]:
```

```
t0, tf = (0, 1000)
sim_ampsn = Simulation(ampsn)
conc_sol, flux_sol = sim_ampsn.simulate(
    ampsn, time=(t0, tf, tf*10 + 1),
    perturbations={"amp_b": "amp_b * 1.1"})

conc_sol.make_aggregate_solution(
    "AMP_Charge_Ratio",
    equation="(amp_c) / (amp_c + adn_c + ade_c + imp_c + ins_c + prpp_c + r1p_c + r5p_c)")

fig_12_4, axes = plt.subplots(nrows=1, ncols=2, figsize=(12, 4))
(ax1, ax2) = axes.flatten()

plot_time_profile(
    conc_sol, observable=ampsn.metabolites, ax=ax1,
    plot_function="loglog",
    xlabel="Time [hr]", ylabel="Concentration [mM]",
    title=("(a)Concentrations", L_FONT));

plot_time_profile(
    conc_sol, observable="AMP_Charge_Ratio", ax=ax2,
    plot_function="semilogx",
    xlabel="Time [hr]", ylabel="Ratio", ylim=(0.4, .6),
    title=("(b) AMP Charge Ratio", L_FONT));
fig_12_3.tight_layout()
```

**Figure 12.4:** (a) The concentrations of the AMP metabolism pathway and (b) The AMP charge after a sudden 10% increase in the AMP concentration at \(t=0\).

The response of the degradation pathways to AMP increase is a rapid conversion of AMP to IMP followed by a slow conversion of IMP to INO that is then rapidly exchanged with plasma, Figure 12.5. This degradation route is kinetically preferred. A sudden decrease in AMP has similar, but opposite responses. If AMP is suddenly reduced in concentration, the flux through this pathway drops.

```
[20]:
```

```
fig_12_5, axes = plt.subplots(nrows=2, ncols=3, figsize=(18, 8))

ylims = [
    [(0.012, 0.017), (0.012, 0.017), (0.008, 0.013)],
    [(0.07, 0.11),(0.0085, 0.0125),(0.000, 0.002)]
]

for i, [rxn, met] in enumerate(zip(["AMPDA", "NTD11", "SK_ins_c"],
                                   ["amp_c", "imp_c", "ins_c"])):
    xlims = (t0, 5/50000) if i == 0 else (t0, 5)
    plot_time_profile(
        flux_sol, observable=rxn, ax=axes[0][i],
        xlim=xlims, ylim=ylims[0][i],
        xlabel="Time [hr]", ylabel="Flux [mM/hr]",
        title=(ampsn.reactions.get_by_id(rxn).name, L_FONT))

    plot_time_profile(
        conc_sol, observable=met, ax=axes[1][i],
        xlim=xlims, ylim=ylims[1][i],
        xlabel="Time (hr)", ylabel="Concentrations [mM]",
        title=(met, L_FONT))
fig_12_5.tight_layout()
```

**Figure 12.5:** The dynamic response of the AMP synthesis/degradation sub-network to a sudden 10% increase in the AMP concentration at \(t=0\). The most notable set of fluxes are shown.

##### Network Integration¶

The AMP metabolic sub-network of Figure 12.1 can be integrated with the glycolysis and pentose pathway network from the last chapter. The result is shown in Figure 12.6. This integrated network represents the core of the metabolic network in the red blood cell, the simplest cell in the human body.

**Figure 12.6:** The core metabolic network in the human red blood cell comprised of glycolysis, the pentose pathway, and adenine nucleotide metabolism. Some of the integration issues discussed in the text are highlighted with dashed ovals.

###### Integration issues¶

Given the many points of contact created between the AMP sub-network and the combined glycolytic and pentose pathway network, there are a few interesting integration issues. They are highlighted with dashed ovals in Figure 12.6 and are as follows:

- The AMP molecule in the two networks connects the two. These two nodes need to be merged into one.
- The R5P molecule appears in both the AMP metabolic subnetwork and the pentose pathway, so these two nodes also need to be merged.
- In the sub-network described above, the stoichiometry of the PRPP synthase reaction is

  \[\begin{equation} \text{R5P} + 2\text{ATP} \rightarrow \text{PRPP} + 2\text{ADP} + \text{H} \tag{12.2} \end{equation}\]

  but in actuality it is

  \[\begin{equation} \text{R5P} + \text{ATP} \rightarrow \text{PRPP} + \text{AMP} + \text{H} \tag{12.3} \end{equation}\]

  this difference disappears since the ApK reaction is in the combined glycolytic and pentose pathway network:

  \[\begin{equation} \text{AMP} + \text{ATP} \leftrightharpoons 2\text{ADP} \tag{12.4} \end{equation}\]

  i.e., if Equations (12.3) and (12.4) are added, one gets Eq. (12.2).
- The ATP cost of driving the biosynthetic pathways to AMP is now a part of the ATP load in the integrated model.
- The AMP exchange reaction disappears, and instead we now have exchange reactions for ADO, ADE, INO, and HYP, and the deamination reactions create an exchange flux for \(\text{NH}\_3\).

###### Merging the models¶

Just as in the last chapter, we start by loading all the models, and then combine them:

```
[21]:
```

```
glycolysis = create_test_model("SB2_Glycolysis")
ppp = create_test_model("SB2_PentosePhosphatePathway")
ampsn = create_test_model("SB2_AMPSalvageNetwork")

fullppp = glycolysis.merge(ppp, inplace=False)
fullppp.id = "Full_PPP"
fullppp.remove_reactions([
    r for r in fullppp.boundary
    if r.id in ["SK_g6p_c", "DM_f6p_c", "DM_g3p_c", "DM_r5p_c"]])
fullppp.remove_boundary_conditions(["g6p_b", "f6p_b", "g3p_b", "r5p_b"])
core_network = fullppp.merge(ampsn, inplace=False)
core_network.id = "Core_Model"
```

```
Ignoring reaction 'SK_h_c' since it already exists.
Ignoring reaction 'SK_h2o_c' since it already exists.
Ignoring reaction 'ATPM' since it already exists.
Ignoring reaction 'SK_amp_c' since it already exists.
Ignoring reaction 'SK_h_c' since it already exists.
Ignoring reaction 'SK_h2o_c' since it already exists.
```

Then a few obsolete exchange reactions have to be removed.

```
[22]:
```

```
for boundary in core_network.boundary:
    print(boundary)
```

```
DM_amp_c: amp_c -->
SK_pyr_c: pyr_c <=>
SK_lac__L_c: lac__L_c <=>
SK_glc__D_c:  <=> glc__D_c
SK_amp_c:  <=> amp_c
SK_h_c: h_c <=>
SK_h2o_c: h2o_c <=>
SK_co2_c: co2_c <=>
SK_adn_c: adn_c <=>
SK_ade_c: ade_c <=>
SK_ins_c: ins_c <=>
SK_hxan_c: hxan_c <=>
SK_nh3_c: nh3_c <=>
SK_pi_c: pi_c <=>
```

```
[23]:
```

```
core_network.remove_reactions([
    r for r in core_network.boundary
    if r.id in ["DM_amp_c", "SK_amp_c"]])
core_network.remove_boundary_conditions(["amp_b"])
```

We then need to change the PRPP synthesis reaction to have the correct stoichiometry and PERC:

```
[24]:
```

```
# Note that reactants have negative coefficients and products have positive coefficients
core_network.reactions.PRPPS.subtract_metabolites({
    core_network.metabolites.atp_c: -1,
    core_network.metabolites.adp_c: 2})
core_network.reactions.PRPPS.add_metabolites({
    core_network.metabolites.amp_c: 1})
```

The merged model contains 40 metabolites and 45 reactions:

```
[25]:
```

```
print(core_network.S.shape)
```

```
(40, 45)
```

Note that the merged model is not in a steady-state:

```
[26]:
```

```
t0, tf = (0, 1e3)
sim_core = Simulation(core_network)
conc_sol, flux_sol = sim_core.simulate(
    core_network, time=(t0, tf, tf*10 + 1))

fig, axes = plt.subplots(nrows=1, ncols=2, figsize=(12, 10))
(ax1, ax2) = axes.flatten()

plot_time_profile(
    conc_sol, ax=ax1, legend="lower outside",
    plot_function="loglog", xlabel="Time [hr]",
    ylabel="Concentration [mM]", title=("Concentrations", L_FONT));

plot_time_profile(
    flux_sol, ax=ax2, legend="lower outside",
    plot_function="semilogx", xlabel="Time [hr]",
    ylabel="Flux [mM/hr]", title=("Fluxes", L_FONT));
fig.tight_layout()
```

###### Organization of the stoichiometric matrix¶

As in the previous chapter, we can perform a set of transformations to group the species and reactions into organized groups.

```
[27]:
```

```
# Define new order for metabolites
new_metabolite_order = [
    "glc__D_c", "g6p_c", "f6p_c", "fdp_c", "dhap_c",
    "g3p_c", "_13dpg_c", "_3pg_c", "_2pg_c", "pep_c",
    "pyr_c", "lac__L_c", "_6pgl_c", "_6pgc_c", "ru5p__D_c",
    "xu5p__D_c", "r5p_c", "s7p_c", "e4p_c", "ade_c", "adn_c",
    "imp_c", "ins_c", "hxan_c", "r1p_c", "prpp_c", "nad_c",
    "nadh_c",  "amp_c", "adp_c", "atp_c", "nadp_c", "nadph_c",
    "gthrd_c", "gthox_c", "pi_c", "h_c", "h2o_c", "co2_c", "nh3_c"]
if len(core_network.metabolites) == len(new_metabolite_order):
    core_network.metabolites = DictList(core_network.metabolites.get_by_any(new_metabolite_order))
# Define new order for reactions
new_reaction_order = [
    "HEX1", "PGI", "PFK", "FBA", "TPI", "GAPD", "PGK", "PGM",
    "ENO", "PYK", "LDH_L", "G6PDH2r", "PGL", "GND", "RPE",
    "RPI", "TKT1", "TKT2", "TALA", "ADNK1", "NTD7", "ADA","AMPDA",
    "NTD11", "PUNP5", "PPM", "PRPPS", "ADPT", "ADK1",
    "ATPM", "DM_nadh", "GTHOr", "GSHR", "SK_glc__D_c", "SK_pyr_c",
    "SK_lac__L_c", "SK_ade_c", "SK_adn_c", "SK_ins_c", "SK_hxan_c",
    "SK_pi_c", "SK_h_c", "SK_h2o_c", "SK_co2_c", "SK_nh3_c"]
if len(core_network.reactions) == len(new_reaction_order):
    core_network.reactions = DictList(core_network.reactions.get_by_any(new_reaction_order))
```

The stoichiometric matrix for the merged core network model is shown in Table 11.11. This matrix has dimensions of 40x45 and its rank is 37. The null is of dimension 8 (=45-37) and the left null space is of dimension 3 (=40-37). The matrix is elementally balanced.

We have used colors in Table 12.10 to illustrate the organized structure of the matrix.

**Table 12.10:** The stoichiometric matrix for the merged core network model in Figure 12.6. The matrix is partitioned to show the glycolytic reactions (yellow) separate from the pentose phosphate pathway (light blue) and the AMP salvage network (light green). The cofactors (light orange) and inorganics (pink) are also grouped and shown. The connectivities, \(\rho\_i\) (red), for a compound, and the participation number, \(\pi\_j\) (cyan), for a reaction are shown. The second block in the
table is the product \(\textbf{ES}\) (blue) to evaluate elemental balancing status of the reactions. All exchange reactions have a participation number of unity and are thus not elementally balanced. The last block in the table has the 8 pathway vectors (purple) for the merged model. These vectors are graphically shown in Figure 12.7. Furthest to the right, we display the time invariant pools (green) that span the left null space.

```
[28]:
```

```
# Define labels
metabolite_ids = [m.id for m in core_network.metabolites]
reaction_ids = [r.id for r in core_network.reactions]

pi_str = r"$\pi_{j}$"
rho_str = r"$\rho_{i}$"
chopsnq = ['C', 'H', 'O', 'P', 'N', 'S', 'q', '[NAD]', '[NADP]']
time_inv_labels = [
    "$N_{\mathrm{tot}}$", "$NP_{\mathrm{tot}}$", "$G_{\mathrm{tot}}$"]
path_labels = ["$p_1$", "$p_2$", "$p_3$", "$p_4$",
               "$p_5$", "$p_6$", "$p_7$", "$p_8$"]

# Make table content from the stoichiometric matrix, elemental balancing of pathways
# participation number, and MinSpan pathways
S_matrix = core_network.update_S(array_type="dense", dtype=np.int64, update_model=False)
ES_matrix = core_network.get_elemental_charge_balancing(dtype=np.int64)
pi = np.count_nonzero(S_matrix, axis=0)
rho = np.count_nonzero(S_matrix, axis=1)
minspan_paths = np.array([
    [1, 1, 1, 1, 1, 2, 2, 2, 2, 2, 2, 0, 0, 0, 0, 0, 0, 0, 0, 0, 0, 0, 0, 0, 0, 0, 0, 0, 0, 2, 0, 0, 0, 1, 0, 2, 0, 0, 0, 0, 0, 2, 0, 0, 0],
    [0, 0, 0, 0, 0, 0, 0, 0, 0, 0, 1, 0, 0, 0, 0, 0, 0, 0, 0, 0, 0, 0, 0, 0, 0, 0, 0, 0, 0, 0,-1, 0, 0, 0,-1, 1, 0, 0, 0, 0, 0,-2, 0, 0, 0],
    [1,-2, 0, 0, 0, 1, 1, 1, 1, 1, 1, 3, 3, 3, 2, 1, 1, 1, 1, 0, 0, 0, 0, 0, 0, 0, 0, 0, 0, 1, 0, 6, 6, 1, 0, 1, 0, 0, 0, 0, 0,13,-3, 3, 0],
    [1, 0, 0, 0, 0, 0, 0, 0, 0, 0, 0, 1, 1, 1, 0, 1, 0, 0, 0, 0, 1, 0, 0, 0, 0, 0, 1, 1,-1,-3, 0, 2, 2, 1, 0, 0,-1, 1, 0, 0, 0, 4, 0, 1, 0],
    [1, 0, 0, 0, 0, 0, 0, 0, 0, 0, 0, 1, 1, 1, 0, 1, 0, 0, 0, 0, 1, 1, 0, 0, 0, 0, 1, 1,-1,-3, 0, 2, 2, 1, 0, 0,-1, 0, 1, 0, 0, 4,-1, 1, 1],
    [1, 0, 0, 0, 0, 0, 0, 0, 0, 0, 0, 1, 1, 1, 0, 1, 0, 0, 0, 0, 0, 0, 1, 1, 0, 0, 1, 1,-1,-3, 0, 2, 2, 1, 0, 0,-1, 0, 1, 0, 0, 4,-1, 1, 1],
    [0, 0, 0, 0, 0, 0, 0, 0, 0, 0, 0, 0, 0, 0, 0, 0, 0, 0, 0, 0, 1, 1, 0, 0, 1, 1, 1, 1,-1,-2, 0, 0, 0, 0, 0, 0,-1, 0, 0, 1, 0, 0,-1, 0, 1],
    [0, 0, 0, 0, 0, 0, 0, 0, 0, 0, 0, 0, 0, 0, 0, 0, 0, 0, 0, 1, 1, 0, 0, 0, 0, 0, 0, 0, 0,-1, 0, 0, 0, 0, 0, 0, 0, 0, 0, 0, 0, 0, 0, 0, 0],
])
table_12_10 = np.vstack((S_matrix, pi, ES_matrix, minspan_paths))

# Determine number of blank entries needed to be added to pad the table,
# Add connectivity number and time invariants to table content
blanks = [""]*(len(table_12_10) - len(metabolite_ids))
rho = np.concatenate((rho, blanks))

lns = np.zeros((3, 40), dtype=np.int64)
lns[0][26:28] = 1
lns[1][31:33] = 1
lns[2][33] = 1
lns[2][34] = 2

time_inv = np.array([np.concatenate([row, blanks]) for row in lns])
table_12_10 = np.vstack([table_12_10.T, rho, time_inv]).T

colors = {"glycolysis": "#ffffe6",    # Yellow
          "ppp": "#e6faff",           # Light blue
          "ampsn": "#d9fad2",
          "cofactor": "#ffe6cc",
          "inorganic": "#fadffa",
          "chopsnq": "#99e6ff",       # Blue
          "pathways": "#b399ff",      # Purple
          "pi": "#99ffff",            # Cyan
          "rho": "#ff9999",           # Red
          "time_invs": "#ccff99",     # Green
          "blank": "#f2f2f2"}         # Grey
bg_color_str = "background-color: "
cofactor_mets = ["nad_c", "nadh_c",  "amp_c", "adp_c", "atp_c",
                 "nadp_c", "nadph_c", "gthrd_c", "gthox_c"]
exch_misc_rxns= ["S_glc__D_e", "EX_pyr_e", "EX_lac__L_e", "EX_ade_e", "EX_adn_e",
                 "EX_ins_e", "EX_hxan_e", "ATPM", "DM_nadh", "GTHOr", "GSHR"]
inorganic_mets = ["pi_c", "h_c", "h2o_c", "co2_c", "nh3_c"]
inorganic_exch = ["EX_pi_e", "EX_h_e", "EX_h2o_e", "EX_co2_e", "EX_nh3_e"]

def highlight_table(df, model):
    df = df.copy()
    condition = lambda mmodel, row, col, c1, c2:  (
        (col not in exch_misc_rxns + inorganic_exch) and (row not in cofactor_mets + inorganic_mets) and (
            (row in mmodel.metabolites and c1) or (col in mmodel.reactions or c2)))
    inorganic_condition = lambda row, col: (col in inorganic_exch or row in inorganic_mets)
    for i, row in enumerate(df.index):
        for j, col in enumerate(df.columns):
            if df.loc[row, col] == "":
                main_key = "blank"
            elif row in pi_str:
                main_key = "pi"
            elif row in chopsnq:
                main_key = "chopsnq"
            elif row in path_labels:
                main_key = "pathways"
            elif col in rho_str:
                main_key = "rho"
            elif col in time_inv_labels:
                main_key = "time_invs"
            elif condition(ampsn, row, col, row not in ["r5p_c"], col in ["ADK1"]):
                main_key = "ampsn"
            elif condition(ppp, row, col, row not in ["g6p_c", "f6p_c", "g3p_c"], False):
                main_key = "ppp"
            elif condition(glycolysis, row, col, True, False):
                main_key = "glycolysis"
            elif ((col in exch_misc_rxns or row in cofactor_mets) and not inorganic_condition(row, col)):
                main_key = "cofactor"
            elif inorganic_condition(row, col):
                main_key = "inorganic"
            else:
                continue
            df.loc[row, col] = bg_color_str + colors[main_key]
    return df

# Create index and column labels
index_labels = np.concatenate((metabolite_ids, [pi_str], chopsnq, path_labels))
column_labels = np.concatenate((reaction_ids, [rho_str], time_inv_labels))
# Create DataFrame
table_12_10 = pd.DataFrame(
    table_12_10, index=index_labels, columns=column_labels)
# Apply colors
table_12_10 = table_12_10.style.apply(
    highlight_table,  model=core_network, axis=None)
table_12_10
```

```
[28]:
```

|  | HEX1 | PGI | PFK | FBA | TPI | GAPD | PGK | PGM | ENO | PYK | LDH\_L | G6PDH2r | PGL | GND | RPE | RPI | TKT1 | TKT2 | TALA | ADNK1 | NTD7 | ADA | AMPDA | NTD11 | PUNP5 | PPM | PRPPS | ADPT | ADK1 | ATPM | DM\_nadh | GTHOr | GSHR | SK\_glc\_\_D\_c | SK\_pyr\_c | SK\_lac\_\_L\_c | SK\_ade\_c | SK\_adn\_c | SK\_ins\_c | SK\_hxan\_c | SK\_pi\_c | SK\_h\_c | SK\_h2o\_c | SK\_co2\_c | SK\_nh3\_c | $\rho\_{i}$ | $N\_{\mathrm{tot}}$ | $NP\_{\mathrm{tot}}$ | $G\_{\mathrm{tot}}$ |
| --- | --- | --- | --- | --- | --- | --- | --- | --- | --- | --- | --- | --- | --- | --- | --- | --- | --- | --- | --- | --- | --- | --- | --- | --- | --- | --- | --- | --- | --- | --- | --- | --- | --- | --- | --- | --- | --- | --- | --- | --- | --- | --- | --- | --- | --- | --- | --- | --- | --- |
| glc\_\_D\_c | -1 | 0 | 0 | 0 | 0 | 0 | 0 | 0 | 0 | 0 | 0 | 0 | 0 | 0 | 0 | 0 | 0 | 0 | 0 | 0 | 0 | 0 | 0 | 0 | 0 | 0 | 0 | 0 | 0 | 0 | 0 | 0 | 0 | 1 | 0 | 0 | 0 | 0 | 0 | 0 | 0 | 0 | 0 | 0 | 0 | 2 | 0 | 0 | 0 |
| g6p\_c | 1 | -1 | 0 | 0 | 0 | 0 | 0 | 0 | 0 | 0 | 0 | -1 | 0 | 0 | 0 | 0 | 0 | 0 | 0 | 0 | 0 | 0 | 0 | 0 | 0 | 0 | 0 | 0 | 0 | 0 | 0 | 0 | 0 | 0 | 0 | 0 | 0 | 0 | 0 | 0 | 0 | 0 | 0 | 0 | 0 | 3 | 0 | 0 | 0 |
| f6p\_c | 0 | 1 | -1 | 0 | 0 | 0 | 0 | 0 | 0 | 0 | 0 | 0 | 0 | 0 | 0 | 0 | 0 | 1 | 1 | 0 | 0 | 0 | 0 | 0 | 0 | 0 | 0 | 0 | 0 | 0 | 0 | 0 | 0 | 0 | 0 | 0 | 0 | 0 | 0 | 0 | 0 | 0 | 0 | 0 | 0 | 4 | 0 | 0 | 0 |
| fdp\_c | 0 | 0 | 1 | -1 | 0 | 0 | 0 | 0 | 0 | 0 | 0 | 0 | 0 | 0 | 0 | 0 | 0 | 0 | 0 | 0 | 0 | 0 | 0 | 0 | 0 | 0 | 0 | 0 | 0 | 0 | 0 | 0 | 0 | 0 | 0 | 0 | 0 | 0 | 0 | 0 | 0 | 0 | 0 | 0 | 0 | 2 | 0 | 0 | 0 |
| dhap\_c | 0 | 0 | 0 | 1 | -1 | 0 | 0 | 0 | 0 | 0 | 0 | 0 | 0 | 0 | 0 | 0 | 0 | 0 | 0 | 0 | 0 | 0 | 0 | 0 | 0 | 0 | 0 | 0 | 0 | 0 | 0 | 0 | 0 | 0 | 0 | 0 | 0 | 0 | 0 | 0 | 0 | 0 | 0 | 0 | 0 | 2 | 0 | 0 | 0 |
| g3p\_c | 0 | 0 | 0 | 1 | 1 | -1 | 0 | 0 | 0 | 0 | 0 | 0 | 0 | 0 | 0 | 0 | 1 | 1 | -1 | 0 | 0 | 0 | 0 | 0 | 0 | 0 | 0 | 0 | 0 | 0 | 0 | 0 | 0 | 0 | 0 | 0 | 0 | 0 | 0 | 0 | 0 | 0 | 0 | 0 | 0 | 6 | 0 | 0 | 0 |
| \_13dpg\_c | 0 | 0 | 0 | 0 | 0 | 1 | -1 | 0 | 0 | 0 | 0 | 0 | 0 | 0 | 0 | 0 | 0 | 0 | 0 | 0 | 0 | 0 | 0 | 0 | 0 | 0 | 0 | 0 | 0 | 0 | 0 | 0 | 0 | 0 | 0 | 0 | 0 | 0 | 0 | 0 | 0 | 0 | 0 | 0 | 0 | 2 | 0 | 0 | 0 |
| \_3pg\_c | 0 | 0 | 0 | 0 | 0 | 0 | 1 | -1 | 0 | 0 | 0 | 0 | 0 | 0 | 0 | 0 | 0 | 0 | 0 | 0 | 0 | 0 | 0 | 0 | 0 | 0 | 0 | 0 | 0 | 0 | 0 | 0 | 0 | 0 | 0 | 0 | 0 | 0 | 0 | 0 | 0 | 0 | 0 | 0 | 0 | 2 | 0 | 0 | 0 |
| \_2pg\_c | 0 | 0 | 0 | 0 | 0 | 0 | 0 | 1 | -1 | 0 | 0 | 0 | 0 | 0 | 0 | 0 | 0 | 0 | 0 | 0 | 0 | 0 | 0 | 0 | 0 | 0 | 0 | 0 | 0 | 0 | 0 | 0 | 0 | 0 | 0 | 0 | 0 | 0 | 0 | 0 | 0 | 0 | 0 | 0 | 0 | 2 | 0 | 0 | 0 |
| pep\_c | 0 | 0 | 0 | 0 | 0 | 0 | 0 | 0 | 1 | -1 | 0 | 0 | 0 | 0 | 0 | 0 | 0 | 0 | 0 | 0 | 0 | 0 | 0 | 0 | 0 | 0 | 0 | 0 | 0 | 0 | 0 | 0 | 0 | 0 | 0 | 0 | 0 | 0 | 0 | 0 | 0 | 0 | 0 | 0 | 0 | 2 | 0 | 0 | 0 |
| pyr\_c | 0 | 0 | 0 | 0 | 0 | 0 | 0 | 0 | 0 | 1 | -1 | 0 | 0 | 0 | 0 | 0 | 0 | 0 | 0 | 0 | 0 | 0 | 0 | 0 | 0 | 0 | 0 | 0 | 0 | 0 | 0 | 0 | 0 | 0 | -1 | 0 | 0 | 0 | 0 | 0 | 0 | 0 | 0 | 0 | 0 | 3 | 0 | 0 | 0 |
| lac\_\_L\_c | 0 | 0 | 0 | 0 | 0 | 0 | 0 | 0 | 0 | 0 | 1 | 0 | 0 | 0 | 0 | 0 | 0 | 0 | 0 | 0 | 0 | 0 | 0 | 0 | 0 | 0 | 0 | 0 | 0 | 0 | 0 | 0 | 0 | 0 | 0 | -1 | 0 | 0 | 0 | 0 | 0 | 0 | 0 | 0 | 0 | 2 | 0 | 0 | 0 |
| \_6pgl\_c | 0 | 0 | 0 | 0 | 0 | 0 | 0 | 0 | 0 | 0 | 0 | 1 | -1 | 0 | 0 | 0 | 0 | 0 | 0 | 0 | 0 | 0 | 0 | 0 | 0 | 0 | 0 | 0 | 0 | 0 | 0 | 0 | 0 | 0 | 0 | 0 | 0 | 0 | 0 | 0 | 0 | 0 | 0 | 0 | 0 | 2 | 0 | 0 | 0 |
| \_6pgc\_c | 0 | 0 | 0 | 0 | 0 | 0 | 0 | 0 | 0 | 0 | 0 | 0 | 1 | -1 | 0 | 0 | 0 | 0 | 0 | 0 | 0 | 0 | 0 | 0 | 0 | 0 | 0 | 0 | 0 | 0 | 0 | 0 | 0 | 0 | 0 | 0 | 0 | 0 | 0 | 0 | 0 | 0 | 0 | 0 | 0 | 2 | 0 | 0 | 0 |
| ru5p\_\_D\_c | 0 | 0 | 0 | 0 | 0 | 0 | 0 | 0 | 0 | 0 | 0 | 0 | 0 | 1 | -1 | -1 | 0 | 0 | 0 | 0 | 0 | 0 | 0 | 0 | 0 | 0 | 0 | 0 | 0 | 0 | 0 | 0 | 0 | 0 | 0 | 0 | 0 | 0 | 0 | 0 | 0 | 0 | 0 | 0 | 0 | 3 | 0 | 0 | 0 |
| xu5p\_\_D\_c | 0 | 0 | 0 | 0 | 0 | 0 | 0 | 0 | 0 | 0 | 0 | 0 | 0 | 0 | 1 | 0 | -1 | -1 | 0 | 0 | 0 | 0 | 0 | 0 | 0 | 0 | 0 | 0 | 0 | 0 | 0 | 0 | 0 | 0 | 0 | 0 | 0 | 0 | 0 | 0 | 0 | 0 | 0 | 0 | 0 | 3 | 0 | 0 | 0 |
| r5p\_c | 0 | 0 | 0 | 0 | 0 | 0 | 0 | 0 | 0 | 0 | 0 | 0 | 0 | 0 | 0 | 1 | -1 | 0 | 0 | 0 | 0 | 0 | 0 | 0 | 0 | 1 | -1 | 0 | 0 | 0 | 0 | 0 | 0 | 0 | 0 | 0 | 0 | 0 | 0 | 0 | 0 | 0 | 0 | 0 | 0 | 4 | 0 | 0 | 0 |
| s7p\_c | 0 | 0 | 0 | 0 | 0 | 0 | 0 | 0 | 0 | 0 | 0 | 0 | 0 | 0 | 0 | 0 | 1 | 0 | -1 | 0 | 0 | 0 | 0 | 0 | 0 | 0 | 0 | 0 | 0 | 0 | 0 | 0 | 0 | 0 | 0 | 0 | 0 | 0 | 0 | 0 | 0 | 0 | 0 | 0 | 0 | 2 | 0 | 0 | 0 |
| e4p\_c | 0 | 0 | 0 | 0 | 0 | 0 | 0 | 0 | 0 | 0 | 0 | 0 | 0 | 0 | 0 | 0 | 0 | -1 | 1 | 0 | 0 | 0 | 0 | 0 | 0 | 0 | 0 | 0 | 0 | 0 | 0 | 0 | 0 | 0 | 0 | 0 | 0 | 0 | 0 | 0 | 0 | 0 | 0 | 0 | 0 | 2 | 0 | 0 | 0 |
| ade\_c | 0 | 0 | 0 | 0 | 0 | 0 | 0 | 0 | 0 | 0 | 0 | 0 | 0 | 0 | 0 | 0 | 0 | 0 | 0 | 0 | 0 | 0 | 0 | 0 | 0 | 0 | 0 | -1 | 0 | 0 | 0 | 0 | 0 | 0 | 0 | 0 | -1 | 0 | 0 | 0 | 0 | 0 | 0 | 0 | 0 | 2 | 0 | 0 | 0 |
| adn\_c | 0 | 0 | 0 | 0 | 0 | 0 | 0 | 0 | 0 | 0 | 0 | 0 | 0 | 0 | 0 | 0 | 0 | 0 | 0 | -1 | 1 | -1 | 0 | 0 | 0 | 0 | 0 | 0 | 0 | 0 | 0 | 0 | 0 | 0 | 0 | 0 | 0 | -1 | 0 | 0 | 0 | 0 | 0 | 0 | 0 | 4 | 0 | 0 | 0 |
| imp\_c | 0 | 0 | 0 | 0 | 0 | 0 | 0 | 0 | 0 | 0 | 0 | 0 | 0 | 0 | 0 | 0 | 0 | 0 | 0 | 0 | 0 | 0 | 1 | -1 | 0 | 0 | 0 | 0 | 0 | 0 | 0 | 0 | 0 | 0 | 0 | 0 | 0 | 0 | 0 | 0 | 0 | 0 | 0 | 0 | 0 | 2 | 0 | 0 | 0 |
| ins\_c | 0 | 0 | 0 | 0 | 0 | 0 | 0 | 0 | 0 | 0 | 0 | 0 | 0 | 0 | 0 | 0 | 0 | 0 | 0 | 0 | 0 | 1 | 0 | 1 | -1 | 0 | 0 | 0 | 0 | 0 | 0 | 0 | 0 | 0 | 0 | 0 | 0 | 0 | -1 | 0 | 0 | 0 | 0 | 0 | 0 | 4 | 0 | 0 | 0 |
| hxan\_c | 0 | 0 | 0 | 0 | 0 | 0 | 0 | 0 | 0 | 0 | 0 | 0 | 0 | 0 | 0 | 0 | 0 | 0 | 0 | 0 | 0 | 0 | 0 | 0 | 1 | 0 | 0 | 0 | 0 | 0 | 0 | 0 | 0 | 0 | 0 | 0 | 0 | 0 | 0 | -1 | 0 | 0 | 0 | 0 | 0 | 2 | 0 | 0 | 0 |
| r1p\_c | 0 | 0 | 0 | 0 | 0 | 0 | 0 | 0 | 0 | 0 | 0 | 0 | 0 | 0 | 0 | 0 | 0 | 0 | 0 | 0 | 0 | 0 | 0 | 0 | 1 | -1 | 0 | 0 | 0 | 0 | 0 | 0 | 0 | 0 | 0 | 0 | 0 | 0 | 0 | 0 | 0 | 0 | 0 | 0 | 0 | 2 | 0 | 0 | 0 |
| prpp\_c | 0 | 0 | 0 | 0 | 0 | 0 | 0 | 0 | 0 | 0 | 0 | 0 | 0 | 0 | 0 | 0 | 0 | 0 | 0 | 0 | 0 | 0 | 0 | 0 | 0 | 0 | 1 | -1 | 0 | 0 | 0 | 0 | 0 | 0 | 0 | 0 | 0 | 0 | 0 | 0 | 0 | 0 | 0 | 0 | 0 | 2 | 0 | 0 | 0 |
| nad\_c | 0 | 0 | 0 | 0 | 0 | -1 | 0 | 0 | 0 | 0 | 1 | 0 | 0 | 0 | 0 | 0 | 0 | 0 | 0 | 0 | 0 | 0 | 0 | 0 | 0 | 0 | 0 | 0 | 0 | 0 | 1 | 0 | 0 | 0 | 0 | 0 | 0 | 0 | 0 | 0 | 0 | 0 | 0 | 0 | 0 | 3 | 1 | 0 | 0 |
| nadh\_c | 0 | 0 | 0 | 0 | 0 | 1 | 0 | 0 | 0 | 0 | -1 | 0 | 0 | 0 | 0 | 0 | 0 | 0 | 0 | 0 | 0 | 0 | 0 | 0 | 0 | 0 | 0 | 0 | 0 | 0 | -1 | 0 | 0 | 0 | 0 | 0 | 0 | 0 | 0 | 0 | 0 | 0 | 0 | 0 | 0 | 3 | 1 | 0 | 0 |
| amp\_c | 0 | 0 | 0 | 0 | 0 | 0 | 0 | 0 | 0 | 0 | 0 | 0 | 0 | 0 | 0 | 0 | 0 | 0 | 0 | 1 | -1 | 0 | -1 | 0 | 0 | 0 | 1 | 1 | 1 | 0 | 0 | 0 | 0 | 0 | 0 | 0 | 0 | 0 | 0 | 0 | 0 | 0 | 0 | 0 | 0 | 6 | 0 | 0 | 0 |
| adp\_c | 1 | 0 | 1 | 0 | 0 | 0 | -1 | 0 | 0 | -1 | 0 | 0 | 0 | 0 | 0 | 0 | 0 | 0 | 0 | 1 | 0 | 0 | 0 | 0 | 0 | 0 | 0 | 0 | -2 | 1 | 0 | 0 | 0 | 0 | 0 | 0 | 0 | 0 | 0 | 0 | 0 | 0 | 0 | 0 | 0 | 7 | 0 | 0 | 0 |
| atp\_c | -1 | 0 | -1 | 0 | 0 | 0 | 1 | 0 | 0 | 1 | 0 | 0 | 0 | 0 | 0 | 0 | 0 | 0 | 0 | -1 | 0 | 0 | 0 | 0 | 0 | 0 | -1 | 0 | 1 | -1 | 0 | 0 | 0 | 0 | 0 | 0 | 0 | 0 | 0 | 0 | 0 | 0 | 0 | 0 | 0 | 8 | 0 | 0 | 0 |
| nadp\_c | 0 | 0 | 0 | 0 | 0 | 0 | 0 | 0 | 0 | 0 | 0 | -1 | 0 | -1 | 0 | 0 | 0 | 0 | 0 | 0 | 0 | 0 | 0 | 0 | 0 | 0 | 0 | 0 | 0 | 0 | 0 | 1 | 0 | 0 | 0 | 0 | 0 | 0 | 0 | 0 | 0 | 0 | 0 | 0 | 0 | 3 | 0 | 1 | 0 |
| nadph\_c | 0 | 0 | 0 | 0 | 0 | 0 | 0 | 0 | 0 | 0 | 0 | 1 | 0 | 1 | 0 | 0 | 0 | 0 | 0 | 0 | 0 | 0 | 0 | 0 | 0 | 0 | 0 | 0 | 0 | 0 | 0 | -1 | 0 | 0 | 0 | 0 | 0 | 0 | 0 | 0 | 0 | 0 | 0 | 0 | 0 | 3 | 0 | 1 | 0 |
| gthrd\_c | 0 | 0 | 0 | 0 | 0 | 0 | 0 | 0 | 0 | 0 | 0 | 0 | 0 | 0 | 0 | 0 | 0 | 0 | 0 | 0 | 0 | 0 | 0 | 0 | 0 | 0 | 0 | 0 | 0 | 0 | 0 | 2 | -2 | 0 | 0 | 0 | 0 | 0 | 0 | 0 | 0 | 0 | 0 | 0 | 0 | 2 | 0 | 0 | 1 |
| gthox\_c | 0 | 0 | 0 | 0 | 0 | 0 | 0 | 0 | 0 | 0 | 0 | 0 | 0 | 0 | 0 | 0 | 0 | 0 | 0 | 0 | 0 | 0 | 0 | 0 | 0 | 0 | 0 | 0 | 0 | 0 | 0 | -1 | 1 | 0 | 0 | 0 | 0 | 0 | 0 | 0 | 0 | 0 | 0 | 0 | 0 | 2 | 0 | 0 | 2 |
| pi\_c | 0 | 0 | 0 | 0 | 0 | -1 | 0 | 0 | 0 | 0 | 0 | 0 | 0 | 0 | 0 | 0 | 0 | 0 | 0 | 0 | 1 | 0 | 0 | 1 | -1 | 0 | 0 | 2 | 0 | 1 | 0 | 0 | 0 | 0 | 0 | 0 | 0 | 0 | 0 | 0 | -1 | 0 | 0 | 0 | 0 | 7 | 0 | 0 | 0 |
| h\_c | 1 | 0 | 1 | 0 | 0 | 1 | 0 | 0 | 0 | -1 | -1 | 1 | 1 | 0 | 0 | 0 | 0 | 0 | 0 | 1 | 0 | 0 | 0 | 0 | 0 | 0 | 1 | 1 | 0 | 1 | 1 | -1 | 2 | 0 | 0 | 0 | 0 | 0 | 0 | 0 | 0 | -1 | 0 | 0 | 0 | 15 | 0 | 0 | 0 |
| h2o\_c | 0 | 0 | 0 | 0 | 0 | 0 | 0 | 0 | 1 | 0 | 0 | 0 | -1 | 0 | 0 | 0 | 0 | 0 | 0 | 0 | -1 | -1 | -1 | -1 | 0 | 0 | 0 | -1 | 0 | -1 | 0 | 0 | 0 | 0 | 0 | 0 | 0 | 0 | 0 | 0 | 0 | 0 | -1 | 0 | 0 | 9 | 0 | 0 | 0 |
| co2\_c | 0 | 0 | 0 | 0 | 0 | 0 | 0 | 0 | 0 | 0 | 0 | 0 | 0 | 1 | 0 | 0 | 0 | 0 | 0 | 0 | 0 | 0 | 0 | 0 | 0 | 0 | 0 | 0 | 0 | 0 | 0 | 0 | 0 | 0 | 0 | 0 | 0 | 0 | 0 | 0 | 0 | 0 | 0 | -1 | 0 | 2 | 0 | 0 | 0 |
| nh3\_c | 0 | 0 | 0 | 0 | 0 | 0 | 0 | 0 | 0 | 0 | 0 | 0 | 0 | 0 | 0 | 0 | 0 | 0 | 0 | 0 | 0 | 1 | 1 | 0 | 0 | 0 | 0 | 0 | 0 | 0 | 0 | 0 | 0 | 0 | 0 | 0 | 0 | 0 | 0 | 0 | 0 | 0 | 0 | 0 | -1 | 3 | 0 | 0 | 0 |
| $\pi\_{j}$ | 5 | 2 | 5 | 3 | 2 | 6 | 4 | 2 | 3 | 5 | 5 | 5 | 4 | 5 | 2 | 2 | 4 | 4 | 4 | 5 | 4 | 4 | 4 | 4 | 4 | 2 | 5 | 6 | 3 | 5 | 3 | 5 | 3 | 1 | 1 | 1 | 1 | 1 | 1 | 1 | 1 | 1 | 1 | 1 | 1 |  |  |  |  |
| C | 0 | 0 | 0 | 0 | 0 | 0 | 0 | 0 | 0 | 0 | 0 | 0 | 0 | 0 | 0 | 0 | 0 | 0 | 0 | 0 | 0 | 0 | 0 | 0 | 0 | 0 | 0 | 0 | 0 | 0 | 0 | 0 | 0 | 6 | -3 | -3 | -5 | -10 | -10 | -5 | 0 | 0 | 0 | -1 | 0 |  |  |  |  |
| H | 0 | 0 | 0 | 0 | 0 | 0 | 0 | 0 | 0 | 0 | 0 | 0 | 0 | 0 | 0 | 0 | 0 | 0 | 0 | 0 | 0 | 0 | 0 | 0 | 0 | 0 | 0 | 0 | 0 | 0 | 0 | 0 | 0 | 12 | -3 | -5 | -5 | -13 | -12 | -4 | -1 | -1 | -2 | 0 | -3 |  |  |  |  |
| O | 0 | 0 | 0 | 0 | 0 | 0 | 0 | 0 | 0 | 0 | 0 | 0 | 0 | 0 | 0 | 0 | 0 | 0 | 0 | 0 | 0 | 0 | 0 | 0 | 0 | 0 | 0 | 0 | 0 | 0 | 0 | 0 | 0 | 6 | -3 | -3 | 0 | -4 | -5 | -1 | -4 | 0 | -1 | -2 | 0 |  |  |  |  |
| P | 0 | 0 | 0 | 0 | 0 | 0 | 0 | 0 | 0 | 0 | 0 | 0 | 0 | 0 | 0 | 0 | 0 | 0 | 0 | 0 | 0 | 0 | 0 | 0 | 0 | 0 | 0 | 0 | 0 | 0 | 0 | 0 | 0 | 0 | 0 | 0 | 0 | 0 | 0 | 0 | -1 | 0 | 0 | 0 | 0 |  |  |  |  |
| N | 0 | 0 | 0 | 0 | 0 | 0 | 0 | 0 | 0 | 0 | 0 | 0 | 0 | 0 | 0 | 0 | 0 | 0 | 0 | 0 | 0 | 0 | 0 | 0 | 0 | 0 | 0 | 0 | 0 | 0 | 0 | 0 | 0 | 0 | 0 | 0 | -5 | -5 | -4 | -4 | 0 | 0 | 0 | 0 | -1 |  |  |  |  |
| S | 0 | 0 | 0 | 0 | 0 | 0 | 0 | 0 | 0 | 0 | 0 | 0 | 0 | 0 | 0 | 0 | 0 | 0 | 0 | 0 | 0 | 0 | 0 | 0 | 0 | 0 | 0 | 0 | 0 | 0 | 0 | 0 | 0 | 0 | 0 | 0 | 0 | 0 | 0 | 0 | 0 | 0 | 0 | 0 | 0 |  |  |  |  |
| q | 0 | 0 | 0 | 0 | 0 | 0 | 0 | 0 | 0 | 0 | 0 | 0 | 0 | 0 | 0 | 0 | 0 | 0 | 0 | 0 | 0 | 0 | 0 | 0 | 0 | 0 | 0 | 0 | 0 | 0 | 2 | 0 | 2 | 0 | 1 | 1 | 0 | 0 | 0 | 0 | 2 | -1 | 0 | 0 | 0 |  |  |  |  |
| [NAD] | 0 | 0 | 0 | 0 | 0 | 0 | 0 | 0 | 0 | 0 | 0 | 0 | 0 | 0 | 0 | 0 | 0 | 0 | 0 | 0 | 0 | 0 | 0 | 0 | 0 | 0 | 0 | 0 | 0 | 0 | 0 | 0 | 0 | 0 | 0 | 0 | 0 | 0 | 0 | 0 | 0 | 0 | 0 | 0 | 0 |  |  |  |  |
| [NADP] | 0 | 0 | 0 | 0 | 0 | 0 | 0 | 0 | 0 | 0 | 0 | 0 | 0 | 0 | 0 | 0 | 0 | 0 | 0 | 0 | 0 | 0 | 0 | 0 | 0 | 0 | 0 | 0 | 0 | 0 | 0 | 0 | 0 | 0 | 0 | 0 | 0 | 0 | 0 | 0 | 0 | 0 | 0 | 0 | 0 |  |  |  |  |
| $p\_1$ | 1 | 1 | 1 | 1 | 1 | 2 | 2 | 2 | 2 | 2 | 2 | 0 | 0 | 0 | 0 | 0 | 0 | 0 | 0 | 0 | 0 | 0 | 0 | 0 | 0 | 0 | 0 | 0 | 0 | 2 | 0 | 0 | 0 | 1 | 0 | 2 | 0 | 0 | 0 | 0 | 0 | 2 | 0 | 0 | 0 |  |  |  |  |
| $p\_2$ | 0 | 0 | 0 | 0 | 0 | 0 | 0 | 0 | 0 | 0 | 1 | 0 | 0 | 0 | 0 | 0 | 0 | 0 | 0 | 0 | 0 | 0 | 0 | 0 | 0 | 0 | 0 | 0 | 0 | 0 | -1 | 0 | 0 | 0 | -1 | 1 | 0 | 0 | 0 | 0 | 0 | -2 | 0 | 0 | 0 |  |  |  |  |
| $p\_3$ | 1 | -2 | 0 | 0 | 0 | 1 | 1 | 1 | 1 | 1 | 1 | 3 | 3 | 3 | 2 | 1 | 1 | 1 | 1 | 0 | 0 | 0 | 0 | 0 | 0 | 0 | 0 | 0 | 0 | 1 | 0 | 6 | 6 | 1 | 0 | 1 | 0 | 0 | 0 | 0 | 0 | 13 | -3 | 3 | 0 |  |  |  |  |
| $p\_4$ | 1 | 0 | 0 | 0 | 0 | 0 | 0 | 0 | 0 | 0 | 0 | 1 | 1 | 1 | 0 | 1 | 0 | 0 | 0 | 0 | 1 | 0 | 0 | 0 | 0 | 0 | 1 | 1 | -1 | -3 | 0 | 2 | 2 | 1 | 0 | 0 | -1 | 1 | 0 | 0 | 0 | 4 | 0 | 1 | 0 |  |  |  |  |
| $p\_5$ | 1 | 0 | 0 | 0 | 0 | 0 | 0 | 0 | 0 | 0 | 0 | 1 | 1 | 1 | 0 | 1 | 0 | 0 | 0 | 0 | 1 | 1 | 0 | 0 | 0 | 0 | 1 | 1 | -1 | -3 | 0 | 2 | 2 | 1 | 0 | 0 | -1 | 0 | 1 | 0 | 0 | 4 | -1 | 1 | 1 |  |  |  |  |
| $p\_6$ | 1 | 0 | 0 | 0 | 0 | 0 | 0 | 0 | 0 | 0 | 0 | 1 | 1 | 1 | 0 | 1 | 0 | 0 | 0 | 0 | 0 | 0 | 1 | 1 | 0 | 0 | 1 | 1 | -1 | -3 | 0 | 2 | 2 | 1 | 0 | 0 | -1 | 0 | 1 | 0 | 0 | 4 | -1 | 1 | 1 |  |  |  |  |
| $p\_7$ | 0 | 0 | 0 | 0 | 0 | 0 | 0 | 0 | 0 | 0 | 0 | 0 | 0 | 0 | 0 | 0 | 0 | 0 | 0 | 0 | 1 | 1 | 0 | 0 | 1 | 1 | 1 | 1 | -1 | -2 | 0 | 0 | 0 | 0 | 0 | 0 | -1 | 0 | 0 | 1 | 0 | 0 | -1 | 0 | 1 |  |  |  |  |
| $p\_8$ | 0 | 0 | 0 | 0 | 0 | 0 | 0 | 0 | 0 | 0 | 0 | 0 | 0 | 0 | 0 | 0 | 0 | 0 | 0 | 1 | 1 | 0 | 0 | 0 | 0 | 0 | 0 | 0 | 0 | -1 | 0 | 0 | 0 | 0 | 0 | 0 | 0 | 0 | 0 | 0 | 0 | 0 | 0 | 0 | 0 |  |  |  |  |

###### The pathway structure:¶

**Figure 12.7:** Pathway maps for the eight pathway vectors for the system formed by coupling glycolysis, the pentose pathway, and the AMP Salvage Network. (From left to right, top to bottom, \(\textbf{p}\_1\) through \(\textbf{p}\_8\)). They span all possible steady state solutions.

There are eight pathway vectors, shown in Table 12.10, that are chosen to match those used above and in the previous chapter. \(\textbf{p}\_1\) is the glycolytic pathway to lactate secretion, \(\textbf{p}\_2\) is the pyruvate-lactate exchange coupled to the NADH cofactor, and \(\textbf{p}\_3\) is the pentose pathway cycle to \(\text{CO}\_2\) formation producing NADPH. These are the same pathways as above. In the last chapter we had AMP exchange that now couples to the five AMP
degradation and salvage pathways that form in the AMP metabolic sub-network. They are as follows:

1. \(\textbf{p}\_4\) is a complicated pathway that imports glucose that is processed through the pentose pathway to form R5P and the formation of \(\text{CO}\_2\). R5P is then combined with an imported adenine to form AMP that then goes through the degradation pathway to adenosine that is secreted. This pathway is a balanced use of the whole network and represents a combination of the AMP biosynthetic and degradation pathways. This pathway corresponds to \(\textbf{p}\_1\) in the AMP
   sub-network.
2. \(\textbf{p}\_5\) is a similar pathway as \(\textbf{p}\_4\) above but producing inosine, and it corresponds to \(\textbf{p}\_2\) in the AMP sub-network. Inosine is produced from AMP by first dephosphorylation to adenosine followed by deamination to inosine.
3. \(\textbf{p}\_6\) is a similar pathway as \(\textbf{p}\_4\) above but producing inosine, and it corresponds to \(\textbf{p}\_3\) in the AMP sub-network. Inosine is produced from AMP by first deamination to IMP followed by dephosphorylation to inosine; the same effect as in \(\textbf{p}\_5\), but in the opposite order.
4. \(\textbf{p}\_7\) is the salvage pathway that has a net import of adenine and secreting hypoxanthine with a deamination step.
5. \(\textbf{p}\_8\) is a futile cycle that consumes ATP through the simultaneous use of ADNK1 and NTD7.

As we saw with the integration of glycolysis and the pentose pathway, the incorporation of the AMP sub-network creates new network-wide pathways. These network-based pathway definitions further show how historical definitions of pathways give way to full network considerations as we form pathways for the entire system. Again, this feature is of great importance to systems biology (Papin 2003, *Systems Biology: Properties of Reconstructed Networks*).

###### Structure of the stoichiometric matrix¶

The matrix can be organized by pathway and metabolic processes. The dashed lines in Table 12.10 divide the matrix up into blocks. Each block in this formulation shows how the metabolites and reactions are coupled. These diagonal blocks describe the metabolites and reactions in each sub-network.

The upper off-diagonal blocks, for instance, show how the metabolites, cofactors and exchanges affect the metabolites in each pathway or sub-network represented on the diagonal. The lower off-diagonal blocks show how the metabolites, cofactors and exchanges participate in the reaction in each pathway or sub-network that is represented by the corresponding block on the diagonal.

##### Defining the Steady State¶

The null space is eight dimensional and we have to specify eight fluxes to fix the steady state. Following the previous chapters and what is given above, we specify: \(v\_{S\_{\textit{glc\_\_D}}} = 1.12\), \(v\_{DM\_{nadh}}=0.2\*v\_{S\_{\textit{glc\_\_D}}}\), \(v\_{GSHR}=0.42\), \(v\_{SK\_{ade}}=-0.014\), \(v\_{ADA}=0.01\), \(v\_{SK\_{adn}}=0.01\), \(v\_{ADNK1}=0.12\), and \(v\_{SK\_{hxan}} = 0.097\) using earlier models and specifying independent fluxes in the pathway vectors.

```
[29]:
```

```
# Set independent fluxes to determine steady state flux vector
independent_fluxes = {
    core_network.reactions.SK_glc__D_c: 1.12,
    core_network.reactions.DM_nadh: 0.2*1.12,
    core_network.reactions.GSHR : 0.42,
    core_network.reactions.SK_ade_c: -0.014,
    core_network.reactions.ADA: 0.01,
    core_network.reactions.SK_adn_c: -0.01,
    core_network.reactions.ADNK1: 0.12,
    core_network.reactions.SK_hxan_c: 0.097}

ssfluxes = core_network.compute_steady_state_fluxes(
    minspan_paths,
    independent_fluxes,
    update_reactions=True)
table_12_11 = pd.DataFrame(list(ssfluxes.values()), index=reaction_ids,
                           columns=[r"$\textbf{v}_{\mathrm{stst}}$"]).T
```

**Table 12.11:** The steady state fluxes as a summation of the MinSpan pathway vectors.

```
[30]:
```

```
table_12_11
```

```
[30]:
```

|  | HEX1 | PGI | PFK | FBA | TPI | GAPD | PGK | PGM | ENO | PYK | LDH\_L | G6PDH2r | PGL | GND | RPE | RPI | TKT1 | TKT2 | TALA | ADNK1 | NTD7 | ADA | AMPDA | NTD11 | PUNP5 | PPM | PRPPS | ADPT | ADK1 | ATPM | DM\_nadh | GTHOr | GSHR | SK\_glc\_\_D\_c | SK\_pyr\_c | SK\_lac\_\_L\_c | SK\_ade\_c | SK\_adn\_c | SK\_ins\_c | SK\_hxan\_c | SK\_pi\_c | SK\_h\_c | SK\_h2o\_c | SK\_co2\_c | SK\_nh3\_c |
| --- | --- | --- | --- | --- | --- | --- | --- | --- | --- | --- | --- | --- | --- | --- | --- | --- | --- | --- | --- | --- | --- | --- | --- | --- | --- | --- | --- | --- | --- | --- | --- | --- | --- | --- | --- | --- | --- | --- | --- | --- | --- | --- | --- | --- | --- |
| $\textbf{v}\_{\mathrm{stst}}$ | 1.120 | 0.910 | 1.105 | 1.105 | 1.105 | 2.308 | 2.308 | 2.308 | 2.308 | 2.308 | 2.084 | 0.210 | 0.210 | 0.210 | 0.195 | 0.015 | 0.098 | 0.098 | 0.098 | 0.120 | 0.120 | 0.010 | 0.014 | 0.014 | 0.097 | 0.097 | 0.014 | 0.014 | -0.014 | 2.243 | 0.224 | 0.420 | 0.420 | 1.120 | 0.224 | 2.084 | -0.014 | -0.010 | -0.073 | 0.097 | 0.000 | 3.596 | -0.317 | 0.210 | 0.024 |

These vectors can be visualized as a bar chart:

```
[31]:
```

```
fig_12_8, ax = plt.subplots(nrows=1, ncols=1, figsize=(10, 5))
# Define indicies for bar chart
indicies = np.arange(len(reaction_ids))+0.5
# Define colors to use
c = plt.cm.coolwarm(np.linspace(0, 1, len(reaction_ids)))
# Plot bar chart
ax.bar(indicies, list(ssfluxes.values()), width=0.8, color=c);
ax.set_xlim([0, len(reaction_ids)]);
# Set labels and adjust ticks
ax.set_xticks(indicies);
ax.set_xticklabels(reaction_ids, rotation="vertical");
ax.set_ylabel("Fluxes (mM/hr)", L_FONT);
ax.set_title("Steady State Fluxes", L_FONT);
ax.plot([0, len(reaction_ids)], [0, 0], "k--");
fig_12_8.tight_layout()
```

**Figure 12.8:** Bar chart of the steady-state fluxes.

**A numerical QC/QA:** Ensure \(\textbf{Sv}\_{\mathrm{stst}} = 0\)

```
[32]:
```

```
pd.DataFrame(
    core_network.S.dot(np.array(list(ssfluxes.values()))),
    index=metabolite_ids,
    columns=[r"$\textbf{Sv}_{\mathrm{stst}}$"],
    dtype=np.int64).T
```

```
[32]:
```

|  | glc\_\_D\_c | g6p\_c | f6p\_c | fdp\_c | dhap\_c | g3p\_c | \_13dpg\_c | \_3pg\_c | \_2pg\_c | pep\_c | pyr\_c | lac\_\_L\_c | \_6pgl\_c | \_6pgc\_c | ru5p\_\_D\_c | xu5p\_\_D\_c | r5p\_c | s7p\_c | e4p\_c | ade\_c | adn\_c | imp\_c | ins\_c | hxan\_c | r1p\_c | prpp\_c | nad\_c | nadh\_c | amp\_c | adp\_c | atp\_c | nadp\_c | nadph\_c | gthrd\_c | gthox\_c | pi\_c | h\_c | h2o\_c | co2\_c | nh3\_c |
| --- | --- | --- | --- | --- | --- | --- | --- | --- | --- | --- | --- | --- | --- | --- | --- | --- | --- | --- | --- | --- | --- | --- | --- | --- | --- | --- | --- | --- | --- | --- | --- | --- | --- | --- | --- | --- | --- | --- | --- | --- |
| $\textbf{Sv}\_{\mathrm{stst}}$ | 0 | 0 | 0 | 0 | 0 | 0 | 0 | 0 | 0 | 0 | 0 | 0 | 0 | 0 | 0 | 0 | 0 | 0 | 0 | 0 | 0 | 0 | 0 | 0 | 0 | 0 | 0 | 0 | 0 | 0 | 0 | 0 | 0 | 0 | 0 | 0 | 0 | 0 | 0 | 0 |

###### Computing the rate constants¶

The kinetic constants can be computed from the steady state values of the concentrations using elementary mass action kinetics. The computation is based on Eq. (10.4) The results from this computation is summarized in Table 12.12. This table has all the reaction properties that we need to complete the MASS model.

```
[33]:
```

```
percs = core_network.calculate_PERCs(
    fluxes={
        r: flux for r, flux in core_network.steady_state_fluxes.items()
        if r.id != "ADK1"}, # Skip ADK1
    update_reactions=True)
```

**Table 12.12:** Combined glycolysis, pentose phosphate pathway, and AMP salvage network enzymes and transport rates.

```
[34]:
```

```
# Get concentration values for substitution into sympy expressions
value_dict = {sym.Symbol(str(met)): ic
              for met, ic in core_network.initial_conditions.items()}
value_dict.update({sym.Symbol(str(met)): bc
                   for met, bc in core_network.boundary_conditions.items()})
table_12_12 = []
# Get symbols and values for table and substitution
for p_key in ["Keq", "kf"]:
    symbol_list, value_list = [], []
    for p_str, value in core_network.parameters[p_key].items():
        symbol_list.append(r"$%s_{\text{%s}}$" % (p_key[0], p_str.split("_", 1)[-1]))
        value_list.append("{0:.3f}".format(value) if value != INF else r"$\infty$")
        value_dict.update({sym.Symbol(p_str): value})
    table_12_12.extend([symbol_list, value_list])

table_12_12.append(["{0:.6f}".format(float(ratio.subs(value_dict)))
                    for ratio in strip_time(core_network.get_mass_action_ratios()).values()])
table_12_12.append(["{0:.6f}".format(float(ratio.subs(value_dict)))
                    for ratio in strip_time(core_network.get_disequilibrium_ratios()).values()])
table_12_12 = pd.DataFrame(np.array(table_12_12).T, index=reaction_ids,
                           columns=[r"$K_{eq}$ Symbol", r"$K_{eq}$ Value", "PERC Symbol",
                                    "PERC Value", r"$\Gamma$", r"$\Gamma/K_{eq}$"])
table_12_12
```

```
[34]:
```

|  | $K\_{eq}$ Symbol | $K\_{eq}$ Value | PERC Symbol | PERC Value | $\Gamma$ | $\Gamma/K\_{eq}$ |
| --- | --- | --- | --- | --- | --- | --- |
| HEX1 | $K\_{\text{HEX1}}$ | 850.000 | $k\_{\text{HEX1}}$ | 0.700 | 0.008809 | 0.000010 |
| PGI | $K\_{\text{PGI}}$ | 0.410 | $k\_{\text{PGI}}$ | 2961.111 | 0.407407 | 0.993677 |
| PFK | $K\_{\text{PFK}}$ | 310.000 | $k\_{\text{PFK}}$ | 34.906 | 0.133649 | 0.000431 |
| FBA | $K\_{\text{FBA}}$ | 0.082 | $k\_{\text{FBA}}$ | 2797.449 | 0.079781 | 0.972937 |
| TPI | $K\_{\text{TPI}}$ | 0.057 | $k\_{\text{TPI}}$ | 33.906 | 0.045500 | 0.796249 |
| GAPD | $K\_{\text{GAPD}}$ | 0.018 | $k\_{\text{GAPD}}$ | 3479.760 | 0.006823 | 0.381183 |
| PGK | $K\_{\text{PGK}}$ | 1800.000 | $k\_{\text{PGK}}$ | 1312381.554 | 1755.073081 | 0.975041 |
| PGM | $K\_{\text{PGM}}$ | 0.147 | $k\_{\text{PGM}}$ | 5017.110 | 0.146184 | 0.994048 |
| ENO | $K\_{\text{ENO}}$ | 1.695 | $k\_{\text{ENO}}$ | 1817.545 | 1.504425 | 0.887608 |
| PYK | $K\_{\text{PYK}}$ | 363000.000 | $k\_{\text{PYK}}$ | 468.247 | 19.570304 | 0.000054 |
| LDH\_L | $K\_{\text{LDH\_L}}$ | 26300.000 | $k\_{\text{LDH\_L}}$ | 1150.285 | 44.132974 | 0.001678 |
| G6PDH2r | $K\_{\text{G6PDH2r}}$ | 1000.000 | $k\_{\text{G6PDH2r}}$ | 21864.589 | 11.875411 | 0.011875 |
| PGL | $K\_{\text{PGL}}$ | 1000.000 | $k\_{\text{PGL}}$ | 122.323 | 21.362698 | 0.021363 |
| GND | $K\_{\text{GND}}$ | 1000.000 | $k\_{\text{GND}}$ | 29287.807 | 43.340651 | 0.043341 |
| RPE | $K\_{\text{RPE}}$ | 3.000 | $k\_{\text{RPE}}$ | 22392.052 | 2.994699 | 0.998233 |
| RPI | $K\_{\text{RPI}}$ | 2.570 | $k\_{\text{RPI}}$ | 2021.058 | 2.566222 | 0.998530 |
| TKT1 | $K\_{\text{TKT1}}$ | 1.200 | $k\_{\text{TKT1}}$ | 2338.070 | 0.932371 | 0.776976 |
| TKT2 | $K\_{\text{TKT2}}$ | 10.300 | $k\_{\text{TKT2}}$ | 1600.141 | 1.921130 | 0.186517 |
| TALA | $K\_{\text{TALA}}$ | 1.050 | $k\_{\text{TALA}}$ | 1237.363 | 0.575416 | 0.548015 |
| ADNK1 | $K\_{\text{ADNK1}}$ | $\infty$ | $k\_{\text{ADNK1}}$ | 62.500 | 13.099557 | 0.000000 |
| NTD7 | $K\_{\text{NTD7}}$ | $\infty$ | $k\_{\text{NTD7}}$ | 1.384 | 0.034591 | 0.000000 |
| ADA | $K\_{\text{ADA}}$ | $\infty$ | $k\_{\text{ADA}}$ | 8.333 | 0.075835 | 0.000000 |
| AMPDA | $K\_{\text{AMPDA}}$ | $\infty$ | $k\_{\text{AMPDA}}$ | 0.161 | 0.010493 | 0.000000 |
| NTD11 | $K\_{\text{NTD11}}$ | $\infty$ | $k\_{\text{NTD11}}$ | 1.400 | 0.250000 | 0.000000 |
| PUNP5 | $K\_{\text{PUNP5}}$ | 0.090 | $k\_{\text{PUNP5}}$ | 83.143 | 0.048000 | 0.533333 |
| PPM | $K\_{\text{PPM}}$ | 13.300 | $k\_{\text{PPM}}$ | 1.643 | 0.211148 | 0.015876 |
| PRPPS | $K\_{\text{PRPPS}}$ | $\infty$ | $k\_{\text{PRPPS}}$ | 0.691 | 0.021393 | 0.000000 |
| ADPT | $K\_{\text{ADPT}}$ | $\infty$ | $k\_{\text{ADPT}}$ | 2800.000 | 108410.125000 | 0.000000 |
| ADK1 | $K\_{\text{ADK1}}$ | 1.650 | $k\_{\text{ADK1}}$ | 100000.000 | 1.650000 | 1.000000 |
| ATPM | $K\_{\text{ATPM}}$ | $\infty$ | $k\_{\text{ATPM}}$ | 1.402 | 0.453125 | 0.000000 |
| DM\_nadh | $K\_{\text{DM\_nadh}}$ | $\infty$ | $k\_{\text{DM\_nadh}}$ | 7.442 | 1.956811 | 0.000000 |
| GTHOr | $K\_{\text{GTHOr}}$ | 100.000 | $k\_{\text{GTHOr}}$ | 53.330 | 0.259372 | 0.002594 |
| GSHR | $K\_{\text{GSHR}}$ | 2.000 | $k\_{\text{GSHR}}$ | 0.041 | 0.011719 | 0.005859 |
| SK\_glc\_\_D\_c | $K\_{\text{SK\_glc\_\_D\_c}}$ | $\infty$ | $k\_{\text{SK\_glc\_\_D\_c}}$ | 1.120 | 1.000000 | 0.000000 |
| SK\_pyr\_c | $K\_{\text{SK\_pyr\_c}}$ | 1.000 | $k\_{\text{SK\_pyr\_c}}$ | 744.186 | 0.995008 | 0.995008 |
| SK\_lac\_\_L\_c | $K\_{\text{SK\_lac\_\_L\_c}}$ | 1.000 | $k\_{\text{SK\_lac\_\_L\_c}}$ | 5.790 | 0.735294 | 0.735294 |
| SK\_ade\_c | $K\_{\text{SK\_ade\_c}}$ | 1.000 | $k\_{\text{SK\_ade\_c}}$ | 100000.000 | 1.000000 | 1.000000 |
| SK\_adn\_c | $K\_{\text{SK\_adn\_c}}$ | 1.000 | $k\_{\text{SK\_adn\_c}}$ | 100000.000 | 1.000000 | 1.000000 |
| SK\_ins\_c | $K\_{\text{SK\_ins\_c}}$ | 1.000 | $k\_{\text{SK\_ins\_c}}$ | 100000.000 | 1.000000 | 1.000000 |
| SK\_hxan\_c | $K\_{\text{SK\_hxan\_c}}$ | 1.000 | $k\_{\text{SK\_hxan\_c}}$ | 100000.000 | 1.000000 | 1.000000 |
| SK\_pi\_c | $K\_{\text{SK\_pi\_c}}$ | 1.000 | $k\_{\text{SK\_pi\_c}}$ | 100000.000 | 1.000000 | 1.000000 |
| SK\_h\_c | $K\_{\text{SK\_h\_c}}$ | 1.000 | $k\_{\text{SK\_h\_c}}$ | 133792.163 | 0.701253 | 0.701253 |
| SK\_h2o\_c | $K\_{\text{SK\_h2o\_c}}$ | 1.000 | $k\_{\text{SK\_h2o\_c}}$ | 100000.000 | 1.000000 | 1.000000 |
| SK\_co2\_c | $K\_{\text{SK\_co2\_c}}$ | 1.000 | $k\_{\text{SK\_co2\_c}}$ | 100000.000 | 1.000000 | 1.000000 |
| SK\_nh3\_c | $K\_{\text{SK\_nh3\_c}}$ | 1.000 | $k\_{\text{SK\_nh3\_c}}$ | 100000.000 | 1.000000 | 1.000000 |

##### Simulating the Dynamic Mass Balance¶

###### Validating the steady state¶

As before, we must first ensure that the system is originally at steady state:

```
[35]:
```

```
t0, tf = (0, 1e3)
sim_core = Simulation(core_network)
sim_core.find_steady_state(
    core_network, strategy="simulate",
    update_values=True)
conc_sol_ss, flux_sol_ss = sim_core.simulate(
    core_network, time=(t0, tf, tf*10 + 1))
# Quickly render and display time profiles
conc_sol_ss.view_time_profile()
```

**Figure 12.9:** The merged model after determining the steady state conditions.

We can compare the differences in the initial state of each model before merging and after.

```
[36]:
```

```
fig_12_10, axes = plt.subplots(1, 2, figsize=(9, 4))
(ax1, ax2) = axes.flatten()

# Compare initial conditions
initial_conditions = {
    m.id: ic for m, ic in glycolysis.initial_conditions.items()
    if m.id in core_network.metabolites}
initial_conditions.update({
    m.id: ic for m, ic in ppp.initial_conditions.items()
    if m.id in core_network.metabolites})
initial_conditions.update({
    m.id: ic for m, ic in ampsn.initial_conditions.items()
    if m.id in core_network.metabolites})

plot_comparison(
    core_network, pd.Series(initial_conditions), compare="concentrations",
    ax=ax1, plot_function="loglog",
    xlabel="Merged Model", ylabel="Independent Models",
    title=("(a) Steady State Concentrations of Species", L_FONT),
    color="blue", xy_line=True, xy_legend="best");

# Compare fluxes
fluxes = {
    r.id: flux for r, flux in glycolysis.steady_state_fluxes.items()
    if r.id in core_network.reactions}
fluxes.update({
    r.id: flux for r, flux in ppp.steady_state_fluxes.items()
    if r.id in core_network.reactions})
fluxes.update({
    r.id: flux for r, flux in ampsn.steady_state_fluxes.items()
    if r.id in core_network.reactions})

plot_comparison(
    core_network, pd.Series(fluxes), compare="fluxes",
    ax=ax2, plot_function="plot",
    xlabel="Merged Model", ylabel="Independent Models",
    title=("(b) Steady State Fluxes of Reactions", L_FONT),
    color="red", xy_line=True, xy_legend="best");
fig_12_10.tight_layout()
```

**Figure 12.10:** Comparisons between the initial conditions of the merged model and the initial conditions of the independent glycolysis, pentose phosphate pathway, and AMP salvage networks for (a) the species and (b) the fluxes.

###### Response to an increased \(k\_{ATPM}\)¶

First, we must ensure that the system is originally at steady state. We perform the same simulation as in the last chapter by increasing the rate of ATP utilization.

```
[37]:
```

```
conc_sol, flux_sol = sim_core.simulate(
    core_network, time=(t0, tf, tf*10 + 1),
    perturbations={"kf_ATPM": "kf_ATPM * 1.5"})
```

```
[38]:
```

```
fig_12_11, axes = plt.subplots(nrows=2, ncols=1, figsize=(10, 8));
(ax1, ax2) = axes.flatten()

plot_time_profile(
    conc_sol, ax=ax1, legend="right outside",
    plot_function="loglog",
    xlabel="Time [hr]", ylabel="Concentration [mM]",
    title=("Concentration Profile", L_FONT));

plot_time_profile(
    flux_sol, ax=ax2, legend="right outside",
    plot_function="semilogx",
    xlabel="Time [hr]", ylabel="Flux [mM/hr]",
    title=("Flux Profile", L_FONT));
fig_12_11.tight_layout()
```

**Figure 12.11:** Simulating the combined system from the steady state with 50% increase in the rate of ATP utilization at \(t = 0\).

###### Relative deviations from the initial state¶

As shown in the figure below, AMP still has the highest perturbation. Note that there is slightly more variation in the concentration profiles at around \(t = 10\) than in combined glycolytic and pentose phosphate pathways. We can see that the flux deviations also follow the same general trend as the combined glycolysis and pentose phosphate pathway simulations, with slightly more variation.

```
[39]:
```

```
fig_12_12, axes = plt.subplots(nrows=2, ncols=1, figsize=(10, 8));
(ax1, ax2) = axes.flatten()

conc_deviation = {met.id: conc_sol[met.id]/ic
                  for met, ic in core_network.initial_conditions.items()}
conc_deviation = MassSolution(
    "Deviation", solution_type="Conc",
    data_dict=conc_deviation,
    time=conc_sol.t, interpolate=False)

flux_deviation = {rxn.id: flux_sol[rxn.id]/ssflux
                  for rxn, ssflux in core_network.steady_state_fluxes.items()
                  if ssflux != 0} # To avoid dividing by 0 for equilibrium fluxes.

flux_deviation = MassSolution(
    "Deviation", solution_type="Flux",
    data_dict=flux_deviation,
    time=flux_sol.t, interpolate=False)

plot_time_profile(
    conc_deviation, ax=ax1, legend="right outside",
    plot_function="semilogx",
    xlabel="Time [hr]", ylabel="Concentration [mM]",
    title=("Concentration Profile", L_FONT));

plot_time_profile(
    flux_deviation, ax=ax2, legend="right outside",
    plot_function="semilogx",
    xlabel="Time [hr]", ylabel="Flux [mM/hr]",
    title=("Flux Profile", L_FONT));
fig_12_12.tight_layout()
```

**Figure 12.12:** (a) Deviation from the steady state of the concentrations as a fraction of the steady state. (b) Deviation from the steady state of the fluxes as a fraction of the steady state.

###### ATP Simulation Node Maps¶

As before, we will examine the node balances to gain a better understanding of the system’s response.

###### The proton node¶

The proton node now has a connectivity of 15 with 11 production reactions and 4 utilization reactions.

```
[40]:
```

```
fig_12_13 = plt.figure(figsize=(17, 6))
gs = fig_12_13.add_gridspec(nrows=3, ncols=2, width_ratios=[1.5, 1])

ax1 = fig_12_13.add_subplot(gs[0, 0])
ax2 = fig_12_13.add_subplot(gs[1, 0])
ax3 = fig_12_13.add_subplot(gs[2, 0])
ax4 = fig_12_13.add_subplot(gs[:, 1])

plot_time_profile(
    conc_sol, observable="h_c", ax=ax1,
    legend="right outside", plot_function="semilogx",
    xlim=(1e-6, tf), ylim=(8.5e-5, 1e-4*1.025),
    xlabel="Time [hr]", ylabel="Concentrations [mM]",
    title=("(a) Proton Concentration", L_FONT));

fluxes_in = ["HEX1", "PFK", "GAPD", "ATPM", "DM_nadh",
             "G6PDH2r", "PGL", "GSHR", "ADNK1", "PRPPS", "ADPT"]
plot_time_profile(
    flux_sol, observable=fluxes_in, ax=ax2,
    legend="right outside", plot_function="semilogx",
    xlim=(1e-6, tf),  ylim=(-0.4, 4),
    xlabel="Time [hr]", ylabel="Fluxes [mM/hr]",
    title=("(b) Fluxes in", L_FONT));

fluxes_out = ["PYK", "LDH_L","SK_h_c", "GTHOr"]
plot_time_profile(
    flux_sol, observable=fluxes_out, ax=ax3,
    legend="right outside", plot_function="semilogx",
    xlim=(1e-6, tf),  ylim=(1.2, 5),
    xlabel="Time [hr]", ylabel="Fluxes [mM/hr]",
    title=("(c) Fluxes out", L_FONT));

for flux_id, variables in zip(["Net_Flux_In", "Net_Flux_Out"],
                              [fluxes_in, fluxes_out]):
    flux_sol.make_aggregate_solution(
        flux_id, equation=" + ".join(variables), variables=variables)

time_points = [t0, 1e-1, 1e0, 1e1, 1e2, tf]
time_point_colors = [
    mpl.colors.to_hex(c)
    for c in mpl.cm.Reds(np.linspace(0.3, 1, len(time_points)))]

plot_phase_portrait(
    flux_sol, x="Net_Flux_In", y="Net_Flux_Out", ax=ax4,
    xlim=(6.5, 10), ylim=(6.5, 10),
    xlabel="Fluxes in [mm/Hr]", ylabel="Fluxes out [mm/Hr]",
    title=("(d) Phase Portrait of Fluxes", L_FONT),
    annotate_time_points=time_points,
    annotate_time_points_color=time_point_colors,
    annotate_time_points_legend="best");
fig_12_13.tight_layout()
```

**Figure 12.13:** The time profiles of the (a) proton concentration, (b) the fluxes that make protons, (c) the fluxes that use protons and (d) the phase portrait of the net flux in and net flux out (darker red colors indicate slower time scales) for an increase in ATP utilization.

###### The ATP Node¶

The ATP node now has a connectivity of 8.

```
[41]:
```

```
fig_12_14 = plt.figure(figsize=(15, 6))
gs = fig_12_14.add_gridspec(nrows=3, ncols=2, width_ratios=[1.5, 1])

ax1 = fig_12_14.add_subplot(gs[0, 0])
ax2 = fig_12_14.add_subplot(gs[1, 0])
ax3 = fig_12_14.add_subplot(gs[2, 0])
ax4 = fig_12_14.add_subplot(gs[:, 1])

plot_time_profile(
    conc_sol, observable="atp_c", ax=ax1,
    legend="right outside", plot_function="semilogx",
    xlim=(1e-6, tf), ylim=(.75, 2),
    xlabel="Time [hr]", ylabel="Concentrations [mM]",
    title=("(a) ATP Concentration", L_FONT));

fluxes_in = ["PGK", "PYK", "ADK1"]
plot_time_profile(
    flux_sol, observable=fluxes_in, ax=ax2,
    legend="right outside", plot_function="semilogx",
    xlim=(1e-6, tf), ylim=(-0.1, 3),
    xlabel="Time [hr]", ylabel="Fluxes [mM/hr]",
    title=("(b) Fluxes in", L_FONT));

fluxes_out = ["HEX1", "PFK", "ATPM", "ADNK1", "PRPPS"]
plot_time_profile(
    flux_sol, observable=fluxes_out, ax=ax3,
    legend="right outside", plot_function="semilogx",
    xlim=(1e-6, tf), ylim=(-0.1, 4),
    xlabel="Time [hr]", ylabel="Fluxes [mM/hr]",
    title=("(c) Fluxes out", L_FONT));

for flux_id, variables in zip(["Net_Flux_In", "Net_Flux_Out"],
                              [fluxes_in, fluxes_out]):
    flux_sol.make_aggregate_solution(
        flux_id, equation=" + ".join(variables), variables=variables)

time_points = [t0, 1e-1, 1e0, 1e1, 1e2, tf]
time_point_colors = [
    mpl.colors.to_hex(c)
    for c in mpl.cm.Reds(np.linspace(0.3, 1, len(time_points)))]

plot_phase_portrait(
    flux_sol, x="Net_Flux_In", y="Net_Flux_Out", ax=ax4,
    xlim=(3.5, 6.0), ylim=(3.5, 6.0),
    xlabel="Fluxes in [mm/Hr]", ylabel="Fluxes out [mm/Hr]",
    title=("(d) Phase Portrait of Fluxes", L_FONT),
    annotate_time_points=time_points,
    annotate_time_points_color=time_point_colors,
    annotate_time_points_legend="best");
fig_12_14.tight_layout()
```

**Figure 12.14:** The time profiles of the (a) ATP concentration, (b) the fluxes that make ATP, (c) the fluxes that use ATP and (d) the phase portrait of the net flux in and net flux out (darker red colors indicate slower time scales) for an increase in ATP utilization.

###### The AMP node¶

From the node map of AMP, we see that the biggest fluxes through the node are \(v\_{ADNK1}\) and \(v\_{NTD7}\).

```
[42]:
```

```
fig_12_15 = plt.figure(figsize=(15, 6))
gs = fig_12_15.add_gridspec(nrows=3, ncols=2, width_ratios=[1.5, 1])

ax1 = fig_12_15.add_subplot(gs[0, 0])
ax2 = fig_12_15.add_subplot(gs[1, 0])
ax3 = fig_12_15.add_subplot(gs[2, 0])
ax4 = fig_12_15.add_subplot(gs[:, 1])

plot_time_profile(conc_sol, observable="amp_c", ax=ax1,
                legend="right outside", plot_function="semilogx",
                xlim=(1e-6, tf), ylim=(.035, 0.25),
                xlabel="Time [hr]", ylabel="Concentrations [mM]",
                title=("(a) AMP Concentration", L_FONT));

fluxes_in = ["ADNK1", "PRPPS", "ATPM"]
plot_time_profile(flux_sol, observable=fluxes_in, ax=ax2,
                legend="right outside", plot_function="semilogx",
                xlim=(1e-6, tf), ylim=(-0.1, 4),
                xlabel="Time [hr]", ylabel="Fluxes [mM/hr]",
                title=("(b) Fluxes in", L_FONT));

fluxes_out = ["PGK", "PYK","ADK1"]
plot_time_profile(flux_sol, observable=fluxes_out, ax=ax3,
                legend="right outside", plot_function="semilogx",
                xlim=(1e-6, tf), ylim=(-0.1, 3),
                xlabel="Time [hr]", ylabel="Fluxes [mM/hr]",
                title=("(c) Fluxes out", L_FONT));

for flux_id, variables in zip(["Net_Flux_In", "Net_Flux_Out"],
                              [fluxes_in, fluxes_out]):
    flux_sol.make_aggregate_solution(
        flux_id, equation=" + ".join(variables), variables=variables)

time_points = [t0, 1e-1, 1e0, 1e1, 1e2, tf]
time_point_colors = [
    mpl.colors.to_hex(c)
    for c in mpl.cm.Reds(np.linspace(0.3, 1, len(time_points)))]

plot_phase_portrait(
    flux_sol, x="Net_Flux_In", y="Net_Flux_Out", ax=ax4,
    xlim=(2.0, 3.6), ylim=(3.5, 5.5),
    xlabel="Fluxes in [mm/Hr]", ylabel="Fluxes out [mm/Hr]",
    title=("(d) Phase Portrait of Fluxes", L_FONT),
    annotate_time_points=time_points,
    annotate_time_points_color=time_point_colors,
    annotate_time_points_legend="best");
fig_12_15.tight_layout()
```

**Figure 12.15:** The time profiles of the (a) AMP concentration, (b) the fluxes that make AMP, (c) the fluxes that use AMP and (d) the phase portrait of the net flux in and net flux out (darker red colors indicate slower time scales) for an increase in ATP utilization.

We highlight two aspects of this simulation:

**1.** *Flux balancing of the AMP node:* Previously, the influx (formation) of AMP into the system has been constant, but the output (degradation) was a linear function of AMP. In the integrated model simulated here, the formation of AMP is now explicitly represented by a biosynthetic pathway. We thus plot the phase portrait of the sum of all the formation and the sum of all degradation fluxes of AMP, Figure 12.16a. The 45 degree line in this diagram represents the steady state. The initial
reaction to the perturbation is motion above the 45 degree line where there is net consumption of AMP. The trajectory turns around and heads towards the steady state line, overshoots it at first, but eventually settles down in the steady state.

The phase portrait of the ATP load flux and the net AMP consumption rate was considered in the earlier chapters and is shown in Figure 12.16b. As before, the sudden increase in the ATP is followed by a net removal of AMP from the system and a dropping ATP load flux to reach the steady state again.

```
[43]:
```

```
fig_12_16, axes = plt.subplots(nrows=1, ncols=2, figsize=(12, 5))
(ax1, ax2) = axes.flatten()

equation_dict = {
    "AMP_Production": ["ADNK1 + ADPT + PRPPS", ["ADNK1", "ADPT", "PRPPS"]],
    "AMP_Consumption":["AMPDA + NTD7", ["AMPDA", "NTD7"]],
    "ATP_Load_Flux": ["ATPM", ["ATPM"]],
    "Net_AMP_Consumption": [
        "-(ADNK1 + ADPT + PRPPS) + (AMPDA + NTD7)", [
            "ADNK1", "ADPT", "PRPPS", "AMPDA", "NTD7"]]}

for sol_id, (equation, variables) in equation_dict.items():
    flux_sol.make_aggregate_solution(
        sol_id, equation=equation, variables=variables)

time_points = [t0, 1e-1, 1e0, 1e1, 1e2, tf]
time_point_colors = [
    mpl.colors.to_hex(c)
    for c in mpl.cm.Reds(np.linspace(0.3, 1, len(time_points)))]

plot_phase_portrait(
    flux_sol, x="AMP_Production", y="AMP_Consumption", ax=ax1,
    xlim=(0, 0.2), ylim=(0, 0.4),
    xlabel="Total AMP Production [mM/hr]",
    ylabel="Total AMP Consumption [mM/hr]",
    title=("(a) Total AMP Production vs. Consumption", L_FONT),
    annotate_time_points=time_points,
    annotate_time_points_color=time_point_colors);

ax1.plot([0, .33], [0, .30], "k--")
ax1.annotate("Net AMP\nProduction", xy=(0.152, 0.2), textcoords="data")
ax1.annotate("Net AMP\nConsumption", xy=(0.152, 0.1), textcoords="data")

plot_phase_portrait(
    flux_sol, x="ATP_Load_Flux", y="Net_AMP_Consumption", ax=ax2,
    xlim=(0, 4), ylim=(-0.2, 0.4),
    xlabel="ATP Load Flux [mM/hr]",
    ylabel="Net AMP Consumption [mM/hr]",
    title=("(b) ATP Load Flux vs. Net AMP Consumption", L_FONT),
    annotate_time_points=time_points,
    annotate_time_points_color=time_point_colors,
    annotate_time_points_legend="right outside");

ax2.plot([-.01, 4], [-.01, -.01], "k--");
fig_12_16.tight_layout()
```

**Figure 12.16:** Dynamic response of the combined glycolytic, pentose pathway, and AMP metabolism network to a 50% increase in the rate of ATP use. (a) The phase portrait of total AMP consumption and production fluxes. The 45 degree line is the steady state, above which there is a net consumption (i.e., efflux) from the AMP node and below which there is a net production (i.e., import) of AMP. (b) The phase portrait of ATP load and net AMP consumption fluxes. See Figures 10.15a and 11.13a for
comparison.

**2.** *Comparison with the coupled glycolytic pentose pathway network from the previous chapter:* The phase portrait trajectory is qualitatively similar to simulated responses in previous chapters where the nucleotide metabolism is not explicitly described. To get a quantitative comparison of the effects of detailing AMP metabolism, we simulate the glycolytic and pentose pathway model of the previous chapter and compare it to the integrated model developed in this chapter (see Figure 12.17).
The time response of ATP (Figure 12.17a) shows a more dampened response of the system with AMP metabolism versus the system without it. The same is true for the AMP response (Figure 12.17b) and the long term transient is less pronounced. The phase portrait of the sum of ATP consuming and producing fluxes (Figure 12.17c) shows how the fluxes come into the new steady state more quickly than when AMP metabolism is not detailed.

Thus, the additional dynamic features of AMP concentration reduce the dampened oscillations. The AMP level is able to more quickly reach the new steady state in the integrated model.

```
[44]:
```

```
t0, tf = (0, 1e2)

fig_12_17 = plt.figure(figsize=(14, 6))
gs = fig_12_17.add_gridspec(nrows=2, ncols=2, width_ratios=[1.5, 1])

ax1 = fig_12_17.add_subplot(gs[0, 0])
ax2 = fig_12_17.add_subplot(gs[1, 0])
ax3 = fig_12_17.add_subplot(gs[:, 1])


equation_dict = {
    "ATP_Consumption": ["ADK1 + PGK + PYK", ["ADK1", "PGK", "PYK"]],
    "ATP_Production": ["ATPM + HEX1 + PFK", ["ATPM", "HEX1", "PFK"]]}

time_points = [t0, 1e-1, 1e0, 1e1, tf]
time_point_colors = [
    mpl.colors.to_hex(c)
    for c in mpl.cm.Reds(np.linspace(0.3, 1, len(time_points)))]

colors = ["grey", "black"]
linestyles = ["--", "-"]
for i, model in enumerate([fullppp, core_network]):
    if i == 0:
        time_points_legend = "right outside"
    else:
        time_points_legend = None
    sim = Simulation(model)
    sim.find_steady_state(model, strategy="simulate",
                          update_values=True)
    conc_sol, flux_sol = sim.simulate(
        model, time=(t0, tf, tf*10 + 1),
        perturbations={"kf_ATPM": "kf_ATPM * 1.5"})
    for sol_id, (equation, variables) in equation_dict.items():
        flux_sol.make_aggregate_solution(
            sol_id, equation=equation, variables=variables)

    plot_time_profile(
        conc_sol, observable="atp_c", ax=ax1,
        ylim=(.08, 1.8),
        xlabel="Time [hr]", ylabel="Concentrations [mM]",
        title=("(a) ATP Concentration", L_FONT),
        linestyle=linestyles[i]);

    plot_time_profile(
        conc_sol, observable="amp_c", ax=ax2, ylim=(0, 0.55),
        xlabel="Time [hr]", ylabel="Concentrations [mM]",
        title=("(b) AMP Concentration", L_FONT),
        linestyle=linestyles[i]);

    plot_phase_portrait(
        flux_sol, x="ATP_Consumption", y="ATP_Production", ax=ax3,
        legend=(model.id, "lower right"),
        xlim=(3, 5.7), ylim=(3, 5.7),
        xlabel="ATP Consumption [mM/hr]", ylabel="ATP Production [mM/hr]",
        title=("(c) ATP Production vs. Consumption", L_FONT),
        linestyle=linestyles[i],
        annotate_time_points=time_points,
        annotate_time_points_color=time_point_colors,
        annotate_time_points_legend="upper right");
fig_12_17.tight_layout()
```

**Figure 12.17:** Dynamic response of the combined glycolytic and pentose pathway network (dashed line) and the glycolytic, pentose pathway, and AMP metabolism network (solid line) to a 50% increase in the rate of ATP use. (a) ATP concentration. (b) AMP concentration. (c) the dynamic phase portrait of ATP consumption and production fluxes.

###### Key Fluxes and all pairwise phase portraits¶

Figure 12.18 and Figure 12.19 are set up to allow the reader to examine all pairwise phase portraits After browsing through many of them, you will find that they resemble each other, showing that the variables move in a highly coordinated manner. We can study the relationship between many variables at once using multi-variate statistics.

```
[45]:
```

```
fig_12_18, ax = plt.subplots(nrows=1, ncols=1, figsize=(20, 22))
plot_tiled_phase_portraits(
    conc_sol, ax=ax, annotate_time_points_legend="lower outside");
fig_12_18.tight_layout()
```

**Figure 12.18:** Phase portraits of all the combined glycolytic, pentose phosphate pathway, and AMP salvage network species.

```
[46]:
```

```
fig_12_19, ax = plt.subplots(nrows=1, ncols=1, figsize=(20, 22))
plot_tiled_phase_portraits(
    flux_sol, ax=ax, annotate_time_points_legend="lower outside");
```

```
[46]:
```

```
<matplotlib.axes._subplots.AxesSubplot at 0x7f7fdb59ce90>
```

**Figure 12.19:** Phase portraits of all the combined glycolytic, pentose phosphate pathway, and AMP salvage network fluxes.

##### Whole Cell Models¶

The network described herein encompasses the core metabolic functions in the human red blood cell. One can continue to build metabolic processes into this network model following the procedures outlined in this chapter. To build a more comprehensive model we would have to account for more details, such as osmotic pressure and electroneutrality (Joshi 1989). The inclusion of these effects come with complex mathematics, but account for very important physiological processes. The sodium potassium
pump would also have to be accounted for as it is a key process in maintaining osmotic balance and cell shape. Another complication that arises pertains to the magnesium ion and the fact that it binds ATP to form MgATP (which is actually the substrate of many of the glycolytic enzymes) and to 23DPG to form Mg23DPG.

The metabolites also bind to macromolecules. Some macromolecules have many ligands leading to a multiplicity of bound states. The bound states will alter the properties of the macromolecules. In the next part of the book we illustrate this phenomena for hemoglobin and for a regulated enzyme, PFK.

**Figure 12.20:** Metabolic machinery and metabolic demand on the red blood cell. Redrawn from (Joshi 1989-1).

These processes can be added in a stepwise fashion to increase the scope of the model and develop it towards a whole cell model. Once comprehensive coverage of the known processes in a cell is achieved, a good physiological representation is obtained. For the simple red blood cell, such a representation is achievable and one can match the ‘metabolic machinery’ with the ‘metabolic demands’ that are placed on a cell (see Figure 12.20). Coordination is achieved through regulation. Clearly, the
metabolic network is satisfying multiple functions simultaneously, an important feature of systems biology.

##### Summary¶

- Network reconstruction proceeds in a stepwise fashion by systematically integrating sub-networks. New issues may arise during each step of the integration process.
- The addition of the AMP sub-network introduces a few integration issues. These include the reactions that have common metabolites in the two networks being integrated, new plasma exchange reactions, and the detailing of stoichiometry that may have been simplified.
- The addition of the AMP synthesis and degradation sub-network introduces five new dimensions in the null space of S where three represent the degradation of AMP and two represent the biosynthesis of AMP. New network level pathways are introduced.
- One can continue to integrate more and more biochemical processes known to occur in a cell using the procedures outlined in this chapter. For simple cells, this process can approach a comprehensive description of the cell and thus approach a whole-cell model. In addition to metabolic processes, a number of physico-chemical processes need to be added, and the functions of macromolecules can be incorporated, as described in the next part of this text.
- A large model can be analyzed and conceptualized at the three levels (biochemistry, systems biology, and physiology) as detailed for glycolysis in Chapter 10.

\(\tiny{\text{© B. Ø. Palsson 2011;}\ \text{This publication is in copyright.}\\ \text{Subject to statutory exception and to the provisions of relevant collective licensing agreements,}\\ \text{no reproduction of any part may take place without the written permission of Cambridge University Press.}}\)

#### Hemoglobin¶

A primary function of the red blood cell (RBC) is to carry oxygen bound to hemoglobin through circulation. Hemoglobin can bind to a number of small molecule ligands that affect its oxygen carrying function. The binding of small molecule ligands to a protein like hemoglobin can be described by elementary chemical equations. As we discussed in in Chapter 5, all such chemical equations can be assembled into a stoichiometric matrix that describes all the bound states of the protein. Such a
stoichiometric matrix describes the functions of a module, or a subnetwork, that has inputs and outputs. Such inputs and outputs represent the network environment in which the protein functions. These environmental parameters are known from the steady state of the network in which it operates. The stoichiometric matrix for the protein module can then be combined with stoichiometric matrix that describes the metabolic network to form a integrated network. In this chapter we describe this process
that leads to an integrated kinetic description of low molecular weight metabolites and macromolecules, using hemoglobin in the RBC as an example.

**MASSpy** will be used to demonstrate some of the topics in this chapter.

```
[1]:
```

```
from mass import (
    MassModel, MassMetabolite, MassReaction,
    Simulation, MassSolution, strip_time)
from mass.test import create_test_model
from mass.util.matrix import nullspace, left_nullspace, matrix_rank
from mass.visualization import (
    plot_time_profile, plot_phase_portrait, plot_tiled_phase_portraits,
    plot_comparison)
```

Other useful packages are also imported at this time.

```
[2]:
```

```
from os import path
from cobra import DictList
import matplotlib as mpl
import matplotlib.pyplot as plt
import numpy as np
import pandas as pd
import sympy as sym
```

Some options and variables used throughout the notebook are also declared here.

```
[3]:
```

```
pd.set_option("display.max_rows", 100)
pd.set_option("display.max_columns", 100)
pd.set_option('display.max_colwidth', None)
pd.options.display.float_format = '{:,.3f}'.format
S_FONT = {"size": "small"}
L_FONT = {"size": "large"}
INF = float("inf")
```

##### Hemoglobin: The Carrier of Oxygen¶

###### Physiological function and oxygen transport¶

RBCs account for approximately 45% of the volume of the blood. This volume fraction is known as the *hematocrit*. With a blood volume of 5 liters, a “standard man” (Seagrave, 1971) has about 2.25 Liters of RBCs, or a total of approximately \(2.5 \*10^{13}\) RBCs. Each RBCl contains approximately 30 picograms of hemoglobin.

Oxygen is carried in the blood in two forms: 1) dissolved in the plasma and, 2) bound to hemoglobin inside the RBC. Oxygen is a non-polar molecule that dissolves poorly in aqueous solution. Its solubility is only about 7 parts per million at \(37^\mathrm{o}\text{C}\). One liter of blood will thus only dissolve about 3.2 ml of oxygen. Conversely, the amount of oxygen carried by hemoglobin is much greater than the amount of oxygen dissolved in the plasma. The approximately 160 gm/liter of
hemoglobin in blood will bind to the equivalent of about 220 ml of oxygen. The ratio between hemoglobin-bound oxygen to that dissolved in plasma is about 70:1. Hemoglobin thus enables oxygen to be transported to tissues at a high rate to meet their oxygen consumption rates. An interruption in oxygen delivery, known as an ischemic event, even for just a few minutes, can have serious consequences. Systemic and prolonged shortage of oxygen is know as anemia, and there are hundreds of millions of
people on the planet that are anemic. Sufficient delivery of oxygen to tissues represents a critical physiological function.

**Figure 13.1:** (a) The structure of human hemoglobin (Koolman, 2005) (reprinted with permission). (b) An image of the packing density of hemoglobin in red cells (© David S. Goodsell 2000).

###### Hemoglobin structure and function¶

Hemoglobin is a relatively small globular protein that is a hetero-tetramer comprised of two \(\alpha\) globin and two \(\beta\) globin molecules. Its molecular weight is about 60 kD (Figure 13.1a). It is by far the most abundant protein in the RBC, accounting for approximately 97% of its protein mass (Beutler, 2001). The concentration of hemoglobin in the RBC is on the order of 7 mM. Thus, RBCs are packed with hemoglobin (Figure 13.1b).

Hemoglobin has four binding sites for oxygen. The oxygen binding site of hemoglobin is occupied by iron held in a heme group. The iron in hemoglobin represents 67% of the total iron in the human body. The hemoglobin-oxygen binding curve has a sigmoidal shape (Figure 13.2d) that results from the fact that hemoglobin’s affinity for oxygen becomes greater as each binding site is filled with oxygen, a phenomena known as *cooperativity*.

The binding curve is characterized by the p50 that is the partial pressure of oxygen at which hemoglobin is 50% saturated with oxygen (see Figure 13.2d). In other words, half of the oxygen binding sites are occupied at this partial pressure. The partial pressure of oxygen in venous blood is about 35-40 mmHg, whereas arterial blood in the lungs has partial pressure of about 90-100 mmHg. Thus hemoglobin leaves the lung saturated with oxygen, and leaves the tissue at more than half saturation.
Slightly less than half of the oxygen on the hemoglobin is delivered into tissues per pass of circulating blood.

**Figure 13.2:** Glycolysis and the binding states of hemoglobin. (a) The formation of 23DPG through the Rapoport-Luebering by-pass on glycolysis. (b) A schematic that shows how the binding of 23DPG displaces oxygen. From Koolman, 2005 (reprinted with permission). 23DPG is abbreviated as BPG in this panel. (c) The reaction schema for oxygen and 23DPG binding to hemoglobin. (d) Hemoglobin displays a sigmoidal, or S-shaped, oxygen binding curve. Normal conditions: 3.1 mM, High 23DPG: 6.0 mM, No
23DPG present: 0 mM.

###### Factors affecting oxygen binding to hemoglobin¶

The hemoglobin-oxygen dissociation curve is affected by many factors, including changes in temperature and pH (Figure 13.2b). One of the most important factors affecting the hemoglobin-oxygen binding curve is the unique RBC metabolite 2,3-biphosphoglycerate (23DPG). Increasing the concentration of 23DPG increases the p50 of hemoglobin-oxygen binding, and thus this metabolite affects hemoglobin-oxygen binding. 23DPG is abundant in RBCs and is on the order of 3 to 4 mM, close to being equimolar
with hemoglobin. It is produced in a by-pass on glycolysis, known as the Rapoport-Luebering shunt (Figure 13.2a). The production and maintenance of 23DPG to regulate the hemoglobin binding curve is a major function of RBC metabolism.

##### Describing The States of Hemoglobin¶

We can easily add the Rapoport-Luebering shunt by-pass to our metabolic model, as shown in previous chapters for other metabolic processes. However, perhaps more importantly, we need to add hemoglobin as a compound to our network. We thus add a protein molecule to the network that we are considering, forming a set of reactions that involve both small metabolites and the proteins that they interact with. To do so, we need to consider the many ligand-bound states of hemoglobin.

###### The many states of hemoglobin¶

Hemoglobin can bind to many small molecule ligands. For illustrative purposes we will consider two here: oxygen and 23DPG. As shown in Figure 13.2b, the binding of 23DPG to hemoglobin prevents oxygen molecules from binding to hemoglobin. The corresponding reaction schema is shown in Figure 13.2c and it can be used to build a chemically detailed picture of this process. Here we will assume that effectively all the oxygen is removed when 23DPG is bound to hemoglobin.

###### The ligand binding reactions¶

The binding of oxygen to hemoglobin proceeds serially giving rise to four chemical reactions:

\[\begin{equation} \text{Hb}\_0 + \text{O}\_2 \leftrightharpoons \text{Hb}\_1 \tag{13.1} \end{equation}\]

\[\begin{equation} \text{Hb}\_1 + \text{O}\_2 \leftrightharpoons \text{Hb}\_2 \tag{13.2} \end{equation}\]

\[\begin{equation} \text{Hb}\_2 + \text{O}\_2 \leftrightharpoons \text{Hb}\_3 \tag{13.3} \end{equation}\]

\[\begin{equation} \text{Hb}\_3 + \text{O}\_2 \leftrightharpoons \text{Hb}\_4 \tag{13.4} \end{equation}\]

As more oxygen molecules are bound, the easier a subsequent binding is, thus leading to cooperativity and a sigmoid binding curve, Figure 13.2d.

23DPG can bind reversibly to \(\text{Hb}\_0\) to form the deoxy state, denoted as DHb

\[\begin{equation} \text{Hb}\_0 +\text{23DPG} \leftrightharpoons \text{DHb} \tag{13.5} \end{equation}\]

To form a network, we add the inputs and outputs for the ligands. 23DPG forms and degrades as:

\[\begin{equation} \stackrel{DPGM}{\longrightarrow} \text{23DPG} \stackrel{DPGase}{\longrightarrow} \tag{13.6} \end{equation}\]

23DPG is formed by a mutase and degraded by a phosphatase. Interestingly, both these enzymatic activities are found on the same enzyme molecule.

Oxygen enters and leaves the system:

\[\text{O}\_{2, \mathrm{plasma}} \stackrel{in}{\underset{out}{\leftrightharpoons}} \text{O}\_2\]

We note that the oxygen concentration in the plasma can be a time varying function that represents the changing oxygen environment that the RBC experiences as it goes through circulation.

###### The hemoglobin subnetwork¶

This set of chemical equations can be put into the **MASSpy**, with the following commands. The *hemoglobin* model has the basic binding equations in it.

```
[4]:
```

```
hemoglobin = create_test_model("SB2_Hemoglobin")
```

**Table 13.1:** The species of the hemoglobin subnetwork, their abbreviations and steady state concentrations. The concentrations given are those typical for the human red blood cell. The index on the compounds is added to that for the glycolysis, pentose,and salvage pathways (Table 12.1).

```
[5]:
```

```
metabolite_ids = [
    m.id for m in hemoglobin.metabolites
    if m.id not in ["pi_c", "h_c", "h2o_c", "_13dpg_c", "_3pg_c"]]

table_13_1 = pd.DataFrame(
    np.array([metabolite_ids,
              [met.name for met in hemoglobin.metabolites
               if met.id in metabolite_ids],
              [hemoglobin.initial_conditions[met] for met in hemoglobin.metabolites
               if met.id in metabolite_ids]]).T,
    index=[i for i in range(41, len(metabolite_ids) + 41)],
    columns=["Abbreviations", "Species", "Initial Concentration"])
table_13_1
```

```
[5]:
```

|  | Abbreviations | Species | Initial Concentration |
| --- | --- | --- | --- |
| 41 | \_23dpg\_c | 2,3-Disphospho-D-glycerate | 3.1 |
| 42 | hb\_c | Hemoglobin | 0.0596253007092338 |
| 43 | hb\_1o2\_c | Oxyhemoglobin (1) | 0.050085289191380965 |
| 44 | hb\_2o2\_c | Oxyhemoglobin (2) | 0.07362532834168697 |
| 45 | hb\_3o2\_c | Oxyhemoglobin (3) | 0.2628417272450733 |
| 46 | hb\_4o2\_c | Oxyhemoglobin (4) | 6.807612746462968 |
| 47 | dhb\_c | Deoxyhemoglobin | 0.046209608049656195 |
| 48 | o2\_c | Oxygen | 0.0200788 |

**Table 13.2:** The reactions of the hemoglobin subnetwork enzymes and transporters, their abbreviations, chemical reactions, and equilibrium constants.The index on the reactions is added to that for the combined glycolysis, pentose, and salvage pathways (Table 12.2).

```
[6]:
```

```
reaction_ids = [r.id for r in hemoglobin.reactions]
table_13_2 = pd.DataFrame(
    np.array([reaction_ids,
              [r.name for r in hemoglobin.reactions
               if r.id in reaction_ids],
              [r.reaction for r in hemoglobin.reactions
               if r.id in reaction_ids]]).T,
    index=[i for i in range(49, len(reaction_ids) + 49)],
    columns=["Abbreviations", "Enzymes/Transporter/Load", "Elementally Balanced Reaction"])
table_13_2
```

```
[6]:
```

|  | Abbreviations | Enzymes/Transporter/Load | Elementally Balanced Reaction |
| --- | --- | --- | --- |
| 49 | DPGM | Diphosphoglyceromutase | \_13dpg\_c <=> \_23dpg\_c + h\_c |
| 50 | DPGase | Diphosphoglycerate phosphatase | \_23dpg\_c + h2o\_c --> \_3pg\_c + pi\_c |
| 51 | HBO1 | Oxygen Loading (1) | hb\_c + o2\_c <=> hb\_1o2\_c |
| 52 | HBO2 | Oxygen Loading (2) | hb\_1o2\_c + o2\_c <=> hb\_2o2\_c |
| 53 | HBO3 | Oxygen Loading (3) | hb\_2o2\_c + o2\_c <=> hb\_3o2\_c |
| 54 | HBO4 | Oxygen Loading (4) | hb\_3o2\_c + o2\_c <=> hb\_4o2\_c |
| 55 | HBDPG | Hemoglobin-23dpg binding | \_23dpg\_c + hb\_c <=> dhb\_c |
| 56 | SK\_o2\_c | Oxygen sink | o2\_c <=> |

**Table 13.3:** The elemental composition and charges of the hemoglobin module intermediates. This table represents the matrix \(\textbf{E}.\)\*

```
[7]:
```

```
table_13_3 = hemoglobin.get_elemental_matrix(array_type="DataFrame",
                                             dtype=np.int64)
table_13_3
```

```
[7]:
```

|  | \_23dpg\_c | hb\_c | hb\_1o2\_c | hb\_2o2\_c | hb\_3o2\_c | hb\_4o2\_c | dhb\_c | \_13dpg\_c | \_3pg\_c | o2\_c | pi\_c | h\_c | h2o\_c |
| --- | --- | --- | --- | --- | --- | --- | --- | --- | --- | --- | --- | --- | --- |
| C | 3 | 0 | 0 | 0 | 0 | 0 | 3 | 3 | 3 | 0 | 0 | 0 | 0 |
| H | 3 | 0 | 0 | 0 | 0 | 0 | 3 | 4 | 4 | 0 | 1 | 1 | 2 |
| O | 10 | 0 | 2 | 4 | 6 | 8 | 10 | 10 | 7 | 2 | 4 | 0 | 1 |
| P | 2 | 0 | 0 | 0 | 0 | 0 | 2 | 2 | 1 | 0 | 1 | 0 | 0 |
| N | 0 | 0 | 0 | 0 | 0 | 0 | 0 | 0 | 0 | 0 | 0 | 0 | 0 |
| S | 0 | 0 | 0 | 0 | 0 | 0 | 0 | 0 | 0 | 0 | 0 | 0 | 0 |
| q | -5 | 0 | 0 | 0 | 0 | 0 | -5 | -4 | -3 | 0 | -2 | 1 | 0 |
| [HB] | 0 | 1 | 1 | 1 | 1 | 1 | 1 | 0 | 0 | 0 | 0 | 0 | 0 |

**Table 13.4:** The elemental and charge balance test on the reactions. All internal reactions are balanced. Exchange reactions are not.

```
[8]:
```

```
table_13_4 = hemoglobin.get_elemental_charge_balancing(array_type="DataFrame",
                                                       dtype=np.int64)
table_13_4
```

```
[8]:
```

|  | DPGM | DPGase | HBO1 | HBO2 | HBO3 | HBO4 | HBDPG | SK\_o2\_c |
| --- | --- | --- | --- | --- | --- | --- | --- | --- |
| C | 0 | 0 | 0 | 0 | 0 | 0 | 0 | 0 |
| H | 0 | 0 | 0 | 0 | 0 | 0 | 0 | 0 |
| O | 0 | 0 | 0 | 0 | 0 | 0 | 0 | -2 |
| P | 0 | 0 | 0 | 0 | 0 | 0 | 0 | 0 |
| N | 0 | 0 | 0 | 0 | 0 | 0 | 0 | 0 |
| S | 0 | 0 | 0 | 0 | 0 | 0 | 0 | 0 |
| q | 0 | 0 | 0 | 0 | 0 | 0 | 0 | 0 |
| [HB] | 0 | 0 | 0 | 0 | 0 | 0 | 0 | 0 |

Table 13.4 shows that \(\textbf{ES} = 0\) for all non-exchange reactions in the hemoglobin. Thus the reactions are charge and elementally balanced. The model passes this QC/QA test.

```
[9]:
```

```
for boundary in hemoglobin.boundary:
    print(boundary)
```

```
SK_o2_c: o2_c <=>
```

###### Placing the Hemoglobin module into a known network environment¶

We will analyze the hemoglobin module by itself first. To do so, we have to add exchange reactions from the network environment first. The numerical values for the concentrations external to the hemoglobin module have to be added. We can then explore the contents of this model of the module alone.

```
[10]:
```

```
for met, ext_conc in zip(["_13dpg_c", "_3pg_c", "pi_c", "h_c", "h2o_c"],
                         [0.000243, 0.0773, 2.5, 8.99757e-5, 1]):

    hemoglobin.add_boundary(met, boundary_type="sink",
                            boundary_condition=ext_conc)

for rxn in hemoglobin.boundary:
    if rxn.id != "SK_o2_e":
        rxn.Keq = 1
        rxn.kf = 100000
```

###### The pathway structure: basis for the null space¶

The dimensions of this matrix are 13x13 and its rank is 12. There is one pathway, the Rapoport-Luebering shunt, that spans the null space of the stoichiometric matrix, see Table 13.5. Note the many exchange fluxes that describe this pathway.

**Table 13.5:** The pathway vector for the stoichiometric matrix for hemoglobin subnetwork.

```
[11]:
```

```
reaction_ids = [r.id for r in hemoglobin.reactions]
ns = nullspace(hemoglobin.S).T
# Iterate through nullspace,
# dividing by the smallest value in each row.
for i, row in enumerate(ns):
    minval = np.min(abs(row[np.nonzero(row)]))
    new_row = np.array(row/minval)
    # Round to ensure the left nullspace is composed of only integers
    ns[i] = np.array([round(value) for value in new_row])

# Ensure positive stoichiometric coefficients if all are negative
for i, space in enumerate(ns):
    ns[i] = np.negative(space) if all([num <= 0 for num in space]) else space
ns = ns.T.astype(np.int64)
# Create label
path_labels = ["R.L. Shunt"]
table_13_5 = pd.DataFrame(ns, index=reaction_ids,
                          columns=path_labels).T
table_13_5
```

```
[11]:
```

|  | DPGM | DPGase | HBO1 | HBO2 | HBO3 | HBO4 | HBDPG | SK\_o2\_c | SK\_\_13dpg\_c | SK\_\_3pg\_c | SK\_pi\_c | SK\_h\_c | SK\_h2o\_c |
| --- | --- | --- | --- | --- | --- | --- | --- | --- | --- | --- | --- | --- | --- |
| R.L. Shunt | 1 | 1 | 0 | 0 | 0 | 0 | 0 | 0 | -1 | 1 | 1 | 1 | -1 |

###### The time invariant pools: the basis for the left null space¶

There is one time invariant, that is simply the total amount of hemoglobin. Hemoglobin does not leave or enter the module and thus stays a constant.

**Table 13.6:** The left null space composed of the time invariant Hemoglobin pool.

```
[12]:
```

```
metabolite_ids = [m.id for m in hemoglobin.metabolites]
lns = left_nullspace(hemoglobin.S, rtol=1e-10)
# Iterate through left nullspace,
# dividing by the smallest value in each row.
for i, row in enumerate(lns):
    minval = np.min(abs(row[np.nonzero(row)]))
    new_row = np.array(row/minval)
    # Round to ensure the left nullspace is composed of only integers
    lns[i] = np.array([round(value) for value in new_row])

# Ensure positive stoichiometric coefficients if all are negative
for i, space in enumerate(lns):
    lns[i] = np.negative(space) if all([num <= 0 for num in space]) else space
lns = lns.astype(np.int64)
# Create labels for the time invariants
time_inv_labels = ["Hb-Total"]
table_13_6 = pd.DataFrame(lns, index=time_inv_labels,
                          columns=metabolite_ids, dtype=np.int64)
table_13_6
```

```
[12]:
```

|  | \_23dpg\_c | hb\_c | hb\_1o2\_c | hb\_2o2\_c | hb\_3o2\_c | hb\_4o2\_c | dhb\_c | \_13dpg\_c | \_3pg\_c | o2\_c | pi\_c | h\_c | h2o\_c |
| --- | --- | --- | --- | --- | --- | --- | --- | --- | --- | --- | --- | --- | --- |
| Hb-Total | 0 | 1 | 1 | 1 | 1 | 1 | 1 | 0 | 0 | 0 | 0 | 0 | 0 |

You can also look up the contents of the null spaces from the table of attributes of the model. The pool is shown to the right of Table 13.7 and the pathway vector towards the bottom of the table.

###### An ‘annotated’ form of the stoichiometric matrix¶

All of the properties of the stoichiometric matrix can be conveniently summarized in a tabular format, Table 13.7. The table succinctly summarizes the chemical and topological properties of S. The matrix has dimensions of 13x13 and a rank of 12. It thus has a one dimensional null space and a one dimensional left null space.

**Table 13.7:** The stoichiometric matrix for the hemoglobin seen in Figure 13.2. The matrix is partitioned to show the intermediates (yellow) separate from the cofactors and to separate the exchange reactions and cofactor loads (orange). The connectivities, \(\rho\_i\) (red), for a compound, and the participation number, \(pi\_j\) (cyan), for a reaction are shown. The second block in the table is the product \(\textbf{ES}\) (blue) to evaluate elemental balancing status of the
reactions. All exchange reactions have a participation number of unity and are thus not elementally balanced. The last block in the table has the pathway vector (purple) for the hemoglobin module. Furthest to the right, we display the time invariant pool(green) that spans the left null space.

```
[13]:
```

```
# Define labels
pi_str = r"$\pi_{j}$"
rho_str = r"$\rho_{i}$"
# Include hemoglobin moiety
chopsnq = ['C', 'H', 'O', 'P', 'N', 'S', 'q', "[Hb]"]

# Make table content from the stoichiometric matrix, elemental balancing of pathways
# participation number, and MinSpan pathways
S_matrix = hemoglobin.update_S(array_type="dense", dtype=np.int64, update_model=False)
ES_matrix = hemoglobin.get_elemental_charge_balancing(dtype=np.int64)
pi = np.count_nonzero(S_matrix, axis=0)
rho = np.count_nonzero(S_matrix, axis=1)
table_13_7 = np.vstack((S_matrix, pi, ES_matrix, ns.T))

# Determine number of blank entries needed to be added to pad the table,
# Add connectivity number and time invariants to table content
blanks = [""]*(len(table_13_7) - len(hemoglobin.metabolites))
rho = np.concatenate((rho, blanks))
time_inv = np.array([np.concatenate([row, blanks]) for row in lns])
table_13_7 = np.vstack([table_13_7.T, rho, time_inv]).T

colors = {"intermediates": "#ffffe6", # Yellow
          "cofactors": "#ffe6cc",     # Orange
          "chopsnq": "#99e6ff",       # Blue
          "pathways": "#b399ff",      # Purple
          "pi": "#99ffff",            # Cyan
          "rho": "#ff9999",           # Red
          "time_invs": "#ccff99",     # Green
          "blank": "#f2f2f2"}         # Grey
bg_color_str = "background-color: "
def highlight_table(df, model, main_shape):
    df = df.copy()
    n_mets, n_rxns = (len(model.metabolites), len(model.reactions))
    # Highlight rows
    for row in df.index:
        other_key, condition = ("blank", lambda i, v: v != "")
        if row == pi_str:        # For participation
            main_key = "pi"
        elif row in chopsnq:     # For elemental balancing
            main_key = "chopsnq"
        elif row in path_labels: # For pathways
            main_key = "pathways"
        else:
            # Distinguish between intermediate and cofactor reactions for model reactions
            main_key, other_key = ("cofactors", "intermediates")
            condition = lambda i, v: (main_shape[1] <= i and i < n_rxns)
        df.loc[row, :] = [bg_color_str + colors[main_key] if condition(i, v)
                          else bg_color_str + colors[other_key]
                          for i, v in enumerate(df.loc[row, :])]

    for col in df.columns:
        condition = lambda i, v: v != bg_color_str + colors["blank"]
        if col == rho_str:
            main_key = "rho"
        elif col in time_inv_labels:
            main_key = "time_invs"
        else:
            # Distinguish intermediates and cofactors for model metabolites
            main_key = "cofactors"
            condition = lambda i, v: (main_shape[0] <= i and i < n_mets)
        df.loc[:, col] = [bg_color_str + colors[main_key] if condition(i, v)
                          else v for i, v in enumerate(df.loc[:, col])]
    return df

# Create index and column labels
index_labels = np.concatenate((metabolite_ids, [pi_str], chopsnq, path_labels))
column_labels = np.concatenate((reaction_ids, [rho_str], time_inv_labels))
# Create DataFrame
table_13_7 = pd.DataFrame(
    table_13_7, index=index_labels, columns=column_labels)
# Apply colors
table_13_7 = table_13_7.style.apply(
    highlight_table,  model=hemoglobin, main_shape=(9, 7), axis=None)
table_13_7
```

```
[13]:
```

|  | DPGM | DPGase | HBO1 | HBO2 | HBO3 | HBO4 | HBDPG | SK\_o2\_c | SK\_\_13dpg\_c | SK\_\_3pg\_c | SK\_pi\_c | SK\_h\_c | SK\_h2o\_c | $\rho\_{i}$ | Hb-Total |
| --- | --- | --- | --- | --- | --- | --- | --- | --- | --- | --- | --- | --- | --- | --- | --- |
| \_23dpg\_c | 1 | -1 | 0 | 0 | 0 | 0 | -1 | 0 | 0 | 0 | 0 | 0 | 0 | 3 | 0 |
| hb\_c | 0 | 0 | -1 | 0 | 0 | 0 | -1 | 0 | 0 | 0 | 0 | 0 | 0 | 2 | 1 |
| hb\_1o2\_c | 0 | 0 | 1 | -1 | 0 | 0 | 0 | 0 | 0 | 0 | 0 | 0 | 0 | 2 | 1 |
| hb\_2o2\_c | 0 | 0 | 0 | 1 | -1 | 0 | 0 | 0 | 0 | 0 | 0 | 0 | 0 | 2 | 1 |
| hb\_3o2\_c | 0 | 0 | 0 | 0 | 1 | -1 | 0 | 0 | 0 | 0 | 0 | 0 | 0 | 2 | 1 |
| hb\_4o2\_c | 0 | 0 | 0 | 0 | 0 | 1 | 0 | 0 | 0 | 0 | 0 | 0 | 0 | 1 | 1 |
| dhb\_c | 0 | 0 | 0 | 0 | 0 | 0 | 1 | 0 | 0 | 0 | 0 | 0 | 0 | 1 | 1 |
| \_13dpg\_c | -1 | 0 | 0 | 0 | 0 | 0 | 0 | 0 | -1 | 0 | 0 | 0 | 0 | 2 | 0 |
| \_3pg\_c | 0 | 1 | 0 | 0 | 0 | 0 | 0 | 0 | 0 | -1 | 0 | 0 | 0 | 2 | 0 |
| o2\_c | 0 | 0 | -1 | -1 | -1 | -1 | 0 | -1 | 0 | 0 | 0 | 0 | 0 | 5 | 0 |
| pi\_c | 0 | 1 | 0 | 0 | 0 | 0 | 0 | 0 | 0 | 0 | -1 | 0 | 0 | 2 | 0 |
| h\_c | 1 | 0 | 0 | 0 | 0 | 0 | 0 | 0 | 0 | 0 | 0 | -1 | 0 | 2 | 0 |
| h2o\_c | 0 | -1 | 0 | 0 | 0 | 0 | 0 | 0 | 0 | 0 | 0 | 0 | -1 | 2 | 0 |
| $\pi\_{j}$ | 3 | 4 | 3 | 3 | 3 | 3 | 3 | 1 | 1 | 1 | 1 | 1 | 1 |  |  |
| C | 0 | 0 | 0 | 0 | 0 | 0 | 0 | 0 | -3 | -3 | 0 | 0 | 0 |  |  |
| H | 0 | 0 | 0 | 0 | 0 | 0 | 0 | 0 | -4 | -4 | -1 | -1 | -2 |  |  |
| O | 0 | 0 | 0 | 0 | 0 | 0 | 0 | -2 | -10 | -7 | -4 | 0 | -1 |  |  |
| P | 0 | 0 | 0 | 0 | 0 | 0 | 0 | 0 | -2 | -1 | -1 | 0 | 0 |  |  |
| N | 0 | 0 | 0 | 0 | 0 | 0 | 0 | 0 | 0 | 0 | 0 | 0 | 0 |  |  |
| S | 0 | 0 | 0 | 0 | 0 | 0 | 0 | 0 | 0 | 0 | 0 | 0 | 0 |  |  |
| q | 0 | 0 | 0 | 0 | 0 | 0 | 0 | 0 | 4 | 3 | 2 | -1 | 0 |  |  |
| [Hb] | 0 | 0 | 0 | 0 | 0 | 0 | 0 | 0 | 0 | 0 | 0 | 0 | 0 |  |  |
| R.L. Shunt | 1 | 1 | 0 | 0 | 0 | 0 | 0 | 0 | -1 | 1 | 1 | 1 | -1 |  |  |

###### Pools and ratios for hemoglobin¶

Hemoglobin will have two basic forms: the oxy-form (i.e., OHb bound to oxygen) and the deoxy-form (i.e., DHb bound to 23DPG). The total amount of oxygen carried on hemoglobin (i.e., the occupancy) at any given time is

\[\begin{equation} \text{OHb} = \text{Hb}\_1 + 2\text{Hb}\_2 + 3\text{Hb}\_3 + 4\text{Hb}\_4 \tag{13.7} \end{equation}\]

while the oxygen carrying capacity is \(4\text{Hb}\_{\mathrm{tot}}\). We can thus define a ratio:

\[\begin{equation} r\_{OHb} = \frac{\text{OHb}}{4\text{Hb}\_{\mathrm{tot}}} = \frac{\text{Hb}\_1 + 2\text{Hb}\_2 + 3\text{Hb}\_3 + 4\text{Hb}\_4}{4\text{Hb}\_{\mathrm{tot}}} \tag{13.8} \end{equation}\]

as the fractional oxygen saturation, or the oxygen charge of hemoglobin, in analogy the Energy Charge introduced earlier in the text. The total amount of hemoglobin is a constant, at circa 7 mM. This number represents the size of the time invariant in the left null space (Eq. 13.13)

Similarly we can define the pool of 23DPG as, as it is either free or bound to hemoglobin:

\[\begin{equation} \text{23DPG}\_{\mathrm{tot}} = \text{DHb} + \text{23DPG} \tag{13.9} \end{equation}\]

This total pool will be time dependent, unlike that of total hemoglobin. We can thus define the state of 23DPG as a regulator by the ratio

\[\begin{equation} r\_{23DPG} = \frac{\text{DHb}}{\text{23DPG}\_{\mathrm{tot}}} = \frac{\text{DHb}}{\text{DHb} + \text{23DPG}} \tag{13.10} \end{equation}\]

These two ratios, \(r\_{OHb}\) and \(r\_{23DPG}\), describe the two functional states of hemoglobin and they will respond to environmental and metabolic perturbations.

###### The steady state¶

The binding of the two ligands, oxygen and 23DPG, to hemoglobin is a rapid process. Since hemoglobin is confined to the RBC, we can use equilibrium assumptions for the binding reactions. The binding of oxygen is at equilibrium

\[\begin{equation} K\_1 = \frac{\text{HB}\_{1}}{\text{HB}\_{0}\*\text{O}\_{2}},\ K\_2 = \frac{\text{HB}\_{2}}{\text{HB}\_{1}\*\text{O}\_{2}},\ K\_3 = \frac{\text{HB}\_{3}}{\text{HB}\_{2}\*\text{O}\_{2}},\ K\_4 = \frac{\text{HB}\_{4}}{\text{HB}\_{3}\*\text{O}\_{2}} \tag{13.11} \end{equation}\]

The binding of 23DPG to hemoglobin is also at equilibrium

\[\begin{equation} K\_d = \frac{\text{DHB}}{\text{HB}\_{0}\*\text{23PDG}} \tag{13.12} \end{equation}\]

The numerical values for the equilibrium constants are given in Table 13.2. The total mass of hemoglobin is a constant

\[\begin{equation} \text{Hb}\_{\mathrm{tot}} = \text{HB}\_{0} + \text{HB}\_{1} + \text{HB}\_{2} + \text{HB}\_{3} + \text{HB}\_{4} + \text{DHB} \tag{13.13} \end{equation}\]

These six equations have six unknowns (the six forms of Hb) and need to be solved simultaneously as a function of the oxygen and 23DPG concentrations. The equilibrium relationships can be combined with the \(\text{HB}\_{0}\) mass balance to:

\[\begin{equation} \text{Hb}\_{\mathrm{tot}} = \text{HB}\_{0}(1 + K\_1\text{O}\_2 + K\_1K\_2\text{O}\_2^2 + K\_1K\_2K\_3\text{O}\_2^3 + K\_1K\_2K\_3K\_4\text{O}\_2^4 + K\_d\text{23DPG}) \tag{13.14} \end{equation}\]

This equation is solved for \(\text{HB}\_{0}\) for given oxygen and 23DPG concentrations. Then all the other forms of hemoglobin can be computed from the equilibrium relationships.

To do this, the equilibrium constants are defined and then the equilibrium expressions are converted into `sympy.Equality` objects for symbolic calculations.

```
[14]:
```

```
hb_total_sym = sym.Symbol("Hb-Total")
met_symbols = {met: sym.Symbol(met) for met in metabolite_ids}
# Iterate through reactions assumed to be at equilibrium
binding_reactions = hemoglobin.reactions.get_by_any(["HBO1", "HBO2", "HBO3", "HBO4", "HBDPG"])

# Initialize a dict to store equations for heme products
heme_product_equations = {}
for rxn in binding_reactions:
    reactants = "*".join([met.id for met in rxn.reactants])
    # Find the hemoglobin form being made as a product (bound to most oxygen)
    heme_product = sym.Symbol(rxn.products[0].id)
    # Set up the equilibrium equation
    equation = sym.Eq(sym.Symbol(rxn.Keq_str),
                      sym.sympify("({0}) / ({1})".format(heme_product, reactants),
                                  locals=met_symbols))
    # Solve the equation for the desired form of hemoglobin
    equation = list(sym.solveset(equation, heme_product)).pop()
    # Substitute previously solved heme product equations into the current one
    heme_product_equations.update({heme_product: equation.subs(heme_product_equations)})

# Specify an equation for the total amount of hemoglobin.
hb_total_equation = "+".join([met for met in metabolite_ids if "hb" in met])
hb_total_equation = sym.Eq(hb_total_sym, sym.sympify(hb_total_equation, locals=met_symbols))
# Substitute in equations for each bound form to have total hemoglobin as a function of
# oxygen, free hemoglobin, and 23dpg concentrations.
hb_total_equation = hb_total_equation.subs(heme_product_equations)
sym.pprint(hb_total_equation)
```

```
Hb-Total = Keq_HBDPG⋅_23dpg_c⋅hb_c + Keq_HBO1⋅Keq_HBO2⋅Keq_HBO3⋅Keq_HBO4⋅hb_c⋅

     4                                        3
o_2_c  + Keq_HBO1⋅Keq_HBO2⋅Keq_HBO3⋅hb_c⋅o_2_c  + Keq_HBO1⋅Keq_HBO2⋅hb_c⋅o_2_c

2
  + Keq_HBO1⋅hb_c⋅o_2_c + hb_c
```

At this point, the numerical values for the equilibrium constant and the total concetration of hemoglobin are specified. The total amount of hemoglobin is 7.3 mM. These values are substituted into the current equations.

```
[15]:
```

```
numerical_values = {hb_total_sym: 7.3}
numerical_values.update({sym.Symbol(Keq): value
                         for Keq, value in hemoglobin.parameters["Keq"].items()})
heme_product_equations = {heme_product: equation.subs(numerical_values)
                          for heme_product, equation in heme_product_equations.items()}
hb_total_equation = hb_total_equation.subs(numerical_values)
sym.pprint(hb_total_equation)
```

```
                                                     4
7.3 = 0.25⋅_23dpg_c⋅hb_c + 702446487.27335⋅hb_c⋅o_2_c  + 544565.932207695⋅hb_c

      3                          2
⋅o_2_c  + 3062.8177448⋅hb_c⋅o_2_c  + 41.8352⋅hb_c⋅o_2_c + hb_c
```

To find the steady state, we have to specify the numerical values of the variables that characterize the network environment. The flux through the Rapoport-Luebering shunt is typically about 0.44 mM/hr (Schrader 1993). The steady state concentration of 23DPG is typically about 3.1 mM (Mehta 2005). The concentration of oxygen that we chose to solve for the steady state is 70 mmHg, that is mid way between 100 mmHg in the lung, and 40 mmHg in tissue. Using these numbers, the computed steady state
concentrations are obtained, as:

```
[16]:
```

```
# Define known concentrations
concentrations = {met_symbols["_23dpg_c"]: 3.1,
                  met_symbols["o2_c"]: 70*2.8684*1e-4}
# Solve for concentration of free hb, add to the concentration dict
sol = list(sym.solveset(hb_total_equation.subs(concentrations), met_symbols["hb_c"])).pop()
concentrations.update({met_symbols["hb_c"]: float(sol)})
# Determine concentrations for the remaining hemoglobin forms
for heme_product, equation in heme_product_equations.items():
    equation = equation.subs(concentrations)
    concentrations.update({heme_product: float(equation)})
```

Once the steady state concentrations have been determined, the hemoglobin module can be updated.

```
[17]:
```

```
for met, value in concentrations.items():
    met = hemoglobin.metabolites.get_by_id(str(met))
    met.ic = value
    print("{0}: {1:.6f}".format(met.id, met.ic))
```

```
_23dpg_c: 3.100000
o2_c: 0.020079
hb_c: 0.059625
hb_1o2_c: 0.050085
hb_2o2_c: 0.073625
hb_3o2_c: 0.262842
hb_4o2_c: 6.807613
dhb_c: 0.046210
```

Here we check to make sure that we are at a steady state.

```
[18]:
```

```
t0, tf = (0, 50)
sim_hb = Simulation(hemoglobin)
conc_sol, flux_sol = sim_hb.simulate(
    hemoglobin, time=(t0, tf, tf*10 + 1))

equation_dict = {
    "$r_{OHb}$": [
        "(hb_1o2_c + 2*hb_2o2_c + 3*hb_3o2_c + 4*hb_4o2_c)/(4 * {0})".format(numerical_values[hb_total_sym]),
        ["hb_1o2_c", "hb_2o2_c", "hb_3o2_c", "hb_4o2_c"]],
    "$r_{23DPG}$": [
        "dhb_c/(_23dpg_c + dhb_c)",
        ["dhb_c", "_23dpg_c"]]}

for ratio_id, (equation, variables) in equation_dict.items():
    conc_sol.make_aggregate_solution(
        ratio_id, equation, variables)

fig, axes = plt.subplots(nrows=2, ncols=2, figsize=(12, 8))
(ax1, ax2, ax3, ax4) = axes.flatten()

plot_time_profile(
    conc_sol, observable=list(equation_dict)[0], ax=ax1,
    xlabel="Time [hr]", ylabel="Ratio",
    xlim=(t0, tf), ylim=(0.9, 1),
    title=(list(equation_dict)[0], L_FONT));

plot_time_profile(
    flux_sol, observable="SK_o2_c", ax=ax2,
    xlabel="Time [hr]", ylabel="Flux [mM/hr]",
    xlim=(t0, tf), ylim=(-1, 1),
    title=("$v_{O_2}$", L_FONT));

plot_time_profile(
    conc_sol, observable="_23dpg_c", ax=ax3,
    xlabel="Time [hr]", ylabel="Concentration [mM]",
    xlim=(t0, tf), ylim=(3.0, 3.2),
    title=("_23dpg_c", L_FONT));

plot_time_profile(
    conc_sol, observable=list(equation_dict)[1], ax=ax4,
    xlabel="Time [hr]", ylabel="Ratio",
    xlim=(t0, tf), ylim=(0, .02),
    title=(list(equation_dict)[1], L_FONT));
fig.tight_layout()
```

We note that the slight decrease in the 23DPG concentration and update the condition accordingly.

```
[19]:
```

```
sim_hb.find_steady_state(
    hemoglobin, strategy="simulate", update_values=True);
```

```
[19]:
```

```
(<MassSolution Hemoglobin_ConcSols at 0x7fa02074f1d0>,
 <MassSolution Hemoglobin_FluxSols at 0x7fa020782bf0>)
```

The two ratios that characterize the state of the hemoglobin module are computed to be:

```
[20]:
```

```
for ratio_id in list(equation_dict):
    print("{0}: {1:.3f}".format(ratio_id, conc_sol[ratio_id][-1]))
```

```
$r_{OHb}$: 0.966
$r_{23DPG}$: 0.015
```

\[\begin{equation} r\_{OHb} = 0.97,\ r\_{23DPG} = 0.015 \tag{13.15} \end{equation}\]

Thus, in this steady state, the oxygen carrying capacity of hemoglobin is 97% utilized. Only 1.5% of the 23DPG is bound to hemoglobin. At this low 23DPG loading, the sequestration of hemoglobin into the deoxy form is highly sensitive to changes in the concentration of the 23DPG, making it an effective regulator. Both of these features meet expectations of physiological function, i.e., almost full use of oxygen carrying capacity while being highly sensitive to a key regulatory signal.

###### Dynamic responses¶

The oxygenated state of hemoglobin will respond to the partial pressure of oxygen in the plasma. At sea level, the partial pressure of oxygen in blood will change from a high of about 100 mmHg leaving the lung and 40 mmHg in venus blood leaving tissues. In the dynamic simulations below, we will use the average oxygen partial pressure of 70 mmHg and an oscillation from 40 to 100 mmHg as a time-variant environ- ment during circulations. The average partial pressure can be dropped to simulate
increased altitude.

The dynamic response of this system around the defined steady state can be performed. There are two cases of particular interest. First would be physiologically meaningful oscillations in the oxygen concentrations in the plasma, and second, changes to the average oxygen concentration in the plasma that would, for instance, correspond to significant altitude changes. The former is rapid while the latter is slow.

###### Normal circulation¶

The average circulation time of a RBC in the human body is about 1 minute. The partial pressure of oxygen in plasma should vary between 100 mmHg and 40 mmHg. To simulate the circulation cycle, we pick a sinusoidal oscillatory forcing function on the oxygen plasma and simulate the response. We center the oscillation on 70 mmHg as the midway point. The reader can formulate different forcing functions that are more physiological, keeping in mind that the average capillary transit time is on the
order of one second. In studying a forcing function that represent such a rapid transit time, one has to be cognizant of that the numerical values of the hemoglobin loading and unloading processes are.

The simulated response of the state of the hemoglobin molecule to a sinusoidal forcing function is shown in Figure 13.3. The fractional oxygen loading, \(r\_{OHb}\), shows how hemoglobin is highly saturated at 100 mmHg and that it drops to a low level at 40 mmHg, Figure 13.3a. The exchange of oxygen in and out of the hemoglobin module shows how oxygen might be flowing in and out of the RBC during the simulated circulation cycle, Figure 13.3b. Integrating the \(v\_{O\_2}\) curve shows that
the oxygen loading and delivery is 2.8 mM/L/cycle. For a blood volume of 4.5 liters, this corresponds to 12.6 mmol/cycle or 0.76 mol/h for 60 cycles per hour. This number is in the range of oxygen consumption for an adult male.

```
[21]:
```

```
(t0, tf) = (0, 0.5)

conc_sol, flux_sol = sim_hb.simulate(
    hemoglobin, time=(t0, tf, 1e6+1),
    perturbations={"o2_b": "(70 + 30*sin(120*pi*t))*2.8684*1e-4"},
    interpolate=True)

fig_13_3, axes = plt.subplots(nrows=2, ncols=2, figsize=(12, 8))
(ax1, ax2, ax3, ax4) = axes.flatten()


for ratio_id, (equation, variables) in equation_dict.items():
    conc_sol.make_aggregate_solution(
        ratio_id, equation, variables)

plot_time_profile(
    conc_sol, observable=list(equation_dict)[0], ax=ax1,
    xlabel="Time [hr]", ylabel="Ratio",
    xlim=(t0, tf), ylim=(0.9, 1),
    title=(list(equation_dict)[0], L_FONT));

plot_time_profile(
    flux_sol, observable="SK_o2_c", ax=ax2,
    xlabel="Time [hr]", ylabel="Flux [mM/hr]",
    xlim=(t0, tf), ylim=(-600, 600),
    title=("$v_{O_2}$", L_FONT));

plot_time_profile(
    conc_sol, observable="_23dpg_c", ax=ax3,
    xlabel="Time [hr]", ylabel="Concentration [mM]",
    xlim=(t0, tf), ylim=(2.9, 3.1),
    title=("_23dpg_c", L_FONT));

plot_time_profile(
    conc_sol, observable=list(equation_dict)[1], ax=ax4,
    xlabel="Time [hr]", ylabel="Ratio",
    xlim=(t0, tf), ylim=(0, .06),
    title=(list(equation_dict)[1], L_FONT));
fig_13_3.tight_layout()
```

**Figure 13.3:** The binding states of hemoglobin during normal circulation. (a) \(r\_{OHb}\). (b) \(v\_{O\_2}\). (c) 23DPG concentration. (d) 23DPG loading of hemoglobin.

The loading and unloading of oxygen on hemoglobin can be visualized by graphing the oxygen loading \(r\_{OHb}\) versus the plasma partial pressure of oxygen on the same plot as the saturation curve, Figure 13.4a. The displacement of 23DPG during circulation is shown in Figure 13.3c and \(r\_{23DPG}\) is graphed in Figure 13.3d. We see how the loading and unloading of oxygen on hemoglobin is a cycle between the saturation curves that correspond the the maximum and minimum concentration of
23DPG during the cycle. Note how narrow the range of operation is, but is sufficient to get high rates of oxygen delivery from the lung to the tissues. Finally, it is useful to look at the \(r\_{OHb}\) vs. \(r\_{23DPG}\) when they are plotted against each other, Figure 13.4b.

```
[22]:
```

```
def saturation_curve(points):
    sol = np.zeros(points.shape)
    # Specify an equation for the oxygen occupancy and capacity of hemoglobin
    Hb_occupancy = sym.sympify(
        "+".join([
            "1*hb_1o2_c", "2*hb_2o2_c", "3*hb_3o2_c", "4*hb_4o2_c" ]),
        locals=met_symbols)
    Hb_capacity = sym.sympify(
        "+".join([
            met for met in metabolite_ids if "hb" in met]),
        locals=met_symbols)*4
    ratio = Hb_occupancy/Hb_capacity
    ratio = ratio.subs(heme_product_equations)
    ratio = ratio.subs({
        met: ic for met, ic in concentrations.items()
        if str(met) != "o2_c" and str(met) != "_23dpg_c"})

    for i, x in enumerate(points): # np.linspace(30, 105, 1000)
        sol[i] = float(ratio.subs({met_symbols["_23dpg_c"]: 0,
                                   met_symbols["o2_c"]: x*2.8684*1e-4}))

    return sol

npoints = 250

fig_13_4, axes = plt.subplots(nrows=1, ncols=2, figsize=(10, 5))
(ax1, ax2) = axes.flatten()

# Plot saturation curve
ax1.plot([(70 + 30*np.sin(120*np.pi*t))
          for t in np.linspace(t0, tf, npoints)],
          conc_sol[list(equation_dict)[0]](np.linspace(t0, tf, npoints)),
         "k")

ax1.plot(np.linspace(30, 105, npoints),
         saturation_curve(np.linspace(30, 105, npoints)),
         "r--", label="Saturation Curve")

# Annotate plot
ax1.annotate("$r_{OHb}$ < Saturation\n Oxygen Loading", xy=(70, .9));
ax1.annotate("$r_{OHb}$ > Saturation\n Oxygen Delivery", xy=(40, .98));
ax1.set_xlim(30, 105);
ax1.set_ylim(0.85, 1);
ax1.set_title("(a) $r_{OHb}$ vs. $pO_{2}$", fontdict=L_FONT);
ax1.legend(loc="lower right");

plot_phase_portrait(
    conc_sol, x=list(equation_dict)[1], y=list(equation_dict)[0], ax=ax2,
    xlabel=list(equation_dict)[1], ylabel=list(equation_dict)[0],
    title=("(b) {0} vs. {1}".format(
        list(equation_dict)[1], list(equation_dict)[0]),
           L_FONT),
    annotate_time_points="endpoints",
    annotate_time_points_labels=True);
fig_13_4.tight_layout()
```

**Figure 13.4:** The dynamic version of the hemoglobin binding curve. (a) \(r\_{OHb}\) vs. \(pO\_2\) in mmHg cycle shown with the hemoglobin saturation curve. (b) The oscillation in the fraction of the 23DPG regulator bound to hemoglobin as a function of \(r\_{OHb}\).

###### Response to altitude change¶

The second situation of interest is a change in the average partial pressure of oxygen. Here, we drop it to 38 mmHg, representing a move to 12000 ft elevation [4]. This change induces a long-term change in the DPG23 concentration (see Figure 13.5) that in turn induces a left shift in the oxygen–Hb binding curve; Figure 13.2.

```
[23]:
```

```
(t0, tf) = (0, 40)

conc_sol, flux_sol = sim_hb.simulate(
    hemoglobin, time=(t0, tf, tf*100 + 1),
    perturbations={"o2_b": "o2_b/70 * 47"})

fig_13_5, axes = plt.subplots(nrows=1, ncols=2, figsize=(12, 4), sharey=True)
(ax1, ax2) = axes.flatten()
plot_time_profile(
    conc_sol, observable="_23dpg_c", ax=ax1,
    xlim=(t0, 0.1), xlabel="Time [hr]", ylabel="Concentration [mM]",
    title=("_23dpg_c Response to Altitude Change (Fast)", L_FONT))

plot_time_profile(
    conc_sol, observable="_23dpg_c", ax=ax2,
    xlim=(t0, tf), xlabel="Time [hr]", ylabel="Concentration [mM]",
    title=("_23dpg_c Response to Altitude Change (Slow)", L_FONT))

ax2.annotate("0 h - 3.10 mM", xy=(30, 2.95));
ax2.annotate("0.034 h - 2.76 mM", xy=(30, 2.94));
ax2.annotate("20 h - 3.01 mM", xy=(30, 2.93));
fig_13_5.tight_layout()
```

**Figure 13.5:** Dynamic response of the 23DPG concentration when the average partial pressure of oxygen drops from 70 mmHg at sea level to to 38 mmHg at 12,000 feet.

##### Integration with the Core Metabolic Network¶

The unique red blood cell metabolite 23DPG is produced by a bypass on glycolysis. This bypass can be integrated with the combined core metabolic model of Chapter 12. This integration is seamless as 23DPG will leave from the 13DPG node and re-enter at the 3PG node.

**Figure 13.6:** The core metabolic network in the human red blood cell comprised of glycolysis, the pentose phosphate pathway, AMP Salvage network, and hemoglobin.

First, we set up the core metabolic network as done in Chapter 12.

```
[24]:
```

```
glycolysis = create_test_model("SB2_Glycolysis")
ppp = create_test_model("SB2_PentosePhosphatePathway")
ampsn = create_test_model("SB2_AMPSalvageNetwork")

core_hb = glycolysis.merge(ppp, inplace=False)
core_hb.merge(ampsn, inplace=True)
core_hb.remove_reactions([
    r for r in core_hb.boundary
    if r.id in [
        "SK_g6p_c", "DM_f6p_c", "DM_g3p_c", "DM_r5p_c",
        "DM_amp_c", "SK_amp_c"]])
core_hb.remove_boundary_conditions([
    "g6p_b", "f6p_b", "g3p_b", "r5p_b", "amp_b"])

# Note that reactants have negative coefficients and products have positive coefficients
core_hb.reactions.PRPPS.subtract_metabolites({
    core_hb.metabolites.atp_c: -1,
    core_hb.metabolites.adp_c: 2})
core_hb.reactions.PRPPS.add_metabolites({
    core_hb.metabolites.amp_c: 1})
```

```
Ignoring reaction 'SK_h_c' since it already exists.
Ignoring reaction 'SK_h2o_c' since it already exists.
Ignoring reaction 'ATPM' since it already exists.
Ignoring reaction 'SK_amp_c' since it already exists.
Ignoring reaction 'SK_h_c' since it already exists.
Ignoring reaction 'SK_h2o_c' since it already exists.
```

We then add the hemoglobin subnetwork to this:

```
[25]:
```

```
hemoglobin = create_test_model("SB2_Hemoglobin")
core_hb.merge(hemoglobin, inplace=True)
core_hb.id = "RBC"
```

###### Organization of the stoichiometric matrix¶

As in the previous chapter, we can perform a set of transformations to group the species and reactions into organized groups.

```
[26]:
```

```
# Define new order for metabolites
new_metabolite_order = [
    "glc__D_c", "g6p_c", "f6p_c", "fdp_c", "dhap_c","g3p_c",
    "_13dpg_c", "_3pg_c", "_2pg_c", "pep_c", "pyr_c", "lac__L_c",
    "_6pgl_c", "_6pgc_c", "ru5p__D_c",  "xu5p__D_c", "r5p_c",
    "s7p_c", "e4p_c", "ade_c", "adn_c", "imp_c", "ins_c", "hxan_c",
    "r1p_c", "prpp_c", "_23dpg_c","hb_c", "hb_1o2_c", "hb_2o2_c",
    "hb_3o2_c", "hb_4o2_c", "dhb_c", "nad_c", "nadh_c", "amp_c",
    "adp_c", "atp_c", "nadp_c", "nadph_c", "gthrd_c", "gthox_c",
    "pi_c", "h_c", "h2o_c", "co2_c", "nh3_c", "o2_c"]
if len(core_hb.metabolites) == len(new_metabolite_order):
    core_hb.metabolites = DictList(core_hb.metabolites.get_by_any(new_metabolite_order))
# Define new order for reactions
new_reaction_order = [
    "HEX1", "PGI", "PFK", "FBA", "TPI", "GAPD", "PGK", "PGM",
    "ENO", "PYK", "LDH_L", "G6PDH2r", "PGL", "GND", "RPE",
    "RPI", "TKT1", "TKT2", "TALA", "ADNK1", "NTD7", "ADA","AMPDA",
    "NTD11", "PUNP5", "PPM", "PRPPS", "ADPT", "ADK1", "DPGM",
    "DPGase", "HBDPG", "HBO1", "HBO2", "HBO3", "HBO4", "ATPM",
    "DM_nadh","GTHOr", "GSHR", "SK_glc__D_c", "SK_pyr_c", "SK_lac__L_c",
    "SK_ade_c", "SK_adn_c", "SK_ins_c", "SK_hxan_c","SK_pi_c",
    "SK_h_c", "SK_h2o_c", "SK_co2_c", "SK_nh3_c", "SK_o2_c"]
if len(core_hb.reactions) == len(new_reaction_order):
    core_hb.reactions = DictList(core_hb.reactions.get_by_any(new_reaction_order))
```

The combined stoichiometric matrix is a straightforward integration of the stoichiometric matrices for the two subsystems being combined, see Table 13.8. Note that the matrix has a 2x2 block diagonal structure, as emphasized by the dashed lines. The lower off-diagonal block has no non-zero entries as none of the compounds in the hemoglobin subnetwork participate in any of the glycolytic reactions. The upper-right off-diagonal block has the enzymes that make and degrade 23DPG and represent the
coupling between the glycolytic and hemoglobin subnetworks. The stoichiometric matrix of the core network with the hemoglobin module has dimensions of 48x53 and its rank is 44. The null is of dimension 9 (=53-44) and the left null space is of dimension 4 (=48-44). The matrix is elementally balanced.

We have used colors in Table 13.8 to illustrate the organized structure of the matrix.

**Table 13.8:** The stoichiometric matrix for the merged core network model in Figure 13.6. The matrix is partitioned to show the glycolytic reactions (yellow) separate from the pentose phosphate pathway (light blue), the AMP salvage network (light green) and the Rapoport-Luebering (R.L.) shunt with the binding states of hemoglobin(light red). The cofactors (light orange) and inorganics (pink) are also grouped and shown. The connectivities, \(\rho\_i\) (red), for a compound, and the
participation number, \(\pi\_j\) (cyan), for a reaction are shown. The second block in the table is the product \(\textbf{ES}\) (blue) to evaluate elemental balancing status of the reactions. All exchange reactions have a participation number of unity and are thus not elementally balanced. The last block in the table has the 9 pathway vectors (purple) for the merged model.

```
[27]:
```

```
# Define labels
metabolite_ids = [m.id for m in core_hb.metabolites]
reaction_ids = [r.id for r in core_hb.reactions]

pi_str = r"$\pi_{j}$"
rho_str = r"$\rho_{i}$"
chopsnq = ['C', 'H', 'O', 'P', 'N', 'S', 'q', '[HB]', '[NAD]', '[NADP]',]
time_inv_labels = ["$N_{\mathrm{tot}}$", "$NP_{\mathrm{tot}}$", "$G_{\mathrm{tot}}$", "$Hb_{\mathrm{tot}}$"]
path_labels = ["$p_1$", "$p_2$", "$p_3$", "$p_4$",
               "$p_5$", "$p_6$", "$p_7$", "$p_8$", "$p_9$"]

# Make table content from the stoichiometric matrix, elemental balancing of pathways
# participation number, and MinSpan pathways
S_matrix = core_hb.update_S(array_type="dense", dtype=np.int64, update_model=False)
ES_matrix = core_hb.get_elemental_charge_balancing(dtype=np.int64)
pi = np.count_nonzero(S_matrix, axis=0)
rho = np.count_nonzero(S_matrix, axis=1)
minspan_paths = np.array([
    [1, 1, 1, 1, 1, 2, 2, 2, 2, 2, 2, 0, 0, 0, 0, 0, 0, 0, 0, 0, 0, 0, 0, 0, 0, 0, 0, 0, 0, 0, 0, 0, 0, 0, 0, 0, 2, 0, 0, 0, 1, 0, 2, 0, 0, 0, 0, 0, 2, 0, 0, 0, 0],
    [0, 0, 0, 0, 0, 0, 0, 0, 0, 0, 1, 0, 0, 0, 0, 0, 0, 0, 0, 0, 0, 0, 0, 0, 0, 0, 0, 0, 0, 0, 0, 0, 0, 0, 0, 0, 0,-1, 0, 0, 0,-1, 1, 0, 0, 0, 0, 0,-2, 0, 0, 0, 0],
    [1,-2, 0, 0, 0, 1, 1, 1, 1, 1, 1, 3, 3, 3, 2, 1, 1, 1, 1, 0, 0, 0, 0, 0, 0, 0, 0, 0, 0, 0, 0, 0, 0, 0, 0, 0, 1, 0, 6, 6, 1, 0, 1, 0, 0, 0, 0, 0,13,-3, 3, 0, 0],
    [1, 0, 0, 0, 0, 0, 0, 0, 0, 0, 0, 1, 1, 1, 0, 1, 0, 0, 0, 0, 1, 0, 0, 0, 0, 0, 1, 1,-1, 0, 0, 0, 0, 0, 0, 0,-3, 0, 2, 2, 1, 0, 0,-1, 1, 0, 0, 0, 4, 0, 1, 0, 0],
    [1, 0, 0, 0, 0, 0, 0, 0, 0, 0, 0, 1, 1, 1, 0, 1, 0, 0, 0, 0, 1, 1, 0, 0, 0, 0, 1, 1,-1, 0, 0, 0, 0, 0, 0, 0,-3, 0, 2, 2, 1, 0, 0,-1, 0, 1, 0, 0, 4,-1, 1, 1, 0],
    [1, 0, 0, 0, 0, 0, 0, 0, 0, 0, 0, 1, 1, 1, 0, 1, 0, 0, 0, 0, 0, 0, 1, 1, 0, 0, 1, 1,-1, 0, 0, 0, 0, 0, 0, 0,-3, 0, 2, 2, 1, 0, 0,-1, 0, 1, 0, 0, 4,-1, 1, 1, 0],
    [0, 0, 0, 0, 0, 0, 0, 0, 0, 0, 0, 0, 0, 0, 0, 0, 0, 0, 0, 0, 1, 1, 0, 0, 1, 1, 1, 1,-1, 0, 0, 0, 0, 0, 0, 0,-2, 0, 0, 0, 0, 0, 0,-1, 0, 0, 1, 0, 0,-1, 0, 1, 0],
    [0, 0, 0, 0, 0, 0, 0, 0, 0, 0, 0, 0, 0, 0, 0, 0, 0, 0, 0, 1, 1, 0, 0, 0, 0, 0, 0, 0, 0, 0, 0, 0, 0, 0, 0, 0,-1, 0, 0, 0, 0, 0, 0, 0, 0, 0, 0, 0, 0, 0, 0, 0, 0],
    [1, 1, 1, 1, 1, 2, 0, 2, 2, 2, 2, 0, 0, 0, 0, 0, 0, 0, 0, 0, 0, 0, 0, 0, 0, 0, 0, 0, 0, 2, 2, 0, 0, 0, 0, 0, 2, 0, 0, 0, 1, 0, 2, 0, 0, 0, 0, 0, 2, 0, 0, 0, 0]
])
table_13_8 = np.vstack((S_matrix, pi, ES_matrix, minspan_paths))

# Determine number of blank entries needed to be added to pad the table,
# Add connectivity number and time invariants to table content
blanks = [""]*(len(table_13_8) - len(metabolite_ids))
rho = np.concatenate((rho, blanks))

lns = np.zeros((4, 48), dtype=np.int64)
lns[0][33:35] = 1
lns[1][38:40] = 1
lns[2][40] = 1
lns[2][41] = 2
lns[3][27:33] = 1

time_inv = np.array([np.concatenate([row, blanks]) for row in lns])
table_13_8 = np.vstack([table_13_8.T, rho, time_inv]).T

colors = {"glycolysis": "#ffffe6",    # Yellow
          "ppp": "#e6faff",           # Light blue
          "ampsn": "#d9fad2",         # Light green
          "hemoglobin": "#ffcccc",    # Light red
          "cofactor": "#ffe6cc",      # Orange
          "inorganic": "#fadffa",     # Pink
          "chopsnq": "#99e6ff",       # Blue
          "pathways": "#b399ff",      # Purple
          "pi": "#99ffff",            # Cyan
          "rho": "#ff9999",           # Red
          "time_invs": "#ccff99",     # Green
          "blank": "#f2f2f2"}         # Grey
bg_color_str = "background-color: "
cofactor_mets = ["nad_c", "nadh_c",  "amp_c", "adp_c", "atp_c",
                 "nadp_c", "nadph_c", "gthrd_c", "gthox_c"]
exch_misc_rxns= ["SK_glc__D_c", "SK_pyr_c", "SK_lac__L_c", "SK_ade_c", "SK_adn_c",
                 "SK_ins_c", "SK_hxan_c", "ATPM", "DM_nadh", "GTHOr", "GSHR"]
inorganic_mets = ["pi_c", "h_c", "h2o_c", "co2_c", "nh3_c", "o2_c"]
inorganic_exch = ["SK_pi_c", "SK_h_c", "SK_h2o_c", "SK_co2_c", "SK_nh3_c", "SK_o2_c"]

def highlight_table(df, model):
    df = df.copy()
    condition = lambda mmodel, row, col, c1, c2:  (
        (col not in exch_misc_rxns + inorganic_exch) and (row not in cofactor_mets + inorganic_mets) and (
            (row in mmodel.metabolites and c1) or (col in mmodel.reactions or c2)))
    inorganic_condition = lambda row, col: (col in inorganic_exch or row in inorganic_mets)
    for i, row in enumerate(df.index):
        for j, col in enumerate(df.columns):
            if df.loc[row, col] == "":
                main_key = "blank"
            elif row in pi_str:
                main_key = "pi"
            elif row in chopsnq:
                main_key = "chopsnq"
            elif row in path_labels:
                main_key = "pathways"
            elif col in rho_str:
                main_key = "rho"
            elif col in time_inv_labels:
                main_key = "time_invs"
            elif condition(hemoglobin, row, col, row not in ["_13dpg_c", "_3pg_c"], False):
                main_key = "hemoglobin"
            elif condition(ampsn, row, col, row not in ["r5p_c"], col in ["ADK1"]):
                main_key = "ampsn"
            elif condition(ppp, row, col, row not in ["g6p_c", "f6p_c", "g3p_c"], False):
                main_key = "ppp"
            elif condition(glycolysis, row, col, True, False):
                main_key = "glycolysis"
            elif ((col in exch_misc_rxns or row in cofactor_mets) and not inorganic_condition(row, col)):
                main_key = "cofactor"
            elif inorganic_condition(row, col):
                main_key = "inorganic"
            else:
                continue
            df.loc[row, col] = bg_color_str + colors[main_key]
    return df

# Create index and column labels
index_labels = np.concatenate((metabolite_ids, [pi_str], chopsnq, path_labels))
column_labels = np.concatenate((reaction_ids, [rho_str], time_inv_labels))
# Create DataFrame
table_13_8 = pd.DataFrame(
    table_13_8, index=index_labels, columns=column_labels)
# Apply colors
table_13_8 = table_13_8.style.apply(
    highlight_table,  model=core_hb, axis=None)
table_13_8
```

```
[27]:
```

|  | HEX1 | PGI | PFK | FBA | TPI | GAPD | PGK | PGM | ENO | PYK | LDH\_L | G6PDH2r | PGL | GND | RPE | RPI | TKT1 | TKT2 | TALA | ADNK1 | NTD7 | ADA | AMPDA | NTD11 | PUNP5 | PPM | PRPPS | ADPT | ADK1 | DPGM | DPGase | HBDPG | HBO1 | HBO2 | HBO3 | HBO4 | ATPM | DM\_nadh | GTHOr | GSHR | SK\_glc\_\_D\_c | SK\_pyr\_c | SK\_lac\_\_L\_c | SK\_ade\_c | SK\_adn\_c | SK\_ins\_c | SK\_hxan\_c | SK\_pi\_c | SK\_h\_c | SK\_h2o\_c | SK\_co2\_c | SK\_nh3\_c | SK\_o2\_c | $\rho\_{i}$ | $N\_{\mathrm{tot}}$ | $NP\_{\mathrm{tot}}$ | $G\_{\mathrm{tot}}$ | $Hb\_{\mathrm{tot}}$ |
| --- | --- | --- | --- | --- | --- | --- | --- | --- | --- | --- | --- | --- | --- | --- | --- | --- | --- | --- | --- | --- | --- | --- | --- | --- | --- | --- | --- | --- | --- | --- | --- | --- | --- | --- | --- | --- | --- | --- | --- | --- | --- | --- | --- | --- | --- | --- | --- | --- | --- | --- | --- | --- | --- | --- | --- | --- | --- | --- |
| glc\_\_D\_c | -1 | 0 | 0 | 0 | 0 | 0 | 0 | 0 | 0 | 0 | 0 | 0 | 0 | 0 | 0 | 0 | 0 | 0 | 0 | 0 | 0 | 0 | 0 | 0 | 0 | 0 | 0 | 0 | 0 | 0 | 0 | 0 | 0 | 0 | 0 | 0 | 0 | 0 | 0 | 0 | 1 | 0 | 0 | 0 | 0 | 0 | 0 | 0 | 0 | 0 | 0 | 0 | 0 | 2 | 0 | 0 | 0 | 0 |
| g6p\_c | 1 | -1 | 0 | 0 | 0 | 0 | 0 | 0 | 0 | 0 | 0 | -1 | 0 | 0 | 0 | 0 | 0 | 0 | 0 | 0 | 0 | 0 | 0 | 0 | 0 | 0 | 0 | 0 | 0 | 0 | 0 | 0 | 0 | 0 | 0 | 0 | 0 | 0 | 0 | 0 | 0 | 0 | 0 | 0 | 0 | 0 | 0 | 0 | 0 | 0 | 0 | 0 | 0 | 3 | 0 | 0 | 0 | 0 |
| f6p\_c | 0 | 1 | -1 | 0 | 0 | 0 | 0 | 0 | 0 | 0 | 0 | 0 | 0 | 0 | 0 | 0 | 0 | 1 | 1 | 0 | 0 | 0 | 0 | 0 | 0 | 0 | 0 | 0 | 0 | 0 | 0 | 0 | 0 | 0 | 0 | 0 | 0 | 0 | 0 | 0 | 0 | 0 | 0 | 0 | 0 | 0 | 0 | 0 | 0 | 0 | 0 | 0 | 0 | 4 | 0 | 0 | 0 | 0 |
| fdp\_c | 0 | 0 | 1 | -1 | 0 | 0 | 0 | 0 | 0 | 0 | 0 | 0 | 0 | 0 | 0 | 0 | 0 | 0 | 0 | 0 | 0 | 0 | 0 | 0 | 0 | 0 | 0 | 0 | 0 | 0 | 0 | 0 | 0 | 0 | 0 | 0 | 0 | 0 | 0 | 0 | 0 | 0 | 0 | 0 | 0 | 0 | 0 | 0 | 0 | 0 | 0 | 0 | 0 | 2 | 0 | 0 | 0 | 0 |
| dhap\_c | 0 | 0 | 0 | 1 | -1 | 0 | 0 | 0 | 0 | 0 | 0 | 0 | 0 | 0 | 0 | 0 | 0 | 0 | 0 | 0 | 0 | 0 | 0 | 0 | 0 | 0 | 0 | 0 | 0 | 0 | 0 | 0 | 0 | 0 | 0 | 0 | 0 | 0 | 0 | 0 | 0 | 0 | 0 | 0 | 0 | 0 | 0 | 0 | 0 | 0 | 0 | 0 | 0 | 2 | 0 | 0 | 0 | 0 |
| g3p\_c | 0 | 0 | 0 | 1 | 1 | -1 | 0 | 0 | 0 | 0 | 0 | 0 | 0 | 0 | 0 | 0 | 1 | 1 | -1 | 0 | 0 | 0 | 0 | 0 | 0 | 0 | 0 | 0 | 0 | 0 | 0 | 0 | 0 | 0 | 0 | 0 | 0 | 0 | 0 | 0 | 0 | 0 | 0 | 0 | 0 | 0 | 0 | 0 | 0 | 0 | 0 | 0 | 0 | 6 | 0 | 0 | 0 | 0 |
| \_13dpg\_c | 0 | 0 | 0 | 0 | 0 | 1 | -1 | 0 | 0 | 0 | 0 | 0 | 0 | 0 | 0 | 0 | 0 | 0 | 0 | 0 | 0 | 0 | 0 | 0 | 0 | 0 | 0 | 0 | 0 | -1 | 0 | 0 | 0 | 0 | 0 | 0 | 0 | 0 | 0 | 0 | 0 | 0 | 0 | 0 | 0 | 0 | 0 | 0 | 0 | 0 | 0 | 0 | 0 | 3 | 0 | 0 | 0 | 0 |
| \_3pg\_c | 0 | 0 | 0 | 0 | 0 | 0 | 1 | -1 | 0 | 0 | 0 | 0 | 0 | 0 | 0 | 0 | 0 | 0 | 0 | 0 | 0 | 0 | 0 | 0 | 0 | 0 | 0 | 0 | 0 | 0 | 1 | 0 | 0 | 0 | 0 | 0 | 0 | 0 | 0 | 0 | 0 | 0 | 0 | 0 | 0 | 0 | 0 | 0 | 0 | 0 | 0 | 0 | 0 | 3 | 0 | 0 | 0 | 0 |
| \_2pg\_c | 0 | 0 | 0 | 0 | 0 | 0 | 0 | 1 | -1 | 0 | 0 | 0 | 0 | 0 | 0 | 0 | 0 | 0 | 0 | 0 | 0 | 0 | 0 | 0 | 0 | 0 | 0 | 0 | 0 | 0 | 0 | 0 | 0 | 0 | 0 | 0 | 0 | 0 | 0 | 0 | 0 | 0 | 0 | 0 | 0 | 0 | 0 | 0 | 0 | 0 | 0 | 0 | 0 | 2 | 0 | 0 | 0 | 0 |
| pep\_c | 0 | 0 | 0 | 0 | 0 | 0 | 0 | 0 | 1 | -1 | 0 | 0 | 0 | 0 | 0 | 0 | 0 | 0 | 0 | 0 | 0 | 0 | 0 | 0 | 0 | 0 | 0 | 0 | 0 | 0 | 0 | 0 | 0 | 0 | 0 | 0 | 0 | 0 | 0 | 0 | 0 | 0 | 0 | 0 | 0 | 0 | 0 | 0 | 0 | 0 | 0 | 0 | 0 | 2 | 0 | 0 | 0 | 0 |
| pyr\_c | 0 | 0 | 0 | 0 | 0 | 0 | 0 | 0 | 0 | 1 | -1 | 0 | 0 | 0 | 0 | 0 | 0 | 0 | 0 | 0 | 0 | 0 | 0 | 0 | 0 | 0 | 0 | 0 | 0 | 0 | 0 | 0 | 0 | 0 | 0 | 0 | 0 | 0 | 0 | 0 | 0 | -1 | 0 | 0 | 0 | 0 | 0 | 0 | 0 | 0 | 0 | 0 | 0 | 3 | 0 | 0 | 0 | 0 |
| lac\_\_L\_c | 0 | 0 | 0 | 0 | 0 | 0 | 0 | 0 | 0 | 0 | 1 | 0 | 0 | 0 | 0 | 0 | 0 | 0 | 0 | 0 | 0 | 0 | 0 | 0 | 0 | 0 | 0 | 0 | 0 | 0 | 0 | 0 | 0 | 0 | 0 | 0 | 0 | 0 | 0 | 0 | 0 | 0 | -1 | 0 | 0 | 0 | 0 | 0 | 0 | 0 | 0 | 0 | 0 | 2 | 0 | 0 | 0 | 0 |
| \_6pgl\_c | 0 | 0 | 0 | 0 | 0 | 0 | 0 | 0 | 0 | 0 | 0 | 1 | -1 | 0 | 0 | 0 | 0 | 0 | 0 | 0 | 0 | 0 | 0 | 0 | 0 | 0 | 0 | 0 | 0 | 0 | 0 | 0 | 0 | 0 | 0 | 0 | 0 | 0 | 0 | 0 | 0 | 0 | 0 | 0 | 0 | 0 | 0 | 0 | 0 | 0 | 0 | 0 | 0 | 2 | 0 | 0 | 0 | 0 |
| \_6pgc\_c | 0 | 0 | 0 | 0 | 0 | 0 | 0 | 0 | 0 | 0 | 0 | 0 | 1 | -1 | 0 | 0 | 0 | 0 | 0 | 0 | 0 | 0 | 0 | 0 | 0 | 0 | 0 | 0 | 0 | 0 | 0 | 0 | 0 | 0 | 0 | 0 | 0 | 0 | 0 | 0 | 0 | 0 | 0 | 0 | 0 | 0 | 0 | 0 | 0 | 0 | 0 | 0 | 0 | 2 | 0 | 0 | 0 | 0 |
| ru5p\_\_D\_c | 0 | 0 | 0 | 0 | 0 | 0 | 0 | 0 | 0 | 0 | 0 | 0 | 0 | 1 | -1 | -1 | 0 | 0 | 0 | 0 | 0 | 0 | 0 | 0 | 0 | 0 | 0 | 0 | 0 | 0 | 0 | 0 | 0 | 0 | 0 | 0 | 0 | 0 | 0 | 0 | 0 | 0 | 0 | 0 | 0 | 0 | 0 | 0 | 0 | 0 | 0 | 0 | 0 | 3 | 0 | 0 | 0 | 0 |
| xu5p\_\_D\_c | 0 | 0 | 0 | 0 | 0 | 0 | 0 | 0 | 0 | 0 | 0 | 0 | 0 | 0 | 1 | 0 | -1 | -1 | 0 | 0 | 0 | 0 | 0 | 0 | 0 | 0 | 0 | 0 | 0 | 0 | 0 | 0 | 0 | 0 | 0 | 0 | 0 | 0 | 0 | 0 | 0 | 0 | 0 | 0 | 0 | 0 | 0 | 0 | 0 | 0 | 0 | 0 | 0 | 3 | 0 | 0 | 0 | 0 |
| r5p\_c | 0 | 0 | 0 | 0 | 0 | 0 | 0 | 0 | 0 | 0 | 0 | 0 | 0 | 0 | 0 | 1 | -1 | 0 | 0 | 0 | 0 | 0 | 0 | 0 | 0 | 1 | -1 | 0 | 0 | 0 | 0 | 0 | 0 | 0 | 0 | 0 | 0 | 0 | 0 | 0 | 0 | 0 | 0 | 0 | 0 | 0 | 0 | 0 | 0 | 0 | 0 | 0 | 0 | 4 | 0 | 0 | 0 | 0 |
| s7p\_c | 0 | 0 | 0 | 0 | 0 | 0 | 0 | 0 | 0 | 0 | 0 | 0 | 0 | 0 | 0 | 0 | 1 | 0 | -1 | 0 | 0 | 0 | 0 | 0 | 0 | 0 | 0 | 0 | 0 | 0 | 0 | 0 | 0 | 0 | 0 | 0 | 0 | 0 | 0 | 0 | 0 | 0 | 0 | 0 | 0 | 0 | 0 | 0 | 0 | 0 | 0 | 0 | 0 | 2 | 0 | 0 | 0 | 0 |
| e4p\_c | 0 | 0 | 0 | 0 | 0 | 0 | 0 | 0 | 0 | 0 | 0 | 0 | 0 | 0 | 0 | 0 | 0 | -1 | 1 | 0 | 0 | 0 | 0 | 0 | 0 | 0 | 0 | 0 | 0 | 0 | 0 | 0 | 0 | 0 | 0 | 0 | 0 | 0 | 0 | 0 | 0 | 0 | 0 | 0 | 0 | 0 | 0 | 0 | 0 | 0 | 0 | 0 | 0 | 2 | 0 | 0 | 0 | 0 |
| ade\_c | 0 | 0 | 0 | 0 | 0 | 0 | 0 | 0 | 0 | 0 | 0 | 0 | 0 | 0 | 0 | 0 | 0 | 0 | 0 | 0 | 0 | 0 | 0 | 0 | 0 | 0 | 0 | -1 | 0 | 0 | 0 | 0 | 0 | 0 | 0 | 0 | 0 | 0 | 0 | 0 | 0 | 0 | 0 | -1 | 0 | 0 | 0 | 0 | 0 | 0 | 0 | 0 | 0 | 2 | 0 | 0 | 0 | 0 |
| adn\_c | 0 | 0 | 0 | 0 | 0 | 0 | 0 | 0 | 0 | 0 | 0 | 0 | 0 | 0 | 0 | 0 | 0 | 0 | 0 | -1 | 1 | -1 | 0 | 0 | 0 | 0 | 0 | 0 | 0 | 0 | 0 | 0 | 0 | 0 | 0 | 0 | 0 | 0 | 0 | 0 | 0 | 0 | 0 | 0 | -1 | 0 | 0 | 0 | 0 | 0 | 0 | 0 | 0 | 4 | 0 | 0 | 0 | 0 |
| imp\_c | 0 | 0 | 0 | 0 | 0 | 0 | 0 | 0 | 0 | 0 | 0 | 0 | 0 | 0 | 0 | 0 | 0 | 0 | 0 | 0 | 0 | 0 | 1 | -1 | 0 | 0 | 0 | 0 | 0 | 0 | 0 | 0 | 0 | 0 | 0 | 0 | 0 | 0 | 0 | 0 | 0 | 0 | 0 | 0 | 0 | 0 | 0 | 0 | 0 | 0 | 0 | 0 | 0 | 2 | 0 | 0 | 0 | 0 |
| ins\_c | 0 | 0 | 0 | 0 | 0 | 0 | 0 | 0 | 0 | 0 | 0 | 0 | 0 | 0 | 0 | 0 | 0 | 0 | 0 | 0 | 0 | 1 | 0 | 1 | -1 | 0 | 0 | 0 | 0 | 0 | 0 | 0 | 0 | 0 | 0 | 0 | 0 | 0 | 0 | 0 | 0 | 0 | 0 | 0 | 0 | -1 | 0 | 0 | 0 | 0 | 0 | 0 | 0 | 4 | 0 | 0 | 0 | 0 |
| hxan\_c | 0 | 0 | 0 | 0 | 0 | 0 | 0 | 0 | 0 | 0 | 0 | 0 | 0 | 0 | 0 | 0 | 0 | 0 | 0 | 0 | 0 | 0 | 0 | 0 | 1 | 0 | 0 | 0 | 0 | 0 | 0 | 0 | 0 | 0 | 0 | 0 | 0 | 0 | 0 | 0 | 0 | 0 | 0 | 0 | 0 | 0 | -1 | 0 | 0 | 0 | 0 | 0 | 0 | 2 | 0 | 0 | 0 | 0 |
| r1p\_c | 0 | 0 | 0 | 0 | 0 | 0 | 0 | 0 | 0 | 0 | 0 | 0 | 0 | 0 | 0 | 0 | 0 | 0 | 0 | 0 | 0 | 0 | 0 | 0 | 1 | -1 | 0 | 0 | 0 | 0 | 0 | 0 | 0 | 0 | 0 | 0 | 0 | 0 | 0 | 0 | 0 | 0 | 0 | 0 | 0 | 0 | 0 | 0 | 0 | 0 | 0 | 0 | 0 | 2 | 0 | 0 | 0 | 0 |
| prpp\_c | 0 | 0 | 0 | 0 | 0 | 0 | 0 | 0 | 0 | 0 | 0 | 0 | 0 | 0 | 0 | 0 | 0 | 0 | 0 | 0 | 0 | 0 | 0 | 0 | 0 | 0 | 1 | -1 | 0 | 0 | 0 | 0 | 0 | 0 | 0 | 0 | 0 | 0 | 0 | 0 | 0 | 0 | 0 | 0 | 0 | 0 | 0 | 0 | 0 | 0 | 0 | 0 | 0 | 2 | 0 | 0 | 0 | 0 |
| \_23dpg\_c | 0 | 0 | 0 | 0 | 0 | 0 | 0 | 0 | 0 | 0 | 0 | 0 | 0 | 0 | 0 | 0 | 0 | 0 | 0 | 0 | 0 | 0 | 0 | 0 | 0 | 0 | 0 | 0 | 0 | 1 | -1 | -1 | 0 | 0 | 0 | 0 | 0 | 0 | 0 | 0 | 0 | 0 | 0 | 0 | 0 | 0 | 0 | 0 | 0 | 0 | 0 | 0 | 0 | 3 | 0 | 0 | 0 | 0 |
| hb\_c | 0 | 0 | 0 | 0 | 0 | 0 | 0 | 0 | 0 | 0 | 0 | 0 | 0 | 0 | 0 | 0 | 0 | 0 | 0 | 0 | 0 | 0 | 0 | 0 | 0 | 0 | 0 | 0 | 0 | 0 | 0 | -1 | -1 | 0 | 0 | 0 | 0 | 0 | 0 | 0 | 0 | 0 | 0 | 0 | 0 | 0 | 0 | 0 | 0 | 0 | 0 | 0 | 0 | 2 | 0 | 0 | 0 | 1 |
| hb\_1o2\_c | 0 | 0 | 0 | 0 | 0 | 0 | 0 | 0 | 0 | 0 | 0 | 0 | 0 | 0 | 0 | 0 | 0 | 0 | 0 | 0 | 0 | 0 | 0 | 0 | 0 | 0 | 0 | 0 | 0 | 0 | 0 | 0 | 1 | -1 | 0 | 0 | 0 | 0 | 0 | 0 | 0 | 0 | 0 | 0 | 0 | 0 | 0 | 0 | 0 | 0 | 0 | 0 | 0 | 2 | 0 | 0 | 0 | 1 |
| hb\_2o2\_c | 0 | 0 | 0 | 0 | 0 | 0 | 0 | 0 | 0 | 0 | 0 | 0 | 0 | 0 | 0 | 0 | 0 | 0 | 0 | 0 | 0 | 0 | 0 | 0 | 0 | 0 | 0 | 0 | 0 | 0 | 0 | 0 | 0 | 1 | -1 | 0 | 0 | 0 | 0 | 0 | 0 | 0 | 0 | 0 | 0 | 0 | 0 | 0 | 0 | 0 | 0 | 0 | 0 | 2 | 0 | 0 | 0 | 1 |
| hb\_3o2\_c | 0 | 0 | 0 | 0 | 0 | 0 | 0 | 0 | 0 | 0 | 0 | 0 | 0 | 0 | 0 | 0 | 0 | 0 | 0 | 0 | 0 | 0 | 0 | 0 | 0 | 0 | 0 | 0 | 0 | 0 | 0 | 0 | 0 | 0 | 1 | -1 | 0 | 0 | 0 | 0 | 0 | 0 | 0 | 0 | 0 | 0 | 0 | 0 | 0 | 0 | 0 | 0 | 0 | 2 | 0 | 0 | 0 | 1 |
| hb\_4o2\_c | 0 | 0 | 0 | 0 | 0 | 0 | 0 | 0 | 0 | 0 | 0 | 0 | 0 | 0 | 0 | 0 | 0 | 0 | 0 | 0 | 0 | 0 | 0 | 0 | 0 | 0 | 0 | 0 | 0 | 0 | 0 | 0 | 0 | 0 | 0 | 1 | 0 | 0 | 0 | 0 | 0 | 0 | 0 | 0 | 0 | 0 | 0 | 0 | 0 | 0 | 0 | 0 | 0 | 1 | 0 | 0 | 0 | 1 |
| dhb\_c | 0 | 0 | 0 | 0 | 0 | 0 | 0 | 0 | 0 | 0 | 0 | 0 | 0 | 0 | 0 | 0 | 0 | 0 | 0 | 0 | 0 | 0 | 0 | 0 | 0 | 0 | 0 | 0 | 0 | 0 | 0 | 1 | 0 | 0 | 0 | 0 | 0 | 0 | 0 | 0 | 0 | 0 | 0 | 0 | 0 | 0 | 0 | 0 | 0 | 0 | 0 | 0 | 0 | 1 | 0 | 0 | 0 | 1 |
| nad\_c | 0 | 0 | 0 | 0 | 0 | -1 | 0 | 0 | 0 | 0 | 1 | 0 | 0 | 0 | 0 | 0 | 0 | 0 | 0 | 0 | 0 | 0 | 0 | 0 | 0 | 0 | 0 | 0 | 0 | 0 | 0 | 0 | 0 | 0 | 0 | 0 | 0 | 1 | 0 | 0 | 0 | 0 | 0 | 0 | 0 | 0 | 0 | 0 | 0 | 0 | 0 | 0 | 0 | 3 | 1 | 0 | 0 | 0 |
| nadh\_c | 0 | 0 | 0 | 0 | 0 | 1 | 0 | 0 | 0 | 0 | -1 | 0 | 0 | 0 | 0 | 0 | 0 | 0 | 0 | 0 | 0 | 0 | 0 | 0 | 0 | 0 | 0 | 0 | 0 | 0 | 0 | 0 | 0 | 0 | 0 | 0 | 0 | -1 | 0 | 0 | 0 | 0 | 0 | 0 | 0 | 0 | 0 | 0 | 0 | 0 | 0 | 0 | 0 | 3 | 1 | 0 | 0 | 0 |
| amp\_c | 0 | 0 | 0 | 0 | 0 | 0 | 0 | 0 | 0 | 0 | 0 | 0 | 0 | 0 | 0 | 0 | 0 | 0 | 0 | 1 | -1 | 0 | -1 | 0 | 0 | 0 | 1 | 1 | 1 | 0 | 0 | 0 | 0 | 0 | 0 | 0 | 0 | 0 | 0 | 0 | 0 | 0 | 0 | 0 | 0 | 0 | 0 | 0 | 0 | 0 | 0 | 0 | 0 | 6 | 0 | 0 | 0 | 0 |
| adp\_c | 1 | 0 | 1 | 0 | 0 | 0 | -1 | 0 | 0 | -1 | 0 | 0 | 0 | 0 | 0 | 0 | 0 | 0 | 0 | 1 | 0 | 0 | 0 | 0 | 0 | 0 | 0 | 0 | -2 | 0 | 0 | 0 | 0 | 0 | 0 | 0 | 1 | 0 | 0 | 0 | 0 | 0 | 0 | 0 | 0 | 0 | 0 | 0 | 0 | 0 | 0 | 0 | 0 | 7 | 0 | 0 | 0 | 0 |
| atp\_c | -1 | 0 | -1 | 0 | 0 | 0 | 1 | 0 | 0 | 1 | 0 | 0 | 0 | 0 | 0 | 0 | 0 | 0 | 0 | -1 | 0 | 0 | 0 | 0 | 0 | 0 | -1 | 0 | 1 | 0 | 0 | 0 | 0 | 0 | 0 | 0 | -1 | 0 | 0 | 0 | 0 | 0 | 0 | 0 | 0 | 0 | 0 | 0 | 0 | 0 | 0 | 0 | 0 | 8 | 0 | 0 | 0 | 0 |
| nadp\_c | 0 | 0 | 0 | 0 | 0 | 0 | 0 | 0 | 0 | 0 | 0 | -1 | 0 | -1 | 0 | 0 | 0 | 0 | 0 | 0 | 0 | 0 | 0 | 0 | 0 | 0 | 0 | 0 | 0 | 0 | 0 | 0 | 0 | 0 | 0 | 0 | 0 | 0 | 1 | 0 | 0 | 0 | 0 | 0 | 0 | 0 | 0 | 0 | 0 | 0 | 0 | 0 | 0 | 3 | 0 | 1 | 0 | 0 |
| nadph\_c | 0 | 0 | 0 | 0 | 0 | 0 | 0 | 0 | 0 | 0 | 0 | 1 | 0 | 1 | 0 | 0 | 0 | 0 | 0 | 0 | 0 | 0 | 0 | 0 | 0 | 0 | 0 | 0 | 0 | 0 | 0 | 0 | 0 | 0 | 0 | 0 | 0 | 0 | -1 | 0 | 0 | 0 | 0 | 0 | 0 | 0 | 0 | 0 | 0 | 0 | 0 | 0 | 0 | 3 | 0 | 1 | 0 | 0 |
| gthrd\_c | 0 | 0 | 0 | 0 | 0 | 0 | 0 | 0 | 0 | 0 | 0 | 0 | 0 | 0 | 0 | 0 | 0 | 0 | 0 | 0 | 0 | 0 | 0 | 0 | 0 | 0 | 0 | 0 | 0 | 0 | 0 | 0 | 0 | 0 | 0 | 0 | 0 | 0 | 2 | -2 | 0 | 0 | 0 | 0 | 0 | 0 | 0 | 0 | 0 | 0 | 0 | 0 | 0 | 2 | 0 | 0 | 1 | 0 |
| gthox\_c | 0 | 0 | 0 | 0 | 0 | 0 | 0 | 0 | 0 | 0 | 0 | 0 | 0 | 0 | 0 | 0 | 0 | 0 | 0 | 0 | 0 | 0 | 0 | 0 | 0 | 0 | 0 | 0 | 0 | 0 | 0 | 0 | 0 | 0 | 0 | 0 | 0 | 0 | -1 | 1 | 0 | 0 | 0 | 0 | 0 | 0 | 0 | 0 | 0 | 0 | 0 | 0 | 0 | 2 | 0 | 0 | 2 | 0 |
| pi\_c | 0 | 0 | 0 | 0 | 0 | -1 | 0 | 0 | 0 | 0 | 0 | 0 | 0 | 0 | 0 | 0 | 0 | 0 | 0 | 0 | 1 | 0 | 0 | 1 | -1 | 0 | 0 | 2 | 0 | 0 | 1 | 0 | 0 | 0 | 0 | 0 | 1 | 0 | 0 | 0 | 0 | 0 | 0 | 0 | 0 | 0 | 0 | -1 | 0 | 0 | 0 | 0 | 0 | 8 | 0 | 0 | 0 | 0 |
| h\_c | 1 | 0 | 1 | 0 | 0 | 1 | 0 | 0 | 0 | -1 | -1 | 1 | 1 | 0 | 0 | 0 | 0 | 0 | 0 | 1 | 0 | 0 | 0 | 0 | 0 | 0 | 1 | 1 | 0 | 1 | 0 | 0 | 0 | 0 | 0 | 0 | 1 | 1 | -1 | 2 | 0 | 0 | 0 | 0 | 0 | 0 | 0 | 0 | -1 | 0 | 0 | 0 | 0 | 16 | 0 | 0 | 0 | 0 |
| h2o\_c | 0 | 0 | 0 | 0 | 0 | 0 | 0 | 0 | 1 | 0 | 0 | 0 | -1 | 0 | 0 | 0 | 0 | 0 | 0 | 0 | -1 | -1 | -1 | -1 | 0 | 0 | 0 | -1 | 0 | 0 | -1 | 0 | 0 | 0 | 0 | 0 | -1 | 0 | 0 | 0 | 0 | 0 | 0 | 0 | 0 | 0 | 0 | 0 | 0 | -1 | 0 | 0 | 0 | 10 | 0 | 0 | 0 | 0 |
| co2\_c | 0 | 0 | 0 | 0 | 0 | 0 | 0 | 0 | 0 | 0 | 0 | 0 | 0 | 1 | 0 | 0 | 0 | 0 | 0 | 0 | 0 | 0 | 0 | 0 | 0 | 0 | 0 | 0 | 0 | 0 | 0 | 0 | 0 | 0 | 0 | 0 | 0 | 0 | 0 | 0 | 0 | 0 | 0 | 0 | 0 | 0 | 0 | 0 | 0 | 0 | -1 | 0 | 0 | 2 | 0 | 0 | 0 | 0 |
| nh3\_c | 0 | 0 | 0 | 0 | 0 | 0 | 0 | 0 | 0 | 0 | 0 | 0 | 0 | 0 | 0 | 0 | 0 | 0 | 0 | 0 | 0 | 1 | 1 | 0 | 0 | 0 | 0 | 0 | 0 | 0 | 0 | 0 | 0 | 0 | 0 | 0 | 0 | 0 | 0 | 0 | 0 | 0 | 0 | 0 | 0 | 0 | 0 | 0 | 0 | 0 | 0 | -1 | 0 | 3 | 0 | 0 | 0 | 0 |
| o2\_c | 0 | 0 | 0 | 0 | 0 | 0 | 0 | 0 | 0 | 0 | 0 | 0 | 0 | 0 | 0 | 0 | 0 | 0 | 0 | 0 | 0 | 0 | 0 | 0 | 0 | 0 | 0 | 0 | 0 | 0 | 0 | 0 | -1 | -1 | -1 | -1 | 0 | 0 | 0 | 0 | 0 | 0 | 0 | 0 | 0 | 0 | 0 | 0 | 0 | 0 | 0 | 0 | -1 | 5 | 0 | 0 | 0 | 0 |
| $\pi\_{j}$ | 5 | 2 | 5 | 3 | 2 | 6 | 4 | 2 | 3 | 5 | 5 | 5 | 4 | 5 | 2 | 2 | 4 | 4 | 4 | 5 | 4 | 4 | 4 | 4 | 4 | 2 | 5 | 6 | 3 | 3 | 4 | 3 | 3 | 3 | 3 | 3 | 5 | 3 | 5 | 3 | 1 | 1 | 1 | 1 | 1 | 1 | 1 | 1 | 1 | 1 | 1 | 1 | 1 |  |  |  |  |  |
| C | 0 | 0 | 0 | 0 | 0 | 0 | 0 | 0 | 0 | 0 | 0 | 0 | 0 | 0 | 0 | 0 | 0 | 0 | 0 | 0 | 0 | 0 | 0 | 0 | 0 | 0 | 0 | 0 | 0 | 0 | 0 | 0 | 0 | 0 | 0 | 0 | 0 | 0 | 0 | 0 | 6 | -3 | -3 | -5 | -10 | -10 | -5 | 0 | 0 | 0 | -1 | 0 | 0 |  |  |  |  |  |
| H | 0 | 0 | 0 | 0 | 0 | 0 | 0 | 0 | 0 | 0 | 0 | 0 | 0 | 0 | 0 | 0 | 0 | 0 | 0 | 0 | 0 | 0 | 0 | 0 | 0 | 0 | 0 | 0 | 0 | 0 | 0 | 0 | 0 | 0 | 0 | 0 | 0 | 0 | 0 | 0 | 12 | -3 | -5 | -5 | -13 | -12 | -4 | -1 | -1 | -2 | 0 | -3 | 0 |  |  |  |  |  |
| O | 0 | 0 | 0 | 0 | 0 | 0 | 0 | 0 | 0 | 0 | 0 | 0 | 0 | 0 | 0 | 0 | 0 | 0 | 0 | 0 | 0 | 0 | 0 | 0 | 0 | 0 | 0 | 0 | 0 | 0 | 0 | 0 | 0 | 0 | 0 | 0 | 0 | 0 | 0 | 0 | 6 | -3 | -3 | 0 | -4 | -5 | -1 | -4 | 0 | -1 | -2 | 0 | -2 |  |  |  |  |  |
| P | 0 | 0 | 0 | 0 | 0 | 0 | 0 | 0 | 0 | 0 | 0 | 0 | 0 | 0 | 0 | 0 | 0 | 0 | 0 | 0 | 0 | 0 | 0 | 0 | 0 | 0 | 0 | 0 | 0 | 0 | 0 | 0 | 0 | 0 | 0 | 0 | 0 | 0 | 0 | 0 | 0 | 0 | 0 | 0 | 0 | 0 | 0 | -1 | 0 | 0 | 0 | 0 | 0 |  |  |  |  |  |
| N | 0 | 0 | 0 | 0 | 0 | 0 | 0 | 0 | 0 | 0 | 0 | 0 | 0 | 0 | 0 | 0 | 0 | 0 | 0 | 0 | 0 | 0 | 0 | 0 | 0 | 0 | 0 | 0 | 0 | 0 | 0 | 0 | 0 | 0 | 0 | 0 | 0 | 0 | 0 | 0 | 0 | 0 | 0 | -5 | -5 | -4 | -4 | 0 | 0 | 0 | 0 | -1 | 0 |  |  |  |  |  |
| S | 0 | 0 | 0 | 0 | 0 | 0 | 0 | 0 | 0 | 0 | 0 | 0 | 0 | 0 | 0 | 0 | 0 | 0 | 0 | 0 | 0 | 0 | 0 | 0 | 0 | 0 | 0 | 0 | 0 | 0 | 0 | 0 | 0 | 0 | 0 | 0 | 0 | 0 | 0 | 0 | 0 | 0 | 0 | 0 | 0 | 0 | 0 | 0 | 0 | 0 | 0 | 0 | 0 |  |  |  |  |  |
| q | 0 | 0 | 0 | 0 | 0 | 0 | 0 | 0 | 0 | 0 | 0 | 0 | 0 | 0 | 0 | 0 | 0 | 0 | 0 | 0 | 0 | 0 | 0 | 0 | 0 | 0 | 0 | 0 | 0 | 0 | 0 | 0 | 0 | 0 | 0 | 0 | 0 | 2 | 0 | 2 | 0 | 1 | 1 | 0 | 0 | 0 | 0 | 2 | -1 | 0 | 0 | 0 | 0 |  |  |  |  |  |
| [HB] | 0 | 0 | 0 | 0 | 0 | 0 | 0 | 0 | 0 | 0 | 0 | 0 | 0 | 0 | 0 | 0 | 0 | 0 | 0 | 0 | 0 | 0 | 0 | 0 | 0 | 0 | 0 | 0 | 0 | 0 | 0 | 0 | 0 | 0 | 0 | 0 | 0 | 0 | 0 | 0 | 0 | 0 | 0 | 0 | 0 | 0 | 0 | 0 | 0 | 0 | 0 | 0 | 0 |  |  |  |  |  |
| [NAD] | 0 | 0 | 0 | 0 | 0 | 0 | 0 | 0 | 0 | 0 | 0 | 0 | 0 | 0 | 0 | 0 | 0 | 0 | 0 | 0 | 0 | 0 | 0 | 0 | 0 | 0 | 0 | 0 | 0 | 0 | 0 | 0 | 0 | 0 | 0 | 0 | 0 | 0 | 0 | 0 | 0 | 0 | 0 | 0 | 0 | 0 | 0 | 0 | 0 | 0 | 0 | 0 | 0 |  |  |  |  |  |
| [NADP] | 0 | 0 | 0 | 0 | 0 | 0 | 0 | 0 | 0 | 0 | 0 | 0 | 0 | 0 | 0 | 0 | 0 | 0 | 0 | 0 | 0 | 0 | 0 | 0 | 0 | 0 | 0 | 0 | 0 | 0 | 0 | 0 | 0 | 0 | 0 | 0 | 0 | 0 | 0 | 0 | 0 | 0 | 0 | 0 | 0 | 0 | 0 | 0 | 0 | 0 | 0 | 0 | 0 |  |  |  |  |  |
| $p\_1$ | 1 | 1 | 1 | 1 | 1 | 2 | 2 | 2 | 2 | 2 | 2 | 0 | 0 | 0 | 0 | 0 | 0 | 0 | 0 | 0 | 0 | 0 | 0 | 0 | 0 | 0 | 0 | 0 | 0 | 0 | 0 | 0 | 0 | 0 | 0 | 0 | 2 | 0 | 0 | 0 | 1 | 0 | 2 | 0 | 0 | 0 | 0 | 0 | 2 | 0 | 0 | 0 | 0 |  |  |  |  |  |
| $p\_2$ | 0 | 0 | 0 | 0 | 0 | 0 | 0 | 0 | 0 | 0 | 1 | 0 | 0 | 0 | 0 | 0 | 0 | 0 | 0 | 0 | 0 | 0 | 0 | 0 | 0 | 0 | 0 | 0 | 0 | 0 | 0 | 0 | 0 | 0 | 0 | 0 | 0 | -1 | 0 | 0 | 0 | -1 | 1 | 0 | 0 | 0 | 0 | 0 | -2 | 0 | 0 | 0 | 0 |  |  |  |  |  |
| $p\_3$ | 1 | -2 | 0 | 0 | 0 | 1 | 1 | 1 | 1 | 1 | 1 | 3 | 3 | 3 | 2 | 1 | 1 | 1 | 1 | 0 | 0 | 0 | 0 | 0 | 0 | 0 | 0 | 0 | 0 | 0 | 0 | 0 | 0 | 0 | 0 | 0 | 1 | 0 | 6 | 6 | 1 | 0 | 1 | 0 | 0 | 0 | 0 | 0 | 13 | -3 | 3 | 0 | 0 |  |  |  |  |  |
| $p\_4$ | 1 | 0 | 0 | 0 | 0 | 0 | 0 | 0 | 0 | 0 | 0 | 1 | 1 | 1 | 0 | 1 | 0 | 0 | 0 | 0 | 1 | 0 | 0 | 0 | 0 | 0 | 1 | 1 | -1 | 0 | 0 | 0 | 0 | 0 | 0 | 0 | -3 | 0 | 2 | 2 | 1 | 0 | 0 | -1 | 1 | 0 | 0 | 0 | 4 | 0 | 1 | 0 | 0 |  |  |  |  |  |
| $p\_5$ | 1 | 0 | 0 | 0 | 0 | 0 | 0 | 0 | 0 | 0 | 0 | 1 | 1 | 1 | 0 | 1 | 0 | 0 | 0 | 0 | 1 | 1 | 0 | 0 | 0 | 0 | 1 | 1 | -1 | 0 | 0 | 0 | 0 | 0 | 0 | 0 | -3 | 0 | 2 | 2 | 1 | 0 | 0 | -1 | 0 | 1 | 0 | 0 | 4 | -1 | 1 | 1 | 0 |  |  |  |  |  |
| $p\_6$ | 1 | 0 | 0 | 0 | 0 | 0 | 0 | 0 | 0 | 0 | 0 | 1 | 1 | 1 | 0 | 1 | 0 | 0 | 0 | 0 | 0 | 0 | 1 | 1 | 0 | 0 | 1 | 1 | -1 | 0 | 0 | 0 | 0 | 0 | 0 | 0 | -3 | 0 | 2 | 2 | 1 | 0 | 0 | -1 | 0 | 1 | 0 | 0 | 4 | -1 | 1 | 1 | 0 |  |  |  |  |  |
| $p\_7$ | 0 | 0 | 0 | 0 | 0 | 0 | 0 | 0 | 0 | 0 | 0 | 0 | 0 | 0 | 0 | 0 | 0 | 0 | 0 | 0 | 1 | 1 | 0 | 0 | 1 | 1 | 1 | 1 | -1 | 0 | 0 | 0 | 0 | 0 | 0 | 0 | -2 | 0 | 0 | 0 | 0 | 0 | 0 | -1 | 0 | 0 | 1 | 0 | 0 | -1 | 0 | 1 | 0 |  |  |  |  |  |
| $p\_8$ | 0 | 0 | 0 | 0 | 0 | 0 | 0 | 0 | 0 | 0 | 0 | 0 | 0 | 0 | 0 | 0 | 0 | 0 | 0 | 1 | 1 | 0 | 0 | 0 | 0 | 0 | 0 | 0 | 0 | 0 | 0 | 0 | 0 | 0 | 0 | 0 | -1 | 0 | 0 | 0 | 0 | 0 | 0 | 0 | 0 | 0 | 0 | 0 | 0 | 0 | 0 | 0 | 0 |  |  |  |  |  |
| $p\_9$ | 1 | 1 | 1 | 1 | 1 | 2 | 0 | 2 | 2 | 2 | 2 | 0 | 0 | 0 | 0 | 0 | 0 | 0 | 0 | 0 | 0 | 0 | 0 | 0 | 0 | 0 | 0 | 0 | 0 | 2 | 2 | 0 | 0 | 0 | 0 | 0 | 2 | 0 | 0 | 0 | 1 | 0 | 2 | 0 | 0 | 0 | 0 | 0 | 2 | 0 | 0 | 0 | 0 |  |  |  |  |  |

###### The pathway structure:¶

The null space of the integrated stoichiometric matrix has a dimension of 9. Eight of the pathways are the same as the previous chapter (Table 12.10). The new pathway \(\textbf{p}\_9\), represents flow through glycolysis and through the Rapoport-Luebering shunt, bypassing the ATP generating PGK. This pathway thus produces no net ATP, but an inorganic phosphate with the degradation of 23DPG. Thus, the 23DPG ligand that regulates the oxygen affinity of hemoglobin costs the red blood cell the
opportunity to make one ATP.

##### Defining the Steady State¶

The steady state concentrations for the glycolytic intermediates can stay the same as before. The new steady state concentration that needs to be added is that of 23DPG. All glycolytic fluxes stay the same except for PGK. The PGK flux needs to be reduced by the amount that goes through the Rapoport-Luebering shunt: 0.441 mM/hr. This creates minor changes in the estimated PERCs from this steady state data.

```
[28]:
```

```
# Set independent fluxes to determine steady state flux vector
independent_fluxes = {
    core_hb.reactions.SK_glc__D_c: 1.12,
    core_hb.reactions.DM_nadh: 0.2*1.12,
    core_hb.reactions.GSHR : 0.42,
    core_hb.reactions.SK_ade_c: -0.014,
    core_hb.reactions.ADA: 0.01,
    core_hb.reactions.SK_adn_c: -0.01,
    core_hb.reactions.ADNK1: 0.12,
    core_hb.reactions.SK_hxan_c: 0.097,
    core_hb.reactions.DPGM: 0.441}

ssfluxes = core_hb.compute_steady_state_fluxes(
    minspan_paths,
    independent_fluxes,
    update_reactions=True)
table_13_8 = pd.DataFrame(list(ssfluxes.values()), index=reaction_ids,
                          columns=[r"$\textbf{v}_{\mathrm{stst}}$"]).T
```

**Table 13.8:** The steady state fluxes as a summation of the MinSpan pathway vectors.

```
[29]:
```

```
table_13_8
```

```
[29]:
```

|  | HEX1 | PGI | PFK | FBA | TPI | GAPD | PGK | PGM | ENO | PYK | LDH\_L | G6PDH2r | PGL | GND | RPE | RPI | TKT1 | TKT2 | TALA | ADNK1 | NTD7 | ADA | AMPDA | NTD11 | PUNP5 | PPM | PRPPS | ADPT | ADK1 | DPGM | DPGase | HBDPG | HBO1 | HBO2 | HBO3 | HBO4 | ATPM | DM\_nadh | GTHOr | GSHR | SK\_glc\_\_D\_c | SK\_pyr\_c | SK\_lac\_\_L\_c | SK\_ade\_c | SK\_adn\_c | SK\_ins\_c | SK\_hxan\_c | SK\_pi\_c | SK\_h\_c | SK\_h2o\_c | SK\_co2\_c | SK\_nh3\_c | SK\_o2\_c |
| --- | --- | --- | --- | --- | --- | --- | --- | --- | --- | --- | --- | --- | --- | --- | --- | --- | --- | --- | --- | --- | --- | --- | --- | --- | --- | --- | --- | --- | --- | --- | --- | --- | --- | --- | --- | --- | --- | --- | --- | --- | --- | --- | --- | --- | --- | --- | --- | --- | --- | --- | --- | --- | --- |
| $\textbf{v}\_{\mathrm{stst}}$ | 1.120 | 0.910 | 1.105 | 1.105 | 1.105 | 2.308 | 1.867 | 2.308 | 2.308 | 2.308 | 2.084 | 0.210 | 0.210 | 0.210 | 0.195 | 0.015 | 0.098 | 0.098 | 0.098 | 0.120 | 0.120 | 0.010 | 0.014 | 0.014 | 0.097 | 0.097 | 0.014 | 0.014 | -0.014 | 0.441 | 0.441 | 0.000 | 0.000 | 0.000 | 0.000 | 0.000 | 2.243 | 0.224 | 0.420 | 0.420 | 1.120 | 0.224 | 2.084 | -0.014 | -0.010 | -0.073 | 0.097 | 0.000 | 3.596 | -0.317 | 0.210 | 0.024 | 0.000 |

These vectors can be visualized as a bar chart:

```
[30]:
```

```
fig_13_7, ax = plt.subplots(nrows=1, ncols=1, figsize=(10, 5))
# Define indicies for bar chart
indicies = np.arange(len(reaction_ids))+0.5
# Define colors to use
c = plt.cm.coolwarm(np.linspace(0, 1, len(reaction_ids)))
# Plot bar chart
ax.bar(indicies, list(ssfluxes.values()), width=0.8, color=c);
ax.set_xlim([0, len(reaction_ids)]);
# Set labels and adjust ticks
ax.set_xticks(indicies);
ax.set_xticklabels(reaction_ids, rotation="vertical");
ax.set_ylabel("Fluxes (mM/hr)", L_FONT);
ax.set_title("Steady State Fluxes", L_FONT);
ax.plot([0, len(reaction_ids)], [0, 0], "k--");
fig_13_7.tight_layout()
```

**Figure 13.7:** Bar chart of the steady-state fluxes.

###### Computing the rate constants¶

The kinetic constants can be computed from the steady state values of the concentrations using elementary mass action kinetics. The computation is based on Eq. (10.4) The results from this computation is summarized in Table 13.9. This table has all the reaction properties that we need to complete the MASS model.

Note that we set some parameters manually for hemoglobin binding and the oxygen exchange to better reflect the binding of oxygen to hemoglobin.

```
[31]:
```

```
percs = core_hb.calculate_PERCs(
    fluxes={
        r: flux for r, flux in core_hb.steady_state_fluxes.items()
        if r.id not in [
            "ADK1", "SK_o2_c", "HBDPG", "HBO1", "HBO2", "HBO3", "HBO4"]}, # Skip ADK1 and HB reactions
    update_reactions=True)
core_hb.reactions.SK_o2_c.kf = 509726
core_hb.reactions.HBDPG.kf =519613
core_hb.reactions.HBO1.kf = 506935
core_hb.reactions.HBO2.kf = 511077
core_hb.reactions.HBO3.kf = 509243
core_hb.reactions.HBO4.kf = 501595
```

**Table 13.9:** Combined glycolysis, pentose phosphate pathway, AMP salvage network, and hemoglobin model enzymes and transport rates.

```
[32]:
```

```
# Get concentration values for substitution into sympy expressions
value_dict = {sym.Symbol(str(met)): ic
              for met, ic in core_hb.initial_conditions.items()}
value_dict.update({sym.Symbol(str(met)): bc
                   for met, bc in core_hb.boundary_conditions.items()})
table_13_9 = []
# Get symbols and values for table and substitution
for p_key in ["Keq", "kf"]:
    symbol_list, value_list = [], []
    for p_str, value in core_hb.parameters[p_key].items():
        symbol_list.append(r"$%s_{\text{%s}}$" % (p_key[0], p_str.split("_", 1)[-1]))
        value_list.append("{0:.3f}".format(value) if value != INF else r"$\infty$")
        value_dict.update({sym.Symbol(p_str): value})
    table_13_9.extend([symbol_list, value_list])

table_13_9.append(["{0:.6f}".format(float(ratio.subs(value_dict)))
                   for ratio in strip_time(core_hb.get_mass_action_ratios()).values()])
table_13_9.append(["{0:.6f}".format(float(ratio.subs(value_dict)))
                   for ratio in strip_time(core_hb.get_disequilibrium_ratios()).values()])
table_13_9 = pd.DataFrame(np.array(table_13_9).T, index=reaction_ids,
                          columns=[r"$K_{eq}$ Symbol", r"$K_{eq}$ Value", "PERC Symbol",
                                   "PERC Value", r"$\Gamma$", r"$\Gamma/K_{eq}$"])
table_13_9
```

```
[32]:
```

|  | $K\_{eq}$ Symbol | $K\_{eq}$ Value | PERC Symbol | PERC Value | $\Gamma$ | $\Gamma/K\_{eq}$ |
| --- | --- | --- | --- | --- | --- | --- |
| HEX1 | $K\_{\text{HEX1}}$ | 850.000 | $k\_{\text{HEX1}}$ | 0.700 | 0.008809 | 0.000010 |
| PGI | $K\_{\text{PGI}}$ | 0.410 | $k\_{\text{PGI}}$ | 2961.111 | 0.407407 | 0.993677 |
| PFK | $K\_{\text{PFK}}$ | 310.000 | $k\_{\text{PFK}}$ | 34.906 | 0.133649 | 0.000431 |
| FBA | $K\_{\text{FBA}}$ | 0.082 | $k\_{\text{FBA}}$ | 2797.449 | 0.079781 | 0.972937 |
| TPI | $K\_{\text{TPI}}$ | 0.057 | $k\_{\text{TPI}}$ | 33.906 | 0.045500 | 0.796249 |
| GAPD | $K\_{\text{GAPD}}$ | 0.018 | $k\_{\text{GAPD}}$ | 3479.760 | 0.006823 | 0.381183 |
| PGK | $K\_{\text{PGK}}$ | 1800.000 | $k\_{\text{PGK}}$ | 1061655.085 | 1755.073081 | 0.975041 |
| PGM | $K\_{\text{PGM}}$ | 0.147 | $k\_{\text{PGM}}$ | 5017.110 | 0.146184 | 0.994048 |
| ENO | $K\_{\text{ENO}}$ | 1.695 | $k\_{\text{ENO}}$ | 1817.545 | 1.504425 | 0.887608 |
| PYK | $K\_{\text{PYK}}$ | 363000.000 | $k\_{\text{PYK}}$ | 468.247 | 19.570304 | 0.000054 |
| LDH\_L | $K\_{\text{LDH\_L}}$ | 26300.000 | $k\_{\text{LDH\_L}}$ | 1150.285 | 44.132974 | 0.001678 |
| G6PDH2r | $K\_{\text{G6PDH2r}}$ | 1000.000 | $k\_{\text{G6PDH2r}}$ | 21864.589 | 11.875411 | 0.011875 |
| PGL | $K\_{\text{PGL}}$ | 1000.000 | $k\_{\text{PGL}}$ | 122.323 | 21.362698 | 0.021363 |
| GND | $K\_{\text{GND}}$ | 1000.000 | $k\_{\text{GND}}$ | 29287.807 | 43.340651 | 0.043341 |
| RPE | $K\_{\text{RPE}}$ | 3.000 | $k\_{\text{RPE}}$ | 22392.052 | 2.994699 | 0.998233 |
| RPI | $K\_{\text{RPI}}$ | 2.570 | $k\_{\text{RPI}}$ | 2021.058 | 2.566222 | 0.998530 |
| TKT1 | $K\_{\text{TKT1}}$ | 1.200 | $k\_{\text{TKT1}}$ | 2338.070 | 0.932371 | 0.776976 |
| TKT2 | $K\_{\text{TKT2}}$ | 10.300 | $k\_{\text{TKT2}}$ | 1600.141 | 1.921130 | 0.186517 |
| TALA | $K\_{\text{TALA}}$ | 1.050 | $k\_{\text{TALA}}$ | 1237.363 | 0.575416 | 0.548015 |
| ADNK1 | $K\_{\text{ADNK1}}$ | $\infty$ | $k\_{\text{ADNK1}}$ | 62.500 | 13.099557 | 0.000000 |
| NTD7 | $K\_{\text{NTD7}}$ | $\infty$ | $k\_{\text{NTD7}}$ | 1.384 | 0.034591 | 0.000000 |
| ADA | $K\_{\text{ADA}}$ | $\infty$ | $k\_{\text{ADA}}$ | 8.333 | 0.075835 | 0.000000 |
| AMPDA | $K\_{\text{AMPDA}}$ | $\infty$ | $k\_{\text{AMPDA}}$ | 0.161 | 0.010493 | 0.000000 |
| NTD11 | $K\_{\text{NTD11}}$ | $\infty$ | $k\_{\text{NTD11}}$ | 1.400 | 0.250000 | 0.000000 |
| PUNP5 | $K\_{\text{PUNP5}}$ | 0.090 | $k\_{\text{PUNP5}}$ | 83.143 | 0.048000 | 0.533333 |
| PPM | $K\_{\text{PPM}}$ | 13.300 | $k\_{\text{PPM}}$ | 1.643 | 0.211148 | 0.015876 |
| PRPPS | $K\_{\text{PRPPS}}$ | $\infty$ | $k\_{\text{PRPPS}}$ | 0.691 | 0.021393 | 0.000000 |
| ADPT | $K\_{\text{ADPT}}$ | $\infty$ | $k\_{\text{ADPT}}$ | 2800.000 | 108410.125000 | 0.000000 |
| ADK1 | $K\_{\text{ADK1}}$ | 1.650 | $k\_{\text{ADK1}}$ | 100000.000 | 1.650000 | 1.000000 |
| DPGM | $K\_{\text{DPGM}}$ | 2300000.000 | $k\_{\text{DPGM}}$ | 1824.937 | 12757.201646 | 0.005547 |
| DPGase | $K\_{\text{DPGase}}$ | $\infty$ | $k\_{\text{DPGase}}$ | 0.142 | 0.062339 | 0.000000 |
| HBDPG | $K\_{\text{HBDPG}}$ | 0.250 | $k\_{\text{HBDPG}}$ | 519613.000 | 0.250000 | 1.000000 |
| HBO1 | $K\_{\text{HBO1}}$ | 41.835 | $k\_{\text{HBO1}}$ | 506935.000 | 41.835200 | 1.000000 |
| HBO2 | $K\_{\text{HBO2}}$ | 73.212 | $k\_{\text{HBO2}}$ | 511077.000 | 73.211500 | 1.000000 |
| HBO3 | $K\_{\text{HBO3}}$ | 177.799 | $k\_{\text{HBO3}}$ | 509243.000 | 177.799000 | 1.000000 |
| HBO4 | $K\_{\text{HBO4}}$ | 1289.920 | $k\_{\text{HBO4}}$ | 501595.000 | 1289.920000 | 1.000000 |
| ATPM | $K\_{\text{ATPM}}$ | $\infty$ | $k\_{\text{ATPM}}$ | 1.402 | 0.453125 | 0.000000 |
| DM\_nadh | $K\_{\text{DM\_nadh}}$ | $\infty$ | $k\_{\text{DM\_nadh}}$ | 7.442 | 1.956811 | 0.000000 |
| GTHOr | $K\_{\text{GTHOr}}$ | 100.000 | $k\_{\text{GTHOr}}$ | 53.330 | 0.259372 | 0.002594 |
| GSHR | $K\_{\text{GSHR}}$ | 2.000 | $k\_{\text{GSHR}}$ | 0.041 | 0.011719 | 0.005859 |
| SK\_glc\_\_D\_c | $K\_{\text{SK\_glc\_\_D\_c}}$ | $\infty$ | $k\_{\text{SK\_glc\_\_D\_c}}$ | 1.120 | 1.000000 | 0.000000 |
| SK\_pyr\_c | $K\_{\text{SK\_pyr\_c}}$ | 1.000 | $k\_{\text{SK\_pyr\_c}}$ | 744.186 | 0.995008 | 0.995008 |
| SK\_lac\_\_L\_c | $K\_{\text{SK\_lac\_\_L\_c}}$ | 1.000 | $k\_{\text{SK\_lac\_\_L\_c}}$ | 5.790 | 0.735294 | 0.735294 |
| SK\_ade\_c | $K\_{\text{SK\_ade\_c}}$ | 1.000 | $k\_{\text{SK\_ade\_c}}$ | 100000.000 | 1.000000 | 1.000000 |
| SK\_adn\_c | $K\_{\text{SK\_adn\_c}}$ | 1.000 | $k\_{\text{SK\_adn\_c}}$ | 100000.000 | 1.000000 | 1.000000 |
| SK\_ins\_c | $K\_{\text{SK\_ins\_c}}$ | 1.000 | $k\_{\text{SK\_ins\_c}}$ | 100000.000 | 1.000000 | 1.000000 |
| SK\_hxan\_c | $K\_{\text{SK\_hxan\_c}}$ | 1.000 | $k\_{\text{SK\_hxan\_c}}$ | 100000.000 | 1.000000 | 1.000000 |
| SK\_pi\_c | $K\_{\text{SK\_pi\_c}}$ | 1.000 | $k\_{\text{SK\_pi\_c}}$ | 100000.000 | 1.000000 | 1.000000 |
| SK\_h\_c | $K\_{\text{SK\_h\_c}}$ | 1.000 | $k\_{\text{SK\_h\_c}}$ | 133792.163 | 0.701253 | 0.701253 |
| SK\_h2o\_c | $K\_{\text{SK\_h2o\_c}}$ | 1.000 | $k\_{\text{SK\_h2o\_c}}$ | 100000.000 | 1.000000 | 1.000000 |
| SK\_co2\_c | $K\_{\text{SK\_co2\_c}}$ | 1.000 | $k\_{\text{SK\_co2\_c}}$ | 100000.000 | 1.000000 | 1.000000 |
| SK\_nh3\_c | $K\_{\text{SK\_nh3\_c}}$ | 1.000 | $k\_{\text{SK\_nh3\_c}}$ | 100000.000 | 1.000000 | 1.000000 |
| SK\_o2\_c | $K\_{\text{SK\_o2\_c}}$ | 1.000 | $k\_{\text{SK\_o2\_c}}$ | 509726.000 | 1.000000 | 1.000000 |

##### Simulating the Dynamic Mass Balance¶

###### Validating the steady state¶

As usual, we must first ensure that the system is originally at steady state:

```
[33]:
```

```
t0, tf = (0, 1e3)
sim_core = Simulation(core_hb)
sim_core.find_steady_state(
    core_hb, strategy="simulate",
    update_values=True)
conc_sol_ss, flux_sol_ss = sim_core.simulate(
    core_hb, time=(t0, tf, tf*10 + 1))
# Quickly render and display time profiles
conc_sol_ss.view_time_profile()
```

**Figure 13.8:** The merged model after determining the steady state conditions.

We can compare the differences in the initial state of each model before merging and after.

```
[34]:
```

```
fig_13_9, axes = plt.subplots(1, 2, figsize=(9, 4))
(ax1, ax2) = axes.flatten()

# Compare initial conditions
initial_conditions = {
    m.id: ic for m, ic in glycolysis.initial_conditions.items()
    if m.id in core_hb.metabolites}
initial_conditions.update({
    m.id: ic for m, ic in ppp.initial_conditions.items()
    if m.id in core_hb.metabolites})
initial_conditions.update({
    m.id: ic for m, ic in ampsn.initial_conditions.items()
    if m.id in core_hb.metabolites})
initial_conditions.update({
    m.id: ic for m, ic in hemoglobin.initial_conditions.items()
    if m.id in core_hb.metabolites})

plot_comparison(
    core_hb, pd.Series(initial_conditions), compare="concentrations",
    ax=ax1, plot_function="loglog",
    xlabel="Merged Model", ylabel="Independent Models",
    title=("(a) Steady State Concentrations of Species", L_FONT),
    color="blue", xy_line=True, xy_legend="best");

# Compare fluxes
fluxes = {
    r.id: flux for r, flux in glycolysis.steady_state_fluxes.items()
    if r.id in core_hb.reactions}
fluxes.update({
    r.id: flux for r, flux in ppp.steady_state_fluxes.items()
    if r.id in core_hb.reactions})
fluxes.update({
    r.id: flux for r, flux in ampsn.steady_state_fluxes.items()
    if r.id in core_hb.reactions})
fluxes.update({
    r.id: flux for r, flux in hemoglobin.steady_state_fluxes.items()
    if r.id in core_hb.reactions})

plot_comparison(
    core_hb, pd.Series(fluxes), compare="fluxes",
    ax=ax2, plot_function="plot",
    xlabel="Merged Model", ylabel="Independent Models",
    title=("(b) Steady State Fluxes of Reactions", L_FONT),
    color="red", xy_line=True, xy_legend="best");
fig_13_9.tight_layout()
```

**Figure 13.9:** Comparisons between the initial conditions of the merged model and the initial conditions of the independent glycolysis, pentose phosphate pathway, AMP salvage network and hemoglobin models for (a) the species and (b) the fluxes.

###### Response to an increased \(k\_{ATPM}\)¶

Once we have ensured that the system is originally at steady state, we perform the same simulation as in the last chapter by increasing the rate of ATP utilization.

```
[35]:
```

```
conc_sol, flux_sol = sim_core.simulate(
    core_hb, time=(t0, tf, tf * 10 + 1),
    perturbations={"kf_ATPM": "kf_ATPM * 1.5"})
```

```
[36]:
```

```
fig_13_10, axes = plt.subplots(nrows=2, ncols=1, figsize=(10, 8));
(ax1, ax2) = axes.flatten()

plot_time_profile(
    conc_sol, ax=ax1, legend="right outside",
    plot_function="loglog",
    xlabel="Time [hr]", ylabel="Concentration [mM]",
    title=("Concentration Profile", L_FONT));

plot_time_profile(
    flux_sol, ax=ax2, legend="right outside",
    plot_function="semilogx",
    xlabel="Time [hr]", ylabel="Flux [mM/hr]",
    title=("Flux Profile", L_FONT));
fig_13_10.tight_layout()
```

**Figure 13.10:** Simulating the combined system from the steady state with 50% increase in the rate of ATP utilization at \(t = 0\).

#### The proton node The proton node now has a connectivity of 16 with 12 production reactions and 4 utilization reactions. The additional proton production reaction is due to the addition of DPGM to the model.

```
[37]:
```

```
fig_13_11 = plt.figure(figsize=(18, 6))
gs = fig_13_11.add_gridspec(nrows=3, ncols=2, width_ratios=[1.5, 1])

ax1 = fig_13_11.add_subplot(gs[0, 0])
ax2 = fig_13_11.add_subplot(gs[1, 0])
ax3 = fig_13_11.add_subplot(gs[2, 0])
ax4 = fig_13_11.add_subplot(gs[:, 1])

plot_time_profile(
    conc_sol, observable="h_c", ax=ax1,
    legend="right outside", plot_function="semilogx",
    xlim=(1e-6, tf), ylim=(8.5e-5, 1e-4*1.025),
    xlabel="Time [hr]", ylabel="Concentrations [mM]",
    title=("(a) Proton Concentration", L_FONT));

fluxes_in = ["HEX1", "PFK", "GAPD", "ATPM", "DM_nadh", "G6PDH2r",
             "PGL", "GSHR", "ADNK1", "PRPPS", "ADPT", "DPGM"]
plot_time_profile(
    flux_sol, observable=fluxes_in, ax=ax2,
    legend="right outside", plot_function="semilogx",
    xlim=(1e-6, tf),  ylim=(-0.4, 3.5),
    xlabel="Time [hr]", ylabel="Fluxes [mM/hr]",
    title=("(b) Fluxes in", L_FONT));

fluxes_out = ["PYK", "LDH_L", "SK_h_c", "GSHR"]
plot_time_profile(
    flux_sol, observable=fluxes_out, ax=ax3,
    legend="right outside", plot_function="semilogx",
    xlim=(1e-6, tf),  ylim=(1.2, 5),
    xlabel="Time [hr]", ylabel="Fluxes [mM/hr]",
    title=("(c) Fluxes out", L_FONT));

for flux_id, variables in zip(["Net_Flux_In", "Net_Flux_Out"],
                              [fluxes_in, fluxes_out]):
    flux_sol.make_aggregate_solution(
        flux_id, equation=" + ".join(variables), variables=variables)

time_points = [t0, 1e-1, 1e0, 1e1, 1e2, tf]
time_point_colors = [
    mpl.colors.to_hex(c)
    for c in mpl.cm.Reds(np.linspace(0.3, 1, len(time_points)))]

plot_phase_portrait(
    flux_sol, x="Net_Flux_In", y="Net_Flux_Out", ax=ax4,
    xlim=(7.5, 9.5), ylim=(7.5, 9.5),
    xlabel="Fluxes in [mm/Hr]", ylabel="Fluxes out [mm/Hr]",
    title=("(d) Phase Portrait of Fluxes", L_FONT),
    annotate_time_points=time_points,
    annotate_time_points_color=time_point_colors,
    annotate_time_points_legend="best");
fig_13_11.tight_layout()
```

**Figure 13.11:** The time profiles of the (a) proton concentration, (b) the fluxes that make protons, (c) the fluxes that use protons and (d) the phase portrait of the net flux in and net flux out (darker red colors indicate slower time scales).

###### Dynamic simulation: normal circulation¶

We can repeat the response to the oscillatory plasma concentration of oxygen that we performed for the hemoglobin subsystem alone (Figure 13.3). The response of the integrated system demonstrates dynamic decoupling between the loading and offloading of oxygen on hemoglobin and glycolytic activity.

The simulation demonstrates dynamic decoupling at the rapid time scales, namely that the loading and off-loading of oxygen on the minute time scale does not have significant ripple effects into glycolytic functions. The oxygen delivery and hemoglobin are unaltered as compared before (Figure 13.3a,b). The input into the Rapoport-Luebering shunt is effectively a constant while the output is slightly oscillatory Figure 13.12a, leading to small drift downwards in the first 0.1 hours and additional
minor downstream effects on lower glycolysis, Figure 13.12b. Notice that the PYK flux is barely perturbed.

```
[38]:
```

```
(t0, tf) = (0, 0.5)

conc_sol, flux_sol = sim_core.simulate(
    core_hb, time=(t0, tf, 1e6+1),
    perturbations={"o2_b": "(70 + 30*sin(120*pi*t))*2.8684*1e-4"},
    interpolate=True)

fig_13_12, axes = plt.subplots(nrows=2, ncols=1, figsize=(10, 8))
(ax1, ax2) = axes.flatten()

plot_time_profile(
    flux_sol, observable=["DPGM", "DPGase"], ax=ax1,
    legend="right outside", xlabel="Time [hr]", ylabel="Flux [mM/hr]",
    title=("(a) Fluxes through R.L. shunt", L_FONT));

plot_time_profile(
    flux_sol, observable=["PGM", "ENO", "PYK"], ax=ax2,
    legend="right outside", xlabel="Time [hr]", ylabel="Flux [mM/hr]",
    title=("(b) Lower glycolytic fluxes", L_FONT));
fig_13_12.tight_layout()
```

**Figure 13.12:** The binding states of hemoglobin during normal circulation as computed from the integrated core network and Rapoport-Luebering shunt model. (a): The fluxes through the Rapoport-Luebering shunt. (b): The lower glycolytic fluxes.

###### Pools and ratios¶

The pools and ratios will be the same as for the two individual subsystems, with the exception of the role of 23DPG. In the total phosphate pool, it will count as a 2 since its two phosphate bonds. However, it has a high-energy bond value of 1 in the glycolytic energy pool \((GP^+)\) as it can only generate one ATP through degradation through pyruvate kinase. In the glycolytic redox pool it has a value of 0 as it cannot generate NADH.

##### Summary¶

- The ligand binding to macromolecules can be described by chemical equations. Stoichiometric matrices that describe the binding states of macromolecules can be formulated. Such matrices can be integrated with stoichiometric matrices describing metabolic networks, thus forming integrated models of small molecules and protein.
- Since hemoglobin is confined to the system, the rapid binding of the ligands lead to equilibrium states. These are not quasi-equilibria since the serial binding is a ‘dead end,’ meaning there is no output on the other end of the series of binding steps. Thus, the ligand bound states of the macromolecule are equilibrium variables that match the steady state values of the ligands. The ligands are in pathways that leave and enter the system and are thus in a steady state.
- The oxygen loading of hemoglobin has fast and slow physiologically meaningful time scales: the one minute circulation time of the red blood cell, during which the oxygen molecules load and unload from hemoglobin, and the slow changes in 23DPG levels, resulting from changes in \(pO\_2\). The former does not show dynamic coupling with glycolysis and the pentose pathway, while the latter does.
- The integration issues here are simple. The role of 23DPG in the pools and ratios needs to be defined. The flux split into the Rapoport-Luebering shunt leads to a different definition of PERCs for PGK, as the flux changes, but the steady state concentrations are assumed to be the same with and without the shunt. This easy integration is a reflection of the MASS procedure and its flexibility.

\(\tiny{\text{© B. Ø. Palsson 2011;}\ \text{This publication is in copyright.}\\ \text{Subject to statutory exception and to the provisions of relevant collective licensing agreements,}\\ \text{no reproduction of any part may take place without the written permission of Cambridge University Press.}}\)

#### Regulated Enzymes¶

The proteins that are of particular interest from a dynamic simulation perspective are those that regulate fluxes. Regulatory enzymes bind to many ligands. Their functional states can be described in a similar fashion as demonstrated for hemoglobin in the previous chapter. Their states and functions can thus be readily integrated into MASS models. In this chapter we detail the molecular mechanisms for a key regulatory enzyme in glycolysis, *phosphofructokinase*. First we describe the enzyme,
then detail the module, or subnetwork, that it represents, and finally integrate them into the metabolic model. We then simulate the altered dynamic network states that result from these regulatory interactions.

**MASSpy** will be used to demonstrate some of the topics in this chapter.

```
[1]:
```

```
from mass import (
    MassModel, MassMetabolite, MassReaction,
    Simulation, MassSolution)
from mass.test import create_test_model
from mass.util.expressions import Keq2k, k2Keq, strip_time
from mass.util.matrix import nullspace, left_nullspace, matrix_rank
from mass.visualization import (
    plot_time_profile, plot_phase_portrait, plot_tiled_phase_portraits,
    plot_comparison)
```

Other useful packages are also imported at this time.

```
[2]:
```

```
from os import path

from cobra import DictList
import matplotlib as mpl
import matplotlib.pyplot as plt
from mpl_toolkits.mplot3d import Axes3D
import numpy as np
import pandas as pd
import sympy as sym
```

Some options and variables used throughout the notebook are also declared here.

```
[3]:
```

```
pd.set_option("display.max_rows", 100)
pd.set_option("display.max_columns", 100)
pd.set_option('display.max_colwidth', None)
pd.options.display.float_format = '{:,.3f}'.format
S_FONT = {"size": "small"}
L_FONT = {"size": "large"}
INF = float("inf")
```

##### Phosphofructokinase¶

Phosphofructokinase (PFK) is a tetrameric enzyme. There are isoforms of the subunits of the enzyme, meaning that there is more than one gene for each subunit and the genes are not identical. The isoforms are differentially expressed in various tissues, and therefore different versions of the enzyme are active in different tissues. The regulation of PFK is quite complicated [Okar01]. Here, for illustrative purposes, we will consider a homotetrameric form of PFK, Figure 14.1 with one activator
(AMP) and one inhibitor (ATP).

**Figure 14.1:** The phosphofructokinase, PFK, subnetwork; the reaction schema for catalysis, for regulation, and the exchanges with the rest of the metabolic network. The numerical values of the dissociation constants are taken from [Joshi 1990]; \(K\_{ATP}\) is 0.068 mM, \(K\_{F6P}\) is 0.1 mM, \(K\_{a}\) is 0.33 mM and \(K\_{i}\) is 0.01 mM. The \(K\_{i}\) binding constant for ATP as an inhibitor is increased by a factor of ten since magnesium complexing of ATP is not
considered here. The allosteric constant \(L\) is 0.0011.

###### The reaction catalyzed¶

PFK is a major regulatory enzyme in glycolysis. It catalyzes the reaction

\[\begin{equation} \text{F6P} + \text{ATP} \stackrel{\text{PFK}}{\rightarrow} \text{FDP} + \text{ADP} + \text{H} \tag{14.1} \end{equation}\]

In Chapter 10 we introduced this reaction as a part of the glycolytic pathway.

###### The subnetwork¶

The detailed reaction mechanism of PFK is shown in Figure 14.1. The reactants, F6P and ATP, and products, FDP and ADP, enter and leave the subnetwork. These exchanges will reach a steady state. The regulators, AMP is an activator and ATP is an inhibitor, also enter and leave the subnetwork. They reach an equilibrium in the steady state, as they have no flow through the subnetwork. The bound states of PFK thus equilibrate with its regulators while the reactants and products will flow through the
subnetwork.

###### Placing the PFK subnetwork into a known network environment¶

We will first analyze the PFK subnetwork by itself. To do so, we have to add exchange reactions from the network environment. The numerical values for the concentrations external to the PFK subnetwork have to be added. We can then explore the properties of this subnetwork model by itself.

First we establish the PFK subnetwork:

```
[4]:
```

```
PFK = create_test_model("SB2_PFK")
```

Then we place the PFK subnetwork into its network context:

```
[5]:
```

```
for met in ["f6p_c", "fdp_c", "amp_c", "adp_c", "atp_c", "h_c"]:
    PFK.add_boundary(met, boundary_type="sink", boundary_condition=1);
PFK
```

```
[5]:
```

|  |  |
| --- | --- |
| **Name** | PFK |
| **Memory address** | 0x07f9cc6d1aad0 |
| **Stoichiometric Matrix** | 26x30 |
| **Matrix Rank** | 25 |
| **Subsystem** | Glycolysis |
| **Number of ligands** | 6 |
| **Number of enzyme module forms** | 20 |
| **Initial conditions defined** | 26/26 |
| **Number of enzyme module reactions** | 24 |
| **Total enzyme concentration** | 3.3e-05 |
| **Enzyme rate** | 1.12 |
| **Number of groups** | 16 |
| **Compartments** | Cytosol |

```
[6]:
```

```
new_metabolite_order = ['f6p_c', 'fdp_c', 'amp_c', 'adp_c', 'atp_c', 'h_c',
                        'pfk_R0_c', 'pfk_R0_A_c', 'pfk_R0_AF_c',
                        'pfk_R1_c', 'pfk_R1_A_c', 'pfk_R1_AF_c',
                        'pfk_R2_c', 'pfk_R2_A_c', 'pfk_R2_AF_c',
                        'pfk_R3_c', 'pfk_R3_A_c', 'pfk_R3_AF_c',
                        'pfk_R4_c', 'pfk_R4_A_c', 'pfk_R4_AF_c',
                        'pfk_T0_c','pfk_T1_c', 'pfk_T2_c', 'pfk_T3_c', 'pfk_T4_c']

if len(PFK.metabolites) == len(new_metabolite_order):
    PFK.metabolites = DictList(PFK.metabolites.get_by_any(new_metabolite_order))

new_reaction_order = ["SK_f6p_c", "SK_fdp_c", "SK_amp_c",
                      "SK_adp_c", "SK_atp_c", "SK_h_c",
                      "PFK_R01", "PFK_R02", "PFK_R03",
                      "PFK_R10", "PFK_R11", "PFK_R12", "PFK_R13",
                      "PFK_R20", "PFK_R21", "PFK_R22", "PFK_R23",
                      "PFK_R30", "PFK_R31", "PFK_R32", "PFK_R33",
                      "PFK_R40", "PFK_R41", "PFK_R42", "PFK_R43",
                      "PFK_L", "PFK_T1", "PFK_T2", "PFK_T3", "PFK_T4"]

if len(PFK.reactions) == len(new_reaction_order):
    PFK.reactions = DictList(PFK.reactions.get_by_any(new_reaction_order))
PFK.update_S(array_type="DataFrame");
```

```
[6]:
```

|  | SK\_f6p\_c | SK\_fdp\_c | SK\_amp\_c | SK\_adp\_c | SK\_atp\_c | SK\_h\_c | PFK\_R01 | PFK\_R02 | PFK\_R03 | PFK\_R10 | PFK\_R11 | PFK\_R12 | PFK\_R13 | PFK\_R20 | PFK\_R21 | PFK\_R22 | PFK\_R23 | PFK\_R30 | PFK\_R31 | PFK\_R32 | PFK\_R33 | PFK\_R40 | PFK\_R41 | PFK\_R42 | PFK\_R43 | PFK\_L | PFK\_T1 | PFK\_T2 | PFK\_T3 | PFK\_T4 |
| --- | --- | --- | --- | --- | --- | --- | --- | --- | --- | --- | --- | --- | --- | --- | --- | --- | --- | --- | --- | --- | --- | --- | --- | --- | --- | --- | --- | --- | --- | --- |
| f6p\_c | -1.000 | 0.000 | 0.000 | 0.000 | 0.000 | 0.000 | 0.000 | -1.000 | 0.000 | 0.000 | 0.000 | -1.000 | 0.000 | 0.000 | 0.000 | -1.000 | 0.000 | 0.000 | 0.000 | -1.000 | 0.000 | 0.000 | 0.000 | -1.000 | 0.000 | 0.000 | 0.000 | 0.000 | 0.000 | 0.000 |
| fdp\_c | 0.000 | -1.000 | 0.000 | 0.000 | 0.000 | 0.000 | 0.000 | 0.000 | 1.000 | 0.000 | 0.000 | 0.000 | 1.000 | 0.000 | 0.000 | 0.000 | 1.000 | 0.000 | 0.000 | 0.000 | 1.000 | 0.000 | 0.000 | 0.000 | 1.000 | 0.000 | 0.000 | 0.000 | 0.000 | 0.000 |
| amp\_c | 0.000 | 0.000 | -1.000 | 0.000 | 0.000 | 0.000 | 0.000 | 0.000 | 0.000 | -1.000 | 0.000 | 0.000 | 0.000 | -1.000 | 0.000 | 0.000 | 0.000 | -1.000 | 0.000 | 0.000 | 0.000 | -1.000 | 0.000 | 0.000 | 0.000 | 0.000 | 0.000 | 0.000 | 0.000 | 0.000 |
| adp\_c | 0.000 | 0.000 | 0.000 | -1.000 | 0.000 | 0.000 | 0.000 | 0.000 | 1.000 | 0.000 | 0.000 | 0.000 | 1.000 | 0.000 | 0.000 | 0.000 | 1.000 | 0.000 | 0.000 | 0.000 | 1.000 | 0.000 | 0.000 | 0.000 | 1.000 | 0.000 | 0.000 | 0.000 | 0.000 | 0.000 |
| atp\_c | 0.000 | 0.000 | 0.000 | 0.000 | -1.000 | 0.000 | -1.000 | 0.000 | 0.000 | 0.000 | -1.000 | 0.000 | 0.000 | 0.000 | -1.000 | 0.000 | 0.000 | 0.000 | -1.000 | 0.000 | 0.000 | 0.000 | -1.000 | 0.000 | 0.000 | 0.000 | -1.000 | -1.000 | -1.000 | -1.000 |
| h\_c | 0.000 | 0.000 | 0.000 | 0.000 | 0.000 | -1.000 | 0.000 | 0.000 | 1.000 | 0.000 | 0.000 | 0.000 | 1.000 | 0.000 | 0.000 | 0.000 | 1.000 | 0.000 | 0.000 | 0.000 | 1.000 | 0.000 | 0.000 | 0.000 | 1.000 | 0.000 | 0.000 | 0.000 | 0.000 | 0.000 |
| pfk\_R0\_c | 0.000 | 0.000 | 0.000 | 0.000 | 0.000 | 0.000 | -1.000 | 0.000 | 1.000 | -1.000 | 0.000 | 0.000 | 0.000 | 0.000 | 0.000 | 0.000 | 0.000 | 0.000 | 0.000 | 0.000 | 0.000 | 0.000 | 0.000 | 0.000 | 0.000 | -1.000 | 0.000 | 0.000 | 0.000 | 0.000 |
| pfk\_R0\_A\_c | 0.000 | 0.000 | 0.000 | 0.000 | 0.000 | 0.000 | 1.000 | -1.000 | 0.000 | 0.000 | 0.000 | 0.000 | 0.000 | 0.000 | 0.000 | 0.000 | 0.000 | 0.000 | 0.000 | 0.000 | 0.000 | 0.000 | 0.000 | 0.000 | 0.000 | 0.000 | 0.000 | 0.000 | 0.000 | 0.000 |
| pfk\_R0\_AF\_c | 0.000 | 0.000 | 0.000 | 0.000 | 0.000 | 0.000 | 0.000 | 1.000 | -1.000 | 0.000 | 0.000 | 0.000 | 0.000 | 0.000 | 0.000 | 0.000 | 0.000 | 0.000 | 0.000 | 0.000 | 0.000 | 0.000 | 0.000 | 0.000 | 0.000 | 0.000 | 0.000 | 0.000 | 0.000 | 0.000 |
| pfk\_R1\_c | 0.000 | 0.000 | 0.000 | 0.000 | 0.000 | 0.000 | 0.000 | 0.000 | 0.000 | 1.000 | -1.000 | 0.000 | 1.000 | -1.000 | 0.000 | 0.000 | 0.000 | 0.000 | 0.000 | 0.000 | 0.000 | 0.000 | 0.000 | 0.000 | 0.000 | 0.000 | 0.000 | 0.000 | 0.000 | 0.000 |
| pfk\_R1\_A\_c | 0.000 | 0.000 | 0.000 | 0.000 | 0.000 | 0.000 | 0.000 | 0.000 | 0.000 | 0.000 | 1.000 | -1.000 | 0.000 | 0.000 | 0.000 | 0.000 | 0.000 | 0.000 | 0.000 | 0.000 | 0.000 | 0.000 | 0.000 | 0.000 | 0.000 | 0.000 | 0.000 | 0.000 | 0.000 | 0.000 |
| pfk\_R1\_AF\_c | 0.000 | 0.000 | 0.000 | 0.000 | 0.000 | 0.000 | 0.000 | 0.000 | 0.000 | 0.000 | 0.000 | 1.000 | -1.000 | 0.000 | 0.000 | 0.000 | 0.000 | 0.000 | 0.000 | 0.000 | 0.000 | 0.000 | 0.000 | 0.000 | 0.000 | 0.000 | 0.000 | 0.000 | 0.000 | 0.000 |
| pfk\_R2\_c | 0.000 | 0.000 | 0.000 | 0.000 | 0.000 | 0.000 | 0.000 | 0.000 | 0.000 | 0.000 | 0.000 | 0.000 | 0.000 | 1.000 | -1.000 | 0.000 | 1.000 | -1.000 | 0.000 | 0.000 | 0.000 | 0.000 | 0.000 | 0.000 | 0.000 | 0.000 | 0.000 | 0.000 | 0.000 | 0.000 |
| pfk\_R2\_A\_c | 0.000 | 0.000 | 0.000 | 0.000 | 0.000 | 0.000 | 0.000 | 0.000 | 0.000 | 0.000 | 0.000 | 0.000 | 0.000 | 0.000 | 1.000 | -1.000 | 0.000 | 0.000 | 0.000 | 0.000 | 0.000 | 0.000 | 0.000 | 0.000 | 0.000 | 0.000 | 0.000 | 0.000 | 0.000 | 0.000 |
| pfk\_R2\_AF\_c | 0.000 | 0.000 | 0.000 | 0.000 | 0.000 | 0.000 | 0.000 | 0.000 | 0.000 | 0.000 | 0.000 | 0.000 | 0.000 | 0.000 | 0.000 | 1.000 | -1.000 | 0.000 | 0.000 | 0.000 | 0.000 | 0.000 | 0.000 | 0.000 | 0.000 | 0.000 | 0.000 | 0.000 | 0.000 | 0.000 |
| pfk\_R3\_c | 0.000 | 0.000 | 0.000 | 0.000 | 0.000 | 0.000 | 0.000 | 0.000 | 0.000 | 0.000 | 0.000 | 0.000 | 0.000 | 0.000 | 0.000 | 0.000 | 0.000 | 1.000 | -1.000 | 0.000 | 1.000 | -1.000 | 0.000 | 0.000 | 0.000 | 0.000 | 0.000 | 0.000 | 0.000 | 0.000 |
| pfk\_R3\_A\_c | 0.000 | 0.000 | 0.000 | 0.000 | 0.000 | 0.000 | 0.000 | 0.000 | 0.000 | 0.000 | 0.000 | 0.000 | 0.000 | 0.000 | 0.000 | 0.000 | 0.000 | 0.000 | 1.000 | -1.000 | 0.000 | 0.000 | 0.000 | 0.000 | 0.000 | 0.000 | 0.000 | 0.000 | 0.000 | 0.000 |
| pfk\_R3\_AF\_c | 0.000 | 0.000 | 0.000 | 0.000 | 0.000 | 0.000 | 0.000 | 0.000 | 0.000 | 0.000 | 0.000 | 0.000 | 0.000 | 0.000 | 0.000 | 0.000 | 0.000 | 0.000 | 0.000 | 1.000 | -1.000 | 0.000 | 0.000 | 0.000 | 0.000 | 0.000 | 0.000 | 0.000 | 0.000 | 0.000 |
| pfk\_R4\_c | 0.000 | 0.000 | 0.000 | 0.000 | 0.000 | 0.000 | 0.000 | 0.000 | 0.000 | 0.000 | 0.000 | 0.000 | 0.000 | 0.000 | 0.000 | 0.000 | 0.000 | 0.000 | 0.000 | 0.000 | 0.000 | 1.000 | -1.000 | 0.000 | 1.000 | 0.000 | 0.000 | 0.000 | 0.000 | 0.000 |
| pfk\_R4\_A\_c | 0.000 | 0.000 | 0.000 | 0.000 | 0.000 | 0.000 | 0.000 | 0.000 | 0.000 | 0.000 | 0.000 | 0.000 | 0.000 | 0.000 | 0.000 | 0.000 | 0.000 | 0.000 | 0.000 | 0.000 | 0.000 | 0.000 | 1.000 | -1.000 | 0.000 | 0.000 | 0.000 | 0.000 | 0.000 | 0.000 |
| pfk\_R4\_AF\_c | 0.000 | 0.000 | 0.000 | 0.000 | 0.000 | 0.000 | 0.000 | 0.000 | 0.000 | 0.000 | 0.000 | 0.000 | 0.000 | 0.000 | 0.000 | 0.000 | 0.000 | 0.000 | 0.000 | 0.000 | 0.000 | 0.000 | 0.000 | 1.000 | -1.000 | 0.000 | 0.000 | 0.000 | 0.000 | 0.000 |
| pfk\_T0\_c | 0.000 | 0.000 | 0.000 | 0.000 | 0.000 | 0.000 | 0.000 | 0.000 | 0.000 | 0.000 | 0.000 | 0.000 | 0.000 | 0.000 | 0.000 | 0.000 | 0.000 | 0.000 | 0.000 | 0.000 | 0.000 | 0.000 | 0.000 | 0.000 | 0.000 | 1.000 | -1.000 | 0.000 | 0.000 | 0.000 |
| pfk\_T1\_c | 0.000 | 0.000 | 0.000 | 0.000 | 0.000 | 0.000 | 0.000 | 0.000 | 0.000 | 0.000 | 0.000 | 0.000 | 0.000 | 0.000 | 0.000 | 0.000 | 0.000 | 0.000 | 0.000 | 0.000 | 0.000 | 0.000 | 0.000 | 0.000 | 0.000 | 0.000 | 1.000 | -1.000 | 0.000 | 0.000 |
| pfk\_T2\_c | 0.000 | 0.000 | 0.000 | 0.000 | 0.000 | 0.000 | 0.000 | 0.000 | 0.000 | 0.000 | 0.000 | 0.000 | 0.000 | 0.000 | 0.000 | 0.000 | 0.000 | 0.000 | 0.000 | 0.000 | 0.000 | 0.000 | 0.000 | 0.000 | 0.000 | 0.000 | 0.000 | 1.000 | -1.000 | 0.000 |
| pfk\_T3\_c | 0.000 | 0.000 | 0.000 | 0.000 | 0.000 | 0.000 | 0.000 | 0.000 | 0.000 | 0.000 | 0.000 | 0.000 | 0.000 | 0.000 | 0.000 | 0.000 | 0.000 | 0.000 | 0.000 | 0.000 | 0.000 | 0.000 | 0.000 | 0.000 | 0.000 | 0.000 | 0.000 | 0.000 | 1.000 | -1.000 |
| pfk\_T4\_c | 0.000 | 0.000 | 0.000 | 0.000 | 0.000 | 0.000 | 0.000 | 0.000 | 0.000 | 0.000 | 0.000 | 0.000 | 0.000 | 0.000 | 0.000 | 0.000 | 0.000 | 0.000 | 0.000 | 0.000 | 0.000 | 0.000 | 0.000 | 0.000 | 0.000 | 0.000 | 0.000 | 0.000 | 0.000 | 1.000 |

With these additions we can re-examine the properties of this model of the PFK subnetwork.

###### Bound states of PFK¶

The enzyme exists in two natural forms, T, or tight, and R, or relaxed, recall Section 5.5.

\[\begin{equation} R\_0 \leftrightharpoons T\_0 \tag{14.2} \end{equation}\]

The R form is catalytically active and the T form is inactive. The two forms are in equilibrium as

\[\begin{equation} L = \frac{T\_0}{R\_0} = 0.0011 \tag{14.3} \end{equation}\]

where \(L\) is the allosteric constant for PFK.

There are many ligands that can bind to PFK to modulate its activity by altering the balance of the T and R forms. Here we will consider AMP as an activator and ATP as an inhibitor. AMP will bind to the R state;

\[\begin{split}\begin{align} R\_0 + \text{AMP} &\leftrightharpoons R\_1 \tag{14.4} \\ \\ R\_1 + \text{AMP} &\leftrightharpoons R\_2 \tag{14.5} \\ \\ R\_2 + \text{AMP} &\leftrightharpoons R\_3 \tag{14.6} \\ \\ R\_3 + \text{AMP} &\leftrightharpoons R\_4 \tag{14.7} \\ \end{align}\end{split}\]

and ATP will bind to the T state:

\[\begin{split}\begin{align} T\_0 + \text{ATP} \leftrightharpoons T\_1 \tag{14.8} \\ \\ T\_1 + \text{ATP} \leftrightharpoons T\_2 \tag{14.9} \\ \\ T\_2 + \text{ATP} \leftrightharpoons T\_3 \tag{14.10}\\ \\ T\_3 + \text{ATP} \leftrightharpoons T\_4 \tag{14.11}\\ \\ \end{align}\end{split}\]

The chemical reaction, see Eq. (14.1), will proceed in three steps:

\(\textbf{1.}\) the binding of the cofactor ATP

\[\begin{equation} \stackrel{\text{substrate in}}{\longrightarrow} \text{ATP} + R\_i \leftrightharpoons R\_{i, A} \tag{14.12} \end{equation}\]

\(\textbf{2.}\) the binding of the substrate F6P

\[\begin{equation} \stackrel{\text{substrate in}}{\longrightarrow} \text{F6P} + R\_{i, A} \leftrightharpoons R\_{i, AF} \tag{14.13} \end{equation}\]

\(\textbf{3.}\)the catalytic conversion to the products

\[\begin{equation} R\_{i, AF} \stackrel{\text{transformation}}{\longrightarrow} R\_{i} + \text{FDP} + \text{ADP} + \text{H} \stackrel{\text{products out}}{\longrightarrow} \end{equation}\]

\(\textbf{4.}\) and the release of the enzyme.

The subscript \(i\ (i=0,1,2,3,4)\) indicates how many activator molecules (AMP) are bound to the R from of the enzyme. The free from \((i=0)\) and all the bound forms are catalytically active.

**Figure 14.2:** Pictorial representation of the PFK subnetwork. The inactive tight \((T\_i)\) states are designated with a square, and the substrate binding sites, designated with an open circle, are not accessible. The shading of the sub-squares indicate that the inhibitor (ATP) occupies that site. The active relaxed form \((R\_i)\) are shown with a circle, where the binding sites for the activator (AMP) are indicated. Shading means that the AMP binding site is occupied. The semi-circle
below the circle shows that the binding sites are now on the surface of the protein and can now be occupied by the reactants (ATP and F6P). The \((R\_{i, AF})\) state catalyzes the reaction.

A pictorial representation of the PKF subnetwork is shown in FIgure 14.2, and a MASS model of the PFK subnetwork is shown below:

###### The elemental matrix¶

The elemental composition of the compounds in the PFK subnetwork are shown in Table 14.1. The low molecular weight compounds are detailed in terms of their elemental composition while the enzyme itself is treated as one moiety. It never leaves the system and is never chemically modified, so we can treat it as one entity.

**Table 14.1:** The elemental composition of the compounds in the PFK subnetwork. The PFK protein molecule is treated as one moiety.

```
[7]:
```

```
table_14_1 = PFK.get_elemental_matrix(array_type="DataFrame",
                                      dtype=np.int64)
table_14_1
```

```
[7]:
```

|  | f6p\_c | fdp\_c | amp\_c | adp\_c | atp\_c | h\_c | pfk\_R0\_c | pfk\_R0\_A\_c | pfk\_R0\_AF\_c | pfk\_R1\_c | pfk\_R1\_A\_c | pfk\_R1\_AF\_c | pfk\_R2\_c | pfk\_R2\_A\_c | pfk\_R2\_AF\_c | pfk\_R3\_c | pfk\_R3\_A\_c | pfk\_R3\_AF\_c | pfk\_R4\_c | pfk\_R4\_A\_c | pfk\_R4\_AF\_c | pfk\_T0\_c | pfk\_T1\_c | pfk\_T2\_c | pfk\_T3\_c | pfk\_T4\_c |
| --- | --- | --- | --- | --- | --- | --- | --- | --- | --- | --- | --- | --- | --- | --- | --- | --- | --- | --- | --- | --- | --- | --- | --- | --- | --- | --- |
| C | 6 | 6 | 10 | 10 | 10 | 0 | 0 | 10 | 16 | 10 | 20 | 26 | 20 | 30 | 36 | 30 | 40 | 46 | 40 | 50 | 56 | 0 | 10 | 20 | 30 | 40 |
| H | 11 | 10 | 12 | 12 | 12 | 1 | 0 | 12 | 23 | 12 | 24 | 35 | 24 | 36 | 47 | 36 | 48 | 59 | 48 | 60 | 71 | 0 | 12 | 24 | 36 | 48 |
| O | 9 | 12 | 7 | 10 | 13 | 0 | 0 | 13 | 22 | 7 | 20 | 29 | 14 | 27 | 36 | 21 | 34 | 43 | 28 | 41 | 50 | 0 | 13 | 26 | 39 | 52 |
| P | 1 | 2 | 1 | 2 | 3 | 0 | 0 | 3 | 4 | 1 | 4 | 5 | 2 | 5 | 6 | 3 | 6 | 7 | 4 | 7 | 8 | 0 | 3 | 6 | 9 | 12 |
| N | 0 | 0 | 5 | 5 | 5 | 0 | 0 | 5 | 5 | 5 | 10 | 10 | 10 | 15 | 15 | 15 | 20 | 20 | 20 | 25 | 25 | 0 | 5 | 10 | 15 | 20 |
| S | 0 | 0 | 0 | 0 | 0 | 0 | 0 | 0 | 0 | 0 | 0 | 0 | 0 | 0 | 0 | 0 | 0 | 0 | 0 | 0 | 0 | 0 | 0 | 0 | 0 | 0 |
| q | -2 | -4 | -2 | -3 | -4 | 1 | 0 | -4 | -6 | -2 | -6 | -8 | -4 | -8 | -10 | -6 | -10 | -12 | -8 | -12 | -14 | 0 | -4 | -8 | -12 | -16 |
| [PFK] | 0 | 0 | 0 | 0 | 0 | 0 | 1 | 1 | 1 | 1 | 1 | 1 | 1 | 1 | 1 | 1 | 1 | 1 | 1 | 1 | 1 | 1 | 1 | 1 | 1 | 1 |

###### The stoichiometric matrix¶

The reactions that correspond to this subnetwork of binding states and catalytic conversions can be summarized in a stoichiometric matrix, shown in Table 14.2. This matrix is 26 x 30 of rank 25.

**Table 14.2:** The stoichiometric matrix for the PFK subnetwork. The last eight columns are the exchange rates (orange), the connectivity numbers (red), and the time invariant pool (lime green). The next set of columns (yellow) are the PFK reaction itself (five sets, one for each \(R\_i, i = (0, 1, 2, 3, 4)\). The next five columns (green) are conversion into the T form and the binding reactions for the inhibitor. The first six rows (orange) are the compounds participating in the reaction,
then the relaxed states of the enzyme (yellow), then the tense states of the enzyme (green), then the participation number(cyan), then the elemental balancing of the reactions (blue), and finally the pathway vectors (purple).

```
[8]:
```

```
metabolite_ids = [m.id for m in PFK.metabolites]
reaction_ids = [r.id for r in PFK.reactions]
# Define labels
pi_str = r"$\pi_{j}$"
rho_str = r"$\rho_{i}$"
chopsnq = ['C', 'H', 'O', 'P', 'N', 'S', 'q', '[PFK]']
path_labels = ["$p_1$", "$p_2$", "$p_3$", "$p_4$", "$p_5$"]
time_inv_labels = ["PFK-Total"]

ns = np.zeros((5, 30))
for i in range(5):
    ns[i][i*3:i*3+3] = 1
lns = np.zeros((1, 26))
lns[0][6:] = 1


# Make table content from the stoichiometric matrix, elemental balancing of pathways
# participation number, and MinSpan pathways
S_matrix = PFK.update_S(array_type="dense", dtype=np.int64, update_model=False)
ES_matrix = PFK.get_elemental_charge_balancing()
pi = np.count_nonzero(S_matrix, axis=0)
rho = np.count_nonzero(S_matrix, axis=1)
table_14_2 = np.vstack((S_matrix, pi, ES_matrix, ns))
# Determine number of blank entries needed to be added to pad the table,
# Add connectivity number and time invariants to table content
blanks = [""]*(len(table_14_2) - len(PFK.metabolites))
rho = np.concatenate((rho, blanks))
time_inv = np.array([np.concatenate([row, blanks]) for row in lns])
table_14_2 = np.vstack([table_14_2.T, rho, time_inv]).T

colors = {"relaxed": "#ffffe6",       # Yellow
          "tight": "#d9fad2",         # Green
          "exchanges": "#ffe6cc",     # Orange
          "chopsnq": "#99e6ff",       # Blue
          "pathways": "#b399ff",      # Purple
          "pi": "#99ffff",            # Cyan
          "rho": "#ff9999",           # Red
          "time_invs": "#ccff99",     # Lime Green
          "blank": "#f2f2f2"}         # Grey
bg_color_str = "background-color: "
def highlight_table(df, model):
    df = df.copy()
    n_mets, n_rxns = (len(model.metabolites), len(model.reactions))
    # Highlight rows
    for row in df.index:
        other_key, condition = ("blank", lambda i, v: v != "")
        if row == pi_str:        # For participation
            main_key = "pi"
        elif row in chopsnq:     # For elemental balancing
            main_key = "chopsnq"
        elif row in path_labels: # For pathways
            main_key = "pathways"
        elif "pfk" not in row:
            main_key = "exchanges"
        elif "pfk_T" in row:
            main_key, other_key = ("exchanges", "tight")
            condition = lambda i, v: (i < len(model.boundary) and i < n_rxns)
        else:
            main_key, other_key = ("exchanges", "relaxed")
            condition = lambda i, v: (i < len(model.boundary) and i < n_rxns)

        df.loc[row, :] = [bg_color_str + colors[main_key] if condition(i, v)
                          else bg_color_str + colors[other_key]
                          for i, v in enumerate(df.loc[row, :])]

    for col in df.columns:
        condition = lambda i, v: v != bg_color_str + colors["blank"]
        if col == rho_str:
            main_key = "rho"
        elif col in time_inv_labels:
            main_key = "time_invs"
        elif "PFK_T" in col or "PFK_L" in col:
            main_key = "tight"
            condition = lambda i, v: (5 < i < n_mets)
        else:
            main_key = "exchanges"
            condition = lambda i, v: (i < n_mets - 20)
        df.loc[:, col] = [bg_color_str + colors[main_key] if condition(i, v)
                          else v for i, v in enumerate(df.loc[:, col])]
    return df

# Create index and column labels
index_labels = np.concatenate((metabolite_ids, [pi_str], chopsnq, path_labels))
column_labels = np.concatenate((reaction_ids, [rho_str], time_inv_labels))
# Create DataFrame
table_14_2 = pd.DataFrame(
    table_14_2, index=index_labels, columns=column_labels)
# Apply colors
table_14_2 = table_14_2.style.apply(
    highlight_table,  model=PFK, axis=None)
table_14_2
```

```
[8]:
```

|  | SK\_f6p\_c | SK\_fdp\_c | SK\_amp\_c | SK\_adp\_c | SK\_atp\_c | SK\_h\_c | PFK\_R01 | PFK\_R02 | PFK\_R03 | PFK\_R10 | PFK\_R11 | PFK\_R12 | PFK\_R13 | PFK\_R20 | PFK\_R21 | PFK\_R22 | PFK\_R23 | PFK\_R30 | PFK\_R31 | PFK\_R32 | PFK\_R33 | PFK\_R40 | PFK\_R41 | PFK\_R42 | PFK\_R43 | PFK\_L | PFK\_T1 | PFK\_T2 | PFK\_T3 | PFK\_T4 | $\rho\_{i}$ | PFK-Total |
| --- | --- | --- | --- | --- | --- | --- | --- | --- | --- | --- | --- | --- | --- | --- | --- | --- | --- | --- | --- | --- | --- | --- | --- | --- | --- | --- | --- | --- | --- | --- | --- | --- |
| f6p\_c | -1.0 | 0.0 | 0.0 | 0.0 | 0.0 | 0.0 | 0.0 | -1.0 | 0.0 | 0.0 | 0.0 | -1.0 | 0.0 | 0.0 | 0.0 | -1.0 | 0.0 | 0.0 | 0.0 | -1.0 | 0.0 | 0.0 | 0.0 | -1.0 | 0.0 | 0.0 | 0.0 | 0.0 | 0.0 | 0.0 | 6 | 0.0 |
| fdp\_c | 0.0 | -1.0 | 0.0 | 0.0 | 0.0 | 0.0 | 0.0 | 0.0 | 1.0 | 0.0 | 0.0 | 0.0 | 1.0 | 0.0 | 0.0 | 0.0 | 1.0 | 0.0 | 0.0 | 0.0 | 1.0 | 0.0 | 0.0 | 0.0 | 1.0 | 0.0 | 0.0 | 0.0 | 0.0 | 0.0 | 6 | 0.0 |
| amp\_c | 0.0 | 0.0 | -1.0 | 0.0 | 0.0 | 0.0 | 0.0 | 0.0 | 0.0 | -1.0 | 0.0 | 0.0 | 0.0 | -1.0 | 0.0 | 0.0 | 0.0 | -1.0 | 0.0 | 0.0 | 0.0 | -1.0 | 0.0 | 0.0 | 0.0 | 0.0 | 0.0 | 0.0 | 0.0 | 0.0 | 5 | 0.0 |
| adp\_c | 0.0 | 0.0 | 0.0 | -1.0 | 0.0 | 0.0 | 0.0 | 0.0 | 1.0 | 0.0 | 0.0 | 0.0 | 1.0 | 0.0 | 0.0 | 0.0 | 1.0 | 0.0 | 0.0 | 0.0 | 1.0 | 0.0 | 0.0 | 0.0 | 1.0 | 0.0 | 0.0 | 0.0 | 0.0 | 0.0 | 6 | 0.0 |
| atp\_c | 0.0 | 0.0 | 0.0 | 0.0 | -1.0 | 0.0 | -1.0 | 0.0 | 0.0 | 0.0 | -1.0 | 0.0 | 0.0 | 0.0 | -1.0 | 0.0 | 0.0 | 0.0 | -1.0 | 0.0 | 0.0 | 0.0 | -1.0 | 0.0 | 0.0 | 0.0 | -1.0 | -1.0 | -1.0 | -1.0 | 10 | 0.0 |
| h\_c | 0.0 | 0.0 | 0.0 | 0.0 | 0.0 | -1.0 | 0.0 | 0.0 | 1.0 | 0.0 | 0.0 | 0.0 | 1.0 | 0.0 | 0.0 | 0.0 | 1.0 | 0.0 | 0.0 | 0.0 | 1.0 | 0.0 | 0.0 | 0.0 | 1.0 | 0.0 | 0.0 | 0.0 | 0.0 | 0.0 | 6 | 0.0 |
| pfk\_R0\_c | 0.0 | 0.0 | 0.0 | 0.0 | 0.0 | 0.0 | -1.0 | 0.0 | 1.0 | -1.0 | 0.0 | 0.0 | 0.0 | 0.0 | 0.0 | 0.0 | 0.0 | 0.0 | 0.0 | 0.0 | 0.0 | 0.0 | 0.0 | 0.0 | 0.0 | -1.0 | 0.0 | 0.0 | 0.0 | 0.0 | 4 | 1.0 |
| pfk\_R0\_A\_c | 0.0 | 0.0 | 0.0 | 0.0 | 0.0 | 0.0 | 1.0 | -1.0 | 0.0 | 0.0 | 0.0 | 0.0 | 0.0 | 0.0 | 0.0 | 0.0 | 0.0 | 0.0 | 0.0 | 0.0 | 0.0 | 0.0 | 0.0 | 0.0 | 0.0 | 0.0 | 0.0 | 0.0 | 0.0 | 0.0 | 2 | 1.0 |
| pfk\_R0\_AF\_c | 0.0 | 0.0 | 0.0 | 0.0 | 0.0 | 0.0 | 0.0 | 1.0 | -1.0 | 0.0 | 0.0 | 0.0 | 0.0 | 0.0 | 0.0 | 0.0 | 0.0 | 0.0 | 0.0 | 0.0 | 0.0 | 0.0 | 0.0 | 0.0 | 0.0 | 0.0 | 0.0 | 0.0 | 0.0 | 0.0 | 2 | 1.0 |
| pfk\_R1\_c | 0.0 | 0.0 | 0.0 | 0.0 | 0.0 | 0.0 | 0.0 | 0.0 | 0.0 | 1.0 | -1.0 | 0.0 | 1.0 | -1.0 | 0.0 | 0.0 | 0.0 | 0.0 | 0.0 | 0.0 | 0.0 | 0.0 | 0.0 | 0.0 | 0.0 | 0.0 | 0.0 | 0.0 | 0.0 | 0.0 | 4 | 1.0 |
| pfk\_R1\_A\_c | 0.0 | 0.0 | 0.0 | 0.0 | 0.0 | 0.0 | 0.0 | 0.0 | 0.0 | 0.0 | 1.0 | -1.0 | 0.0 | 0.0 | 0.0 | 0.0 | 0.0 | 0.0 | 0.0 | 0.0 | 0.0 | 0.0 | 0.0 | 0.0 | 0.0 | 0.0 | 0.0 | 0.0 | 0.0 | 0.0 | 2 | 1.0 |
| pfk\_R1\_AF\_c | 0.0 | 0.0 | 0.0 | 0.0 | 0.0 | 0.0 | 0.0 | 0.0 | 0.0 | 0.0 | 0.0 | 1.0 | -1.0 | 0.0 | 0.0 | 0.0 | 0.0 | 0.0 | 0.0 | 0.0 | 0.0 | 0.0 | 0.0 | 0.0 | 0.0 | 0.0 | 0.0 | 0.0 | 0.0 | 0.0 | 2 | 1.0 |
| pfk\_R2\_c | 0.0 | 0.0 | 0.0 | 0.0 | 0.0 | 0.0 | 0.0 | 0.0 | 0.0 | 0.0 | 0.0 | 0.0 | 0.0 | 1.0 | -1.0 | 0.0 | 1.0 | -1.0 | 0.0 | 0.0 | 0.0 | 0.0 | 0.0 | 0.0 | 0.0 | 0.0 | 0.0 | 0.0 | 0.0 | 0.0 | 4 | 1.0 |
| pfk\_R2\_A\_c | 0.0 | 0.0 | 0.0 | 0.0 | 0.0 | 0.0 | 0.0 | 0.0 | 0.0 | 0.0 | 0.0 | 0.0 | 0.0 | 0.0 | 1.0 | -1.0 | 0.0 | 0.0 | 0.0 | 0.0 | 0.0 | 0.0 | 0.0 | 0.0 | 0.0 | 0.0 | 0.0 | 0.0 | 0.0 | 0.0 | 2 | 1.0 |
| pfk\_R2\_AF\_c | 0.0 | 0.0 | 0.0 | 0.0 | 0.0 | 0.0 | 0.0 | 0.0 | 0.0 | 0.0 | 0.0 | 0.0 | 0.0 | 0.0 | 0.0 | 1.0 | -1.0 | 0.0 | 0.0 | 0.0 | 0.0 | 0.0 | 0.0 | 0.0 | 0.0 | 0.0 | 0.0 | 0.0 | 0.0 | 0.0 | 2 | 1.0 |
| pfk\_R3\_c | 0.0 | 0.0 | 0.0 | 0.0 | 0.0 | 0.0 | 0.0 | 0.0 | 0.0 | 0.0 | 0.0 | 0.0 | 0.0 | 0.0 | 0.0 | 0.0 | 0.0 | 1.0 | -1.0 | 0.0 | 1.0 | -1.0 | 0.0 | 0.0 | 0.0 | 0.0 | 0.0 | 0.0 | 0.0 | 0.0 | 4 | 1.0 |
| pfk\_R3\_A\_c | 0.0 | 0.0 | 0.0 | 0.0 | 0.0 | 0.0 | 0.0 | 0.0 | 0.0 | 0.0 | 0.0 | 0.0 | 0.0 | 0.0 | 0.0 | 0.0 | 0.0 | 0.0 | 1.0 | -1.0 | 0.0 | 0.0 | 0.0 | 0.0 | 0.0 | 0.0 | 0.0 | 0.0 | 0.0 | 0.0 | 2 | 1.0 |
| pfk\_R3\_AF\_c | 0.0 | 0.0 | 0.0 | 0.0 | 0.0 | 0.0 | 0.0 | 0.0 | 0.0 | 0.0 | 0.0 | 0.0 | 0.0 | 0.0 | 0.0 | 0.0 | 0.0 | 0.0 | 0.0 | 1.0 | -1.0 | 0.0 | 0.0 | 0.0 | 0.0 | 0.0 | 0.0 | 0.0 | 0.0 | 0.0 | 2 | 1.0 |
| pfk\_R4\_c | 0.0 | 0.0 | 0.0 | 0.0 | 0.0 | 0.0 | 0.0 | 0.0 | 0.0 | 0.0 | 0.0 | 0.0 | 0.0 | 0.0 | 0.0 | 0.0 | 0.0 | 0.0 | 0.0 | 0.0 | 0.0 | 1.0 | -1.0 | 0.0 | 1.0 | 0.0 | 0.0 | 0.0 | 0.0 | 0.0 | 3 | 1.0 |
| pfk\_R4\_A\_c | 0.0 | 0.0 | 0.0 | 0.0 | 0.0 | 0.0 | 0.0 | 0.0 | 0.0 | 0.0 | 0.0 | 0.0 | 0.0 | 0.0 | 0.0 | 0.0 | 0.0 | 0.0 | 0.0 | 0.0 | 0.0 | 0.0 | 1.0 | -1.0 | 0.0 | 0.0 | 0.0 | 0.0 | 0.0 | 0.0 | 2 | 1.0 |
| pfk\_R4\_AF\_c | 0.0 | 0.0 | 0.0 | 0.0 | 0.0 | 0.0 | 0.0 | 0.0 | 0.0 | 0.0 | 0.0 | 0.0 | 0.0 | 0.0 | 0.0 | 0.0 | 0.0 | 0.0 | 0.0 | 0.0 | 0.0 | 0.0 | 0.0 | 1.0 | -1.0 | 0.0 | 0.0 | 0.0 | 0.0 | 0.0 | 2 | 1.0 |
| pfk\_T0\_c | 0.0 | 0.0 | 0.0 | 0.0 | 0.0 | 0.0 | 0.0 | 0.0 | 0.0 | 0.0 | 0.0 | 0.0 | 0.0 | 0.0 | 0.0 | 0.0 | 0.0 | 0.0 | 0.0 | 0.0 | 0.0 | 0.0 | 0.0 | 0.0 | 0.0 | 1.0 | -1.0 | 0.0 | 0.0 | 0.0 | 2 | 1.0 |
| pfk\_T1\_c | 0.0 | 0.0 | 0.0 | 0.0 | 0.0 | 0.0 | 0.0 | 0.0 | 0.0 | 0.0 | 0.0 | 0.0 | 0.0 | 0.0 | 0.0 | 0.0 | 0.0 | 0.0 | 0.0 | 0.0 | 0.0 | 0.0 | 0.0 | 0.0 | 0.0 | 0.0 | 1.0 | -1.0 | 0.0 | 0.0 | 2 | 1.0 |
| pfk\_T2\_c | 0.0 | 0.0 | 0.0 | 0.0 | 0.0 | 0.0 | 0.0 | 0.0 | 0.0 | 0.0 | 0.0 | 0.0 | 0.0 | 0.0 | 0.0 | 0.0 | 0.0 | 0.0 | 0.0 | 0.0 | 0.0 | 0.0 | 0.0 | 0.0 | 0.0 | 0.0 | 0.0 | 1.0 | -1.0 | 0.0 | 2 | 1.0 |
| pfk\_T3\_c | 0.0 | 0.0 | 0.0 | 0.0 | 0.0 | 0.0 | 0.0 | 0.0 | 0.0 | 0.0 | 0.0 | 0.0 | 0.0 | 0.0 | 0.0 | 0.0 | 0.0 | 0.0 | 0.0 | 0.0 | 0.0 | 0.0 | 0.0 | 0.0 | 0.0 | 0.0 | 0.0 | 0.0 | 1.0 | -1.0 | 2 | 1.0 |
| pfk\_T4\_c | 0.0 | 0.0 | 0.0 | 0.0 | 0.0 | 0.0 | 0.0 | 0.0 | 0.0 | 0.0 | 0.0 | 0.0 | 0.0 | 0.0 | 0.0 | 0.0 | 0.0 | 0.0 | 0.0 | 0.0 | 0.0 | 0.0 | 0.0 | 0.0 | 0.0 | 0.0 | 0.0 | 0.0 | 0.0 | 1.0 | 1 | 1.0 |
| $\pi\_{j}$ | 1.0 | 1.0 | 1.0 | 1.0 | 1.0 | 1.0 | 3.0 | 3.0 | 5.0 | 3.0 | 3.0 | 3.0 | 5.0 | 3.0 | 3.0 | 3.0 | 5.0 | 3.0 | 3.0 | 3.0 | 5.0 | 3.0 | 3.0 | 3.0 | 5.0 | 2.0 | 3.0 | 3.0 | 3.0 | 3.0 |  |  |
| C | -6.0 | -6.0 | -10.0 | -10.0 | -10.0 | 0.0 | 0.0 | 0.0 | 0.0 | 0.0 | 0.0 | 0.0 | 0.0 | 0.0 | 0.0 | 0.0 | 0.0 | 0.0 | 0.0 | 0.0 | 0.0 | 0.0 | 0.0 | 0.0 | 0.0 | 0.0 | 0.0 | 0.0 | 0.0 | 0.0 |  |  |
| H | -11.0 | -10.0 | -12.0 | -12.0 | -12.0 | -1.0 | 0.0 | 0.0 | 0.0 | 0.0 | 0.0 | 0.0 | 0.0 | 0.0 | 0.0 | 0.0 | 0.0 | 0.0 | 0.0 | 0.0 | 0.0 | 0.0 | 0.0 | 0.0 | 0.0 | 0.0 | 0.0 | 0.0 | 0.0 | 0.0 |  |  |
| O | -9.0 | -12.0 | -7.0 | -10.0 | -13.0 | 0.0 | 0.0 | 0.0 | 0.0 | 0.0 | 0.0 | 0.0 | 0.0 | 0.0 | 0.0 | 0.0 | 0.0 | 0.0 | 0.0 | 0.0 | 0.0 | 0.0 | 0.0 | 0.0 | 0.0 | 0.0 | 0.0 | 0.0 | 0.0 | 0.0 |  |  |
| P | -1.0 | -2.0 | -1.0 | -2.0 | -3.0 | 0.0 | 0.0 | 0.0 | 0.0 | 0.0 | 0.0 | 0.0 | 0.0 | 0.0 | 0.0 | 0.0 | 0.0 | 0.0 | 0.0 | 0.0 | 0.0 | 0.0 | 0.0 | 0.0 | 0.0 | 0.0 | 0.0 | 0.0 | 0.0 | 0.0 |  |  |
| N | 0.0 | 0.0 | -5.0 | -5.0 | -5.0 | 0.0 | 0.0 | 0.0 | 0.0 | 0.0 | 0.0 | 0.0 | 0.0 | 0.0 | 0.0 | 0.0 | 0.0 | 0.0 | 0.0 | 0.0 | 0.0 | 0.0 | 0.0 | 0.0 | 0.0 | 0.0 | 0.0 | 0.0 | 0.0 | 0.0 |  |  |
| S | 0.0 | 0.0 | 0.0 | 0.0 | 0.0 | 0.0 | 0.0 | 0.0 | 0.0 | 0.0 | 0.0 | 0.0 | 0.0 | 0.0 | 0.0 | 0.0 | 0.0 | 0.0 | 0.0 | 0.0 | 0.0 | 0.0 | 0.0 | 0.0 | 0.0 | 0.0 | 0.0 | 0.0 | 0.0 | 0.0 |  |  |
| q | 2.0 | 4.0 | 2.0 | 3.0 | 4.0 | -1.0 | 0.0 | 0.0 | 0.0 | 0.0 | 0.0 | 0.0 | 0.0 | 0.0 | 0.0 | 0.0 | 0.0 | 0.0 | 0.0 | 0.0 | 0.0 | 0.0 | 0.0 | 0.0 | 0.0 | 0.0 | 0.0 | 0.0 | 0.0 | 0.0 |  |  |
| [PFK] | 0.0 | 0.0 | 0.0 | 0.0 | 0.0 | 0.0 | 0.0 | 0.0 | 0.0 | 0.0 | 0.0 | 0.0 | 0.0 | 0.0 | 0.0 | 0.0 | 0.0 | 0.0 | 0.0 | 0.0 | 0.0 | 0.0 | 0.0 | 0.0 | 0.0 | 0.0 | 0.0 | 0.0 | 0.0 | 0.0 |  |  |
| $p\_1$ | 1.0 | 1.0 | 1.0 | 0.0 | 0.0 | 0.0 | 0.0 | 0.0 | 0.0 | 0.0 | 0.0 | 0.0 | 0.0 | 0.0 | 0.0 | 0.0 | 0.0 | 0.0 | 0.0 | 0.0 | 0.0 | 0.0 | 0.0 | 0.0 | 0.0 | 0.0 | 0.0 | 0.0 | 0.0 | 0.0 |  |  |
| $p\_2$ | 0.0 | 0.0 | 0.0 | 1.0 | 1.0 | 1.0 | 0.0 | 0.0 | 0.0 | 0.0 | 0.0 | 0.0 | 0.0 | 0.0 | 0.0 | 0.0 | 0.0 | 0.0 | 0.0 | 0.0 | 0.0 | 0.0 | 0.0 | 0.0 | 0.0 | 0.0 | 0.0 | 0.0 | 0.0 | 0.0 |  |  |
| $p\_3$ | 0.0 | 0.0 | 0.0 | 0.0 | 0.0 | 0.0 | 1.0 | 1.0 | 1.0 | 0.0 | 0.0 | 0.0 | 0.0 | 0.0 | 0.0 | 0.0 | 0.0 | 0.0 | 0.0 | 0.0 | 0.0 | 0.0 | 0.0 | 0.0 | 0.0 | 0.0 | 0.0 | 0.0 | 0.0 | 0.0 |  |  |
| $p\_4$ | 0.0 | 0.0 | 0.0 | 0.0 | 0.0 | 0.0 | 0.0 | 0.0 | 0.0 | 1.0 | 1.0 | 1.0 | 0.0 | 0.0 | 0.0 | 0.0 | 0.0 | 0.0 | 0.0 | 0.0 | 0.0 | 0.0 | 0.0 | 0.0 | 0.0 | 0.0 | 0.0 | 0.0 | 0.0 | 0.0 |  |  |
| $p\_5$ | 0.0 | 0.0 | 0.0 | 0.0 | 0.0 | 0.0 | 0.0 | 0.0 | 0.0 | 0.0 | 0.0 | 0.0 | 1.0 | 1.0 | 1.0 | 0.0 | 0.0 | 0.0 | 0.0 | 0.0 | 0.0 | 0.0 | 0.0 | 0.0 | 0.0 | 0.0 | 0.0 | 0.0 | 0.0 | 0.0 |  |  |

###### The null spaces of the stoichiometric matrix¶

The null space is 5 (=30-25) dimensional. The five pathway vectors spanning the null space are simply the input of the reagents, the PFK reaction itself and the output of the products, see Table 14.2. Each pathway is catalyzed by one of the five active R forms. Thus these form 5 parallel pathways through the subnetwork where the flux through each is determined by the relative amount of the R forms of the enzyme.

The left null space is one dimensional (=26-25). It is spanned by a vector that represents the conservation of the PFK enzyme itself, see Table 14.2

###### Pools and ratios¶

The enzyme is in two key states, the relaxed (R) and the tight (T) state. The binding of the activator, AMP, pulls the enzyme towards the catalytically active relaxed state while binding of the the inhibitor, ATP, pulls it towards the catalytically inactive tight state, creating a tug of war over the catalytic capacity of the enzyme. The fraction of the total enzyme that is in the R form can be computed:

\[\begin{equation} r\_\mathrm{R} = \frac{\Sigma\_{i=0}^{4}(R\_i + R\_{i, A} + R\_{i, AF})}{\text{PFK}\_{\mathrm{tot}}} \tag{14.15} \end{equation}\]

The relaxed states are then pooled into the form that is loaded with substrates that carry out the reaction. We thus define the fraction of the enzyme R states that is loaded with the substrates and is producing the product as;

\[\begin{equation} r\_\mathrm{cat} = \frac{\Sigma\_{i=0}^{4}R\_{i, AF}}{\Sigma\_{i=0}^{4}(R\_i + R\_{i, A} + R\_{i, AF})} \tag{14.16} \end{equation}\]

This fraction of the enzyme generates the products.

##### The Steady State¶

###### The reaction rate¶

The steady state flux through the PFK subnetwork is given by

\[\begin{split}\begin{align} v\_{PFK} &= k\_{PFK} \Sigma\_{i=0}^{4}R\_{i, AF} \tag{14.17} \\ &= k\_{PFK}\text{PFK}\_{\mathrm{tot}}r\_\mathrm{R}r\_\mathrm{cat} \tag{14.18} \\ \end{align}\end{split}\]

In this equation we know the steady state flux and the total amount of enzyme. We will use the glycolytic flux in Chapter 10 of 1.12 mM/hr. The concentration of PFK in the RBC is about 0.000033 mM (Albe 1990).

###### Integration of the PFK subnetwork into the metabolic network¶

We can compute \(r\_\mathrm{R}\) and \(r\_\mathrm{cat}\) from equilibrium binding of the regulatory ligands to PFK. We know the steady state concentrations of these ligands in the network; ATP is about 1.6 mM and AMP is about 0.087 mM. We also know the steady state concentration of the reactants ATP and F6P, the latter is about 0.02 mM. The algebra associated with these computations is quite intricate, albeit simple in principle. Its complexity was foreshadowed in Chapter 5.

Once we have computed the PERC, \(k\_{PFK}\), we can integrate the PFK subnetwork into the glycolytic model. Such integration procedures can be performed for any enzyme of interest in a given network.

###### Solving for the steady state¶

The steady state equations are

\[\begin{equation} \textbf{Sv(x)} = 0 \tag{14.19} \end{equation}\]

The elementary forms of the rate laws

\[\begin{split}\begin{align} &v\_{R\_{0, 1}} : k^+\_AR\_{0} &&- k^-\_AR\_{0, A} \\ &v\_{R\_{0, 2}} : k^+\_FR\_{0,A} &&- k^-\_FR\_{0, AF} \\ &v\_{R\_{0, 3}} : k\_{PFK}R\_{0, AF} && \\ &v\_{R\_{1, 0}} : 4k^+\_{a}R\_{0} &&- k^-\_{a}R\_{1} \\ &v\_{R\_{1, 1}} : k^+\_AR\_{1} &&- k^-\_AR\_{1, A} \\ &v\_{R\_{1, 2}} : k^+\_FR\_{1,A} &&- k^-\_FR\_{1, AF} \\ &v\_{R\_{1, 3}} : k\_{PFK}R\_{1, AF} && \\ &v\_{R\_{2, 0}} : 3k^+\_{a}R\_{1} &&- 2k^-\_{a}R\_{2} \\ &v\_{R\_{2, 1}} : k^+\_AR\_{2} &&- k^-\_AR\_{2, A} \\ &v\_{R\_{2, 2}} : k^+\_FR\_{2,A} &&- k^-\_FR\_{2, AF} \\ &v\_{R\_{2, 3}} : k\_{PFK}R\_{2, AF} && \\ &v\_{R\_{3, 0}} : 2k^+\_{a}R\_{2} &&- 3k^-\_{a}R\_{3} \\ &v\_{R\_{3, 1}} : k^+\_AR\_{3} &&- k^-\_AR\_{3, A} \\ &v\_{R\_{3, 2}} : k^+\_FR\_{3,A} &&- k^-\_FR\_{3, AF} \\ &v\_{R\_{3, 3}} : k\_{PFK}R\_{3, AF} && \\ &v\_{R\_{4, 0}} : k^+\_{a}R\_{3} &&- 4k^-\_{a}R\_{4} \\ &v\_{R\_{4, 1}} : k^+\_AR\_{4} &&- k^-\_AR\_{4, A} \\ &v\_{R\_{4, 2}} : k^+\_FR\_{4,A} &&- k^-\_FR\_{4, AF} \\ &v\_{R\_{4, 3}} : k\_{PFK}R\_{4, AF} && \\ &v\_{L} : k^+R\_{0} &&- k^-T\_{0} \\ &v\_{T\_{1}} : 4k^+\_{i}T\_{0} &&- k^-\_{i}T\_{1} \\ &v\_{T\_{2}} : 3k^+\_{i}T\_{1} &&- 2k^-\_{i}T\_{2} \\ &v\_{T\_{3}} : 2k^+\_{i}T\_{2} &&- 3k^-\_{i}T\_{3} \\ &v\_{T\_{4}} : k^+\_{i}T\_{3} &&- 4k^-\_{i}T\_{4} \\ \end{align}\end{split}\]

\[\tag{14.20}\]

can be introduced into this equation to form 19 algebraic equations in 20 concentration variables as unknowns, where

\[\begin{equation} L = \frac{k^+}{k^-},\ K\_i = \frac{k^+\_i}{k^-\_i},\ K\_a = \frac{k^+\_a}{k^-\_a},\ K\_F = \frac{k^+\_F}{k^-\_F},\ K\_A = \frac{k^+\_A}{k^-\_A} \tag{14.21} \end{equation}\]

and where the forward rate constants \({k^+\_F}\), \({k^+\_A}\), \({k^+\_a}\), and \({k^+\_i}\) contain the steady state concentrations of the corresponding ligand. This makes all the rate laws linear.

The total mass balance on all the forms of the enzyme

\[\begin{equation} \text{PFK}\_{\mathrm{tot}} = \underset{i=0}{\stackrel{4}{\sum}}(R\_i + R\_{i, A} + R\_{i, AF}) + \underset{i=0}{\stackrel{4}{\sum}} T\_{i} \tag{14.22} \end{equation}\]

will lead to 20 algebraic equations with 20 concentration variables.

In addition, the reaction rate \(v\_{PFK}\) is the input flux for the reactants and the output flux for the product in the steady state. The reaction rate is:

\[\begin{equation} v\_{PFK} = k\_{PFK} \underset{i=0}{\stackrel{4}{\sum}}R\_{i, AF} \tag{14.23} \end{equation}\]

where \(k\_{PFK}\) is unknown. Since the binding step of the activator and the inhibitor is at equilibrium at steady state only the corresponding equilibrium constants will appear in the steady state equation. However, since the binding of the reactants are in a steady state we will need numerical values for \(k^-\_F\) and \(k^-\_A\).

In principle, we can thus specify \(k^-\_F\) and \(k^-\_A\) and solve 20 equations for the 20 concentration variables and \(k\_{PFK}\), given numerical values for the total amount of enzyme and the 5 binding constants in Eq. (14.21). In practice, the choices for \(k^-\_F\) and \(k^-\_A\) are restricted for the computed concentrations and \(k\_{PFK}\) to take on positive values.

###### The steady state solution¶

The solution to the 19 algebraic equations with the reaction rate law (Eq. (14.23)) is given in Table 14.3. The total PFK can then be computed from Eq. (14.22).

First, we will remove the ligands from the equations by substituting them for a value of 1 to account for the forward rate consants including the steady state concentrations of their corresponding ligands. Next, we will identify the enzyme forms and store them in a list. Finally, we define a dictionary of the ordinary differential equations as sympy expressions with the ligand concentrations lumped into the rate constants.

```
[9]:
```

```
n_sites = 4
n_binding_steps = 3

enzyme_forms = [sym.Symbol(met.id) for met in PFK.metabolites
                if "pfk" in met.id]
conc_subs = {sym.Symbol(met.id): 1 for met in PFK.metabolites
             if "pfk" not in met.id}

ode_dict = {sym.Symbol(met.id): sym.Eq(ode.subs(conc_subs), 0)
            for met, ode in Keq2k(strip_time(PFK.odes)).items()
            if "pfk" in met.id}
```

We then identify equations for the unknown concentrations we wish to solve for in each reaction. We will treat the completely free form of the enzyme with no activators or inhibitors as our dependent variable.

```
[10]:
```

```
enzyme_forms.reverse()

pfk_solutions = {}
for pfk_form in enzyme_forms:
    if "pfk_R0_c" == str(pfk_form):
        continue
    sol = sym.solveset(ode_dict[pfk_form].subs(pfk_solutions), pfk_form)
    pfk_solutions[pfk_form] = list(sol).pop()
    pfk_solutions.update({pfk_form: sol.subs(pfk_solutions)
                          for pfk_form, sol in pfk_solutions.items()})
```

We also determine the catalyzation reactions and isolate them in order to create our equation for \(v\_{PFK}\).

```
[11]:
```

```
catalyzation_reactions = ["PFK_R{0:d}{1:d}".format(i, n_binding_steps)
                          for i in range(n_sites + 1)]
v_PFK_sym = sym.Symbol("v_PFK")
v_PFK_equation = v_PFK_sym - sym.simplify(sum(strip_time([
    PFK.reactions.get_by_id(rxn).rate for rxn in catalyzation_reactions])))
sym.pprint(v_PFK_equation)
```

```
-kf_PFK⋅(pfk_R0_AF_c + pfk_R1_AF_c + pfk_R2_AF_c + pfk_R3_AF_c + pfk_R4_AF_c)
+ v_PFK
```

We utilize the reaction rate equation and solve for the our final unknown concentration variable in terms of the rate constants. Once it has been solved for, we substitute the solution back into our other equations.

```
[12]:
```

```
sol = sym.solveset(v_PFK_equation.subs(pfk_solutions), "pfk_R0_c")
pfk_solutions[sym.Symbol("pfk_R0_c")] = list(sol).pop()
pfk_solutions = {met: sym.simplify(solution.subs(pfk_solutions))
                 for met, solution in pfk_solutions.items()}
```

The first column in Table 14.3 shows the solution for \(R\_{i, 0}\). Summing up these columns gives the total amount of the \(R\_{i, 0}\) forms The next three columns show the relative distribution of the \(R\_{i, 0}\), \(R\_{i, A}\), and \(R\_{i, AF}\) forms. These relative amounts are the same for all the activator bound states of the enzyme, i.e., the same for all \(i\).

Thus the relative amount of the enzyme in the different activator bound states is given by the relative amount of the \(R\_{i, 0}\) forms, shown in the fourth column of the table.

The solution for \(T\_{0}\) is:

\[\begin{equation} T\_0 = \frac{(k\_i^-)^4Lv\_{PFK}(k\_A^-(k\_F^- + k\_{PFK}) + k\_F^+k\_{PFK})}{k\_A^+k\_F^+k\_{PFK}(k\_i^- + k\_i^+)^4} \tag{14.24} \end{equation}\]

and then the relative amount of the \(T\_{i}\) forms is given in the last column of Table 14.3.

**Table 14.3:** The steady-state solution for the enzyme concentrations in the PFK sub-network.

```
[13]:
```

```
table_14_3 = []

R10, T00 = (pfk_solutions[sym.Symbol(k)] for k in ["pfk_R0_AF_c", "pfk_T0_c"])
for i in range(5):
    keys = ("pfk_R{0:d}_c|pfk_R{0:d}_A_c|pfk_R{0:d}_AF_c|pfk_T{0:d}_c"
            .format(i).split("|"))
    Ri0, RiA, RiAF, Ti0 = (pfk_solutions[sym.Symbol(k)] for k in keys)
    num_denom_zip = zip([Ri0, Ri0, RiA, RiAF, RiAF, Ti0], [1, Ri0, Ri0, Ri0, R10, T00])
    ratios = [sym.collect(numerator/denominator, "kf_PFK") for numerator, denominator in num_denom_zip]
    table_14_3.append(["${0}$".format(sym.latex(ratio)) for ratio in ratios])

column_labels = ["$R_{i,0}$", "$R_{i,0} / R_{i,0}$", "$R_{i,A} / R_{i,0}$",
                 "$R_{i,AF} / R_{i,0}$", "$R_{i,0} / R_{0,0}$", "$T_{i} / T_{0}$"]
table_14_3 = pd.DataFrame(
    np.array(table_14_3), columns=column_labels)

table_14_3.index.rename("i", inplace=True);
table_14_3
```

```
[13]:
```

|  | $R\_{i,0}$ | $R\_{i,0} / R\_{i,0}$ | $R\_{i,A} / R\_{i,0}$ | $R\_{i,AF} / R\_{i,0}$ | $R\_{i,0} / R\_{0,0}$ | $T\_{i} / T\_{0}$ |
| --- | --- | --- | --- | --- | --- | --- |
| i |  |  |  |  |  |  |
| 0 | $\frac{kr\_{PFK ACT}^{4} v\_{PFK} \left(kf\_{PFK} \left(kf\_{PFK F} + kr\_{PFK A}\right) + kr\_{PFK A} kr\_{PFK F}\right)}{kf\_{PFK} kf\_{PFK A} kf\_{PFK F} \left(kf\_{PFK ACT} + kr\_{PFK ACT}\right)^{4}}$ | $1$ | $\frac{kf\_{PFK A} \left(kf\_{PFK} + kr\_{PFK F}\right)}{kf\_{PFK} \left(kf\_{PFK F} + kr\_{PFK A}\right) + kr\_{PFK A} kr\_{PFK F}}$ | $\frac{kf\_{PFK A} kf\_{PFK F}}{kf\_{PFK} \left(kf\_{PFK F} + kr\_{PFK A}\right) + kr\_{PFK A} kr\_{PFK F}}$ | $1$ | $1$ |
| 1 | $\frac{4 kf\_{PFK ACT} kr\_{PFK ACT}^{3} v\_{PFK} \left(kf\_{PFK} \left(kf\_{PFK F} + kr\_{PFK A}\right) + kr\_{PFK A} kr\_{PFK F}\right)}{kf\_{PFK} kf\_{PFK A} kf\_{PFK F} \left(kf\_{PFK ACT} + kr\_{PFK ACT}\right)^{4}}$ | $1$ | $\frac{kf\_{PFK A} \left(kf\_{PFK} + kr\_{PFK F}\right)}{kf\_{PFK} \left(kf\_{PFK F} + kr\_{PFK A}\right) + kr\_{PFK A} kr\_{PFK F}}$ | $\frac{kf\_{PFK A} kf\_{PFK F}}{kf\_{PFK} \left(kf\_{PFK F} + kr\_{PFK A}\right) + kr\_{PFK A} kr\_{PFK F}}$ | $\frac{4 kf\_{PFK ACT}}{kr\_{PFK ACT}}$ | $\frac{4 kf\_{PFK I}}{kr\_{PFK I}}$ |
| 2 | $\frac{6 kf\_{PFK ACT}^{2} kr\_{PFK ACT}^{2} v\_{PFK} \left(kf\_{PFK} \left(kf\_{PFK F} + kr\_{PFK A}\right) + kr\_{PFK A} kr\_{PFK F}\right)}{kf\_{PFK} kf\_{PFK A} kf\_{PFK F} \left(kf\_{PFK ACT} + kr\_{PFK ACT}\right)^{4}}$ | $1$ | $\frac{kf\_{PFK A} \left(kf\_{PFK} + kr\_{PFK F}\right)}{kf\_{PFK} \left(kf\_{PFK F} + kr\_{PFK A}\right) + kr\_{PFK A} kr\_{PFK F}}$ | $\frac{kf\_{PFK A} kf\_{PFK F}}{kf\_{PFK} \left(kf\_{PFK F} + kr\_{PFK A}\right) + kr\_{PFK A} kr\_{PFK F}}$ | $\frac{6 kf\_{PFK ACT}^{2}}{kr\_{PFK ACT}^{2}}$ | $\frac{6 kf\_{PFK I}^{2}}{kr\_{PFK I}^{2}}$ |
| 3 | $\frac{4 kf\_{PFK ACT}^{3} kr\_{PFK ACT} v\_{PFK} \left(kf\_{PFK} \left(kf\_{PFK F} + kr\_{PFK A}\right) + kr\_{PFK A} kr\_{PFK F}\right)}{kf\_{PFK} kf\_{PFK A} kf\_{PFK F} \left(kf\_{PFK ACT} + kr\_{PFK ACT}\right)^{4}}$ | $1$ | $\frac{kf\_{PFK A} \left(kf\_{PFK} + kr\_{PFK F}\right)}{kf\_{PFK} \left(kf\_{PFK F} + kr\_{PFK A}\right) + kr\_{PFK A} kr\_{PFK F}}$ | $\frac{kf\_{PFK A} kf\_{PFK F}}{kf\_{PFK} \left(kf\_{PFK F} + kr\_{PFK A}\right) + kr\_{PFK A} kr\_{PFK F}}$ | $\frac{4 kf\_{PFK ACT}^{3}}{kr\_{PFK ACT}^{3}}$ | $\frac{4 kf\_{PFK I}^{3}}{kr\_{PFK I}^{3}}$ |
| 4 | $\frac{kf\_{PFK ACT}^{4} v\_{PFK} \left(kf\_{PFK} \left(kf\_{PFK F} + kr\_{PFK A}\right) + kr\_{PFK A} kr\_{PFK F}\right)}{kf\_{PFK} kf\_{PFK A} kf\_{PFK F} \left(kf\_{PFK ACT} + kr\_{PFK ACT}\right)^{4}}$ | $1$ | $\frac{kf\_{PFK A} \left(kf\_{PFK} + kr\_{PFK F}\right)}{kf\_{PFK} \left(kf\_{PFK F} + kr\_{PFK A}\right) + kr\_{PFK A} kr\_{PFK F}}$ | $\frac{kf\_{PFK A} kf\_{PFK F}}{kf\_{PFK} \left(kf\_{PFK F} + kr\_{PFK A}\right) + kr\_{PFK A} kr\_{PFK F}}$ | $\frac{kf\_{PFK ACT}^{4}}{kr\_{PFK ACT}^{4}}$ | $\frac{kf\_{PFK I}^{4}}{kr\_{PFK I}^{4}}$ |

###### Numerical values¶

We now introduce the numerical values, \(K\_i=0.1/1.6\), \(K\_a=0.033/0.0867\), \(K\_A=0.068/1.6\), \(K\_F=0.1/0.0198\), \(v\_{PFK}=1.12 \text{mM/hr}\), and \(K\_L=1/0.0011\) using the dissociation constant values given earlier in the chapter and the steady state concentrations of the ligands. We can introduce these into the solution and sum over all the forms of the enzyme to get

```
[14]:
```

```
# Extract steady state values from glycolysis
glycolysis = create_test_model("SB2_Glycolysis")
abbrev_dict = {"PFK_A": "atp_c", "PFK_F": "f6p_c", "PFK_ACT": "amp_c", "PFK_I": "atp_c", "PFK_L": ""}

k2K = {sym.Symbol("kr_" + p): sym.Symbol("kf_" + p)*sym.Symbol("K_" + p) for p in abbrev_dict.keys()}
pfk_solutions = {met: sym.simplify(solution.subs(pfk_solutions).subs(k2K))
                 for met, solution in pfk_solutions.items()}
K_values = dict(zip(["K_" + p for p in abbrev_dict], [0.068, 0.1, 0.033, 0.1, 0.0011]))


numerical_values = {}
for abbrev, ligand_id in abbrev_dict.items():
    K_str = "K_" + abbrev
    if ligand_id:
        ligand = glycolysis.metabolites.get_by_id(ligand_id)
        numerical_value = K_values[K_str]/glycolysis.initial_conditions[ligand]
    else:
        numerical_value = 1/K_values[K_str]
    numerical_values[sym.Symbol(K_str)] = numerical_value

pfk_total_sym = sym.Symbol("PFK-Total")
numerical_values.update({
    v_PFK_sym: glycolysis.reactions.PFK.steady_state_flux,
    pfk_total_sym: 0.033e-3})

pfk_total_equation = sym.Eq(pfk_total_sym, sum(enzyme_forms))
pfk_total_equation = sym.simplify(pfk_total_equation.subs(pfk_solutions).subs(numerical_values))
sym.pprint(sym.N(pfk_total_equation, 3))
```

```
  1.19       1.71      7.14
──────── + ──────── + ────── = 3.3e-5
kf_PFK_F   kf_PFK_A   kf_PFK
```

\[\begin{equation} \text{PFK}\_{\mathrm{tot}} = 1.71/k\_A^+ + 1.19/k\_F^+ + 7.14/k\_{PFK}^= 0.000033 \text{mM} \tag{14.25} \end{equation}\]

The three parameter values are thus not independent as stated above. This equation can be plotted in three dimensions, Figure 14.3. For the simulations below we choose \(k\_F^+ = 1\*10^6 \text{1/h/mM}\) and \(k\_A^+ = 2\*10^5 \text{1/h/mM}\) and compute \(k\_{PFK}=3.07\*10^5 \text{1/h}\) from equation 14.25. With these values about 90% of the enzyme is in the R form \(r\_\mathrm{R}=0.896)\), and about 12% of R is in the \(R\_{i, AF}\) forms \((r\_\mathrm{cat}=0.123)\). With these
values the relative flux load through the five forms of \(R\_{i, AF}\) is:

\[\begin{split}\begin{align} &i, \ &\text{fraction} \\ &0, \ &0.00577 \\ &1, \ &0.0607 \\ &2, \ &0.239 \\ &3, \ &0.419 \\ &4, \ &0.275 \\ \end{align}\end{split}\]

\[\tag{14.26}\]

Thus most of the flux is carried by \(R\_{3, AF}\) for these parameter values and steady state concentrations of ligands.

```
[15]:
```

```
kf_PFK_sym = sym.Symbol("kf_PFK")
kf_PFK_sol = sym.solve([3.3e-05 - pfk_total_equation.lhs], kf_PFK_sym)[kf_PFK_sym]

args = tuple(sym.Symbol(param) for param in sorted(
    str(symbol) for symbol in kf_PFK_sol.atoms(sym.Symbol)))
kf_PFK_sol = sym.lambdify(args, sym.log(kf_PFK_sol, 10), modules="numpy")

fig_14_3 = plt.figure(figsize=(6, 6))
ax = fig_14_3.add_subplot(111, projection="3d")

ka  = np.geomspace(1e5, 1e7, 50)
kf  = np.geomspace(1e5, 1e7, 50)
ka, kf = np.meshgrid(ka, kf)
# Plot the surface.
surf = ax.plot_surface(ka, kf, kf_PFK_sol(ka, kf),
                       linewidth=0, antialiased=True, vmin=5.35, vmax=5.5,
                       cmap=plt.cm.coolwarm, zorder=0)
ax.view_init(30, 70);
ax.set_xlim(1e4, 1e7);
ax.set_ylim(1e4, 1e7);
ax.set_zlim(5.35, 5.5);
ax.zaxis.set_major_locator(mpl.ticker.LinearLocator());
ax.zaxis.set_major_formatter(mpl.ticker.FormatStrFormatter('%.02f'));
ax.plot([2e5], [1e6], [kf_PFK_sol(2e5, 1e6)],
        markerfacecolor='k', markeredgecolor='k',
        marker='o', markersize=5, zorder=10);
fig_14_3.tight_layout()
```

**Figure 14.3:** (a) 3D plot and (b) density plot of the relationships between the three key PERCs \((k\_{F}^+,\ k\_{A}^+,\ k\_{PFK})\) of PFK given in Eq (14.25), with z-axis in logarithmic scale. The black dot shows the particular combination chosen.

##### Integration of PFK with Glycolysis¶

The PFK subnetwork can now be integrated with the glycolysis model of chapter 10. The integration process is straight forward. Though simulation we can compare the networks with and without the PFK subnetwork. The integration is performed as:

```
[16]:
```

```
# Load glycolysis model
glycolysis = create_test_model("SB2_Glycolysis")

# Remove phosphate constraint in glycolysis as in Section 10.9
glycolysis.add_boundary(
    metabolite=glycolysis.metabolites.get_by_id("pi_c"),
    boundary_type="sink",  boundary_condition=2.5)
# Set forward rate constant
glycolysis.reactions.SK_pi_c.kf = 0.23
glycolysis.reactions.SK_pi_c.Keq = 1

# Load PFK module
PFK = create_test_model("SB2_PFK")

# Add PFK module to system
glycolysis_PFK = glycolysis.merge(PFK, inplace=False)
# Delete old PFK reaction
glycolysis_PFK.remove_reactions([glycolysis_PFK.reactions.PFK])
```

This results in an integrated network whose stoichiometric matrix is of dimensions 40 x 45 and of rank 38

```
[17]:
```

```
# Define new order for metabolites
new_metabolite_order = [
    "glc__D_c", "g6p_c", "f6p_c",  "pfk_R0_c", "pfk_R0_A_c", "pfk_R0_AF_c",
    "pfk_R1_c", "pfk_R1_A_c", "pfk_R1_AF_c", "pfk_R2_c", "pfk_R2_A_c", "pfk_R2_AF_c",
    "pfk_R3_c", "pfk_R3_A_c", "pfk_R3_AF_c","pfk_R4_c", "pfk_R4_A_c", "pfk_R4_AF_c",
    "pfk_T0_c","pfk_T1_c", "pfk_T2_c", "pfk_T3_c", "pfk_T4_c", "fdp_c", "dhap_c","g3p_c",
    "_13dpg_c", "_3pg_c", "_2pg_c", "pep_c", "pyr_c", "lac__L_c", "nad_c", "nadh_c",
    "amp_c", "adp_c", "atp_c","pi_c", "h_c", "h2o_c"]

if len(glycolysis_PFK.metabolites) == len(new_metabolite_order):
    glycolysis_PFK.metabolites = DictList(glycolysis_PFK.metabolites.get_by_any(new_metabolite_order))
# Define new order for reactions
new_reaction_order = [
    "HEX1", "PGI", "PFK_R01", "PFK_R02", "PFK_R03", "PFK_R10", "PFK_R11", "PFK_R12", "PFK_R13",
    "PFK_R20", "PFK_R21", "PFK_R22", "PFK_R23", "PFK_R30", "PFK_R31", "PFK_R32", "PFK_R33",
    "PFK_R40", "PFK_R41", "PFK_R42", "PFK_R43", "PFK_L", "PFK_T1", "PFK_T2", "PFK_T3", "PFK_T4",
    "TPI","FBA", "GAPD", "PGK", "PGM", "ENO", "PYK", "LDH_L", "DM_amp_c", "ADK1", "SK_pyr_c",
    "SK_lac__L_c", "ATPM", "DM_nadh",  "SK_glc__D_c", "SK_amp_c", "SK_h_c", "SK_h2o_c", "SK_pi_c"]

if len(glycolysis_PFK.reactions) == len(new_reaction_order):
    glycolysis_PFK.reactions = DictList(glycolysis_PFK.reactions.get_by_any(new_reaction_order))
glycolysis_PFK.update_S(array_type="DataFrame", dtype=np.int64);
```

```
[17]:
```

|  | HEX1 | PGI | PFK\_R01 | PFK\_R02 | PFK\_R03 | PFK\_R10 | PFK\_R11 | PFK\_R12 | PFK\_R13 | PFK\_R20 | PFK\_R21 | PFK\_R22 | PFK\_R23 | PFK\_R30 | PFK\_R31 | PFK\_R32 | PFK\_R33 | PFK\_R40 | PFK\_R41 | PFK\_R42 | PFK\_R43 | PFK\_L | PFK\_T1 | PFK\_T2 | PFK\_T3 | PFK\_T4 | TPI | FBA | GAPD | PGK | PGM | ENO | PYK | LDH\_L | DM\_amp\_c | ADK1 | SK\_pyr\_c | SK\_lac\_\_L\_c | ATPM | DM\_nadh | SK\_glc\_\_D\_c | SK\_amp\_c | SK\_h\_c | SK\_h2o\_c | SK\_pi\_c |
| --- | --- | --- | --- | --- | --- | --- | --- | --- | --- | --- | --- | --- | --- | --- | --- | --- | --- | --- | --- | --- | --- | --- | --- | --- | --- | --- | --- | --- | --- | --- | --- | --- | --- | --- | --- | --- | --- | --- | --- | --- | --- | --- | --- | --- | --- |
| glc\_\_D\_c | -1 | 0 | 0 | 0 | 0 | 0 | 0 | 0 | 0 | 0 | 0 | 0 | 0 | 0 | 0 | 0 | 0 | 0 | 0 | 0 | 0 | 0 | 0 | 0 | 0 | 0 | 0 | 0 | 0 | 0 | 0 | 0 | 0 | 0 | 0 | 0 | 0 | 0 | 0 | 0 | 1 | 0 | 0 | 0 | 0 |
| g6p\_c | 1 | -1 | 0 | 0 | 0 | 0 | 0 | 0 | 0 | 0 | 0 | 0 | 0 | 0 | 0 | 0 | 0 | 0 | 0 | 0 | 0 | 0 | 0 | 0 | 0 | 0 | 0 | 0 | 0 | 0 | 0 | 0 | 0 | 0 | 0 | 0 | 0 | 0 | 0 | 0 | 0 | 0 | 0 | 0 | 0 |
| f6p\_c | 0 | 1 | 0 | -1 | 0 | 0 | 0 | -1 | 0 | 0 | 0 | -1 | 0 | 0 | 0 | -1 | 0 | 0 | 0 | -1 | 0 | 0 | 0 | 0 | 0 | 0 | 0 | 0 | 0 | 0 | 0 | 0 | 0 | 0 | 0 | 0 | 0 | 0 | 0 | 0 | 0 | 0 | 0 | 0 | 0 |
| pfk\_R0\_c | 0 | 0 | -1 | 0 | 1 | -1 | 0 | 0 | 0 | 0 | 0 | 0 | 0 | 0 | 0 | 0 | 0 | 0 | 0 | 0 | 0 | -1 | 0 | 0 | 0 | 0 | 0 | 0 | 0 | 0 | 0 | 0 | 0 | 0 | 0 | 0 | 0 | 0 | 0 | 0 | 0 | 0 | 0 | 0 | 0 |
| pfk\_R0\_A\_c | 0 | 0 | 1 | -1 | 0 | 0 | 0 | 0 | 0 | 0 | 0 | 0 | 0 | 0 | 0 | 0 | 0 | 0 | 0 | 0 | 0 | 0 | 0 | 0 | 0 | 0 | 0 | 0 | 0 | 0 | 0 | 0 | 0 | 0 | 0 | 0 | 0 | 0 | 0 | 0 | 0 | 0 | 0 | 0 | 0 |
| pfk\_R0\_AF\_c | 0 | 0 | 0 | 1 | -1 | 0 | 0 | 0 | 0 | 0 | 0 | 0 | 0 | 0 | 0 | 0 | 0 | 0 | 0 | 0 | 0 | 0 | 0 | 0 | 0 | 0 | 0 | 0 | 0 | 0 | 0 | 0 | 0 | 0 | 0 | 0 | 0 | 0 | 0 | 0 | 0 | 0 | 0 | 0 | 0 |
| pfk\_R1\_c | 0 | 0 | 0 | 0 | 0 | 1 | -1 | 0 | 1 | -1 | 0 | 0 | 0 | 0 | 0 | 0 | 0 | 0 | 0 | 0 | 0 | 0 | 0 | 0 | 0 | 0 | 0 | 0 | 0 | 0 | 0 | 0 | 0 | 0 | 0 | 0 | 0 | 0 | 0 | 0 | 0 | 0 | 0 | 0 | 0 |
| pfk\_R1\_A\_c | 0 | 0 | 0 | 0 | 0 | 0 | 1 | -1 | 0 | 0 | 0 | 0 | 0 | 0 | 0 | 0 | 0 | 0 | 0 | 0 | 0 | 0 | 0 | 0 | 0 | 0 | 0 | 0 | 0 | 0 | 0 | 0 | 0 | 0 | 0 | 0 | 0 | 0 | 0 | 0 | 0 | 0 | 0 | 0 | 0 |
| pfk\_R1\_AF\_c | 0 | 0 | 0 | 0 | 0 | 0 | 0 | 1 | -1 | 0 | 0 | 0 | 0 | 0 | 0 | 0 | 0 | 0 | 0 | 0 | 0 | 0 | 0 | 0 | 0 | 0 | 0 | 0 | 0 | 0 | 0 | 0 | 0 | 0 | 0 | 0 | 0 | 0 | 0 | 0 | 0 | 0 | 0 | 0 | 0 |
| pfk\_R2\_c | 0 | 0 | 0 | 0 | 0 | 0 | 0 | 0 | 0 | 1 | -1 | 0 | 1 | -1 | 0 | 0 | 0 | 0 | 0 | 0 | 0 | 0 | 0 | 0 | 0 | 0 | 0 | 0 | 0 | 0 | 0 | 0 | 0 | 0 | 0 | 0 | 0 | 0 | 0 | 0 | 0 | 0 | 0 | 0 | 0 |
| pfk\_R2\_A\_c | 0 | 0 | 0 | 0 | 0 | 0 | 0 | 0 | 0 | 0 | 1 | -1 | 0 | 0 | 0 | 0 | 0 | 0 | 0 | 0 | 0 | 0 | 0 | 0 | 0 | 0 | 0 | 0 | 0 | 0 | 0 | 0 | 0 | 0 | 0 | 0 | 0 | 0 | 0 | 0 | 0 | 0 | 0 | 0 | 0 |
| pfk\_R2\_AF\_c | 0 | 0 | 0 | 0 | 0 | 0 | 0 | 0 | 0 | 0 | 0 | 1 | -1 | 0 | 0 | 0 | 0 | 0 | 0 | 0 | 0 | 0 | 0 | 0 | 0 | 0 | 0 | 0 | 0 | 0 | 0 | 0 | 0 | 0 | 0 | 0 | 0 | 0 | 0 | 0 | 0 | 0 | 0 | 0 | 0 |
| pfk\_R3\_c | 0 | 0 | 0 | 0 | 0 | 0 | 0 | 0 | 0 | 0 | 0 | 0 | 0 | 1 | -1 | 0 | 1 | -1 | 0 | 0 | 0 | 0 | 0 | 0 | 0 | 0 | 0 | 0 | 0 | 0 | 0 | 0 | 0 | 0 | 0 | 0 | 0 | 0 | 0 | 0 | 0 | 0 | 0 | 0 | 0 |
| pfk\_R3\_A\_c | 0 | 0 | 0 | 0 | 0 | 0 | 0 | 0 | 0 | 0 | 0 | 0 | 0 | 0 | 1 | -1 | 0 | 0 | 0 | 0 | 0 | 0 | 0 | 0 | 0 | 0 | 0 | 0 | 0 | 0 | 0 | 0 | 0 | 0 | 0 | 0 | 0 | 0 | 0 | 0 | 0 | 0 | 0 | 0 | 0 |
| pfk\_R3\_AF\_c | 0 | 0 | 0 | 0 | 0 | 0 | 0 | 0 | 0 | 0 | 0 | 0 | 0 | 0 | 0 | 1 | -1 | 0 | 0 | 0 | 0 | 0 | 0 | 0 | 0 | 0 | 0 | 0 | 0 | 0 | 0 | 0 | 0 | 0 | 0 | 0 | 0 | 0 | 0 | 0 | 0 | 0 | 0 | 0 | 0 |
| pfk\_R4\_c | 0 | 0 | 0 | 0 | 0 | 0 | 0 | 0 | 0 | 0 | 0 | 0 | 0 | 0 | 0 | 0 | 0 | 1 | -1 | 0 | 1 | 0 | 0 | 0 | 0 | 0 | 0 | 0 | 0 | 0 | 0 | 0 | 0 | 0 | 0 | 0 | 0 | 0 | 0 | 0 | 0 | 0 | 0 | 0 | 0 |
| pfk\_R4\_A\_c | 0 | 0 | 0 | 0 | 0 | 0 | 0 | 0 | 0 | 0 | 0 | 0 | 0 | 0 | 0 | 0 | 0 | 0 | 1 | -1 | 0 | 0 | 0 | 0 | 0 | 0 | 0 | 0 | 0 | 0 | 0 | 0 | 0 | 0 | 0 | 0 | 0 | 0 | 0 | 0 | 0 | 0 | 0 | 0 | 0 |
| pfk\_R4\_AF\_c | 0 | 0 | 0 | 0 | 0 | 0 | 0 | 0 | 0 | 0 | 0 | 0 | 0 | 0 | 0 | 0 | 0 | 0 | 0 | 1 | -1 | 0 | 0 | 0 | 0 | 0 | 0 | 0 | 0 | 0 | 0 | 0 | 0 | 0 | 0 | 0 | 0 | 0 | 0 | 0 | 0 | 0 | 0 | 0 | 0 |
| pfk\_T0\_c | 0 | 0 | 0 | 0 | 0 | 0 | 0 | 0 | 0 | 0 | 0 | 0 | 0 | 0 | 0 | 0 | 0 | 0 | 0 | 0 | 0 | 1 | -1 | 0 | 0 | 0 | 0 | 0 | 0 | 0 | 0 | 0 | 0 | 0 | 0 | 0 | 0 | 0 | 0 | 0 | 0 | 0 | 0 | 0 | 0 |
| pfk\_T1\_c | 0 | 0 | 0 | 0 | 0 | 0 | 0 | 0 | 0 | 0 | 0 | 0 | 0 | 0 | 0 | 0 | 0 | 0 | 0 | 0 | 0 | 0 | 1 | -1 | 0 | 0 | 0 | 0 | 0 | 0 | 0 | 0 | 0 | 0 | 0 | 0 | 0 | 0 | 0 | 0 | 0 | 0 | 0 | 0 | 0 |
| pfk\_T2\_c | 0 | 0 | 0 | 0 | 0 | 0 | 0 | 0 | 0 | 0 | 0 | 0 | 0 | 0 | 0 | 0 | 0 | 0 | 0 | 0 | 0 | 0 | 0 | 1 | -1 | 0 | 0 | 0 | 0 | 0 | 0 | 0 | 0 | 0 | 0 | 0 | 0 | 0 | 0 | 0 | 0 | 0 | 0 | 0 | 0 |
| pfk\_T3\_c | 0 | 0 | 0 | 0 | 0 | 0 | 0 | 0 | 0 | 0 | 0 | 0 | 0 | 0 | 0 | 0 | 0 | 0 | 0 | 0 | 0 | 0 | 0 | 0 | 1 | -1 | 0 | 0 | 0 | 0 | 0 | 0 | 0 | 0 | 0 | 0 | 0 | 0 | 0 | 0 | 0 | 0 | 0 | 0 | 0 |
| pfk\_T4\_c | 0 | 0 | 0 | 0 | 0 | 0 | 0 | 0 | 0 | 0 | 0 | 0 | 0 | 0 | 0 | 0 | 0 | 0 | 0 | 0 | 0 | 0 | 0 | 0 | 0 | 1 | 0 | 0 | 0 | 0 | 0 | 0 | 0 | 0 | 0 | 0 | 0 | 0 | 0 | 0 | 0 | 0 | 0 | 0 | 0 |
| fdp\_c | 0 | 0 | 0 | 0 | 1 | 0 | 0 | 0 | 1 | 0 | 0 | 0 | 1 | 0 | 0 | 0 | 1 | 0 | 0 | 0 | 1 | 0 | 0 | 0 | 0 | 0 | 0 | -1 | 0 | 0 | 0 | 0 | 0 | 0 | 0 | 0 | 0 | 0 | 0 | 0 | 0 | 0 | 0 | 0 | 0 |
| dhap\_c | 0 | 0 | 0 | 0 | 0 | 0 | 0 | 0 | 0 | 0 | 0 | 0 | 0 | 0 | 0 | 0 | 0 | 0 | 0 | 0 | 0 | 0 | 0 | 0 | 0 | 0 | -1 | 1 | 0 | 0 | 0 | 0 | 0 | 0 | 0 | 0 | 0 | 0 | 0 | 0 | 0 | 0 | 0 | 0 | 0 |
| g3p\_c | 0 | 0 | 0 | 0 | 0 | 0 | 0 | 0 | 0 | 0 | 0 | 0 | 0 | 0 | 0 | 0 | 0 | 0 | 0 | 0 | 0 | 0 | 0 | 0 | 0 | 0 | 1 | 1 | -1 | 0 | 0 | 0 | 0 | 0 | 0 | 0 | 0 | 0 | 0 | 0 | 0 | 0 | 0 | 0 | 0 |
| \_13dpg\_c | 0 | 0 | 0 | 0 | 0 | 0 | 0 | 0 | 0 | 0 | 0 | 0 | 0 | 0 | 0 | 0 | 0 | 0 | 0 | 0 | 0 | 0 | 0 | 0 | 0 | 0 | 0 | 0 | 1 | -1 | 0 | 0 | 0 | 0 | 0 | 0 | 0 | 0 | 0 | 0 | 0 | 0 | 0 | 0 | 0 |
| \_3pg\_c | 0 | 0 | 0 | 0 | 0 | 0 | 0 | 0 | 0 | 0 | 0 | 0 | 0 | 0 | 0 | 0 | 0 | 0 | 0 | 0 | 0 | 0 | 0 | 0 | 0 | 0 | 0 | 0 | 0 | 1 | -1 | 0 | 0 | 0 | 0 | 0 | 0 | 0 | 0 | 0 | 0 | 0 | 0 | 0 | 0 |
| \_2pg\_c | 0 | 0 | 0 | 0 | 0 | 0 | 0 | 0 | 0 | 0 | 0 | 0 | 0 | 0 | 0 | 0 | 0 | 0 | 0 | 0 | 0 | 0 | 0 | 0 | 0 | 0 | 0 | 0 | 0 | 0 | 1 | -1 | 0 | 0 | 0 | 0 | 0 | 0 | 0 | 0 | 0 | 0 | 0 | 0 | 0 |
| pep\_c | 0 | 0 | 0 | 0 | 0 | 0 | 0 | 0 | 0 | 0 | 0 | 0 | 0 | 0 | 0 | 0 | 0 | 0 | 0 | 0 | 0 | 0 | 0 | 0 | 0 | 0 | 0 | 0 | 0 | 0 | 0 | 1 | -1 | 0 | 0 | 0 | 0 | 0 | 0 | 0 | 0 | 0 | 0 | 0 | 0 |
| pyr\_c | 0 | 0 | 0 | 0 | 0 | 0 | 0 | 0 | 0 | 0 | 0 | 0 | 0 | 0 | 0 | 0 | 0 | 0 | 0 | 0 | 0 | 0 | 0 | 0 | 0 | 0 | 0 | 0 | 0 | 0 | 0 | 0 | 1 | -1 | 0 | 0 | -1 | 0 | 0 | 0 | 0 | 0 | 0 | 0 | 0 |
| lac\_\_L\_c | 0 | 0 | 0 | 0 | 0 | 0 | 0 | 0 | 0 | 0 | 0 | 0 | 0 | 0 | 0 | 0 | 0 | 0 | 0 | 0 | 0 | 0 | 0 | 0 | 0 | 0 | 0 | 0 | 0 | 0 | 0 | 0 | 0 | 1 | 0 | 0 | 0 | -1 | 0 | 0 | 0 | 0 | 0 | 0 | 0 |
| nad\_c | 0 | 0 | 0 | 0 | 0 | 0 | 0 | 0 | 0 | 0 | 0 | 0 | 0 | 0 | 0 | 0 | 0 | 0 | 0 | 0 | 0 | 0 | 0 | 0 | 0 | 0 | 0 | 0 | -1 | 0 | 0 | 0 | 0 | 1 | 0 | 0 | 0 | 0 | 0 | 1 | 0 | 0 | 0 | 0 | 0 |
| nadh\_c | 0 | 0 | 0 | 0 | 0 | 0 | 0 | 0 | 0 | 0 | 0 | 0 | 0 | 0 | 0 | 0 | 0 | 0 | 0 | 0 | 0 | 0 | 0 | 0 | 0 | 0 | 0 | 0 | 1 | 0 | 0 | 0 | 0 | -1 | 0 | 0 | 0 | 0 | 0 | -1 | 0 | 0 | 0 | 0 | 0 |
| amp\_c | 0 | 0 | 0 | 0 | 0 | -1 | 0 | 0 | 0 | -1 | 0 | 0 | 0 | -1 | 0 | 0 | 0 | -1 | 0 | 0 | 0 | 0 | 0 | 0 | 0 | 0 | 0 | 0 | 0 | 0 | 0 | 0 | 0 | 0 | -1 | 1 | 0 | 0 | 0 | 0 | 0 | 1 | 0 | 0 | 0 |
| adp\_c | 1 | 0 | 0 | 0 | 1 | 0 | 0 | 0 | 1 | 0 | 0 | 0 | 1 | 0 | 0 | 0 | 1 | 0 | 0 | 0 | 1 | 0 | 0 | 0 | 0 | 0 | 0 | 0 | 0 | -1 | 0 | 0 | -1 | 0 | 0 | -2 | 0 | 0 | 1 | 0 | 0 | 0 | 0 | 0 | 0 |
| atp\_c | -1 | 0 | -1 | 0 | 0 | 0 | -1 | 0 | 0 | 0 | -1 | 0 | 0 | 0 | -1 | 0 | 0 | 0 | -1 | 0 | 0 | 0 | -1 | -1 | -1 | -1 | 0 | 0 | 0 | 1 | 0 | 0 | 1 | 0 | 0 | 1 | 0 | 0 | -1 | 0 | 0 | 0 | 0 | 0 | 0 |
| pi\_c | 0 | 0 | 0 | 0 | 0 | 0 | 0 | 0 | 0 | 0 | 0 | 0 | 0 | 0 | 0 | 0 | 0 | 0 | 0 | 0 | 0 | 0 | 0 | 0 | 0 | 0 | 0 | 0 | -1 | 0 | 0 | 0 | 0 | 0 | 0 | 0 | 0 | 0 | 1 | 0 | 0 | 0 | 0 | 0 | -1 |
| h\_c | 1 | 0 | 0 | 0 | 1 | 0 | 0 | 0 | 1 | 0 | 0 | 0 | 1 | 0 | 0 | 0 | 1 | 0 | 0 | 0 | 1 | 0 | 0 | 0 | 0 | 0 | 0 | 0 | 1 | 0 | 0 | 0 | -1 | -1 | 0 | 0 | 0 | 0 | 1 | 1 | 0 | 0 | -1 | 0 | 0 |
| h2o\_c | 0 | 0 | 0 | 0 | 0 | 0 | 0 | 0 | 0 | 0 | 0 | 0 | 0 | 0 | 0 | 0 | 0 | 0 | 0 | 0 | 0 | 0 | 0 | 0 | 0 | 0 | 0 | 0 | 0 | 0 | 0 | 1 | 0 | 0 | 0 | 0 | 0 | 0 | -1 | 0 | 0 | 0 | 0 | -1 | 0 |

###### The stoichiometric matrix: glycolysis with PFK module¶

The properties of the stoichiometric matrix are shown in Table 14.4. All the reactions are elementally balanced except for the exchange reactions. The matrix has dimensions of 40 x 45 and a rank of 38 It thus has a 7 dimensional null space and a two dimensional left null space.

**Table 14.4:** The annotated stoichiometric matrix for glycolysis and the PFK enzyme.

```
[18]:
```

```
# Define labels
metabolite_ids = [m.id for m in glycolysis_PFK.metabolites]
reaction_ids = [r.id for r in glycolysis_PFK.reactions]

pi_str = r"$\pi_{j}$"
rho_str = r"$\rho_{i}$"
chopsnq = ['C', 'H', 'O', 'P', 'N', 'S', 'q', '[PFK]', '[NAD]']
time_inv_labels = ["$N_{\mathrm{tot}}$", "$PFK_{\mathrm{tot}}$"]
path_labels = ["$p_1$", "$p_2$", "$p_3$", "$p_4$", "$p_5$", "$p_6$", "$p_7$", ]

# Make table content from the stoichiometric matrix, elemental balancing of pathways
# participation number, and MinSpan pathways
S_matrix = glycolysis_PFK.update_S(array_type="dense", dtype=np.int64, update_model=False)
ES_matrix = glycolysis_PFK.get_elemental_charge_balancing(dtype=np.int64)
pi = np.count_nonzero(S_matrix, axis=0)
rho = np.count_nonzero(S_matrix, axis=1)
minspan_paths = np.array([
    [1, 1, 1, 1, 1, 0, 0, 0, 0, 0, 0, 0, 0, 0, 0, 0, 0, 0, 0, 0, 0, 0, 0, 0, 0, 0, 1, 1, 2, 2, 2, 2, 2, 2, 0, 0, 0, 2, 2, 0, 1, 0, 2, 0, 0],
    [1, 1, 0, 0, 0, 0, 1, 1, 1, 0, 0, 0, 0, 0, 0, 0, 0, 0, 0, 0, 0, 0, 0, 0, 0, 0, 1, 1, 2, 2, 2, 2, 2, 2, 0, 0, 0, 2, 2, 0, 1, 0, 2, 0, 0],
    [1, 1, 0, 0, 0, 0, 0, 0, 0, 0, 1, 1, 1, 0, 0, 0, 0, 0, 0, 0, 0, 0, 0, 0, 0, 0, 1, 1, 2, 2, 2, 2, 2, 2, 0, 0, 0, 2, 2, 0, 1, 0, 2, 0, 0],
    [1, 1, 0, 0, 0, 0, 0, 0, 0, 0, 0, 0, 0, 0, 1, 1, 1, 0, 0, 0, 0, 0, 0, 0, 0, 0, 1, 1, 2, 2, 2, 2, 2, 2, 0, 0, 0, 2, 2, 0, 1, 0, 2, 0, 0],
    [1, 1, 0, 0, 0, 0, 0, 0, 0, 0, 0, 0, 0, 0, 0, 0, 0, 0, 1, 1, 1, 0, 0, 0, 0, 0, 1, 1, 2, 2, 2, 2, 2, 2, 0, 0, 0, 2, 2, 0, 1, 0, 2, 0, 0],
    [0, 0, 0, 0, 0, 0, 0, 0, 0, 0, 0, 0, 0, 0, 0, 0, 0, 0, 1, 1, 1, 0, 0, 0, 0, 0, 0, 0, 0, 0, 0, 0, 0,-1, 0, 0, 1,-1, 0, 1, 0, 0, 2, 0, 0],
    [0, 0, 0, 0, 0, 0, 0, 0, 0, 0, 0, 0, 0, 0, 0, 0, 0, 0, 1, 1, 1, 0, 0, 0, 0, 0, 0, 0, 0, 0, 0, 0, 0, 0, 1, 0, 0, 0, 0, 0, 0, 1, 0, 0, 0],
])
table_14_4 = np.vstack((S_matrix, pi, ES_matrix, minspan_paths))

# Determine number of blank entries needed to be added to pad the table,
# Add connectivity number and time invariants to table content
blanks = [""]*(len(table_14_4) - len(metabolite_ids))
rho = np.concatenate((rho, blanks))

lns = np.zeros((2, 40), dtype=np.int64)
lns[0][32:34] = 1
lns[1][2:22] = 1

time_inv = np.array([np.concatenate([row, blanks]) for row in lns])
table_14_4 = np.vstack([table_14_4.T, rho, time_inv]).T

colors = {"glycolysis": "#ffffe6",    # Yellow
          "pfk": "#F4D03F",           # Dark Yellow
          "cofactor": "#ffe6cc",      # Orange
          "inorganic": "#fadffa",     # Pink
          "chopsnq": "#99e6ff",       # Blue
          "pathways": "#b399ff",      # Purple
          "pi": "#99ffff",            # Cyan
          "rho": "#ff9999",           # Red
          "time_invs": "#ccff99",     # Green
          "blank": "#f2f2f2"}         # Grey
bg_color_str = "background-color: "
cofactor_mets = ["nad_c", "nadh_c",  "amp_c", "adp_c", "atp_c",]
exch_misc_rxns= ["DM_amp_c", "SK_pyr_c", "SK_lac__L_c",
                 "SK_glc__D_c", "SK_amp_c", "ADK1",
                 "ATPM", "DM_nadh"]
inorganic_mets = ["pi_c", "h_c", "h2o_c"]
inorganic_exch = ["SK_h_c", "SK_h2o_c", "SK_pi_c"]

def highlight_table(df, model):
    df = df.copy()
    condition = lambda mmodel, row, col:  (
        (col not in exch_misc_rxns + inorganic_exch) and (row not in cofactor_mets + inorganic_mets) and (
            (row in mmodel.metabolites) or (col in mmodel.reactions)))
    inorganic_condition = lambda row, col: (col in inorganic_exch or row in inorganic_mets)
    for i, row in enumerate(df.index):
        for j, col in enumerate(df.columns):
            if df.loc[row, col] == "":
                main_key = "blank"
            elif row in pi_str:
                main_key = "pi"
            elif row in chopsnq:
                main_key = "chopsnq"
            elif row in path_labels:
                main_key = "pathways"
            elif col in rho_str:
                main_key = "rho"
            elif col in time_inv_labels:
                main_key = "time_invs"
            elif condition(glycolysis, row, col):
                main_key = "glycolysis"
            elif condition(PFK, row, col):
                main_key = "pfk"
            elif ((col in exch_misc_rxns or row in cofactor_mets) and not inorganic_condition(row, col)):
                main_key = "cofactor"
            elif inorganic_condition(row, col):
                main_key = "inorganic"
            else:
                continue
            df.loc[row, col] = bg_color_str + colors[main_key]
    return df

# Create index and column labels
index_labels = np.concatenate((metabolite_ids, [pi_str], chopsnq, path_labels))
column_labels = np.concatenate((reaction_ids, [rho_str], time_inv_labels))
# Create DataFrame
table_14_4 = pd.DataFrame(
    table_14_4, index=index_labels, columns=column_labels)
# Apply colors
table_14_4 = table_14_4.style.apply(
    highlight_table,  model=glycolysis_PFK, axis=None)
table_14_4
```

```
[18]:
```

|  | HEX1 | PGI | PFK\_R01 | PFK\_R02 | PFK\_R03 | PFK\_R10 | PFK\_R11 | PFK\_R12 | PFK\_R13 | PFK\_R20 | PFK\_R21 | PFK\_R22 | PFK\_R23 | PFK\_R30 | PFK\_R31 | PFK\_R32 | PFK\_R33 | PFK\_R40 | PFK\_R41 | PFK\_R42 | PFK\_R43 | PFK\_L | PFK\_T1 | PFK\_T2 | PFK\_T3 | PFK\_T4 | TPI | FBA | GAPD | PGK | PGM | ENO | PYK | LDH\_L | DM\_amp\_c | ADK1 | SK\_pyr\_c | SK\_lac\_\_L\_c | ATPM | DM\_nadh | SK\_glc\_\_D\_c | SK\_amp\_c | SK\_h\_c | SK\_h2o\_c | SK\_pi\_c | $\rho\_{i}$ | $N\_{\mathrm{tot}}$ | $PFK\_{\mathrm{tot}}$ |
| --- | --- | --- | --- | --- | --- | --- | --- | --- | --- | --- | --- | --- | --- | --- | --- | --- | --- | --- | --- | --- | --- | --- | --- | --- | --- | --- | --- | --- | --- | --- | --- | --- | --- | --- | --- | --- | --- | --- | --- | --- | --- | --- | --- | --- | --- | --- | --- | --- |
| glc\_\_D\_c | -1 | 0 | 0 | 0 | 0 | 0 | 0 | 0 | 0 | 0 | 0 | 0 | 0 | 0 | 0 | 0 | 0 | 0 | 0 | 0 | 0 | 0 | 0 | 0 | 0 | 0 | 0 | 0 | 0 | 0 | 0 | 0 | 0 | 0 | 0 | 0 | 0 | 0 | 0 | 0 | 1 | 0 | 0 | 0 | 0 | 2 | 0 | 0 |
| g6p\_c | 1 | -1 | 0 | 0 | 0 | 0 | 0 | 0 | 0 | 0 | 0 | 0 | 0 | 0 | 0 | 0 | 0 | 0 | 0 | 0 | 0 | 0 | 0 | 0 | 0 | 0 | 0 | 0 | 0 | 0 | 0 | 0 | 0 | 0 | 0 | 0 | 0 | 0 | 0 | 0 | 0 | 0 | 0 | 0 | 0 | 2 | 0 | 0 |
| f6p\_c | 0 | 1 | 0 | -1 | 0 | 0 | 0 | -1 | 0 | 0 | 0 | -1 | 0 | 0 | 0 | -1 | 0 | 0 | 0 | -1 | 0 | 0 | 0 | 0 | 0 | 0 | 0 | 0 | 0 | 0 | 0 | 0 | 0 | 0 | 0 | 0 | 0 | 0 | 0 | 0 | 0 | 0 | 0 | 0 | 0 | 6 | 0 | 1 |
| pfk\_R0\_c | 0 | 0 | -1 | 0 | 1 | -1 | 0 | 0 | 0 | 0 | 0 | 0 | 0 | 0 | 0 | 0 | 0 | 0 | 0 | 0 | 0 | -1 | 0 | 0 | 0 | 0 | 0 | 0 | 0 | 0 | 0 | 0 | 0 | 0 | 0 | 0 | 0 | 0 | 0 | 0 | 0 | 0 | 0 | 0 | 0 | 4 | 0 | 1 |
| pfk\_R0\_A\_c | 0 | 0 | 1 | -1 | 0 | 0 | 0 | 0 | 0 | 0 | 0 | 0 | 0 | 0 | 0 | 0 | 0 | 0 | 0 | 0 | 0 | 0 | 0 | 0 | 0 | 0 | 0 | 0 | 0 | 0 | 0 | 0 | 0 | 0 | 0 | 0 | 0 | 0 | 0 | 0 | 0 | 0 | 0 | 0 | 0 | 2 | 0 | 1 |
| pfk\_R0\_AF\_c | 0 | 0 | 0 | 1 | -1 | 0 | 0 | 0 | 0 | 0 | 0 | 0 | 0 | 0 | 0 | 0 | 0 | 0 | 0 | 0 | 0 | 0 | 0 | 0 | 0 | 0 | 0 | 0 | 0 | 0 | 0 | 0 | 0 | 0 | 0 | 0 | 0 | 0 | 0 | 0 | 0 | 0 | 0 | 0 | 0 | 2 | 0 | 1 |
| pfk\_R1\_c | 0 | 0 | 0 | 0 | 0 | 1 | -1 | 0 | 1 | -1 | 0 | 0 | 0 | 0 | 0 | 0 | 0 | 0 | 0 | 0 | 0 | 0 | 0 | 0 | 0 | 0 | 0 | 0 | 0 | 0 | 0 | 0 | 0 | 0 | 0 | 0 | 0 | 0 | 0 | 0 | 0 | 0 | 0 | 0 | 0 | 4 | 0 | 1 |
| pfk\_R1\_A\_c | 0 | 0 | 0 | 0 | 0 | 0 | 1 | -1 | 0 | 0 | 0 | 0 | 0 | 0 | 0 | 0 | 0 | 0 | 0 | 0 | 0 | 0 | 0 | 0 | 0 | 0 | 0 | 0 | 0 | 0 | 0 | 0 | 0 | 0 | 0 | 0 | 0 | 0 | 0 | 0 | 0 | 0 | 0 | 0 | 0 | 2 | 0 | 1 |
| pfk\_R1\_AF\_c | 0 | 0 | 0 | 0 | 0 | 0 | 0 | 1 | -1 | 0 | 0 | 0 | 0 | 0 | 0 | 0 | 0 | 0 | 0 | 0 | 0 | 0 | 0 | 0 | 0 | 0 | 0 | 0 | 0 | 0 | 0 | 0 | 0 | 0 | 0 | 0 | 0 | 0 | 0 | 0 | 0 | 0 | 0 | 0 | 0 | 2 | 0 | 1 |
| pfk\_R2\_c | 0 | 0 | 0 | 0 | 0 | 0 | 0 | 0 | 0 | 1 | -1 | 0 | 1 | -1 | 0 | 0 | 0 | 0 | 0 | 0 | 0 | 0 | 0 | 0 | 0 | 0 | 0 | 0 | 0 | 0 | 0 | 0 | 0 | 0 | 0 | 0 | 0 | 0 | 0 | 0 | 0 | 0 | 0 | 0 | 0 | 4 | 0 | 1 |
| pfk\_R2\_A\_c | 0 | 0 | 0 | 0 | 0 | 0 | 0 | 0 | 0 | 0 | 1 | -1 | 0 | 0 | 0 | 0 | 0 | 0 | 0 | 0 | 0 | 0 | 0 | 0 | 0 | 0 | 0 | 0 | 0 | 0 | 0 | 0 | 0 | 0 | 0 | 0 | 0 | 0 | 0 | 0 | 0 | 0 | 0 | 0 | 0 | 2 | 0 | 1 |
| pfk\_R2\_AF\_c | 0 | 0 | 0 | 0 | 0 | 0 | 0 | 0 | 0 | 0 | 0 | 1 | -1 | 0 | 0 | 0 | 0 | 0 | 0 | 0 | 0 | 0 | 0 | 0 | 0 | 0 | 0 | 0 | 0 | 0 | 0 | 0 | 0 | 0 | 0 | 0 | 0 | 0 | 0 | 0 | 0 | 0 | 0 | 0 | 0 | 2 | 0 | 1 |
| pfk\_R3\_c | 0 | 0 | 0 | 0 | 0 | 0 | 0 | 0 | 0 | 0 | 0 | 0 | 0 | 1 | -1 | 0 | 1 | -1 | 0 | 0 | 0 | 0 | 0 | 0 | 0 | 0 | 0 | 0 | 0 | 0 | 0 | 0 | 0 | 0 | 0 | 0 | 0 | 0 | 0 | 0 | 0 | 0 | 0 | 0 | 0 | 4 | 0 | 1 |
| pfk\_R3\_A\_c | 0 | 0 | 0 | 0 | 0 | 0 | 0 | 0 | 0 | 0 | 0 | 0 | 0 | 0 | 1 | -1 | 0 | 0 | 0 | 0 | 0 | 0 | 0 | 0 | 0 | 0 | 0 | 0 | 0 | 0 | 0 | 0 | 0 | 0 | 0 | 0 | 0 | 0 | 0 | 0 | 0 | 0 | 0 | 0 | 0 | 2 | 0 | 1 |
| pfk\_R3\_AF\_c | 0 | 0 | 0 | 0 | 0 | 0 | 0 | 0 | 0 | 0 | 0 | 0 | 0 | 0 | 0 | 1 | -1 | 0 | 0 | 0 | 0 | 0 | 0 | 0 | 0 | 0 | 0 | 0 | 0 | 0 | 0 | 0 | 0 | 0 | 0 | 0 | 0 | 0 | 0 | 0 | 0 | 0 | 0 | 0 | 0 | 2 | 0 | 1 |
| pfk\_R4\_c | 0 | 0 | 0 | 0 | 0 | 0 | 0 | 0 | 0 | 0 | 0 | 0 | 0 | 0 | 0 | 0 | 0 | 1 | -1 | 0 | 1 | 0 | 0 | 0 | 0 | 0 | 0 | 0 | 0 | 0 | 0 | 0 | 0 | 0 | 0 | 0 | 0 | 0 | 0 | 0 | 0 | 0 | 0 | 0 | 0 | 3 | 0 | 1 |
| pfk\_R4\_A\_c | 0 | 0 | 0 | 0 | 0 | 0 | 0 | 0 | 0 | 0 | 0 | 0 | 0 | 0 | 0 | 0 | 0 | 0 | 1 | -1 | 0 | 0 | 0 | 0 | 0 | 0 | 0 | 0 | 0 | 0 | 0 | 0 | 0 | 0 | 0 | 0 | 0 | 0 | 0 | 0 | 0 | 0 | 0 | 0 | 0 | 2 | 0 | 1 |
| pfk\_R4\_AF\_c | 0 | 0 | 0 | 0 | 0 | 0 | 0 | 0 | 0 | 0 | 0 | 0 | 0 | 0 | 0 | 0 | 0 | 0 | 0 | 1 | -1 | 0 | 0 | 0 | 0 | 0 | 0 | 0 | 0 | 0 | 0 | 0 | 0 | 0 | 0 | 0 | 0 | 0 | 0 | 0 | 0 | 0 | 0 | 0 | 0 | 2 | 0 | 1 |
| pfk\_T0\_c | 0 | 0 | 0 | 0 | 0 | 0 | 0 | 0 | 0 | 0 | 0 | 0 | 0 | 0 | 0 | 0 | 0 | 0 | 0 | 0 | 0 | 1 | -1 | 0 | 0 | 0 | 0 | 0 | 0 | 0 | 0 | 0 | 0 | 0 | 0 | 0 | 0 | 0 | 0 | 0 | 0 | 0 | 0 | 0 | 0 | 2 | 0 | 1 |
| pfk\_T1\_c | 0 | 0 | 0 | 0 | 0 | 0 | 0 | 0 | 0 | 0 | 0 | 0 | 0 | 0 | 0 | 0 | 0 | 0 | 0 | 0 | 0 | 0 | 1 | -1 | 0 | 0 | 0 | 0 | 0 | 0 | 0 | 0 | 0 | 0 | 0 | 0 | 0 | 0 | 0 | 0 | 0 | 0 | 0 | 0 | 0 | 2 | 0 | 1 |
| pfk\_T2\_c | 0 | 0 | 0 | 0 | 0 | 0 | 0 | 0 | 0 | 0 | 0 | 0 | 0 | 0 | 0 | 0 | 0 | 0 | 0 | 0 | 0 | 0 | 0 | 1 | -1 | 0 | 0 | 0 | 0 | 0 | 0 | 0 | 0 | 0 | 0 | 0 | 0 | 0 | 0 | 0 | 0 | 0 | 0 | 0 | 0 | 2 | 0 | 1 |
| pfk\_T3\_c | 0 | 0 | 0 | 0 | 0 | 0 | 0 | 0 | 0 | 0 | 0 | 0 | 0 | 0 | 0 | 0 | 0 | 0 | 0 | 0 | 0 | 0 | 0 | 0 | 1 | -1 | 0 | 0 | 0 | 0 | 0 | 0 | 0 | 0 | 0 | 0 | 0 | 0 | 0 | 0 | 0 | 0 | 0 | 0 | 0 | 2 | 0 | 1 |
| pfk\_T4\_c | 0 | 0 | 0 | 0 | 0 | 0 | 0 | 0 | 0 | 0 | 0 | 0 | 0 | 0 | 0 | 0 | 0 | 0 | 0 | 0 | 0 | 0 | 0 | 0 | 0 | 1 | 0 | 0 | 0 | 0 | 0 | 0 | 0 | 0 | 0 | 0 | 0 | 0 | 0 | 0 | 0 | 0 | 0 | 0 | 0 | 1 | 0 | 0 |
| fdp\_c | 0 | 0 | 0 | 0 | 1 | 0 | 0 | 0 | 1 | 0 | 0 | 0 | 1 | 0 | 0 | 0 | 1 | 0 | 0 | 0 | 1 | 0 | 0 | 0 | 0 | 0 | 0 | -1 | 0 | 0 | 0 | 0 | 0 | 0 | 0 | 0 | 0 | 0 | 0 | 0 | 0 | 0 | 0 | 0 | 0 | 6 | 0 | 0 |
| dhap\_c | 0 | 0 | 0 | 0 | 0 | 0 | 0 | 0 | 0 | 0 | 0 | 0 | 0 | 0 | 0 | 0 | 0 | 0 | 0 | 0 | 0 | 0 | 0 | 0 | 0 | 0 | -1 | 1 | 0 | 0 | 0 | 0 | 0 | 0 | 0 | 0 | 0 | 0 | 0 | 0 | 0 | 0 | 0 | 0 | 0 | 2 | 0 | 0 |
| g3p\_c | 0 | 0 | 0 | 0 | 0 | 0 | 0 | 0 | 0 | 0 | 0 | 0 | 0 | 0 | 0 | 0 | 0 | 0 | 0 | 0 | 0 | 0 | 0 | 0 | 0 | 0 | 1 | 1 | -1 | 0 | 0 | 0 | 0 | 0 | 0 | 0 | 0 | 0 | 0 | 0 | 0 | 0 | 0 | 0 | 0 | 3 | 0 | 0 |
| \_13dpg\_c | 0 | 0 | 0 | 0 | 0 | 0 | 0 | 0 | 0 | 0 | 0 | 0 | 0 | 0 | 0 | 0 | 0 | 0 | 0 | 0 | 0 | 0 | 0 | 0 | 0 | 0 | 0 | 0 | 1 | -1 | 0 | 0 | 0 | 0 | 0 | 0 | 0 | 0 | 0 | 0 | 0 | 0 | 0 | 0 | 0 | 2 | 0 | 0 |
| \_3pg\_c | 0 | 0 | 0 | 0 | 0 | 0 | 0 | 0 | 0 | 0 | 0 | 0 | 0 | 0 | 0 | 0 | 0 | 0 | 0 | 0 | 0 | 0 | 0 | 0 | 0 | 0 | 0 | 0 | 0 | 1 | -1 | 0 | 0 | 0 | 0 | 0 | 0 | 0 | 0 | 0 | 0 | 0 | 0 | 0 | 0 | 2 | 0 | 0 |
| \_2pg\_c | 0 | 0 | 0 | 0 | 0 | 0 | 0 | 0 | 0 | 0 | 0 | 0 | 0 | 0 | 0 | 0 | 0 | 0 | 0 | 0 | 0 | 0 | 0 | 0 | 0 | 0 | 0 | 0 | 0 | 0 | 1 | -1 | 0 | 0 | 0 | 0 | 0 | 0 | 0 | 0 | 0 | 0 | 0 | 0 | 0 | 2 | 0 | 0 |
| pep\_c | 0 | 0 | 0 | 0 | 0 | 0 | 0 | 0 | 0 | 0 | 0 | 0 | 0 | 0 | 0 | 0 | 0 | 0 | 0 | 0 | 0 | 0 | 0 | 0 | 0 | 0 | 0 | 0 | 0 | 0 | 0 | 1 | -1 | 0 | 0 | 0 | 0 | 0 | 0 | 0 | 0 | 0 | 0 | 0 | 0 | 2 | 0 | 0 |
| pyr\_c | 0 | 0 | 0 | 0 | 0 | 0 | 0 | 0 | 0 | 0 | 0 | 0 | 0 | 0 | 0 | 0 | 0 | 0 | 0 | 0 | 0 | 0 | 0 | 0 | 0 | 0 | 0 | 0 | 0 | 0 | 0 | 0 | 1 | -1 | 0 | 0 | -1 | 0 | 0 | 0 | 0 | 0 | 0 | 0 | 0 | 3 | 0 | 0 |
| lac\_\_L\_c | 0 | 0 | 0 | 0 | 0 | 0 | 0 | 0 | 0 | 0 | 0 | 0 | 0 | 0 | 0 | 0 | 0 | 0 | 0 | 0 | 0 | 0 | 0 | 0 | 0 | 0 | 0 | 0 | 0 | 0 | 0 | 0 | 0 | 1 | 0 | 0 | 0 | -1 | 0 | 0 | 0 | 0 | 0 | 0 | 0 | 2 | 0 | 0 |
| nad\_c | 0 | 0 | 0 | 0 | 0 | 0 | 0 | 0 | 0 | 0 | 0 | 0 | 0 | 0 | 0 | 0 | 0 | 0 | 0 | 0 | 0 | 0 | 0 | 0 | 0 | 0 | 0 | 0 | -1 | 0 | 0 | 0 | 0 | 1 | 0 | 0 | 0 | 0 | 0 | 1 | 0 | 0 | 0 | 0 | 0 | 3 | 1 | 0 |
| nadh\_c | 0 | 0 | 0 | 0 | 0 | 0 | 0 | 0 | 0 | 0 | 0 | 0 | 0 | 0 | 0 | 0 | 0 | 0 | 0 | 0 | 0 | 0 | 0 | 0 | 0 | 0 | 0 | 0 | 1 | 0 | 0 | 0 | 0 | -1 | 0 | 0 | 0 | 0 | 0 | -1 | 0 | 0 | 0 | 0 | 0 | 3 | 1 | 0 |
| amp\_c | 0 | 0 | 0 | 0 | 0 | -1 | 0 | 0 | 0 | -1 | 0 | 0 | 0 | -1 | 0 | 0 | 0 | -1 | 0 | 0 | 0 | 0 | 0 | 0 | 0 | 0 | 0 | 0 | 0 | 0 | 0 | 0 | 0 | 0 | -1 | 1 | 0 | 0 | 0 | 0 | 0 | 1 | 0 | 0 | 0 | 7 | 0 | 0 |
| adp\_c | 1 | 0 | 0 | 0 | 1 | 0 | 0 | 0 | 1 | 0 | 0 | 0 | 1 | 0 | 0 | 0 | 1 | 0 | 0 | 0 | 1 | 0 | 0 | 0 | 0 | 0 | 0 | 0 | 0 | -1 | 0 | 0 | -1 | 0 | 0 | -2 | 0 | 0 | 1 | 0 | 0 | 0 | 0 | 0 | 0 | 10 | 0 | 0 |
| atp\_c | -1 | 0 | -1 | 0 | 0 | 0 | -1 | 0 | 0 | 0 | -1 | 0 | 0 | 0 | -1 | 0 | 0 | 0 | -1 | 0 | 0 | 0 | -1 | -1 | -1 | -1 | 0 | 0 | 0 | 1 | 0 | 0 | 1 | 0 | 0 | 1 | 0 | 0 | -1 | 0 | 0 | 0 | 0 | 0 | 0 | 14 | 0 | 0 |
| pi\_c | 0 | 0 | 0 | 0 | 0 | 0 | 0 | 0 | 0 | 0 | 0 | 0 | 0 | 0 | 0 | 0 | 0 | 0 | 0 | 0 | 0 | 0 | 0 | 0 | 0 | 0 | 0 | 0 | -1 | 0 | 0 | 0 | 0 | 0 | 0 | 0 | 0 | 0 | 1 | 0 | 0 | 0 | 0 | 0 | -1 | 3 | 0 | 0 |
| h\_c | 1 | 0 | 0 | 0 | 1 | 0 | 0 | 0 | 1 | 0 | 0 | 0 | 1 | 0 | 0 | 0 | 1 | 0 | 0 | 0 | 1 | 0 | 0 | 0 | 0 | 0 | 0 | 0 | 1 | 0 | 0 | 0 | -1 | -1 | 0 | 0 | 0 | 0 | 1 | 1 | 0 | 0 | -1 | 0 | 0 | 12 | 0 | 0 |
| h2o\_c | 0 | 0 | 0 | 0 | 0 | 0 | 0 | 0 | 0 | 0 | 0 | 0 | 0 | 0 | 0 | 0 | 0 | 0 | 0 | 0 | 0 | 0 | 0 | 0 | 0 | 0 | 0 | 0 | 0 | 0 | 0 | 1 | 0 | 0 | 0 | 0 | 0 | 0 | -1 | 0 | 0 | 0 | 0 | -1 | 0 | 3 | 0 | 0 |
| $\pi\_{j}$ | 5 | 2 | 3 | 3 | 5 | 3 | 3 | 3 | 5 | 3 | 3 | 3 | 5 | 3 | 3 | 3 | 5 | 3 | 3 | 3 | 5 | 2 | 3 | 3 | 3 | 3 | 2 | 3 | 6 | 4 | 2 | 3 | 5 | 5 | 1 | 3 | 1 | 1 | 5 | 3 | 1 | 1 | 1 | 1 | 1 |  |  |  |
| C | 0 | 0 | 0 | 0 | 0 | 0 | 0 | 0 | 0 | 0 | 0 | 0 | 0 | 0 | 0 | 0 | 0 | 0 | 0 | 0 | 0 | 0 | 0 | 0 | 0 | 0 | 0 | 0 | 0 | 0 | 0 | 0 | 0 | 0 | -10 | 0 | -3 | -3 | 0 | 0 | 6 | 10 | 0 | 0 | 0 |  |  |  |
| H | 0 | 0 | 0 | 0 | 0 | 0 | 0 | 0 | 0 | 0 | 0 | 0 | 0 | 0 | 0 | 0 | 0 | 0 | 0 | 0 | 0 | 0 | 0 | 0 | 0 | 0 | 0 | 0 | 0 | 0 | 0 | 0 | 0 | 0 | -12 | 0 | -3 | -5 | 0 | 0 | 12 | 12 | -1 | -2 | -1 |  |  |  |
| O | 0 | 0 | 0 | 0 | 0 | 0 | 0 | 0 | 0 | 0 | 0 | 0 | 0 | 0 | 0 | 0 | 0 | 0 | 0 | 0 | 0 | 0 | 0 | 0 | 0 | 0 | 0 | 0 | 0 | 0 | 0 | 0 | 0 | 0 | -7 | 0 | -3 | -3 | 0 | 0 | 6 | 7 | 0 | -1 | -4 |  |  |  |
| P | 0 | 0 | 0 | 0 | 0 | 0 | 0 | 0 | 0 | 0 | 0 | 0 | 0 | 0 | 0 | 0 | 0 | 0 | 0 | 0 | 0 | 0 | 0 | 0 | 0 | 0 | 0 | 0 | 0 | 0 | 0 | 0 | 0 | 0 | -1 | 0 | 0 | 0 | 0 | 0 | 0 | 1 | 0 | 0 | -1 |  |  |  |
| N | 0 | 0 | 0 | 0 | 0 | 0 | 0 | 0 | 0 | 0 | 0 | 0 | 0 | 0 | 0 | 0 | 0 | 0 | 0 | 0 | 0 | 0 | 0 | 0 | 0 | 0 | 0 | 0 | 0 | 0 | 0 | 0 | 0 | 0 | -5 | 0 | 0 | 0 | 0 | 0 | 0 | 5 | 0 | 0 | 0 |  |  |  |
| S | 0 | 0 | 0 | 0 | 0 | 0 | 0 | 0 | 0 | 0 | 0 | 0 | 0 | 0 | 0 | 0 | 0 | 0 | 0 | 0 | 0 | 0 | 0 | 0 | 0 | 0 | 0 | 0 | 0 | 0 | 0 | 0 | 0 | 0 | 0 | 0 | 0 | 0 | 0 | 0 | 0 | 0 | 0 | 0 | 0 |  |  |  |
| q | 0 | 0 | 0 | 0 | 0 | 0 | 0 | 0 | 0 | 0 | 0 | 0 | 0 | 0 | 0 | 0 | 0 | 0 | 0 | 0 | 0 | 0 | 0 | 0 | 0 | 0 | 0 | 0 | 0 | 0 | 0 | 0 | 0 | 0 | 2 | 0 | 1 | 1 | 0 | 2 | 0 | -2 | -1 | 0 | 2 |  |  |  |
| [PFK] | 0 | 0 | 0 | 0 | 0 | 0 | 0 | 0 | 0 | 0 | 0 | 0 | 0 | 0 | 0 | 0 | 0 | 0 | 0 | 0 | 0 | 0 | 0 | 0 | 0 | 0 | 0 | 0 | 0 | 0 | 0 | 0 | 0 | 0 | 0 | 0 | 0 | 0 | 0 | 0 | 0 | 0 | 0 | 0 | 0 |  |  |  |
| [NAD] | 0 | 0 | 0 | 0 | 0 | 0 | 0 | 0 | 0 | 0 | 0 | 0 | 0 | 0 | 0 | 0 | 0 | 0 | 0 | 0 | 0 | 0 | 0 | 0 | 0 | 0 | 0 | 0 | 0 | 0 | 0 | 0 | 0 | 0 | 0 | 0 | 0 | 0 | 0 | 0 | 0 | 0 | 0 | 0 | 0 |  |  |  |
| $p\_1$ | 1 | 1 | 1 | 1 | 1 | 0 | 0 | 0 | 0 | 0 | 0 | 0 | 0 | 0 | 0 | 0 | 0 | 0 | 0 | 0 | 0 | 0 | 0 | 0 | 0 | 0 | 1 | 1 | 2 | 2 | 2 | 2 | 2 | 2 | 0 | 0 | 0 | 2 | 2 | 0 | 1 | 0 | 2 | 0 | 0 |  |  |  |
| $p\_2$ | 1 | 1 | 0 | 0 | 0 | 0 | 1 | 1 | 1 | 0 | 0 | 0 | 0 | 0 | 0 | 0 | 0 | 0 | 0 | 0 | 0 | 0 | 0 | 0 | 0 | 0 | 1 | 1 | 2 | 2 | 2 | 2 | 2 | 2 | 0 | 0 | 0 | 2 | 2 | 0 | 1 | 0 | 2 | 0 | 0 |  |  |  |
| $p\_3$ | 1 | 1 | 0 | 0 | 0 | 0 | 0 | 0 | 0 | 0 | 1 | 1 | 1 | 0 | 0 | 0 | 0 | 0 | 0 | 0 | 0 | 0 | 0 | 0 | 0 | 0 | 1 | 1 | 2 | 2 | 2 | 2 | 2 | 2 | 0 | 0 | 0 | 2 | 2 | 0 | 1 | 0 | 2 | 0 | 0 |  |  |  |
| $p\_4$ | 1 | 1 | 0 | 0 | 0 | 0 | 0 | 0 | 0 | 0 | 0 | 0 | 0 | 0 | 1 | 1 | 1 | 0 | 0 | 0 | 0 | 0 | 0 | 0 | 0 | 0 | 1 | 1 | 2 | 2 | 2 | 2 | 2 | 2 | 0 | 0 | 0 | 2 | 2 | 0 | 1 | 0 | 2 | 0 | 0 |  |  |  |
| $p\_5$ | 1 | 1 | 0 | 0 | 0 | 0 | 0 | 0 | 0 | 0 | 0 | 0 | 0 | 0 | 0 | 0 | 0 | 0 | 1 | 1 | 1 | 0 | 0 | 0 | 0 | 0 | 1 | 1 | 2 | 2 | 2 | 2 | 2 | 2 | 0 | 0 | 0 | 2 | 2 | 0 | 1 | 0 | 2 | 0 | 0 |  |  |  |
| $p\_6$ | 0 | 0 | 0 | 0 | 0 | 0 | 0 | 0 | 0 | 0 | 0 | 0 | 0 | 0 | 0 | 0 | 0 | 0 | 1 | 1 | 1 | 0 | 0 | 0 | 0 | 0 | 0 | 0 | 0 | 0 | 0 | 0 | 0 | -1 | 0 | 0 | 1 | -1 | 0 | 1 | 0 | 0 | 2 | 0 | 0 |  |  |  |
| $p\_7$ | 0 | 0 | 0 | 0 | 0 | 0 | 0 | 0 | 0 | 0 | 0 | 0 | 0 | 0 | 0 | 0 | 0 | 0 | 1 | 1 | 1 | 0 | 0 | 0 | 0 | 0 | 0 | 0 | 0 | 0 | 0 | 0 | 0 | 0 | 1 | 0 | 0 | 0 | 0 | 0 | 0 | 1 | 0 | 0 | 0 |  |  |  |

###### The null spaces: glycolysis with PFK module¶

The null space is thus 7 (=45-38) dimensional. It fundamentally has the same pathways as the previous models alone. The difference is that pathway 1 in Figure 10.2 now becomes five pathways \((\textbf{p}\_1\) through \(\textbf{p}\_5\) in Table 14.4), one through each of the \(R\_i\) forms of PFK, consistent with the properties of the stoichiometric matrix for the PFK subnetwork. The remaining pathways are the same as the ones seen in previous models and become \(\textbf{p}\_6\) and
in Table 14.4 to make a total of 7 pathways.

The left null space is 2 (=40-38) dimensional. It has the same pools as the previous models, namely the total NAD pool and the total PFK pool that originates from the stoichiometric matrix of the PFK subnetwork, Table 14.2.

##### Simulating the Dynamic Mass Balance¶

###### Defining the Steady State:¶

The PFK subnetwork is set up to be in balance with its metabolic network environment. This balance is ensured by keeping the substrate and regulator concentrations the same and the total flux through the reaction the same. The steady state of the integrated model will thus be the same as for the glycolysis alone. Perturbations away from the steady state will be different.

```
[19]:
```

```
sim_PFK = Simulation(glycolysis_PFK)

t0, tf = (0, 1e4)
sim_PFK.find_steady_state(
    glycolysis_PFK, strategy="simulate", update_values=True,
    tfinal=1e5, steps=1e5)

conc_sol_ss, flux_sol_ss = sim_PFK.simulate(
    glycolysis_PFK, time=(t0, tf, tf*10 + 1))
conc_sol_ss.view_time_profile()
```

###### Response to an increased \(k\_{ATPM}\)¶

We now perturb the integrated model by increasing the rate of ATP utilization as in Chapters 10. We show the results in the form of the same key flux phase portraits.

```
[20]:
```

```
conc_sol, flux_sol = sim_PFK.simulate(
    glycolysis_PFK, time=(t0, tf, tf * 10 + 1),
    perturbations={"kf_ATPM": "kf_ATPM * 1.5"})
```

```
[21]:
```

```
fig_14_4, ax = plt.subplots(nrows=1, ncols=1, figsize=(12, 5));

plot_time_profile(
    conc_sol, ax=ax, legend="right outside",
    plot_function="semilogx", xlim=(1e-6, tf),
    xlabel="Time [hr]", ylabel="Concentration [mM]",
    title=("Concentration Profile", L_FONT));
fig_14_4.tight_layout()
```

**Figure 14.4:** Simulating the combined system from the steady state with 50% increase in the rate of ATP utilization at \(t = 0.\)\*

###### The proton node¶

The proton node has a connectivity of 12 with 9 production reactions and 3 utilization reactions.

```
[22]:
```

```
fig_14_5 = plt.figure(figsize=(15, 6))
gs = fig_14_5.add_gridspec(nrows=3, ncols=2, width_ratios=[1.5, 1])

ax1 = fig_14_5.add_subplot(gs[0, 0])
ax2 = fig_14_5.add_subplot(gs[1, 0])
ax3 = fig_14_5.add_subplot(gs[2, 0])
ax4 = fig_14_5.add_subplot(gs[:, 1])

plot_time_profile(
    conc_sol, observable="h_c", ax=ax1,
    legend="right outside", plot_function="semilogx",
    xlim=(1e-6, tf), ylim=(7.7e-5, 1e-4*1.025),
    xlabel="Time [hr]", ylabel="Concentrations [mM]",
    title=("(a) Proton Concentration", L_FONT));

fluxes_in = ["HEX1", "GAPD", "ATPM", "DM_nadh",
             "PFK_R03", "PFK_R13", "PFK_R23", "PFK_R33", "PFK_R43"]
plot_time_profile(
    flux_sol, observable=fluxes_in, ax=ax2,
    legend="right outside", plot_function="semilogx",
    xlim=(1e-6, tf),  ylim=(0, 4),
    xlabel="Time [hr]", ylabel="Fluxes [mM/hr]",
    title=("(b) Fluxes in", L_FONT));

fluxes_out = ["PYK", "LDH_L", "SK_h_c"]
plot_time_profile(
    flux_sol, observable=fluxes_out, ax=ax3,
    legend="right outside", plot_function="semilogx",
    xlim=(1e-6, tf),  ylim=(1, 4),
    xlabel="Time [hr]", ylabel="Fluxes [mM/hr]",
    title=("(c) Fluxes out", L_FONT));

for flux_id, variables in zip(["Net_Flux_In", "Net_Flux_Out"],
                              [fluxes_in, fluxes_out]):
    flux_sol.make_aggregate_solution(
        flux_id, equation=" + ".join(variables), variables=variables)

time_points = [t0, 1e-1, 1e0, 1e1, 1e2, tf]
time_point_colors = [
    mpl.colors.to_hex(c)
    for c in mpl.cm.Reds(np.linspace(0.3, 1, len(time_points)))]

plot_phase_portrait(
    flux_sol, x="Net_Flux_In", y="Net_Flux_Out", ax=ax4,
    xlim=(5, 9), ylim=(5, 9),
    xlabel="Fluxes in [mm/Hr]", ylabel="Fluxes out [mm/Hr]",
    title=("(d) Phase Portrait of Fluxes", L_FONT),
    annotate_time_points=time_points,
    annotate_time_points_color=time_point_colors,
    annotate_time_points_legend="best");
fig_14_5.tight_layout()
```

**Figure 14.5:** The time profiles of the (a) proton concentration, (b) the fluxes that make protons, (c) the fluxes that use protons and (d) the phase portrait of the net flux in and net flux out (darker red colors indicate slower time scales).

```
[23]:
```

```
sim_glycolysis = Simulation(glycolysis)
sim_glycolysis.simulate(
    glycolysis, time=(t0, tf),
    perturbations={"kf_ATPM": "kf_ATPM * 1.5"});

linestyles = ["--", "-"]
colors = ["grey", "black"]

fig_14_6, ax = plt.subplots(nrows=1, ncols=1, figsize=(5, 5))

for i, (model, sim) in enumerate(zip([glycolysis, glycolysis_PFK],
                                     [sim_glycolysis, sim_PFK])):
    flux_solution = sim.flux_solutions.get_by_id(
        "_".join((model.id, "FluxSols")))
    plot_phase_portrait(
        flux_solution, x="ATPM", y="SK_h_c", ax=ax,
        legend=(model.id, "lower right"),
        xlabel="ATPM [mm/Hr]", ylabel="SK_h_c [mm/Hr]",
        xlim=(1.5, 3.5), ylim=(2, 4),
        title=("Phase Portrait of ATPM vs. SK_h_c", L_FONT),
        color=colors[i], linestyle=linestyles[i],
        annotate_time_points=time_points,
        annotate_time_points_color=time_point_colors,
        annotate_time_points_legend="best")
fig_14_6.tight_layout()
```

**Figure 14.6:** A phase portrait of the instantly imbalanced ATP demand flux and the proton exchange rate with (solid line) and without (dashed line) coupling of the PFK sub-network to the glycolytic network.

###### Key fluxes and pairwise phase portraits¶

Here we examine the key fluxes through the PFK enzyme and in the integrated model.

```
[24]:
```

```
PFK_module = glycolysis_PFK.enzyme_modules.PFK

# Create equations for flux through various enzyme module forms groups
equations_dict = {}
for i in range(0, 5):
    PFK_rxns = ["PFK_R{0:d}{1:d}".format(i, j) for j in range(1, 4)]
    equations_dict["R{0:d}i".format(i)] = [" + ".join(PFK_rxns),
                                           PFK_rxns]
# Obtain reactions responsible for catalysis
catalyzation_rxns = PFK_module.enzyme_module_reactions_categorized.get_by_id("catalyzation")
catalyzation_rxns = [m.id for m in catalyzation_rxns.members]

# Create equation for net flux through enzyme
equations_dict["PFK"] = [" + ".join(catalyzation_rxns),
                         catalyzation_rxns]

# Create flux solutions for equations
for flux_id, (equation, variables) in equations_dict.items():
    flux_sol.make_aggregate_solution(
        flux_id, equation=equation, variables=variables)

fig_14_7, axes = plt.subplots(nrows=2, ncols=1, figsize=(8, 6))
(ax1, ax2) = axes.flatten()

plot_time_profile(
    flux_sol, observable=list(equations_dict)[:-1], ax=ax1,
    legend="right outside", plot_function="loglog",
    xlim=(1e-6, tf), ylim=(1e-5, 1e1),
    xlabel="Time [hr]", ylabel="Flux [mM/hr]",
    title=("(a) Fluxes through R-States", L_FONT));

for i, (model, sim) in enumerate(zip([glycolysis, glycolysis_PFK],
                                     [sim_glycolysis, sim_PFK])):
    flux_solution = sim.flux_solutions.get_by_id(
        "_".join((model.id, "FluxSols")))

    plot_time_profile(
        flux_solution, observable="PFK", ax=ax2,
        legend=[model.id, "right outside"], plot_function="semilogx",
        xlim=(1e-6, tf), ylim=(.7, 1.3),
        xlabel="Time [hr]", ylabel="Flux [mM/hr]",
        color=colors[i], linestyle=linestyles[i],
        title=("(b) Net Flux through PFK", L_FONT));
fig_14_7.tight_layout()
```

**Figure 14.7:** (a): The individual fluxes through the R-forms of the PFK enzyme and (b) the PFK flux from the module. The new PFK flux is the sum of all fluxes through the R-forms. The dashed line is the PFK flux in the glycolytic model without the coupling of the PFK sub-network\*

```
[25]:
```

```
fig_14_8, axes = plt.subplots(nrows=2, ncols=3, figsize=(17, 10))
axes = axes.flatten()

time_points = [t0, 1e-1, 1e0, 1e1, 1e2, tf]
time_point_colors = [
    mpl.colors.to_hex(c)
    for c in mpl.cm.Reds(np.linspace(0.3, 1, len(time_points)))]

pairings = [
    ("ATPM", "DM_amp_c"), ("ATPM", "SK_pyr_c"), ("DM_amp_c", "SK_pyr_c"),
    ("GAPD", "LDH_L"), ("LDH_L", "DM_nadh"), ("HEX1", "PYK")]
xlims = [
    (1.80, 3.40), (1.80, 3.40), (0.00, 0.08),
    (1.70, 2.45), (1.55, 2.200), (0.88, 1.22)]
ylims = [
    (0.00, 0.08), (0.17, 0.31), (0.17, 0.31),
    (1.55, 2.20), (0.17, 0.245), (1.70, 2.45)]

linestyles = ["--", "-"]
colors = ["grey", "black"]

for j, (model, sim) in enumerate(zip([glycolysis, glycolysis_PFK],
                                [sim_glycolysis, sim_PFK])):
    flux_sol = sim.flux_solutions.get_by_id(
        "_".join((model.id, "FluxSols")))
    for i, ax in enumerate(axes):
        x_i, y_i = pairings[i]
        # Create legends
        legend = None
        time_points_legend = None
        if i == 2:
            legend = [model.id, "lower right outside"]
        if i == len(axes) - 1:
            time_points_legend = "upper right outside"


        plot_phase_portrait(
            flux_sol, x=x_i, y=y_i, ax=ax, legend=legend,
            xlabel=x_i, ylabel=y_i,
            xlim=xlims[i], ylim=ylims[i],
            title=("Phase Portrait of {0} vs. {1}".format(
                x_i, y_i), L_FONT),
            color=colors[j], linestyle=linestyles[j],
            annotate_time_points=time_points,
            annotate_time_points_color=time_point_colors,
            annotate_time_points_legend=time_points_legend)
fig_14_8.tight_layout()
```

**Figure 14.8:** The dynamic response of key fluxes in glycolysis. Detailed pairwise phase portraits: (a): \(v\_{ATPM}\ \textit{vs.}\ v\_{SK\_{PYR}}\). (b): \(v\_{GAPD}\ \textit{vs.}\ v\_{LDH\_{L}}\). (c): \(v\_{LDH\_{L}}\ \textit{vs.}\ v\_{DM\_{NADH}}\). (d): \(v\_{HEX1}\ \textit{vs.}\ v\_{PYK}\) Each panel compares the solution with (solid line) and without (dashed line) coupling of the PFK sub-network to the glycolytic network. The unregulated responses can be seen in in Figure 10.15. The
fluxes are in units of mM/h. The perturbation is reflected in the instantaneous move of the flux state from the initial steady state to an unsteady state, as indicated by the arrow placing the initial point at \(t = 0^+\). The system then returns to its steady state at \(t \rightarrow \infty\).

###### Ratios:¶

###### Increase in the rate of ATP use¶

The overall dynamic responses to this 50% increase in ATP utilization rate is similar with and without regulation of PFK. The enzyme in the steady state is slightly below 90% in the R form. Thus, there is little room for regulation to change. In Figure 14.9, we show the fraction of PFK in the R state versus the energy charge the response of the integrated system. The initial response to the drop in the energy charge, that results from the increased ATP usage rate, leads to an increase in PFK
that is in the R state. The fraction \(r\_{\mathrm{R}}\) reaches almost unity, which represents the full possible regulatory response. After the response dies down, the energy charge returns to a value close to the initial one, but the fraction of PFK in the R state, \(r\_{\mathrm{R}}\) settles down at about 97%, slightly above where it was before.

###### Decrease in the rate of ATP use¶

The regulatory action of PFK in this steady state to an increase energy usage is thus limited. To illustrate the non-linear nature of enzyme regulation, we now simulate the response of the integrated network to a drop 15% in the rate of use of ATP. The response is quite asymmetric to the response to an increase in ATP usage rate, Figure 14.9. The build up of ATP that results from the initial drop in its rate of usage rapidly inhibits PFK and the R fraction, \(r\_{\mathrm{R}}\), drops to about
40%. As the network then approaches its eventual steady state the ATP concentration drops, with \(r\_{\mathrm{R}}\) returning to about 85% and the energy charge settling at a level that that is higher than the initial point.

```
[26]:
```

```
fig_14_9 = plt.figure(figsize=(13, 5))
gs = fig_14_9.add_gridspec(nrows=2, ncols=2, width_ratios=[1, 1.5])

ax1 = fig_14_9.add_subplot(gs[:, 0])
ax2 = fig_14_9.add_subplot(gs[0, 1])
ax3 = fig_14_9.add_subplot(gs[1, 1])

# Create pool for relaxed enzyme fraction (active fraction) and energy charge
relaxed_fraction = PFK.make_enzyme_fraction(
    "forms", top="Relaxed", bottom="Equation")
relaxed_fraction = str(strip_time(relaxed_fraction))

equations_dict = {}
equations_dict["r_R"] = [relaxed_fraction,
                         [m.id for m in PFK_module.enzyme_module_forms]]
equations_dict["EC"] = ["(2*atp_c + adp_c)/(2*(atp_c + adp_c + amp_c))",
                        ["atp_c", "adp_c", "amp_c"]]
# Create pooled solutions for equations
for pool_id, (equation, variables) in equations_dict.items():
    conc_sol_ss.make_aggregate_solution(
        pool_id, equation=equation, variables=variables)

ax1.plot([conc_sol_ss["EC"]]*2, [0, 1.05], color="grey", linestyle="--")
ax1.plot([0, 1.05], [conc_sol_ss["r_R"]]*2, color="grey", linestyle="--")

colors = ["blue", "red"]
ylims = [(0.6, 1.05), (0.3, 1.05)]

perturbations = dict(zip(
    ["50% increase in ATP utilization", "15% decrease in ATP utilization"],
    [{"kf_ATPM": "kf_ATPM * 1.5"}, {"kf_ATPM": "kf_ATPM * 0.85"}]))
for i, (perturb_type, perturb_dict) in enumerate(perturbations.items()):
    conc_sol, flux_sol = sim_PFK.simulate(
        glycolysis_PFK, time=(t0, tf, tf * 10 + 1),
        perturbations=perturb_dict)

    # Create pooled solutions for equations
    for pool_id, (equation, variables) in equations_dict.items():
        conc_sol.make_aggregate_solution(
            pool_id, equation=equation, variables=variables)

    plot_phase_portrait(
        conc_sol, x="EC", y="r_R", ax=ax1,
        legend=[perturb_type, "lower outside"],
        color=colors[i], linestyle="--",
        xlabel="Energy Charge", ylabel="$r_R$",
        xlim=(0.55, 1.05), ylim=(0.3, 1.05),
        title=("Phase Portrait of E.C. vs. $r_R$", L_FONT),
        annotate_time_points="endpoints",
        annotate_time_points_color=["xkcd:light purple",
                                    "xkcd:purple"],
        annotate_time_points_legend="lower left")

    if i == 0:
        ax = ax2
        legend = "lower right outside"
    else:
        ax = ax3
        legend = None

    plot_time_profile(
        conc_sol, ax=ax, observable=["EC", "r_R"], legend=legend,
        xlabel="Time [hr]", ylabel="Ratios",
        xlim=(t0, 100), ylim=ylims[i],
        title=("Ratio Time Profiles: " + perturb_type, L_FONT));
fig_14_9.tight_layout()
```

**Figure 14.9:** The dynamic response of key ratios. (a): The phase portrait for the energy charge versus \(r\_{\mathrm{R}}\) for a change in ATP utilization rate \((k\_{ATPM})\) where blue represents a 50% increase in ATP utilization and red represents a decrease 15% decrease in ATP utilization. (b): Time profile for the \(r\_{\mathrm{R}}\) and energy charge for an increase in ATP utilization. (c): Time profile for the \(r\_{\mathrm{R}}\) and energy charge for an decrease in ATP
utilization\*

##### Integration of PFK with RBC Metabolic Network¶

The PFK subnetwork can be integrated with the core network model of chapter 13. The integration process is straight forward. Though simulation we can compare the networks with and without the PFK subnetwork. The integration is performed as:

```
[27]:
```

```
# Create RBC model
glycolysis = create_test_model("SB2_Glycolysis")
ppp = create_test_model("SB2_PentosePhosphatePathway")
ampsn = create_test_model("SB2_AMPSalvageNetwork")
hemoglobin = create_test_model("SB2_Hemoglobin")

RBC_PFK = glycolysis.merge(ppp, inplace=False)
RBC_PFK.merge(ampsn, inplace=True)
RBC_PFK.remove_reactions([
    r for r in RBC_PFK.boundary
    if r.id in [
        "SK_g6p_c", "DM_f6p_c", "DM_g3p_c",
        "DM_r5p_c", "DM_amp_c", "SK_amp_c"]])
RBC_PFK.remove_boundary_conditions([
    "g6p_b", "f6p_b", "g3p_b", "r5p_b", "amp_b"])

RBC_PFK.reactions.PRPPS.subtract_metabolites({
    RBC_PFK.metabolites.atp_c: -1,
    RBC_PFK.metabolites.adp_c: 2})
RBC_PFK.reactions.PRPPS.add_metabolites({
    RBC_PFK.metabolites.amp_c: 1})
RBC_PFK.merge(hemoglobin, inplace=True)
RBC_PFK.id = "RBC"

# Load PFK module
PFK = create_test_model("SB2_PFK")
# Update the module with the parameters determined in section 14.2.

# Add PFK module to system
RBC_PFK = RBC_PFK.merge(PFK, inplace=False)
# Delete old PFK reaction
RBC_PFK.remove_reactions([RBC_PFK.reactions.PFK])
```

```
Ignoring reaction 'SK_h_c' since it already exists.
Ignoring reaction 'SK_h2o_c' since it already exists.
Ignoring reaction 'ATPM' since it already exists.
Ignoring reaction 'SK_amp_c' since it already exists.
Ignoring reaction 'SK_h_c' since it already exists.
Ignoring reaction 'SK_h2o_c' since it already exists.
```

This results in an integrated network whose stoichiometric matrix is of dimensions 68 x 76 and of rank 63

```
[28]:
```

```
# Define new order for metabolites
new_metabolite_order = [
    "glc__D_c", "g6p_c", "f6p_c", "pfk_R0_c", "pfk_R0_A_c", "pfk_R0_AF_c", "pfk_R1_c", "pfk_R1_A_c", "pfk_R1_AF_c",
    "pfk_R2_c", "pfk_R2_A_c", "pfk_R2_AF_c", "pfk_R3_c", "pfk_R3_A_c", "pfk_R3_AF_c","pfk_R4_c", "pfk_R4_A_c",
    "pfk_R4_AF_c", "pfk_T0_c","pfk_T1_c", "pfk_T2_c", "pfk_T3_c", "pfk_T4_c", "fdp_c", "dhap_c", "g3p_c",
    "_13dpg_c", "_3pg_c", "_2pg_c", "pep_c", "pyr_c", "lac__L_c", "_6pgl_c", "_6pgc_c", "ru5p__D_c", "xu5p__D_c",
    "r5p_c", "s7p_c", "e4p_c", "ade_c", "adn_c", "imp_c", "ins_c", "hxan_c", "r1p_c", "prpp_c", "_23dpg_c","hb_c",
    "hb_1o2_c", "hb_2o2_c", "hb_3o2_c", "hb_4o2_c", "dhb_c", "nad_c", "nadh_c", "amp_c", "adp_c", "atp_c",
    "nadp_c", "nadph_c", "gthrd_c", "gthox_c", "pi_c", "h_c", "h2o_c", "co2_c", "nh3_c", "o2_c"]

if len(RBC_PFK.metabolites) == len(new_metabolite_order):
    RBC_PFK.metabolites = DictList(RBC_PFK.metabolites.get_by_any(new_metabolite_order))
# Define new order for reactions
new_reaction_order = [
    "HEX1", "PGI", "PFK_R01", "PFK_R02", "PFK_R03", "PFK_R11", "PFK_R12", "PFK_R13", "PFK_R21", "PFK_R22", "PFK_R23",
    "PFK_R31", "PFK_R32", "PFK_R33","PFK_R41", "PFK_R42", "PFK_R43", "PFK_R10", "PFK_R20", "PFK_R30","PFK_R40",
    "PFK_T1", "PFK_T2", "PFK_T3", "PFK_T4", "PFK_L", "FBA", "TPI", "GAPD", "PGK", "PGM", "ENO", "PYK", "LDH_L",
    "G6PDH2r", "PGL", "GND", "RPE", "RPI", "TKT1", "TKT2", "TALA", "ADNK1", "NTD7", "ADA","AMPDA", "NTD11", "PUNP5",
    "PPM", "PRPPS", "ADPT", "ADK1", "DPGM", "DPGase", "HBDPG", "HBO1", "HBO2", "HBO3", "HBO4", "ATPM", "DM_nadh",
    "GTHOr", "GSHR", "SK_glc__D_c", "SK_pyr_c", "SK_lac__L_c", "SK_ade_c", "SK_adn_c", "SK_ins_c", "SK_hxan_c",
    "SK_pi_c", "SK_h_c", "SK_h2o_c", "SK_co2_c", "SK_nh3_c", "SK_o2_c"]
if len(RBC_PFK.reactions) == len(new_reaction_order):
    RBC_PFK.reactions = DictList(RBC_PFK.reactions.get_by_any(new_reaction_order))
RBC_PFK.update_S(array_type="DataFrame", dtype=np.int64);
```

```
[28]:
```

|  | HEX1 | PGI | PFK\_R01 | PFK\_R02 | PFK\_R03 | PFK\_R11 | PFK\_R12 | PFK\_R13 | PFK\_R21 | PFK\_R22 | PFK\_R23 | PFK\_R31 | PFK\_R32 | PFK\_R33 | PFK\_R41 | PFK\_R42 | PFK\_R43 | PFK\_R10 | PFK\_R20 | PFK\_R30 | PFK\_R40 | PFK\_T1 | PFK\_T2 | PFK\_T3 | PFK\_T4 | PFK\_L | FBA | TPI | GAPD | PGK | PGM | ENO | PYK | LDH\_L | G6PDH2r | PGL | GND | RPE | RPI | TKT1 | TKT2 | TALA | ADNK1 | NTD7 | ADA | AMPDA | NTD11 | PUNP5 | PPM | PRPPS | ADPT | ADK1 | DPGM | DPGase | HBDPG | HBO1 | HBO2 | HBO3 | HBO4 | ATPM | DM\_nadh | GTHOr | GSHR | SK\_glc\_\_D\_c | SK\_pyr\_c | SK\_lac\_\_L\_c | SK\_ade\_c | SK\_adn\_c | SK\_ins\_c | SK\_hxan\_c | SK\_pi\_c | SK\_h\_c | SK\_h2o\_c | SK\_co2\_c | SK\_nh3\_c | SK\_o2\_c |
| --- | --- | --- | --- | --- | --- | --- | --- | --- | --- | --- | --- | --- | --- | --- | --- | --- | --- | --- | --- | --- | --- | --- | --- | --- | --- | --- | --- | --- | --- | --- | --- | --- | --- | --- | --- | --- | --- | --- | --- | --- | --- | --- | --- | --- | --- | --- | --- | --- | --- | --- | --- | --- | --- | --- | --- | --- | --- | --- | --- | --- | --- | --- | --- | --- | --- | --- | --- | --- | --- | --- | --- | --- | --- | --- | --- | --- |
| glc\_\_D\_c | -1 | 0 | 0 | 0 | 0 | 0 | 0 | 0 | 0 | 0 | 0 | 0 | 0 | 0 | 0 | 0 | 0 | 0 | 0 | 0 | 0 | 0 | 0 | 0 | 0 | 0 | 0 | 0 | 0 | 0 | 0 | 0 | 0 | 0 | 0 | 0 | 0 | 0 | 0 | 0 | 0 | 0 | 0 | 0 | 0 | 0 | 0 | 0 | 0 | 0 | 0 | 0 | 0 | 0 | 0 | 0 | 0 | 0 | 0 | 0 | 0 | 0 | 0 | 1 | 0 | 0 | 0 | 0 | 0 | 0 | 0 | 0 | 0 | 0 | 0 | 0 |
| g6p\_c | 1 | -1 | 0 | 0 | 0 | 0 | 0 | 0 | 0 | 0 | 0 | 0 | 0 | 0 | 0 | 0 | 0 | 0 | 0 | 0 | 0 | 0 | 0 | 0 | 0 | 0 | 0 | 0 | 0 | 0 | 0 | 0 | 0 | 0 | -1 | 0 | 0 | 0 | 0 | 0 | 0 | 0 | 0 | 0 | 0 | 0 | 0 | 0 | 0 | 0 | 0 | 0 | 0 | 0 | 0 | 0 | 0 | 0 | 0 | 0 | 0 | 0 | 0 | 0 | 0 | 0 | 0 | 0 | 0 | 0 | 0 | 0 | 0 | 0 | 0 | 0 |
| f6p\_c | 0 | 1 | 0 | -1 | 0 | 0 | -1 | 0 | 0 | -1 | 0 | 0 | -1 | 0 | 0 | -1 | 0 | 0 | 0 | 0 | 0 | 0 | 0 | 0 | 0 | 0 | 0 | 0 | 0 | 0 | 0 | 0 | 0 | 0 | 0 | 0 | 0 | 0 | 0 | 0 | 1 | 1 | 0 | 0 | 0 | 0 | 0 | 0 | 0 | 0 | 0 | 0 | 0 | 0 | 0 | 0 | 0 | 0 | 0 | 0 | 0 | 0 | 0 | 0 | 0 | 0 | 0 | 0 | 0 | 0 | 0 | 0 | 0 | 0 | 0 | 0 |
| pfk\_R0\_c | 0 | 0 | -1 | 0 | 1 | 0 | 0 | 0 | 0 | 0 | 0 | 0 | 0 | 0 | 0 | 0 | 0 | -1 | 0 | 0 | 0 | 0 | 0 | 0 | 0 | -1 | 0 | 0 | 0 | 0 | 0 | 0 | 0 | 0 | 0 | 0 | 0 | 0 | 0 | 0 | 0 | 0 | 0 | 0 | 0 | 0 | 0 | 0 | 0 | 0 | 0 | 0 | 0 | 0 | 0 | 0 | 0 | 0 | 0 | 0 | 0 | 0 | 0 | 0 | 0 | 0 | 0 | 0 | 0 | 0 | 0 | 0 | 0 | 0 | 0 | 0 |
| pfk\_R0\_A\_c | 0 | 0 | 1 | -1 | 0 | 0 | 0 | 0 | 0 | 0 | 0 | 0 | 0 | 0 | 0 | 0 | 0 | 0 | 0 | 0 | 0 | 0 | 0 | 0 | 0 | 0 | 0 | 0 | 0 | 0 | 0 | 0 | 0 | 0 | 0 | 0 | 0 | 0 | 0 | 0 | 0 | 0 | 0 | 0 | 0 | 0 | 0 | 0 | 0 | 0 | 0 | 0 | 0 | 0 | 0 | 0 | 0 | 0 | 0 | 0 | 0 | 0 | 0 | 0 | 0 | 0 | 0 | 0 | 0 | 0 | 0 | 0 | 0 | 0 | 0 | 0 |
| pfk\_R0\_AF\_c | 0 | 0 | 0 | 1 | -1 | 0 | 0 | 0 | 0 | 0 | 0 | 0 | 0 | 0 | 0 | 0 | 0 | 0 | 0 | 0 | 0 | 0 | 0 | 0 | 0 | 0 | 0 | 0 | 0 | 0 | 0 | 0 | 0 | 0 | 0 | 0 | 0 | 0 | 0 | 0 | 0 | 0 | 0 | 0 | 0 | 0 | 0 | 0 | 0 | 0 | 0 | 0 | 0 | 0 | 0 | 0 | 0 | 0 | 0 | 0 | 0 | 0 | 0 | 0 | 0 | 0 | 0 | 0 | 0 | 0 | 0 | 0 | 0 | 0 | 0 | 0 |
| pfk\_R1\_c | 0 | 0 | 0 | 0 | 0 | -1 | 0 | 1 | 0 | 0 | 0 | 0 | 0 | 0 | 0 | 0 | 0 | 1 | -1 | 0 | 0 | 0 | 0 | 0 | 0 | 0 | 0 | 0 | 0 | 0 | 0 | 0 | 0 | 0 | 0 | 0 | 0 | 0 | 0 | 0 | 0 | 0 | 0 | 0 | 0 | 0 | 0 | 0 | 0 | 0 | 0 | 0 | 0 | 0 | 0 | 0 | 0 | 0 | 0 | 0 | 0 | 0 | 0 | 0 | 0 | 0 | 0 | 0 | 0 | 0 | 0 | 0 | 0 | 0 | 0 | 0 |
| pfk\_R1\_A\_c | 0 | 0 | 0 | 0 | 0 | 1 | -1 | 0 | 0 | 0 | 0 | 0 | 0 | 0 | 0 | 0 | 0 | 0 | 0 | 0 | 0 | 0 | 0 | 0 | 0 | 0 | 0 | 0 | 0 | 0 | 0 | 0 | 0 | 0 | 0 | 0 | 0 | 0 | 0 | 0 | 0 | 0 | 0 | 0 | 0 | 0 | 0 | 0 | 0 | 0 | 0 | 0 | 0 | 0 | 0 | 0 | 0 | 0 | 0 | 0 | 0 | 0 | 0 | 0 | 0 | 0 | 0 | 0 | 0 | 0 | 0 | 0 | 0 | 0 | 0 | 0 |
| pfk\_R1\_AF\_c | 0 | 0 | 0 | 0 | 0 | 0 | 1 | -1 | 0 | 0 | 0 | 0 | 0 | 0 | 0 | 0 | 0 | 0 | 0 | 0 | 0 | 0 | 0 | 0 | 0 | 0 | 0 | 0 | 0 | 0 | 0 | 0 | 0 | 0 | 0 | 0 | 0 | 0 | 0 | 0 | 0 | 0 | 0 | 0 | 0 | 0 | 0 | 0 | 0 | 0 | 0 | 0 | 0 | 0 | 0 | 0 | 0 | 0 | 0 | 0 | 0 | 0 | 0 | 0 | 0 | 0 | 0 | 0 | 0 | 0 | 0 | 0 | 0 | 0 | 0 | 0 |
| pfk\_R2\_c | 0 | 0 | 0 | 0 | 0 | 0 | 0 | 0 | -1 | 0 | 1 | 0 | 0 | 0 | 0 | 0 | 0 | 0 | 1 | -1 | 0 | 0 | 0 | 0 | 0 | 0 | 0 | 0 | 0 | 0 | 0 | 0 | 0 | 0 | 0 | 0 | 0 | 0 | 0 | 0 | 0 | 0 | 0 | 0 | 0 | 0 | 0 | 0 | 0 | 0 | 0 | 0 | 0 | 0 | 0 | 0 | 0 | 0 | 0 | 0 | 0 | 0 | 0 | 0 | 0 | 0 | 0 | 0 | 0 | 0 | 0 | 0 | 0 | 0 | 0 | 0 |
| pfk\_R2\_A\_c | 0 | 0 | 0 | 0 | 0 | 0 | 0 | 0 | 1 | -1 | 0 | 0 | 0 | 0 | 0 | 0 | 0 | 0 | 0 | 0 | 0 | 0 | 0 | 0 | 0 | 0 | 0 | 0 | 0 | 0 | 0 | 0 | 0 | 0 | 0 | 0 | 0 | 0 | 0 | 0 | 0 | 0 | 0 | 0 | 0 | 0 | 0 | 0 | 0 | 0 | 0 | 0 | 0 | 0 | 0 | 0 | 0 | 0 | 0 | 0 | 0 | 0 | 0 | 0 | 0 | 0 | 0 | 0 | 0 | 0 | 0 | 0 | 0 | 0 | 0 | 0 |
| pfk\_R2\_AF\_c | 0 | 0 | 0 | 0 | 0 | 0 | 0 | 0 | 0 | 1 | -1 | 0 | 0 | 0 | 0 | 0 | 0 | 0 | 0 | 0 | 0 | 0 | 0 | 0 | 0 | 0 | 0 | 0 | 0 | 0 | 0 | 0 | 0 | 0 | 0 | 0 | 0 | 0 | 0 | 0 | 0 | 0 | 0 | 0 | 0 | 0 | 0 | 0 | 0 | 0 | 0 | 0 | 0 | 0 | 0 | 0 | 0 | 0 | 0 | 0 | 0 | 0 | 0 | 0 | 0 | 0 | 0 | 0 | 0 | 0 | 0 | 0 | 0 | 0 | 0 | 0 |
| pfk\_R3\_c | 0 | 0 | 0 | 0 | 0 | 0 | 0 | 0 | 0 | 0 | 0 | -1 | 0 | 1 | 0 | 0 | 0 | 0 | 0 | 1 | -1 | 0 | 0 | 0 | 0 | 0 | 0 | 0 | 0 | 0 | 0 | 0 | 0 | 0 | 0 | 0 | 0 | 0 | 0 | 0 | 0 | 0 | 0 | 0 | 0 | 0 | 0 | 0 | 0 | 0 | 0 | 0 | 0 | 0 | 0 | 0 | 0 | 0 | 0 | 0 | 0 | 0 | 0 | 0 | 0 | 0 | 0 | 0 | 0 | 0 | 0 | 0 | 0 | 0 | 0 | 0 |
| pfk\_R3\_A\_c | 0 | 0 | 0 | 0 | 0 | 0 | 0 | 0 | 0 | 0 | 0 | 1 | -1 | 0 | 0 | 0 | 0 | 0 | 0 | 0 | 0 | 0 | 0 | 0 | 0 | 0 | 0 | 0 | 0 | 0 | 0 | 0 | 0 | 0 | 0 | 0 | 0 | 0 | 0 | 0 | 0 | 0 | 0 | 0 | 0 | 0 | 0 | 0 | 0 | 0 | 0 | 0 | 0 | 0 | 0 | 0 | 0 | 0 | 0 | 0 | 0 | 0 | 0 | 0 | 0 | 0 | 0 | 0 | 0 | 0 | 0 | 0 | 0 | 0 | 0 | 0 |
| pfk\_R3\_AF\_c | 0 | 0 | 0 | 0 | 0 | 0 | 0 | 0 | 0 | 0 | 0 | 0 | 1 | -1 | 0 | 0 | 0 | 0 | 0 | 0 | 0 | 0 | 0 | 0 | 0 | 0 | 0 | 0 | 0 | 0 | 0 | 0 | 0 | 0 | 0 | 0 | 0 | 0 | 0 | 0 | 0 | 0 | 0 | 0 | 0 | 0 | 0 | 0 | 0 | 0 | 0 | 0 | 0 | 0 | 0 | 0 | 0 | 0 | 0 | 0 | 0 | 0 | 0 | 0 | 0 | 0 | 0 | 0 | 0 | 0 | 0 | 0 | 0 | 0 | 0 | 0 |
| pfk\_R4\_c | 0 | 0 | 0 | 0 | 0 | 0 | 0 | 0 | 0 | 0 | 0 | 0 | 0 | 0 | -1 | 0 | 1 | 0 | 0 | 0 | 1 | 0 | 0 | 0 | 0 | 0 | 0 | 0 | 0 | 0 | 0 | 0 | 0 | 0 | 0 | 0 | 0 | 0 | 0 | 0 | 0 | 0 | 0 | 0 | 0 | 0 | 0 | 0 | 0 | 0 | 0 | 0 | 0 | 0 | 0 | 0 | 0 | 0 | 0 | 0 | 0 | 0 | 0 | 0 | 0 | 0 | 0 | 0 | 0 | 0 | 0 | 0 | 0 | 0 | 0 | 0 |
| pfk\_R4\_A\_c | 0 | 0 | 0 | 0 | 0 | 0 | 0 | 0 | 0 | 0 | 0 | 0 | 0 | 0 | 1 | -1 | 0 | 0 | 0 | 0 | 0 | 0 | 0 | 0 | 0 | 0 | 0 | 0 | 0 | 0 | 0 | 0 | 0 | 0 | 0 | 0 | 0 | 0 | 0 | 0 | 0 | 0 | 0 | 0 | 0 | 0 | 0 | 0 | 0 | 0 | 0 | 0 | 0 | 0 | 0 | 0 | 0 | 0 | 0 | 0 | 0 | 0 | 0 | 0 | 0 | 0 | 0 | 0 | 0 | 0 | 0 | 0 | 0 | 0 | 0 | 0 |
| pfk\_R4\_AF\_c | 0 | 0 | 0 | 0 | 0 | 0 | 0 | 0 | 0 | 0 | 0 | 0 | 0 | 0 | 0 | 1 | -1 | 0 | 0 | 0 | 0 | 0 | 0 | 0 | 0 | 0 | 0 | 0 | 0 | 0 | 0 | 0 | 0 | 0 | 0 | 0 | 0 | 0 | 0 | 0 | 0 | 0 | 0 | 0 | 0 | 0 | 0 | 0 | 0 | 0 | 0 | 0 | 0 | 0 | 0 | 0 | 0 | 0 | 0 | 0 | 0 | 0 | 0 | 0 | 0 | 0 | 0 | 0 | 0 | 0 | 0 | 0 | 0 | 0 | 0 | 0 |
| pfk\_T0\_c | 0 | 0 | 0 | 0 | 0 | 0 | 0 | 0 | 0 | 0 | 0 | 0 | 0 | 0 | 0 | 0 | 0 | 0 | 0 | 0 | 0 | -1 | 0 | 0 | 0 | 1 | 0 | 0 | 0 | 0 | 0 | 0 | 0 | 0 | 0 | 0 | 0 | 0 | 0 | 0 | 0 | 0 | 0 | 0 | 0 | 0 | 0 | 0 | 0 | 0 | 0 | 0 | 0 | 0 | 0 | 0 | 0 | 0 | 0 | 0 | 0 | 0 | 0 | 0 | 0 | 0 | 0 | 0 | 0 | 0 | 0 | 0 | 0 | 0 | 0 | 0 |
| pfk\_T1\_c | 0 | 0 | 0 | 0 | 0 | 0 | 0 | 0 | 0 | 0 | 0 | 0 | 0 | 0 | 0 | 0 | 0 | 0 | 0 | 0 | 0 | 1 | -1 | 0 | 0 | 0 | 0 | 0 | 0 | 0 | 0 | 0 | 0 | 0 | 0 | 0 | 0 | 0 | 0 | 0 | 0 | 0 | 0 | 0 | 0 | 0 | 0 | 0 | 0 | 0 | 0 | 0 | 0 | 0 | 0 | 0 | 0 | 0 | 0 | 0 | 0 | 0 | 0 | 0 | 0 | 0 | 0 | 0 | 0 | 0 | 0 | 0 | 0 | 0 | 0 | 0 |
| pfk\_T2\_c | 0 | 0 | 0 | 0 | 0 | 0 | 0 | 0 | 0 | 0 | 0 | 0 | 0 | 0 | 0 | 0 | 0 | 0 | 0 | 0 | 0 | 0 | 1 | -1 | 0 | 0 | 0 | 0 | 0 | 0 | 0 | 0 | 0 | 0 | 0 | 0 | 0 | 0 | 0 | 0 | 0 | 0 | 0 | 0 | 0 | 0 | 0 | 0 | 0 | 0 | 0 | 0 | 0 | 0 | 0 | 0 | 0 | 0 | 0 | 0 | 0 | 0 | 0 | 0 | 0 | 0 | 0 | 0 | 0 | 0 | 0 | 0 | 0 | 0 | 0 | 0 |
| pfk\_T3\_c | 0 | 0 | 0 | 0 | 0 | 0 | 0 | 0 | 0 | 0 | 0 | 0 | 0 | 0 | 0 | 0 | 0 | 0 | 0 | 0 | 0 | 0 | 0 | 1 | -1 | 0 | 0 | 0 | 0 | 0 | 0 | 0 | 0 | 0 | 0 | 0 | 0 | 0 | 0 | 0 | 0 | 0 | 0 | 0 | 0 | 0 | 0 | 0 | 0 | 0 | 0 | 0 | 0 | 0 | 0 | 0 | 0 | 0 | 0 | 0 | 0 | 0 | 0 | 0 | 0 | 0 | 0 | 0 | 0 | 0 | 0 | 0 | 0 | 0 | 0 | 0 |
| pfk\_T4\_c | 0 | 0 | 0 | 0 | 0 | 0 | 0 | 0 | 0 | 0 | 0 | 0 | 0 | 0 | 0 | 0 | 0 | 0 | 0 | 0 | 0 | 0 | 0 | 0 | 1 | 0 | 0 | 0 | 0 | 0 | 0 | 0 | 0 | 0 | 0 | 0 | 0 | 0 | 0 | 0 | 0 | 0 | 0 | 0 | 0 | 0 | 0 | 0 | 0 | 0 | 0 | 0 | 0 | 0 | 0 | 0 | 0 | 0 | 0 | 0 | 0 | 0 | 0 | 0 | 0 | 0 | 0 | 0 | 0 | 0 | 0 | 0 | 0 | 0 | 0 | 0 |
| fdp\_c | 0 | 0 | 0 | 0 | 1 | 0 | 0 | 1 | 0 | 0 | 1 | 0 | 0 | 1 | 0 | 0 | 1 | 0 | 0 | 0 | 0 | 0 | 0 | 0 | 0 | 0 | -1 | 0 | 0 | 0 | 0 | 0 | 0 | 0 | 0 | 0 | 0 | 0 | 0 | 0 | 0 | 0 | 0 | 0 | 0 | 0 | 0 | 0 | 0 | 0 | 0 | 0 | 0 | 0 | 0 | 0 | 0 | 0 | 0 | 0 | 0 | 0 | 0 | 0 | 0 | 0 | 0 | 0 | 0 | 0 | 0 | 0 | 0 | 0 | 0 | 0 |
| dhap\_c | 0 | 0 | 0 | 0 | 0 | 0 | 0 | 0 | 0 | 0 | 0 | 0 | 0 | 0 | 0 | 0 | 0 | 0 | 0 | 0 | 0 | 0 | 0 | 0 | 0 | 0 | 1 | -1 | 0 | 0 | 0 | 0 | 0 | 0 | 0 | 0 | 0 | 0 | 0 | 0 | 0 | 0 | 0 | 0 | 0 | 0 | 0 | 0 | 0 | 0 | 0 | 0 | 0 | 0 | 0 | 0 | 0 | 0 | 0 | 0 | 0 | 0 | 0 | 0 | 0 | 0 | 0 | 0 | 0 | 0 | 0 | 0 | 0 | 0 | 0 | 0 |
| g3p\_c | 0 | 0 | 0 | 0 | 0 | 0 | 0 | 0 | 0 | 0 | 0 | 0 | 0 | 0 | 0 | 0 | 0 | 0 | 0 | 0 | 0 | 0 | 0 | 0 | 0 | 0 | 1 | 1 | -1 | 0 | 0 | 0 | 0 | 0 | 0 | 0 | 0 | 0 | 0 | 1 | 1 | -1 | 0 | 0 | 0 | 0 | 0 | 0 | 0 | 0 | 0 | 0 | 0 | 0 | 0 | 0 | 0 | 0 | 0 | 0 | 0 | 0 | 0 | 0 | 0 | 0 | 0 | 0 | 0 | 0 | 0 | 0 | 0 | 0 | 0 | 0 |
| \_13dpg\_c | 0 | 0 | 0 | 0 | 0 | 0 | 0 | 0 | 0 | 0 | 0 | 0 | 0 | 0 | 0 | 0 | 0 | 0 | 0 | 0 | 0 | 0 | 0 | 0 | 0 | 0 | 0 | 0 | 1 | -1 | 0 | 0 | 0 | 0 | 0 | 0 | 0 | 0 | 0 | 0 | 0 | 0 | 0 | 0 | 0 | 0 | 0 | 0 | 0 | 0 | 0 | 0 | -1 | 0 | 0 | 0 | 0 | 0 | 0 | 0 | 0 | 0 | 0 | 0 | 0 | 0 | 0 | 0 | 0 | 0 | 0 | 0 | 0 | 0 | 0 | 0 |
| \_3pg\_c | 0 | 0 | 0 | 0 | 0 | 0 | 0 | 0 | 0 | 0 | 0 | 0 | 0 | 0 | 0 | 0 | 0 | 0 | 0 | 0 | 0 | 0 | 0 | 0 | 0 | 0 | 0 | 0 | 0 | 1 | -1 | 0 | 0 | 0 | 0 | 0 | 0 | 0 | 0 | 0 | 0 | 0 | 0 | 0 | 0 | 0 | 0 | 0 | 0 | 0 | 0 | 0 | 0 | 1 | 0 | 0 | 0 | 0 | 0 | 0 | 0 | 0 | 0 | 0 | 0 | 0 | 0 | 0 | 0 | 0 | 0 | 0 | 0 | 0 | 0 | 0 |
| \_2pg\_c | 0 | 0 | 0 | 0 | 0 | 0 | 0 | 0 | 0 | 0 | 0 | 0 | 0 | 0 | 0 | 0 | 0 | 0 | 0 | 0 | 0 | 0 | 0 | 0 | 0 | 0 | 0 | 0 | 0 | 0 | 1 | -1 | 0 | 0 | 0 | 0 | 0 | 0 | 0 | 0 | 0 | 0 | 0 | 0 | 0 | 0 | 0 | 0 | 0 | 0 | 0 | 0 | 0 | 0 | 0 | 0 | 0 | 0 | 0 | 0 | 0 | 0 | 0 | 0 | 0 | 0 | 0 | 0 | 0 | 0 | 0 | 0 | 0 | 0 | 0 | 0 |
| pep\_c | 0 | 0 | 0 | 0 | 0 | 0 | 0 | 0 | 0 | 0 | 0 | 0 | 0 | 0 | 0 | 0 | 0 | 0 | 0 | 0 | 0 | 0 | 0 | 0 | 0 | 0 | 0 | 0 | 0 | 0 | 0 | 1 | -1 | 0 | 0 | 0 | 0 | 0 | 0 | 0 | 0 | 0 | 0 | 0 | 0 | 0 | 0 | 0 | 0 | 0 | 0 | 0 | 0 | 0 | 0 | 0 | 0 | 0 | 0 | 0 | 0 | 0 | 0 | 0 | 0 | 0 | 0 | 0 | 0 | 0 | 0 | 0 | 0 | 0 | 0 | 0 |
| pyr\_c | 0 | 0 | 0 | 0 | 0 | 0 | 0 | 0 | 0 | 0 | 0 | 0 | 0 | 0 | 0 | 0 | 0 | 0 | 0 | 0 | 0 | 0 | 0 | 0 | 0 | 0 | 0 | 0 | 0 | 0 | 0 | 0 | 1 | -1 | 0 | 0 | 0 | 0 | 0 | 0 | 0 | 0 | 0 | 0 | 0 | 0 | 0 | 0 | 0 | 0 | 0 | 0 | 0 | 0 | 0 | 0 | 0 | 0 | 0 | 0 | 0 | 0 | 0 | 0 | -1 | 0 | 0 | 0 | 0 | 0 | 0 | 0 | 0 | 0 | 0 | 0 |
| lac\_\_L\_c | 0 | 0 | 0 | 0 | 0 | 0 | 0 | 0 | 0 | 0 | 0 | 0 | 0 | 0 | 0 | 0 | 0 | 0 | 0 | 0 | 0 | 0 | 0 | 0 | 0 | 0 | 0 | 0 | 0 | 0 | 0 | 0 | 0 | 1 | 0 | 0 | 0 | 0 | 0 | 0 | 0 | 0 | 0 | 0 | 0 | 0 | 0 | 0 | 0 | 0 | 0 | 0 | 0 | 0 | 0 | 0 | 0 | 0 | 0 | 0 | 0 | 0 | 0 | 0 | 0 | -1 | 0 | 0 | 0 | 0 | 0 | 0 | 0 | 0 | 0 | 0 |
| \_6pgl\_c | 0 | 0 | 0 | 0 | 0 | 0 | 0 | 0 | 0 | 0 | 0 | 0 | 0 | 0 | 0 | 0 | 0 | 0 | 0 | 0 | 0 | 0 | 0 | 0 | 0 | 0 | 0 | 0 | 0 | 0 | 0 | 0 | 0 | 0 | 1 | -1 | 0 | 0 | 0 | 0 | 0 | 0 | 0 | 0 | 0 | 0 | 0 | 0 | 0 | 0 | 0 | 0 | 0 | 0 | 0 | 0 | 0 | 0 | 0 | 0 | 0 | 0 | 0 | 0 | 0 | 0 | 0 | 0 | 0 | 0 | 0 | 0 | 0 | 0 | 0 | 0 |
| \_6pgc\_c | 0 | 0 | 0 | 0 | 0 | 0 | 0 | 0 | 0 | 0 | 0 | 0 | 0 | 0 | 0 | 0 | 0 | 0 | 0 | 0 | 0 | 0 | 0 | 0 | 0 | 0 | 0 | 0 | 0 | 0 | 0 | 0 | 0 | 0 | 0 | 1 | -1 | 0 | 0 | 0 | 0 | 0 | 0 | 0 | 0 | 0 | 0 | 0 | 0 | 0 | 0 | 0 | 0 | 0 | 0 | 0 | 0 | 0 | 0 | 0 | 0 | 0 | 0 | 0 | 0 | 0 | 0 | 0 | 0 | 0 | 0 | 0 | 0 | 0 | 0 | 0 |
| ru5p\_\_D\_c | 0 | 0 | 0 | 0 | 0 | 0 | 0 | 0 | 0 | 0 | 0 | 0 | 0 | 0 | 0 | 0 | 0 | 0 | 0 | 0 | 0 | 0 | 0 | 0 | 0 | 0 | 0 | 0 | 0 | 0 | 0 | 0 | 0 | 0 | 0 | 0 | 1 | -1 | -1 | 0 | 0 | 0 | 0 | 0 | 0 | 0 | 0 | 0 | 0 | 0 | 0 | 0 | 0 | 0 | 0 | 0 | 0 | 0 | 0 | 0 | 0 | 0 | 0 | 0 | 0 | 0 | 0 | 0 | 0 | 0 | 0 | 0 | 0 | 0 | 0 | 0 |
| xu5p\_\_D\_c | 0 | 0 | 0 | 0 | 0 | 0 | 0 | 0 | 0 | 0 | 0 | 0 | 0 | 0 | 0 | 0 | 0 | 0 | 0 | 0 | 0 | 0 | 0 | 0 | 0 | 0 | 0 | 0 | 0 | 0 | 0 | 0 | 0 | 0 | 0 | 0 | 0 | 1 | 0 | -1 | -1 | 0 | 0 | 0 | 0 | 0 | 0 | 0 | 0 | 0 | 0 | 0 | 0 | 0 | 0 | 0 | 0 | 0 | 0 | 0 | 0 | 0 | 0 | 0 | 0 | 0 | 0 | 0 | 0 | 0 | 0 | 0 | 0 | 0 | 0 | 0 |
| r5p\_c | 0 | 0 | 0 | 0 | 0 | 0 | 0 | 0 | 0 | 0 | 0 | 0 | 0 | 0 | 0 | 0 | 0 | 0 | 0 | 0 | 0 | 0 | 0 | 0 | 0 | 0 | 0 | 0 | 0 | 0 | 0 | 0 | 0 | 0 | 0 | 0 | 0 | 0 | 1 | -1 | 0 | 0 | 0 | 0 | 0 | 0 | 0 | 0 | 1 | -1 | 0 | 0 | 0 | 0 | 0 | 0 | 0 | 0 | 0 | 0 | 0 | 0 | 0 | 0 | 0 | 0 | 0 | 0 | 0 | 0 | 0 | 0 | 0 | 0 | 0 | 0 |
| s7p\_c | 0 | 0 | 0 | 0 | 0 | 0 | 0 | 0 | 0 | 0 | 0 | 0 | 0 | 0 | 0 | 0 | 0 | 0 | 0 | 0 | 0 | 0 | 0 | 0 | 0 | 0 | 0 | 0 | 0 | 0 | 0 | 0 | 0 | 0 | 0 | 0 | 0 | 0 | 0 | 1 | 0 | -1 | 0 | 0 | 0 | 0 | 0 | 0 | 0 | 0 | 0 | 0 | 0 | 0 | 0 | 0 | 0 | 0 | 0 | 0 | 0 | 0 | 0 | 0 | 0 | 0 | 0 | 0 | 0 | 0 | 0 | 0 | 0 | 0 | 0 | 0 |
| e4p\_c | 0 | 0 | 0 | 0 | 0 | 0 | 0 | 0 | 0 | 0 | 0 | 0 | 0 | 0 | 0 | 0 | 0 | 0 | 0 | 0 | 0 | 0 | 0 | 0 | 0 | 0 | 0 | 0 | 0 | 0 | 0 | 0 | 0 | 0 | 0 | 0 | 0 | 0 | 0 | 0 | -1 | 1 | 0 | 0 | 0 | 0 | 0 | 0 | 0 | 0 | 0 | 0 | 0 | 0 | 0 | 0 | 0 | 0 | 0 | 0 | 0 | 0 | 0 | 0 | 0 | 0 | 0 | 0 | 0 | 0 | 0 | 0 | 0 | 0 | 0 | 0 |
| ade\_c | 0 | 0 | 0 | 0 | 0 | 0 | 0 | 0 | 0 | 0 | 0 | 0 | 0 | 0 | 0 | 0 | 0 | 0 | 0 | 0 | 0 | 0 | 0 | 0 | 0 | 0 | 0 | 0 | 0 | 0 | 0 | 0 | 0 | 0 | 0 | 0 | 0 | 0 | 0 | 0 | 0 | 0 | 0 | 0 | 0 | 0 | 0 | 0 | 0 | 0 | -1 | 0 | 0 | 0 | 0 | 0 | 0 | 0 | 0 | 0 | 0 | 0 | 0 | 0 | 0 | 0 | -1 | 0 | 0 | 0 | 0 | 0 | 0 | 0 | 0 | 0 |
| adn\_c | 0 | 0 | 0 | 0 | 0 | 0 | 0 | 0 | 0 | 0 | 0 | 0 | 0 | 0 | 0 | 0 | 0 | 0 | 0 | 0 | 0 | 0 | 0 | 0 | 0 | 0 | 0 | 0 | 0 | 0 | 0 | 0 | 0 | 0 | 0 | 0 | 0 | 0 | 0 | 0 | 0 | 0 | -1 | 1 | -1 | 0 | 0 | 0 | 0 | 0 | 0 | 0 | 0 | 0 | 0 | 0 | 0 | 0 | 0 | 0 | 0 | 0 | 0 | 0 | 0 | 0 | 0 | -1 | 0 | 0 | 0 | 0 | 0 | 0 | 0 | 0 |
| imp\_c | 0 | 0 | 0 | 0 | 0 | 0 | 0 | 0 | 0 | 0 | 0 | 0 | 0 | 0 | 0 | 0 | 0 | 0 | 0 | 0 | 0 | 0 | 0 | 0 | 0 | 0 | 0 | 0 | 0 | 0 | 0 | 0 | 0 | 0 | 0 | 0 | 0 | 0 | 0 | 0 | 0 | 0 | 0 | 0 | 0 | 1 | -1 | 0 | 0 | 0 | 0 | 0 | 0 | 0 | 0 | 0 | 0 | 0 | 0 | 0 | 0 | 0 | 0 | 0 | 0 | 0 | 0 | 0 | 0 | 0 | 0 | 0 | 0 | 0 | 0 | 0 |
| ins\_c | 0 | 0 | 0 | 0 | 0 | 0 | 0 | 0 | 0 | 0 | 0 | 0 | 0 | 0 | 0 | 0 | 0 | 0 | 0 | 0 | 0 | 0 | 0 | 0 | 0 | 0 | 0 | 0 | 0 | 0 | 0 | 0 | 0 | 0 | 0 | 0 | 0 | 0 | 0 | 0 | 0 | 0 | 0 | 0 | 1 | 0 | 1 | -1 | 0 | 0 | 0 | 0 | 0 | 0 | 0 | 0 | 0 | 0 | 0 | 0 | 0 | 0 | 0 | 0 | 0 | 0 | 0 | 0 | -1 | 0 | 0 | 0 | 0 | 0 | 0 | 0 |
| hxan\_c | 0 | 0 | 0 | 0 | 0 | 0 | 0 | 0 | 0 | 0 | 0 | 0 | 0 | 0 | 0 | 0 | 0 | 0 | 0 | 0 | 0 | 0 | 0 | 0 | 0 | 0 | 0 | 0 | 0 | 0 | 0 | 0 | 0 | 0 | 0 | 0 | 0 | 0 | 0 | 0 | 0 | 0 | 0 | 0 | 0 | 0 | 0 | 1 | 0 | 0 | 0 | 0 | 0 | 0 | 0 | 0 | 0 | 0 | 0 | 0 | 0 | 0 | 0 | 0 | 0 | 0 | 0 | 0 | 0 | -1 | 0 | 0 | 0 | 0 | 0 | 0 |
| r1p\_c | 0 | 0 | 0 | 0 | 0 | 0 | 0 | 0 | 0 | 0 | 0 | 0 | 0 | 0 | 0 | 0 | 0 | 0 | 0 | 0 | 0 | 0 | 0 | 0 | 0 | 0 | 0 | 0 | 0 | 0 | 0 | 0 | 0 | 0 | 0 | 0 | 0 | 0 | 0 | 0 | 0 | 0 | 0 | 0 | 0 | 0 | 0 | 1 | -1 | 0 | 0 | 0 | 0 | 0 | 0 | 0 | 0 | 0 | 0 | 0 | 0 | 0 | 0 | 0 | 0 | 0 | 0 | 0 | 0 | 0 | 0 | 0 | 0 | 0 | 0 | 0 |
| prpp\_c | 0 | 0 | 0 | 0 | 0 | 0 | 0 | 0 | 0 | 0 | 0 | 0 | 0 | 0 | 0 | 0 | 0 | 0 | 0 | 0 | 0 | 0 | 0 | 0 | 0 | 0 | 0 | 0 | 0 | 0 | 0 | 0 | 0 | 0 | 0 | 0 | 0 | 0 | 0 | 0 | 0 | 0 | 0 | 0 | 0 | 0 | 0 | 0 | 0 | 1 | -1 | 0 | 0 | 0 | 0 | 0 | 0 | 0 | 0 | 0 | 0 | 0 | 0 | 0 | 0 | 0 | 0 | 0 | 0 | 0 | 0 | 0 | 0 | 0 | 0 | 0 |
| \_23dpg\_c | 0 | 0 | 0 | 0 | 0 | 0 | 0 | 0 | 0 | 0 | 0 | 0 | 0 | 0 | 0 | 0 | 0 | 0 | 0 | 0 | 0 | 0 | 0 | 0 | 0 | 0 | 0 | 0 | 0 | 0 | 0 | 0 | 0 | 0 | 0 | 0 | 0 | 0 | 0 | 0 | 0 | 0 | 0 | 0 | 0 | 0 | 0 | 0 | 0 | 0 | 0 | 0 | 1 | -1 | -1 | 0 | 0 | 0 | 0 | 0 | 0 | 0 | 0 | 0 | 0 | 0 | 0 | 0 | 0 | 0 | 0 | 0 | 0 | 0 | 0 | 0 |
| hb\_c | 0 | 0 | 0 | 0 | 0 | 0 | 0 | 0 | 0 | 0 | 0 | 0 | 0 | 0 | 0 | 0 | 0 | 0 | 0 | 0 | 0 | 0 | 0 | 0 | 0 | 0 | 0 | 0 | 0 | 0 | 0 | 0 | 0 | 0 | 0 | 0 | 0 | 0 | 0 | 0 | 0 | 0 | 0 | 0 | 0 | 0 | 0 | 0 | 0 | 0 | 0 | 0 | 0 | 0 | -1 | -1 | 0 | 0 | 0 | 0 | 0 | 0 | 0 | 0 | 0 | 0 | 0 | 0 | 0 | 0 | 0 | 0 | 0 | 0 | 0 | 0 |
| hb\_1o2\_c | 0 | 0 | 0 | 0 | 0 | 0 | 0 | 0 | 0 | 0 | 0 | 0 | 0 | 0 | 0 | 0 | 0 | 0 | 0 | 0 | 0 | 0 | 0 | 0 | 0 | 0 | 0 | 0 | 0 | 0 | 0 | 0 | 0 | 0 | 0 | 0 | 0 | 0 | 0 | 0 | 0 | 0 | 0 | 0 | 0 | 0 | 0 | 0 | 0 | 0 | 0 | 0 | 0 | 0 | 0 | 1 | -1 | 0 | 0 | 0 | 0 | 0 | 0 | 0 | 0 | 0 | 0 | 0 | 0 | 0 | 0 | 0 | 0 | 0 | 0 | 0 |
| hb\_2o2\_c | 0 | 0 | 0 | 0 | 0 | 0 | 0 | 0 | 0 | 0 | 0 | 0 | 0 | 0 | 0 | 0 | 0 | 0 | 0 | 0 | 0 | 0 | 0 | 0 | 0 | 0 | 0 | 0 | 0 | 0 | 0 | 0 | 0 | 0 | 0 | 0 | 0 | 0 | 0 | 0 | 0 | 0 | 0 | 0 | 0 | 0 | 0 | 0 | 0 | 0 | 0 | 0 | 0 | 0 | 0 | 0 | 1 | -1 | 0 | 0 | 0 | 0 | 0 | 0 | 0 | 0 | 0 | 0 | 0 | 0 | 0 | 0 | 0 | 0 | 0 | 0 |
| hb\_3o2\_c | 0 | 0 | 0 | 0 | 0 | 0 | 0 | 0 | 0 | 0 | 0 | 0 | 0 | 0 | 0 | 0 | 0 | 0 | 0 | 0 | 0 | 0 | 0 | 0 | 0 | 0 | 0 | 0 | 0 | 0 | 0 | 0 | 0 | 0 | 0 | 0 | 0 | 0 | 0 | 0 | 0 | 0 | 0 | 0 | 0 | 0 | 0 | 0 | 0 | 0 | 0 | 0 | 0 | 0 | 0 | 0 | 0 | 1 | -1 | 0 | 0 | 0 | 0 | 0 | 0 | 0 | 0 | 0 | 0 | 0 | 0 | 0 | 0 | 0 | 0 | 0 |
| hb\_4o2\_c | 0 | 0 | 0 | 0 | 0 | 0 | 0 | 0 | 0 | 0 | 0 | 0 | 0 | 0 | 0 | 0 | 0 | 0 | 0 | 0 | 0 | 0 | 0 | 0 | 0 | 0 | 0 | 0 | 0 | 0 | 0 | 0 | 0 | 0 | 0 | 0 | 0 | 0 | 0 | 0 | 0 | 0 | 0 | 0 | 0 | 0 | 0 | 0 | 0 | 0 | 0 | 0 | 0 | 0 | 0 | 0 | 0 | 0 | 1 | 0 | 0 | 0 | 0 | 0 | 0 | 0 | 0 | 0 | 0 | 0 | 0 | 0 | 0 | 0 | 0 | 0 |
| dhb\_c | 0 | 0 | 0 | 0 | 0 | 0 | 0 | 0 | 0 | 0 | 0 | 0 | 0 | 0 | 0 | 0 | 0 | 0 | 0 | 0 | 0 | 0 | 0 | 0 | 0 | 0 | 0 | 0 | 0 | 0 | 0 | 0 | 0 | 0 | 0 | 0 | 0 | 0 | 0 | 0 | 0 | 0 | 0 | 0 | 0 | 0 | 0 | 0 | 0 | 0 | 0 | 0 | 0 | 0 | 1 | 0 | 0 | 0 | 0 | 0 | 0 | 0 | 0 | 0 | 0 | 0 | 0 | 0 | 0 | 0 | 0 | 0 | 0 | 0 | 0 | 0 |
| nad\_c | 0 | 0 | 0 | 0 | 0 | 0 | 0 | 0 | 0 | 0 | 0 | 0 | 0 | 0 | 0 | 0 | 0 | 0 | 0 | 0 | 0 | 0 | 0 | 0 | 0 | 0 | 0 | 0 | -1 | 0 | 0 | 0 | 0 | 1 | 0 | 0 | 0 | 0 | 0 | 0 | 0 | 0 | 0 | 0 | 0 | 0 | 0 | 0 | 0 | 0 | 0 | 0 | 0 | 0 | 0 | 0 | 0 | 0 | 0 | 0 | 1 | 0 | 0 | 0 | 0 | 0 | 0 | 0 | 0 | 0 | 0 | 0 | 0 | 0 | 0 | 0 |
| nadh\_c | 0 | 0 | 0 | 0 | 0 | 0 | 0 | 0 | 0 | 0 | 0 | 0 | 0 | 0 | 0 | 0 | 0 | 0 | 0 | 0 | 0 | 0 | 0 | 0 | 0 | 0 | 0 | 0 | 1 | 0 | 0 | 0 | 0 | -1 | 0 | 0 | 0 | 0 | 0 | 0 | 0 | 0 | 0 | 0 | 0 | 0 | 0 | 0 | 0 | 0 | 0 | 0 | 0 | 0 | 0 | 0 | 0 | 0 | 0 | 0 | -1 | 0 | 0 | 0 | 0 | 0 | 0 | 0 | 0 | 0 | 0 | 0 | 0 | 0 | 0 | 0 |
| amp\_c | 0 | 0 | 0 | 0 | 0 | 0 | 0 | 0 | 0 | 0 | 0 | 0 | 0 | 0 | 0 | 0 | 0 | -1 | -1 | -1 | -1 | 0 | 0 | 0 | 0 | 0 | 0 | 0 | 0 | 0 | 0 | 0 | 0 | 0 | 0 | 0 | 0 | 0 | 0 | 0 | 0 | 0 | 1 | -1 | 0 | -1 | 0 | 0 | 0 | 1 | 1 | 1 | 0 | 0 | 0 | 0 | 0 | 0 | 0 | 0 | 0 | 0 | 0 | 0 | 0 | 0 | 0 | 0 | 0 | 0 | 0 | 0 | 0 | 0 | 0 | 0 |
| adp\_c | 1 | 0 | 0 | 0 | 1 | 0 | 0 | 1 | 0 | 0 | 1 | 0 | 0 | 1 | 0 | 0 | 1 | 0 | 0 | 0 | 0 | 0 | 0 | 0 | 0 | 0 | 0 | 0 | 0 | -1 | 0 | 0 | -1 | 0 | 0 | 0 | 0 | 0 | 0 | 0 | 0 | 0 | 1 | 0 | 0 | 0 | 0 | 0 | 0 | 0 | 0 | -2 | 0 | 0 | 0 | 0 | 0 | 0 | 0 | 1 | 0 | 0 | 0 | 0 | 0 | 0 | 0 | 0 | 0 | 0 | 0 | 0 | 0 | 0 | 0 | 0 |
| atp\_c | -1 | 0 | -1 | 0 | 0 | -1 | 0 | 0 | -1 | 0 | 0 | -1 | 0 | 0 | -1 | 0 | 0 | 0 | 0 | 0 | 0 | -1 | -1 | -1 | -1 | 0 | 0 | 0 | 0 | 1 | 0 | 0 | 1 | 0 | 0 | 0 | 0 | 0 | 0 | 0 | 0 | 0 | -1 | 0 | 0 | 0 | 0 | 0 | 0 | -1 | 0 | 1 | 0 | 0 | 0 | 0 | 0 | 0 | 0 | -1 | 0 | 0 | 0 | 0 | 0 | 0 | 0 | 0 | 0 | 0 | 0 | 0 | 0 | 0 | 0 | 0 |
| nadp\_c | 0 | 0 | 0 | 0 | 0 | 0 | 0 | 0 | 0 | 0 | 0 | 0 | 0 | 0 | 0 | 0 | 0 | 0 | 0 | 0 | 0 | 0 | 0 | 0 | 0 | 0 | 0 | 0 | 0 | 0 | 0 | 0 | 0 | 0 | -1 | 0 | -1 | 0 | 0 | 0 | 0 | 0 | 0 | 0 | 0 | 0 | 0 | 0 | 0 | 0 | 0 | 0 | 0 | 0 | 0 | 0 | 0 | 0 | 0 | 0 | 0 | 1 | 0 | 0 | 0 | 0 | 0 | 0 | 0 | 0 | 0 | 0 | 0 | 0 | 0 | 0 |
| nadph\_c | 0 | 0 | 0 | 0 | 0 | 0 | 0 | 0 | 0 | 0 | 0 | 0 | 0 | 0 | 0 | 0 | 0 | 0 | 0 | 0 | 0 | 0 | 0 | 0 | 0 | 0 | 0 | 0 | 0 | 0 | 0 | 0 | 0 | 0 | 1 | 0 | 1 | 0 | 0 | 0 | 0 | 0 | 0 | 0 | 0 | 0 | 0 | 0 | 0 | 0 | 0 | 0 | 0 | 0 | 0 | 0 | 0 | 0 | 0 | 0 | 0 | -1 | 0 | 0 | 0 | 0 | 0 | 0 | 0 | 0 | 0 | 0 | 0 | 0 | 0 | 0 |
| gthrd\_c | 0 | 0 | 0 | 0 | 0 | 0 | 0 | 0 | 0 | 0 | 0 | 0 | 0 | 0 | 0 | 0 | 0 | 0 | 0 | 0 | 0 | 0 | 0 | 0 | 0 | 0 | 0 | 0 | 0 | 0 | 0 | 0 | 0 | 0 | 0 | 0 | 0 | 0 | 0 | 0 | 0 | 0 | 0 | 0 | 0 | 0 | 0 | 0 | 0 | 0 | 0 | 0 | 0 | 0 | 0 | 0 | 0 | 0 | 0 | 0 | 0 | 2 | -2 | 0 | 0 | 0 | 0 | 0 | 0 | 0 | 0 | 0 | 0 | 0 | 0 | 0 |
| gthox\_c | 0 | 0 | 0 | 0 | 0 | 0 | 0 | 0 | 0 | 0 | 0 | 0 | 0 | 0 | 0 | 0 | 0 | 0 | 0 | 0 | 0 | 0 | 0 | 0 | 0 | 0 | 0 | 0 | 0 | 0 | 0 | 0 | 0 | 0 | 0 | 0 | 0 | 0 | 0 | 0 | 0 | 0 | 0 | 0 | 0 | 0 | 0 | 0 | 0 | 0 | 0 | 0 | 0 | 0 | 0 | 0 | 0 | 0 | 0 | 0 | 0 | -1 | 1 | 0 | 0 | 0 | 0 | 0 | 0 | 0 | 0 | 0 | 0 | 0 | 0 | 0 |
| pi\_c | 0 | 0 | 0 | 0 | 0 | 0 | 0 | 0 | 0 | 0 | 0 | 0 | 0 | 0 | 0 | 0 | 0 | 0 | 0 | 0 | 0 | 0 | 0 | 0 | 0 | 0 | 0 | 0 | -1 | 0 | 0 | 0 | 0 | 0 | 0 | 0 | 0 | 0 | 0 | 0 | 0 | 0 | 0 | 1 | 0 | 0 | 1 | -1 | 0 | 0 | 2 | 0 | 0 | 1 | 0 | 0 | 0 | 0 | 0 | 1 | 0 | 0 | 0 | 0 | 0 | 0 | 0 | 0 | 0 | 0 | -1 | 0 | 0 | 0 | 0 | 0 |
| h\_c | 1 | 0 | 0 | 0 | 1 | 0 | 0 | 1 | 0 | 0 | 1 | 0 | 0 | 1 | 0 | 0 | 1 | 0 | 0 | 0 | 0 | 0 | 0 | 0 | 0 | 0 | 0 | 0 | 1 | 0 | 0 | 0 | -1 | -1 | 1 | 1 | 0 | 0 | 0 | 0 | 0 | 0 | 1 | 0 | 0 | 0 | 0 | 0 | 0 | 1 | 1 | 0 | 1 | 0 | 0 | 0 | 0 | 0 | 0 | 1 | 1 | -1 | 2 | 0 | 0 | 0 | 0 | 0 | 0 | 0 | 0 | -1 | 0 | 0 | 0 | 0 |
| h2o\_c | 0 | 0 | 0 | 0 | 0 | 0 | 0 | 0 | 0 | 0 | 0 | 0 | 0 | 0 | 0 | 0 | 0 | 0 | 0 | 0 | 0 | 0 | 0 | 0 | 0 | 0 | 0 | 0 | 0 | 0 | 0 | 1 | 0 | 0 | 0 | -1 | 0 | 0 | 0 | 0 | 0 | 0 | 0 | -1 | -1 | -1 | -1 | 0 | 0 | 0 | -1 | 0 | 0 | -1 | 0 | 0 | 0 | 0 | 0 | -1 | 0 | 0 | 0 | 0 | 0 | 0 | 0 | 0 | 0 | 0 | 0 | 0 | -1 | 0 | 0 | 0 |
| co2\_c | 0 | 0 | 0 | 0 | 0 | 0 | 0 | 0 | 0 | 0 | 0 | 0 | 0 | 0 | 0 | 0 | 0 | 0 | 0 | 0 | 0 | 0 | 0 | 0 | 0 | 0 | 0 | 0 | 0 | 0 | 0 | 0 | 0 | 0 | 0 | 0 | 1 | 0 | 0 | 0 | 0 | 0 | 0 | 0 | 0 | 0 | 0 | 0 | 0 | 0 | 0 | 0 | 0 | 0 | 0 | 0 | 0 | 0 | 0 | 0 | 0 | 0 | 0 | 0 | 0 | 0 | 0 | 0 | 0 | 0 | 0 | 0 | 0 | -1 | 0 | 0 |
| nh3\_c | 0 | 0 | 0 | 0 | 0 | 0 | 0 | 0 | 0 | 0 | 0 | 0 | 0 | 0 | 0 | 0 | 0 | 0 | 0 | 0 | 0 | 0 | 0 | 0 | 0 | 0 | 0 | 0 | 0 | 0 | 0 | 0 | 0 | 0 | 0 | 0 | 0 | 0 | 0 | 0 | 0 | 0 | 0 | 0 | 1 | 1 | 0 | 0 | 0 | 0 | 0 | 0 | 0 | 0 | 0 | 0 | 0 | 0 | 0 | 0 | 0 | 0 | 0 | 0 | 0 | 0 | 0 | 0 | 0 | 0 | 0 | 0 | 0 | 0 | -1 | 0 |
| o2\_c | 0 | 0 | 0 | 0 | 0 | 0 | 0 | 0 | 0 | 0 | 0 | 0 | 0 | 0 | 0 | 0 | 0 | 0 | 0 | 0 | 0 | 0 | 0 | 0 | 0 | 0 | 0 | 0 | 0 | 0 | 0 | 0 | 0 | 0 | 0 | 0 | 0 | 0 | 0 | 0 | 0 | 0 | 0 | 0 | 0 | 0 | 0 | 0 | 0 | 0 | 0 | 0 | 0 | 0 | 0 | -1 | -1 | -1 | -1 | 0 | 0 | 0 | 0 | 0 | 0 | 0 | 0 | 0 | 0 | 0 | 0 | 0 | 0 | 0 | 0 | -1 |

###### The stoichiometric matrix: PFK with RBC metabolic network¶

The properties of the stoichiometric matrix are shown in Table 14.5. All the reactions are elementally balanced except for the exchange reactions. The matrix has dimensions of 68 x 76 and a rank of 63 It thus has a 13 dimensional null space and a five dimensional left null space.

**Table 14.5:** The stoichiometric matrix for glycolysis, pentose phosphate pathway, AMP degradation and biosynthetic pathways, the Rapoport-Luebering (R.L.) shunt, the binding states of hemoglobin, and the PFK enzyme.

```
[29]:
```

```
# Define labels
metabolite_ids = [m.id for m in RBC_PFK.metabolites]
reaction_ids = [r.id for r in RBC_PFK.reactions]

pi_str = r"$\pi_{j}$"
rho_str = r"$\rho_{i}$"
chopsnq = ['C', 'H', 'O', 'P', 'N', 'S', 'q', '[PFK]', '[HB]', '[NAD]', '[NADP]']
time_inv_labels = ["$N_{\mathrm{tot}}$", "$NP_{\mathrm{tot}}$", "$G_{\mathrm{tot}}$",
                   "$Hb_{\mathrm{tot}}$", "$PFK_{\mathrm{tot}}$"]
path_labels = ["$p_1$", "$p_2$", "$p_3$", "$p_4$", "$p_5$",
               "$p_6$", "$p_7$", "$p_8$", "$p_9$", "$p_{10}$",
               "$p_{11}$", "$p_{12}$", "$p_{13}$"]

# Make table content from the stoichiometric matrix, elemental balancing of pathways
# participation number, and MinSpan pathways
S_matrix = RBC_PFK.update_S(array_type="dense", dtype=np.int64, update_model=False)
ES_matrix = RBC_PFK.get_elemental_charge_balancing(dtype=np.int64)
pi = np.count_nonzero(S_matrix, axis=0)
rho = np.count_nonzero(S_matrix, axis=1)
minspan_paths = np.array([
    [1, 1, 1, 1, 1, 0, 0, 0, 0, 0, 0, 0, 0, 0, 0, 0, 0, 0, 0, 0, 0, 0, 0, 0, 0, 0, 1, 1, 2, 2, 2, 2, 2, 2, 0, 0, 0, 0, 0, 0, 0, 0, 0, 0, 0, 0, 0, 0, 0, 0, 0, 0, 0, 0, 0, 0, 0, 0, 0, 2, 0, 0, 0, 1, 0, 2, 0, 0, 0, 0, 0, 2, 0, 0, 0, 0],
    [1, 1, 0, 0, 0, 1, 1, 1, 0, 0, 0, 0, 0, 0, 0, 0, 0, 0, 0, 0, 0, 0, 0, 0, 0, 0, 1, 1, 2, 2, 2, 2, 2, 2, 0, 0, 0, 0, 0, 0, 0, 0, 0, 0, 0, 0, 0, 0, 0, 0, 0, 0, 0, 0, 0, 0, 0, 0, 0, 2, 0, 0, 0, 1, 0, 2, 0, 0, 0, 0, 0, 2, 0, 0, 0, 0],
    [1, 1, 0, 0, 0, 0, 0, 0, 1, 1, 1, 0, 0, 0, 0, 0, 0, 0, 0, 0, 0, 0, 0, 0, 0, 0, 1, 1, 2, 2, 2, 2, 2, 2, 0, 0, 0, 0, 0, 0, 0, 0, 0, 0, 0, 0, 0, 0, 0, 0, 0, 0, 0, 0, 0, 0, 0, 0, 0, 2, 0, 0, 0, 1, 0, 2, 0, 0, 0, 0, 0, 2, 0, 0, 0, 0],
    [1, 1, 0, 0, 0, 0, 0, 0, 0, 0, 0, 1, 1, 1, 0, 0, 0, 0, 0, 0, 0, 0, 0, 0, 0, 0, 1, 1, 2, 2, 2, 2, 2, 2, 0, 0, 0, 0, 0, 0, 0, 0, 0, 0, 0, 0, 0, 0, 0, 0, 0, 0, 0, 0, 0, 0, 0, 0, 0, 2, 0, 0, 0, 1, 0, 2, 0, 0, 0, 0, 0, 2, 0, 0, 0, 0],
    [1, 1, 0, 0, 0, 0, 0, 0, 0, 0, 0, 0, 0, 0, 1, 1, 1, 0, 0, 0, 0, 0, 0, 0, 0, 0, 1, 1, 2, 2, 2, 2, 2, 2, 0, 0, 0, 0, 0, 0, 0, 0, 0, 0, 0, 0, 0, 0, 0, 0, 0, 0, 0, 0, 0, 0, 0, 0, 0, 2, 0, 0, 0, 1, 0, 2, 0, 0, 0, 0, 0, 2, 0, 0, 0, 0],
    [0, 0, 0, 0, 0, 0, 0, 0, 0, 0, 0, 0, 0, 0, 0, 0, 0, 0, 0, 0, 0, 0, 0, 0, 0, 0, 0, 0, 0, 0, 0, 0, 0, 1, 0, 0, 0, 0, 0, 0, 0, 0, 0, 0, 0, 0, 0, 0, 0, 0, 0, 0, 0, 0, 0, 0, 0, 0, 0, 0,-1, 0, 0, 0,-1, 1, 0, 0, 0, 0, 0,-2, 0, 0, 0, 0],
    [1,-2, 0, 0, 0, 0, 0, 0, 0, 0, 0, 0, 0, 0, 0, 0, 0, 0, 0, 0, 0, 0, 0, 0, 0, 0, 0, 0, 1, 1, 1, 1, 1, 1, 3, 3, 3, 2, 1, 1, 1, 1, 0, 0, 0, 0, 0, 0, 0, 0, 0, 0, 0, 0, 0, 0, 0, 0, 0, 1, 0, 6, 6, 1, 0, 1, 0, 0, 0, 0, 0,13,-3, 3, 0, 0],
    [1, 0, 0, 0, 0, 0, 0, 0, 0, 0, 0, 0, 0, 0, 0, 0, 0, 0, 0, 0, 0, 0, 0, 0, 0, 0, 0, 0, 0, 0, 0, 0, 0, 0, 1, 1, 1, 0, 1, 0, 0, 0, 0, 1, 0, 0, 0, 0, 0, 1, 1,-1, 0, 0, 0, 0, 0, 0, 0,-3, 0, 2, 2, 1, 0, 0,-1, 1, 0, 0, 0, 4, 0, 1, 0, 0],
    [1, 0, 0, 0, 0, 0, 0, 0, 0, 0, 0, 0, 0, 0, 0, 0, 0, 0, 0, 0, 0, 0, 0, 0, 0, 0, 0, 0, 0, 0, 0, 0, 0, 0, 1, 1, 1, 0, 1, 0, 0, 0, 0, 1, 1, 0, 0, 0, 0, 1, 1,-1, 0, 0, 0, 0, 0, 0, 0,-3, 0, 2, 2, 1, 0, 0,-1, 0, 1, 0, 0, 4,-1, 1, 1, 0],
    [1, 0, 0, 0, 0, 0, 0, 0, 0, 0, 0, 0, 0, 0, 0, 0, 0, 0, 0, 0, 0, 0, 0, 0, 0, 0, 0, 0, 0, 0, 0, 0, 0, 0, 1, 1, 1, 0, 1, 0, 0, 0, 0, 0, 0, 1, 1, 0, 0, 1, 1,-1, 0, 0, 0, 0, 0, 0, 0,-3, 0, 2, 2, 1, 0, 0,-1, 0, 1, 0, 0, 4,-1, 1, 1, 0],
    [0, 0, 0, 0, 0, 0, 0, 0, 0, 0, 0, 0, 0, 0, 0, 0, 0, 0, 0, 0, 0, 0, 0, 0, 0, 0, 0, 0, 0, 0, 0, 0, 0, 0, 0, 0, 0, 0, 0, 0, 0, 0, 0, 1, 1, 0, 0, 1, 1, 1, 1,-1, 0, 0, 0, 0, 0, 0, 0,-2, 0, 0, 0, 0, 0, 0,-1, 0, 0, 1, 0, 0,-1, 0, 1, 0],
    [0, 0, 0, 0, 0, 0, 0, 0, 0, 0, 0, 0, 0, 0, 0, 0, 0, 0, 0, 0, 0, 0, 0, 0, 0, 0, 0, 0, 0, 0, 0, 0, 0, 0, 0, 0, 0, 0, 0, 0, 0, 0, 1, 1, 0, 0, 0, 0, 0, 0, 0, 0, 0, 0, 0, 0, 0, 0, 0,-1, 0, 0, 0, 0, 0, 0, 0, 0, 0, 0, 0, 0, 0, 0, 0, 0],
    [1, 1, 0, 0, 0, 0, 0, 0, 1, 1, 1, 0, 0, 0, 0, 0, 0, 0, 0, 0, 0, 0, 0, 0, 0, 0, 1, 1, 2, 0, 2, 2, 2, 2, 0, 0, 0, 0, 0, 0, 0, 0, 0, 0, 0, 0, 0, 0, 0, 0, 0, 0, 2, 2, 0, 0, 0, 0, 0, 2, 0, 0, 0, 1, 0, 2, 0, 0, 0, 0, 0, 2, 0, 0, 0, 0]
])
table_14_5 = np.vstack((S_matrix, pi, ES_matrix, minspan_paths))

# Determine number of blank entries needed to be added to pad the table,
# Add connectivity number and time invariants to table content
blanks = [""]*(len(table_14_5) - len(metabolite_ids))
rho = np.concatenate((rho, blanks))

lns = np.zeros((5, 68), dtype=np.int64)
lns[0][53:55] = 1
lns[1][58:60] = 1
lns[2][60] = 1
lns[2][61] = 2
lns[3][47:53] = 1
lns[4][3:23] = 1

time_inv = np.array([np.concatenate([row, blanks]) for row in lns])
table_14_5 = np.vstack([table_14_5.T, rho, time_inv]).T

colors = {"glycolysis": "#ffffe6",    # Yellow
          "ppp": "#e6faff",           # Light blue
          "ampsn": "#d9fad2",
          "hemoglobin": "#ffcccc",
          "pfk": "#F4D03F",
          "cofactor": "#ffe6cc",
          "inorganic": "#fadffa",
          "chopsnq": "#99e6ff",       # Blue
          "pathways": "#b399ff",      # Purple
          "pi": "#99ffff",            # Cyan
          "rho": "#ff9999",           # Red
          "time_invs": "#ccff99",     # Green
          "blank": "#f2f2f2"}         # Grey
bg_color_str = "background-color: "
cofactor_mets = ["nad_c", "nadh_c",  "amp_c", "adp_c", "atp_c",
                 "nadp_c", "nadph_c", "gthrd_c", "gthox_c"]
exch_misc_rxns= ["SK_glc__D_c", "SK_pyr_c", "SK_lac__L_c", "SK_ade_c", "SK_adn_c",
                 "SK_ins_c", "SK_hxan_c", "ATPM", "DM_nadh", "GTHOr", "GSHR"]
inorganic_mets = ["pi_c", "h_c", "h2o_c", "co2_c", "nh3_c", "o2_c"]
inorganic_exch = ["SK_pi_c", "SK_h_c", "SK_h2o_c", "SK_co2_c", "SK_nh3_c", "SK_o2_c"]

def highlight_table(df, model):
    df = df.copy()
    condition = lambda mmodel, row, col, c1, c2:  (
        (col not in exch_misc_rxns + inorganic_exch) and (row not in cofactor_mets + inorganic_mets) and (
            (row in mmodel.metabolites and c1) or (col in mmodel.reactions or c2)))
    inorganic_condition = lambda row, col: (col in inorganic_exch or row in inorganic_mets)
    for i, row in enumerate(df.index):
        for j, col in enumerate(df.columns):
            if df.loc[row, col] == "":
                main_key = "blank"
            elif row in pi_str:
                main_key = "pi"
            elif row in chopsnq:
                main_key = "chopsnq"
            elif row in path_labels:
                main_key = "pathways"
            elif col in rho_str:
                main_key = "rho"
            elif col in time_inv_labels:
                main_key = "time_invs"
            elif condition(hemoglobin, row, col, row not in ["_13dpg_c", "_3pg_c"], False):
                main_key = "hemoglobin"
            elif condition(ampsn, row, col, row not in ["r5p_c"], col in ["ADK1"]):
                main_key = "ampsn"
            elif condition(ppp, row, col, row not in ["g6p_c", "f6p_c", "g3p_c"], False):
                main_key = "ppp"
            elif condition(glycolysis, row, col, True, False):
                main_key = "glycolysis"
            elif condition(PFK, row, col, True, False):
                main_key = "pfk"
            elif ((col in exch_misc_rxns or row in cofactor_mets) and not inorganic_condition(row, col)):
                main_key = "cofactor"
            elif inorganic_condition(row, col):
                main_key = "inorganic"
            else:
                continue
            df.loc[row, col] = bg_color_str + colors[main_key]
    return df

# Create index and column labels
index_labels = np.concatenate((metabolite_ids, [pi_str], chopsnq, path_labels))
column_labels = np.concatenate((reaction_ids, [rho_str], time_inv_labels))
# Create DataFrame
table_14_5 = pd.DataFrame(
    table_14_5, index=index_labels, columns=column_labels)
# Apply colors
table_14_5 = table_14_5.style.apply(
    highlight_table,  model=RBC_PFK, axis=None)
table_14_5
```

```
[29]:
```

|  | HEX1 | PGI | PFK\_R01 | PFK\_R02 | PFK\_R03 | PFK\_R11 | PFK\_R12 | PFK\_R13 | PFK\_R21 | PFK\_R22 | PFK\_R23 | PFK\_R31 | PFK\_R32 | PFK\_R33 | PFK\_R41 | PFK\_R42 | PFK\_R43 | PFK\_R10 | PFK\_R20 | PFK\_R30 | PFK\_R40 | PFK\_T1 | PFK\_T2 | PFK\_T3 | PFK\_T4 | PFK\_L | FBA | TPI | GAPD | PGK | PGM | ENO | PYK | LDH\_L | G6PDH2r | PGL | GND | RPE | RPI | TKT1 | TKT2 | TALA | ADNK1 | NTD7 | ADA | AMPDA | NTD11 | PUNP5 | PPM | PRPPS | ADPT | ADK1 | DPGM | DPGase | HBDPG | HBO1 | HBO2 | HBO3 | HBO4 | ATPM | DM\_nadh | GTHOr | GSHR | SK\_glc\_\_D\_c | SK\_pyr\_c | SK\_lac\_\_L\_c | SK\_ade\_c | SK\_adn\_c | SK\_ins\_c | SK\_hxan\_c | SK\_pi\_c | SK\_h\_c | SK\_h2o\_c | SK\_co2\_c | SK\_nh3\_c | SK\_o2\_c | $\rho\_{i}$ | $N\_{\mathrm{tot}}$ | $NP\_{\mathrm{tot}}$ | $G\_{\mathrm{tot}}$ | $Hb\_{\mathrm{tot}}$ | $PFK\_{\mathrm{tot}}$ |
| --- | --- | --- | --- | --- | --- | --- | --- | --- | --- | --- | --- | --- | --- | --- | --- | --- | --- | --- | --- | --- | --- | --- | --- | --- | --- | --- | --- | --- | --- | --- | --- | --- | --- | --- | --- | --- | --- | --- | --- | --- | --- | --- | --- | --- | --- | --- | --- | --- | --- | --- | --- | --- | --- | --- | --- | --- | --- | --- | --- | --- | --- | --- | --- | --- | --- | --- | --- | --- | --- | --- | --- | --- | --- | --- | --- | --- | --- | --- | --- | --- | --- | --- |
| glc\_\_D\_c | -1 | 0 | 0 | 0 | 0 | 0 | 0 | 0 | 0 | 0 | 0 | 0 | 0 | 0 | 0 | 0 | 0 | 0 | 0 | 0 | 0 | 0 | 0 | 0 | 0 | 0 | 0 | 0 | 0 | 0 | 0 | 0 | 0 | 0 | 0 | 0 | 0 | 0 | 0 | 0 | 0 | 0 | 0 | 0 | 0 | 0 | 0 | 0 | 0 | 0 | 0 | 0 | 0 | 0 | 0 | 0 | 0 | 0 | 0 | 0 | 0 | 0 | 0 | 1 | 0 | 0 | 0 | 0 | 0 | 0 | 0 | 0 | 0 | 0 | 0 | 0 | 2 | 0 | 0 | 0 | 0 | 0 |
| g6p\_c | 1 | -1 | 0 | 0 | 0 | 0 | 0 | 0 | 0 | 0 | 0 | 0 | 0 | 0 | 0 | 0 | 0 | 0 | 0 | 0 | 0 | 0 | 0 | 0 | 0 | 0 | 0 | 0 | 0 | 0 | 0 | 0 | 0 | 0 | -1 | 0 | 0 | 0 | 0 | 0 | 0 | 0 | 0 | 0 | 0 | 0 | 0 | 0 | 0 | 0 | 0 | 0 | 0 | 0 | 0 | 0 | 0 | 0 | 0 | 0 | 0 | 0 | 0 | 0 | 0 | 0 | 0 | 0 | 0 | 0 | 0 | 0 | 0 | 0 | 0 | 0 | 3 | 0 | 0 | 0 | 0 | 0 |
| f6p\_c | 0 | 1 | 0 | -1 | 0 | 0 | -1 | 0 | 0 | -1 | 0 | 0 | -1 | 0 | 0 | -1 | 0 | 0 | 0 | 0 | 0 | 0 | 0 | 0 | 0 | 0 | 0 | 0 | 0 | 0 | 0 | 0 | 0 | 0 | 0 | 0 | 0 | 0 | 0 | 0 | 1 | 1 | 0 | 0 | 0 | 0 | 0 | 0 | 0 | 0 | 0 | 0 | 0 | 0 | 0 | 0 | 0 | 0 | 0 | 0 | 0 | 0 | 0 | 0 | 0 | 0 | 0 | 0 | 0 | 0 | 0 | 0 | 0 | 0 | 0 | 0 | 8 | 0 | 0 | 0 | 0 | 0 |
| pfk\_R0\_c | 0 | 0 | -1 | 0 | 1 | 0 | 0 | 0 | 0 | 0 | 0 | 0 | 0 | 0 | 0 | 0 | 0 | -1 | 0 | 0 | 0 | 0 | 0 | 0 | 0 | -1 | 0 | 0 | 0 | 0 | 0 | 0 | 0 | 0 | 0 | 0 | 0 | 0 | 0 | 0 | 0 | 0 | 0 | 0 | 0 | 0 | 0 | 0 | 0 | 0 | 0 | 0 | 0 | 0 | 0 | 0 | 0 | 0 | 0 | 0 | 0 | 0 | 0 | 0 | 0 | 0 | 0 | 0 | 0 | 0 | 0 | 0 | 0 | 0 | 0 | 0 | 4 | 0 | 0 | 0 | 0 | 1 |
| pfk\_R0\_A\_c | 0 | 0 | 1 | -1 | 0 | 0 | 0 | 0 | 0 | 0 | 0 | 0 | 0 | 0 | 0 | 0 | 0 | 0 | 0 | 0 | 0 | 0 | 0 | 0 | 0 | 0 | 0 | 0 | 0 | 0 | 0 | 0 | 0 | 0 | 0 | 0 | 0 | 0 | 0 | 0 | 0 | 0 | 0 | 0 | 0 | 0 | 0 | 0 | 0 | 0 | 0 | 0 | 0 | 0 | 0 | 0 | 0 | 0 | 0 | 0 | 0 | 0 | 0 | 0 | 0 | 0 | 0 | 0 | 0 | 0 | 0 | 0 | 0 | 0 | 0 | 0 | 2 | 0 | 0 | 0 | 0 | 1 |
| pfk\_R0\_AF\_c | 0 | 0 | 0 | 1 | -1 | 0 | 0 | 0 | 0 | 0 | 0 | 0 | 0 | 0 | 0 | 0 | 0 | 0 | 0 | 0 | 0 | 0 | 0 | 0 | 0 | 0 | 0 | 0 | 0 | 0 | 0 | 0 | 0 | 0 | 0 | 0 | 0 | 0 | 0 | 0 | 0 | 0 | 0 | 0 | 0 | 0 | 0 | 0 | 0 | 0 | 0 | 0 | 0 | 0 | 0 | 0 | 0 | 0 | 0 | 0 | 0 | 0 | 0 | 0 | 0 | 0 | 0 | 0 | 0 | 0 | 0 | 0 | 0 | 0 | 0 | 0 | 2 | 0 | 0 | 0 | 0 | 1 |
| pfk\_R1\_c | 0 | 0 | 0 | 0 | 0 | -1 | 0 | 1 | 0 | 0 | 0 | 0 | 0 | 0 | 0 | 0 | 0 | 1 | -1 | 0 | 0 | 0 | 0 | 0 | 0 | 0 | 0 | 0 | 0 | 0 | 0 | 0 | 0 | 0 | 0 | 0 | 0 | 0 | 0 | 0 | 0 | 0 | 0 | 0 | 0 | 0 | 0 | 0 | 0 | 0 | 0 | 0 | 0 | 0 | 0 | 0 | 0 | 0 | 0 | 0 | 0 | 0 | 0 | 0 | 0 | 0 | 0 | 0 | 0 | 0 | 0 | 0 | 0 | 0 | 0 | 0 | 4 | 0 | 0 | 0 | 0 | 1 |
| pfk\_R1\_A\_c | 0 | 0 | 0 | 0 | 0 | 1 | -1 | 0 | 0 | 0 | 0 | 0 | 0 | 0 | 0 | 0 | 0 | 0 | 0 | 0 | 0 | 0 | 0 | 0 | 0 | 0 | 0 | 0 | 0 | 0 | 0 | 0 | 0 | 0 | 0 | 0 | 0 | 0 | 0 | 0 | 0 | 0 | 0 | 0 | 0 | 0 | 0 | 0 | 0 | 0 | 0 | 0 | 0 | 0 | 0 | 0 | 0 | 0 | 0 | 0 | 0 | 0 | 0 | 0 | 0 | 0 | 0 | 0 | 0 | 0 | 0 | 0 | 0 | 0 | 0 | 0 | 2 | 0 | 0 | 0 | 0 | 1 |
| pfk\_R1\_AF\_c | 0 | 0 | 0 | 0 | 0 | 0 | 1 | -1 | 0 | 0 | 0 | 0 | 0 | 0 | 0 | 0 | 0 | 0 | 0 | 0 | 0 | 0 | 0 | 0 | 0 | 0 | 0 | 0 | 0 | 0 | 0 | 0 | 0 | 0 | 0 | 0 | 0 | 0 | 0 | 0 | 0 | 0 | 0 | 0 | 0 | 0 | 0 | 0 | 0 | 0 | 0 | 0 | 0 | 0 | 0 | 0 | 0 | 0 | 0 | 0 | 0 | 0 | 0 | 0 | 0 | 0 | 0 | 0 | 0 | 0 | 0 | 0 | 0 | 0 | 0 | 0 | 2 | 0 | 0 | 0 | 0 | 1 |
| pfk\_R2\_c | 0 | 0 | 0 | 0 | 0 | 0 | 0 | 0 | -1 | 0 | 1 | 0 | 0 | 0 | 0 | 0 | 0 | 0 | 1 | -1 | 0 | 0 | 0 | 0 | 0 | 0 | 0 | 0 | 0 | 0 | 0 | 0 | 0 | 0 | 0 | 0 | 0 | 0 | 0 | 0 | 0 | 0 | 0 | 0 | 0 | 0 | 0 | 0 | 0 | 0 | 0 | 0 | 0 | 0 | 0 | 0 | 0 | 0 | 0 | 0 | 0 | 0 | 0 | 0 | 0 | 0 | 0 | 0 | 0 | 0 | 0 | 0 | 0 | 0 | 0 | 0 | 4 | 0 | 0 | 0 | 0 | 1 |
| pfk\_R2\_A\_c | 0 | 0 | 0 | 0 | 0 | 0 | 0 | 0 | 1 | -1 | 0 | 0 | 0 | 0 | 0 | 0 | 0 | 0 | 0 | 0 | 0 | 0 | 0 | 0 | 0 | 0 | 0 | 0 | 0 | 0 | 0 | 0 | 0 | 0 | 0 | 0 | 0 | 0 | 0 | 0 | 0 | 0 | 0 | 0 | 0 | 0 | 0 | 0 | 0 | 0 | 0 | 0 | 0 | 0 | 0 | 0 | 0 | 0 | 0 | 0 | 0 | 0 | 0 | 0 | 0 | 0 | 0 | 0 | 0 | 0 | 0 | 0 | 0 | 0 | 0 | 0 | 2 | 0 | 0 | 0 | 0 | 1 |
| pfk\_R2\_AF\_c | 0 | 0 | 0 | 0 | 0 | 0 | 0 | 0 | 0 | 1 | -1 | 0 | 0 | 0 | 0 | 0 | 0 | 0 | 0 | 0 | 0 | 0 | 0 | 0 | 0 | 0 | 0 | 0 | 0 | 0 | 0 | 0 | 0 | 0 | 0 | 0 | 0 | 0 | 0 | 0 | 0 | 0 | 0 | 0 | 0 | 0 | 0 | 0 | 0 | 0 | 0 | 0 | 0 | 0 | 0 | 0 | 0 | 0 | 0 | 0 | 0 | 0 | 0 | 0 | 0 | 0 | 0 | 0 | 0 | 0 | 0 | 0 | 0 | 0 | 0 | 0 | 2 | 0 | 0 | 0 | 0 | 1 |
| pfk\_R3\_c | 0 | 0 | 0 | 0 | 0 | 0 | 0 | 0 | 0 | 0 | 0 | -1 | 0 | 1 | 0 | 0 | 0 | 0 | 0 | 1 | -1 | 0 | 0 | 0 | 0 | 0 | 0 | 0 | 0 | 0 | 0 | 0 | 0 | 0 | 0 | 0 | 0 | 0 | 0 | 0 | 0 | 0 | 0 | 0 | 0 | 0 | 0 | 0 | 0 | 0 | 0 | 0 | 0 | 0 | 0 | 0 | 0 | 0 | 0 | 0 | 0 | 0 | 0 | 0 | 0 | 0 | 0 | 0 | 0 | 0 | 0 | 0 | 0 | 0 | 0 | 0 | 4 | 0 | 0 | 0 | 0 | 1 |
| pfk\_R3\_A\_c | 0 | 0 | 0 | 0 | 0 | 0 | 0 | 0 | 0 | 0 | 0 | 1 | -1 | 0 | 0 | 0 | 0 | 0 | 0 | 0 | 0 | 0 | 0 | 0 | 0 | 0 | 0 | 0 | 0 | 0 | 0 | 0 | 0 | 0 | 0 | 0 | 0 | 0 | 0 | 0 | 0 | 0 | 0 | 0 | 0 | 0 | 0 | 0 | 0 | 0 | 0 | 0 | 0 | 0 | 0 | 0 | 0 | 0 | 0 | 0 | 0 | 0 | 0 | 0 | 0 | 0 | 0 | 0 | 0 | 0 | 0 | 0 | 0 | 0 | 0 | 0 | 2 | 0 | 0 | 0 | 0 | 1 |
| pfk\_R3\_AF\_c | 0 | 0 | 0 | 0 | 0 | 0 | 0 | 0 | 0 | 0 | 0 | 0 | 1 | -1 | 0 | 0 | 0 | 0 | 0 | 0 | 0 | 0 | 0 | 0 | 0 | 0 | 0 | 0 | 0 | 0 | 0 | 0 | 0 | 0 | 0 | 0 | 0 | 0 | 0 | 0 | 0 | 0 | 0 | 0 | 0 | 0 | 0 | 0 | 0 | 0 | 0 | 0 | 0 | 0 | 0 | 0 | 0 | 0 | 0 | 0 | 0 | 0 | 0 | 0 | 0 | 0 | 0 | 0 | 0 | 0 | 0 | 0 | 0 | 0 | 0 | 0 | 2 | 0 | 0 | 0 | 0 | 1 |
| pfk\_R4\_c | 0 | 0 | 0 | 0 | 0 | 0 | 0 | 0 | 0 | 0 | 0 | 0 | 0 | 0 | -1 | 0 | 1 | 0 | 0 | 0 | 1 | 0 | 0 | 0 | 0 | 0 | 0 | 0 | 0 | 0 | 0 | 0 | 0 | 0 | 0 | 0 | 0 | 0 | 0 | 0 | 0 | 0 | 0 | 0 | 0 | 0 | 0 | 0 | 0 | 0 | 0 | 0 | 0 | 0 | 0 | 0 | 0 | 0 | 0 | 0 | 0 | 0 | 0 | 0 | 0 | 0 | 0 | 0 | 0 | 0 | 0 | 0 | 0 | 0 | 0 | 0 | 3 | 0 | 0 | 0 | 0 | 1 |
| pfk\_R4\_A\_c | 0 | 0 | 0 | 0 | 0 | 0 | 0 | 0 | 0 | 0 | 0 | 0 | 0 | 0 | 1 | -1 | 0 | 0 | 0 | 0 | 0 | 0 | 0 | 0 | 0 | 0 | 0 | 0 | 0 | 0 | 0 | 0 | 0 | 0 | 0 | 0 | 0 | 0 | 0 | 0 | 0 | 0 | 0 | 0 | 0 | 0 | 0 | 0 | 0 | 0 | 0 | 0 | 0 | 0 | 0 | 0 | 0 | 0 | 0 | 0 | 0 | 0 | 0 | 0 | 0 | 0 | 0 | 0 | 0 | 0 | 0 | 0 | 0 | 0 | 0 | 0 | 2 | 0 | 0 | 0 | 0 | 1 |
| pfk\_R4\_AF\_c | 0 | 0 | 0 | 0 | 0 | 0 | 0 | 0 | 0 | 0 | 0 | 0 | 0 | 0 | 0 | 1 | -1 | 0 | 0 | 0 | 0 | 0 | 0 | 0 | 0 | 0 | 0 | 0 | 0 | 0 | 0 | 0 | 0 | 0 | 0 | 0 | 0 | 0 | 0 | 0 | 0 | 0 | 0 | 0 | 0 | 0 | 0 | 0 | 0 | 0 | 0 | 0 | 0 | 0 | 0 | 0 | 0 | 0 | 0 | 0 | 0 | 0 | 0 | 0 | 0 | 0 | 0 | 0 | 0 | 0 | 0 | 0 | 0 | 0 | 0 | 0 | 2 | 0 | 0 | 0 | 0 | 1 |
| pfk\_T0\_c | 0 | 0 | 0 | 0 | 0 | 0 | 0 | 0 | 0 | 0 | 0 | 0 | 0 | 0 | 0 | 0 | 0 | 0 | 0 | 0 | 0 | -1 | 0 | 0 | 0 | 1 | 0 | 0 | 0 | 0 | 0 | 0 | 0 | 0 | 0 | 0 | 0 | 0 | 0 | 0 | 0 | 0 | 0 | 0 | 0 | 0 | 0 | 0 | 0 | 0 | 0 | 0 | 0 | 0 | 0 | 0 | 0 | 0 | 0 | 0 | 0 | 0 | 0 | 0 | 0 | 0 | 0 | 0 | 0 | 0 | 0 | 0 | 0 | 0 | 0 | 0 | 2 | 0 | 0 | 0 | 0 | 1 |
| pfk\_T1\_c | 0 | 0 | 0 | 0 | 0 | 0 | 0 | 0 | 0 | 0 | 0 | 0 | 0 | 0 | 0 | 0 | 0 | 0 | 0 | 0 | 0 | 1 | -1 | 0 | 0 | 0 | 0 | 0 | 0 | 0 | 0 | 0 | 0 | 0 | 0 | 0 | 0 | 0 | 0 | 0 | 0 | 0 | 0 | 0 | 0 | 0 | 0 | 0 | 0 | 0 | 0 | 0 | 0 | 0 | 0 | 0 | 0 | 0 | 0 | 0 | 0 | 0 | 0 | 0 | 0 | 0 | 0 | 0 | 0 | 0 | 0 | 0 | 0 | 0 | 0 | 0 | 2 | 0 | 0 | 0 | 0 | 1 |
| pfk\_T2\_c | 0 | 0 | 0 | 0 | 0 | 0 | 0 | 0 | 0 | 0 | 0 | 0 | 0 | 0 | 0 | 0 | 0 | 0 | 0 | 0 | 0 | 0 | 1 | -1 | 0 | 0 | 0 | 0 | 0 | 0 | 0 | 0 | 0 | 0 | 0 | 0 | 0 | 0 | 0 | 0 | 0 | 0 | 0 | 0 | 0 | 0 | 0 | 0 | 0 | 0 | 0 | 0 | 0 | 0 | 0 | 0 | 0 | 0 | 0 | 0 | 0 | 0 | 0 | 0 | 0 | 0 | 0 | 0 | 0 | 0 | 0 | 0 | 0 | 0 | 0 | 0 | 2 | 0 | 0 | 0 | 0 | 1 |
| pfk\_T3\_c | 0 | 0 | 0 | 0 | 0 | 0 | 0 | 0 | 0 | 0 | 0 | 0 | 0 | 0 | 0 | 0 | 0 | 0 | 0 | 0 | 0 | 0 | 0 | 1 | -1 | 0 | 0 | 0 | 0 | 0 | 0 | 0 | 0 | 0 | 0 | 0 | 0 | 0 | 0 | 0 | 0 | 0 | 0 | 0 | 0 | 0 | 0 | 0 | 0 | 0 | 0 | 0 | 0 | 0 | 0 | 0 | 0 | 0 | 0 | 0 | 0 | 0 | 0 | 0 | 0 | 0 | 0 | 0 | 0 | 0 | 0 | 0 | 0 | 0 | 0 | 0 | 2 | 0 | 0 | 0 | 0 | 1 |
| pfk\_T4\_c | 0 | 0 | 0 | 0 | 0 | 0 | 0 | 0 | 0 | 0 | 0 | 0 | 0 | 0 | 0 | 0 | 0 | 0 | 0 | 0 | 0 | 0 | 0 | 0 | 1 | 0 | 0 | 0 | 0 | 0 | 0 | 0 | 0 | 0 | 0 | 0 | 0 | 0 | 0 | 0 | 0 | 0 | 0 | 0 | 0 | 0 | 0 | 0 | 0 | 0 | 0 | 0 | 0 | 0 | 0 | 0 | 0 | 0 | 0 | 0 | 0 | 0 | 0 | 0 | 0 | 0 | 0 | 0 | 0 | 0 | 0 | 0 | 0 | 0 | 0 | 0 | 1 | 0 | 0 | 0 | 0 | 1 |
| fdp\_c | 0 | 0 | 0 | 0 | 1 | 0 | 0 | 1 | 0 | 0 | 1 | 0 | 0 | 1 | 0 | 0 | 1 | 0 | 0 | 0 | 0 | 0 | 0 | 0 | 0 | 0 | -1 | 0 | 0 | 0 | 0 | 0 | 0 | 0 | 0 | 0 | 0 | 0 | 0 | 0 | 0 | 0 | 0 | 0 | 0 | 0 | 0 | 0 | 0 | 0 | 0 | 0 | 0 | 0 | 0 | 0 | 0 | 0 | 0 | 0 | 0 | 0 | 0 | 0 | 0 | 0 | 0 | 0 | 0 | 0 | 0 | 0 | 0 | 0 | 0 | 0 | 6 | 0 | 0 | 0 | 0 | 0 |
| dhap\_c | 0 | 0 | 0 | 0 | 0 | 0 | 0 | 0 | 0 | 0 | 0 | 0 | 0 | 0 | 0 | 0 | 0 | 0 | 0 | 0 | 0 | 0 | 0 | 0 | 0 | 0 | 1 | -1 | 0 | 0 | 0 | 0 | 0 | 0 | 0 | 0 | 0 | 0 | 0 | 0 | 0 | 0 | 0 | 0 | 0 | 0 | 0 | 0 | 0 | 0 | 0 | 0 | 0 | 0 | 0 | 0 | 0 | 0 | 0 | 0 | 0 | 0 | 0 | 0 | 0 | 0 | 0 | 0 | 0 | 0 | 0 | 0 | 0 | 0 | 0 | 0 | 2 | 0 | 0 | 0 | 0 | 0 |
| g3p\_c | 0 | 0 | 0 | 0 | 0 | 0 | 0 | 0 | 0 | 0 | 0 | 0 | 0 | 0 | 0 | 0 | 0 | 0 | 0 | 0 | 0 | 0 | 0 | 0 | 0 | 0 | 1 | 1 | -1 | 0 | 0 | 0 | 0 | 0 | 0 | 0 | 0 | 0 | 0 | 1 | 1 | -1 | 0 | 0 | 0 | 0 | 0 | 0 | 0 | 0 | 0 | 0 | 0 | 0 | 0 | 0 | 0 | 0 | 0 | 0 | 0 | 0 | 0 | 0 | 0 | 0 | 0 | 0 | 0 | 0 | 0 | 0 | 0 | 0 | 0 | 0 | 6 | 0 | 0 | 0 | 0 | 0 |
| \_13dpg\_c | 0 | 0 | 0 | 0 | 0 | 0 | 0 | 0 | 0 | 0 | 0 | 0 | 0 | 0 | 0 | 0 | 0 | 0 | 0 | 0 | 0 | 0 | 0 | 0 | 0 | 0 | 0 | 0 | 1 | -1 | 0 | 0 | 0 | 0 | 0 | 0 | 0 | 0 | 0 | 0 | 0 | 0 | 0 | 0 | 0 | 0 | 0 | 0 | 0 | 0 | 0 | 0 | -1 | 0 | 0 | 0 | 0 | 0 | 0 | 0 | 0 | 0 | 0 | 0 | 0 | 0 | 0 | 0 | 0 | 0 | 0 | 0 | 0 | 0 | 0 | 0 | 3 | 0 | 0 | 0 | 0 | 0 |
| \_3pg\_c | 0 | 0 | 0 | 0 | 0 | 0 | 0 | 0 | 0 | 0 | 0 | 0 | 0 | 0 | 0 | 0 | 0 | 0 | 0 | 0 | 0 | 0 | 0 | 0 | 0 | 0 | 0 | 0 | 0 | 1 | -1 | 0 | 0 | 0 | 0 | 0 | 0 | 0 | 0 | 0 | 0 | 0 | 0 | 0 | 0 | 0 | 0 | 0 | 0 | 0 | 0 | 0 | 0 | 1 | 0 | 0 | 0 | 0 | 0 | 0 | 0 | 0 | 0 | 0 | 0 | 0 | 0 | 0 | 0 | 0 | 0 | 0 | 0 | 0 | 0 | 0 | 3 | 0 | 0 | 0 | 0 | 0 |
| \_2pg\_c | 0 | 0 | 0 | 0 | 0 | 0 | 0 | 0 | 0 | 0 | 0 | 0 | 0 | 0 | 0 | 0 | 0 | 0 | 0 | 0 | 0 | 0 | 0 | 0 | 0 | 0 | 0 | 0 | 0 | 0 | 1 | -1 | 0 | 0 | 0 | 0 | 0 | 0 | 0 | 0 | 0 | 0 | 0 | 0 | 0 | 0 | 0 | 0 | 0 | 0 | 0 | 0 | 0 | 0 | 0 | 0 | 0 | 0 | 0 | 0 | 0 | 0 | 0 | 0 | 0 | 0 | 0 | 0 | 0 | 0 | 0 | 0 | 0 | 0 | 0 | 0 | 2 | 0 | 0 | 0 | 0 | 0 |
| pep\_c | 0 | 0 | 0 | 0 | 0 | 0 | 0 | 0 | 0 | 0 | 0 | 0 | 0 | 0 | 0 | 0 | 0 | 0 | 0 | 0 | 0 | 0 | 0 | 0 | 0 | 0 | 0 | 0 | 0 | 0 | 0 | 1 | -1 | 0 | 0 | 0 | 0 | 0 | 0 | 0 | 0 | 0 | 0 | 0 | 0 | 0 | 0 | 0 | 0 | 0 | 0 | 0 | 0 | 0 | 0 | 0 | 0 | 0 | 0 | 0 | 0 | 0 | 0 | 0 | 0 | 0 | 0 | 0 | 0 | 0 | 0 | 0 | 0 | 0 | 0 | 0 | 2 | 0 | 0 | 0 | 0 | 0 |
| pyr\_c | 0 | 0 | 0 | 0 | 0 | 0 | 0 | 0 | 0 | 0 | 0 | 0 | 0 | 0 | 0 | 0 | 0 | 0 | 0 | 0 | 0 | 0 | 0 | 0 | 0 | 0 | 0 | 0 | 0 | 0 | 0 | 0 | 1 | -1 | 0 | 0 | 0 | 0 | 0 | 0 | 0 | 0 | 0 | 0 | 0 | 0 | 0 | 0 | 0 | 0 | 0 | 0 | 0 | 0 | 0 | 0 | 0 | 0 | 0 | 0 | 0 | 0 | 0 | 0 | -1 | 0 | 0 | 0 | 0 | 0 | 0 | 0 | 0 | 0 | 0 | 0 | 3 | 0 | 0 | 0 | 0 | 0 |
| lac\_\_L\_c | 0 | 0 | 0 | 0 | 0 | 0 | 0 | 0 | 0 | 0 | 0 | 0 | 0 | 0 | 0 | 0 | 0 | 0 | 0 | 0 | 0 | 0 | 0 | 0 | 0 | 0 | 0 | 0 | 0 | 0 | 0 | 0 | 0 | 1 | 0 | 0 | 0 | 0 | 0 | 0 | 0 | 0 | 0 | 0 | 0 | 0 | 0 | 0 | 0 | 0 | 0 | 0 | 0 | 0 | 0 | 0 | 0 | 0 | 0 | 0 | 0 | 0 | 0 | 0 | 0 | -1 | 0 | 0 | 0 | 0 | 0 | 0 | 0 | 0 | 0 | 0 | 2 | 0 | 0 | 0 | 0 | 0 |
| \_6pgl\_c | 0 | 0 | 0 | 0 | 0 | 0 | 0 | 0 | 0 | 0 | 0 | 0 | 0 | 0 | 0 | 0 | 0 | 0 | 0 | 0 | 0 | 0 | 0 | 0 | 0 | 0 | 0 | 0 | 0 | 0 | 0 | 0 | 0 | 0 | 1 | -1 | 0 | 0 | 0 | 0 | 0 | 0 | 0 | 0 | 0 | 0 | 0 | 0 | 0 | 0 | 0 | 0 | 0 | 0 | 0 | 0 | 0 | 0 | 0 | 0 | 0 | 0 | 0 | 0 | 0 | 0 | 0 | 0 | 0 | 0 | 0 | 0 | 0 | 0 | 0 | 0 | 2 | 0 | 0 | 0 | 0 | 0 |
| \_6pgc\_c | 0 | 0 | 0 | 0 | 0 | 0 | 0 | 0 | 0 | 0 | 0 | 0 | 0 | 0 | 0 | 0 | 0 | 0 | 0 | 0 | 0 | 0 | 0 | 0 | 0 | 0 | 0 | 0 | 0 | 0 | 0 | 0 | 0 | 0 | 0 | 1 | -1 | 0 | 0 | 0 | 0 | 0 | 0 | 0 | 0 | 0 | 0 | 0 | 0 | 0 | 0 | 0 | 0 | 0 | 0 | 0 | 0 | 0 | 0 | 0 | 0 | 0 | 0 | 0 | 0 | 0 | 0 | 0 | 0 | 0 | 0 | 0 | 0 | 0 | 0 | 0 | 2 | 0 | 0 | 0 | 0 | 0 |
| ru5p\_\_D\_c | 0 | 0 | 0 | 0 | 0 | 0 | 0 | 0 | 0 | 0 | 0 | 0 | 0 | 0 | 0 | 0 | 0 | 0 | 0 | 0 | 0 | 0 | 0 | 0 | 0 | 0 | 0 | 0 | 0 | 0 | 0 | 0 | 0 | 0 | 0 | 0 | 1 | -1 | -1 | 0 | 0 | 0 | 0 | 0 | 0 | 0 | 0 | 0 | 0 | 0 | 0 | 0 | 0 | 0 | 0 | 0 | 0 | 0 | 0 | 0 | 0 | 0 | 0 | 0 | 0 | 0 | 0 | 0 | 0 | 0 | 0 | 0 | 0 | 0 | 0 | 0 | 3 | 0 | 0 | 0 | 0 | 0 |
| xu5p\_\_D\_c | 0 | 0 | 0 | 0 | 0 | 0 | 0 | 0 | 0 | 0 | 0 | 0 | 0 | 0 | 0 | 0 | 0 | 0 | 0 | 0 | 0 | 0 | 0 | 0 | 0 | 0 | 0 | 0 | 0 | 0 | 0 | 0 | 0 | 0 | 0 | 0 | 0 | 1 | 0 | -1 | -1 | 0 | 0 | 0 | 0 | 0 | 0 | 0 | 0 | 0 | 0 | 0 | 0 | 0 | 0 | 0 | 0 | 0 | 0 | 0 | 0 | 0 | 0 | 0 | 0 | 0 | 0 | 0 | 0 | 0 | 0 | 0 | 0 | 0 | 0 | 0 | 3 | 0 | 0 | 0 | 0 | 0 |
| r5p\_c | 0 | 0 | 0 | 0 | 0 | 0 | 0 | 0 | 0 | 0 | 0 | 0 | 0 | 0 | 0 | 0 | 0 | 0 | 0 | 0 | 0 | 0 | 0 | 0 | 0 | 0 | 0 | 0 | 0 | 0 | 0 | 0 | 0 | 0 | 0 | 0 | 0 | 0 | 1 | -1 | 0 | 0 | 0 | 0 | 0 | 0 | 0 | 0 | 1 | -1 | 0 | 0 | 0 | 0 | 0 | 0 | 0 | 0 | 0 | 0 | 0 | 0 | 0 | 0 | 0 | 0 | 0 | 0 | 0 | 0 | 0 | 0 | 0 | 0 | 0 | 0 | 4 | 0 | 0 | 0 | 0 | 0 |
| s7p\_c | 0 | 0 | 0 | 0 | 0 | 0 | 0 | 0 | 0 | 0 | 0 | 0 | 0 | 0 | 0 | 0 | 0 | 0 | 0 | 0 | 0 | 0 | 0 | 0 | 0 | 0 | 0 | 0 | 0 | 0 | 0 | 0 | 0 | 0 | 0 | 0 | 0 | 0 | 0 | 1 | 0 | -1 | 0 | 0 | 0 | 0 | 0 | 0 | 0 | 0 | 0 | 0 | 0 | 0 | 0 | 0 | 0 | 0 | 0 | 0 | 0 | 0 | 0 | 0 | 0 | 0 | 0 | 0 | 0 | 0 | 0 | 0 | 0 | 0 | 0 | 0 | 2 | 0 | 0 | 0 | 0 | 0 |
| e4p\_c | 0 | 0 | 0 | 0 | 0 | 0 | 0 | 0 | 0 | 0 | 0 | 0 | 0 | 0 | 0 | 0 | 0 | 0 | 0 | 0 | 0 | 0 | 0 | 0 | 0 | 0 | 0 | 0 | 0 | 0 | 0 | 0 | 0 | 0 | 0 | 0 | 0 | 0 | 0 | 0 | -1 | 1 | 0 | 0 | 0 | 0 | 0 | 0 | 0 | 0 | 0 | 0 | 0 | 0 | 0 | 0 | 0 | 0 | 0 | 0 | 0 | 0 | 0 | 0 | 0 | 0 | 0 | 0 | 0 | 0 | 0 | 0 | 0 | 0 | 0 | 0 | 2 | 0 | 0 | 0 | 0 | 0 |
| ade\_c | 0 | 0 | 0 | 0 | 0 | 0 | 0 | 0 | 0 | 0 | 0 | 0 | 0 | 0 | 0 | 0 | 0 | 0 | 0 | 0 | 0 | 0 | 0 | 0 | 0 | 0 | 0 | 0 | 0 | 0 | 0 | 0 | 0 | 0 | 0 | 0 | 0 | 0 | 0 | 0 | 0 | 0 | 0 | 0 | 0 | 0 | 0 | 0 | 0 | 0 | -1 | 0 | 0 | 0 | 0 | 0 | 0 | 0 | 0 | 0 | 0 | 0 | 0 | 0 | 0 | 0 | -1 | 0 | 0 | 0 | 0 | 0 | 0 | 0 | 0 | 0 | 2 | 0 | 0 | 0 | 0 | 0 |
| adn\_c | 0 | 0 | 0 | 0 | 0 | 0 | 0 | 0 | 0 | 0 | 0 | 0 | 0 | 0 | 0 | 0 | 0 | 0 | 0 | 0 | 0 | 0 | 0 | 0 | 0 | 0 | 0 | 0 | 0 | 0 | 0 | 0 | 0 | 0 | 0 | 0 | 0 | 0 | 0 | 0 | 0 | 0 | -1 | 1 | -1 | 0 | 0 | 0 | 0 | 0 | 0 | 0 | 0 | 0 | 0 | 0 | 0 | 0 | 0 | 0 | 0 | 0 | 0 | 0 | 0 | 0 | 0 | -1 | 0 | 0 | 0 | 0 | 0 | 0 | 0 | 0 | 4 | 0 | 0 | 0 | 0 | 0 |
| imp\_c | 0 | 0 | 0 | 0 | 0 | 0 | 0 | 0 | 0 | 0 | 0 | 0 | 0 | 0 | 0 | 0 | 0 | 0 | 0 | 0 | 0 | 0 | 0 | 0 | 0 | 0 | 0 | 0 | 0 | 0 | 0 | 0 | 0 | 0 | 0 | 0 | 0 | 0 | 0 | 0 | 0 | 0 | 0 | 0 | 0 | 1 | -1 | 0 | 0 | 0 | 0 | 0 | 0 | 0 | 0 | 0 | 0 | 0 | 0 | 0 | 0 | 0 | 0 | 0 | 0 | 0 | 0 | 0 | 0 | 0 | 0 | 0 | 0 | 0 | 0 | 0 | 2 | 0 | 0 | 0 | 0 | 0 |
| ins\_c | 0 | 0 | 0 | 0 | 0 | 0 | 0 | 0 | 0 | 0 | 0 | 0 | 0 | 0 | 0 | 0 | 0 | 0 | 0 | 0 | 0 | 0 | 0 | 0 | 0 | 0 | 0 | 0 | 0 | 0 | 0 | 0 | 0 | 0 | 0 | 0 | 0 | 0 | 0 | 0 | 0 | 0 | 0 | 0 | 1 | 0 | 1 | -1 | 0 | 0 | 0 | 0 | 0 | 0 | 0 | 0 | 0 | 0 | 0 | 0 | 0 | 0 | 0 | 0 | 0 | 0 | 0 | 0 | -1 | 0 | 0 | 0 | 0 | 0 | 0 | 0 | 4 | 0 | 0 | 0 | 0 | 0 |
| hxan\_c | 0 | 0 | 0 | 0 | 0 | 0 | 0 | 0 | 0 | 0 | 0 | 0 | 0 | 0 | 0 | 0 | 0 | 0 | 0 | 0 | 0 | 0 | 0 | 0 | 0 | 0 | 0 | 0 | 0 | 0 | 0 | 0 | 0 | 0 | 0 | 0 | 0 | 0 | 0 | 0 | 0 | 0 | 0 | 0 | 0 | 0 | 0 | 1 | 0 | 0 | 0 | 0 | 0 | 0 | 0 | 0 | 0 | 0 | 0 | 0 | 0 | 0 | 0 | 0 | 0 | 0 | 0 | 0 | 0 | -1 | 0 | 0 | 0 | 0 | 0 | 0 | 2 | 0 | 0 | 0 | 0 | 0 |
| r1p\_c | 0 | 0 | 0 | 0 | 0 | 0 | 0 | 0 | 0 | 0 | 0 | 0 | 0 | 0 | 0 | 0 | 0 | 0 | 0 | 0 | 0 | 0 | 0 | 0 | 0 | 0 | 0 | 0 | 0 | 0 | 0 | 0 | 0 | 0 | 0 | 0 | 0 | 0 | 0 | 0 | 0 | 0 | 0 | 0 | 0 | 0 | 0 | 1 | -1 | 0 | 0 | 0 | 0 | 0 | 0 | 0 | 0 | 0 | 0 | 0 | 0 | 0 | 0 | 0 | 0 | 0 | 0 | 0 | 0 | 0 | 0 | 0 | 0 | 0 | 0 | 0 | 2 | 0 | 0 | 0 | 0 | 0 |
| prpp\_c | 0 | 0 | 0 | 0 | 0 | 0 | 0 | 0 | 0 | 0 | 0 | 0 | 0 | 0 | 0 | 0 | 0 | 0 | 0 | 0 | 0 | 0 | 0 | 0 | 0 | 0 | 0 | 0 | 0 | 0 | 0 | 0 | 0 | 0 | 0 | 0 | 0 | 0 | 0 | 0 | 0 | 0 | 0 | 0 | 0 | 0 | 0 | 0 | 0 | 1 | -1 | 0 | 0 | 0 | 0 | 0 | 0 | 0 | 0 | 0 | 0 | 0 | 0 | 0 | 0 | 0 | 0 | 0 | 0 | 0 | 0 | 0 | 0 | 0 | 0 | 0 | 2 | 0 | 0 | 0 | 0 | 0 |
| \_23dpg\_c | 0 | 0 | 0 | 0 | 0 | 0 | 0 | 0 | 0 | 0 | 0 | 0 | 0 | 0 | 0 | 0 | 0 | 0 | 0 | 0 | 0 | 0 | 0 | 0 | 0 | 0 | 0 | 0 | 0 | 0 | 0 | 0 | 0 | 0 | 0 | 0 | 0 | 0 | 0 | 0 | 0 | 0 | 0 | 0 | 0 | 0 | 0 | 0 | 0 | 0 | 0 | 0 | 1 | -1 | -1 | 0 | 0 | 0 | 0 | 0 | 0 | 0 | 0 | 0 | 0 | 0 | 0 | 0 | 0 | 0 | 0 | 0 | 0 | 0 | 0 | 0 | 3 | 0 | 0 | 0 | 0 | 0 |
| hb\_c | 0 | 0 | 0 | 0 | 0 | 0 | 0 | 0 | 0 | 0 | 0 | 0 | 0 | 0 | 0 | 0 | 0 | 0 | 0 | 0 | 0 | 0 | 0 | 0 | 0 | 0 | 0 | 0 | 0 | 0 | 0 | 0 | 0 | 0 | 0 | 0 | 0 | 0 | 0 | 0 | 0 | 0 | 0 | 0 | 0 | 0 | 0 | 0 | 0 | 0 | 0 | 0 | 0 | 0 | -1 | -1 | 0 | 0 | 0 | 0 | 0 | 0 | 0 | 0 | 0 | 0 | 0 | 0 | 0 | 0 | 0 | 0 | 0 | 0 | 0 | 0 | 2 | 0 | 0 | 0 | 1 | 0 |
| hb\_1o2\_c | 0 | 0 | 0 | 0 | 0 | 0 | 0 | 0 | 0 | 0 | 0 | 0 | 0 | 0 | 0 | 0 | 0 | 0 | 0 | 0 | 0 | 0 | 0 | 0 | 0 | 0 | 0 | 0 | 0 | 0 | 0 | 0 | 0 | 0 | 0 | 0 | 0 | 0 | 0 | 0 | 0 | 0 | 0 | 0 | 0 | 0 | 0 | 0 | 0 | 0 | 0 | 0 | 0 | 0 | 0 | 1 | -1 | 0 | 0 | 0 | 0 | 0 | 0 | 0 | 0 | 0 | 0 | 0 | 0 | 0 | 0 | 0 | 0 | 0 | 0 | 0 | 2 | 0 | 0 | 0 | 1 | 0 |
| hb\_2o2\_c | 0 | 0 | 0 | 0 | 0 | 0 | 0 | 0 | 0 | 0 | 0 | 0 | 0 | 0 | 0 | 0 | 0 | 0 | 0 | 0 | 0 | 0 | 0 | 0 | 0 | 0 | 0 | 0 | 0 | 0 | 0 | 0 | 0 | 0 | 0 | 0 | 0 | 0 | 0 | 0 | 0 | 0 | 0 | 0 | 0 | 0 | 0 | 0 | 0 | 0 | 0 | 0 | 0 | 0 | 0 | 0 | 1 | -1 | 0 | 0 | 0 | 0 | 0 | 0 | 0 | 0 | 0 | 0 | 0 | 0 | 0 | 0 | 0 | 0 | 0 | 0 | 2 | 0 | 0 | 0 | 1 | 0 |
| hb\_3o2\_c | 0 | 0 | 0 | 0 | 0 | 0 | 0 | 0 | 0 | 0 | 0 | 0 | 0 | 0 | 0 | 0 | 0 | 0 | 0 | 0 | 0 | 0 | 0 | 0 | 0 | 0 | 0 | 0 | 0 | 0 | 0 | 0 | 0 | 0 | 0 | 0 | 0 | 0 | 0 | 0 | 0 | 0 | 0 | 0 | 0 | 0 | 0 | 0 | 0 | 0 | 0 | 0 | 0 | 0 | 0 | 0 | 0 | 1 | -1 | 0 | 0 | 0 | 0 | 0 | 0 | 0 | 0 | 0 | 0 | 0 | 0 | 0 | 0 | 0 | 0 | 0 | 2 | 0 | 0 | 0 | 1 | 0 |
| hb\_4o2\_c | 0 | 0 | 0 | 0 | 0 | 0 | 0 | 0 | 0 | 0 | 0 | 0 | 0 | 0 | 0 | 0 | 0 | 0 | 0 | 0 | 0 | 0 | 0 | 0 | 0 | 0 | 0 | 0 | 0 | 0 | 0 | 0 | 0 | 0 | 0 | 0 | 0 | 0 | 0 | 0 | 0 | 0 | 0 | 0 | 0 | 0 | 0 | 0 | 0 | 0 | 0 | 0 | 0 | 0 | 0 | 0 | 0 | 0 | 1 | 0 | 0 | 0 | 0 | 0 | 0 | 0 | 0 | 0 | 0 | 0 | 0 | 0 | 0 | 0 | 0 | 0 | 1 | 0 | 0 | 0 | 1 | 0 |
| dhb\_c | 0 | 0 | 0 | 0 | 0 | 0 | 0 | 0 | 0 | 0 | 0 | 0 | 0 | 0 | 0 | 0 | 0 | 0 | 0 | 0 | 0 | 0 | 0 | 0 | 0 | 0 | 0 | 0 | 0 | 0 | 0 | 0 | 0 | 0 | 0 | 0 | 0 | 0 | 0 | 0 | 0 | 0 | 0 | 0 | 0 | 0 | 0 | 0 | 0 | 0 | 0 | 0 | 0 | 0 | 1 | 0 | 0 | 0 | 0 | 0 | 0 | 0 | 0 | 0 | 0 | 0 | 0 | 0 | 0 | 0 | 0 | 0 | 0 | 0 | 0 | 0 | 1 | 0 | 0 | 0 | 1 | 0 |
| nad\_c | 0 | 0 | 0 | 0 | 0 | 0 | 0 | 0 | 0 | 0 | 0 | 0 | 0 | 0 | 0 | 0 | 0 | 0 | 0 | 0 | 0 | 0 | 0 | 0 | 0 | 0 | 0 | 0 | -1 | 0 | 0 | 0 | 0 | 1 | 0 | 0 | 0 | 0 | 0 | 0 | 0 | 0 | 0 | 0 | 0 | 0 | 0 | 0 | 0 | 0 | 0 | 0 | 0 | 0 | 0 | 0 | 0 | 0 | 0 | 0 | 1 | 0 | 0 | 0 | 0 | 0 | 0 | 0 | 0 | 0 | 0 | 0 | 0 | 0 | 0 | 0 | 3 | 1 | 0 | 0 | 0 | 0 |
| nadh\_c | 0 | 0 | 0 | 0 | 0 | 0 | 0 | 0 | 0 | 0 | 0 | 0 | 0 | 0 | 0 | 0 | 0 | 0 | 0 | 0 | 0 | 0 | 0 | 0 | 0 | 0 | 0 | 0 | 1 | 0 | 0 | 0 | 0 | -1 | 0 | 0 | 0 | 0 | 0 | 0 | 0 | 0 | 0 | 0 | 0 | 0 | 0 | 0 | 0 | 0 | 0 | 0 | 0 | 0 | 0 | 0 | 0 | 0 | 0 | 0 | -1 | 0 | 0 | 0 | 0 | 0 | 0 | 0 | 0 | 0 | 0 | 0 | 0 | 0 | 0 | 0 | 3 | 1 | 0 | 0 | 0 | 0 |
| amp\_c | 0 | 0 | 0 | 0 | 0 | 0 | 0 | 0 | 0 | 0 | 0 | 0 | 0 | 0 | 0 | 0 | 0 | -1 | -1 | -1 | -1 | 0 | 0 | 0 | 0 | 0 | 0 | 0 | 0 | 0 | 0 | 0 | 0 | 0 | 0 | 0 | 0 | 0 | 0 | 0 | 0 | 0 | 1 | -1 | 0 | -1 | 0 | 0 | 0 | 1 | 1 | 1 | 0 | 0 | 0 | 0 | 0 | 0 | 0 | 0 | 0 | 0 | 0 | 0 | 0 | 0 | 0 | 0 | 0 | 0 | 0 | 0 | 0 | 0 | 0 | 0 | 10 | 0 | 0 | 0 | 0 | 0 |
| adp\_c | 1 | 0 | 0 | 0 | 1 | 0 | 0 | 1 | 0 | 0 | 1 | 0 | 0 | 1 | 0 | 0 | 1 | 0 | 0 | 0 | 0 | 0 | 0 | 0 | 0 | 0 | 0 | 0 | 0 | -1 | 0 | 0 | -1 | 0 | 0 | 0 | 0 | 0 | 0 | 0 | 0 | 0 | 1 | 0 | 0 | 0 | 0 | 0 | 0 | 0 | 0 | -2 | 0 | 0 | 0 | 0 | 0 | 0 | 0 | 1 | 0 | 0 | 0 | 0 | 0 | 0 | 0 | 0 | 0 | 0 | 0 | 0 | 0 | 0 | 0 | 0 | 11 | 0 | 0 | 0 | 0 | 0 |
| atp\_c | -1 | 0 | -1 | 0 | 0 | -1 | 0 | 0 | -1 | 0 | 0 | -1 | 0 | 0 | -1 | 0 | 0 | 0 | 0 | 0 | 0 | -1 | -1 | -1 | -1 | 0 | 0 | 0 | 0 | 1 | 0 | 0 | 1 | 0 | 0 | 0 | 0 | 0 | 0 | 0 | 0 | 0 | -1 | 0 | 0 | 0 | 0 | 0 | 0 | -1 | 0 | 1 | 0 | 0 | 0 | 0 | 0 | 0 | 0 | -1 | 0 | 0 | 0 | 0 | 0 | 0 | 0 | 0 | 0 | 0 | 0 | 0 | 0 | 0 | 0 | 0 | 16 | 0 | 0 | 0 | 0 | 0 |
| nadp\_c | 0 | 0 | 0 | 0 | 0 | 0 | 0 | 0 | 0 | 0 | 0 | 0 | 0 | 0 | 0 | 0 | 0 | 0 | 0 | 0 | 0 | 0 | 0 | 0 | 0 | 0 | 0 | 0 | 0 | 0 | 0 | 0 | 0 | 0 | -1 | 0 | -1 | 0 | 0 | 0 | 0 | 0 | 0 | 0 | 0 | 0 | 0 | 0 | 0 | 0 | 0 | 0 | 0 | 0 | 0 | 0 | 0 | 0 | 0 | 0 | 0 | 1 | 0 | 0 | 0 | 0 | 0 | 0 | 0 | 0 | 0 | 0 | 0 | 0 | 0 | 0 | 3 | 0 | 1 | 0 | 0 | 0 |
| nadph\_c | 0 | 0 | 0 | 0 | 0 | 0 | 0 | 0 | 0 | 0 | 0 | 0 | 0 | 0 | 0 | 0 | 0 | 0 | 0 | 0 | 0 | 0 | 0 | 0 | 0 | 0 | 0 | 0 | 0 | 0 | 0 | 0 | 0 | 0 | 1 | 0 | 1 | 0 | 0 | 0 | 0 | 0 | 0 | 0 | 0 | 0 | 0 | 0 | 0 | 0 | 0 | 0 | 0 | 0 | 0 | 0 | 0 | 0 | 0 | 0 | 0 | -1 | 0 | 0 | 0 | 0 | 0 | 0 | 0 | 0 | 0 | 0 | 0 | 0 | 0 | 0 | 3 | 0 | 1 | 0 | 0 | 0 |
| gthrd\_c | 0 | 0 | 0 | 0 | 0 | 0 | 0 | 0 | 0 | 0 | 0 | 0 | 0 | 0 | 0 | 0 | 0 | 0 | 0 | 0 | 0 | 0 | 0 | 0 | 0 | 0 | 0 | 0 | 0 | 0 | 0 | 0 | 0 | 0 | 0 | 0 | 0 | 0 | 0 | 0 | 0 | 0 | 0 | 0 | 0 | 0 | 0 | 0 | 0 | 0 | 0 | 0 | 0 | 0 | 0 | 0 | 0 | 0 | 0 | 0 | 0 | 2 | -2 | 0 | 0 | 0 | 0 | 0 | 0 | 0 | 0 | 0 | 0 | 0 | 0 | 0 | 2 | 0 | 0 | 1 | 0 | 0 |
| gthox\_c | 0 | 0 | 0 | 0 | 0 | 0 | 0 | 0 | 0 | 0 | 0 | 0 | 0 | 0 | 0 | 0 | 0 | 0 | 0 | 0 | 0 | 0 | 0 | 0 | 0 | 0 | 0 | 0 | 0 | 0 | 0 | 0 | 0 | 0 | 0 | 0 | 0 | 0 | 0 | 0 | 0 | 0 | 0 | 0 | 0 | 0 | 0 | 0 | 0 | 0 | 0 | 0 | 0 | 0 | 0 | 0 | 0 | 0 | 0 | 0 | 0 | -1 | 1 | 0 | 0 | 0 | 0 | 0 | 0 | 0 | 0 | 0 | 0 | 0 | 0 | 0 | 2 | 0 | 0 | 2 | 0 | 0 |
| pi\_c | 0 | 0 | 0 | 0 | 0 | 0 | 0 | 0 | 0 | 0 | 0 | 0 | 0 | 0 | 0 | 0 | 0 | 0 | 0 | 0 | 0 | 0 | 0 | 0 | 0 | 0 | 0 | 0 | -1 | 0 | 0 | 0 | 0 | 0 | 0 | 0 | 0 | 0 | 0 | 0 | 0 | 0 | 0 | 1 | 0 | 0 | 1 | -1 | 0 | 0 | 2 | 0 | 0 | 1 | 0 | 0 | 0 | 0 | 0 | 1 | 0 | 0 | 0 | 0 | 0 | 0 | 0 | 0 | 0 | 0 | -1 | 0 | 0 | 0 | 0 | 0 | 8 | 0 | 0 | 0 | 0 | 0 |
| h\_c | 1 | 0 | 0 | 0 | 1 | 0 | 0 | 1 | 0 | 0 | 1 | 0 | 0 | 1 | 0 | 0 | 1 | 0 | 0 | 0 | 0 | 0 | 0 | 0 | 0 | 0 | 0 | 0 | 1 | 0 | 0 | 0 | -1 | -1 | 1 | 1 | 0 | 0 | 0 | 0 | 0 | 0 | 1 | 0 | 0 | 0 | 0 | 0 | 0 | 1 | 1 | 0 | 1 | 0 | 0 | 0 | 0 | 0 | 0 | 1 | 1 | -1 | 2 | 0 | 0 | 0 | 0 | 0 | 0 | 0 | 0 | -1 | 0 | 0 | 0 | 0 | 20 | 0 | 0 | 0 | 0 | 0 |
| h2o\_c | 0 | 0 | 0 | 0 | 0 | 0 | 0 | 0 | 0 | 0 | 0 | 0 | 0 | 0 | 0 | 0 | 0 | 0 | 0 | 0 | 0 | 0 | 0 | 0 | 0 | 0 | 0 | 0 | 0 | 0 | 0 | 1 | 0 | 0 | 0 | -1 | 0 | 0 | 0 | 0 | 0 | 0 | 0 | -1 | -1 | -1 | -1 | 0 | 0 | 0 | -1 | 0 | 0 | -1 | 0 | 0 | 0 | 0 | 0 | -1 | 0 | 0 | 0 | 0 | 0 | 0 | 0 | 0 | 0 | 0 | 0 | 0 | -1 | 0 | 0 | 0 | 10 | 0 | 0 | 0 | 0 | 0 |
| co2\_c | 0 | 0 | 0 | 0 | 0 | 0 | 0 | 0 | 0 | 0 | 0 | 0 | 0 | 0 | 0 | 0 | 0 | 0 | 0 | 0 | 0 | 0 | 0 | 0 | 0 | 0 | 0 | 0 | 0 | 0 | 0 | 0 | 0 | 0 | 0 | 0 | 1 | 0 | 0 | 0 | 0 | 0 | 0 | 0 | 0 | 0 | 0 | 0 | 0 | 0 | 0 | 0 | 0 | 0 | 0 | 0 | 0 | 0 | 0 | 0 | 0 | 0 | 0 | 0 | 0 | 0 | 0 | 0 | 0 | 0 | 0 | 0 | 0 | -1 | 0 | 0 | 2 | 0 | 0 | 0 | 0 | 0 |
| nh3\_c | 0 | 0 | 0 | 0 | 0 | 0 | 0 | 0 | 0 | 0 | 0 | 0 | 0 | 0 | 0 | 0 | 0 | 0 | 0 | 0 | 0 | 0 | 0 | 0 | 0 | 0 | 0 | 0 | 0 | 0 | 0 | 0 | 0 | 0 | 0 | 0 | 0 | 0 | 0 | 0 | 0 | 0 | 0 | 0 | 1 | 1 | 0 | 0 | 0 | 0 | 0 | 0 | 0 | 0 | 0 | 0 | 0 | 0 | 0 | 0 | 0 | 0 | 0 | 0 | 0 | 0 | 0 | 0 | 0 | 0 | 0 | 0 | 0 | 0 | -1 | 0 | 3 | 0 | 0 | 0 | 0 | 0 |
| o2\_c | 0 | 0 | 0 | 0 | 0 | 0 | 0 | 0 | 0 | 0 | 0 | 0 | 0 | 0 | 0 | 0 | 0 | 0 | 0 | 0 | 0 | 0 | 0 | 0 | 0 | 0 | 0 | 0 | 0 | 0 | 0 | 0 | 0 | 0 | 0 | 0 | 0 | 0 | 0 | 0 | 0 | 0 | 0 | 0 | 0 | 0 | 0 | 0 | 0 | 0 | 0 | 0 | 0 | 0 | 0 | -1 | -1 | -1 | -1 | 0 | 0 | 0 | 0 | 0 | 0 | 0 | 0 | 0 | 0 | 0 | 0 | 0 | 0 | 0 | 0 | -1 | 5 | 0 | 0 | 0 | 0 | 0 |
| $\pi\_{j}$ | 5 | 2 | 3 | 3 | 5 | 3 | 3 | 5 | 3 | 3 | 5 | 3 | 3 | 5 | 3 | 3 | 5 | 3 | 3 | 3 | 3 | 3 | 3 | 3 | 3 | 2 | 3 | 2 | 6 | 4 | 2 | 3 | 5 | 5 | 5 | 4 | 5 | 2 | 2 | 4 | 4 | 4 | 5 | 4 | 4 | 4 | 4 | 4 | 2 | 5 | 6 | 3 | 3 | 4 | 3 | 3 | 3 | 3 | 3 | 5 | 3 | 5 | 3 | 1 | 1 | 1 | 1 | 1 | 1 | 1 | 1 | 1 | 1 | 1 | 1 | 1 |  |  |  |  |  |  |
| C | 0 | 0 | 0 | 0 | 0 | 0 | 0 | 0 | 0 | 0 | 0 | 0 | 0 | 0 | 0 | 0 | 0 | 0 | 0 | 0 | 0 | 0 | 0 | 0 | 0 | 0 | 0 | 0 | 0 | 0 | 0 | 0 | 0 | 0 | 0 | 0 | 0 | 0 | 0 | 0 | 0 | 0 | 0 | 0 | 0 | 0 | 0 | 0 | 0 | 0 | 0 | 0 | 0 | 0 | 0 | 0 | 0 | 0 | 0 | 0 | 0 | 0 | 0 | 6 | -3 | -3 | -5 | -10 | -10 | -5 | 0 | 0 | 0 | -1 | 0 | 0 |  |  |  |  |  |  |
| H | 0 | 0 | 0 | 0 | 0 | 0 | 0 | 0 | 0 | 0 | 0 | 0 | 0 | 0 | 0 | 0 | 0 | 0 | 0 | 0 | 0 | 0 | 0 | 0 | 0 | 0 | 0 | 0 | 0 | 0 | 0 | 0 | 0 | 0 | 0 | 0 | 0 | 0 | 0 | 0 | 0 | 0 | 0 | 0 | 0 | 0 | 0 | 0 | 0 | 0 | 0 | 0 | 0 | 0 | 0 | 0 | 0 | 0 | 0 | 0 | 0 | 0 | 0 | 12 | -3 | -5 | -5 | -13 | -12 | -4 | -1 | -1 | -2 | 0 | -3 | 0 |  |  |  |  |  |  |
| O | 0 | 0 | 0 | 0 | 0 | 0 | 0 | 0 | 0 | 0 | 0 | 0 | 0 | 0 | 0 | 0 | 0 | 0 | 0 | 0 | 0 | 0 | 0 | 0 | 0 | 0 | 0 | 0 | 0 | 0 | 0 | 0 | 0 | 0 | 0 | 0 | 0 | 0 | 0 | 0 | 0 | 0 | 0 | 0 | 0 | 0 | 0 | 0 | 0 | 0 | 0 | 0 | 0 | 0 | 0 | 0 | 0 | 0 | 0 | 0 | 0 | 0 | 0 | 6 | -3 | -3 | 0 | -4 | -5 | -1 | -4 | 0 | -1 | -2 | 0 | -2 |  |  |  |  |  |  |
| P | 0 | 0 | 0 | 0 | 0 | 0 | 0 | 0 | 0 | 0 | 0 | 0 | 0 | 0 | 0 | 0 | 0 | 0 | 0 | 0 | 0 | 0 | 0 | 0 | 0 | 0 | 0 | 0 | 0 | 0 | 0 | 0 | 0 | 0 | 0 | 0 | 0 | 0 | 0 | 0 | 0 | 0 | 0 | 0 | 0 | 0 | 0 | 0 | 0 | 0 | 0 | 0 | 0 | 0 | 0 | 0 | 0 | 0 | 0 | 0 | 0 | 0 | 0 | 0 | 0 | 0 | 0 | 0 | 0 | 0 | -1 | 0 | 0 | 0 | 0 | 0 |  |  |  |  |  |  |
| N | 0 | 0 | 0 | 0 | 0 | 0 | 0 | 0 | 0 | 0 | 0 | 0 | 0 | 0 | 0 | 0 | 0 | 0 | 0 | 0 | 0 | 0 | 0 | 0 | 0 | 0 | 0 | 0 | 0 | 0 | 0 | 0 | 0 | 0 | 0 | 0 | 0 | 0 | 0 | 0 | 0 | 0 | 0 | 0 | 0 | 0 | 0 | 0 | 0 | 0 | 0 | 0 | 0 | 0 | 0 | 0 | 0 | 0 | 0 | 0 | 0 | 0 | 0 | 0 | 0 | 0 | -5 | -5 | -4 | -4 | 0 | 0 | 0 | 0 | -1 | 0 |  |  |  |  |  |  |
| S | 0 | 0 | 0 | 0 | 0 | 0 | 0 | 0 | 0 | 0 | 0 | 0 | 0 | 0 | 0 | 0 | 0 | 0 | 0 | 0 | 0 | 0 | 0 | 0 | 0 | 0 | 0 | 0 | 0 | 0 | 0 | 0 | 0 | 0 | 0 | 0 | 0 | 0 | 0 | 0 | 0 | 0 | 0 | 0 | 0 | 0 | 0 | 0 | 0 | 0 | 0 | 0 | 0 | 0 | 0 | 0 | 0 | 0 | 0 | 0 | 0 | 0 | 0 | 0 | 0 | 0 | 0 | 0 | 0 | 0 | 0 | 0 | 0 | 0 | 0 | 0 |  |  |  |  |  |  |
| q | 0 | 0 | 0 | 0 | 0 | 0 | 0 | 0 | 0 | 0 | 0 | 0 | 0 | 0 | 0 | 0 | 0 | 0 | 0 | 0 | 0 | 0 | 0 | 0 | 0 | 0 | 0 | 0 | 0 | 0 | 0 | 0 | 0 | 0 | 0 | 0 | 0 | 0 | 0 | 0 | 0 | 0 | 0 | 0 | 0 | 0 | 0 | 0 | 0 | 0 | 0 | 0 | 0 | 0 | 0 | 0 | 0 | 0 | 0 | 0 | 2 | 0 | 2 | 0 | 1 | 1 | 0 | 0 | 0 | 0 | 2 | -1 | 0 | 0 | 0 | 0 |  |  |  |  |  |  |
| [PFK] | 0 | 0 | 0 | 0 | 0 | 0 | 0 | 0 | 0 | 0 | 0 | 0 | 0 | 0 | 0 | 0 | 0 | 0 | 0 | 0 | 0 | 0 | 0 | 0 | 0 | 0 | 0 | 0 | 0 | 0 | 0 | 0 | 0 | 0 | 0 | 0 | 0 | 0 | 0 | 0 | 0 | 0 | 0 | 0 | 0 | 0 | 0 | 0 | 0 | 0 | 0 | 0 | 0 | 0 | 0 | 0 | 0 | 0 | 0 | 0 | 0 | 0 | 0 | 0 | 0 | 0 | 0 | 0 | 0 | 0 | 0 | 0 | 0 | 0 | 0 | 0 |  |  |  |  |  |  |
| [HB] | 0 | 0 | 0 | 0 | 0 | 0 | 0 | 0 | 0 | 0 | 0 | 0 | 0 | 0 | 0 | 0 | 0 | 0 | 0 | 0 | 0 | 0 | 0 | 0 | 0 | 0 | 0 | 0 | 0 | 0 | 0 | 0 | 0 | 0 | 0 | 0 | 0 | 0 | 0 | 0 | 0 | 0 | 0 | 0 | 0 | 0 | 0 | 0 | 0 | 0 | 0 | 0 | 0 | 0 | 0 | 0 | 0 | 0 | 0 | 0 | 0 | 0 | 0 | 0 | 0 | 0 | 0 | 0 | 0 | 0 | 0 | 0 | 0 | 0 | 0 | 0 |  |  |  |  |  |  |
| [NAD] | 0 | 0 | 0 | 0 | 0 | 0 | 0 | 0 | 0 | 0 | 0 | 0 | 0 | 0 | 0 | 0 | 0 | 0 | 0 | 0 | 0 | 0 | 0 | 0 | 0 | 0 | 0 | 0 | 0 | 0 | 0 | 0 | 0 | 0 | 0 | 0 | 0 | 0 | 0 | 0 | 0 | 0 | 0 | 0 | 0 | 0 | 0 | 0 | 0 | 0 | 0 | 0 | 0 | 0 | 0 | 0 | 0 | 0 | 0 | 0 | 0 | 0 | 0 | 0 | 0 | 0 | 0 | 0 | 0 | 0 | 0 | 0 | 0 | 0 | 0 | 0 |  |  |  |  |  |  |
| [NADP] | 0 | 0 | 0 | 0 | 0 | 0 | 0 | 0 | 0 | 0 | 0 | 0 | 0 | 0 | 0 | 0 | 0 | 0 | 0 | 0 | 0 | 0 | 0 | 0 | 0 | 0 | 0 | 0 | 0 | 0 | 0 | 0 | 0 | 0 | 0 | 0 | 0 | 0 | 0 | 0 | 0 | 0 | 0 | 0 | 0 | 0 | 0 | 0 | 0 | 0 | 0 | 0 | 0 | 0 | 0 | 0 | 0 | 0 | 0 | 0 | 0 | 0 | 0 | 0 | 0 | 0 | 0 | 0 | 0 | 0 | 0 | 0 | 0 | 0 | 0 | 0 |  |  |  |  |  |  |
| $p\_1$ | 1 | 1 | 1 | 1 | 1 | 0 | 0 | 0 | 0 | 0 | 0 | 0 | 0 | 0 | 0 | 0 | 0 | 0 | 0 | 0 | 0 | 0 | 0 | 0 | 0 | 0 | 1 | 1 | 2 | 2 | 2 | 2 | 2 | 2 | 0 | 0 | 0 | 0 | 0 | 0 | 0 | 0 | 0 | 0 | 0 | 0 | 0 | 0 | 0 | 0 | 0 | 0 | 0 | 0 | 0 | 0 | 0 | 0 | 0 | 2 | 0 | 0 | 0 | 1 | 0 | 2 | 0 | 0 | 0 | 0 | 0 | 2 | 0 | 0 | 0 | 0 |  |  |  |  |  |  |
| $p\_2$ | 1 | 1 | 0 | 0 | 0 | 1 | 1 | 1 | 0 | 0 | 0 | 0 | 0 | 0 | 0 | 0 | 0 | 0 | 0 | 0 | 0 | 0 | 0 | 0 | 0 | 0 | 1 | 1 | 2 | 2 | 2 | 2 | 2 | 2 | 0 | 0 | 0 | 0 | 0 | 0 | 0 | 0 | 0 | 0 | 0 | 0 | 0 | 0 | 0 | 0 | 0 | 0 | 0 | 0 | 0 | 0 | 0 | 0 | 0 | 2 | 0 | 0 | 0 | 1 | 0 | 2 | 0 | 0 | 0 | 0 | 0 | 2 | 0 | 0 | 0 | 0 |  |  |  |  |  |  |
| $p\_3$ | 1 | 1 | 0 | 0 | 0 | 0 | 0 | 0 | 1 | 1 | 1 | 0 | 0 | 0 | 0 | 0 | 0 | 0 | 0 | 0 | 0 | 0 | 0 | 0 | 0 | 0 | 1 | 1 | 2 | 2 | 2 | 2 | 2 | 2 | 0 | 0 | 0 | 0 | 0 | 0 | 0 | 0 | 0 | 0 | 0 | 0 | 0 | 0 | 0 | 0 | 0 | 0 | 0 | 0 | 0 | 0 | 0 | 0 | 0 | 2 | 0 | 0 | 0 | 1 | 0 | 2 | 0 | 0 | 0 | 0 | 0 | 2 | 0 | 0 | 0 | 0 |  |  |  |  |  |  |
| $p\_4$ | 1 | 1 | 0 | 0 | 0 | 0 | 0 | 0 | 0 | 0 | 0 | 1 | 1 | 1 | 0 | 0 | 0 | 0 | 0 | 0 | 0 | 0 | 0 | 0 | 0 | 0 | 1 | 1 | 2 | 2 | 2 | 2 | 2 | 2 | 0 | 0 | 0 | 0 | 0 | 0 | 0 | 0 | 0 | 0 | 0 | 0 | 0 | 0 | 0 | 0 | 0 | 0 | 0 | 0 | 0 | 0 | 0 | 0 | 0 | 2 | 0 | 0 | 0 | 1 | 0 | 2 | 0 | 0 | 0 | 0 | 0 | 2 | 0 | 0 | 0 | 0 |  |  |  |  |  |  |
| $p\_5$ | 1 | 1 | 0 | 0 | 0 | 0 | 0 | 0 | 0 | 0 | 0 | 0 | 0 | 0 | 1 | 1 | 1 | 0 | 0 | 0 | 0 | 0 | 0 | 0 | 0 | 0 | 1 | 1 | 2 | 2 | 2 | 2 | 2 | 2 | 0 | 0 | 0 | 0 | 0 | 0 | 0 | 0 | 0 | 0 | 0 | 0 | 0 | 0 | 0 | 0 | 0 | 0 | 0 | 0 | 0 | 0 | 0 | 0 | 0 | 2 | 0 | 0 | 0 | 1 | 0 | 2 | 0 | 0 | 0 | 0 | 0 | 2 | 0 | 0 | 0 | 0 |  |  |  |  |  |  |
| $p\_6$ | 0 | 0 | 0 | 0 | 0 | 0 | 0 | 0 | 0 | 0 | 0 | 0 | 0 | 0 | 0 | 0 | 0 | 0 | 0 | 0 | 0 | 0 | 0 | 0 | 0 | 0 | 0 | 0 | 0 | 0 | 0 | 0 | 0 | 1 | 0 | 0 | 0 | 0 | 0 | 0 | 0 | 0 | 0 | 0 | 0 | 0 | 0 | 0 | 0 | 0 | 0 | 0 | 0 | 0 | 0 | 0 | 0 | 0 | 0 | 0 | -1 | 0 | 0 | 0 | -1 | 1 | 0 | 0 | 0 | 0 | 0 | -2 | 0 | 0 | 0 | 0 |  |  |  |  |  |  |
| $p\_7$ | 1 | -2 | 0 | 0 | 0 | 0 | 0 | 0 | 0 | 0 | 0 | 0 | 0 | 0 | 0 | 0 | 0 | 0 | 0 | 0 | 0 | 0 | 0 | 0 | 0 | 0 | 0 | 0 | 1 | 1 | 1 | 1 | 1 | 1 | 3 | 3 | 3 | 2 | 1 | 1 | 1 | 1 | 0 | 0 | 0 | 0 | 0 | 0 | 0 | 0 | 0 | 0 | 0 | 0 | 0 | 0 | 0 | 0 | 0 | 1 | 0 | 6 | 6 | 1 | 0 | 1 | 0 | 0 | 0 | 0 | 0 | 13 | -3 | 3 | 0 | 0 |  |  |  |  |  |  |
| $p\_8$ | 1 | 0 | 0 | 0 | 0 | 0 | 0 | 0 | 0 | 0 | 0 | 0 | 0 | 0 | 0 | 0 | 0 | 0 | 0 | 0 | 0 | 0 | 0 | 0 | 0 | 0 | 0 | 0 | 0 | 0 | 0 | 0 | 0 | 0 | 1 | 1 | 1 | 0 | 1 | 0 | 0 | 0 | 0 | 1 | 0 | 0 | 0 | 0 | 0 | 1 | 1 | -1 | 0 | 0 | 0 | 0 | 0 | 0 | 0 | -3 | 0 | 2 | 2 | 1 | 0 | 0 | -1 | 1 | 0 | 0 | 0 | 4 | 0 | 1 | 0 | 0 |  |  |  |  |  |  |
| $p\_9$ | 1 | 0 | 0 | 0 | 0 | 0 | 0 | 0 | 0 | 0 | 0 | 0 | 0 | 0 | 0 | 0 | 0 | 0 | 0 | 0 | 0 | 0 | 0 | 0 | 0 | 0 | 0 | 0 | 0 | 0 | 0 | 0 | 0 | 0 | 1 | 1 | 1 | 0 | 1 | 0 | 0 | 0 | 0 | 1 | 1 | 0 | 0 | 0 | 0 | 1 | 1 | -1 | 0 | 0 | 0 | 0 | 0 | 0 | 0 | -3 | 0 | 2 | 2 | 1 | 0 | 0 | -1 | 0 | 1 | 0 | 0 | 4 | -1 | 1 | 1 | 0 |  |  |  |  |  |  |
| $p\_{10}$ | 1 | 0 | 0 | 0 | 0 | 0 | 0 | 0 | 0 | 0 | 0 | 0 | 0 | 0 | 0 | 0 | 0 | 0 | 0 | 0 | 0 | 0 | 0 | 0 | 0 | 0 | 0 | 0 | 0 | 0 | 0 | 0 | 0 | 0 | 1 | 1 | 1 | 0 | 1 | 0 | 0 | 0 | 0 | 0 | 0 | 1 | 1 | 0 | 0 | 1 | 1 | -1 | 0 | 0 | 0 | 0 | 0 | 0 | 0 | -3 | 0 | 2 | 2 | 1 | 0 | 0 | -1 | 0 | 1 | 0 | 0 | 4 | -1 | 1 | 1 | 0 |  |  |  |  |  |  |
| $p\_{11}$ | 0 | 0 | 0 | 0 | 0 | 0 | 0 | 0 | 0 | 0 | 0 | 0 | 0 | 0 | 0 | 0 | 0 | 0 | 0 | 0 | 0 | 0 | 0 | 0 | 0 | 0 | 0 | 0 | 0 | 0 | 0 | 0 | 0 | 0 | 0 | 0 | 0 | 0 | 0 | 0 | 0 | 0 | 0 | 1 | 1 | 0 | 0 | 1 | 1 | 1 | 1 | -1 | 0 | 0 | 0 | 0 | 0 | 0 | 0 | -2 | 0 | 0 | 0 | 0 | 0 | 0 | -1 | 0 | 0 | 1 | 0 | 0 | -1 | 0 | 1 | 0 |  |  |  |  |  |  |
| $p\_{12}$ | 0 | 0 | 0 | 0 | 0 | 0 | 0 | 0 | 0 | 0 | 0 | 0 | 0 | 0 | 0 | 0 | 0 | 0 | 0 | 0 | 0 | 0 | 0 | 0 | 0 | 0 | 0 | 0 | 0 | 0 | 0 | 0 | 0 | 0 | 0 | 0 | 0 | 0 | 0 | 0 | 0 | 0 | 1 | 1 | 0 | 0 | 0 | 0 | 0 | 0 | 0 | 0 | 0 | 0 | 0 | 0 | 0 | 0 | 0 | -1 | 0 | 0 | 0 | 0 | 0 | 0 | 0 | 0 | 0 | 0 | 0 | 0 | 0 | 0 | 0 | 0 |  |  |  |  |  |  |
| $p\_{13}$ | 1 | 1 | 0 | 0 | 0 | 0 | 0 | 0 | 1 | 1 | 1 | 0 | 0 | 0 | 0 | 0 | 0 | 0 | 0 | 0 | 0 | 0 | 0 | 0 | 0 | 0 | 1 | 1 | 2 | 0 | 2 | 2 | 2 | 2 | 0 | 0 | 0 | 0 | 0 | 0 | 0 | 0 | 0 | 0 | 0 | 0 | 0 | 0 | 0 | 0 | 0 | 0 | 2 | 2 | 0 | 0 | 0 | 0 | 0 | 2 | 0 | 0 | 0 | 1 | 0 | 2 | 0 | 0 | 0 | 0 | 0 | 2 | 0 | 0 | 0 | 0 |  |  |  |  |  |  |

###### The null spaces: PFK with RBC metabolic network¶

The null space is thus 13 (=76-63) dimensional. It fundamentally has the same pathways as the previous models alone. The difference is that pathway 1 in Figure 10.2 now becomes five pathways \((\textbf{p}\_1\) through \(\textbf{p}\_5\) in Table 14.4), one through each of the \(R\_i\) forms of PFK, consistent with the properties of the stoichiometric matrix for the PFK subnetwork. The remaining pathways are the same as the ones seen in previous models and become \(\textbf{p}\_6\)
through \(\textbf{p}\_{13}\) in Table 14.4 to make a total of 13 pathways.

The left null space is 5 (=68-63) dimensional. It has the same pools as the previous models, namely the total phosphate pool, NAD and NADP pools, the total hemoglobin pool,and the total PFK pool that originates from the stoichiometric matrix of the PFK subnetwork, Table 14.2.

##### Simulating the RBC Metabolic Network with PFK¶

###### Defining the Steady State¶

We start by ensuring our model is at steady state.

```
[30]:
```

```
sim_RBC_PFK = Simulation(RBC_PFK)
sim_RBC_PFK.find_steady_state(
    RBC_PFK, strategy="simulate", update_values=True);

t0, tf = (0, 1e4)
conc_sol_ss, flux_sol_ss = sim_RBC_PFK.simulate(
    RBC_PFK, time=(t0, tf, tf*10 + 1))
conc_sol_ss.view_time_profile()
```

###### Response to an increased \(k\_{ATPM}\): PFK with RBC metabolic network¶

We now perturb the integrated model by increasing the rate of ATP utilization by 25%. Note that the perturbation is smaller than in previous chapters to allow for a steady state to be reached, even with the additional regulatory mechanisms in the larger RBC model.

```
[31]:
```

```
t0, tf = (0, 1e4)
conc_sol, flux_sol = sim_RBC_PFK.simulate(
    RBC_PFK, time=(t0, tf, tf*10 + 1),
    perturbations={"kf_ATPM": "kf_ATPM * 1.25"});
```

```
[32]:
```

```
fig_14_11, ax = plt.subplots(nrows=1, ncols=1, figsize=(12, 5));

plot_time_profile(
    conc_sol, ax=ax, legend="right outside",
    plot_function="semilogx", xlim=(1e-6, tf),
    xlabel="Time [hr]", ylabel="Concentration [mM]",
    title=("Concentration Profile", L_FONT));
fig_14_11.tight_layout()
```

**Figure 14.11:** Simulating the combined system from the steady state with 25% increase in the rate of ATP utilization at \(t = 0.\)\*

###### The proton node: PFK with RBC metabolic network¶

The proton node now has a connectivity of 20 with 16 production reactions and 4 utilization reactions.

```
[33]:
```

```
fig_14_12 = plt.figure(figsize=(18, 6))
gs = fig_14_12.add_gridspec(nrows=3, ncols=2, width_ratios=[1.5, 1])

ax1 = fig_14_12.add_subplot(gs[0, 0])
ax2 = fig_14_12.add_subplot(gs[1, 0])
ax3 = fig_14_12.add_subplot(gs[2, 0])
ax4 = fig_14_12.add_subplot(gs[:, 1])

plot_time_profile(
    conc_sol, observable="h_c", ax=ax1,
    legend="right outside", plot_function="semilogx",
    xlim=(1e-6, tf), ylim=(1e-4 - 5e-6, 1e-4 + 5e-6),
    xlabel="Time [hr]", ylabel="Concentrations [mM]",
    title=("(a) Proton Concentration", L_FONT));

fluxes_in = ["HEX1", "PFK_R03", "PFK_R13", "PFK_R23", "PFK_R33", "PFK_R43",
             "GAPD", "ATPM", "DM_nadh", "G6PDH2r", "PGL",
             "GSHR", "ADNK1", "PRPPS", "ADPT", "DPGM"]
plot_time_profile(
    flux_sol, observable=fluxes_in, ax=ax2,
    legend="right outside", plot_function="semilogx",
    xlim=(1e-6, tf), ylim=(-0.2, 3.5),
    xlabel="Time [hr]", ylabel="Fluxes [mM/hr]",
    title=("(b) Fluxes in", L_FONT));


fluxes_out = ["PYK", "LDH_L", "SK_h_c", "GSHR"]
plot_time_profile(
    flux_sol, observable=fluxes_out, ax=ax3,
    legend="right outside", plot_function="semilogx",
    xlim=(1e-6, tf),  ylim=(-.1, 5),
    xlabel="Time [hr]", ylabel="Fluxes [mM/hr]",
    title=("(c) Fluxes out", L_FONT));

time_points = [t0, 1e-1, 1e0, 1e1, 1e2, tf]
time_point_colors = [
    mpl.colors.to_hex(c)
    for c in mpl.cm.Reds(np.linspace(0.3, 1, len(time_points)))]

linestyles = [":", "--", "-"]
colors = ["xkcd:grey", "xkcd:dark grey", "black"]

for i, model, in enumerate([glycolysis, glycolysis_PFK, RBC_PFK]):
    sim = Simulation(model)
    flux_solution = sim.simulate(
        model, time=(t0, tf, tf*10 + 1),
        perturbations={"kf_ATPM": "kf_ATPM * 1.25"})[1]

    model_fluxes_in = [r for r in fluxes_in if r in model.reactions]
    if model.id == "Glycolysis":
        model_fluxes_in += ["PFK"]
    model_fluxes_out = [r for r in fluxes_out if r in model.reactions]

    for flux_id, variables in zip(["Net_Flux_In", "Net_Flux_Out"],
                                  [model_fluxes_in, model_fluxes_out]):
        flux_solution.make_aggregate_solution(
            flux_id, equation=" + ".join(variables), variables=variables)

    plot_phase_portrait(
        flux_solution, x="Net_Flux_In", y="Net_Flux_Out", ax=ax4,
        legend=[model.id, "lower right"],
        xlim=(6, 9), ylim=(6, 9),
        xlabel="Fluxes in [mm/Hr]", ylabel="Fluxes out [mm/Hr]",
        title=("(d) Phase Portrait of Fluxes", L_FONT),
        color=colors[i], linestyle=linestyles[i],
        annotate_time_points=time_points,
        annotate_time_points_color=time_point_colors,
        annotate_time_points_legend="best");
fig_14_12.tight_layout()
```

**Figure 14.12:** The time profiles of the (a) proton concentration, (b) the fluxes that make protons, (c) the fluxes that use protons and (d) the phase portrait of the net flux in and net flux out (darker red colors indicate slower time scales).

```
[34]:
```

```
fig_14_13, ax = plt.subplots(nrows=1, ncols=1, figsize=(5, 5))

for i, model, in enumerate([glycolysis, glycolysis_PFK, RBC_PFK]):
    sim = Simulation(model)
    flux_solution = sim.simulate(
        model, time=(t0, tf, tf*10 + 1),
        perturbations={"kf_ATPM": "kf_ATPM * 1.25"})[1]

    plot_phase_portrait(
        flux_solution, x="ATPM", y="SK_h_c", ax=ax,
        legend=(model.id, "right outside"),
        xlabel="ATPM [mm/Hr]", ylabel="SK_h_c [mm/Hr]",
        xlim=(1, 3.), ylim=(2.5, 4),
        title=("Phase Portrait of ATPM vs. SK_h_c", L_FONT),
        color=colors[i], linestyle=linestyles[i],
        annotate_time_points=time_points,
        annotate_time_points_color=time_point_colors,
        annotate_time_points_legend="best")
fig_14_13.tight_layout()
```

**Figure 14.13:** A phase portrait of the instantly imbalanced ATP demand flux and the proton exchange rate with the core network coupled to the PFK sub-network (black line), and the glycolytic network with (dark grey dashed line) and without (ligh grey dashed line) coupling of the PFK sub-network.

###### Key fluxes and pairwise phase portraits: PFK with RBC metabolic network¶

Here we examine the key fluxes through the PFK enzyme and in the integrated model.

```
[35]:
```

```
PFK_module = RBC_PFK.enzyme_modules.PFK

# Create equations for flux through various enzyme module form groups
equations_dict = {}
for i in range(0, 5):
    PFK_rxns = ["PFK_R{0:d}{1:d}".format(i, j) for j in range(1, 4)]
    equations_dict["R{0:d}i".format(i)] = [" + ".join(PFK_rxns),
                                           PFK_rxns]
# Obtain reactions responsible for catalysis
catalyzation_rxns = PFK_module.enzyme_module_reactions_categorized.get_by_id("catalyzation")
catalyzation_rxns = [m.id for m in catalyzation_rxns.members]

# Create equation for net flux through enzyme
equations_dict["PFK"] = [" + ".join(catalyzation_rxns),
                         catalyzation_rxns]

# Create flux solutions for equations
for flux_id, (equation, variables) in equations_dict.items():
    flux_sol.make_aggregate_solution(
        flux_id, equation=equation, variables=variables)

fig_14_14, axes = plt.subplots(nrows=2, ncols=1, figsize=(8, 6))
(ax1, ax2) = axes.flatten()

plot_time_profile(
    flux_sol, observable=list(equations_dict)[:-1], ax=ax1,
    legend="right outside", plot_function="loglog",
    xlim=(1e-6, tf), ylim=(1e-2, 1e1),
    xlabel="Time [hr]", ylabel="Flux [mM/hr]",
    title=("(a) Fluxes through R-States", L_FONT));

for i, model, in enumerate([glycolysis, glycolysis_PFK, RBC_PFK]):
    sim = Simulation(model)
    flux_solution = sim.simulate(
        model, time=(t0, tf, tf*10 + 1),
        perturbations={"kf_ATPM": "kf_ATPM * 1.25"})[1]

    if i > 0:
        # Create flux solutions for equations
        flux_solution.make_aggregate_solution(
            "PFK", equation=equations_dict["PFK"][0],
            variables=equations_dict["PFK"][1])

    plot_time_profile(
        flux_solution, observable="PFK", ax=ax2,
        legend=[model.id, "right outside"], plot_function="semilogx",
        xlim=(1e-6, tf), ylim=(.9, 1.25),
        xlabel="Time [hr]", ylabel="Flux [mM/hr]",
        color=colors[i], linestyle=linestyles[i],
        title=("(b) Net Flux through PFK", L_FONT));
fig_14_14.tight_layout()
```

**Figure 14.14:** (a): The individual fluxes through the R-forms of the PFK enzyme and (b) the PFK flux from the module. The new PFK flux is the sum of all fluxes through the R-forms.

```
[36]:
```

```
fig_14_15, axes = plt.subplots(nrows=2, ncols=2, figsize=(10, 10))
axes = axes.flatten()

time_points = [t0, 1e-1, 1e0, 1e1, 1e2, tf]
time_point_colors = [
    mpl.colors.to_hex(c)
    for c in mpl.cm.Reds(np.linspace(0.3, 1, len(time_points)))]

pairings = [("ATPM", "SK_pyr_c"), ("GAPD", "LDH_L"),
            ("LDH_L", "DM_nadh"), ("HEX1", "PYK")]
xlims = [(1.20, 2.20), (1.90, 2.5), (1.75, 2.25), (1.03, 1.18)]
ylims = [(0.05, 0.35), (1.75, 2.25), (.19,.25), (1.70, 2.55)]

sim = Simulation(RBC_PFK)
flux_sol = sim.simulate(
    RBC_PFK, time=(t0, tf, tf*10 + 1),
    perturbations={"kf_ATPM": "kf_ATPM * 1.25"})[1]

for i, ax in enumerate(axes):
    time_points_legend = None
    if i == len(axes) - 1:
        time_points_legend = "upper right outside"
    x_i, y_i = pairings[i]
    plot_phase_portrait(
        flux_sol, x=x_i, y=y_i, ax=ax,
        xlabel=x_i, ylabel=y_i,
        xlim=xlims[i], ylim=ylims[i],
        title=("Phase Portrait of {0} vs. {1}".format(
            x_i, y_i), L_FONT),
        annotate_time_points=time_points,
        annotate_time_points_color=time_point_colors,
        annotate_time_points_legend=time_points_legend)
fig_14_15.tight_layout()
```

**Figure 14.15:** The dynamic response of key fluxes in glycolysis. Detailed pairwise phase portraits: (a): \(v\_{ATPM}\ \textit{vs.}\ v\_{SK\_{PYR}}\). (b): \(v\_{GAPD}\ \textit{vs.}\ v\_{LDH\_{L}}\). (c): \(v\_{LDH\_{L}}\ \textit{vs.}\ v\_{DM\_{NADH}}\). (d): \(v\_{HEX1}\ \textit{vs.}\ v\_{PYK}\) The fluxes are in units of mM/h. The perturbation is reflected in the instantaneous move of the flux state from the initial steady state to an unsteady state, as indicated by the arrow placing the
initial point at \(t = 0^+\). The system then returns to its steady state at \(t \rightarrow \infty\).

\(\tiny{\text{© B. Ø. Palsson 2011;}\ \text{This publication is in copyright.}\\ \text{Subject to statutory exception and to the provisions of relevant collective licensing agreements,}\\ \text{no reproduction of any part may take place without the written permission of Cambridge University Press.}}\)

#### Glycolysis Model Construction¶

Based on Chapter 10 of [Pal11]

To construct a model of glycolysis, first we import **MASSpy** and other essential packages. Constants used throughout the notebook are also defined.

```
[1]:
```

```
from os import path

import matplotlib.pyplot as plt

from cobra import DictList

from mass import (
    MassConfiguration, MassMetabolite, MassModel,
    MassReaction, Simulation, UnitDefinition)
from mass.io import json, sbml
from mass.util import qcqa_model

mass_config = MassConfiguration()

mass_config.irreversible_Keq = float("inf")
```

##### Model Construction¶

The first step of creating a model of glycolysis is to define the `MassModel`.

```
[2]:
```

```
glycolysis = MassModel("Glycolysis")
```

###### Metabolites¶

The next step is to define all of the metabolites using the `MassMetabolite` object. Some considerations for this step include the following:

1. It is important to use a clear and consistent format for identifiers and names when defining the `MassMetabolite` objects for various reasons, some of which include improvements to model clarity and utility, assurance of unique identifiers (required to add metabolites to the model), and consistency when collaborating and communicating with others.
2. In order to ensure our model is physiologically accurate, it is important to provide the `formula` argument with a string representing the chemical formula for each metabolite, and the `charge` argument with an integer representing the metabolite’s ionic charge (Note that neutrally charged metabolites are provided with 0). These attributes can always be set later if necessary using the `formula` and `charge` attribute set methods.
3. To indicate that the cytosol is the cellular compartment in which the reactions occur, the string “c” is provided to the `compartment` argument.

This model will be created using identifiers and names found in the BiGG Database.

In this model, there are 20 metabolites inside the cytosol compartment.

```
[3]:
```

```
glc__D_c = MassMetabolite(
    "glc__D_c",
    name="D-Glucose",
    formula="C6H12O6",
    charge=0,
    compartment="c",
    fixed=False)

g6p_c = MassMetabolite(
    "g6p_c",
    name="D-Glucose 6-phosphate",
    formula="C6H11O9P",
    charge=-2,
    compartment="c",
    fixed=False)

f6p_c = MassMetabolite(
    "f6p_c",
    name="D-Fructose 6-phosphate",
    formula="C6H11O9P",
    charge=-2,
    compartment="c",
    fixed=False)

fdp_c = MassMetabolite(
    "fdp_c",
    name="D-Fructose 1,6-bisphosphate",
    formula="C6H10O12P2",
    charge=-4,
    compartment="c",
    fixed=False)

dhap_c = MassMetabolite(
    "dhap_c",
    name="Dihydroxyacetone phosphate",
    formula="C3H5O6P",
    charge=-2,
    compartment="c",
    fixed=False)

g3p_c = MassMetabolite(
    "g3p_c",
    name="Glyceraldehyde 3-phosphate",
    formula="C3H5O6P",
    charge=-2,
    compartment="c",
    fixed=False)

_13dpg_c = MassMetabolite(
    "_13dpg_c",
    name="3-Phospho-D-glyceroyl phosphate",
    formula="C3H4O10P2",
    charge=-4,
    compartment="c",
    fixed=False)

_3pg_c = MassMetabolite(
    "_3pg_c",
    name="3-Phospho-D-glycerate",
    formula="C3H4O7P",
    charge=-3,
    compartment="c",
    fixed=False)

_2pg_c = MassMetabolite(
    "_2pg_c",
    name="D-Glycerate 2-phosphate",
    formula="C3H4O7P",
    charge=-3,
    compartment="c",
    fixed=False)

pep_c = MassMetabolite(
    "pep_c",
    name="Phosphoenolpyruvate",
    formula="C3H2O6P",
    charge=-3,
    compartment="c",
    fixed=False)

pyr_c = MassMetabolite(
    "pyr_c",
    name="Pyruvate",
    formula="C3H3O3",
    charge=-1,
    compartment="c",
    fixed=False)

lac__L_c = MassMetabolite(
    "lac__L_c",
    name="L-Lactate",
    formula="C3H5O3",
    charge=-1,
    compartment="c",
    fixed=False)

nad_c = MassMetabolite(
    "nad_c",
    name="Nicotinamide adenine dinucleotide",
    formula="[NAD]-C21H26N7O14P2",
    charge=-1,
    compartment="c",
    fixed=False)

nadh_c = MassMetabolite(
    "nadh_c",
    name="Nicotinamide adenine dinucleotide - reduced",
    formula="[NAD]-C21H27N7O14P2",
    charge=-2,
    compartment="c",
    fixed=False)

atp_c = MassMetabolite(
    "atp_c",
    name="ATP",
    formula="C10H12N5O13P3",
    charge=-4,
    compartment="c",
    fixed=False)

adp_c = MassMetabolite(
    "adp_c",
    name="ADP",
    formula="C10H12N5O10P2",
    charge=-3,
    compartment="c",
    fixed=False)

amp_c = MassMetabolite(
    "amp_c",
    name="AMP",
    formula="C10H12N5O7P",
    charge=-2,
    compartment="c",
    fixed=False)

pi_c = MassMetabolite(
    "pi_c",
    name="Phosphate",
    formula="HPO4",
    charge=-2,
    compartment="c",
    fixed=False)

h_c = MassMetabolite(
    "h_c",
    name="H+",
    formula="H",
    charge=1,
    compartment="c",
    fixed=False)

h2o_c = MassMetabolite(
    "h2o_c",
    name="H2O",
    formula="H2O",
    charge=0,
    compartment="c",
    fixed=False)
```

###### Reactions¶

Once all of the `MassMetabolite` objects for each metabolite, the next step is to define all of the reactions that occur and their stoichiometry.

1. As with the metabolites, it is also important to use a clear and consistent format for identifiers and names when defining when defining the `MassReaction` objects.
2. To make this model useful for integration with other models, it is important to provide a string to the `subsystem` argument. By providing the subsystem, the reactions can be easily obtained even when integrated with a significantly larger model through the `subsystem` attribute.
3. After the creation of each `MassReaction` object, the metabolites are added to the reaction using a dictionary where keys are the `MassMetabolite` objects and values are the stoichiometric coefficients (reactants have negative coefficients, products have positive ones).

This model will be created using identifiers and names found in the BiGG Database.

In this model, there are 14 reactions occuring inside the cytosol compartment.

```
[4]:
```

```
HEX1 = MassReaction(
    "HEX1",
    name="Hexokinase (D-glucose:ATP)",
    subsystem=glycolysis.id,
    reversible=True)
HEX1.add_metabolites({
    glc__D_c: -1,
    atp_c: -1,
    adp_c: 1,
    g6p_c: 1,
    h_c: 1})

PGI = MassReaction(
    "PGI",
    name="Glucose-6-phosphate isomerase",
    subsystem=glycolysis.id,
    reversible=True)
PGI.add_metabolites({
    g6p_c: -1,
    f6p_c: 1})

PFK = MassReaction(
    "PFK",
    name="Phosphofructokinase",
    subsystem=glycolysis.id,
    reversible=True)
PFK.add_metabolites({
    f6p_c: -1,
    atp_c: -1,
    fdp_c: 1,
    adp_c: 1,
    h_c: 1})

FBA = MassReaction(
    "FBA",
    name="Fructose-bisphosphate aldolase",
    subsystem=glycolysis.id,
    reversible=True)
FBA.add_metabolites({
    fdp_c: -1,
    dhap_c: 1,
    g3p_c: 1})

TPI = MassReaction(
    "TPI",
    name="Triose-phosphate isomerase",
    subsystem=glycolysis.id,
    reversible=True)
TPI.add_metabolites({
    dhap_c: -1,
    g3p_c: 1})

GAPD = MassReaction(
    "GAPD",
    name="Glyceraldehyde-3-phosphate dehydrogenase",
    subsystem=glycolysis.id,
    reversible=True)
GAPD.add_metabolites({
    g3p_c: -1,
    nad_c: -1,
    pi_c: -1,
    _13dpg_c: 1,
    h_c: 1,
    nadh_c: 1})

PGK = MassReaction(
    "PGK",
    name="Phosphoglycerate kinase",
    subsystem=glycolysis.id,
    reversible=True)
PGK.add_metabolites({
    _13dpg_c: -1,
    adp_c: -1,
    _3pg_c: 1,
    atp_c: 1})

PGM = MassReaction(
    "PGM",
    name="Phosphoglycerate mutase",
    subsystem=glycolysis.id,
    reversible=True)
PGM.add_metabolites({
    _3pg_c: -1,
    _2pg_c: 1})

ENO = MassReaction(
    "ENO",
    name="Enolase",
    subsystem=glycolysis.id,
    reversible=True)
ENO.add_metabolites({
    _2pg_c: -1,
    h2o_c: 1,
    pep_c: 1})

PYK = MassReaction(
    "PYK",
    name="Pyruvate kinase",
    subsystem=glycolysis.id,
    reversible=True)
PYK.add_metabolites({
    pep_c: -1,
    h_c: -1,
    adp_c: -1,
    atp_c: 1,
    pyr_c: 1})

LDH_L = MassReaction(
    "LDH_L",
    name="L-lactate dehydrogenase",
    subsystem=glycolysis.id,
    reversible=True)
LDH_L.add_metabolites({
    h_c: -1,
    nadh_c: -1,
    pyr_c: -1,
    lac__L_c: 1,
    nad_c: 1})

ADK1 = MassReaction(
    "ADK1",
    name="Adenylate kinase",
    subsystem="Misc.",
    reversible=True)
ADK1.add_metabolites({
    adp_c: -2,
    amp_c: 1,
    atp_c: 1})

ATPM = MassReaction(
    "ATPM",
    name="ATP maintenance requirement",
    subsystem="Pseudoreaction",
    reversible=False)
ATPM.add_metabolites({
    atp_c: -1,
    h2o_c: -1,
    adp_c: 1,
    h_c: 1,
    pi_c: 1})

DM_nadh = MassReaction(
    "DM_nadh",
    name="Demand NADH",
    subsystem="Pseudoreaction",
    reversible=False)
DM_nadh.add_metabolites({
    nadh_c: -1,
    nad_c: 1,
    h_c: 1})
```

After generating the reactions, all reactions are added to the model through the `MassModel.add_reactions` class method. Adding the `MassReaction` objects will also add their associated `MassMetabolite` objects if they have not already been added to the model.

```
[5]:
```

```
glycolysis.add_reactions([
    HEX1, PGI, PFK, FBA, TPI, GAPD, PGK,
    PGM, ENO, PYK, LDH_L, ADK1, ATPM, DM_nadh])

for reaction in glycolysis.reactions:
    print(reaction)
```

```
HEX1: atp_c + glc__D_c <=> adp_c + g6p_c + h_c
PGI: g6p_c <=> f6p_c
PFK: atp_c + f6p_c <=> adp_c + fdp_c + h_c
FBA: fdp_c <=> dhap_c + g3p_c
TPI: dhap_c <=> g3p_c
GAPD: g3p_c + nad_c + pi_c <=> _13dpg_c + h_c + nadh_c
PGK: _13dpg_c + adp_c <=> _3pg_c + atp_c
PGM: _3pg_c <=> _2pg_c
ENO: _2pg_c <=> h2o_c + pep_c
PYK: adp_c + h_c + pep_c <=> atp_c + pyr_c
LDH_L: h_c + nadh_c + pyr_c <=> lac__L_c + nad_c
ADK1: 2 adp_c <=> amp_c + atp_c
ATPM: atp_c + h2o_c --> adp_c + h_c + pi_c
DM_nadh: nadh_c --> h_c + nad_c
```

###### Boundary reactions¶

After generating the reactions, the next step is to add the boundary reactions and boundary conditions (the concentrations of the boundary ‘metabolites’ of the system). This can easily be done using the `MassModel.add_boundary` method. With the generation of the boundary reactions, the system becomes an open system, allowing for the flow of mass through the biochemical pathways of the model. Once added, the model will be able to return the boundary conditions as a dictionary through the
`MassModel.boundary_conditions` attribute.

All boundary reactions are originally created with the metabolite as the reactant. However, there are times where it would be preferable to represent the metabolite as the product. For these situtations, the `MassReaction.reverse_stoichiometry` method can be used with its `inplace` argument to create a new `MassReaction` or simply reverse the stoichiometry for the current `MassReaction.`

In this model, there are 7 boundary reactions that must be defined.

```
[6]:
```

```
SK_glc__D_c = glycolysis.add_boundary(
    metabolite=glc__D_c, boundary_type="sink", subsystem="Pseudoreaction",
    boundary_condition=1)
SK_glc__D_c.reverse_stoichiometry(inplace=True)

SK_lac__L_c = glycolysis.add_boundary(
    metabolite=lac__L_c, boundary_type="sink", subsystem="Pseudoreaction",
    boundary_condition=1)

SK_pyr_c = glycolysis.add_boundary(
    metabolite=pyr_c, boundary_type="sink", subsystem="Pseudoreaction",
    boundary_condition=0.06)

SK_h_c = glycolysis.add_boundary(
    metabolite=h_c, boundary_type="sink", subsystem="Pseudoreaction",
    boundary_condition=6.30957e-05)

SK_h2o_c = glycolysis.add_boundary(
    metabolite=h2o_c, boundary_type="sink", subsystem="Pseudoreaction",
    boundary_condition=1)

SK_amp_c = glycolysis.add_boundary(
    metabolite=amp_c, boundary_type="sink", subsystem="Pseudoreaction",
    boundary_condition=1)
SK_amp_c.reverse_stoichiometry(inplace=True)

DM_amp_c = glycolysis.add_boundary(
    metabolite=amp_c, boundary_type="demand", subsystem="Pseudoreaction",
    boundary_condition=1)

print("Boundary Reactions and Values\n-----------------------------")
for reaction in glycolysis.boundary:
    boundary_met = reaction.boundary_metabolite
    bc_value = glycolysis.boundary_conditions.get(boundary_met)
    print("{0}\n{1}: {2}\n".format(
        reaction, boundary_met, bc_value))
```

```
Boundary Reactions and Values
-----------------------------
SK_glc__D_c:  <=> glc__D_c
glc__D_b: 1.0

SK_lac__L_c: lac__L_c <=>
lac__L_b: 1.0

SK_pyr_c: pyr_c <=>
pyr_b: 0.06

SK_h_c: h_c <=>
h_b: 6.30957e-05

SK_h2o_c: h2o_c <=>
h2o_b: 1.0

SK_amp_c:  <=> amp_c
amp_b: 1.0

DM_amp_c: amp_c -->
amp_b: 1.0
```

###### Ordering of internal species and reactions¶

Sometimes, it is also desirable to reorder the metabolite and reaction objects inside the model to follow the physiology. To reorder the internal objects, one can use `cobra.DictList` containers and the `DictList.get_by_any` method with the list of object identifiers in the desirable order. To ensure all objects are still present and not forgotten in the model, a small QA check is also performed.

```
[7]:
```

```
new_metabolite_order = [
    "glc__D_c", "g6p_c", "f6p_c", "fdp_c", "dhap_c",
    "g3p_c", "_13dpg_c", "_3pg_c", "_2pg_c", "pep_c",
    "pyr_c", "lac__L_c", "nad_c", "nadh_c", "amp_c",
    "adp_c", "atp_c", "pi_c", "h_c", "h2o_c"]

if len(glycolysis.metabolites) == len(new_metabolite_order):
    glycolysis.metabolites = DictList(
        glycolysis.metabolites.get_by_any(new_metabolite_order))

new_reaction_order = [
    "HEX1", "PGI", "PFK", "FBA", "TPI",
    "GAPD", "PGK", "PGM", "ENO", "PYK",
    "LDH_L", "DM_amp_c", "ADK1", "SK_pyr_c",
    "SK_lac__L_c", "ATPM", "DM_nadh", "SK_glc__D_c",
    "SK_amp_c", "SK_h_c", "SK_h2o_c"]

if len(glycolysis.reactions) == len(new_reaction_order):
    glycolysis.reactions = DictList(
        glycolysis.reactions.get_by_any(new_reaction_order))

glycolysis.update_S(array_type="DataFrame", dtype=int)
```

```
[7]:
```

|  | HEX1 | PGI | PFK | FBA | TPI | GAPD | PGK | PGM | ENO | PYK | ... | DM\_amp\_c | ADK1 | SK\_pyr\_c | SK\_lac\_\_L\_c | ATPM | DM\_nadh | SK\_glc\_\_D\_c | SK\_amp\_c | SK\_h\_c | SK\_h2o\_c |
| --- | --- | --- | --- | --- | --- | --- | --- | --- | --- | --- | --- | --- | --- | --- | --- | --- | --- | --- | --- | --- | --- |
| glc\_\_D\_c | -1 | 0 | 0 | 0 | 0 | 0 | 0 | 0 | 0 | 0 | ... | 0 | 0 | 0 | 0 | 0 | 0 | 1 | 0 | 0 | 0 |
| g6p\_c | 1 | -1 | 0 | 0 | 0 | 0 | 0 | 0 | 0 | 0 | ... | 0 | 0 | 0 | 0 | 0 | 0 | 0 | 0 | 0 | 0 |
| f6p\_c | 0 | 1 | -1 | 0 | 0 | 0 | 0 | 0 | 0 | 0 | ... | 0 | 0 | 0 | 0 | 0 | 0 | 0 | 0 | 0 | 0 |
| fdp\_c | 0 | 0 | 1 | -1 | 0 | 0 | 0 | 0 | 0 | 0 | ... | 0 | 0 | 0 | 0 | 0 | 0 | 0 | 0 | 0 | 0 |
| dhap\_c | 0 | 0 | 0 | 1 | -1 | 0 | 0 | 0 | 0 | 0 | ... | 0 | 0 | 0 | 0 | 0 | 0 | 0 | 0 | 0 | 0 |
| g3p\_c | 0 | 0 | 0 | 1 | 1 | -1 | 0 | 0 | 0 | 0 | ... | 0 | 0 | 0 | 0 | 0 | 0 | 0 | 0 | 0 | 0 |
| \_13dpg\_c | 0 | 0 | 0 | 0 | 0 | 1 | -1 | 0 | 0 | 0 | ... | 0 | 0 | 0 | 0 | 0 | 0 | 0 | 0 | 0 | 0 |
| \_3pg\_c | 0 | 0 | 0 | 0 | 0 | 0 | 1 | -1 | 0 | 0 | ... | 0 | 0 | 0 | 0 | 0 | 0 | 0 | 0 | 0 | 0 |
| \_2pg\_c | 0 | 0 | 0 | 0 | 0 | 0 | 0 | 1 | -1 | 0 | ... | 0 | 0 | 0 | 0 | 0 | 0 | 0 | 0 | 0 | 0 |
| pep\_c | 0 | 0 | 0 | 0 | 0 | 0 | 0 | 0 | 1 | -1 | ... | 0 | 0 | 0 | 0 | 0 | 0 | 0 | 0 | 0 | 0 |
| pyr\_c | 0 | 0 | 0 | 0 | 0 | 0 | 0 | 0 | 0 | 1 | ... | 0 | 0 | -1 | 0 | 0 | 0 | 0 | 0 | 0 | 0 |
| lac\_\_L\_c | 0 | 0 | 0 | 0 | 0 | 0 | 0 | 0 | 0 | 0 | ... | 0 | 0 | 0 | -1 | 0 | 0 | 0 | 0 | 0 | 0 |
| nad\_c | 0 | 0 | 0 | 0 | 0 | -1 | 0 | 0 | 0 | 0 | ... | 0 | 0 | 0 | 0 | 0 | 1 | 0 | 0 | 0 | 0 |
| nadh\_c | 0 | 0 | 0 | 0 | 0 | 1 | 0 | 0 | 0 | 0 | ... | 0 | 0 | 0 | 0 | 0 | -1 | 0 | 0 | 0 | 0 |
| amp\_c | 0 | 0 | 0 | 0 | 0 | 0 | 0 | 0 | 0 | 0 | ... | -1 | 1 | 0 | 0 | 0 | 0 | 0 | 1 | 0 | 0 |
| adp\_c | 1 | 0 | 1 | 0 | 0 | 0 | -1 | 0 | 0 | -1 | ... | 0 | -2 | 0 | 0 | 1 | 0 | 0 | 0 | 0 | 0 |
| atp\_c | -1 | 0 | -1 | 0 | 0 | 0 | 1 | 0 | 0 | 1 | ... | 0 | 1 | 0 | 0 | -1 | 0 | 0 | 0 | 0 | 0 |
| pi\_c | 0 | 0 | 0 | 0 | 0 | -1 | 0 | 0 | 0 | 0 | ... | 0 | 0 | 0 | 0 | 1 | 0 | 0 | 0 | 0 | 0 |
| h\_c | 1 | 0 | 1 | 0 | 0 | 1 | 0 | 0 | 0 | -1 | ... | 0 | 0 | 0 | 0 | 1 | 1 | 0 | 0 | -1 | 0 |
| h2o\_c | 0 | 0 | 0 | 0 | 0 | 0 | 0 | 0 | 1 | 0 | ... | 0 | 0 | 0 | 0 | -1 | 0 | 0 | 0 | 0 | -1 |

20 rows × 21 columns

##### Model Parameterization¶

###### Steady State fluxes¶

Steady state fluxes can be computed as a summation of the MinSpan pathway vectors. Pathways are obtained using MinSpan.

Using these pathways and literature sources, independent fluxes can be defined in order to calculate the steady state flux vector. For this model, flux and concentration data are obtained from the historical human red blood cell (RBC) metabolic model (Heinrich 1977, Jamshidi 2002, Joshi 1988, Joshi 1990, Joshi 1989-II).

From the literature, it is known that:

1. A typical uptake rate of the RBC of glucose is about 1.12 mM/hr.
2. Based on experimental data, the steady state load on NADH is set at 20% of the glucose uptake rate, or \(0.2 \* 1.12 = 0.244\).
3. The synthesis rate of AMP is measured to be 0.014 mM/hr.

With these pathays and numerical values, the steady state flux vector can be computed as the weighted sum of the corresponding basis vectors. The steady state flux vector is computed as an inner product:

```
[8]:
```

```
minspan_paths = [
    [1, 1, 1, 1, 1, 2, 2, 2, 2, 2, 2, 0, 0, 0, 2, 2, 0, 1, 0, 2, 0],
    [0, 0, 0, 0, 0, 0, 0, 0, 0, 0, -1, 0, 0, 1, -1, 0, 1, 0, 0, 2, 0],
    [0, 0, 0, 0, 0, 0, 0, 0, 0, 0, 0, 1, 0, 0, 0, 0, 0, 0, 1, 0, 0]]
glycolysis.compute_steady_state_fluxes(
    pathways=minspan_paths,
    independent_fluxes={
        SK_glc__D_c: 1.12,
        DM_nadh: .2 * 1.12,
        DM_amp_c: 0.014},
    update_reactions=True)

print("Steady State Fluxes\n-------------------")
for reaction, steady_state_flux in glycolysis.steady_state_fluxes.items():
    print("{0}: {1:.6f}".format(reaction.flux_symbol_str, steady_state_flux))
```

```
Steady State Fluxes
-------------------
v_HEX1: 1.120000
v_PGI: 1.120000
v_PFK: 1.120000
v_FBA: 1.120000
v_TPI: 1.120000
v_GAPD: 2.240000
v_PGK: 2.240000
v_PGM: 2.240000
v_ENO: 2.240000
v_PYK: 2.240000
v_LDH_L: 2.016000
v_DM_amp_c: 0.014000
v_ADK1: 0.000000
v_SK_pyr_c: 0.224000
v_SK_lac__L_c: 2.016000
v_ATPM: 2.240000
v_DM_nadh: 0.224000
v_SK_glc__D_c: 1.120000
v_SK_amp_c: 0.014000
v_SK_h_c: 2.688000
v_SK_h2o_c: 0.000000
```

###### Initial Conditions¶

Once the network has been built, the concentrations can be added to the metabolites. These concentrations are also treated as the initial conditions required to integrate and simulate the model’s ordinary differential equations (ODEs). The metabolite concentrations are added to each individual metabolite using the `MassMetabolite.initial_condition` (alias: `MassMetabolite.ic`) attribute setter methods. Once added, the model will be able to return the initial conditions as a dictionary
through the `MassModel.initial_conditions` attribute.

```
[9]:
```

```
glc__D_c.ic = 1.0
g6p_c.ic = 0.0486
f6p_c.ic = 0.0198
fdp_c.ic = 0.0146
g3p_c.ic = 0.00728
dhap_c.ic = 0.16
_13dpg_c.ic = 0.000243
_3pg_c.ic = 0.0773
_2pg_c.ic = 0.0113
pep_c.ic = 0.017
pyr_c.ic = 0.060301
lac__L_c.ic = 1.36
atp_c.ic = 1.6
adp_c.ic = 0.29
amp_c.ic = 0.0867281
h_c.ic = 0.0000899757
nad_c.ic = 0.0589
nadh_c.ic = 0.0301
pi_c.ic = 2.5
h2o_c.ic = 1.0

print("Initial Conditions\n------------------")
for metabolite, ic_value in glycolysis.initial_conditions.items():
    print("{0}: {1}".format(metabolite, ic_value))
```

```
Initial Conditions
------------------
glc__D_c: 1.0
g6p_c: 0.0486
f6p_c: 0.0198
fdp_c: 0.0146
dhap_c: 0.16
g3p_c: 0.00728
_13dpg_c: 0.000243
_3pg_c: 0.0773
_2pg_c: 0.0113
pep_c: 0.017
pyr_c: 0.060301
lac__L_c: 1.36
nad_c: 0.0589
nadh_c: 0.0301
amp_c: 0.0867281
adp_c: 0.29
atp_c: 1.6
pi_c: 2.5
h_c: 8.99757e-05
h2o_c: 1.0
```

###### Equilibirum Constants¶

After adding initial conditions and steady state fluxes, the equilibrium constants are defined using the `MassReaction.equilibrium_constant` (alias: `MassReaction.Keq`) setter method.

```
[10]:
```

```
HEX1.Keq = 850
PGI.Keq = 0.41
PFK.Keq = 310
FBA.Keq = 0.082
TPI.Keq = 0.0571429
GAPD.Keq = 0.0179
PGK.Keq = 1800
PGM.Keq = 0.147059
ENO.Keq = 1.69492
PYK.Keq = 363000
LDH_L.Keq = 26300
ADK1.Keq = 1.65

SK_glc__D_c.Keq = mass_config.irreversible_Keq
SK_lac__L_c.Keq = 1
SK_pyr_c.Keq = 1
SK_h_c.Keq = 1
SK_h2o_c.Keq = 1
SK_amp_c.Keq = mass_config.irreversible_Keq

print("Equilibrium Constants\n---------------------")
for reaction in glycolysis.reactions:
    print("{0}: {1}".format(reaction.Keq_str, reaction.Keq))
```

```
Equilibrium Constants
---------------------
Keq_HEX1: 850
Keq_PGI: 0.41
Keq_PFK: 310
Keq_FBA: 0.082
Keq_TPI: 0.0571429
Keq_GAPD: 0.0179
Keq_PGK: 1800
Keq_PGM: 0.147059
Keq_ENO: 1.69492
Keq_PYK: 363000
Keq_LDH_L: 26300
Keq_DM_amp_c: inf
Keq_ADK1: 1.65
Keq_SK_pyr_c: 1
Keq_SK_lac__L_c: 1
Keq_ATPM: inf
Keq_DM_nadh: inf
Keq_SK_glc__D_c: inf
Keq_SK_amp_c: inf
Keq_SK_h_c: 1
Keq_SK_h2o_c: 1
```

###### Calculation of PERCs¶

By defining the equilibrium constant and steady state parameters, the values of the pseudo rate constants (PERCs) can be calculated and added to the model using the `MassModel.calculate_PERCs` method.

```
[11]:
```

```
glycolysis.calculate_PERCs(update_reactions=True)

print("Forward Rate Constants\n----------------------")
for reaction in glycolysis.reactions:
    print("{0}: {1:.6f}".format(reaction.kf_str, reaction.kf))
```

```
Forward Rate Constants
----------------------
kf_HEX1: 0.700007
kf_PGI: 3644.444444
kf_PFK: 35.368784
kf_FBA: 2834.567901
kf_TPI: 34.355728
kf_GAPD: 3376.749242
kf_PGK: 1273531.269741
kf_PGM: 4868.589299
kf_ENO: 1763.740525
kf_PYK: 454.385552
kf_LDH_L: 1112.573989
kf_DM_amp_c: 0.161424
kf_ADK1: 100000.000000
kf_SK_pyr_c: 744.186047
kf_SK_lac__L_c: 5.600000
kf_ATPM: 1.400000
kf_DM_nadh: 7.441860
kf_SK_glc__D_c: 1.120000
kf_SK_amp_c: 0.014000
kf_SK_h_c: 100000.000000
kf_SK_h2o_c: 100000.000000
```

##### QC/QA Model¶

Before simulating the model, it is important to ensure that the model is elementally balanced, and that the model can simulate. Therefore, the `qcqa_model` function from the `mass.util.qcqa` submodule is used to provide a report on the model quality and indicate whether simulation is possible and if not, what parameters and/or initial conditions are missing.

Generally, pseudoreactions (e.g. boundary exchanges, sinks, demands) are not elementally balanced. The `qcqa_model` function does not include elemental balancing of boundary reactions. However, some models contain pseudoreactions reprsenting a simplified mechanism, and show up in the returned report. The elemental imbalance of these pseudoreactions is therefore expected in certain reaction and should not be a cause for concern.

In this model of glycolysis, the NADH demand reaction is a simplified pseudoreaction that generates electrons for certain redox processes and is not expected to be balanced.

```
[12]:
```

```
qcqa_model(glycolysis, parameters=True, concentrations=True,
           fluxes=True, superfluous=True, elemental=True)
```

```
╒══════════════════════════════════════════════╕
│ MODEL ID: Glycolysis                         │
│ SIMULATABLE: True                            │
│ PARAMETERS NUMERICALY CONSISTENT: True       │
╞══════════════════════════════════════════════╡
│ ============================================ │
│             CONSISTENCY CHECKS               │
│ ============================================ │
│ Elemental                                    │
│ ----------------------                       │
│ DM_nadh: {charge: 2.0}                       │
│ ============================================ │
╘══════════════════════════════════════════════╛
```

From the results of the QC/QA test, it can be seen that the model can be simulated and is numerically consistent.

##### Steady State and Model Validation¶

To find the steady state of the model and perform simulations, the model must first be loaded into a `Simulation`. In order to load a model into a `Simulation`, the model must be simulatable, meaning there are no missing numerical values that would prevent the integration of the ODEs that comprise the model. The `verbose` argument can be used while loading a model to produce a message indicating the successful loading of a model, or why a model could not load.

Once loaded into a `Simulation`, the `find_steady_state` method can be used with the `update_values` argument in order to update the initial conditions and fluxes of the model to a steady state (if necessary). The model can be simulated using the `simulate` method by passing the model to simulate, and a tuple containing the start time and the end time. The number of time points can also be included, but is optional.

After a successful simulation, two `MassSolution` objects are returned. The first `MassSolution` contains the concentration results of the simulation, and the second contains the flux results of the simulation.

To visually validate the steady state of the model, concentration and flux solutions can be plotted using the `plot_time_profile` function from `mass.visualization`. Alternatively, the `MassSolution.view_time_profile` property can be used to quickly generate a time profile for the results.

```
[13]:
```

```
# Setup simulation object
sim = Simulation(glycolysis, verbose=True)
# Simulate from 0 to 1000 with 10001 points in the output
conc_sol, flux_sol = sim.simulate(glycolysis, time=(0, 1e3, 1e4 + 1))
# Quickly render and display time profiles
conc_sol.view_time_profile()
```

```
Successfully loaded MassModel 'Glycolysis' into RoadRunner.
```

###### Storing information and references¶

###### Compartment¶

Because the character “c” represents the cytosol compartment, it is recommended to define and set the compartment in the `MassModel.compartments` attribute.

```
[14]:
```

```
glycolysis.compartments = {"c": "Cytosol"}
print(glycolysis.compartments)
```

```
{'c': 'Cytosol'}
```

###### Units¶

All of the units for the numerical values used in this model are “Millimoles” for amount and “Liters” for volume (giving a concentration unit of ‘Millimolar’), and “Hours” for time. In order to ensure that future users understand the numerical values for model, it is important to define the `MassModel.units` attribute.

The `MassModel.units` is a `cobra.DictList` that contains only `UnitDefinition` objects from the `mass.core.unit` submodule. Each `UnitDefinition` is created from `Unit` objects representing the base units that comprise the `UnitDefinition`. These `Units` are stored in the `list_of_units` attribute. Pre-built units can be viewed using the `print_defined_unit_values` function from the `mass.core.unit` submodule. Alternatively, custom units can also be created using the
`UnitDefinition.create_unit` function. For more information about units, please see the module docstring for `mass.core.unit` submodule.

**Note:** It is important to note that this attribute will NOT track units, but instead acts as a reference for the user and others so that they can perform necessary unit conversions.

```
[15]:
```

```
# Using pre-build units to define UnitDefinitions
concentration = UnitDefinition("mM", name="Millimolar",
                               list_of_units=["millimole", "per_litre"])
time = UnitDefinition("hr", name="hour", list_of_units=["hour"])

# Add units to model
glycolysis.add_units([concentration, time])
print(glycolysis.units)
```

```
[<UnitDefinition Millimolar "mM" at 0x7f9b8534dd90>, <UnitDefinition hour "hr" at 0x7f9b8534de10>]
```

##### Export¶

After validation, the model is ready to be saved. The model can either be exported as a “.json” file or as an “.sbml” (“.xml”) file using their repsective submodules in `mass.io`.

To export the model, only the path to the directory and the model object itself need to be specified.

###### Export using SBML¶

```
[16]:
```

```
sbml.write_sbml_model(mass_model=glycolysis, filename="SB2_" + glycolysis.id + ".xml")
```

###### Export using JSON¶

```
[17]:
```

```
json.save_json_model(mass_model=glycolysis, filename="SB2_" + glycolysis.id + ".json")
```

#### Pentose Phosphate Pathway Model Construction¶

Based on Chapter 11 of [Pal11]

To construct a model of the pentose phosphate pathway (PPP), first we import **MASSpy** and other essential packages. Constants used throughout the notebook are also defined.

```
[1]:
```

```
from os import path

import matplotlib.pyplot as plt

from cobra import DictList

from mass import (
    MassConfiguration, MassMetabolite, MassModel,
    MassReaction, Simulation, UnitDefinition)
from mass.io import json, sbml
from mass.util import qcqa_model

mass_config = MassConfiguration()

mass_config.irreversible_Keq = float("inf")
```

##### Model Construction¶

The first step of creating a model of the PPP is to define the `MassModel`.

```
[2]:
```

```
ppp = MassModel("PentosePhosphatePathway")
```

###### Metabolites¶

The next step is to define all of the metabolites using the `MassMetabolite` object. Some considerations for this step include the following:

1. It is important to use a clear and consistent format for identifiers and names when defining the `MassMetabolite` objects for various reasons, some of which include improvements to model clarity and utility, assurance of unique identifiers (required to add metabolites to the model), and consistency when collaborating and communicating with others.
2. In order to ensure our model is physiologically accurate, it is important to provide the `formula` argument with a string representing the chemical formula for each metabolite, and the `charge` argument with an integer representing the metabolite’s ionic charge (Note that neutrally charged metabolites are provided with 0). These attributes can always be set later if necessary using the `formula` and `charge` attribute set methods.
3. To indicate that the cytosol is the cellular compartment in which the reactions occur, the string “c” is provided to the `compartment` argument.

This model will be created using identifiers and names found in the BiGG Database.

In this model, there are 17 metabolites inside the cytosol compartment.

```
[3]:
```

```
g6p_c = MassMetabolite(
    "g6p_c",
    name="D-Glucose 6-phosphate",
    formula="C6H11O9P",
    charge=-2,
    compartment="c",
    fixed=False)

_6pgl_c = MassMetabolite(
    "_6pgl_c",
    name="6-Phospho-D-gluco-1,5-lactone",
    formula="C6H9O9P",
    charge=-2,
    compartment="c",
    fixed=False)

_6pgc_c = MassMetabolite(
    "_6pgc_c",
    name="6-Phospho-D-gluconate",
    formula="C6H10O10P",
    charge=-3,
    compartment="c",
    fixed=False)

ru5p__D_c = MassMetabolite(
    "ru5p__D_c",
    name="D-Ribulose 5-phosphate",
    formula="C5H9O8P",
    charge=-2,
    compartment="c",
    fixed=False)

r5p_c = MassMetabolite(
    "r5p_c",
    name="Alpha-D-Ribose 5-phosphate",
    formula="C5H9O8P",
    charge=-2,
    compartment="c",
    fixed=False)

xu5p__D_c = MassMetabolite(
    "xu5p__D_c",
    name="D-Xylulose 5-phosphate",
    formula="C5H9O8P",
    charge=-2,
    compartment="c",
    fixed=False)

g3p_c = MassMetabolite(
    "g3p_c",
    name="Glyceraldehyde 3-phosphate",
    formula="C3H5O6P",
    charge=-2,
    compartment="c",
    fixed=False)

s7p_c = MassMetabolite(
    "s7p_c",
    name="Sedoheptulose 7-phosphate",
    formula="C7H13O10P",
    charge=-2,
    compartment="c",
    fixed=False)

f6p_c = MassMetabolite(
    "f6p_c",
    name="D-Fructose 6-phosphate",
    formula="C6H11O9P",
    charge=-2,
    compartment="c",
    fixed=False)

e4p_c = MassMetabolite(
    "e4p_c",
    name="D-Erythrose 4-phosphate",
    formula="C4H7O7P",
    charge=-2,
    compartment="c",
    fixed=False)

h_c = MassMetabolite(
    "h_c",
    name="H+",
    formula="H",
    charge=1,
    compartment="c",
    fixed=False)

nadp_c = MassMetabolite(
    "nadp_c",
    name="Nicotinamide adenine dinucleotide phosphate",
    formula="[NADP]-C21H25N7O17P3",
    charge=-3,
    compartment="c",
    fixed=False)

nadph_c = MassMetabolite(
    "nadph_c",
    name="Nicotinamide adenine dinucleotide phosphate - reduced",
    formula="[NADP]-C21H26N7O17P3",
    charge=-4,
    compartment="c",
    fixed=False)

h2o_c = MassMetabolite(
    "h2o_c",
    name="H2O",
    formula="H2O",
    charge=0,
    compartment="c",
    fixed=False)

gthox_c = MassMetabolite(
    "gthox_c",
    name="Oxidized glutathione",
    formula="C20H30N6O12S2",
    charge=-2,
    compartment="c",
    fixed=False)

gthrd_c = MassMetabolite(
    "gthrd_c",
    name="Reduced glutathione",
    formula="C10H16N3O6S",
    charge=-1,
    compartment="c",
    fixed=False)

co2_c = MassMetabolite(
    "co2_c",
    name="CO2",
    formula="CO2",
    charge=0,
    compartment="c",
    fixed=False)
```

###### Reactions¶

Once all of the `MassMetabolite` objects for each metabolite, the next step is to define all of the reactions that occur and their stoichiometry.

1. As with the metabolites, it is also important to use a clear and consistent format for identifiers and names when defining when defining the `MassReaction` objects.
2. To make this model useful for integration with other models, it is important to provide a string to the `subsystem` argument. By providing the subsystem, the reactions can be easily obtained even when integrated with a significantly larger model through the `subsystem` attribute.
3. After the creation of each `MassReaction` object, the metabolites are added to the reaction using a dictionary where keys are the `MassMetabolite` objects and values are the stoichiometric coefficients (reactants have negative coefficients, products have positive ones).

This model will be created using identifiers and names found in the BiGG Database.

In this model, there are 10 reactions occuring inside the cytosol compartment.

```
[4]:
```

```
G6PDH2r = MassReaction(
    "G6PDH2r",
    name="Glucose 6-phosphate dehydrogenase",
    subsystem=ppp.id,
    reversible=True)
G6PDH2r.add_metabolites({
    g6p_c: -1,
    nadp_c: -1,
    _6pgl_c: 1,
    nadph_c: 1,
    h_c: 1})

PGL = MassReaction(
    "PGL",
    name="6-phosphogluconolactonase",
    subsystem=ppp.id,
    reversible=True)
PGL.add_metabolites({
    _6pgl_c: -1,
    h2o_c: -1,
    _6pgc_c: 1,
    h_c: 1})

GND = MassReaction(
    "GND",
    name="Phosphogluconate dehydrogenase",
    subsystem=ppp.id,
    reversible=True)
GND.add_metabolites({
    _6pgc_c: -1,
    nadp_c: -1,
    nadph_c: 1,
    co2_c: 1,
    ru5p__D_c: 1})

RPI = MassReaction(
    "RPI",
    name="Ribulose 5-Phosphate Isomerase",
    subsystem=ppp.id,
    reversible=True)
RPI.add_metabolites({
    ru5p__D_c: -1,
    r5p_c: 1})

RPE = MassReaction(
    "RPE",
    name="Ribulose 5-phosphate 3-epimerase",
    subsystem=ppp.id,
    reversible=True)
RPE.add_metabolites({
    ru5p__D_c: -1,
    xu5p__D_c: 1})

TKT1 = MassReaction(
    "TKT1",
    name="Transketolase",
    subsystem=ppp.id,
    reversible=True)
TKT1.add_metabolites({
    r5p_c: -1,
    xu5p__D_c: -1,
    g3p_c: 1,
    s7p_c: 1})

TALA = MassReaction(
    "TALA",
    name="Transaldolase",
    subsystem=ppp.id,
    reversible=True)
TALA.add_metabolites({
    g3p_c: -1,
    s7p_c: -1,
    e4p_c: 1,
    f6p_c: 1})

TKT2 = MassReaction(
    "TKT2",
    name="Transketolase",
    subsystem=ppp.id,
    reversible=True)
TKT2.add_metabolites({
    e4p_c: -1,
    xu5p__D_c: -1,
    f6p_c: 1,
    g3p_c: 1})

GTHOr = MassReaction(
    "GTHOr",
    name="Glutathione oxidoreductase",
    subsystem="Misc.",
    reversible=True)
GTHOr.add_metabolites({
    gthox_c: -1,
    h_c: -1,
    nadph_c: -1,
    gthrd_c: 2,
    nadp_c: 1})

GSHR = MassReaction(
    "GSHR",
    name="Glutathione-disulfide reductase",
    subsystem="Misc.",
    reversible=True)
GSHR.add_metabolites({
    gthrd_c: -2,
    gthox_c: 1,
    h_c: 2})
```

After generating the reactions, all reactions are added to the model through the `MassModel.add_reactions` class method. Adding the `MassReaction` objects will also add their associated `MassMetabolite` objects if they have not already been added to the model.

```
[5]:
```

```
ppp.add_reactions([
    G6PDH2r, PGL, GND, RPI, RPE, TKT1, TALA, TKT2, GTHOr, GSHR])

for reaction in ppp.reactions:
    print(reaction)
```

```
G6PDH2r: g6p_c + nadp_c <=> _6pgl_c + h_c + nadph_c
PGL: _6pgl_c + h2o_c <=> _6pgc_c + h_c
GND: _6pgc_c + nadp_c <=> co2_c + nadph_c + ru5p__D_c
RPI: ru5p__D_c <=> r5p_c
RPE: ru5p__D_c <=> xu5p__D_c
TKT1: r5p_c + xu5p__D_c <=> g3p_c + s7p_c
TALA: g3p_c + s7p_c <=> e4p_c + f6p_c
TKT2: e4p_c + xu5p__D_c <=> f6p_c + g3p_c
GTHOr: gthox_c + h_c + nadph_c <=> 2 gthrd_c + nadp_c
GSHR: 2 gthrd_c <=> gthox_c + 2 h_c
```

###### Boundary reactions¶

After generating the reactions, the next step is to add the boundary reactions and boundary conditions (the concentrations of the boundary ‘metabolites’ of the system). This can easily be done using the `MassModel.add_boundary` method. With the generation of the boundary reactions, the system becomes an open system, allowing for the flow of mass through the biochemical pathways of the model. Once added, the model will be able to return the boundary conditions as a dictionary through the
`MassModel.boundary_conditions` attribute.

All boundary reactions are originally created with the metabolite as the reactant. However, there are times where it would be preferable to represent the metabolite as the product. For these situtations, the `MassReaction.reverse_stoichiometry` method can be used with its `inplace` argument to create a new `MassReaction` or simply reverse the stoichiometry for the current `MassReaction.`

In this model, there are 7 boundary reactions that must be defined.

```
[6]:
```

```
DM_f6p_c = ppp.add_boundary(
    metabolite=f6p_c, boundary_type="demand", subsystem="Pseudoreaction",
    boundary_condition=1)

DM_r5p_c = ppp.add_boundary(
    metabolite=r5p_c, boundary_type="demand", subsystem="Pseudoreaction",
    boundary_condition=1)

DM_g3p_c = ppp.add_boundary(
    metabolite=g3p_c, boundary_type="demand", subsystem="Pseudoreaction",
    boundary_condition=1)
SK_g6p_c = ppp.add_boundary(
    metabolite=g6p_c, boundary_type="sink", subsystem="Pseudoreaction",
    boundary_condition=1)
SK_g6p_c.reverse_stoichiometry(inplace=True)

SK_h_c = ppp.add_boundary(
    metabolite=h_c, boundary_type="sink", subsystem="Pseudoreaction",
    boundary_condition=6.30957e-05)

SK_h2o_c = ppp.add_boundary(
    metabolite=h2o_c, boundary_type="sink", subsystem="Pseudoreaction",
    boundary_condition=1)

SK_co2_c = ppp.add_boundary(
    metabolite=co2_c, boundary_type="sink", subsystem="Pseudoreaction",
    boundary_condition=1)

print("Boundary Reactions and Values\n-----------------------------")
for reaction in ppp.boundary:
    boundary_met = reaction.boundary_metabolite
    bc_value = ppp.boundary_conditions.get(boundary_met)
    print("{0}\n{1}: {2}\n".format(
        reaction, boundary_met, bc_value))
```

```
Boundary Reactions and Values
-----------------------------
DM_f6p_c: f6p_c -->
f6p_b: 1.0

DM_r5p_c: r5p_c -->
r5p_b: 1.0

DM_g3p_c: g3p_c -->
g3p_b: 1.0

SK_g6p_c:  <=> g6p_c
g6p_b: 1.0

SK_h_c: h_c <=>
h_b: 6.30957e-05

SK_h2o_c: h2o_c <=>
h2o_b: 1.0

SK_co2_c: co2_c <=>
co2_b: 1.0
```

###### Ordering of internal species and reactions¶

Sometimes, it is also desirable to reorder the metabolite and reaction objects inside the model to follow the physiology. To reorder the internal objects, one can use `cobra.DictList` containers and the `DictList.get_by_any` method with the list of object identifiers in the desirable order. To ensure all objects are still present and not forgotten in the model, a small QA check is also performed.

```
[7]:
```

```
new_metabolite_order = [
    "f6p_c", "g6p_c", "g3p_c", "_6pgl_c", "_6pgc_c",
    "ru5p__D_c", "xu5p__D_c", "r5p_c", "s7p_c", "e4p_c",
    "nadp_c", "nadph_c", "gthrd_c", "gthox_c", "co2_c",
    "h_c", "h2o_c"]

if len(ppp.metabolites) == len(new_metabolite_order):
    ppp.metabolites = DictList(
        ppp.metabolites.get_by_any(new_metabolite_order))

new_reaction_order = [
    "G6PDH2r", "PGL", "GND", "RPE", "RPI",
    "TKT1", "TKT2", "TALA", "GTHOr", "GSHR",
    "SK_g6p_c", "DM_f6p_c", "DM_g3p_c","DM_r5p_c",
    "SK_co2_c", "SK_h_c", "SK_h2o_c"]

if len(ppp.reactions) == len(new_reaction_order):
    ppp.reactions = DictList(
        ppp.reactions.get_by_any(new_reaction_order))

ppp.update_S(array_type="DataFrame", dtype=int)
```

```
[7]:
```

|  | G6PDH2r | PGL | GND | RPE | RPI | TKT1 | TKT2 | TALA | GTHOr | GSHR | SK\_g6p\_c | DM\_f6p\_c | DM\_g3p\_c | DM\_r5p\_c | SK\_co2\_c | SK\_h\_c | SK\_h2o\_c |
| --- | --- | --- | --- | --- | --- | --- | --- | --- | --- | --- | --- | --- | --- | --- | --- | --- | --- |
| f6p\_c | 0 | 0 | 0 | 0 | 0 | 0 | 1 | 1 | 0 | 0 | 0 | -1 | 0 | 0 | 0 | 0 | 0 |
| g6p\_c | -1 | 0 | 0 | 0 | 0 | 0 | 0 | 0 | 0 | 0 | 1 | 0 | 0 | 0 | 0 | 0 | 0 |
| g3p\_c | 0 | 0 | 0 | 0 | 0 | 1 | 1 | -1 | 0 | 0 | 0 | 0 | -1 | 0 | 0 | 0 | 0 |
| \_6pgl\_c | 1 | -1 | 0 | 0 | 0 | 0 | 0 | 0 | 0 | 0 | 0 | 0 | 0 | 0 | 0 | 0 | 0 |
| \_6pgc\_c | 0 | 1 | -1 | 0 | 0 | 0 | 0 | 0 | 0 | 0 | 0 | 0 | 0 | 0 | 0 | 0 | 0 |
| ru5p\_\_D\_c | 0 | 0 | 1 | -1 | -1 | 0 | 0 | 0 | 0 | 0 | 0 | 0 | 0 | 0 | 0 | 0 | 0 |
| xu5p\_\_D\_c | 0 | 0 | 0 | 1 | 0 | -1 | -1 | 0 | 0 | 0 | 0 | 0 | 0 | 0 | 0 | 0 | 0 |
| r5p\_c | 0 | 0 | 0 | 0 | 1 | -1 | 0 | 0 | 0 | 0 | 0 | 0 | 0 | -1 | 0 | 0 | 0 |
| s7p\_c | 0 | 0 | 0 | 0 | 0 | 1 | 0 | -1 | 0 | 0 | 0 | 0 | 0 | 0 | 0 | 0 | 0 |
| e4p\_c | 0 | 0 | 0 | 0 | 0 | 0 | -1 | 1 | 0 | 0 | 0 | 0 | 0 | 0 | 0 | 0 | 0 |
| nadp\_c | -1 | 0 | -1 | 0 | 0 | 0 | 0 | 0 | 1 | 0 | 0 | 0 | 0 | 0 | 0 | 0 | 0 |
| nadph\_c | 1 | 0 | 1 | 0 | 0 | 0 | 0 | 0 | -1 | 0 | 0 | 0 | 0 | 0 | 0 | 0 | 0 |
| gthrd\_c | 0 | 0 | 0 | 0 | 0 | 0 | 0 | 0 | 2 | -2 | 0 | 0 | 0 | 0 | 0 | 0 | 0 |
| gthox\_c | 0 | 0 | 0 | 0 | 0 | 0 | 0 | 0 | -1 | 1 | 0 | 0 | 0 | 0 | 0 | 0 | 0 |
| co2\_c | 0 | 0 | 1 | 0 | 0 | 0 | 0 | 0 | 0 | 0 | 0 | 0 | 0 | 0 | -1 | 0 | 0 |
| h\_c | 1 | 1 | 0 | 0 | 0 | 0 | 0 | 0 | -1 | 2 | 0 | 0 | 0 | 0 | 0 | -1 | 0 |
| h2o\_c | 0 | -1 | 0 | 0 | 0 | 0 | 0 | 0 | 0 | 0 | 0 | 0 | 0 | 0 | 0 | 0 | -1 |

##### Model Parameterization¶

###### Steady State fluxes¶

Steady state fluxes can be computed as a summation of the MinSpan pathway vectors. Pathways are obtained using MinSpan.

Using these pathways and literature sources, independent fluxes can be defined in order to calculate the steady state flux vector. For this model, the flux of glucose-6-phosphate uptake is fixed at 0.21 mM/hr, and the flux of Alpha-D-Ribose 5-phosphate uptake is fixed at 0.01 mM/hr. The former value represents a typical flux through the pentose pathway while the latter will couple at a low flux level to the AMP pathways

With these pathays and numerical values, the steady state flux vector can be computed as the weighted sum of the corresponding basis vectors. The steady state flux vector is computed as an inner product:

```
[8]:
```

```
minspan_paths = [
    [1, 1, 1, 2/3, 1/3, 1/3, 1/3, 1/3, 2, 2, 1, 2/3, 1/3, 0, 1, 4, -1],
    [1, 1, 1,  0,   1,   0,   0,   0,  2, 2, 1,  0,   0,  1, 1, 4, -1]]
ppp.compute_steady_state_fluxes(
    pathways=minspan_paths,
    independent_fluxes={
        SK_g6p_c: 0.21,
        DM_r5p_c: 0.01},
    update_reactions=True)

print("Steady State Fluxes\n-------------------")
for reaction, steady_state_flux in ppp.steady_state_fluxes.items():
    print("{0}: {1:.6f}".format(reaction.flux_symbol_str, steady_state_flux))
```

```
Steady State Fluxes
-------------------
v_G6PDH2r: 0.210000
v_PGL: 0.210000
v_GND: 0.210000
v_RPE: 0.133333
v_RPI: 0.076667
v_TKT1: 0.066667
v_TKT2: 0.066667
v_TALA: 0.066667
v_GTHOr: 0.420000
v_GSHR: 0.420000
v_SK_g6p_c: 0.210000
v_DM_f6p_c: 0.133333
v_DM_g3p_c: 0.066667
v_DM_r5p_c: 0.010000
v_SK_co2_c: 0.210000
v_SK_h_c: 0.840000
v_SK_h2o_c: -0.210000
```

###### Initial Conditions¶

Once the network has been built, the concentrations can be added to the metabolites. These concentrations are also treated as the initial conditions required to integrate and simulate the model’s ordinary differential equations (ODEs). The metabolite concentrations are added to each individual metabolite using the `MassMetabolite.initial_condition` (alias: `MassMetabolite.ic`) attribute setter methods. Once added, the model will be able to return the initial conditions as a dictionary
through the `MassModel.initial_conditions` attribute.

```
[9]:
```

```
g6p_c.ic = 0.0486
f6p_c.ic = 0.0198
g3p_c.ic = 0.00728
_6pgl_c.ic = 0.00175424
_6pgc_c.ic = 0.0374753
ru5p__D_c.ic = 0.00493679
xu5p__D_c.ic = 0.0147842
r5p_c.ic = 0.0126689
s7p_c.ic = 0.023988
e4p_c.ic = 0.00507507
nadp_c.ic = 0.0002
nadph_c.ic = 0.0658
gthrd_c.ic = 3.2
gthox_c.ic = 0.12
co2_c.ic = 1
h_c.ic = 0.0000714957
h2o_c.ic = 1

print("Initial Conditions\n------------------")
for metabolite, ic_value in ppp.initial_conditions.items():
    print("{0}: {1}".format(metabolite, ic_value))
```

```
Initial Conditions
------------------
f6p_c: 0.0198
g6p_c: 0.0486
g3p_c: 0.00728
_6pgl_c: 0.00175424
_6pgc_c: 0.0374753
ru5p__D_c: 0.00493679
xu5p__D_c: 0.0147842
r5p_c: 0.0126689
s7p_c: 0.023988
e4p_c: 0.00507507
nadp_c: 0.0002
nadph_c: 0.0658
gthrd_c: 3.2
gthox_c: 0.12
co2_c: 1
h_c: 7.14957e-05
h2o_c: 1
```

###### Equilibirum Constants¶

After adding initial conditions and steady state fluxes, the equilibrium constants are defined using the `MassReaction.equilibrium_constant` (alias: `MassReaction.Keq`) setter method.

```
[10]:
```

```
G6PDH2r.Keq = 1000
PGL.Keq = 1000
GND.Keq = 1000
RPE.Keq = 3
RPI.Keq = 2.57
TKT1.Keq = 1.2
TKT2.Keq = 10.3
TALA.Keq = 1.05
GTHOr.Keq = 100
GSHR.Keq = 2

SK_g6p_c.Keq = 1
SK_h_c.Keq = 1
SK_h2o_c.Keq = 1
SK_co2_c.Keq = 1

print("Equilibrium Constants\n---------------------")
for reaction in ppp.reactions:
    print("{0}: {1}".format(reaction.Keq_str, reaction.Keq))
```

```
Equilibrium Constants
---------------------
Keq_G6PDH2r: 1000
Keq_PGL: 1000
Keq_GND: 1000
Keq_RPE: 3
Keq_RPI: 2.57
Keq_TKT1: 1.2
Keq_TKT2: 10.3
Keq_TALA: 1.05
Keq_GTHOr: 100
Keq_GSHR: 2
Keq_SK_g6p_c: 1
Keq_DM_f6p_c: inf
Keq_DM_g3p_c: inf
Keq_DM_r5p_c: inf
Keq_SK_co2_c: 1
Keq_SK_h_c: 1
Keq_SK_h2o_c: 1
```

###### Calculation of PERCs¶

By defining the equilibrium constant and steady state parameters, the values of the pseudo rate constants (PERCs) can be calculated and added to the model using the `MassModel.calculate_PERCs` method.

```
[11]:
```

```
ppp.calculate_PERCs(update_reactions=True)

print("Forward Rate Constants\n----------------------")
for reaction in ppp.reactions:
    print("{0}: {1:.6f}".format(reaction.kf_str, reaction.kf))
```

```
Forward Rate Constants
----------------------
kf_G6PDH2r: 21864.589249
kf_PGL: 122.323112
kf_GND: 29287.807474
kf_RPE: 15284.677111
kf_RPI: 10564.620051
kf_TKT1: 1595.951975
kf_TKT2: 1092.246435
kf_TALA: 844.616138
kf_GTHOr: 53.329812
kf_GSHR: 0.041257
kf_SK_g6p_c: 0.220727
kf_DM_f6p_c: 6.734007
kf_DM_g3p_c: 9.157509
kf_DM_r5p_c: 0.789335
kf_SK_co2_c: 100000.000000
kf_SK_h_c: 100000.000000
kf_SK_h2o_c: 100000.000000
```

##### QC/QA Model¶

Before simulating the model, it is important to ensure that the model is elementally balanced, and that the model can simulate. Therefore, the `qcqa_model` function from the `mass.util.qcqa` submodule is used to provide a report on the model quality and indicate whether simulation is possible and if not, what parameters and/or initial conditions are missing.

Generally, pseudoreactions (e.g. boundary exchanges, sinks, demands) are not elementally balanced. The `qcqa_model` function does not include elemental balancing of boundary reactions. However, some models contain pseudoreactions reprsenting a simplified mechanism, and show up in the returned report. The elemental imbalance of these pseudoreactions is therefore expected in certain reaction and should not be a cause for concern.

In this model of the PPP, the GSHR reaction is a simplified pseudoreaction that represents oxidative stress due to gluthathione and is not expected to be balanced.

```
[12]:
```

```
qcqa_model(ppp, parameters=True, concentrations=True,
           fluxes=True, superfluous=True, elemental=True)
```

```
╒══════════════════════════════════════════════╕
│ MODEL ID: PentosePhosphatePathway            │
│ SIMULATABLE: True                            │
│ PARAMETERS NUMERICALY CONSISTENT: True       │
╞══════════════════════════════════════════════╡
│ ============================================ │
│             CONSISTENCY CHECKS               │
│ ============================================ │
│ Elemental                                    │
│ -------------------                          │
│ GSHR: {charge: 2.0}                          │
│ ============================================ │
╘══════════════════════════════════════════════╛
```

From the results of the QC/QA test, it can be seen that the model can be simulated and is numerically consistent.

##### Steady State and Model Validation¶

To find the steady state of the model and perform simulations, the model must first be loaded into a `Simulation`. In order to load a model into a `Simulation`, the model must be simulatable, meaning there are no missing numerical values that would prevent the integration of the ODEs that comprise the model. The `verbose` argument can be used while loading a model to produce a message indicating the successful loading of a model, or why a model could not load.

Once loaded into a `Simulation`, the `find_steady_state` method can be used with the `update_values` argument in order to update the initial conditions and fluxes of the model to a steady state (if necessary). The model can be simulated using the `simulate` method by passing the model to simulate, and a tuple containing the start time and the end time. The number of time points can also be included, but is optional.

After a successful simulation, two `MassSolution` objects are returned. The first `MassSolution` contains the concentration results of the simulation, and the second contains the flux results of the simulation.

To visually validate the steady state of the model, concentration and flux solutions can be plotted using the `plot_time_profile` function from `mass.visualization`. Alternatively, the `MassSolution.view_time_profile` property can be used to quickly generate a time profile for the results.

```
[13]:
```

```
# Setup simulation object
sim = Simulation(ppp, verbose=True)
# Simulate from 0 to 1000 with 10001 points in the output
conc_sol, flux_sol = sim.simulate(ppp, time=(0, 1e3, 1e4 + 1))
# Quickly render and display time profiles
conc_sol.view_time_profile()
```

```
Successfully loaded MassModel 'PentosePhosphatePathway' into RoadRunner.
```

###### Storing information and references¶

###### Compartment¶

Because the character “c” represents the cytosol compartment, it is recommended to define and set the compartment in the `MassModel.compartments` attribute.

```
[14]:
```

```
ppp.compartments = {"c": "Cytosol"}
print(ppp.compartments)
```

```
{'c': 'Cytosol'}
```

###### Units¶

All of the units for the numerical values used in this model are “Millimoles” for amount and “Liters” for volume (giving a concentration unit of ‘Millimolar’), and “Hours” for time. In order to ensure that future users understand the numerical values for model, it is important to define the `MassModel.units` attribute.

The `MassModel.units` is a `cobra.DictList` that contains only `UnitDefinition` objects from the `mass.core.unit` submodule. Each `UnitDefinition` is created from `Unit` objects representing the base units that comprise the `UnitDefinition`. These `Units` are stored in the `list_of_units` attribute. Pre-built units can be viewed using the `print_defined_unit_values` function from the `mass.core.unit` submodule. Alternatively, custom units can also be created using the
`UnitDefinition.create_unit` method. For more information about units, please see the module docstring for `mass.core.unit` submodule.

**Note:** It is important to note that this attribute will NOT track units, but instead acts as a reference for the user and others so that they can perform necessary unit conversions.

```
[15]:
```

```
# Using pre-build units to define UnitDefinitions
concentration = UnitDefinition("mM", name="Millimolar",
                               list_of_units=["millimole", "per_litre"])
time = UnitDefinition("hr", name="hour", list_of_units=["hour"])

# Add units to model
ppp.add_units([concentration, time])
print(ppp.units)
```

```
[<UnitDefinition Millimolar "mM" at 0x7fea3a1a3450>, <UnitDefinition hour "hr" at 0x7fea3a1a34d0>]
```

##### Export¶

After validation, the model is ready to be saved. The model can either be exported as a “.json” file or as an “.sbml” (“.xml”) file using their repsective submodules in `mass.io`.

To export the model, only the path to the directory and the model object itself need to be specified.

###### Export using SBML¶

```
[16]:
```

```
sbml.write_sbml_model(mass_model=ppp, filename="SB2_" + ppp.id + ".xml")
```

###### Export using JSON¶

```
[17]:
```

```
json.save_json_model(mass_model=ppp, filename="SB2_" + ppp.id + ".json")
```

#### AMP Salvage Network Model Construction¶

Based on Chapter 12 of [Pal11]

To construct a model of the AMP Salvage Network (AMPSN), first we import **MASSpy** and other essential packages. Constants used throughout the notebook are also defined.

```
[1]:
```

```
from os import path

import matplotlib.pyplot as plt

from cobra import DictList

from mass import (
    MassConfiguration, MassMetabolite, MassModel,
    MassReaction, Simulation, UnitDefinition)
from mass.io import json, sbml
from mass.util.qcqa import qcqa_model

mass_config = MassConfiguration()

mass_config.irreversible_Keq = float("inf")
```

##### Model Construction¶

The first step of creating a model of the AMPSN is to define the `MassModel`.

```
[2]:
```

```
ampsn = MassModel("AMPSalvageNetwork")
```

###### Metabolites¶

The next step is to define all of the metabolites using the `MassMetabolite` object. Some considerations for this step include the following:

1. It is important to use a clear and consistent format for identifiers and names when defining the `MassMetabolite` objects for various reasons, some of which include improvements to model clarity and utility, assurance of unique identifiers (required to add metabolites to the model), and consistency when collaborating and communicating with others.
2. In order to ensure our model is physiologically accurate, it is important to provide the `formula` argument with a string representing the chemical formula for each metabolite, and the `charge` argument with an integer representing the metabolite’s ionic charge (Note that neutrally charged metabolites are provided with 0). These attributes can always be set later if necessary using the `formula` and `charge` attribute set methods.
3. To indicate that the cytosol is the cellular compartment in which the reactions occur, the string “c” is provided to the `compartment` argument.

This model will be created using identifiers and names found in the BiGG Database.

In this model, there are 15 metabolites inside the cytosol compartment.

```
[3]:
```

```
adn_c = MassMetabolite(
    "adn_c",
    name="Adenosine",
    formula="C10H13N5O4",
    charge=0,
    compartment="c",
    fixed=False)

ade_c = MassMetabolite(
    "ade_c",
    name="Adenine",
    formula="C5H5N5",
    charge=0,
    compartment="c",
    fixed=False)

imp_c = MassMetabolite(
    "imp_c",
    name="Inosine monophosphate",
    formula="C10H11N4O8P",
    charge=-2,
    compartment="c",
    fixed=False)

ins_c = MassMetabolite(
    "ins_c",
    name="Inosine",
    formula="C10H12N4O5",
    charge=0,
    compartment="c",
    fixed=False)

hxan_c = MassMetabolite(
    "hxan_c",
    name="Hypoxanthine",
    formula="C5H4N4O",
    charge=0,
    compartment="c",
    fixed=False)

r1p_c = MassMetabolite(
    "r1p_c",
    name="Alpha-D-Ribose 1-phosphate",
    formula="C5H9O8P",
    charge=-2,
    compartment="c",
    fixed=False)

r5p_c = MassMetabolite(
    "r5p_c",
    name="Alpha-D-Ribose 5-phosphate",
    formula="C5H9O8P",
    charge=-2,
    compartment="c",
    fixed=False)

prpp_c = MassMetabolite(
    "prpp_c",
    name="5-Phospho-alpha-D-ribose 1-diphosphate",
    formula="C5H8O14P3",
    charge=-5,
    compartment="c",
    fixed=False)

atp_c = MassMetabolite(
    "atp_c",
    name="ATP",
    formula="C10H12N5O13P3",
    charge=-4,
    compartment="c",
    fixed=False)

adp_c = MassMetabolite(
    "adp_c",
    name="ADP",
    formula="C10H12N5O10P2",
    charge=-3,
    compartment="c",
    fixed=False)

amp_c = MassMetabolite(
    "amp_c",
    name="AMP",
    formula="C10H12N5O7P",
    charge=-2,
    compartment="c",
    fixed=False)

h_c = MassMetabolite(
    "h_c",
    name="H+",
    formula="H",
    charge=1,
    compartment="c",
    fixed=False)

h2o_c = MassMetabolite(
    "h2o_c",
    name="H2O",
    formula="H2O",
    charge=0,
    compartment="c",
    fixed=False)

pi_c = MassMetabolite(
    "pi_c",
    name="Phosphate",
    formula="HPO4",
    charge=-2,
    compartment="c",
    fixed=False)

nh3_c = MassMetabolite(
    "nh3_c",
    name="Ammonia",
    formula="H3N",
    charge=0,
    compartment="c",
    fixed=False)
```

###### Reactions¶

Once all of the `MassMetabolite` objects for each metabolite, the next step is to define all of the reactions that occur and their stoichiometry.

1. As with the metabolites, it is also important to use a clear and consistent format for identifiers and names when defining when defining the `MassReaction` objects.
2. To make this model useful for integration with other models, it is important to provide a string to the `subsystem` argument. By providing the subsystem, the reactions can be easily obtained even when integrated with a significantly larger model through the `subsystem` attribute.
3. After the creation of each `MassReaction` object, the metabolites are added to the reaction using a dictionary where keys are the `MassMetabolite` objects and values are the stoichiometric coefficients (reactants have negative coefficients, products have positive ones).

This model will be created using identifiers and names found in the BiGG Database.

In this model, there are 10 reactions occuring inside the cytosol compartment.

```
[4]:
```

```
ADNK1 = MassReaction(
    "ADNK1",
    name="Adenosine kinase",
    subsystem=ampsn.id,
    reversible=False)
ADNK1.add_metabolites({
    adn_c: -1,
    atp_c: -1,
    adp_c: 1,
    amp_c: 1,
    h_c: 1})

NTD7 = MassReaction(
    "NTD7",
    name="5'-nucleotidase (AMP)",
    subsystem=ampsn.id,
    reversible=False)
NTD7.add_metabolites({
    amp_c: -1,
    h2o_c: -1,
    adn_c: 1,
    pi_c: 1})

AMPDA = MassReaction(
    "AMPDA",
    name="Adenosine monophosphate deaminase",
    subsystem=ampsn.id,
    reversible=False)
AMPDA.add_metabolites({
    amp_c: -1,
    h2o_c: -1,
    imp_c: 1,
    nh3_c: 1})

NTD11 = MassReaction(
    "NTD11",
    name="5'-nucleotidase (IMP)",
    subsystem=ampsn.id,
    reversible=False)
NTD11.add_metabolites({
    imp_c: -1,
    h2o_c: -1,
    ins_c: 1,
    pi_c: 1})

ADA = MassReaction(
    "ADA",
    name="Adenosine deaminase",
    subsystem=ampsn.id,
    reversible=False)
ADA.add_metabolites({
    adn_c: -1,
    h2o_c: -1,
    ins_c: 1,
    nh3_c: 1})

PUNP5 = MassReaction(
    "PUNP5",
    name="Purine-nucleoside phosphorylase (Inosine)",
    subsystem=ampsn.id,
    reversible=True)
PUNP5.add_metabolites({
    ins_c: -1,
    pi_c: -1,
    hxan_c: 1,
    r1p_c: 1})

PPM = MassReaction(
    "PPM",
    name="Phosphopentomutase",
    subsystem=ampsn.id,
    reversible=True)
PPM.add_metabolites({
    r1p_c: -1,
    r5p_c: 1})
# Must account for lack of ADK1 reaction in this model.
# Therefore, coefficients of atp_c and adp_c are -2 and 2
PRPPS = MassReaction(
    "PRPPS",
    name="Phosphoribosylpyrophosphate synthetase",
    subsystem=ampsn.id,
    reversible=False)
PRPPS.add_metabolites({
    atp_c: -2,
    r5p_c: -1,
    adp_c: 2,
    h_c: 1,
    prpp_c: 1})

ADPT = MassReaction(
    "ADPT",
    subsystem=ampsn.id,
    reversible=False)
ADPT.add_metabolites({
    ade_c: -1,
    h2o_c: -1,
    prpp_c: -1,
    amp_c: 1,
    h_c: 1,
    pi_c: 2
})

ATPM = MassReaction(
    "ATPM",
    name="ATP maintenance requirement",
    subsystem="Misc.",
    reversible=False)
ATPM.add_metabolites({
    adp_c: -1,
    h_c: -1,
    pi_c: -1,
    atp_c: 1,
    h2o_c: 1})
```

After generating the reactions, all reactions are added to the model through the `MassModel.add_reactions` class method. Adding the `MassReaction` objects will also add their associated `MassMetabolite` objects if they have not already been added to the model.

```
[5]:
```

```
ampsn.add_reactions([
    ADNK1, NTD7, AMPDA, NTD11, ADA, PUNP5, PPM, PRPPS, ADPT, ATPM])

for reaction in ampsn.reactions:
    print(reaction)
```

```
ADNK1: adn_c + atp_c --> adp_c + amp_c + h_c
NTD7: amp_c + h2o_c --> adn_c + pi_c
AMPDA: amp_c + h2o_c --> imp_c + nh3_c
NTD11: h2o_c + imp_c --> ins_c + pi_c
ADA: adn_c + h2o_c --> ins_c + nh3_c
PUNP5: ins_c + pi_c <=> hxan_c + r1p_c
PPM: r1p_c <=> r5p_c
PRPPS: 2 atp_c + r5p_c --> 2 adp_c + h_c + prpp_c
ADPT: ade_c + h2o_c + prpp_c --> amp_c + h_c + 2 pi_c
ATPM: adp_c + h_c + pi_c --> atp_c + h2o_c
```

###### Boundary reactions¶

After generating the reactions, the next step is to add the boundary reactions and boundary conditions (the concentrations of the boundary ‘metabolites’ of the system). This can easily be done using the `MassModel.add_boundary` method. With the generation of the boundary reactions, the system becomes an open system, allowing for the flow of mass through the biochemical pathways of the model. Once added, the model will be able to return the boundary conditions as a dictionary through the
`MassModel.boundary_conditions` attribute.

All boundary reactions are originally created with the metabolite as the reactant. However, there are times where it would be preferable to represent the metabolite as the product. For these situtations, the `MassReaction.reverse_stoichiometry` method can be used with its `inplace` argument to create a new `MassReaction` or simply reverse the stoichiometry for the current `MassReaction.`

In this model, there are 8 boundary reactions that must be defined.

```
[6]:
```

```
SK_adn_c = ampsn.add_boundary(
    metabolite=adn_c, boundary_type="sink", subsystem="Pseudoreaction",
    boundary_condition=1.2e-3)

SK_ade_c = ampsn.add_boundary(
    metabolite=ade_c, boundary_type="sink", subsystem="Pseudoreaction",
    boundary_condition=1.0e-3)

SK_ins_c = ampsn.add_boundary(
    metabolite=ins_c, boundary_type="sink", subsystem="Pseudoreaction",
    boundary_condition=1.0e-3)

SK_hxan_c = ampsn.add_boundary(
    metabolite=hxan_c, boundary_type="sink", subsystem="Pseudoreaction",
    boundary_condition=2.0e-3)

SK_amp_c = ampsn.add_boundary(
    metabolite=amp_c, boundary_type="sink", subsystem="Pseudoreaction",
    boundary_condition=8.67281e-2)

SK_h_c = ampsn.add_boundary(
    metabolite=h_c, boundary_type="sink", subsystem="Pseudoreaction",
    boundary_condition=6.30957e-5)

SK_h2o_c = ampsn.add_boundary(
    metabolite=h2o_c, boundary_type="sink", subsystem="Pseudoreaction",
    boundary_condition=1)

SK_pi_c = ampsn.add_boundary(
    metabolite=pi_c, boundary_type="sink", subsystem="Pseudoreaction",
    boundary_condition=2.5)

SK_nh3_c = ampsn.add_boundary(
    metabolite=nh3_c, boundary_type="sink", subsystem="Pseudoreaction",
    boundary_condition=9.1002e-2)

print("Boundary Reactions and Values\n-----------------------------")
for reaction in ampsn.boundary:
    boundary_met = reaction.boundary_metabolite
    bc_value = ampsn.boundary_conditions.get(boundary_met)
    print("{0}\n{1}: {2}\n".format(
        reaction, boundary_met, bc_value))
```

```
Boundary Reactions and Values
-----------------------------
SK_adn_c: adn_c <=>
adn_b: 0.0012

SK_ade_c: ade_c <=>
ade_b: 0.001

SK_ins_c: ins_c <=>
ins_b: 0.001

SK_hxan_c: hxan_c <=>
hxan_b: 0.002

SK_amp_c: amp_c <=>
amp_b: 0.0867281

SK_h_c: h_c <=>
h_b: 6.30957e-05

SK_h2o_c: h2o_c <=>
h2o_b: 1.0

SK_pi_c: pi_c <=>
pi_b: 2.5

SK_nh3_c: nh3_c <=>
nh3_b: 0.091002
```

###### Ordering of internal species and reactions¶

Sometimes, it is also desirable to reorder the metabolite and reaction objects inside the model to follow the physiology. To reorder the internal objects, one can use `cobra.DictList` containers and the `DictList.get_by_any` method with the list of object identifiers in the desirable order. To ensure all objects are still present and not forgotten in the model, a small QA check is also performed.

```
[7]:
```

```
new_metabolite_order = [
    "adn_c", "ade_c", "imp_c", "ins_c", "hxan_c",
    "r1p_c", "r5p_c", "prpp_c", "atp_c", "adp_c",
    "amp_c", "pi_c", "nh3_c", "h_c", "h2o_c"]

if len(ampsn.metabolites) == len(new_metabolite_order):
    ampsn.metabolites = DictList(
        ampsn.metabolites.get_by_any(new_metabolite_order))

new_reaction_order = [
    "ADNK1", "NTD7", "AMPDA", "NTD11",
    "ADA", "PUNP5", "PPM", "PRPPS",
    "ADPT", "ATPM", "SK_adn_c", "SK_ade_c",
    "SK_ins_c", "SK_hxan_c", "SK_nh3_c", "SK_pi_c",
    "SK_amp_c", "SK_h_c", "SK_h2o_c"]

if len(ampsn.reactions) == len(new_reaction_order):
    ampsn.reactions = DictList(
        ampsn.reactions.get_by_any(new_reaction_order))

ampsn.update_S(array_type="DataFrame", dtype=int)
```

```
[7]:
```

|  | ADNK1 | NTD7 | AMPDA | NTD11 | ADA | PUNP5 | PPM | PRPPS | ADPT | ATPM | SK\_adn\_c | SK\_ade\_c | SK\_ins\_c | SK\_hxan\_c | SK\_nh3\_c | SK\_pi\_c | SK\_amp\_c | SK\_h\_c | SK\_h2o\_c |
| --- | --- | --- | --- | --- | --- | --- | --- | --- | --- | --- | --- | --- | --- | --- | --- | --- | --- | --- | --- |
| adn\_c | -1 | 1 | 0 | 0 | -1 | 0 | 0 | 0 | 0 | 0 | -1 | 0 | 0 | 0 | 0 | 0 | 0 | 0 | 0 |
| ade\_c | 0 | 0 | 0 | 0 | 0 | 0 | 0 | 0 | -1 | 0 | 0 | -1 | 0 | 0 | 0 | 0 | 0 | 0 | 0 |
| imp\_c | 0 | 0 | 1 | -1 | 0 | 0 | 0 | 0 | 0 | 0 | 0 | 0 | 0 | 0 | 0 | 0 | 0 | 0 | 0 |
| ins\_c | 0 | 0 | 0 | 1 | 1 | -1 | 0 | 0 | 0 | 0 | 0 | 0 | -1 | 0 | 0 | 0 | 0 | 0 | 0 |
| hxan\_c | 0 | 0 | 0 | 0 | 0 | 1 | 0 | 0 | 0 | 0 | 0 | 0 | 0 | -1 | 0 | 0 | 0 | 0 | 0 |
| r1p\_c | 0 | 0 | 0 | 0 | 0 | 1 | -1 | 0 | 0 | 0 | 0 | 0 | 0 | 0 | 0 | 0 | 0 | 0 | 0 |
| r5p\_c | 0 | 0 | 0 | 0 | 0 | 0 | 1 | -1 | 0 | 0 | 0 | 0 | 0 | 0 | 0 | 0 | 0 | 0 | 0 |
| prpp\_c | 0 | 0 | 0 | 0 | 0 | 0 | 0 | 1 | -1 | 0 | 0 | 0 | 0 | 0 | 0 | 0 | 0 | 0 | 0 |
| atp\_c | -1 | 0 | 0 | 0 | 0 | 0 | 0 | -2 | 0 | 1 | 0 | 0 | 0 | 0 | 0 | 0 | 0 | 0 | 0 |
| adp\_c | 1 | 0 | 0 | 0 | 0 | 0 | 0 | 2 | 0 | -1 | 0 | 0 | 0 | 0 | 0 | 0 | 0 | 0 | 0 |
| amp\_c | 1 | -1 | -1 | 0 | 0 | 0 | 0 | 0 | 1 | 0 | 0 | 0 | 0 | 0 | 0 | 0 | -1 | 0 | 0 |
| pi\_c | 0 | 1 | 0 | 1 | 0 | -1 | 0 | 0 | 2 | -1 | 0 | 0 | 0 | 0 | 0 | -1 | 0 | 0 | 0 |
| nh3\_c | 0 | 0 | 1 | 0 | 1 | 0 | 0 | 0 | 0 | 0 | 0 | 0 | 0 | 0 | -1 | 0 | 0 | 0 | 0 |
| h\_c | 1 | 0 | 0 | 0 | 0 | 0 | 0 | 1 | 1 | -1 | 0 | 0 | 0 | 0 | 0 | 0 | 0 | -1 | 0 |
| h2o\_c | 0 | -1 | -1 | -1 | -1 | 0 | 0 | 0 | -1 | 1 | 0 | 0 | 0 | 0 | 0 | 0 | 0 | 0 | -1 |

##### Model Parameterization¶

###### Steady State fluxes¶

Steady state fluxes can be computed as a summation of the MinSpan pathway vectors. Pathways are obtained using MinSpan.

Using these pathways and literature sources, independent fluxes can be defined in order to calculate the steady state flux vector. Because we have five pathways, we have to specify five fluxes to set the steady state.

1. In a steady state, the synthesis of AMP is balanced by degradation, that is \(v\_{EX\_{amp}}=0\). Thus, the sum of the flux through the first three pathways must be balanced by the fourth to make the AMP exchange rate zero. Note that the fifth pathway has no net AMP exchange rate.
2. The fifth pathway is uniquely defined by either the exchange rate of hypoxanthine or adenine. These two exchange rates are not independent. The uptake rate of adenine is approximately 0.014 mM/hr (Joshi, 1990).
3. The exchange rate of adenosine would specify the relative rate of pathways one and four. The rate of \(v\_{ADNK1}\) is set to 0.12 mM/hr, specifying the flux through \(\textbf{p}\_{4}\). The net uptake rate of adenosine is set at 0.01 mM/hr, specifying the flux of \(\textbf{p}\_{1}\) to be 0.11 mM/hr.
4. Since \(\textbf{p}\_{1}\) and \(\textbf{p}\_{4}\) differ by 0.01 mM/hr in favor of AMP synthesis, it means that the sum of \(\textbf{p}\_{2}\) and \(\textbf{p}\_{3}\) has to be 0.01 mM/hr. To specify the contributions to that sum of the two pathways, we would have to know one of the internal rates, such as the deaminases or the phosphorylases. We set the flux of adenosine deaminase to 0.01 mM/hr as it a very low flux enzyme based on an earlier model (Joshi, 1990).
5. This assignment sets the flux of \(\textbf{p}\_{2}\) to zero and \(\textbf{p}\_{3}\) to 0.01 mM/hr. We pick the flux through \(\textbf{p}\_{2}\) to be zero since it overlaps with \(\textbf{p}\_{5}\) and gives flux values to all the reactions in the pathways.

With these pathays and numerical values, the steady state flux vector can be computed as the weighted sum of the corresponding basis vectors. The steady state flux vector is computed as an inner product:

```
[8]:
```

```
minspan_paths = [
    [0, 1, 0, 0, 0, 0, 0, 0, 0, 0, 1, 0, 0, 0, 0, 1,-1, 1,-1],
    [0, 0, 1, 1, 0, 0, 0, 0, 0, 0, 0, 0, 1, 0, 1, 1,-1, 1,-2],
    [0, 1, 0, 0, 1, 0, 0, 0, 0, 0, 0, 0, 1, 0, 1, 1,-1, 1,-2],
    [1, 0, 0, 0, 0, 0, 0, 0, 0, 1,-1, 0, 0, 0, 0,-1, 1,-1, 1],
    [0, 0, 1, 1, 0, 1, 1, 1, 1, 2, 0,-1, 0, 1, 1, 0, 0, 0,-1]]
ampsn.compute_steady_state_fluxes(
    pathways=minspan_paths,
    independent_fluxes={
        SK_amp_c: 0.0,
        SK_ade_c: -0.014,
        ADNK1: 0.12,
        SK_adn_c: -0.01,
        AMPDA: 0.014},
    update_reactions=True)

print("Steady State Fluxes\n-------------------")
for reaction, steady_state_flux in ampsn.steady_state_fluxes.items():
    print("{0}: {1:.6f}".format(reaction.flux_symbol_str, steady_state_flux))
```

```
Steady State Fluxes
-------------------
v_ADNK1: 0.120000
v_NTD7: 0.120000
v_AMPDA: 0.014000
v_NTD11: 0.014000
v_ADA: 0.010000
v_PUNP5: 0.014000
v_PPM: 0.014000
v_PRPPS: 0.014000
v_ADPT: 0.014000
v_ATPM: 0.148000
v_SK_adn_c: -0.010000
v_SK_ade_c: -0.014000
v_SK_ins_c: 0.010000
v_SK_hxan_c: 0.014000
v_SK_nh3_c: 0.024000
v_SK_pi_c: 0.000000
v_SK_amp_c: 0.000000
v_SK_h_c: 0.000000
v_SK_h2o_c: -0.024000
```

###### Initial Conditions¶

Once the network has been built, the concentrations can be added to the metabolites. These concentrations are also treated as the initial conditions required to integrate and simulate the model’s ordinary differential equations (ODEs). The metabolite concentrations are added to each individual metabolite using the `MassMetabolite.initial_condition` (alias: `MassMetabolite.ic`) attribute setter methods. Once added, the model will be able to return the initial conditions as a dictionary
through the `MassModel.initial_conditions` attribute.

```
[9]:
```

```
adn_c.ic = 1.2e-3
ade_c.ic = 1.0e-3
imp_c.ic = 1.0e-2
ins_c.ic = 1.0e-3
hxan_c.ic = 2.0e-3
r1p_c.ic = 6.0e-2
r5p_c.ic = 4.94e-3
prpp_c.ic = 5.0e-3
atp_c.ic = 1.6
adp_c.ic = 0.29
amp_c.ic = 8.67281e-2
pi_c.ic = 2.5
nh3_c.ic = 9.1002e-2
h_c.ic = 6.30957e-5
h2o_c.ic = 1

print("Initial Conditions\n------------------")
for metabolite, ic_value in ampsn.initial_conditions.items():
    print("{0}: {1}".format(metabolite, ic_value))
```

```
Initial Conditions
------------------
adn_c: 0.0012
ade_c: 0.001
imp_c: 0.01
ins_c: 0.001
hxan_c: 0.002
r1p_c: 0.06
r5p_c: 0.00494
prpp_c: 0.005
atp_c: 1.6
adp_c: 0.29
amp_c: 0.0867281
pi_c: 2.5
nh3_c: 0.091002
h_c: 6.30957e-05
h2o_c: 1
```

###### Equilibirum Constants¶

After adding initial conditions and steady state fluxes, the equilibrium constants are defined using the `MassReaction.equilibrium_constant` (alias: `MassReaction.Keq`) setter method.

```
[10]:
```

```
PUNP5.Keq = 0.09
PPM.Keq = 13.3

SK_adn_c.Keq = 1
SK_ade_c.Keq = 1
SK_ins_c.Keq = 1
SK_hxan_c.Keq = 1
SK_amp_c.Keq = 1
SK_h_c.Keq = 1
SK_h2o_c.Keq = 1
SK_pi_c.Keq = 1
SK_nh3_c.Keq = 1

print("Equilibrium Constants\n---------------------")
for reaction in ampsn.reactions:
    print("{0}: {1}".format(reaction.Keq_str, reaction.Keq))
```

```
Equilibrium Constants
---------------------
Keq_ADNK1: inf
Keq_NTD7: inf
Keq_AMPDA: inf
Keq_NTD11: inf
Keq_ADA: inf
Keq_PUNP5: 0.09
Keq_PPM: 13.3
Keq_PRPPS: inf
Keq_ADPT: inf
Keq_ATPM: inf
Keq_SK_adn_c: 1
Keq_SK_ade_c: 1
Keq_SK_ins_c: 1
Keq_SK_hxan_c: 1
Keq_SK_nh3_c: 1
Keq_SK_pi_c: 1
Keq_SK_amp_c: 1
Keq_SK_h_c: 1
Keq_SK_h2o_c: 1
```

###### Calculation of PERCs¶

By defining the equilibrium constant and steady state parameters, the values of the pseudo rate constants (PERCs) can be calculated and added to the model using the `MassModel.calculate_PERCs` method.

```
[11]:
```

```
percs = ampsn.calculate_PERCs(update_reactions=True)

print("Forward Rate Constants\n----------------------")
for reaction in ampsn.reactions:
    print("{0}: {1:.6f}".format(reaction.kf_str, reaction.kf))
```

```
Forward Rate Constants
----------------------
kf_ADNK1: 62.500000
kf_NTD7: 1.383635
kf_AMPDA: 0.161424
kf_NTD11: 1.400000
kf_ADA: 8.333333
kf_PUNP5: 12.000000
kf_PPM: 0.234787
kf_PRPPS: 1.107034
kf_ADPT: 2800.000000
kf_ATPM: 0.204138
kf_SK_adn_c: 100000.000000
kf_SK_ade_c: 100000.000000
kf_SK_ins_c: 100000.000000
kf_SK_hxan_c: 100000.000000
kf_SK_nh3_c: 100000.000000
kf_SK_pi_c: 100000.000000
kf_SK_amp_c: 100000.000000
kf_SK_h_c: 100000.000000
kf_SK_h2o_c: 100000.000000
```

##### QC/QA Model¶

Before simulating the model, it is important to ensure that the model is elementally balanced, and that the model can simulate. Therefore, the `qcqa_model` function from the `mass.util.qcqa` submodule is used to provide a report on the model quality and indicate whether simulation is possible and if not, what parameters and/or initial conditions are missing.

Generally, pseudoreactions (e.g. boundary exchanges, sinks, demands) are not elementally balanced. The `qcqa_model` function does not include elemental balancing of boundary reactions. However, some models contain pseudoreactions reprsenting a simplified mechanism, and show up in the returned report. The elemental imbalance of these pseudoreactions is therefore expected in certain reaction and should not be a cause for concern.

```
[12]:
```

```
qcqa_model(ampsn, parameters=True, concentrations=True,
           fluxes=True, superfluous=True, elemental=True)
```

```
╒══════════════════════════════════════════╕
│ MODEL ID: AMPSalvageNetwork              │
│ SIMULATABLE: True                        │
│ PARAMETERS NUMERICALY CONSISTENT: True   │
╞══════════════════════════════════════════╡
╘══════════════════════════════════════════╛
```

From the results of the QC/QA test, it can be seen that the model can be simulated and is numerically consistent.

##### Steady State and Model Validation¶

To find the steady state of the model and perform simulations, the model must first be loaded into a `Simulation`. In order to load a model into a `Simulation`, the model must be simulatable, meaning there are no missing numerical values that would prevent the integration of the ODEs that comprise the model. The `verbose` argument can be used while loading a model to produce a message indicating the successful loading of a model, or why a model could not load.

Once loaded into a `Simulation`, the `find_steady_state` method can be used with the `update_values` argument in order to update the initial conditions and fluxes of the model to a steady state (if necessary). The model can be simulated using the `simulate` method by passing the model to simulate, and a tuple containing the start time and the end time. The number of time points can also be included, but is optional.

After a successful simulation, two `MassSolution` objects are returned. The first `MassSolution` contains the concentration results of the simulation, and the second contains the flux results of the simulation.

To visually validate the steady state of the model, concentration and flux solutions can be plotted using the `plot_time_profile` function from `mass.visualization`. Alternatively, the `MassSolution.view_time_profile` property can be used to quickly generate a time profile for the results.

```
[13]:
```

```
# Setup simulation object
sim = Simulation(ampsn, verbose=True)
# Simulate from 0 to 1000 with 10001 points in the output
conc_sol, flux_sol = sim.simulate(ampsn, time=(0, 1e3, 1e4 + 1))
# Quickly render and display time profiles
conc_sol.view_time_profile()
```

```
Successfully loaded MassModel 'AMPSalvageNetwork' into RoadRunner.
```

###### Storing information and references¶

###### Compartment¶

Because the character “c” represents the cytosol compartment, it is recommended to define and set the compartment in the `MassModel.compartments` attribute.

```
[14]:
```

```
ampsn.compartments = {"c": "Cytosol"}
print(ampsn.compartments)
```

```
{'c': 'Cytosol'}
```

###### Units¶

All of the units for the numerical values used in this model are “Millimoles” for amount and “Liters” for volume (giving a concentration unit of ‘Millimolar’), and “Hours” for time. In order to ensure that future users understand the numerical values for model, it is important to define the `MassModel.units` attribute.

The `MassModel.units` is a `cobra.DictList` that contains only `UnitDefinition` objects from the `mass.core.unit` submodule. Each `UnitDefinition` is created from `Unit` objects representing the base units that comprise the `UnitDefinition`. These `Units` are stored in the `list_of_units` attribute. Pre-built units can be viewed using the `print_defined_unit_values` function from the `mass.core.unit` submodule. Alternatively, custom units can also be created using the
`UnitDefinition.create_unit` method. For more information about units, please see the module docstring for `mass.core.unit` submodule.

**Note:** It is important to note that this attribute will NOT track units, but instead acts as a reference for the user and others so that they can perform necessary unit conversions.

```
[15]:
```

```
# Using pre-build units to define UnitDefinitions
concentration = UnitDefinition("mM", name="Millimolar",
                               list_of_units=["millimole", "per_litre"])
time = UnitDefinition("hr", name="hour", list_of_units=["hour"])

# Add units to model
ampsn.add_units([concentration, time])
print(ampsn.units)
```

```
[<UnitDefinition Millimolar "mM" at 0x7fb557adf6d0>, <UnitDefinition hour "hr" at 0x7fb557adfad0>]
```

##### Export¶

After validation, the model is ready to be saved. The model can either be exported as a “.json” file or as an “.sbml” (“.xml”) file using their repsective submodules in `mass.io`.

To export the model, only the path to the directory and the model object itself need to be specified.

###### Export using SBML¶

```
[16]:
```

```
sbml.write_sbml_model(mass_model=ampsn, filename="SB2_" + ampsn.id + ".xml")
```

###### Export using JSON¶

```
[17]:
```

```
json.save_json_model(mass_model=ampsn, filename="SB2_" + ampsn.id + ".json")
```

#### Hemoglobin Model Construction¶

Based on Chapter 13 of [Pal11]

To construct a module of hemoglobin, first we import **MASSpy** and other essential packages. Constants used throughout the notebook are also defined.

```
[1]:
```

```
from os import path

import matplotlib.pyplot as plt

from sympy import Equality, Symbol, solveset, sympify, pprint

from cobra import DictList

from mass import (
    MassConfiguration, MassMetabolite, MassModel,
    MassReaction, Simulation, UnitDefinition)
from mass.test import create_test_model
from mass.io import json, sbml
from mass.util import strip_time, qcqa_model

mass_config = MassConfiguration()

mass_config.irreversible_Keq = float("inf")
```

##### Model Construction¶

The first step of creating a model of hemoglobin is to define the `MassModel`.

```
[2]:
```

```
hemoglobin = MassModel("Hemoglobin")
```

###### Metabolites¶

The next step is to define all of the metabolites using the `MassMetabolite` object. Some considerations for this step include the following:

1. It is important to use a clear and consistent format for identifiers and names when defining the `MassMetabolite` objects for various reasons, some of which include improvements to model clarity and utility, assurance of unique identifiers (required to add metabolites to the model), and consistency when collaborating and communicating with others.
2. In order to ensure our model is physiologically accurate, it is important to provide the `formula` argument with a string representing the chemical formula for each metabolite, and the `charge` argument with an integer representing the metabolite’s ionic charge (Note that neutrally charged metabolites are provided with 0). These attributes can always be set later if necessary using the `formula` and `charge` attribute set methods. To include the Hemoglobin macromolecule in the
   formula, brackets are used (e.g., [HB]).
3. To indicate that the cytosol is the cellular compartment in which the reactions occur, the string “c” is provided to the `compartment` argument.

This model will be created using identifiers and names found in the BiGG Database.

In this model, there are 13 metabolites inside the cytosol compartment. Note that for metabolites without BiGG identifiers are given ones that are similar to BiGG style.

```
[3]:
```

```
hb_c = MassMetabolite(
    "hb_c",
    name="Hemoglobin",
    formula="[HB]",
    charge=0,
    compartment="c",
    fixed=False)

hb_1o2_c = MassMetabolite(
    "hb_1o2_c",
    name="Oxyhemoglobin (1)",
    formula="[HB]-O2",
    charge=0,
    compartment="c",
    fixed=False)

hb_2o2_c = MassMetabolite(
    "hb_2o2_c",
    name="Oxyhemoglobin (2)",
    formula="[HB]-O4",
    charge=0,
    compartment="c",
    fixed=False)

hb_3o2_c = MassMetabolite(
    "hb_3o2_c",
    name="Oxyhemoglobin (3)",
    formula="[HB]-O6",
    charge=0,
    compartment="c",
    fixed=False)

hb_4o2_c = MassMetabolite(
    "hb_4o2_c",
    name="Oxyhemoglobin (4)",
    formula="[HB]-O8",
    charge=0,
    compartment="c",
    fixed=False)

dhb_c = MassMetabolite(
    "dhb_c",
    name="Deoxyhemoglobin",
    formula="[HB]-C3H3O10P2",
    charge=-5,
    compartment="c",
    fixed=False)

_23dpg_c = MassMetabolite(
    "_23dpg_c",
    name="2,3-Disphospho-D-glycerate",
    formula="C3H3O10P2",
    charge=-5,
    compartment="c",
    fixed=False)

_13dpg_c = MassMetabolite(
    "_13dpg_c",
    name="3-Phospho-D-glyceroyl phosphate",
    formula="C3H4O10P2",
    charge=-4,
    compartment="c",
    fixed=False)

_3pg_c = MassMetabolite(
    "_3pg_c",
    name="3-Phospho-D-glycerate",
    formula="C3H4O7P",
    charge=-3,
    compartment="c",
    fixed=False)

o2_c = MassMetabolite(
    "o2_c",
    name="Oxygen",
    formula="O2",
    charge=0,
    compartment="c",
    fixed=False)

h_c = MassMetabolite(
    "h_c",
    name="H+",
    formula="H",
    charge=1,
    compartment="c",
    fixed=False)

pi_c = MassMetabolite(
    "pi_c",
    name="Phosphate",
    formula="HPO4",
    charge=-2,
    compartment="c",
    fixed=False)

h2o_c = MassMetabolite(
    "h2o_c",
    name="H2O",
    formula="H2O",
    charge=0,
    compartment="c",
    fixed=False)
```

###### Reactions¶

Once all of the `MassMetabolite` objects for each metabolite, the next step is to define all of the reactions that occur and their stoichiometry.

1. As with the metabolites, it is also important to use a clear and consistent format for identifiers and names when defining when defining the `MassReaction` objects.
2. To make this model useful for integration with other models, it is important to provide a string to the `subsystem` argument. By providing the subsystem, the reactions can be easily obtained even when integrated with a significantly larger model through the `subsystem` attribute.
3. After the creation of each `MassReaction` object, the metabolites are added to the reaction using a dictionary where keys are the `MassMetabolite` objects and values are the stoichiometric coefficients (reactants have negative coefficients, products have positive ones).

This model will be created using identifiers and names found in the BiGG Database.

In this model, there are 7 reactions occuring inside the cytosol compartment.

```
[4]:
```

```
DPGase = MassReaction(
    "DPGase",
    name="Diphosphoglycerate phosphatase",
    subsystem=hemoglobin.id,
    reversible=False)
DPGase.add_metabolites({
    h2o_c: -1,
    _23dpg_c: -1,
    _3pg_c: 1,
    pi_c: 1})

DPGM = MassReaction(
    "DPGM",
    name="Diphosphoglyceromutase",
    subsystem=hemoglobin.id,
    reversible=True)
DPGM.add_metabolites({
    _13dpg_c: -1,
    _23dpg_c: 1,
    h_c: 1})

HBDPG = MassReaction(
    "HBDPG",
    name="Hemoglobin-23dpg binding",
    subsystem=hemoglobin.id,
    reversible=True)
HBDPG.add_metabolites({
    hb_c: -1,
    _23dpg_c: -1,
    dhb_c: 1})

HBO1 = MassReaction(
    "HBO1",
    name="Oxygen Loading (1)",
    subsystem=hemoglobin.id,
    reversible=True)
HBO1.add_metabolites({
    hb_c: -1,
    o2_c: -1,
    hb_1o2_c: 1})

HBO2 = MassReaction(
    "HBO2",
    name="Oxygen Loading (2)",
    subsystem=hemoglobin.id,
    reversible=True)
HBO2.add_metabolites({
    hb_1o2_c: -1,
    o2_c: -1,
    hb_2o2_c: 1})

HBO3 = MassReaction(
    "HBO3",
    name="Oxygen Loading (3)",
    subsystem=hemoglobin.id,
    reversible=True)
HBO3.add_metabolites({
    hb_2o2_c: -1,
    o2_c: -1,
    hb_3o2_c: 1})

HBO4 = MassReaction(
    "HBO4",
    name="Oxygen Loading (4)",
    subsystem=hemoglobin.id,
    reversible=True)
HBO4.add_metabolites({
    hb_3o2_c: -1,
    o2_c: -1,
    hb_4o2_c: 1})
```

After generating the reactions, all reactions are added to the model through the `MassModel.add_reactions` class method. Adding the `MassReaction` objects will also add their associated `MassMetabolite` objects if they have not already been added to the model.

```
[5]:
```

```
hemoglobin.add_reactions([
    DPGase, DPGM, HBDPG, HBO1, HBO2, HBO3, HBO4])

for reaction in hemoglobin.reactions:
    print(reaction)
```

```
DPGase: _23dpg_c + h2o_c --> _3pg_c + pi_c
DPGM: _13dpg_c <=> _23dpg_c + h_c
HBDPG: _23dpg_c + hb_c <=> dhb_c
HBO1: hb_c + o2_c <=> hb_1o2_c
HBO2: hb_1o2_c + o2_c <=> hb_2o2_c
HBO3: hb_2o2_c + o2_c <=> hb_3o2_c
HBO4: hb_3o2_c + o2_c <=> hb_4o2_c
```

###### Boundary reactions¶

After generating the reactions, the next step is to add the boundary reactions and boundary conditions (the concentrations of the boundary ‘metabolites’ of the system). This can easily be done using the `MassModel.add_boundary` method. With the generation of the boundary reactions, the system becomes an open system, allowing for the flow of mass through the biochemical pathways of the model. Once added, the model will be able to return the boundary conditions as a dictionary through the
`MassModel.boundary_conditions` attribute.

All boundary reactions are originally created with the metabolite as the reactant. However, there are times where it would be preferable to represent the metabolite as the product. For these situtations, the `MassReaction.reverse_stoichiometry` method can be used with its `inplace` argument to create a new `MassReaction` or simply reverse the stoichiometry for the current `MassReaction.`

In this model, there is 1 boundary reaction that must be defined.

```
[6]:
```

```
SK_o2_c = hemoglobin.add_boundary(
    metabolite=o2_c, boundary_type="sink", subsystem="Pseudoreaction",
    boundary_condition=0.0200788)

print("Boundary Reactions and Values\n-----------------------------")
for reaction in hemoglobin.boundary:
    boundary_met = reaction.boundary_metabolite
    bc_value = hemoglobin.boundary_conditions.get(boundary_met)
    print("{0}\n{1}: {2}\n".format(
        reaction, boundary_met, bc_value))
```

```
Boundary Reactions and Values
-----------------------------
SK_o2_c: o2_c <=>
o2_b: 0.0200788
```

###### Ordering of internal species and reactions¶

Sometimes, it is also desirable to reorder the metabolite and reaction objects inside the model to follow the physiology. To reorder the internal objects, one can use `cobra.DictList` containers and the `DictList.get_by_any` method with the list of object identifiers in the desirable order. To ensure all objects are still present and not forgotten in the model, a small QA check is also performed.

```
[7]:
```

```
new_metabolite_order = [
    "_23dpg_c", "hb_c", "hb_1o2_c", "hb_2o2_c",
    "hb_3o2_c", "hb_4o2_c", "dhb_c", "_13dpg_c",
    "_3pg_c",  "o2_c", "pi_c", "h_c", "h2o_c"]

if len(hemoglobin.metabolites) == len(new_metabolite_order):
    hemoglobin.metabolites = DictList(
        hemoglobin.metabolites.get_by_any(new_metabolite_order))

new_reaction_order = [
    "DPGM", "DPGase", "HBO1", "HBO2",
    "HBO3", "HBO4", "HBDPG", "SK_o2_c"]

if len(hemoglobin.reactions) == len(new_reaction_order):
    hemoglobin.reactions = DictList(
        hemoglobin.reactions.get_by_any(new_reaction_order))

hemoglobin.update_S(array_type="DataFrame", dtype=int)
```

```
[7]:
```

|  | DPGM | DPGase | HBO1 | HBO2 | HBO3 | HBO4 | HBDPG | SK\_o2\_c |
| --- | --- | --- | --- | --- | --- | --- | --- | --- |
| \_23dpg\_c | 1 | -1 | 0 | 0 | 0 | 0 | -1 | 0 |
| hb\_c | 0 | 0 | -1 | 0 | 0 | 0 | -1 | 0 |
| hb\_1o2\_c | 0 | 0 | 1 | -1 | 0 | 0 | 0 | 0 |
| hb\_2o2\_c | 0 | 0 | 0 | 1 | -1 | 0 | 0 | 0 |
| hb\_3o2\_c | 0 | 0 | 0 | 0 | 1 | -1 | 0 | 0 |
| hb\_4o2\_c | 0 | 0 | 0 | 0 | 0 | 1 | 0 | 0 |
| dhb\_c | 0 | 0 | 0 | 0 | 0 | 0 | 1 | 0 |
| \_13dpg\_c | -1 | 0 | 0 | 0 | 0 | 0 | 0 | 0 |
| \_3pg\_c | 0 | 1 | 0 | 0 | 0 | 0 | 0 | 0 |
| o2\_c | 0 | 0 | -1 | -1 | -1 | -1 | 0 | -1 |
| pi\_c | 0 | 1 | 0 | 0 | 0 | 0 | 0 | 0 |
| h\_c | 1 | 0 | 0 | 0 | 0 | 0 | 0 | 0 |
| h2o\_c | 0 | -1 | 0 | 0 | 0 | 0 | 0 | 0 |

###### Computing the steady state concentrations.¶

The binding of the two ligands, oxygen and DPG23, to hemoglobin is a rapid process. Since hemoglobin is confined to the RBC, we can use equilibrium assumptions for the binding reactions.

1. The binding of oxygen is at equilibrium for each form of oxygenated hemoglobin.
2. The binding of DPG23 to hemoglobin is also at equilibrium
3. The total mass of hemoglobin is a constant

These six equations have six unknowns (the six forms of Hb) and need to be solved simultaneously as a function of the oxygen and DPG23 concentrations. The equilibrium relationships can be combined with the \(\text{Hb}\_{\mathrm{tot}}\) mass balance, and this equation is solved for \(\text{Hb}\_{\mathrm{0}}\) for given oxygen and 23DPG concentrations. Then the steady state concentrations for all other forms of hemoglobin can be computed from the equilibrium relationships.

To do this, the **SymPy** package is utilized. The metabolites and equilibrium constants are defined as `sympy.Symbol` objects, and then the equilibrium expressions are converted into `sympy.Equality` objects for symbolic calculations.

```
[8]:
```

```
metabolites = {metabolite.id: Symbol(metabolite.id)
               for metabolite in hemoglobin.metabolites}

concentration_equations = {}
# Iterate through reactions assumed to be at equilibrium
for reaction in [HBO1, HBO2, HBO3, HBO4, HBDPG]:
    equilibrium_expression = Equality(
        Symbol(reaction.Keq_str),
        strip_time(reaction.get_mass_action_ratio()))
    # Find the hemoglobin form being made as a product (bound to most oxygen)
    hb_product = [
        Symbol(metabolite.id) for metabolite in reaction.products
        if metabolite.id not in ["_23dpg_c", "hb_c", "o2_c"]].pop()
    # Solve equation for the desired form hemoglobin
    equation = solveset(equilibrium_expression, hb_product)
    equation = next(iter(equation))
    # Update equilibrium expression dict with the equation
    # for the bound form of hemoglobin. These equations will
    # be dependent on hb_c, o2_c, and _23dpg_c.
    concentration_equations.update({
        hb_product: equation.subs(concentration_equations)})
# Specify an equation for the total amount of hemoglobin
HB_total_symbol = Symbol("HB-Total")
HB_total = Equality(
    HB_total_symbol,
    sympify("+".join([
        metabolite.id for metabolite in hemoglobin.metabolites
        if "hb" in metabolite.id]), locals=metabolites))
HB_total = HB_total.subs(concentration_equations)
pprint(HB_total)
```

```
HB-Total = Keq_HBDPG⋅_23dpg_c⋅hb_c + Keq_HBO1⋅Keq_HBO2⋅Keq_HBO3⋅Keq_HBO4⋅hb_c⋅

     4                                        3
o_2_c  + Keq_HBO1⋅Keq_HBO2⋅Keq_HBO3⋅hb_c⋅o_2_c  + Keq_HBO1⋅Keq_HBO2⋅hb_c⋅o_2_c

2
  + Keq_HBO1⋅hb_c⋅o_2_c + hb_c
```

At this point, the numerical values for the equilibrium constant and the total concetration of hemoglobin are specified. The total amount of hemoglobin is a constant, at circa 7.3 mM. These values are substituted into the current equations.

```
[9]:
```

```
numerical_values = {HB_total_symbol: 7.3}

DPGM.Keq = 2.3*1e6
HBO1.Keq = 41.8352
HBO2.Keq = 73.2115
HBO3.Keq = 177.799
HBO4.Keq = 1289.92
HBDPG.Keq = 1/4
SK_o2_c.Keq = 1

numerical_values.update({
    Symbol(reaction.Keq_str): reaction.Keq
    for reaction in hemoglobin.reactions})

concentration_equations.update({
    hb_form: equation.subs(numerical_values)
    for hb_form, equation in concentration_equations.items()})
HB_total = HB_total.subs(numerical_values)
pprint(HB_total)
```

```
                                                     4
7.3 = 0.25⋅_23dpg_c⋅hb_c + 702446487.27335⋅hb_c⋅o_2_c  + 544565.932207695⋅hb_c

      3                          2
⋅o_2_c  + 3062.8177448⋅hb_c⋅o_2_c  + 41.8352⋅hb_c⋅o_2_c + hb_c
```

To find the steady state, we have to specify the numerical values of the variables that characterize the network environment. The flux through the Rapoport-Luebering shunt is typically about 0.44 mM/hr (Schrader 1993). The steady state concentration of 23DPG is typically about 3.1 mM (Mehta 2005). The concentration of oxygen that we chose to solve for the steady state is 70 mmHg, that is mid way between 100 mmHg in the lung, and 40 mmHg in tissue. Using these numbers, the computed steady state
concentrations are obtained, as:

```
[10]:
```

```
# Define known concentrations
concentrations = {
    metabolites["_23dpg_c"]: 3.1,
    metabolites["o2_c"]: 70*2.8684*1e-4}
# Convert the solution into a numerical value
hb_conc =  next(iter(solveset(
    HB_total.subs(concentrations),
    Symbol("hb_c"))))
concentrations.update({metabolites["hb_c"]: hb_conc})
# Solve for the rest of the hemoglobin concentrations
for hb_form, equation in concentration_equations.items():
    equation = equation.subs(concentrations)
    concentrations.update({hb_form: equation})
```

Once the steady state concentrations have been determined, the hemoglobin module can be updated. The remaining concentrations are obtained from the glycolysis module.

```
[11]:
```

```
glycolysis = create_test_model("SB2_Glycolysis.json")

for metabolite_symbol, value_symbol in concentrations.items():
    metabolite = hemoglobin.metabolites.get_by_id(str(metabolite_symbol))
    metabolite.ic = float(value_symbol)

for met in hemoglobin.metabolites:
    if met.ic is None:
        met.ic = glycolysis.metabolites.get_by_id(str(met)).ic

for metabolite, concentration in hemoglobin.initial_conditions.items():
    print("{0}: {1:.6f}".format(metabolite, concentration))
```

```
_23dpg_c: 3.100000
hb_c: 0.059625
hb_1o2_c: 0.050085
hb_2o2_c: 0.073625
hb_3o2_c: 0.262842
hb_4o2_c: 6.807613
dhb_c: 0.046210
_13dpg_c: 0.000243
_3pg_c: 0.077300
o2_c: 0.020079
pi_c: 2.500000
h_c: 0.000090
h2o_c: 1.000000
```

With the steady state concentrations and steady state flux values, the PERCs can be calculated. For this module, the PERCs for the binding of hemoglobin to oxygen will be set manually to better reflect the physiology.

**Note:** Reactions at equilibrium have a steady state flux of 0.

```
[12]:
```

```
DPGM.v = 0.441
DPGase.v = 0.441
HBO1.v = 0
HBO2.v = 0
HBO3.v = 0
HBO4.v = 0
HBDPG.v = 0
SK_o2_c.v = 0

hemoglobin.calculate_PERCs(update_reactions=True)

HBO1.kf = 506935
HBO2.kf = 511077
HBO3.kf = 509243
HBO4.kf = 501595
HBDPG.kf =519613
SK_o2_c.kf = 509726
```

##### QC/QA Model¶

Before simulating the model, it is important to ensure that the model is elementally balanced, and that the model can simulate. Therefore, the `qcqa_model` function from `mass.util.qcqa` is used to provide a report on the model quality and indicate whether simulation is possible and if not, what parameters and/or initial conditions are missing.

```
[13]:
```

```
qcqa_model(hemoglobin,  parameters=True, concentrations=True,
           fluxes=True, superfluous=True, elemental=True)
```

```
╒══════════════════════════════════════════╕
│ MODEL ID: Hemoglobin                     │
│ SIMULATABLE: True                        │
│ PARAMETERS NUMERICALY CONSISTENT: True   │
╞══════════════════════════════════════════╡
╘══════════════════════════════════════════╛
```

From the results of the QC/QA test, it can be seen that the model can be simulated and is numerically consistent.

##### Steady State and Model Validation¶

In order to determine whether the module can be successfully integrated into a model, another model can be loaded, merged with the module, and simulated. To validate this module, it will be merged with a glycolysis model.

To find the steady state of the model and perform simulations, the model must first be loaded into a `Simulation`. In order to load a model into a `Simulation`, the model must be simulatable, meaning there are no missing numerical values that would prevent the integration of the ODEs that comprise the model. The `verbose` argument can be used while loading a model to produce a message indicating the successful loading of a model, or why a model could not load.

Once loaded into a `Simulation`, the `find_steady_state` method can be used with the `update_values` argument in order to update the initial conditions and fluxes of the model to a steady state (if necessary). The model can be simulated using the `simulate` method by passing the model to simulate, and a tuple containing the start time and the end time. The number of time points can also be included, but is optional.

After a successful simulation, two `MassSolution` objects are returned. The first `MassSolution` contains the concentration results of the simulation, and the second contains the flux results of the simulation.

To visually validate the steady state of the model, concentration and flux solutions can be plotted using the `plot_time_profile` function from `mass.visualization`. Alternatively, the `MassSolution.view_time_profile` property can be used to quickly generate a time profile for the results.

```
[14]:
```

```
glyc_hb = glycolysis.merge(hemoglobin, inplace=False)

# Setup simulation object, ensure model is at steady state
sim = Simulation(glyc_hb, verbose=True)
sim.find_steady_state(glyc_hb, strategy="simulate", update_values=True)
# Simulate from 0 to 1000 with 10001 points in the output
conc_sol, flux_sol = sim.simulate(glyc_hb, time=(0, 1e3, 1e4 + 1))
# Quickly render and display time profiles
conc_sol.view_time_profile()
```

```
Successfully loaded MassModel 'Glycolysis_Hemoglobin' into RoadRunner.
```

###### Storing information and references¶

###### Compartment¶

Because the character “c” represents the cytosol compartment, it is recommended to define and set the compartment in the `MassModel.compartments` attribute.

```
[15]:
```

```
hemoglobin.compartments = {"c": "Cytosol"}
print(hemoglobin.compartments)
```

```
{'c': 'Cytosol'}
```

###### Units¶

All of the units for the numerical values used in this model are “Millimoles” for amount and “Liters” for volume (giving a concentration unit of ‘Millimolar’), and “Hours” for time. In order to ensure that future users understand the numerical values for model, it is important to define the `MassModel.units` attribute.

The `MassModel.units` is a `cobra.DictList` that contains only `UnitDefinition` objects from the `mass.core.unit` submodule. Each `UnitDefinition` is created from `Unit` objects representing the base units that comprise the `UnitDefinition`. These `Units` are stored in the `list_of_units` attribute. Pre-built units can be viewed using the `print_defined_unit_values` function from the `mass.core.unit` submodule. Alternatively, custom units can also be created using the
`UnitDefinition.create_unit` method. For more information about units, please see the module docstring for `mass.core.unit` submodule.

**Note:** It is important to note that this attribute will NOT track units, but instead acts as a reference for the user and others so that they can perform necessary unit conversions.

```
[16]:
```

```
# Using pre-build units to define UnitDefinitions
concentration = UnitDefinition("mM", name="Millimolar",
                               list_of_units=["millimole", "per_litre"])
time = UnitDefinition("hr", name="hour", list_of_units=["hour"])

# Add units to model
hemoglobin.add_units([concentration, time])
print(hemoglobin.units)
```

```
[<UnitDefinition Millimolar "mM" at 0x7f9d473a5d90>, <UnitDefinition hour "hr" at 0x7f9d474a9f50>]
```

##### Export¶

After validation, the model is ready to be saved. The model can either be exported as a “.json” file or as an “.sbml” (“.xml”) file using their repsective submodules in `mass.io`.

To export the model, only the path to the directory and the model object itself need to be specified.

###### Export using SBML¶

```
[17]:
```

```
sbml.write_sbml_model(mass_model=hemoglobin, filename="SB2_" + hemoglobin.id + ".xml")
```

###### Export using JSON¶

```
[18]:
```

```
json.save_json_model(mass_model=hemoglobin, filename="SB2_" + hemoglobin.id + ".json")
```

#### Phosphofructokinase (PFK) Model Construction¶

Based on Chapter 14 of [Pal11]

To construct the phosphofructokinase module, first we import **MASSpy** and other essential packages. Constants used throughout the notebook are also defined.

```
[1]:
```

```
from operator import attrgetter
from os import path

import matplotlib.pyplot as plt

import numpy as np

from scipy import optimize

import sympy as sym

from cobra import DictList

from mass import MassConfiguration, MassMetabolite, Simulation, UnitDefinition
from mass.enzyme_modules import EnzymeModule
from mass.io import json, sbml
from mass.test import create_test_model
from mass.util.expressions import Keq2k, k2Keq, strip_time
from mass.util.matrix import matrix_rank
from mass.util.qcqa import qcqa_model

mass_config = MassConfiguration()

mass_config.irreversible_Keq = float("inf")
```

Note that the total enzyme concentration of PFK is \(33 nM = 0.033 \mu M = 0.000033 mM\).

For the construction of the `EnzymeModule` for PFK, the following assumptions were made:

1. The enzyme is a homotetramer.
2. The enzyme binding and catalyzation of substrates occurs in an ordered sequential mechanism.
3. The mechanism of allosteric regulation is based on the Monod-Wyman-Changeux (MWC) model for allosteric transitions of homoproteins.

##### Module Construction¶

The first step of creating the PFK module is to define the `EnzymeModule`. The `EnzymeModule` is an extension of the `MassModel`, with additional enzyme-specific attributes that aid in the construction, validation, and utilization of the module.

**Note:** All `EnzymeModule` specific attributes start will start the prefix “enzyme” or “enzyme\_module”.

```
[2]:
```

```
PFK = EnzymeModule("PFK", name="Phosphofructokinase",
                   subsystem="Glycolysis")
```

###### Metabolites¶

###### Ligands¶

The next step is to define all of the metabolites using the `MassMetabolite` object. For `EnzymeModule` objects, the `MassMetabolite` objects will be refered to as ligands, for these `MassMetabolite` form a complex with the enzyme to serve some biological purpose. Some considerations for this step include the following:

1. It is important to use a clear and consistent format for identifiers and names when defining the `MassMetabolite` objects for various reasons, some of which include improvements to model clarity and utility, assurance of unique identifiers (required to add metabolites to the model), and consistency when collaborating and communicating with others.
2. In order to ensure our model is physiologically accurate, it is important to provide the `formula` argument with a string representing the chemical formula for each metabolite, and the `charge` argument with an integer representing the metabolite’s ionic charge (Note that neutrally charged metabolites are provided with 0). These attributes can always be set later if necessary using the `formula` and `charge` attribute setter methods.
3. To indicate that the cytosol is the cellular compartment in which the reactions occur, the string “c” is provided to the `compartment` argument.

This model will be created using identifiers and names found in the BiGG Database.

The ligands correspond to the activators, inhibitors, cofactors, substrates, and products involved in the enzyme catalyzed reaction. In this model, there are 6 species which must be considered.

```
[3]:
```

```
f6p_c = MassMetabolite(
    "f6p_c",
    name="D-Fructose 6-phosphate",
    formula="C6H11O9P",
    charge=-2,
    compartment="c")
fdp_c = MassMetabolite(
    "fdp_c",
    name="D-Fructose 1,6-bisphosphate",
    formula="C6H10O12P2",
    charge=-4,
    compartment="c")
atp_c = MassMetabolite(
    "atp_c",
    name="ATP",
    formula="C10H12N5O13P3",
    charge=-4,
    compartment="c")
adp_c = MassMetabolite(
    "adp_c",
    name="ADP",
    formula="C10H12N5O10P2",
    charge=-3,
    compartment="c")
amp_c = MassMetabolite(
    "amp_c",
    name="AMP",
    formula="C10H12N5O7P",
    charge=-2,
    compartment="c")
h_c = MassMetabolite(
    "h_c",
    name="H+",
    formula="H",
    charge=1,
    compartment="c")
```

After generating the ligands, they are added to the `EnzymeModule` through the `add_metabolites` method. The ligands of the `EnzymeModule` can be viewed as a `DictList` through the `enzyme_module_ligands` attribute.

```
[4]:
```

```
# Add the metabolites to the EnzymeModule
PFK.add_metabolites([f6p_c, fdp_c, atp_c, adp_c, amp_c, h_c])
# Access DictList of ligands and print
print("All {0} Ligands: {1}".format(
    PFK.id, "; ".join([m.id for m in PFK.enzyme_module_ligands])))
```

```
All PFK Ligands: f6p_c; fdp_c; atp_c; adp_c; amp_c; h_c
```

The `enzyme_module_ligands_categorized` attribute can be used to assign metabolites to groups of user-defined categories by providing a dictionary where keys are the categories and values are the metabolites. Note that any metabolite can be placed in more than one category.

```
[5]:
```

```
PFK.enzyme_module_ligands_categorized =  {
    "Substrates": f6p_c,
    "Cofactors": atp_c,
    "Activators": amp_c,
    "Inhibitors": atp_c,
    "Products": [fdp_c, adp_c, h_c]}

# Access DictList of ligands and print
print("All {0} ligands ({1} total):\n{2}\n".format(
    PFK.id, len(PFK.enzyme_module_ligands),
    str([m.id for m in PFK.enzyme_module_ligands])))

# Access categorized attribute for ligands and print
for group in PFK.enzyme_module_ligands_categorized:
    print("{0}: {1}".format(
        group.id, str([m.id for m in group.members])))
```

```
All PFK ligands (6 total):
['f6p_c', 'fdp_c', 'atp_c', 'adp_c', 'amp_c', 'h_c']

Substrates: ['f6p_c']
Cofactors: ['atp_c']
Activators: ['amp_c']
Inhibitors: ['atp_c']
Products: ['fdp_c', 'adp_c', 'h_c']
```

###### EnzymeModuleForms¶

The next step is to define the various states of the enzyme and enzyme-ligand complexes. These states can be represented through an `EnzymeModuleForm` object. Just like how `EnzymeModule` objects extend `MassModels`, the `EnzymeModuleForm` objects extend `MassMetabolite` objects, giving them the same functionality as a `MassMetabolite`. However, there are two important additional attrubutes that are specific to the `EnzymeModuleForm`.

- The first attribute is the `enzyme_module_id`. It is meant to hold the identifier or name of the corresponding `EnzymeModule`.
- The second attribute is the `bound_metabolites` attribute, designed to contain metabolites bound to the enzymatic site(s).
- Automatic generation of the `name`, `formula`, and `charge` attributes attributes utilize the `bound_metabolites` attribute, which can aid in identification of `EnzymeModuleForm` and mass and charge balancing of the reactions.

The most convenient way to make an `EnzymeModuleForm` is through the `EnzymeModule.make_enzyme_module_form` method. There are several reasons to use this method to generate the `EnzymeModuleForm` objects:

1. The only requirement to creating an `EnzymeModuleForm` is the identifier.
2. A string can optionally be provided for the `name` argument to set the corresponding `name` attribute, or it can automatically be generated and set by setting the string “Automatic” (case sensitve).
3. The `enzyme_module_id`, `formula` and `charge` attributes are set based on the identifier of the EnzymeModule and the MassMetabolite objects found in `bound_metabolites`.
4. Just like the `enzyme_module_ligands_categorized` attribute, there is the `enzyme_module_forms_categorized` attribute that behaves in a similar manner. Categories can be set at the time of construction by providing a string or a list of strings to the `categories` argument.
5. `EnzymeModuleForm` objects are automatically added to the `EnzymeModule` once created.

For this module, there are 20 `EnzymeModuleForm` objects that must be created. Because of the assumptions made for this module, a loop can be used to help automate the construction of the `EnzymeModuleForm` objects.

```
[6]:
```

```
# Number of identical subunits
n_subunits = 4

for i in range(n_subunits + 1):
    # Make enzyme module forms per number of bound activators (Up to 4 Total)
    PFK.make_enzyme_module_form(
        "pfk_R{0:d}_c".format(i),
        name="Automatic",
        categories=("Relaxed", "Free_Catalytic"),
        bound_metabolites={amp_c: i},
        compartment="c");

    PFK.make_enzyme_module_form(
        "pfk_R{0:d}_A_c".format(i),
        name="Automatic",
        categories=("Relaxed", "Complexed_ATP"),
        bound_metabolites={atp_c: 1, amp_c: i},
        compartment="c");

    PFK.make_enzyme_module_form(
        "pfk_R{0:d}_AF_c".format(i),
        name="Automatic",
        categories=("Relaxed", "Complexed_ATP_F6P"),
        bound_metabolites={atp_c: 1, f6p_c: 1, amp_c: i},
        compartment="c");

    # Make enzyme module forms per number of bound inhibitors (Up to 4 Total)
    PFK.make_enzyme_module_form(
        "pfk_T{0:d}_c".format(i),
        name="Automatic",
        categories="Tense",
        bound_metabolites={atp_c: i},
        compartment="c");

# Access DictList of enzyme module forms and print
print("All {0} enzyme module forms ({1} total):\n{2}\n".format(
    PFK.id, len(PFK.enzyme_module_forms),
    str([m.id for m in PFK.enzyme_module_forms])))

# Access categorized attribute for enzyme module forms and print
for group in PFK.enzyme_module_forms_categorized:
    print("{0}: {1}\n".format(
        group.id, str(sorted([m.id for m in group.members]))))
```

```
All PFK enzyme module forms (20 total):
['pfk_R0_c', 'pfk_R0_A_c', 'pfk_R0_AF_c', 'pfk_T0_c', 'pfk_R1_c', 'pfk_R1_A_c', 'pfk_R1_AF_c', 'pfk_T1_c', 'pfk_R2_c', 'pfk_R2_A_c', 'pfk_R2_AF_c', 'pfk_T2_c', 'pfk_R3_c', 'pfk_R3_A_c', 'pfk_R3_AF_c', 'pfk_T3_c', 'pfk_R4_c', 'pfk_R4_A_c', 'pfk_R4_AF_c', 'pfk_T4_c']

Relaxed: ['pfk_R0_AF_c', 'pfk_R0_A_c', 'pfk_R0_c', 'pfk_R1_AF_c', 'pfk_R1_A_c', 'pfk_R1_c', 'pfk_R2_AF_c', 'pfk_R2_A_c', 'pfk_R2_c', 'pfk_R3_AF_c', 'pfk_R3_A_c', 'pfk_R3_c', 'pfk_R4_AF_c', 'pfk_R4_A_c', 'pfk_R4_c']

Free_Catalytic: ['pfk_R0_c', 'pfk_R1_c', 'pfk_R2_c', 'pfk_R3_c', 'pfk_R4_c']

Complexed_ATP: ['pfk_R0_A_c', 'pfk_R1_A_c', 'pfk_R2_A_c', 'pfk_R3_A_c', 'pfk_R4_A_c']

Complexed_ATP_F6P: ['pfk_R0_AF_c', 'pfk_R1_AF_c', 'pfk_R2_AF_c', 'pfk_R3_AF_c', 'pfk_R4_AF_c']

Tense: ['pfk_T0_c', 'pfk_T1_c', 'pfk_T2_c', 'pfk_T3_c', 'pfk_T4_c']
```

##### Reactions¶

###### EnzymeModuleReactions¶

Once all of the `MassMetabolite` and `EnzymeModuleForm` objects have been created, the next step is to define all of the enzyme-ligand binding reactions and conformation trasitions that occur in its mechanism.

These reactions can be represented through an `EnzymeModuleReaction` object. As with the previous enzyme objects, `EnzymeModuleReactions` extend `MassReaction` objects to maintain the same functionality. However, as with the `EnzymeModuleForm`, the `EnzymeModuleReaction` has additional enzyme-specific attributes, such as the `enzyme_module_id`.

The most conveient way to make an `EnzymeModuleReaction` is through the `EnzymeModule.make_enzyme_module_reaction` method. There are several reasons to use this method to generate the EnzymeModuleReactions:

1. The only requirement to creating an `EnzymeModuleReaction` is an identifier.
2. A string can optionally be provided for the `name` argument to set the corresponding `name` attribute, or it can automatically be generated and set by setting the string “Automatic” (case sensitve).
3. There is an `enzyme_module_reactions_categorized` attribute that behaves in a similar manner as the previous categorized attributes. Categories can be set at the time of construction by providing a string or a list of strings to the `categories` argument.
4. `MassMetabolite` and `EnzymeModuleForm` objects that already exist in the `EnzymeModule` can be directly added to the newly created `EnzymeModuleReaction` by providing a dictionary to the optional `metabolites_to_add` argument using string identifiers (or the objects) as keys and their stoichiometric coefficients as the values.
5. `EnzymeModuleReactions` are automatically added to the `EnzymeModule` once created.

For this module, there are 24 `EnzymeModuleReactions` that must be created. Because of the assumptions made for this module, a loop can be used to help automate the construction of the `EnzymeModuleReactions`.

```
[7]:
```

```
for i in range(n_subunits + 1):
    # Make reactions for enzyme-ligand binding and catalytzation per number of bound activators (Up to 4 Total)
    PFK.make_enzyme_module_reaction(
        "PFK_R{0:d}1".format(i),
        name="Automatic",
        subsystem="Glycolysis",
        reversible=True,
        categories="atp_c_binding",
        metabolites_to_add={
            "pfk_R{0:d}_c".format(i): -1,
            "atp_c": -1,
            "pfk_R{0:d}_A_c".format(i): 1})

    PFK.make_enzyme_module_reaction(
        "PFK_R{0:d}2".format(i),
        name="Automatic",
        subsystem="Glycolysis",
        reversible=True,
        categories="f6p_c_binding",
        metabolites_to_add={
            "pfk_R{0:d}_A_c".format(i): -1,
            "f6p_c": -1,
            "pfk_R{0:d}_AF_c".format(i): 1})

    PFK.make_enzyme_module_reaction(
        "PFK_R{0:d}3".format(i),
        name="Automatic",
        subsystem="Glycolysis",
        reversible=False,
        categories="catalyzation",
        metabolites_to_add={
            "pfk_R{0:d}_AF_c".format(i): -1,
            "pfk_R{0:d}_c".format(i): 1,
            "adp_c": 1,
            "fdp_c": 1,
            "h_c": 1})

    if i < n_subunits:
        # Make enzyme reactions for enzyme-activator binding
        PFK.make_enzyme_module_reaction(
            "PFK_R{0:d}0".format(i + 1),
            name="Automatic",
            subsystem="Glycolysis",
            reversible=True,
            categories="amp_c_activation",
            metabolites_to_add={
                "pfk_R{0:d}_c".format(i): -1,
                "amp_c": -1,
                "pfk_R{0:d}_c".format(i + 1): 1})

        # Make enzyme reactions for enzyme-inhibitor binding
        PFK.make_enzyme_module_reaction(
            "PFK_T{0:d}".format(i + 1),
            name="Automatic",
            subsystem="Glycolysis",
            reversible=True,
            categories="atp_c_inhibition",
            metabolites_to_add={
                "pfk_T{0:d}_c".format(i): -1,
                "atp_c": -1,
                "pfk_T{0:d}_c".format(i + 1): 1})

# Make reaction representing enzyme transition from R to T state
PFK.make_enzyme_module_reaction(
    "PFK_L",
    name="Automatic",
    subsystem="Glycolysis",
    reversible=True,
    categories="RT_transition",
    metabolites_to_add={
        "pfk_R0_c": -1,
        "pfk_T0_c": 1})

# Access DictList of enzyme module reactions and print
print("All {0} enzyme module reactions ({1} total):\n{2}\n".format(
    PFK.id, len(PFK.enzyme_module_reactions),
    str([m.name for m in PFK.enzyme_module_reactions])))

# Access categorized attribute for enzyme module reactions and print
for group in PFK.enzyme_module_reactions_categorized:
    print("{0}: {1}\n".format(
        group.id, str(sorted([m.id for m in group.members]))))
```

```
All PFK enzyme module reactions (24 total):
['pfk_R0-atp binding', 'pfk_R0_A-f6p binding', 'pfk_R0_AF catalyzation', 'pfk_R0-amp binding', 'pfk_T0-atp binding', 'pfk_R1-atp binding', 'pfk_R1_A-f6p binding', 'pfk_R1_AF catalyzation', 'pfk_R1-amp binding', 'pfk_T1-atp binding', 'pfk_R2-atp binding', 'pfk_R2_A-f6p binding', 'pfk_R2_AF catalyzation', 'pfk_R2-amp binding', 'pfk_T2-atp binding', 'pfk_R3-atp binding', 'pfk_R3_A-f6p binding', 'pfk_R3_AF catalyzation', 'pfk_R3-amp binding', 'pfk_T3-atp binding', 'pfk_R4-atp binding', 'pfk_R4_A-f6p binding', 'pfk_R4_AF catalyzation', 'pfk_R0-pfk_T0 transition']

atp_c_binding: ['PFK_R01', 'PFK_R11', 'PFK_R21', 'PFK_R31', 'PFK_R41']

f6p_c_binding: ['PFK_R02', 'PFK_R12', 'PFK_R22', 'PFK_R32', 'PFK_R42']

catalyzation: ['PFK_R03', 'PFK_R13', 'PFK_R23', 'PFK_R33', 'PFK_R43']

amp_c_activation: ['PFK_R10', 'PFK_R20', 'PFK_R30', 'PFK_R40']

atp_c_inhibition: ['PFK_T1', 'PFK_T2', 'PFK_T3', 'PFK_T4']

RT_transition: ['PFK_L']
```

###### Create and Unify Rate Parameters¶

The next step is to unify rate parameters of binding steps that are not unique, allowing for those parameter values to be defined once and stored in the same place. Therefore, custom rate laws with custom parameters are used to reduce the number of parameters that need to be defined and better represent the module.

The rate law parameters can be unified using the `EnzymeModule.unify_rate_parameters` class method. This method requires a list of reactions whose rate laws that should be identical, along with a string representation of the new identifier to use on the unified parameters. There is also the optional `prefix` argument, which if set to True, will ensure the new parameter identifiers are prefixed with the `EnzymeModule` identifier. This can be used to help prevent custom parameters from being
replaced when multiple models are merged.

###### Allosteric Transitions: Symmetry Model¶

Once rate parameters are unified, the allosteric regulation of this enzyme must be accounted for. Because this module is to be based on the (Monod-Wyman-Changeux) MWC model for ligand binding and allosteric regulation, the rate laws of the allosteric binding reactions must be adjusted to reflect the symmetry in the module using the number of identical binding sites to help determine the scalars for the parameters.

For this module, PFK is considered a homotetramer, meaning it has four identical subunits \(\nu = 4\). Each subunit can be allosterically activated by AMP or inhibited by ATP. The helper functions `k2Keq`, `Keq2k`, and `strip_time` from the `mass.util` submodule will be used to help facilitate the rate law changes in this example so that the final rate laws are dependent on the forward rate (kf) and equilibrium (Keq) constants.

```
[8]:
```

```
abbreviations = ["A", "F", "I", "ACT"]
ligands = [atp_c, f6p_c, atp_c, amp_c]

for met, unified_id in zip(ligands, abbreviations):
    category = {"A": "binding",
                "F": "binding",
                "I": "inhibition",
                "ACT": "activation"}[unified_id]
    group = PFK.enzyme_module_reactions_categorized.get_by_id(
        "_".join((met.id, category)))
    reactions = sorted(group.members, key=attrgetter("id"))
    PFK.unify_rate_parameters(reactions, unified_id,
                              rate_type=2, enzyme_prefix=True)
    # Add the coefficients to make symmetry model rate laws for activation and inhibition
    if unified_id in ["I", "ACT"]:
        for i, reaction in enumerate(reactions):
            custom_rate = str(strip_time((reaction.rate)))
            custom_rate = custom_rate.replace(
                "kf_", "{0:d}*kf_".format(n_subunits - i))
            custom_rate = custom_rate.replace(
                "kr_", "{0:d}*kr_".format(i + 1))
            PFK.add_custom_rate(reaction, custom_rate)

PFK.unify_rate_parameters(
    PFK.enzyme_module_reactions_categorized.get_by_id("catalyzation").members,
    "PFK")
# Update rate laws to be in terms of kf and Keq
PFK.custom_rates.update(k2Keq(PFK.custom_rates))

# Access categorized attribute for enzyme module reactions and print
for group in PFK.enzyme_module_reactions_categorized:
    header = "Category: " + group.id
    print("\n" + header + "\n" + "-" * len(header))
    for reaction in sorted(group.members, key=attrgetter("id")):
        print(reaction.id + ": " + str(reaction.rate))
```

```
Category: atp_c_binding
-----------------------
PFK_R01: kf_PFK_A*(atp_c(t)*pfk_R0_c(t) - pfk_R0_A_c(t)/Keq_PFK_A)
PFK_R11: kf_PFK_A*(atp_c(t)*pfk_R1_c(t) - pfk_R1_A_c(t)/Keq_PFK_A)
PFK_R21: kf_PFK_A*(atp_c(t)*pfk_R2_c(t) - pfk_R2_A_c(t)/Keq_PFK_A)
PFK_R31: kf_PFK_A*(atp_c(t)*pfk_R3_c(t) - pfk_R3_A_c(t)/Keq_PFK_A)
PFK_R41: kf_PFK_A*(atp_c(t)*pfk_R4_c(t) - pfk_R4_A_c(t)/Keq_PFK_A)

Category: f6p_c_binding
-----------------------
PFK_R02: kf_PFK_F*(f6p_c(t)*pfk_R0_A_c(t) - pfk_R0_AF_c(t)/Keq_PFK_F)
PFK_R12: kf_PFK_F*(f6p_c(t)*pfk_R1_A_c(t) - pfk_R1_AF_c(t)/Keq_PFK_F)
PFK_R22: kf_PFK_F*(f6p_c(t)*pfk_R2_A_c(t) - pfk_R2_AF_c(t)/Keq_PFK_F)
PFK_R32: kf_PFK_F*(f6p_c(t)*pfk_R3_A_c(t) - pfk_R3_AF_c(t)/Keq_PFK_F)
PFK_R42: kf_PFK_F*(f6p_c(t)*pfk_R4_A_c(t) - pfk_R4_AF_c(t)/Keq_PFK_F)

Category: catalyzation
----------------------
PFK_R03: kf_PFK*pfk_R0_AF_c(t)
PFK_R13: kf_PFK*pfk_R1_AF_c(t)
PFK_R23: kf_PFK*pfk_R2_AF_c(t)
PFK_R33: kf_PFK*pfk_R3_AF_c(t)
PFK_R43: kf_PFK*pfk_R4_AF_c(t)

Category: amp_c_activation
--------------------------
PFK_R10: kf_PFK_ACT*(4*amp_c(t)*pfk_R0_c(t) - pfk_R1_c(t)/Keq_PFK_ACT)
PFK_R20: kf_PFK_ACT*(3*amp_c(t)*pfk_R1_c(t) - 2*pfk_R2_c(t)/Keq_PFK_ACT)
PFK_R30: kf_PFK_ACT*(2*amp_c(t)*pfk_R2_c(t) - 3*pfk_R3_c(t)/Keq_PFK_ACT)
PFK_R40: kf_PFK_ACT*(amp_c(t)*pfk_R3_c(t) - 4*pfk_R4_c(t)/Keq_PFK_ACT)

Category: atp_c_inhibition
--------------------------
PFK_T1: kf_PFK_I*(4*atp_c(t)*pfk_T0_c(t) - pfk_T1_c(t)/Keq_PFK_I)
PFK_T2: kf_PFK_I*(3*atp_c(t)*pfk_T1_c(t) - 2*pfk_T2_c(t)/Keq_PFK_I)
PFK_T3: kf_PFK_I*(2*atp_c(t)*pfk_T2_c(t) - 3*pfk_T3_c(t)/Keq_PFK_I)
PFK_T4: kf_PFK_I*(atp_c(t)*pfk_T3_c(t) - 4*pfk_T4_c(t)/Keq_PFK_I)

Category: RT_transition
-----------------------
PFK_L: kf_PFK_L*(pfk_R0_c(t) - pfk_T0_c(t)/Keq_PFK_L)
```

##### The Steady State¶

###### Solve steady state concentrations symbolically¶

To determine the steady state of the enzyme, a dictionary of the ordinary differential equations as symbolic expressions for each of the `EnzymeModuleForm` objects. The ligands are first removed from the equations by assuming their values are taken into account in a lumped rate constant parameter.

For handling of all symbolic expressions, the **SymPy** package is used.

```
[9]:
```

```
# Make a dictionary of ODEs and lump ligands into rate parameters by giving them a value of 1
ode_dict = {}
lump_ligands = {sym.Symbol(met.id): 1 for met in PFK.enzyme_module_ligands}
for enzyme_module_form in PFK.enzyme_module_forms:
    symbol_key = sym.Symbol(enzyme_module_form.id)
    ode = sym.Eq(strip_time(enzyme_module_form.ode), 0)
    ode_dict[symbol_key] = ode.subs(lump_ligands)

rank = matrix_rank(PFK.S[6:])
print("Rank Deficiency: {0}".format(len(ode_dict) - rank))
```

```
Rank Deficiency: 1
```

In order to solve the system of ODEs for the steady state concentrations, an additional equation is required due to the rank deficiency of the stoichiometric matrix. Therefore, the equation for the steady state flux through the enzyme, which will be referred to as the “enzyme net flux equation”, must be defined.

To define the enzyme net flux equation, the `EnzymeModule.make_enzyme_netflux_equation` class method can be used.

- This equation is made by providing a reaction, or a list of reactions to add together.
- Passing a bool to `use_rates` argument determines whether a symbolic equation is a summation of the flux symbols returned by `EnzymeModuleReaction.flux_symbol_str`, or a summation of the rates laws for those reactions.
- The `update_enzyme` argument determines whether the new rate equation is set in the `enzyme_rate_equation` attribute.

The flux through the enzyme typically corresponds to the sum of the fluxes through the catalytic reaction steps. Because the catalyzation reactions were assigned to the “catalyzation” cateogry, they can be accessed through the `enzyme_module_reactions_categorized` attribute to create the equation for \(v\_{\mathrm{PFK}}\).

```
[10]:
```

```
reactions = PFK.enzyme_module_reactions_categorized.get_by_id(
    "catalyzation").members
PFK.make_enzyme_rate_equation(
    reactions,
    use_rates=True, update_enzyme=True)
sym.pprint(PFK.enzyme_rate_equation)
```

```
kf_PFK⋅(pfk_R0_AF_c(t) + pfk_R1_AF_c(t) + pfk_R2_AF_c(t) + pfk_R3_AF_c(t) + pf
k_R4_AF_c(t))
```

The next step is to identify equations for the unknown concentrations in each reaction. These equations will need to be solved with a dependent variable before accounting for the enzyme net flux equation. The completely free form of the enzyme with no bound species will be treated as the dependent variable.

To verify that all equations are in terms of the lumped rate parameters, and the dependent variable, the solutions can be iterated through using the atoms method to identify the equation arguments. There should be no `EnzymeModuleForm` identifiers with the exception of the dependent variable.

```
[11]:
```

```
# Get enzyme module forms
enzyme_module_forms = PFK.enzyme_module_forms.copy()
# Reverse list for increased performance (due to symmetry assumption)
# by solving for the most activated/inhibitors bound first.
enzyme_module_forms.reverse()

enzyme_solutions = {}
for enzyme_module_form in enzyme_module_forms:
    # Skip dependent variable
    if "pfk_R0_c" == str(enzyme_module_form):
        continue
    enzyme_module_form = sym.Symbol(enzyme_module_form.id)
    # Susbtitute in previous solutions and solve for the enzyme module form,
    equation = ode_dict[enzyme_module_form]
    sol = sym.solveset(equation.subs(enzyme_solutions), enzyme_module_form)
    enzyme_solutions[enzyme_module_form] = list(sol)[0]
    # Update the dictionary of solutions with the solutions
    enzyme_solutions.update({
        enzyme_module_form: sol.subs(enzyme_solutions)
        for enzyme_module_form, sol in enzyme_solutions.items()})
args = set()
for sol in enzyme_solutions.values():
    args.update(sol.atoms(sym.Symbol))
print(args)
```

```
{Keq_PFK_L, kf_PFK, kf_PFK_A, Keq_PFK_A, kf_PFK_F, pfk_R0_c, Keq_PFK_I, Keq_PFK_ACT, Keq_PFK_F}
```

The enzyme net flux equation can then be utilized as the last equation required to solve for the final unknown concentration variable in terms of the rate and equilibrium constants, allowing for all of the concentration variables to be defined in terms of the rate and equilibrium constants. Once the unknown variable has been solved for, the solution can be substituted back into the other equations. Because `sympy.solveset` function expects the input equations to be equal to 0, the
`EnzymeModule.enzyme_rate_error` method with the `use_values` argument set to `False` to get the appropriate expression.

```
[12]:
```

```
enzyme_rate_equation = strip_time(PFK.enzyme_rate_error(False))
print("Enzyme Net Flux Equation\n" + "-"*24)
sym.pprint(enzyme_rate_equation)

# Solve for last unknown concentration symbolically
sol = sym.solveset(enzyme_rate_equation.subs(enzyme_solutions), "pfk_R0_c")

# Update solution dictionary with the new solution
enzyme_solutions[sym.Symbol("pfk_R0_c")] = list(sol)[0]

# Update solutions with free variable solutions
enzyme_solutions = {
    enzyme_module_form: sym.simplify(solution.subs(enzyme_solutions))
    for enzyme_module_form, solution in enzyme_solutions.items()}

args = set()
for sol in enzyme_solutions.values():
    args.update(sol.atoms(sym.Symbol))
print("\n", args)
```

```
Enzyme Net Flux Equation
------------------------
-kf_PFK⋅(pfk_R0_AF_c + pfk_R1_AF_c + pfk_R2_AF_c + pfk_R3_AF_c + pfk_R4_AF_c)
+ v_PFK

 {Keq_PFK_L, kf_PFK, kf_PFK_A, Keq_PFK_A, kf_PFK_F, Keq_PFK_I, v_PFK, Keq_PFK_ACT, Keq_PFK_F}
```

###### Numerical Values¶

At this point, numerical values are defined for the dissociation constants and the concentrations of the substrates, cofactors, activators, and inhibitors. Providing these numerical values will speed up the subsequent calculations.

To do this, experimental data is used to define the dissociations constants for the different binding steps under the QEA. The concentrations of the non-enzyme species are taken from the glycolysis model. Experimental data gives the following for the dissociation constants:

\[K\_i=0.1 mM,\
K\_a=0.033 mM,\
K\_A=0.068 mM,\
K\_F=0.1 mM\]

and an allosteric constant of \(K\_L = 0.0011\).

**Note:** The \(K\_i\) binding constant for ATP as an inhibitor was increased by a factor of ten since magnesium complexing of ATP is not considered here.

```
[13]:
```

```
numerical_values = {}

# Get ligand IDs and parameter IDs
ligand_ids = sorted([str(ligand) for ligand in PFK.enzyme_module_ligands])
parameter_ids = ["_".join((PFK.id, abbrev)) for abbrev in abbreviations + ["L"]]
print("Ligand IDs: " + str(ligand_ids))
print("Parameter IDs: " + str(parameter_ids))

# Load the glycolysis model to extract steady state values
glycolysis = create_test_model("SB2_Glycolysis")

# Get the steady state flux value and add to numerical values
PFK.enzyme_rate = glycolysis.reactions.get_by_id(PFK.id).steady_state_flux
numerical_values.update({PFK.enzyme_flux_symbol_str: PFK.enzyme_rate})

# Get the steady state concentration values and add to numerical values
initial_conditions = {
    str(ligand): glycolysis.initial_conditions[glycolysis.metabolites.get_by_id(ligand)]
    for ligand in ligand_ids}

# Define parameter values and add to numerical values
# Because of the QEA, invert dissociation constants for Keq
parameter_values = {
    "Keq_" + parameter_id: value
    for parameter_id, value in zip(parameter_ids, [1/0.068, 1/0.1, 1/0.1, 1/0.033, 0.0011])}

# Display numerical values
print("\nNumerical Values\n----------------")
for k, v in numerical_values.items():
    print("{0} = {1}".format(k, v))
```

```
Ligand IDs: ['adp_c', 'amp_c', 'atp_c', 'f6p_c', 'fdp_c', 'h_c']
Parameter IDs: ['PFK_A', 'PFK_F', 'PFK_I', 'PFK_ACT', 'PFK_L']

Numerical Values
----------------
v_PFK = 1.12
```

The next step is to define the numerical values, \(K\_i=0.1/1.6\), \(K\_a=0.033/0.0867\), \(K\_A=0.068/1.6\), \(K\_F=0.1/0.0198\), \(v\_{PFK}=1.12 \text{mM/hr}\), and \(K\_L=1/0.0011\) using the dissociation constant values and the steady state concentrations of the ligands and introduce them into the solution to get the steady state concentrations of the enzyme module forms in terms of the rate constants. The values of the equilirbium constants and initial conditions are also
stored for later use.

```
[14]:
```

```
# Match abbreviations to their corresponding ligands
abbreviation_dict = {"PFK_A": "atp_c", "PFK_F": "f6p_c", "PFK_ACT": "amp_c", "PFK_I": "atp_c", "PFK_L": ""}

k2K = {sym.Symbol("kr_" + p): sym.Symbol("kf_" + p)*sym.Symbol("K_" + p) for p in abbreviation_dict.keys()}
enzyme_solutions = {met: sym.simplify(Keq2k(solution).subs(enzyme_solutions).subs(k2K))
                    for met, solution in enzyme_solutions.items()}
K_values = dict(zip(["K_" + p for p in abbreviation_dict], [0.068, 0.1, 0.033, 0.1, 0.0011]))

for abbrev, ligand_id in abbreviation_dict.items():
    K_str = "K_" + abbrev
    if ligand_id:
        numerical_value = K_values[K_str]/initial_conditions[ligand_id]
    else:
        numerical_value = 1/K_values[K_str]
    numerical_values[sym.Symbol(K_str)] = numerical_value

enzyme_solutions = {met: sym.simplify(solution.subs(numerical_values))
                    for met, solution in enzyme_solutions.items()}

# Display numerical values
print("\nNumerical Values\n----------------")
for k, v in numerical_values.items():
    print("{0} = {1}".format(k, v))
```

```
Numerical Values
----------------
v_PFK = 1.12
K_PFK_A = 0.0425
K_PFK_F = 5.05050505050505
K_PFK_ACT = 0.3804995151513754
K_PFK_I = 0.0625
K_PFK_L = 909.090909090909
```

The last part of this step is to simplify the solutions for the enzyme module forms and, as a QA check, ensure that only rate constants are the only symbolic arguments in the solutions.

```
[15]:
```

```
# Substitute values into equations
enzyme_solutions = {
    enzyme_module_form: sym.simplify(solution.subs(numerical_values))
    for enzyme_module_form, solution in enzyme_solutions.items()}

args = set()
for sol in enzyme_solutions.values():
    args.update(sol.atoms(sym.Symbol))
print(args)
```

```
{kf_PFK, kf_PFK_A, kf_PFK_F}
```

###### Determine rate constants¶

###### Total Enzyme Concentration and \(r\_{T}\)¶

After solving for the enzyme module forms, the next step is to define equations for the total enzyme concentration and for the fraction of the enzyme in the T state. These two equations can be used as constraints for determining the rate parameters. To view the equation for the total enzyme concentration, we can use the `EnzymeModule.enzyme_concentration_total_equation` property.

```
[16]:
```

```
sym.pprint(PFK.enzyme_concentration_total_equation)
```

```
pfk_R0_AF_c(t) + pfk_R0_A_c(t) + pfk_R0_c(t) + pfk_R1_AF_c(t) + pfk_R1_A_c(t)
+ pfk_R1_c(t) + pfk_R2_AF_c(t) + pfk_R2_A_c(t) + pfk_R2_c(t) + pfk_R3_AF_c(t)
+ pfk_R3_A_c(t) + pfk_R3_c(t) + pfk_R4_AF_c(t) + pfk_R4_A_c(t) + pfk_R4_c(t) +
 pfk_T0_c(t) + pfk_T1_c(t) + pfk_T2_c(t) + pfk_T3_c(t) + pfk_T4_c(t)
```

The total concentration of PFK is 33 nM (=0.000033 mM). The `EnzymeModule.enzyme_concentration_total` atrribute can be used to set and store this concentration.

```
[17]:
```

```
PFK.enzyme_concentration_total = 33e-6
print(PFK.enzyme_concentration_total)
```

```
3.3e-05
```

To determine the rate constants, an optimization problem where the objective function is to minimize the error between the measured and calculated total enzyme concentrations. To create the objective function, the `EnzymeModule.enzyme_concentration_total_error` method with the `use_values` argument set as False to get the symbolic expression of the constraint.

```
[18]:
```

```
enzyme_total_constraint = abs(strip_time(PFK.enzyme_concentration_total_error(use_values=False)))
sym.pprint(enzyme_total_constraint)
```

```
│-PFK_Total + pfk_R0_AF_c + pfk_R0_A_c + pfk_R0_c + pfk_R1_AF_c + pfk_R1_A_c +
 pfk_R1_c + pfk_R2_AF_c + pfk_R2_A_c + pfk_R2_c + pfk_R3_AF_c + pfk_R3_A_c + p
fk_R3_c + pfk_R4_AF_c + pfk_R4_A_c + pfk_R4_c + pfk_T0_c + pfk_T1_c + pfk_T2_c
 + pfk_T3_c + pfk_T4_c│
```

Substitute the solutions for the enzyme forms to get an equation for the error in the enzyme total concentration in terms of the rate constants.

```
[19]:
```

```
# Substitute value for enzyme concentration total
enzyme_total_constraint = enzyme_total_constraint.subs({PFK.enzyme_total_symbol_str: PFK.enzyme_concentration_total})
# Substitute solutions into constraint and simplify
enzyme_total_constraint = sym.simplify(enzyme_total_constraint.subs(enzyme_solutions))
sym.pprint(enzyme_total_constraint)
```

```
│          1.19283868483391   1.71385140785683   7.14443780219149│
│-3.3e-5 + ──────────────── + ──────────────── + ────────────────│
│              kf_PFK_F           kf_PFK_A            kf_PFK     │
```

To create the objective function in a format suitable for the minimization method from the `scipy.optimize` submodule, the `sympy.lambdify` function can be used to convert the symbolic expression into a lambda function with the rate constants as the arguments. This lambda function can then be used to generate the objective function for the `optimize.minimize` method.

```
[20]:
```

```
# Create a sorted tuple of the arguments to ensure the input format does not change
args = tuple(sorted([str(arg) for arg in list(args)]))
# Create the objective function as a lambda function
objective_function = lambda x: sym.lambdify(args, enzyme_total_constraint)(*x)
```

Another constraint can be set on the amount of inhibited enzyme in the steady state of the system using the T fraction (denoted as \(r\_{T}\)). This fraction is simply the amount of inhibited enzyme over the total amount of enzyme. The enzyme is inhibited between 10-15% under physiological conditions (Ponce et al. Biochimica et Biophysica Acta 1971 250(1):63-74)

To make the fraction as a symbolic expression, we can use the `EnzymeModule.make_enzyme_fraction` method. This method is designed to assist in making fractions and ratios by passing to the function: 1. A string to the `categorized_attr` argument identifying which categorized attribute (either “forms” for the `EnzymeModule.enzyme_module_forms_categorized` or “reactions” for the `EnzymeModule.enzyme_module_reactions_categorized`). 2. A string for the `top` argument and a string for the
`bottom` argument identifying the categories to sum and use in the numerator and the denominator, respectively. 3. A bool to the `use_values` argument indicating whether to substitute numerical values into the expression to return a float or to keep the ratio as a **SymPy** expression.

**Note:** The string “Equation” can be passed to either the `top` or `bottom` arguments to utilize the equation stored either in `enzyme_concentration_total_equation` (for `categorized_attr`=”forms”), or `enzyme_rate_equation` (for `categorized_attr`=”reactions”).

```
[21]:
```

```
# Set the values for the constraint bounds
r_T_lb, r_T_ub = (0.10, 0.15)
# Make a symbolic expression for enzyme fraction.
r_T_expr = PFK.make_enzyme_fraction(
    categorized_attr="forms", top="Tense", bottom="Equation",
    use_values=False)
# Substitute solutions into the expression to make
# solely dependent on the rate constants
r_T_expr = sym.simplify(strip_time(r_T_expr).subs(enzyme_solutions))

# Make lambda functions for the T fraction constraint
r_T_lb_constraint = lambda x: sym.lambdify(args, r_T_expr - r_T_lb)(*x)
r_T_ub_constraint = lambda x: sym.lambdify(args, r_T_ub - r_T_expr)(*x)
```

Lastly, we place lower and upper bounds on the rate constants to ensure that the values are non-negative and are within physiological limits, and then we solve the optmization problem. Once the optimization has finished, we check whether it was successful, and if so, what the optimality and errors are associated with this particular solution instance.

```
[22]:
```

```
print("Ordered Args: {0}\n".format(str(args)))
# Set arguments for minimization
kf_bounds = ((1e2, 1e8), (1e2, 1e8), (1e2, 1e8))
initial_guess = [
    3.07e5,
    2e5,
    1e6,]

# Find a feasible solution
sol = optimize.minimize(
    objective_function, x0=initial_guess,
    method="trust-constr",
    bounds=kf_bounds,
    options={"gtol": 1e-20, "xtol": 1e-20, "maxiter": 1e4, "disp": True})

# Check whether optimzation was successful
print("\nOptimization Success: {0}".format(sol.success))
if sol.success:
    # Update the paramter values dictionary with the feasible solution
    parameter_values.update(dict(zip(args, [round(x) for x in sol.x])))
    print("Optimization Optimality: {0:.4e}".format(sol.optimality))
    print("Parameter Solutions: {:}".format(str({arg: parameter_values[arg] for arg in args})))
    # Plug solutions back into constraints for validation
    print("Optimization Error: {0:.4e}".format(enzyme_total_constraint.subs(parameter_values)))
```

```
Ordered Args: ('kf_PFK', 'kf_PFK_A', 'kf_PFK_F')

`xtol` termination condition is satisfied.
Number of iterations: 104, function evaluations: 224, CG iterations: 116, optimality: 3.60e-11, constraint violation: 0.00e+00, execution time: 0.63 s.

Optimization Success: True
Optimization Optimality: 3.6029e-11
Parameter Solutions: {'kf_PFK': 307263, 'kf_PFK_A': 200325, 'kf_PFK_F': 1000059}
Optimization Error: 1.2079e-11
```

With a successful optimization, the module is updated with the parameter values. The inhibition and activation reactions are set to have a high forward rate constant and the allosteric transition even higher, limiting the amount of unbound enzyme and ensuring that the dynamics are determined by the dissociation and allosteric constants.

**Note:** This assumption for the rate constants can be made because none of the enzyme concentrations are dependendent on the activation, inhibition, and allosteric rate constants.

```
[23]:
```

```
# Add the activation, inhibition, and allosteric rate constants
for abbrev, value in zip(["I", "ACT", "L"], [1e6, 1e6, 1e6**2]):
    # Account for the enzyme prefix if used in the previous function
    to_join = ("kf", PFK.id, abbrev)
    param = "_".join(to_join)
    parameter_values.update({param: value})

# Display numerical values
for k, v in parameter_values.items():
    print("{0} = {1}".format(k, v))
```

```
Keq_PFK_A = 14.705882352941176
Keq_PFK_F = 10.0
Keq_PFK_I = 10.0
Keq_PFK_ACT = 30.3030303030303
Keq_PFK_L = 0.0011
kf_PFK = 307263
kf_PFK_A = 200325
kf_PFK_F = 1000059
kf_PFK_I = 1000000.0
kf_PFK_ACT = 1000000.0
kf_PFK_L = 1000000000000.0
```

###### Solve steady state concentrations numerically¶

Once the rate constants have been defined, the steady state concentrations of the enzyme can be determined.

```
[24]:
```

```
# Substitute values into equations
initial_conditions.update({
    str(enzyme_module_form): float(sym.simplify(solution.subs(parameter_values)))
    for enzyme_module_form, solution in enzyme_solutions.items()})

for header, dictlist in zip(["Ligand", "\nEnzyme"], [PFK.enzyme_module_ligands, PFK.enzyme_module_forms]):
    header += " Concentrations"
    print("\n".join([header, "-" * len(header)]))
    for form in dictlist:
        ic = initial_conditions[form.id]
        print("{0} = {1}".format(form.id, ic))
```

```
Ligand Concentrations
---------------------
f6p_c = 0.0198
fdp_c = 0.0146
atp_c = 1.6
adp_c = 0.29
amp_c = 0.0867281
h_c = 8.99757e-05

Enzyme Concentrations
----------------------
pfk_R0_c = 3.705684451779081e-08
pfk_R0_A_c = 1.1270977736701491e-07
pfk_R0_AF_c = 2.1036774576199985e-08
pfk_T0_c = 4.0762528969569896e-11
pfk_R1_c = 3.895599656998077e-07
pfk_R1_A_c = 1.1848611930259641e-06
pfk_R1_AF_c = 2.2114902898450058e-07
pfk_T1_c = 2.6088018540524733e-09
pfk_R2_c = 1.5357179846004314e-06
pfk_R2_A_c = 4.670943637948869e-06
pfk_R2_AF_c = 8.71810686394121e-07
pfk_T2_c = 6.261124449725935e-08
pfk_R3_c = 2.690705109903528e-06
pfk_R3_A_c = 8.183880139927137e-06
pfk_R3_AF_c = 1.5274845331446054e-06
pfk_T3_c = 6.678532746374332e-07
pfk_R4_c = 1.7678768321380616e-06
pfk_R4_A_c = 5.377063448209202e-06
pfk_R4_AF_c = 1.0036047828713532e-06
pfk_T4_c = 2.6714130985497327e-06
```

###### Set Initial Conditions and Parameters¶

Once the steady state concentrations have been determined, the initial conditions and parameters are added to the module. All custom parameter are added to the custom\_parameter attribute. The allosteric transition uses the standard parameter identifiers (returned by `kf_str` and `Keq_str` properties of the `EnzymeModuleReaction`), so they are popped out of the custom parameters and set through their respective attribute setter methods.

```
[25]:
```

```
# Set initial conditions
for met, concentration in initial_conditions.items():
    PFK.metabolites.get_by_id(str(met)).ic = concentration

# Add the custom parameters and values for kf and Keq to model
PFK.custom_parameters.update(parameter_values)
# PFK_L uses standard reaction parameters and not custom parameters
PFK_L = PFK.enzyme_module_reactions.PFK_L
PFK_L.kf = PFK.custom_parameters.pop(PFK_L.kf_str)
PFK_L.Keq = PFK.custom_parameters.pop(PFK_L.Keq_str)

# Set parameter values in reaction fields
for group in PFK.enzyme_module_reactions_categorized:
    if group.id == "atp_c_binding":
        param_id = "PFK_A"
    elif group.id == "f6p_c_binding":
        param_id = "PFK_F"
    elif group.id == "catalyzation":
        param_id = "PFK"
    elif group.id == "atp_c_inhibition":
        param_id = "PFK_I"
    elif group.id == "amp_c_activation":
        param_id = "PFK_ACT"
    else:
        continue
    for reaction in group.members:
        kf, Keq = ("kf_" + param_id, "Keq_" + param_id)
        if kf in PFK.custom_parameters:
            reaction.kf = PFK.custom_parameters[kf]
        if Keq in PFK.custom_parameters:
            reaction.Keq = PFK.custom_parameters[Keq]
```

###### Ordering of internal species and reactions¶

Sometimes, it is also desirable to reorder the metabolite and reaction objects inside the model to follow the physiology. To reorder the internal objects, one can use `cobra.DictList` containers and the `DictList.get_by_any` method with the list of object identifiers in the desirable order. To ensure all objects are still present and not forgotten in the model, a small QA check is also performed.

```
[26]:
```

```
new_metabolite_order = ['f6p_c', 'fdp_c', 'amp_c', 'adp_c', 'atp_c', 'h_c',
                        'pfk_R0_c', 'pfk_R0_A_c', 'pfk_R0_AF_c',
                        'pfk_R1_c', 'pfk_R1_A_c', 'pfk_R1_AF_c',
                        'pfk_R2_c', 'pfk_R2_A_c', 'pfk_R2_AF_c',
                        'pfk_R3_c', 'pfk_R3_A_c', 'pfk_R3_AF_c',
                        'pfk_R4_c', 'pfk_R4_A_c', 'pfk_R4_AF_c',
                        'pfk_T0_c','pfk_T1_c', 'pfk_T2_c', 'pfk_T3_c', 'pfk_T4_c']

if len(glycolysis.metabolites) == len(new_metabolite_order):
    PFK.metabolites = DictList(
        PFK.metabolites.get_by_any(new_metabolite_order))

if len(PFK.metabolites) == len(new_metabolite_order):
    PFK.metabolites = DictList(PFK.metabolites.get_by_any(new_metabolite_order))

new_reaction_order = ["PFK_R01", 'PFK_R02', "PFK_R03", "PFK_R10",
                      "PFK_R11", "PFK_R12", "PFK_R13", "PFK_R20",
                      "PFK_R21", "PFK_R22", "PFK_R23", "PFK_R30",
                      "PFK_R31", "PFK_R32", "PFK_R33", "PFK_R40",
                      "PFK_R41", "PFK_R42", "PFK_R43", "PFK_L",
                      "PFK_T1", "PFK_T2", "PFK_T3", "PFK_T4"]

if len(PFK.reactions) == len(new_reaction_order):
    PFK.reactions = DictList(
        PFK.reactions.get_by_any(new_reaction_order))

PFK.update_S(array_type="DataFrame", dtype=int)
```

```
[26]:
```

|  | PFK\_R01 | PFK\_R02 | PFK\_R03 | PFK\_R10 | PFK\_R11 | PFK\_R12 | PFK\_R13 | PFK\_R20 | PFK\_R21 | PFK\_R22 | ... | PFK\_R33 | PFK\_R40 | PFK\_R41 | PFK\_R42 | PFK\_R43 | PFK\_L | PFK\_T1 | PFK\_T2 | PFK\_T3 | PFK\_T4 |
| --- | --- | --- | --- | --- | --- | --- | --- | --- | --- | --- | --- | --- | --- | --- | --- | --- | --- | --- | --- | --- | --- |
| f6p\_c | 0 | -1 | 0 | 0 | 0 | -1 | 0 | 0 | 0 | -1 | ... | 0 | 0 | 0 | -1 | 0 | 0 | 0 | 0 | 0 | 0 |
| fdp\_c | 0 | 0 | 1 | 0 | 0 | 0 | 1 | 0 | 0 | 0 | ... | 1 | 0 | 0 | 0 | 1 | 0 | 0 | 0 | 0 | 0 |
| amp\_c | 0 | 0 | 0 | -1 | 0 | 0 | 0 | -1 | 0 | 0 | ... | 0 | -1 | 0 | 0 | 0 | 0 | 0 | 0 | 0 | 0 |
| adp\_c | 0 | 0 | 1 | 0 | 0 | 0 | 1 | 0 | 0 | 0 | ... | 1 | 0 | 0 | 0 | 1 | 0 | 0 | 0 | 0 | 0 |
| atp\_c | -1 | 0 | 0 | 0 | -1 | 0 | 0 | 0 | -1 | 0 | ... | 0 | 0 | -1 | 0 | 0 | 0 | -1 | -1 | -1 | -1 |
| h\_c | 0 | 0 | 1 | 0 | 0 | 0 | 1 | 0 | 0 | 0 | ... | 1 | 0 | 0 | 0 | 1 | 0 | 0 | 0 | 0 | 0 |
| pfk\_R0\_c | -1 | 0 | 1 | -1 | 0 | 0 | 0 | 0 | 0 | 0 | ... | 0 | 0 | 0 | 0 | 0 | -1 | 0 | 0 | 0 | 0 |
| pfk\_R0\_A\_c | 1 | -1 | 0 | 0 | 0 | 0 | 0 | 0 | 0 | 0 | ... | 0 | 0 | 0 | 0 | 0 | 0 | 0 | 0 | 0 | 0 |
| pfk\_R0\_AF\_c | 0 | 1 | -1 | 0 | 0 | 0 | 0 | 0 | 0 | 0 | ... | 0 | 0 | 0 | 0 | 0 | 0 | 0 | 0 | 0 | 0 |
| pfk\_R1\_c | 0 | 0 | 0 | 1 | -1 | 0 | 1 | -1 | 0 | 0 | ... | 0 | 0 | 0 | 0 | 0 | 0 | 0 | 0 | 0 | 0 |
| pfk\_R1\_A\_c | 0 | 0 | 0 | 0 | 1 | -1 | 0 | 0 | 0 | 0 | ... | 0 | 0 | 0 | 0 | 0 | 0 | 0 | 0 | 0 | 0 |
| pfk\_R1\_AF\_c | 0 | 0 | 0 | 0 | 0 | 1 | -1 | 0 | 0 | 0 | ... | 0 | 0 | 0 | 0 | 0 | 0 | 0 | 0 | 0 | 0 |
| pfk\_R2\_c | 0 | 0 | 0 | 0 | 0 | 0 | 0 | 1 | -1 | 0 | ... | 0 | 0 | 0 | 0 | 0 | 0 | 0 | 0 | 0 | 0 |
| pfk\_R2\_A\_c | 0 | 0 | 0 | 0 | 0 | 0 | 0 | 0 | 1 | -1 | ... | 0 | 0 | 0 | 0 | 0 | 0 | 0 | 0 | 0 | 0 |
| pfk\_R2\_AF\_c | 0 | 0 | 0 | 0 | 0 | 0 | 0 | 0 | 0 | 1 | ... | 0 | 0 | 0 | 0 | 0 | 0 | 0 | 0 | 0 | 0 |
| pfk\_R3\_c | 0 | 0 | 0 | 0 | 0 | 0 | 0 | 0 | 0 | 0 | ... | 1 | -1 | 0 | 0 | 0 | 0 | 0 | 0 | 0 | 0 |
| pfk\_R3\_A\_c | 0 | 0 | 0 | 0 | 0 | 0 | 0 | 0 | 0 | 0 | ... | 0 | 0 | 0 | 0 | 0 | 0 | 0 | 0 | 0 | 0 |
| pfk\_R3\_AF\_c | 0 | 0 | 0 | 0 | 0 | 0 | 0 | 0 | 0 | 0 | ... | -1 | 0 | 0 | 0 | 0 | 0 | 0 | 0 | 0 | 0 |
| pfk\_R4\_c | 0 | 0 | 0 | 0 | 0 | 0 | 0 | 0 | 0 | 0 | ... | 0 | 1 | -1 | 0 | 1 | 0 | 0 | 0 | 0 | 0 |
| pfk\_R4\_A\_c | 0 | 0 | 0 | 0 | 0 | 0 | 0 | 0 | 0 | 0 | ... | 0 | 0 | 1 | -1 | 0 | 0 | 0 | 0 | 0 | 0 |
| pfk\_R4\_AF\_c | 0 | 0 | 0 | 0 | 0 | 0 | 0 | 0 | 0 | 0 | ... | 0 | 0 | 0 | 1 | -1 | 0 | 0 | 0 | 0 | 0 |
| pfk\_T0\_c | 0 | 0 | 0 | 0 | 0 | 0 | 0 | 0 | 0 | 0 | ... | 0 | 0 | 0 | 0 | 0 | 1 | -1 | 0 | 0 | 0 |
| pfk\_T1\_c | 0 | 0 | 0 | 0 | 0 | 0 | 0 | 0 | 0 | 0 | ... | 0 | 0 | 0 | 0 | 0 | 0 | 1 | -1 | 0 | 0 |
| pfk\_T2\_c | 0 | 0 | 0 | 0 | 0 | 0 | 0 | 0 | 0 | 0 | ... | 0 | 0 | 0 | 0 | 0 | 0 | 0 | 1 | -1 | 0 |
| pfk\_T3\_c | 0 | 0 | 0 | 0 | 0 | 0 | 0 | 0 | 0 | 0 | ... | 0 | 0 | 0 | 0 | 0 | 0 | 0 | 0 | 1 | -1 |
| pfk\_T4\_c | 0 | 0 | 0 | 0 | 0 | 0 | 0 | 0 | 0 | 0 | ... | 0 | 0 | 0 | 0 | 0 | 0 | 0 | 0 | 0 | 1 |

26 rows × 24 columns

##### Module Validation¶

###### QC/QA model¶

Before saving the module, it is important to ensure that the module is elementally balanced, and that the module can be integrated into a larger network for simulation. Therefore, the `qcqa_model` function from `mass.util.qcqa` is used to provide a report on the module quality and and indicate whether simulation is possible and if not, what parameters and/or initial conditions are missing.

```
[27]:
```

```
qcqa_model(PFK, parameters=True, concentrations=True,
           fluxes=False, superfluous=True, elemental=True)
```

```
╒══════════════════════════════════════════╕
│ MODEL ID: PFK                            │
│ SIMULATABLE: True                        │
│ PARAMETERS NUMERICALY CONSISTENT: True   │
╞══════════════════════════════════════════╡
╘══════════════════════════════════════════╛
```

###### Constraint Satisfaction and Error Values¶

Another QA check we perform is to substitute the steady state numerical values back into the constraints used in determining the rate constants in order to ensure that the constraints remain satisified, and that errors are small.

```
[28]:
```

```
t_fraction = PFK.make_enzyme_fraction("forms", top="Tense",
                                      bottom="Equation", use_values=True)
print("Enzyme T-fraction: {:.4f}".format(t_fraction))

print("Concentration Absolute Error: {0:.4e}".format(
    abs(PFK.enzyme_concentration_total_error(use_values=True))))
print("Flux Absolute Error: {0:.4e}".format(
    abs(PFK.enzyme_rate_error(use_values=True))))
```

```
Enzyme T-fraction: 0.1032
Concentration Absolute Error: 1.2079e-11
Flux Absolute Error: 2.2204e-16
```

###### Add Enzyme to MassModel¶

In order to determine whether the module can be successfully integrated into a model, another model can be loaded, merged with the module, and simulated. To validate this module, it will be merged with a glycolysis model.

To integrate the `EnzymeModule` into the `MassModel`, the reaction that the EnzymeModule will be replacing is first removed. The `MassModel.merge` method can then be utilized to add the `EnzymeModule` to the `MassModel`.

When merging an `EnzymeModule` and a `MassModel`, the `EnzymeModule` should always be merged into the `MassModel`.

```
[29]:
```

```
# Load and merge glycolysis with PFK model
glycolysis = create_test_model("SB2_Glycolysis.json")
# Remove the PFK MassReaction, then merge the EnzymeModule into the MassModel
glycolysis.remove_reactions([glycolysis.reactions.get_by_id("PFK")])
glycolysis_PFK = glycolysis.merge(PFK)
glycolysis_PFK
```

```
[29]:
```

|  |  |
| --- | --- |
| **Name** | Glycolysis |
| **Memory address** | 0x07f7fc34cec90 |
| **Stoichiometric Matrix** | 40x44 |
| **Matrix Rank** | 37 |
| **Number of metabolites** | 40 |
| **Initial conditions defined** | 40/40 |
| **Number of reactions** | 44 |
| **Number of genes** | 0 |
| **Number of enzyme modules** | 1 |
| **Number of groups** | 16 |
| **Objective expression** | 0 |
| **Compartments** | Cytosol |

Using `MassModel.merge` class method enables the `EnzymeModule` and `MassModel` to be merged like as if they were both `MassModel` objects. However, all attributes specific to the `EnzymeModule` (e.g the categorized attributes) are condensed into a speciailzed container called an `EnzymeModuleDict`.

The `EnzymeModuleDict` behaves like an ordered dictionary, but is unique in that its contents can be accessed as if they were attributes. These attributes can be viewed using `EnzymeModuleDict.keys` method. All `EnzymeModuleDicts` associated with a `MassModel` can be accessed via `MassModel.enzyme_modules` attribute.

```
[30]:
```

```
print(str(glycolysis_PFK.enzyme_modules) + "\n")
print("Attribute Accessors:\n-------------------\n" + "\n".join(list(
    glycolysis_PFK.enzyme_modules.PFK.keys())) + "\n")
glycolysis_PFK.enzyme_modules.PFK
```

```
[<EnzymeModuleDict PFK at 0x7f7fc2f88680>]

Attribute Accessors:
-------------------
id
name
subsystem
enzyme_module_ligands
enzyme_module_forms
enzyme_module_reactions
enzyme_module_ligands_categorized
enzyme_module_forms_categorized
enzyme_module_reactions_categorized
enzyme_concentration_total
enzyme_rate
enzyme_concentration_total_equation
enzyme_rate_equation
S
model
```

```
[30]:
```

|  |  |
| --- | --- |
| **Name** | PFK |
| **Memory address** | 0x07f7fc2f88680 |
| **Stoichiometric Matrix** | 26x24 |
| **Matrix Rank** | 20 |
| **Subsystem** | Glycolysis |
| **Number of Ligands** | 6 |
| **Number of EnzymeForms** | 20 |
| **Number of EnzymeModuleReactions** | 24 |
| **Enzyme Concentration Total** | 3.3e-05 |
| **Enzyme Net Flux** | 1.12 |

###### Validate Steady State¶

To find the steady state of the model and perform simulations, the model must first be loaded into a `Simulation`. In order to load a model into a `Simulation`, the model must be simulatable, meaning there are no missing numerical values that would prevent the integration of the ODEs that comprise the model. The `verbose` argument can be used while loading a model to produce a message indicating the successful loading of a model, or why a model could not load.

Once loaded into a `Simulation`, the `find_steady_state` method can be used with the `update_values` argument in order to update the initial conditions and fluxes of the model to a steady state. The model can then be simulated using the `simulate` method by passing the model to simulate, and a tuple containing the start time and the end time. The number of time points can also be included, but is optional.

After a successful simulation, two `MassSolution` objects are returned. The first `MassSolution` contains the concentration results of the simulation, and the second contains the flux results of the simulation.

To visually validate the steady state of the model, concentration and flux solutions can be plotted using the `plot_time_profile` function from `mass.visualization`. Alternatively, the `MassSolution.view_time_profile` property can be used to quickly generate a time profile for the results.

```
[31]:
```

```
# Setup simulation object, ensure model is at steady state
sim = Simulation(glycolysis_PFK, verbose=True)
sim.find_steady_state(glycolysis_PFK, strategy="simulate",
                      update_values=True, verbose=True,
                      tfinal=1e4, steps=1e6)

# Simulate from 0 to 1000 with 10001 points in the output
conc_sol, flux_sol = sim.simulate(
    glycolysis_PFK,time=(0, 1e3, 1e4 + 1))
# Quickly render and display time profiles
conc_sol.view_time_profile()
```

```
Successfully loaded MassModel 'Glycolysis' into RoadRunner.
Setting output selections
Setting simulation values for 'Glycolysis'
Setting output selections
Getting time points
Simulating 'Glycolysis'
Found steady state for 'Glycolysis'.
Updating 'Glycolysis' values
Adding 'Glycolysis' simulation solutions to output
```

###### Storing information and references¶

###### Compartment¶

Because the character “c” represents the cytosol compartment, it is recommended to define and set the compartment in the `EnzymeModule.compartments` attribute.

```
[32]:
```

```
PFK.compartments = {"c": "Cytosol"}
print(PFK.compartments)
```

```
{'c': 'Cytosol'}
```

###### Units¶

All of the units for the numerical values used in this model are “Millimoles” for amount and “Liters” for volume (giving a concentration unit of ‘Millimolar’), and “Hours” for time. In order to ensure that future users understand the numerical values for model, it is important to define the `MassModel.units` attribute.

The `MassModel.units` is a `cobra.DictList` that contains only `UnitDefinition` objects from the `mass.core.unit` submodule. Each `UnitDefinition` is created from `Unit` objects representing the base units that comprise the `UnitDefinition`. These `Units` are stored in the `list_of_units` attribute. Pre-built units can be viewed using the `print_defined_unit_values` function from the `mass.core.unit` submodule. Alternatively, custom units can also be created using the
`UnitDefinition.create_unit` method. For more information about units, please see the module docstring for `mass.core.unit` submodule.

**Note:** It is important to note that this attribute will NOT track units, but instead acts as a reference for the user and others so that they can perform necessary unit conversions.

```
[33]:
```

```
# Using pre-build units to define UnitDefinitions
concentration = UnitDefinition("mM", name="Millimolar",
                               list_of_units=["millimole", "per_litre"])
time = UnitDefinition("hr", name="hour", list_of_units=["hour"])

# Add units to model
PFK.add_units([concentration, time])
print(PFK.units)
```

```
[<UnitDefinition Millimolar "mM" at 0x7f7fbf2a9110>, <UnitDefinition hour "hr" at 0x7f7fbf2a9050>]
```

##### Export¶

After validation, the model is ready to be saved. The model can either be exported as a “.json” file or as an “.sbml” (“.xml”) file using their repsective submodules in `mass.io`.

To export the model, only the path to the directory and the model object itself need to be specified.

###### Export using SBML¶

```
[34]:
```

```
sbml.write_sbml_model(mass_model=PFK, filename="SB2_" + PFK.id + ".xml")
```

###### Export using JSON¶

```
[35]:
```

```
json.save_json_model(mass_model=PFK, filename="SB2_" + PFK.id + ".json")
```

## API¶

Not sure how to use a specific method or function? Try searching the API Reference!

### API Reference¶

This page contains auto-generated API reference documentation 1.

#### `mass`¶

##### Subpackages¶

###### `mass.core`¶

###### Submodules¶

###### `mass.core.mass_configuration`¶

Define the global configuration values through the `MassConfiguration`.

Involved in model construction:
:   - `boundary_compartment()`
    - `default_compartment()`
    - `irreversible_Keq()`
    - `irreversible_kr()`
    - `exclude_metabolites_from_rates()`
    - `exclude_compartment_volumes_in_rates()`
    - `model_creator()`
    - `decimal_precision()`

Involved in model simulation:
:   - `decimal_precision()`
    - `steady_state_threshold()`

Involved in flux balance analysis (FBA):
:   - `solver()`
    - `tolerance()`
    - `processes()`
    - `lower_bound()`
    - `upper_bound()`
    - `bounds()`

Involved in thermodynamics:
:   - `decimal_precision()`
    - `solver()`
    - `tolerance()`
    - `processes()`

Notes

- Some attributes are used in multiple `mass` submodules, such as
  `decimal_precision`.
- The `MassConfiguration` shares the configuration attributes of the
  `Configuration` class from `cobra`.

###### Module Contents¶

###### Classes¶

|  |  |
| --- | --- |
| `MassBaseConfiguration` | Define global configuration values honored by `mass` functions. |
| `MassConfiguration` | Define the configuration to be `Singleton` based. |

*class* `mass.core.mass_configuration.``MassBaseConfiguration`[source]¶
:   Define global configuration values honored by `mass` functions.

    Notes

    The `MassConfiguration` should be always be used over the
    `MassBaseConfiguration` in order for global configuration to work
    as intended.

    *property* `boundary_compartment`(*self*)[source]¶
    :   Get or set the default value for the boundary compartment.

        Parameters
        :   **compartment\_dict** (*dict*) – A `dict` containing the identifier of the boundary compartment
            mapped to the name of the boundary compartment.

    *property* `default_compartment`(*self*)[source]¶
    :   Get or set the default value for the default compartment.

        Parameters
        :   **compartment\_dict** (*dict*) – A `dict` containing the identifier of the default compartment
            mapped to the name of the default compartment.

    *property* `irreversible_Keq`(*self*)[source]¶
    :   Get or set the default `Keq` value of an irreversible reaction.

        Notes

        `MassReaction.equilibrium_constant`s cannot be negative.

        Parameters
        :   **value** (*float*) – A non-negative number for the equilibrium constant (Keq)
            of the reaction.

        Raises
        :   **ValueError** – Occurs when trying to set a negative value.

    *property* `irreversible_kr`(*self*)[source]¶
    :   Get or set the default `kr` value of an irreversible reaction.

        Notes

        `MassReaction.reverse_rate_constant`s cannot be negative.

        Parameters
        :   **value** (*float*) – A non-negative number for the reverse rate constant (kr)
            of the reaction.

        Raises
        :   **ValueError** – Occurs when trying to set a negative value.

    *property* `model_creator`(*self*)[source]¶
    :   Get or set values for the `dict` representing the model creator.

        Notes

        - A read-only copy of the `dict` is returned.
        - To successfully export a model creator, all keys must have non-empty
          string values.

        Parameters
        :   **creator\_dict** (*dict*) –

            A `dict` containing the model creator information where keys
            are SBML creator fields and values are strings. Keys can only
            be the following:

            - ’familyName’
            - ’givenName’
            - ’organization’
            - ’email’

            Values must be strings or `None`.

    *property* `exclude_metabolites_from_rates`(*self*)[source]¶
    :   Get or set the metabolites that should be excluded from rates.

        Default is `dict("elements", [{"H": 2, "O": 1}, {"H": 1}])` to
        remove the hydrogen and water metabolites using the
        `elements` attribute to filter out the hydrogen
        and water in all rates except the hydrogen and water exchange
        reactions.

        Parameters
        :   **to\_exclude** (*dict*) – A dict where keys should correspond to a metabolite attribute to
            utilize for filtering, and values are lists that contain the items
            to exclude that would be returned by the metabolite attribute.
            Does not apply to boundary reactions.

    *property* `exclude_compartment_volumes_in_rates`(*self*)[source]¶
    :   Get or set whether to exclude the compartment volumes in rates.

        The boundary compartment will always excluded.

        Parameters
        :   **value** (*bool*) – Whether to exclude the compartment volumes in rate expressions.

    *property* `decimal_precision`(*self*)[source]¶
    :   Get or set the default decimal precision when rounding.

        Positive numbers indicated digits to the right of the
        decimal place, negative numbers indicate digits to the left of the
        decimal place.

        Notes

        The `decimal_precison` is applied as follows:

        ```
        new_value = round(value, decimal_precison)
        ```

        Parameters
        :   **precision** (*int* *or* *None*) – An integer indicating how many digits from the decimal should
            rounding occur. If `None`, no rounding will occur.

    *property* `steady_state_threshold`(*self*)[source]¶
    :   Get or set the steady state threshold when using roadrunner solvers.

        A threshold for determining whether the RoadRunner steady state solver
        is at steady state. The steady state solver returns a value indicating
        how close the solution is to the steady state, where smaller values
        are better. Values less than the threshold indicate steady state.

        Notes

        - With simulations. the absolute difference between the last two points
          must be less than the steady state threshold.
        - With steady state solvers, the sum of squares of the steady state
          solution must be less than the steady state threshold.
        - Steady state threshold values cannot be negative.

        Parameters
        :   **threshold** (*float*) – The threshold for determining whether a steady state occurred.

        Raises
        :   **ValueError** – Occurs when trying to set a negative value.

    *property* `solver`(*self*)[source]¶
    :   Get or set the solver utilized for optimization.

        The solver choices are the ones
        provided by optlang and solvers installed in the environment.

        Parameters
        :   **solver** (*str*) –

            The solver to utilize in optimizations. Valid solvers typically
            include:

            > - `"glpk"`
            > - `"cplex"`
            > - `"gurobi"`

        Raises
        :   **cobra.exceptions.SolverNotFound** – Occurs for invalid solver values.

    *property* `tolerance`(*self*)[source]¶
    :   Get or set the tolerance value utilized by the optimization solver.

        Parameters
        :   **tol** (*float*) – The tolerance value to set.

    *property* `lower_bound`(*self*)[source]¶
    :   Get or set the default value of the lower bound for reactions.

        Parameters
        :   **bound** (*float*) – The default bound value to set.

    *property* `upper_bound`(*self*)[source]¶
    :   Get or set the default value of the lower bound for reactions.

        Parameters
        :   **bound** (*float*) – The default bound value to set.

    *property* `bounds`(*self*)[source]¶
    :   Get or set the default lower and upper bounds for reactions.

        Parameters
        :   **bounds** (*tuple of floats*) – A tuple of floats to set as the new default bounds in the form
            of `(lower_bound, upper_bound)`.

        Raises
        :   **AssertionError** – Occurs when lower bound is greater than the upper bound.

    *property* `processes`(*self*)[source]¶
    :   Return the default number of processes to use when possible.

        Number of processes to use where multiprocessing is
        possible. The default number corresponds to the number of available
        cores (hyperthreads).

    *property* `shared_state`(*self*)[source]¶
    :   Return a read-only `dict` for shared configuration attributes.

    `_repr_html_`(*self*)[source]¶
    :   Return the HTML representation of the MassConfiguration.

        Warning

        This method is intended for internal use only.

    `__repr__`(*self*)[source]¶
    :   Override default `repr()` for the MassConfiguration.

        Warning

        This method is intended for internal use only.

*class* `mass.core.mass_configuration.``MassConfiguration`[source]¶
:   Bases: `mass.core.mass_configuration.MassBaseConfiguration`

    Define the configuration to be `Singleton` based.

###### `mass.core.mass_metabolite`¶

MassMetabolite is a class for holding information regarding metabolites.

The `MassMetabolite` class inherits and extends the
`Metabolite` class in `cobra`. It contains
additional information required for simulations and other `mass`
functions and workflows.

Some key differences between the
`cobra.Metabolite` and the
`mass.MassMetabolite` are
listed below:

> - Unlike the `cobra.Metabolite`
>   the `id` initialization argument has been replaced with
>   `id_or_specie` to be clear in that a new `MassMetabolite` can
>   be instantiated with the same properties of a previously created
>   metabolite by passing the metabolite object to this argument.
> - The `formula`s can have moieties by placing them within square
>   brackets (e.g. `"[ENZYME]"`)

###### Module Contents¶

###### Classes¶

|  |  |
| --- | --- |
| `MassMetabolite` | Class for holding information regarding a metabolite. |

*class* `mass.core.mass_metabolite.``MassMetabolite`(*id\_or\_specie=None*, *name=''*, *formula=None*, *charge=None*, *compartment=None*, *fixed=False*)[source]¶
:   Bases: `cobra.core.metabolite.Metabolite`

    Class for holding information regarding a metabolite.

    Parameters
    :   - **id\_or\_specie** (*str**,* *Metabolite**,* *MassMetabolite*) – A string identifier to associate with the metabolite, or an existing
          metabolite object. If an existing metabolite object is
          provided, a new `MassMetabolite` is instantiated with the same
          properties as the original metabolite.
        - **name** (*str*) – A human readable name for the metabolite.
        - **formula** (*str*) – Chemical formula associated with the metabolite (e.g. H2O).
        - **charge** (*float*) – The charge number associated with the metabolite.
        - **compartment** (*str*) – Compartment of the metabolite.
        - **fixed** (*bool*) – Whether the metabolite concentration should remain at a fixed value.
          Default is `False`.

    *property* `elements`(*self*)[source]¶
    :   Get or set `dict` of elements in the `formula`.

        Parameters
        :   **elements\_dict** (*dict*) – A `dict` representing the elements of the chemical formula where
            keys are elements and values are the amount.

        Notes

        - Enzyme and macromolecule moieties can be recognized by enclosing them
          in brackets (e.g. [ENZYME]) when defining the chemical formula.
          They are treated as one entity and therefore are only counted once.
        - Overrides
          `elements()` of the
          `cobra.Metabolite`
          to allow for the use of moieties.

    *property* `initial_condition`(*self*)[source]¶
    :   Get or set the initial condition of the metabolite.

        When using a HistoryManager context, this attribute can be set
        temporarily, reversed when the exiting the context.

        Notes

        Initial conditions of metabolites cannot be negative.

        Parameters
        :   **value** (*float*) – A non-negative number for the concentration of the metabolite.

        Raises
        :   **ValueError** – Occurs when trying to set a negative value.

    *property* `fixed`(*self*)[source]¶
    :   Get or set whether the metabolite remains constant.

        When using a HistoryManager context, this attribute can be set
        temporarily, reversed when the exiting the context.

        Parameters
        :   **value** (*bool*) – Whether the metabolite should remain constant, meaning that the
            metabolite ODE is 0.

    *property* `ordinary_differential_equation`(*self*)[source]¶
    :   Return a `sympy` expression of the metabolite’s associated ODE.

        Will return `None` if metabolite is not associated with a
        `MassReaction`, or `0`. if the `fixed` attribute
        is set as `True`.

    *property* `formula_weight`(*self*)[source]¶
    :   Calculate and return the formula weight of the metabolite.

        Does not consider any moieties enclosed in brackets.

        Notes

        Overrides `formula_weight()`
        of the `cobra.Metabolite`
        to allow for the use of moieties.

    *property* `model`(*self*)[source]¶
    :   Return the `MassModel` associated with the metabolite.

    *property* `ic`(*self*)[source]¶
    :   Alias for the `initial_condition`.

    *property* `ode`(*self*)[source]¶
    :   Alias for the `ordinary_differential_equation`.

    `_remove_compartment_from_id_str`(*self*)[source]¶
    :   Remove the compartment from the ID str of the metabolite.

        Warning

        This method is intended for internal use only.

    `_repr_html_`(*self*)[source]¶
    :   HTML representation of the overview for the MassMetabolite.

        Warning

        This method is intended for internal use only.

    `__dir__`(*self*)[source]¶
    :   Override default dir() implementation to list only public items.

        Warning

        This method is intended for internal use only.

###### `mass.core.mass_model`¶

MassModel is a class for holding information regarding a `mass` model.

The `MassModel` class inherits and extends the
`Model` class in `cobra`. It contains additional
information required for simulations and other `mass` functions and
workflows.

Some key differences between the
`cobra.Model` and the
`mass.MassModel` are
listed below:

> - When instantiating a `MassModel` from a
>   `cobra.Model`, any associated
>   `cobra.Metabolite` will also
>   be converted into a `MassMetabolite`, and any associated
>   `cobra.Reaction` will also
>   be converted into a `MassReaction`.
>
>   Additionally, any groups associated with the model containing
>   `cobra.Metabolites` or
>   `cobra.Reactions` will be updated
>   with the correspondining `mass` objects.
> - No `cobra` object can be directly added to the `MassModel`
>   with the exception of `Gene` and
>   `Group` objects. Any `cobra` object will
>   first be instantiated into its equivalent `mass` object before
>   beimg added to the `MassModel`.

###### Module Contents¶

###### Classes¶

|  |  |
| --- | --- |
| `MassModel` | Class representation of a model. |

`mass.core.mass_model.``LOGGER`[source]¶
:   Logger for `mass_model` submodule.

    Type
    :   logging.Logger

`mass.core.mass_model.``CHOPNSQ` *= ['C', 'H', 'O', 'P', 'N', 'S', 'q']*[source]¶
:   Contains the six most abundant elements and charge for molecules.

    Type
    :   list

*class* `mass.core.mass_model.``MassModel`(*id\_or\_model=None*, *name=None*, *array\_type='dense'*, *dtype=np.float64*)[source]¶
:   Bases: `cobra.core.model.Model`

    Class representation of a model.

    Parameters
    :   - **id\_or\_model** (*str**,* *Model**,* *MassModel*) – A string identifier to associate with the model, or an existing
          `MassModel`. If an existing `MassModel` is provided,
          a new `MassModel` object is instantiated with the same
          properties as the original model.
        - **name** (*str*) – A human readable name for the model.
        - **array\_type** (*str*) – A string identifiying the desired format for the returned matrix.
          Valid matrix types include `'dense'`, `'dok'`, `'lil'`,
          `'DataFrame'`, and `'symbolic'` Default is `'DataFrame'`.
          See the `matrix` module documentation for more information
          on the `array_type`.
        - **dtype** (*data-type*) – The desired array data-type for the stoichiometric matrix. If `None`
          then the data-type will default to `numpy.float64`.

    `reactions`¶
    :   A `DictList` where the keys are reaction
        identifiers and the values are the associated
        `MassReaction`s.

        Type
        :   DictList

    `metabolites`¶
    :   A `DictList` where the keys are metabolite
        identifiers and the values are the associated
        `MassMetabolite`s.

        Type
        :   DictList

    `genes`¶
    :   A `DictList` where the keys are gene
        identifiers and the values are the associated
        `Gene`s.

        Type
        :   DictList

    `groups`¶
    :   A `DictList` where the keys are group
        identifiers and the values are the associated
        `Group`s.

        Type
        :   DictList

    `enzyme_modules`¶
    :   A `DictList` where the keys are enzyme
        module identifiers and the values are the associated
        `EnzymeModuleDict`s.

        Type
        :   DictList

    `custom_rates`¶
    :   A `dict` to store custom rate expressions for specific reactions,
        where the keys are `MassReaction`s and values are the
        custom rate expressions given as `sympy` expressions. Custom rate
        expressions will always be prioritized over automatically generated
        mass action rates.

        Type
        :   dict

    `custom_parameters`¶
    :   A `dict` to store the custom parameters for the custom rates,
        where key:value pairs are the string identifiers for the parameters
        and their corresponding numerical value.

        Type
        :   dict

    `boundary_conditions`¶
    :   A `dict` to store boundary conditions, where keys are string
        identifiers for ‘boundary metabolites’ of boundary reactions, and
        values are the corresponding boundary condition numerical value or
        function of time. Note that boundary conditions are treated as
        parameters and NOT as species.

        Type
        :   dict

    `units`¶
    :   `DictList` of `UnitDefinition`s
        to store in the model for referencing.

        Type
        :   DictList

    Warning

    - Note that the `MassModel` does NOT track units, and it is
      therefore incumbent upon the user to maintain unit consistency the model.
    - Note that boundary conditions are considered parameters and NOT as
      species in a reaction.

    *property* `stoichiometric_matrix`(*self*)[source]¶
    :   Return the stoichiometric matrix.

    *property* `S`(*self*)[source]¶
    :   Alias for the `stoichiometric_matrix`.

    *property* `ordinary_differential_equations`(*self*)[source]¶
    :   Return a `dict` of ODEs for the metabolites.

    *property* `odes`(*self*)[source]¶
    :   Alias for the `ordinary_differential_equations`.

    *property* `initial_conditions`(*self*)[source]¶
    :   Get `dict` of `MassMetabolite.initial_condition`s.

    *property* `ics`(*self*)[source]¶
    :   Alias for the `initial_conditions`.

    *property* `fixed`(*self*)[source]¶
    :   Return a `dict` of all metabolite fixed conditions.

    *property* `rates`(*self*)[source]¶
    :   Return a `dict` of reaction rate expressions.

        If a reaction has an associated custom rate expression, the custom rate
        will be prioritized and returned in the `dict` instead of the
        automatically generated rate law expression.

    *property* `steady_state_fluxes`(*self*)[source]¶
    :   Return a `dict` of all reaction steady state fluxes.

    *property* `v`(*self*)[source]¶
    :   Alias for the `steady_state_fluxes`.

    *property* `boundary`(*self*)[source]¶
    :   Return a `list` of boundary reactions in the model.

    *property* `boundary_metabolites`(*self*)[source]¶
    :   Return a sorted `list` of all ‘boundary metabolites’ in the model.

        See also

        `MassReaction.boundary_metabolite`

    *property* `exchanges`(*self*)[source]¶
    :   Return exchange reactions in the model.

        Exchange reactions are reactions that exchange mass with the exterior.
        Uses annotations and heuristics to exclude non-exchanges such as sink
        and demand reactions.

    *property* `demands`(*self*)[source]¶
    :   Return demand reactions in the model.

        Demands are irreversible reactions that accumulate or consume a
        metabolite in the inside of the model.

    *property* `sinks`(*self*)[source]¶
    :   Return sink reactions in the model.

        Sinks are reversible reactions that accumulate or consume a metabolite
        in the inside of the model.

    *property* `irreversible_reactions`(*self*)[source]¶
    :   Return a `list` of all irreversible reactions in the model.

    *property* `parameters`(*self*)[source]¶
    :   Return all parameters associateed with the model.

    *property* `compartments`(*self*)[source]¶
    :   Get or set a `dict` of all metabolite compartments.

        Assigning a `dict` to this property updates the model’s
        `dict` of compartment descriptions with the new values.

        Notes

        - Setter extends `compartments()` of
          the `cobra.Model` to enable
          resetting the attribute by setting an empty `dict`

        Parameters
        :   **compartment\_dict** (*dict*) – A `dict` mapping compartments abbreviations to full names.
            An empty `dict` will reset the compartments.

    *property* `conc_solver`(*self*)[source]¶
    :   Return the `ConcSolver` associated with the model.

    `update_S`(*self*, *array\_type=None*, *dtype=None*, *update\_model=True*)[source]¶
    :   Update the stoichiometric matrix of the model.

        Parameters
        :   - **array\_type** (*str*) – A string identifiying the desired format for the returned matrix.
              Valid matrix types include `'dense'`, `'dok'`, `'lil'`,
              `'DataFrame'`, and `'symbolic'`
              Default is the current `array_type`. See the `matrix`
              module documentation for more information on the `array_type`.
            - **dtype** (*data-type*) – The desired array data-type for the stoichiometric matrix.
              If `None` then the data-type will default to the
              current `dtype`.
            - **update\_model** (*bool*) – If `True`, will update the stored stoichiometric matrix,
              the matrix type, and the data-type for the model.

        Returns
        :   The stoichiometric matrix for the `MassModel` returned
            as the given `array_type` and with a data-type of `dtype`.

        Return type
        :   matrix of type `array_type`

    `add_metabolites`(*self*, *metabolite\_list*)[source]¶
    :   Add a `list` of metabolites to the model.

        The change is reverted upon exit when using the `MassModel`
        as a context.

        Parameters
        :   **metabolite\_list** (*list*) – A `list` containing `MassMetabolite`s to add to
            the model.

    `remove_metabolites`(*self*, *metabolite\_list*, *destructive=False*)[source]¶
    :   Remove a `list` of metabolites from the model.

        The change is reverted upon exit when using the `MassModel`
        as a context.

        Parameters
        :   - **metabolite\_list** (*list*) –

              A list containing `MassMetabolite`s to remove
              :   from the model.
            - **destructive** (*bool*) – If `False`, the metabolites are removed from all associated
              reactions. If `True`, also remove associated
              `MassReaction`s from the model.

    `add_boundary_conditions`(*self*, *boundary\_conditions*)[source]¶
    :   Add boundary conditions values for the given boundary metabolites.

        Boundary condition values can be a numerical value, or they can be a
        string or `sympy` expression representing a function of time.
        The function must only depend on time.

        Parameters
        :   **boundary\_conditions** (*dict*) – A `dict` of boundary conditions containing the
            ‘boundary metabolites’ and their corresponding value. The string
            representing the ‘boundary\_metabolite’ must exist in the `list`
            returned by `MassModel.boundary_metabolites`.

        See also

        `boundary_metabolites`

    `remove_boundary_conditions`(*self*, *boundary\_metabolite\_list*)[source]¶
    :   Remove the boundary condition for a list of boundary metabolites.

        Parameters
        :   **metabolite\_list** (*list*) – A `list` of metabolites to remove the boundary conditions for.
            Boundary metabolites must already exist in the model in order
            for them to be removed.

        See also

        `boundary_metabolites`

    `add_reactions`(*self*, *reaction\_list*)[source]¶
    :   Add reactions to the model.

        The change is reverted upon exit when using the `MassModel`
        as a context.

        Notes

        - `MassReaction`s with identifiers identical to an
          existing reaction are ignored.
        - Extends `cobra.core.model.Model.add_reactions()` by
          first ensuring that the reactions to be added are
          `MassReactions`s and not
          `cobra.Reactions`.
          and error message raised reflects the `mass` object.
        - If a `cobra.Reaction` is
          provided. a warning is raised and a `MassReaction`
          will be instantiated using the
          `cobra.Reaction`.

        Parameters
        :   **reaction\_list** (*list*) – A `list` of `MassReaction`s to add to the model.

    `remove_reactions`(*self*, *reactions*, *remove\_orphans=False*)[source]¶
    :   Remove reactions from the model.

        The change is reverted upon exit when using the `MassModel`
        as a context.

        Notes

        Extends `cobra.core.model.Model.remove_reactions()` by also
        removing any custom rates along with the reaction (and custom
        parameters if `remove_orphans=True`). Also removes
        the boundary condition if the reaction is a boundary reaction with
        a defined boundary condition.

        Parameters
        :   - **reaction\_list** (*list*) – A `list` of `MassReaction`s to be removed
              from the model.
            - **remove\_orphans** (*bool*) – If `True`, will also remove orphaned genes and metabolites from
              the model. If a custom rate is removed, the orphaned custom
              parameters will also be removed.

    `add_boundary`(*self*, *metabolite*, *boundary\_type='exchange'*, *reaction\_id=None*, *boundary\_condition=0.0*, *\*\*kwargs*)[source]¶
    :   Add a boundary reaction for a given metabolite.

        Accepted `kwargs` are passed to the underlying function for boundary
        reaction creation,
        `cobra.Model.add_boundary`,
        and initialization of the `MassReaction`.

        There are three different types of pre-defined boundary reactions:
        exchange, demand, and sink reactions.

        > - An exchange reaction is a reversible, unbalanced reaction that
        >   adds to or removes an extracellular metabolite from the
        >   extracellular compartment.
        > - A demand reaction is an irreversible reaction that consumes an
        >   intracellular metabolite.
        > - A sink is similar to an exchange but specifically for
        >   intracellular metabolites.

        Notes

        - Extends `cobra.core.model.Model.add_boundary()` by allowing
          for metabolite identifers of existing metabolites in the model,
          boundary conditions and reaction subsystem to be set, and
          utilizes default bounds from the `MassConfiguration`. for
          creation of custom boundary reaction types.
        - To set the reaction `boundary_type` to something else, the desired
          identifier of the created reaction must be specified. The name will
          be given by the metabolite name and the given `boundary_type`, and
          the reaction will be set its reversible attribute to `True`.

          Bounds will be set to the defaults specified in the
          `MassConfiguration`.

        Parameters
        :   - **metabolite** (*MassMetabolite* *or* *str*) – Any `MassMetabolite`, or an identifier of a metabolite
              that already exists in the model. The metabolite compartment is
              not checked but it is encouraged to stick to the definition of
              exchanges, demands, and sinks.
            - **boundary\_type** (*str*) – One of the pre-defined boundary types, or a user-defined type.
              Pre-defined boundary types include `"exchange"`, `"demand"`,
              and `"sink"`. Using one of the pre-defined reaction types is
              easiest. To create a user-defined kind of boundary reaction
              choose any other string, e.g., ‘my-boundary’.
            - **reaction\_id** (*str*) – The ID of the resulting reaction. This takes precedence over the
              auto-generated identifiers but beware that it might make boundary
              reactions harder to identify afterwards when using
              `boundary` or specifically
              `exchanges` etc.
            - **boundary\_condition** (*float**,* *str**,* *Basic*) – The boundary condition value to set. Must be an `int`,
              `float`, or a `sympy` expression dependent only on time.
              Default value is 0.
            - **\*\*kwargs** –

              subsystem :
              :   `str` for subsystem where the reaction is meant to occur.

              lb :
              :   `float` for the lower bound of the resulting reaction, or
                  `None` to use the default specified in the
                  `MassConfiguration`.

              ub :
              :   `float` for the upper bound of the resulting reaction, or
                  `None` to use the default specified in the
                  `MassConfiguration`.

              sbo\_term :
              :   `str` for the SBO term. A correct SBO term is set for the
                  available boundary types. If a custom boundary type is chosen,
                  a suitable SBO term should also be set.

        Returns
        :   The `MassReaction` of the new boundary reaction.

        Return type
        :   MassReaction

    `get_rate_expressions`(*self*, *reaction\_list=None*, *rate\_type=0*, *update\_reactions=False*)[source]¶
    :   Get the rate expressions for a `list` of reactions in the model.

        Notes

        If a reaction has a custom rate in the `MassModel.custom_rates`
        attribute, it will be returned only when the `rate_type=0`.

        Parameters
        :   - **reaction\_list** (*list*) – A `list` of `MassReaction`s to get the rate
              expressions for. Reactions must already exist in the model.
              If `None`, then return the rates for all reactions in the model.
            - **rate\_type** (*int*) –

              The type of rate law to return. Must be 0, 1, 2, or 3.

              > - If 0, the currrent rate expression is returned.
              > - Type 1 will utilize the `forward_rate_constant` and the
              >   `equilibrium_constant`.
              > - Type 2 will utilize the `forward_rate_constant` and the
              >   `reverse_rate_constant`.
              > - Type 3 will utilize the `equilibrium_constant` and the
              >   `reverse_rate_constant`.

              Default is `0.`
            - **update\_reactions** (*bool*) – If `True`, update the `MassReaction` rate in addition
              to returning the rate expressions. Will not remove a custom
              rate.

        Returns
        :   A `dict` of reaction rates where keys are the reaction ids
            and values are the rate law expressions.

        Return type
        :   dict

    `get_mass_action_ratios`(*self*, *reaction\_list=None*, *sympy\_expr=True*)[source]¶
    :   Get mass action ratios for a `list` of reactions in the model.

        Parameters
        :   - **reaction\_list** (*list*) – A `list` of `MassReaction`s to get the mass action
              ratios for. Reactions must already exist in the model.
              If `None`, then return the ratios for all reactions in the model.
            - **sympy\_expr** (*bool*) – If `True` then return the mass action ratios as a `sympy`
              expression, otherwise return the ratio as a human readable string.

        Returns
        :   A `dict` of mass action ratios where keys are the
            reaction ids and values are the ratios.

        Return type
        :   dict

    `get_disequilibrium_ratios`(*self*, *reaction\_list=None*, *sympy\_expr=True*)[source]¶
    :   Get disequilibrium ratios for a `list` of reactions in the model.

        Parameters
        :   - **reaction\_list** (*list*) – A `list` of `MassReaction`s to get the disequilibrium
              ratios for. Reactions must already exist in the model.
              If `None`, then return the ratios for all reactions in the model.
            - **sympy\_expr** (*bool*) – If `True` then return the disequilibrium ratios as a `sympy`
              expression, otherwise return the ratio as a human readable string.

        Returns
        :   A `dict` of mass action ratios where keys are the
            reaction ids and values are the ratios.

        Return type
        :   dict

    `add_custom_rate`(*self*, *reaction*, *custom\_rate*, *custom\_parameters=None*)[source]¶
    :   Add a custom rate for a reaction to the model.

        The change is reverted upon exit when using the `MassModel`
        as a context.

        Notes

        - Metabolites must already exist in the `MassModel`.
        - Default parameters of a `MassReaction` are automatically
          taken into account and do not need to be defined as additional
          custom parameters.

        Parameters
        :   - **reaction** (*MassReaction*) – The reaction associated with the custom rate.
            - **custom\_rate** (*str*) – The string representation of the custom rate expression. The string
              representation of the custom rate will be used to create a
              `sympy` expression that represents the custom rate.
            - **custom\_parameters** (*dict*) – A `dict` of custom parameters for the custom rate where the
              key:value pairs are the strings representing the custom parameters
              and their numerical values. The string representation of the custom
              parametes will be used to create the symbols needed for the sympy
              expression of the custom rate. If `None`, then parameters are
              assumed to already exist in the model.

        See also

        `all_parameter_ids()`
        :   Lists the default reaction parameters automatically accounted for.

    `remove_custom_rate`(*self*, *reaction*, *remove\_orphans=True*)[source]¶
    :   Remove the custom rate for a given reaction from the model.

        The change is reverted upon exit when using the `MassModel`
        as a context.

        Parameters
        :   - **reaction** (*MassReaction*) – The reaction assoicated with the custom rate to be removed.
            - **remove\_orphans** (*bool*) – If `True`, then remove any orphaned custom parameters from
              the model.

    `reset_custom_rates`(*self*)[source]¶
    :   Reset all custom rate expressions and parameters in a model.

        The change is reverted upon exit when using the `MassModel`
        as a context.

        Warning

        Using this method will remove all custom rates and custom rate
        parameters in the model. To remove a specific rate without affecting
        the other custom rates or parameters, use `remove_custom_rate()`
        instead.

    `add_units`(*self*, *unit\_defs*)[source]¶
    :   Add a `UnitDefinition` to the model `units`.

        The change is reverted upon exit when using the `MassModel`
        as a context.

        Notes

        The model will not automatically track or convert units. Therefore,
        it is up to the user to ensure unit consistency in the model.

        Parameters
        :   **unit\_defs** (*list*) – A `list` of `UnitDefinition`s to add to the model.

    `remove_units`(*self*, *unit\_defs*)[source]¶
    :   Remove a `UnitDefinition` from the model `units`.

        The change is reverted upon exit when using the `MassModel`
        as a context.

        Notes

        The model will not automatically track or convert units. Therefore,
        it is up to the user to ensure unit consistency in the model.

        Parameters
        :   **unit\_defs** (*list*) – A `list` of `UnitDefinition`s or their string
            identifiers to remove from the model.

    `reset_units`(*self*)[source]¶
    :   Reset all unit definitions in a model.

        The change is reverted upon exit when using the `MassModel`
        as a context.

        Warning

        Using this method will remove all `UnitDefinition`s from
        the model. To remove a `UnitDefinition` without affecting
        other units, use `remove_units()` instead.

    `get_elemental_matrix`(*self*, *array\_type=None*, *dtype=None*)[source]¶
    :   Get the elemental matrix for a model.

        Parameters
        :   - **array\_type** (*str*) – A string identifiying the desired format for the returned matrix.
              Valid matrix types include `'dense'`, `'dok'`, `'lil'`,
              `'DataFrame'`, and `'symbolic'`
              Default is `'dense'`. See the `matrix` module
              documentation for more information on the `array_type`.
            - **dtype** (*data-type*) – The desired array data-type for the matrix. If `None` then
              the data-type will default to `numpy.float64`.

        Returns
        :   The elemntal matrix for the `MassModel` returned
            as the given `array_type` and with a data-type of `dtype`.

        Return type
        :   matrix of type `array_type`

    `get_elemental_charge_balancing`(*self*, *array\_type=None*, *dtype=None*)[source]¶
    :   Get the elemental charge balance as a matrix for a model.

        Parameters
        :   - **array\_type** (*str*) – A string identifiying the desired format for the returned matrix.
              Valid matrix types include `'dense'`, `'dok'`, `'lil'`,
              `'DataFrame'`, and `'symbolic'`
              Default is `'dense'`. See the `matrix` module
              documentation for more information on the `array_type`.
            - **dtype** (*data-type*) – The desired array data-type for the matrix. If `None` then
              the data-type will default to `numpy.float64`.

        Returns
        :   The charge balancing matrix for the `MassModel` returned
            as the given `array_type` and with a data-type of `dtype`.

        Return type
        :   matrix of type `array_type`

    `repair`(*self*, *rebuild\_index=True*, *rebuild\_relationships=True*)[source]¶
    :   Update all indicies and pointers in the model.

        Notes

        Extends `repair()` of the
        `cobra.Model` to include the
        `MassModel.enzyme_modules` and `MassModel.units`.

        Parameters
        :   - **rebuild\_index** (*bool*) – If `True`, then rebuild the indicies kept in the reactions,
              metabolites, and genes.
            - **rebuild\_relationships** (*bool*) – If `True`, then reset all associations between the reactions,
              metabolites, genes, enzyme\_modules, and the `MassModel`,
              and rebuilds them.

    `copy`(*self*)[source]¶
    :   Create a partial “deepcopy” of the `MassModel`.

        All of the `MassMetabolite`s, `MassReaction`s,
        `Gene`s and `EnzymeModuleDict`s,
        the boundary conditions, custom\_rates, custom\_parameters, and the
        stoichiometric matrix are created anew, but in a faster fashion
        than `deepcopy`.

        Notes

        Overrides `copy()` of the
        `cobra.Model` so that all objects
        are `mass` objects and additional attributes of specific to the
        `MassModel`.

    `merge`(*self*, *right*, *prefix\_existing=None*, *inplace=True*, *objective='left'*)[source]¶
    :   Merge two models into one model with the objects from both.

        The reactions, metabolites, genes, enzyme modules, boundary conditions,
        custom rate expressions, rate parameters, compartments, units, notes,
        and annotations from the right model are also copied to left model.
        However, note that in cases where identifiers for objects are identical
        or a `dict` item has an identical key(s), priority will be given
        to what already exists in the left model.

        Custom constraints and variables from right models are also copied
        to left model, however note that, constraints and variables are
        assumed to be the same if they have the same name.

        Notes

        - When merging an `EnzymeModule` into a `MassModel`,
          the enzyme module is converted to an `EnzymeModuleDict` and
          stored in a `DictList` accessible via the
          `enzyme_modules` attribute. If an `EnzymeModuleDict`
          already exists in the model, it will be replaced.
        - Extends `merge()` of the
          `cobra.Model` by including
          additional `MassModel` attributes.

        Parameters
        :   - **right** (*MassModel*) – The model to merge into the left model.
            - **prefix\_existing** (*str*) – If provided, the string is used to prefix the reaction identifier
              of a reaction in the second model if that reaction already exists
              within the first model. Will also apply prefix to identifiers
              of enzyme modules in the second model.
            - **inplace** (*bool*) – If `True` then add reactions from second (right) model directly
              to the first (left) model. Otherwise, create a new model leaving
              the left model untouched. When done within the model as context,
              changes to the models are reverted upon exit.
            - **objective** (*str*) – One of `"left"`, `"right"` or `"sum"` for setting the
              objective of the resulting model to that of the corresponding
              model or the sum of both. Default is `"left"`.

        Returns
        :   A new `MassModel` or `self` representing the
            merged model.

        Return type
        :   MassModel

    `compute_steady_state_fluxes`(*self*, *pathways*, *independent\_fluxes*, *update\_reactions=False*)[source]¶
    :   Calculate the unique steady state flux for each reaction.

        The unique steady state flux for each reaction in the
        `MassModel` is calculated using defined pathways, independently
        defined fluxes, and steady state concentrations, where index of values
        in the pathways must correspond to the index of the reaction in
        `MassModel.reactions`.

        Notes

        The number of individually defined fluxes must be the same as the
        number of pathways in order to determine the solution. For best
        results, the number of pathways to specify must equal the dimension
        of the right nullspace.

        Parameters
        :   - **pathways** (*array-like*) – An array-like object that define the pathways through the reaction
              network of the model. The given pathway vectors must be the same
              length as the number of reactions in the model, with indicies of
              values in the pathway vector corresponding to the indicies of
              reactions in the `reactions` attribute.
            - **independent\_fluxes** (*dict*) – A `dict` of steady state fluxes where `MassReaction`s
              are keys and fluxes are values to utilize in order to calculate
              all other steady state fluxes. Must be the same length as the
              number of specified pathways.
            - **update\_reactions** (*bool*) – If `True` then update the `MassReaction.steady_state_flux`
              with the calculated steady state flux value for each reaction.

        Returns
        :   A `dict` where key:value pairs are the `MassReaction`s
            with their corresponding calculated steady state fluxes.

        Return type
        :   dict

        Warning

        The indicies of the values in the pathway vector must correspond to the
        indicies of the reactions in the `reactions` attribute in order
        for the method to work as intended.

    `calculate_PERCs`(*self*, *at\_equilibrium\_default=100000*, *update\_reactions=False*, *verbose=False*, *\*\*kwargs*)[source]¶
    :   Calculate pseudo-order rate constants for reactions in the model.

        Pseudo-order rate constants (PERCs) are considered to be the same as
        `forward_rate_constant` attributes, and are
        calculated based on the steady state concentrations and fluxes.

        Notes

        - All fluxes and concentrations used in calculations must be provided,
          including relevant boundary conditions. By default, the relevant
          values are taken from objects associated with the model.
        - To calculate PERCs for a subset of model reactions, use the
          `fluxes` kwawrg.

        Parameters
        :   - **at\_equilibrium\_default** (*float*) – The value to set the pseudo-order rate constant if the reaction is
              at equilibrium. Default is `100,000`.
            - **update\_reactions** (*bool*) – If `True` then will update the values for the
              `forward_rate_constant` attributes with the
              calculated PERC values.
            - **verbose** (*bool*) – Whether to output more verbose messages for errors and logging.
            - **\*\*kwargs** –

              fluxes :
              :   A `dict` of reaction fluxes where `MassReaction`s
                  are keys and fluxes are the values. Only reactions provided
                  will have their PERCs calculated. If `None`, PERCs are
                  calculated using the current steady state fluxes for all
                  reactions in the model.

                  Default is `None`.

              concentrationsdict
              :   A `dict` of concentrations necessary for the PERC
                  calculations, where `MassMetabolite`s are keys and
                  concentrations are the values. If `None`, the relevant
                  concentrations that exist in the model are used.

                  Default is `None`.

        Returns
        :   A `dict` where keys are strings identifers of the pseudo-order
            rate constants (as given by `MassReaction.kf_str`) and
            values are the calculated PERC values.

        Return type
        :   dict

    `build_model_from_string`(*self*, *model\_str*, *verbose=True*, *reaction\_split=';'*, *reaction\_id\_split=':'*, *\*\*kwargs*)[source]¶
    :   Create a `MassModel` from strings of reaction equations.

        Accepted `kwargs` are passed to the underlying function for reaction
        creation, `MassReaction.build_reaction_from_string()`.

        Takes a string representation of the reactions and uses the
        specifications supplied in the optional arguments to first infer a set
        of reactions and their identifiers, then to infer metabolites,
        metabolite compartments, and stoichiometries for the reactions. It
        also infers the reversibility of the reaction from the reaction arrow.
        For example:

        ```
        '''
        RID_1: S + E <=> ES;
        RID_2: ES -> E + P;
        RID_3: E + I <=> EI;
        '''
        ```

        where `RID` represents the identifier to assign the
        `MassReaction`.

        Parameters
        :   - **model** (*str*) – A string representing the reaction formulas (equation) for the
              model.
            - **verbose** (*bool*) – Setting the verbosity of the function.
            - **reaction\_split** (*str*) – Dividing individual reaction entries. Default is `";"`.
            - **reaction\_id\_split** (*str*) – Dividing individual reaction entries from their identifiers.
              Default is `":"`.
            - **\*\*kwargs** –

              fwd\_arrow :
              :   `re.compile()` or `None` for forward irreversible
                  reaction arrows. If `None`, the arrow is expected to
                  be `'-->'` or `'==>'`.

              rev\_arrow :
              :   `re.compile()` or `None` for backward irreversible
                  reaction arrows. If `None`, the arrow is expected to
                  be `'<--'` or `'<=='`.

              reversible\_arrow :
              :   `re.compile()` or `None` for reversible reaction arrows.
                  If `None`, the arrow is expected to
                  be `'<=>'` or `'<->'`.

              term\_splitstr
              :   Dividing individual metabolite entries. Default is `"+"`.

        See also

        `MassReaction.build_reaction_from_string()`
        :   Base method for building reactions.

    `update_parameters`(*self*, *parameters*, *verbose=True*)[source]¶
    :   Update the parameters associated with the MassModel.

        Parameters can be the following:

        > - `MassReaction.forward_rate_constant` (
        >   `kf`)
        > - `MassReaction.reverse_rate_constant` (
        >   `kr`)
        > - `MassReaction.equilibrium_constant` (
        >   `Keq`)
        > - `MassReaction.steady_state_flux` (
        >   `v`)
        > - `boundary_conditions`
        > - `custom_parameters`

        Notes

        The reactions must already exist in the model in order to change
        associated parameters. Any identifiers that are not identifiers of
        standard reaction parameter or of any ‘boundary metabolites’ will be
        set as a custom parameter.

        Parameters
        :   - **parameters** (*dict*) – A `dict` containing the parameter identifiers as strings and
              their corresponding values to set in the model.
            - **verbose** (*bool*) – If `True` then display the warnings that may be raised when
              setting reaction parameters. Default is `True`.

        See also

        `MassReaction.all_parameter_ids`
        :   Lists the default reaction parameter identifiers.

        `MassModel.boundary_metabolites`
        :   Lists the ‘boundary metabolites’ found in the model.

    `update_initial_conditions`(*self*, *initial\_conditions*, *verbose=True*)[source]¶
    :   Update the initial conditions of the model.

        Can also be used to update initial conditions of fixed metabolites to
        change the concentration value at which the metabolite is fixed.

        Notes

        The metabolite(s) must already exist in the model to set the initial
        conditions. Initial conditions for the metabolites are accessed through
        `MassMetabolite.initial_condition`. If an initial condition for
        a metabolite already exists, it will be replaced.

        Parameters
        :   - **initial\_conditions** (*dict*) – A `dict` where metabolites are the keys and the initial
              conditions are the values.
            - **verbose** (*bool*) – If `True` then display the warnings that may be raised when
              setting metabolite initial conditions. Default is `True`.

    `update_custom_rates`(*self*, *custom\_rates*, *custom\_parameters=None*)[source]¶
    :   Update the custom rates of the model.

        Parameters
        :   - **custom\_rates** (*dict*) – A `dict` where `MassReaction`s or their string
              identifiers are the keys and the rates are the string
              representations of the custom rate expression.
            - **custom\_parameters** (*dict*) – A `dict` of custom parameters for the custom rates, where the
              key:value pairs are the strings representing the custom parameters
              and their numerical values. If a custom parameter already exists in
              the model, it will be updated.

        Notes

        The reaction(s) must already exist in the model to set the custom rate.

        See also

        `add_custom_rate()`

    `has_equivalent_odes`(*self*, *right*, *verbose=False*)[source]¶
    :   Determine whether `odes` between two models are equivalent.

        Notes

        The ODEs between two models are compared to determine whether the
        models can be considered equivalent, meaning that the models contain
        the same metabolites, reactions, and rate expressions such that they
        require the same set of parameters and initial conditions for
        simulation.

        Parameters
        :   - **right** (*MassModel*) – The `MassModel` to compare to the left model (`self`).
            - **verbose** (*bool*) – If `True`, display the reason(s) for the differences in the left
              and right models. Default is `False`.

        Returns
        :   Returns a bool indicating whether the model ODEs are equivalent.

        Return type
        :   bool

    `set_steady_state_fluxes_from_solver`(*self*)[source]¶
    :   Set reaction steady state fluxes based on the state of the solver.

        Only works when reaction is associated with a model that has been
        optimized.

    `_cobra_to_mass_repair`(*self*)[source]¶
    :   Convert associated cobra objects to mass objects for self.

        Warning

        This method is intended for internal use only.

    `_mk_stoich_matrix`(*self*, *array\_type=None*, *dtype=None*, *update\_model=True*)[source]¶
    :   Return the stoichiometric matrix for a given MassModel.

        The rows represent the chemical species and the columns represent the
        reaction. S[i, j] therefore contains the quantity of species ‘i’
        produced (positive) or consumed (negative) by reaction ‘j’.

        Warning

        This method is intended for internal use only. To safely update the
        stoichiometric matrix, use `update_S()` instead.

    `_get_all_parameters`(*self*)[source]¶
    :   Get a dict containing all of defined model parameters in the model.

        Warning

        This method is intended for internal use only.

    `_copy_model_metabolites`(*self*, *new\_model*)[source]¶
    :   Copy the metabolites in creating a partial “deepcopy” of model.

        Warning

        This method is intended for internal use only.

    `_copy_model_genes`(*self*, *new\_model*)[source]¶
    :   Copy the genes in creating a partial “deepcopy” of model.

        Warning

        This method is intended for internal use only.

    `_copy_model_reactions`(*self*, *new\_model*)[source]¶
    :   Copy the reactions in creating a partial “deepcopy” of model.

        Warning

        This method is intended for internal use only.

    `_copy_model_enzyme_modules`(*self*, *new\_model*)[source]¶
    :   Copy the enzyme\_modules in creating a partial “deepcopy” of model.

        Warning

        This method is intended for internal use only.

    `_copy_model_groups`(*self*, *new\_model*)[source]¶
    :   Copy the groups in creating a partial “deepcopy” of model.

        Warning

        This method is intended for internal use only.

    `_repr_html_`(*self*)[source]¶
    :   HTML representation of the overview for the MassModel.

        Warning

        This method is intended for internal use only.

    `__setstate__`(*self*, *state*)[source]¶
    :   Ensure all objects in the model point to the MassModel.

        Extends
        `Model.__setstate__`
        to include enzyme\_modules

        Warning

        This method is intended for internal use only.

    `__getstate__`(*self*)[source]¶
    :   Get the state for serialization.

        Ensures that the context stack is cleared prior to serialization,
        since partial functions cannot be pickled reliably

        Warning

        This method is intended for internal use only.

    `__dir__`(*self*)[source]¶
    :   Override default dir() implementation to list only public items.

        Warning

        This method is intended for internal use only.

###### `mass.core.mass_reaction`¶

MassReaction is a class for holding information regarding reactions.

The `MassReaction` class inherits and extends the
`Reaction` class in `cobra`. It contains
additional information required for simulations and other `mass`
functions and workflows.

Some key differences between the
`cobra.Reaction` and the
`mass.MassReaction` are
listed below:

> - When instantiating a `MassReaction` from a
>   `cobra.Reaction`, any associated
>   `cobra.Metabolite` will also
>   be converted into a `MassMetabolite`.
> - Unlike the `cobra.Reaction`
>   which initializes the `lower_bound`
>   at a default value of `0.0`, the `MassReaction` initializes the
>   `lower_bound` as `None` to utilize
>   the default value set in the `MassConfiguration`.
> - The `MassReaction` contains both the
>   `steady_state_flux` and the inherited
>   `cobra.Reaction.flux`
>   attributes. Note that these attributes **DO NOT** refer to the same flux
>   value unless specifically set as such. The
>   `flux` refers to the flux value in
>   the most recent optimization solution, while the
>   `steady_state_flux` refers to the net steady
>   state flux through the reaction.
> - The `MassReaction` contains both the
>   `reversible`
>   and `reversibility` attributes.
>
>   Note that these attributes are **NOT** the same. While the
>   `reversible` refers the the kinetic reversibility of
>   the reaction and thus determines how the `rate` is
>   set, while the inhertied
>   `cobra.Reaction.reversibility`
>   refers to the computed reversibility based on the current lower
>   and upper bounds.
> - The arrow in the string representation of the reaction is dependent on
>   the `MassReaction.reversible` rather than the inhertied
>   `cobra.Reaction.reversibility`
>   attribute.

###### Module Contents¶

###### Classes¶

|  |  |
| --- | --- |
| `MassReaction` | Class for holding kinetic information regarding a biochemical reaction. |

*class* `mass.core.mass_reaction.``MassReaction`(*id\_or\_reaction=None*, *name=''*, *subsystem=''*, *reversible=True*, *steady\_state\_flux=None*, *\*\*kwargs*)[source]¶
:   Bases: `cobra.core.reaction.Reaction`

    Class for holding kinetic information regarding a biochemical reaction.

    Accepted `kwargs` are passed onto the initialization method for the
    base class `Reaction`.

    Parameters
    :   - **id\_or\_reaction** (*str**,* *Reaction**,* *MassReaction*) – A string identifier to associate with the reaction, or an existing
          reaction. If an existing reaction object is provided, a new
          `MassReaction` object is instantiated with the same properties
          as the original reaction.
        - **name** (*str*) – A human readable name for the reaction.
        - **subsystem** (*str*) – The subsystem where the reaction is meant to occur.
        - **reversible** (*bool*) – The kinetic reversibility of the reaction. Irreversible reactions have
          an equilibrium constant and a reverse rate constant as set in the
          `irreversible_Keq` and
          `irreversible_kr` attributes of the
          `MassConfiguration`. Default is `True`.
        - **\*\*kwargs** –

          lower\_boundfloat or None
          :   The lower flux bound for optimization. If `None` then
              the default bound from the `MassConfiguration` is used.

              Default is `None`.

          upper\_boundfloat or None
          :   The upper flux bound for optimization. If `None` then
              the default bound from the `MassConfiguration` is used.

              Default is `None`.

    *property* `reversible`(*self*)[source]¶
    :   Get or set the kinetic reversibility of the reaction.

        When using a HistoryManager context, this attribute can be set
        temporarily, reversed when the exiting the context.

        Parameters
        :   **reversible** (*bool*) – The kinetic reversibility of the reaction.

        Warning

        Changing the `reversible` attribute will reset the
        `equilibrium_constant` and the `reverse_rate_constant`
        to the initialization defaults.

    *property* `steady_state_flux`(*self*)[source]¶
    :   Get or set the steady state flux of the reaction.

        When using a HistoryManager context, this attribute can be set
        temporarily, reversed when the exiting the context.

        Parameters
        :   **flux\_value** (*bool*) – The steady state flux value of the reaction.

    *property* `forward_rate_constant`(*self*)[source]¶
    :   Get or set the forward rate constant (kf) of the reaction.

        When using a HistoryManager context, this attribute can be set
        temporarily, reversed when the exiting the context.

        Notes

        Forward rate constants cannot be negative.

        Parameters
        :   **value** (*float*) – A non-negative number for the forward rate constant (kf) of the
            reaction.

        Raises
        :   **ValueError** – Occurs when trying to set a negative value.

    *property* `reverse_rate_constant`(*self*)[source]¶
    :   Get or set the reverse rate constant (kr) of the reaction.

        When using a HistoryManager context, this attribute can be set
        temporarily, reversed when the exiting the context.

        Notes

        - Reverse rate constants cannot be negative.
        - If reaction is not reversible, will warn the user instead of setting
          the parameter value.

        Parameters
        :   **value** (*float*) – A non-negative number for the reverse rate constant (kr) of the
            reaction.

        Raises
        :   **ValueError** – Occurs when trying to set a negative value.

    *property* `equilibrium_constant`(*self*)[source]¶
    :   Get or set the equilibrium constant (Keq) of the reaction.

        When using a HistoryManager context, this attribute can be set
        temporarily, reversed when the exiting the context.

        Notes

        - Equilibrium constants cannot be negative.
        - If reaction is not reversible, will warn the user instead of setting
          the parameter value.

        Parameters
        :   **value** (*float*) – A non-negative number for the equilibrium constant (Keq)
            of the reaction.

        Raises
        :   **ValueError** – Occurs when trying to set a negative value.

    *property* `parameters`(*self*)[source]¶
    :   Return a `dict` of rate and equilibrium constants.

        Notes

        The `reverse_rate_constant` is only included for reversible
        reactions. Additionally, only rate and equilibrium constants are
        accessed here. Steady state fluxes can be accessed through the
        `steady_state_flux` attribute, and custom parameters can only be
        accessed through the model.

    *property* `metabolites`(*self*)[source]¶
    :   Return the metabolites of a reaction as a read only copy.

    *property* `reactants`(*self*)[source]¶
    :   Return a `list` of reactants for the reaction.

    *property* `products`(*self*)[source]¶
    :   Return a `list` of products for the reaction.

    *property* `stoichiometry`(*self*)[source]¶
    :   Return a `list` containing the stoichiometry for the reaction.

    *property* `rate`(*self*)[source]¶
    :   Return the current rate as a `sympy` expression.

        If reaction has a custom rate in its associated `MassModel`,
        the custom rate will be returned instead.

    *property* `model`(*self*)[source]¶
    :   Return the `MassModel` associated with the reaction.

    *property* `reaction`(*self*)[source]¶
    :   Get or set the reaction as a human readable string.

        Parameters
        :   **reaction\_str** (*str*) – String representation of the reaction.

        Warning

        Care must be taken when setting a reaction using this method.
        See documentation for `build_reaction_from_string()` for more
        information.

        See also

        `build_reaction_string()`
        :   Base function for getter method.

    *property* `compartments`(*self*)[source]¶
    :   Return the set of compartments where the metabolites are located.

    *property* `boundary`(*self*)[source]¶
    :   Determine whether or not the reaction is a boundary reaction.

        Will return `True` if the reaction has no products or no reactants
        and only one metabolite.

        Notes

        These are reactions with a sink or a source term (e.g. ‘A –> ‘)

    *property* `boundary_metabolite`(*self*)[source]¶
    :   Return an ‘boundary’ metabolite for bounary reactions.

        Notes

        The ‘boundary\_metabolite’ represents the metabolite that corresponds
        to the empty part of a boundary reaction through a string. It’s primary
        use is for setting of the `boundary_conditions`
        without creating a `MassMetabolite` object. Therefore it is
        not counted as a metabolite but instead as a parameter.

        Returns
        :   **boundary\_metabolite** – String representation of the boundary metabolite of the reaction,
            or `None` if the reaction is not considered a boundary reaction.

        Return type
        :   str

        See also

        `boundary()`
        :   Method must return `True` to get the boundary\_metabolite.

    *property* `genes`(*self*)[source]¶
    :   Return a `frozenset` of the genes associated with the reaction.

    *property* `gene_reaction_rule`(*self*)[source]¶
    :   Get or set the gene reaction rule for the reaction.

        Parameters
        :   **new\_rule** (*str*) – String representation of the new reaction rule.

        Notes

        New genes will be associated with the reaction and old genes will be
        dissociated from the reaction.

    *property* `gene_name_reaction_rule`(*self*)[source]¶
    :   Display gene\_reaction\_rule with names.

        Warning

        Do NOT use this string for computation. It is intended to give a
        representation of the rule using more familiar gene names instead
        of the often cryptic ids.

    *property* `functional`(*self*)[source]¶
    :   Check if all required enzymes for the reaction are functional.

        Returns
        :   Returns `True` if the gene-protein-reaction (GPR) rule is
            fulfilled for the reaction, or if the reaction does not have an
            assoicated `MassModel`. Otherwise returns `False`.

        Return type
        :   bool

    *property* `flux_symbol_str`(*self*)[source]¶
    :   Return the string representation for the reaction flux symbol.

    *property* `all_parameter_ids`(*self*)[source]¶
    :   Return `list` of strings representing non-custom parameters.

    *property* `kf_str`(*self*)[source]¶
    :   Return the string representation of the forward rate constant.

    *property* `Keq_str`(*self*)[source]¶
    :   Return the string representation of the equilibrium constant.

    *property* `kr_str`(*self*)[source]¶
    :   Return the string representation of the reverse rate constant.

    *property* `kf`(*self*)[source]¶
    :   Alias for the `forward_rate_constant`.

    *property* `kr`(*self*)[source]¶
    :   Alias for the `reverse_rate_constant`.

    *property* `Keq`(*self*)[source]¶
    :   Alias for the `equilibrium_constant`.

    *property* `S`(*self*)[source]¶
    :   Alias for the `stoichiometry`.

    *property* `v`(*self*)[source]¶
    :   Alias for the `steady_state_flux`.

    `reverse_stoichiometry`(*self*, *inplace=False*, *reverse\_parameters=False*, *reverse\_bounds=True*, *reverse\_flux=True*)[source]¶
    :   Reverse the stoichiometry of the reaction.

        Reversing the stoichiometry will turn the products into the reactants
        and the reactants into the products.

        Notes

        To avoid errors when reversing the reaction equilibrium constant:

        > - If `self.equilibrium_constant=0.` then
        >   `new_reaction.equilibrium_constant=float("inf")`
        > - If `self.equilibrium_constant=float("inf")` then
        >   `new_reaction.equilibrium_constant=0.`

        Parameters
        :   - **inplace** (*bool*) – If `True`, modify the reaction directly. Otherwise a new reaction
              is created, modified, and returned.
            - **reverse\_parameters** (*bool*) –

              If `True` then also switch the reaction rate constants and
              inverse the equilibrium constants such that:

              ```
              new_reaction.forward_rate_constant = self.reverse_rate_constant
              new_reaction.reverse_rate_constant = self.forward_rate_constant
              new_reaction.equilibrium_constant = 1/self.equilibrium_constant
              ```

              Default is `False`.
            - **reverse\_bounds** (*bool*) –

              If `True` then also switch the lower and upper bounds with one
              another such that:

              ```
              new_reaction.bounds = (-self.upper_bound, -self.lower_bound)
              ```

              Default is `True`.
            - **reverse\_flux** (*bool*) –
              > If `True` then also switch the direction of the flux such that:
              >
              > ```
              > new_reaction.steady_state_flux = -self.steady_state_flux
              > ```

              Default is `True`.

        Returns
        :   **new\_reaction** – Returns the original `MassReaction` if `inplace=True`.
            Otherwise return a modified copy of the original reaction.

        Return type
        :   MassReaction

    `get_mass_action_rate`(*self*, *rate\_type=1*, *update\_reaction=False*, *destructive=False*)[source]¶
    :   Get the mass action rate law for the reaction.

        Parameters
        :   - **rate\_type** (*int*) –

              The type of rate law to return. Must be 1, 2, or 3.

              > - Type 1 will utilize the `forward_rate_constant` and the
              >   `equilibrium_constant`.
              > - Type 2 will utilize the `forward_rate_constant` and the
              >   `reverse_rate_constant`.
              > - Type 3 will utilize the `equilibrium_constant` and the
              >   `reverse_rate_constant`.

              Default is `1`.
            - **update\_reaction** (*bool*) – Whether to update the `MassReaction.rate` attribute
              in addition to returning the rate law. Default is `False`.
            - **destructive** (*bool*) – If `True` and the reaction has a custom rate law in its
              associated model, then setting `update_reaction=True` will
              replace the rate law and remove the custom rate law and orphaned
              parameters from the model. Default is `False`.

        Returns
        :   **rate\_expression** – The rate law as a `sympy` expression. If the reaction has no
            metabolites associated, `None` will be returned.

        Return type
        :   `sympy.core.basic.Basic` or `None`

        Warning

        Setting `update_reaction=True` will not remove any associated
        custom rate laws from the model unless `destructive=True` as well.

    `get_forward_mass_action_rate_expression`(*self*, *rate\_type=None*)[source]¶
    :   Get the forward mass action rate expression for the reaction.

        Parameters
        :   **rate\_type** (*int**,* *None*) –

            The type of rate law to return. Must be 1, 2, or 3.

            > - Type 1 and 2 will utilize the `forward_rate_constant`.
            > - Type 3 will utilize the `equilibrium_constant` and the
            >   `reverse_rate_constant`.

            If `None`, the current rate type will be used.
            Default is `None`.

        Returns
        :   **fwd\_rate** – The forward rate as a `sympy` expression. If the reaction
            has no metabolites associated, `None` will be returned.

        Return type
        :   `sympy.core.basic.Basic` or `None`

    `get_reverse_mass_action_rate_expression`(*self*, *rate\_type=1*)[source]¶
    :   Get the reverse mass action rate expression for the reaction.

        Parameters
        :   **rate\_type** (*int**,* *None*) –

            The type of rate law to return. Must be 1, 2, or 3.

            > - Type 1 will utilize the `forward_rate_constant` and the
            >   `equilibrium_constant`.
            > - Type 2 and 3 will utilize the `reverse_rate_constant`.

            If `None`, the current rate type will be used.
            Default is `None`.

        Returns
        :   **rev\_rate** – The reverse rate as a `sympy` expression. If the reaction
            has no metabolites associated, `None` will be returned.

        Return type
        :   `sympy.core.basic.Basic` or `None`

    `get_mass_action_ratio`(*self*)[source]¶
    :   Get the mass action ratio as a `sympy` expression.

        Returns
        :   The mass action ratio as a sympy expression.

        Return type
        :   `sympy.core.basic.Basic`

    `get_disequilibrium_ratio`(*self*)[source]¶
    :   Get the disequilibrium ratio as a `sympy` expression.

        Returns
        :   The disequilibrium ratio as a sympy expression.

        Return type
        :   `sympy.core.basic.Basic`

    `copy`(*self*)[source]¶
    :   Copy a reaction.

        The reaction parameters, referenced metabolites, and genes are also
        copied.

    `get_coefficient`(*self*, *metabolite\_id*)[source]¶
    :   Return the coefficient of a metabolite in the reaction.

        Parameters
        :   **metabolite\_id** (*str* *or* *MassMetabolite*) – The `MassMetabolite` or the string identifier of the
            metabolite whose coefficient is desired.

    `get_coefficients`(*self*, *metabolite\_ids*)[source]¶
    :   Return coefficients for a `list` of metabolites in the reaction.

        Parameters
        :   **metabolite\_ids** (*iterable*) – Iterable containing the `MassMetabolite`s or
            their string identifiers.

    `add_metabolites`(*self*, *metabolites\_to\_add*, *combine=True*, *reversibly=True*)[source]¶
    :   Add metabolites and their coefficients to the reaction.

        If the final coefficient for a metabolite is 0 then it is removed from
        the reaction.

        The change is reverted upon exit when using the `MassModel`
        as a context.

        Notes

        - A final coefficient of < 0 implies a reactant and a final
          coefficient of > 0 implies a product.
        - Extends `add_metabolites()` of the
          `cobra.Reaction` by first
          ensuring that the metabolites to be added are
          `MassMetabolite`s and not
          `cobra.Metabolites`.
          and error message raised reflects the `mass` object.
        - If a `cobra.Metabolite` is
          provided. a warning is raised and a `MassMetabolite`
          will be instantiated using the
          `cobra.Metabolite`.

        Parameters
        :   - **metabolites\_to\_add** (*dict*) – A `dict` with `MassMetabolite`s or metabolite
              identifiers as keys and stoichiometric coefficients as values. If
              keys are strings (id of a metabolite), the reaction must already
              be part of a `MassModel` and a metabolite with the given
              id must already exist in the `MassModel`.
            - **combine** (*bool*) – Describes the behavior of existing metabolites.
              If `True`, the metabolite coefficients are combined together.
              If `False` the coefficients are replaced.
            - **reversibly** (*bool*) – Whether to add the change to the context to make the change
              reversible (primarily intended for internal use).

        See also

        `subtract_metabolites()`

    `subtract_metabolites`(*self*, *metabolites*, *combine=True*, *reversibly=True*)[source]¶
    :   Subtract metabolites and their coefficients from the reaction.

        This function will ‘subtract’ metabolites from a reaction by adding
        the given metabolites with `-1 * coeffcient`. If the final
        coefficient for a metabolite is 0, the metabolite is removed from the
        reaction.

        The change is reverted upon exit when using the `MassModel`
        as a context.

        Notes

        - A final coefficient of < 0 implies a reactant and a final
          coefficient of > 0 implies a product.
        - Extends `cobra.core.reaction.Reaction.subtract_metabolites()` of
          the `cobra.Reaction` by first
          ensuring that the metabolites to be added are
          `MassMetabolite`s and not
          `cobra.Metabolites`.
          and error message raised reflects the `mass` object.
        - If a `cobra.Metabolite` is
          provided. a warning is raised and a `MassMetabolite`
          will be instantiated using the
          `cobra.Metabolite`.

        Parameters
        :   - **metabolites** (*dict*) – A `dict` with `MassMetabolite`s or their identifiers
              as keys and stoichiometric coefficients as values. If keys are
              strings (id of a metabolite), the reaction must already be part of
              a `MassModel` and a metabolite with the given id must
              already exist in the `MassModel`.
            - **combine** (*bool*) – Describes the behavior of existing metabolites.
              If `True`, the metabolite coefficients are combined together.
              If `False` the coefficients are replaced.
            - **reversibly** (*bool*) – Whether to add the change to the context to make the change
              reversible (primarily intended for internal use).

        See also

        `add_metabolites()`

    `build_reaction_string`(*self*, *use\_metabolite\_names=False*)[source]¶
    :   Generate a human readable string to represent the reaction.

        Notes

        Overrides `build_reaction_string()`
        of the `cobra.Reaction` so that
        the reaction arrow depends on `MassReaction.reversible` rather
        than the inherited
        `cobra.Reaction.reversibility`
        attribute.

        Parameters
        :   **use\_metabolite\_names** (*bool*) – If `True`, use the metabolite names instead of their identifiers.
            Default is `False`.

        Returns
        :   **reaction\_string** – A string representation of the reaction.

        Return type
        :   str

    `check_mass_balance`(*self*)[source]¶
    :   Compute the mass and charge balances for the reaction.

        Returns
        :   Returns a `dict` of `{element: amount}` for unbalanced
            elements, with the “charge” treated as an element in the `dict`.
            For a balanced reaction, an empty `dict` is returned.

        Return type
        :   dict

    `build_reaction_from_string`(*self*, *reaction\_str*, *verbose=True*, *fwd\_arrow=None*, *rev\_arrow=None*, *reversible\_arrow=None*, *term\_split='+'*)[source]¶
    :   Build reaction from reaction equation `reaction_str` using parser.

        Takes a string representation of the reaction and uses the
        specifications supplied in the optional arguments to infer a set of
        metabolites, metabolite compartments, and stoichiometries for the
        reaction. It also infers the refversibility of the reaction from the
        reaction arrow.

        For example:

        > - `'A + B <=> C'` for reversible reactions, A & B are reactants.
        > - `'A + B --> C'` for irreversible reactions, A & B are
        >   reactants.
        > - `'A + B <-- C'` for irreversible reactions, A & B are products.

        The change is reverted upon exit when using the `MassModel`
        as a context.

        Notes

        Extends
        `build_reaction_from_string()`
        of the `cobra.Reaction`
        in order to change how the irreversible backwards arrow is
        interpreted, affecting the assignment of reactants and products
        rather than how the bounds are set.

        Parameters
        :   - **reaction\_str** (*str*) – A string containing the reaction formula (equation).
            - **verbose** (*bool*) – Setting the verbosity of the function. Default is `True`.
            - **fwd\_arrow** (*re.compile**,* *None*) – For forward irreversible reaction arrows. If `None`, the
              arrow is expected to be `'-->'` or `'==>'`.
            - **rev\_arrow** (*re.compile**,* *None*) – For backward irreversible reaction arrows. If `None`, the
              arrow is expected to be `'<--'` or `'<=='`.
            - **reversible\_arrow** (*re.compile**,* *None*) – For reversible reaction arrows. If `None`, the arrow is expected
              to be `'<=>'` or `'<->'`.
            - **term\_split** (*str*) – Dividing individual metabolite entries. Default is `"+"`.

    `knock_out`(*self*)[source]¶
    :   Knockout reaction by setting its bounds to zero.

    `_cobra_to_mass_repair`(*self*)[source]¶
    :   Convert associated cobra.Metabolites to MassMetabolites for self.

        Warning

        This method is intended for internal use only.

    `_associate_gene`(*self*, *cobra\_gene*)[source]¶
    :   Associates a `Gene` with the reaction.

        Parameters
        :   **cobra\_gene** (*Gene*) – `Gene` to be assoicated with the reaction.

        Warning

        This method is intended for internal use only.

    `_dissociate_gene`(*self*, *cobra\_gene*)[source]¶
    :   Dissociates a `Gene` with the reaction.

        Parameters
        :   **cobra\_gene** (*Gene*) – `Gene` to be disassociated with the
            reaction.

        Warning

        This method is intended for internal use only.

    `_make_boundary_metabolites`(*self*)[source]¶
    :   Make the boundary metabolite.

        Warning

        This method is intended for internal use only.

    `_repr_html_`(*self*)[source]¶
    :   HTML representation of the overview for the MassReaction.

        Warning

        This method is intended for internal use only.

    `__copy__`(*self*)[source]¶
    :   Create a copy of the MassReaction.

        Warning

        This method is intended for internal use only.

    `__deepcopy__`(*self*, *memo*)[source]¶
    :   Create a deepcopy of the MassReaction.

        Warning

        This method is intended for internal use only.

    `__str__`(*self*)[source]¶
    :   Create an id string with the stoichiometry.

        Warning

        This method is intended for internal use only.

    `__dir__`(*self*)[source]¶
    :   Override default dir() implementation to list only public items.

        Warning

        This method is intended for internal use only.

###### `mass.core.mass_solution`¶

MassSolution is a class for storing the simulation results.

After a `Simulation` is used to simulate a `mass` model,
`MassSolution`s are created to store the results computed over the
time interval specified when simulating. These results are divided into two
categories:

> - Concentration solutions (abbreviated as ConcSols)
> - Reaction Flux solutions (abbreviated as FluxSols)

`MassSolution`s are therefore given an identifier of the
following format: `{id_or_model}_{solution_type}Sols` where `id_or_model`
and `solution_type` correspond to the simulated model and resulting solution
category, respectively.

All solutions in a `MassSolution` can be accessed via attribute
accessors. A `MassSolution` also contains standard `dict` methods.

All functions from the `mass.visualization` submodule are designed
to work seamlessly with `MassSolution`s, provided they are properly
created.

###### Module Contents¶

###### Classes¶

|  |  |
| --- | --- |
| `MassSolution` | Container to store the solutions for the simulation of a model. |

*class* `mass.core.mass_solution.``MassSolution`(*id\_or\_model*, *solution\_type=''*, *data\_dict=None*, *time=None*, *interpolate=False*, *initial\_values=None*)[source]¶
:   Bases: `mass.util.dict_with_id.DictWithID`

    Container to store the solutions for the simulation of a model.

    Parameters
    :   - **id\_or\_model** (*str**,* *MassModel*) – A `MassModel` or a string identifier to associate with the
          stored solutions. If a `MassModel` is provided, then the model
          identifier will be used.
        - **solution\_type** (*str*) – The type of solution being stored. Must be `'Conc'` or `'flux'`.
        - **data\_dict** (*dict*) – A dict containing the solutions to store. If `None` provided then
          the `MassSolution` will be initialized with no solutions.
          Solutions can be added or changed later using various `dict`
          methods (e.g. `update()`).
        - **time** (*array-like*) – An array-like object containing the time points used in obtaining the
          solutions.
        - **interpolate** (*bool*) –

          If `True` then all solutions are converted and stored as
          `scipy.interpolate.interp1d` objects.
          If `False`, solutions are converted and stored as
          `numpy.ndarray`s.

          Default value is `False`.

    *property* `simulation`(*self*)[source]¶
    :   Return the associated `Simulation`.

    *property* `time`(*self*)[source]¶
    :   Get or set the time points stored in the `MassSolution`.

        Notes

        If the solutions stored in the `MassSolution` are
        `numpy.ndarray`s and the numerical arrays of the solutions
        will be recomputed to correspond to the new time points using
        `scipy.interpolate.interp1d` interpolating functions

        Parameters
        :   **value** (*array-like*) – An array-like object containing the time points used in calculating
            the solutions to be stored.

    *property* `t`(*self*)[source]¶
    :   Shorthand method to get or set the stored time points.

        Notes

        If the solutions stored in the `MassSolution` are
        `numpy.ndarray`s and the numerical arrays of the solutions
        will be recomputed to correspond to the new time points using
        `interp1d` interpolating functions

        Parameters
        :   **value** (*array-like*) – An array-like object containing the time points used in calculating
            the solutions to be stored.

    *property* `interpolate`(*self*)[source]¶
    :   Get or set whether solutions are stored as interpolating functions.

        Parameters
        :   **value** (*bool*) – If `True`, solutions are stored in the `MassSolution` as
            `interp1d` objects. Otherwise solutions
            are stored as arrays.

    *property* `initial_values`(*self*)[source]¶
    :   Get or set a `dict` of the initial values for solution variables.

        Notes

        Primarily for storing the intial values used in a simulation and for
        calculating deviations from the initial state.

        Parameters
        :   **initial\_values** (*dict*) – A `dict` containining the variables stored in the
            `MassSolution` and their initial values.

    `view_time_profile`(*self*, *deviation=False*, *plot\_function='loglog'*)[source]¶
    :   Generate a quick view of the time profile for the solution.

        See `visualization` documentation for more information.

        Parameters
        :   - **deviation** (*bool*) – Whether to plot time profiles as a deviation from their
              initial value.
            - **plot\_function** (*str*) –

              The plotting function to use. Accepted values are the following:

              > - `"plot"` for a linear x-axis and a linear y-axis
              >   via `Axes.plot()`
              > - `"loglog"` for a logarithmic x-axis and a logarithmic y-axis
              >   via `Axes.loglog()`
              > - `"semilogx`” for a logarithmic x-axis and a linear y-axis
              >   via `Axes.semilogx()`
              > - `"semilogy"` for a linear x-axis and a logarithmic y-axis
              >   via `Axes.semilogy()`

        Notes

        Will clear and use the current axis (accessible via
        `matplotlib.pyplot.gca()`).

    `view_tiled_phase_portraits`(*self*)[source]¶
    :   Generate a preview of the phase portraits for the solution.

        See `visualization` documentation for more information.

        Notes

        Will clear and use the current axis (accessible via
        `matplotlib.pyplot.gca()`).

    `to_frame`(*self*)[source]¶
    :   Return the stored solutions as a `pandas.DataFrame`.

    `make_aggregate_solution`(*self*, *aggregate\_id*, *equation*, *variables=None*, *parameters=None*, *update=True*)[source]¶
    :   Make a new aggregate variable and its solution from an equation.

        Parameters
        :   - **aggregate\_id** (*str*) – An identifier for the solution to be made.
            - **equation** (*str*) – A string representing the equation of the new solution.
            - **variables** (*iterable* *or* *None*) – Either an iterable of object identifiers or the objects themselves
              representing keys in the `MassSolution` that are used as
              variables in equation. If `None`, then all keys of the solution
              object are checked as variables, potentially resulting in lower
              performance time.
            - **parameters** (*dict* *or* *None*) – A `dict` of additional parameters to use, where key:value pairs
              are the parameter identifiers and their numerical values.
              If `None` then it is assumed that there are no additional
              parameters in the equation.
            - **update** (*bool*) – Whether to add the new solution into the `MassSolution`.
              via the `update()` method. Default is `True`.

        Returns
        :   **solution** – A `dict` containing where the key is the `aggregate_id` and the
            value is the newly created solution as the same type as the
            variable solutions

        Return type
        :   dict

        Raises
        :   **SympifyError** – Raised if the `equation_str` could not be interpreted.

    `__getattribute__`(*self*, *name*)[source]¶
    :   Override of default `getattr()` to enable attribute accessors.

        Warning

        This method is intended for internal use only.

    `__dir__`(*self*)[source]¶
    :   Override of default `dir()` to include solution accessors.

        Warning

        This method is intended for internal use only.

###### `mass.core.units`¶

Unit and UnitDefinition implementation based on SBML specifications.

The `units` module contains the `Unit` and
`UnitDefinition` classes based on the implementation of units in
SBML.

Note that `mass` does not support automatic unit tracking to ensure
unit consistency. Therefore, it is incumbent upon the user to maintain unit
consistency as they use the various `mass` modules and functions.

To view valid units, use the `print_defined_unit_values()` function.
Please send a PR if you want to add something to the pre-built
`Unit` s.

###### Module Contents¶

###### Classes¶

|  |  |
| --- | --- |
| `Unit` | Manage units via this implementation of the SBML Unit specifications. |
| `UnitDefinition` | Manage units via implementation of SBML UnitDefinition specifications. |

###### Functions¶

|  |  |
| --- | --- |
| `print_defined_unit_values`(value=’Units’) | Print the pre-defined unit quantities in the `units` submodule. |

`mass.core.units.``SBML_BASE_UNIT_KINDS_DICT`[source]¶
:   Contains SBML base units and their `int` values.

    Type
    :   `DictWithID`

`mass.core.units.``SI_PREFIXES_DICT`[source]¶
:   Contains SI unit prefixes and scale values.

    Type
    :   `DictWithID`

*class* `mass.core.units.``Unit`(*kind*, *exponent*, *scale*, *multiplier*)[source]¶
:   Manage units via this implementation of the SBML Unit specifications.

    Parameters
    :   - **kind** (*str* *or* *int*) – A string representing the SBML Level 3 recognized base unit or its
          corresponding SBML integer value as defined in
          `SBML_BASE_UNIT_KINDS_DICT`.
        - **exponent** (*int*) – The unit exponent.
        - **scale** (*int* *or* *str*) – An integer representing the scale of the unit, or a string for one of
          the pre-defined SI scales in `SI_PREFIXES_DICT`.
        - **multiplier** (*float*) – A number used to multiply the unit by a real-numbered factor, enabling
          units that are not necessarily a power-of-ten multiple.

    *property* `kind`(*self*)[source]¶
    :   Return the unit kind of the `Unit`.

        Parameters
        :   **kind** (*str*) – An SBML recognized unit kind as a string.

    *property* `exponent`(*self*)[source]¶
    :   Return the exponent of the `Unit`.

        Parameters
        :   **exponent** (*int*) – The exponent of the unit as an integer.

    *property* `scale`(*self*)[source]¶
    :   Return the scale of the `Unit`.

        Parameters
        :   **scale** (*int* *or* *str*) – An integer representing the scale of the unit, or a
            string from the pre-defined SI prefixes. Not case sensitive.

    *property* `multiplier`(*self*)[source]¶
    :   Get or set the multiplier of the `Unit`.

        Parameters
        :   **multiplier** (*float*) – A numerical value representing a multiplier for the unit.

    `__str__`(*self*)[source]¶
    :   Override of default `str` implementation.

        Warning

        This method is intended for internal use only.

    `__repr__`(*self*)[source]¶
    :   Override of default `repr()` implementation.

        Warning

        This method is intended for internal use only.

`mass.core.units.``PREDEFINED_UNITS_DICT`[source]¶
:   Contains pre-built `Unit`s.

    Type
    :   `DictWithID`

*class* `mass.core.units.``UnitDefinition`(*id=None*, *name=''*, *list\_of\_units=None*)[source]¶
:   Bases: `cobra.core.object.Object`

    Manage units via implementation of SBML UnitDefinition specifications.

    Parameters
    :   - **id** (*str*) – The identifier to associate with the unit definition
        - **name** (*str*) – A human readable name for the unit definition.

    `list_of_units`¶
    :   A list containing `Unit`s that are needed to define the
        `UnitDefinition`, or a string that corresponds with the
        pre-defined units. Invalid units are ignored.

        Type
        :   list

    `create_unit`(*self*, *kind*, *exponent=1*, *scale=0*, *multiplier=1*)[source]¶
    :   Create a `Unit` and add it to the `UnitDefinition`.

        Parameters
        :   - **kind** (*str*) – A string representing the SBML Level 3 recognized base unit.
            - **exponent** (*int*) – The exponent on the unit. Default is `1`.
            - **scale** (*int* *or* *str*) – An integer representing the scale of the unit, or
              a string for one of the pre-defined scales. Default is `0.`
            - **multiplier** (*float*) – A number used to multiply the unit by a real-numbered factor,
              enabling units that are not necessarily a power-of-ten multiple.
              Default is `1.`

    `add_units`(*self*, *new\_units*)[source]¶
    :   Add `Unit`s to the `list_of_units`.

        Parameters
        :   **new\_units** (*list*) – A list of `Unit`s and the string identifiers of pre-built
            units to add to the `list_of_units`

    `remove_units`(*self*, *units\_to\_remove*)[source]¶
    :   Remove `Unit`s from the `list_of_units`.

        Parameters
        :   **units\_to\_remove** (*list*) – A list of `Unit`s and/or the string corresponding to the
            unit `Unit.kind` to remove from the `list_of_units`.

    `_units_to_alter`(*self*, *units*)[source]¶
    :   Create a set of units to alter in the unit definition.

        Warning

        This method is intended for internal use only.

    `__repr__`(*self*)[source]¶
    :   Override of default `repr()` implementation.

        Warning

        This method is intended for internal use only.

    `__iter__`(*self*)[source]¶
    :   Override of default `iter()` implementation.

        Warning

        This method is intended for internal use only.

`mass.core.units.``print_defined_unit_values`(*value='Units'*)[source]¶
:   Print the pre-defined unit quantities in the `units` submodule.

    Parameters
    :   **value** (*str*) –

        A string representing which pre-defined values to display.
        Must be one of the following:

        > - `"Scales"`
        > - `"BaseUnitKinds"`
        > - `"Units"`
        > - `"all"`

        Default is `"Units"` to display all pre-defined `Unit`s.

###### `mass.enzyme_modules`¶

###### Submodules¶

###### `mass.enzyme_modules.enzyme_module`¶

EnzymeModule is a class for handling reconstructions of enzymes.

The `EnzymeModule` is a reconstruction an enzyme’s mechanism and
behavior in a context of a larger system. To aid in the reconstruction process,
the `EnzymeModule` contains various methods to build and add associated
`EnzymeModuleForm` and `EnzymeModuleReaction`s
of the enzyme module (see `make_enzyme_module_forms()` and
`make_enzyme_module_reaction()` methods, respecitvely).

Given the wide variety of enzymes and the various interactions it can have
with ligands (e.g. catalyzation, inhibition, activation, etc.), the enzyme
module has the following “categorized dict” attributes:

> - `EnzymeModule.enzyme_module_ligands_categorized`
> - `EnzymeModule.enzyme_module_forms_categorized`
> - `EnzymeModule.enzyme_module_reactions_categorized`

These “categorized dict” attributes allow the user to define categories and
place various objects into one or more categories in the respective
“categorized dict” attribute (ligands a.k.a.
`MassMetabolite`s, `EnzymeModuleForm`, and
`EnzymeModuleReaction`s). Utilizing categories with these attributes
can help with the management of complex enzymes, and are preserved upon
merging an `EnzymeModule` into a `MassModel`.

Because the `EnzymeModule` is a subclass of the `MassModel`, it
can be merged with a `MassModel` representing the larger network in
which the enzyme is a part of. For best results, an `EnzymeModule`
should always be merged into the model as follows:

```
model.merge(enzyme_module, inplace=False)
# OR
new_model = model.merge(enzyme_module, inplace=True)
```

Once merged, the `EnzymeModuleForm` and
`EnzymeModuleReaction`s of the `EnzymeModule` are treated
like any other `MassMetabolite` or `MassReaction`.

Therefore, to prevent the loss of the enzyme specific information that was
stored in the `EnzymeModule`, the enzyme module is converted into an
ordered dictionary known as an `EnzymeModuleDict`, which contains
most of the enzyme specific attribute information. Note that all enzyme
specific attribute names start with either `"enzyme"` or `"enzyme_module"`.

During the model merging process, the `EnzymeModuleDict` is created,
then stored in the `MassModel.enzyme_modules` attribute for access at
a later time. See the `enzyme_module_dict` documentation for more
information about the `EnzymeModuleDict`.

###### Module Contents¶

###### Classes¶

|  |  |
| --- | --- |
| `EnzymeModule` | Class representation of an enzyme module reconstruction. |

*class* `mass.enzyme_modules.enzyme_module.``EnzymeModule`(*id\_or\_model=None*, *name=None*, *subsystem=''*, *array\_type='dense'*, *dtype=np.float64*)[source]¶
:   Bases: `mass.core.mass_model.MassModel`

    Class representation of an enzyme module reconstruction.

    Parameters
    :   - **id\_or\_model** (*str**,* *MassModel**,* *EnzymeModule*) – A string identifier to associate with the `EnzymeModule`, or an
          existing model object. If an existing model object is provided, a new
          `EnzymeModule` object is instantiated with the same properties
          as the original model.
        - **name** (*str*) – A human readable name for the model.
        - **subsystem** (*str*) – The subsystem in which the enzyme module is a part of.
        - **array\_type** (*str*) – A string identifiying the desired format for the returned matrix.
          Valid matrix types include `'dense'`, `'dok'`, `'lil'`,
          `'DataFrame'`, and `'symbolic'` Default is `'DataFrame'`.
          See the `matrix` module documentation for more information
          on the `array_type`.
        - **dtype** (*data-type*) – The desired array data-type for the stoichiometric matrix. If `None`
          then the data-type will default to `numpy.float64`.

    `enzyme_module_ligands`¶
    :   A `DictList` where the keys are the
        metabolite identifiers and the values are the associated
        `MassMetabolite`s.

        Type
        :   DictList

    `enzyme_module_forms`¶
    :   A `DictList` where the keys are the
        `EnzymeModuleForm` identifiers and the values are the
        associated `EnzymeModuleForm`.

        Type
        :   DictList

    `enzyme_module_reactions`¶
    :   A `DictList` where keys are the
        reaction identifiers and the values are the associated
        `EnzymeModuleReaction`s.

        Type
        :   DictList

    *property* `enzyme_total_symbol_str`(*self*)[source]¶
    :   Get the symbol as a string for the total enzyme concentration.

    *property* `enzyme_flux_symbol_str`(*self*)[source]¶
    :   Get the symbol as a string for the net flux through the enzyme.

    *property* `enzyme_concentration_total`(*self*)[source]¶
    :   Get or set the total concentration value.

        Notes

        The total concentration of the enzyme cannot be negative.

        Parameters
        :   **concentration** (*float*) – A non-negative number for the concentration of the enzyme.

        Raises
        :   **ValueError** – Occurs when trying to set a negative value.

    *property* `enzyme_rate`(*self*)[source]¶
    :   Get or set the flux through the enzyme.

        Parameters
        :   **value** (*float*) – The value of the net flux through the enzyme.

    *property* `enzyme_concentration_total_equation`(*self*)[source]¶
    :   Return the total concentration equation.

        Notes

        - Will sum the `EnzymeModuleForm` that have their
          `enzyme_module_id` match the
          `EnzymeModule.id`.
        - If no `EnzymeModuleForm` are found to have an
          `enzyme_module_id` that matches the
          `EnzymeModule.id`, all `EnzymeModuleForm`
          in the the model will be used.

        Returns
        :   A `sympy` expression of the sum of the
            `EnzymeModuleForm`.

        Return type
        :   Basic

    *property* `enzyme_rate_equation`(*self*)[source]¶
    :   Get or set the net flux rate equation of the enzyme.

        Parameters
        :   **equation** (*str**,* *Basic*) – Either a string representing the equationcthat will be sympified
            via the `sympify()` function., or a
            `sympy` expression representing the of the expression.

        Returns
        :   A `sympy` expression representing the net flux through the
            enzyme.

        Return type
        :   Basic

    *property* `enzyme_module_ligands_categorized`(*self*)[source]¶
    :   Get or set categories for ligands.

        Notes

        - A ligand must already exist in the `EnzymeModule` as a
          `MassMetabolite` in order to set its category.
        - If categories already exists, their existing contents are updated.
        - Setting an empty `list` for a category in the dict will cause that
          particular category group to be removed completely from the model.
        - Setting an empty `dict` will cause ALL category groups to be
          removed completely from the model.

        Parameters
        :   **value** (*Group* *or* *dict*) –

            Either a `cobra.Group` to add
            to the categorized ligands, or a `dict` where keys are strings
            representing categories for the ligands, and values are `lists`
            containing the corresponding `MassMetabolite`s or
            their identifiers.

            An empty `list` will remove the corresponding category from the
            model and attribute.

            An empty `dict` will remove all categories from the attribute
            and the model.

    *property* `enzyme_module_forms_categorized`(*self*)[source]¶
    :   Get or set categories for enzyme module forms.

        Notes

        - An enzyme module form must already exist in the
          `EnzymeModule` as an `EnzymeModuleForm` in order
          to set its category.
        - If categories already exists, their existing contents are updated.
        - Setting an empty `list` for a category in the dict will cause that
          particular category group to be removed completely from the model.
        - Setting an empty `dict` will cause ALL category groups to be
          removed completely from the model.

        Parameters
        :   **value** (*Group* *or* *dict*) –

            Either a `cobra.Group` to add
            to the categorized enzyme module forms, or a `dict` where keys
            are strings representing categories for the enzyme module forms,
            and values are `lists` containing the corresponding
            `EnzymeModuleForm`s or their identifiers.

            An empty `list` will remove the corresponding category from the
            model and attribute.

            An empty `dict` will remove all categories from the attribute
            and the model.

    *property* `enzyme_module_reactions_categorized`(*self*)[source]¶
    :   Get or set categories for enzyme module reactions.

        Notes

        - An enzyme module reaction must already exist in the
          `EnzymeModule` as an `EnzymeModuleReaction` in order
          to set its category.
        - If categories already exists, their existing contents are updated.
        - Setting an empty `list` for a category in the dict will cause that
          particular category group to be removed completely from the model.
        - Setting an empty `dict` will cause ALL category groups to be
          removed completely from the model.

        Parameters
        :   **value** (*Group* *or* *dict*) –

            Either a `cobra.Group` to add
            to the categorized enzyme module reaction, or a `dict` where keys
            are strings representing categories for the enzyme module
            reactions, and values are `lists` containing the corresponding
            `EnzymeModuleReactions`s or their identifiers.

            An empty `list` will remove the corresponding category from the
            model and attribute.

            An empty `dict` will remove all categories from the attribute
            and the model.

    `make_enzyme_module_form`(*self*, *id=None*, *name='automatic'*, *categories=None*, *bound\_metabolites=None*, *compartment=None*)[source]¶
    :   Create and add an `EnzymeModuleForm` to the module.

        Notes

        - Upon creation, the `EnzymeModuleForm` is added to the
          `EnzymeModule`.
        - If `MassMetabolite`s in the `bound_metabolites`
          argument do not already exist in the `EnzymeModule`,
          they will also be added.

        Parameters
        :   - **id** (*str*) – A string identifier to associate with the enzymatic forms.
            - **name** (*str*) – Either a human readable name for the enzyme module forms, or the
              string `"automatic"`. If set to `"automatic"`, a name will be
              generated based on the identifier of the enzyme module forms and
              its bound ligands.
            - **categories** (*str* *or* *list*) – A string representing the category, or a list of strings
              containing several categories for the enzyme module forms.
            - **bound\_metabolites** (*dict*) – A `dict` representing the ligands bound to the enzyme,
              with `MassMetabolite`s or their identifiers as
              keys and the number bound as values.
            - **compartment** (*str*) – The compartment where the enzyme module forms is located.

        Returns
        :   The newly created `EnzymeModuleForm`.

        Return type
        :   EnzymeModuleForm

        See also

        `EnzymeModuleForm.generate_enzyme_module_forms_name()`
        :   Automatic generation of the `name` for an `EnzymeModuleForm`.

    `make_enzyme_module_reaction`(*self*, *id=None*, *name=''*, *subsystem=None*, *reversible=True*, *categories=None*, *metabolites\_to\_add=None*)[source]¶
    :   Create and add an `EnzymeModuleReaction` to the module.

        Notes

        - When adding metabolites, a final coefficient of < 0 implies a
          reactant and a final coefficient of > 0 implies a product.

        Parameters
        :   - **id** (*str*) – The identifier associated with the enzyme module reaction.
            - **name** (*str*) – A human readable name for the enzyme module reaction. If name
              is set to match `"automatic"`, a name will be generated based
              on the `EnzymeModuleForm` and their bound ligands.
            - **subsystem** (*str*) – The subsystem where the reaction is meant to occur.
            - **reversible** (*bool*) – The kinetic reversibility of the reaction. Irreversible reactions
              have an equilibrium constant and a reverse rate constant as set
              in the `irreversible_Keq` and
              `irreversible_kr` attributes of the
              `MassConfiguration`. Default is `True`.
            - **categories** (*str* *or* *list*) – A string representing the category, or a list of strings
              containing several categories for the enzyme module reactions.
            - **metabolites\_to\_add** (*dict*) – A `dict` with `MassMetabolite`s and
              `EnzymeModuleForm` or their identifiers as keys and
              stoichiometric coefficients as values. If keys are string
              identifiers then the `MassMetabolite`s and
              `EnzymeModuleForm` must already be a part of model.

        Returns
        :   The newly created `EnzymeModuleReaction`.

        Return type
        :   EnzymeModuleReaction

        See also

        `EnzymeModuleReaction.generate_enzyme_module_reaction_name()`
        :   Automatic generation of the `name` for an `EnzymeModuleReaction`.

    `unify_rate_parameters`(*self*, *reaction\_list*, *new\_parameter\_id*, *rate\_type=1*, *enzyme\_prefix=False*)[source]¶
    :   Unify rate law parameters for a list of enzyme module reactions.

        After unification, the new parameters and rate laws are placed into the
        `custom_parameters` and `custom_rates` attributes, repsectively.

        Parameters
        :   - **reaction\_list** (*list*) – A `list` containing `EnzymeModuleReaction`s or
              their string identifiers. `EnzymeModuleReaction`s must
              already exist in the `EnzymeModule`.
            - **new\_parameter\_id** (*str*) – The new parameter ID to use for the unified reaction parameters.
              The forward rate, reverse rate, and/or equilibrium constants in
              the current rate law will have the reaction ID component replaced
              with the `new_parameter_id` in the parameter ID.
            - **rate\_type** (*int*) –

              The type of rate law to utilize in unification. Must be 1, 2, or 3.

              > - Type 1 will utilize the `forward_rate_constant` and the
              >   `equilibrium_constant`.
              > - Type 2 will utilize the `forward_rate_constant` and the
              >   `reverse_rate_constant`.
              > - Type 3 will utilize the `equilibrium_constant` and the
              >   `reverse_rate_constant`.

              Default is `1`.
            - **enzyme\_prefix** (*bool*) – If `True`, add the `EnzymeModule` ID as a prefix to the
              `new_parameter_id` before using the `new_parameter_id` in
              the rate parameter unification. Default is `False`.

    `make_enzyme_rate_equation`(*self*, *enzyme\_module\_reactions*, *use\_rates=False*, *update\_enzyme=False*)[source]¶
    :   Create an equation representing the net flux through the enzyme.

        The left side of the rate equation will always be the flux symbol of
        the enzyme, accessible via `enzyme_flux_symbol_str`.

        Parameters
        :   - **enzyme\_module\_reactions** (*list*) – A list containing the `EnzymeModuleReaction`s or their
              identifiers to be summed for the equation.
            - **use\_rates** (*bool*) – If `True`, then the rates of the provided reactions are used in
              creating the expression. Otherwise the arguments in the expression
              are left as the `EnzymeModuleReaction.flux_symbol_str`s.
            - **update\_enzyme** (*bool*) – If `True`, update the `enzyme_rate_equation` attribute
              and, if necessary, the `enzyme_module_reactions` attribute
              of the module in addition to returning the generated equation.
              Otherwise just return the net flux equation without making any
              updates. Default is `False`.

        Returns
        :   A `sympy` expression of the net flux equation.

        Return type
        :   Basic

    `sum_enzyme_module_form_concentrations`(*self*, *enzyme\_module\_forms*, *use\_values=False*)[source]¶
    :   Sum the forms concentrations for a list of enzyme module forms.

        Parameters
        :   - **enzyme\_module\_forms** (*list*) – A `list` containing the `EnzymeModuleForm` or their
              identifiers to be summed. Forms must already exist in the
              `EnzymeModule`.
            - **use\_values** (*bool*) – If `True`, then numerical values are substituted into the
              expression. Otherwise arguments in the expression are left as
              `sympy` symbols.

        Returns
        :   The sum of the concentrations for the given enzyme module forms
            as a `float` if `use_values` is `True` and all values are
            present. Otherwise returns a `sympy` expression representing
            the sum of the given enzyme module forms.

        Return type
        :   float or Basic

    `sum_enzyme_module_reaction_fluxes`(*self*, *enzyme\_module\_reactions*, *use\_values=False*)[source]¶
    :   Sum the enzyme reaction steady state fluxes for a list of reactions.

        Parameters
        :   - **enzyme\_module\_reactions** (*list*) – A `list` a containing the `EnzymeModuleReaction` or
              their identifiers to be summed. Reactions must already exist in
              the module and must be considered an enzyme module reaction.
            - **use\_values** (*bool*) – If `True`, then numerical values are substituted into the
              expression. Otherwise arguments in the expression are left as
              `sympy` symbols.

        Returns
        :   The sum of the steady state fluxes for the given enzyme reaction
            as a `float` if `use_values` is `True` and all values are
            present. Otherwise returns a `sympy` expression representing
            the sum of the enzyme module reaction fluxes.

        Return type
        :   float or Basic

    `enzyme_concentration_total_error`(*self*, *use\_values=False*)[source]¶
    :   Return the error for the total enzyme concentrations.

        The error of the total enzyme concentration is defined to be the
        difference between the `enzyme_concentration_total` value and
        the sum of all `EnzymeModuleForm` initial conditions
        in the model.

        Notes

        Positive values indicate the value in the
        `enzyme_concentration_total` attribute is greater than the value
        calculated using the expression from the
        `enzyme_concentration_total_equation` attribute.

        Parameters
        :   **use\_values** (*bool*) – If `True`, then numerical values are substituted into the
            expression. Otherwise arguments in the expression are left as
            `sympy` symbols.

        Returns
        :   The error between the set `enzyme_concentration_total` and
            the sum of the `EnzymeModuleForm` initial condition
            values in the model as a `float` if `use_values` is `True`
            and all values are present. Otherwise returns a `sympy`
            expression representing the error.

        Return type
        :   float or Basic

    `enzyme_rate_error`(*self*, *use\_values=False*)[source]¶
    :   Return the error for the net flux through the enzyme.

        The error of the enzyme net flux is defined to be the difference
        between the `enzyme_rate` value and the
        calculated value for the `enzyme_rate_equation`.

        Notes

        Positive values indicate the value in `enzyme_rate`
        attribute is greater than the value calculated using the expression
        from the `enzyme_rate_equation` attribute.

        Parameters
        :   **use\_values** (*bool*) – If `True`, then numerical values are substituted into the
            expression. Otherwise arguments in the expression are left as
            `sympy` symbols.

        Returns
        :   The error between the set `enzyme_rate` and the
            calculated value for the `enzyme_rate_equation`
            attribute as a `float` if `use_values` is `True` and all
            values are present in the model. Otherwise returns
            a `sympy` expression representing the error.

        Return type
        :   float or Basic

    `make_enzyme_fraction`(*self*, *categorized\_attr*, *top*, *bottom*, *use\_values=False*)[source]¶
    :   Make the expression for a ratio of categorized enzyme objects.

        Notes

        The string `"Equation"` can be passed to either `top` or
        `bottom` to utilize the equation in the corresponding attribute
        (i.e. `enzyme_concentration_total_equation` for `'forms'`
        and `enzyme_rate_equation` for `'reactions'`).

        Parameters
        :   - **categorized\_attr** (*str*) – Either a string representing which categorized attribute
              to use or the attribute itself to use in making the
              enzyme ratio expression. Use the string `'forms'` for
              `enzyme_module_forms_categorized`, or `'reactions'` for
              `enzyme_module_reactions_categorized`.
            - **top** (*str*) – A string representing a category in the categorized attribute.
              The summation expression of
              the objects in the corresponding list is used as the top
              (numerator) of the fraction to be made. Alternatively, the
              string `"Equation"` can be provided to utilize an
              equation attribute.
            - **bottom** (*str*) – A string representing a category in the categorized attribute.
              The summation expression of the objects in the corresponding list
              is used as the bottom (denominator) of the fraction to be made.
              Alternatively, the string `"Equation"` can be provided to
              utilize an equation attribute.
            - **use\_values** (*bool*) – If `True`, then numerical values are substituted into the
              expression. Otherwise arguments in the expression are left as
              `sympy` symbols.

        Returns
        :   The fraction either calculated and returned as float if
            `use_values` is `True` and all values are present in the
            model, or a `sympy` expression representing the formula
            for the fraction.

        Return type
        :   float or Basic

    `add_metabolites`(*self*, *metabolite\_list*)[source]¶
    :   Add a list of metabolites and enzyme forms to the module.

        The change is reverted upon exit when using the `EnzymeModule`
        as a context.

        Notes

        Extends from `MassModel.add_metabolites()`.

        Parameters
        :   - **metabolite\_list** (*list*) – A list of `MassMetabolite`s and
              `EnzymeModuleForm` to add to the `EnzymeModule`.
            - **add\_initial\_conditons** (*bool*) – If `True`, the initial conditions associated with the species
              are also added to the model. Otherwise, the species are added
              without their initial conditions.

    `remove_metabolites`(*self*, *metabolite\_list*, *destructive=False*)[source]¶
    :   Remove a list of metabolites and enzyme forms from the module.

        The species’ initial conditions will also be removed from the model.

        The change is reverted upon exit when using the `EnzymeModule`
        as a context.

        Notes

        Extends from `MassModel.remove_metabolites()`.

        Parameters
        :   - **metabolite\_list** (*list*) – A list of `MassMetabolite`s and
              `EnzymeModuleForm` to remove from the
              `EnzymeModule`.
            - **destructive** (*bool*) – If `False`, the species are removed from all associated
              `EnzymeModuleReaction`s . If `True`, also remove
              associated `EnzymeModuleReaction`s from the
              `EnzymeModule`.

    `add_reactions`(*self*, *reaction\_list*)[source]¶
    :   Add a list of reactions to the `EnzymeModule`.

        `MassReaction`s and `EnzymeModuleReaction`s
        with identifiers identical to an existing reaction are ignored.

        The change is reverted upon exit when using the `EnzymeModule`
        as a context.

        Notes

        Extends from `MassModel.add_reactions()`.

        Parameters
        :   **reaction\_list** (*list*) – A list of `MassReaction` and
            `EnzymeModuleReaction` to add.

    `remove_reactions`(*self*, *reactions*, *remove\_orphans=False*)[source]¶
    :   Remove reactions from the `EnzymeModule`.

        The change is reverted upon exit when using the `EnzymeModule`
        as a context.

        Notes

        Extends from `MassModel.remove_reactions()`.

        Parameters
        :   - **reactions** (*list*) – A list of `MassReaction` and
              `EnzymeModuleReaction` to remove from the
              `EnzymeModule`.
            - **remove\_orphans** (*bool*) – If `True`, will also remove orphaned genes,
              `MassMetabolite`s, and `EnzymeModuleForm`
              from the `EnzymeModule`.

    `repair`(*self*, *rebuild\_index=True*, *rebuild\_relationships=True*)[source]¶
    :   Update all indicies and pointers in the model.

        In addition to updating indicies and pointers, the
        `enzyme_module_reactions` attribute will be updated to
        ensure it contains all existing reactions involving
        `EnzymeModuleForm`.

        Notes

        Extends from `MassModel.repair()`.

        Parameters
        :   - **rebuild\_index** (*bool*) – If `True`, then rebuild the indicies kept in the reactions,
              metabolites, and genes.
            - **rebuild\_relationships** (*bool*) – If `True`, then reset all associations between the reactions,
              metabolites, genes, and the model, and rebuilds them.

    `copy`(*self*)[source]¶
    :   Create a partial “deepcopy” of the EnzymeModule.

        All of the `MassMetabolite`s, `MassReaction`s,
        `Gene`s, `EnzymeModuleForm`,
        `EnzymeModuleReaction`s, and `EnzymeModuleDict`s,
        the boundary conditions, custom rates, custom parameters, and the
        stoichiometric matrix are created anew, but in a faster fashion than
        `deepcopy`.

        Notes

        - Overrides `MassModel.copy()` in order to exclude more items
          to not copy by ref.

    `merge`(*self*, *right*, *prefix\_existing=None*, *inplace=True*, *objective='left'*)[source]¶
    :   Merge two models into one model with the objects from both.

        The reactions, metabolites, genes, enzyme modules, boundary conditions,
        custom rate expressions, rate parameters, compartments, units, notes,
        and annotations from the right model are also copied to left model.
        However, note that in cases where identifiers for objects are identical
        or a dict item has an identical key(s), priority will be given to what
        already exists in the left model.

        Notes

        - When merging an `EnzymeModule` into a `MassModel`,
          the enzyme module is converted to an `EnzymeModuleDict` and
          stored in a `DictList` accessible via the
          `enzyme_modules` attribute. If an `EnzymeModuleDict`
          already exists in the model, it will be replaced.
        - If an `EnzymeModule` already exists in the model, it will
          be replaced.
        - When merging an `EnzymeModule` with another
          `EnzymeModule`, a new `EnzymeModule` will be returned,
          where the EnzymeModule is a copy of the ‘left’ model (`self`)
          with the `'right'` model is contained within.
        - Overrides `MassModel.merge()`.

        Parameters
        :   - **right** (*MassModel*) – The model to merge into the left model. If a `MassModel`
              then the first model refers to the `right` model and the second
              model refers to the `left` model. Otherwise the first model
              refers to the `left` model and the second model refers to the
              `right` model.
            - **prefix\_existing** (*str*) – If provided, the string is used to prefix the reaction identifier
              of a reaction in the second model if that reaction already exists
              within the first model. Will also apply prefix to identifiers
              of enzyme modules in the second model.
            - **inplace** (*bool*) – If `True` then add reactions from second model directly to the
              first model. Otherwise, create a new model leaving the first model
              untouched. When done within the model as context, changes to the
              models are reverted upon exit.
            - **objective** (*str*) – One of `"left"`, `"right"` or `"sum"` for setting the
              objective of the resulting model to that of the corresponding
              model or the sum of both. Default is `"left"`. Note that when
              merging a `MassModel` with an `EnzymeModule`,
              `"left"` will refer to the `MassModel`.

        Returns
        :   A new `MassModel` or `EnzymeModule`
            representing the merged model.

        Return type
        :   MassModel or EnzymeModule

    `_set_category_attribute`(*self*, *item*, *attr*, *to\_filter*)[source]¶
    :   Set the categorized attribute after ensuring it is valid.

        Warning

        This method is intended for internal use only.

    `_set_enzyme_object_category`(*self*, *attr*, *category*, *object\_list*)[source]¶
    :   Add a list of objects to a new or existing category.

        Notes

        - If a category already exists, the objects will be added to the
          corresponding `cobra.Group`.
        - The objects to be categorized must already exist in the
          `EnzymeModule`.
        - An empty `object_list` will cause the group representing the
          category to be removed.

        Parameters
        :   - **category** (*str*) – A string representing the category for the list of objects to be
              categorized.
            - **object\_list** (*list*) –

              A `list` containing the objects to be categorized.
              The `list` must contain ONLY one of the following `mass`
              object types:

              > - `MassMetabolite`s representing enzyme ligands.
              > - `EnzymeModuleForm`s representing enzymatic
              >   forms.
              > - `EnzymeModuleReaction`s representing enzymatic
              >   binding reactions ligands.

    `_update_object_pointers`(*self*)[source]¶
    :   Update objects in the attributes to be the objects from the model.

        Warning

        This method is intended for internal use only.

    `_get_current_enzyme_module_objs`(*self*, *attr*, *update\_enzyme=True*)[source]¶
    :   Get the enzyme module objects for ‘attr’ that exist in the model.

        Parameters
        :   - **attr** (*str {'ligands'**,* *'forms'**,* *'reactions'}*) – A string representing which attribute to update.
            - **update\_enzyme** (*bool*) – If True, update the enzyme\_module\_reactions attribute of the
              EnzymeModule.

        Warning

        This method is intended for internal use only.

    `_make_summation_expr`(*self*, *items*, *object\_type*)[source]¶
    :   Create a sympy expression of the summation of the given items.

        Warning

        This method is intended for internal use only.

    `_sub_values_into_expr`(*self*, *expr*, *object\_type*, *additional=None*)[source]¶
    :   Substitute values into an expression and try to return a float.

        Warning

        This method is intended for internal use only.

    `_add_self_to_model`(*self*, *model*, *prefix\_existing*, *inplace*, *objective*)[source]¶
    :   Add self to the model and return the MassModel object.

        Warning

        This method is intended for internal use only.

    `_repr_html_`(*self*)[source]¶
    :   HTML representation of the overview for the EnzymeModule.

        Warning

        This method is intended for internal use only.

###### `mass.enzyme_modules.enzyme_module_dict`¶

EnzymeModuleDict is a class representing the EnzymeModule after merging.

This object is intended to represent the `EnzymeModule` once merged
into a `MassModel` in order to retain `EnzymeModule` specific
attributes of the `EnzymeModule` without the need of storing the
`EnzymeModule` object itself.

When merging an `EnzymeModule` into another model, the
`EnzymeModule` is converted into an `EnzymeModuleDict`, allowing
for most of the enzyme specific attribute information of the
`EnzymeModule` to be preserved during the merging process, and accessed
after the merging. All keys of the `EnzymeModuleDict` can be used as
attribute accessors. Additionally `EnzymeModuleDict` is a subclass of
an `OrderedDictWithID` which in turn is a subclass of an
`OrderedDict`, thereby inheriting its methods and behavior.

The `EnzymeModule` attributes preserved in the
`EnzymeModuleDict` are the following:

> - `EnzymeModule.id`
> - `EnzymeModule.name`
> - `EnzymeModule.subsystem`
> - `EnzymeModule.enzyme_module_ligands`
> - `EnzymeModule.enzyme_module_forms`
> - `EnzymeModule.enzyme_module_reactions`
> - `EnzymeModule.enzyme_module_ligands_categorized`
> - `EnzymeModule.enzyme_module_forms_categorized`
> - `EnzymeModule.enzyme_module_reactions_categorized`
> - `EnzymeModule.enzyme_concentration_total`
> - `EnzymeModule.enzyme_rate`
> - `EnzymeModule.enzyme_concentration_total_equation`
> - `EnzymeModule.enzyme_rate_equation`
> - `EnzymeModule.S`

If one of the above attributes has not been set, it will be added to the
`EnzymeModuleDict` as its default value. This means that the above
attributes can *always* be found in an `EnzymeModuleDict`.

Note that this class is not intended to be used for construction of an
`EnzymeModule`, but rather a representation of one after construction.
See the `enzyme_module` documentation for more information on
constructing `EnzymeModule`s.

###### Module Contents¶

###### Classes¶

|  |  |
| --- | --- |
| `EnzymeModuleDict` | Container to store `EnzymeModule` information after merging. |

*class* `mass.enzyme_modules.enzyme_module_dict.``EnzymeModuleDict`(*id\_or\_enzyme=None*)[source]¶
:   Bases: `mass.util.dict_with_id.OrderedDictWithID`

    Container to store `EnzymeModule` information after merging.

    Parameters
    :   **enzyme** (*EnzymeModule* *or* *EnzymeModuleDict*) – The `EnzymeModule` to be converted into an
        `EnzymeModuleDict`, or an existing `EnzymeModuleDict`.
        If an existing `EnzymeModuleDict` is provided, a new
        `EnzymeModuleDict` is instantiated with the same information
        as the original.

    `copy`(*self*)[source]¶
    :   Copy an `EnzymeModuleDict`.

    `_set_missing_to_defaults`(*self*)[source]¶
    :   Set all of the missing attributes to their default values.

        Warning

        This method is intended for internal use only.

    `_update_object_pointers`(*self*, *model=None*)[source]¶
    :   Update objects in the EnzymeModuleDict to point to the given model.

        Warning

        This method is intended for internal use only.

    `_make_enzyme_stoichiometric_matrix`(*self*, *update=False*)[source]¶
    :   Return the S matrix based on enzyme forms, reactions, and ligands.

        Warning

        This method is intended for internal use only.

    `_fix_order`(*self*)[source]¶
    :   Fix the order of the items in the EnzymeModuleDict.

        Warning

        This method is intended for internal use only.

    `_repr_html_`(*self*)[source]¶
    :   HTML representation of the overview for the EnzymeModuleDict.

        Warning

        This method is intended for internal use only.

    `__getattr__`(*self*, *name*)[source]¶
    :   Override of default `getattr` implementation.

        Warning

        This method is intended for internal use only.

    `__setattr__`(*self*, *name*, *value*)[source]¶
    :   Override of default `setattr` implementation.

        Warning

        This method is intended for internal use only.

    `__delattr__`(*self*, *name*)[source]¶
    :   Override of default `delattr` implementation.

        Warning

        This method is intended for internal use only.

    `__dir__`(*self*)[source]¶
    :   Override of default `dir` implementation to include the keys.

        Warning

        This method is intended for internal use only.

    `__copy__`(*self*)[source]¶
    :   Create a copy of the EnzymeModuleDict.

        Warning

        This method is intended for internal use only.

    `__deepcopy__`(*self*, *memo*)[source]¶
    :   Create a deepcopy of the EnzymeModuleDict.

        Warning

        This method is intended for internal use only.

`mass.enzyme_modules.enzyme_module_dict.``_ORDERED_ENZYMEMODULE_DICT_DEFAULTS`[source]¶

###### `mass.enzyme_modules.enzyme_module_form`¶

EnzymeModuleForm is a class for holding information regarding enzyme module forms.

The `EnzymeModuleForm` class inherits and extends the
`MassMetabolite` class. It is designed to represent various bound
states and conformations of the enzymes represented through the
`EnzymeModule` class.

The enzyme specific attributes on the `EnzymeModuleForm` are the
following:

> - `enzyme_module_id`
> - `"bound_metabolites"`

The `EnzymeModuleForm` contains the attribute
`MassMetabolite`(s) that could be bound to the sites on the enzyme.

Some other important points about the `EnzymeModuleForm` include:

> - If the `name` attribute is not set upon initializing, it is
>   automatically generated using the enzyme specific attributes.
> - If the `formula` or charge attributes are not set upon
>   initialization, it is inferred using the formulas and charges set on the
>   `MassMetabolite`(s) found in
>   `bound_metabolites`. A moiety is also included
>   for the formula using the `enzyme_module_id`.
> - The purpose of the generated formula and charge is to ensure reactions
>   remained mass and charge balanaced as metabolite species are bound and
>   altered by the `EnzymeModuleReaction`s of the
>   `EnzymeModule`.

###### Module Contents¶

###### Classes¶

|  |  |
| --- | --- |
| `EnzymeModuleForm` | Class representing an enzyme forms of an `EnzymeModule`. |

*class* `mass.enzyme_modules.enzyme_module_form.``EnzymeModuleForm`(*id\_or\_specie=None*, *enzyme\_module\_id=''*, *bound\_metabolites=None*, *\*\*kwargs*)[source]¶
:   Bases: `mass.core.mass_metabolite.MassMetabolite`

    Class representing an enzyme forms of an `EnzymeModule`.

    Accepted `kwargs` are passed to the initialization method of the base
    class, `MassMetabolite`.

    Parameters
    :   - **id\_or\_specie** (*str**,* *MassMetabolite**,* *EnzymeModuleForm*) – A string identifier to associate with the enzyme module forms, or an
          existing metabolite object. If an existing metabolite object is
          provided, a new `EnzymeModuleForm` is instantiated with the
          same properties as the original metabolite.
        - **enzyme\_module\_id** (*str*) – The identifier of the associated `EnzymeModule`.
        - **bound\_metabolites** (*dict*) – A `dict` representing the ligands bound to the enzyme,
          with `MassMetabolite`s or their identifiers as
          keys and the number bound as values.
        - **\*\*kwargs** –

          name :
          :   `str` representing a human readable name for the enzyme module
              form.

          formula :
          :   `str` representing a chemical formula associated with the
              enzyme module form.

          charge :
          :   `float` representing the charge number associated with the
              enzyme module form.

          compartment :
          :   `str` representing the compartment where the enzyme module
              form is located.

          fixed :
          :   `bool` indicating whether the enzyme module form
              concentration should remain at a fixed value. Default is `False`.

    *property* `bound_metabolites`(*self*)[source]¶
    :   Get or set metabolites bound to the enzyme’s site(s).

        Notes

        Assigning a `dict` to this property updates the current `dict` of
        ligands bound at the enzyme site(s) with the new values.

        Parameters
        :   **value** (*dict*) – A `dict` where keys are `MassMetabolite` and values
            are the number currently bound to the site(s).
            An empty `dict` will reset the bound ligands.

    `generate_enzyme_module_form_name`(*self*, *update\_enzyme=False*)[source]¶
    :   Generate name for the enzyme module form based on bound ligands.

        Notes

        - The `bound_metabolites` attribute is used
          in generating the name.
        - If the `enzyme_module_id` attributes are
          not set, the string `'Enzyme'` will be used in its place.

        Parameters
        :   **update\_enzyme** (*bool*) – If `True`, update the `name` attribute of the enzyme
            module form in addition to returning the generated name.
            Default is `False`.

        Returns
        :   String representing the name of the `EnzymeModuleForm`.

        Return type
        :   str

    `generate_form_formula`(*self*, *update\_enzyme=False*)[source]¶
    :   Generate the chemical formula for the enzyme module form.

        This function is primarily utilized for keeping reactions between
        `EnzymeModuleForm` mass and charge balanced.

        Notes

        The `bound_metabolites` attribute is used
        in generating the formula.

        Parameters
        :   **update\_enzyme** (*bool*) – If `True`, update the `formula` attribute of the enzyme
            module form in addition to returning the generated formula.
            Default is `False`.

        Returns
        :   String representing the formula of the
            `EnzymeModuleForm`.

        Return type
        :   str

    `generate_form_charge`(*self*, *update\_enzyme=False*)[source]¶
    :   Generate the charge for the enzyme module form.

        This function is primarily utilized for keeping reactions between
        `EnzymeModuleForm` mass and charge balanced.

        Notes

        The `bound_metabolites` attribute is used
        in generating the charge.

        Parameters
        :   **update\_enzyme** (*bool*) – If `True`, update the `charge` attribute of the enzyme
            module form in addition to returning the generated charge.
            Default is `False`.

        Returns
        :   Value representing the charge of the `EnzymeModuleForm`.

        Return type
        :   float

    `_set_id_with_model`(*self*, *value*)[source]¶
    :   Set the id of the EnzymeModuleForm to the associated MassModel.

        Warning

        This method is intended for internal use only.

    `_repair_bound_obj_pointers`(*self*)[source]¶
    :   Repair object pointer for metabolites in bound dict attributes.

        Requires a model to be associated with the EnzymeModuleForm.

        Warning

        This method is intended for internal use only.

    `_repr_html_`(*self*)[source]¶
    :   HTML representation of the overview for the EnzymeModuleForm.

        Warning

        This method is intended for internal use only.

###### `mass.enzyme_modules.enzyme_module_reaction`¶

EnzymeModuleReaction is a class for holding information regarding enzyme module reactions.

The `EnzymeModuleReaction` class inherits and extends the
`MassReaction` class. It is designed to represent the reactions
and transitions involving `EnzymeModuleForm`s represented in the
`EnzymeModule` class.

The enzyme specific attributes on the `EnzymeModuleReaction` are the
following:

> - `enzyme_module_id`

Some other important points about the `EnzymeModuleReaction` include:

> - If the `name` attribute is not set upon initializing, it is
>   automatically generated using the enzyme specific attributes of the
>   associated `EnzymeModuleForm`s.
> - Even though `MassReaction`s are also catalyzed by enzymes, an
>   enzyme module reaction in the context of this module will refer to
>   reactions that involve `EnzymeModuleForm`(s) and are
>   associated with an `EnzymeModule`.

###### Module Contents¶

###### Classes¶

|  |  |
| --- | --- |
| `EnzymeModuleReaction` | Class representing an enzyme reaction in an `EnzymeModule`. |

*class* `mass.enzyme_modules.enzyme_module_reaction.``EnzymeModuleReaction`(*id\_or\_reaction=None*, *enzyme\_module\_id=''*, *\*\*kwargs*)[source]¶
:   Bases: `mass.core.mass_reaction.MassReaction`

    Class representing an enzyme reaction in an `EnzymeModule`.

    Accepted `kwargs` are passed to the initialization method of the base
    class, `MassReaction`.

    Parameters
    :   - **id\_or\_reaction** (*str**,* *MassReaction**,* *EnzymeModuleReaction*) – A string identifier to associate with the enzyme module reaction,
          or an existing reaction object. If an existing reaction object is
          provided, a new `EnzymeModuleReaction` is instantiated with the
          same properties as the original reaction.
        - **enzyme\_module\_id** (*str*) – The identifier of the associated `EnzymeModule`.
        - **\*\*kwargs** –

          name :
          :   `str` representing a human readable name for the enzyme
              module reaction.

          subsystem :
          :   `str` representing the subsystem where the enzyme
              module reaction is meant to occur.

          reversible :
          :   `bool` indicating the the kinetic reversibility of the reaction.
              Irreversible reactions have an equilibrium constant and a
              reverse rate constant as set in the
              `irreversible_Keq` and
              `irreversible_kr` attributes of the
              `MassConfiguration`. Default is `True`.

          steady\_state\_flux :
          :   `float` representing the stored (typically steady state) flux
              for the reaction.

    `generate_enzyme_module_reaction_name`(*self*, *update\_enzyme=False*)[source]¶
    :   Generate name for an enzyme module reaction based on bound ligands.

        Notes

        - The `bound_metabolites` attribute of the
          associated `EnzymeModuleForm` is used in generating
          the name.

        Parameters
        :   **update\_enzyme** (*bool*) – If `True`, update the `name` attribute of the enzyme
            module reaction in addition to returning the generated name.
            Default is `False`.

        Returns
        :   String representing the name of the `EnzymeModuleReaction`.

        Return type
        :   str

###### `mass.io`¶

###### Submodules¶

###### `mass.io.dict`¶

Module to convert or create `mass` objects into or from dictionaries.

Converting objects into dictionaries allow for the exportation of
`MassModel`s in various formats. These formats include:

> - JSON format using the functions in
>   `json`.

###### Module Contents¶

###### Functions¶

|  |  |
| --- | --- |
| `model_to_dict`(model, sort=False) | Convert a `MassModel` into a serializable dictionary. |
| `model_from_dict`(obj) | Create a `MassModel` from a dictionary. |
| `metabolite_to_dict`(metabolite) | Convert a `MassMetabolite` into a serializable dictionary. |
| `metabolite_from_dict`(metabolite) | Create a `MassMetabolite` from a dictionary. |
| `reaction_to_dict`(reaction) | Convert a `MassReaction` into a serializable dictionary. |
| `reaction_from_dict`(reaction, model) | Create a `MassReaction` from a dictionary. |
| `enzyme_to_dict`(enzyme) | Convert an `EnzymeModuleDict` into a serializable dictionary. |
| `enzyme_from_dict`(enzyme, model) | Create an `EnzymeModuleDict` from a dictionary. |
| `unit_to_dict`(unit\_definition) | Convert an `UnitDefintion` into a serializable dictionary. |
| `unit_from_dict`(unit\_definition) | Create an `UnitDefintion` from a dictionary. |

`mass.io.dict.``model_to_dict`(*model*, *sort=False*)[source]¶
:   Convert a `MassModel` into a serializable dictionary.

    Parameters
    :   - **model** (*MassModel* *or* *EnzymeModule*) – The model to represent as a dictionary.
        - **sort** (*bool*) – Whether to sort the metabolites, reactions, genes, and enzyme modules
          or maintain the order defined in the model. If the model is an
          `EnzymeModule`, the
          `enzyme_module_ligands`,
          `enzyme_module_forms`, and
          `enzyme_module_reactions` attributes are also
          included. Default is `False`.

    Returns
    :   A dictionary with elements corresponding to the model attributes as
        which are in turn lists containing dictionaries holding all attribute
        information to form the corresponding object.

    Return type
    :   OrderedDict

    See also

    `model_from_dict()`

`mass.io.dict.``model_from_dict`(*obj*)[source]¶
:   Create a `MassModel` from a dictionary.

    Notes

    The `enzyme_module_ligands`,
    `enzyme_module_forms`, and
    `enzyme_module_reactions` attributes are used to
    determine whether the model should be initialized as an
    `EnzymeModule` or as a `MassModel`. At least one of these
    three attributes must be present in order for an `EnzymeModule`
    to be created.

    Parameters
    :   **obj** (*dict*) – A dictionary with elements corresponding to the model attributes as
        which are in turn lists containing dictionaries holding all attribute
        information to form the corresponding object.

    Returns
    :   The generated model or enzyme module.

    Return type
    :   MassModel or EnzymeModule

    See also

    `model_to_dict()`

`mass.io.dict.``metabolite_to_dict`(*metabolite*)[source]¶
:   Convert a `MassMetabolite` into a serializable dictionary.

    Parameters
    :   **metabolite** (*MassMetabolite*) – The metabolite to represent as a dictionary.

    Returns
    :   A dictionary with elements corresponding to metabolite attributes.

    Return type
    :   OrderedDict

    See also

    `metabolite_from_dict()`

`mass.io.dict.``metabolite_from_dict`(*metabolite*)[source]¶
:   Create a `MassMetabolite` from a dictionary.

    Notes

    The presence of the `enzyme_module_id`
    attribute is used to determine whether the dictionary should be
    initialized as an `EnzymeModuleForm` or as a
    `MassMetabolite`.

    Parameters
    :   **metabolite** (*dict*) – A dictionary with elements corresponding to the metabolite attributes.

    Returns
    :   The generated metabolite.

    Return type
    :   MassMetabolite or EnzymeModuleForm

    See also

    `metabolite_to_dict()`

`mass.io.dict.``reaction_to_dict`(*reaction*)[source]¶
:   Convert a `MassReaction` into a serializable dictionary.

    Parameters
    :   **reaction** (*MassReaction*) – The reaction to represent as a dictionary.

    Returns
    :   A dictionary with elements corresponding to reaction attributes.

    Return type
    :   OrderedDict

    See also

    `reaction_from_dict()`

`mass.io.dict.``reaction_from_dict`(*reaction*, *model*)[source]¶
:   Create a `MassReaction` from a dictionary.

    Notes

    The presence of the `EnzymeModuleReaction.enzyme_module_id`
    attribute is used to determine whether the dictionary should be initialized
    as an `EnzymeModuleReaction` or as a `MassReaction`.

    Parameters
    :   - **reaction** (*dict*) – A dictionary with elements corresponding to the reaction attributes.
        - **model** (*MassModel*) – The model to assoicate with the reaction.

    Returns
    :   The generated reaction.

    Return type
    :   MassReaction or EnzymeModuleReaction

    See also

    `reaction_to_dict()`

`mass.io.dict.``enzyme_to_dict`(*enzyme*)[source]¶
:   Convert an `EnzymeModuleDict` into a serializable dictionary.

    Parameters
    :   **enzyme** (*EnzymeModuleDict*) – The enzyme module to represent as a dictionary.

    Returns
    :   A dictionary with elements corresponding to the enzyme module
        attributes.

    Return type
    :   OrderedDict

    See also

    `enzyme_from_dict()`

`mass.io.dict.``enzyme_from_dict`(*enzyme*, *model*)[source]¶
:   Create an `EnzymeModuleDict` from a dictionary.

    Parameters
    :   - **enzyme** (*dict*) – A dictionary with elements corresponding to the enzyme module
          dictionary attributes.
        - **model** (*MassModel*) – The model to assoicate with the enzyme module dictionary.

    Returns
    :   The generated enzyme module dictionary.

    Return type
    :   EnzymeModuleDict

    See also

    `enzyme_to_dict()`

`mass.io.dict.``unit_to_dict`(*unit\_definition*)[source]¶
:   Convert an `UnitDefintion` into a serializable dictionary.

    Parameters
    :   **unit\_definition** (*UnitDefintion*) – The unit definition to represent as a dictionary.

    Returns
    :   A dictionary with elements corresponding to the unit definition
        attributes.

    Return type
    :   OrderedDict

    See also

    `unit_from_dict()`

`mass.io.dict.``unit_from_dict`(*unit\_definition*)[source]¶
:   Create an `UnitDefintion` from a dictionary.

    Parameters
    :   **unit\_definition** (*dict*) – A dictionary with elements corresponding to the unit definition
        attributes.

    Returns
    :   The generated unit definition.

    Return type
    :   UnitDefintion

    See also

    `unit_to_dict()`

###### `mass.io.json`¶

Create or load models in JSON format.

Models created in JSON format can also be viewed using the
Escher network visualization tool. See the
Escher web-based tool
or the
Python package
documentation for more information on Escher.

To enable faster JSON export, the `simplejson` package can be installed
during the `mass` installation process as follows:

```
# Installs simplejson package.
pip install masspy["json"]
# Or to install all additional packages.
pip install masspy["all"]
```

If the `visualization` submodule is installed, see the
`mass.visualiation.escher` documentation for more information on using
`mass` with `escher` (Coming soon).

###### Module Contents¶

###### Functions¶

|  |  |
| --- | --- |
| `to_json`(mass\_model, sort=False, \*\*kwargs) | Return the model as a JSON document. |
| `from_json`(document) | Load a model from a JSON document. |
| `save_json_model`(mass\_model, filename, sort=False, pretty=False, \*\*kwargs) | Write the model to a file in JSON format. |
| `load_json_model`(filename) | Load the model from a file in JSON format. |

`mass.io.json.``to_json`(*mass\_model*, *sort=False*, *\*\*kwargs*)[source]¶
:   Return the model as a JSON document.

    `kwargs` are passed on to `json.dumps`

    Parameters
    :   - **mass\_model** (*MassModel* *or* *EnzymeModule*) – The `mass` model to represent.
        - **sort** (*bool*) – Whether to sort the objects in the lists representing attributes, or
          to maintain the order defined in the model. Default is `False`.

    Returns
    :   String representation of the `mass` model as a JSON document.

    Return type
    :   str

    See also

    `save_json_model()`
    :   Write directly to a file.

    `json.dumps()`
    :   Base function.

`mass.io.json.``from_json`(*document*)[source]¶
:   Load a model from a JSON document.

    Parameters
    :   **document** (*str*) – The JSON document representation of a `mass` model.

    Returns
    :   The `mass` model as represented in the JSON document.

    Return type
    :   MassModel or EnzymeModule

    See also

    `load_json_model()`
    :   Load directly from a file.

`mass.io.json.``save_json_model`(*mass\_model*, *filename*, *sort=False*, *pretty=False*, *\*\*kwargs*)[source]¶
:   Write the model to a file in JSON format.

    `kwargs` are passed on to `json.dump`

    Parameters
    :   - **mass\_model** (*MassModel* *or* *EnzymeModule*) – The `mass` model to represent.
        - **filename** (*str* *or* *file-like*) – File path or descriptor the the JSON representation should be
          written to.
        - **sort** (*bool*) – Whether to sort the objects in the lists representing attributes, or
          to maintain the order defined in the model. Default is `False`.
        - **pretty** (*bool*) – Whether to format the JSON more compactly (default), or in a more
          verbose but easier to read fashion. Default is `False`.
          Can be partially overwritten by the `kwargs`.

    See also

    `to_json()`
    :   Create a string represenation of the model in JSON format.

    `json.dump()`
    :   Base function.

`mass.io.json.``load_json_model`(*filename*)[source]¶
:   Load the model from a file in JSON format.

    Parameters
    :   **filename** (*str* *or* *file-like*) – File path or descriptor the contains JSON document describing the
        `mass` model to be loaded.

    Returns
    :   The `mass` model as represented in the JSON formatted file.

    Return type
    :   MassModel or EnzymeModule

    See also

    `from_json()`
    :   Load a model from a string representation in JSON format.

`mass.io.json.``JSON_SCHEMA`[source]¶
:   The generic JSON schema for representing a model in `mass`.

    Type
    :   dict

###### `mass.io.sbml`¶

SBML import and export using the `python-libsbml` package.

- The SBML importer supports all versions of SBML
  that are compatible with the `roadrunner` package.
- The `sbml` module supports the latest version of
  python-libsbml
  that is compatible with the `roadrunner` package.
- The SBML importer supports the ‘fbc’ and ‘groups’ package extension.
- The SBML exporter writes SBML Level 3 models.
- Annotation information is stored on the `mass` objects.
- Information from the ‘groups’ package is read.
- All equations are written via MathML.

Parsing of models using the
fbc extension
was implemented as efficiently as possible, whereas (discouraged) fallback
solutions are not optimized for efficiency. Futhermore, because the SBML
kinetic law is used for the reaction kinetic laws, fbc information will NOT
be written into or read from the SBML kinetic laws. Whether the fbc package
extension is disabled or enabled will not change this behavior with kinetic
laws.

Notes are only supported in a minimal way relevant for kinetic models, i.e.
structured information from notes in the form:

```
"<p>key: value</p>"
```

Notes are read into the `notes` attribute of `mass` objects when
reading SBML files. On writing, the `notes` attribute of `mass`
objects dictionary is serialized to the SBML notes information.

Attribute information for `EnzymeModuleForm`s and
`EnzymeModuleReaction`s are written into the SBML object notes
field. Upon import of the SBML, the information is read into the
enzyme specific attribute as long as the `"key"` in the notes matches the
attribute name precisely.

The information specific to attributes of the `EnzymeModule` and
`EnzymeModuleDict` information is stored using the
groups extension
by creating an SBML ‘group’ representing the enzyme module containing
additional SBML group objects for enzyme module ligands, forms, and reactions
for the categories of the enzyme module categorized dictionary attributes.
The remaining information is written to the the notes field of the main SBML
group for the enzyme module. Disabling use of the ‘groups’ package extension
will result in the loss of the enzyme specific information, but it will not
prevent `EnzymeModuleForm`s and `EnzymeModuleReaction`s
from being written to the SBML model as species and reactions, respectively.

Annotations are read and written via `annotation` attribute for
`mass` objects.

Some SBML related issues are still open, please refer to the respective issue:

- update annotation format and support qualifiers (depends on decision
  for new annotation format; https://github.com/opencobra/cobrapy/issues/684)
- write compartment annotations and notes (depends on updated first-class
  compartments; see https://github.com/opencobra/cobrapy/issues/760)
- support compression on file handles (depends on solution for
  https://github.com/opencobra/cobrapy/issues/812)

###### Module Contents¶

###### Functions¶

|  |  |
| --- | --- |
| `read_sbml_model`(filename, f\_replace=None, \*\*kwargs) | Read SBML model from the given filename into a `mass` model. |
| `write_sbml_model`(mass\_model, filename, f\_replace=None, \*\*kwargs) | Write `mass` model to `filename` in SBML format. |
| `validate_sbml_model`(filename, check\_model=True, internal\_consistency=True, check\_units\_consistency=False, check\_modeling\_practice=False, \*\*kwargs) | Validate the SBML model and returns the model along with the errors. |
| `validate_sbml_model_export`(mass\_model, filename, f\_replace=None, \*\*kwargs) | Validate export of a `mass` model to SBML, returning any errors. |

`mass.io.sbml.``LOGGER`[source]¶
:   Logger for the `sbml` submodule.

    Type
    :   logging.Logger

`mass.io.sbml.``SBML_LEVEL_VERSION` *= [3, 1]*[source]¶
:   Current level and version supported for SBML export.

    Type
    :   tuple

`mass.io.sbml.``FBC_VERSION` *= 2*[source]¶
:   Current version of the SBML ‘fbc’ package extension.

    Type
    :   int

`mass.io.sbml.``GROUPS_VERSION` *= 1*[source]¶
:   Current version of the SBML ‘groups’ package extension.

    Type
    :   int

`mass.io.sbml.``CHAR_RE`[source]¶
:   Regex for ASCII character removal.

    Type
    :   `re.Pattern`

`mass.io.sbml.``MASS_MOIETY_RE`[source]¶
:   Regex for `mass` moiety replacements.

    Type
    :   `re.Pattern`

`mass.io.sbml.``SBML_MOIETY_RE`[source]¶
:   Regex for SBML moiety replacements.

    Type
    :   `re.Pattern`

`mass.io.sbml.``RATE_CONSTANT_RE`[source]¶
:   Regex for recognizing and rate constants.

    Type
    :   `re.Pattern`

`mass.io.sbml.``SBO_MODELING_FRAMEWORK` *= SBO:0000062*[source]¶
:   SBO term for the modeling framework

    Type
    :   str

`mass.io.sbml.``COBRA_FLUX_UNIT`[source]¶
:   Unit definition for `cobra` flux units.

    Type
    :   `UnitDefintion`

`mass.io.sbml.``NUMBER_ID_PREFIX` *= \_*[source]¶
:   String to use as a prefix for identifiers starting with a number.

    Type
    :   str

`mass.io.sbml.``F_GENE` *= F\_GENE*[source]¶
:   Key in `F_REPLACE` for the gene prefix clipping function.

    Type
    :   str

`mass.io.sbml.``F_GENE_REV` *= F\_GENE\_REV*[source]¶
:   Key in `F_REPLACE` for the gene prefix adding function.

    Type
    :   str

`mass.io.sbml.``F_SPECIE` *= F\_SPECIE*[source]¶
:   Key in `F_REPLACE` for the specie prefix clipping function.

    Type
    :   str

`mass.io.sbml.``F_SPECIE_REV` *= F\_SPECIE\_REV*[source]¶
:   Key in `F_REPLACE` for the specie prefix adding function.

    Type
    :   str

`mass.io.sbml.``F_REACTION` *= F\_REACTION*[source]¶
:   Key in `F_REPLACE` for the reaction prefix clipping function.

    Type
    :   str

`mass.io.sbml.``F_REACTION_REV` *= F\_REACTION\_REV*[source]¶
:   Key in `F_REPLACE` for the reaction prefix adding function.

    Type
    :   str

`mass.io.sbml.``F_REPLACE`[source]¶
:   Contains functions for ID clipping/adding of prefixes.

    Type
    :   dict

`mass.io.sbml.``ASCII_REPLACE`[source]¶
:   Contains ASCII characters and the value for their replacement.

    Type
    :   dict

`mass.io.sbml.``read_sbml_model`(*filename*, *f\_replace=None*, *\*\*kwargs*)[source]¶
:   Read SBML model from the given filename into a `mass` model.

    If the given filename ends with the suffix `'.gz'` (for example,
    `'myfile.xml.gz'`), the file is assumed to be compressed in gzip
    format and will be automatically decompressed upon reading. Similarly,
    if the given filename ends with `'.zip'` or `'.bz2'`, the file is
    assumed to be compressed in zip or bzip2 format (respectively).

    Files whose names lack these suffixes will be read uncompressed. Note that
    if the file is in zip format but the archive contains more than one file,
    only the first file in the archive will be read and the rest are ignored.

    To read a gzip/zip file, `libSBML` needs to be configured and linked
    with the `zlib` library at compile time. It also needs to be linked
    with the `bz2` library to read files in bzip2 format.
    (Both of these are the default configurations for `libSBML`.)

    This function supports SBML with FBC-v1 and FBC-v2. FBC-v1 models
    are converted to FBC-v2 models before reading.

    The parser tries to fall back to information in notes dictionaries
    if information is not available in the FBC packages, e.g.,
    CHARGE, FORMULA on species, or GENE\_ASSOCIATION, SUBSYSTEM on reactions.

    Notes

    - Provided file handles cannot be opened in binary mode, i.e., use:

      ```
      with open(path, "r" as f):
          read_sbml_model(f)
      ```
    - File handles to compressed files are not supported yet.

    Parameters
    :   - **filename** (*path to SBML file**,* *SBML string**, or* *SBML file handle*) – SBML which is read into a `mass` model.
        - **f\_replace** (*dict*) –

          Dictionary of replacement functions for gene, specie, and reaction. By
          default the following id changes are performed on import: clip `'G_'`
          from genes, clip `'M_'` from species, clip `'R_'` from reactions.

          If no replacements should be performed, set `f_replace={}`.
        - **\*\*kwargs** –

          number :
          :   In which data type should the stoichiometry be parsed. Can be
              `float` or `int`.

              Default is `float`.

          set\_missing\_bounds :
          :   `bool` indicating whether to set missing bounds to the
              default bounds from the `MassConfiguration`.

              Default is `True`.

          remove\_char :
          :   `bool` indicating whether to remove ASCII characters from IDs.

              Default is `True`.

          stop\_on\_conversion\_failbool
          :   `bool` indicating whether to stop trying to load the model if a
              conversion process fails. If `False`, then the loading of the
              model will be attempted anyways, despite a potential loss of
              information.

              Default is `True`.

    Returns
    :   The generated `mass` model.

    Return type
    :   MassModel or EnzymeModule

    Raises
    :   **MassSBMLError** – Errors due to :mass model specific requirements.

`mass.io.sbml.``write_sbml_model`(*mass\_model*, *filename*, *f\_replace=None*, *\*\*kwargs*)[source]¶
:   Write `mass` model to `filename` in SBML format.

    The created model is SBML level 3 version 1 core (L3V1) using packages
    ‘fbc-v2’ and ‘groups-v1’ for optimal exporting. Not including these
    packages may result in some information loss when exporting the model.

    If the given filename ends with the suffix `'.gz'` (for example,
    `'myfile.xml.gz'`), `libSBML` assumes the caller wants the file
    to be written compressed in gzip format. Similarly, if the given filename
    ends with `'.zip'` or `'.bz2'`, `libSBML` assumes the caller
    wants the file to be compressed in zip or bzip2 format (respectively).
    Files whose names lack these suffixes will be written uncompressed.

    Special considerations for the zip format: If the given filename ends with
    `'.zip'`, the file placed in the zip archive will have the suffix
    `".xml"` or `".sbml"`. For example, the file in the zip archive will
    be named `"test.xml"` if the given filename is `"test.xml.zip"` or
    `"test.zip"`. Similarly, the filename in the archive will be
    `"test.sbml"` if the given filename is `"test.sbml.zip"`.

    Parameters
    :   - **mass\_model** (*MassModel* *or* *EnzymeModule*) – The `mass` model to write to into an SBML compliant modle file.
        - **filename** (*str*) – Path to which the model should be written
        - **f\_replace** (*dict*) –

          Dictionary of replacement functions for gene, specie, and reaction. By
          default the following id changes are performed on import: add `'G_'`
          to genes, add `'M_'` to species, add `'R_'` to reactions.

          If no replacements should be performed,set `f_replace={}`.
        - **\*\*kwargs** –

          use\_fbc\_package :
          :   `bool` indicating whether SBML ‘fbc’ package extension
              should be used.

              Default is `True`.

          use\_groups\_package :
          :   `bool` indicating whether SBML ‘groups’ package extension
              should be used.

              Default is `True`.

          units :
          :   `bool` indicating whether units should be written into
              the SBMLDocument.

              Default is `True`.

          local\_parameters :
          :   `bool` indicating whether reaction kinetic parameters should be
              written as local parameters of the kinetic law (default),
              or as global model parameters in the SBML model file.

              Default is `True` to write parameters as local parameters.

          write\_objective :
          :   `bool` indicating whether the model objective(s) should also be
              written into the SBML model file.

              Default is `False`.

    Raises
    :   **MassSBMLError** – Errors due to :mass model specific requirements.

    Warning

    - Setting the `use_fbc_package=False` may result in some information
      loss when writing the model.
    - Setting the `use_groups_package=False` may result in some information
      loss when writing the model. Information lost will include some
      attributes associated with enzyme modules and will likely result in an
      exported `EnzymeModule` becoming a `MassModel` upon
      reloading the model.

`mass.io.sbml.``validate_sbml_model`(*filename*, *check\_model=True*, *internal\_consistency=True*, *check\_units\_consistency=False*, *check\_modeling\_practice=False*, *\*\*kwargs*)[source]¶
:   Validate the SBML model and returns the model along with the errors.

    `kwargs` are passed to `read_sbml_model()`.

    Parameters
    :   - **filename** (*str*) – The filename (or SBML string) of the SBML model to be validated.
        - **check\_model** (*bool*) – Check some basic model properties. Default is `True`.
        - **internal\_consistency** (*bool*) – Check internal consistency. Default is `True`.
        - **check\_units\_consistency** (*bool*) – Check consistency of units. Default is `False`.
        - **check\_modeling\_practice** (*bool*) – Check modeling practice. Default is `False`.
        - **\*\*kwargs** –

          number :
          :   In which data type should the stoichiometry be parsed. Can be
              `float` or `int`.

              Default is `float`.

          set\_missing\_bounds :
          :   `bool` indicating whether to set missing bounds to the
              default bounds from the `MassConfiguration`.

              Default is `True`.

          remove\_char :
          :   `bool` indicating whether to remove ASCII characters from IDs.

              Default is `True`.

          stop\_on\_conversion\_failbool
          :   `bool` indicating whether to stop trying to load the model if a
              conversion process fails. If `False`, then the loading of the
              model will be attempted anyways, despite a potential loss of
              information.

              Default is `True`.

    Returns
    :   - *tuple (model, errors)*
        - **model** (`MassModel` or `EnzymeModule`, or `None`) – The `mass` model if the file could be read successfully.
          If the file was not successfully read, `None` will be returned.
        - **errors** (*dict*) – Warnings and errors grouped by their respective types.

`mass.io.sbml.``validate_sbml_model_export`(*mass\_model*, *filename*, *f\_replace=None*, *\*\*kwargs*)[source]¶
:   Validate export of a `mass` model to SBML, returning any errors.

    If no SBML errors or MASS fatal errors occur, the model will be written to
    the `'filename'`.

    `kwargs` are passed to either `write_sbml_model()` or
    `validate_sbml_model()`.

    Parameters
    :   - **mass\_model** (*MassModel* *or* *EnzymeModule*) – The `mass` model to write to into an SBML compliant modle file.
        - **filename** (*str*) – Path to which the model should be written
        - **f\_replace** (*dict*) –

          Dictionary of replacement functions for gene, specie, and reaction. By
          default the following id changes are performed on import: add `'G_'`
          to genes, add `'M_'` to species, add `'R_'` to reactions.

          If no replacements should be performed,set `f_replace={}`.
        - **\*\*kwargs** –

          use\_fbc\_package :
          :   `bool` indicating whether SBML ‘fbc’ package extension
              should be used.

              Default is `True`.

          use\_groups\_package :
          :   `bool` indicating whether SBML ‘groups’ package extension
              should be used.

              Default is `True`.

          units :
          :   `bool` indicating whether units should be written into
              the SBMLDocument.

              Default is `True`.

          local\_parameters :
          :   `bool` indicating whether reaction kinetic parameters should be
              written as local parameters of the kinetic law (default),
              or as global model parameters in the SBML model file.

              Default is `True` to write parameters as local parameters.

          write\_objective :
          :   `bool` indicating whether the model objective(s) should also be
              written into the SBML model file.

              Default is `False`.

          check\_modelbool
          :   `bool` indicating whether to check some basic model properties.

              Default is `True`.

          internal\_consistencybool
          :   `bool` indicating whether to check internal consistency.

              Default is `True`.

          check\_units\_consistencybool
          :   `bool` indicating whether to check consistency of units.

              Default is `False`.

          check\_modeling\_practicebool
          :   `bool` indicating whether to check modeling practice.

              Default is `False`.

          number :
          :   In which data type should the stoichiometry be parsed. Can be
              `float` or `int`.

              Default is `float`.

          set\_missing\_bounds :
          :   `bool` indicating whether to set missing bounds to the
              default bounds from the `MassConfiguration`.

              Default is `True`.

          remove\_char :
          :   `bool` indicating whether to remove ASCII characters from IDs.

              Default is `True`.

          stop\_on\_conversion\_failbool
          :   `bool` indicating whether to stop trying to load the model if a
              conversion process fails. If `False`, then the loading of the
              model will be attempted anyways, despite a potential loss of
              information.

              Default is `True`.

    Returns
    :   - *tuple (success, errors)*
        - **success** (*bool*) – `bool` indicating whether the model was successfully exported to
          `'filename'`.
        - **errors** (*dict*) – Warnings and errors grouped by their respective types.

###### `mass.simulation`¶

###### Submodules¶

###### `mass.simulation.ensemble`¶

Module to create and manage an ensemble of models.

The method of the `ensemble` submodule are designed to generate an
ensemble of models. It contains various methods to assist in generating
multiple models from existing `MassModel`s, using flux data or
concentration data in `pandas.DataFrame`s (e.g. generated from
`conc_sampling`). There are also methods to help ensure that
models are thermodynamically feasible and can reach steady states with or
without perturbations applied.

In addition to containing various methods that can be combined into
an ensemble generation workflow, the `ensemble` submodule contains the
`generate_ensemble_of_models()` function, which is optimized for
performance when generating a large number of models.

The `generate_ensemble_of_models()` function also ensures that the user
input is valid before generating models to reduce the likelihood of a user
error causing the model generation process to stop before completion. However,
there is time spent in function’s setup, meaining that when generating a
smaller number of models, performance gains may not be seen.

###### Module Contents¶

###### Functions¶

|  |  |
| --- | --- |
| `create_models_from_flux_data`(reference\_model, data=None, raise\_error=False, \*\*kwargs) | Generate ensemble of models for a given set of flux data. |
| `create_models_from_concentration_data`(reference\_model, data=None, raise\_error=False, \*\*kwargs) | Generate ensemble of models for a given set of concentration data. |
| `ensure_positive_percs`(models, reactions=None, raise\_error=False, update\_values=False, \*\*kwargs) | Seperate models based on whether all calculated PERCs are positive. |
| `ensure_steady_state`(models, strategy=’simulate’, perturbations=None, solver\_options=None, update\_values=False, \*\*kwargs) | Seperate models based on whether a steady state can be reached. |
| `generate_ensemble_of_models`(reference\_model, flux\_data=None, conc\_data=None, ensure\_positive\_percs=None, strategy=None, perturbations=None, \*\*kwargs) | Generate an ensemble of models for given data sets. |

`mass.simulation.ensemble.``create_models_from_flux_data`(*reference\_model*, *data=None*, *raise\_error=False*, *\*\*kwargs*)[source]¶
:   Generate ensemble of models for a given set of flux data.

    Parameters
    :   - **reference\_model** (*iterable**,* *None*) – A `MassModel` object to treat as the reference model.
        - **data** (*pandas.DataFrame*) – A `pandas.DataFrame` containing the flux data for generation
          of the models. Each row is a different set of flux values to
          generate a model for, and each column corresponds to the reaction
          identifier for the flux value.
        - **raise\_error** (*bool*) – Whether to raise an error upon failing to generate a model from a
          given reference. Default is `False`.
        - **\*\*kwargs** –

          verbose :
          :   `bool` indicating the verbosity of the function.

              Default is `False`.

          suffix :
          :   `str` representing the suffix to append to generated models.

              Default is `'_F'`.

    Returns
    :   **new\_models** – A `list` of successfully generated `MassModel` objects.

    Return type
    :   list

    Raises
    :   **MassEnsembleError** – Raised if generation of a model fails and `raise_error=True`.

`mass.simulation.ensemble.``create_models_from_concentration_data`(*reference\_model*, *data=None*, *raise\_error=False*, *\*\*kwargs*)[source]¶
:   Generate ensemble of models for a given set of concentration data.

    Parameters
    :   - **reference\_model** (*iterable**,* *None*) – A `MassModel` object to treat as the reference model.
        - **data** (*pandas.DataFrame*) – A `pandas.DataFrame` containing the concentration data for
          generation of the models. Each row is a different set of
          concentration values to generate a model for, and each column
          corresponds to the metabolite identifier for the concentraiton
          value.
        - **raise\_error** (*bool*) – Whether to raise an error upon failing to generate a model from a
          given reference. Default is `False`.
        - **\*\*kwargs** –

          verbose :
          :   `bool` indicating the verbosity of the function.

              Default is `False`.

          suffix :
          :   `str` representing the suffix to append to generated models.

              Default is `'_C'`.

    Returns
    :   **new\_models** – A `list` of successfully generated `MassModel` objects.

    Return type
    :   list

    Raises
    :   **MassEnsembleError** – Raised if generation of a model fails and `raise_error=True`.

`mass.simulation.ensemble.``ensure_positive_percs`(*models*, *reactions=None*, *raise\_error=False*, *update\_values=False*, *\*\*kwargs*)[source]¶
:   Seperate models based on whether all calculated PERCs are positive.

    Parameters
    :   - **models** (*iterable*) – An iterable of `MassModel` objects to use for PERC
          calculations.
        - **reactions** (*iterable*) – An iterable of reaction identifiers to calculate the
          PERCs for. If `None`, all reactions in the model will be used.
        - **raise\_error** (*bool*) – Whether to raise an error upon failing to generate a model from a
          given reference. Default is `False`.
        - **update\_values** (*bool*) – Whether to update the PERC values for models that generate all
          positive PERCs. Default is `False`.
        - **\*\*kwargs** –

          verbose :
          :   `bool` indicating the verbosity of the function.

              Default is `False`.

          at\_equilibrium\_default :
          :   `float` value to set the pseudo-order rate constant if the
              reaction is at equilibrium.

              Default is `100,000`.

    Returns
    :   - *tuple (positive, negative)*
        - **positive** (*list*) – A `list` of `MassModel` objects whose calculated PERC
          values were postiive.
        - **negative** (*list*) – A `list` of `MassModel` objects whose calculated PERC
          values were negative.

    Raises
    :   **MassEnsembleError** – Raised if PERC calculation fails and `raise_error=True`.

`mass.simulation.ensemble.``ensure_steady_state`(*models*, *strategy='simulate'*, *perturbations=None*, *solver\_options=None*, *update\_values=False*, *\*\*kwargs*)[source]¶
:   Seperate models based on whether a steady state can be reached.

    All `kwargs` are passed to `find_steady_state()`.

    Parameters
    :   - **models** (*MassModel**,* *iterable*) – A `MassModel` or an iterable of `MassModel` objects to
          find a steady state for.
        - **strategy** (*str*) –

          The strategy for finding the steady state. Must be one of the
          following:

          > - `'simulate'`
          > - `'nleq1'`
          > - `'nleq2'`
        - **perturbations** (*dict*) – A `dict` of perturbations to incorporate into the simulation.
          Models must reach a steady state with the given pertubration to be
          considered as feasible.
          See `simulation` documentation for more
          information on valid perturbations.
        - **solver\_options** (*dict*) – A dict of options to pass to the solver utilized in determining a
          steady state. Solver options should be for the
          `roadrunner.Integrator` if `strategy="simulate"`, otherwise
          options should correspond to the `roadrunner.SteadyStateSolver`.
        - **update\_values** (*bool*) – Whether to update the model with the steady state results.
          Default is `False`. Only updates models that reached steady state.
        - **\*\*kwargs** –

          verbose :
          :   `bool` indicating the verbosity of the method.

              Default is `False`.

          steps :
          :   `int` indicating number of steps at which the output is
              sampled where the samples are evenly spaced and
              `steps = (number of time points) - 1.`
              Steps and number of time points may not both be specified.
              Only valid for `strategy='simulate'`.

              Default is `None`.

          tfinal :
          :   `float` indicating the final time point to use in when
              simulating to long times to find a steady state.
              Only valid for `strategy='simulate'`.

              Default is `1e8`.

          num\_attempts :
          :   `int` indicating the number of attempts the steady state
              solver should make before determining that a steady state
              cannot be found. Only valid for `strategy='nleq1'` or
              `strategy='nleq2'`.

              Default is `2`.

          decimal\_precision :
          :   `bool` indicating whether to apply the
              `decimal_precision` attribute of
              the `MassConfiguration` to the solution values.

              Default is `False`.

    Returns
    :   - *tuple (feasible, infeasible)*
        - **feasible** (*list*) – A `list` of `MassModel` objects that could successfully reach
          a steady state.
        - **infeasible** (*list*) – A `list` of `MassModel` objects that could not successfully
          reach a steady state.

`mass.simulation.ensemble.``generate_ensemble_of_models`(*reference\_model*, *flux\_data=None*, *conc\_data=None*, *ensure\_positive\_percs=None*, *strategy=None*, *perturbations=None*, *\*\*kwargs*)[source]¶
:   Generate an ensemble of models for given data sets.

    This function is optimized for performance when generating a large
    ensemble of models when compared to the combination of various individual
    methods of the `ensemble` submodule used. However, this function may
    not provide as much control over the process when compared to utilizing a
    combination of other methods defined in the `ensemble` submodule.

    Notes

    - Only one data set is required to generate the ensemble, meaning that
      a flux data set can be given without a concentration data set, and vice
      versa.
    - If `x` flux data samples and `y` concentration data samples are
      provided, `x * y` total models will be generated.
    - If models deemed `infeasible` are to be returned, ensure the
      `return_infeasible` kwarg is set to `True`.

    Parameters
    :   - **reference\_model** (*MassModel*) – The reference model used in generating the ensemble.
        - **flux\_data** (*pandas.DataFrame* *or* *None*) – A `pandas.DataFrame` containing the flux data for generation
          of the models. Each row is a different set of flux values to
          generate a model for, and each column corresponds to the reaction
          identifier for the flux value.
        - **conc\_data** (*pandas.DataFrame* *or* *None*) – A `pandas.DataFrame` containing the concentration data for
          generation of the models. Each row is a different set of
          concentration values to generate a model for, and each column
          corresponds to the metabolite identifier for the concentraiton
          value.
        - **ensure\_positive\_percs** – A `list` of reactions to calculate PERCs for, ensure they
          are postive, and update feasible models with the new PERC values.
          If `None`, no PERCs will be checked.
        - **strategy** (*str**,* *None*) –

          The strategy for finding the steady state.
          Must be one of the following:

          > - `'simulate'`
          > - `'nleq1'`
          > - `'nleq2'`

          If a `strategy` is given, models must reach a steady state to be
          considered feasible. All feasible models are updated to steady state.
          If `None`, no attempts will be made to determine whether a generated
          model can reach a steady state.
        - **perturbations** (*dict*) –

          A `dict` of perturbations to incorporate into the simulation,
          or a list of perturbation dictionaries where each `dict` is applied
          to a simulation. Models must reach a steady state with all given
          pertubration dictionaries to be considered feasible.
          See `simulation` documentation for more
          information on valid perturbations.

          Ignored if `strategy=None`.
        - **\*\*kwargs** –

          solver\_options :
          :   `dict` of options to pass to the solver utilized in determining
              a steady state. Solver options should be for the
              `roadrunner.Integrator` if `strategy="simulate"`,
              otherwise options should correspond to the
              `roadrunner.SteadyStateSolver`.

              Default is `None`.

          verbose :
          :   `bool` indicating the verbosity of the function.

              Default is `False`.

          decimal\_precision :
          :   `bool` indicating whether to apply the
              `decimal_precision` attribute of
              the `MassConfiguration` to the solution values.

              Default is `False`.

          flux\_suffix :
          :   `str` representing the suffix to append to generated models
              indicating the flux data set used.

              Default is `'_F'`.

          conc\_suffix :
          :   `str` representing the suffix to append to generated models
              indicating the conc data set used.

              Default is `'_C'`.

          at\_equilibrium\_default :
          :   `float` value to set the pseudo-order rate constant if the
              reaction is at equilibrium.

              Default is `100,000`. Ignored if `ensure_positive_percs=None`.

          return\_infeasible :
          :   `bool` indicating whether to generate and return an
              `Ensemble` containing the models deemed infeasible.

              Default is `False`.

    Returns
    :   - **feasible** (*list*) – A `list` containing the MassModel objects that are deemed
          feasible by sucessfully passing through all PERC and simulation
          checks in the ensemble building processes.
        - **infeasible** (*list*) – A `list` containing the MassModel objects that are deemed
          infeasible by failing to passing through one of the PERC or
          simulation checks in the ensemble building processes.

###### `mass.simulation.simulation`¶

The Simulation module addresses the simulation of `MassModel`s.

The `Simulation` is designed to address all aspects related to the
simulation of one or more `MassModel`s. These aspects include
initializing and compiling the model into a `roadrunner.RoadRunner`
instance (the ODE integrator), storing the numerical values necessary for the
simulations, and handling the simulation results by creating and storing
`MassSolution`s.

A `Simulation` is initialized by providing a simulatable
`MassModel` that can be converted into an SBML compliant model.

Multiple models can be added to the `Simualtion` in order to simulate
an ensemble of models. To add an additional model to the ensemble, the model
must meet three criteria:

> 1. The model must have equivalent ODEs to the
>    `reference_model`
> 2. The model must not have the same ID as the
>    `reference_model`.
> 3. The model must have all numerical values needed for simulation.

Perturbations can be implemented for a given simulation as long as they
follow the following guidelines:

> 1. Perturbations are `dict`s where the `{key: value}` pairs are the
>    variables to be perturbed and the new numerical value or value change.
> 2. To scale the current value of a variable, the value should be a `str`
>    representing the formula for altering perturbation variable, where the
>    variable in the `str` is identical to the perturbation key.
> 3. If providing a formula `str` as the perturbation value, it must be
>    possible to ‘sympify’ the string using the
>    `sympify()` function. It must only have one
>    variable, identical to the perturbation key.
> 4. Only boundary conditions can be changed to have functions of time that
>    represent the external concentration at that point in time.
>    If a perturbation value is to be a string representing a function, it
>    must be a function, it may only contain the time variable `'t'` and
>    the boundary metabolite variable.

Some examples of perturbations following the guidelines for a model containing
the specie with ID `'MID_c'`, boundary metabolite with ID `'MID_b'`, and
reaction with ID `'RID'`:

> - Altering `initial_conditions` (ICs):
>
>   > - `{'MID_c': 2}` Change the IC value to 2.
>   > - `{'MID_c': 'MID_c * 1.5'}` Increase current IC value by 50%.
> - Altering `parameters`:
>
>   > - `{'kf_RID': 'kf_RID * 0.75'}` Decrease kf parameter value by 25%.
>   > - `{'Keq_RID': 100}` Change Keq parameter value to 100.
> - Altering `boundary_conditions` (BCs):
>
>   > - `{'MID_b': 'sin(2 * pi * t)'}` Change BC to a `sin` function.
>   > - `{'MID_b': 'MID_b + cos(t)'}` Add `cos` function to current BC
>   >   value.

Note that perturbations using functions of time may take longer to implement
than other perturbations.

All simulation results are returned as `MassSolution`s.
Each simulated model has a corresponding `MassSolution`s stored
in the `Simulation`. These solution objects are stored until being
replaced by a new `MassSolution` upon resimulating the model. This
means that there can only be one concentration solution and one flux solution
per simulated model. A failed simulation of a model will return an empty
`MassSolution`.

Though the `Simulation` utilizes the `roadrunner` package, the
standard `logging` module will be used for `mass` logging purposes in
the `simulation` submodule. Therefore, the roadrunner logger is disabled
upon loading the `simulation` submodule. However, because the
`Simulation` utilizes the `roadrunner` package for simulating
models, the `roadrunner.Logger` can be accessed via the
`RR_LOGGER` variable for those who wish to utilize it. See the
`roadrunner` documentation for more information on how to configure the
`roadrunner.Logger`.

###### Module Contents¶

###### Classes¶

|  |  |
| --- | --- |
| `Simulation` | Class for managing setup and result handling of simulations. |

`mass.simulation.simulation.``LOGGER`[source]¶
:   Logger for `simulation` submodule.

    Type
    :   logging.Logger

`mass.simulation.simulation.``RR_LOGGER`[source]¶
:   The logger for the `roadrunner`.

    Type
    :   roadrunner.Logger

*class* `mass.simulation.simulation.``Simulation`(*reference\_model*, *id=None*, *name=None*, *verbose=False*, *\*\*kwargs*)[source]¶
:   Bases: `cobra.core.object.Object`

    Class for managing setup and result handling of simulations.

    The `Simulation` class is designed to address all aspects related
    to the simulation of `MassModel` objects, including setting the
    solver and solver options, perturbation of concentrations and parameters,
    simulation of a single model or an ensemble of models, and handling
    of simulation results.

    Parameters
    :   - **reference\_model** (*MassModel*) – The model to load for simulation. The model will be set as the
          `Simulation.reference_model`.
        - **id** (*str* *or* *None*) – An identifier to associate with the `Simulation`. If `None`
          then one is automatically created based on the model identifier.
        - **name** (*str*) – A human readable name for the `Simulation`.
        - **verbose** (*bool*) – Whether to provide a QCQA report and more verbose messages when trying
          to load the model. Default is `False`.
        - **\*kwargs** –

          variable\_step\_size :
          :   `bool` indicating whether to initialize the integrator with a
              variable time step for simulations.

              Default is `True`.

          allow\_approx :
          :   `bool` indicating whether to allow the steady state solver to
              approximate the steady state solution in cases where NLEQ methods
              fail to converge to steady state due to a singular Jacobian matrix.

              Default is `True`.

    *property* `reference_model`(*self*)[source]¶
    :   Return the reference model of the `Simulation`.

    *property* `models`(*self*)[source]¶
    :   Return the IDs of models that exist in the `Simulation`.

    *property* `roadrunner`(*self*)[source]¶
    :   Return the `RoadRunner` instance.

    *property* `concentration_solutions`(*self*)[source]¶
    :   Get a copy of stored `MassSolution`s for concentrations.

        Returns
        :   Contains all `MassSolution` objects for concentrations.

        Return type
        :   DictList

    *property* `flux_solutions`(*self*)[source]¶
    :   Get a copy of the stored `MassSolution`s for fluxes.

        Returns
        :   Contains all `MassSolution` objects for fluxes.

        Return type
        :   DictList

    *property* `integrator`(*self*)[source]¶
    :   Return the `roadrunner.roadrunner.Integrator`.

    *property* `steady_state_solver`(*self*)[source]¶
    :   Return the `roadrunner.roadrunner.SteadyStateSolver`.

    `set_new_reference_model`(*self*, *model*, *verbose=False*)[source]¶
    :   Set a new reference model for the `Simulation`.

        To set a new reference model, the model must meet three criteria:

        > 1. The model must have equivalent ODEs to the
        >    `reference_model`.
        > 2. The model must not have the same ID as the
        >    `reference_model`
        > 3. The model must have all numerical values needed for simulation.

        If the criteria is not met, a warning is raised and the reference model
        will not change.

        After changing the reference model, the previous reference model will
        remain included in the `Simulation`.

        Parameters
        :   - **model** (*MassModel* *or* *str*) – Either a new or existing `MassModel`, or the string
              identifer of an existing model in the `Simulation` to be set
              as the new reference model.
            - **verbose** (*bool*) – Whether to output additional and more verbose messages.
              Default is `False`.

    `get_model_simulation_values`(*self*, *model*)[source]¶
    :   Return two dictionaries containing initial and parameter values.

        Parameters
        :   **model** (*MassModel* *or* *str*) – The model or its identifier whose values are to be returned.

        Returns
        :   - *tuple (init\_conds, parameters)*
            - **init\_conds** (`DictWithID`) – A `DictWithID` containing
              initial conditions.
            - **parameters** (`DictWithID`) – A `DictWithID` containing
              model parameters.

    `add_models`(*self*, *models*, *verbose=False*, *disable\_safe\_load=False*)[source]¶
    :   Add the model values to the `Simulation`.

        To add a model to the `Simulation`, three criteria must be met:

        > 1. The model must have equivalent ODEs to the
        >    `reference_model`
        > 2. The model must not have the same ID as the
        >    `reference_model`.
        > 3. The model must have all numerical values needed for simulation.

        Notes

        - Only the model values are added to the `Simulation`.
        - If a model already exists in the Simulation, it will be replaced.
        - To verify that the model has equivalent ODEs to the reference model,
          use `MassModel.has_equivalent_odes()`.

        Parameters
        :   - **models** (*iterable of models*) – An iterable containing the `MassModel`s to add.
            - **verbose** (*bool*) – Whether to print if loading of models succeeds or fails.
              Default is `False`.
            - **disable\_safe\_load** (*bool*) – Whether to disable criteria checks. Default is `False`.

        Warning

        Use the `disable_safe_load` argument with caution, as setting the
        value as `disable_safe_load=True` will reduce the time it takes to
        add models, but models that do not adhere to the three criteria may
        create unexepcted downstream errors.

    `remove_models`(*self*, *models*, *verbose=False*)[source]¶
    :   Remove the model values from the `Simulation`.

        Notes

        The `reference_model` cannot be removed from the
        `Simulation`. In order to remove the current reference model,
        the reference model must first be changed to a different model using
        the `set_new_reference_model()` method.

        Parameters
        :   - **models** (*iterable of models* *or* *their identifiers*) – An iterable of `MassModel`s or their string identifiers
              to be removed.
            - **verbose** (*bool*) – Whether to print if removal of models succeeds.
              Default is `False`.

    `get_model_objects`(*self*, *models=None*)[source]¶
    :   Return the loaded models as `MassModel`s.

        Notes

        With the exception of the `reference_model`, only
        the numerical values of a mdoel are stored in order to improve
        performance. Therefore, when using this method to retrieve the
        `MassModel`s, all models are created anew, meaning that
        they will NOT be the same `MassModel`s that were loaded
        into the Simulation.

        Parameters
        :   **models** (*iterable of model identifiers*) – An iterable of strings containing the model identifiers of the
            desired `MassModel`s to return. If `None` then all
            models in the `Simulation` will be returned.

        Returns
        :   **mass\_models** – A `DictList` containing all of the
            `MassModel`s.

        Return type
        :   DictList

    `simulate`(*self*, *models=None*, *time=None*, *perturbations=None*, *\*\*kwargs*)[source]¶
    :   Simulate models and return results as `MassSolution`s.

        A simulation is carried out by simultaneously integrating the ODEs
        of the `models` to compute their solutions over the time interval
        specified by `time`, while temporarily incorporating events and
        changes specified in `perturbations`.

        Parameters
        :   - **models** (*iterable of models* *or* *their string identifiers**,* *None*) – The models to simulate. If `None` then all models loaded into
              the simulation object will be used. All models must already
              exist in the `Simulation`.
            - **time** (*tuple*) – Either a `tuple` containing the initial and final time points, or
              a `tuple` containing the initial time point, final time point,
              and the number of time points to use.
            - **perturbations** (*dict*) – A `dict` of perturbations to incorporate into the simulation.
              See `simulation` documentation for more
              information on valid perturbations.
            - **\*\*kwargs** –

              verbose :
              :   `bool` indicating the verbosity of the method.

                  Default is `False`.

              steps :
              :   `int` indicating number of steps at which the output is
                  sampled where the samples are evenly spaced and
                  `steps = (number of time points) - 1.`
                  Steps and number of time points may not both be specified.

                  Default is `None`.

              interpolate :
              :   `bool` indicating whether simulation results should be
                  returned to as interpolating functions.

                  Default is `False`.

              update\_solutions :
              :   `bool` indicating whether to replace the stored solutions in
                  the simulation with the new simulation results.

                  Default is `True`.

              decimal\_precision :
              :   `bool` indicating whether to apply the
                  `decimal_precision` attribute of
                  the `MassConfiguration` to the solution values.

                  Default is `False`.

        Returns
        :   - *tuple (conc\_solutions, flux\_solutions)*
            - **conc\_solutions** (*MassSolution or DictList*) – If only one model was simulated, the return type is
              a `MassSolution` containing the concentration solutions.
              If multiple models were simulated, the return type is a
              `DictList` of
              `MassSolution`s containing the concentration solutions.
              If a simulation failed, the corresponding `MassSolution`
              will be returned as empty.
            - **flux\_solutions** (*MassSolution or DictList*) – If only one model was simulated, the return type is
              a `MassSolution` containing the flux solutions.
              If multiple models were simulated, the return type is a
              `DictList` of
              `MassSolution`s containing the flux solutions.
              If a simulation failed, the corresponding `MassSolution`
              will be returned as empty.

        See also

        `integrator`
        :   Access the integrator utilized in simulations.

    `find_steady_state`(*self*, *models=None*, *strategy='nleq2'*, *perturbations=None*, *update\_values=False*, *\*\*kwargs*)[source]¶
    :   Find steady states for models.

        The steady state is found by carrying out the provided strategy.

        - The `'simulate'` strategy will simulate the model for a long time
          (default `1e8`), and ensure the absolute difference between
          solutions at the final two time points is less than the
          `steady_state_threshold` in the
          `MassConfiguration`.
        - Other strategies involve using the
          `roadrunner.roadrunner.SteadyStateSolver` class to determine
          the steady state through global Newtonian methods. The steady state
          is found when the sum of squares of the rates of change is less than
          the `steady_state_threshold` in the
          `MassConfiguration`.

        Parameters
        :   - **models** (*iterable of models* *or* *their string identifiers**,* *None*) – The models to simulate. If `None` then all models loaded into
              the simulation object will be used. All models must already
              exist in the `Simulation`.
            - **strategy** (*str*) –

              The strategy for finding the steady state. Must be one of the
              following:

              > - `'simulate'`
              > - `'nleq1'`
              > - `'nleq2'`
            - **perturbations** (*dict*) – A `dict` of perturbations to incorporate into the simulation.
              See `simulation` documentation for more
              information on valid perturbations.
            - **update\_values** (*bool*) – Whether to update the model with the steady state results.
              Default is `False`.
            - **\*\*kwargs** –

              verbose :
              :   `bool` indicating the verbosity of the method.

                  Default is `False`.

              steps :
              :   `int` indicating number of steps at which the output is
                  sampled where the samples are evenly spaced and
                  `steps = (number of time points) - 1.`
                  Steps and number of time points may not both be specified.
                  Only valid for `strategy='simulate'`.

                  Default is `None`.

              tfinal :
              :   `float` indicating the final time point to use in when
                  simulating to long times to find a steady state.
                  Only valid for `strategy='simulate'`.

                  Default is `1e8`.

              num\_attempts :
              :   `int` indicating the number of attempts the steady state
                  solver should make before determining that a steady state
                  cannot be found. Only valid for `strategy='nleq1'` or
                  `strategy='nleq2'`.

                  Default is `2`.

              decimal\_precision :
              :   `bool` indicating whether to apply the
                  `decimal_precision` attribute of
                  the `MassConfiguration` to the solution values.

                  Default is `False`.

        Returns
        :   - *tuple (conc\_solutions, flux\_solutions)*
            - **conc\_solutions** (*MassSolution or DictList*) – If only one model was simulated, the return type is
              a `MassSolution` containing the concentration solutions.
              If multiple models were simulated, the return type is a
              `DictList` of
              `MassSolution`s containing the concentration solutions.
              If a simulation failed, the corresponding `MassSolution`
              will be returned as empty.
            - **flux\_solutions** (*MassSolution or DictList*) – If only one model was simulated, the return type is
              a `MassSolution` containing the flux solutions.
              If multiple models were simulated, the return type is a
              `DictList` of
              `MassSolution`s containing the flux solutions.
              If a simulation failed, the corresponding `MassSolution`
              will be returned as empty.

        See also

        `integrator`
        :   Access the integrator utilized in the `"simulate"` strategy.

        `steady_state_solver`
        :   Access the steady state solver utilized in root finding strategies i.e. `"nleq1"` and `"nleq2"`.

    `_make_rr_selections`(*self*, *selections=None*, *include\_time=True*, *verbose=False*)[source]¶
    :   Set the observable output of the simulation.

        Warning

        This method is intended for internal use only.

    `_format_perturbations_input`(*self*, *perturbations*, *verbose=False*)[source]¶
    :   Check and format the perturbation input.

        Perturbations are checked before simulations are carried out to limit
        fails during the simulation due to bad syntax or values.

        Warning

        This method is intended for internal use only.

    `_set_simulation_values`(*self*, *model*, *perturbations*, *verbose=False*)[source]¶
    :   Set the simulation numerical values in the roadrunner instance.

        Warning

        This method is intended for internal use only.

    `_set_values_in_roadrunner`(*self*, *model*, *reset*, *sim\_values\_to\_set*)[source]¶
    :   Set the roadrunner values to reflect the given model.

        Warning

        This method is intended for internal use only.

    `_make_mass_solutions`(*self*, *model*, *selections*, *results*, *update\_values=False*, *\*\*kwargs*)[source]¶
    :   Make the MassSolutions using the results of the Simulation.

        Warning

        This method is intended for internal use only.

    `_update_stored_solutions`(*self*, *solution\_type*, *solutions*)[source]¶
    :   Update stored MassSolutions with new MassSolution objects.

        Warning

        This method is intended for internal use only.

    `_reset_roadrunner`(*self*, *reset*)[source]¶
    :   Reset the RoadRunner to its the original state.

        Warning

        This method is intended for internal use only.

    `_find_steady_state_simulate`(*self*, *model*, *\*\*kwargs*)[source]¶
    :   Find the steady state of a model through simulation of the model.

        Warning

        This method is intended for internal use only.

    `_find_steady_state_solver`(*self*, *model*, *\*\*kwargs*)[source]¶
    :   Find the steady state of the model using a RoadRunner solver method.

        Warning

        This method is intended for internal use only.

    `update_model_simulation_values`(*self*, *model*, *initial\_conditions=None*, *parameters=None*, *verbose=False*)[source]¶
    :   Update the simulation values for a given model.

        Parameters
        :   - **model** (*MassModel* *or* *its string identifier.*) – A previously loaded MassModel.
            - **initial\_conditions** (*dict* *or* *None*) – A `dict` containing initial conditions to update.
            - **parameters** (*dict* *or* *None*) – A `dict` containing parameters to update. If `None` provided,
              will attempt to extract values from the given MassModel

    `_update_mass_model_with_values`(*self*, *mass\_model*, *value\_dict=None*)[source]¶
    :   Update the MassModel object with the stored model values.

        Warning

        This method is intended for internal use only.

    `_get_all_values_for_sim`(*self*, *mass\_model*)[source]¶
    :   Get all model values as a single dict.

        Warning

        This method is intended for internal use only.

    `_add_model_values_to_simulation`(*self*, *model*, *verbose*)[source]¶
    :   Add model values to the simulation.

        Warning

        This method is intended for internal use only.

###### `mass.test`¶

Module containing functions for testing various `mass` methods.

There is the `test_all()` function to run all tests. Note that the testing
requirements must be installed (e.g. `pytest`) for this function to work.

There are also functions for viewing and loading pre-built example
`MassModel` objects via the `json` or
`sbml` submodules, as well as functions to view
Escher
maps that correspond to certain pre-defined models.

###### Package Contents¶

###### Functions¶

|  |  |
| --- | --- |
| `create_test_model`(model\_name, io=’json’) | Return a `mass.MassModel` for testing. |
| `view_test_models`() | Print the test models that can be loaded. |
| `view_test_maps`() | Print the test models that can be loaded. |
| `test_all`(args=None) | Alias for running all unit-tests on installed `mass`. |

`mass.test.``FILE_EXTENSIONS` *= ['.xml', '.json']*[source]¶
:   list of recognized file extensions.

    Type
    :   list

`mass.test.``MASS_DIR`[source]¶
:   The directory location of where `mass` is installed.

    Type
    :   str

`mass.test.``DATA_DIR`[source]¶
:   The directory location of the test data model files and maps.

    Type
    :   str

`mass.test.``MODELS_DIR`[source]¶
:   The directory location of the pre-built `MassModel` files.

    Type
    :   str

`mass.test.``MAPS_DIR`[source]¶
:   The directory location of the pre-made `escher` maps files.

    Type
    :   str

`mass.test.``create_test_model`(*model\_name*, *io='json'*)[source]¶
:   Return a `mass.MassModel` for testing.

    Parameters
    :   - **model\_name** (*str*) – The name of the test model to load. Valid model names can be printed
          and viewed using the `view_test_models()` function.
        - **io** (str `{'sbml', 'json'}`) – A string representing the `mass.io` module to use to load the
          model. Default is `"sbml"`. Case sensitive.

    Returns
    :   The loaded `MassModel`

    Return type
    :   MassModel

`mass.test.``view_test_models`()[source]¶
:   Print the test models that can be loaded.

`mass.test.``view_test_maps`()[source]¶
:   Print the test models that can be loaded.

`mass.test.``test_all`(*args=None*)[source]¶
:   Alias for running all unit-tests on installed `mass`.

###### `mass.thermo`¶

###### Subpackages¶

###### `mass.thermo.conc_sampling`¶

This module contains the Hit-and-Run concentration samplers.

Key components in the `thermo.conc_sampling` module are the following:

> 1. The `sample_concentrations()` function, a function
>    to call one of the concentration sampler methods and valid
>    concentration distributions from the :associated `mass` model
>    of the `ConcSolver`.
>    Currently provides two sampling methods:
>
>    > 1. `'optgp'` to utilize the `ConcOptGPSampler`
>    > 2. `'achr'` to utilize the `ConcACHRSampler`
> 2. The `ConcOptGPSampler` is parallel optimized sampler with
>    fast convergence and parallel execution based on [MHM14], with its
>    implementation similar to the Python `cobra` package.
>    See the `conc_optgp` documentation for more information.
> 3. The `ConcACHRSampler` is a sampler that utilizes an
>    Artifial Centering Hit-and-Run (ACHR) sampler for a low memory
>    footprint and good convergence based on [KS98], with its
>    implementation similar to the Python `cobra` package.
>    See the `conc_achr` documentation for more information.
> 4. The `ConcHRSampler` is the base class for the
>    samplers. All current samplers are derived from this class and all new
>    samplers should be derived from this class to provide a unified
>    interface for concentration sampling.
>    See the `conc_hr_sampler` documentation for more information.

To properly use the concentration samplers and associated functions, note
the following:

> - It is required that a model has been loaded into a `ConcSolver`
>   instance and that the solver has been setup for concentraiton sampling
>   via the `ConcSolver.setup_sampling_problem()` method.
> - All numerical values to be utilized by the solver must be defined. This
>   includes:
>
>   > - Metabolite concentrations for all included metabolites, accessed via
>   >   `MassMetabolite.initial_condition`.
>   > - Reaction equilibrium constants for all included reactions, accessed
>   >   via `MassReaction.equilibrium_constant`.
>   > - Reaction steady state fluxes for all included reactions, accessed
>   >   `MassReaction.steady_state_flux`.
> - In order to perform the sampling, all numerical values are transformed
>   from linear space into logarithmic space before the solver is populated.
>   Therefore, all variables and constraints in the solver will exist in
>   logspace.
>
>   However, all numerical solution values are transformed from logspace
>   back into a linear space before being returned, unless otherwise
>   specified.

###### Submodules¶

###### `mass.thermo.conc_sampling.conc_achr`¶

Provides concentration sampling through an ACHR sampler.

Based on sampling implementations in `cobra.sampling.achr`

###### Module Contents¶

###### Classes¶

|  |  |
| --- | --- |
| `ConcACHRSampler` | Artificial Centering Hit-and-Run sampler for concentration sampling. |

*class* `mass.thermo.conc_sampling.conc_achr.``ConcACHRSampler`(*concentration\_solver*, *thinning=100*, *nproj=None*, *seed=None*)[source]¶
:   Bases: `mass.thermo.conc_sampling.conc_hr_sampler.ConcHRSampler`

    Artificial Centering Hit-and-Run sampler for concentration sampling.

    A sampler with low memory footprint and good convergence [KS98].

    Notes

    ACHR generates samples by choosing new directions from the sampling space’s
    center and the warmup points. The implementation used here is the similar
    as in the Python `cobra` package.

    This implementation uses only the initial warmup points to generate new
    directions and not any other previous iterates. This usually gives better
    mixing since the startup points are chosen to span the space in a wide
    manner. This also makes the generated sampling chain quasi-markovian since
    the center converges rapidly.

    Memory usage is roughly in the order of:

    ```
    (number included reactions + number included metabolites)^2
    ```

    due to the required nullspace matrices and warmup points. So large
    models easily take up a few GB of RAM.

    Parameters
    :   - **concentration\_solver** (*ConcSolver*) – The `ConcSolver` to use in generating samples.
        - **thinning** (*int*) – The thinning factor for the generated sampling chain as a positive
          `int` > 0. A thinning factor of 10 means samples are returned every
          10 steps.
        - **nproj** (*int* *or* *None*) –

          A positive `int` > 0 indicating how often to reporject the sampling
          point into the feasibility space. Avoids numerical issues at the cost
          of lower sampling. If `None` then the value is determined via the
          following:

          ```
          nproj = int(min(len(self.concentration_solver.variables)**3, 1e6))
          ```

          Default is `None`
        - **seed** (*int* *or* *None*) –

          A positive `int` > 0 indiciating random number seed that should be
          used. If `None` provided, the current time stamp is used.

          Default is `None`.

    `concentration_solver`¶
    :   The `ConcSolver` used to generate samples.

        Type
        :   ConcSolver

    `feasibility_tol`¶
    :   The tolerance used for checking equalities feasibility.

        Type
        :   float

    `bounds_tol`¶
    :   The tolerance used for checking bounds feasibility.

        Type
        :   float

    `thinning`¶
    :   The currently used thinning factor.

        Type
        :   int

    `n_samples`¶
    :   The total number of samples that have been generated by this
        sampler instance.

        Type
        :   int

    `retries`¶
    :   The overall of sampling retries the sampler has observed. Larger
        values indicate numerical instabilities.

        Type
        :   int

    `problem`¶
    :   A `namedtuple` whose attributes define the entire
        sampling problem in matrix form. See docstring of
        `Problem` for more information.

        Type
        :   collections.namedtuple

    `warmup`¶
    :   A matrix of with as many columns as variables in the model of the
        `ConcSolver` and more than 3 rows containing a warmup
        sample in each row. `None` if no warmup points have been generated
        yet.

        Type
        :   numpy.matrix

    `nproj`[source]¶
    :   How often to reproject the sampling point into the feasibility space.

        Type
        :   int

    `sample`(*self*, *n*, *concs=True*)[source]¶
    :   Generate a set of samples.

        This is the basic sampling function for all hit-and-run samplers.

        Notes

        Performance of this function linearly depends on the number
        of variables in the model of the `ConcSolver`
        and the thinning factor.

        Parameters
        :   - **n** (*int*) – The number of samples that are generated at once.
            - **concs** (*bool*) – Whether to return concentrations or the internal solver variables.
              If `False` will return a variable for each metabolite and
              reaction equilibrium constant as well as all additional variables
              that may have been defined in the model of the
              `ConcSolver`.

        Returns
        :   A matrix with `n` rows, each containing a concentration sample.

        Return type
        :   numpy.matrix

    `__single_iteration`(*self*)[source]¶
    :   Perform a single iteration of sampling.

        Warning

        This method is intended for internal use only.

###### `mass.thermo.conc_sampling.conc_hr_sampler`¶

Provide base class for Hit-and-Run concentration samplers.

New samplers should derive from the abstract `ConcHRSampler` class
where possible to provide a uniform interface.”“

Based on sampling implementations in `cobra.sampling.hr_sampler`.

###### Module Contents¶

###### Classes¶

|  |  |
| --- | --- |
| `ConcHRSampler` | The abstract base class for hit and run concentration samplers. |

###### Functions¶

|  |  |
| --- | --- |
| `step`(sampler, x, delta, fraction=None, tries=0) | Sample new feasible point from point `x` in the direction `delta`. |

`mass.thermo.conc_sampling.conc_hr_sampler.``LOGGER`[source]¶
:   Logger for `conc_hr_sampler` submodule.

    Type
    :   logging.Logger

`mass.thermo.conc_sampling.conc_hr_sampler.``MAX_TRIES` *= 100*[source]¶
:   Maximum number of retries for sampling.

    Type
    :   int

*class* `mass.thermo.conc_sampling.conc_hr_sampler.``ConcHRSampler`(*concentration\_solver*, *thinning*, *nproj=None*, *seed=None*)[source]¶
:   The abstract base class for hit and run concentration samplers.

    Parameters
    :   - **concentration\_solver** (*ConcSolver*) – The `ConcSolver` to use in generating samples.
        - **thinning** (*int*) – The thinning factor for the generated sampling chain as a positive
          `int` > 0. A thinning factor of 10 means samples are returned every
          10 steps.
        - **nproj** (*int* *or* *None*) –

          A positive `int` > 0 indicating how often to reporject the sampling
          point into the feasibility space. Avoids numerical issues at the cost
          of lower samplimg. If `None` then the value is determined via the
          following:

          ```
          nproj = int(min(len(self.concentration_solver.variables)**3, 1e6))
          ```

          Default is `None`
        - **seed** (*int* *or* *None*) –

          A positive `int` > 0 indiciating random number seed that should be
          used. If `None` provided, the current time stamp is used.

          Default is `None`.

    `concentration_solver`¶
    :   The `ConcSolver` used to generate samples.

        Type
        :   ConcSolver

    `feasibility_tol`¶
    :   The tolerance used for checking equalities feasibility.

        Type
        :   float

    `bounds_tol`¶
    :   The tolerance used for checking bounds feasibility.

        Type
        :   float

    `thinning`¶
    :   The currently used thinning factor.

        Type
        :   int

    `n_samples`¶
    :   The total number of samples that have been generated by this
        sampler instance.

        Type
        :   int

    `retries`¶
    :   The overall of sampling retries the sampler has observed. Larger
        values indicate numerical instabilities.

        Type
        :   int

    `problem`¶
    :   A `namedtuple` whose attributes define the entire
        sampling problem in matrix form. See docstring of
        `Problem` for more information.

        Type
        :   collections.namedtuple

    `warmup`¶
    :   A matrix of with as many columns as reactions in the model and more
        than 3 rows containing a warmup sample in each row. None if no warmup
        points have been generated yet.

        Type
        :   numpy.matrix

    *property* `nproj`(*self*)[source]¶
    :   Get or set `nproj` value.

        Parameters
        :   **value** (*int* *or* *None*) –

            A positive `int` > 0 indicating how often to reporject the
            sampling point into the feasibility space. Avoids numerical issues
            at the cost of lower sampling. If `None` then the value is
            determined via the following:

            ```
            nproj = int(min(len(self.concentration_solver.variables)**3, 1e6))
            ```

    *property* `seed`(*self*)[source]¶
    :   Get or set `nproj` value.

        Parameters
        :   **value** (*int* *or* *None*) – A positive `int` > 0 indiciating random number seed that should
            be used. If `None` provided, the current time stamp is used.

    `generate_cva_warmup`(*self*)[source]¶
    :   Generate the warmup points for the sampler.

        Generates warmup points by setting each concentration as the sole
        objective and minimizing/maximizing it. Also caches the projection of
        the warmup points into the nullspace for non-homogenous problems.

    `sample`(*self*, *n*, *concs=True*)[source]¶
    :   Abstract sampling function.

        Should be overwritten by child classes.

    `batch`(*self*, *batch\_size*, *batch\_num*, *concs=True*)[source]¶
    :   Create a batch generator.

        This is useful to generate `n` batches of `m` samples each.

        Parameters
        :   - **batch\_size** (*int*) – The number of samples contained in each batch (`m`).
            - **batch\_num** (*int*) – The number of batches in the generator (`n`).
            - **concs** (*boolean*) – Whether to return concentrations or the internal solver variables.
              If `False` will return a variable for each metabolite and
              reaction Keq as well as all additional variables that may have
              been defined in the model.

        Yields
        :   *pandas.core.frame.DataFrame* – A `pandas.DataFrame` with dimensions `(batch_size x n_m)`
            containing a valid concentration sample for a total of
            `n_m` metabolites (or variables if `concs=False`) in each row.

    `_reproject`(*self*, *p*)[source]¶
    :   Reproject a point into the feasibility region.

        This function is guarunteed to return a new feasible point. However,
        no guaruntees in terms of proximity to the original point can be made.

        Parameters
        :   **p** (*numpy.ndarray*) – The current sample point.

        Returns
        :   A new feasible point. If p was feasible it wil return p.

        Return type
        :   numpy.ndarray

        Warning

        This method is intended for internal use only.

    `_random_point`(*self*)[source]¶
    :   Find an approximately random point in the concentration cone.

        Warning

        This method is intended for internal use only.

    `_is_redundant`(*self*, *matrix*, *cutoff=None*)[source]¶
    :   Identify redundant rows in a matrix that can be removed.

        Warning

        This method is intended for internal use only.

    `_bounds_dist`(*self*, *p*)[source]¶
    :   Get the lower and upper bound distances. Negative is bad.

        Warning

        This method is intended for internal use only.

    `__build_problem`(*self*)[source]¶
    :   Build the matrix representation of the sampling problem.

        Warning

        This method is intended for internal use only.

`mass.thermo.conc_sampling.conc_hr_sampler.``step`(*sampler*, *x*, *delta*, *fraction=None*, *tries=0*)[source]¶
:   Sample new feasible point from point `x` in the direction `delta`.

    Has to be declared outside of class to be used for multiprocessing

    Parameters
    :   - **sampler** (*ConcHRSampler*) – The sampler instance.
        - **x** (*float*) – The starting point from which to sample.
        - **delta** (*float*) – The direction to travel from the point at `x`.
        - **fraction** (*float* *or* *None*) – The fraction of the alpha range to use in determining alpha.
          If `None` then the `np.random.uniform()` function to get alpha.
        - **tries** (*int*) – Number of tries. If the number of tries is greater than the
          `MAX_TRIES`, a `RuntimeError` will be raised.

    Returns
    :   The new feasible point.

    Return type
    :   float

    Raises
    :   **RunTimeError** – Raised when `tries > MAX_TRIES`

###### `mass.thermo.conc_sampling.conc_optgp`¶

Provides concentration sampling through an OptGP sampler.

Based on sampling implementations in `cobra.sampling.optgp`

###### Module Contents¶

###### Classes¶

|  |  |
| --- | --- |
| `ConcOptGPSampler` | A parallel optimized sampler. |

*class* `mass.thermo.conc_sampling.conc_optgp.``ConcOptGPSampler`(*concentration\_solver*, *processes=None*, *thinning=100*, *nproj=None*, *seed=None*)[source]¶
:   Bases: `mass.thermo.conc_sampling.conc_hr_sampler.ConcHRSampler`

    A parallel optimized sampler.

    A parallel sampler with fast convergence and parallel execution
    [MHM14].

    Notes

    The sampler is very similar to artificial centering where each process
    samples its own chain. The implementation used here is the similar
    as in the Python `cobra` package.

    Initial points are chosen randomly from the warmup points followed by a
    linear transformation that pulls the points a little bit towards the
    center of the sampling space.

    If the number of processes used is larger than the one requested,
    number of samples is adjusted to the smallest multiple of the number of
    processes larger than the requested sample number. For instance, if you
    have 3 processes and request 8 samples you will receive 9.

    Memory usage is roughly in the order of:

    ```
    (number included reactions + number included metabolites)^2
    ```

    due to the required nullspace matrices and warmup points. So large
    models easily take up a few GB of RAM. However, most of the large matrices
    are kept in shared memory. So the RAM usage is independent of the number
    of processes.

    Parameters
    :   - **concentration\_solver** (*ConcSolver*) – The `ConcSolver` to use in generating samples.
        - **thinning** (*int*) – The thinning factor for the generated sampling chain as a positive
          `int` > 0. A thinning factor of 10 means samples are returned every
          10 steps.
        - **processes** (*int* *or* *None*) –

          The number of processes used to generate samples. If `None` the
          number of processes specified in the `MassConfiguration` is
          utilized. Only valid for `method='optgp'`.

          Default is `None`.
        - **nproj** (*int* *or* *None*) –

          A positive `int` > 0 indicating how often to reporject the sampling
          point into the feasibility space. Avoids numerical issues at the cost
          of lower samplimg. If `None` then the value is determined via the
          following:

          ```
          nproj = int(min(len(self.concentration_solver.variables)**3, 1e6))
          ```

          Default is `None`
        - **seed** (*int* *or* *None*) –

          A positive `int` > 0 indiciating random number seed that should be
          used. If `None` provided, the current time stamp is used.

          Default is `None`.

    `concentration_solver`¶
    :   The `ConcSolver` used to generate samples.

        Type
        :   ConcSolver

    `feasibility_tol`¶
    :   The tolerance used for checking equalities feasibility.

        Type
        :   float

    `bounds_tol`¶
    :   The tolerance used for checking bounds feasibility.

        Type
        :   float

    `thinning`¶
    :   The currently used thinning factor.

        Type
        :   int

    `n_samples`¶
    :   The total number of samples that have been generated by this
        sampler instance.

        Type
        :   int

    `retries`¶
    :   The overall of sampling retries the sampler has observed. Larger
        values indicate numerical instabilities.

        Type
        :   int

    `problem`¶
    :   A `namedtuple` whose attributes define the entire
        sampling problem in matrix form. See docstring of
        `Problem` for more information.

        Type
        :   collections.namedtuple

    `warmup`¶
    :   A matrix of with as many columns as variables in the model of the
        `ConcSolver` and more than 3 rows containing a warmup
        sample in each row. `None` if no warmup points have been generated
        yet.

        Type
        :   numpy.matrix

    `nproj`[source]¶
    :   How often to reproject the sampling point into the feasibility space.

        Type
        :   int

    `sample`(*self*, *n*, *concs=True*)[source]¶
    :   Generate a set of samples.

        This is the basic sampling function for all hit-and-run samplers.

        Notes

        Performance of this function linearly depends on the number
        of metabolites in your model and the thinning factor.

        If the number of processes is larger than one, computation is split
        across as the CPUs of your machine. This may shorten computation time.

        However, there is also overhead in setting up parallel computation so
        it is recommended to calculate large numbers of samples at once
        (`n` > 1000).

        Parameters
        :   - **n** (*int*) – The number of samples that are generated at once.
            - **concs** (*boolean*) – Whether to return concentrations or the internal solver variables.
              If `False` will return a variable for each metabolite and
              reaction Keq as well as all additional variables that may have
              been defined in the model.

        Returns
        :   A matrix with `n` rows, each containing a concentration sample.

        Return type
        :   numpy.matrix

    `__getstate__`(*self*)[source]¶
    :   Return the object for serialization.

        Warning

        This method is intended for internal use only.

###### `mass.thermo.conc_sampling.conc_sampling`¶

Module implementing concentration sampling for `mass` models.

Based on sampling implementations in `cobra.sampling.sampling`

###### Module Contents¶

###### Functions¶

|  |  |
| --- | --- |
| `sample_concentrations`(concentration\_solver, n, method=’optgp’, thinning=100, processes=1, seed=None) | Sample valid concentration distributions from a `mass` model. |

`mass.thermo.conc_sampling.conc_sampling.``sample_concentrations`(*concentration\_solver*, *n*, *method='optgp'*, *thinning=100*, *processes=1*, *seed=None*)[source]¶
:   Sample valid concentration distributions from a `mass` model.

    This function samples valid concentration distributions from a
    `mass` model using a `ConcSolver`.

    Currently supports two methods.

    1. `'optgp'` which uses the `ConcOptGPSampler` that supports
       parallel sampling [MHM14]. Requires large numbers of samples to be
       performant (n > 1000). For smaller samples, `'achr'` might be better
       suited.
    2. `'achr'` which uses artificial centering hit-and-run via the
       `ConcACHRSampler`. This is a single process method with
       good convergence [KS98].

    Parameters
    :   - **concentration\_solver** (*ConcSolver*) – The `ConcSolver` to use in generating samples.
        - **n** (*int*) – The number of samples to obtain. When using `'method=optgp'`, this
          must be a multiple of `processes`, otherwise a larger number of
          samples will be returned.
        - **method** (*str*) – The sampling algorithm to use. Default is `'optgp'`.
        - **thinning** (*int*) –

          The thinning factor for the generated sampling chain as a positive
          `int` > 0. A thinning factor of 10 means samples are returned every
          10 steps. If set to one, all iterates are returned.

          Default is `100`.
        - **processes** (*int* *or* *None*) –

          The number of processes used to generate samples. If `None` the
          number of processes specified in the `MassConfiguration` is
          utilized. Only valid for `method='optgp'`.

          Default is `1`.
        - **seed** (*int* *or* *None*) –

          A positive `int` > 0 indiciating random number seed that should be
          used. If `None` provided, the current time stamp is used.

          Default is `None`.

    Returns
    :   The generated concentration samples. Each row corresponds to a sample
        of the concentrations and the columns are the metabolites.

    Return type
    :   pandas.DataFrame

    See also

    `ConcSolver.setup_sampling_problem()`
    :   For setup of the sampling problem in the given `ConcSolver`.

###### Submodules¶

###### `mass.thermo.conc_solution`¶

Provide unified interfaces for optimization solutions for concentrations.

Based on solution implementations in `cobra.core.solution`

###### Module Contents¶

###### Classes¶

|  |  |
| --- | --- |
| `ConcSolution` | A unified interface to a `ConcSolver` optimization solution. |

###### Functions¶

|  |  |
| --- | --- |
| `get_concentration_solution`(concentration\_solver, metabolites=None, reactions=None, raise\_error=False, \*\*kwargs) | Generate a solution representation of a `ConcSolver` state. |
| `update_model_with_concentration_solution`(model, concentration\_solution, concentrations=True, Keqs=True, inplace=True) | Update a `mass` model with values from a `ConcSolution`. |

*class* `mass.thermo.conc_solution.``ConcSolution`(*objective\_value*, *status*, *concentrations*, *Keqs*, *concentration\_reduced\_costs=None*, *Keq\_reduced\_costs=None*, *shadow\_prices=None*)[source]¶
:   A unified interface to a `ConcSolver` optimization solution.

    Notes

    The `ConcSolution` is meant to be constructed by
    `get_concentration_solution()` please look at that function to fully
    understand the `ConcSolution` class.

    `objective_value`¶
    :   The (optimal) value for the objective function.

        Type
        :   float

    `status`¶
    :   The solver status related to the solution.

        Type
        :   str

    `concentrations`¶
    :   Contains the metabolite concentrations which are the primal values
        of metabolite variables.

        Type
        :   pandas.Series

    `concentration_reduced_costs`¶
    :   Contains metabolite reduced costs, which are the dual values of
        metabolites variables.

        Type
        :   pandas.Series

    `Keqs`¶
    :   Contains the reaction equilibrium constant values, which are primal
        values of Keq variables.

        Type
        :   pandas.Series

    `Keq_reduced_costs`¶
    :   Contains reaction equilibrium constant reduced costs, which are the
        dual values of Keq variables.

        Type
        :   pandas.Series

    `shadow_prices`¶
    :   Contains reaction shadow prices (dual values of constraints).

        Type
        :   pandas.Series

    `get_primal_by_id`[source]¶

    `concentrations_to_frame`(*self*)[source]¶
    :   Get a `pandas.DataFrame` of concs. and reduced costs.

    `Keqs_to_frame`(*self*)[source]¶
    :   Get a `pandas.DataFrame` of Keqs and reduced costs.

    `to_frame`(*self*)[source]¶
    :   Get a `pandas.DataFrame` of variables and reduced costs.

    `_repr_html_`(*self*)[source]¶
    :   HTML representation of the overview for the ConcSolution.

        Warning

        This method is intended for internal use only.

    `__repr__`(*self*)[source]¶
    :   Set string representation of the solution instance.

        Warning

        This method is intended for internal use only.

    `__getitem__`(*self*, *variable*)[source]¶
    :   Return the value of a metabolite concentration or reaction Keq.

        Parameters
        :   **variable** (*str*) – A variable ID for a variable in the solution.

        Warning

        This method is intended for internal use only.

    `__dir__`(*self*)[source]¶
    :   Override default dir() implementation to list only public items.

        Warning

        This method is intended for internal use only.

`mass.thermo.conc_solution.``get_concentration_solution`(*concentration\_solver*, *metabolites=None*, *reactions=None*, *raise\_error=False*, *\*\*kwargs*)[source]¶
:   Generate a solution representation of a `ConcSolver` state.

    Parameters
    :   - **concentration\_solver** (*ConcSolver*) – The `ConcSolver` containing the mathematical problem solved.
        - **metabolites** (*list*) – An iterable of `MassMetabolite` objects.
          Uses `ConcSolver.included_metabolites` by default.
        - **reactions** (*list*) – An iterable of `MassReaction` objects.
          Uses `ConcSolver.included_reactions` by default.
        - **raise\_error** (*bool*) – Whether to raise an OptimizationError if solver status is not optimal.
        - **\*\*kwargs** –

          decimal\_precision :
          :   `bool` indicating whether to apply the
              `decimal_precision` attribute of
              the `MassConfiguration` to the solution values.

              Default is `False`.

    Returns
    :   The solution of the optimization as a `ConcSolution` object.

    Return type
    :   ConcSolution

`mass.thermo.conc_solution.``update_model_with_concentration_solution`(*model*, *concentration\_solution*, *concentrations=True*, *Keqs=True*, *inplace=True*)[source]¶
:   Update a `mass` model with values from a `ConcSolution`.

    Parameters
    :   - **model** (*MassModel*) – A `mass` model to update with the new solution values.
        - **concentration\_solution** (*ConcSolution*) – The `ConcSolution` containing the solution values to use.
        - **concentrations** (*bool*) – Whether to update the metabolite concentrations of the model
          (the `MassMetabolite.initial_condition` values).
        - **Keqs** (*bool*) – Whether to update the reaction equilibrium constants of the model
          (the `MassReaction.equilibrium_constant` values).
        - **inplace** (*bool*) – Whether to modify the given model or to modify a copy of the model.

    Returns
    :   Either the given model if `inplace=True`, or a new copy of the model
        `inplace=False`.

    Return type
    :   MassModel

###### `mass.thermo.conc_solver`¶

Module handling `optlang.interface.Model` for concentration problems.

The purpose of the `ConcSolver` is to provide an interface to assist
with setting up various problems involving optimization-like problems
involving metabolite concentrations. Note that all internal solver variables
exist in logarithmic space and therefore all associated numerical values are
transformed from linear space into log space before being added to the solver.
Unless specified otherwise, all numerical solutions will be transformed back
into linear space from logspace before being returned.

Upon initialization, a generic problem is created, represented by an
`optlang.interface.Model`. The generic problem includes variables for
the metabolite concentration and reaction equilibrium constants, along with
thermodynamic constraints for each reaction with the direction of the
constraint (i.e. greater/less than) dependent on the sign of the steady
state flux.

In addition to creating a generic problem, the `ConcSolver` has the
following functions available to create a specific type of problem:

> - `ConcSolver.setup_feasible_qp_problem()`
> - `ConcSolver.setup_sampling_problem()`

If custom objectives, constraints and/or variables are to be used with the
solver, it is recommended to run one of the above functions first to setup the
problem, and then tailor the solver with customizations for that problem.

Notes

- An `optlang.interface.Model` represents an optimization problem and
  contains the variables, constraints, and objectives that make up the problem.
  See the `optlang` documentation for more information.
- All numerical values to be utilized by the solver must be defined. This
  includes:

  > - Metabolite concentrations for all included metabolites, accessed via
  >   `MassMetabolite.initial_condition`.
  > - Reaction equilibrium constants for all included reactions, accessed
  >   via `MassReaction.equilibrium_constant`.
  > - Reaction steady state fluxes for all included reactions, accessed
  >   `MassReaction.steady_state_flux`.

###### Module Contents¶

###### Classes¶

|  |  |
| --- | --- |
| `ConcSolver` | Class providing an interface for concentration mathematical problems. |

###### Functions¶

|  |  |
| --- | --- |
| `concentration_constraint_matricies`(concentration\_solver, array\_type=’dense’, zero\_tol=1e-06) | Create a matrix representation of the problem. |

*class* `mass.thermo.conc_solver.``ConcSolver`(*model*, *excluded\_metabolites=None*, *excluded\_reactions=None*, *equilibrium\_reactions=None*, *constraint\_buffer=0*, *\*\*kwargs*)[source]¶
:   Class providing an interface for concentration mathematical problems.

    Upon initialization, a generic problem is created, represented by an
    `optlang.Model`. The generic problem includes variables for the
    metabolite concentration and reaction equilibrium constants, along with
    thermodynamic constraints for each reaction with the direction of the
    constraint (i.e. greater/less than) dependent on the sign of the steady
    state flux.

    Notes

    - All internal solver variables exist in logarithmic space and therefore
      all associated numerical values are transformed from linear space into
      log space before being added to the solver.
    - Unless specified otherwise, all numerical solutions will be transformed
      back into linear space from logspace before being returned.
    - Boundary reactions (a.k.a. reactions with only one metabolite involved)
      are excluded automatically. To work with reactions that cross compartment
      boundaries, `MassMetabolite` objects need to be defined for
      the metabolites in both compartments.

    Parameters
    :   - **model** (*MassModel*) – The `mass` model to associated with the `ConcSolver`
          instance. The model is used to populate the solver with typical
          variables and constraints upon initialization.
        - **excluded\_metabolites** (*iterable* *or* *None*) – An iterable of model `MassMetabolite` objects or their
          identifiers in populating the solver with metabolite concentration
          variables and reaction constraints. If `None`, no metabolites are
          excluded.
        - **excluded\_reactions** (*iterable* *or* *None*) – An iterable of model `MassReaction` objects or their
          identifiers in populating the solver with reaction equilibrium constant
          variables and reaction constraints. If `None`, no reactions are
          excluded.
        - **equilibrium\_reactions** (*iterable* *or* *None*) – An iterable of model `MassReaction` objects or their
          identifiers that are intended to be at equilibrium. Reactions with
          steady state flux values equal to 0. are typically ignored unless
          they are specified in the `ConcSolver.equilibrium_reactions`
        - **constraint\_buffer** (*float* *or* *None*) –

          A `float` value to use as a constraint buffer for all constraints.

          Default is 0..
        - **\*\*kwargs** –

          exclude\_infinite\_Keqs :
          :   `bool` indicating whether to exclude reactions with equilibrium
              constant values of infinity from the concentration solver.

              Default is `True`.

          fixed\_conc\_bounds :
          :   An iterable containing metabolites whose concentrations are
              to be set as fixed variables, meaning that their lower and
              upper bounds are equal to the base value.

          fixed\_Keq\_bounds :
          :   An iterable containing reactions whose equilibrium constants
              are to be set as fixed variables, meaning that their lower and
              upper bounds are equal to the base value.

          decimal\_precision :
          :   `bool` indicating whether to apply the
              `decimal_precision` attribute of
              the `MassConfiguration` to the bound values.

              Default is `False`.

          zero\_value\_log\_substitute :
          :   `float` value to substitute for 0 when trying to take the
              logarithm of 0 to avoid a domain error.

              Default is `1e-10`.

    `problem_type`¶
    :   The type of mathematical problem that the concentration solver has
        been setup to solve.

        Type
        :   str

    `excluded_metabolites`¶
    :   A `list` of metabolite identifiers for model metabolites to exclude
        in populating the solver with metabolite concentration variables and
        reaction constraints.

        Type
        :   list

    `excluded_reactions`¶
    :   A `list` of reaction identifiers for model reactions to exclude
        in populating the solver with reaction equilibrium constant variables
        and reaction constraints.

        Type
        :   list

    `equilibrium_reactions`¶
    :   A `list` of reaction identifiers for model reactions that are
        intended to be at equilibrium.

        Type
        :   list

    `constraint_buffer`¶
    :   A value to utilize when setting a constraint buffer.

        Type
        :   float

    *property* `model`(*self*)[source]¶
    :   Return the model associated with the `ConcSolver`.

    *property* `solver`(*self*)[source]¶
    :   Get or set the attached solver of the `ConcSolver`.

        When using a `HistoryManager` context,
        this attribute can be set temporarily, reversed when the exiting
        the context.

        Parameters
        :   **value** (*str*) – The optimization solver for problems concerning metabolite
            concentrations. The solver choices are the ones provided by
            `optlang` and solvers installed in your environment. Valid
            solvers typically include: `"glpk"`, `"cplex"`, `"gurobi"`

        Notes

        - Like the `Model.solver`
          attribute, the concentration solver instance is the associated
          solver object, which manages the interaction with the associated
          solver, e.g. glpk.
        - This property is useful for accessing the concentration optimization
          problem directly and for defining additional constraints.

    *property* `tolerance`(*self*)[source]¶
    :   Get or set the tolerance for the solver of the `ConcSolver`.

        When using a `HistoryManager` context,
        this attribute can be set temporarily, reversed when the exiting
        the context.

        Parameters
        :   **value** (*float*) – The tolerance of the concentration solver.

    *property* `objective`(*self*)[source]¶
    :   Get or set the solver objective.

        When using a `HistoryManager` context,
        this attribute can be set temporarily, reversed when the exiting
        the context.

        Parameters
        :   **value** (*dict**,* *str**,* *int**,* *MassMetabolite**,* *Objective**, or* *Basic*) –

            The following are allowable values to set as the objective.

            > - `dict` where metabolites are keys, linear coefficients as
            >   values.
            > - `str` identifier of a `MassMetabolite` or the
            >   metabolite object itself.
            > - `int` metabolite index in `MassModel.metabolites`
            > - An `Objective` or a `sympy`
            >   expression to be directly interpreted as objectives.

    *property* `objective_direction`(*self*)[source]¶
    :   Get or set the objective direction.

        When using a `HistoryManager` context,
        this attribute can be set temporarily, reversed when the exiting
        the context.

        Parameters
        :   **value** (*str*) – The objective direction. Can be either `"max"` for the maximum,
            or `"min"` for the minimum.

    *property* `problem`(*self*)[source]¶
    :   Return the interface to the underlying mathematical problem.

        Solutions to the `ConcSolver` are obtained by formulating a
        mathematical problem and solving it. The `optlang` package is
        used to accomplish that and with this property, the problem interface
        can be accessed directly.

        Returns
        :   The problem interface that defines methods for interacting with
            the problem and associated solver directly.

        Return type
        :   optlang.interface

    *property* `variables`(*self*)[source]¶
    :   Return the mathematical variables in the `ConcSolver`.

        In a `ConcSolver`, most variables are metabolites and reaction
        equilibrium constants. However, for specific use cases, it may also be
        useful to have other types of variables. This property defines all
        variables currently associated with the underlying problem of the
        `ConcSolver`.

        Notes

        - All variables exist in logspace.

        Returns
        :   A container with all associated variables.

        Return type
        :   optlang.container.Container

    *property* `constraints`(*self*)[source]¶
    :   Return the constraints in the `ConcSolver`.

        In a `ConcSolver`, most constraints are thermodynamic
        constraints relating the reaction equilibrium constant to the reaction
        metabolite concentrations.However, for specific use cases, it may also
        be useful to have other types of constraints. This property defines
        all constraints currently associated with the underlying problem of
        the `ConcSolver`.

        Notes

        - All constraints exist in logspace.

        Returns
        :   A container with all associated constraints.

        Return type
        :   optlang.container.Container

    *property* `included_metabolites`(*self*)[source]¶
    :   Return a `list` of metabolite identifiers included in the solver.

        These are the metabolites not in the
        `ConcSolver.excluded_metabolites` attribute.

    *property* `included_reactions`(*self*)[source]¶
    :   Return a `list` of reaction identifiers included in the solver.

        These are the reactions not in the
        `ConcSolver.excluded_reactions` attribute.

    *property* `zero_value_log_substitute`(*self*)[source]¶
    :   Get or set the a value to substitute for 0 when taking the log of 0.

        A value of `1e-10` means that instead of attempting `log(0)` which
        causes a `ValueError`, it will be instead calculated as
        `log(1e-10)`.

        Parameters
        :   **value** (*float*) – A positive value to use instead of 0 when taking the logarithm.

    `setup_sampling_problem`(*self*, *metabolites=None*, *reactions=None*, *conc\_percent\_deviation=0.2*, *Keq\_percent\_deviation=0.2*, *\*\*kwargs*)[source]¶
    :   Set up the solver’s mathematical problem for concentraiton sampling.

        Notes

        - This involves changing solver variable bounds based on the percent
          deviation of the base value, removing the objective, and setting the
          `problem_type` to `"sampling"`.
        - If a percent deviation value is large enough to create a negative
          lower bound, it is set as the
          `zero_value_log_substitute` value
          to ensure that returned values are not negative.

        Parameters
        :   - **metabolites** (*iterable* *or* *None*) – An iterable of metabolites whose concentration variable bounds
              are to be changed. If `None`, all metabolites except those that
              are in the `ConcSolver.excluded_metabolites` list are used.
            - **reactions** (*iterable* *or* *None*) – An iterable of reactions whose equilibrium constant variable bounds
              are to be changed. If `None`, all reactions except those that
              are in the `ConcSolver.excluded_reactions` list are used.
            - **conc\_percent\_deviation** (*float*) –

              A non-negative number indicating the percent to deviate from the
              initial concentration to set as the lower and upper bounds for
              sampling.

              If a value of 0. is given, all given reaction equilibrium
              constants are set as fixed variables. Default is `0.2` for
              a 20% deviation from the base value.
            - **Keq\_percent\_deviation** (*float*) –

              A non-negative number indicating the percent to deviate from the
              base reaction equilibrium constant to set as the lower and
              upper bounds for sampling.

              If a value of 0. is given, all given reaction equilibrium
              constants are set as fixed variables. Default is `0.2` for
              a 20% deviation from the base value.
            - **\*\*kwargs** –

              fixed\_conc\_bounds :
              :   An iterable containing metabolites whose concentrations are
                  to be set as fixed variables, meaning that their lower and
                  upper bounds are equal to the base value.

              fixed\_Keq\_bounds :
              :   An iterable containing reactions whose equilibrium constants
                  are to be set as fixed variables, meaning that their lower and
                  upper bounds are equal to the base value.

              decimal\_precision :
              :   `bool` indicating whether to apply the
                  `decimal_precision` attribute of
                  the `MassConfiguration` to the bound values.

                  Default is `False`.

    `setup_feasible_qp_problem`(*self*, *metabolites=None*, *reactions=None*, *\*\*kwargs*)[source]¶
    :   Set up the solver’s mathematical problem for feasible conc. QP.

        Notes

        - This involves changing solver variable bounds to `[0, inf]`,
          setting the objective as a QP problem, and setting the
          `problem_type` to `"feasible_qp"`.
        - The `ConcSolver.solver` must have QP capabilities.

        Parameters
        :   - **metabolites** (*iterable* *or* *None*) – An iterable of metabolites whose concentration variable bounds
              are to be changed. If `None`, all metabolites except those that
              are in the `ConcSolver.excluded_metabolites` list are used.
            - **reactions** (*iterable* *or* *None*) – An iterable of reactions whose equilibrium constant variable bounds
              are to be changed. If `None`, all reactions except those that
              are in the `ConcSolver.excluded_reactions` list are used.
            - **\*\*kwargs** –

              fixed\_conc\_bounds :
              :   An iterable containing metabolites whose concentrations are
                  to be set as fixed variables, meaning that their lower and
                  upper bounds are equal to the base value.

              fixed\_Keq\_bounds :
              :   An iterable containing reactions whose equilibrium constants
                  are to be set as fixed variables, meaning that their lower and
                  upper bounds are equal to the base value.

              decimal\_precision :
              :   `bool` indicating whether to apply the
                  `decimal_precision` attribute of
                  the `MassConfiguration` to the bound values.

                  Default is `False`.

        Raises
        :   **TypeError** – Raised when the current solver does not have QP capabilities

        See also

        `choose_solver()`
        :   Method to choose a solver with QP capabilities

    `choose_solver`(*self*, *solver=None*, *qp=False*)[source]¶
    :   Choose a solver given a solver name.

        This will choose a solver compatible with the
        `ConcSolver` and required capabilities.

        Also respects `ConcSolver.solver` where it can.

        Parameters
        :   - **solver** (*str*) – The name of the solver to be used.
            - **qp** (*boolean*) – Whether the solver needs Quadratic Programming capabilities.
              Default is `False`.

        Returns
        :   **solver** – Returns a valid solver for the problem.

        Return type
        :   optlang.interface.Model

        Raises
        :   **SolverNotFound** – If no suitable solver could be found.

    `add_cons_vars`(*self*, *what*, *\*\*kwargs*)[source]¶
    :   Add constraints and variables to the solver’s problem.

        Useful for variables and constraints that cannot be expressed through
        metabolite concentrations and reaction equilibrium constants.

        Additions are reversed upon exit if the solver itself is used as
        context.

        Parameters
        :   - **what** (*list**,* *tuple*) – Either a `list` or a `tuple` of variables or constraints to
              add to the solver. Must be of `optlang.interface.Variable`
              or `optlang.interface.Constraint`.
            - **\*\*kwargs** (*keyword arguments*) – Passed to solver.add().

    `remove_cons_vars`(*self*, *what*)[source]¶
    :   Remove constraints and variables from the solver’s problem.

        Remove variables and constraints that were added directly to the
        solver’s underlying mathematical problem. Removals are reversed
        upon exit if the model itself is used as context.

        Parameters
        :   **what** (*list**,* *tuple*) – Either a `list` or a `tuple` of variables or constraints to
            remove from the solver. Must be of
            `optlang.interface.Variable` or
            `optlang.interface.Constraint`.

    `reset_constraints`(*self*)[source]¶
    :   Reset the constraints.

        Ensures constraints are updated if solver’s problem changes in any way.

    `add_excluded_metabolites`(*self*, *metabolites*, *reset\_problem=False*)[source]¶
    :   Add metabolites to the exclusion list for problem creation.

        Note that this will not remove metabolites from the current problem.
        The problem must first be reset in order for changes to take effect.

        Parameters
        :   - **metabolites** (*iterable*) – An iterable of `MassMetabolite` objects or their
              identifiers to be added to the
              `excluded_metabolites`.
            - **reset\_problem** (*bool*) – Whether to reset the underlying mathematical problem of the solver
              to a generic one after adding additional metabolites to exclude.
              If `False` then it is incumbent upon the user to remove the
              newly excluded metabolites from the solver.

    `remove_excluded_metabolites`(*self*, *metabolites*, *reset\_problem=False*)[source]¶
    :   Remove metabolites from the exclusion list for problem creation.

        Note that this will not add metabolites to the current problem.
        The problem must first be reset in order for changes to take effect.

        Parameters
        :   - **metabolites** (*iterable*) – An iterable of `MassMetabolite` objects or their
              identifiers to be removed from the
              `excluded_metabolites`.
            - **reset\_problem** (*bool*) – Whether to reset the underlying mathematical problem of the solver
              to a generic one after removing additional metabolites to exclude.
              If `False` then it is incumbent upon the user to add the
              newly included metabolites to the solver.

    `add_excluded_reactions`(*self*, *reactions*, *reset\_problem=False*)[source]¶
    :   Add reactions to the exclusion list for problem creation.

        Note that this will not remove reaction equilibrium constants or
        constraints from the current problem. The problem must first be reset
        in order for changes to take effect.

        Parameters
        :   - **reactions** (*iterable*) – An iterable of `MassReaction` objects or their
              identifiers to be added to the
              `excluded_reactions`.
            - **reset\_problem** (*bool*) – Whether to reset the underlying mathematical problem of the solver
              to a generic one after adding additional reactions to exclude.
              If `False` then it is incumbent upon the user to remove the
              newly excluded reactions from the solver.

    `remove_excluded_reactions`(*self*, *reactions*, *reset\_problem=False*)[source]¶
    :   Remove reactions from the exclusion list for problem creation.

        Note that this will not add reaction equilibrium constants or
        constraints to the current problem. The problem must first be reset
        in order for changes to take effect.

        Parameters
        :   - **reactions** (*iterable*) – An iterable of `MassReaction` objects or their
              identifiers to be removed from the
              `excluded_reactions`.
            - **reset\_problem** (*bool*) – Whether to reset the underlying mathematical problem of the solver
              to a generic one after removing additional reactions to exclude.
              If `False` then it is incumbent upon the user to add the
              newly included reaction equilibrium constants and constraints
              to the solver.

    `add_equilibrium_reactions`(*self*, *reactions*, *reset\_problem=False*)[source]¶
    :   Add additional reaction to the equilibrium reaction list.

        The problem must first be reset in order for changes to take effect
        as a result of adding additional equilibrium reactions.

        Parameters
        :   - **reactions** (*iterable*) – An iterable of `MassReaction` objects or their
              identifiers to be added to the
              `equilibrium_reactions`.
            - **reset\_problem** (*bool*) – Whether to reset the underlying mathematical problem of the solver
              to a generic one after adding additional equilibrium reactions.
              If `False` then it is incumbent upon the user to make the
              changes necessary for the mathematical problem of the solver.

    `remove_equilibrium_reactions`(*self*, *reactions*, *reset\_problem=False*)[source]¶
    :   Remove reactions from the equilibrium reaction list.

        The problem must first be reset in order for changes to take effect
        as a result of removing equilibrium reactions.

        Parameters
        :   - **reactions** (*iterable*) – An iterable of `MassReaction` objects or their
              identifiers to be removed from the
              `equilibrium_reactions`.
            - **reset\_problem** (*bool*) – Whether to reset the underlying mathematical problem of the solver
              to a generic one after removing equilibrium reactions.
              If `False` then it is incumbent upon the user to make the
              changes necessary for the mathematical problem of the solver.

    `optimize`(*self*, *objective\_sense=None*, *raise\_error=False*, *\*\*kwargs*)[source]¶
    :   Optimize the `ConcSolver`.

        Notes

        Only the most commonly used parameters are presented here. Additional
        parameters for solvers may be available and specified with the
        appropriate keyword argument.

        Parameters
        :   - **objective\_sense** (*str* *or* *None*) – Either `"maximize"` or `"minimize"` indicating whether
              variables should be maximized or minimized. In case of `None`,
              the previous direction is used.
            - **raise\_error** (*bool*) – If `True`, raise an `OptimizationError`
              if solver status is not optimal.
            - **\*\*kwargs** –

              decimal\_precision :
              :   `bool` indicating whether to apply the
                  `decimal_precision` attribute of
                  the `MassConfiguration` to the solution values.

                  Default is `False`.

    `slim_optimize`(*self*, *error\_value=float('nan')*, *message=None*, *\*\*kwargs*)[source]¶
    :   Optimize model without creating a `ConcSolution` object.

        Creating a full solution object implies fetching shadow prices and
        solution values for all variables and constraints in the solver.

        This necessarily takes some time and in cases where only one
        or two values are of interest, it is recommended to instead use this
        function which does not create a solution object, returning only the
        value of the objective.

        Note however that the `slim_optimize()` method uses
        efficient means to fetch values so if you need solution values or
        shadow prices for more than say 4 metabolites/reactions, then the
        total speed increase of `slim_optimize()` versus
        `optimize()` is expected to be small or even negative
        depending on other methods of fetching values after optimization.

        Parameters
        :   - **error\_value** (*float* *or* *None*) – The value to return if optimization failed due to e.g.
              infeasibility. If `None`, raise
              `OptimizationError` if the
              optimization fails.
            - **message** (*string*) – Error message to use if the optimization did not succeed.
            - **\*\*kwargs** –

              decimal\_precision :
              :   `bool` indicating whether to apply the
                  `decimal_precision` attribute of
                  the `MassConfiguration` to the solution values.

                  Default is `False`.

        Returns
        :   The objective value.

        Return type
        :   float

    `set_objective`(*self*, *value*, *additive=False*)[source]¶
    :   Set the model objective.

        Parameters
        :   - **value** (*optlang.interface.Objective**,* *Basic**, or* *dict*) – If the objective is linear, the value can be a new
              `optlang.interface.Objective` object or a `dict` with
              linear coefficients where each key is a metabolite and the element
              the new coefficient (`float`).
            - **additive** (*bool*) – If `True`, add the terms to the current objective, otherwise
              start with an empty objective.

    `add_metabolite_var_to_problem`(*self*, *metabolite*, *lower\_bound=None*, *upper\_bound=None*, *\*\*kwargs*)[source]¶
    :   Add a metabolite concentration variable to the problem.

        The variable in linear space is represented as:

        ```
        log(x_lb) <= log(x) <= log(x_ub)
        ```

        where

        > - `x` is the metabolite concentration variable.
        > - `x_lb` is the lower bound for the concentration.
        > - `x_ub` is the upper bound for the concentration.

        Parameters
        :   - **metabolite** (*MassMetabolite*) – The metabolite whose concentration should be added as a variable
              to the solver.
            - **lower\_bound** (*float*) – A non-negative number for the lower bound of the variable. If
              `bound_type=deviation` then value is treated as a percentage.
              Otherwise value is treated as the lower bound in linear space.
            - **upper\_bound** (*float*) – A non-negative number for the upper bound of the variable. If
              `bound_type=deviation` then value is treated as a percentage.
              Otherwise value is treated as the lower bound in linear space.
            - **\*\*kwargs** –

              bound\_type :
              :   Either `"deviation"` to indicate that bound values are
                  percentages representing deviations of the base concentration
                  value, or `"absolute"` to indicate that the bound values
                  should be not be treated as percentages but as the bound values
                  themselves (in linear space).

                  Default is `deviation`.

              concentration :
              :   A non-negative number to treat as the base concentration value
                  in setting percent deviation bounds. Ignored if
                  `bound_type=absolute`

                  Default is the current metabolite concentration accessed via
                  `MassMetabolite.initial_condition`.

              decimal\_precision :
              :   `bool` indicating whether to apply the
                  `decimal_precision` attribute of
                  the `MassConfiguration` to the bound values.

                  Default is `False`.

    `add_reaction_Keq_var_to_problem`(*self*, *reaction*, *lower\_bound=None*, *upper\_bound=None*, *\*\*kwargs*)[source]¶
    :   Add a reaction equilibrium constant variable to the problem.

        The variable in linear space is represented as:

        ```
        log(Keq_lb) <= log(Keq) <= log(Keq_ub)
        ```

        where

        > - `Keq` is the equilibrium constant variable for the reaction.
        > - `Keq_lb` is the lower bound for the equilibrium constant.
        > - `Keq_ub` is the upper bound for the equilibrium constant.

        Parameters
        :   - **reaction** (*MassReaction*) – The reaction whose equilibrium constant should be added as a
              variable to the solver.
            - **lower\_bound** (*float*) – A non-negative number for the lower bound of the variable. If
              `bound_type=deviation` then value is treated as a percentage.
              Otherwise value is treated as the lower bound in linear space.
            - **upper\_bound** (*float*) – A non-negative number for the upper bound of the variable. If
              `bound_type=deviation` then value is treated as a percentage.
              Otherwise value is treated as the lower bound in linear space.
            - **\*\*kwargs** –

              bound\_type :
              :   Either `"deviation"` to indicate that bound values are
                  percentages representing deviations of the base equilibrium
                  constant value, or `"absolute"` to indicate that the
                  bound values should be not be treated as percentages but as
                  the bound values themselves (in linear space).

                  Default is `deviation`.

              Keq :
              :   A non-negative number to treat as the base equilibrium
                  constant value in setting percent deviation bounds. Ignored if
                  `bound_type=absolute`

                  Default is the current reaction equilibrium constant accessed
                  via `MassReaction.equilibrium_constant`.

              steady\_state\_flux :
              :   The steady state flux value of the reaction. If set as 0.,
                  the creation of the equilibrium constant variable will depend
                  on whether the reaction is defined as an equilibrium reaction.

                  Default is the current reaction steady state flux accessed
                  via `MassReaction.steady_state_flux`.

              decimal\_precision :
              :   `bool` indicating whether to apply the
                  `decimal_precision` attribute of
                  the `MassConfiguration` to the bound values.

                  Default is `False`.

    `add_concentration_Keq_cons_to_problem`(*self*, *reaction*, *epsilon=None*, *\*\*kwargs*)[source]¶
    :   Add constraint using the reaction metabolite stoichiometry and Keq.

        The constraint in linear space is represented as:

        ```
        S^T * log(x) <= log(Keq) - epsilon if v > 0
        S^T * log(x) >= log(Keq) + epsilon if v < 0
        ```

        where

        > - `S^T` is the transposed stoichiometry for the reaction.
        > - `x` is the vector of concentration variables.
        > - `Keq` is the equilibrium constant variable for the reaction.
        > - `v` is the steady state flux value for the reaction.
        > - `epsilon` is a buffer for the constraint .

        Parameters
        :   - **reaction** (*MassReaction*) – The reaction whose metabolite stoichiometry and equilibrium
              constant is used to create the constraint to be added.
            - **epsilon** (*float*) –

              The buffer for the constraint.

              Default is `None` to use `ConcSolver.constraint_buffer`.
            - **\*\*kwargs** –

              bound\_type :
              :   Either `"deviation"` to indicate that bound values are
                  percentages representing deviations of the base equilibrium
                  constant value, or `"absolute"` to indicate that the
                  bound values should be not be treated as percentages but as
                  the bound values themselves (in linear space). Only used
                  if the variables necessary for the constraint do not already
                  exist.

                  Default is `deviation`.

              steady\_state\_flux :
              :   The steady state flux value of the reaction. Determines whether
                  the contraint is set up as less than or as greater than.
                  If set as 0., the creation of the reaction constraint will
                  depend on whether the reaction is defined as an equilibrium
                  reaction.

                  Default is the current reaction steady state flux accessed
                  via `MassReaction.steady_state_flux`.

              decimal\_precision :
              :   `bool` indicating whether to apply the
                  `decimal_precision` attribute of
                  the `MassConfiguration` to the bound values.

                  Default is `False`.

    `update_model_with_solution`(*self*, *concentration\_solution*, *\*\*kwargs*)[source]¶
    :   Update `ConcSolver.model` using a `ConcSolution`.

        Parameters
        :   - **concentration\_solution** (*ConcSolution*) – The `ConcSolution` containing the solution values to use
              in updating the model.
            - **\*\*kwargs** –

              concentrations :
              :   `bool` indicating whether to update the metabolite
                  concentrations of the model (the
                  `MassMetabolite.initial_condition` values).

                  Default is `True`.

              Keqs :
              :   `bool` indicating whether to update the reaction equilibrium
                  constants of the model (the
                  `MassReaction.equilibrium_constant` values).

                  Default is `True`.

              inplace :
              :   `bool` indicating whether to modify the current
                  `ConcSolver.model` or to copy the model, then modify and
                  set the model copy as the new `ConcSolver.model`. If
                  `False`, the association with the old model is removed and
                  an association with the new model is created.

                  Default is `True`.

    `_create_variable`(*self*, *mass\_obj*, *lower\_bound*, *upper\_bound*, *\*\*kwargs*)[source]¶
    :   Create an optlang variable for the solver.

        Warning

        This method is intended for internal use only.

    `_initialize_solver`(*self*, *reset\_problem=False*, *\*\*kwargs*)[source]¶
    :   Initialize the solver as a generic problem involving concentraitons.

        Warning

        This method is intended for internal use only.

    `_get_included_metabolites`(*self*, *metabolites=None*)[source]¶
    :   Return a list of MassMetabolite objects for included metabolites.

        Warning

        This method is intended for internal use only.

    `_get_included_reactions`(*self*, *reactions=None*)[source]¶
    :   Return a list of MassReaction objects for included reactions.

        Warning

        This method is intended for internal use only.

    `_check_for_missing_values`(*self*, *model*, *concentrations=True*, *equilibrium\_constants=True*, *steady\_state\_fluxes=True*)[source]¶
    :   Determine missing values that prevent setup of a problem.

        Warning

        This method is intended for internal use only.

    `__dir__`(*self*)[source]¶
    :   Override default dir() implementation to list only public items.

        Warning

        This method is intended for internal use only.

    `__repr__`(*self*)[source]¶
    :   Override default repr.

        Warning

        This method is intended for internal use only.

    `__enter__`(*self*)[source]¶
    :   Record all future changes, undoing them when \_\_exit\_\_ is called.

        Warning

        This method is intended for internal use only.

    `__exit__`(*self*, *type*, *value*, *traceback*)[source]¶
    :   Pop the top context manager and trigger the undo functions.

        Warning

        This method is intended for internal use only.

`mass.thermo.conc_solver.``concentration_constraint_matricies`(*concentration\_solver*, *array\_type='dense'*, *zero\_tol=1e-06*)[source]¶
:   Create a matrix representation of the problem.

    This is used for alternative solution approaches that do not use optlang.
    The function will construct the equality matrix, inequality matrix and
    bounds for the complete problem.

    Parameters
    :   - **concentration\_solver** (*ConcSolver*) – The `ConcSolver` containing the mathematical problem.
        - **array\_type** (*str*) – A string identifiying the desired format for the returned matrix.
          Valid matrix types include `'dense'`, `'dok'`, `'lil'`,
          `'DataFrame'`, and `'symbolic'`
          Default is the current `dense`. See the `matrix`
          module documentation for more information on the `array_type`.
        - **zero\_tol** (*float*) – The zero tolerance used to judge whether two bounds are the same.

    Returns
    :   A named tuple consisting of 6 matrices and 2 vectors:

        - `"equalities"` is a matrix `S` such that `S*vars = b`.
          It includes a row for each equality constraint and a column for
          each variable.
        - `"b"` the right side of the equality equation such that
          `S*vars = b.`
        - `"inequalities"` is a matrix `M` such that
          `lb <= M*vars <= ub`. It contains a row for each inequality and
          as many columns as variables.
        - `"bounds"` is a compound matrix `[lb ub]` containing the lower
          and upper bounds for the inequality constraints in `M`.
        - `"variable_fixed"` is a boolean vector indicating whether the
          variable at that index is fixed (`lower bound == upper_bound`) and
          is thus bounded by an equality constraint.
        - `"variable_bounds"` is a compound matrix `[lb ub]` containing
          the lower and upper bounds for all variables.

    Return type
    :   collections.namedtuple

###### `mass.util`¶

###### Submodules¶

###### `mass.util.dict_with_id`¶

DictWithID and OrderedDictWithID are dictionaries with identifers attributes.

The `dict_with_id` submodule utilizes the built-in `type` to
dynamically create the `DictWithID` and `OrderedDictWithID`
classes based on whether the parent class should be a standard `dict`
or an `OrderedDict`, therefore allowing each object to
inherit its parent class methods and generable behavior.

The `DictWithID` and `OrderedDictWithID` classes are primarily
used for in order to use dictionaries with the speciailized
`DictList` container for both performance gains
and user convenience.

###### Module Contents¶

`mass.util.dict_with_id.``DictWithID`[source]¶
:   Has ID attribute, inherits `dict` methods

    Type
    :   class

`mass.util.dict_with_id.``OrderedDictWithID`[source]¶
:   Has ID attribute, inherits `OrderedDict` methods.

    Type
    :   class

###### `mass.util.expressions`¶

Handles generation and manipulation of `sympy` expressions.

###### Module Contents¶

###### Functions¶

|  |  |
| --- | --- |
| `Keq2k`(sympy\_expr, simplify=False) | Replace `'Keq'` symbols with `'kf/kr'` in `sympy` expressions. |
| `k2Keq`(sympy\_expr, simplify=False) | Replace `'kr'` symbols with `'kf/Keq'` in `sympy` expressions. |
| `strip_time`(sympy\_expr) | Strip the time dependency in `sympy` expressions. |
| `generate_mass_action_rate_expression`(reaction, rate\_type=1) | Generate the mass action rate law for the reaction. |
| `generate_forward_mass_action_rate_expression`(reaction, rate\_type=1) | Generate the forward mass action rate expression for the reaction. |
| `generate_reverse_mass_action_rate_expression`(reaction, rate\_type=1) | Generate the reverse mass action rate expression for the reaction. |
| `generate_mass_action_ratio`(reaction) | Generate the mass action ratio for a given reaction. |
| `generate_disequilibrium_ratio`(reaction) | Generate the disequilibrium ratio for a given reaction. |
| `create_custom_rate`(reaction, custom\_rate, custom\_parameters=None) | Create a `sympy` expression for a given custom rate law. |
| `generate_ode`(metabolite) | Generate the ODE for a given metabolite as a `sympy` expression. |

`mass.util.expressions.``Keq2k`(*sympy\_expr*, *simplify=False*)[source]¶
:   Replace `'Keq'` symbols with `'kf/kr'` in `sympy` expressions.

    Parameters
    :   - **sympy\_expr** (*Basic**,* *dict**, or* *list*) – A `sympy` expression, a list of `sympy` expressions, or a
          dictionary with `sympy` expressions as the values.
        - **simplify** (*bool*) – If `True` then try to simplify the expression after making the
          substitution. Otherwise leave the expression as is.

    Returns
    :   The `sympy` expression(s) with the substitution made, returned as
        the same type as the original input.

    Return type
    :   Basic, dict, or list

`mass.util.expressions.``k2Keq`(*sympy\_expr*, *simplify=False*)[source]¶
:   Replace `'kr'` symbols with `'kf/Keq'` in `sympy` expressions.

    Parameters
    :   - **sympy\_expr** (*Basic**,* *dict**, or* *list*) – A `sympy` expression, a list of `sympy` expressions, or a
          dictionary with `sympy` expressions as the values.
        - **simplify** (*bool*) – If `True` then try to simplify the expression after making the
          substitution. Otherwise leave the expression as is.

    Returns
    :   The `sympy` expression(s) with the substitution made, returned as
        the same type as the original input.

    Return type
    :   Basic, dict, or list

`mass.util.expressions.``strip_time`(*sympy\_expr*)[source]¶
:   Strip the time dependency in `sympy` expressions.

    Parameters
    :   **sympy\_expr** (*Basic**,* *dict**, or* *list*) – A `sympy` expression, a list of `sympy` expressions, or a
        dictionary with `sympy` expressions as the values.

    Returns
    :   The `sympy` expression(s) with the time dependency removed,
        returned as the same type as the original input.

    Return type
    :   Basic, dict, or list

`mass.util.expressions.``generate_mass_action_rate_expression`(*reaction*, *rate\_type=1*)[source]¶
:   Generate the mass action rate law for the reaction.

    Parameters
    :   - **reaction** (*MassReaction*) – The reaction to generate the rate expression for.
        - **rate\_type** (*int*) –

          The type of rate law to return. Must be 1, 2, or 3.

          > - Type 1 will utilize the
          >   `forward_rate_constant` and the
          >   `equilibrium_constant`.
          > - Type 2 will utilize the
          >   `forward_rate_constant` and the
          >   `reverse_rate_constant`.
          > - Type 3 will utilize the
          >   `equilibrium_constant` and the
          >   `reverse_rate_constant`.

          Default is `1`.

    Returns
    :   The rate law as a `sympy` expression. If the reaction has no
        metabolites associated, `None` will be returned.

    Return type
    :   Basic or None

`mass.util.expressions.``generate_forward_mass_action_rate_expression`(*reaction*, *rate\_type=1*)[source]¶
:   Generate the forward mass action rate expression for the reaction.

    Parameters
    :   - **reaction** (*MassReaction*) – The reaction to generate the rate expression for.
        - **rate\_type** (*int*) –

          The type of rate law to return. Must be 1, 2, or 3.

          > - Type 1 and 2 will utilize the
          >   `forward_rate_constant`.
          > - Type 3 will utilize the
          >   `equilibrium_constant` and the
          >   `reverse_rate_constant`.

          Default is 1.

    Returns
    :   The forward rate as a `sympy` expression. If the reaction
        has no metabolites associated, `None` will be returned.

    Return type
    :   Basic or None

`mass.util.expressions.``generate_reverse_mass_action_rate_expression`(*reaction*, *rate\_type=1*)[source]¶
:   Generate the reverse mass action rate expression for the reaction.

    Parameters
    :   - **reaction** (*MassReaction*) – The reaction to generate the rate expression for.
        - **rate\_type** (*int*) –

          The type of rate law to return. Must be 1, 2, or 3.

          > - Type 1 will utilize the
          >   `forward_rate_constant` and the
          >   `equilibrium_constant`.
          > - Type 2 and 3 will utilize the
          >   `reverse_rate_constant`.

          Default is 1.

    Returns
    :   The reverse rate as a `sympy` expression. If the reaction
        has no metabolites associated, `None` will be returned.

    Return type
    :   Basic or None

`mass.util.expressions.``generate_mass_action_ratio`(*reaction*)[source]¶
:   Generate the mass action ratio for a given reaction.

    Parameters
    :   **reaction** (*MassReaction*) – The reaction to generate the mass action ratio for.

    Returns
    :   The mass action ratio as a `sympy` expression.

    Return type
    :   Basic

`mass.util.expressions.``generate_disequilibrium_ratio`(*reaction*)[source]¶
:   Generate the disequilibrium ratio for a given reaction.

    Parameters
    :   **reaction** (*MassReaction*) – The reaction to generate the disequilibrium ratio for.

    Returns
    :   The disequilibrium ratio as a `sympy` expression.

    Return type
    :   Basic

`mass.util.expressions.``create_custom_rate`(*reaction*, *custom\_rate*, *custom\_parameters=None*)[source]¶
:   Create a `sympy` expression for a given custom rate law.

    Notes

    - Metabolites must already exist in the `MassModel` or
      `MassReaction`.
    - Default parameters of a `MassReaction` are automatically
      taken into account and do not need to be defined as additional
      custom parameters.

    Parameters
    :   - **reaction** (*MassReaction*) – The reaction associated with the custom rate.
        - **custom\_rate** (*str*) – The custom rate law as a str. The string representation of the
          custom rate law will be used to create the expression through the
          `sympify()` function.
        - **custom\_parameters** (*list of str*) – The custom parameter(s) of the custom rate law as a list of strings.
          The string representation of the custom parameters will be used for
          creation and recognition of the custom parameter symbols in the
          `sympy` expression. If `None` then parameters are assumed to be
          one or more of the reaction rate or equilibrium constants.

    Returns
    :   A `sympy` expression of the custom rate. If no metabolites are
        assoicated with the reaction, `None` will be returned.

    Return type
    :   Basic or None

    See also

    `MassReaction.all_parameter_ids`
    :   List of default reaction parameters automatically accounted for.

`mass.util.expressions.``generate_ode`(*metabolite*)[source]¶
:   Generate the ODE for a given metabolite as a `sympy` expression.

    Parameters
    :   **metabolite** (*MassMetabolite*) – The metabolite to generate the ODE for.

    Returns
    :   **ode** – A `sympy` expression of the metabolite ODE. If the metabolite
        is not associated with any reactions, then `None` will be returned.

    Return type
    :   Basic or None

###### `mass.util.matrix`¶

Containins basic matrix operations that can be applied to a `mass` model.

To assist with various matrix operations using different packages, the
following values can be provided to the `array_type` argument to set the
return type of the output. Valid matrix types include:

- `'dense'` for a `numpy.ndarray`
- `'dok'` for a `scipy.sparse.dok_matrix`
- `'lil'` for a `scipy.sparse.lil_matrix`
- `'DataFrame'` for a `pandas.DataFrame`
- `'symbolic'` for a
  `sympy.MutableDenseMatrix`

For all matrix types, species (excluding genes) are the row indicies and
reactions are the column indicies.

There are also several methods that are nearly identical to `scipy.linalg`
methods, with the main exception being that matrix conversions are performed
beforehand to ensure that valid input is passed to the `scipy` method.
These methods include:

- `svd()`
- `eig()`

###### Module Contents¶

###### Functions¶

|  |  |
| --- | --- |
| `gradient`(model, use\_parameter\_values=True, use\_concentration\_values=True, array\_type=’dense’) | Create the gradient matrix for a given model. |
| `kappa`(model, use\_parameter\_values=True, use\_concentration\_values=True, array\_type=’dense’) | Create the kappa matrix for a given model. |
| `gamma`(model, use\_parameter\_values=True, use\_concentration\_values=True, array\_type=’dense’) | Create the gamma matrix for a given model. |
| `jacobian`(model, jacobian\_type=’species’, use\_parameter\_values=True, use\_concentration\_values=True, array\_type=’dense’) | Get the jacobian matrix for a given model. |
| `nullspace`(matrix, atol=1e-13, rtol=0, decimal\_precision=False) | Compute an approximate basis for the nullspace of a matrix. |
| `left_nullspace`(matrix, atol=1e-13, rtol=0, decimal\_precision=False) | Compute an approximate basis for the left nullspace of a matrix. |
| `columnspace`(matrix, atol=1e-13, rtol=0, decimal\_precision=False) | Compute an approximate basis for the columnspace of a matrix. |
| `rowspace`(matrix, atol=1e-13, rtol=0, decimal\_precision=False) | Compute an approximate basis for the rowspace of a matrix. |
| `matrix_rank`(matrix, atol=1e-13, rtol=0) | Estimate the rank (i.e. the dimension of the nullspace) of a matrix. |
| `svd`(matrix, \*\*kwargs) | Get the singular value decomposition of a matrix. |
| `eig`(matrix, left=False, right=False, \*\*kwargs) | Get the eigenvalues of a matrix. |
| `convert_matrix`(matrix, array\_type, dtype, row\_ids=None, col\_ids=None) | Convert a matrix to a different type. |

`mass.util.matrix.``gradient`(*model*, *use\_parameter\_values=True*, *use\_concentration\_values=True*, *array\_type='dense'*)[source]¶
:   Create the gradient matrix for a given model.

    Parameters
    :   - **model** (*MassModel*) – The `MassModel` to construct the matrix for.
        - **use\_parameter\_values** (*bool*) – Whether to substitute the numerical values for parameters into the
          matrix. If `True` then numerical values of the kinetic parameters
          are substituted into the matrix. Otherwise parameters in the matrix
          are left as symbols. Default is `True`.
        - **use\_concentration\_values** (*bool*) – Whether to substitute the numerical values for concentrations into the
          matrix. If `True` then numerical values of the initial conditions
          are substituted into the matrix. Otherwise species concentrations in
          the matrix are left as symbols. Default is `True`.
        - **array\_type** (*str*) – A string identifiying the desired format for the returned matrix.
          Default is `'dense'`. See the `matrix` module documentation
          for more information on the `array_type`

    Returns
    :   The gradient matrix for the model.

    Return type
    :   matrix of type `array_type`

`mass.util.matrix.``kappa`(*model*, *use\_parameter\_values=True*, *use\_concentration\_values=True*, *array\_type='dense'*)[source]¶
:   Create the kappa matrix for a given model.

    Notes

    The kappa matrix is the diagnolization of the norms for the rows in the
    gradient matrix.

    Parameters
    :   - **model** (*MassModel*) – The `MassModel` to construct the matrix for.
        - **use\_parameter\_values** (*bool*) – Whether to substitute the numerical values for parameters into the
          matrix. If `True` then numerical values of the kinetic parameters
          are substituted into the matrix. Otherwise parameters in the matrix
          are left as symbols. Default is `True`.
        - **use\_concentration\_values** (*bool*) – Whether to substitute the numerical values for concentrations into the
          matrix. If `True` then numerical values of the initial conditions
          are substituted into the matrix. Otherwise species concentrations in
          the matrix are left as symbols. Default is `True`.
        - **array\_type** (*str*) – A string identifiying the desired format for the returned matrix.
          Default is `'dense'`. See the `matrix` module documentation
          for more information on the `array_type`.

    Returns
    :   The kappa matrix for the model.

    Return type
    :   matrix of type `array_type`

`mass.util.matrix.``gamma`(*model*, *use\_parameter\_values=True*, *use\_concentration\_values=True*, *array\_type='dense'*)[source]¶
:   Create the gamma matrix for a given model.

    Notes

    The gamma matrix is composed of the 1-norms of the gradient matrix.

    Parameters
    :   - **model** (*MassModel*) – The `MassModel` to construct the matrix for.
        - **use\_parameter\_values** (*bool*) – Whether to substitute the numerical values for parameters into the
          matrix. If `True` then numerical values of the kinetic parameters
          are substituted into the matrix. Otherwise parameters in the matrix
          are left as symbols. Default is `True`.
        - **use\_concentration\_values** (*bool*) – Whether to substitute the numerical values for concentrations into
          the matrix. If `True` then numerical values of the initial
          conditions are substituted into the matrix. Otherwise species
          concentrations in the matrix are left as symbols. Default is `True`.
        - **array\_type** (*str*) – A string identifiying the desired format for the returned matrix.
          Default is `'dense'`. See the `matrix` module documentation
          for more information on the `array_type`.

    Returns
    :   The gamma matrix for the model.

    Return type
    :   matrix of type `array_type`

`mass.util.matrix.``jacobian`(*model*, *jacobian\_type='species'*, *use\_parameter\_values=True*, *use\_concentration\_values=True*, *array\_type='dense'*)[source]¶
:   Get the jacobian matrix for a given model.

    Parameters
    :   - **model** (*MassModel*) – The `MassModel` to construct the matrix for.
        - **jacobian\_type** (*str*) – Either the string `'species'` to obtain the jacobian matrix with
          respect to species, or the string `'reactions'` to obtain the
          jacobian matrix with respect to the reactions.
          Default is `'reactions'`.
        - **use\_parameter\_values** (*bool*) – Whether to substitute the numerical values for parameters into the
          matrix. If `True` then numerical values of the kinetic parameters
          are substituted into the matrix. Otherwise parameters in the matrix
          are left as symbols. Default is `True`.
        - **use\_concentration\_values** (*bool*) – Whether to substitute the numerical values for concentrations into the
          matrix. If `True` then numerical values of the initial conditions
          are substituted into the matrix. Otherwise species concentrations in
          the matrix are left as symbols. Default is `True`.
        - **array\_type** (*str*) – A string identifiying the desired format for the returned matrix.
          Default is `'dense'`. See the `matrix` module documentation
          for more information on the `array_type`.

    Returns
    :   The jacobian matrix for the model.

    Return type
    :   matrix of type `array_type`

`mass.util.matrix.``nullspace`(*matrix*, *atol=1e-13*, *rtol=0*, *decimal\_precision=False*)[source]¶
:   Compute an approximate basis for the nullspace of a matrix.

    The algorithm used by this function is based on singular value
    decomposition.

    Notes

    - If both `atol` and `rtol` are positive, the combined tolerance
      is the maximum of the two; that is:

      ```
      tol = max(atol, rtol * smax)
      ```

      Singular values smaller than `tol` are considered to be zero.
    - Similar to the `cobra.util.array.nullspace` function, but includes
      utlization of the `decimal_precision` in
      the `MassConfiguration` and sets values below the tolerance to 0.
    - Taken from the numpy cookbook and extended.

    Parameters
    :   - **matrix** (*array-like*) – The matrix to decompose. The matrix should be at most 2-D. A 1-D
          array with length `k` will be treated as a 2-D with shape `(1, k)`.
        - **atol** (*float*) – The absolute tolerance for a zero singular value. Singular values
          smaller than `atol` are considered to be zero.
        - **rtol** (*float*) – The relative tolerance. Singular values less than `rtol * smax` are
          considered to be zero, where `smax` is the largest singular value.
        - **decimal\_precision** (*bool*) – Whether to apply the `decimal_precision`
          set in the `MassConfiguration` to the nullspace values
          before comparing to the tolerance. Default is `False`.

    Returns
    :   **ns** – If `matrix` is an array with shape `(m, k)`, then `ns` will be
        an array with shape `(k, n)`, where `n` is the estimated dimension
        of the nullspace of `matrix`. The columns of `ns` are a basis for
        the nullspace; each element in the dot product of the matrix
        and the nullspace will be approximately 0.

    Return type
    :   numpy.ndarray

`mass.util.matrix.``left_nullspace`(*matrix*, *atol=1e-13*, *rtol=0*, *decimal\_precision=False*)[source]¶
:   Compute an approximate basis for the left nullspace of a matrix.

    The algorithm used by this function is based on singular value
    decomposition.

    Notes

    If both `atol` and `rtol` are positive, the combined tolerance is the
    maximum of the two; that is:

    ```
    tol = max(atol, rtol * smax)
    ```

    Singular values smaller than `tol` are considered to be zero.

    Parameters
    :   - **matrix** (*array-like*) – The matrix to decompose. The matrix should be at most 2-D. A 1-D
          array with length `k` will be treated as a 2-D with shape `(1, k)`.
        - **atol** (*float*) – The absolute tolerance for a zero singular value. Singular values
          smaller than `atol` are considered to be zero.
        - **rtol** (*float*) – The relative tolerance. Singular values less than `rtol * smax` are
          considered to be zero, where `smax` is the largest singular value.
        - **decimal\_precision** (*bool*) – Whether to apply the `decimal_precision`
          set in the `MassConfiguration` to the left nullspace values
          before comparing to the tolerance. Default is `False`.

    Returns
    :   **lns** – If `matrix` is an array with shape `(m, k)`, then `lns` will be
        an array with shape `(n, m)`, where `n` is the estimated dimension
        of the left nullspace of `matrix`. The rows of `lns` are a basis
        for the left nullspace; each element in the dot product of the matrix
        and the left nullspace will be approximately 0.

    Return type
    :   numpy.ndarray

    See also

    `nullspace()`
    :   Base function.

`mass.util.matrix.``columnspace`(*matrix*, *atol=1e-13*, *rtol=0*, *decimal\_precision=False*)[source]¶
:   Compute an approximate basis for the columnspace of a matrix.

    This function utilizes the `scipy.linalg.qr()` function to obtain an
    orthogonal basis for the columnspace of the matrix.

    Notes

    If both `atol` and `rtol` are positive, the combined tolerance is the
    maximum of the two; that is:

    ```
    tol = max(atol, rtol * smax)
    ```

    Singular values smaller than `tol` are considered to be zero.

    Parameters
    :   - **matrix** (*array-like*) – The matrix to decompose. The matrix should be at most 2-D. A 1-D
          array with length `k` will be treated as a 2-D with shape `(1, k)`.
        - **atol** (*float*) – The absolute tolerance for a zero singular value. Singular values
          smaller than `atol` are considered to be zero.
        - **rtol** (*float*) – The relative tolerance. Singular values less than `rtol * smax` are
          considered to be zero, where `smax` is the largest singular value.
        - **decimal\_precision** (*bool*) – Whether to apply the `decimal_precision`
          set in the `MassConfiguration` to the columnspace values
          before comparing to the tolerance. Default is `False`.

    Returns
    :   **cs** – If `matrix` is an array with shape `(m, k)`, then `cs` will be
        an array with shape `(m, n)`, where `n` is the estimated dimension
        of the columnspace of `matrix`. The columns of `cs` are a basis
        for the columnspace.

    Return type
    :   numpy.ndarray

`mass.util.matrix.``rowspace`(*matrix*, *atol=1e-13*, *rtol=0*, *decimal\_precision=False*)[source]¶
:   Compute an approximate basis for the rowspace of a matrix.

    This function utilizes the `scipy.linalg.qr()` function to obtain an
    orthogonal basis for the rowspace of the matrix.

    Notes

    If both `atol` and `rtol` are positive, the combined tolerance is the
    maximum of the two; that is:

    ```
    tol = max(atol, rtol * smax)
    ```

    Singular values smaller than `tol` are considered to be zero.

    Parameters
    :   - **matrix** (*array-like*) – The matrix to decompose. The matrix should be at most 2-D. A 1-D
          array with length `k` will be treated as a 2-D with shape `(1, k)`.
        - **atol** (*float*) – The absolute tolerance for a zero singular value. Singular values
          smaller than `atol` are considered to be zero.
        - **rtol** (*float*) – The relative tolerance. Singular values less than `rtol * smax` are
          considered to be zero, where `smax` is the largest singular value.
        - **decimal\_precision** (*bool*) – Whether to apply the `decimal_precision`
          set in the `MassConfiguration` to the rowspace values
          before comparing to the tolerance. Default is `False`.

    Returns
    :   **rs** – If `matrix` is an array with shape `(m, k)`, then `rs` will be
        an array with shape `(n, k)`, where `n` is the estimated dimension
        of the rowspace of `matrix`. The columns of `rs` are a basis for
        the rowspace.

    Return type
    :   numpy.ndarray

    See also

    `columnspace()`
    :   Base function.

`mass.util.matrix.``matrix_rank`(*matrix*, *atol=1e-13*, *rtol=0*)[source]¶
:   Estimate the rank (i.e. the dimension of the nullspace) of a matrix.

    The algorithm used by this function is based on singular value
    decomposition. Taken from the `scipy` cookbook.

    Notes

    If both `atol` and `rtol` are positive, the combined tolerance is the
    maximum of the two; that is:

    ```
    tol = max(atol, rtol * smax)
    ```

    Singular values smaller than `tol` are considered to be zero.

    Parameters
    :   - **matrix** (*array-like*) – The matrix to obtain the rank for. The matrix should be at most
          2-D. A 1-D array with length `k` will be treated as a 2-D with
          shape `(1, k)`.
        - **atol** (*float*) – The absolute tolerance for a zero singular value. Singular values
          smaller than `atol` are considered to be zero.
        - **rtol** (*float*) – The relative tolerance. Singular values less than `rtol * smax` are
          considered to be zero, where `smax` is the largest singular value.

    Returns
    :   **rank** – The estimated rank of the matrix.

    Return type
    :   int

    See also

    `numpy.linalg.matrix_rank()`
    :   `mass.util.matrix.matrix_rank()` is nearly identical to this function, but it does not provide the option of the absolute tolerance.

`mass.util.matrix.``svd`(*matrix*, *\*\*kwargs*)[source]¶
:   Get the singular value decomposition of a matrix.

    `kwargs` are passed on to `scipy.linalg.svd()`.

    Parameters
    :   **matrix** (*array-like*) – The matrix to decompose. The matrix should be at most 2-D. A 1-D
        array with length `k` will be treated as a 2-D with shape `(1, k)`.

    Returns
    :   - **U** (*ndarray*) – Unitary matrix having left singular vectors as columns.
          Of shape `(M, M)` or `(M, K)`, depending on full\_matrices.
        - **s** (*ndarray*) – The singular values, sorted in non-increasing order.
          Of shape `(K, )`, with `K = min(M, N)`.
        - **Vh** (*ndarray*) – Unitary matrix having right singular vectors as rows.
          Of shape `(N, N)` or `(K, N)` depending on full\_matrices.
        - For `compute_uv=False`, only `s` is returned.

    See also

    `scipy.linalg.svd()`
    :   Base function.

`mass.util.matrix.``eig`(*matrix*, *left=False*, *right=False*, *\*\*kwargs*)[source]¶
:   Get the eigenvalues of a matrix.

    `kwargs` are passed on to `scipy.linalg.eig()`

    Parameters
    :   - **matrix** (*array-like*) – The matrix to decompose. The matrix should be at most 2-D. A 1-D
          array with length `k` will be treated as a 2-D with shape `(1, k)`.
        - **left** (*bool*) – Whether to calculate and return left eigenvectors.
          Default is `False`.
        - **right** (*bool*) – Whether to calculate and return right eigenvectors.
          Default is `True`.

    Returns
    :   - **w** (*(M, ) double or complex ndarray*) – The eigenvalues, each repeated according to its multiplicity.
        - **vl** (*(M, M) double or complex ndarray*) – The normalized left eigenvector corresponding to the eigenvalue
          `w[i]` is the column v[:,i]. Only returned if `left=True`.
        - **vr** (*(M, M) double or complex ndarray*) – The normalized right eigenvector corresponding to the eigenvalue
          `w[i]` is the column `vr[:,i]`. Only returned if `right=True`.

    See also

    `scipy.linalg.eig()`
    :   Base function.

`mass.util.matrix.``convert_matrix`(*matrix*, *array\_type*, *dtype*, *row\_ids=None*, *col\_ids=None*)[source]¶
:   Convert a matrix to a different type.

    Parameters
    :   - **matrix** (*array-like*) – The matrix to convert.
        - **array\_type** (*str*) – A string identifiying the desired format for the returned matrix.
          Valid matrix types include `'dense'`, `'dok'`, `'lil'`,
          `'DataFrame'`, and `'symbolic'` See the `matrix` module
          documentation for more information on the `array_type`.
        - **dtype** (*data-type*) – The desired array data-type for the matrix.
        - **row\_ids** (*array-like*) – The idenfifiers for each row. Only used if type is `'DataFrame'`.
        - **col\_ids** (*array-like*) – The idenfifiers for each column. Only used if type is `'DataFrame'`.

    Warning

    This method is NOT the safest way to convert a matrix to another type.
    To safely convert a matrix into another type, use the `'array_type'`
    argument in the method that returns the desired matrix.

###### `mass.util.qcqa`¶

Module containing functions to assess the quality of a model.

###### Module Contents¶

###### Functions¶

|  |  |
| --- | --- |
| `qcqa_model`(model, \*\*kwargs) | Check the model quality and print a summary of the results. |
| `get_missing_reaction_parameters`(model, reaction\_list=None, simulation\_only=True) | Identify the missing parameters for reactions in a model. |
| `get_missing_custom_parameters`(model, reaction\_list=None, simulation\_only=True) | Identify the missing custom parameters in a model. |
| `get_missing_steady_state_fluxes`(model, reaction\_list=None) | Identify the missing steady state flux values for reactions in a model. |
| `get_missing_initial_conditions`(model, metabolite\_list=None, simulation\_only=True) | Identify the missing initial conditions for metabolites in a model. |
| `get_missing_boundary_conditions`(model, metabolite\_list=None, simulation\_only=True) | Identify the missing boundary conditions for metabolites in a model. |
| `check_superfluous_consistency`(model, reaction\_list=None) | Check parameters of model reactions to ensure numerical consistentency. |
| `check_elemental_consistency`(model, reaction\_list=None) | Check the reactions in the model to ensure elemental consistentency. |
| `check_reaction_parameters`(model, reaction\_list=None, simulation\_only=True) | Check the model reactions for missing and superfluous parameters. |
| `is_simulatable`(model) | Determine whether a model can be simulated. |

`mass.util.qcqa.``qcqa_model`(*model*, *\*\*kwargs*)[source]¶
:   Check the model quality and print a summary of the results.

    Notes

    Checking the model quality involves running a series of quality control and
    assessment tests to determine consistency (e.g. elemental)
    in the model, missing values, and whether the model can be simulated.

    Parameters
    :   - **model** (*MassModel*) – The model to inspect.
        - **\*\*kwargs** –

          parameters :
          :   `bool` indicating whether to check for undefined parameters in
              the model.

              Default is `False.`

          concentrations :
          :   `bool` indicating whether to check for undefined initial and
              boundary conditions in the model.

              Default is `False.`

          fluxes :
          :   `bool` indicating whether to check for undefined steady state
              fluxes in the model.

              > Default is `False.`

          superfluous :
          :   `bool` indicating whether to check for superfluous parameters
              in the model and ensure existing parameters are consistent with
              one another if superfluous parameters are present.

              > Default is `False.`

          elemental :
          :   `bool` indicating whether to check for elemental consistency in
              the model. Boundary reactions are ignored.

              Default is `False.`

          simulation\_only :
          :   Only check for undefined values necessary for simulating the model.

              Default is `True`.

`mass.util.qcqa.``get_missing_reaction_parameters`(*model*, *reaction\_list=None*, *simulation\_only=True*)[source]¶
:   Identify the missing parameters for reactions in a model.

    Notes

    Will include the default reaction parameters in custom rate laws. To get
    missing custom parameters for reactions with custom rate expressions,
    use `get_missing_custom_parameters()` instead.

    Parameters
    :   - **model** (*MassModel*) – The model to inspect.
        - **reaction\_list** (*iterable*) – An iterable of `MassReaction`s in the model to be checked.
          If `None` then all reactions in the model will be utilized.
        - **simulation\_only** – Only check for undefined values necessary for simulating the model.

    Returns
    :   **missing** – A `dict` with `MassReaction`s as keys and a string
        identifying the missing parameters as values. Will return as an
        empty `dict` if there are no missing values.

    Return type
    :   dict

    See also

    `MassReaction.all_parameter_ids`
    :   List of default reaction parameters.

`mass.util.qcqa.``get_missing_custom_parameters`(*model*, *reaction\_list=None*, *simulation\_only=True*)[source]¶
:   Identify the missing custom parameters in a model.

    Notes

    Will not include default reaction parameters. To get missing standard
    reaction parameters for reactions with custom rate laws, use
    `get_missing_reaction_parameters()` instead.

    Parameters
    :   - **model** (*MassModel*) – The model to inspect.
        - **reaction\_list** (*iterable*) – An iterable of `MassReaction`s in the model to be checked.
          If `None` then all reactions in the model will be utilized.
        - **simulation\_only** – Only check for undefined values necessary for simulating the model.

    Returns
    :   **missing** – A `dict` with `MassReaction`s as keys and a string
        identifying the missing custom parameters as values. Will return as an
        empty `dict` if there are no missing values.

    Return type
    :   dict

    See also

    `MassReaction.all_parameter_ids`
    :   List of default reaction parameters.

`mass.util.qcqa.``get_missing_steady_state_fluxes`(*model*, *reaction\_list=None*)[source]¶
:   Identify the missing steady state flux values for reactions in a model.

    Parameters
    :   - **model** (*MassModel*) – The model to inspect.
        - **reaction\_list** (*iterable*) – An iterable of `MassReaction`s in the model to be checked.
          If `None` then all reactions in the model will be utilized.

    Returns
    :   **missing** – List of `MassReaction`s with missing steady state fluxes.
        Will return as an empty `list` if there are no missing values.

    Return type
    :   list

`mass.util.qcqa.``get_missing_initial_conditions`(*model*, *metabolite\_list=None*, *simulation\_only=True*)[source]¶
:   Identify the missing initial conditions for metabolites in a model.

    Notes

    Does not include boundary conditions.

    Parameters
    :   - **model** (*MassModel*) – The model to inspect.
        - **metabolite\_list** (*iterable*) – An iterable of `MassMetabolite`s in the model to be checked.
          If `None` then all metabolites in the model will be utilized.
        - **simulation\_only** – Only check for undefined values necessary for simulating the model.

    Returns
    :   **missing** – List of `MassMetabolite`s with missing initial conditions.
        Will return as an empty `list` if there are no missing values.

    Return type
    :   list

`mass.util.qcqa.``get_missing_boundary_conditions`(*model*, *metabolite\_list=None*, *simulation\_only=True*)[source]¶
:   Identify the missing boundary conditions for metabolites in a model.

    Parameters
    :   - **model** (*MassModel*) – The model to inspect.
        - **metabolite\_list** (*iterable*) – An iterable of ‘boundary metabolites’ or `MassMetabolite`s
          in the model to be checked. If `None` then all ‘boundary metabolites’
          in the model will be utilized.
        - **simulation\_only** – Only check for undefined values necessary for simulating the model.

    Returns
    :   **missing** – List of metabolites with missing boundary conditions.
        Will return as an empty `list` if there are no missing values.

    Return type
    :   list

    See also

    `MassModel.boundary_metabolites`
    :   List of boundary metabolites found in the model.

`mass.util.qcqa.``check_superfluous_consistency`(*model*, *reaction\_list=None*)[source]¶
:   Check parameters of model reactions to ensure numerical consistentency.

    Parameter numerical consistency includes checking reaction rate and
    equilibrium constants to ensure they are mathematically consistent with
    one another. If there are no superfluous parameters, the existing
    parameters are considered consistent.

    Notes

    The MassConfiguration.decimal\_precision is used to round the value of
    `abs(rxn.kr - rxn.kf/rxn.Keq)` before comparison.

    Parameters
    :   - **model** (*MassModel*) – The model to inspect.
        - **reaction\_list** (*iterable*) – An iterable of `MassReaction`s in the model to be checked.
          If `None` then all reactions in the model will be utilized.

    Returns
    :   **inconsistent** – A `dict` with `MassReaction`s as keys and a string
        identifying the incosistencies as values. Will return as an
        empty `dict` if there are no inconsistencies.

    Return type
    :   dict

`mass.util.qcqa.``check_elemental_consistency`(*model*, *reaction\_list=None*)[source]¶
:   Check the reactions in the model to ensure elemental consistentency.

    Elemental consistency includes checking reactions to ensure they are mass
    and charged balanced. Boundary reactions are ignored because they are
    typically unbalanced.

    Parameters
    :   - **model** (*MassModel*) – The model to inspect.
        - **reaction\_list** (*iterable*) – An iterable of `MassReaction`s in the model to be checked.
          If `None` then all reactions in the model will be utilized.

    Returns
    :   **inconsistent** – A `dict` with `MassReaction`s as keys and a string
        identifying the incosistencies as values. Will return as an
        empty `dict` if there are no inconsistencies.

    Return type
    :   dict

`mass.util.qcqa.``check_reaction_parameters`(*model*, *reaction\_list=None*, *simulation\_only=True*)[source]¶
:   Check the model reactions for missing and superfluous parameters.

    Parameters
    :   - **model** (*MassModel*) – The model to inspect.
        - **reaction\_list** (*iterable*) – An iterable of `MassReaction`s in the model to be checked.
          If `None` then all reactions in the model will be utilized.
        - **simulation\_only** – Only check for undefined values necessary for simulating the model.

    Returns
    :   - *tuple (missing, superfluous)*
        - **missing** (*dict*) – A `dict` with `MassReaction`s as keys and a string
          identifying the missing parameters as values. Will return as an
          empty `dict` if there are no missing values.
        - **superfluous** (*dict*) – A `dict` with `MassReaction`s as keys and superfluous
          parameters as values. Will return as an empty `dict` if there are no
          superfluous values.

`mass.util.qcqa.``is_simulatable`(*model*)[source]¶
:   Determine whether a model can be simulated.

    Parameters
    :   **model** (*MassModel*) – The model to inspect.

    Returns
    :   - *tuple (simulate\_check, consistency\_check)*
        - **simulate\_check** (*bool*) – `True` if the model can be simulated, `False` otherwise.
        - **consistency\_check** (*bool*) – `True` if the model has no issues with numerical consistency,
          `False` otherwise.

###### `mass.util.util`¶

Contains utility functions to assist in various `mass` functions.

###### Module Contents¶

###### Classes¶

|  |  |
| --- | --- |
| `ColorFormatter` | Colored Formatter for logging output. |

###### Functions¶

|  |  |
| --- | --- |
| `show_versions`() | Print dependency information. |
| `ensure_iterable`(item) | Ensure the given item is an returned as an iterable. |
| `ensure_non_negative_value`(value, exclude\_zero=False) | Ensure provided value is a non-negative value, or `None`. |

`mass.util.util.``LOG_COLORS`[source]¶
:   Contains logger levels and corresponding color codes.

    Type
    :   dict

`mass.util.util.``show_versions`()[source]¶
:   Print dependency information.

`mass.util.util.``ensure_iterable`(*item*)[source]¶
:   Ensure the given item is an returned as an iterable.

    Parameters
    :   **item** (*object*) – The item to ensure is returned as an iterable.

`mass.util.util.``ensure_non_negative_value`(*value*, *exclude\_zero=False*)[source]¶
:   Ensure provided value is a non-negative value, or `None`.

    Parameters
    :   **value** (*float*) – The value to ensure is non-negative

    Raises
    :   **ValueError** – Occurs if the value is negative.

*class* `mass.util.util.``ColorFormatter`(*fmt=None*, *datefmt=None*, *style='%'*)[source]¶
:   Bases: `logging.Formatter`

    Colored Formatter for logging output.

    Based on
    http://uran198.github.io/en/python/2016/07/12/colorful-python-logging.html

    `format`(*self*, *record*, *\*args*, *\*\*kwargs*)[source]¶
    :   Set logger format.

###### `mass.visualization`¶

Contains function for visualizing simulation results.

This module contains the various functions for visualization of solutions
returned in `MassSolution`s after simulation of models.

In general, it is recommended to pass an `matplotlib.axes.Axes` instance
into the visualization function in order to guaruntee more control over the
generated plot and ensure that the plot be placed onto a figure as expected.
If an `Axes` is not passed into the function, then the
currrent axes instance will be used, accessed via:

```
matplotlib.pyplot.gca()
```

All functions will return the axes instance utilzed in generating the plot.

The `legend` input format for the plotting function must be one of the
following:

> 1. An iterable of legend labels as strings
>    (e.g. `legend=["A", "B", ...]`).
> 2. A `str` representing the location of the legend, or an
>    `int` between 0 and 14 (inclusive) corresponding to the
>    legend location (e.g. `legend="best"` or `legend=1`).
> 3. An iterable of the format `(labels, loc)` to set both
>    the legend labels and location, where the `labels` and `loc`
>    in the iterable follows the formats specified above in both
>    **1** and **2**, respectively.
>    (e.g. `legend=(["A", "B", ...], "best")`)

Valid legend location strings and the integer corresponding to the legend
location codes are the following:

> | Location String (str) | Location Code (int) |
> | --- | --- |
> | ‘best’ | 0 |
> | ‘upper right’ | 1 |
> | ‘upper left’ | 2 |
> | ‘lower left’ | 3 |
> | ‘lower right’ | 4 |
> | ‘right’ | 5 |
> | ‘center left’ | 6 |
> | ‘center right’ | 7 |
> | ‘lower center’ | 8 |
> | ‘upper center’ | 9 |
> | ‘center’ | 10 |
> | ‘upper right outside’ | 11 |
> | ‘upper left outside’ | 12 |
> | ‘lower left outside’ | 13 |
> | ‘lower right outside’ | 14 |
> | ‘right outside’ | 15 |
> | ‘left outside’ | 16 |
> | ‘lower outside’ | 17 |
> | ‘upper outside’ | 18 |

Some other important things to note about the legend include the following:

> - Note that the last four “outside” locations are NOT
>   `matplotlib.legend` location values, but rather they utilize the
>   legend kwarg `bbox_to_anchor` to place the legend outside of the plot.
> - If only legend labels are provided (i.e. format specified above in
>   **1**), the default legend location specified in the
>   `matplotlib.rcsetup` will be used.
> - If only the legend location is provided (i.e. format specified above
>   in **2**), the corresponding keys in `mass_solution` will be used as
>   default legend labels.
> - If invalid input is provided (e.g. too many legend labels provided,
>   invalid legend location), a warning will be issued and the default
>   values will be used.
> - See the `matplotlib.legend` documentation for additional control
>   over the plot legend.

The following are optional `kwargs` that can be passed to the functions
of the `visualiation` module.

> time\_vector :
> :   `iterable` of values to treat as time points for the solutions. If
>     provided, the original solutions in the `MassSolution` input
>     will be converted into interpolating functions and the solutions are
>     recalculated based on the provided `time_vector`. If `None` then
>     the current `time` values will be used.
>
>     Default is `None`.
>
> plot\_function :
> :   > `str` representing the plotting function to use. Accepted values are
>     > the following:
>     >
>     > > For all functions:
>     > >
>     > > > - `"plot"` for a linear x-axis and a linear y-axis
>     > > >   via `Axes.plot()`
>     > > > - `"loglog"` for a logarithmic x-axis and a logarithmic y-axis
>     > > >   via `Axes.loglog()`
>     > >
>     > > In addition, for functions in
>     > > `time_profiles` and
>     > > `phase_portraits` submodules only:
>     > >
>     > > > - `"semilogx`” for a logarithmic x-axis and a linear y-axis
>     > > >   via `Axes.semilogx()`
>     > > > - `"semilogy"` for a linear x-axis and a logarithmic y-axis
>     > > >   via `Axes.semilogy()`
>
>     Default is `"plot"`.
>
> title :
> :   Either a `str` to set as the title or a `tuple` of length 2 where
>     the first value is the title string and the second value is a `dict`
>     of font options. Arguments are passed to the corresponding setter method
>     `Axes.set_title()`.
>
>     Default is `None`.
>
> xlabel :
> :   Either a `str` to set as the xlabel or a `tuple` of length 2 where
>     the first value is the xlabel string and the second value is a `dict`
>     of font options. Arguments are passed to the corresponding setter method
>     `Axes.set_xlabel()`.
>
>     Default is `None`. Not valid for `plot_tiled_phase_portraits()`
>
> ylabel :
> :   Either a `str` to set as the ylabel or a `tuple` of length 2 where
>     the first value is the ylabel string and the second value is a `dict`
>     of font options. Arguments are passed to the corresponding setter method
>     `Axes.set_ylabel()`.
>
>     Default is `None`. Not valid for `plot_tiled_phase_portraits()`
>
> xlim :
> :   `tuple` of form `(xmin, xmax)` containing numerical values
>     specifying the limits of the x-axis. Passed to the corresponding setter
>     method `Axes.set_xlim()`.
>     For `plot_tiled_phase_portraits()`, the limits will be applied to
>     all tiles containing phase portrait plots.
>
>     Default is `None`.
>
> ylim :
> :   `tuple` of form `(ymin, ymax)` containing numerical values
>     specifying the limits of the y-axis. Passed to the corresponding setter
>     method `Axes.set_ylim()`.
>     For `plot_tiled_phase_portraits()`, the limits will be applied to
>     all tiles containing phase portrait plots.
>
>     Default is `None`.
>
> xmargin :
> :   `float` value greater than -0.5 to set as the padding for the x-axis
>     data limits prior to autoscaling. Passed to the corresponding setter
>     method `Axes.set_xmargin()`.
>     For `plot_tiled_phase_portraits()`, the margins will be applied to
>     all tiles containing phase portrait plots.
>
>     Default is `0.15` for `plot_tiled_phase_portraits()`,
>     otherwise `None`.
>
> ymargin :
> :   `float` value greater than -0.5 to set as the padding for the y-axis
>     data limits prior to autoscaling. Passed to the corresponding setter
>     method `Axes.set_ymargin()`.
>     For `plot_tiled_phase_portraits()`, the margins will be applied to
>     all tiles containing phase portrait plots.
>
>     Default is `0.15` for `plot_tiled_phase_portraits()`,
>     otherwise `None`.
>
> color :
> :   Value or `iterable` of values representing valid
>     `matplotlib.colors` values to use as line colors. If a single color
>     is provided, that color will be applied to all solutions being plotted.
>     If an `iterable` of color values is provided, it must be equal to the
>     number of solutions to be plotted.
>     For `plot_tiled_phase_portraits()`, the colors will be applied to
>     all tiles containing phase portrait plots.
>
>     Default is `None`. Ignored if the kwarg `prop_cycler` is also
>     provided.
>
> linestyle :
> :   Value or `iterable` of values representing valid `matplotlib`
>     linestyles. If a single linestyle is provided, that linestyle will be
>     applied to all solutions being plotted. If an `iterable` is provided,
>     it must be equal to the number of solutions to be plotted.
>     For `plot_tiled_phase_portraits()`, the linestyles will be applied
>     to all tiles containing phase portrait plots.
>
>     Default is `None` to use default value in `matplotlib.rcsetup`.
>     Ignored if the kwarg `prop_cycler` is also provided.
>
> linewidth :
> :   `float` value representing the linewidth (in points) to set.
>
>     Default is `None` to use default value in `matplotlib.rcsetup`.
>     Ignored if the kwarg `prop_cycler` is also provided.
>
> marker :
> :   Value or `iterable` of values representing valud `matplotlib`
>     marker values to use as line markers. If a single marker is provided,
>     that marker will be applied to all solutions being plotted.
>     If an `iterable` is provided, it must be equal to the number of
>     solutions to be plotted.
>     For `plot_tiled_phase_portraits()`, the markers will be applied
>     to all tiles containing phase portrait plots.
>
>     For functions in `comparision`, default is `"o"`, otherwise
>     default is `None` to use default value in `matplotlib.rcsetup`.
>     Ignored if the kwarg `prop_cycler` is also provided.
>
> markersize :
> :   `float` value representing the size of the marker (in points) to set.
>     For `plot_tiled_phase_portraits()`, the markersizes will be applied
>     to all tiles containing phase portrait plots. Ignored if the kwarg
>     `prop_cycler` is also provided.
>
>     Default is `None` to use default value in `matplotlib.rcsetup`.
>     Ignored if the kwarg `prop_cycler` is also provided.
>
> grid :
> :   Either a `bool` or a `tuple` of form `(which, axis)` where the
>     values for `which` and `axis` are one of the following:
>
>     > - `which` arguement must be `"major"`, `"minor"`, or `"both"`
>     > - `axis` argument must be `"x"`, `"y"`, or `"both"`
>
>     If `grid=False` then grid lines will be removed.
>     If `grid=True`, then grid lines will be created based on the
>     default values in `matplotlib.rcsetup` unless overridden by
>     `grid_color`, `grid_linestyle`, or `grid_linewidth` kwargs. Passed
>     to the setter method `Axes.grid()`.
>     For `plot_tiled_phase_portraits()`, the grid lines will be
>     applied to all tiles containing phase portrait plots.
>
>     Default is `None`.
>
> grid\_color :
> :   Value representing a valid `matplotlib.colors` value to use as
>     the color of the grid lines. For `plot_tiled_phase_portraits()`,
>     the color of the grid lines will be applied to all tiles containing
>     phase portrait plots.
>
>     Default is `None` to use default value in `matplotlib.rcsetup`.
>     Ignored if the kwarg `grid` is `None`.
>
> grid\_linestyle :
> :   Value representing a valid `matplotlib` value to use as the
>     linestyles of the grid lines. For `plot_tiled_phase_portraits()`,
>     the linestyle of the grid lines will be applied to all tiles containing
>     phase portrait plots.
>
>     Default is `None` to use default value in `matplotlib.rcsetup`.
>     Ignored if the kwarg `grid` is `None`.
>
> grid\_linewidth :
> :   > `float` value representing the grid linewidth (in points) to set.
>
>     Default is `None` to use default value in `matplotlib.rcsetup`.
>     Ignored if the kwarg `grid` is `None`.
>
> legend\_ncol :
> :   `int` indicating the number of columns to use in the legend.
>     If `None` the the following formula is applied:
>
>     ```
>     ncols = int(ceil(sqrt(N_total) / 3))
>     ```
>
>     where `N_total` is equal to the total number of solution lines.
>
>     Default is `None`. Not valid for `plot_tiled_phase_portraits()`
>
> deviation :
> :   `bool` indicating whether to plot the deviation from the initial value
>     for the observable variables.
>
>     Default is `False`.
>
> deviation\_zero\_centered :
> :   `bool` indicating whether to center deviations around zero for
>     for the observable variables.
>
>     Default is `False`.
>     Ignored if the kwarg `deviation=False`.
>
> deviation\_normalization :
> :   `str` indicating how to normalize the plotted deviation values. Can be
>     one of the following:
>
>     > - ‘initial’ to normalize the solution by dividing by the initial value
>     >   of the solution variable
>     > - ‘range’ to normalize the solution through dividing by the range of
>     >   solution values (maximum value - minimum value)
>
>     Default is `initial`.
>     Ignored if the kwarg `deviation=False`.
>
> annotate\_time\_points :
> :   Either the string `"endpoints"` or an `iterable` containing the
>     numerical values for the time points of interest to be annotated by
>     plotting points for the solutions at the given time points.
>     If `annotate_time_points="endpoints"` then only the initial and final
>     time points will be utilized. If `None` then no time points will be
>     annotated. For `plot_tiled_phase_portraits()`, the time points
>     will be applied to all tiles containing phase portrait plots.
>
>     Default is `None`.
>
> annotate\_time\_points\_color :
> :   Value or `iterable` of values representing valid
>     `matplotlib.colors` values to use as time point colors. If a single
>     color is provided, that color will be applied to all time points being
>     plotted. If an `iterable` of color values is provided, it must be
>     equal to the number of time points to be plotted.
>     For `plot_tiled_phase_portraits()`, the colors will be applied to
>     all tiles containing phase portrait plots.
>
>     Default is `None`.
>
> annotate\_time\_points\_marker :
> :   Value or `iterable` of values representing valud `matplotlib`
>     marker values to use as time point markers. If a single marker is
>     provided, that marker will be applied to all solutions being plotted.
>     If an `iterable` is provided, it must be equal to the number of time
>     points to be plotted.
>     For `plot_tiled_phase_portraits()`, the markers will be applied
>     to all tiles containing phase portrait plots.
>
>     Default is `None`.
>
> annotate\_time\_points\_markersize :
> :   `float` value representing the size of the marker (in points) to set.
>     For `plot_tiled_phase_portraits()`, the markersizes will be applied
>     to all tiles containing phase portrait plots.
>
>     Default is `None` to use default value in `matplotlib.rcsetup`.
>
> annotate\_time\_points\_labels :
> :   `bool` indicating whether to annotate the time points with their
>     labels on the plot itself.
>
>     Default is `False`.
>
> annotate\_time\_points\_legend :
> :   A `str` representing the location of the legend, or an `int`
>     between 0 and 14 (inclusive) corresponding to the location to use for
>     the legend of annotated time points. Cannot be the same location value
>     as the plot’s `legend` location. If `None`, no legend is created.
>
>     Default is `None`.
>
> prop\_cycle :
> :   A valid `matplotlib.rcsetup.cycler()` instance to use in the plot.
>     If provided, then the `color`, `linestyle`, `linewidth`,
>     `marker`, and `markersize` kwargs are ignored.
>
>     Default is `None`.
>
> mean\_line\_alpha :
> :   `float` indicating the alpha (opacity) value to use for the line
>     representing the mean solution.
>
>     Default is `1.0`.
>     Only valid for ensemble visualization functions.
>
> interval\_fill\_alpha :
> :   `float` indicating the alpha (opacity) value to use in shading the
>     interval.
>
>     Default is `0.5`.
>     Only valid for ensemble visualization functions.
>
> interval\_border\_alpha :
> :   `float` indicating the alpha (opacity) value for border lines of the
>     interval.
>
>     Default is `0.5`.
>     Only valid for ensemble visualization functions.
>
> CI\_distribution :
> :   Either `"t"` to calculate the confidence interval using a
>     t-distribution or `"z"` to calculate the confidence interval using a
>     z-distribution.
>
>     Default is `"t"`.
>     Only valid for ensemble visualization functions.
>
> tile\_ticks\_on :
> :   `bool` indicating whether to leave tick marks on tiles containing
>     phase portraits.
>
>     Default is `False`.
>     Only valid for `plot_tiled_phase_portraits()`.
>
> tile\_xlabel\_fontdict :
> :   `Font properties`
>     to set using a `dict`. Applied to all tile ylabels.
>
>     Default is `None`.
>     Only valid for `plot_tiled_phase_portraits()`.
>
> tile\_ylabel\_fontdict :
> :   `Font properties`
>     to set using a `dict`. Applied to all tile ylabels.
>
>     Default is `None`.
>     Only valid for `plot_tiled_phase_portraits()`.
>
> data\_tile\_fontsize :
> :   Valid `fontsize` value
>     representing the fontsize to be used for the data values on tiles
>     displaying additional data.
>
>     Default is `large`.
>     Only valid for `plot_tiled_phase_portraits()`. Ignored if no
>     additional data is provided (i.e. `additional_data=None`).
>
> data\_tile\_color :
> :   Value representing a valid `matplotlib.colors` value to use as
>     the facecolor of the tiles displaying additional data.
>
>     Default is `None` to utilize the color `"lightgray"`.
>     Only valid for `plot_tiled_phase_portraits()`. Ignored if no
>     additional data is provided (i.e. `additional_data=None`).
>
> diag\_tile\_color :
> :   Value representing a valid `matplotlib.colors` value to use as
>     the facecolor of the tiles on the diagonal.
>
>     Default is `None` to utilize the color `"black"`.
>     Only valid for `plot_tiled_phase_portraits()`.
>
> empty\_tile\_color :
> :   Value representing a valid `matplotlib.colors` value to use as
>     the facecolor of the empty tiles.
>
>     Default is `None` to utilize the color `"white"`.
>     Only valid for `plot_tiled_phase_portraits()`.
>
> xy\_line :
> :   Whether to plot a line with equation `y=x`.
>     If `xy_line=True`, then the line will be created based on the default
>     values in `matplotlib.rcsetup` unless overridden by
>     `xy_linecolor`, `xy_linestyle`, or `xy_linewidth` kwargs.
>
>     Ignored if the kwarg `xy_line=False`.
>     Only valid for functions in the `comparison` submodule.
>
> xy\_linecolor :
> :   Value representing a valid `matplotlib.colors` value to use as
>     the color of the `y=x` line.
>
>     Default is `None` to use default value in `matplotlib.rcsetup`.
>     Ignored if the kwarg `xy_line=False`.
>     Only valid for functions in the `comparison` submodule.
>
> xy\_linestyle :
> :   Value representing a valid `matplotlib` value to use as the
>     style of the `y=x` line.
>
>     Default is `None` to use default value in `matplotlib.rcsetup`.
>     Ignored if the kwarg `xy_line=False`.
>     Only valid for functions in the `comparison` submodule.
>
> xy\_linewidth :
> :   > `float` value representing the linewidth (in points) to set for
>     > the `y=x` line.
>
>     Default is `None` to use default value in `matplotlib.rcsetup`.
>     Ignored if the kwarg `xy_line=False`.
>     Only valid for functions in the `comparison` submodule.
>
> xy\_legend :
> :   `str` indicating where to place a legend for the `y=x` line.
>
>     Default is `None`.
>     Ignored if the kwarg `xy_line=False`.
>     Only valid for functions in the `comparison` submodule.

###### Submodules¶

###### `mass.visualization.comparison`¶

Contains function for visually comparing values in various objects.

See the `mass.visualization` documentation for general information
on `mass.visualization` functions.

This module contains the following functions for visually comparing a set of
values in one object against a similar set of valeus in another object.

> - `plot_comparison()`

###### Module Contents¶

###### Functions¶

|  |  |
| --- | --- |
| `plot_comparison`(x, y, compare=None, observable=None, ax=None, legend=None, \*\*kwargs) | Plot values of two objects for comparision. |
| `get_comparison_default_kwargs`(function\_name) | Get default `kwargs` for plotting functions in `comparison`. |

`mass.visualization.comparison.``plot_comparison`(*x*, *y*, *compare=None*, *observable=None*, *ax=None*, *legend=None*, *\*\*kwargs*)[source]¶
:   Plot values of two objects for comparision.

    This function can take two `MassModel`, `ConcSolution`,
    `cobra.Solution`, or
    `pandas.Series` objects and plot them against one another in a
    calibration plot.

    Accepted `kwargs` are passed onto various `matplotlib` methods
    utilized in the function. See the `visualization` module
    documentation for more detailed information about the possible `kwargs`.

    Notes

    - If a `pandas.Series`, the index must correspond to the identifier
      of the assoicated object. (e.g. a metabolite identifier for
      `compare="concentrations"`, or a reaction identifier for
      `compare="Keqs"`)

    Parameters
    :   - **x** (*MassModel**,* *ConcSolution**,* *Solution**,* *Series*) – The object to access for x-axis values.
        - **y** (*MassModel**,* *ConcSolution**,* *Solution**,* *Series*) – The object to access for y-axis values.
        - **compare** (*str*) –

          The values to be compared. Must be one of the following:

          > - `"concentrations"` for `MassModel` and
          >   `ConcSolution` objects.
          > - `"Keqs"` for `MassModel` and
          >   `ConcSolution` objects.
          > - `"fluxes"` for `MassModel` and
          >   `cobra.Solution` objects.
          > - `"kfs"` for `MassModel` objects.

          Not required if both `x` and `y` are `pandas.Series`.
        - **observable** (*iterable*) – An iterable containing string identifiers of `mass` objects
          or the objects themselves corresponding to the object or index where
          the value is located.
        - **ax** (*matplotlib.axes.Axes**,* *None*) – An `Axes` instance to plot the data on.
          If `None` then the current axes instance is used.
        - **legend** (*iterable**,* *str**,* *int*) –

          There are three possible input formats for the legend:

          > 1. An iterable of legend labels as strings.
          > 2. A `str` representing the location of the legend, or an
          >    `int` between 0 and 14 (inclusive) corresponding to the
          >    legend location.
          > 3. An iterable of the format `(labels, loc)` to set both
          >    the legend labels and location, where `labels` and `loc`
          >    follows the labels specified in **1** and **2**.

          See the `visualization` documentation for more information
          about legend and valid legend locations.
        - **\*\*kwargs** –
          - plot\_function
          - title
          - xlabel
          - ylabel
          - xlim
          - ylim
          - grid
          - grid\_color
          - grid\_linestyle
          - grid\_linewidth
          - prop\_cycle
          - color
          - marker
          - markersize
          - legend\_ncol
          - xy\_line
          - xy\_linecolor
          - xy\_linewidth
          - xy\_linestyle
          - xy\_legend

          See `visualization` documentation for more information on
          optional `kwargs`.

    Returns
    :   **ax** – The `Axes` instance containing the newly
        created plot.

    Return type
    :   matplotlib.axes.Axes

`mass.visualization.comparison.``get_comparison_default_kwargs`(*function\_name*)[source]¶
:   Get default `kwargs` for plotting functions in `comparison`.

    Parameters
    :   **function\_name** (*str*) –

        The name of the plotting function to get the `kwargs` for.
        Valid values include the following:

        > - `"plot_comparison"`

    Returns
    :   Default `kwarg` values for the given `function_name`.

    Return type
    :   dict

###### `mass.visualization.phase_portraits`¶

Contains function for visualizing phase portraits of simulation results.

See the `mass.visualization` documentation for general information
on `mass.visualization` functions.

This module contains the following functions for visualization of
time-dependent solutions returned in `MassSolution`s after
simulation of models.

> - `plot_phase_portrait()`
> - `plot_ensemble_phase_portrait()`
> - `plot_tiled_phase_portrait()`

###### Module Contents¶

###### Functions¶

|  |  |
| --- | --- |
| `plot_phase_portrait`(mass\_solution, x, y, ax=None, legend=None, \*\*kwargs) | Plot phase portraits of solutions in a given `MassSolution`. |
| `plot_ensemble_phase_portrait`(mass\_solution\_list, x, y, ax=None, legend=None, \*\*kwargs) | Plot a phase portrait for an ensemble of class:~.MassSolution objects. |
| `plot_tiled_phase_portraits`(mass\_solution, observable=None, ax=None, plot\_tile\_placement=’all’, additional\_data=None, \*\*kwargs) | Plot phase portraits of solutions in a given `MassSolution`. |
| `get_phase_portrait_default_kwargs`(function\_name) | Get default `kwargs` for plotting functions in `phase_portraits`. |

`mass.visualization.phase_portraits.``plot_phase_portrait`(*mass\_solution*, *x*, *y*, *ax=None*, *legend=None*, *\*\*kwargs*)[source]¶
:   Plot phase portraits of solutions in a given `MassSolution`.

    Accepted `kwargs` are passed onto various `matplotlib` methods
    utilized in the function. See the `visualization` module
    documentation for more detailed information about the possible `kwargs`.

    Notes

    - To prevent any changes to the original `MassSolution`, a copy of
      the `MassSolution` is created and used.

    Parameters
    :   - **mass\_solution** (*MassSolution*) – The `MassSolution` containing the time-dependent solutions
          to be plotted.
        - **x** (`mass` object or its string identifier) – The string identifier of a `mass` object or the object itself
          that corresponds to the key for the desired solution in the
          `MassSolution` for the x-axis of the phase portrait.
        - **y** (`mass` object or its string identifier) – The string identifier of a `mass` object or the object itself
          that corresponds to the key for the desired solution in the
          `MassSolution` for the y-axis of the phase portrait.
        - **ax** (*matplotlib.axes.Axes**,* *None*) – An `Axes` instance to plot the data on.
          If `None` then the current axes instance is used.
        - **legend** (*iterable**,* *str**,* *int*) –

          There are three possible input formats for the legend:

          > 1. An iterable of legend labels as strings.
          > 2. A `str` representing the location of the legend, or an
          >    `int` between 0 and 14 (inclusive) corresponding to the
          >    legend location.
          > 3. An iterable of the format `(labels, loc)` to set both
          >    the legend labels and location, where `labels` and `loc`
          >    follows the format specified in **1** and **2**.

          See the `visualization` documentation for more information
          about legend and valid legend locations.
        - **\*\*kwargs** –
          - time\_vector
          - plot\_function
          - title
          - xlabel
          - ylabel
          - xlim
          - ylim
          - grid
          - grid\_color
          - grid\_linestyle
          - grid\_linewidth
          - prop\_cycle
          - color
          - linestyle
          - linewidth
          - marker
          - markersize
          - legend\_ncol
          - annotate\_time\_points
          - annotate\_time\_points\_color
          - annotate\_time\_points\_marker
          - annotate\_time\_points\_markersize
          - annotate\_time\_points\_labels
          - annotate\_time\_points\_legend
          - deviation
          - deviation\_zero\_centered
          - deviation\_normalization

          See `visualization` documentation for more information on
          optional `kwargs`.

    Returns
    :   **ax** – The `Axes` instance containing the newly
        created plot.

    Return type
    :   matplotlib.axes.Axes

`mass.visualization.phase_portraits.``plot_ensemble_phase_portrait`(*mass\_solution\_list*, *x*, *y*, *ax=None*, *legend=None*, *\*\*kwargs*)[source]¶
:   Plot a phase portrait for an ensemble of class:~.MassSolution objects.

    The plotted lines represent the mean for the values of a particular
    solution specified in `observable`.

    Accepted `kwargs` are passed onto various `matplotlib` methods
    utilized in the function. See the `visualization` module
    documentation for more detailed information about the possible `kwargs`.

    Notes

    - To prevent any changes to the original `MassSolution`, copies of
      `MassSolution` objects are created and used.

    Parameters
    :   - **mass\_solution\_list** (*iterable*) – An iterable of `MassSolution` objects containing the
          time-dependent solutions to be plotted.
        - **x** (`mass` object or its string identifier) – The string identifier of a `mass` object or the object itself
          that corresponds to the key for the desired solution in the
          `MassSolution` for the x-axis of the phase portrait.
        - **y** (`mass` object or its string identifier) – The string identifier of a `mass` object or the object itself
          that corresponds to the key for the desired solution in the
          `MassSolution` for the y-axis of the phase portrait.
        - **ax** (*matplotlib.axes.Axes**,* *None*) – An `Axes` instance to plot the data on.
          If `None` then the current axes instance is used.
        - **legend** (*iterable**,* *str**,* *int*) –

          There are three possible input formats for the legend:

          > 1. An iterable of legend labels as strings.
          > 2. A `str` representing the location of the legend, or an
          >    `int` between 0 and 14 (inclusive) corresponding to the
          >    legend location.
          > 3. An iterable of the format `(labels, loc)` to set both
          >    the legend labels and location, where `labels` and `loc`
          >    follows the format specified in **1** and **2**.

          See the `visualization` documentation for more information
          about legend and valid legend locations.
        - **\*\*kwargs** –
          - time\_vector
          - plot\_function
          - title
          - xlabel
          - ylabel
          - xlim
          - ylim
          - grid
          - grid\_color
          - grid\_linestyle
          - grid\_linewidth
          - prop\_cycle
          - color
          - linestyle
          - linewidth
          - marker
          - markersize
          - legend\_ncol
          - annotate\_time\_points
          - annotate\_time\_points\_color
          - annotate\_time\_points\_marker
          - annotate\_time\_points\_markersize
          - annotate\_time\_points\_labels
          - annotate\_time\_points\_legend
          - deviation
          - deviation\_zero\_centered
          - deviation\_normalization

          See `visualization` documentation for more information on
          optional `kwargs`.

    Returns
    :   **ax** – The `Axes` instance containing the newly
        created plot.

    Return type
    :   matplotlib.axes.Axes

`mass.visualization.phase_portraits.``plot_tiled_phase_portraits`(*mass\_solution*, *observable=None*, *ax=None*, *plot\_tile\_placement='all'*, *additional\_data=None*, *\*\*kwargs*)[source]¶
:   Plot phase portraits of solutions in a given `MassSolution`.

    Accepted `kwargs` are passed onto various matplotlib methods in utilized
    in the function. See the `visualization` module documentation
    for more detailed information about the possible `kwargs`.

    Notes

    - To prevent any changes to the original `MassSolution`, a copy of
      the `MassSolution` will be created and used.
    - `i` and `j` represent the number of rows and columns, respectively.

    Parameters
    :   - **mass\_solution** (*MassSolution*) – The `MassSolution` containing the time-dependent solutions
          to be plotted.
        - **observable** (*iterable*) – An iterable containing string identifiers of the `mass` objects
          or the objects themselves that correspond to the keys for the desired
          solutions in the `MassSolution`.
        - **ax** (*matplotlib.axes.Axes**,* *None*) – An `Axes` instance to plot the data on.
          If `None` then the current axes instance is used.
        - **plot\_tile\_placement** (*str*) –

          A string representing the location to place the tiles containing
          phase portrait plots. Must be one of the following:

          > - `"lower"` to place plot tiles on the lower left triangular
          >   section `(i < j)` on the figure tiles.
          > - `"upper"` to place plot tiles on the upper right triangular
          >   section `(i > j)` on the figure tiles.
          > - `all` to place plot tiles on the lower left triangular
          >   section `(i < j)` AND on the upper right triangular section
          >   `(i > j)` on the figure tiles.
        - **additional\_data** (*array\_like**,* *None*) – A matrix of shape `(N, N)` where `N_obs` is the number
          of observables provided, or the number of keys in the
          `MassSolution` if `observable=None`. The value at `(i, j)`
          of the matrix must correspond to the empty tile that the data should
          be displayed on. All other values are ignored. If `None` then no
          data will be displayed and tiles will be left empty.
        - **\*\*kwargs** –
          - time\_vector
          - plot\_function
          - title
          - xlim
          - ylim
          - grid
          - grid\_color
          - grid\_linestyle
          - grid\_linewidth
          - prop\_cycle
          - color
          - linestyle
          - linewidth
          - marker
          - markersize
          - annotate\_time\_points
          - annotate\_time\_points\_color
          - annotate\_time\_points\_marker
          - annotate\_time\_points\_markersize
          - annotate\_time\_points\_legend
          - annotate\_time\_points\_zorder
          - tile\_ticks\_on
          - tile\_xlabel\_fontdict
          - tile\_ylabel\_fontdict
          - data\_tile\_fontsize
          - data\_tile\_color
          - diag\_tile\_color
          - empty\_tile\_color

          See `visualization` documentation for more information on
          optional `kwargs`.

    Returns
    :   **ax** – The `Axes` instance containing the newly
        created plot.

    Return type
    :   matplotlib.axes.Axes

`mass.visualization.phase_portraits.``get_phase_portrait_default_kwargs`(*function\_name*)[source]¶
:   Get default `kwargs` for plotting functions in `phase_portraits`.

    Parameters
    :   **function\_name** (*str*) –

        The name of the plotting function to get the `kwargs` for.
        Valid values include the following:

        > - `"plot_phase_portrait"`
        > - `"plot_tiled_phase_portraits"`

    Returns
    :   Default `kwarg` values for the given `function_name`.

    Return type
    :   dict

###### `mass.visualization.time_profiles`¶

Contains function for visualizing time profiles of simulation results.

See the `mass.visualization` documentation for general information
on `mass.visualization` functions.

This module contains the following functions for visualization of
time-dependent solutions returned in `MassSolution`s after
simulation of models.

> - `plot_time_profile()`
> - `plot_ensemble_time_profile()`

###### Module Contents¶

###### Functions¶

|  |  |
| --- | --- |
| `plot_time_profile`(mass\_solution, observable=None, ax=None, legend=None, \*\*kwargs) | Plot time profiles of solutions in a given `MassSolution`. |
| `plot_ensemble_time_profile`(mass\_solution\_list, observable, ax=None, legend=None, interval\_type=None, \*\*kwargs) | Plot time profiles for an ensemble of class:~.MassSolution objects. |
| `get_time_profile_default_kwargs`(function\_name) | Get default `kwargs` for plotting functions in `time_profiles`. |

`mass.visualization.time_profiles.``plot_time_profile`(*mass\_solution*, *observable=None*, *ax=None*, *legend=None*, *\*\*kwargs*)[source]¶
:   Plot time profiles of solutions in a given `MassSolution`.

    Accepted `kwargs` are passed onto various `matplotlib` methods
    utilized in the function. See the `visualization` module
    documentation for more detailed information about the possible `kwargs`.

    Notes

    - To prevent any changes to the original `MassSolution`, a copy of
      the `MassSolution` is created and used.

    Parameters
    :   - **mass\_solution** (*MassSolution*) – The `MassSolution` containing the time-dependent solutions
          to be plotted.
        - **observable** (*iterable**,* *None*) – An iterable containing string identifiers of the `mass` objects
          or the objects themselves that correspond to the keys for the desired
          solutions in the `MassSolution`. If `None` then all solutions
          are plotted.
        - **ax** (*matplotlib.axes.Axes**,* *None*) – An `Axes` instance to plot the data on.
          If `None` then the current axes instance is used.
        - **legend** (*iterable**,* *str**,* *int*) –

          There are three possible input formats for the legend:

          > 1. An iterable of legend labels as strings.
          > 2. A `str` representing the location of the legend, or an
          >    `int` between 0 and 14 (inclusive) corresponding to the
          >    legend location.
          > 3. An iterable of the format `(labels, loc)` to set both
          >    the legend labels and location, where `labels` and `loc`
          >    follows the labels specified in **1** and **2**.

          See the `visualization` documentation for more information
          about legend and valid legend locations.
        - **\*\*kwargs** –
          - time\_vector
          - plot\_function
          - title
          - xlabel
          - ylabel
          - xlim
          - ylim
          - grid
          - grid\_color
          - grid\_linestyle
          - grid\_linewidth
          - prop\_cycle
          - color
          - linestyle
          - linewidth
          - marker
          - markersize
          - legend\_ncol
          - annotate\_time\_points
          - annotate\_time\_points\_color
          - annotate\_time\_points\_marker
          - annotate\_time\_points\_markersize
          - annotate\_time\_points\_labels
          - annotate\_time\_points\_legend
          - annotate\_time\_points\_zorder
          - deviation
          - deviation\_zero\_centered
          - deviation\_normalization

          See `visualization` documentation for more information on
          optional `kwargs`.

    Returns
    :   **ax** – The `Axes` instance containing the newly
        created plot.

    Return type
    :   matplotlib.axes.Axes

`mass.visualization.time_profiles.``plot_ensemble_time_profile`(*mass\_solution\_list*, *observable*, *ax=None*, *legend=None*, *interval\_type=None*, *\*\*kwargs*)[source]¶
:   Plot time profiles for an ensemble of class:~.MassSolution objects.

    The plotted lines represent the mean for the values of a particular
    solution specified in `observable`.

    Accepted `kwargs` are passed onto various `matplotlib` methods
    utilized in the function. See the `visualization` module
    documentation for more detailed information about the possible `kwargs`.

    Notes

    - To prevent any changes to the original `MassSolution`, copies of
      `MassSolution` objects are created and used.

    Parameters
    :   - **mass\_solution\_list** (*iterable*) – An iterable of `MassSolution` objects containing the
          time-dependent solutions to be plotted.
        - **observable** (*iterable*) – An iterable containing string identifiers of the `mass` objects
          or the objects themselves that correspond to the keys for the desired
          solutions in the `MassSolution`.
        - **ax** (*matplotlib.axes.Axes**,* *None*) – An `Axes` instance to plot the data on.
          If `None` then the current axes instance is used.
        - **legend** (*iterable**,* *str**,* *int*) –

          There are three possible input formats for the legend:

          > 1. An iterable of legend labels as strings.
          > 2. A `str` representing the location of the legend, or an
          >    `int` between 0 and 14 (inclusive) corresponding to the
          >    legend location.
          > 3. An iterable of the format `(labels, loc)` to set both
          >    the legend labels and location, where `labels` and `loc`
          >    follows the labels specified in **1** and **2**.

          See the `visualization` documentation for more information
          about legend and valid legend locations.
        - **interval\_type** (*str**,* *None*) –

          The type of interval to display with the plotted mean of the solution.
          Can be one of the following:

          > - `"range"`: Interval shading occurs from the minimum to the
          >   maximum value for each time point.
          > - `"CI="`: Interval shading occurs for a confidence interval.
          >   (e.g. confidence interval of 95% is specified as `"CI=95.0"`.)
          > - `None` to prevent interval shading from occurring.

          Default is `None`
        - **\*\*kwargs** –
          - time\_vector
          - plot\_function
          - title
          - xlabel
          - ylabel
          - xlim
          - ylim
          - grid
          - grid\_color
          - grid\_linestyle
          - grid\_linewidth
          - prop\_cycle
          - color
          - linestyle
          - linewidth
          - marker
          - markersize
          - legend\_ncol
          - annotate\_time\_points
          - annotate\_time\_points\_color
          - annotate\_time\_points\_marker
          - annotate\_time\_points\_markersize
          - annotate\_time\_points\_labels
          - annotate\_time\_points\_legend
          - deviation
          - deviation\_zero\_centered
          - deviation\_normalization
          - mean\_line\_alpha
          - interval\_fill\_alpha
          - interval\_border\_alpha
          - CI\_distribution

          See `visualization` documentation for more information on
          optional `kwargs`.

    Returns
    :   **ax** – The `Axes` instance containing the newly
        created plot.

    Return type
    :   matplotlib.axes.Axes

`mass.visualization.time_profiles.``get_time_profile_default_kwargs`(*function\_name*)[source]¶
:   Get default `kwargs` for plotting functions in `time_profiles`.

    Parameters
    :   **function\_name** (*str*) –

        The name of the plotting function to get the `kwargs` for.
        Valid values include the following:

        > - `"plot_time_profile"`

    Returns
    :   Default `kwarg` values for the given `function_name`.

    Return type
    :   dict

###### `mass.visualization.visualization_util`¶

Contains internal functions common to various visualization functions.

Warning

The functions found in this module are NOT intended for direct use.

###### Package Contents¶

###### Functions¶

|  |  |
| --- | --- |
| `plot_comparison`(x, y, compare=None, observable=None, ax=None, legend=None, \*\*kwargs) | Plot values of two objects for comparision. |
| `plot_ensemble_time_profile`(mass\_solution\_list, observable, ax=None, legend=None, interval\_type=None, \*\*kwargs) | Plot time profiles for an ensemble of class:~.MassSolution objects. |
| `plot_time_profile`(mass\_solution, observable=None, ax=None, legend=None, \*\*kwargs) | Plot time profiles of solutions in a given `MassSolution`. |
| `plot_ensemble_phase_portrait`(mass\_solution\_list, x, y, ax=None, legend=None, \*\*kwargs) | Plot a phase portrait for an ensemble of class:~.MassSolution objects. |
| `plot_phase_portrait`(mass\_solution, x, y, ax=None, legend=None, \*\*kwargs) | Plot phase portraits of solutions in a given `MassSolution`. |
| `plot_tiled_phase_portraits`(mass\_solution, observable=None, ax=None, plot\_tile\_placement=’all’, additional\_data=None, \*\*kwargs) | Plot phase portraits of solutions in a given `MassSolution`. |

`mass.visualization.``plot_comparison`(*x*, *y*, *compare=None*, *observable=None*, *ax=None*, *legend=None*, *\*\*kwargs*)[source]¶
:   Plot values of two objects for comparision.

    This function can take two `MassModel`, `ConcSolution`,
    `cobra.Solution`, or
    `pandas.Series` objects and plot them against one another in a
    calibration plot.

    Accepted `kwargs` are passed onto various `matplotlib` methods
    utilized in the function. See the `visualization` module
    documentation for more detailed information about the possible `kwargs`.

    Notes

    - If a `pandas.Series`, the index must correspond to the identifier
      of the assoicated object. (e.g. a metabolite identifier for
      `compare="concentrations"`, or a reaction identifier for
      `compare="Keqs"`)

    Parameters
    :   - **x** (*MassModel**,* *ConcSolution**,* *Solution**,* *Series*) – The object to access for x-axis values.
        - **y** (*MassModel**,* *ConcSolution**,* *Solution**,* *Series*) – The object to access for y-axis values.
        - **compare** (*str*) –

          The values to be compared. Must be one of the following:

          > - `"concentrations"` for `MassModel` and
          >   `ConcSolution` objects.
          > - `"Keqs"` for `MassModel` and
          >   `ConcSolution` objects.
          > - `"fluxes"` for `MassModel` and
          >   `cobra.Solution` objects.
          > - `"kfs"` for `MassModel` objects.

          Not required if both `x` and `y` are `pandas.Series`.
        - **observable** (*iterable*) – An iterable containing string identifiers of `mass` objects
          or the objects themselves corresponding to the object or index where
          the value is located.
        - **ax** (*matplotlib.axes.Axes**,* *None*) – An `Axes` instance to plot the data on.
          If `None` then the current axes instance is used.
        - **legend** (*iterable**,* *str**,* *int*) –

          There are three possible input formats for the legend:

          > 1. An iterable of legend labels as strings.
          > 2. A `str` representing the location of the legend, or an
          >    `int` between 0 and 14 (inclusive) corresponding to the
          >    legend location.
          > 3. An iterable of the format `(labels, loc)` to set both
          >    the legend labels and location, where `labels` and `loc`
          >    follows the labels specified in **1** and **2**.

          See the `visualization` documentation for more information
          about legend and valid legend locations.
        - **\*\*kwargs** –
          - plot\_function
          - title
          - xlabel
          - ylabel
          - xlim
          - ylim
          - grid
          - grid\_color
          - grid\_linestyle
          - grid\_linewidth
          - prop\_cycle
          - color
          - marker
          - markersize
          - legend\_ncol
          - xy\_line
          - xy\_linecolor
          - xy\_linewidth
          - xy\_linestyle
          - xy\_legend

          See `visualization` documentation for more information on
          optional `kwargs`.

    Returns
    :   **ax** – The `Axes` instance containing the newly
        created plot.

    Return type
    :   matplotlib.axes.Axes

`mass.visualization.``plot_ensemble_time_profile`(*mass\_solution\_list*, *observable*, *ax=None*, *legend=None*, *interval\_type=None*, *\*\*kwargs*)[source]¶
:   Plot time profiles for an ensemble of class:~.MassSolution objects.

    The plotted lines represent the mean for the values of a particular
    solution specified in `observable`.

    Accepted `kwargs` are passed onto various `matplotlib` methods
    utilized in the function. See the `visualization` module
    documentation for more detailed information about the possible `kwargs`.

    Notes

    - To prevent any changes to the original `MassSolution`, copies of
      `MassSolution` objects are created and used.

    Parameters
    :   - **mass\_solution\_list** (*iterable*) – An iterable of `MassSolution` objects containing the
          time-dependent solutions to be plotted.
        - **observable** (*iterable*) – An iterable containing string identifiers of the `mass` objects
          or the objects themselves that correspond to the keys for the desired
          solutions in the `MassSolution`.
        - **ax** (*matplotlib.axes.Axes**,* *None*) – An `Axes` instance to plot the data on.
          If `None` then the current axes instance is used.
        - **legend** (*iterable**,* *str**,* *int*) –

          There are three possible input formats for the legend:

          > 1. An iterable of legend labels as strings.
          > 2. A `str` representing the location of the legend, or an
          >    `int` between 0 and 14 (inclusive) corresponding to the
          >    legend location.
          > 3. An iterable of the format `(labels, loc)` to set both
          >    the legend labels and location, where `labels` and `loc`
          >    follows the labels specified in **1** and **2**.

          See the `visualization` documentation for more information
          about legend and valid legend locations.
        - **interval\_type** (*str**,* *None*) –

          The type of interval to display with the plotted mean of the solution.
          Can be one of the following:

          > - `"range"`: Interval shading occurs from the minimum to the
          >   maximum value for each time point.
          > - `"CI="`: Interval shading occurs for a confidence interval.
          >   (e.g. confidence interval of 95% is specified as `"CI=95.0"`.)
          > - `None` to prevent interval shading from occurring.

          Default is `None`
        - **\*\*kwargs** –
          - time\_vector
          - plot\_function
          - title
          - xlabel
          - ylabel
          - xlim
          - ylim
          - grid
          - grid\_color
          - grid\_linestyle
          - grid\_linewidth
          - prop\_cycle
          - color
          - linestyle
          - linewidth
          - marker
          - markersize
          - legend\_ncol
          - annotate\_time\_points
          - annotate\_time\_points\_color
          - annotate\_time\_points\_marker
          - annotate\_time\_points\_markersize
          - annotate\_time\_points\_labels
          - annotate\_time\_points\_legend
          - deviation
          - deviation\_zero\_centered
          - deviation\_normalization
          - mean\_line\_alpha
          - interval\_fill\_alpha
          - interval\_border\_alpha
          - CI\_distribution

          See `visualization` documentation for more information on
          optional `kwargs`.

    Returns
    :   **ax** – The `Axes` instance containing the newly
        created plot.

    Return type
    :   matplotlib.axes.Axes

`mass.visualization.``plot_time_profile`(*mass\_solution*, *observable=None*, *ax=None*, *legend=None*, *\*\*kwargs*)[source]¶
:   Plot time profiles of solutions in a given `MassSolution`.

    Accepted `kwargs` are passed onto various `matplotlib` methods
    utilized in the function. See the `visualization` module
    documentation for more detailed information about the possible `kwargs`.

    Notes

    - To prevent any changes to the original `MassSolution`, a copy of
      the `MassSolution` is created and used.

    Parameters
    :   - **mass\_solution** (*MassSolution*) – The `MassSolution` containing the time-dependent solutions
          to be plotted.
        - **observable** (*iterable**,* *None*) – An iterable containing string identifiers of the `mass` objects
          or the objects themselves that correspond to the keys for the desired
          solutions in the `MassSolution`. If `None` then all solutions
          are plotted.
        - **ax** (*matplotlib.axes.Axes**,* *None*) – An `Axes` instance to plot the data on.
          If `None` then the current axes instance is used.
        - **legend** (*iterable**,* *str**,* *int*) –

          There are three possible input formats for the legend:

          > 1. An iterable of legend labels as strings.
          > 2. A `str` representing the location of the legend, or an
          >    `int` between 0 and 14 (inclusive) corresponding to the
          >    legend location.
          > 3. An iterable of the format `(labels, loc)` to set both
          >    the legend labels and location, where `labels` and `loc`
          >    follows the labels specified in **1** and **2**.

          See the `visualization` documentation for more information
          about legend and valid legend locations.
        - **\*\*kwargs** –
          - time\_vector
          - plot\_function
          - title
          - xlabel
          - ylabel
          - xlim
          - ylim
          - grid
          - grid\_color
          - grid\_linestyle
          - grid\_linewidth
          - prop\_cycle
          - color
          - linestyle
          - linewidth
          - marker
          - markersize
          - legend\_ncol
          - annotate\_time\_points
          - annotate\_time\_points\_color
          - annotate\_time\_points\_marker
          - annotate\_time\_points\_markersize
          - annotate\_time\_points\_labels
          - annotate\_time\_points\_legend
          - annotate\_time\_points\_zorder
          - deviation
          - deviation\_zero\_centered
          - deviation\_normalization

          See `visualization` documentation for more information on
          optional `kwargs`.

    Returns
    :   **ax** – The `Axes` instance containing the newly
        created plot.

    Return type
    :   matplotlib.axes.Axes

`mass.visualization.``plot_ensemble_phase_portrait`(*mass\_solution\_list*, *x*, *y*, *ax=None*, *legend=None*, *\*\*kwargs*)[source]¶
:   Plot a phase portrait for an ensemble of class:~.MassSolution objects.

    The plotted lines represent the mean for the values of a particular
    solution specified in `observable`.

    Accepted `kwargs` are passed onto various `matplotlib` methods
    utilized in the function. See the `visualization` module
    documentation for more detailed information about the possible `kwargs`.

    Notes

    - To prevent any changes to the original `MassSolution`, copies of
      `MassSolution` objects are created and used.

    Parameters
    :   - **mass\_solution\_list** (*iterable*) – An iterable of `MassSolution` objects containing the
          time-dependent solutions to be plotted.
        - **x** (`mass` object or its string identifier) – The string identifier of a `mass` object or the object itself
          that corresponds to the key for the desired solution in the
          `MassSolution` for the x-axis of the phase portrait.
        - **y** (`mass` object or its string identifier) – The string identifier of a `mass` object or the object itself
          that corresponds to the key for the desired solution in the
          `MassSolution` for the y-axis of the phase portrait.
        - **ax** (*matplotlib.axes.Axes**,* *None*) – An `Axes` instance to plot the data on.
          If `None` then the current axes instance is used.
        - **legend** (*iterable**,* *str**,* *int*) –

          There are three possible input formats for the legend:

          > 1. An iterable of legend labels as strings.
          > 2. A `str` representing the location of the legend, or an
          >    `int` between 0 and 14 (inclusive) corresponding to the
          >    legend location.
          > 3. An iterable of the format `(labels, loc)` to set both
          >    the legend labels and location, where `labels` and `loc`
          >    follows the format specified in **1** and **2**.

          See the `visualization` documentation for more information
          about legend and valid legend locations.
        - **\*\*kwargs** –
          - time\_vector
          - plot\_function
          - title
          - xlabel
          - ylabel
          - xlim
          - ylim
          - grid
          - grid\_color
          - grid\_linestyle
          - grid\_linewidth
          - prop\_cycle
          - color
          - linestyle
          - linewidth
          - marker
          - markersize
          - legend\_ncol
          - annotate\_time\_points
          - annotate\_time\_points\_color
          - annotate\_time\_points\_marker
          - annotate\_time\_points\_markersize
          - annotate\_time\_points\_labels
          - annotate\_time\_points\_legend
          - deviation
          - deviation\_zero\_centered
          - deviation\_normalization

          See `visualization` documentation for more information on
          optional `kwargs`.

    Returns
    :   **ax** – The `Axes` instance containing the newly
        created plot.

    Return type
    :   matplotlib.axes.Axes

`mass.visualization.``plot_phase_portrait`(*mass\_solution*, *x*, *y*, *ax=None*, *legend=None*, *\*\*kwargs*)[source]¶
:   Plot phase portraits of solutions in a given `MassSolution`.

    Accepted `kwargs` are passed onto various `matplotlib` methods
    utilized in the function. See the `visualization` module
    documentation for more detailed information about the possible `kwargs`.

    Notes

    - To prevent any changes to the original `MassSolution`, a copy of
      the `MassSolution` is created and used.

    Parameters
    :   - **mass\_solution** (*MassSolution*) – The `MassSolution` containing the time-dependent solutions
          to be plotted.
        - **x** (`mass` object or its string identifier) – The string identifier of a `mass` object or the object itself
          that corresponds to the key for the desired solution in the
          `MassSolution` for the x-axis of the phase portrait.
        - **y** (`mass` object or its string identifier) – The string identifier of a `mass` object or the object itself
          that corresponds to the key for the desired solution in the
          `MassSolution` for the y-axis of the phase portrait.
        - **ax** (*matplotlib.axes.Axes**,* *None*) – An `Axes` instance to plot the data on.
          If `None` then the current axes instance is used.
        - **legend** (*iterable**,* *str**,* *int*) –

          There are three possible input formats for the legend:

          > 1. An iterable of legend labels as strings.
          > 2. A `str` representing the location of the legend, or an
          >    `int` between 0 and 14 (inclusive) corresponding to the
          >    legend location.
          > 3. An iterable of the format `(labels, loc)` to set both
          >    the legend labels and location, where `labels` and `loc`
          >    follows the format specified in **1** and **2**.

          See the `visualization` documentation for more information
          about legend and valid legend locations.
        - **\*\*kwargs** –
          - time\_vector
          - plot\_function
          - title
          - xlabel
          - ylabel
          - xlim
          - ylim
          - grid
          - grid\_color
          - grid\_linestyle
          - grid\_linewidth
          - prop\_cycle
          - color
          - linestyle
          - linewidth
          - marker
          - markersize
          - legend\_ncol
          - annotate\_time\_points
          - annotate\_time\_points\_color
          - annotate\_time\_points\_marker
          - annotate\_time\_points\_markersize
          - annotate\_time\_points\_labels
          - annotate\_time\_points\_legend
          - deviation
          - deviation\_zero\_centered
          - deviation\_normalization

          See `visualization` documentation for more information on
          optional `kwargs`.

    Returns
    :   **ax** – The `Axes` instance containing the newly
        created plot.

    Return type
    :   matplotlib.axes.Axes

`mass.visualization.``plot_tiled_phase_portraits`(*mass\_solution*, *observable=None*, *ax=None*, *plot\_tile\_placement='all'*, *additional\_data=None*, *\*\*kwargs*)[source]¶
:   Plot phase portraits of solutions in a given `MassSolution`.

    Accepted `kwargs` are passed onto various matplotlib methods in utilized
    in the function. See the `visualization` module documentation
    for more detailed information about the possible `kwargs`.

    Notes

    - To prevent any changes to the original `MassSolution`, a copy of
      the `MassSolution` will be created and used.
    - `i` and `j` represent the number of rows and columns, respectively.

    Parameters
    :   - **mass\_solution** (*MassSolution*) – The `MassSolution` containing the time-dependent solutions
          to be plotted.
        - **observable** (*iterable*) – An iterable containing string identifiers of the `mass` objects
          or the objects themselves that correspond to the keys for the desired
          solutions in the `MassSolution`.
        - **ax** (*matplotlib.axes.Axes**,* *None*) – An `Axes` instance to plot the data on.
          If `None` then the current axes instance is used.
        - **plot\_tile\_placement** (*str*) –

          A string representing the location to place the tiles containing
          phase portrait plots. Must be one of the following:

          > - `"lower"` to place plot tiles on the lower left triangular
          >   section `(i < j)` on the figure tiles.
          > - `"upper"` to place plot tiles on the upper right triangular
          >   section `(i > j)` on the figure tiles.
          > - `all` to place plot tiles on the lower left triangular
          >   section `(i < j)` AND on the upper right triangular section
          >   `(i > j)` on the figure tiles.
        - **additional\_data** (*array\_like**,* *None*) – A matrix of shape `(N, N)` where `N_obs` is the number
          of observables provided, or the number of keys in the
          `MassSolution` if `observable=None`. The value at `(i, j)`
          of the matrix must correspond to the empty tile that the data should
          be displayed on. All other values are ignored. If `None` then no
          data will be displayed and tiles will be left empty.
        - **\*\*kwargs** –
          - time\_vector
          - plot\_function
          - title
          - xlim
          - ylim
          - grid
          - grid\_color
          - grid\_linestyle
          - grid\_linewidth
          - prop\_cycle
          - color
          - linestyle
          - linewidth
          - marker
          - markersize
          - annotate\_time\_points
          - annotate\_time\_points\_color
          - annotate\_time\_points\_marker
          - annotate\_time\_points\_markersize
          - annotate\_time\_points\_legend
          - annotate\_time\_points\_zorder
          - tile\_ticks\_on
          - tile\_xlabel\_fontdict
          - tile\_ylabel\_fontdict
          - data\_tile\_fontsize
          - data\_tile\_color
          - diag\_tile\_color
          - empty\_tile\_color

          See `visualization` documentation for more information on
          optional `kwargs`.

    Returns
    :   **ax** – The `Axes` instance containing the newly
        created plot.

    Return type
    :   matplotlib.axes.Axes

##### Submodules¶

###### `mass.exceptions`¶

This module contains Exceptions specific to `mass` module.

###### Module Contents¶

*exception* `mass.exceptions.``MassSBMLError`[source]¶
:   Bases: `Exception`

    SBML error class.

*exception* `mass.exceptions.``MassSimulationError`[source]¶
:   Bases: `Exception`

    Simulation error class.

*exception* `mass.exceptions.``MassEnsembleError`[source]¶
:   Bases: `Exception`

    Simulation error class.

1
:   Created with sphinx-autoapi

### Frequently Asked Questions (FAQs)¶

Have a question? Try searching the FAQs!

#### How do I install MASSpy?¶

There are several ways to install MASSpy. To use `pip` to install MASSpy from PyPI

```
pip install masspy
```

Check out the Quick Start Guide to learn more about getting started!

#### How do I cite MASSpy?¶

A manuscript is in preparation for publication and will be the proper reference for citing the **MASSpy** software package in the future. In the meantime, feel free to cite the preprint [HZK+20], which can be found at bioRxiv.

#### How do I change the rate expression for a reaction?¶

Use the `MassModel.add_custom_rate()` method.

```
[1]:
```

```
import mass.test

model = mass.test.create_test_model("textbook")
```

```
Using license file /Users/zhaiman/opt/licenses/gurobi.lic
Academic license - for non-commercial use only
```

When metabolites are added to reactions, **MASSpy** will generates rate expressions automatically based on mass action kinetics and the kinetic reversibility given by the `MassReaction.reversible` attribute.

```
[2]:
```

```
print(model.reactions.PGI.rate)
```

```
kf_PGI*(g6p_c(t) - f6p_c(t)/Keq_PGI)
```

If a reaction is associated with a model, a custom rate expression can be set using the `MassModel.add_custom_rate()` method. The `add_custom_rate()` method requires the corresponding reaction object and a string representing the custom rate expression to set. For example, to set a simple Michaelis Menten rate expression with \(V\_{max}\) and \(K\_{m}\) parameters:

```
[3]:
```

```
custom_parameter_dict = {"vmax_PGI": None, "Km_PGI": None}

model.add_custom_rate(
    model.reactions.PGI,
    custom_rate="(vmax_PGI * g6p_c)/(Km_PGI + g6p_c)",
    custom_parameters=custom_parameter_dict)
print(model.reactions.PGI.rate)
```

```
vmax_PGI*g6p_c(t)/(Km_PGI + g6p_c(t))
```

The reaction rate expression is converted from a string to a symbolic expression using the `sympy.sympify()` function. All metabolites and standard reaction parameters (i.e. returned by the `MassReaction.all_parameter_ids`), and boundary conditions are recognized. However, all additional parameters must be set as a custom parameter in the `MassModel.custom_parameters` attribute.

```
[4]:
```

```
print("Recognized Parameters: {!r}".format(model.reactions.PGI.all_parameter_ids))
print("Custom Parameters: {!r}".format(list(custom_parameter_dict)))
```

```
Recognized Parameters: ['kf_PGI', 'Keq_PGI', 'kr_PGI', 'v_PGI']
Custom Parameters: ['vmax_PGI', 'Km_PGI']
```

Additional information about the underlying `sympify()` function can be found here.

### Code Repositories¶

This page contains the links to various code and container repositories associated with MASSpy:

#### MASSpy Repositories¶

- Links to repositories for the MASSpy package: DockerHub | GitHub
- Links to repositories for associated with the MASSpy publication: GitHub

#### COBRA Repositories¶

Links to repositories for COnstraint-Based Reconstruction and Analysis (COBRA) packages compatible with MASSpy:

- **openCOBRA**:
  Homepage
- **COBRApy**:
  Homepage |
  GitHub |
  Documentation
- **Escher**:
  Homepage |
  GitHub |
  Documentation
- **Optlang**:
  GitHub |
  Documentation

#### Scientific computing in MASSpy¶

Several packages for scientific computing are utilized within the MASSpy framework are commonly used in conjuction with MASSpy
to implement a variety of workflows. Many of these packages are seen throughout the documentation, including those used to build the documentation for MASSpy!

As a helpful reference, links to the documentation homepages are provided for a select set of software:

- NumPy
- SciPy library
- Matplotlib
- pandas
- SymPy
- libRoadRunner
- iPython (Jupyter)
- Docker

### Works Cited¶

The following is a list of all references cited throughout the **MASSpy** documentation.

Atk68
:   Daniel E. Atkinson. Energy charge of the adenylate pool as a regulatory parameter. interaction with feedback modifiers. *Biochemistry*, 7(11):4030–4034, 11 1968. URL: https://doi.org/10.1021/bi00851a033, doi:10.1021/bi00851a033.

DZK+16
:   Bin Du, Daniel C. Zielinski, Erol S. Kavvas, Andreas Dräger, Justin Tan, Zhen Zhang, Kayla E. Ruggiero, Garri A. Arzumanyan, and Bernhard O. Palsson. Evaluation of rate law approximations in bottom-up kinetic models of metabolism. *BMC Systems Biology*, 10(1):40, 2016. URL: https://doi.org/10.1186/s12918-016-0283-2, doi:10.1186/s12918-016-0283-2.

ELPH13
:   Ali Ebrahim, Joshua A. Lerman, Bernhard O. Palsson, and Daniel R. Hyduke. Cobrapy: constraints-based reconstruction and analysis for python. *BMC Systems Biology*, 7(1):74, 2013. URL: https://doi.org/10.1186/1752-0509-7-74, doi:10.1186/1752-0509-7-74.

HZK+20
:   Zachary B. Haiman, Daniel C. Zielinski, Yuko Koike, James T. Yurkovich, and Bernhard O. Palsson. Masspy: building, simulating, and visualizing dynamic biological models in python using mass action kinetics. *bioRxiv*, 2020. URL: https://www.biorxiv.org/content/early/2020/07/31/2020.07.31.230334, arXiv:https://www.biorxiv.org/content/early/2020/07/31/2020.07.31.230334.full.pdf, doi:10.1101/2020.07.31.230334.

HDR13
:   Joshua J. Hamilton, Vivek Dwivedi, and Jennifer L. Reed. Quantitative assessment of thermodynamic constraints on the solution space of genome-scale metabolic models. *Biophysical Journal*, 105(2):512 – 522, 2013. URL: http://www.sciencedirect.com/science/article/pii/S0006349513006851, doi:https://doi.org/10.1016/j.bpj.2013.06.011.

HRR78
:   R. Heinrich, S. M. Rapoport, and T. A. Rapoport. Metabolic regulation and mathematical models. *Progress in Biophysics and Molecular Biology*, 32:1–82, 1978. URL: http://www.sciencedirect.com/science/article/pii/0079610778900172, doi:https://doi.org/10.1016/0079-6107(78)90017-2.

JWPB02
:   Neema Jamshidi, Sharon J Wiback, and Bernhard Ø Palsson B. In silico model-driven assessment of the effects of single nucleotide polymorphisms (snps) on human red blood cell metabolism. *Genome research*, 12(11):1687–1692, 11 2002. URL: https://www.ncbi.nlm.nih.gov/pubmed/12421755, doi:10.1101/gr.329302.

JCS17
:   Kristian Jensen, Joao G.r. Cardoso, and Nikolaus Sonnenschein. Optlang: an algebraic modeling language for mathematical optimization. *Journal of Open Source Software*, 2(9):139, 2017. URL: https://doi.org/10.21105/joss.00139, doi:10.21105/joss.00139.

JP89a
:   Abhay Joshi and Bernhard O. Palsson. Metabolic dynamics in the human red cell: part i—a comprehensive kinetic model. *Journal of Theoretical Biology*, 141(4):515–528, 1989. URL: http://www.sciencedirect.com/science/article/pii/S0022519389802334, doi:https://doi.org/10.1016/S0022-5193(89)80233-4.

JP89b
:   Abhay Joshi and Bernhard O. Palsson. Metabolic dynamics in the human red cell: part ii—interactions with the environment. *Journal of Theoretical Biology*, 141(4):529–545, 1989. URL: http://www.sciencedirect.com/science/article/pii/S0022519389802346, doi:https://doi.org/10.1016/S0022-5193(89)80234-6.

JP90
:   Abhay Joshi and Bernhard O. Palsson. Metabolic dynamics in the human red cell. part iii—metabolic reaction rates. *Journal of Theoretical Biology*, 142(1):41–68, 1990. URL: http://www.sciencedirect.com/science/article/pii/S0022519305800128, doi:https://doi.org/10.1016/S0022-5193(05)80012-8.

KS98
:   David E. Kaufman and Robert L. Smith. Direction choice for accelerated convergence in hit-and-run sampling. *Operations Research*, 46(1):84–95, 1998. URL: https://pubsonline.informs.org/doi/abs/10.1287/opre.46.1.84, arXiv:https://pubsonline.informs.org/doi/pdf/10.1287/opre.46.1.84, doi:10.1287/opre.46.1.84.

KDragerE+15
:   Zachary A. King, Andreas Dräger, Ali Ebrahim, Nikolaus Sonnenschein, Nathan E. Lewis, and Bernhard O. Palsson. Escher: a web application for building, sharing, and embedding data-rich visualizations of biological pathways. *PLOS Computational Biology*, 11(8):e1004321–, 08 2015. URL: https://doi.org/10.1371/journal.pcbi.1004321.

KummelPH06
:   Anne Kümmel, Sven Panke, and Matthias Heinemann. Putative regulatory sites unraveled by network-embedded thermodynamic analysis of metabolome data. *Molecular systems biology*, 2:2006.0034–2006.0034, 2006. URL: https://www.ncbi.nlm.nih.gov/pubmed/16788595, doi:10.1038/msb4100074.

MHM14
:   Wout Megchelenbrink, Martijn Huynen, and Elena Marchiori. Optgpsampler: an improved tool for uniformly sampling the solution-space of genome-scale metabolic networks. *PLOS ONE*, 9(2):1–8, 02 2014. URL: https://doi.org/10.1371/journal.pone.0086587, doi:10.1371/journal.pone.0086587.

MK99
:   P J Mulquiney and P W Kuchel. Model of 2,3-bisphosphoglycerate metabolism in the human erythrocyte based on detailed enzyme kinetic equations: equations and parameter refinement. *The Biochemical journal*, 342 Pt 3(Pt 3):581–596, 09 1999. URL: https://www.ncbi.nlm.nih.gov/pubmed/10477269.

Pal11
:   Bernhard Ø. Palsson. *Systems Biology: Simulation of Dynamic Network States*. Cambridge University Press, 2011. doi:10.1017/CBO9780511736179.

SQF+11
:   Jan Schellenberger, Richard Que, Ronan M T Fleming, Ines Thiele, Jeffrey D Orth, Adam M Feist, Daniel C Zielinski, Aarash Bordbar, Nathan E Lewis, Sorena Rahmanian, Joseph Kang, Daniel R Hyduke, and Bernhard Ø Palsson. Quantitative prediction of cellular metabolism with constraint-based models: the cobra toolbox v2.0. *Nature Protocols*, 6(9):1290–1307, 2011. URL: https://doi.org/10.1038/nprot.2011.308, doi:10.1038/nprot.2011.308.

YAHP18
:   James T. Yurkovich, Miguel A. Alcantar, Zachary B. Haiman, and Bernhard O. Palsson. Network-level allosteric effects are elucidated by detailing how ligand-binding events modulate utilization of catalytic potentials. *PLOS Computational Biology*, 14(8):e1006356–, 08 2018. URL: https://doi.org/10.1371/journal.pcbi.1006356.

# Indices and tables¶

- Index
- Module Index
- Search Page

---

© Copyright 2019, Z. Haiman
Revision `3ddbbd0c`.

Built with Sphinx using a theme provided by Read the Docs.

Read the Docs
v: v0.1.1

Versions
:   latest
:   stable
:   v0.1.1
:   docs

Downloads
:   html

On Read the Docs
:   Project Home
:   Builds

---

Free document hosting provided by Read the Docs.
