# Supplementary figures and images for: MASSpy: Building, simulating, and visualizing dynamic biological models in Python using mass action kinetics (part 3 of 3)
Source: PLoS Comput Biol. 2021 Jan 28;17(1):e1008208. doi: 10.1371/journal.pcbi.1008208 (PMC7872247; doi:10.1371/journal.pcbi.1008208)

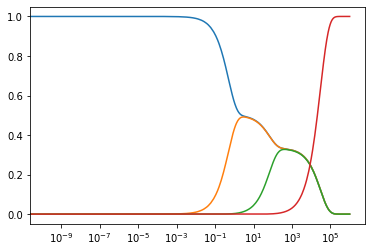

Supplement: S2 File — The latest version of the MASSpy documentation can be found at https://masspy.readthedocs.io. (ZIP) [file pcbi.1008208.s004.zip › masspy-v0.1.1/_images/education_sb2_chapters_sb2_chapter3_87_1.png]

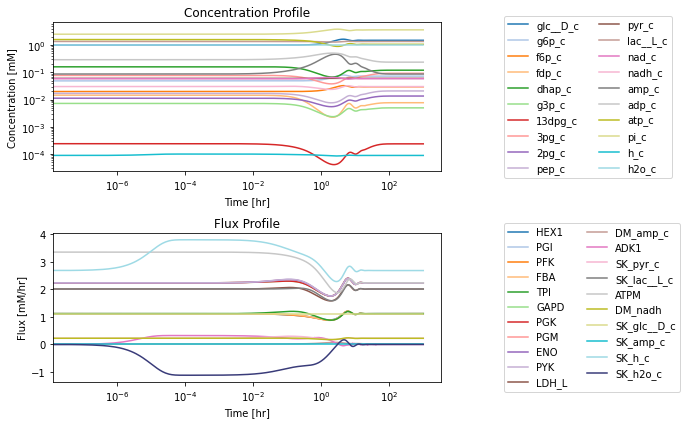

Supplement: S2 File — The latest version of the MASSpy documentation can be found at https://masspy.readthedocs.io. (ZIP) [file pcbi.1008208.s004.zip › masspy-v0.1.1/_images/education_sb2_chapters_sb2_chapter10_49_0.png]

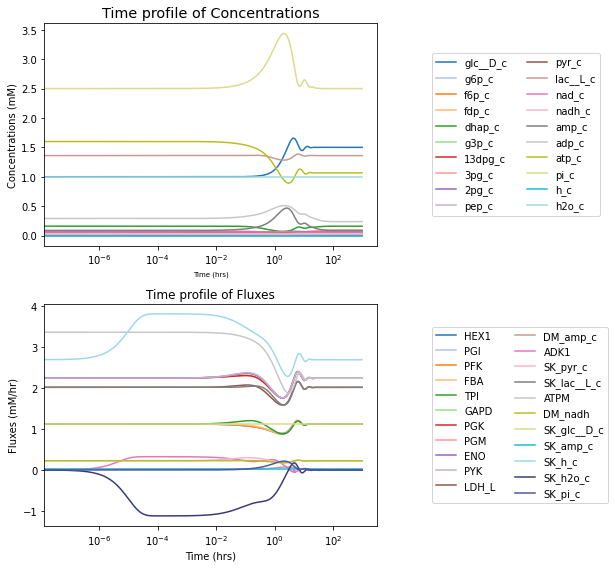

Supplement: S2 File — The latest version of the MASSpy documentation can be found at https://masspy.readthedocs.io. (ZIP) [file pcbi.1008208.s004.zip › masspy-v0.1.1/_images/tutorials_plot_visualization_21_0.png]

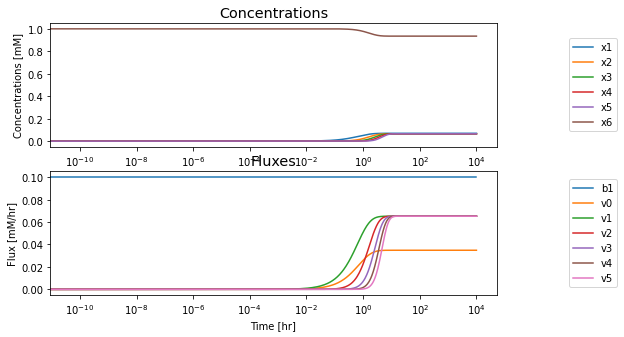

Supplement: S2 File — The latest version of the MASSpy documentation can be found at https://masspy.readthedocs.io. (ZIP) [file pcbi.1008208.s004.zip › masspy-v0.1.1/_images/education_sb2_chapters_sb2_chapter9_15_1.png]

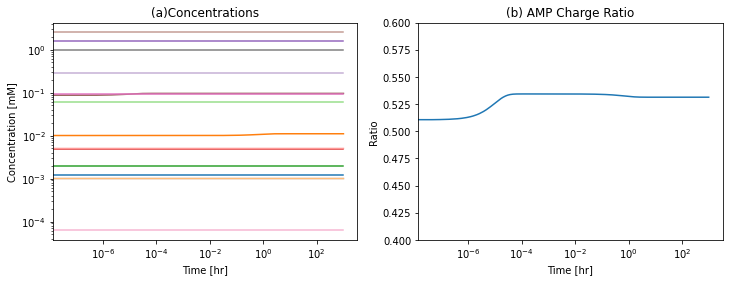

Supplement: S2 File — The latest version of the MASSpy documentation can be found at https://masspy.readthedocs.io. (ZIP) [file pcbi.1008208.s004.zip › masspy-v0.1.1/_images/education_sb2_chapters_sb2_chapter12_36_0.png]

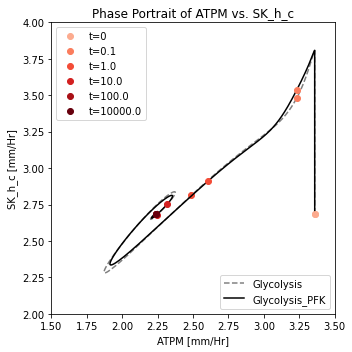

Supplement: S2 File — The latest version of the MASSpy documentation can be found at https://masspy.readthedocs.io. (ZIP) [file pcbi.1008208.s004.zip › masspy-v0.1.1/_images/education_sb2_chapters_sb2_chapter14_46_0.png]

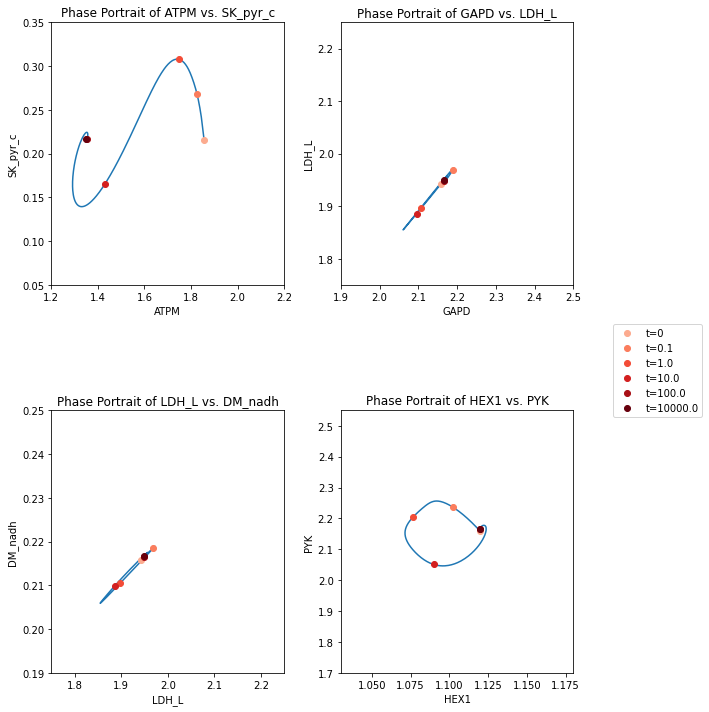

Supplement: S2 File — The latest version of the MASSpy documentation can be found at https://masspy.readthedocs.io. (ZIP) [file pcbi.1008208.s004.zip › masspy-v0.1.1/_images/education_sb2_chapters_sb2_chapter14_74_0.png]

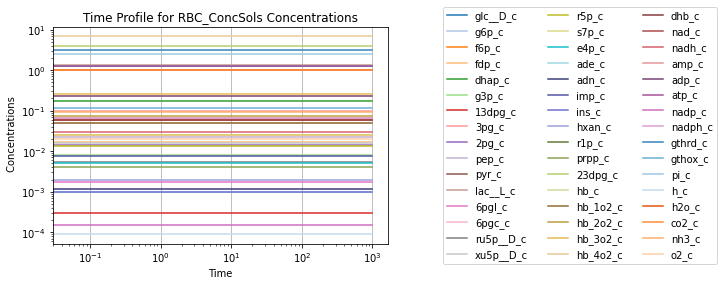

Supplement: S2 File — The latest version of the MASSpy documentation can be found at https://masspy.readthedocs.io. (ZIP) [file pcbi.1008208.s004.zip › masspy-v0.1.1/_images/education_sb2_chapters_sb2_chapter13_68_0.png]

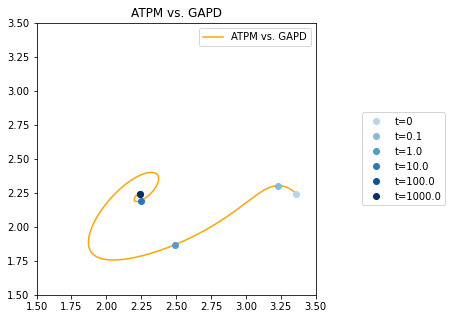

Supplement: S2 File — The latest version of the MASSpy documentation can be found at https://masspy.readthedocs.io. (ZIP) [file pcbi.1008208.s004.zip › masspy-v0.1.1/_images/tutorials_plot_visualization_40_1.png]

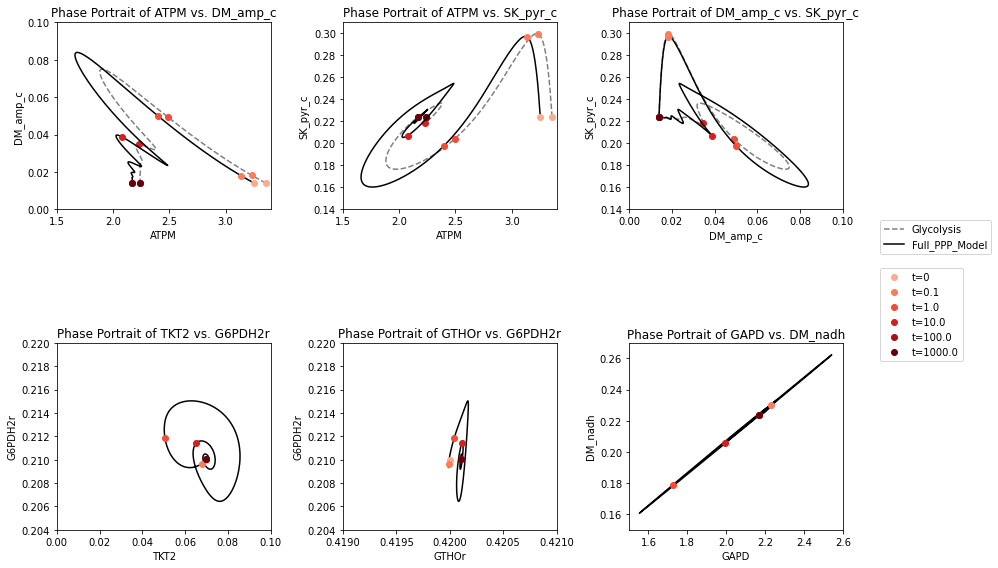

Supplement: S2 File — The latest version of the MASSpy documentation can be found at https://masspy.readthedocs.io. (ZIP) [file pcbi.1008208.s004.zip › masspy-v0.1.1/_images/education_sb2_chapters_sb2_chapter11_80_0.png]

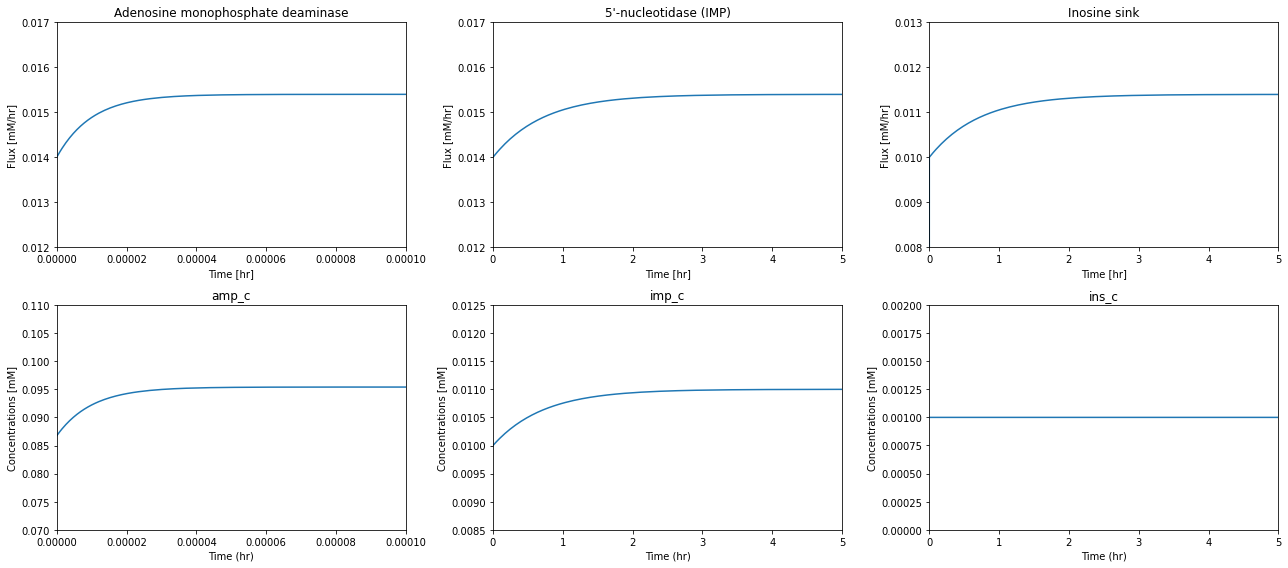

Supplement: S2 File — The latest version of the MASSpy documentation can be found at https://masspy.readthedocs.io. (ZIP) [file pcbi.1008208.s004.zip › masspy-v0.1.1/_images/education_sb2_chapters_sb2_chapter12_38_0.png]

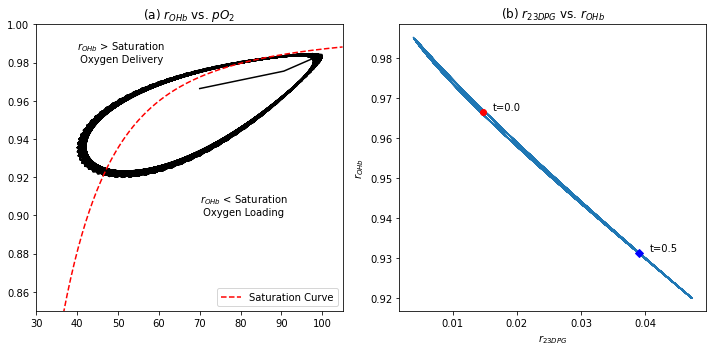

Supplement: S2 File — The latest version of the MASSpy documentation can be found at https://masspy.readthedocs.io. (ZIP) [file pcbi.1008208.s004.zip › masspy-v0.1.1/_images/education_sb2_chapters_sb2_chapter13_44_0.png]

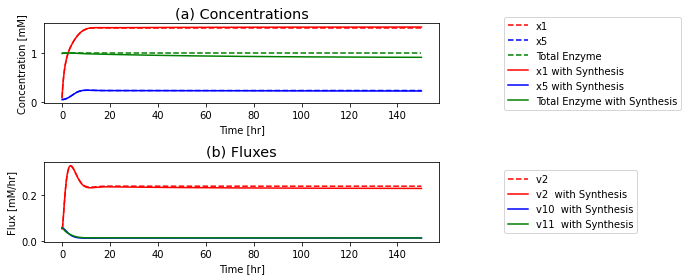

Supplement: S2 File — The latest version of the MASSpy documentation can be found at https://masspy.readthedocs.io. (ZIP) [file pcbi.1008208.s004.zip › masspy-v0.1.1/_images/education_sb2_chapters_sb2_chapter9_142_0.png]

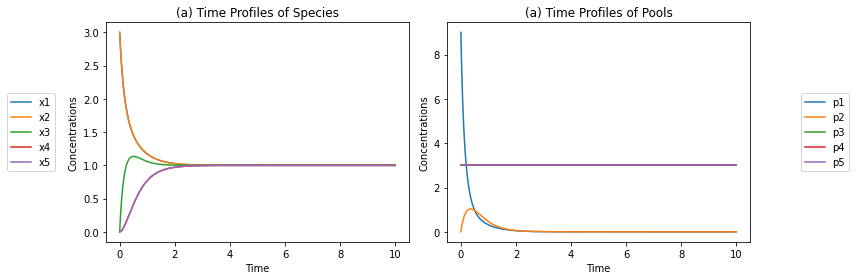

Supplement: S2 File — The latest version of the MASSpy documentation can be found at https://masspy.readthedocs.io. (ZIP) [file pcbi.1008208.s004.zip › masspy-v0.1.1/_images/education_sb2_chapters_sb2_chapter4_26_0.png]

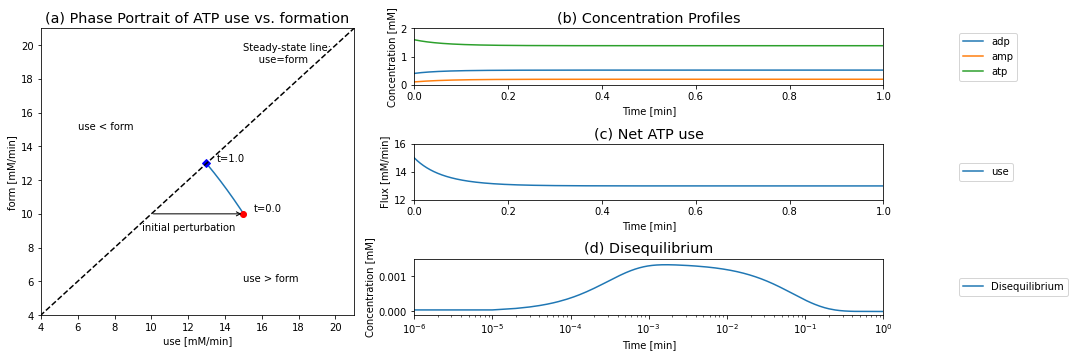

Supplement: S2 File — The latest version of the MASSpy documentation can be found at https://masspy.readthedocs.io. (ZIP) [file pcbi.1008208.s004.zip › masspy-v0.1.1/_images/education_sb2_chapters_sb2_chapter8_31_0.png]

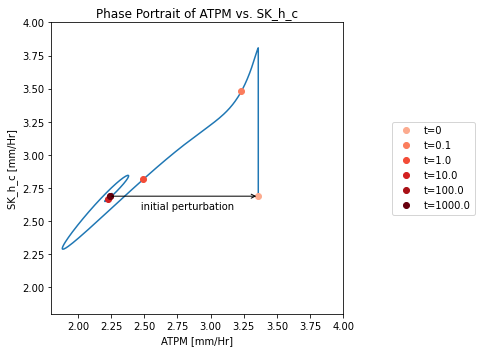

Supplement: S2 File — The latest version of the MASSpy documentation can be found at https://masspy.readthedocs.io. (ZIP) [file pcbi.1008208.s004.zip › masspy-v0.1.1/_images/education_sb2_chapters_sb2_chapter10_60_0.png]

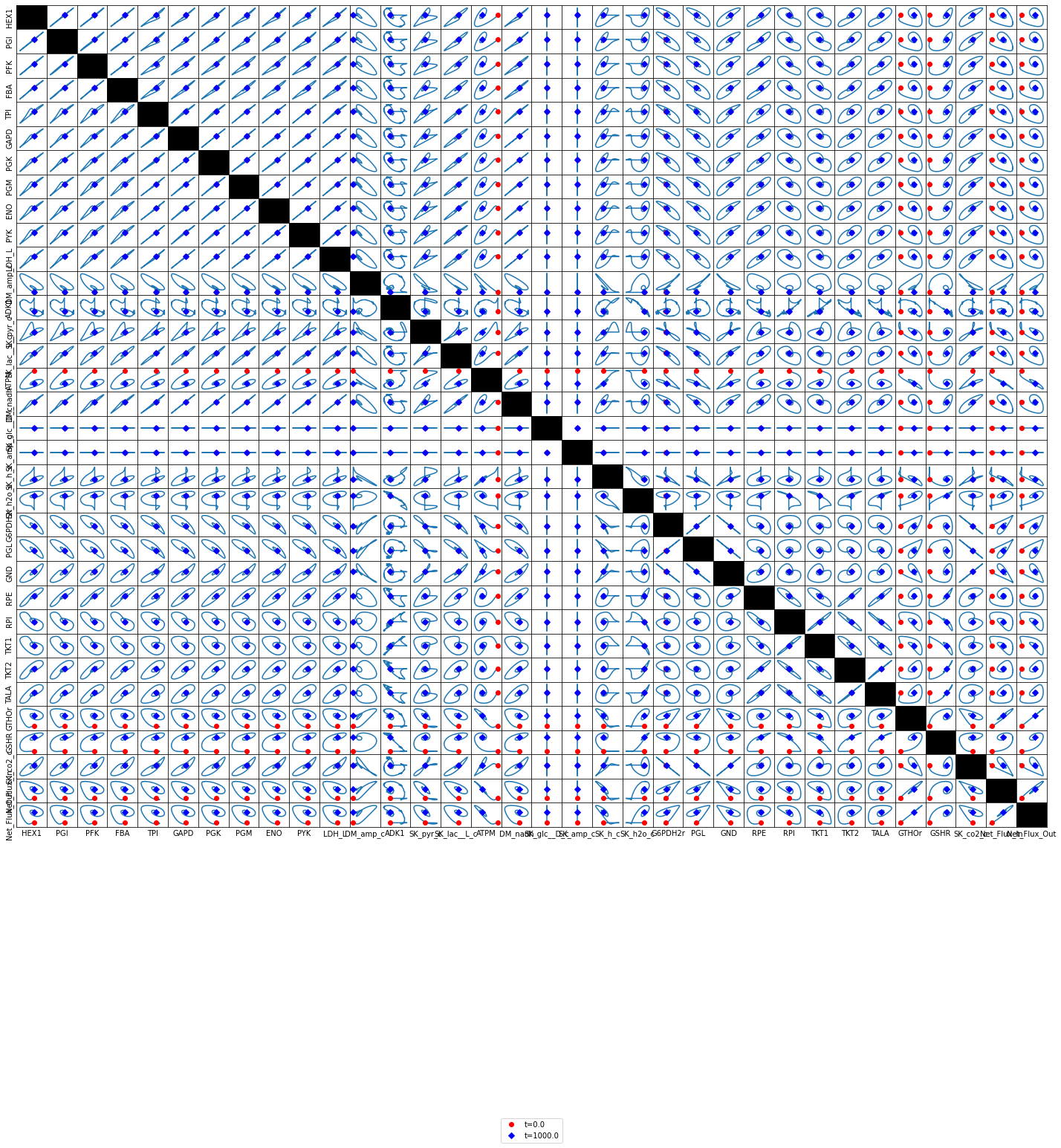

Supplement: S2 File — The latest version of the MASSpy documentation can be found at https://masspy.readthedocs.io. (ZIP) [file pcbi.1008208.s004.zip › masspy-v0.1.1/_images/education_sb2_chapters_sb2_chapter11_92_0.png]

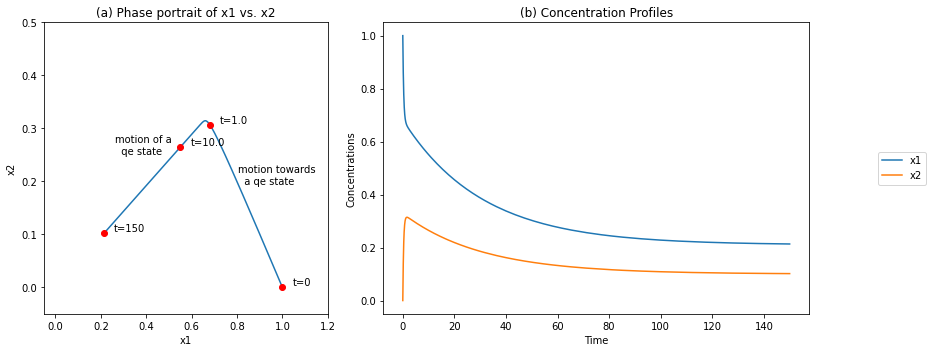

Supplement: S2 File — The latest version of the MASSpy documentation can be found at https://masspy.readthedocs.io. (ZIP) [file pcbi.1008208.s004.zip › masspy-v0.1.1/_images/education_sb2_chapters_sb2_chapter6_8_0.png]

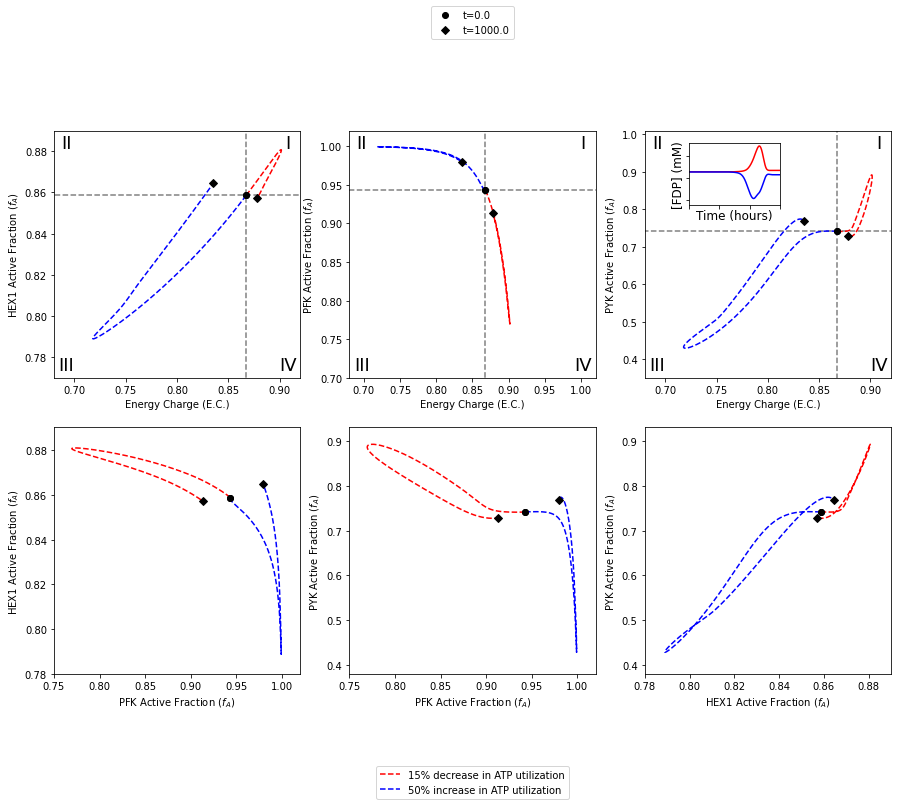

Supplement: S2 File — The latest version of the MASSpy documentation can be found at https://masspy.readthedocs.io. (ZIP) [file pcbi.1008208.s004.zip › masspy-v0.1.1/_images/gallery_visualization_catalytic_potential_visualizations_48_0.png]

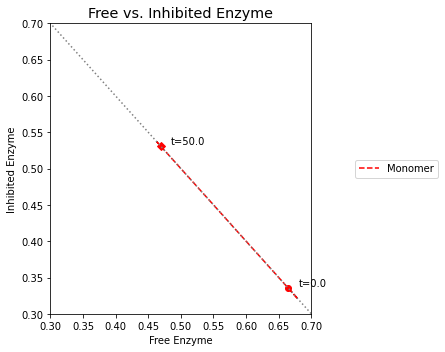

Supplement: S2 File — The latest version of the MASSpy documentation can be found at https://masspy.readthedocs.io. (ZIP) [file pcbi.1008208.s004.zip › masspy-v0.1.1/_images/education_sb2_chapters_sb2_chapter9_54_0.png]

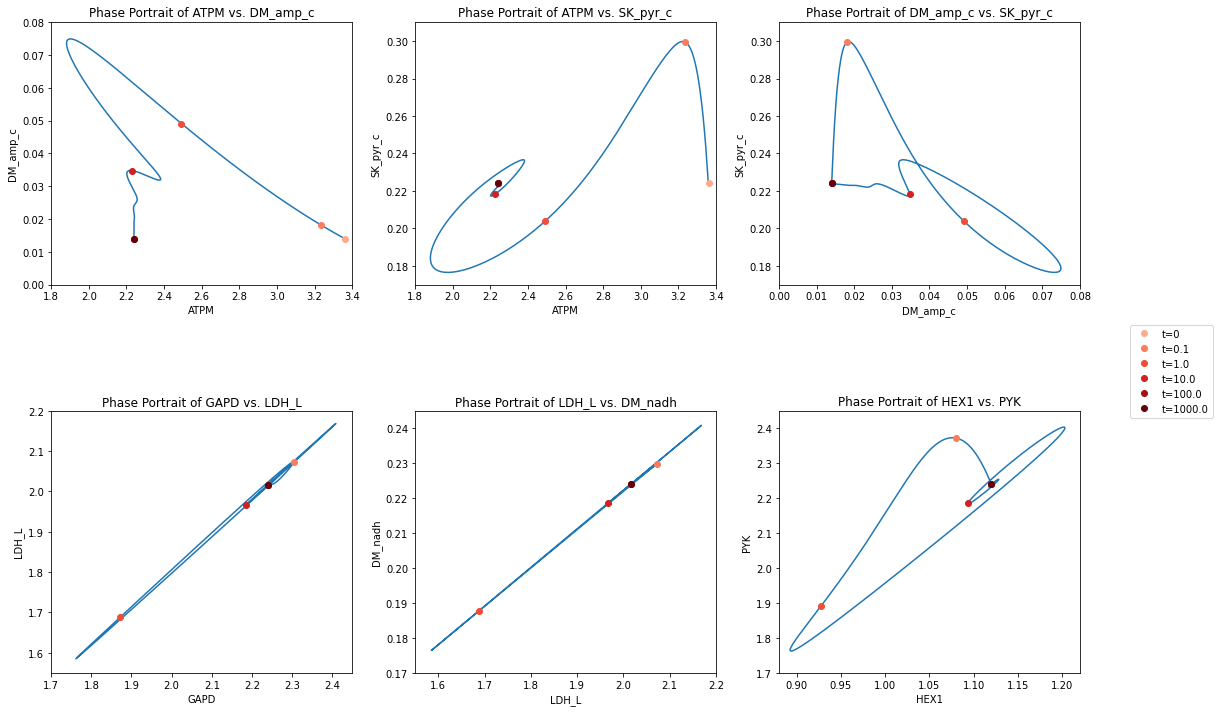

Supplement: S2 File — The latest version of the MASSpy documentation can be found at https://masspy.readthedocs.io. (ZIP) [file pcbi.1008208.s004.zip › masspy-v0.1.1/_images/education_sb2_chapters_sb2_chapter10_72_0.png]

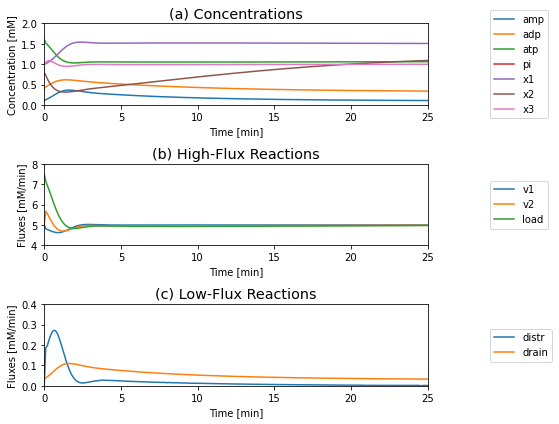

Supplement: S2 File — The latest version of the MASSpy documentation can be found at https://masspy.readthedocs.io. (ZIP) [file pcbi.1008208.s004.zip › masspy-v0.1.1/_images/education_sb2_chapters_sb2_chapter8_81_0.png]

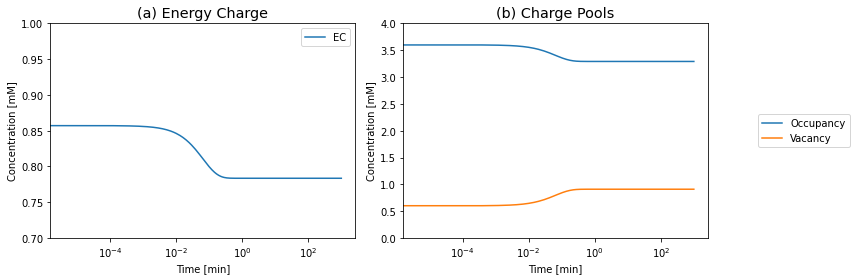

Supplement: S2 File — The latest version of the MASSpy documentation can be found at https://masspy.readthedocs.io. (ZIP) [file pcbi.1008208.s004.zip › masspy-v0.1.1/_images/education_sb2_chapters_sb2_chapter8_34_0.png]

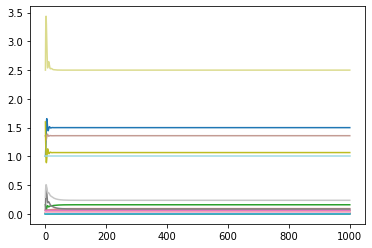

Supplement: S2 File — The latest version of the MASSpy documentation can be found at https://masspy.readthedocs.io. (ZIP) [file pcbi.1008208.s004.zip › masspy-v0.1.1/_images/tutorials_plot_visualization_11_1.png]

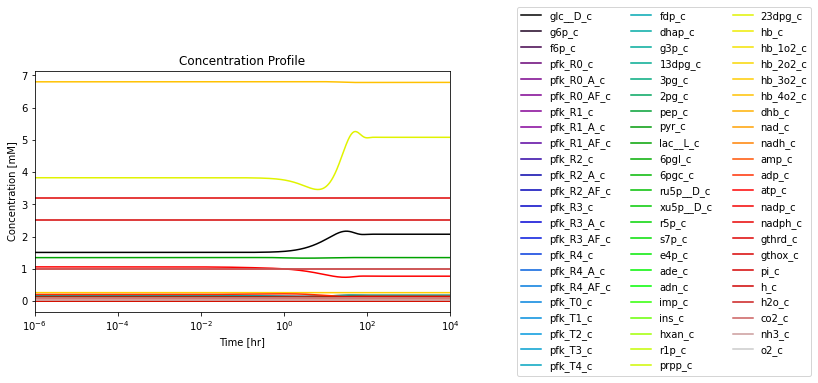

Supplement: S2 File — The latest version of the MASSpy documentation can be found at https://masspy.readthedocs.io. (ZIP) [file pcbi.1008208.s004.zip › masspy-v0.1.1/_images/education_sb2_chapters_sb2_chapter14_66_0.png]

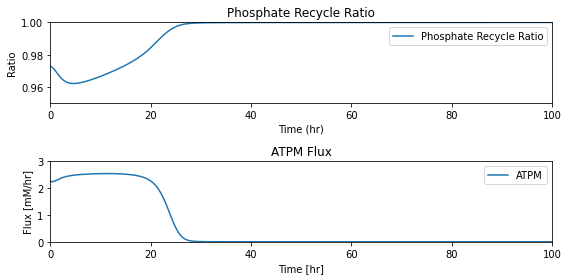

Supplement: S2 File — The latest version of the MASSpy documentation can be found at https://masspy.readthedocs.io. (ZIP) [file pcbi.1008208.s004.zip › masspy-v0.1.1/_images/education_sb2_chapters_sb2_chapter10_93_0.png]

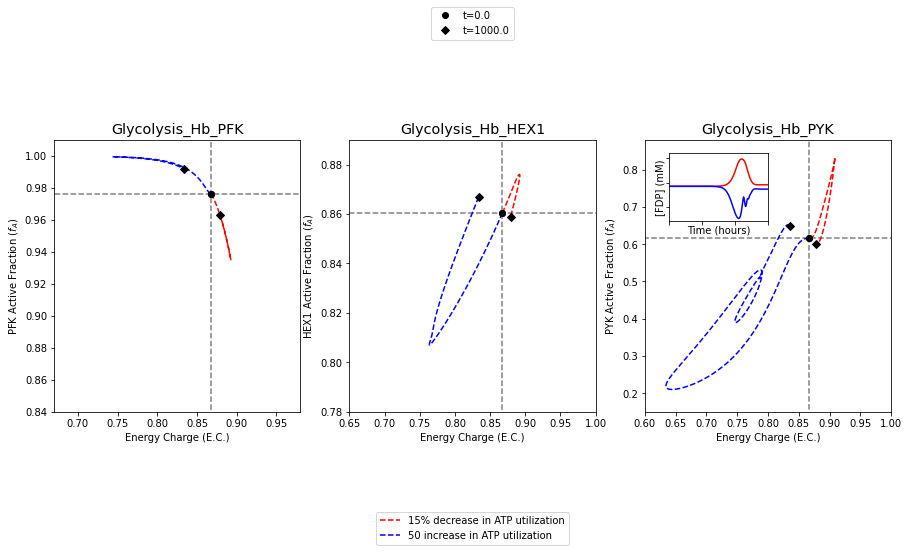

Supplement: S2 File — The latest version of the MASSpy documentation can be found at https://masspy.readthedocs.io. (ZIP) [file pcbi.1008208.s004.zip › masspy-v0.1.1/_images/gallery_visualization_catalytic_potential_visualizations_4_1.png]

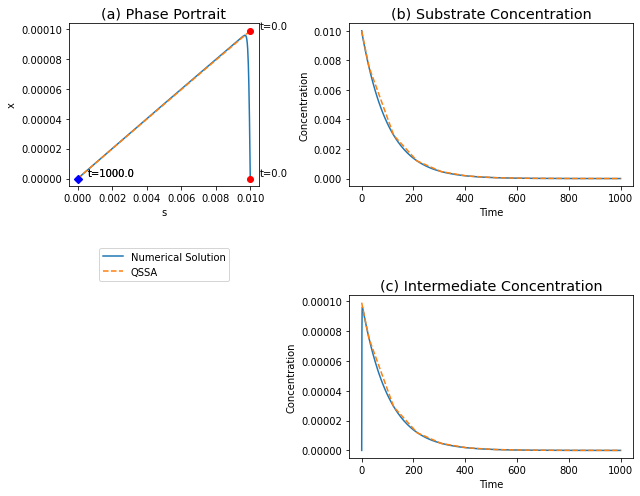

Supplement: S2 File — The latest version of the MASSpy documentation can be found at https://masspy.readthedocs.io. (ZIP) [file pcbi.1008208.s004.zip › masspy-v0.1.1/_images/education_sb2_chapters_sb2_chapter5_14_0.png]

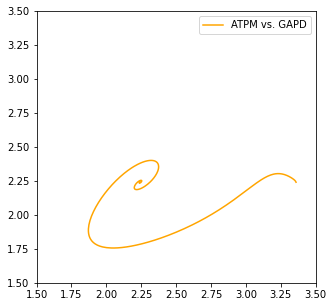

Supplement: S2 File — The latest version of the MASSpy documentation can be found at https://masspy.readthedocs.io. (ZIP) [file pcbi.1008208.s004.zip › masspy-v0.1.1/_images/tutorials_plot_visualization_38_1.png]

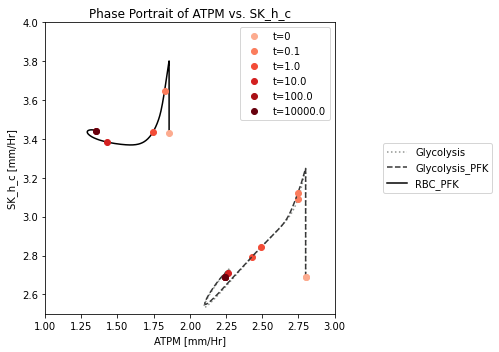

Supplement: S2 File — The latest version of the MASSpy documentation can be found at https://masspy.readthedocs.io. (ZIP) [file pcbi.1008208.s004.zip › masspy-v0.1.1/_images/education_sb2_chapters_sb2_chapter14_70_0.png]

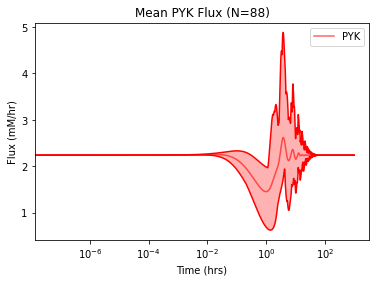

Supplement: S2 File — The latest version of the MASSpy documentation can be found at https://masspy.readthedocs.io. (ZIP) [file pcbi.1008208.s004.zip › masspy-v0.1.1/_images/tutorials_ensemble_modeling_44_1.png]

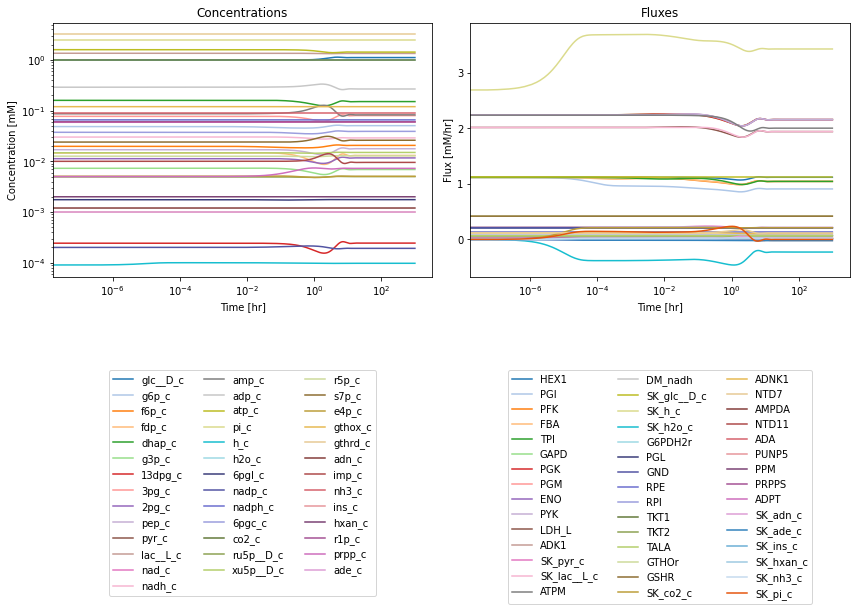

Supplement: S2 File — The latest version of the MASSpy documentation can be found at https://masspy.readthedocs.io. (ZIP) [file pcbi.1008208.s004.zip › masspy-v0.1.1/_images/education_sb2_chapters_sb2_chapter12_50_0.png]

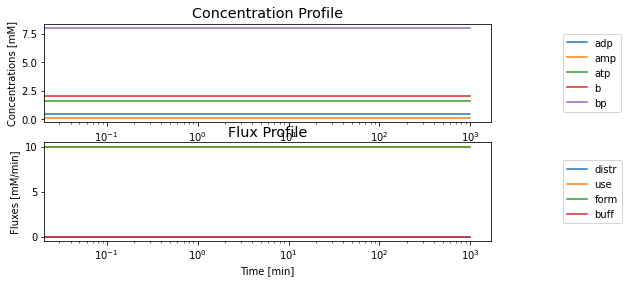

Supplement: S2 File — The latest version of the MASSpy documentation can be found at https://masspy.readthedocs.io. (ZIP) [file pcbi.1008208.s004.zip › masspy-v0.1.1/_images/education_sb2_chapters_sb2_chapter8_53_1.png]
