# Supplementary material for: MASSpy: Building, simulating, and visualizing dynamic biological models in Python using mass action kinetics
Source: PLoS Comput Biol. 2021 Jan 28;17(1):e1008208. doi: 10.1371/journal.pcbi.1008208 (PMC7872247; doi:10.1371/journal.pcbi.1008208)
Supplement: S3 File — All files necessary to reproduce the results presented in the validation, ensemble modeling feature demonstration, and case study. Alternatively, these files can be found at https://github.com/SBRG/MASSpy-publication. (ZIP) [file pcbi.1008208.s005.zip › MASSpy-publication-master/case-study/data/analysis_figures/medium_direct_comparison_075_025/Gibbs_Glucose_Pyruvate_all_enzymes.pdf]

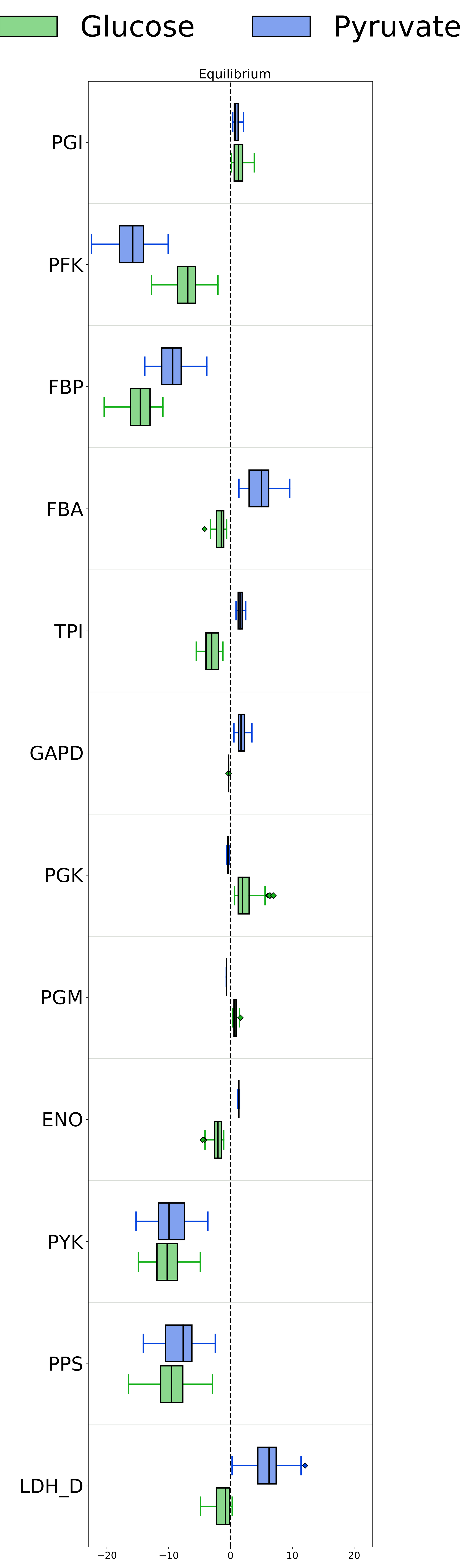

Glucose
Pyruvate
Equilibrium
PGI
PFK
FBP
FBA
TPI
GAPD
PGK
PGM
ENO
PYK
PPS
LDH_D
−20
−10
0
10
20
