# Supplementary material for: MASSpy: Building, simulating, and visualizing dynamic biological models in Python using mass action kinetics
Source: PLoS Comput Biol. 2021 Jan 28;17(1):e1008208. doi: 10.1371/journal.pcbi.1008208 (PMC7872247; doi:10.1371/journal.pcbi.1008208)
Supplement: S3 File — All files necessary to reproduce the results presented in the validation, ensemble modeling feature demonstration, and case study. Alternatively, these files can be found at https://github.com/SBRG/MASSpy-publication. (ZIP) [file pcbi.1008208.s005.zip › MASSpy-publication-master/ensemble-modeling/data/figures/MASSpy-Pub-Fig3_PanelD2.pdf]

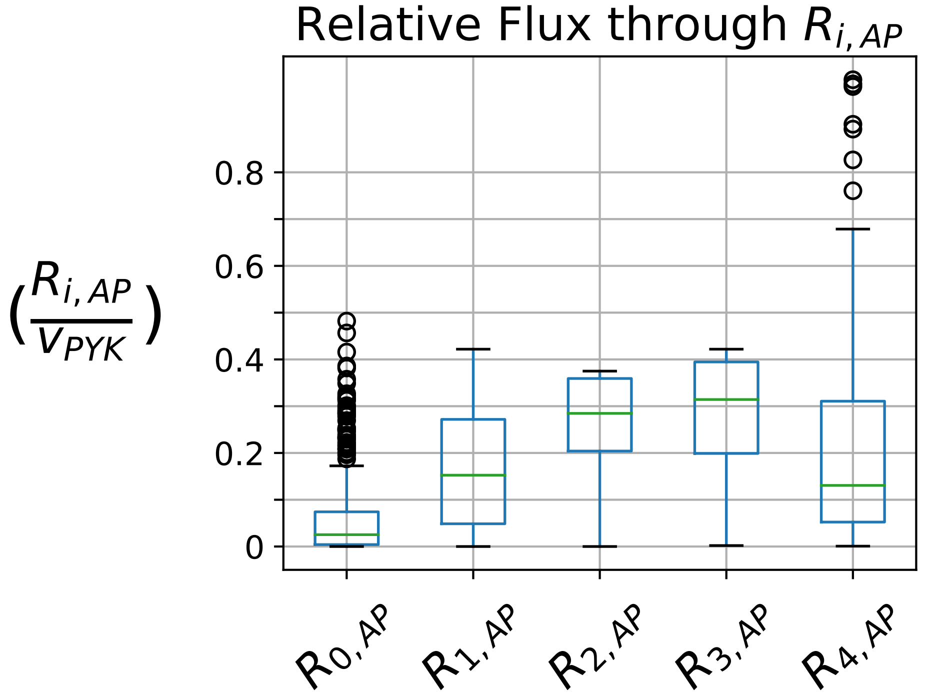

Relative Flux through $R_{i,AP}$
$(\frac{R_{i,AP}}{v_{PYK}})$
0
0.2
0.4
0.6
0.8
$R_{0,AP}$
$R_{1,AP}$
$R_{2,AP}$
$R_{3,AP}$
$R_{4,AP}$
