# Supplementary material for: MASSpy: Building, simulating, and visualizing dynamic biological models in Python using mass action kinetics
Source: PLoS Comput Biol. 2021 Jan 28;17(1):e1008208. doi: 10.1371/journal.pcbi.1008208 (PMC7872247; doi:10.1371/journal.pcbi.1008208)
Supplement: S3 File — All files necessary to reproduce the results presented in the validation, ensemble modeling feature demonstration, and case study. Alternatively, these files can be found at https://github.com/SBRG/MASSpy-publication. (ZIP) [file pcbi.1008208.s005.zip › MASSpy-publication-master/ensemble-modeling/data/figures/ensemble_simulation_results_three_panes.pdf]

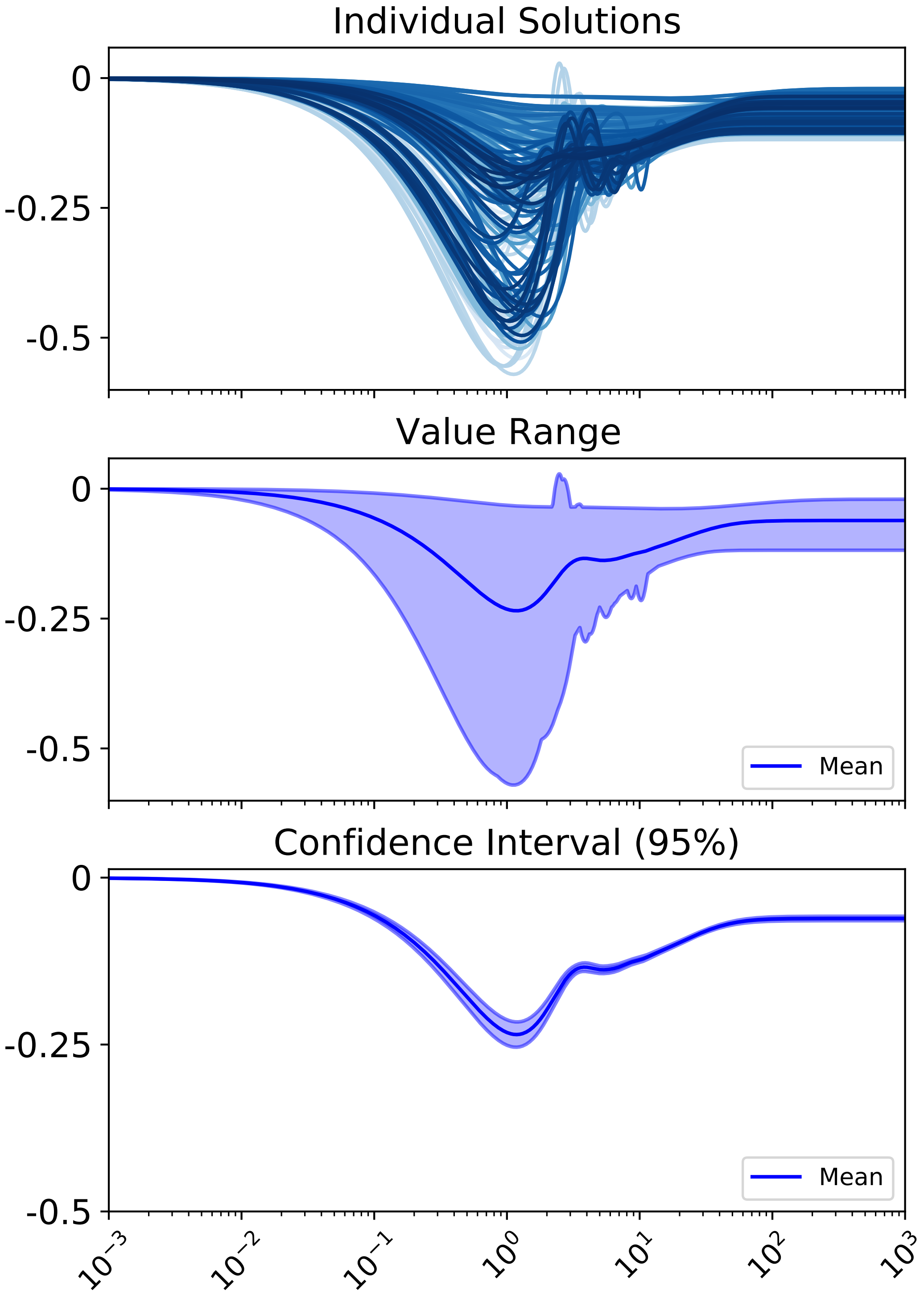

Individual Solutions
0
-0.25
-0.5
Value Range
Mean
Confidence Interval (95%)
10^{-3}
10^{-2}
10^{-1}
10^{0}
10^{1}
10^{2}
10^{3}
