# Supplementary material for: MASSpy: Building, simulating, and visualizing dynamic biological models in Python using mass action kinetics
Source: PLoS Comput Biol. 2021 Jan 28;17(1):e1008208. doi: 10.1371/journal.pcbi.1008208 (PMC7872247; doi:10.1371/journal.pcbi.1008208)
Supplement: S3 File — All files necessary to reproduce the results presented in the validation, ensemble modeling feature demonstration, and case study. Alternatively, these files can be found at https://github.com/SBRG/MASSpy-publication. (ZIP) [file pcbi.1008208.s005.zip › MASSpy-publication-master/validation/data/figures/MASSpy-Pub-Fig2_PanelB.pdf]

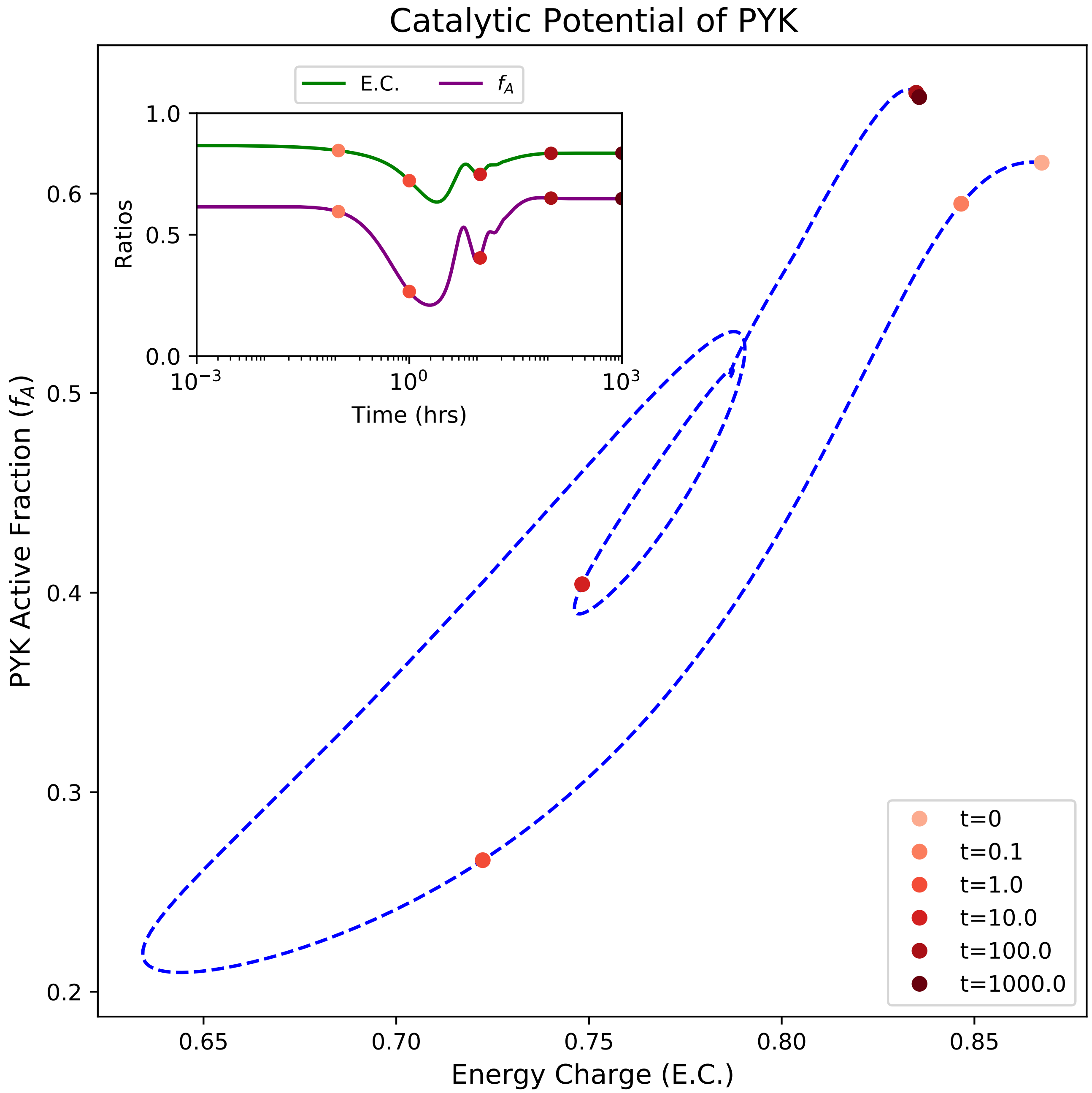

Catalytic Potential of PYK
PYK Active Fraction (f_A)
Energy Charge (E.C.)
0.2
0.3
0.4
0.5
0.6
0.65
0.70
0.75
0.80
0.85
E.C.
f_A
Ratios
0.0
0.5
1.0
10^{-3}
10^{0}
10^{3}
Time (hrs)
t=0
t=0.1
t=1.0
t=10.0
t=100.0
t=1000.0
