# Supplementary material for: MASSpy: Building, simulating, and visualizing dynamic biological models in Python using mass action kinetics
Source: PLoS Comput Biol. 2021 Jan 28;17(1):e1008208. doi: 10.1371/journal.pcbi.1008208 (PMC7872247; doi:10.1371/journal.pcbi.1008208)
Supplement: S3 File — All files necessary to reproduce the results presented in the validation, ensemble modeling feature demonstration, and case study. Alternatively, these files can be found at https://github.com/SBRG/MASSpy-publication. (ZIP) [file pcbi.1008208.s005.zip › MASSpy-publication-master/validation/data/figures/MASSpy-Pub-Fig2_PanelC.pdf]

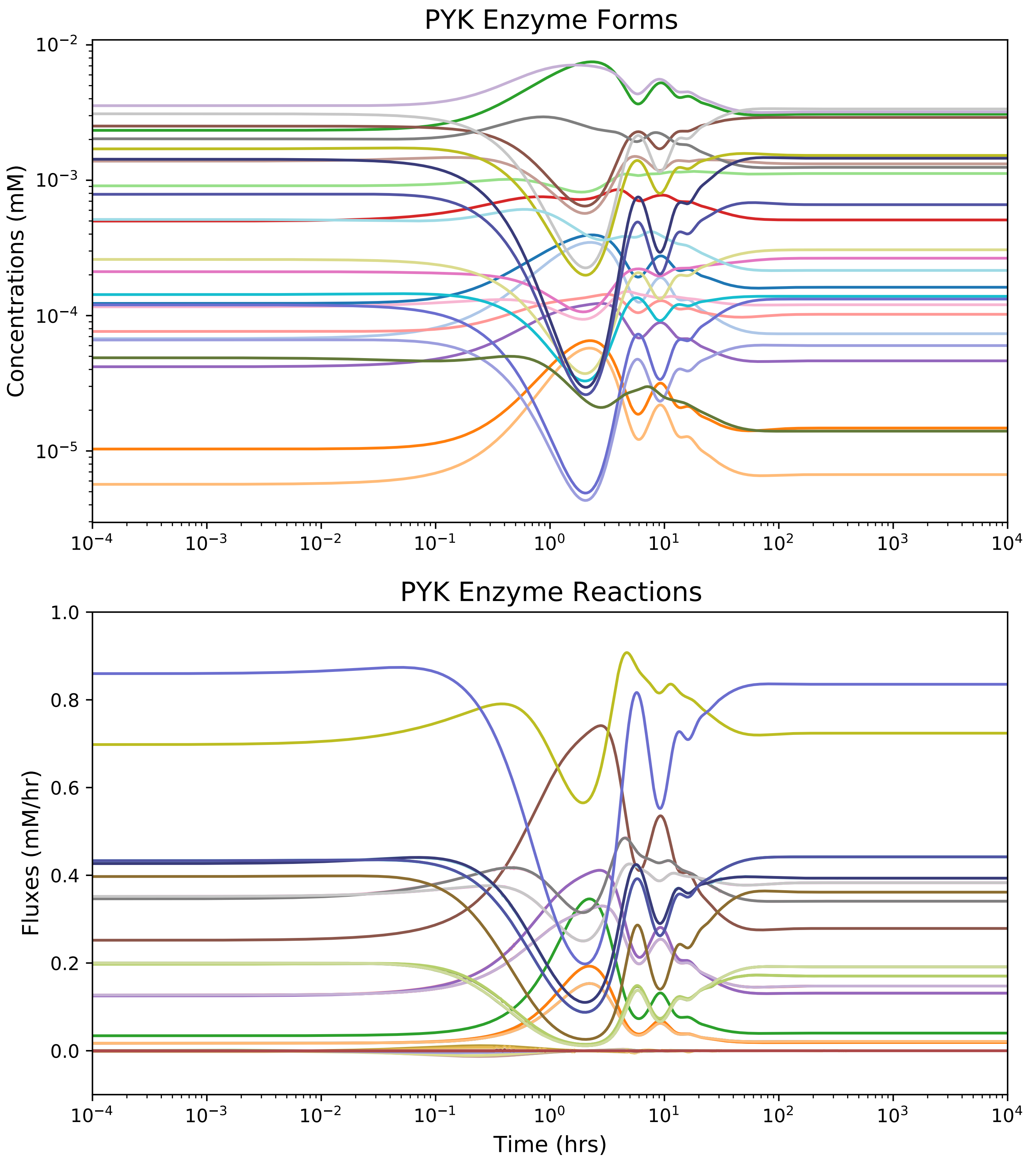
PYK Enzyme Forms
Concentrations (mM)
10^{-2}
10^{-3}
10^{-4}
10^{-5}
10^{-4} 10^{-3} 10^{-2} 10^{-1} 10^{0} 10^{1} 10^{2} 10^{3} 10^{4}
PYK Enzyme Reactions
Fluxes (mM/hr)
1.0
0.8
0.6
0.4
0.2
0.0
10^{-4} 10^{-3} 10^{-2} 10^{-1} 10^{0} 10^{1} 10^{2} 10^{3} 10^{4}
Time (hrs)
