# Supplementary material for: MASSpy: Building, simulating, and visualizing dynamic biological models in Python using mass action kinetics
Source: PLoS Comput Biol. 2021 Jan 28;17(1):e1008208. doi: 10.1371/journal.pcbi.1008208 (PMC7872247; doi:10.1371/journal.pcbi.1008208)
Supplement: S3 File — All files necessary to reproduce the results presented in the validation, ensemble modeling feature demonstration, and case study. Alternatively, these files can be found at https://github.com/SBRG/MASSpy-publication. (ZIP) [file pcbi.1008208.s005.zip › MASSpy-publication-master/validation/data/figures/Reproduced-Fig2.pdf]

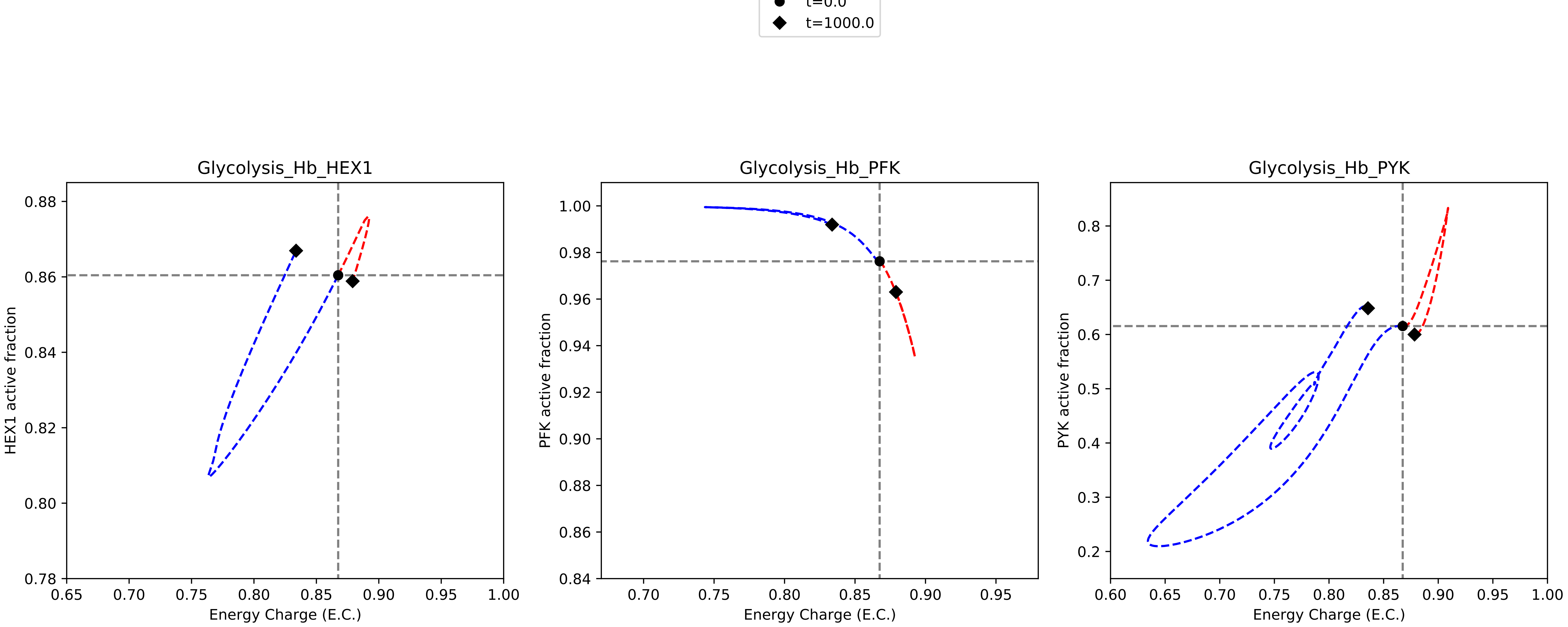

t=0.0
t=1000.0
Glycolysis_Hb_HEX1
HEX1 active fraction
Energy Charge (E.C.)
0.78
0.80
0.82
0.84
0.86
0.88
0.65
0.70
0.75
0.80
0.85
0.90
0.95
1.00
Glycolysis_Hb_PFK
PFK active fraction
Energy Charge (E.C.)
0.84
0.86
0.88
0.90
0.92
0.94
0.96
0.98
1.00
0.70
0.75
0.80
0.85
0.90
0.95
Glycolysis_Hb_PYK
PYK active fraction
Energy Charge (E.C.)
0.2
0.3
0.4
0.5
0.6
0.7
0.8
0.60
0.65
0.70
0.75
0.80
0.85
0.90
0.95
1.00
