# Supplementary material for: MASSpy: Building, simulating, and visualizing dynamic biological models in Python using mass action kinetics
Source: PLoS Comput Biol. 2021 Jan 28;17(1):e1008208. doi: 10.1371/journal.pcbi.1008208 (PMC7872247; doi:10.1371/journal.pcbi.1008208)
Supplement: S3 File — All files necessary to reproduce the results presented in the validation, ensemble modeling feature demonstration, and case study. Alternatively, these files can be found at https://github.com/SBRG/MASSpy-publication. (ZIP) [file pcbi.1008208.s005.zip › MASSpy-publication-master/validation/data/figures/Reproduced-Fig4.pdf]

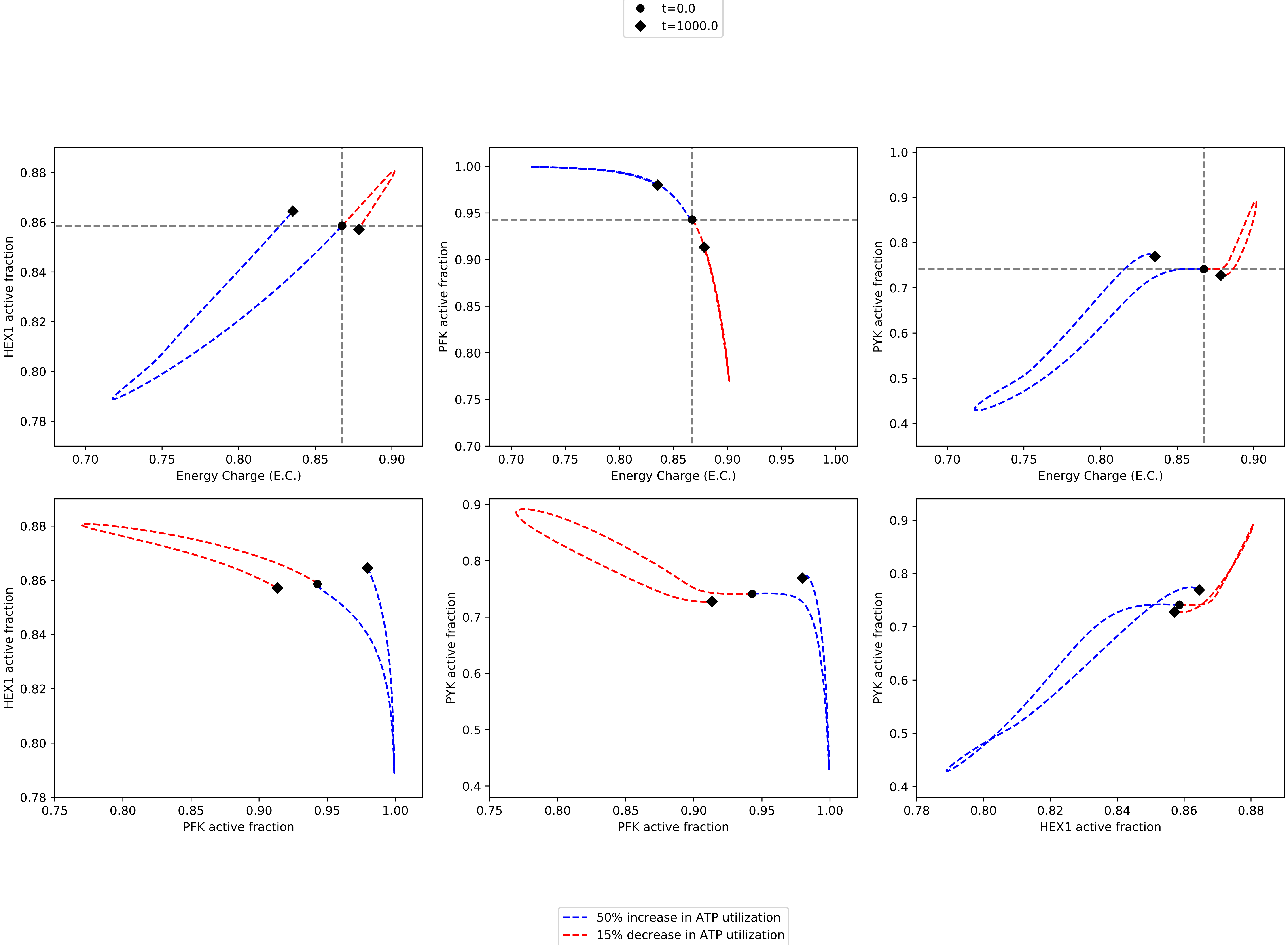

t=0.0
t=1000.0
HEX1 active fraction
PFK active fraction
PYK active fraction
Energy Charge (E.C.)
0.78
0.80
0.82
0.84
0.86
0.88
0.70
0.75
0.80
0.85
0.90
0.95
1.00
0.4
0.5
0.6
0.7
0.8
0.9
1.0
50% increase in ATP utilization
15% decrease in ATP utilization
