# Supplementary material for: MASSpy: Building, simulating, and visualizing dynamic biological models in Python using mass action kinetics
Source: PLoS Comput Biol. 2021 Jan 28;17(1):e1008208. doi: 10.1371/journal.pcbi.1008208 (PMC7872247; doi:10.1371/journal.pcbi.1008208)
Supplement: S3 File — All files necessary to reproduce the results presented in the validation, ensemble modeling feature demonstration, and case study. Alternatively, these files can be found at https://github.com/SBRG/MASSpy-publication. (ZIP) [file pcbi.1008208.s005.zip › MASSpy-publication-master/validation/data/figures/Reproduced-Supplemental-FigA.pdf]

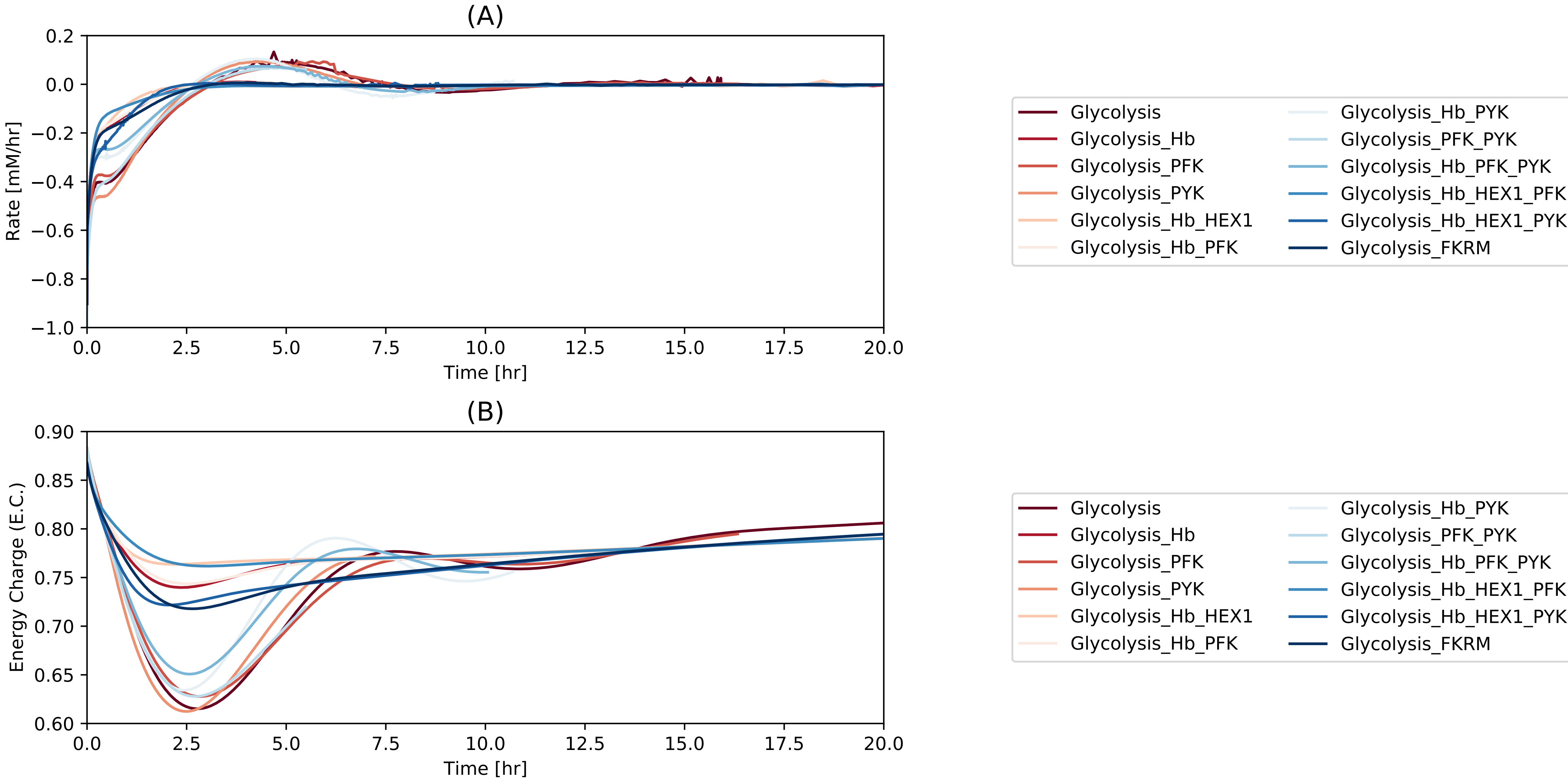
(A)
Rate [mM/hr]
0.2
0.0
−0.2
−0.4
−0.6
−0.8
−1.0
0.0
2.5
5.0
7.5
10.0
12.5
15.0
17.5
20.0
Time [hr]
Glycolysis
Glycolysis_Hb
Glycolysis_PFK
Glycolysis_PYK
Glycolysis_Hb_HEX1
Glycolysis_Hb_PFK
Glycolysis_Hb_PYK
Glycolysis_PFK_PYK
Glycolysis_Hb_PFK_PYK
Glycolysis_Hb_HEX1_PFK
Glycolysis_Hb_HEX1_PYK
Glycolysis_FKRM
(B)
Energy Charge (E.C.)
0.90
0.85
0.80
0.75
0.70
0.65
0.60
0.0
2.5
5.0
7.5
10.0
12.5
15.0
17.5
20.0
Time [hr]
Glycolysis
Glycolysis_Hb
Glycolysis_PFK
Glycolysis_PYK
Glycolysis_Hb_HEX1
Glycolysis_Hb_PFK
Glycolysis_Hb_PYK
Glycolysis_PFK_PYK
Glycolysis_Hb_PFK_PYK
Glycolysis_Hb_HEX1_PFK
Glycolysis_Hb_HEX1_PYK
Glycolysis_FKRM
