# Supplementary material for: MASSpy: Building, simulating, and visualizing dynamic biological models in Python using mass action kinetics
Source: PLoS Comput Biol. 2021 Jan 28;17(1):e1008208. doi: 10.1371/journal.pcbi.1008208 (PMC7872247; doi:10.1371/journal.pcbi.1008208)
Supplement: S3 File — All files necessary to reproduce the results presented in the validation, ensemble modeling feature demonstration, and case study. Alternatively, these files can be found at https://github.com/SBRG/MASSpy-publication. (ZIP) [file pcbi.1008208.s005.zip › MASSpy-publication-master/validation/data/figures/Reproduced-Supplemental-FigB.pdf]

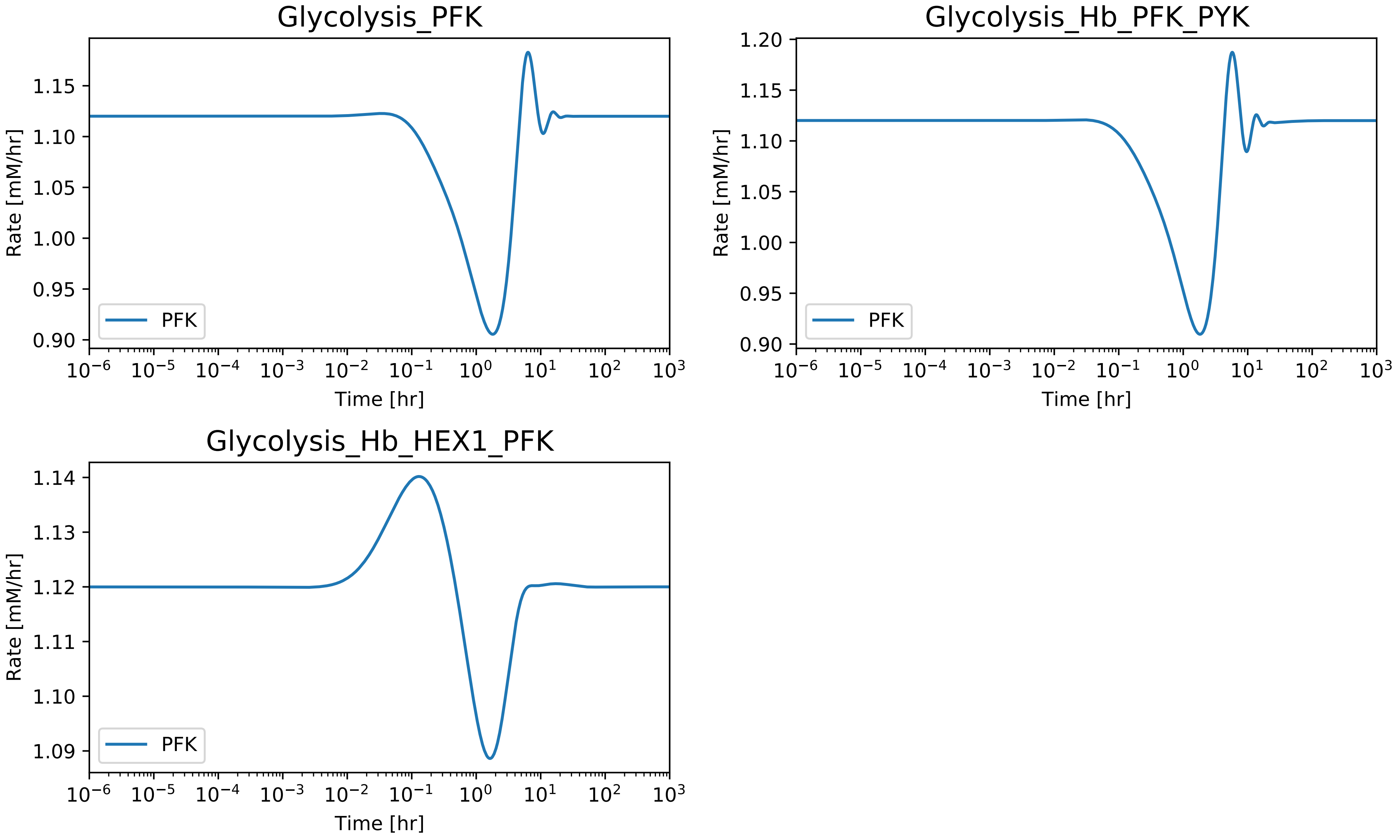

Glycolysis_PFK
Glycolysis_Hb_PFK_PYK
Glycolysis_Hb_HEX1_PFK
Rate [mM/hr]
Time [hr]
PFK
1.15
1.10
1.05
1.00
0.95
0.90
1.20
1.14
1.13
1.12
1.11
1.10
1.09
10^{-6}
10^{-5}
10^{-4}
10^{-3}
10^{-2}
10^{-1}
10^{0}
10^{1}
10^{2}
10^{3}
