# Supplementary material for: MASSpy: Building, simulating, and visualizing dynamic biological models in Python using mass action kinetics
Source: PLoS Comput Biol. 2021 Jan 28;17(1):e1008208. doi: 10.1371/journal.pcbi.1008208 (PMC7872247; doi:10.1371/journal.pcbi.1008208)
Supplement: S3 File — All files necessary to reproduce the results presented in the validation, ensemble modeling feature demonstration, and case study. Alternatively, these files can be found at https://github.com/SBRG/MASSpy-publication. (ZIP) [file pcbi.1008208.s005.zip › MASSpy-publication-master/validation/data/figures/Reproduced-Supplemental-FigD.pdf]

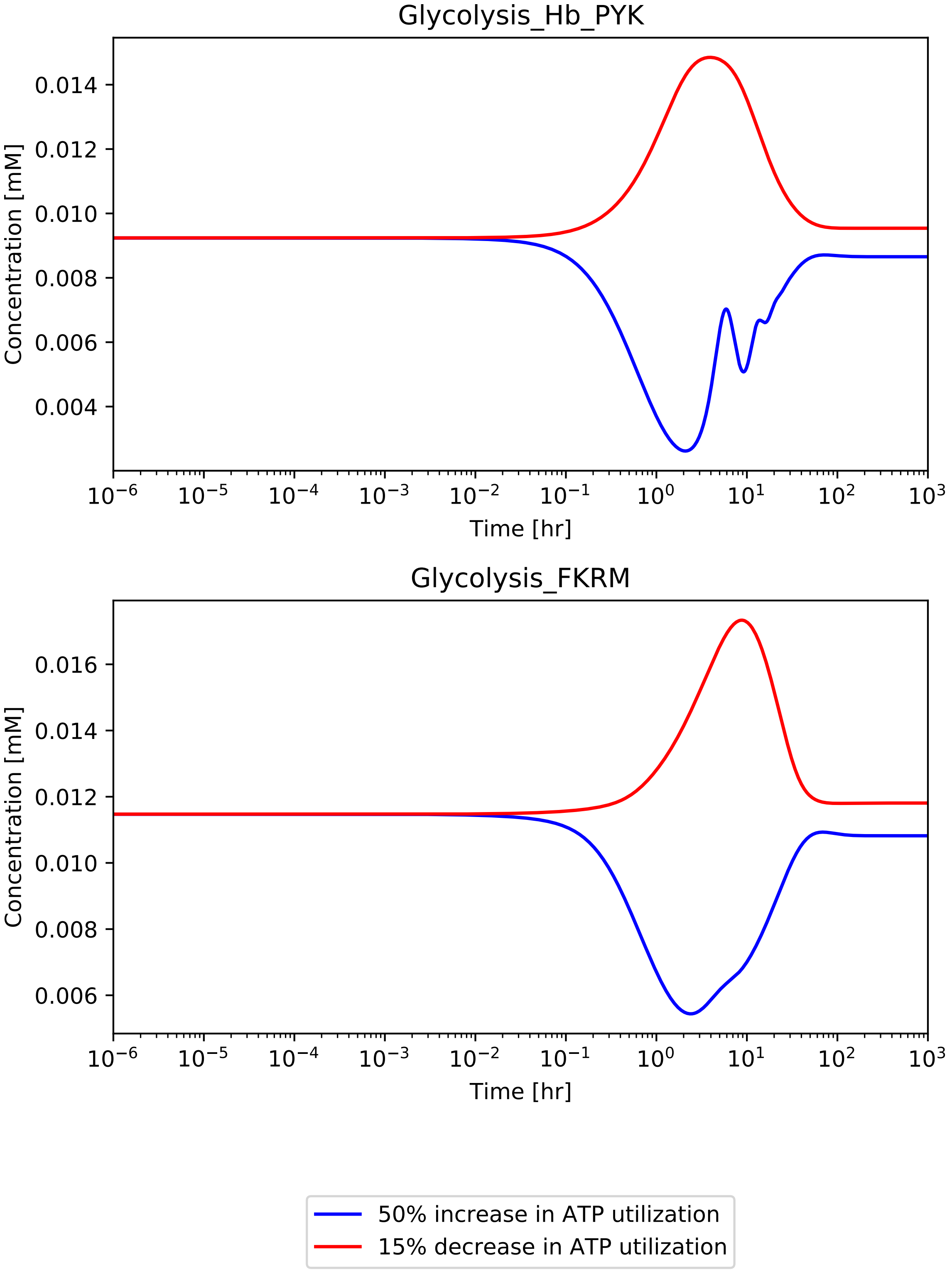
Glycolysis_Hb_PYK
Concentration [mM]
0.014
0.012
0.010
0.008
0.006
0.004
10^{-6} 10^{-5} 10^{-4} 10^{-3} 10^{-2} 10^{-1} 10^{0} 10^{1} 10^{2} 10^{3}
Time [hr]
Glycolysis_FKRM
Concentration [mM]
0.016
0.014
0.012
0.010
0.008
0.006
10^{-6} 10^{-5} 10^{-4} 10^{-3} 10^{-2} 10^{-1} 10^{0} 10^{1} 10^{2} 10^{3}
Time [hr]
50% increase in ATP utilization
15% decrease in ATP utilization
