# Supplementary material for: MASSpy: Building, simulating, and visualizing dynamic biological models in Python using mass action kinetics
Source: PLoS Comput Biol. 2021 Jan 28;17(1):e1008208. doi: 10.1371/journal.pcbi.1008208 (PMC7872247; doi:10.1371/journal.pcbi.1008208)
Supplement: S3 File — All files necessary to reproduce the results presented in the validation, ensemble modeling feature demonstration, and case study. Alternatively, these files can be found at https://github.com/SBRG/MASSpy-publication. (ZIP) [file pcbi.1008208.s005.zip › MASSpy-publication-master/validation/data/figures/Reproduced-Supplemental-FigF.pdf]

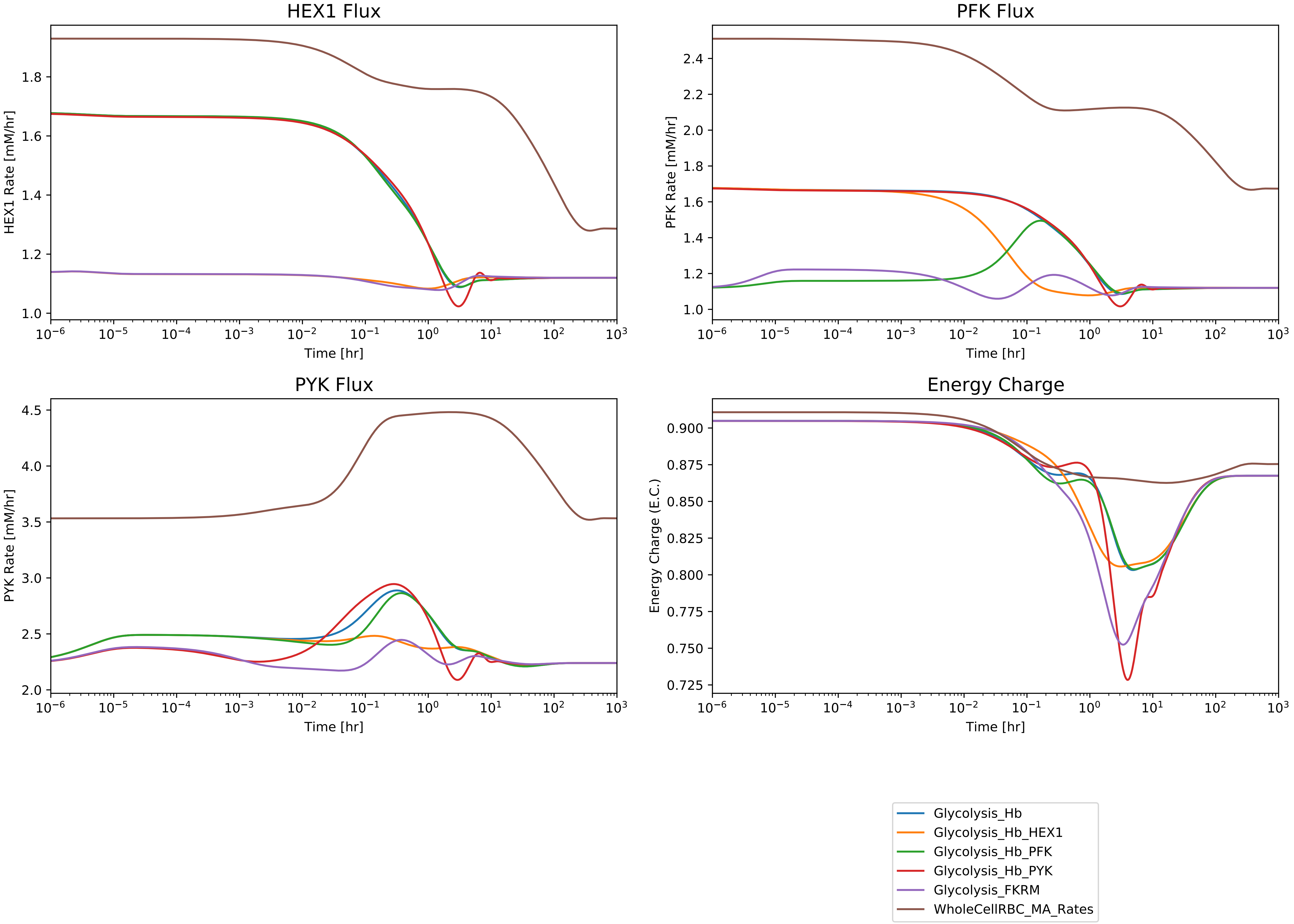
HEX1 Flux
HEX1 Rate [mM/hr]
Time [hr]
PFK Flux
PFK Rate [mM/hr]
Time [hr]
PYK Flux
PYK Rate [mM/hr]
Time [hr]
Energy Charge
Energy Charge (E.C.)
Time [hr]
Glycolysis_Hb
Glycolysis_Hb_HEX1
Glycolysis_Hb_PFK
Glycolysis_Hb_PYK
Glycolysis_FKRM
WholeCellRBC_MA_Rates
